# Opportunities for Integrated Ecological Analysis across Inland Australia with Standardised Data from Ausplots Rangelands

**DOI:** 10.1371/journal.pone.0170137

**Published:** 2017-01-17

**Authors:** Greg R. Guerin, Ben Sparrow, Andrew Tokmakoff, Anita Smyth, Emrys Leitch, Zdravko Baruch, Andrew J. Lowe

**Affiliations:** Terrestrial Ecosystem Research Network, Adelaide-node, School of Biological Sciences, University of Adelaide, North Terrace, Adelaide, South Australia, Australia; Technical University in Zvolen, SLOVAKIA

## Abstract

Australian rangelands ecosystems cover 81% of the continent but are understudied and continental-scale research has been limited in part by a lack of precise data that are standardised between jurisdictions. We present a new dataset from *AusPlots Rangelands* that enables integrative rangelands analysis due to its geographic scope and standardised methodology. The method provides data on vegetation and soils, enabling comparison of a suite of metrics including fractional vegetation cover, basal area, and species richness, diversity, and composition. Cover estimates are robust and repeatable, allowing comparisons among environments and detection of modest change. The 442 field plots presented here span a rainfall gradient of 129–1437 mm Mean annual precipitation with varying seasonality. Vegetation measurements include vouchered vascular plant species, growth form, basal area, height, cover and substrate type from 1010 point intercepts as well as systematically recorded absences, which are useful for predictive modelling and validation of remote sensing applications. Leaf and soil samples are sampled for downstream chemical and genomic analysis. We overview the sampling of vegetation parameters and environments, applying the data to the question of how species abundance distributions (SADs) vary over climatic gradients, a key question for the influence of environmental change on ecosystem processes. We found linear relationships between SAD shape and rainfall within grassland and shrubland communities, indicating more uneven abundance in deserts and suggesting relative abundance may shift as a consequence of climate change, resulting in altered diversity and ecosystem function. The standardised data of *AusPlots* enables such analyses at large spatial scales, and the testing of predictions through time with longitudinal sampling. In future, the *AusPlots* field program will be directed towards improving coverage of space, under-represented environments, vegetation types and fauna and, increasingly, re-sampling of established plots. Providing up-to-date data access methods to enhance re-use is also a priority.

## Introduction

Rangelands make up 81% of the Australian landmass according to an accepted spatial definition encompassing inland and northern Australia, and consist of a variety of vegetation types in which the predominant land-use is extensive, low-intensity livestock grazing [1]. Much of the rangelands is characterised by highly variable climate, old and nutrient poor soils and vegetation largely adapted to tolerate such harsh conditions [2,3]. Many rangelands ecosystems are fragile and susceptible to large-scale change from both natural and anthropocentric events as well as species declines [4,5]. Despite their vast spatial extent and heterogeneity, Australia rangeland ecosystems as a whole remain relatively poorly studied [3], although by no means without exceptions at local and regional scales [4,6]. Tropical savannas, for example, have been investigated somewhat more intensively than deserts [3,7,8].

Baseline information on these systems is essential to determine their current condition, while ongoing surveillance monitoring can track changes occurring in these environments and inform management decisions in these areas [5]. In addition, continental-scale ecological research across the Australian rangelands has been limited in part by a lack of data sources that are standardised between both repeated measurements (precision problem) and data collection efforts undertaken in different government jurisdictions (compatibility problem) in a spatially consistent manner.

To address some of these perceived gaps, the Australian Terrestrial Ecosystem Research Network (TERN) established *AusPlots*, a new surveillance monitoring capability, to collect standardised, plot-based monitoring data throughout Australian ecosystems [9]. The data and samples obtained are establishing ecosystem benchmarks for the Australian rangelands as part of the *AusPlots Rangelands* sub-program (hereafter *AusPlots*) and help address some key questions on ecosystem function raised by Morton et al. [10], including understanding of soil fertility, plant life histories and productivity. In addition, the data and sample analysis will address some key knowledge gaps in understanding environmental change and help direct environmental management [11]. The grasslands, shrublands and sparse woodlands of the rangelands were the first systems to be measured, with widespread data now collected over much of Australia’s rangelands areas.

We present here an overview of data derived from *AusPlots* that will allow integrative analysis across Australian rangelands ecosystems. The geographic scope of sampling is the entirety of the Australian rangelands and drylands, comprising the inland and northern reaches of the continent, spanning Australian State and Territory borders. In addition, all data are collected according to a well-defined, precise and standardised field methodology. For example, vegetation cover and structure are measured in a precise, objective manner, which means that detailed comparisons can be made between plot samples with minimal uncertainty due measurement method or observer variation. Visual estimates are minimised in favour of quantitative measures.

The *AusPlots* dataset is dynamic and growing over time. The data are freely available for download and re-use (under the conditions of the open access data licence) via the TERN’s Advanced Ecological Knowledge Observation System (ÆKOS; www.aekos.org.au; see TERN AusPlots [12]). The ÆKOS data portal delivers high quality, integrated, research data (at the site level) for environmental change analysis of mainly Australian terrestrial ecosystems. The portal is the gateway to accessing Australian ecology data and provides free access to, and information about, *in situ* species and environment plot data for intelligible and ethical reuse of other people’s research data. Up-to-date *AusPlots* data are publicly accessible as a customised ‘site by species’ csv flat file (or shapefile) or a relational MySQL/Postgres database for all integrated site data (TERN Eco-informatics www.ecoinformaticis.org.au). Data can alternatively be explored, visualised and downloaded via the interactive website 'Soils2Satellites' (www.soils2satellites.org.au). The processed data used here are also available for download as a static dataset [13].

These data are useful not only for specific questions relating to Australian rangelands ecosystems, but also for testing ecological theories and analysis methods or software where systematically gathered vegetation (composition, cover, growth forms and structure) and soil data over continental scales are desirable.

Here, we describe and visualise the variation in vegetation and environments sampled by *AusPlots* and explore the completeness of species sampling. We demonstrate an application of the data to an important ecological question: *Do plant species relative abundance patterns vary in a predictable way over continental-scale edaphic and climatic gradients*? This is a key question for community assembly and predictive modelling applications because abundant species characterise communities and ecosystem function [14,15].

The tendency for ecological communities to be made up of few abundant species and many rare species has been well established empirically, a pattern described by species abundance distributions, or SADs [16]. While there have been numerous empirical examples of SADs that fit this basic pattern, there is no consensus about the most appropriate or useful probability distributions or models that can be fitted to field data to describe the shape of those distributions, in fact the distribution or model that best fits a given SAD may itself be an informative ecological indicator [16].

Perhaps because of a focus on which statistical or ecological model best describes SADs, there has been relatively little advancement on questions of how SAD shape or species evenness (i.e. model parameters) vary along major climatic gradients, despite a well established finding that the percent importance of the top ranked species increased with latitude globally and in stressed environments regionally [17]. Disturbance may also influence SADs, which makes them a potential ecological indicator of successional stage and condition [17–19].

## Materials and methods

### Study area

**Fig 1:**
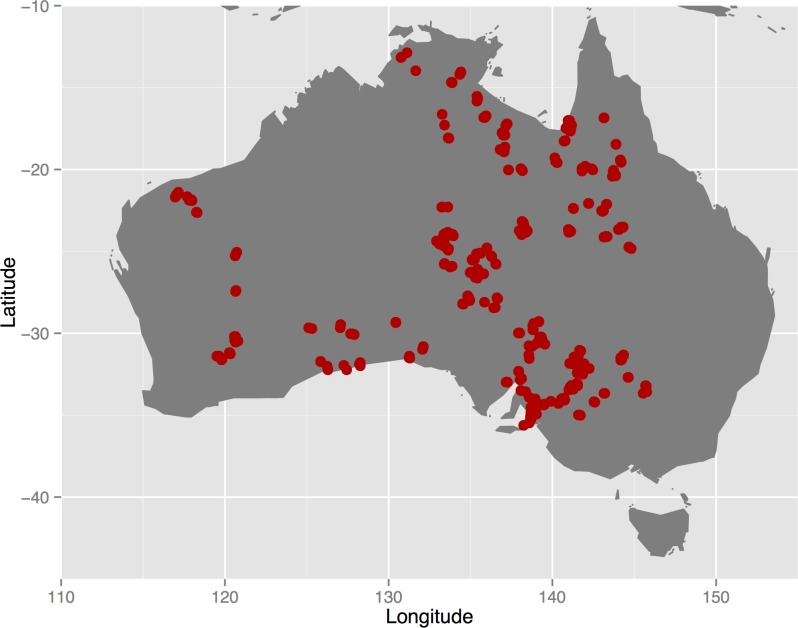
Location of *AusPlots* within Australia.

The study sites are largely confined to the Australian rangelands, according to an established spatial definition [20,21], with a small number of sites extending into the adjoining Mediterranean climate zone in the south (Fig 1). Across this area there is rainfall gradient from north (summer dominant monsoonal) to south (winter dominant), with central arid areas characterised by on average very low, aseasonal rainfall characterised by long droughts punctuated by high rainfall events [8,22,23]. The highly variable rainfall is a major driver of spatial patterns and change in rangelands [24], with temporal and spatial variability particularly evident and important in arid areas [22,23]. Ecosystems present in the study area include tropical woodlands and savannas (northern), tussock grasses (mid-north downs country), hummock grasslands, shrublands, mulga and other *Acacia* woodlands (mid-latitudes), and chenopod shrublands (southern regions; [25]).

The site selection procedure consisted of stratifying bioregions based on clustering of climate and landscape attributes and 'Major Vegetation Groups' (MVGs), then selecting representative bioregions and locating plots at finer scales to cover biophysical and disturbance gradients whilst setting target minima for vegetation types and considering logistical aspects such as access and opportunities to integrate with existing sites [26].

### Field method

A set of 442 field sites was sampled with a standard methodology in the present dataset, with 17 of those sites measured twice for vegetation parameters (Fig 1). The *AusPlots* survey method is made up of a series of individual modules, which are described in full in a protocols manual [26]. The modules involve: plot selection and layout; photo-panoramas; vascular plant vouchering; plant tissue sampling for genetic and chemical analysis; point intercept; basal area; structural summary; leaf area index; and soils and soil sampling for genomic and chemical analysis. Field data are recorded directly onto mobile (tablet) devices before being stored in cloud-based server infrastructure and ultimately sent to the ÆKOS data repository. For reference, we give below a brief outline of the field modules most relevant to data presented here.

Plot layout: 1 ha (100 x 100 m) plots are permanently marked over a homogenous patch of terrestrial vegetation. Structural summary: the dominant species in three vegetation strata (lower, middle and upper) are identified visually by the observer. The vegetation is then categorised into MVGs according to the Australian National Vegetation Information System (NVIS; [27]) by overlaying point locations with the MVG layer in ArcGis and manually correcting based on the structural summary description. Sites classified to the same MVG may have different species composition but a similar structural formation, often defined by the dominant genus, for example '*Acacia* Forests and Woodlands'. Vascular plant vouchering: vascular plants within the plot are detected visually, with a herbarium voucher taken for each unique taxon, which is assigned a barcode and identified then permanently stored in a herbarium. Point intercept: 10 x 100 m long transects are laid out within the plot in a grid pattern. A staff with laser pointer and densitometer is used to record species, growth form and height plus substrate type every 1 m along the transects, resulting in a total of 1010 (10 x 101) point intercept hits for the plot.

All sites were sampled under permits issued by State and Territory authorities and with individual permission from private landholders as follows: NSW–Office of Environment and Heritage and NSW National Parks and Wildlife Service (Western LLS, Murray LLS, Riverina LLS); NT–Parks and Wildlife Commission Northern Territory (multiple locations); QLD–Department of Environment and Heritage Protection (multiple locations); SA–Department of Environment, Water and Natural Resources (whole State except Wilderness Protection Areas); VIC–Department of Environment and Primary Industries (Murray-Sunset NP, Alpine NP); WA–Department of Parks and Wildlife (whole state).

### Data extraction and compilation

An R [28] script which connects directly to an internal *AusPlots* PostgreSQL relational database was used to extract site and vegetation data via a set of queries. The extraction process generates a series of individual data files for each plot containing the raw data (e.g. basal area, point intercept, vouchers, site information, structural summaries, soil bulk density, soil characterisation and soil sub-sites). Additional scripts were used to compile the extracted raw data from individual plots into a single data table and to re-shape those data tables for presentation and downstream analysis. For example, point intercept hits from all plots were compiled together and labelled by unique plot, visit and hit identifiers. These data tables were further processed to calculate the percent cover of each species and generate species cover-abundance against sites matrices. Foliage Projective Cover (FPC) was calculated for each species as the number of point intercept hits within a plot, excluding 'in canopy sky' hits, divided by the total number of hits multiplied by 100 (percent). An alternative cover metric is Opaque Canopy Cover, in which 'in canopy sky' hits are included and the canopy is therefore treated as a solid convex polygon.

### Environmental and spatial data

A map layer of the Australian Rangelands (Australian Government) was converted to a 0.1 degree resolution raster and the centroid coordinates of grid cells were extracted, resulting in a set of 55,643 spatial points representing the rangelands. Environmental data for these rangelands coordinates and the coordinates for *AusPlots* field sites were extracted from selected spatial environmental (climate, landscape and soil) layers (S1 Appendix). Bioclimatic variables were obtained from WorldClim [29] grid with 3' resolution. Variables presented here are: Mean (annual) temperature, Mean annual precipitation (MAP), Mean Maximum Temperature of the Warmest Month and Precipitation Seasonality. The following soil and landscape variables were obtained from the Terrestrial Ecosystem Research Network's 'Soil and Landscape Grid of Australia' and 'National Elevation and Terrain' datasets via the CSIRO data portal (https://data.csiro.au/) in the form of 3'' resolution spatial grids: total phosphorus; total nitrogen, available water capacity; soil depth; percent sand; elevational relief over 1000 m [30–35]. Where relevant, values for the top soil layer were selected. These variables were selected to represent major biophysical gradients and to visualise the sampling of a diverse set of ecologically relevant variables at *AusPlots* sites.

### Descriptive statistics

Descriptive statistics and associated plots were generated to visualise the sampled variation in basic vegetation parameters as well as the environmental space represented by the field plots. Boxplots for variables show the median value and interquartile range (IQR) with whiskers extending to data that fall no more than 1.5 times outside the IQR. Scatterplots show the extent of coverage of *AusPlots* in the context of the climate space occupied by the Australian rangelands and the major latitudinal gradients evident in the bioclimatic variables. For each plot, we calculated the Shannon Diversity and Simpson Dominance indices based on species presences and cover (FPC) scored from the point intercept module.

### Species sampling and cover estimates

Vascular plant species were sampled via herbarium vouchers taken for all species observed within the plot during exhaustive visual searches as well as during the collection of point intercept data. We compared species richness in plots as measured by these methods. We also assessed the completeness of species sampling in the point intercept module by generating species accumulation curves, whereby individual point intercept samples were rarefied to visualise the accumulation of species with increasing point intercepts.

For the point-intercept module, we calculated cumulative cover (FPC) estimates for each recorded species with increasing sampling. Point-intercepts were added sequentially to a data pool and cover (FPC) was calculated with each addition. Point intercept hits were added in an order equivalent to that in which they were collected, that is, transects were added in a zigzagging pattern across the plot and hits along each transect added in numerical order (i.e. transect East1-West1 hits 0 to 100, West2-East2 hits 0–100, and so on). This gives a more realistic visualisation of the sampling required to produce stable cover estimates, because randomising the order of point-intercepts across the plot would assume that additional intercepts are taken at random locations, which is not the case in the field. For each analysis, intercept points were treated as samples that may record zero (substrate only), one or multiple species.

### Species dominance analysis

Species abundance distributions (SADs) were fitted to cover-abundance profiles for species in each plot using Maximum Likelihood, an appropriate fitting method for SADs [19]. We used the Pareto distribution because we were interested in comparing empirical SAD estimates rather than testing process-based models. Such power law functions have been shown to be appropriate for plant cover-abundance data and to perform better than the lognormal distribution for abundance distribution extremities (i.e. species with the highest and lowest abundances) [16,36]. Traditionally though, lognormal SADs have been fitted to community abundance profiles. Although they have been increasingly criticised in the literature as inappropriate, despite good empirical fitting [18,37,38], we calculated coefficients for lognormal SADs for comparison with Pareto (assessed via AIC (Akaike Information Criterion), see Ulrich et al. [39] for comparable example) and previous studies.

Plots in which less than six species were recorded in the point intercept module were excluded from the SAD analysis because, although low diversity observations could be ecologically relevant, the model fitting is likely to be unreliable, or impossible, with small numbers of species [16]. The shape parameter α of the Pareto model describes how rapidly the expected number of species declines with increasingly abundance in the distribution, and from this, relative abundance distributions (RADs) can be predicted and compared to their empirical equivalent, Whittaker plots [16]. A higher value of alpha indicates a higher left skew, so that abundance is less evenly shared among the species (more species have low proportional abundance). For the lognormal shape parameter σ the reverse is true, where more negative values represent less even abundances.

The relationship of the shape parameters (modelled for abundance profiles for each plot) to environmental variables was explored with scatterplots and regression. Linear models were assessed for fit and relevance through a combination of residual plots, R^2^ values, and slope. Because diagnostic plots of Ordinary Least Squares (OLS) models indicated significant outliers and data points that may be unduly leveraging the model fit, we fitted models using robust regression and the resulting weights were used in the calculation of R^2^ (R^2^_WLS_; [40]). Slope coefficients were bootstrapped with 1000 replicates.

To assess whether any patterns detected among SADs related to changes in vegetation structure (for example woodland versus grassland) between bioclimatic regions rather than ecological dominance effects *per se*, we partitioned subsets of field plots by MVG and used five of the most frequently sampled vegetation types to ensure adequate sample size (n = 37–74).

### Software

All data extraction, compilation, visualisation and analysis was conducted in R [28] using a suite of packages including 'RPostgresSQL', ‘vegan’, ‘raster’, ‘sads’ and 'MASS' [36,41–44].

## Results

### Descriptive statistics

**Fig 2:**
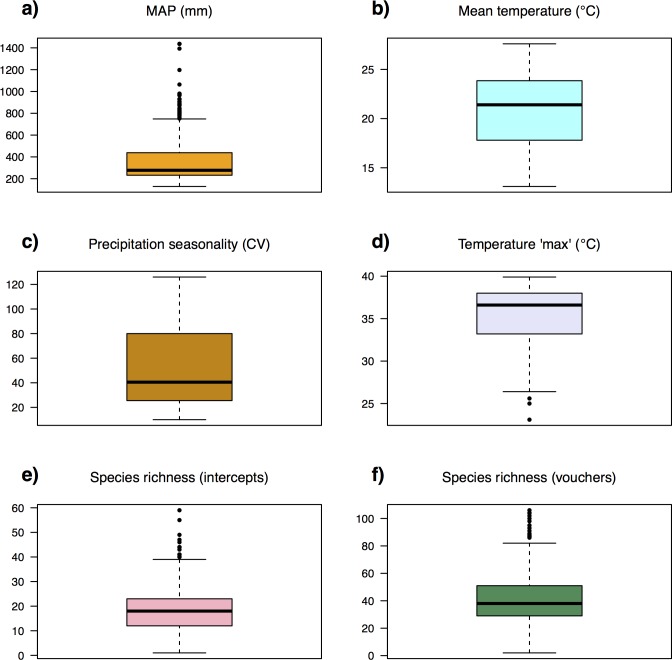
Boxplots of climatic variables as sampled by *AusPlots* and species richness. Bold line represents median, coloured box the interquartile range, whiskers up to 1.5x interquartile range from median, points outliers: (a) Mean annual precipitation (MAP); (b) Mean temperature; (c) Precipitation seasonality (coefficient of variation); (d) Mean maximum temperature of the warmest month; (e) Species richness (point intercepts); (f) Species richness (vouchers).

**Fig 3:**
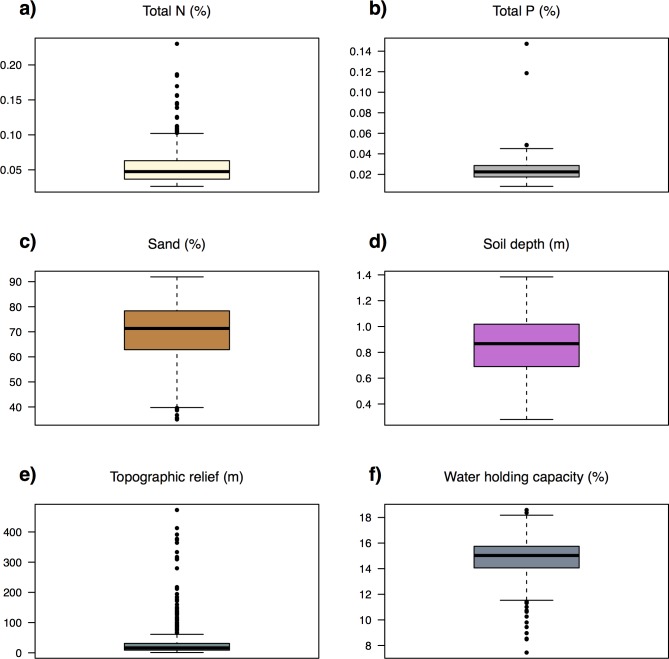
Boxplots of soil and landscape variables as sampled by *AusPlots*. Bold line represents median, coloured box the interquartile range, whiskers up to 1.5x interquartile range from median, points outliers: (a) Total nitrogen; (b) Total phosphorus; (c) Percent sand; (d) Soil depth; (e) Topographic (elevational) relief within 1 km; (f) Available water capacity.

**Fig 4:**
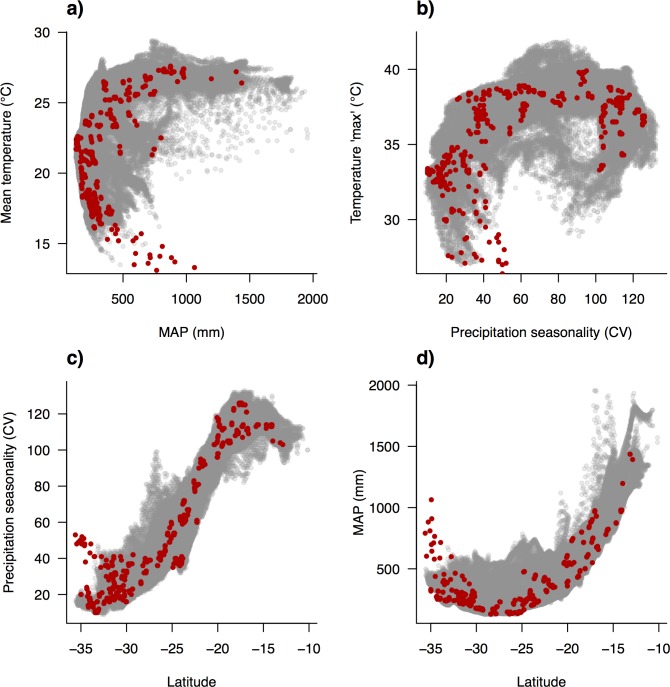
Scatterplots of climatic variables as sampled by *AusPlots*. *AusPlots* (red) are shown in the context of the climate space of the Australian Rangelands as a whole (grey): (a) Mean temperature versus Mean annual precipitation (MAP); (b) Mean maximum temperature of the warmest month versus Precipitation seasonality (coefficient of variation); (c) Precipitation seasonality versus latitude; (d) Mean annual precipitation versus latitude.

Vegetation recorded in *AusPlots* was classified into 22 MVGs (Table 1). Field plots spanned a major continental gradient in MAP of 129–1437 mm and Precipitation Seasonality ranging from the aseasonal interior deserts to the strongly seasonal tropical north (mainly summer rainfall) and somewhat seasonal Mediterranean-climate south (winter rainfall zone), with scores (coefficient of variation in monthly data) varying between 10 and 126 (Fig 2). Landscapes sampled by *AusPlots* are predominately– but not exclusively–characterised by low topographic relief and soils that are sandy and low in N and P (Fig 3). A large proportion of the climate space occupied by the Australian rangelands has been sampled by *AusPlots* (Fig 4). Shannon Diversity and Simpson Dominance Indices values for plots ranged between 0–3.17 and 0–0.93, respectively (S1 Appendix).

**Table 1:** Vegetation groups sampled by *AusPlots*.

Major Vegetation Group (MVG)	Number of plots
***Acacia* Forests, Woodlands and Open Woodlands**	**42**
***Acacia* Shrublands**	**37**
*Callitris* Forests and Woodlands	4
*Casuarina* Forests and Woodlands	6
**Chenopod Shrublands, Samphire Shrublands and Forblands**	**74**
Cleared, non-native vegetation, buildings	1
Eucalypt Low Open Forests	3
Eucalypt Open Forests	10
Eucalypt Open Woodlands	28
**Eucalypt Woodlands**	**56**
Heathlands	2
Hummock Grasslands	18
Inland aquatic—freshwater, salt lakes, lagoons	1
Low Closed Forests and Tall Closed Shrublands	1
Mallee Open Woodlands and Sparse Mallee Shrublands	18
Mallee Woodlands and Shrublands	29
*Melaleuca* Forests and Woodlands	17
Other Forests and Woodlands	8
Other Open Woodlands	2
Other Shrublands	39
Tropical Eucalypt Woodlands/Grasslands	2
**Tussock Grasslands**	**60**

Bold entries mark those used to create data subsets for analysis of Relative Abundance Profiles (refer to text). Classification based on NVIS [27].

### Species sampling and cover estimates

**Fig 5:**
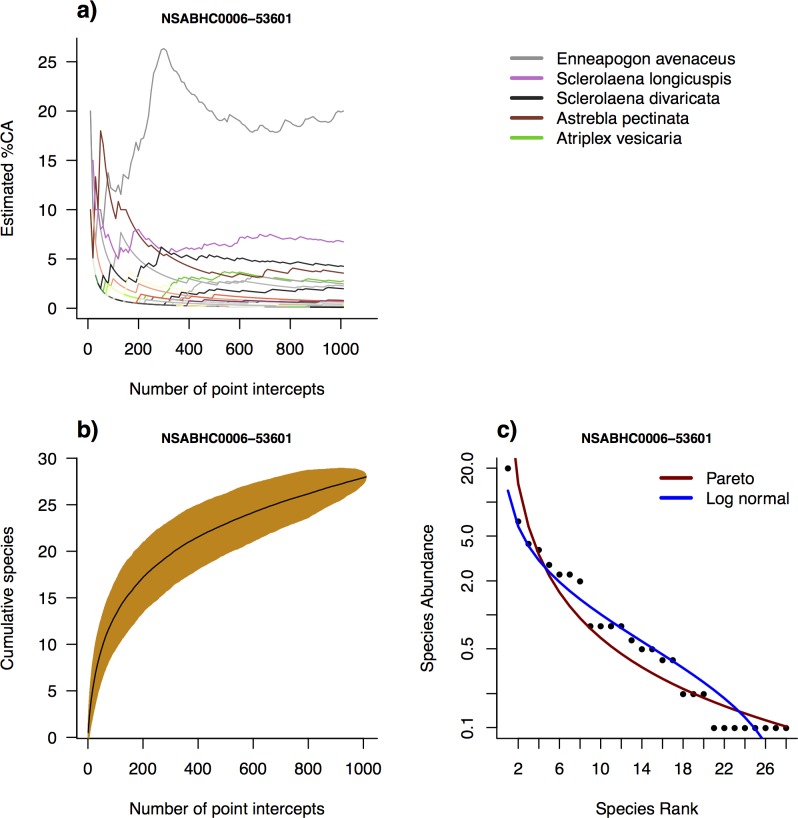
**Sampling and relative abundance example for a Tussock Grassland plot (NSABH0006-53601):** (a) Cumulative cover abundance (%CA; Foliage Projective Cover) for species with point intercepts taken across the plot. Five most abundant species are labelled; (b) Species accumulation curve with point intercepts within a plot (1000 random replicates); (c) Modelled Rank Abundance Distributions over a Whittaker plot.

**Fig 6:**
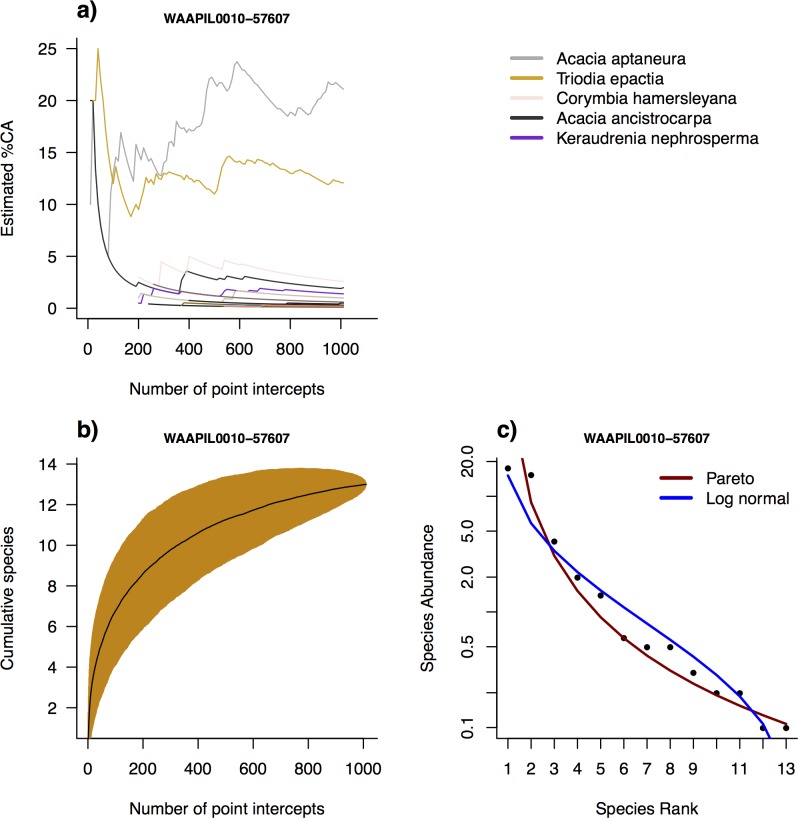
**Sampling and relative abundance example for an *Acacia* shrubland plot (WAAPIL0010-57607):** (a) Cumulative cover abundance (%CA; Foliage Projective Cover) for species with point intercepts taken across the plot. Five most abundant species are labelled; (b) Species accumulation curve with point intercepts within a plot (1000 random replicates); (c) Modelled Rank Abundance Distributions over a Whittaker plot.

Species accumulation curves varied between plots but in general became relatively flat by 1010 point intercept hits, indicating that additional sampling effort would be unlikely to detect significantly more species using this method (Figs 5 and 6; S2 Appendix). Observed species richness was approximately doubled from vouchered sampling via visual searches within the plot (mean 42; range 2–106) compared to the point-intercept module (mean 19; range 1–59; Fig 2) although this ratio varied from plot to plot. Overall, more than 3,000 taxa were recorded.

Cumulative cover (FPC) estimates for individual species within each plot show that cover estimates are usually relatively unstable with small numbers of intercepts (i.e. cover estimates change quickly with the addition of samples), while estimates become stable with the inclusion of the full set of intercepts (i.e. additional sampling points do not change the estimates; Figs 5 and 6; S3 Appendix).

### Species dominance analysis

**Fig 7:**
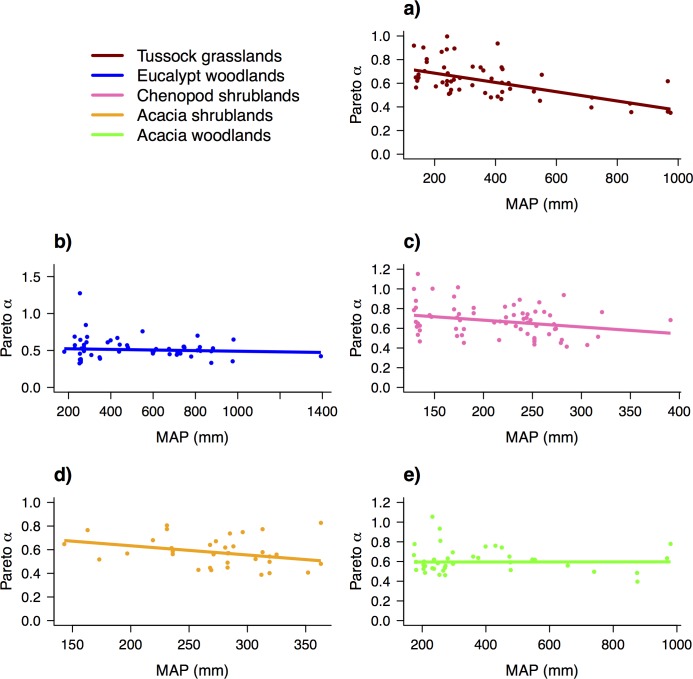
Relationship between Species Abundance Distributions (SADs) and Mean annual precipitation. Robust linear regressions with the predictor variable Mean annual precipitation (MAP) and the response variable shape coefficients of SADs models fit to abundance data for *AusPlots* using the Pareto (power-law) distribution: (a) Tussock grasslands; (b) Eucalypt woodlands; (c) Chenopod shrublands; (d) *Acacia* shrublands; (e) *Acacia* woodlands. Frequency of vegetation groups is shown in Table 1 and regression statistics are shown in Table 2. Note that Chenopod shrublands and *Acacia* shrublands (figure panels (c) and (d)) only occur across the most arid part of the continental MAP gradient (shown) making the regressions appear flatter despite the slopes being the highest of the five models shown–see Table 2.

SADs were fitted for all plots save 17 in which five or fewer species were recorded (Figs 5 and 6, S4 and S5 Appendices). Pareto models performed better than lognormal in terms of the lowest AIC in 12x as many cases, although visually in some cases lognormal appeared a closer fit for middle-range abundances (S1 Appendix). The shape parameters α and σ for Pareto and lognormal, respectively, were linearly related to MAP (Fig 7; S6 Appendix; Table 2), which corresponded to an approximately quadratic relationship with latitude (data not shown). There was no relationship between SAD shape and any other environmental variable tested (data not shown).

**Table 2:** Regression statistics for Species Abundance Distributions (SADs) along a continental Mean annual precipitation (MAP) gradient for vegetation group subsets.

Vegetation Group	Response	Coefficient (x 10^−4^)	R^2^_WLS_	Bootstrap lower 90% CI (x 10^−4^)	Bootstrap upper 90% CI (x 10^−4^)
Tussock Grasslands	Pareto α	-3.93	0.49	-5.33	-2.79
	Lognormal σ	17.74	0.68	13.23	28.49
Eucalypt Woodlands	Pareto α	-0.41	0.67	-1.55	0.52
	Lognormal σ	2.41	0.19	-1.92	7.34
Chenopod Shrublands	Pareto α	-6.94	0.32	-12.69	0.27
	Lognormal σ	14.73	0.30	-0.73	29.72
*Acacia* Shrublands	Pareto α	-7.81	0.59	-16.5	-1.33
	Lognormal σ	24.7	0.29	2.63	45.94
*Acacia* Forests and Woodlands	Pareto α	0.03	0.61	-2.08	1.83
	Lognormal σ	0.62	0.17	-5.58	6.98
Combined	Pareto α	-1.93	0.39	-2.37	-1.42
	Lognormal σ	6.61	0.26	4.62	8.38

Note coefficients are not comparable between Pareto and lognormal. Refer to Table 1 for MVGs and sample sizes. Coefficients are considered more robust if bootstrapped Confidence Intervals (CI) do not overlap zero.

Shape–MAP regressions were repeated for the MVG subsets: 'Tussock Grasslands', 'Eucalypt Woodlands', 'Chenopod Shrublands, Samphire Shrublands and Forblands', *'Acacia* Shrublands' and *'Acacia* Forests and Woodlands', the five most frequently sampled vegetation groups. There were linear relationships for all shrublands and grasslands whereas slope coefficients for woodlands were small and bootstrapped 90% Confidence Intervals (CIs) strongly overlapped zero (Fig 7; S6 Appendix; Table 2). Bootstrapped CIs for chenopod communities also marginally overlapped zero. Slopes were steepest for Pareto in chenopod and *Acacia* shrublands and for lognormal, in shrublands and grasslands.

There was no correlation between SAD shape and species richness (Pearson's r = 0.06 and -0.21 for Pareto and lognormal, respectively), while Shannon Diversity (Pearson's r = 0.76), and Simpson Dominance Index (Pearson's r = 0.57) were correlated with species richness. The Simpson and Shannon metrics were almost perfectly correlated (Pearon's r = 0.94), while SAD shape was not correlated with either (Pearson's r = -0.15 to 0.11).

## Discussion

Robust ecosystem monitoring that can report on condition and trajectory in rangelands is needed to inform management and requires precise and objective measurements of tractable indicators with generalised links to climate and disturbance regimes [5]. The *AusPlots* program provides standardised and quantitative information on rangelands and drylands vegetation and soils at continental scale, enabling comparisons of a suite of metrics such as fractional vegetation cover (the proportion of living and dead vegetation and vegetation free substrate), basal area plus species richness, diversity and composition.

The core field module of *AusPlots* is the point intercept survey, which records substrate, plant species, growth form and height at each of 1010 intercepts located along 10 transects arranged in a grid within 1 ha plots. The visualisations of raw point intercept data for each plot presented here (Figs 5 and 6; S2 and S3 Appendices) demonstrate that the number and arrangement of intercepts employed is adequate for maximising species detection and for stable estimation of cover, in that estimates of species richness and cover become relatively stable by 1010 hits. Species accumulation curves can be used to assess sampling completeness and their shape is also informative of patchiness and relative abundance [45]. However, the vouchering of plant species within *AusPlots* based on visual searches typically records around double the number of species compared to point intercept, which suggests that point intercepts are useful for precise measurements of structure, cover and relative abundance, while vouchered species composition are suitable for applications where total floral diversity (species presence/absence) is more important.

To demonstrate an application of the point intercept data, we used *AusPlots*' robust measure of Foliage Projective Cover to test for continent-wide patterns in the relative abundance of plant species. The relationships found between species abundance distributions (SADs) and Mean annual precipitation when tested over all plots suggest that cover-abundance is typically more evenly distributed among species in wetter environments. However, when plots were grouped by vegetation type, this pattern was present within shrub and grass dominated vegetation types but absent (or at best weak) within tree dominated vegetation types. The reasons behind the different responses among vegetation types are not evident from our exploratory analysis but are worthy of further investigation. The results suggests that, for some vegetation types, changes to relative abundance may be a consequence of climate change and decreases or increases to rainfall in the future, which may result in altered diversity and therefore ecosystem structure and function [15]. For example, experimental results have suggested that the evenness of abundances within a community influences its potential for plant invasions [46] and that higher unevenness can result in lower biomass [47] and functional resilience to environmental stress [48].

SADs capture an ecologically relevant property of plant communities that can be used to test theories of community assembly as well as an indicator for monitoring across diverse species assemblages [19]. More research is needed to determine empirically how disturbance (e.g. grazing and fire regimes) influences relative abundance and SADs [1,19,49]. For our data, there was an empirical distinction between SAD shape as a measure of evenness and more traditional diversity metrics such as Shannon Diversity, which is mathematically related to species richness.

Combined, the visualisation and analysis of the point intercept and species cover data presented here are intended to highlight that the standardised and precise nature of these data enable a range of analyses at ecological community–indeed continental–level. The cover estimates are robust and repeatable, allowing for comparisons among sites and environments as well as detection of modest changes in vegetation structure and relative abundance. While we have presented species-based examples of the point intercept data, these data also have applications in providing cover estimates for plant growth forms or fractional cover (the proportional cover of photosynthetic and non-photosynthetic vegetation and bare substrate), for example to structurally classify survey plots according to formulae based on plant height and opaque canopy cover.

### Potential applications and re-use

The dataset presented here and its future iterations may be useful for a range of applications in raw and more processed formats. Species composition (identity and relative abundance) is a standard metric for a range of ecological analyses including ordination and classification. Basal area is a key predictor of woody plant biomass across species [50] and is measured in the *AusPlots* method using a basal wedge, with work in development to also estimate from photo-point data. Basal area can be included in ecological models and in measurement and models of above ground carbon storage. Additionally, what isn't recorded in *AusPlots* may be useful as a resource for ecosystem science, as spatially independent sites in different environments with systematically recorded absences of species (but potentially also plant growth forms or structural vegetation types) are needed for model training and validation. The point intercept module provides a range of information on vegetation structure, cover, composition and heterogeneity.

Environmental and climatic parameters associated with plot locations can be inferred via intersection with high-resolution, interpolated spatial layers, a strategy employed here for exploring the breadth of sampling and associated ecological change. For some applications, *in situ* soil attributes, in particular, may be a more appropriate data resource that will be available in future via analysis of archived soil samples.

### Future directions

The future *AusPlots* field program will be directed towards improving spatial coverage, including the targeting of under-represented regions of environmental space. Increasingly, effort will be invested in re-sampling established plots, with decreasing emphasis on new plots over time. There are also methods currently undergoing consultation within the ecosystem science community to expand the habitats targeted and the range of attributes that are measured to be more spatially and taxonomically comprehensive. For example, forests are already sampled with a separate protocol by *AusPlots Forests* [51] but draft methods protocols also exist for woodlands, fauna and condition. Taking these measurements in more intensively managed or used landscapes would further increase the relevance and breadth of the dataset.

The flip-side to the on-going field program for *AusPlots* is enabling access to the data and physical samples that are collected. Providing alternative data access methods will make re-use of the data easier and keep up with the latest science methods and data trends, such as public querying of data directly through the R software package. To this end, we have developed an early prototype of an R package that will provide helper functions to assist with extraction of *AusPlots* data from ÆKOS directly from R in a process that makes use of ÆKOS’ externally visible SPARQL interface and makes it easier to extract and manipulate published *AuPlots* data.

## Conclusions

We present the first collated dataset sampled by the *AusPlots* program, giving an overview of the breadth of sampling in terms of space, environments and vegetation. The 442 field plots established to date across inland Australia have recorded over 3,000 vascular plant taxa in 22 major vegetation types including savanna, eucalypt woodland, chenopod shrubland and grassland. The standardised and quantitative nature of the data collection combined with an open access data approach and the broad spatial scope make this a useful data set for many applications including analysis of vegetation cover (by species, growth form or fractional cover) and species composition modelling, which benefits from systematically recorded absences. The distribution of relative species abundances as measured in the field plots with point intercepts provide a high level ecological and condition indicator that can be compared among a set of highly heterogeneous and widespread habitats. In-filling of spatial and environmental sampling gaps and, increasingly, a push towards temporal re-visits of baseline sites will increase the utility of the dataset as it develops in future.

## Supporting Information

S1 Appendix Site data. Details of *AusPlots* with environmental variables, metrics and model parameters.
(XLSX)

S2 Appendix Species accumulation curves for *AusPlots*. Species accumulation curves with point intercepts within a plot (1000 random replicates).
(PDF)

S3 Appendix Cumulative percent cover plots for *AusPlots*. Cumulative cover abundance (%CA; Foliage Projective Cover) for species with point intercepts taken across plots. Five most abundant species are labelled.
(PDF)

S4 Appendix Modelled Rank Abundance Distributions (Pareto) over Whittaker plots for *AusPlots*. Plots of species abundance (percent cover) against species rank.
(PDF)

S5 Appendix Modelled Rank Abundance Distributions (Lognormal) over Whittaker plots for *AusPlots*. Plots of species abundance (percent cover) against species rank.
(PDF)

S6 Appendix Robust linear regressions. Regressions in subsets by vegetation group with predictor variable Mean annual precipitation (MAP) and response variable shape coefficients of SADs models fit to abundance data for *AuspPlots* using the lognormal distribution.
(PDF)
