# Supplementary material for: Opportunities for Integrated Ecological Analysis across Inland Australia with Standardised Data from Ausplots Rangelands
Source: PLoS One. 2017 Jan 17;12(1):e0170137. doi: 10.1371/journal.pone.0170137 (PMC5241013; doi:10.1371/journal.pone.0170137)
Supplement: S2 Appendix — Species accumulation curves with point intercepts within a plot (1000 random replicates). (PDF) [file pone.0170137.s002.pdf]

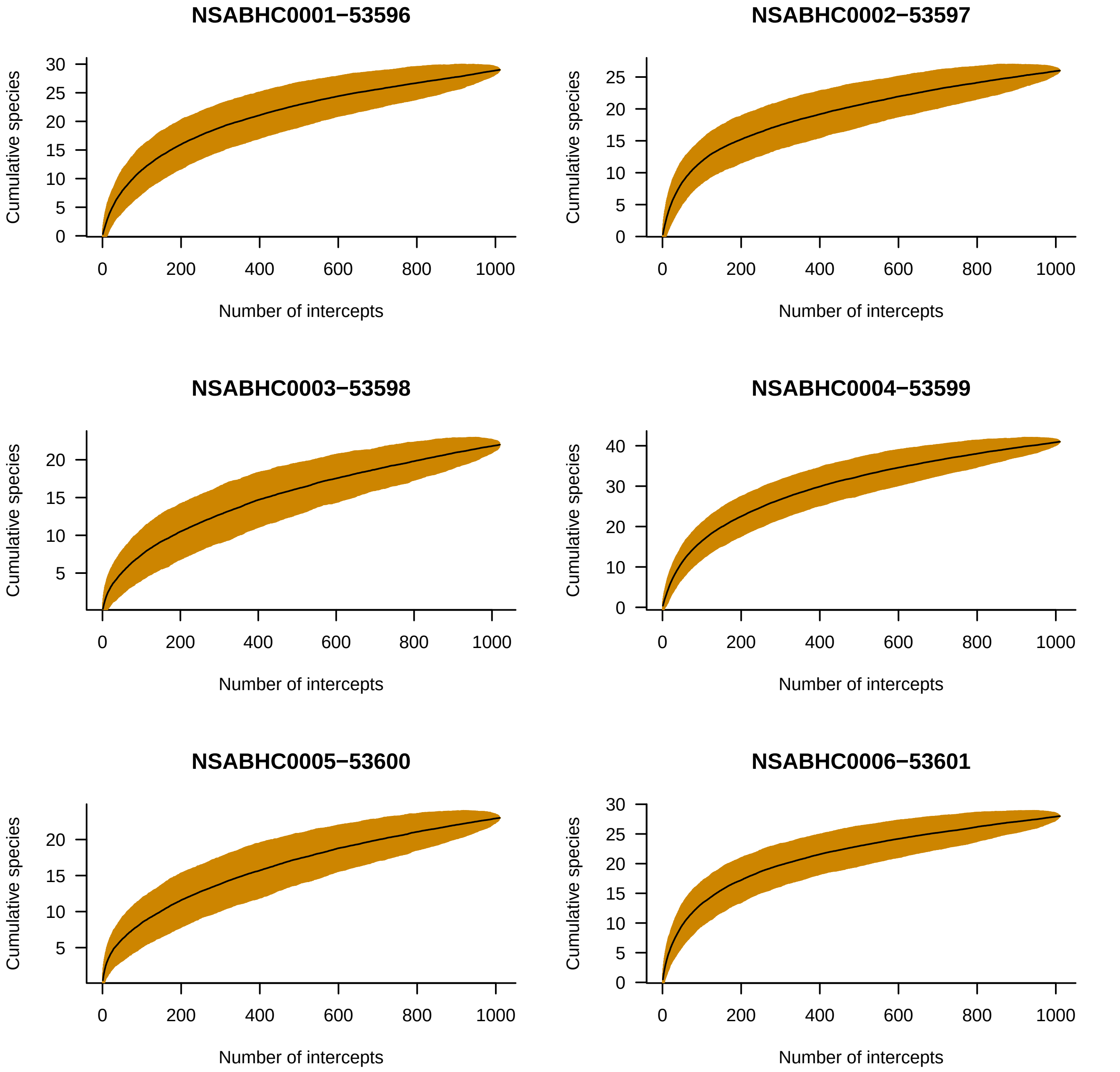
NSABHC0001−53596
Cumulative species
Number of intercepts
NSABHC0002−53597
Cumulative species
Number of intercepts
NSABHC0003−53598
Cumulative species
Number of intercepts
NSABHC0004−53599
Cumulative species
Number of intercepts
NSABHC0005−53600
Cumulative species
Number of intercepts
NSABHC0006−53601
Cumulative species
Number of intercepts

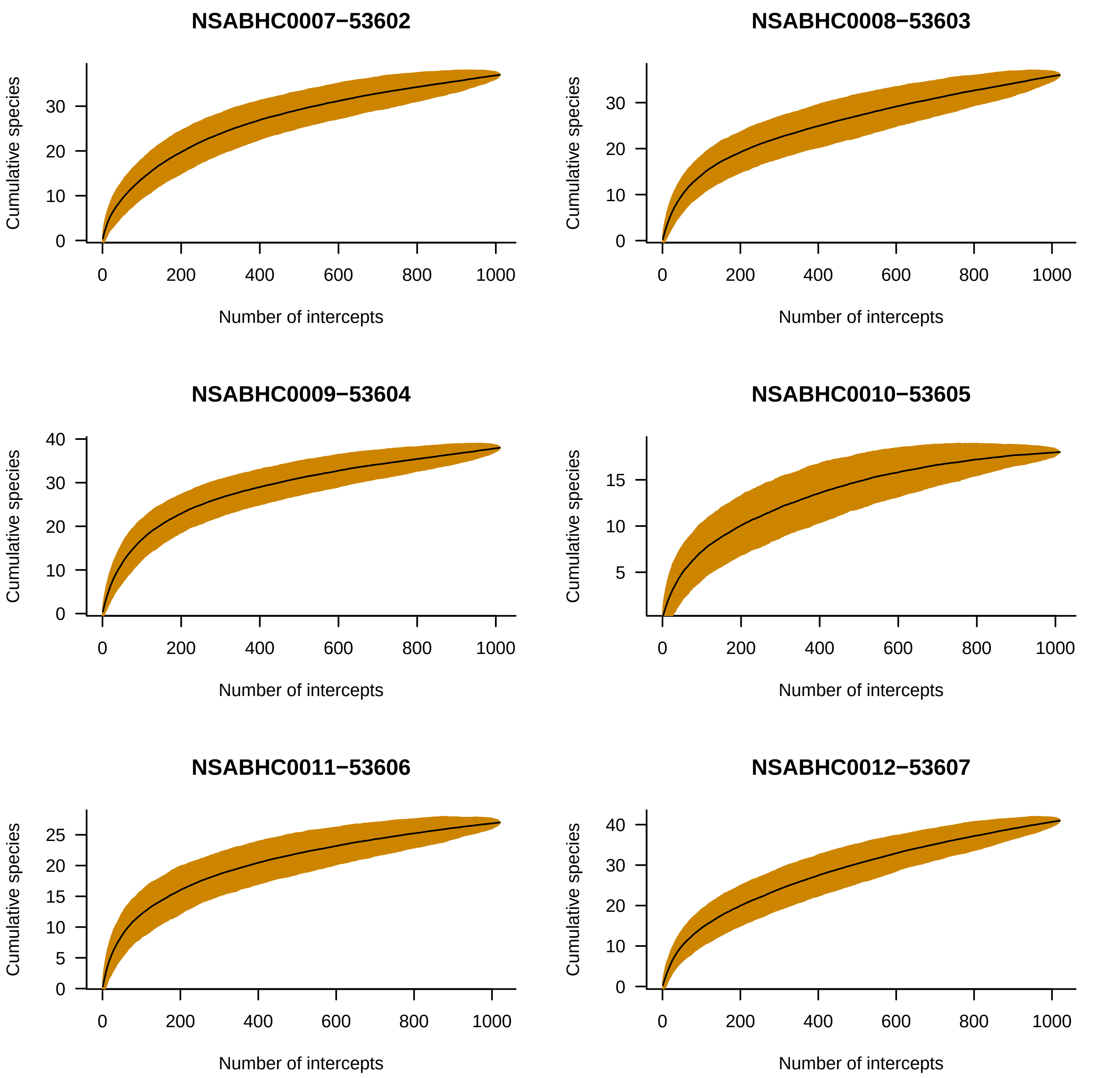
NSABHC0007−53602
NSABHC0008−53603
NSABHC0009−53604
NSABHC0010−53605
NSABHC0011−53606
NSABHC0012−53607
Cumulative species
Number of intercepts
0
200
400
600
800
1000
0
5
10
15
20
25
30
40

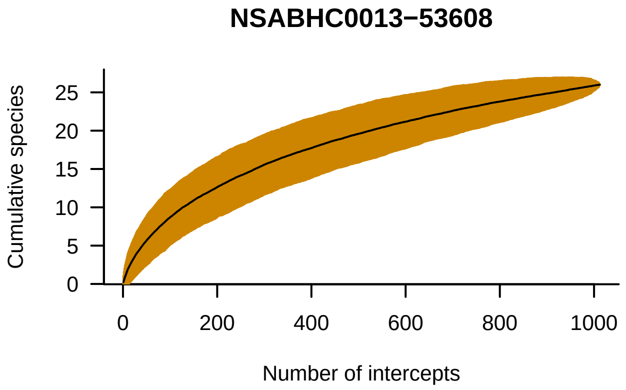
NSABHC0013−53608
Cumulative species
0
5
10
15
20
25
0
200
400
600
800
1000
Number of intercepts

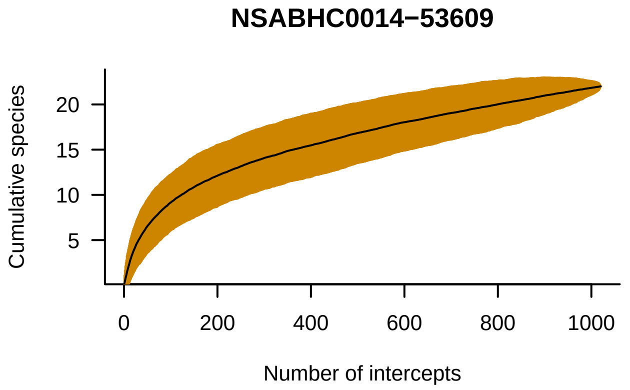
NSABHC0014−53609
Cumulative species
5
10
15
20
0
200
400
600
800
1000
Number of intercepts

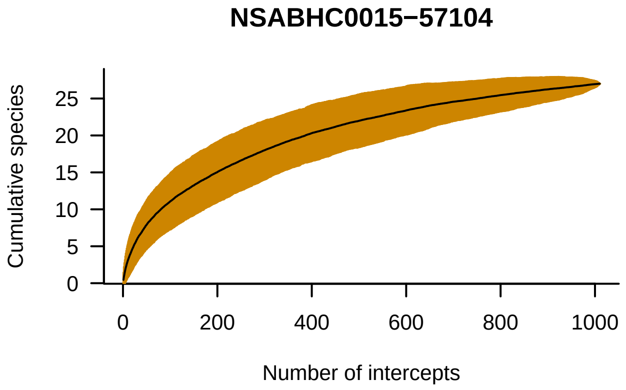
NSABHC0015−57104
Cumulative species
0
5
10
15
20
25
0
200
400
600
800
1000
Number of intercepts

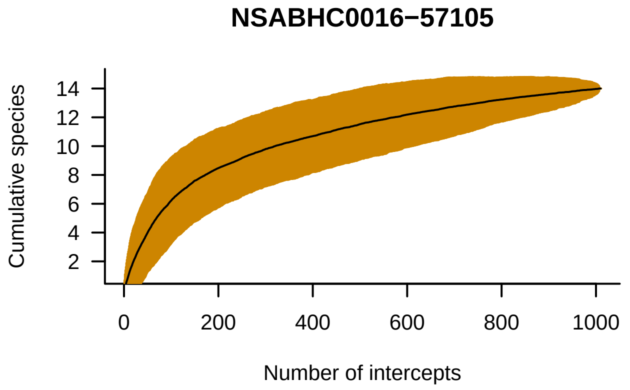
NSABHC0016−57105
Cumulative species
2
4
6
8
10
12
14
0
200
400
600
800
1000
Number of intercepts

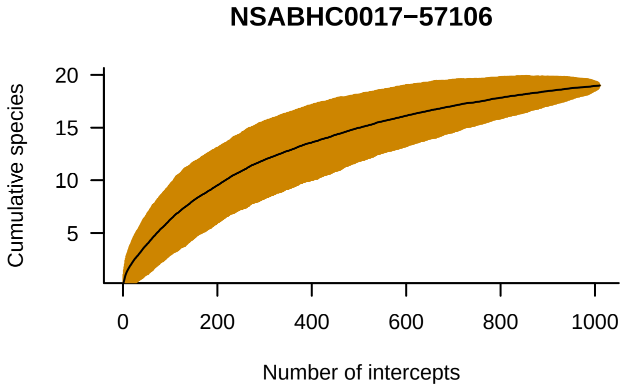
NSABHC0017−57106
Cumulative species
5
10
15
20
0
200
400
600
800
1000
Number of intercepts

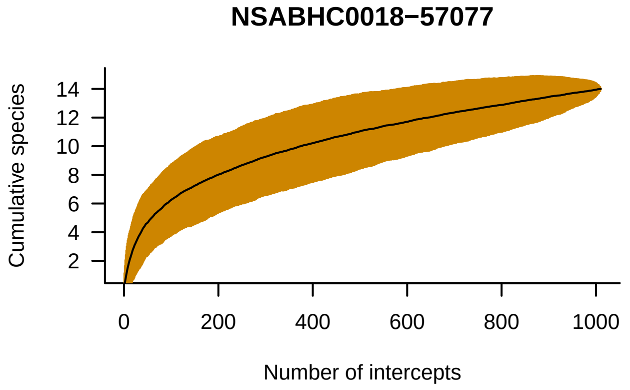
NSABHC0018−57077
Cumulative species
2
4
6
8
10
12
14
0
200
400
600
800
1000
Number of intercepts

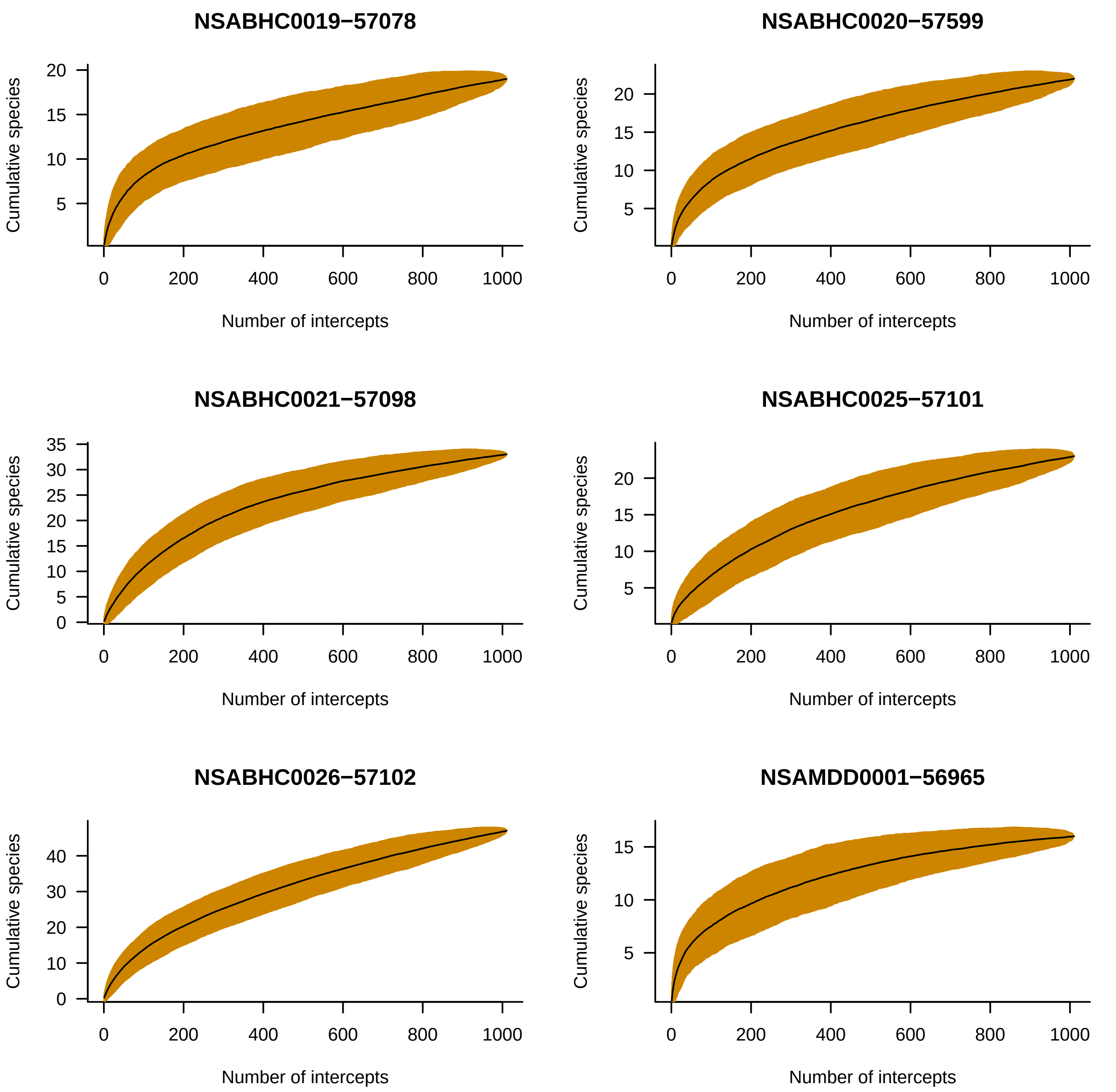
NSABHC0019−57078
Cumulative species
20
15
10
5
0
200
400
600
800
1000
Number of intercepts
NSABHC0020−57599
Cumulative species
20
15
10
5
0
200
400
600
800
1000
Number of intercepts
NSABHC0021−57098
Cumulative species
35
30
25
20
15
10
5
0
0
200
400
600
800
1000
Number of intercepts
NSABHC0025−57101
Cumulative species
20
15
10
5
0
200
400
600
800
1000
Number of intercepts
NSABHC0026−57102
Cumulative species
40
30
20
10
0
0
200
400
600
800
1000
Number of intercepts
NSAMDD0001−56965
Cumulative species
15
10
5
0
200
400
600
800
1000
Number of intercepts

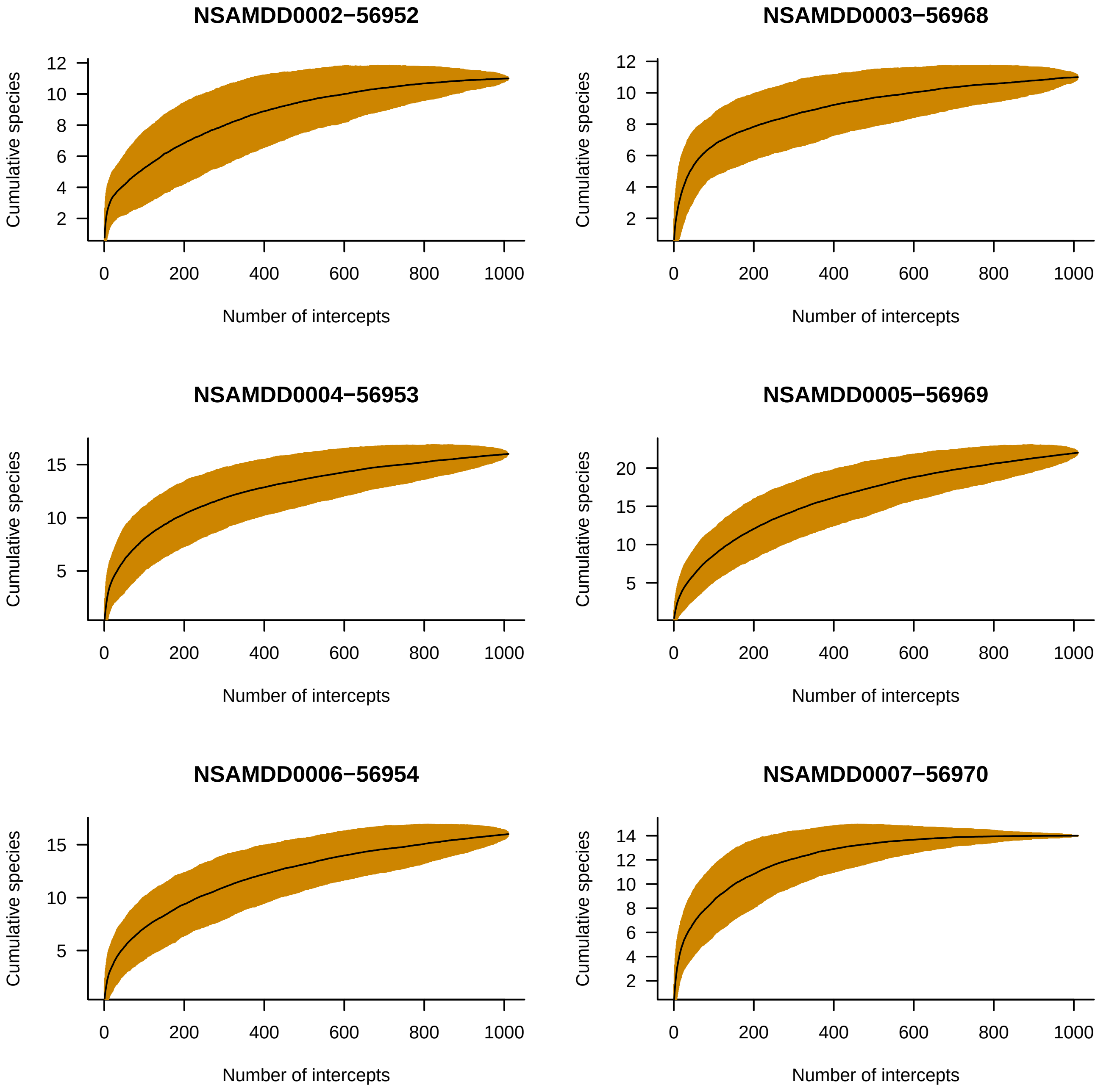
NSAMDD0002−56952
Cumulative species
Number of intercepts
NSAMDD0003−56968
Cumulative species
Number of intercepts
NSAMDD0004−56953
Cumulative species
Number of intercepts
NSAMDD0005−56969
Cumulative species
Number of intercepts
NSAMDD0006−56954
Cumulative species
Number of intercepts
NSAMDD0007−56970
Cumulative species
Number of intercepts
0
200
400
600
800
1000

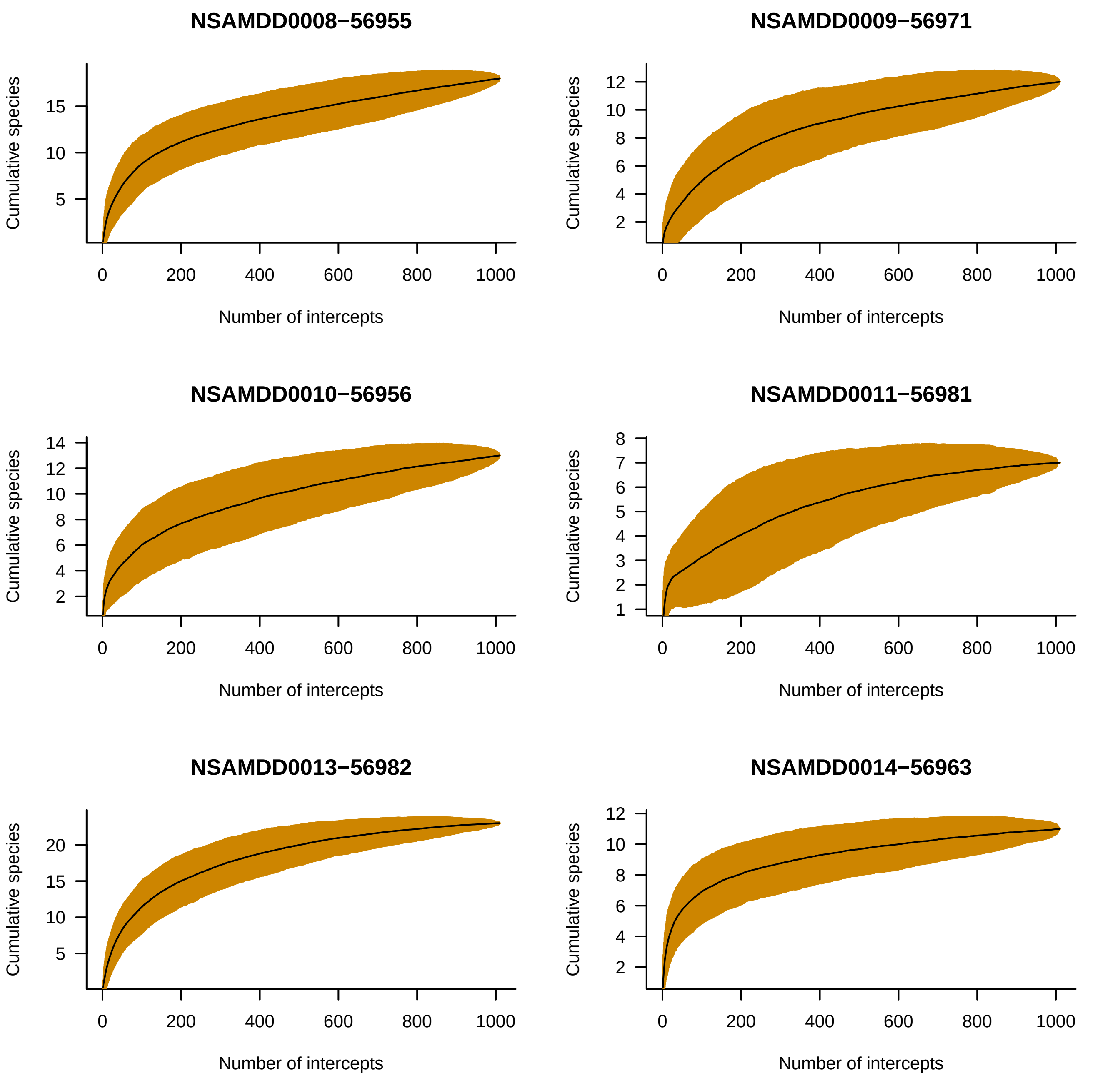
NSAMDD0008−56955
NSAMDD0009−56971
NSAMDD0010−56956
NSAMDD0011−56981
NSAMDD0013−56982
NSAMDD0014−56963
Cumulative species
Number of intercepts
0
200
400
600
800
1000

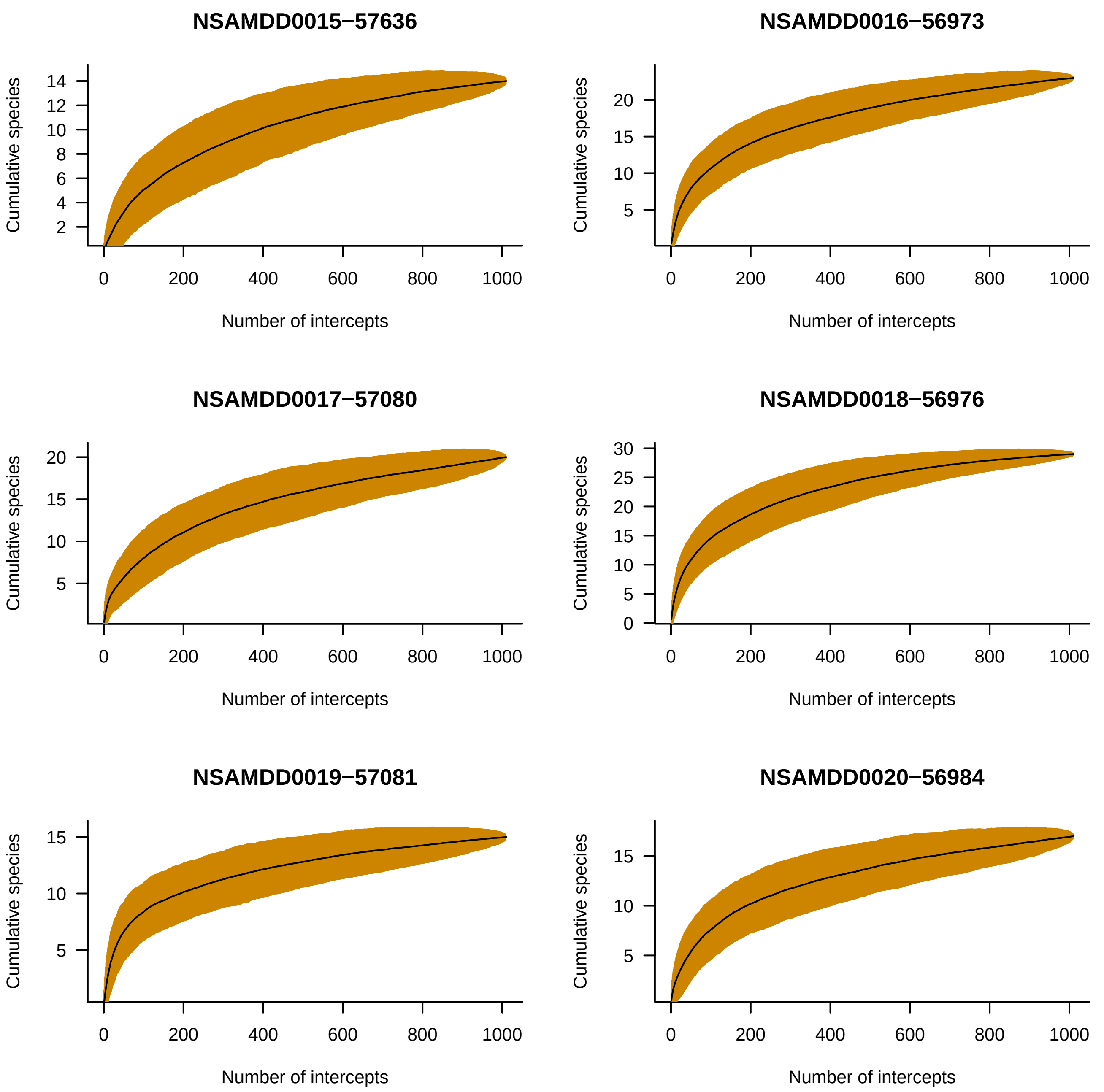
NSAMDD0015−57636
Cumulative species
2 4 6 8 10 12 14
0 200 400 600 800 1000
Number of intercepts
NSAMDD0016−56973
Cumulative species
5 10 15 20
0 200 400 600 800 1000
Number of intercepts
NSAMDD0017−57080
Cumulative species
5 10 15 20
0 200 400 600 800 1000
Number of intercepts
NSAMDD0018−56976
Cumulative species
0 5 10 15 20 25 30
0 200 400 600 800 1000
Number of intercepts
NSAMDD0019−57081
Cumulative species
5 10 15
0 200 400 600 800 1000
Number of intercepts
NSAMDD0020−56984
Cumulative species
5 10 15
0 200 400 600 800 1000
Number of intercepts

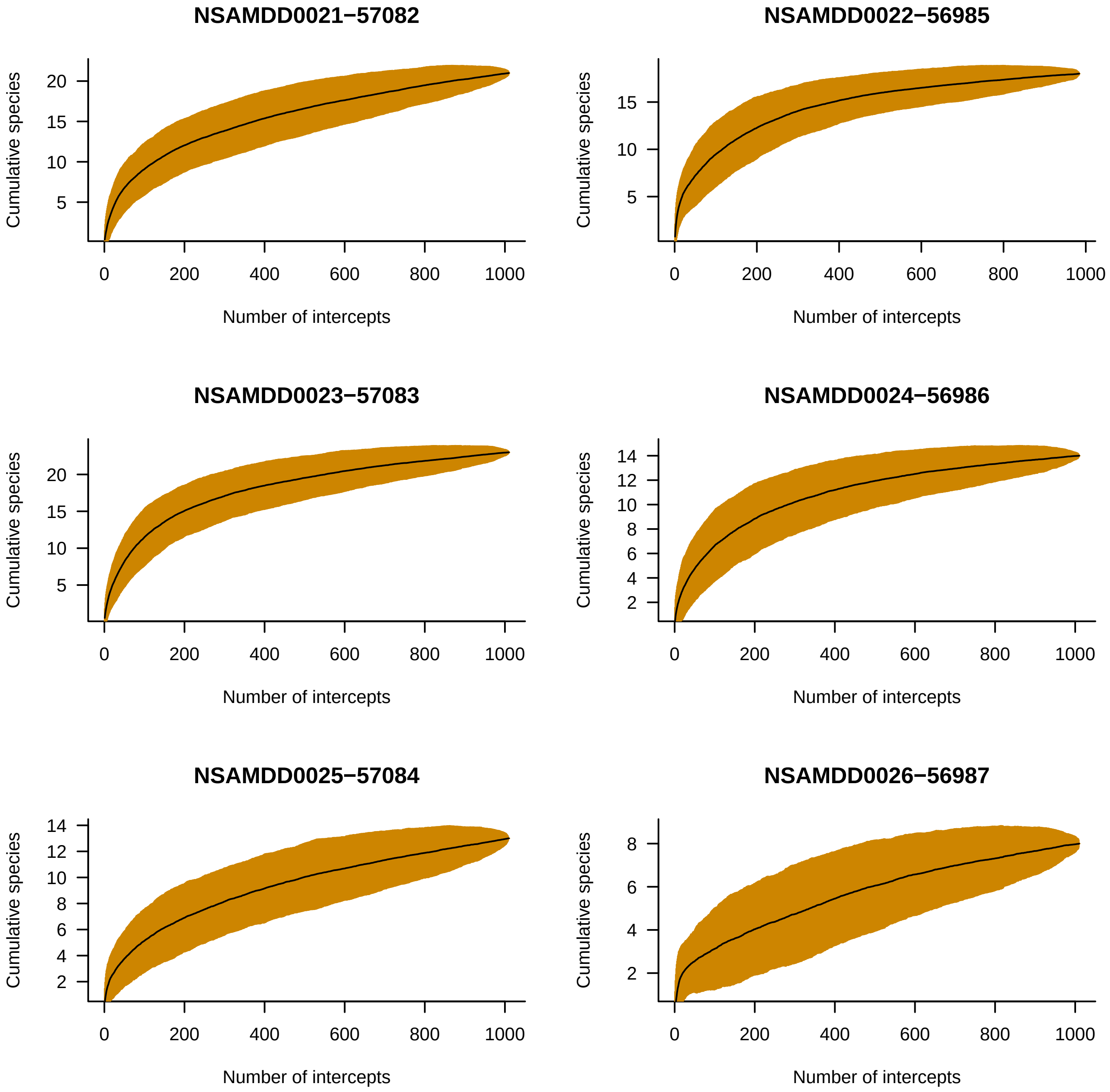
NSAMDD0021−57082
NSAMDD0022−56985
NSAMDD0023−57083
NSAMDD0024−56986
NSAMDD0025−57084
NSAMDD0026−56987
Cumulative species
Number of intercepts
0
200
400
600
800
1000
5
10
15
20
2
4
6
8
12
14

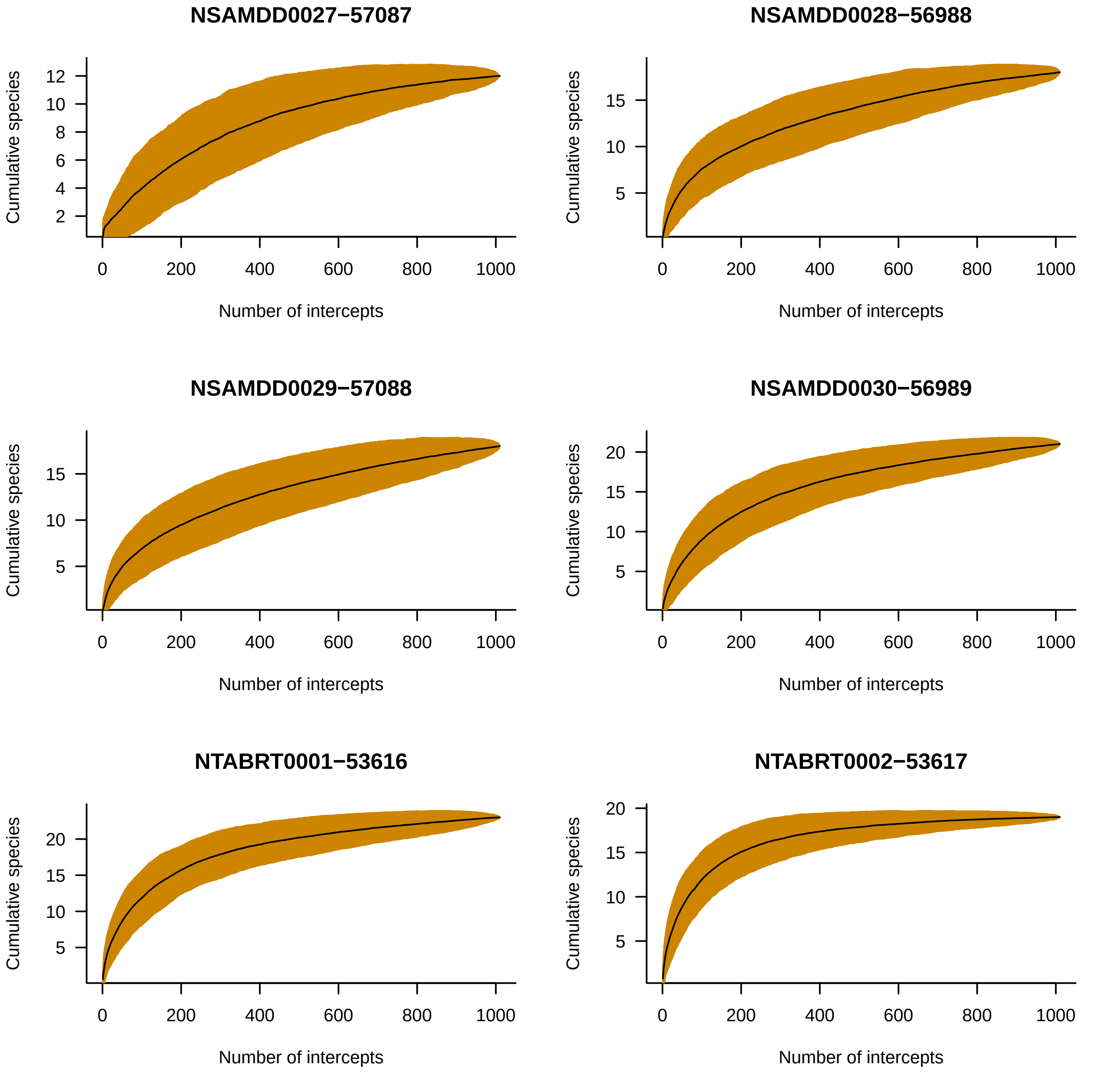

NSAMDD0027−57087
NSAMDD0028−56988
NSAMDD0029−57088
NSAMDD0030−56989
NTABRT0001−53616
NTABRT0002−53617
Cumulative species
Number of intercepts
0
200
400
600
800
1000

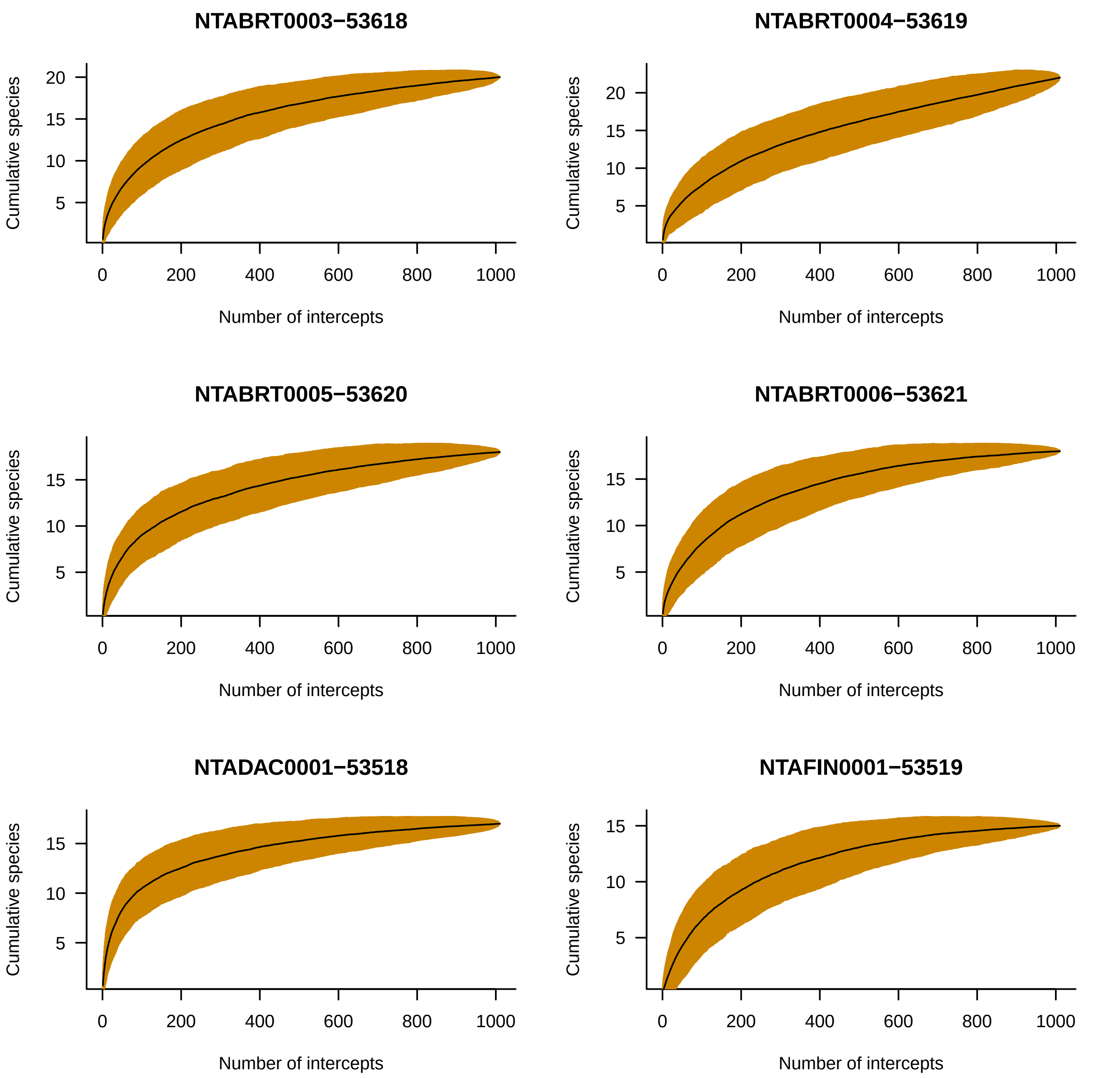
NTABRT0003−53618
NTABRT0004−53619
NTABRT0005−53620
NTABRT0006−53621
NTADAC0001−53518
NTAFIN0001−53519
Cumulative species
Number of intercepts
0
200
400
600
800
1000
5
10
15
20

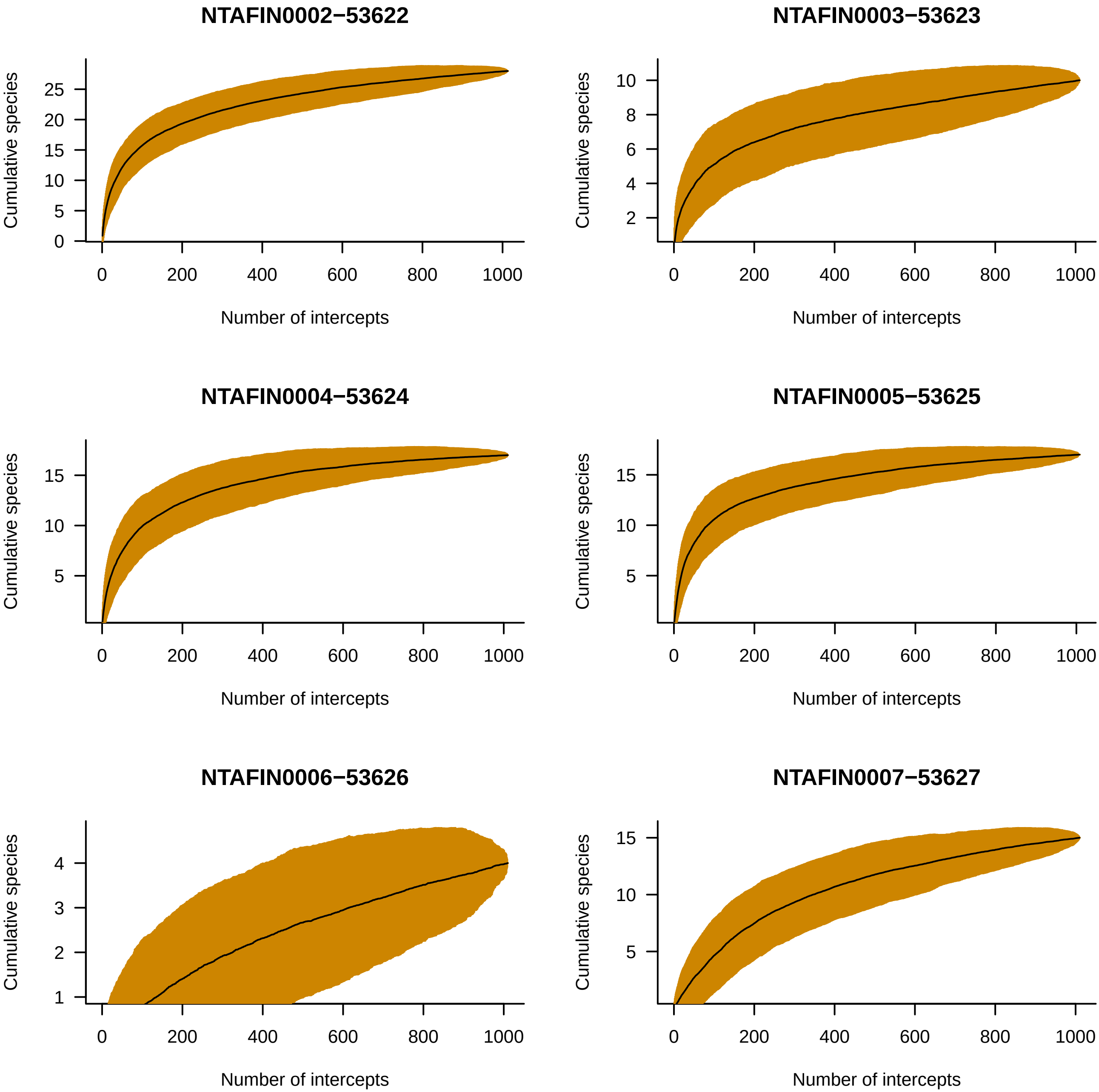

NTAFIN0002−53622
NTAFIN0003−53623
NTAFIN0004−53624
NTAFIN0005−53625
NTAFIN0006−53626
NTAFIN0007−53627
Cumulative species
Number of intercepts
0
200
400
600
800
1000

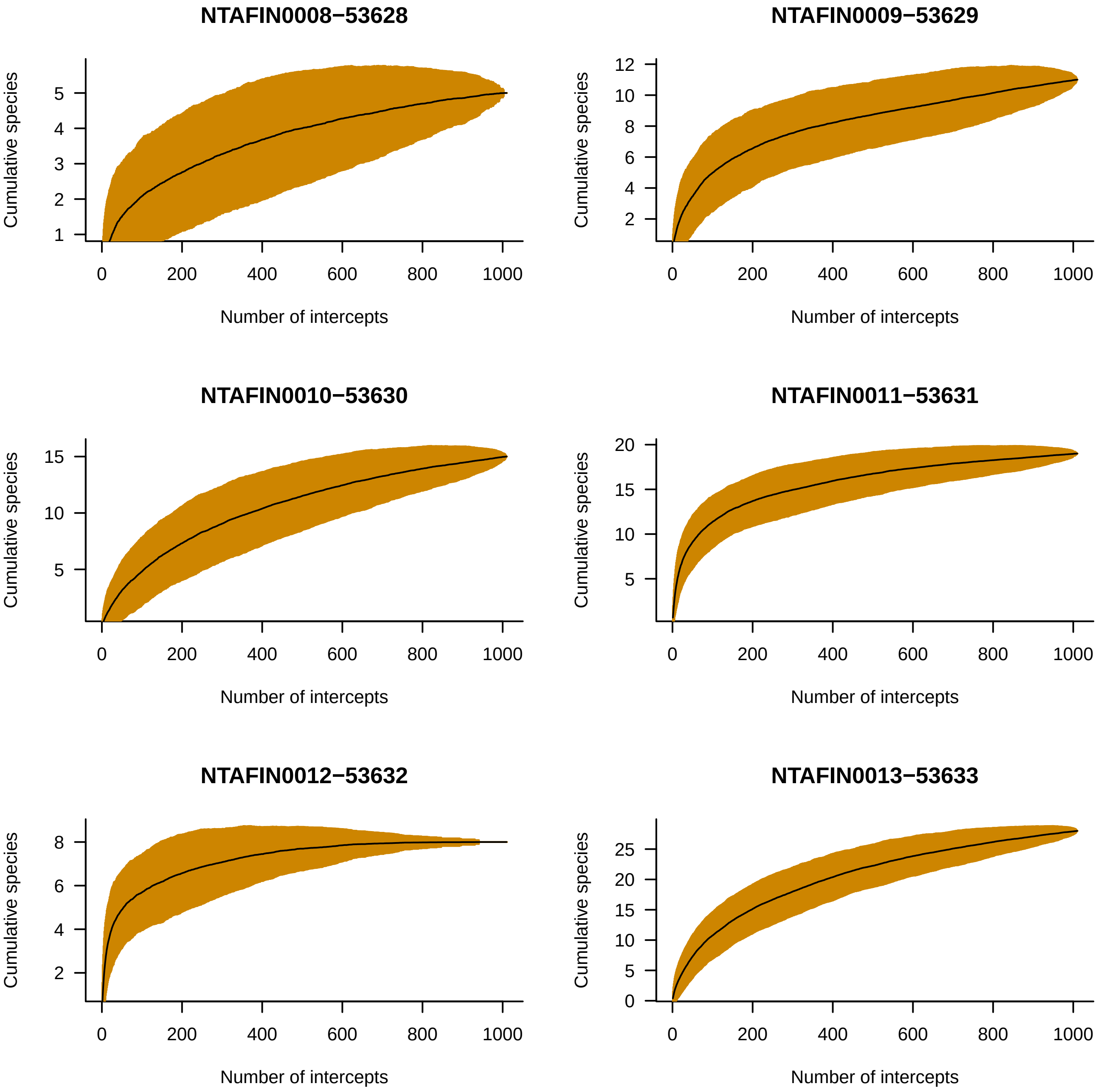

NTAFIN0008−53628
NTAFIN0009−53629
NTAFIN0010−53630
NTAFIN0011−53631
NTAFIN0012−53632
NTAFIN0013−53633
Cumulative species
Number of intercepts
0
200
400
600
800
1000

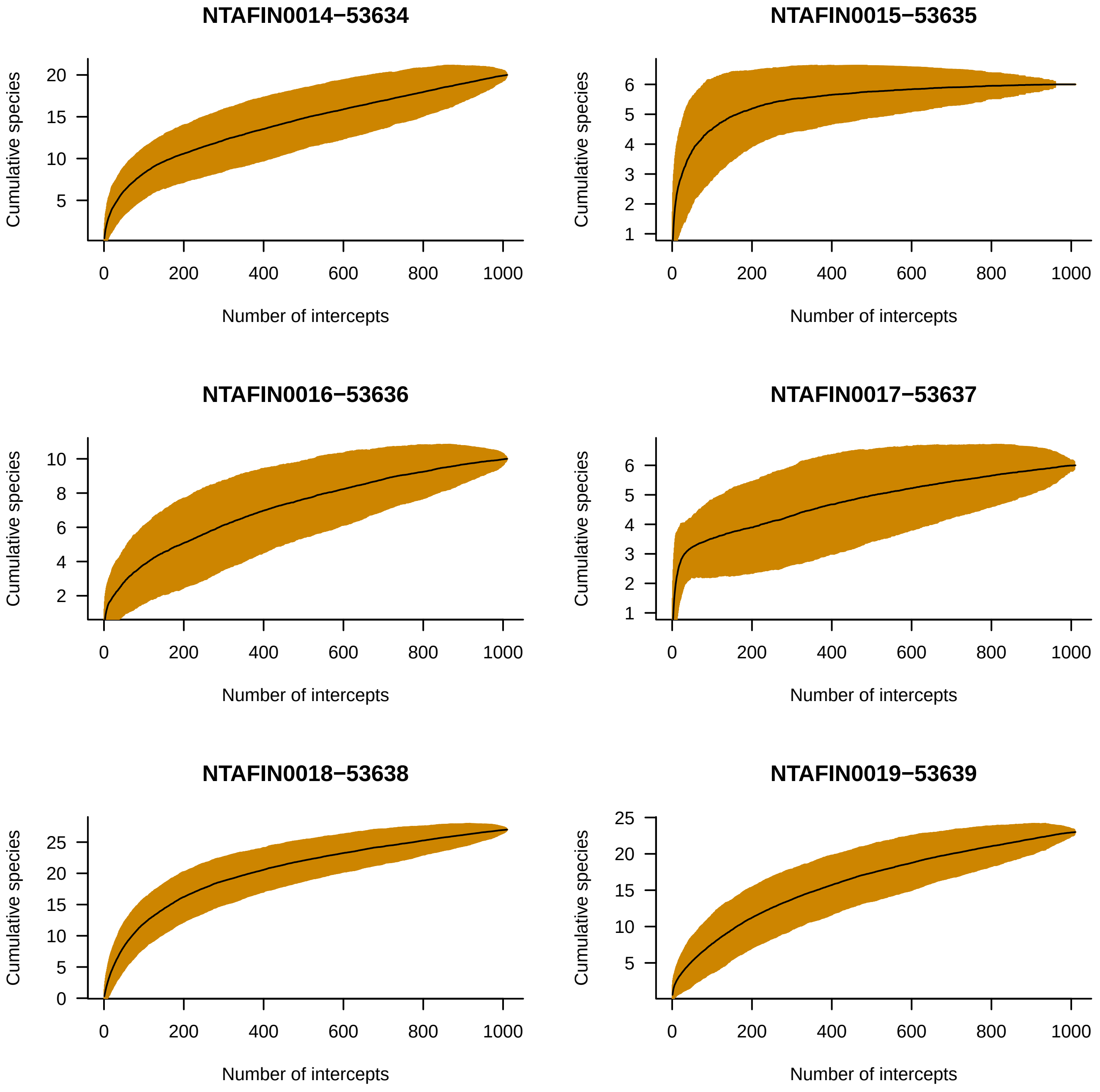
NTAFIN0014−53634
Cumulative species
Number of intercepts
0
200
400
600
800
1000
5
10
15
20
NTAFIN0015−53635
Cumulative species
Number of intercepts
1
2
3
4
5
6
NTAFIN0016−53636
Cumulative species
Number of intercepts
2
4
6
8
10
NTAFIN0017−53637
Cumulative species
Number of intercepts
NTAFIN0018−53638
Cumulative species
Number of intercepts
0
5
10
15
20
25
NTAFIN0019−53639
Cumulative species
Number of intercepts

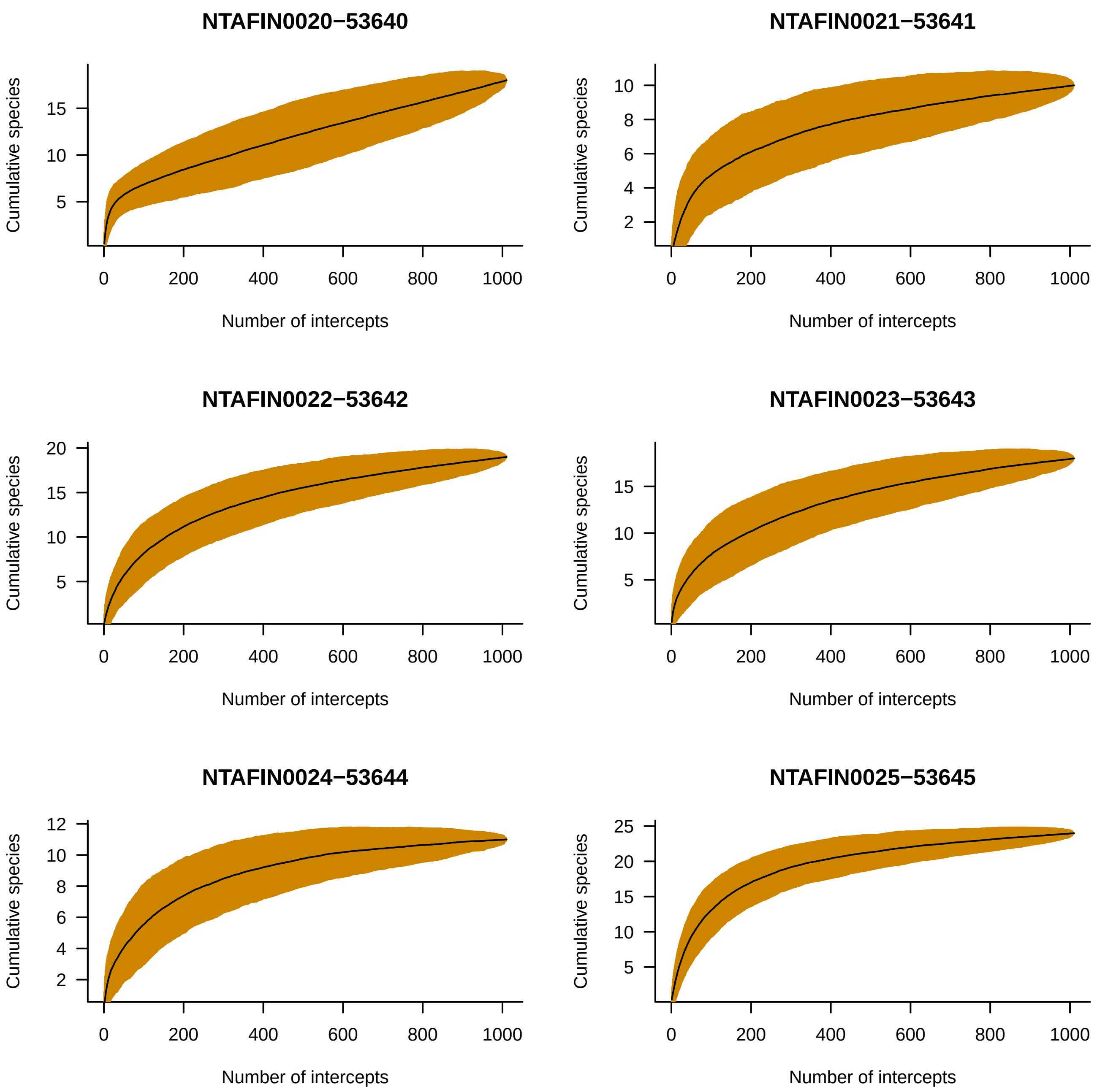

NTAFIN0020−53640
NTAFIN0021−53641
NTAFIN0022−53642
NTAFIN0023−53643
NTAFIN0024−53644
NTAFIN0025−53645
Cumulative species
Number of intercepts
0
200
400
600
800
1000

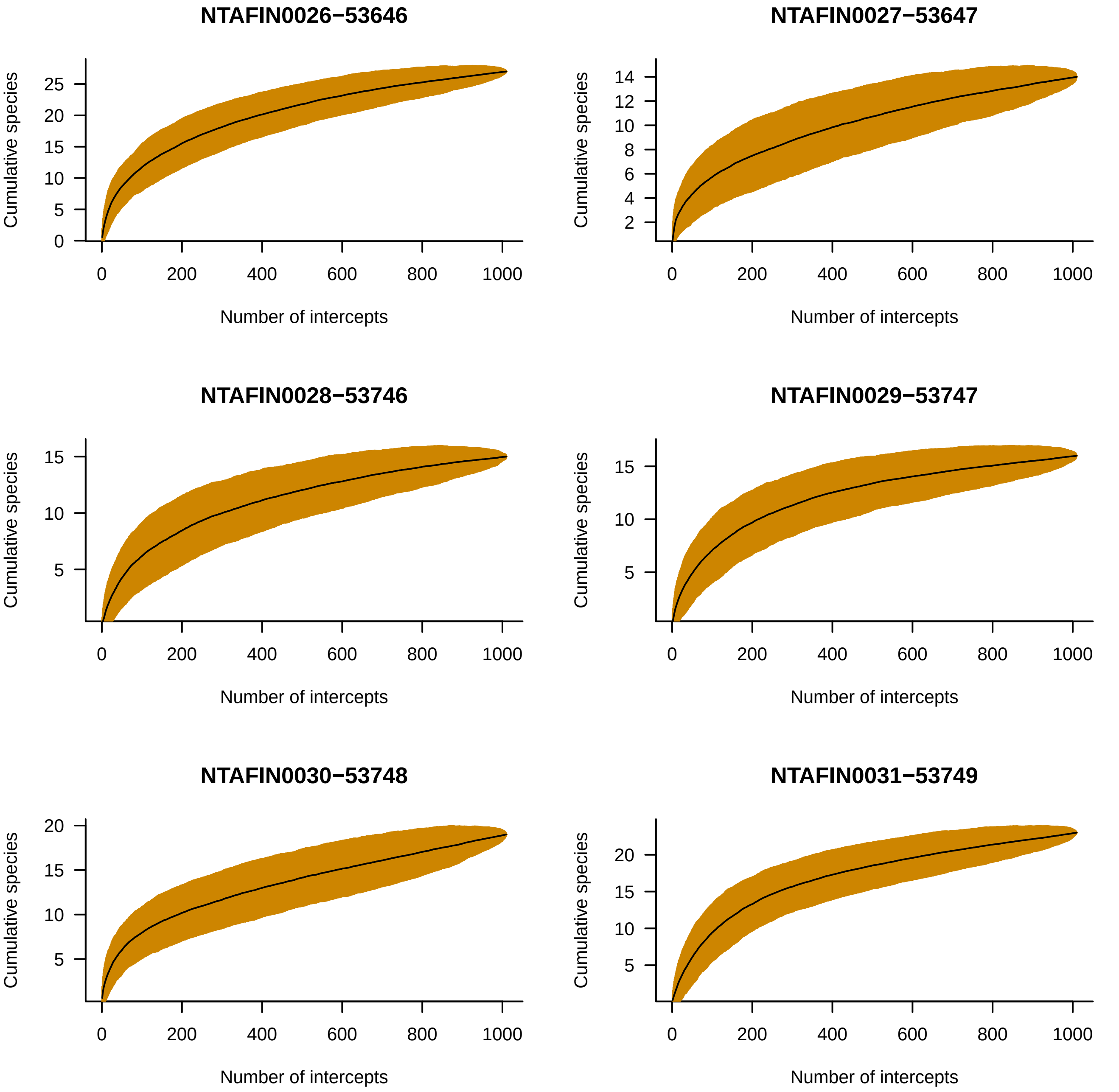
NTAFIN0026−53646
Cumulative species
Number of intercepts
NTAFIN0027−53647
Cumulative species
Number of intercepts
NTAFIN0028−53746
Cumulative species
Number of intercepts
NTAFIN0029−53747
Cumulative species
Number of intercepts
NTAFIN0030−53748
Cumulative species
Number of intercepts
NTAFIN0031−53749
Cumulative species
Number of intercepts

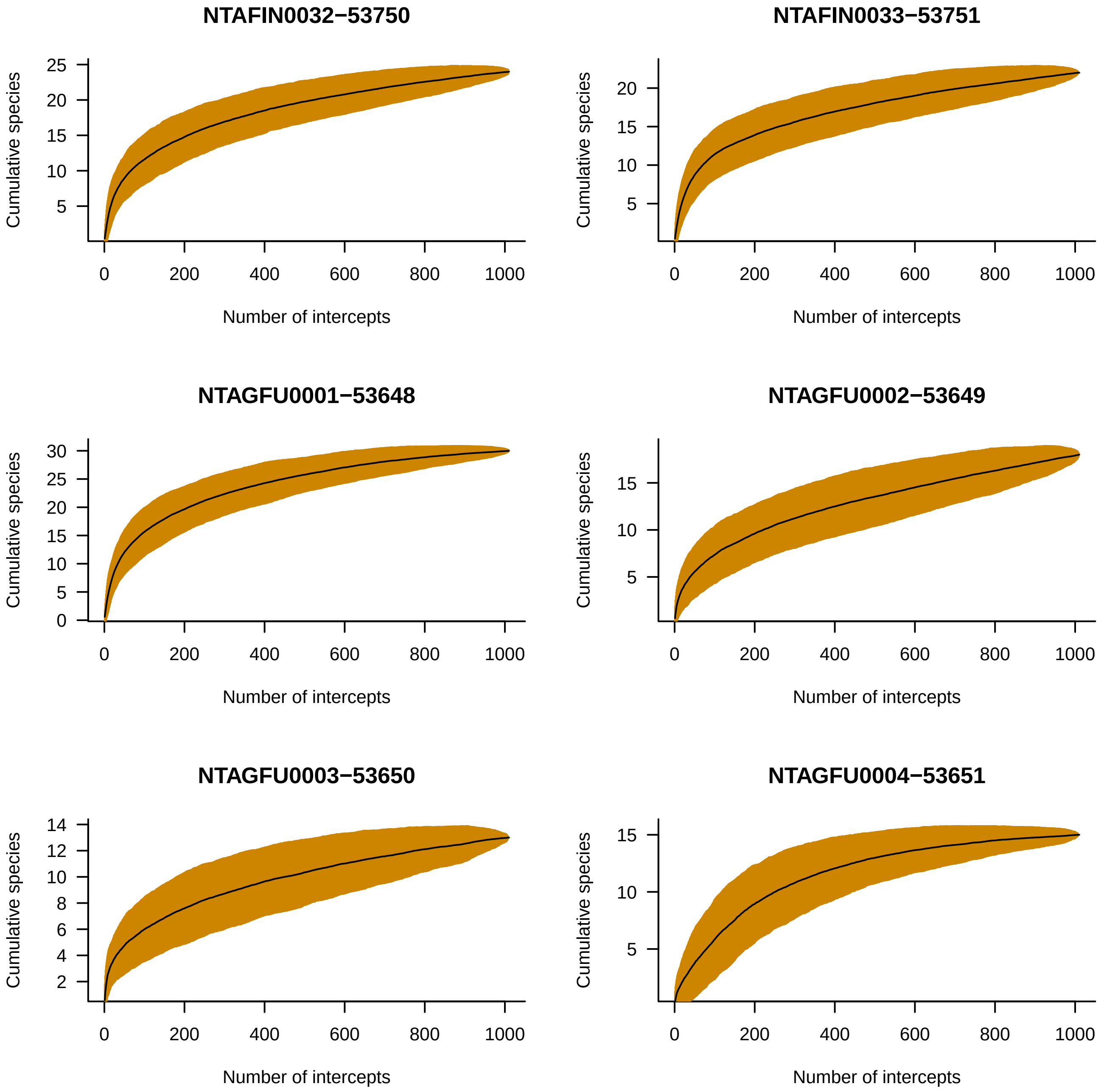
NTAFIN0032−53750
Cumulative species
25
20
15
10
5
0
200
400
600
800
1000
Number of intercepts
NTAFIN0033−53751
Cumulative species
20
15
10
5
0
200
400
600
800
1000
Number of intercepts
NTAGFU0001−53648
Cumulative species
30
25
20
15
10
5
0
0
200
400
600
800
1000
Number of intercepts
NTAGFU0002−53649
Cumulative species
15
10
5
0
200
400
600
800
1000
Number of intercepts
NTAGFU0003−53650
Cumulative species
14
12
10
8
6
4
2
0
200
400
600
800
1000
Number of intercepts
NTAGFU0004−53651
Cumulative species
15
10
5
0
200
400
600
800
1000
Number of intercepts

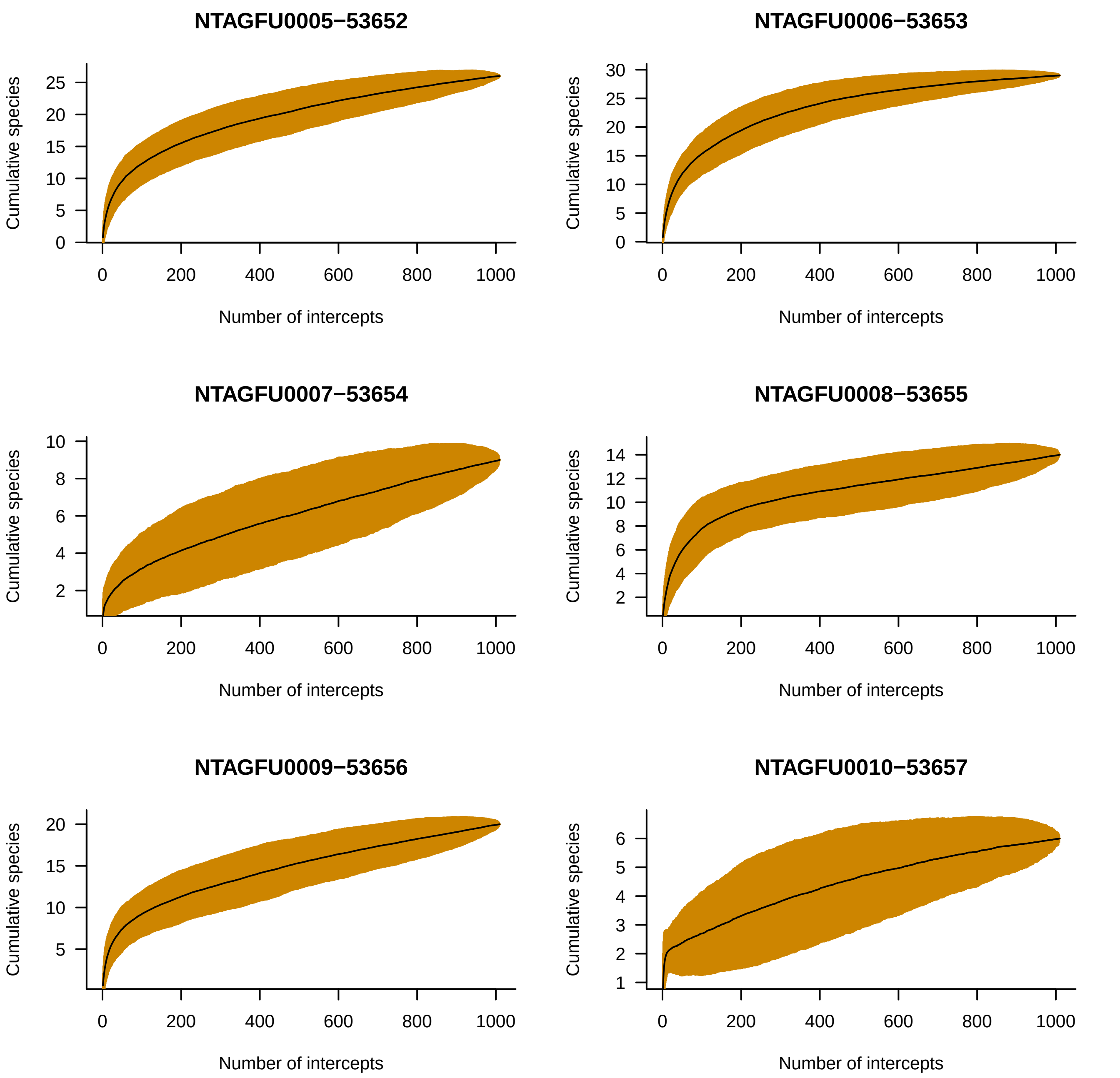
NTAGFU0005−53652
Cumulative species
Number of intercepts
NTAGFU0006−53653
Cumulative species
Number of intercepts
NTAGFU0007−53654
Cumulative species
Number of intercepts
NTAGFU0008−53655
Cumulative species
Number of intercepts
NTAGFU0009−53656
Cumulative species
Number of intercepts
NTAGFU0010−53657
Cumulative species
Number of intercepts

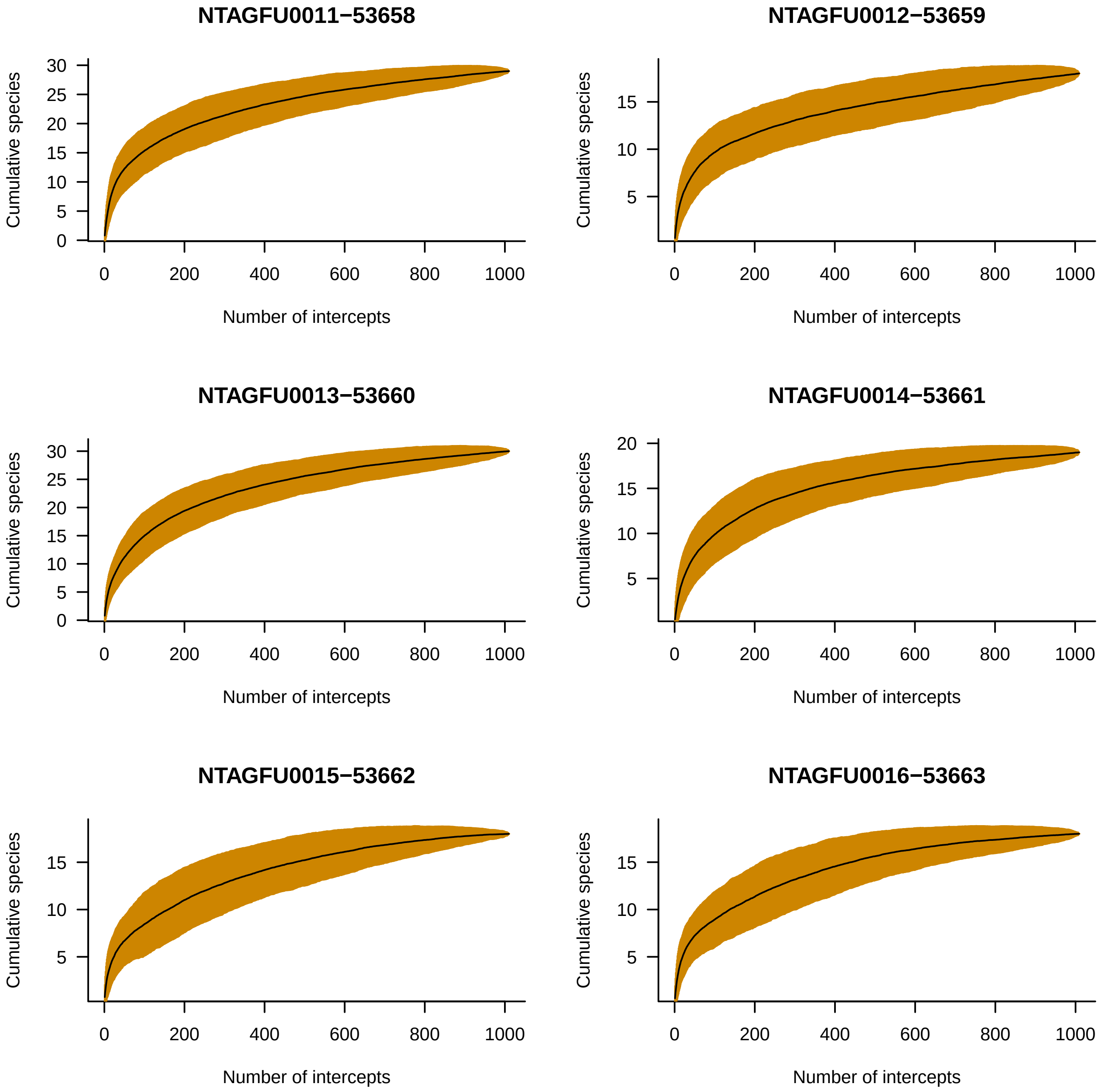
NTAGFU0011−53658
NTAGFU0012−53659
NTAGFU0013−53660
NTAGFU0014−53661
NTAGFU0015−53662
NTAGFU0016−53663
Cumulative species
Number of intercepts
0
200
400
600
800
1000
0
5
10
15
20
25
30

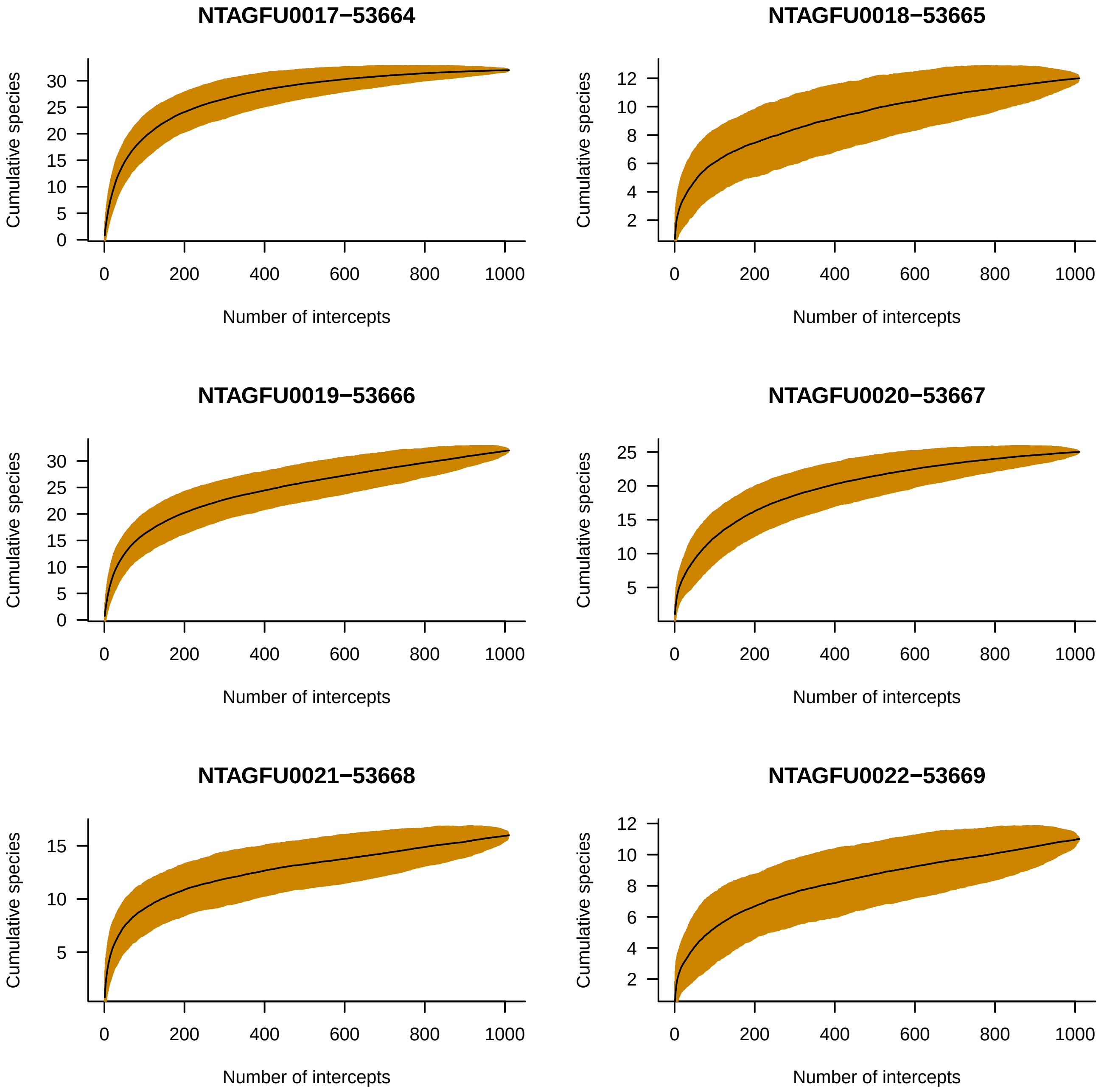
NTAGFU0017−53664
NTAGFU0018−53665
NTAGFU0019−53666
NTAGFU0020−53667
NTAGFU0021−53668
NTAGFU0022−53669
Cumulative species
Number of intercepts
0
200
400
600
800
1000

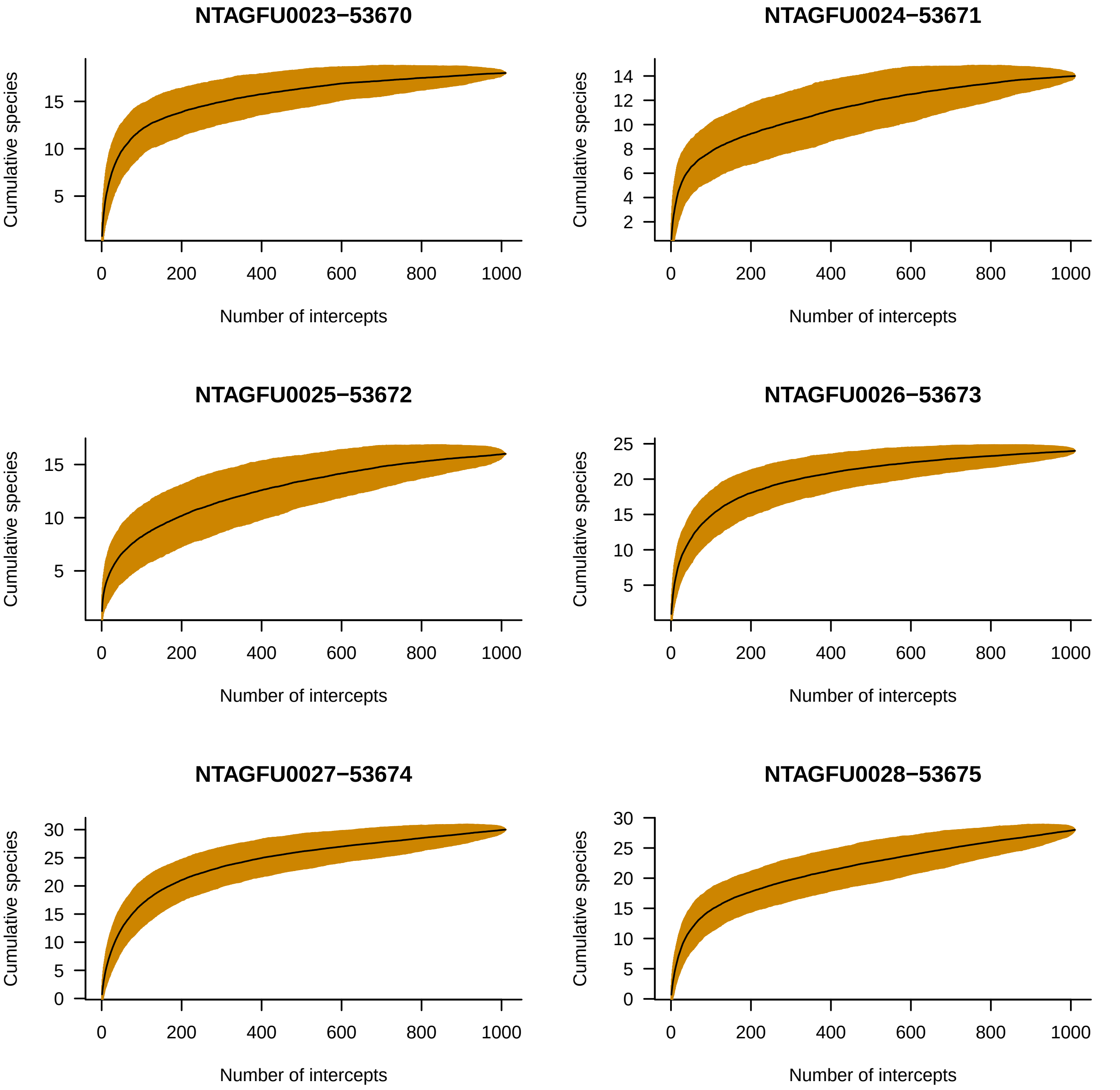
NTAGFU0023−53670
NTAGFU0024−53671
NTAGFU0025−53672
NTAGFU0026−53673
NTAGFU0027−53674
NTAGFU0028−53675
Cumulative species
Number of intercepts
0
200
400
600
800
1000

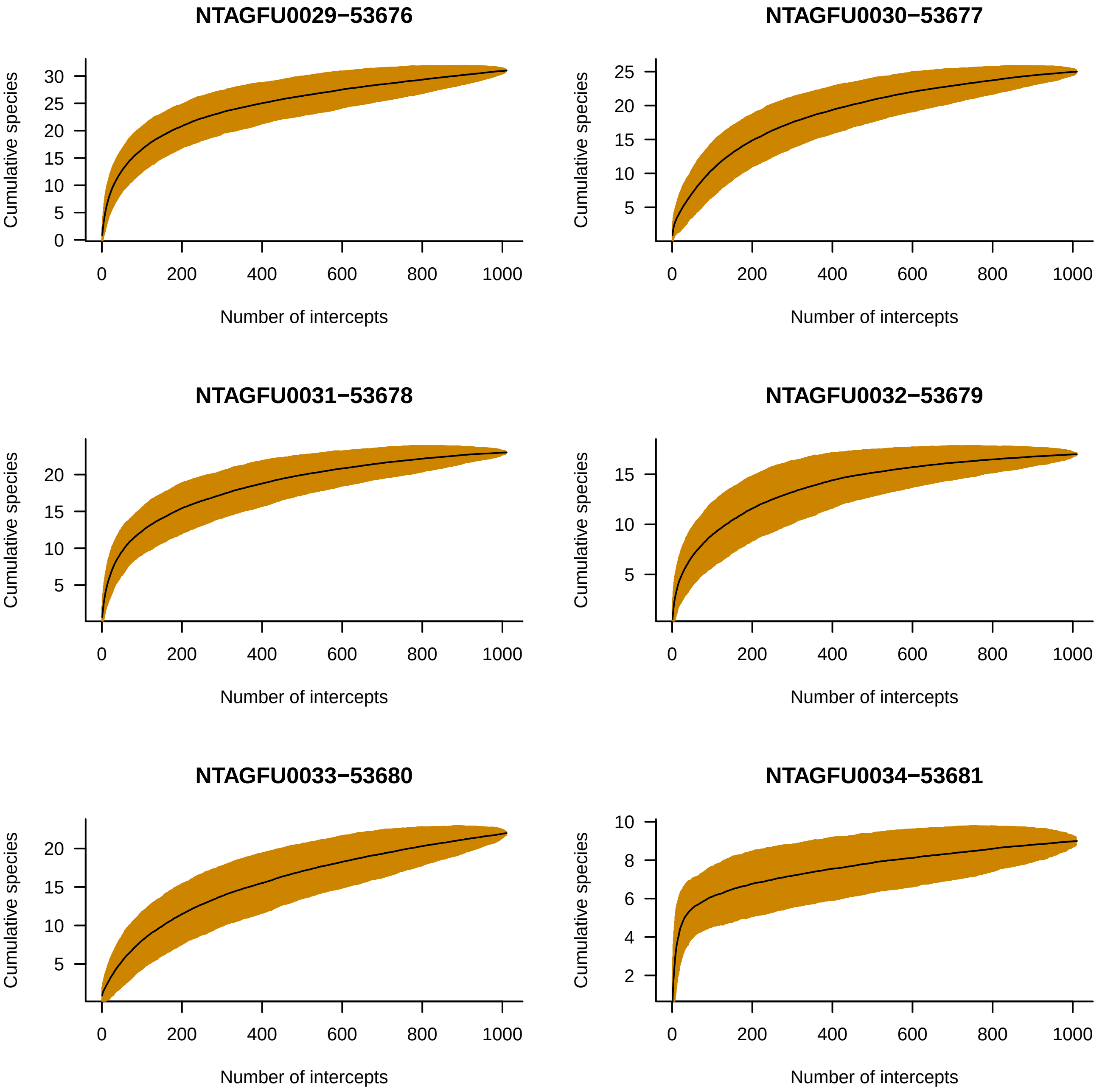
NTAGFU0029−53676
NTAGFU0030−53677
NTAGFU0031−53678
NTAGFU0032−53679
NTAGFU0033−53680
NTAGFU0034−53681
Cumulative species
Number of intercepts
0
200
400
600
800
1000

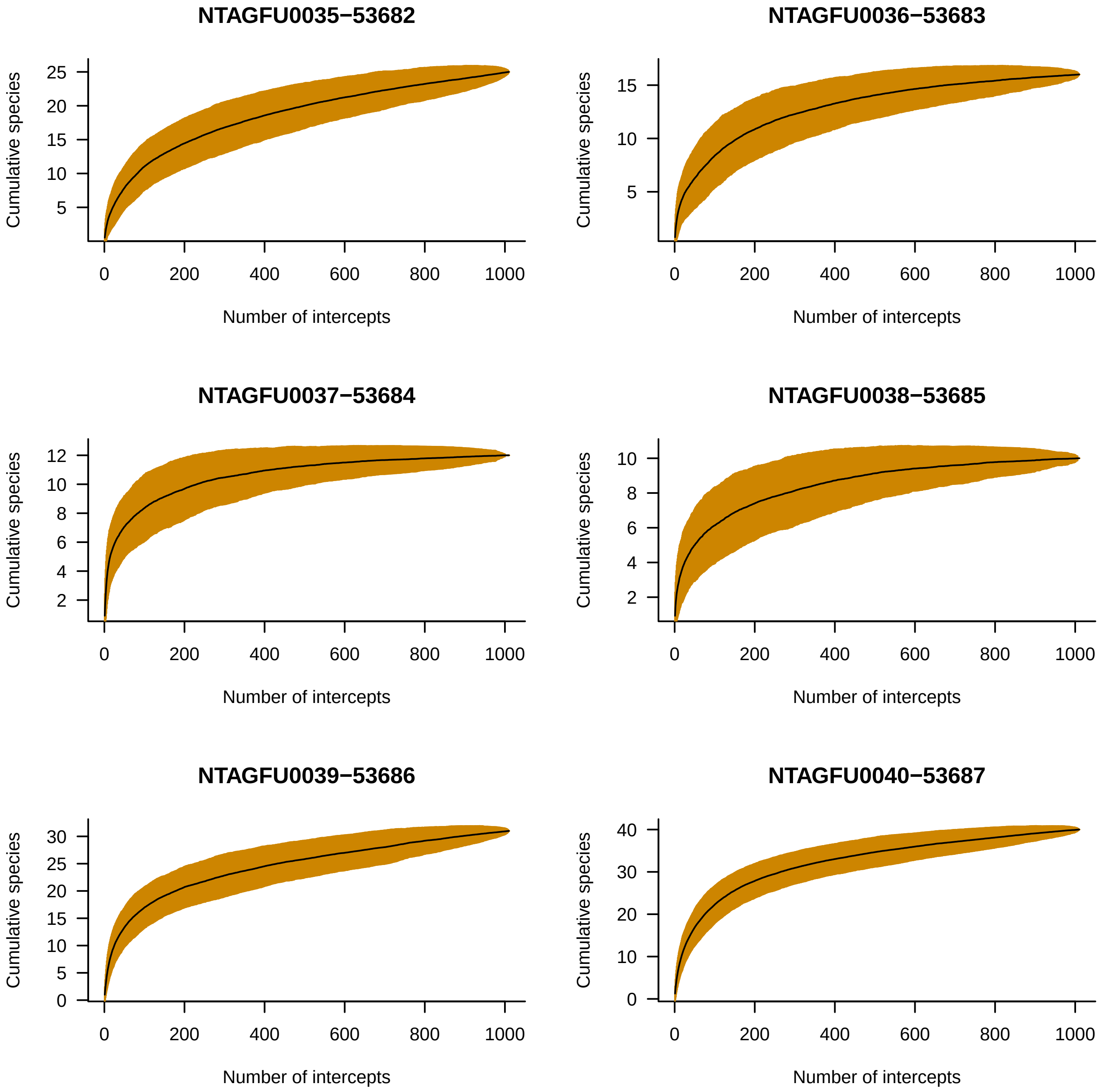
NTAGFU0035−53682
NTAGFU0036−53683
NTAGFU0037−53684
NTAGFU0038−53685
NTAGFU0039−53686
NTAGFU0040−53687
Cumulative species
Number of intercepts
0
200
400
600
800
1000
5
10
15
20
25
30
40
2
4
6
8
12

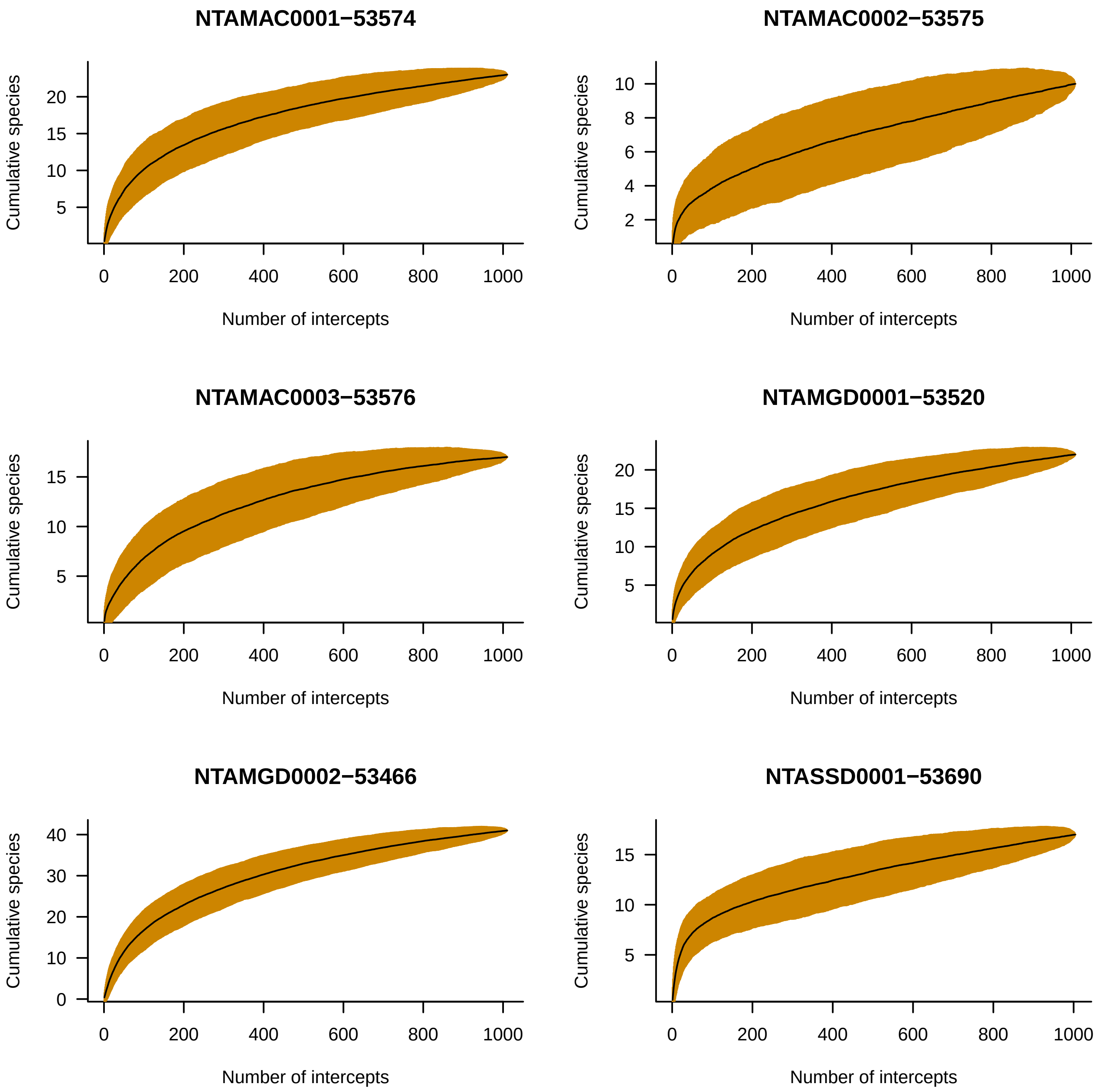
NTAMAC0001−53574
Cumulative species
5
10
15
20
0
200
400
600
800
1000
Number of intercepts
NTAMAC0002−53575
Cumulative species
2
4
6
8
10
0
200
400
600
800
1000
Number of intercepts
NTAMAC0003−53576
Cumulative species
5
10
15
0
200
400
600
800
1000
Number of intercepts
NTAMGD0001−53520
Cumulative species
5
10
15
20
0
200
400
600
800
1000
Number of intercepts
NTAMGD0002−53466
Cumulative species
0
10
20
30
40
0
200
400
600
800
1000
Number of intercepts
NTASSD0001−53690
Cumulative species
5
10
15
0
200
400
600
800
1000
Number of intercepts

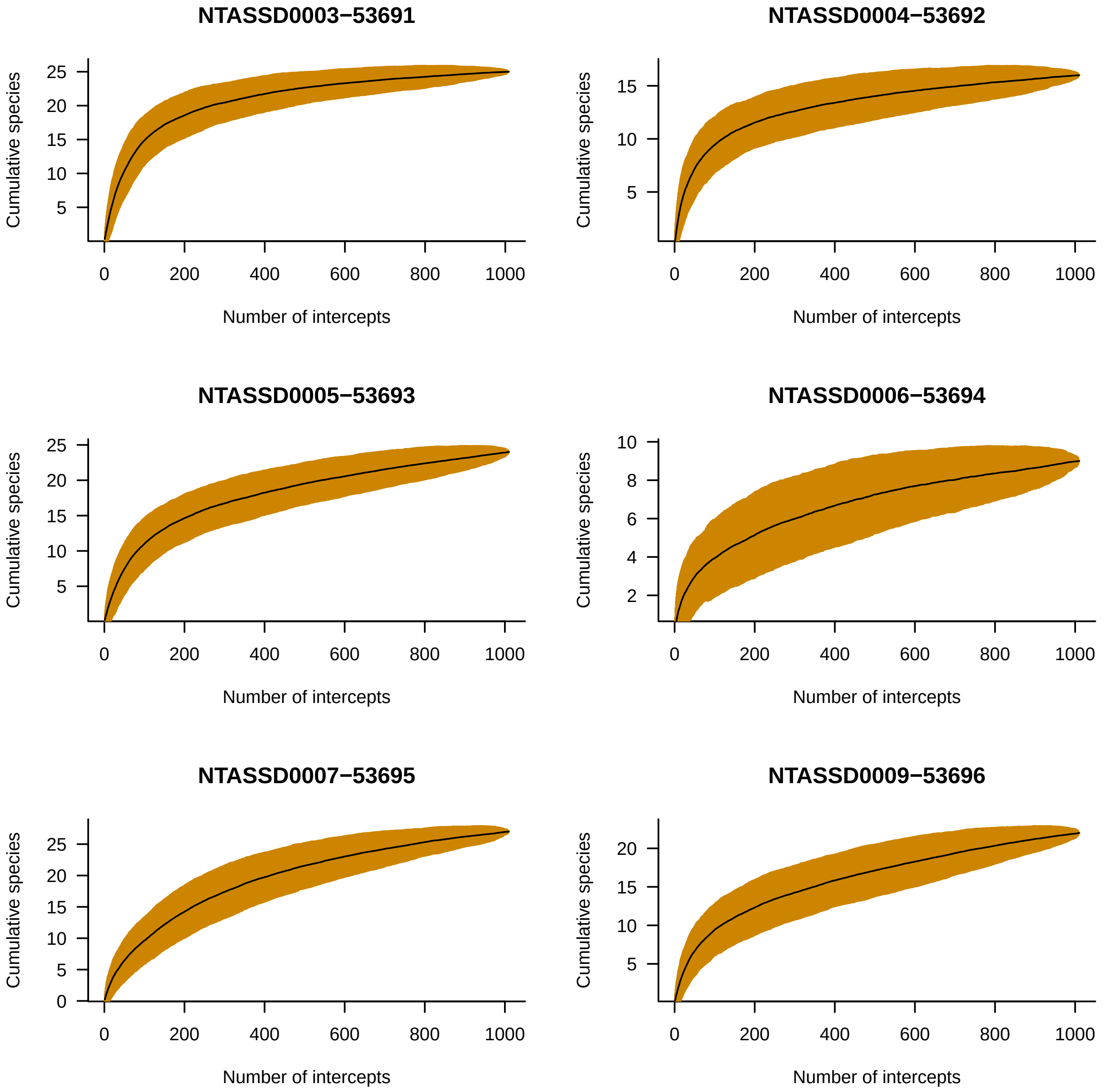
NTASSD0003−53691
NTASSD0004−53692
NTASSD0005−53693
NTASSD0006−53694
NTASSD0007−53695
NTASSD0009−53696
Cumulative species
Number of intercepts
0
200
400
600
800
1000
5
10
15
20
25
2
4
6
8

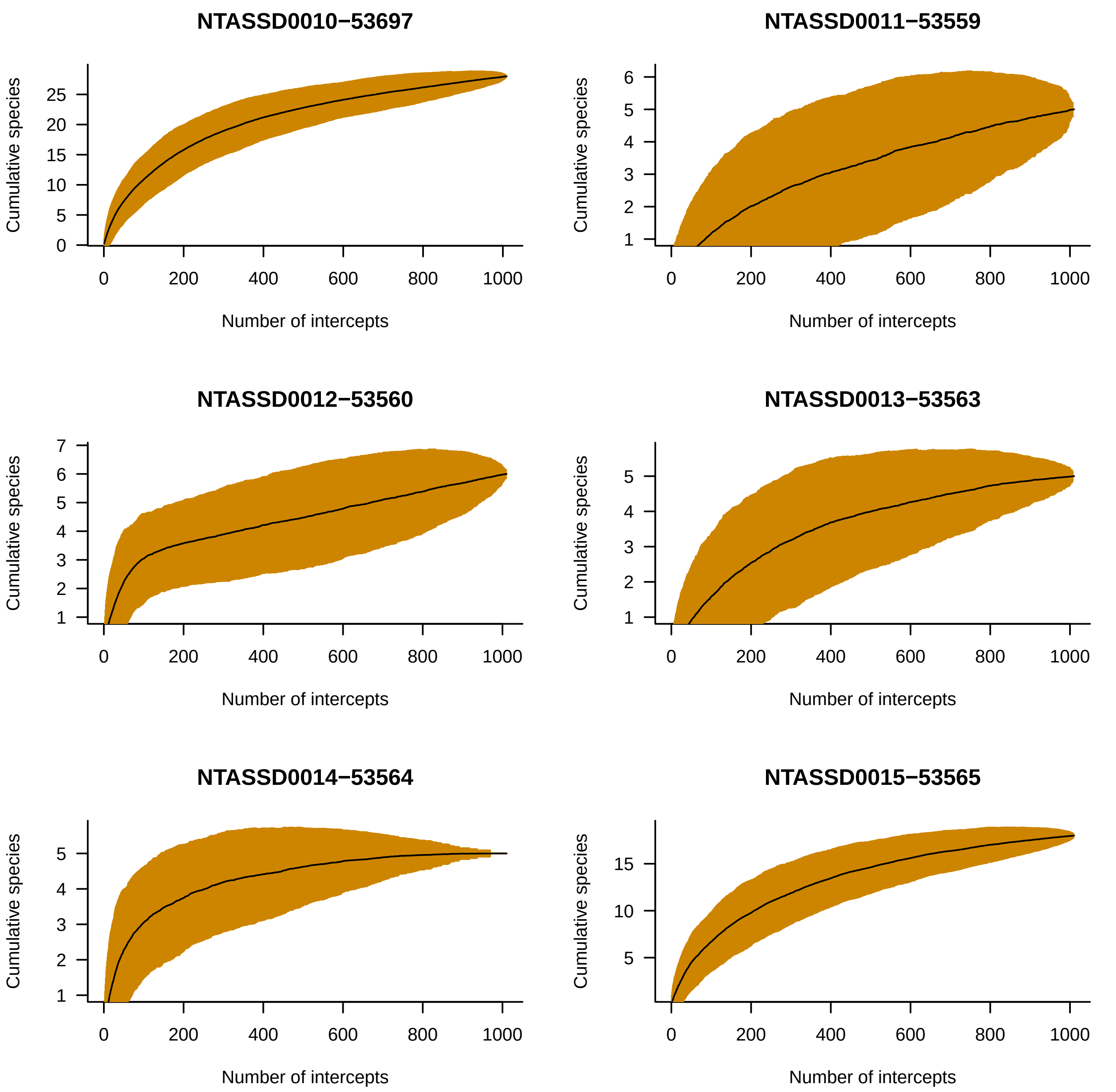
NTASSD0010−53697
Cumulative species
0
5
10
15
20
25
0
200
400
600
800
1000
Number of intercepts
NTASSD0011−53559
Cumulative species
1
2
3
4
5
6
0
200
400
600
800
1000
Number of intercepts
NTASSD0012−53560
Cumulative species
1
2
3
4
5
6
7
0
200
400
600
800
1000
Number of intercepts
NTASSD0013−53563
Cumulative species
1
2
3
4
5
0
200
400
600
800
1000
Number of intercepts
NTASSD0014−53564
Cumulative species
1
2
3
4
5
0
200
400
600
800
1000
Number of intercepts
NTASSD0015−53565
Cumulative species
5
10
15
0
200
400
600
800
1000
Number of intercepts

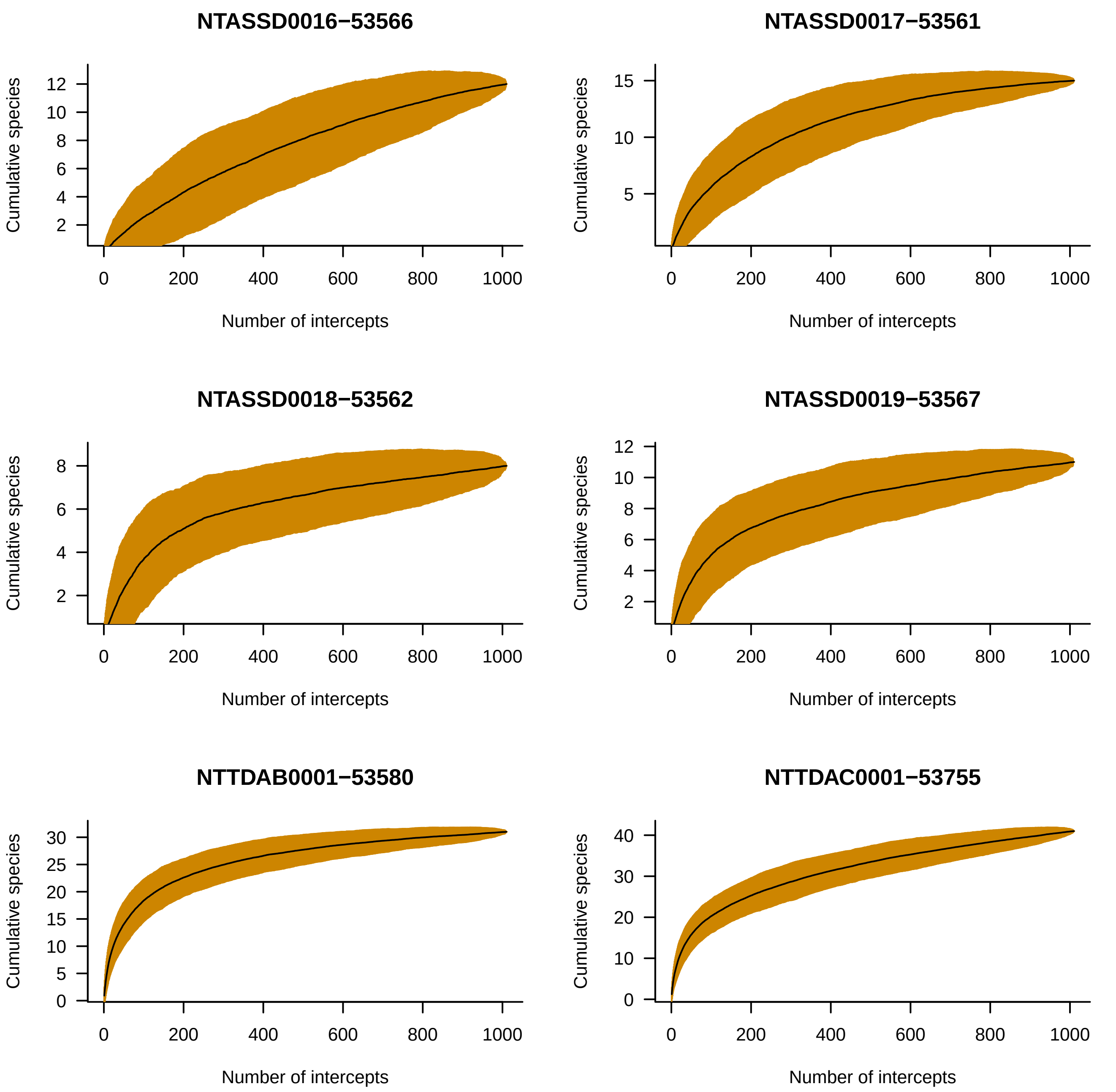
NTASSD0016−53566
NTASSD0017−53561
NTASSD0018−53562
NTASSD0019−53567
NTTDAB0001−53580
NTTDAC0001−53755
Cumulative species
Number of intercepts
0 200 400 600 800 1000
2 4 6 8 10 12
5 10 15
2 4 6 8
2 4 6 8 10 12
0 5 10 15 20 25 30
0 10 20 30 40

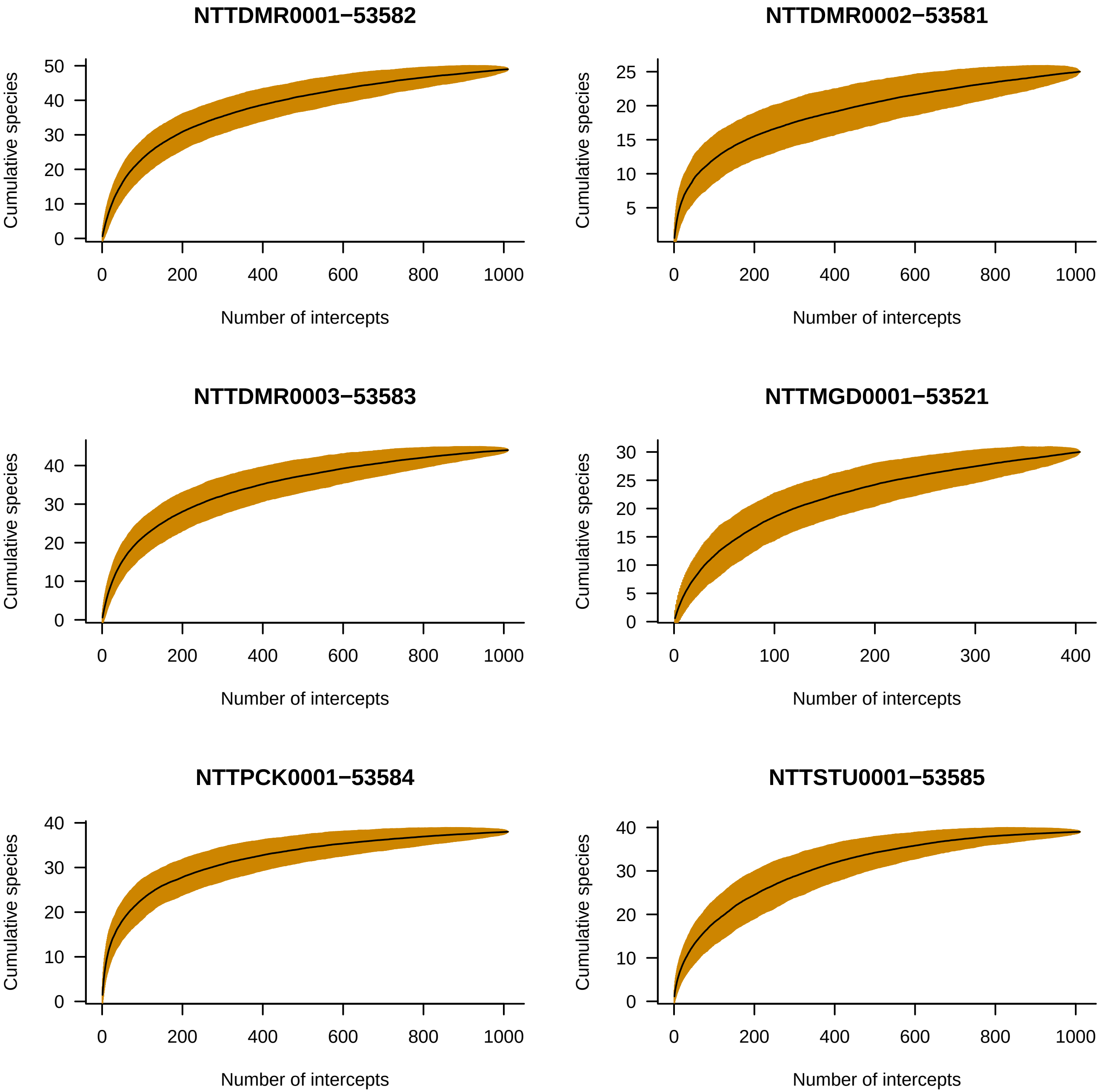
NTTDMR0001−53582
NTTDMR0002−53581
NTTDMR0003−53583
NTTMGD0001−53521
NTTPCK0001−53584
NTTSTU0001−53585
Cumulative species
Number of intercepts
0
200
400
600
800
1000
100
300
10
20
30
40
50
5
15
25

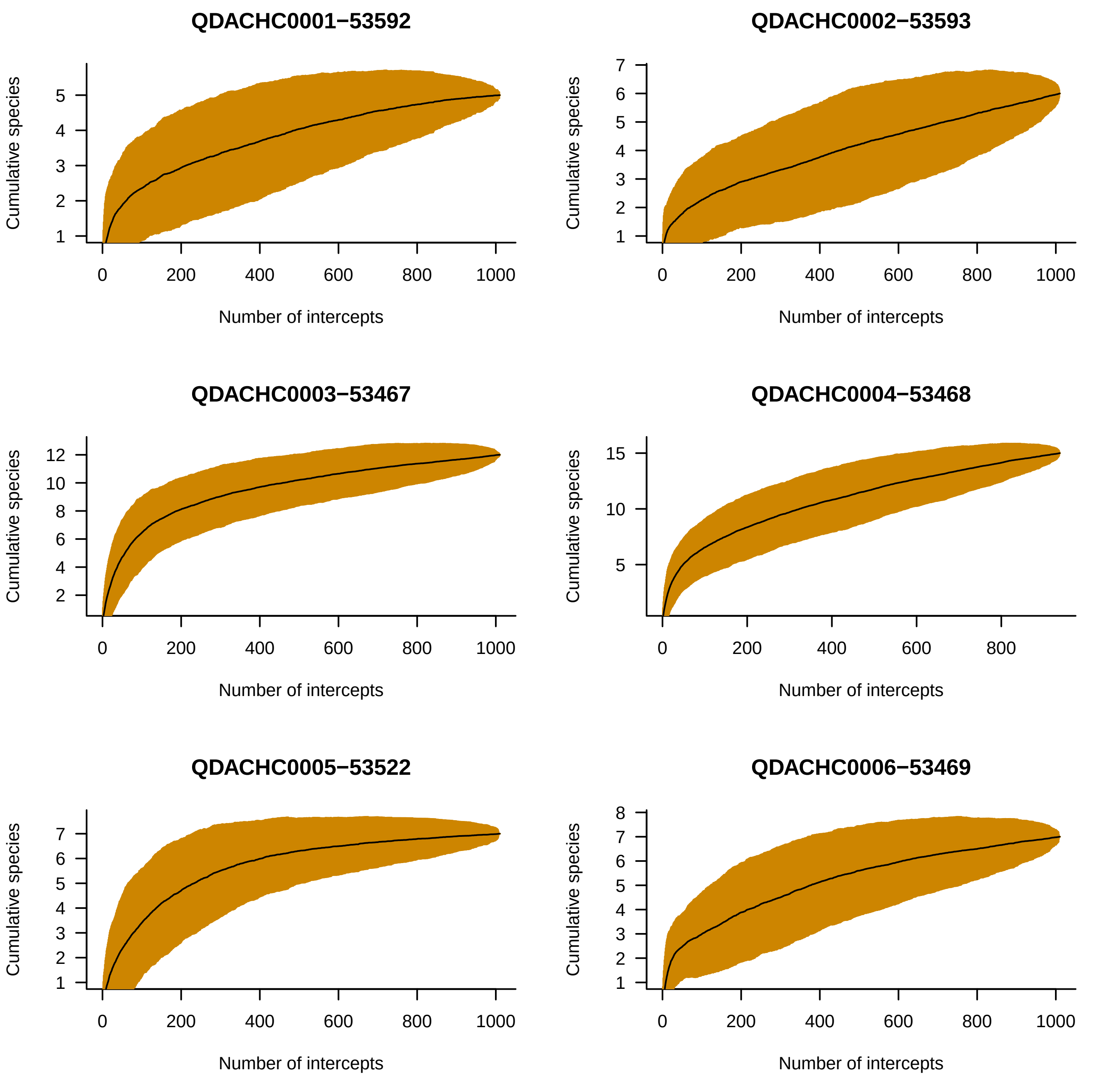
QDACHC0001−53592
Cumulative species
Number of intercepts
QDACHC0002−53593
Cumulative species
Number of intercepts
QDACHC0003−53467
Cumulative species
Number of intercepts
QDACHC0004−53468
Cumulative species
Number of intercepts
QDACHC0005−53522
Cumulative species
Number of intercepts
QDACHC0006−53469
Cumulative species
Number of intercepts

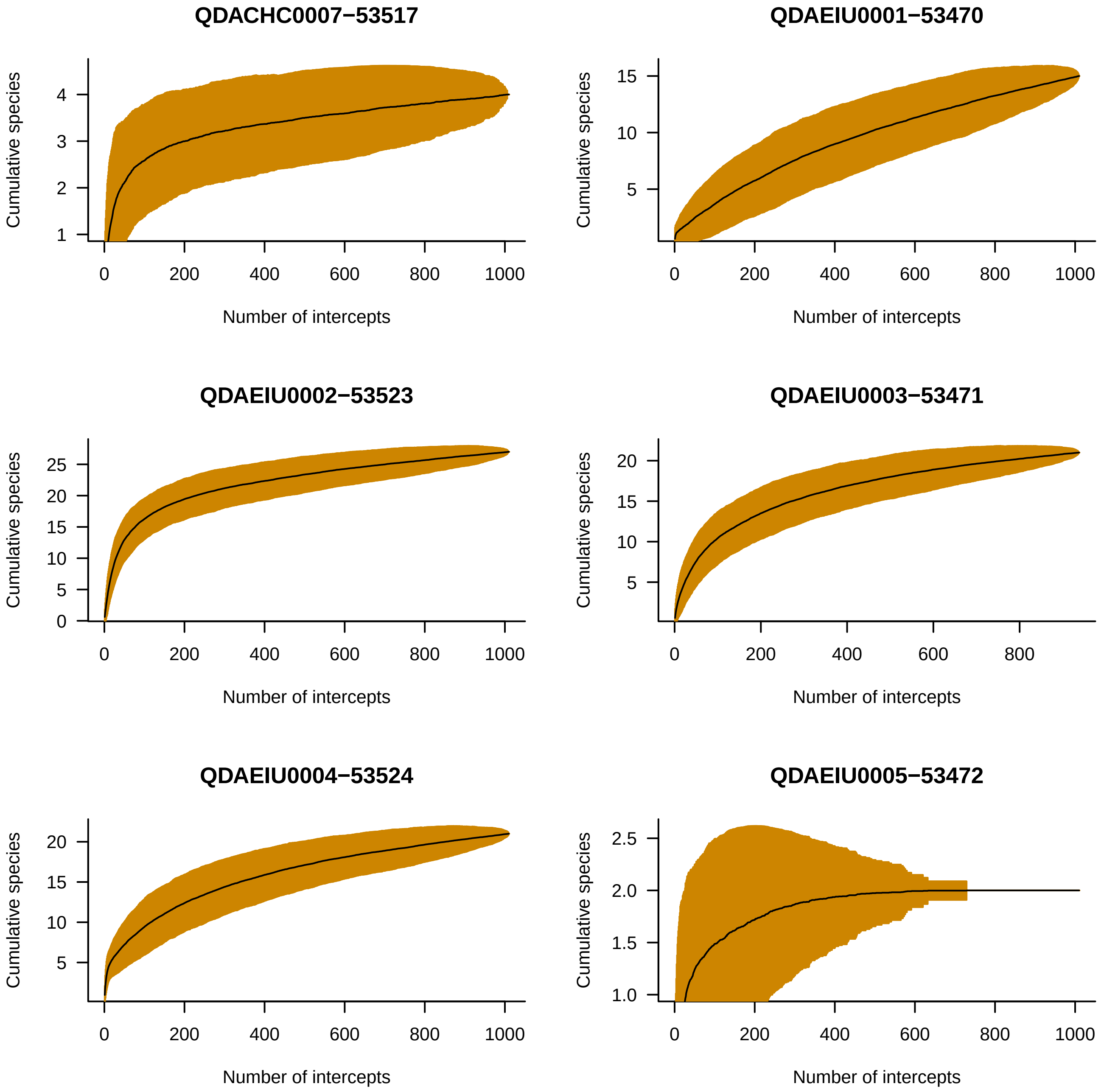
QDACHC0007−53517
QDAEIU0001−53470
QDAEIU0002−53523
QDAEIU0003−53471
QDAEIU0004−53524
QDAEIU0005−53472
Cumulative species
Number of intercepts
0
200
400
600
800
1000
1
2
3
4
5
10
15
20
25
1.0
1.5
2.0
2.5

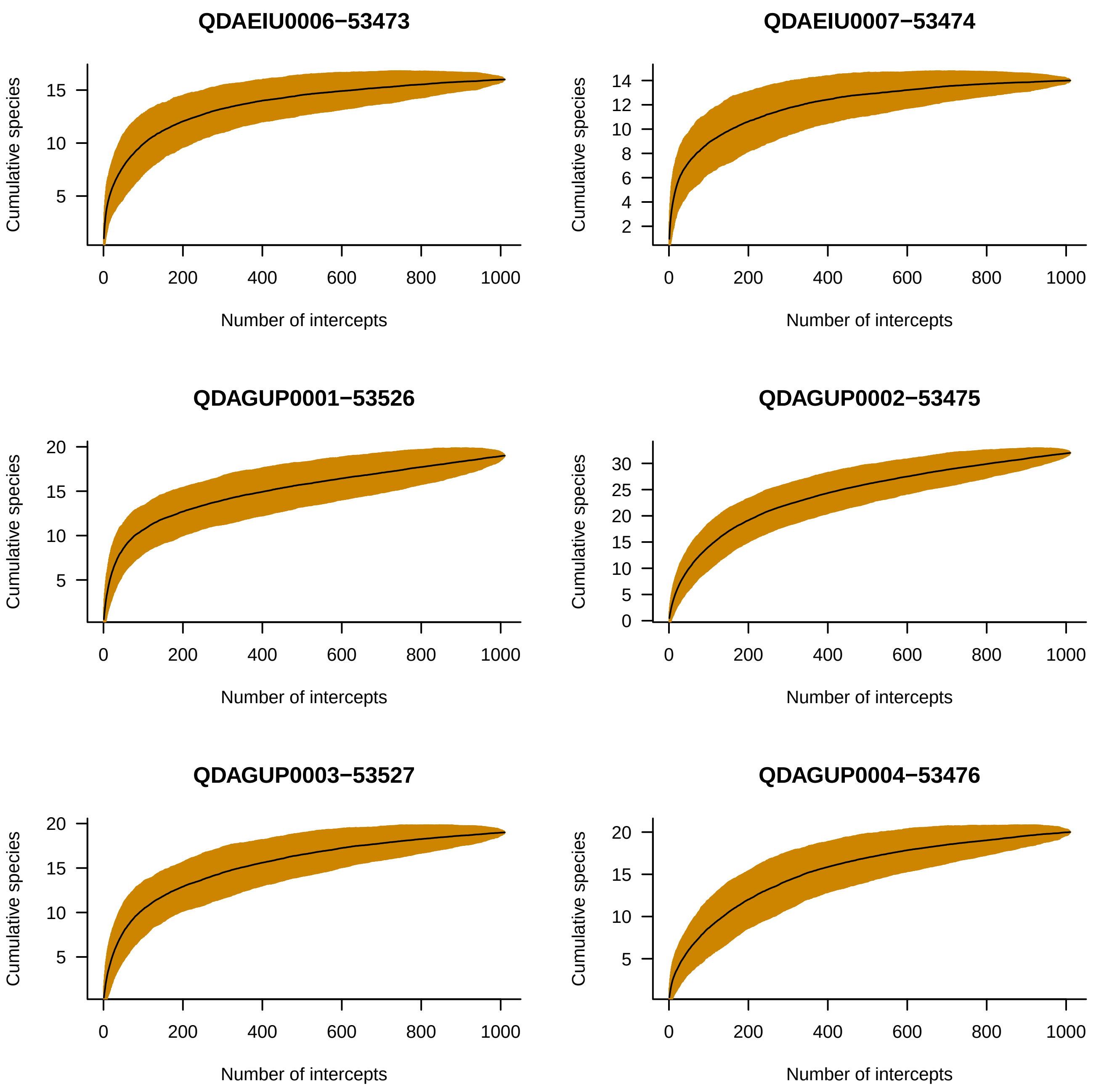
QDAEIU0006−53473
QDAEIU0007−53474
QDAGUP0001−53526
QDAGUP0002−53475
QDAGUP0003−53527
QDAGUP0004−53476
Cumulative species
Number of intercepts
0
200
400
600
800
1000
5
10
15
20
25
30
2
4
6
8
12
14

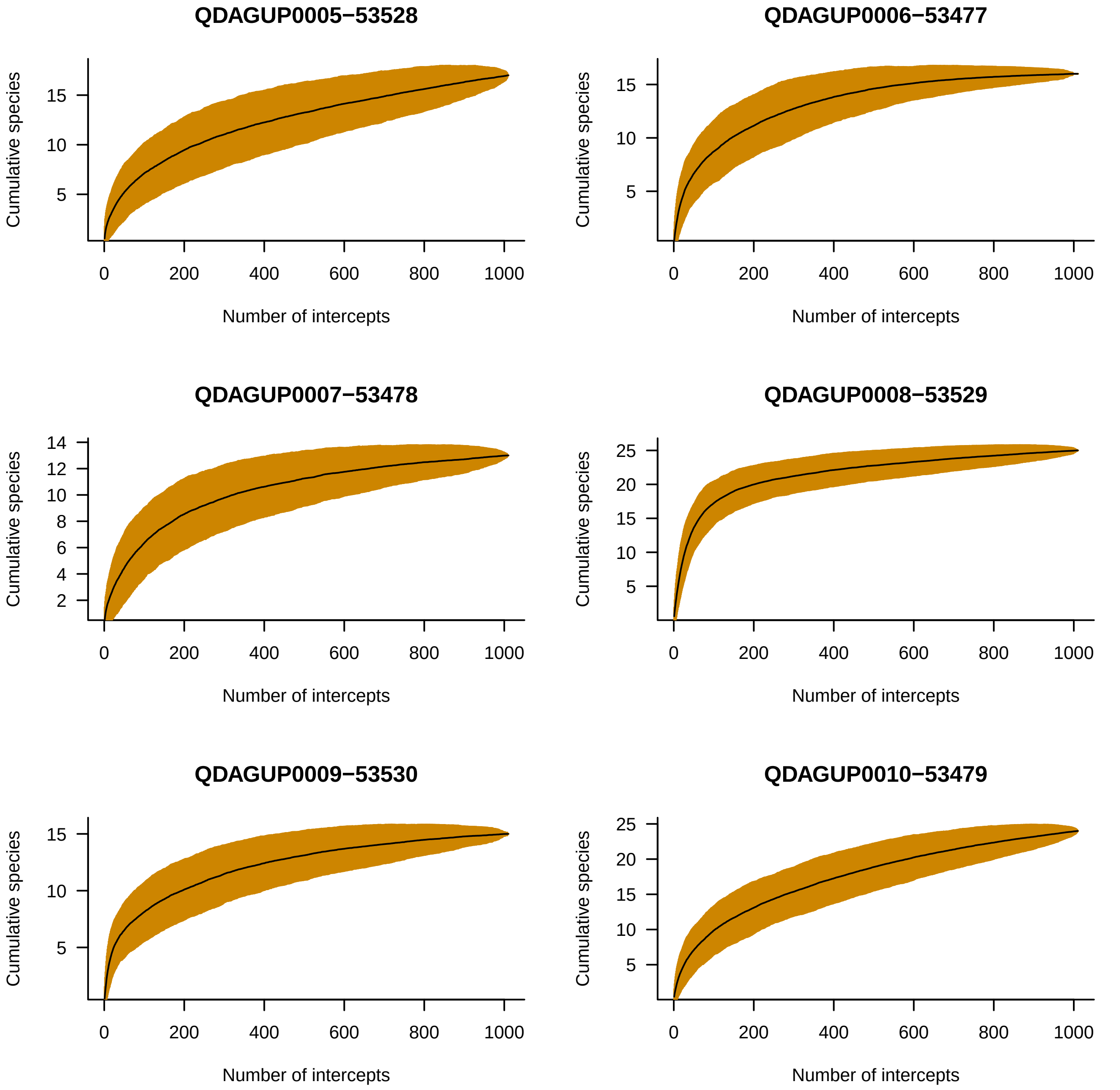

QDAGUP0005−53528
QDAGUP0006−53477
QDAGUP0007−53478
QDAGUP0008−53529
QDAGUP0009−53530
QDAGUP0010−53479
Cumulative species
Number of intercepts
0 200 400 600 800 1000

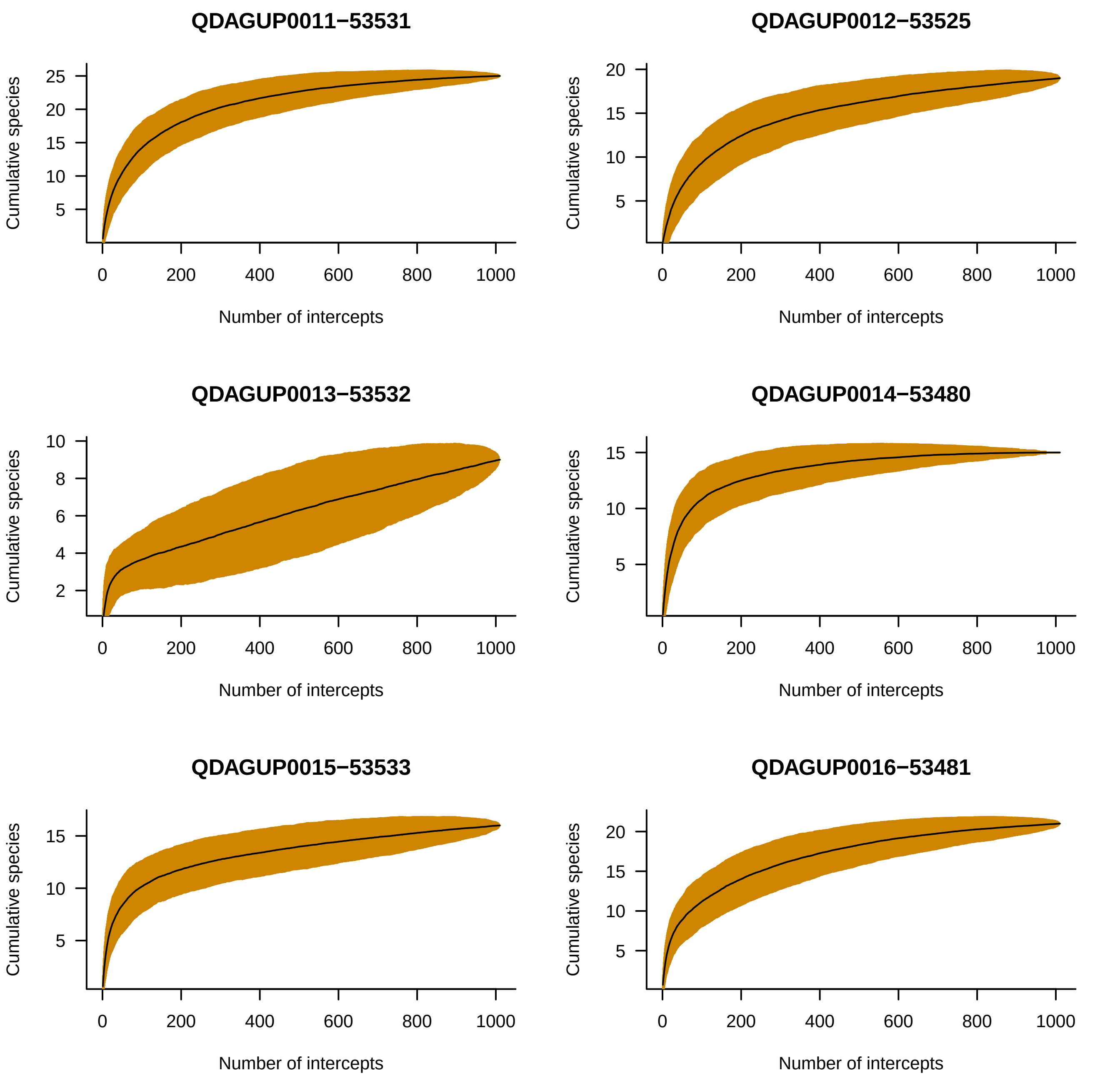
QDAGUP0011−53531
QDAGUP0012−53525
QDAGUP0013−53532
QDAGUP0014−53480
QDAGUP0015−53533
QDAGUP0016−53481
Cumulative species
Number of intercepts

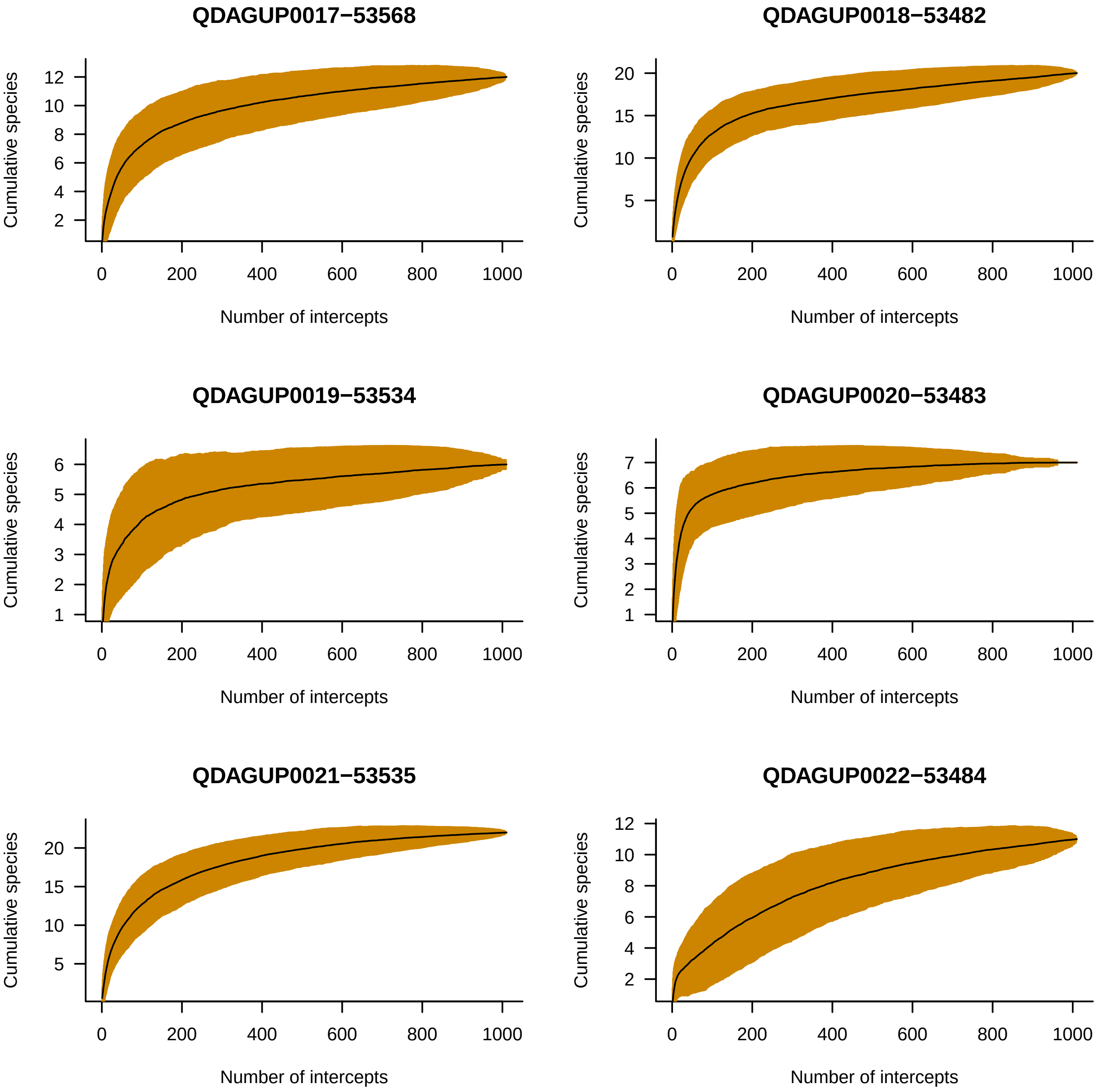
QDAGUP0017−53568
QDAGUP0018−53482
QDAGUP0019−53534
QDAGUP0020−53483
QDAGUP0021−53535
QDAGUP0022−53484
Cumulative species
Number of intercepts
0
200
400
600
800
1000

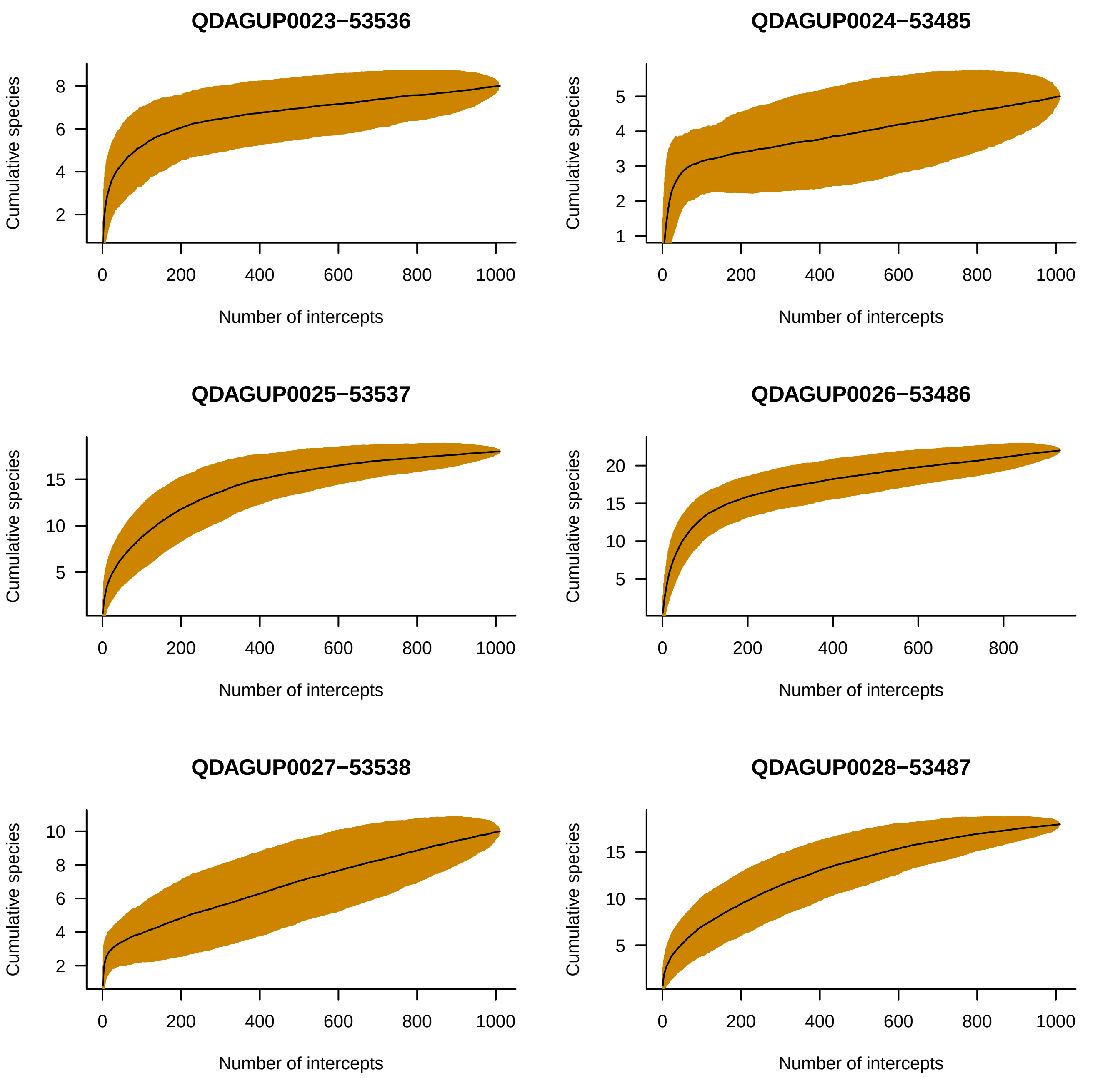
QDAGUP0023−53536
QDAGUP0024−53485
QDAGUP0025−53537
QDAGUP0026−53486
QDAGUP0027−53538
QDAGUP0028−53487
Cumulative species
Number of intercepts

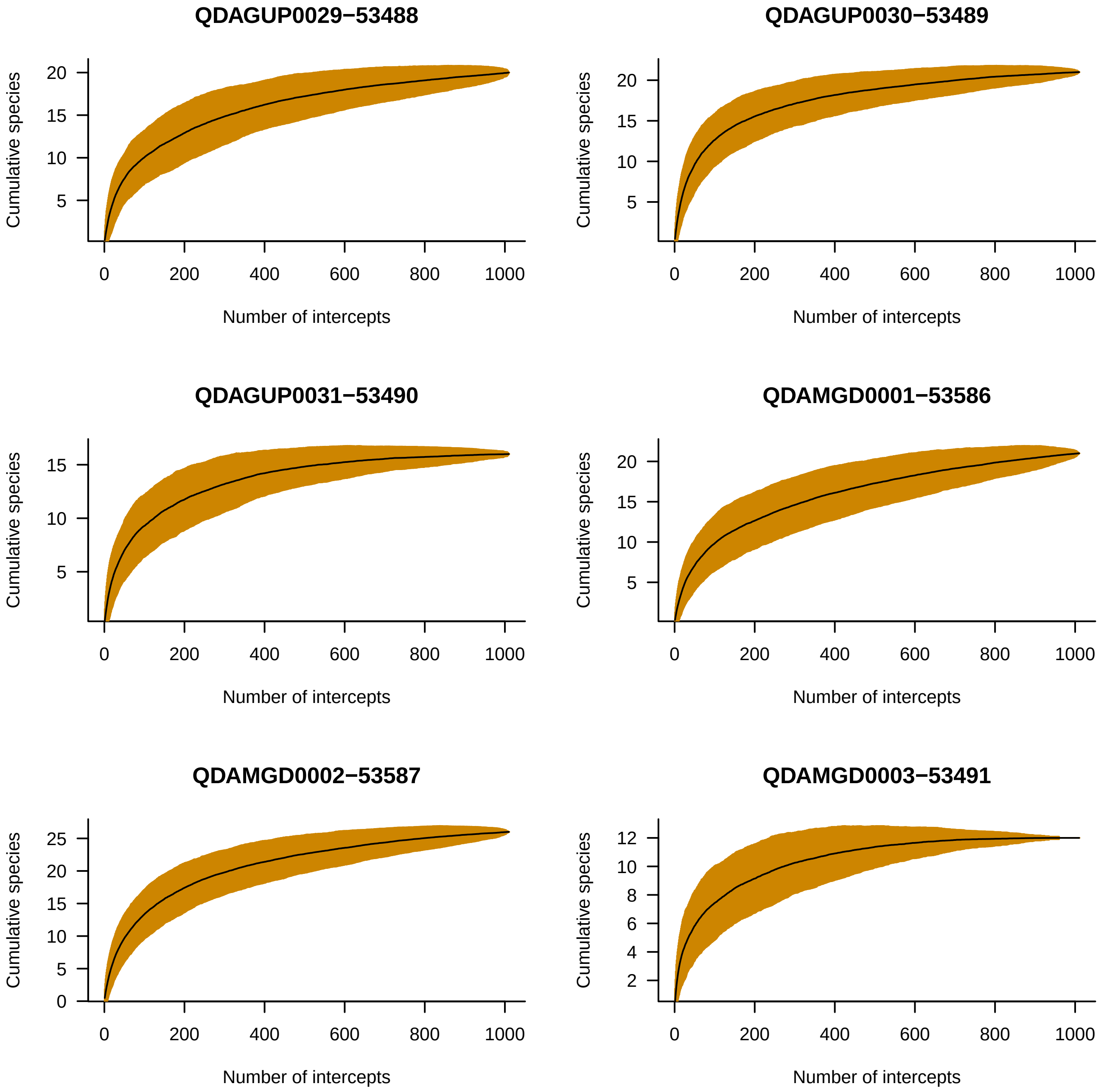
QDAGUP0029−53488
QDAGUP0030−53489
QDAGUP0031−53490
QDAMGD0001−53586
QDAMGD0002−53587
QDAMGD0003−53491
Cumulative species
Number of intercepts

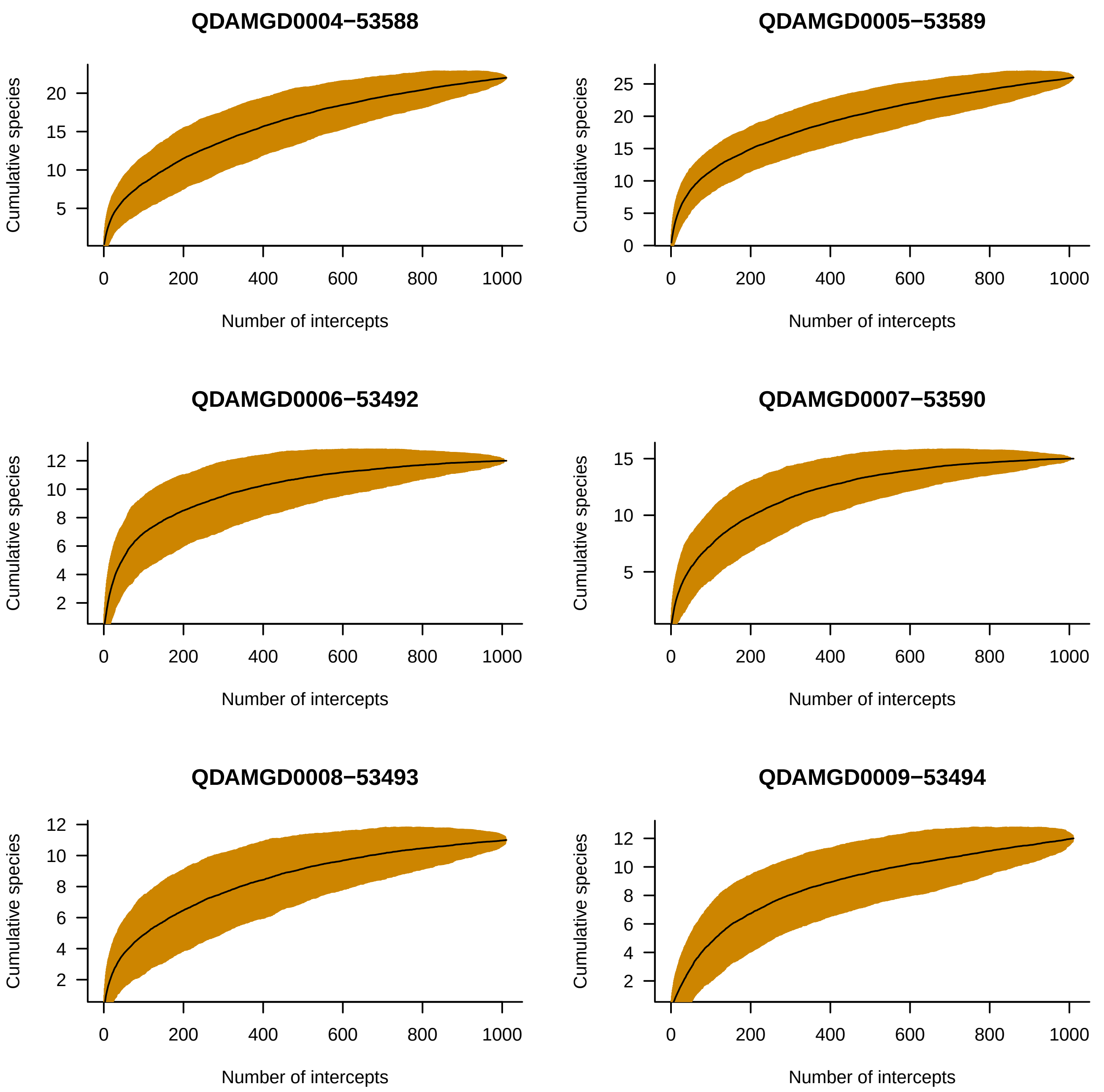
QDAMGD0004−53588
QDAMGD0005−53589
QDAMGD0006−53492
QDAMGD0007−53590
QDAMGD0008−53493
QDAMGD0009−53494
Cumulative species
Number of intercepts
0
200
400
600
800
1000

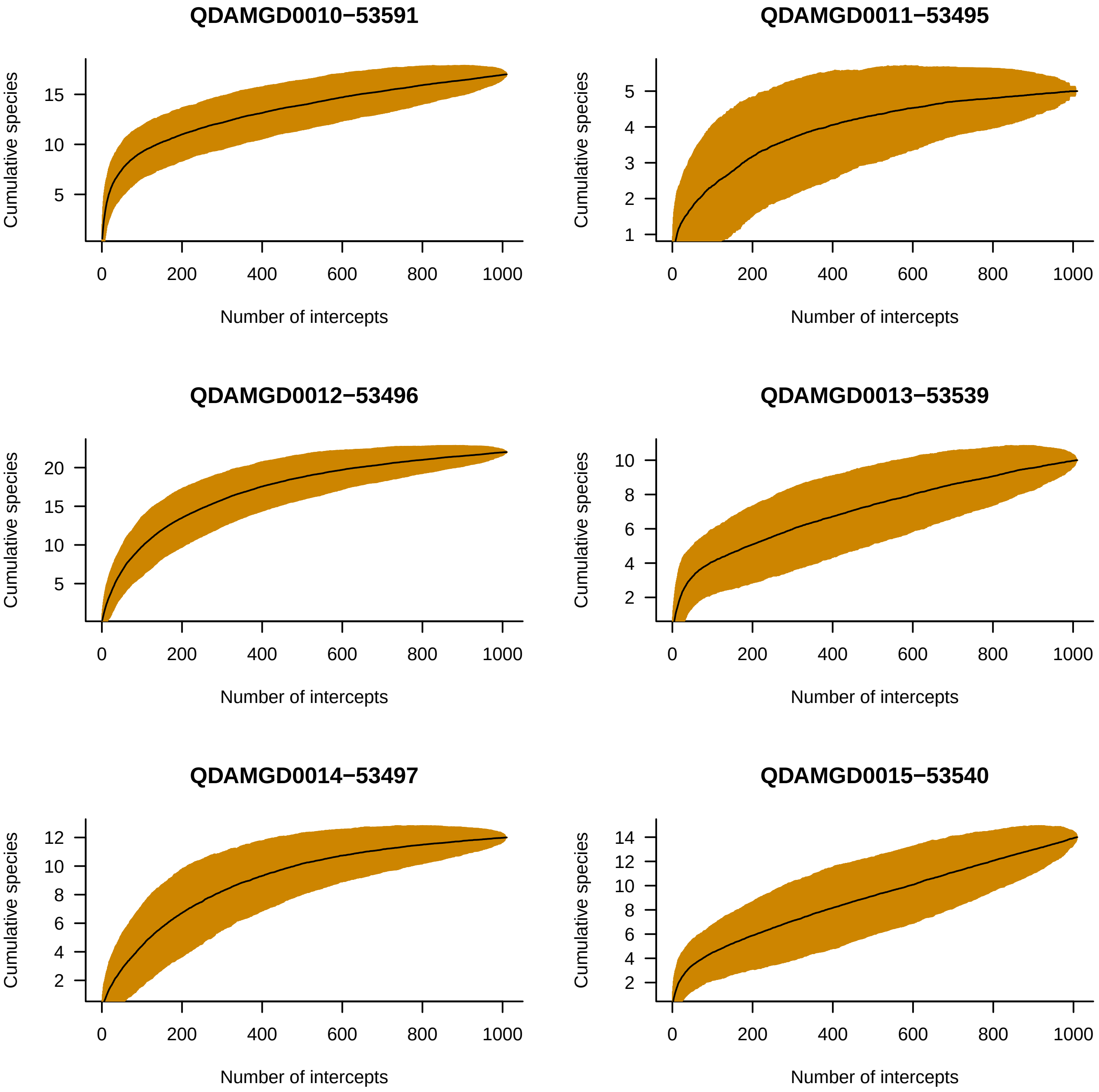
QDAMGD0010−53591
QDAMGD0011−53495
QDAMGD0012−53496
QDAMGD0013−53539
QDAMGD0014−53497
QDAMGD0015−53540
Cumulative species
Number of intercepts
0
200
400
600
800
1000

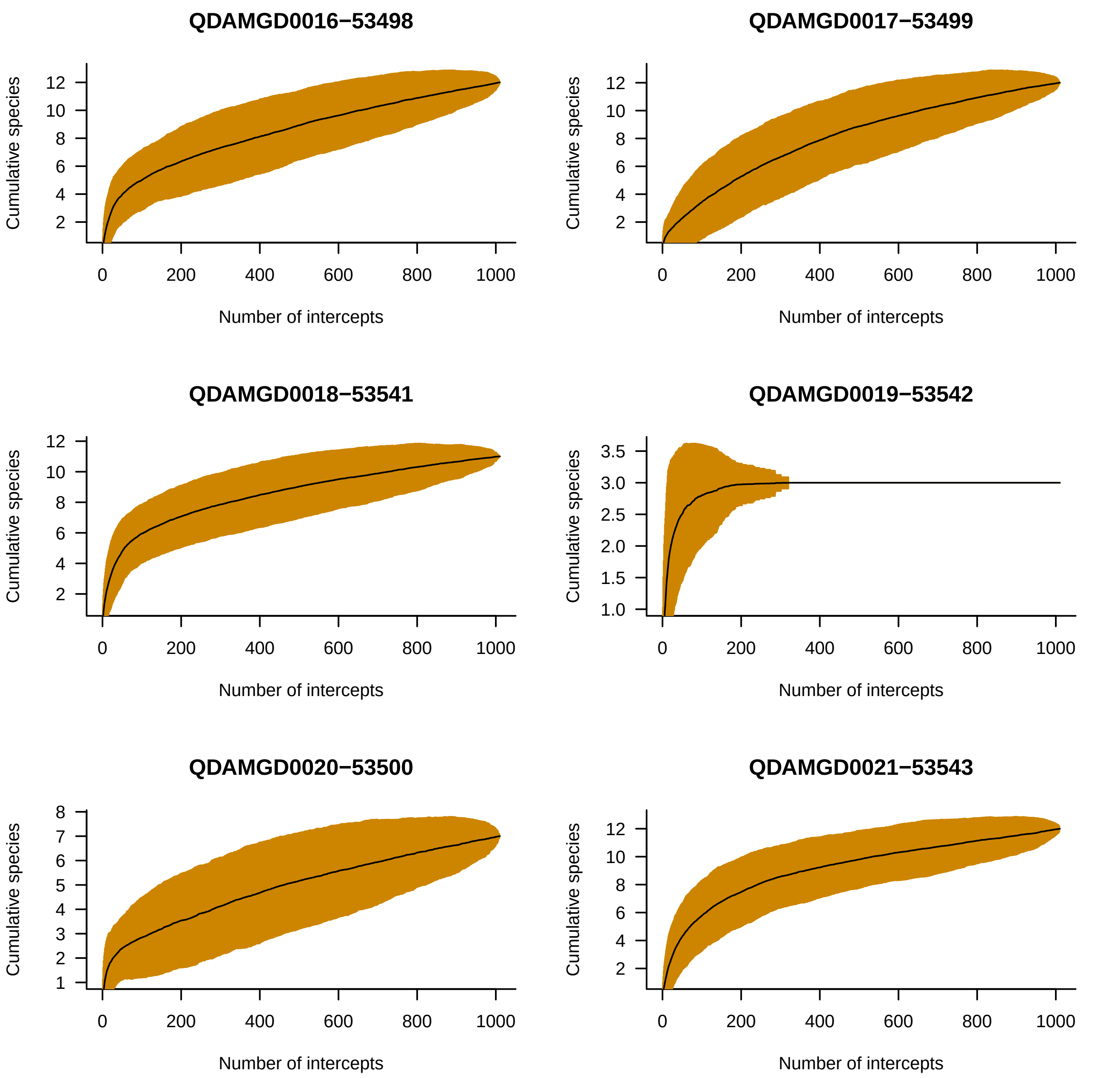
QDAMGD0016−53498
QDAMGD0017−53499
QDAMGD0018−53541
QDAMGD0019−53542
QDAMGD0020−53500
QDAMGD0021−53543
Cumulative species
Number of intercepts
0
200
400
600
800
1000
2
4
6
8
10
12
1.0
1.5
2.0
2.5
3.0
3.5
1
3
5
7

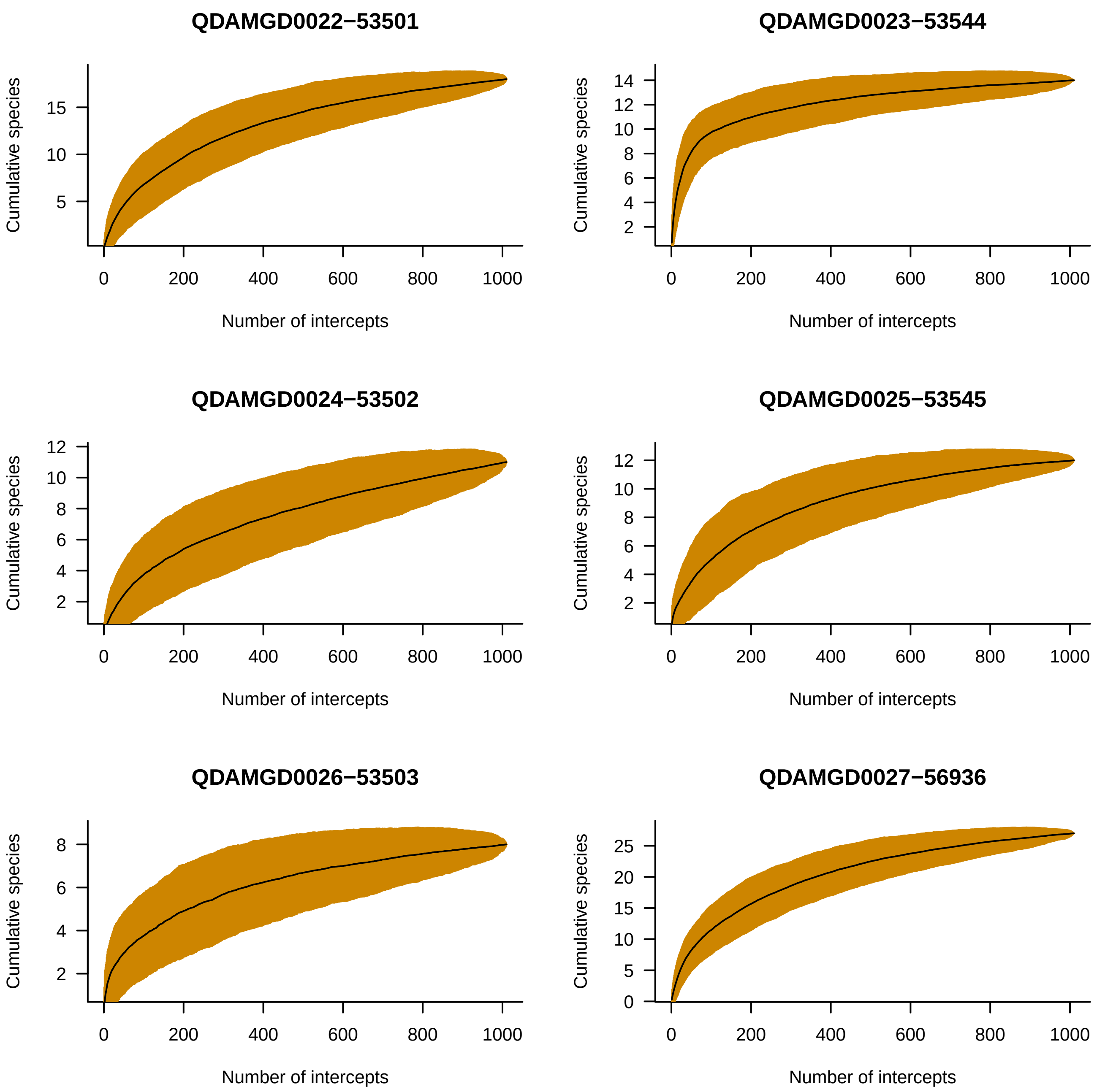
QDAMGD0022−53501
QDAMGD0023−53544
QDAMGD0024−53502
QDAMGD0025−53545
QDAMGD0026−53503
QDAMGD0027−56936
Cumulative species
Number of intercepts
0
200
400
600
800
1000

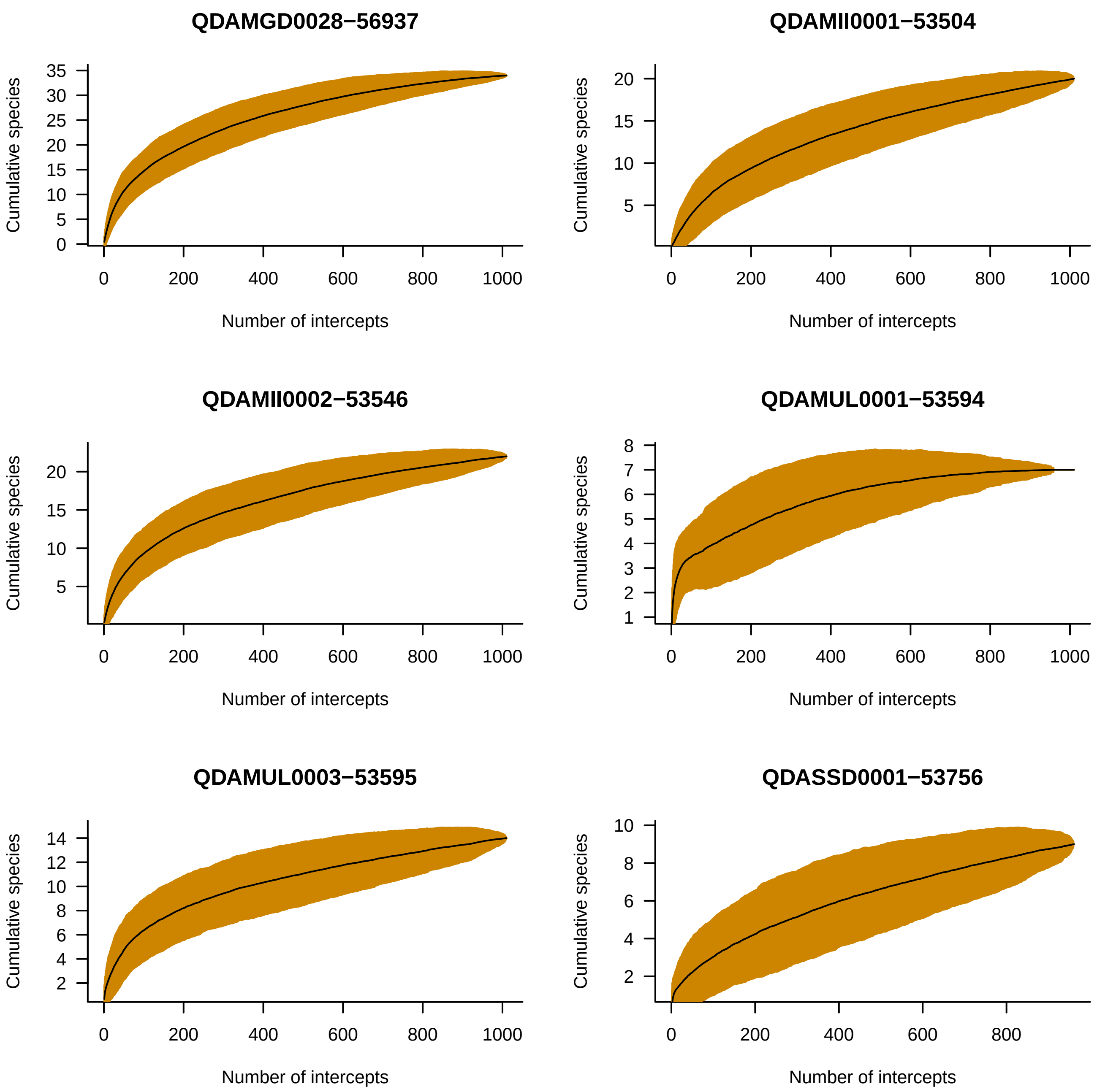
QDAMGD0028−56937
QDAMII0001−53504
QDAMII0002−53546
QDAMUL0001−53594
QDAMUL0003−53595
QDASSD0001−53756
Cumulative species
Number of intercepts
0
200
400
600
800
1000

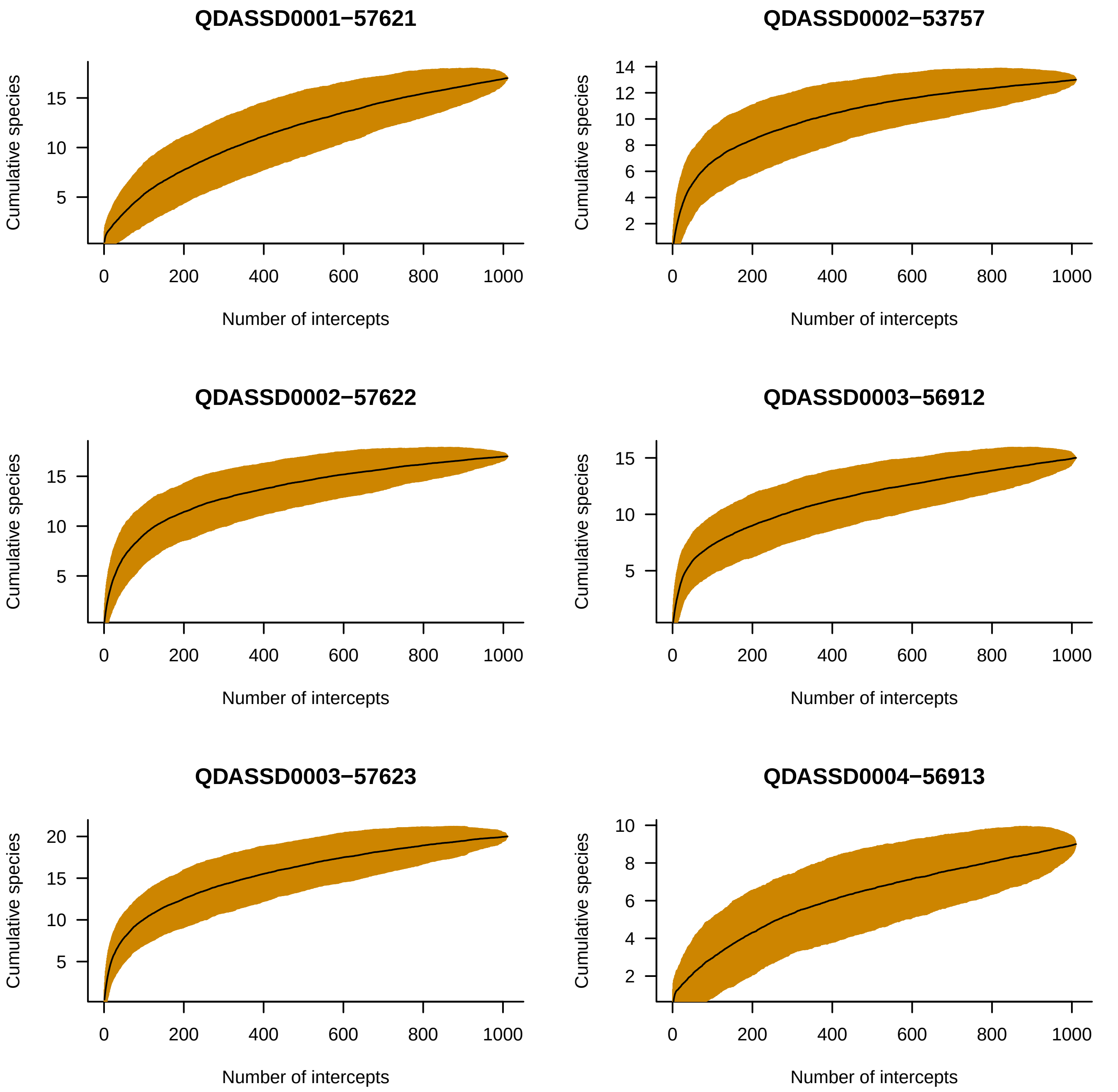
QDASSD0001−57621
QDASSD0002−53757
QDASSD0002−57622
QDASSD0003−56912
QDASSD0003−57623
QDASSD0004−56913
Cumulative species
Number of intercepts
0
200
400
600
800
1000
5
10
15
20
2
4
6
8
12
14

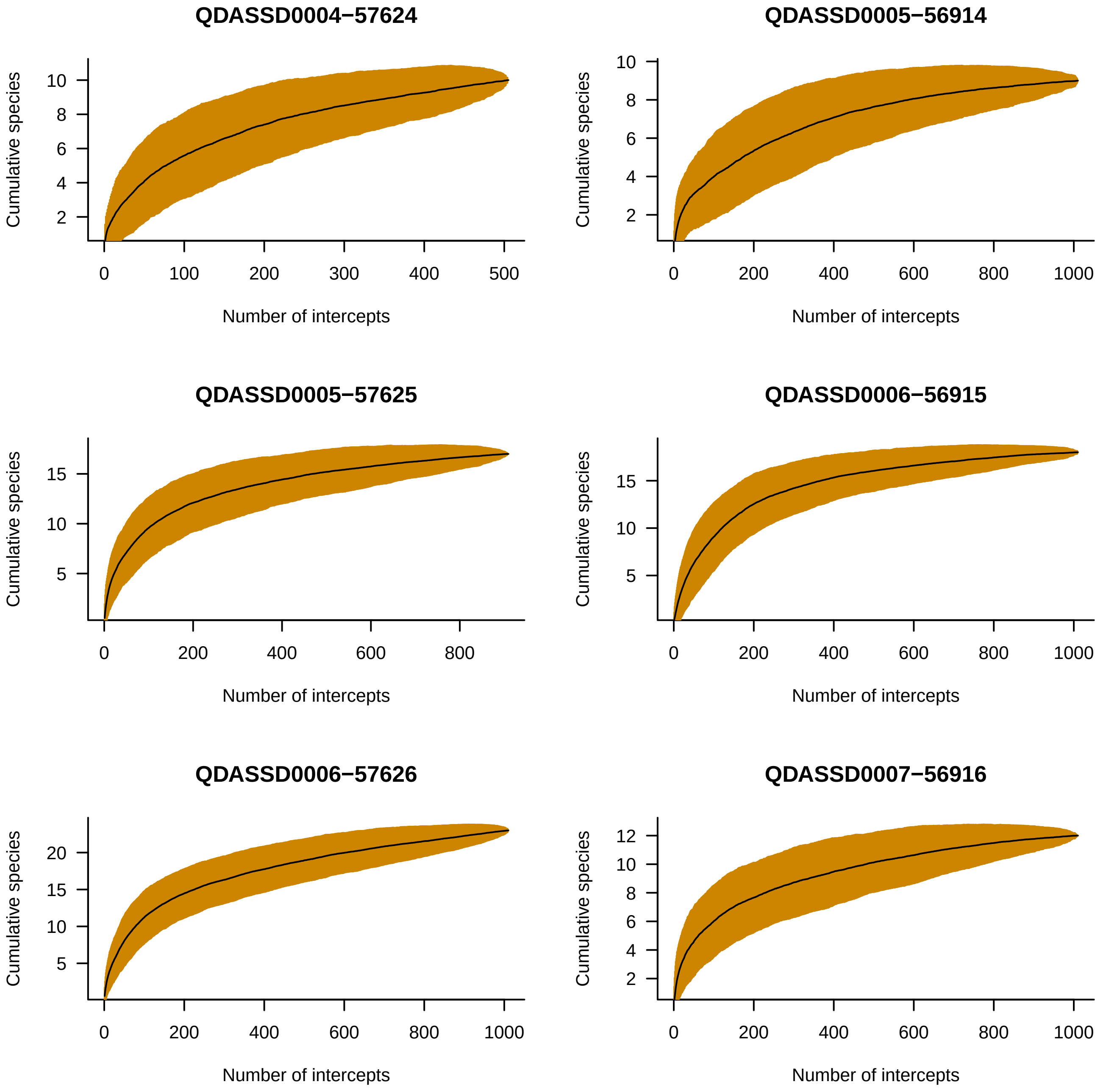
QDASSD0004−57624
Cumulative species
Number of intercepts
QDASSD0005−56914
Cumulative species
Number of intercepts
QDASSD0005−57625
Cumulative species
Number of intercepts
QDASSD0006−56915
Cumulative species
Number of intercepts
QDASSD0006−57626
Cumulative species
Number of intercepts
QDASSD0007−56916
Cumulative species
Number of intercepts

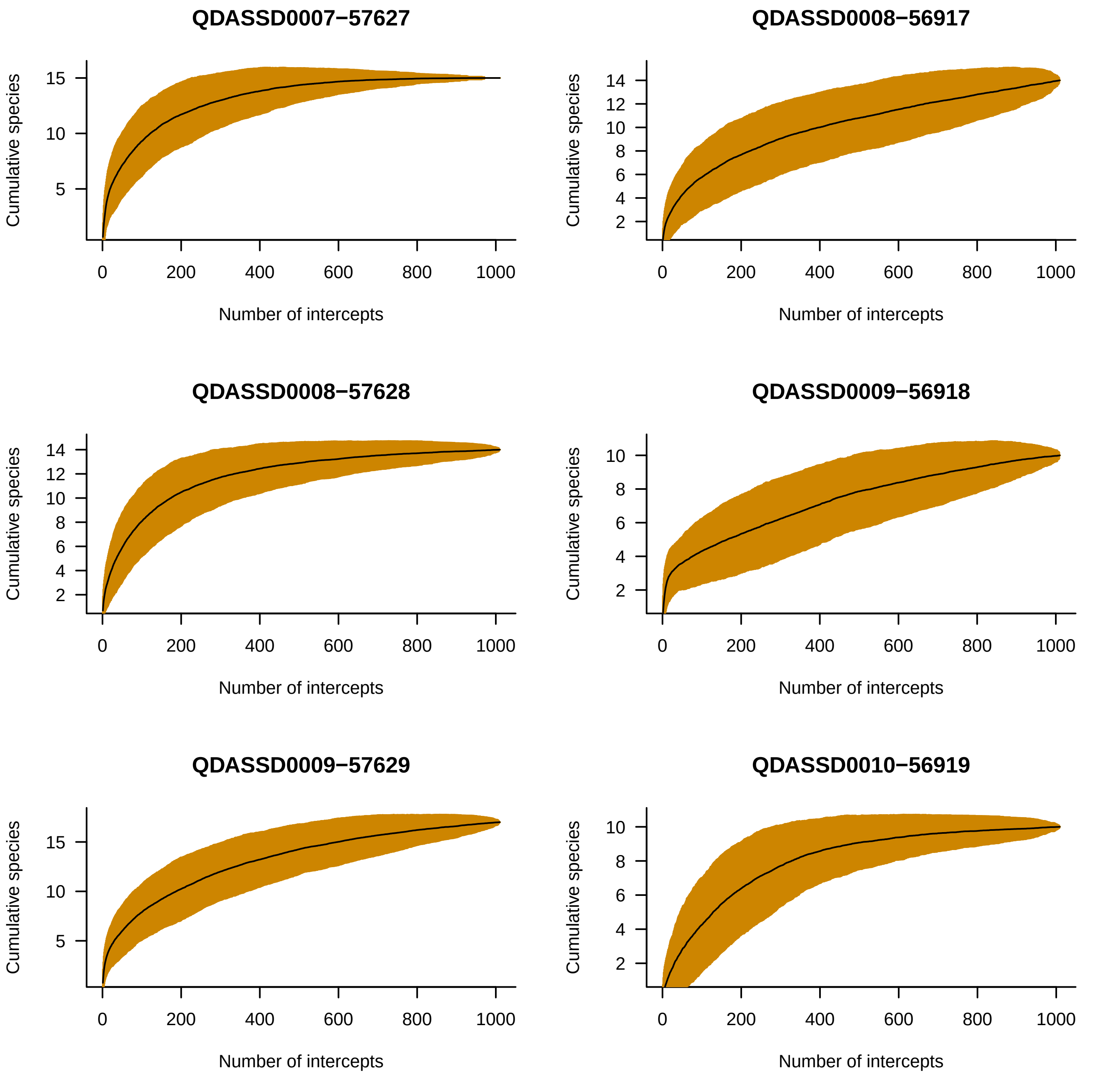
QDASSD0007−57627
QDASSD0008−56917
QDASSD0008−57628
QDASSD0009−56918
QDASSD0009−57629
QDASSD0010−56919
Cumulative species
Number of intercepts
0
200
400
600
800
1000

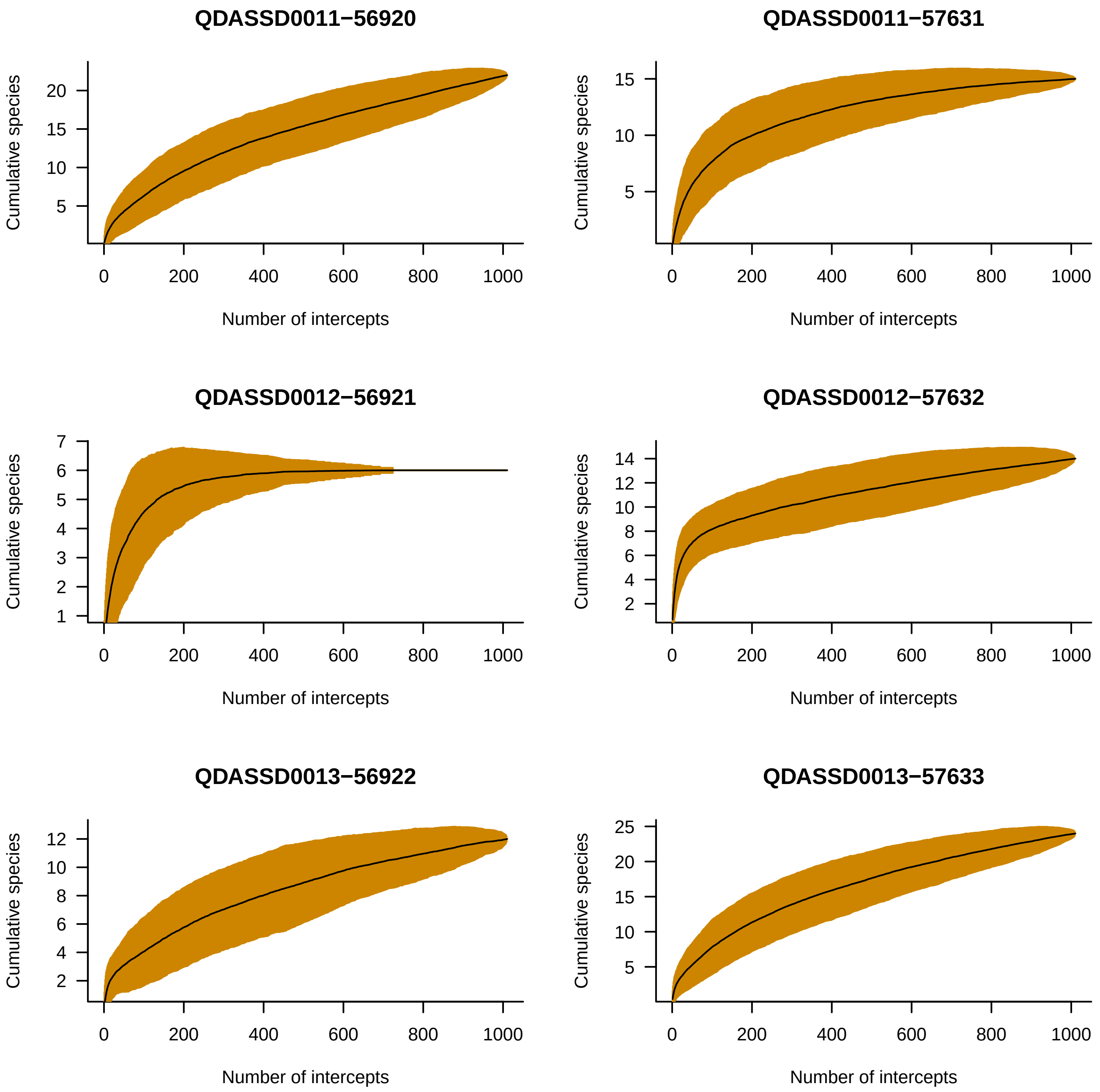
QDASSD0011−56920
QDASSD0011−57631
QDASSD0012−56921
QDASSD0012−57632
QDASSD0013−56922
QDASSD0013−57633
Cumulative species
Number of intercepts
0
200
400
600
800
1000

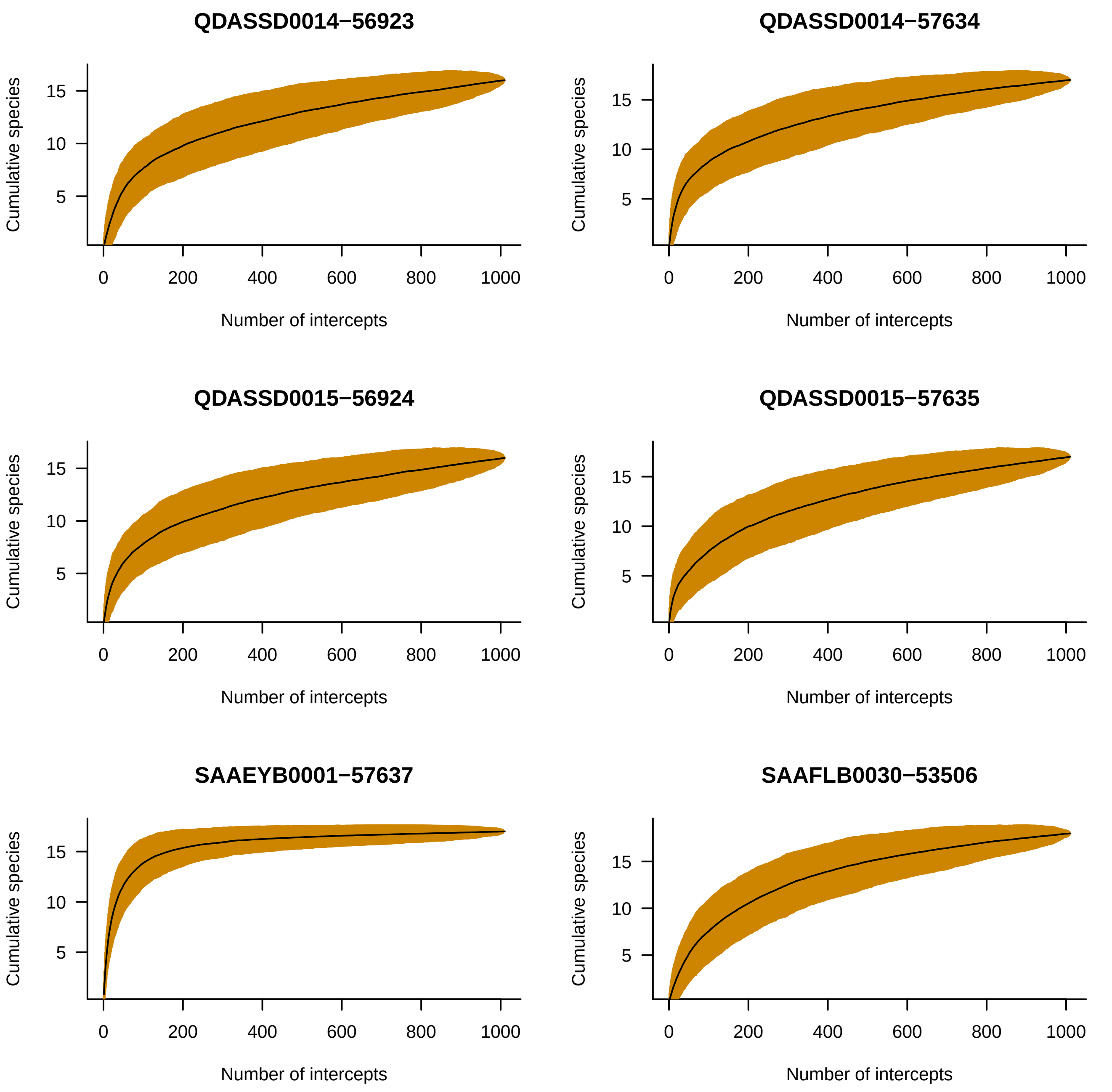
QDASSD0014−56923
QDASSD0014−57634
QDASSD0015−56924
QDASSD0015−57635
SAAEYB0001−57637
SAAFLB0030−53506
Cumulative species
Number of intercepts
0
200
400
600
800
1000
5
10
15

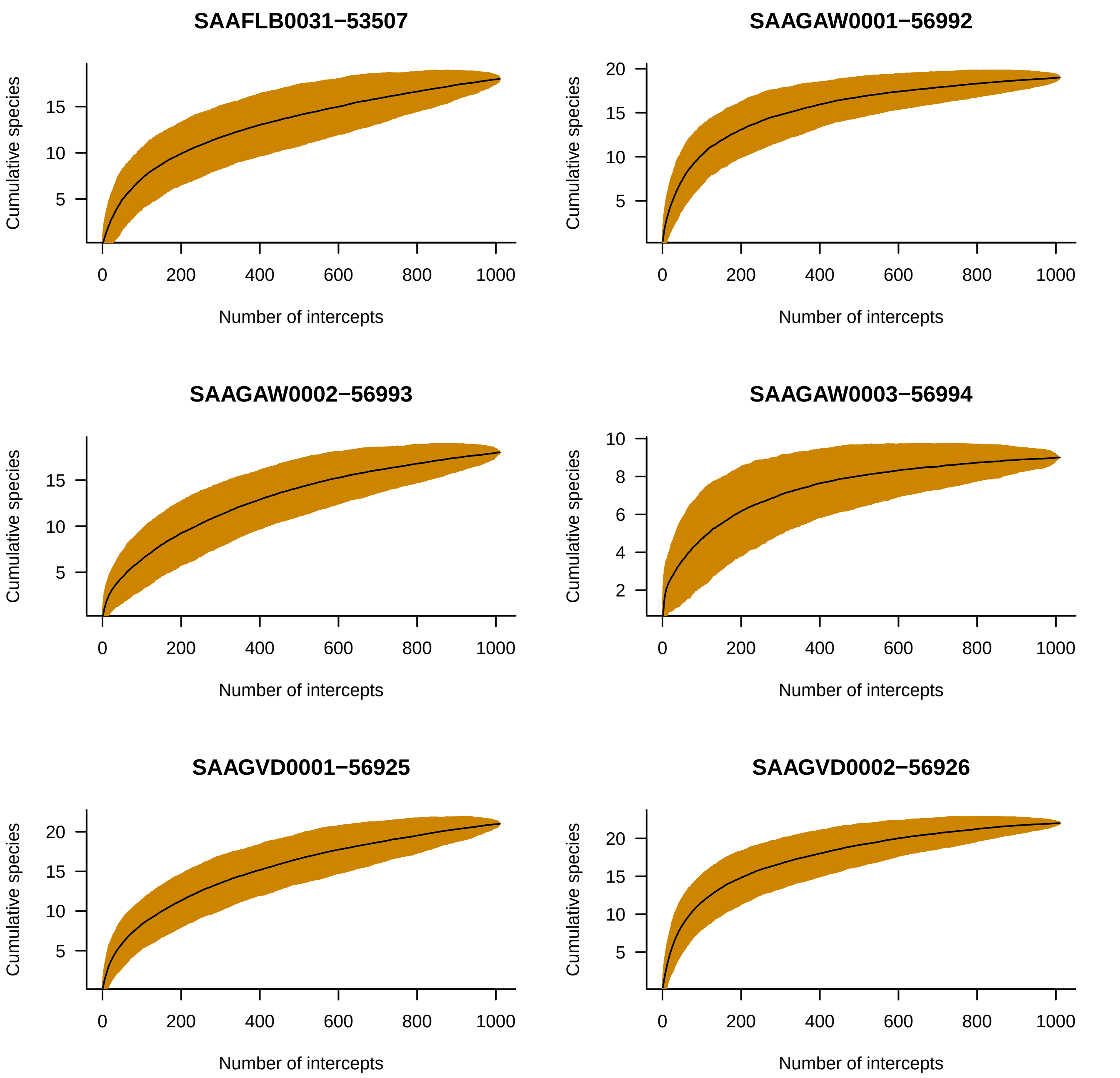
SAAFLB0031−53507
SAAGAW0001−56992
SAAGAW0002−56993
SAAGAW0003−56994
SAAGVD0001−56925
SAAGVD0002−56926
Cumulative species
Number of intercepts

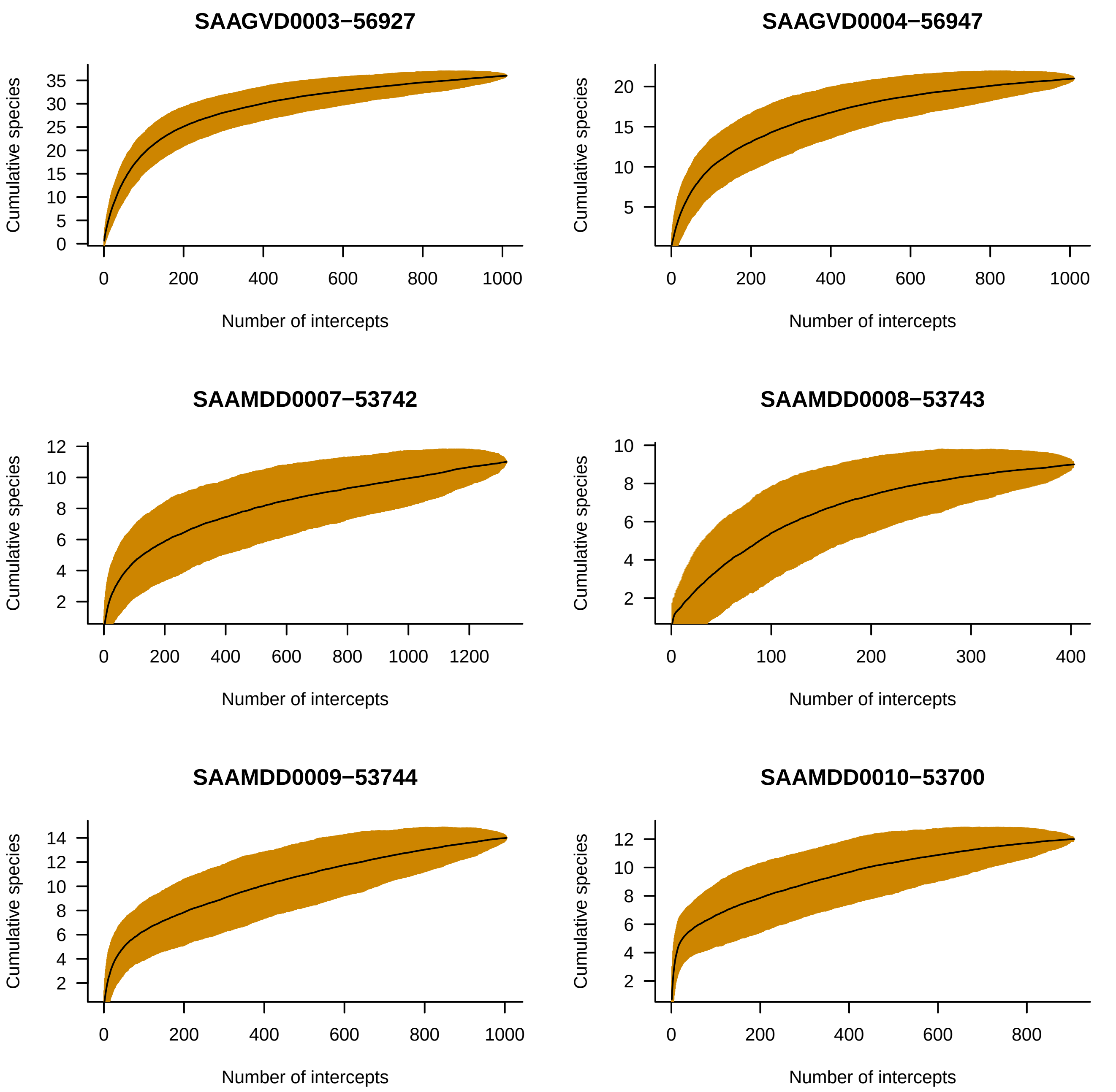
SAAGVD0003−56927
SAAGVD0004−56947
SAAMDD0007−53742
SAAMDD0008−53743
SAAMDD0009−53744
SAAMDD0010−53700
Cumulative species
Number of intercepts

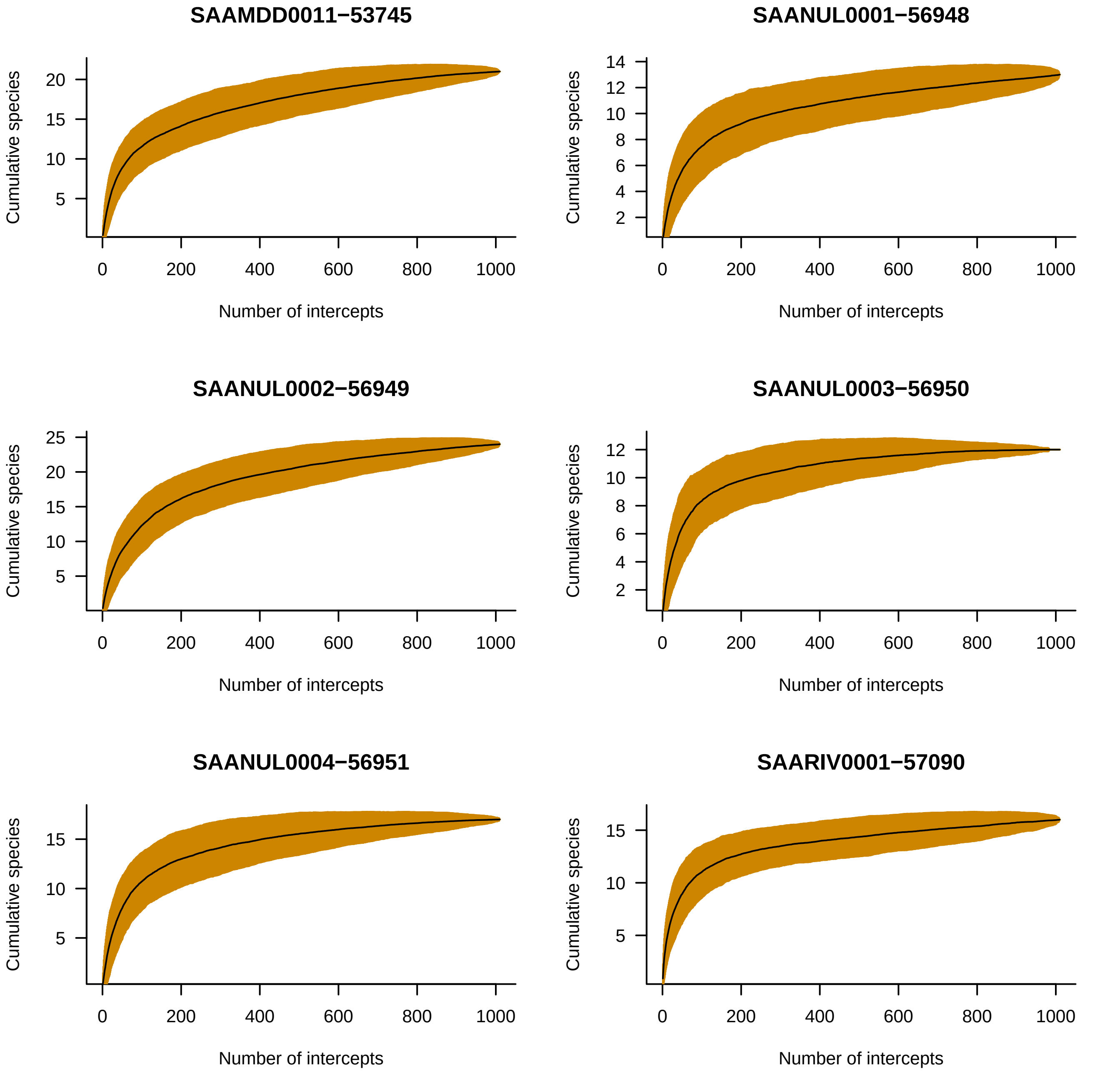
SAAMDD0011−53745
SAANUL0001−56948
SAANUL0002−56949
SAANUL0003−56950
SAANUL0004−56951
SAARIV0001−57090
Cumulative species
Number of intercepts
0
200
400
600
800
1000

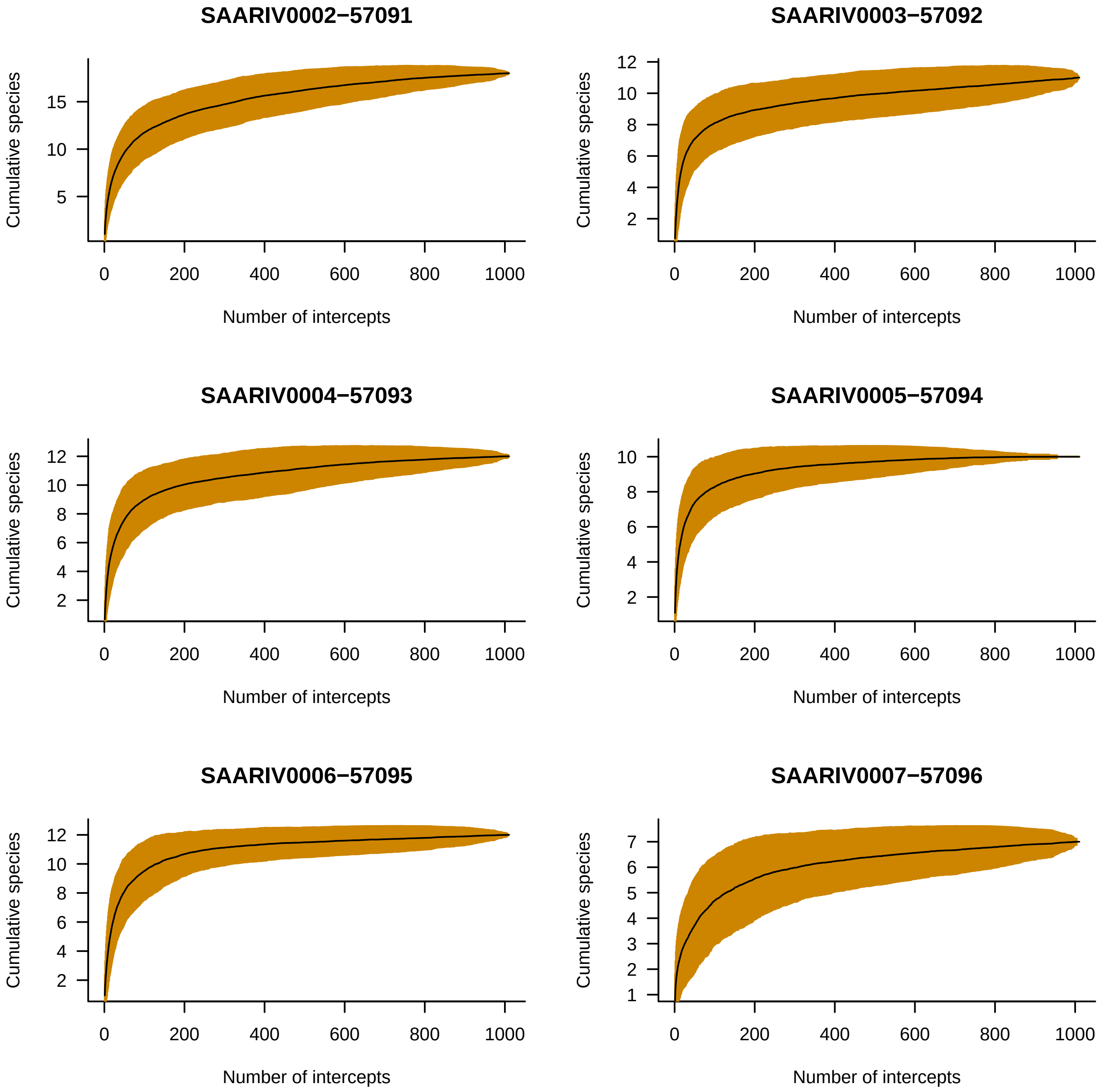
SAARIV0002−57091
SAARIV0003−57092
SAARIV0004−57093
SAARIV0005−57094
SAARIV0006−57095
SAARIV0007−57096
Cumulative species
Number of intercepts
0
200
400
600
800
1000

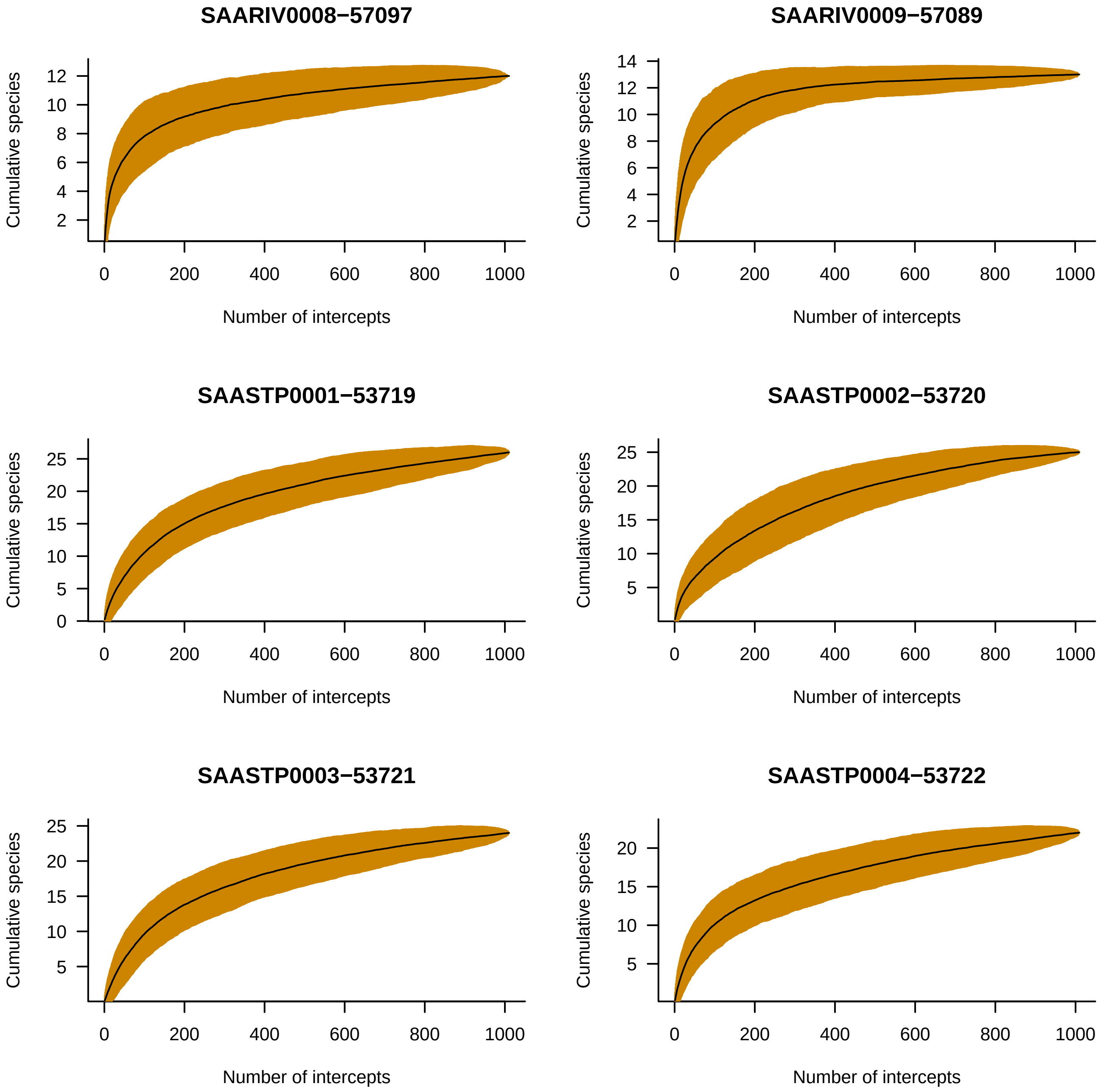
SAARIV0008−57097
SAARIV0009−57089
SAASTP0001−53719
SAASTP0002−53720
SAASTP0003−53721
SAASTP0004−53722
Cumulative species
Number of intercepts
0
200
400
600
800
1000

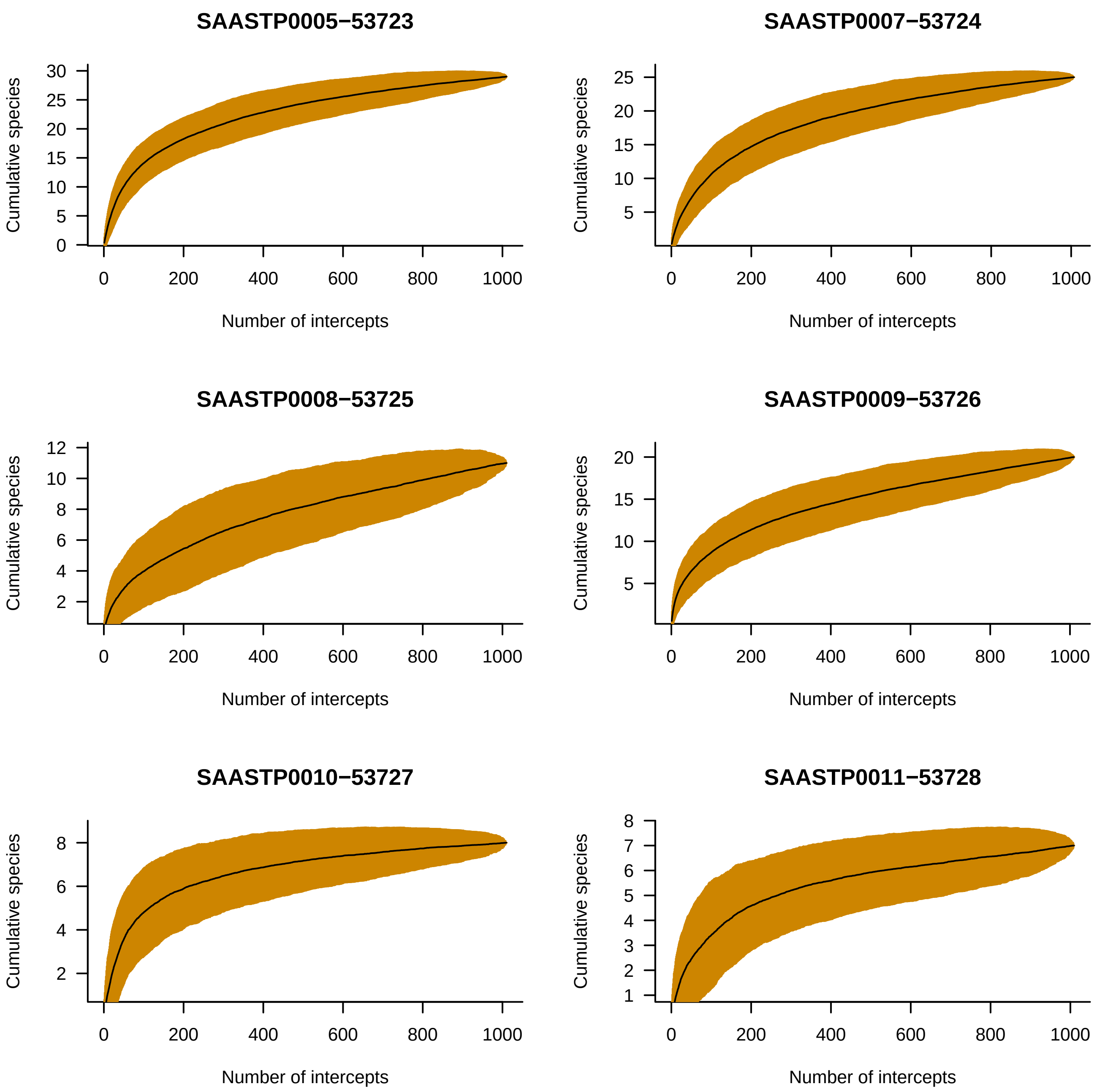
SAASTP0005−53723
Cumulative species
Number of intercepts
SAASTP0007−53724
Cumulative species
Number of intercepts
SAASTP0008−53725
Cumulative species
Number of intercepts
SAASTP0009−53726
Cumulative species
Number of intercepts
SAASTP0010−53727
Cumulative species
Number of intercepts
SAASTP0011−53728
Cumulative species
Number of intercepts

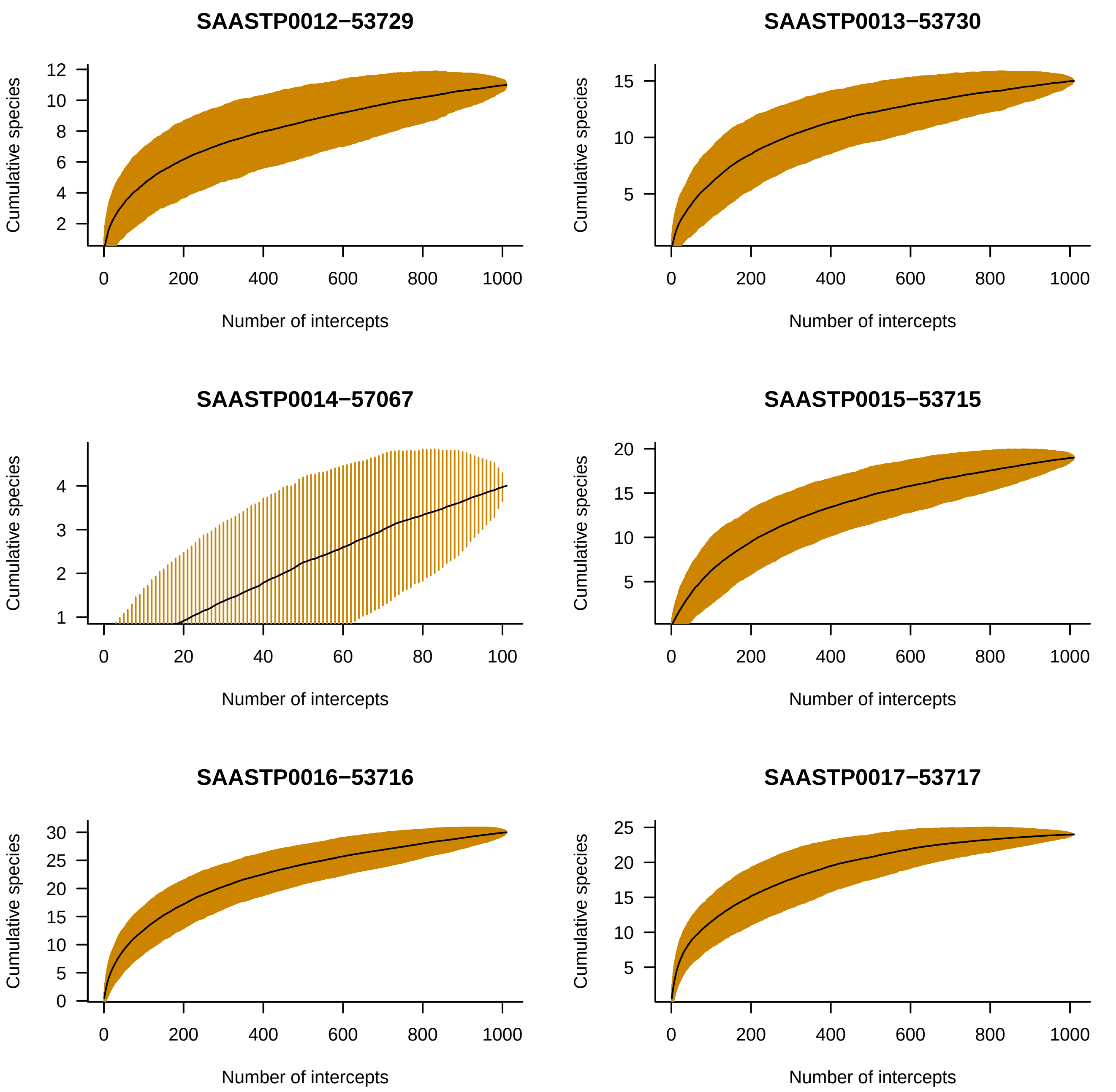
SAASTP0012−53729
Cumulative species
2
4
6
8
10
12
0
200
400
600
800
1000
Number of intercepts
SAASTP0013−53730
Cumulative species
5
10
15
0
200
400
600
800
1000
Number of intercepts
SAASTP0014−57067
Cumulative species
1
2
3
4
0
20
40
60
80
100
Number of intercepts
SAASTP0015−53715
Cumulative species
5
10
15
20
0
200
400
600
800
1000
Number of intercepts
SAASTP0016−53716
Cumulative species
0
5
10
15
20
25
30
0
200
400
600
800
1000
Number of intercepts
SAASTP0017−53717
Cumulative species
5
10
15
20
25
0
200
400
600
800
1000
Number of intercepts

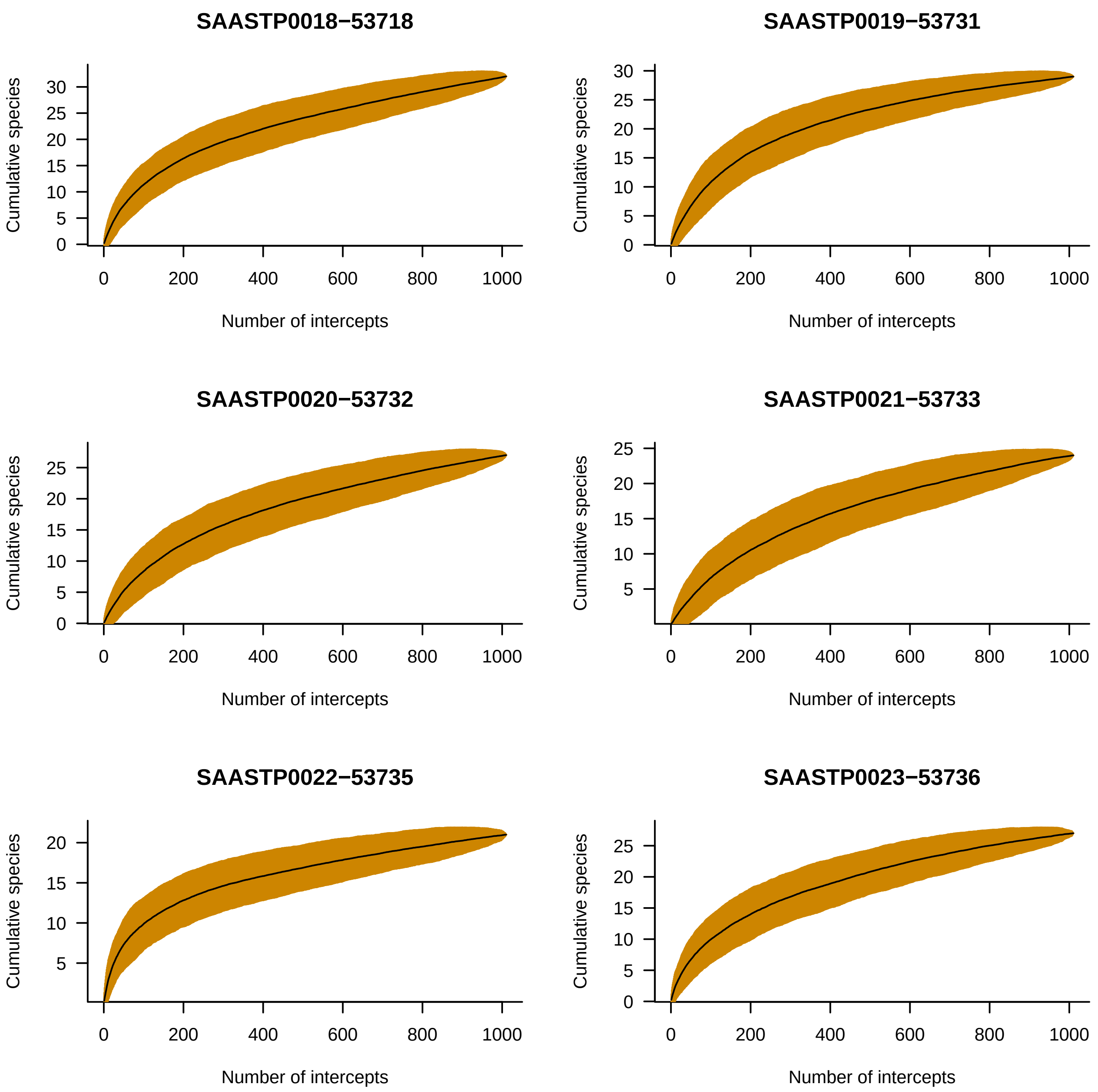
SAASTP0018−53718
Cumulative species
0
5
10
15
20
25
30
0
200
400
600
800
1000
Number of intercepts
SAASTP0019−53731
Cumulative species
0
5
10
15
20
25
30
0
200
400
600
800
1000
Number of intercepts
SAASTP0020−53732
Cumulative species
0
5
10
15
20
25
0
200
400
600
800
1000
Number of intercepts
SAASTP0021−53733
Cumulative species
5
10
15
20
25
0
200
400
600
800
1000
Number of intercepts
SAASTP0022−53735
Cumulative species
5
10
15
20
0
200
400
600
800
1000
Number of intercepts
SAASTP0023−53736
Cumulative species
0
5
10
15
20
25
0
200
400
600
800
1000
Number of intercepts

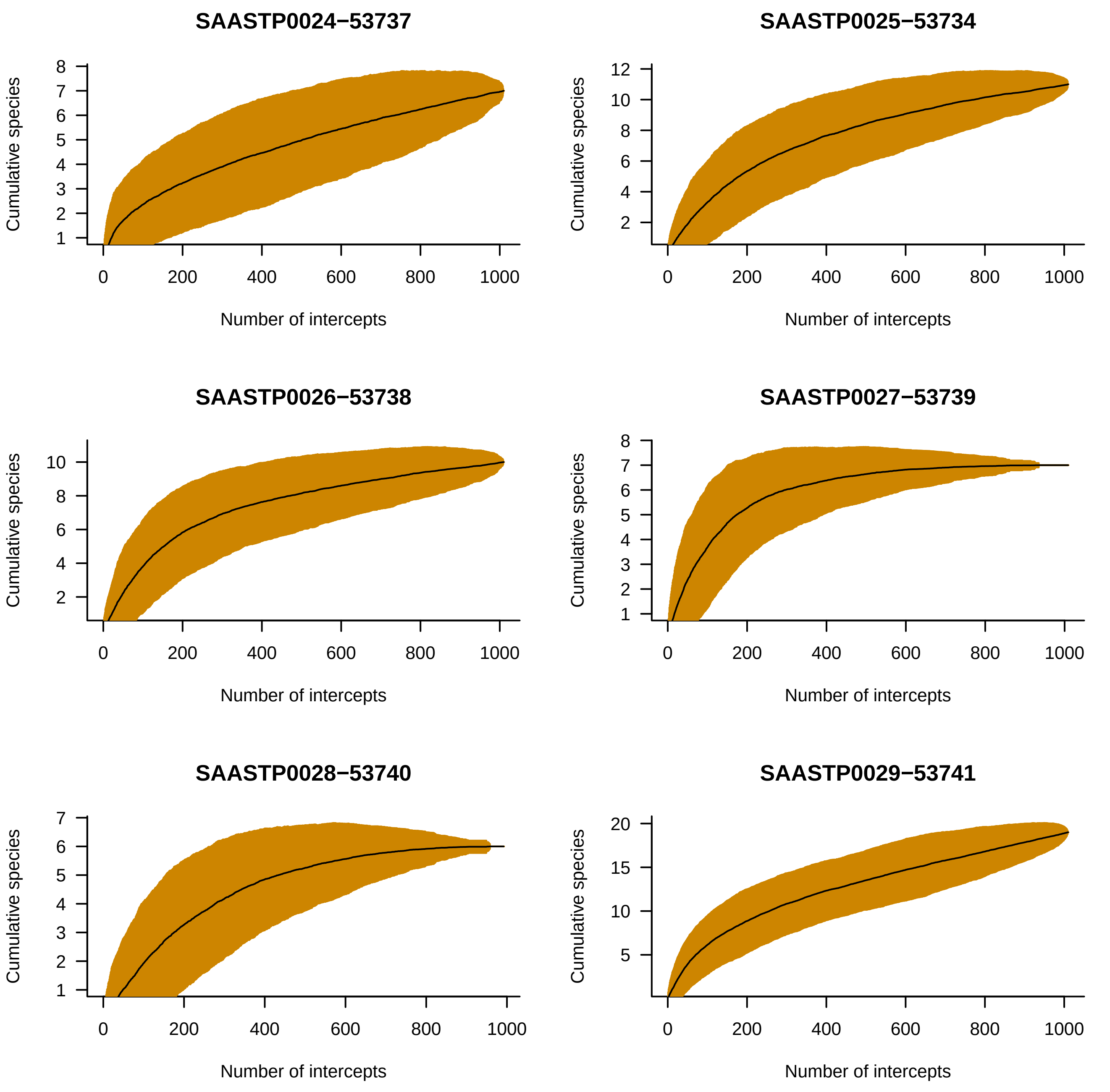
SAASTP0024−53737
SAASTP0025−53734
SAASTP0026−53738
SAASTP0027−53739
SAASTP0028−53740
SAASTP0029−53741
Cumulative species
Number of intercepts
0
200
400
600
800
1000

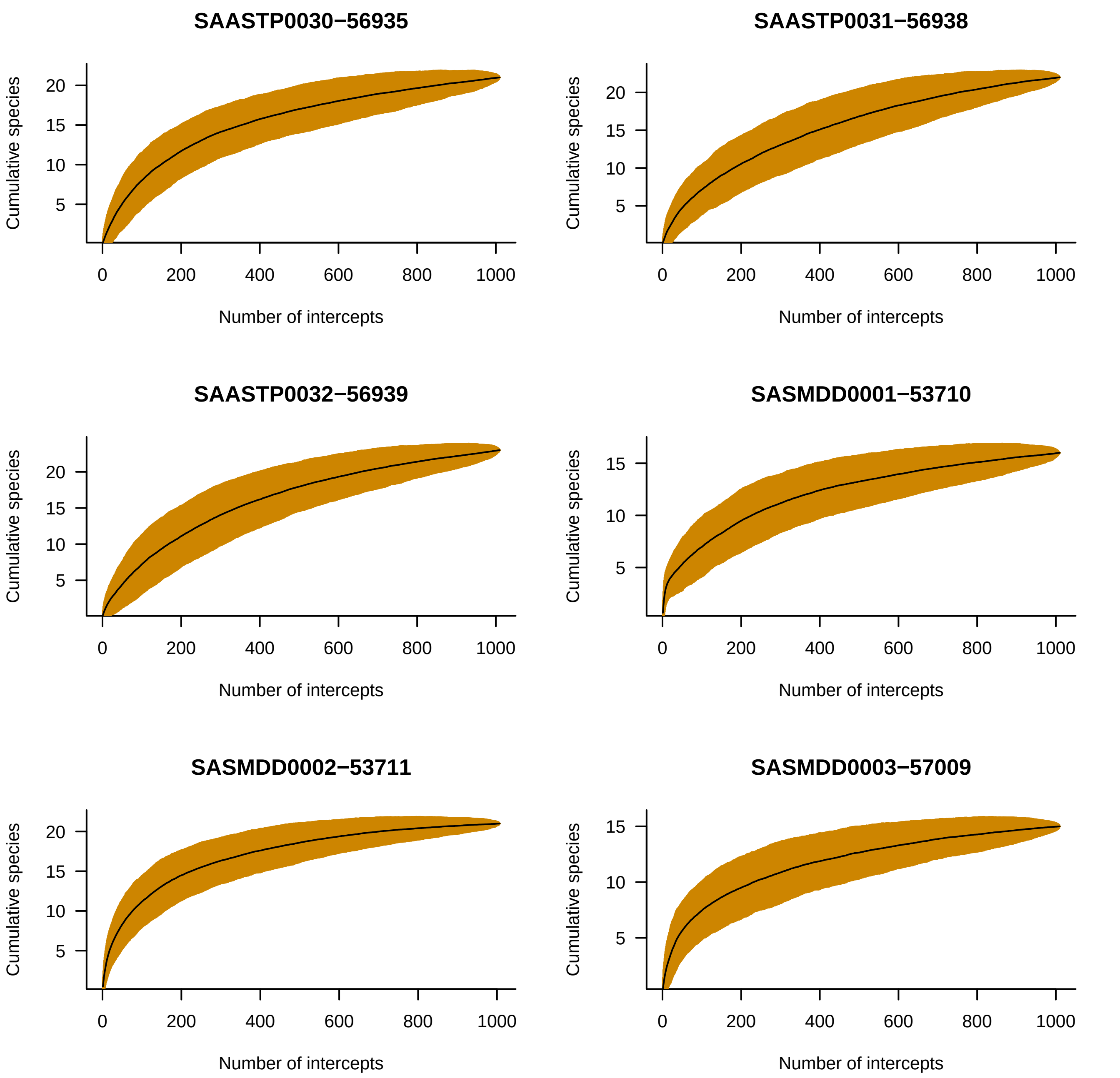
SAASTP0030−56935
SAASTP0031−56938
SAASTP0032−56939
SASMDD0001−53710
SASMDD0002−53711
SASMDD0003−57009
Cumulative species
Number of intercepts
0
200
400
600
800
1000
5
10
15
20

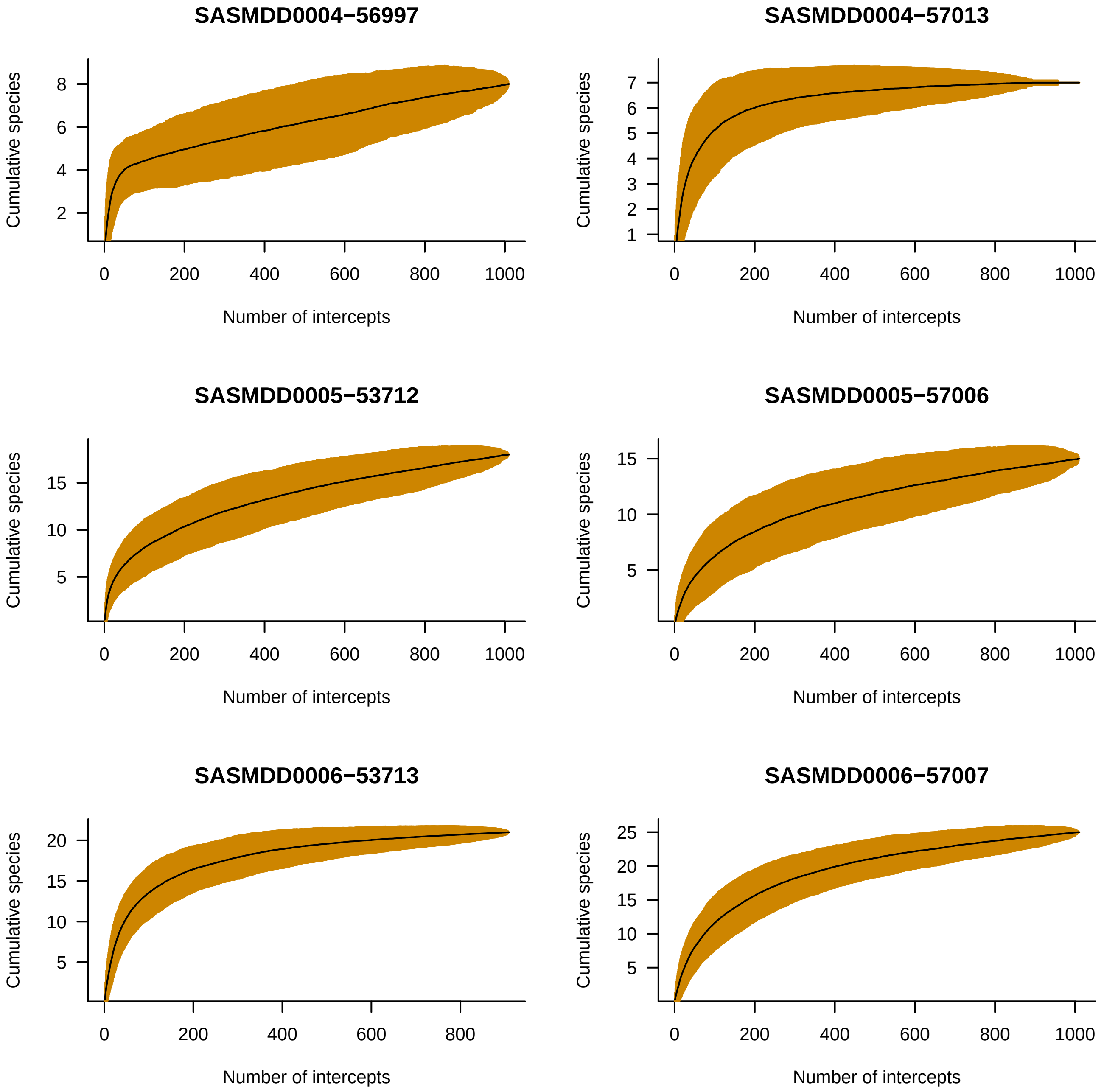
SASMDD0004−56997
SASMDD0004−57013
SASMDD0005−53712
SASMDD0005−57006
SASMDD0006−53713
SASMDD0006−57007
Cumulative species
Number of intercepts
0
200
400
600
800
1000
2
4
6
8
1
3
5
7
10
15
20
25

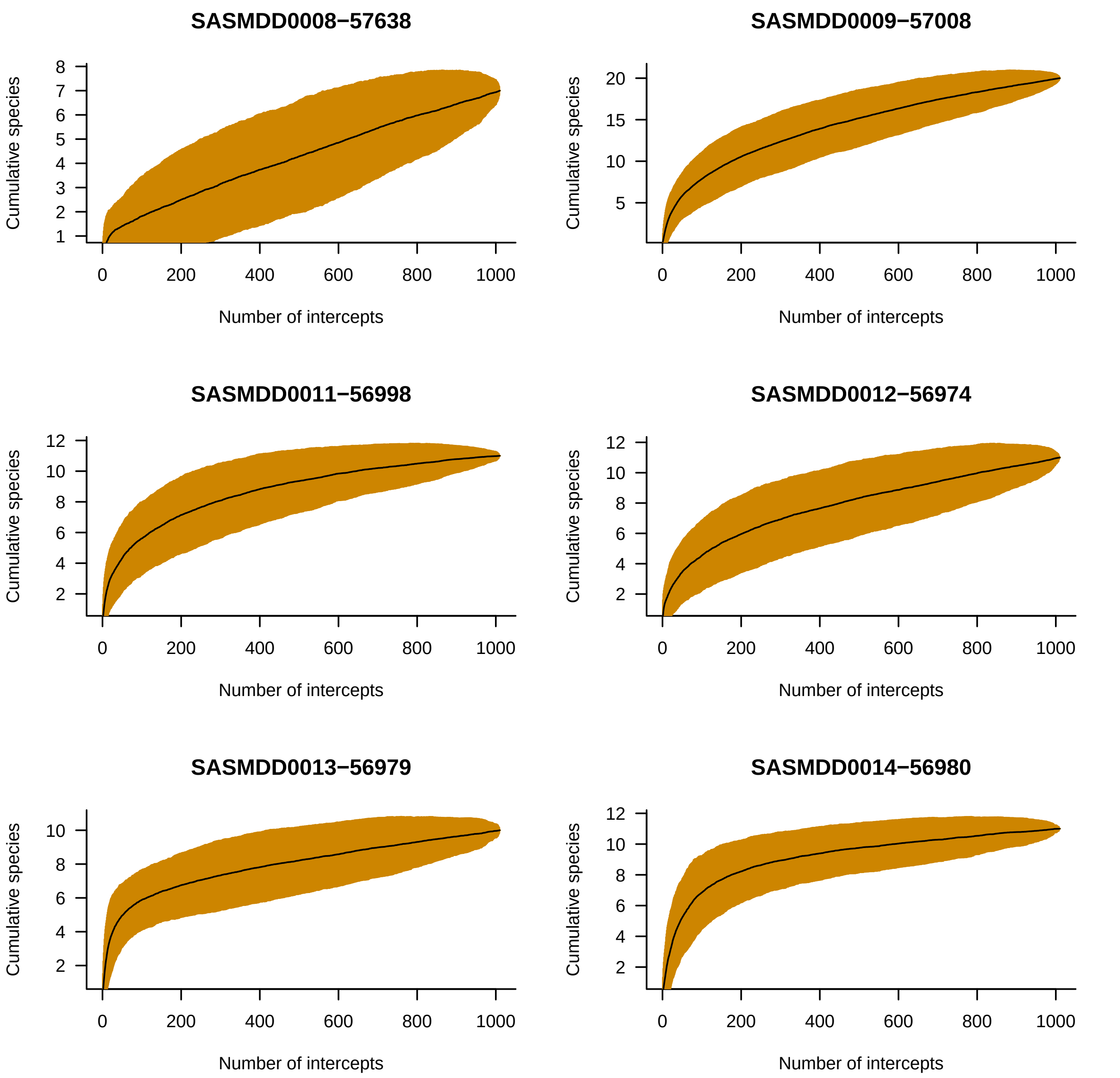
SASMDD0008−57638
Cumulative species
1 2 3 4 5 6 7 8
0 200 400 600 800 1000
Number of intercepts
SASMDD0009−57008
Cumulative species
5 10 15 20
0 200 400 600 800 1000
Number of intercepts
SASMDD0011−56998
Cumulative species
2 4 6 8 10 12
0 200 400 600 800 1000
Number of intercepts
SASMDD0012−56974
Cumulative species
2 4 6 8 10 12
0 200 400 600 800 1000
Number of intercepts
SASMDD0013−56979
Cumulative species
2 4 6 8 10
0 200 400 600 800 1000
Number of intercepts
SASMDD0014−56980
Cumulative species
2 4 6 8 10 12
0 200 400 600 800 1000
Number of intercepts

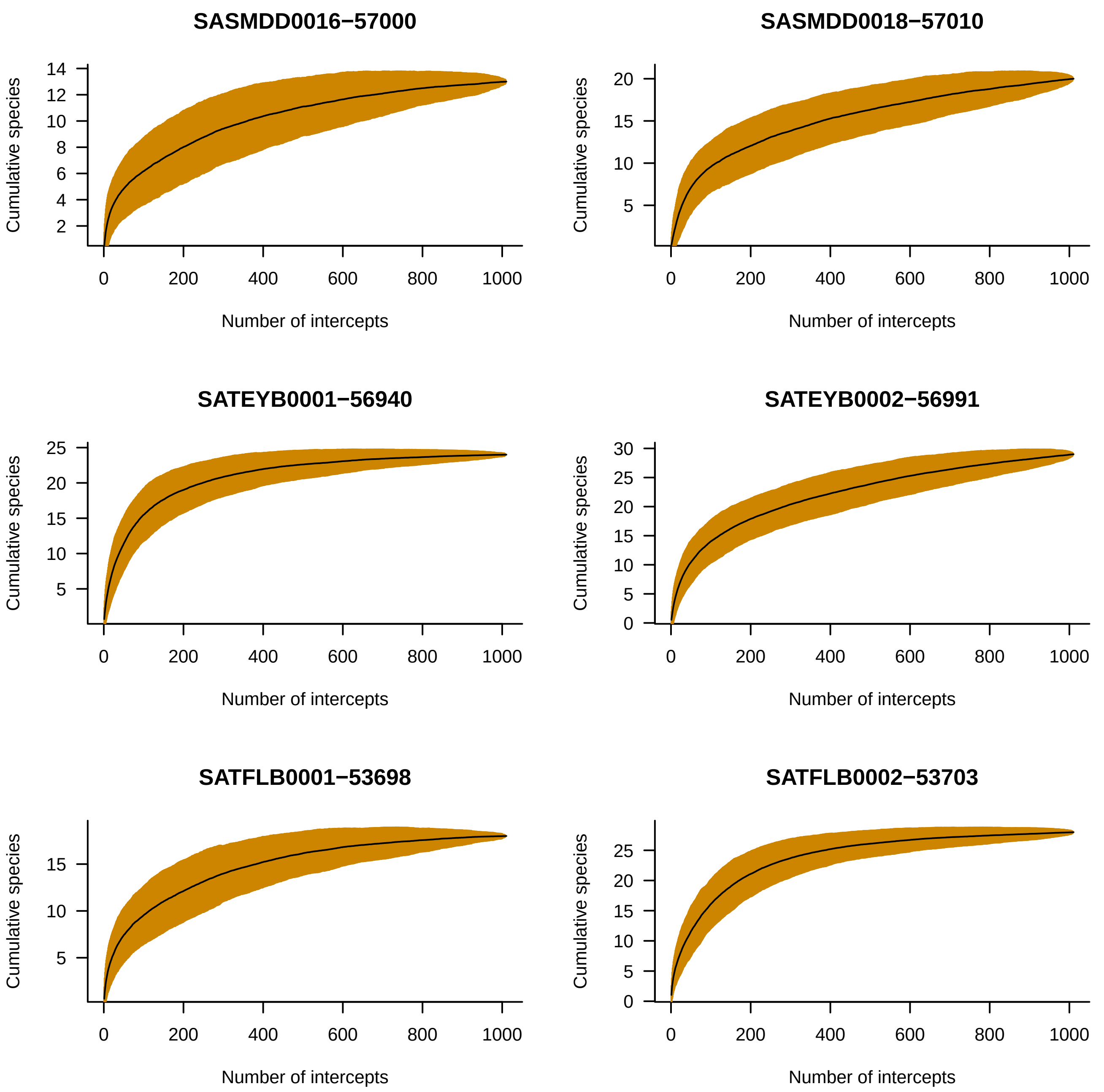
SASMDD0016−57000
SASMDD0018−57010
SATEYB0001−56940
SATEYB0002−56991
SATFLB0001−53698
SATFLB0002−53703
Cumulative species
Number of intercepts
0
200
400
600
800
1000

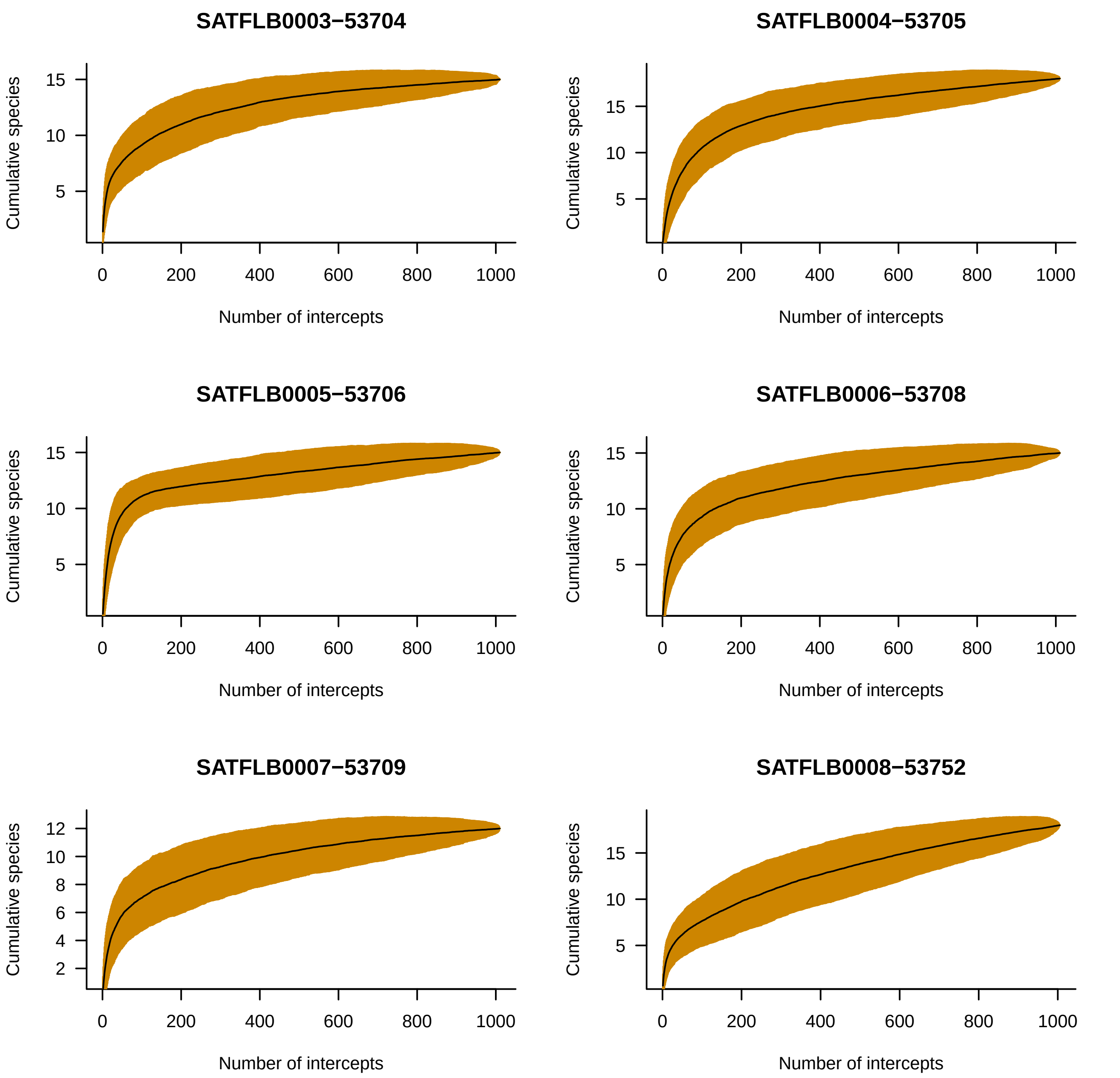
SATFLB0003−53704
SATFLB0004−53705
SATFLB0005−53706
SATFLB0006−53708
SATFLB0007−53709
SATFLB0008−53752
Cumulative species
Number of intercepts
0
200
400
600
800
1000
5
10
15
2
4
6
8
12

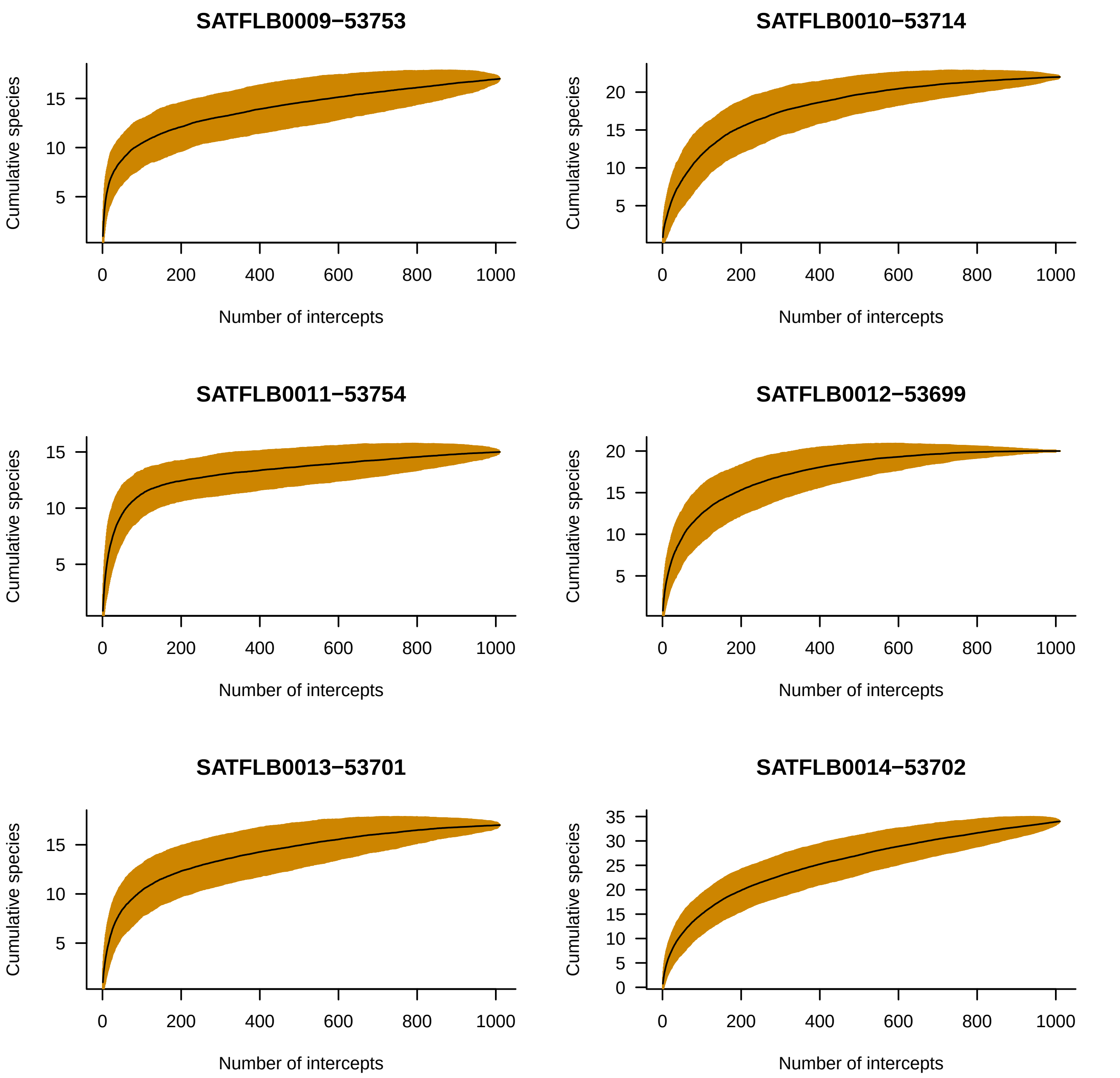
SATFLB0009−53753
SATFLB0010−53714
SATFLB0011−53754
SATFLB0012−53699
SATFLB0013−53701
SATFLB0014−53702
Cumulative species
Number of intercepts
0
200
400
600
800
1000
5
10
15
20
25
30
35

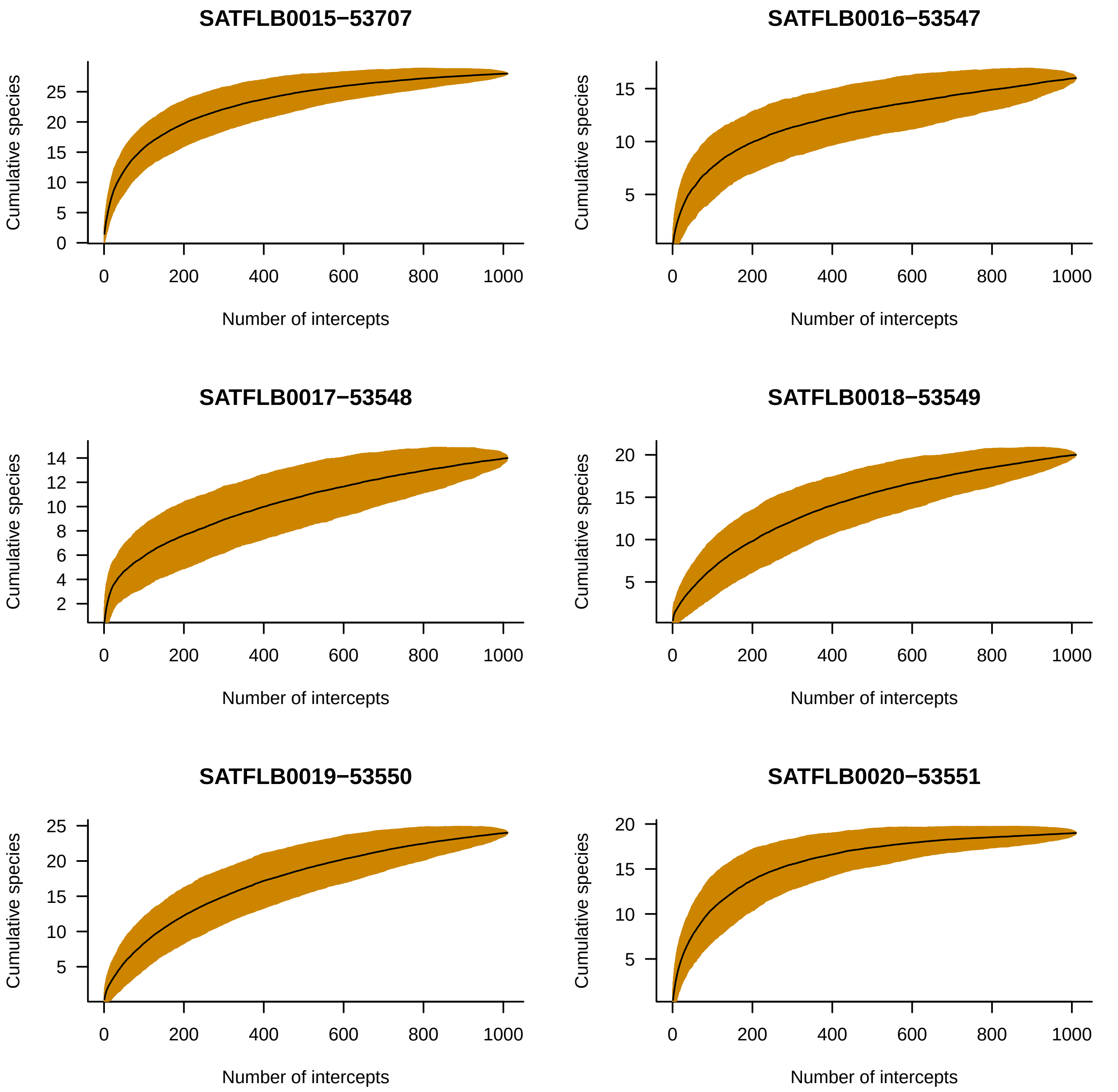
SATFLB0015−53707
SATFLB0016−53547
SATFLB0017−53548
SATFLB0018−53549
SATFLB0019−53550
SATFLB0020−53551
Cumulative species
Number of intercepts
0
200
400
600
800
1000

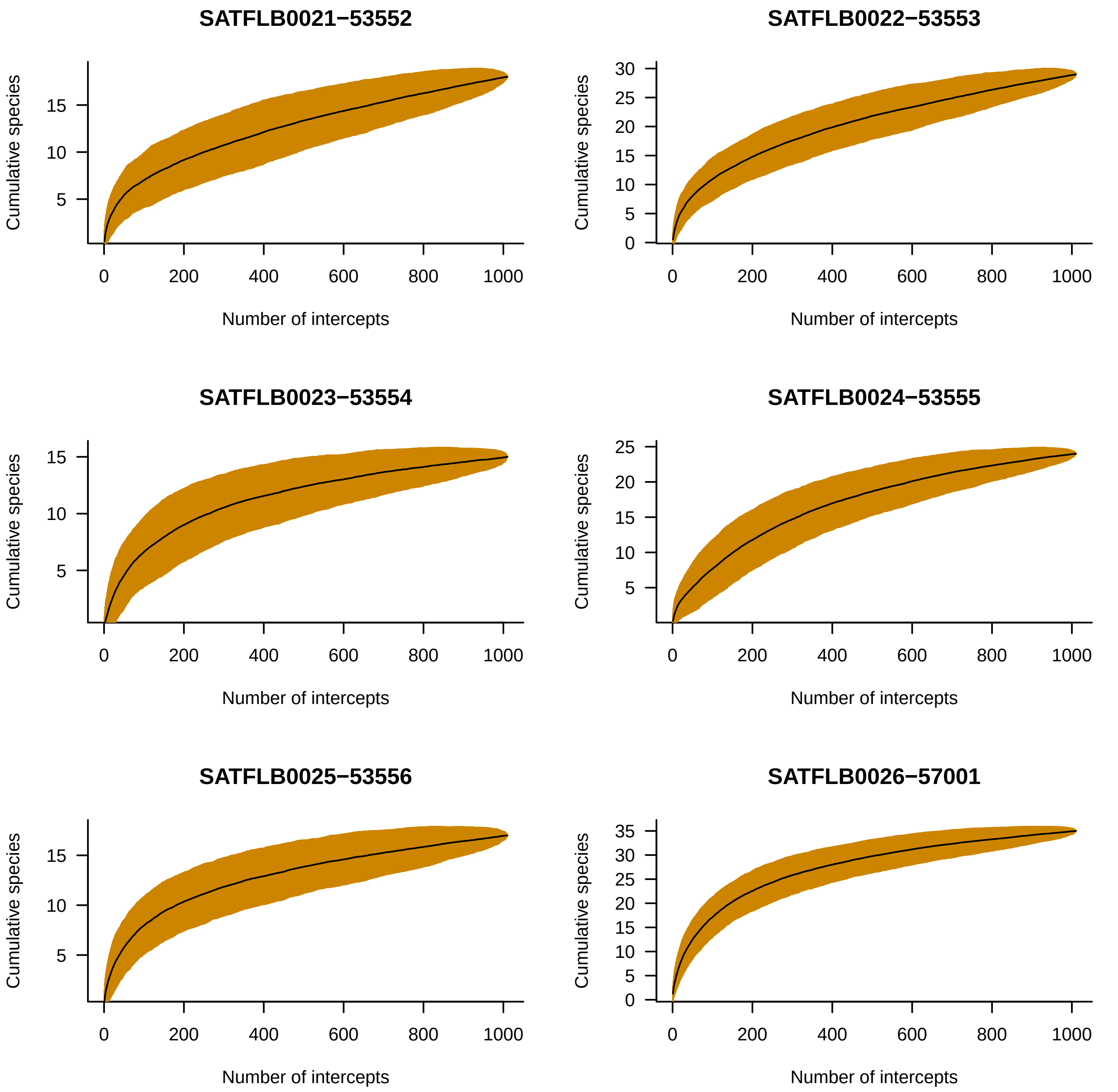

SATFLB0021−53552
SATFLB0022−53553
SATFLB0023−53554
SATFLB0024−53555
SATFLB0025−53556
SATFLB0026−57001
Cumulative species
Number of intercepts
0
200
400
600
800
1000
5
10
15
20
25
30
35

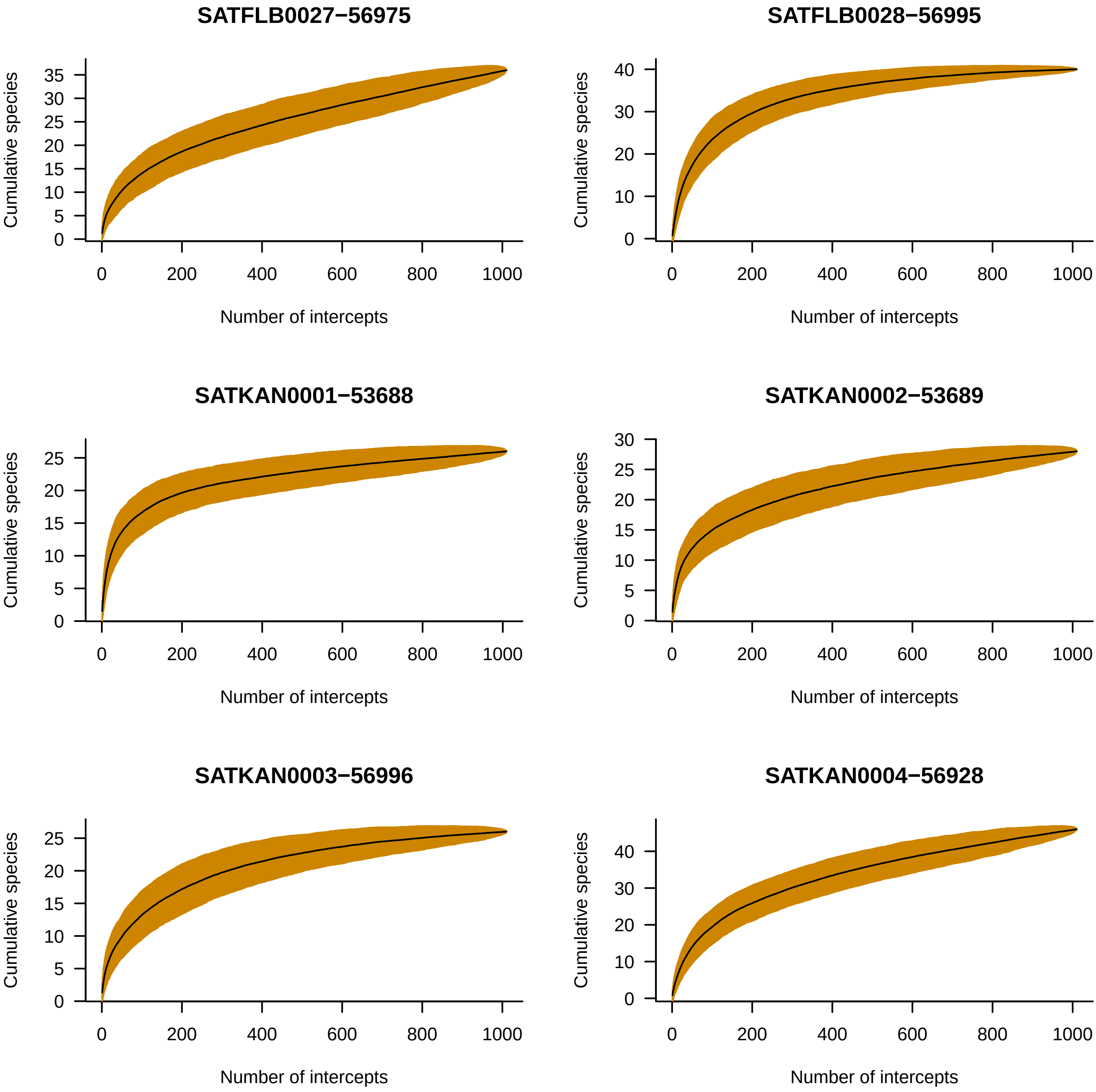

SATFLB0027−56975
SATFLB0028−56995
SATKAN0001−53688
SATKAN0002−53689
SATKAN0003−56996
SATKAN0004−56928
Cumulative species
Number of intercepts
0
200
400
600
800
1000
0
5
10
15
20
25
30
35
40

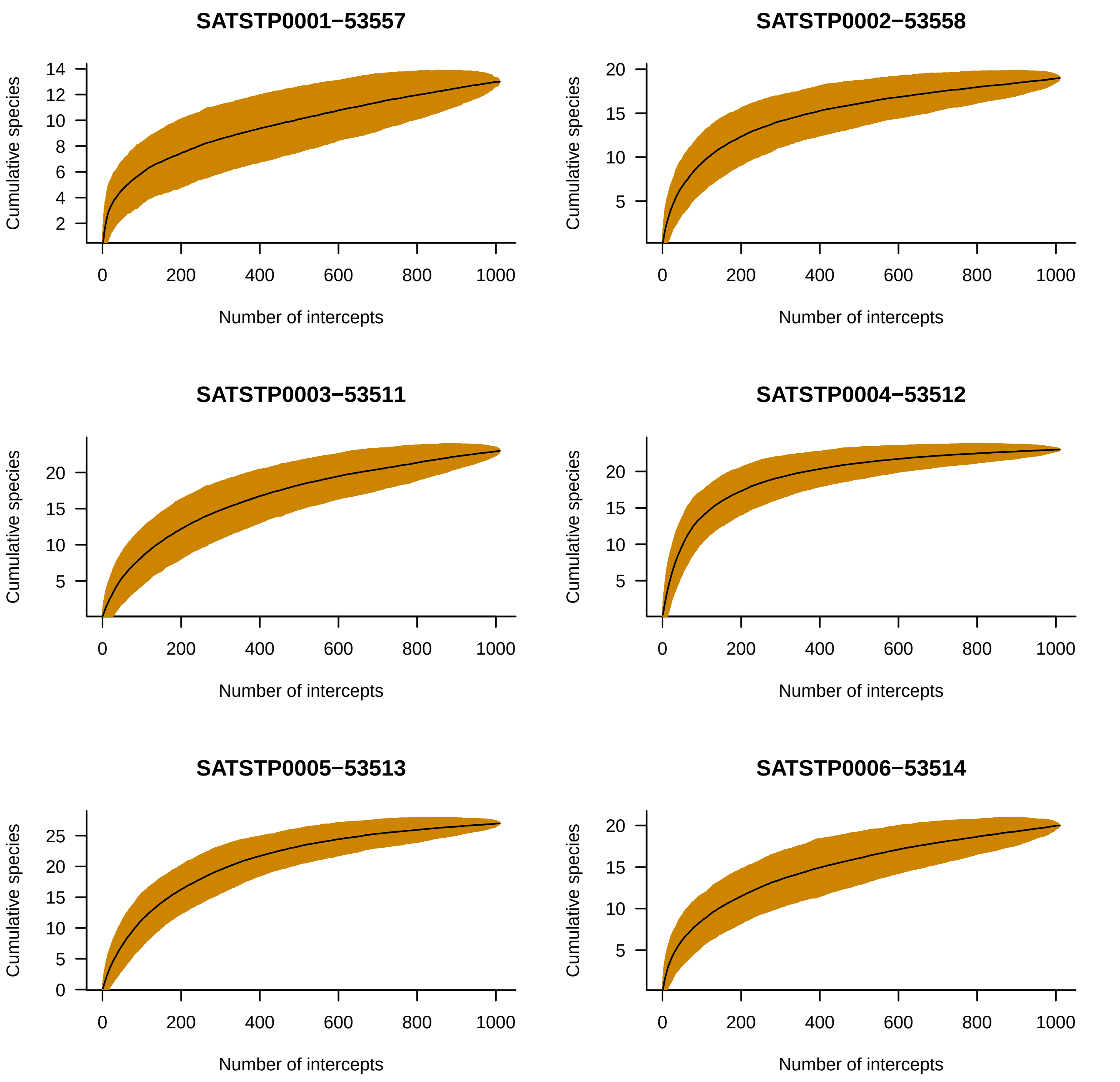
SATSTP0001−53557
SATSTP0002−53558
SATSTP0003−53511
SATSTP0004−53512
SATSTP0005−53513
SATSTP0006−53514
Cumulative species
Number of intercepts
0 200 400 600 800 1000
2 4 6 8 10 12 14
5 10 15 20
5 10 15 20
5 10 15 20
0 5 10 15 20 25
5 10 15 20

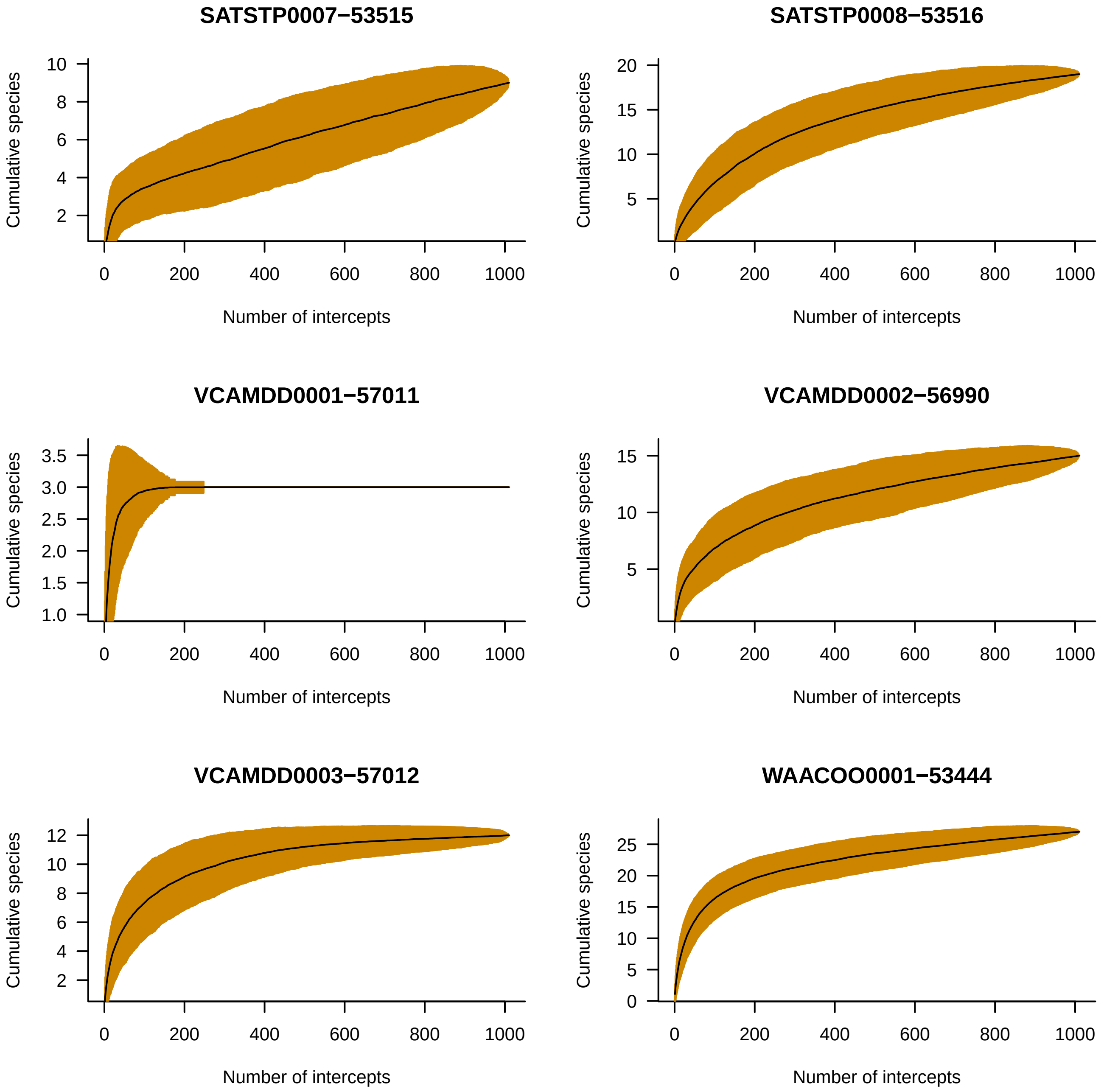
SATSTP0007−53515
SATSTP0008−53516
VCAMDD0001−57011
VCAMDD0002−56990
VCAMDD0003−57012
WAACOO0001−53444
Cumulative species
Number of intercepts
0 200 400 600 800 1000
2 4 6 8 10
5 10 15 20
1.0 1.5 2.0 2.5 3.0 3.5
5 10 15
2 4 6 8 10 12
0 5 10 15 20 25

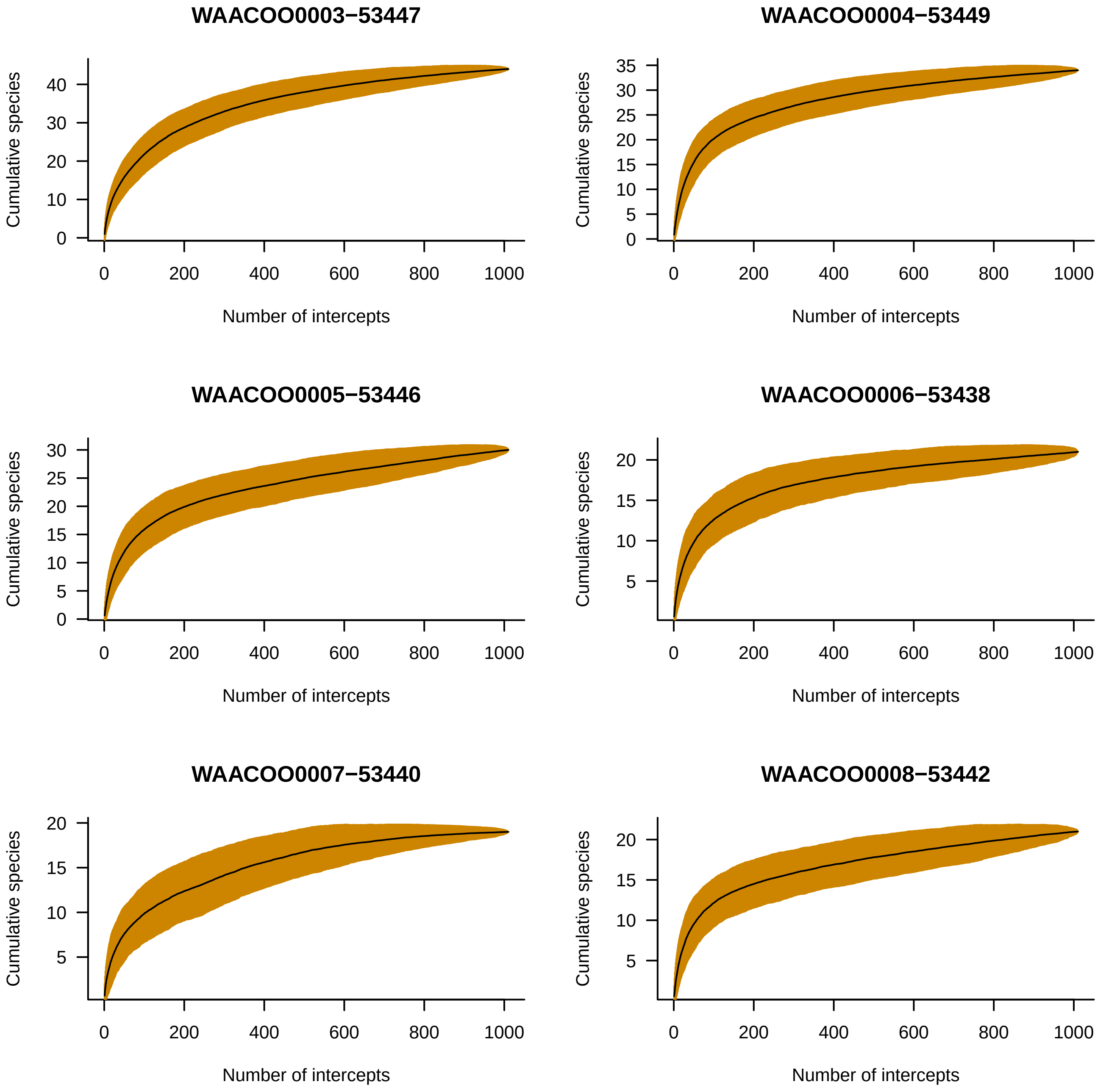
WAACOO0003−53447
WAACOO0004−53449
WAACOO0005−53446
WAACOO0006−53438
WAACOO0007−53440
WAACOO0008−53442
Cumulative species
Number of intercepts
0
200
400
600
800
1000
0
5
10
15
20
25
30
35
40

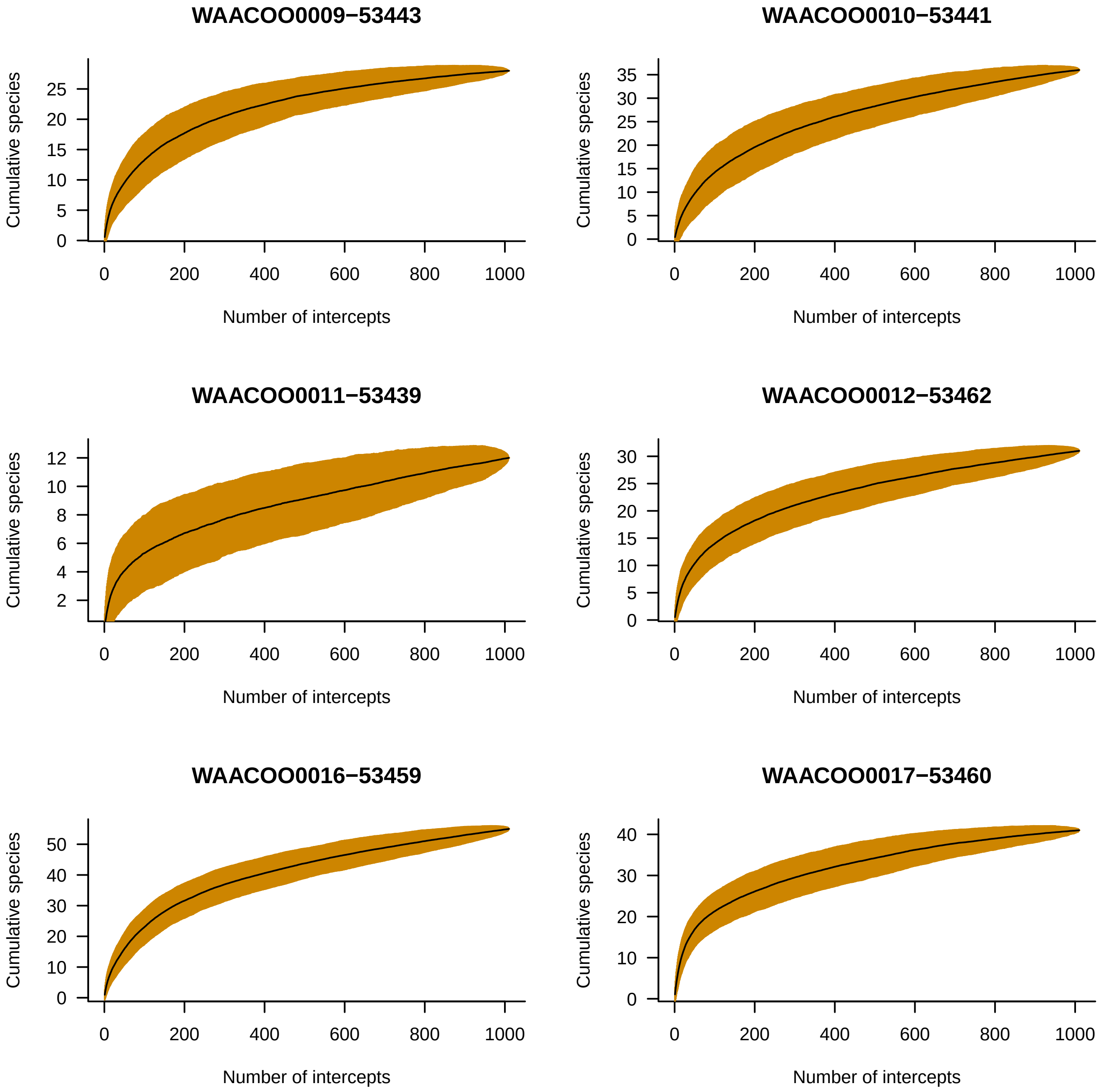
WAACOO0009−53443
WAACOO0010−53441
WAACOO0011−53439
WAACOO0012−53462
WAACOO0016−53459
WAACOO0017−53460
Cumulative species
Number of intercepts
0
200
400
600
800
1000

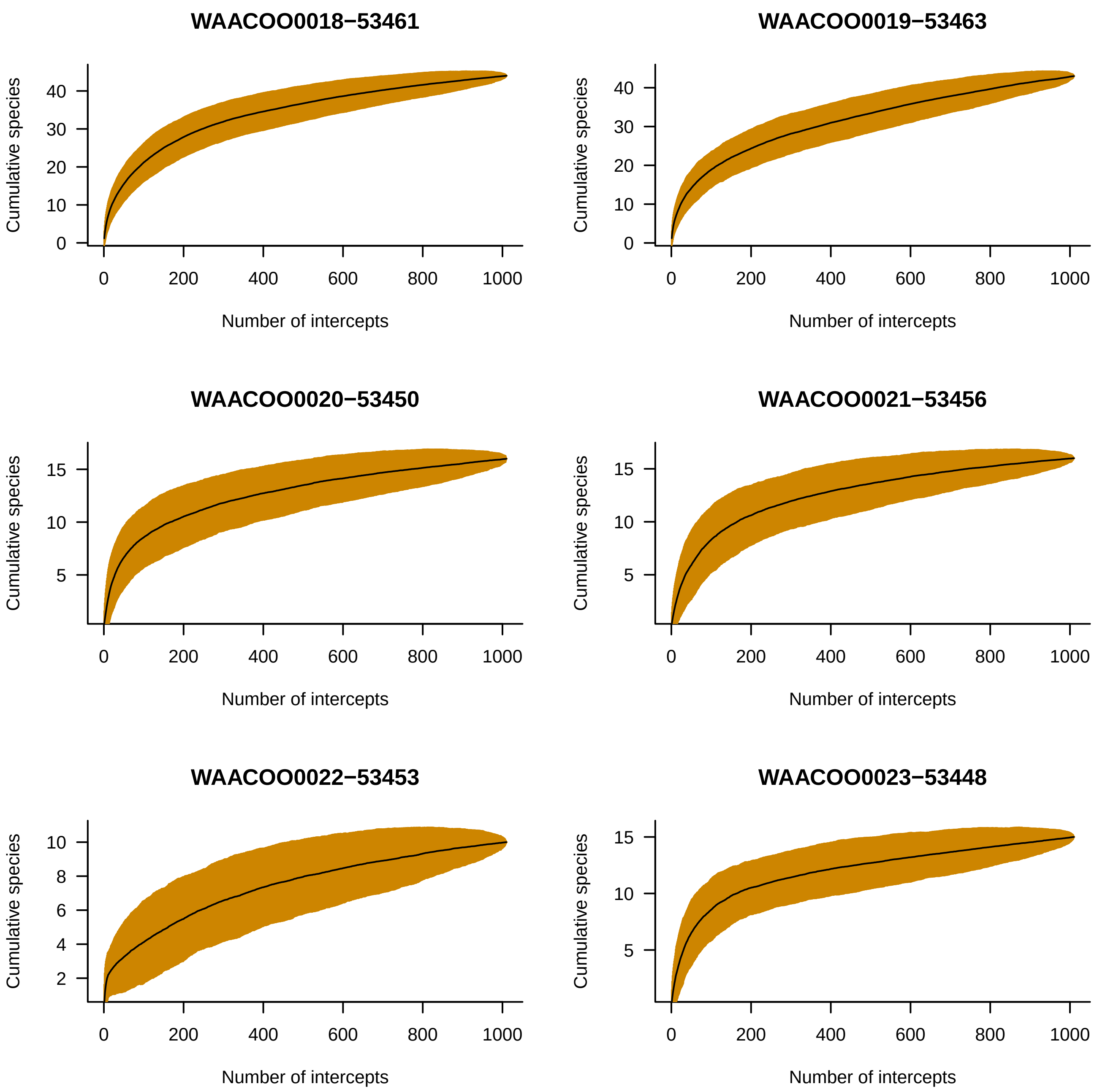
WAACOO0018−53461
WAACOO0019−53463
WAACOO0020−53450
WAACOO0021−53456
WAACOO0022−53453
WAACOO0023−53448
Cumulative species
Number of intercepts
0
200
400
600
800
1000
0
2
4
5
6
8
10
15
20
30
40

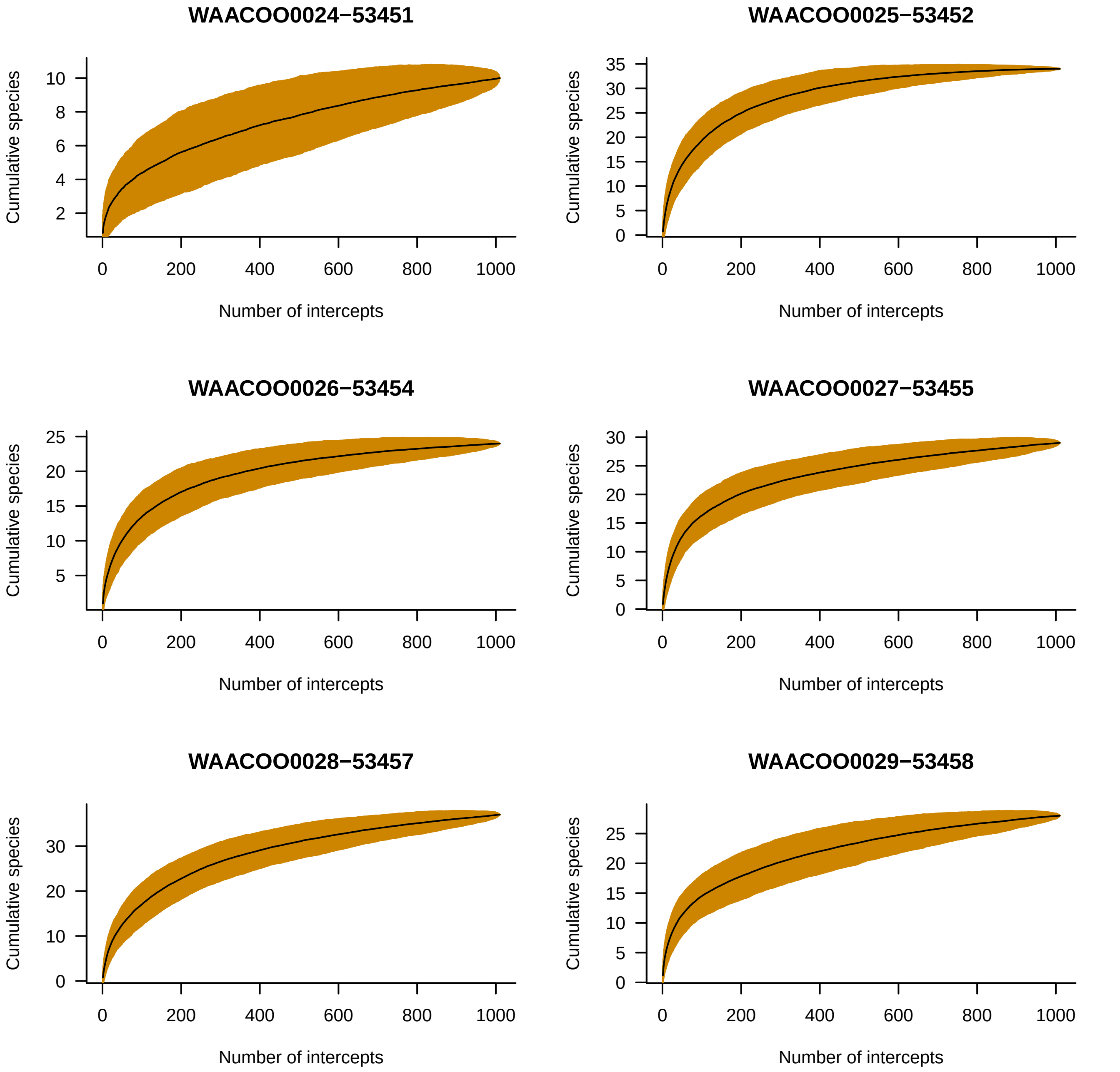

WAACOO0024−53451
WAACOO0025−53452
WAACOO0026−53454
WAACOO0027−53455
WAACOO0028−53457
WAACOO0029−53458
Cumulative species
Number of intercepts
0
200
400
600
800
1000

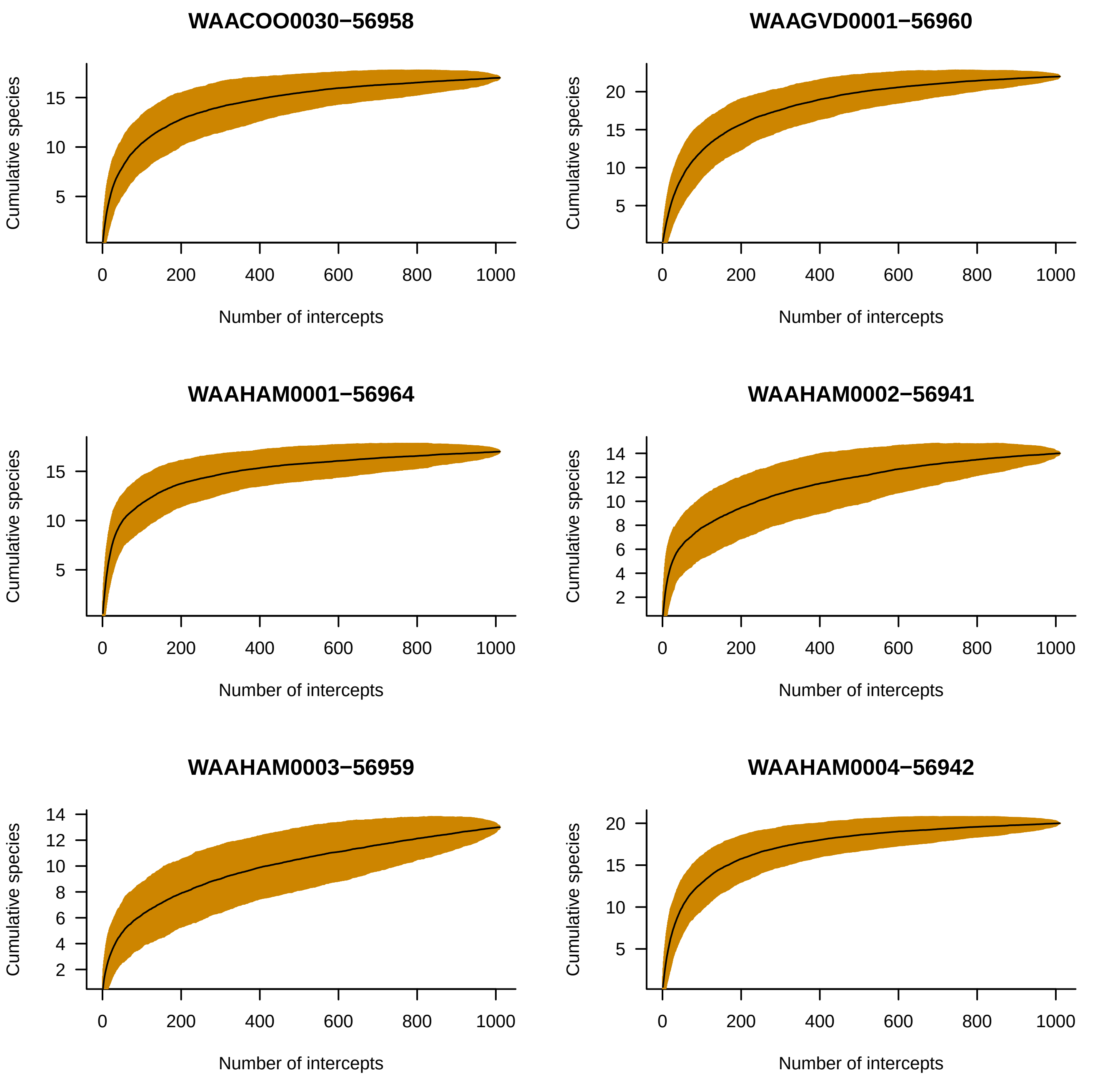
WAACOO0030−56958
WAAGVD0001−56960
WAAHAM0001−56964
WAAHAM0002−56941
WAAHAM0003−56959
WAAHAM0004−56942
Cumulative species
Number of intercepts
0
200
400
600
800
1000

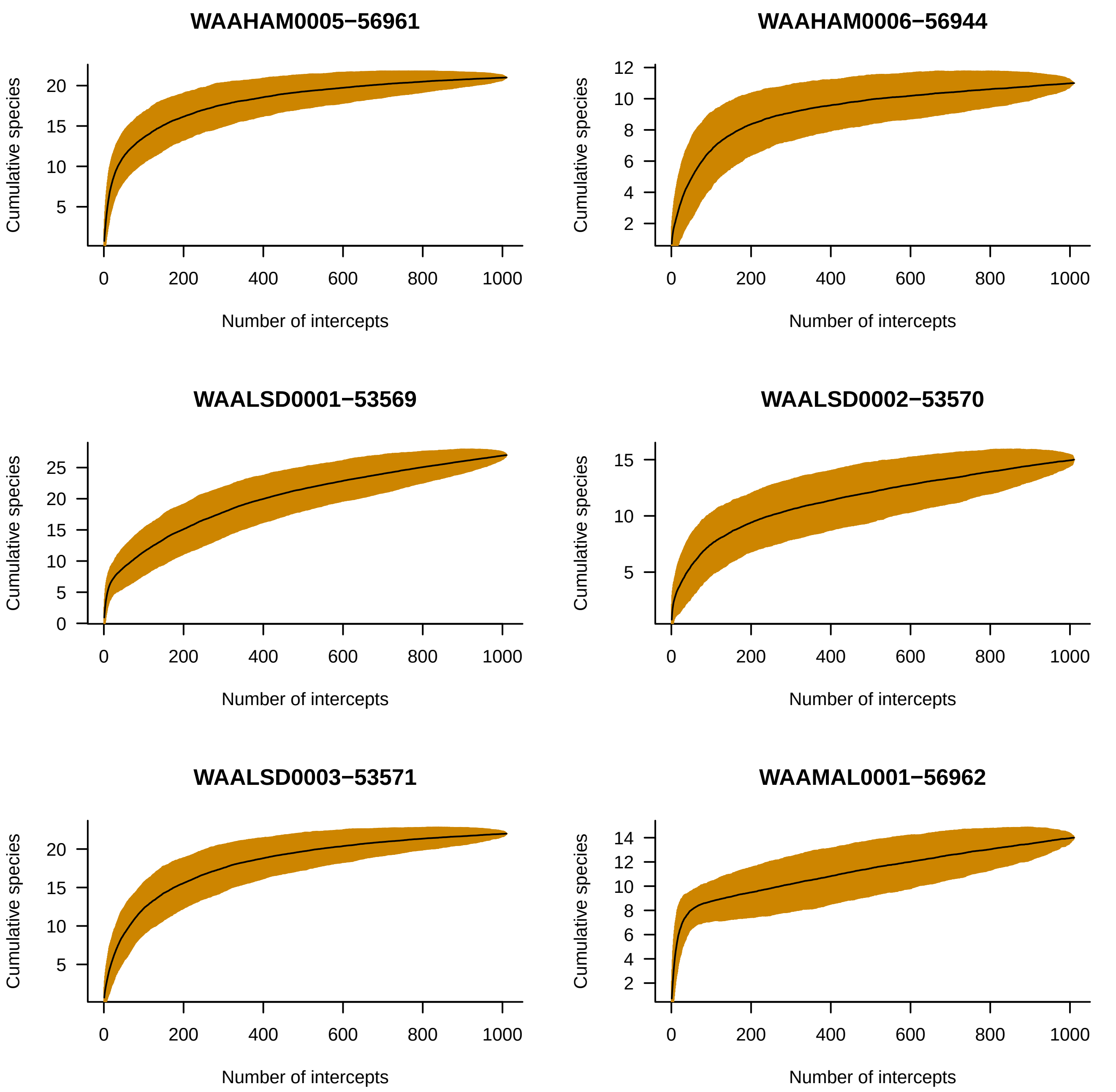
WAAHAM0005−56961
WAAHAM0006−56944
WAALSD0001−53569
WAALSD0002−53570
WAALSD0003−53571
WAAMAL0001−56962
Cumulative species
Number of intercepts
0 200 400 600 800 1000

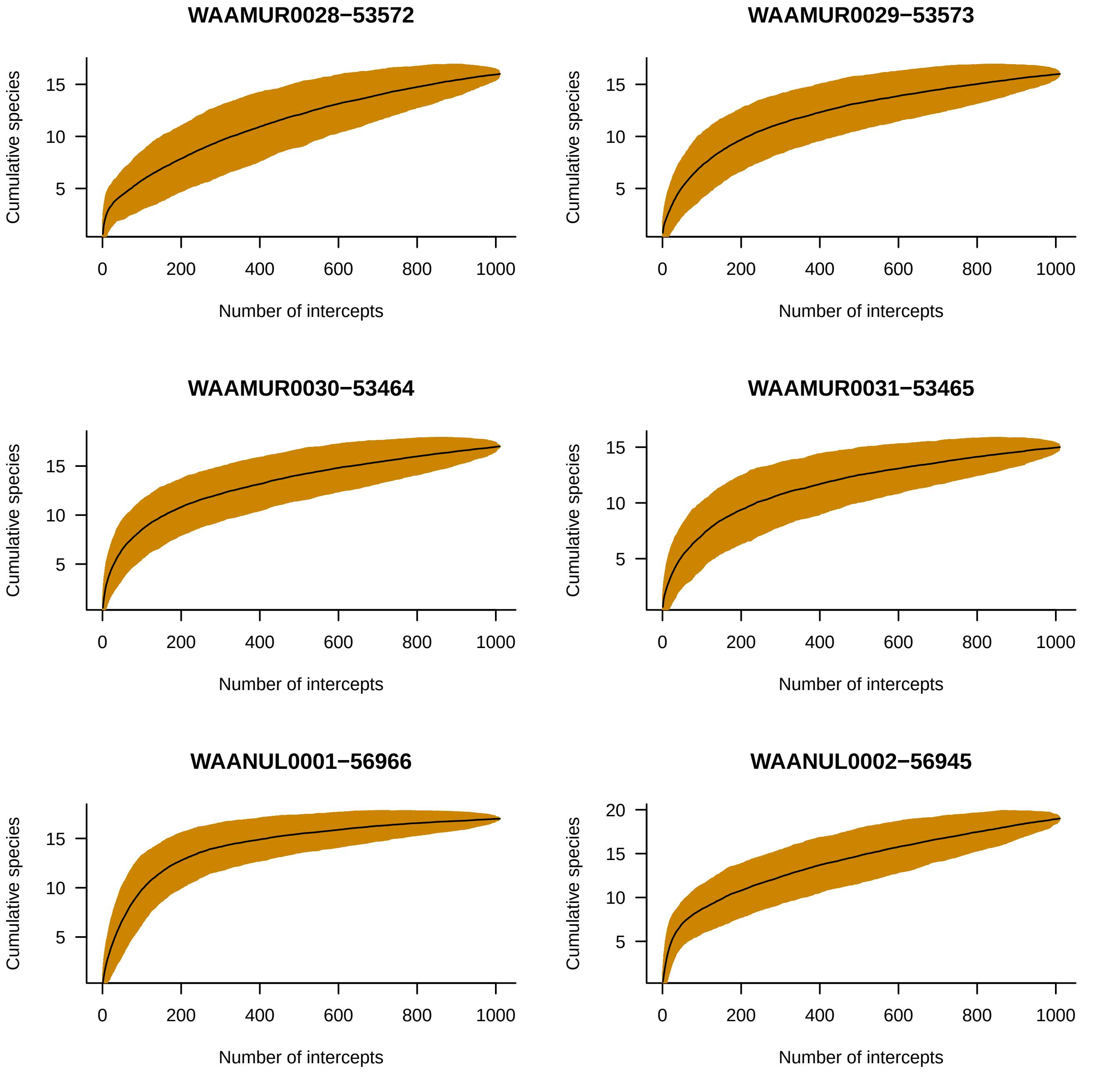
WAAMUR0028−53572
WAAMUR0029−53573
WAAMUR0030−53464
WAAMUR0031−53465
WAANUL0001−56966
WAANUL0002−56945
Cumulative species
Number of intercepts
0
200
400
600
800
1000
5
10
15
20

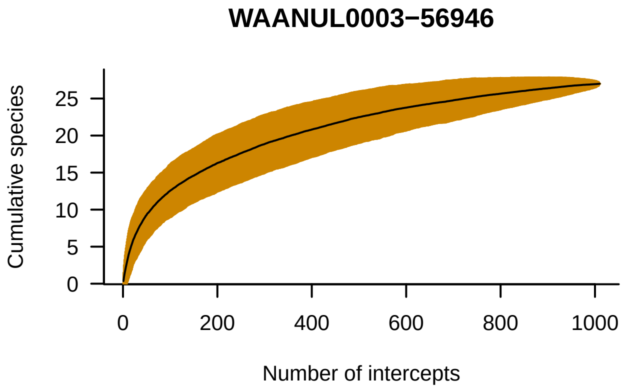
WAANUL0003−56946
Cumulative species
0
5
10
15
20
25
0
200
400
600
800
1000
Number of intercepts

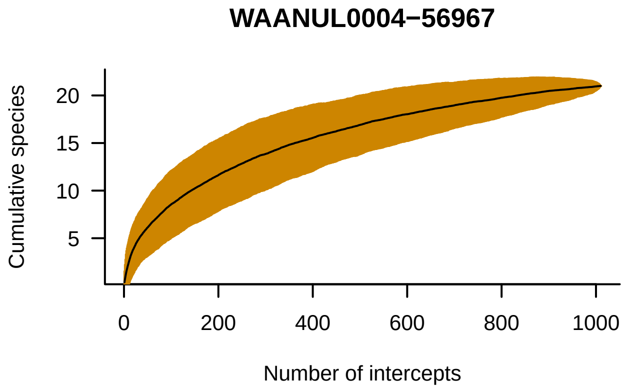
WAANUL0004−56967
Cumulative species
5
10
15
20
0
200
400
600
800
1000
Number of intercepts

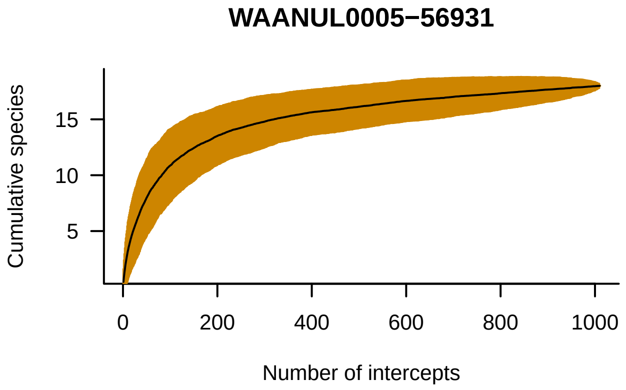
WAANUL0005−56931
Cumulative species
5
10
15
0
200
400
600
800
1000
Number of intercepts

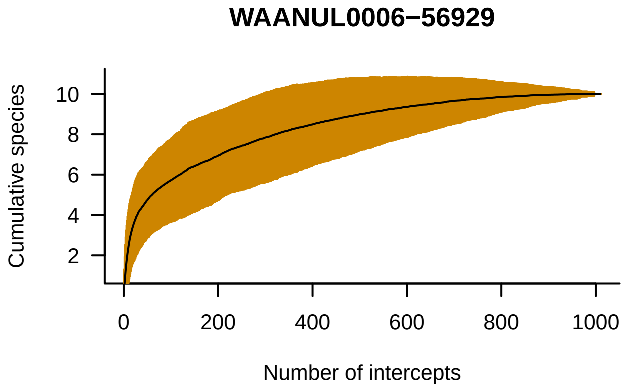
WAANUL0006−56929
Cumulative species
2
4
6
8
10
0
200
400
600
800
1000
Number of intercepts

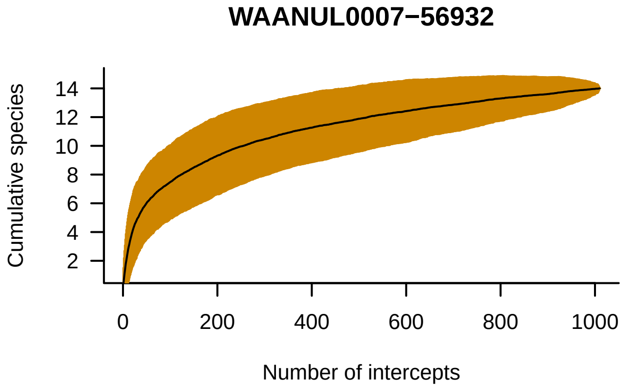
WAANUL0007−56932
Cumulative species
2
4
6
8
10
12
14
0
200
400
600
800
1000
Number of intercepts

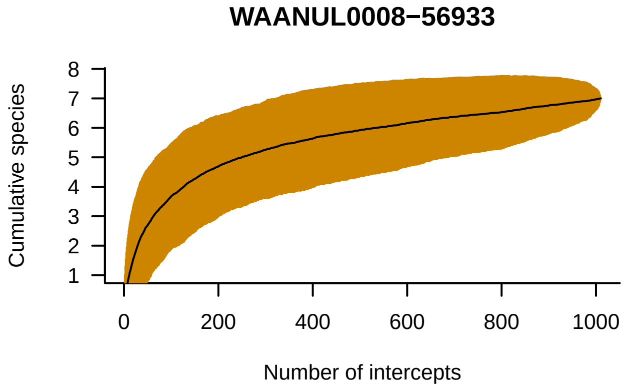
WAANUL0008−56933
Cumulative species
1
2
3
4
5
6
7
8
0
200
400
600
800
1000
Number of intercepts

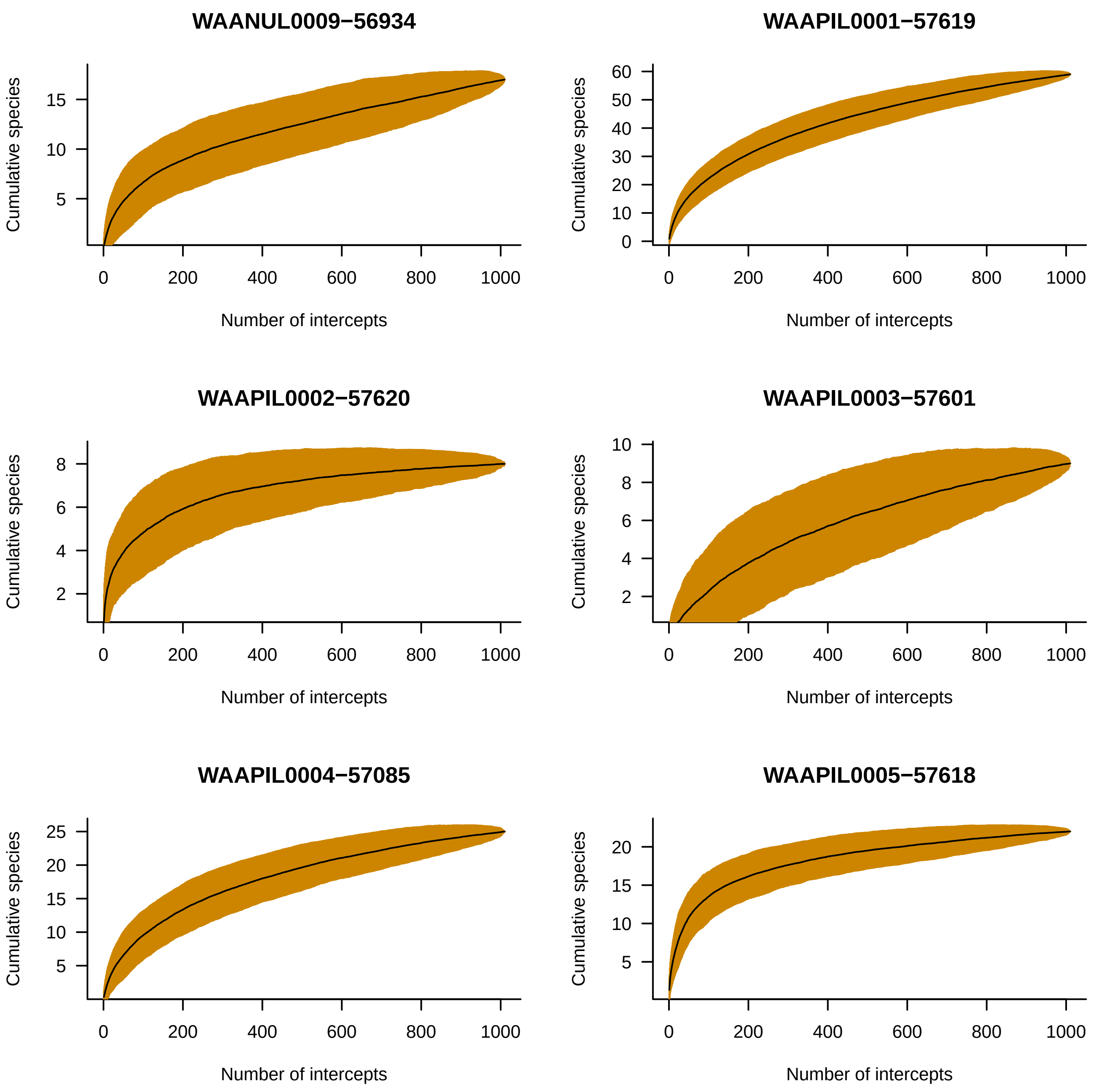
WAANUL0009−56934
WAAPIL0001−57619
WAAPIL0002−57620
WAAPIL0003−57601
WAAPIL0004−57085
WAAPIL0005−57618
Cumulative species
Number of intercepts
0
200
400
600
800
1000

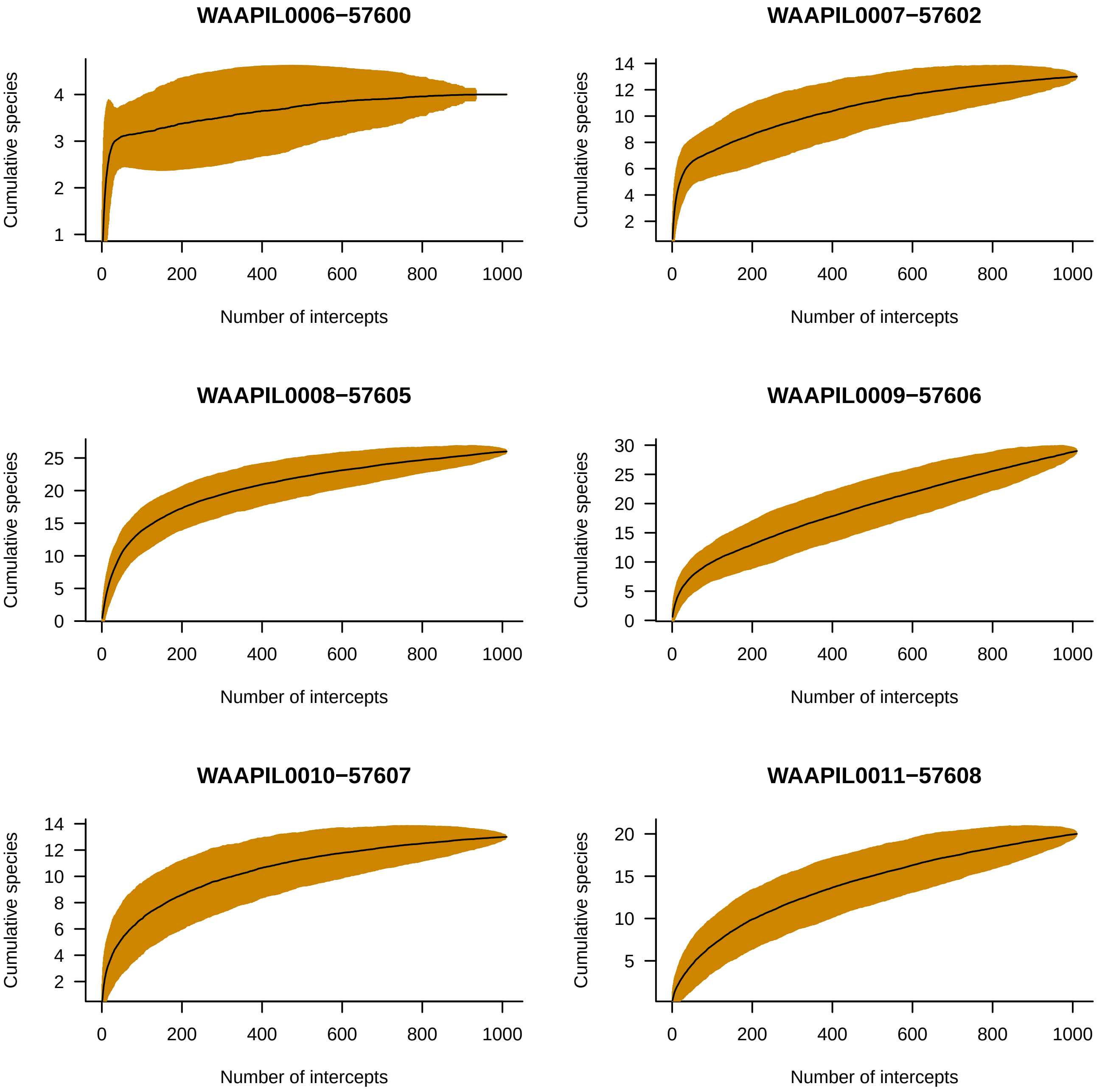
WAAPIL0006−57600
WAAPIL0007−57602
WAAPIL0008−57605
WAAPIL0009−57606
WAAPIL0010−57607
WAAPIL0011−57608
Cumulative species
Number of intercepts
0
200
400
600
800
1000

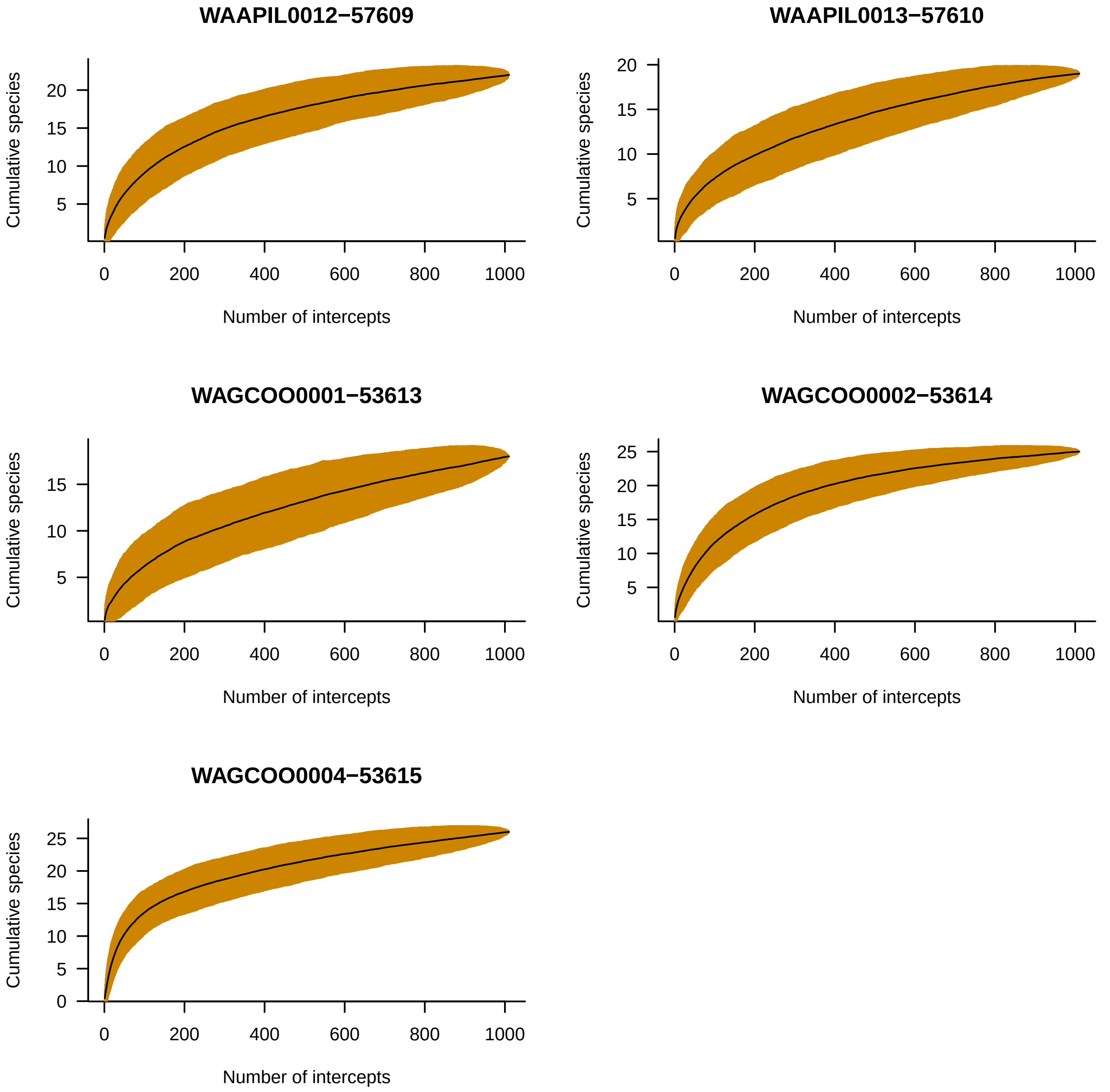
WAAPIL0012−57609
WAAPIL0013−57610
WAGCOO0001−53613
WAGCOO0002−53614
WAGCOO0004−53615
Cumulative species
Number of intercepts
0
200
400
600
800
1000
5
10
15
20
25
