# Supplementary material for: Opportunities for Integrated Ecological Analysis across Inland Australia with Standardised Data from Ausplots Rangelands
Source: PLoS One. 2017 Jan 17;12(1):e0170137. doi: 10.1371/journal.pone.0170137 (PMC5241013; doi:10.1371/journal.pone.0170137)
Supplement: S3 Appendix — Cumulative cover abundance (%CA; Foliage Projective Cover) for species with point intercepts taken across plots. Five most abundant species are labelled. (PDF) [file pone.0170137.s003.pdf]

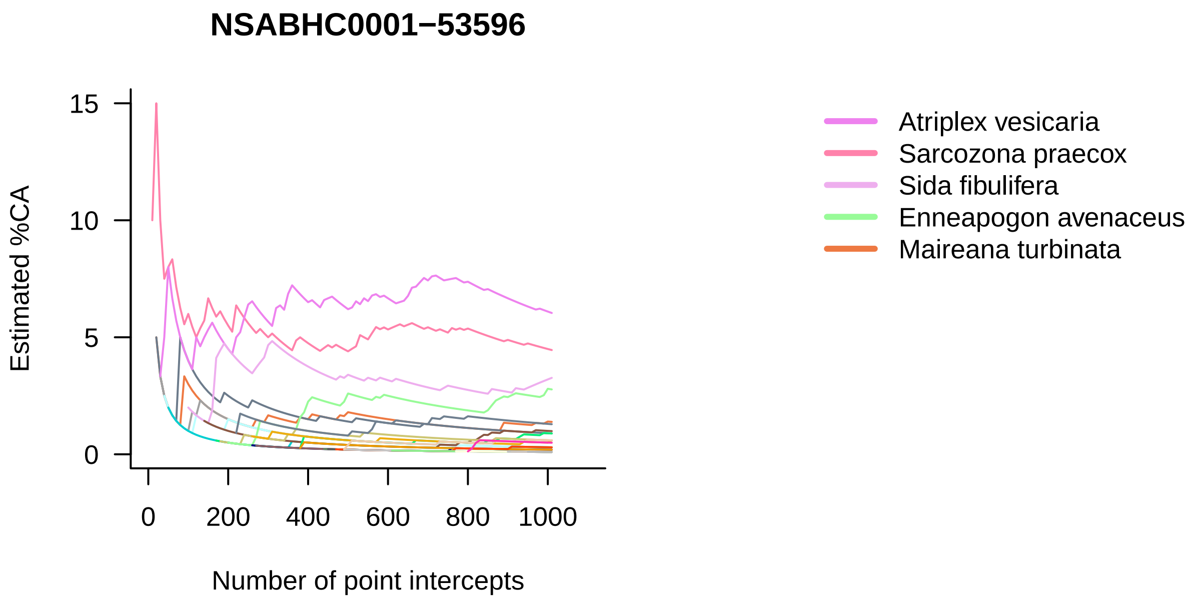
NSABHC0001−53596
Estimated %CA
0
5
10
15
0
200
400
600
800
1000
Number of point intercepts
Atriplex vesicaria
Sarcozona praecox
Sida fibulifera
Enneapogon avenaceus
Maireana turbinata

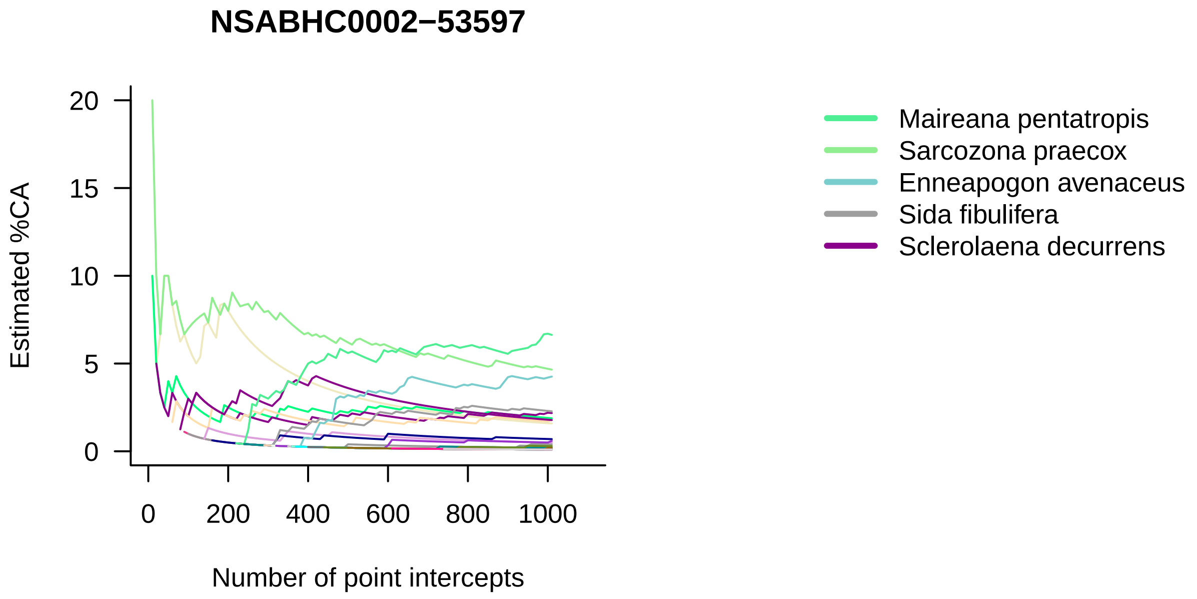
NSABHC0002−53597
Estimated %CA
0
5
10
15
20
0
200
400
600
800
1000
Number of point intercepts
Maireana pentatropis
Sarcozona praecox
Enneapogon avenaceus
Sida fibulifera
Sclerolaena decurrens

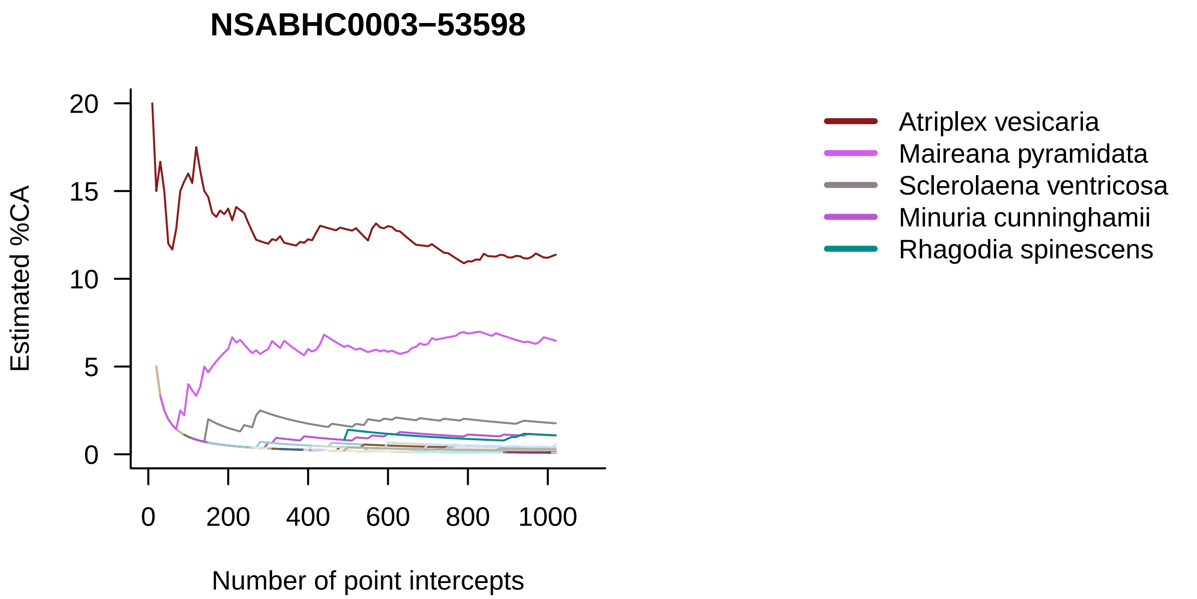
NSABHC0003−53598
Estimated %CA
Number of point intercepts
0
5
10
15
20
0
200
400
600
800
1000
Atriplex vesicaria
Maireana pyramidata
Sclerolaena ventricosa
Minuria cunninghamii
Rhagodia spinescens

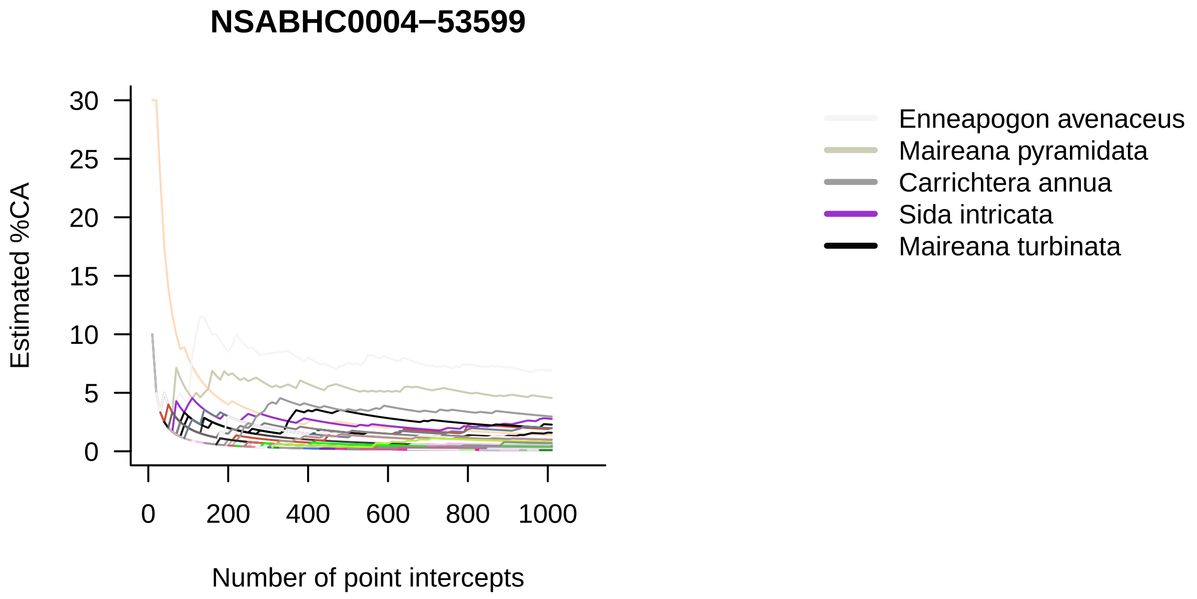
NSABHC0004−53599
Estimated %CA
Number of point intercepts
0
5
10
15
20
25
30
0
200
400
600
800
1000
Enneapogon avenaceus
Maireana pyramidata
Carrichtera annua
Sida intricata
Maireana turbinata

**NSABHC0005−53600**

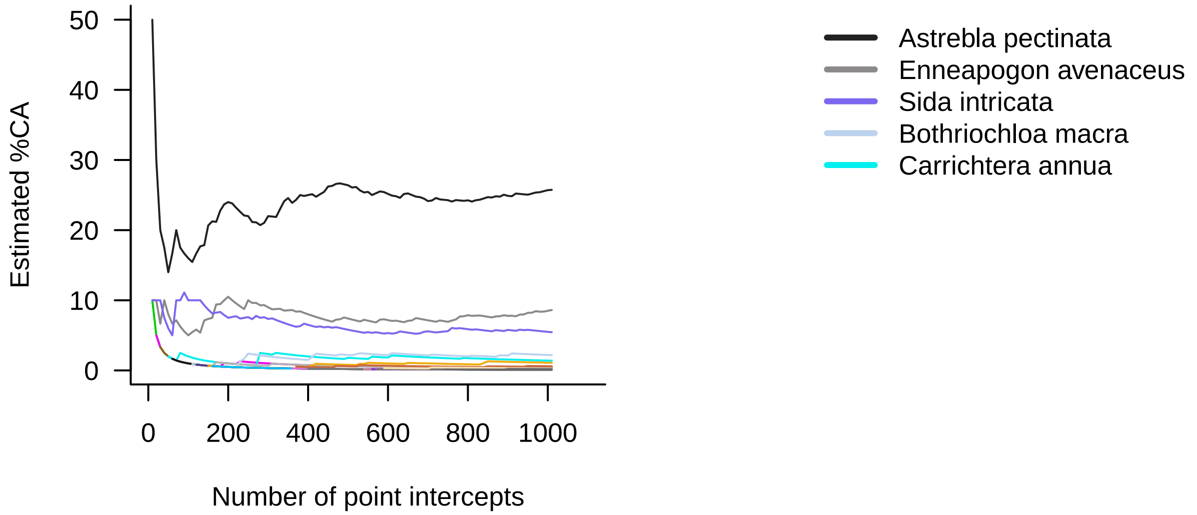


**NSABHC0006−53601**

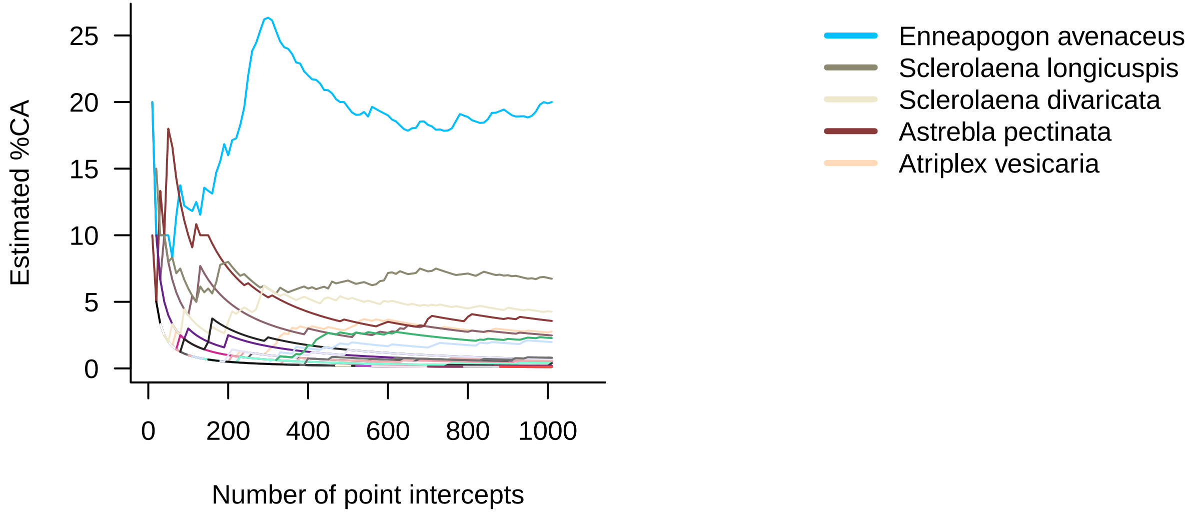

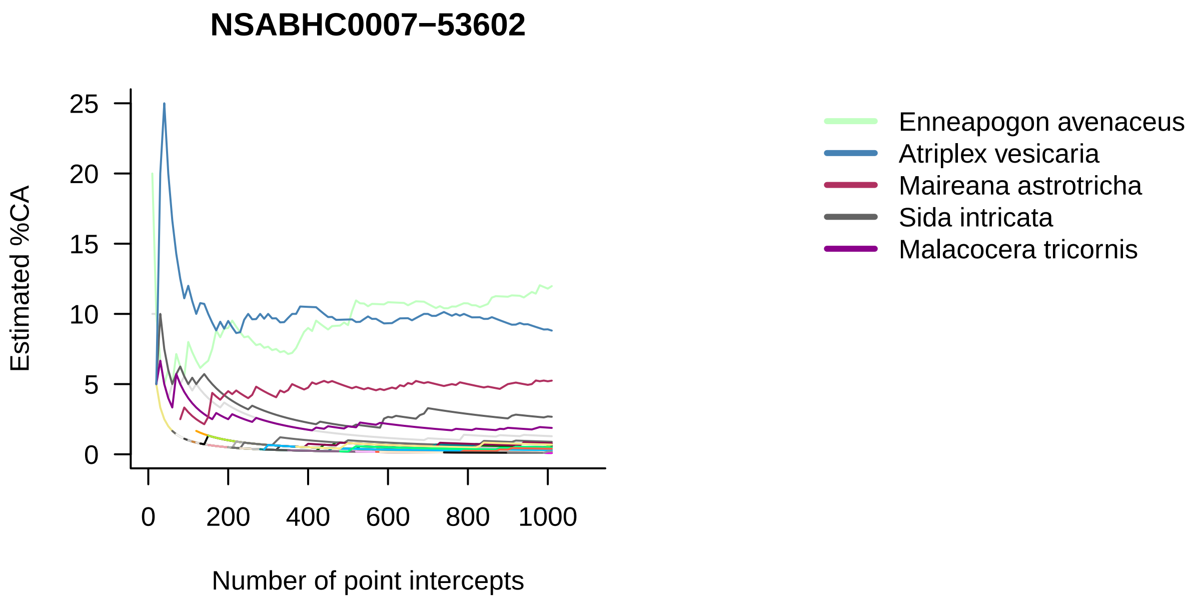
NSABHC0007−53602
Estimated %CA
0
5
10
15
20
25
0
200
400
600
800
1000
Number of point intercepts
Enneapogon avenaceus
Atriplex vesicaria
Maireana astrotricha
Sida intricata
Malacocera tricornis

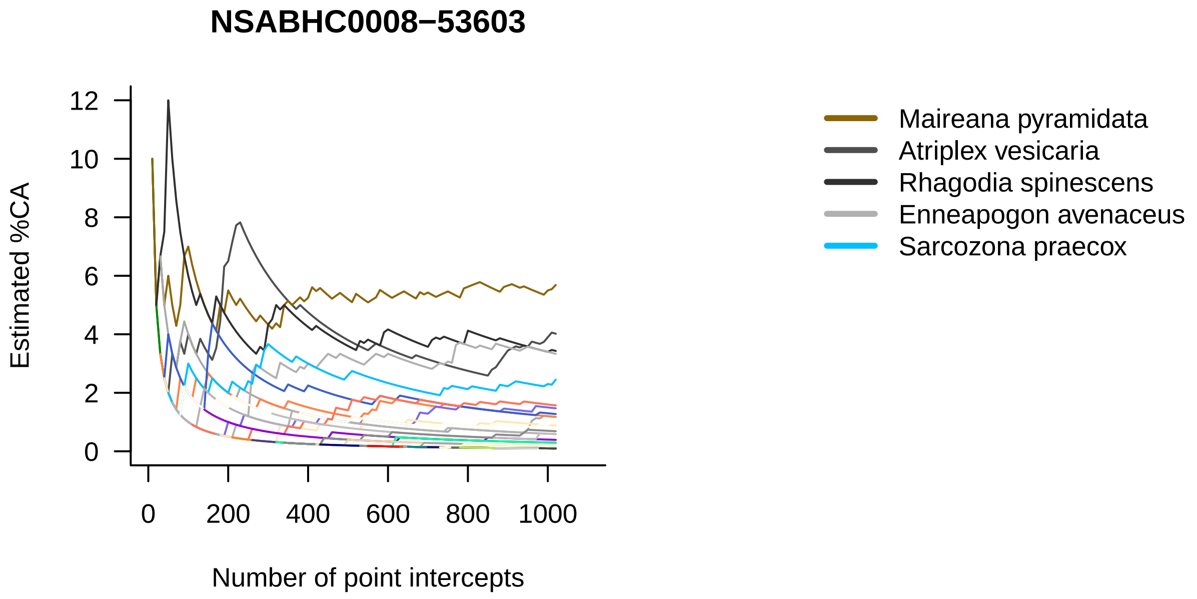
NSABHC0008−53603
Estimated %CA
0
2
4
6
8
10
12
0
200
400
600
800
1000
Number of point intercepts
Maireana pyramidata
Atriplex vesicaria
Rhagodia spinescens
Enneapogon avenaceus
Sarcozona praecox

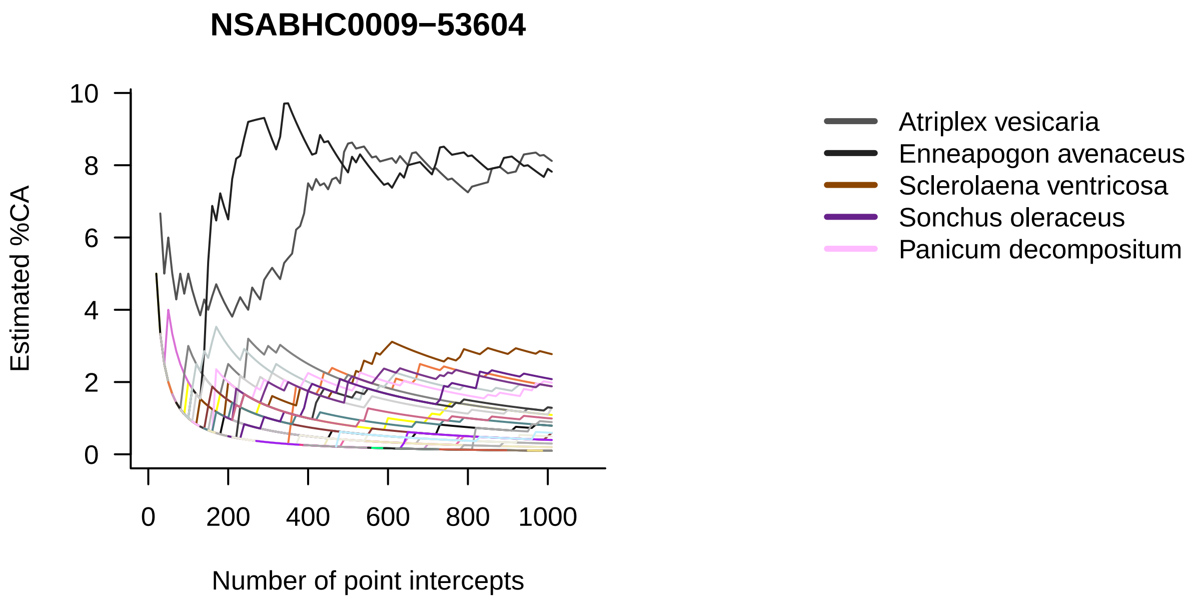
NSABHC0009−53604
Estimated %CA
Number of point intercepts
0
2
4
6
8
10
0
200
400
600
800
1000
Atriplex vesicaria
Enneapogon avenaceus
Sclerolaena ventricosa
Sonchus oleraceus
Panicum decompositum

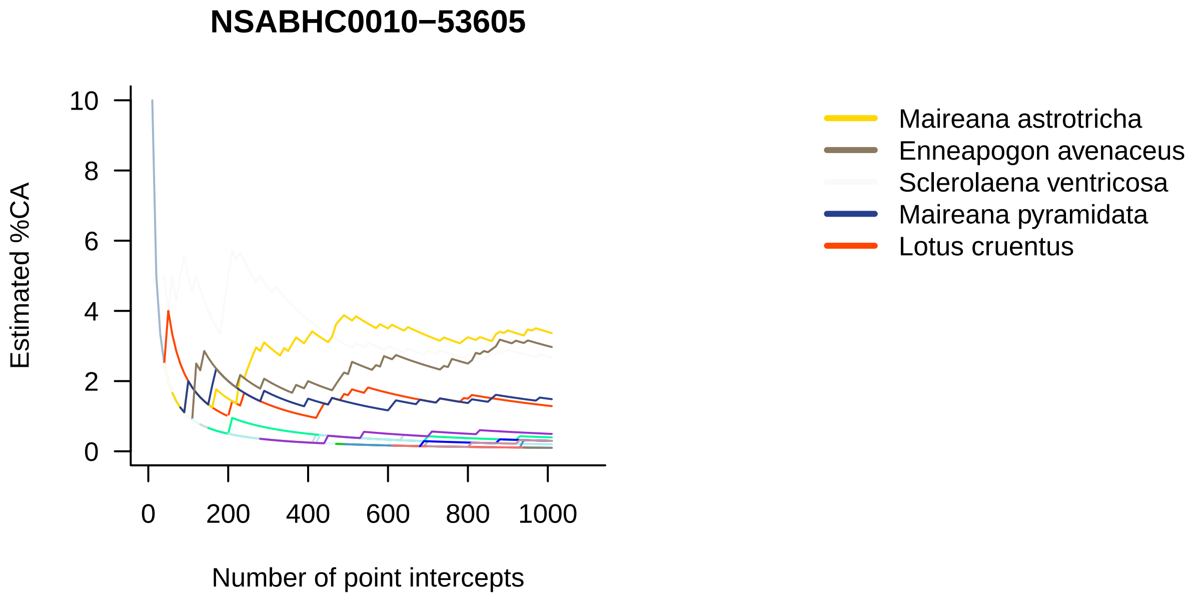
NSABHC0010−53605
Estimated %CA
Number of point intercepts
0
2
4
6
8
10
0
200
400
600
800
1000
Maireana astrotricha
Enneapogon avenaceus
Sclerolaena ventricosa
Maireana pyramidata
Lotus cruentus

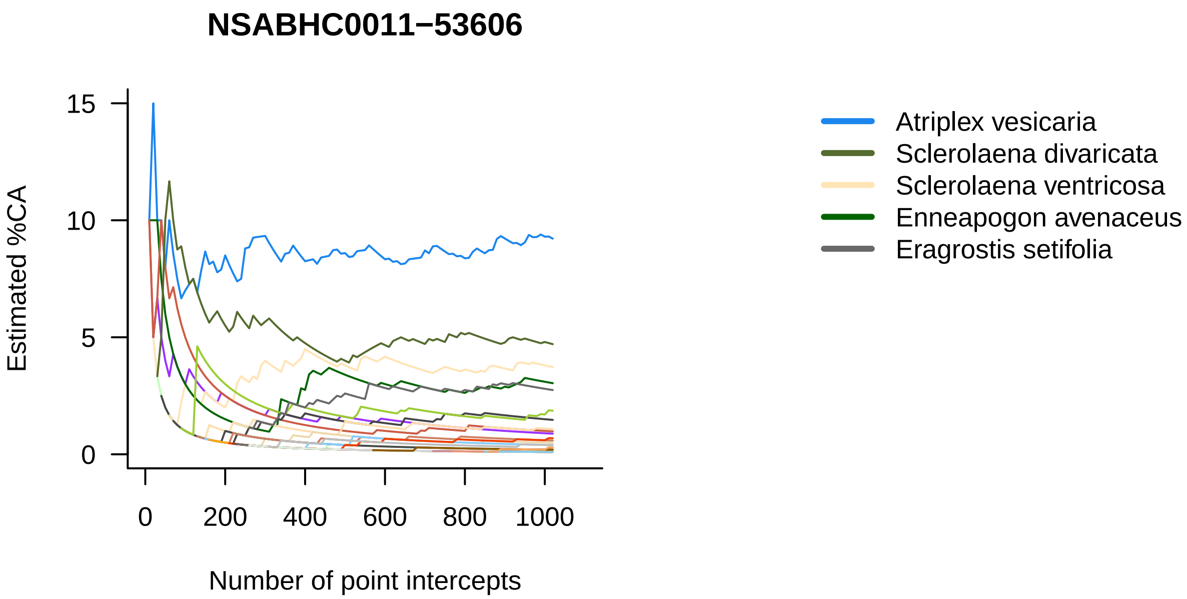
NSABHC0011−53606
Estimated %CA
15
10
5
0
0
200
400
600
800
1000
Number of point intercepts
Atriplex vesicaria
Sclerolaena divaricata
Sclerolaena ventricosa
Enneapogon avenaceus
Eragrostis setifolia

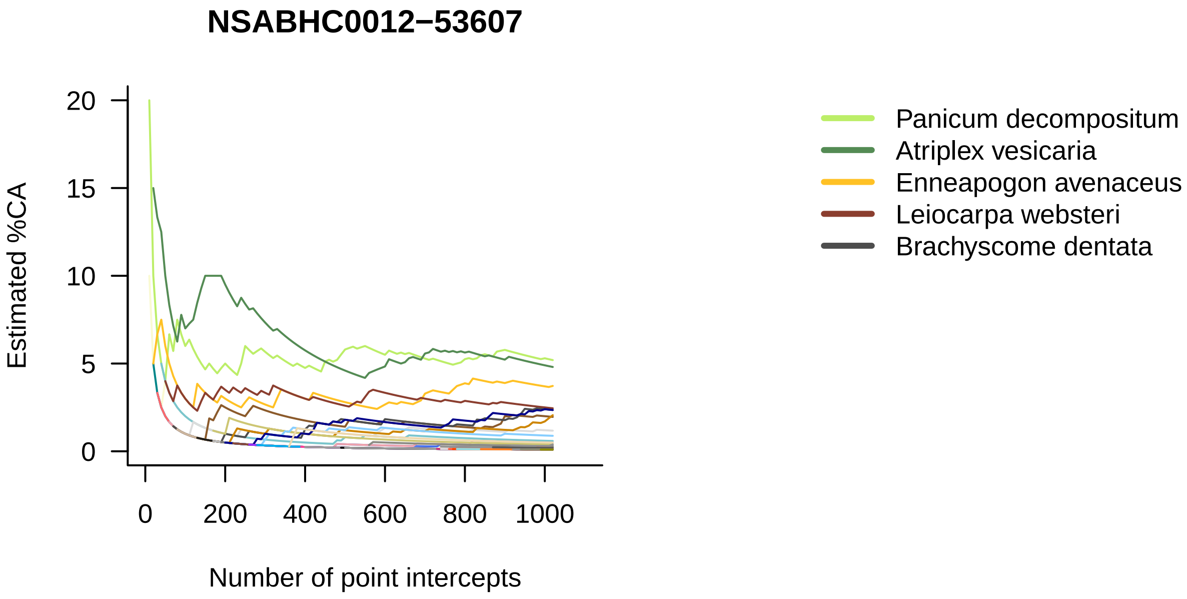
NSABHC0012−53607
Estimated %CA
20
15
10
5
0
0
200
400
600
800
1000
Number of point intercepts
Panicum decompositum
Atriplex vesicaria
Enneapogon avenaceus
Leiocarpa websteri
Brachyscome dentata

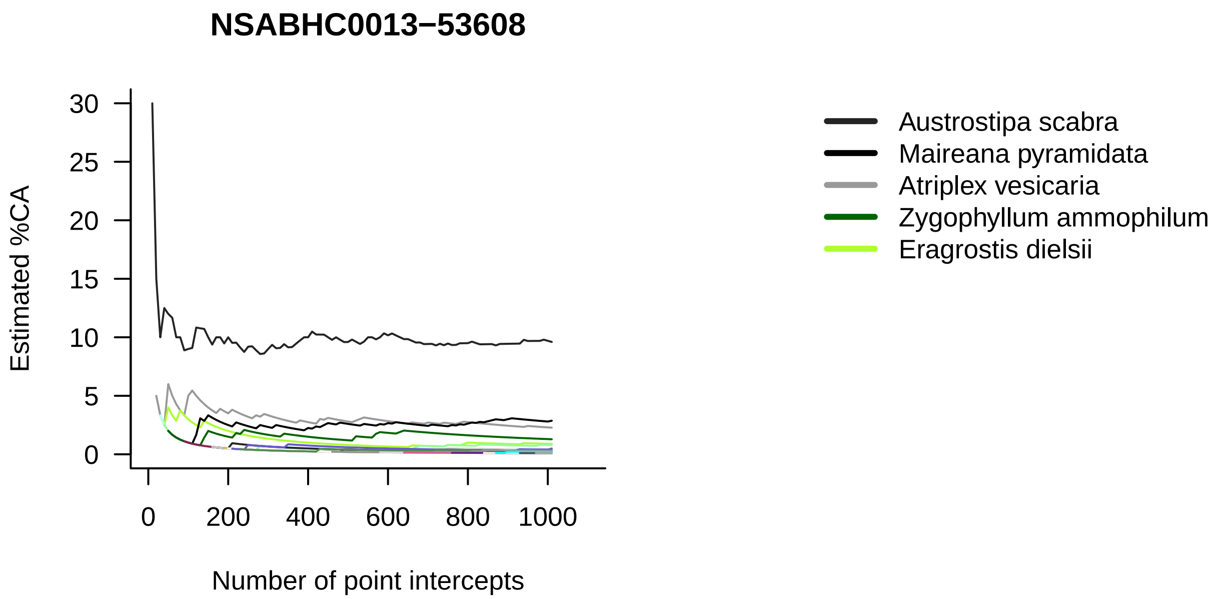
NSABHC0013−53608
Estimated %CA
30
25
20
15
10
5
0
0
200
400
600
800
1000
Number of point intercepts
Austrostipa scabra
Maireana pyramidata
Atriplex vesicaria
Zygophyllum ammophilum
Eragrostis dielsii

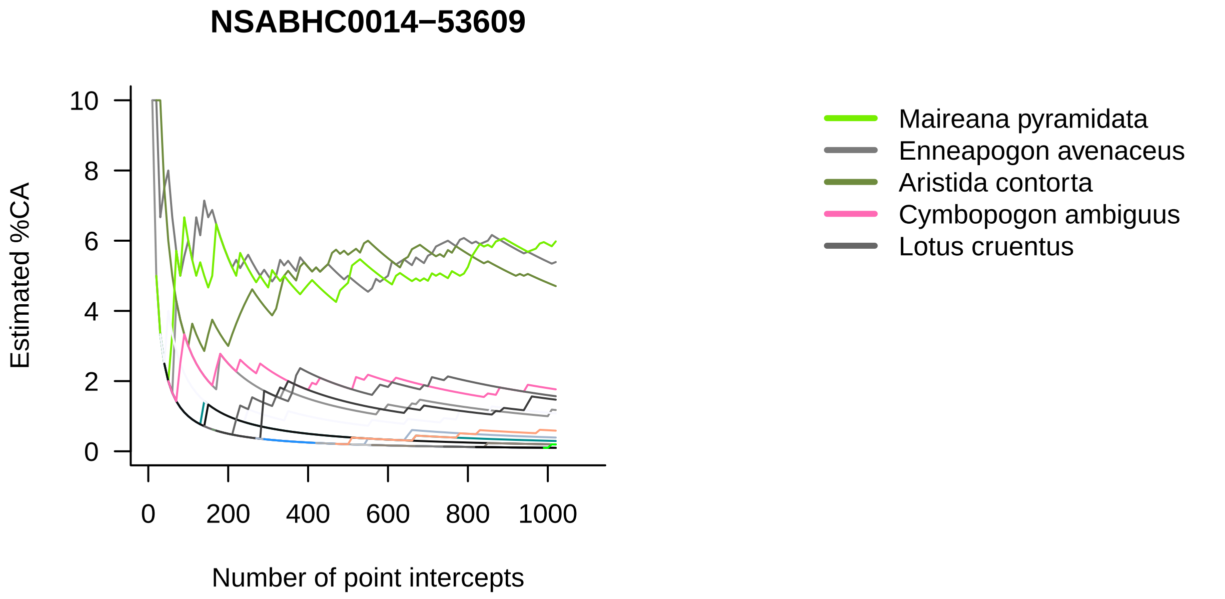
NSABHC0014−53609
Estimated %CA
10
8
6
4
2
0
0
200
400
600
800
1000
Number of point intercepts
Maireana pyramidata
Enneapogon avenaceus
Aristida contorta
Cymbopogon ambiguus
Lotus cruentus

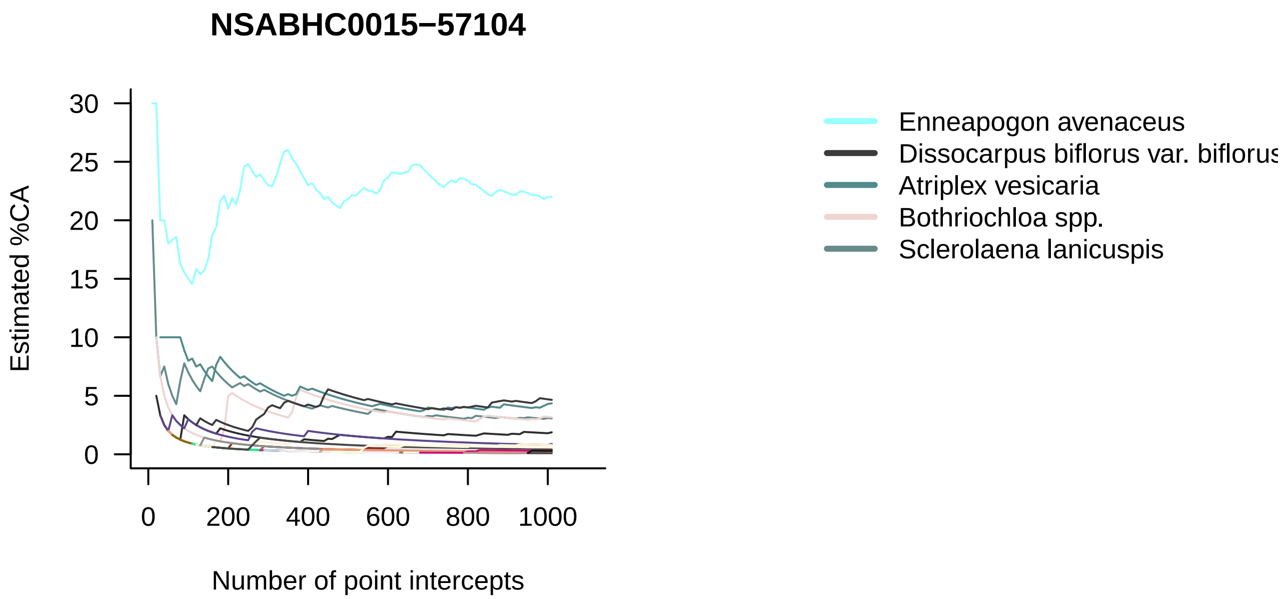
NSABHC0015−57104
Estimated %CA
30
25
20
15
10
5
0
0
200
400
600
800
1000
Number of point intercepts
Enneapogon avenaceus
Dissocarpus biflorus var. biflorus
Atriplex vesicaria
Bothriochloa spp.
Sclerolaena lanicuspis

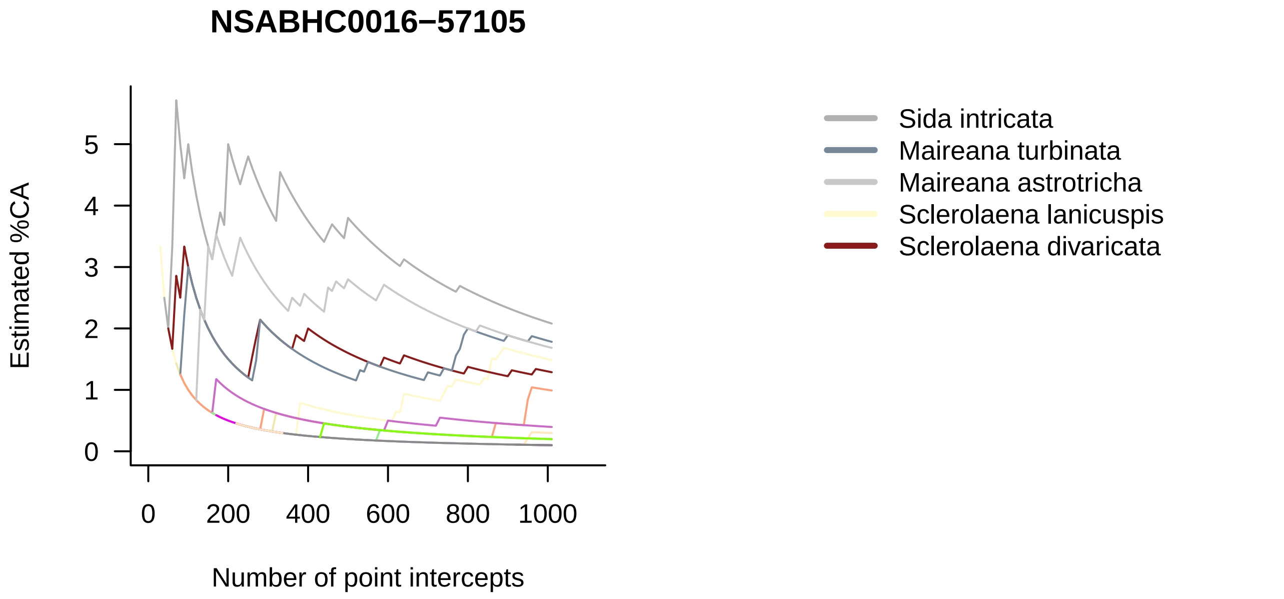
NSABHC0016−57105
Estimated %CA
5
4
3
2
1
0
0
200
400
600
800
1000
Number of point intercepts
Sida intricata
Maireana turbinata
Maireana astrotricha
Sclerolaena lanicuspis
Sclerolaena divaricata

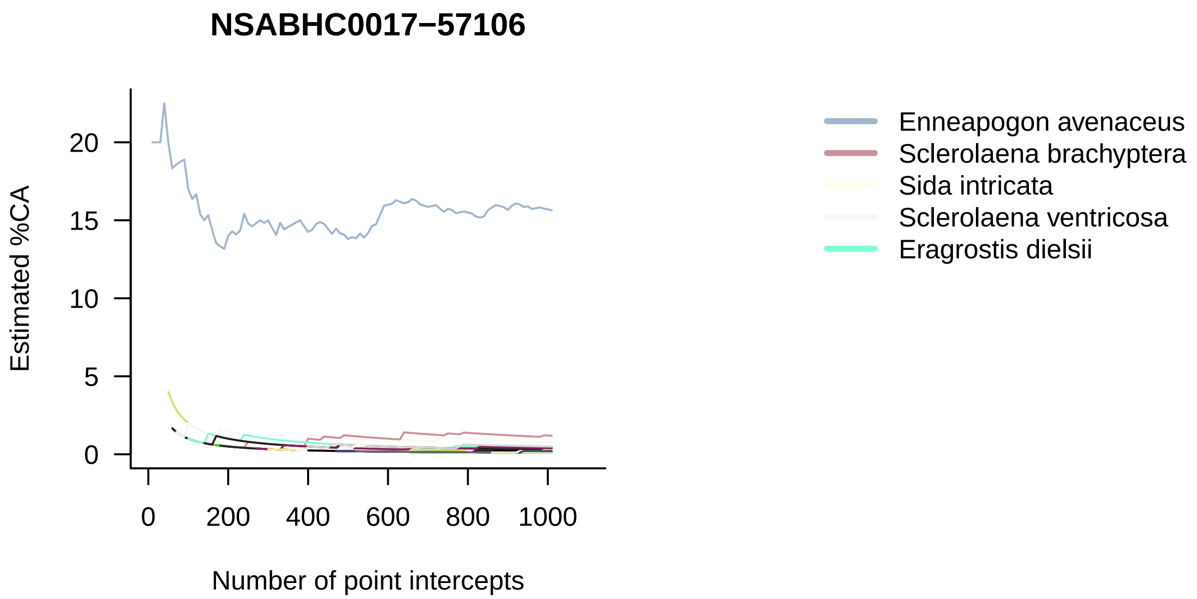
NSABHC0017−57106
Estimated %CA
Number of point intercepts
0
5
10
15
20
0
200
400
600
800
1000
Enneapogon avenaceus
Sclerolaena brachyptera
Sida intricata
Sclerolaena ventricosa
Eragrostis dielsii

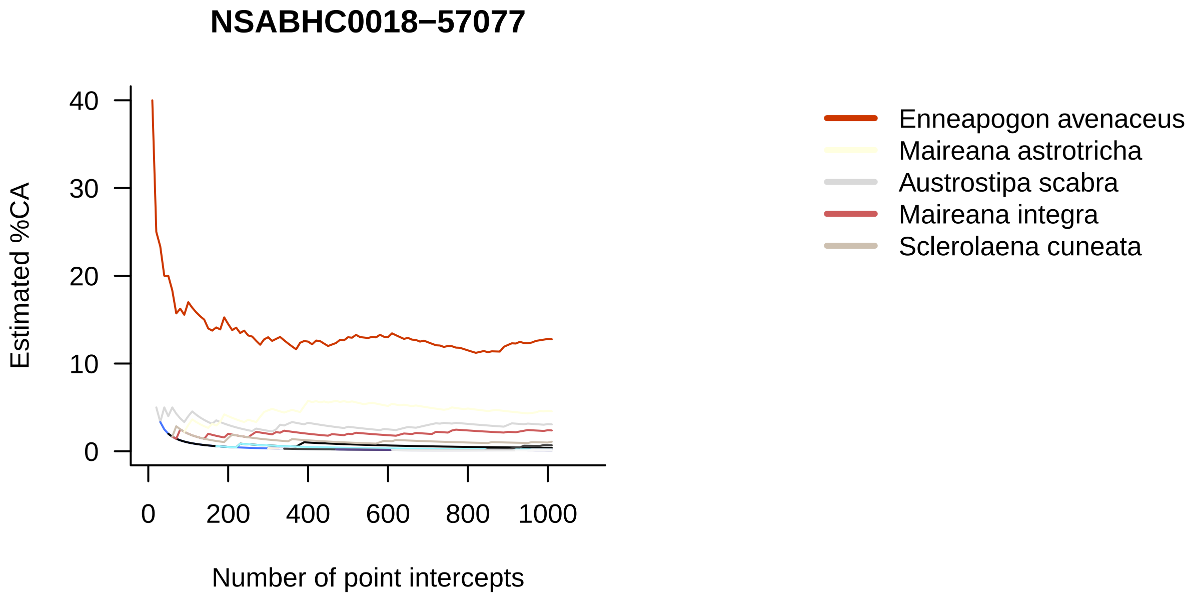
NSABHC0018−57077
Estimated %CA
Number of point intercepts
0
10
20
30
40
0
200
400
600
800
1000
Enneapogon avenaceus
Maireana astrotricha
Austrostipa scabra
Maireana integra
Sclerolaena cuneata

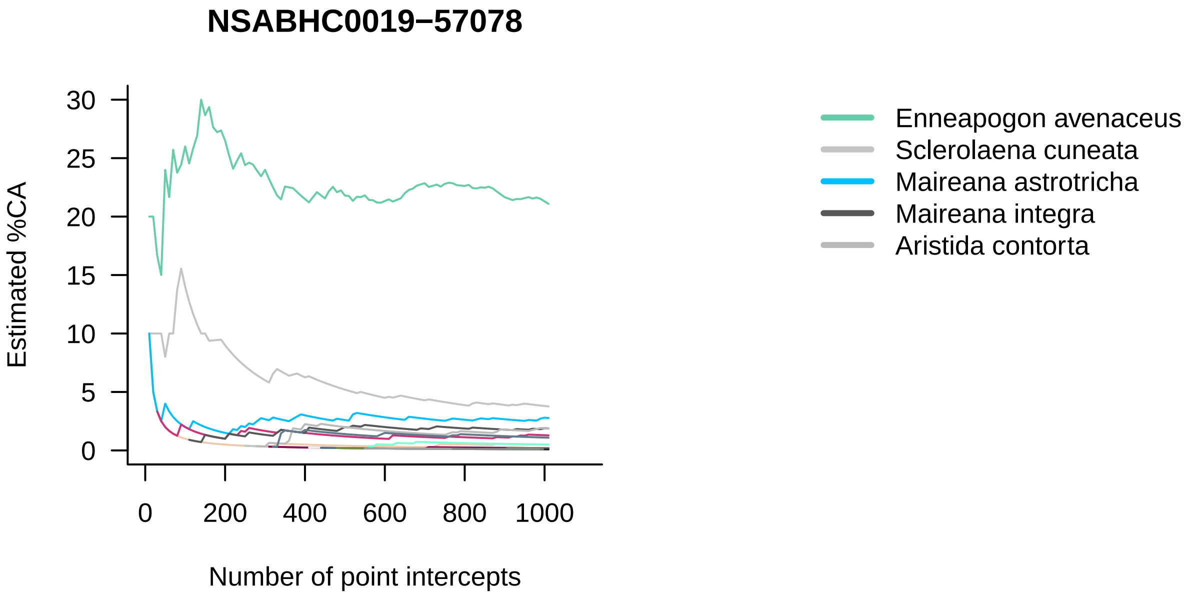
NSABHC0019−57078
Estimated %CA
0
5
10
15
20
25
30
0
200
400
600
800
1000
Number of point intercepts
Enneapogon avenaceus
Sclerolaena cuneata
Maireana astrotricha
Maireana integra
Aristida contorta

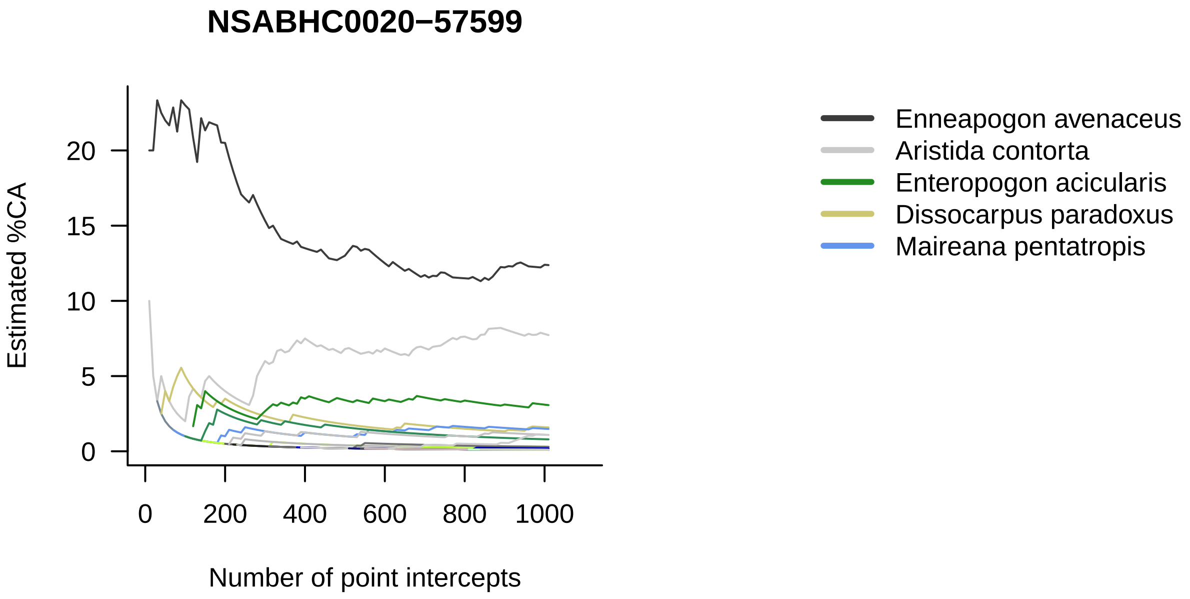
NSABHC0020−57599
Estimated %CA
0
5
10
15
20
0
200
400
600
800
1000
Number of point intercepts
Enneapogon avenaceus
Aristida contorta
Enteropogon acicularis
Dissocarpus paradoxus
Maireana pentatropis

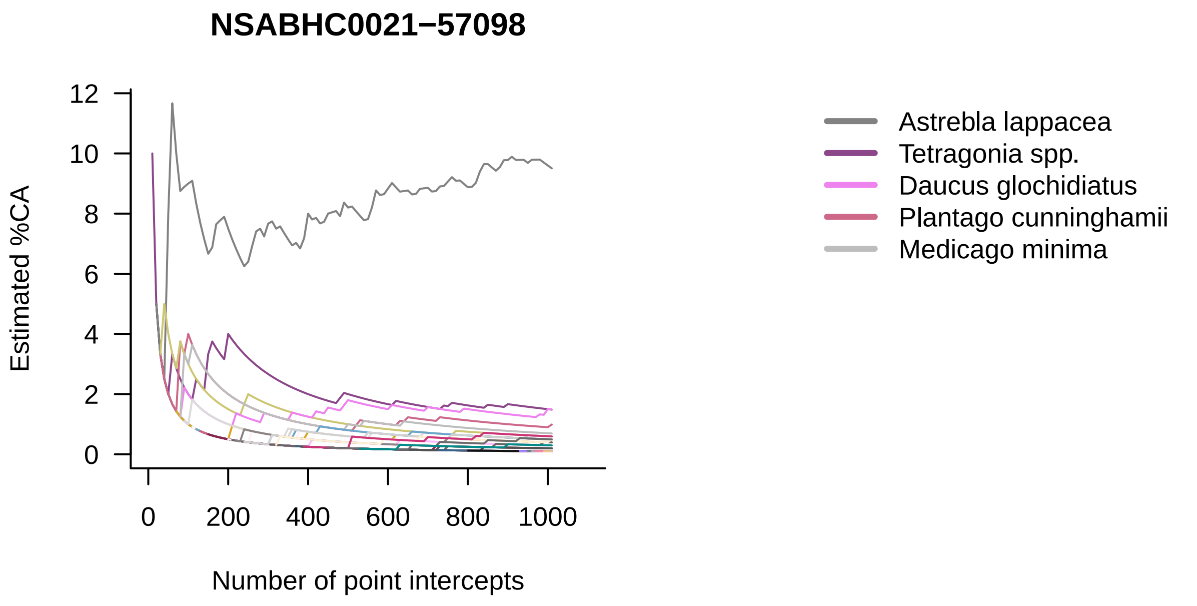
NSABHC0021−57098
Estimated %CA
12
10
8
6
4
2
0
0
200
400
600
800
1000
Number of point intercepts
Astrebla lappacea
Tetragonia spp.
Daucus glochidiatus
Plantago cunninghamii
Medicago minima

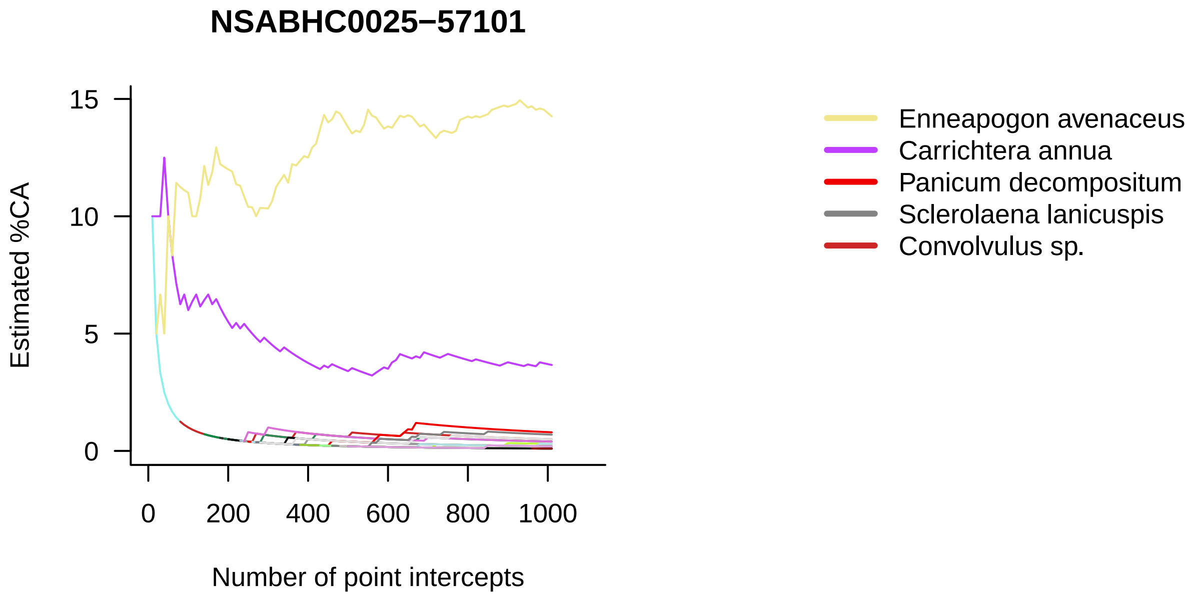
NSABHC0025−57101
Estimated %CA
15
10
5
0
0
200
400
600
800
1000
Number of point intercepts
Enneapogon avenaceus
Carrichtera annua
Panicum decompositum
Sclerolaena lanicuspis
Convolvulus sp.

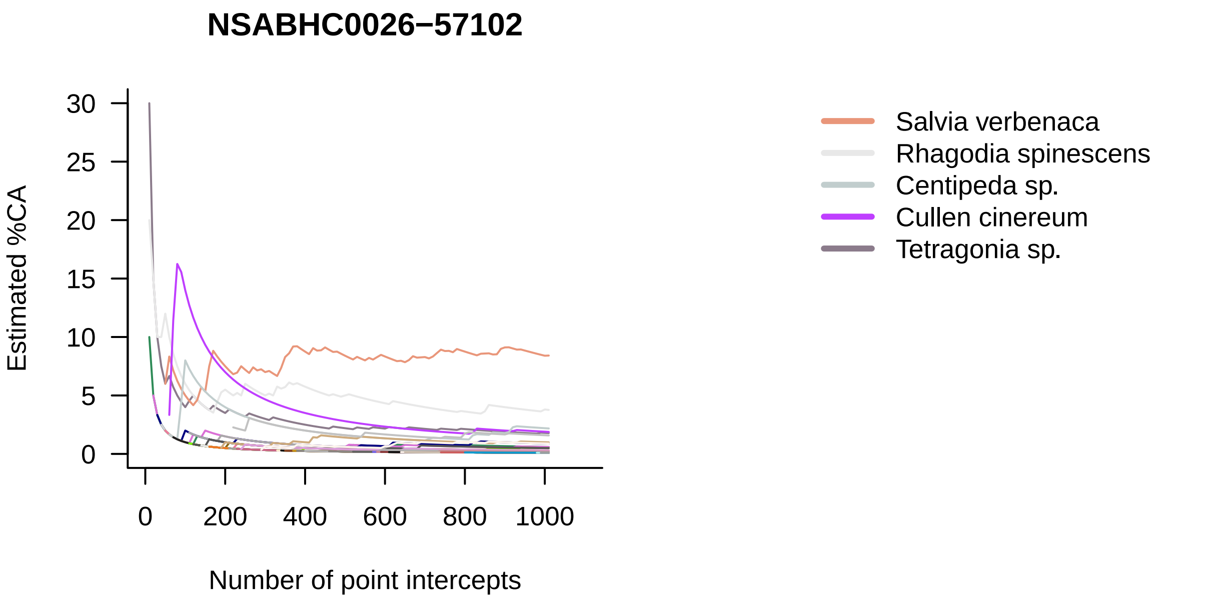
NSABHC0026−57102
Estimated %CA
30
25
20
15
10
5
0
0
200
400
600
800
1000
Number of point intercepts
Salvia verbenaca
Rhagodia spinescens
Centipeda sp.
Cullen cinereum
Tetragonia sp.

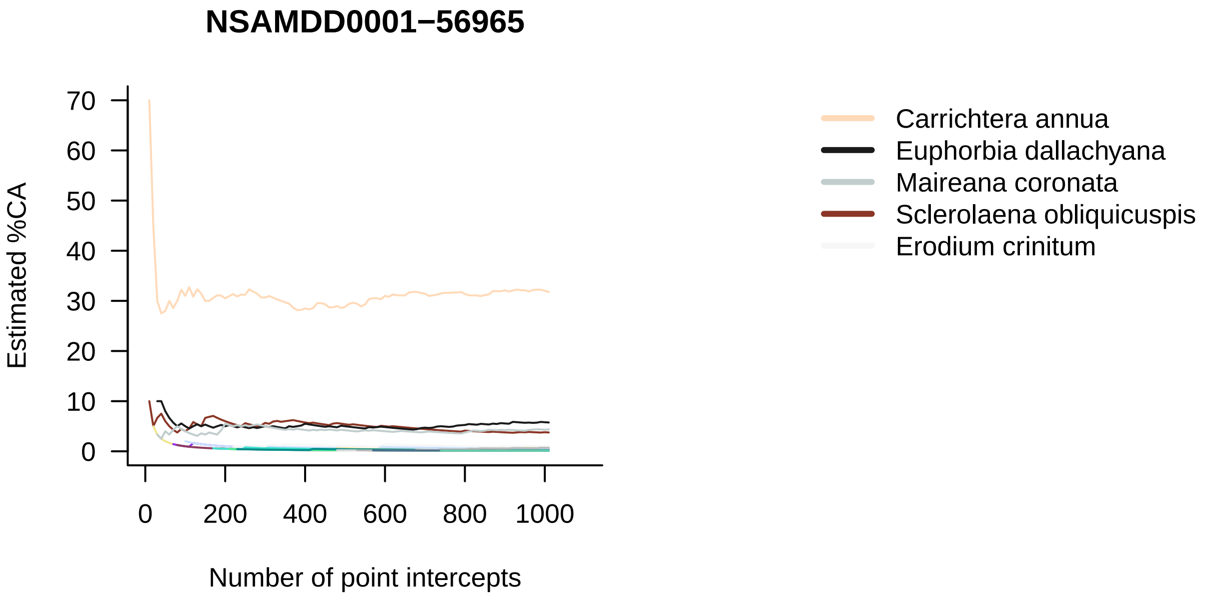
NSAMDD0001−56965
Estimated %CA
70
60
50
40
30
20
10
0
0
200
400
600
800
1000
Number of point intercepts
Carrichtera annua
Euphorbia dallachyana
Maireana coronata
Sclerolaena obliquicuspis
Erodium crinitum

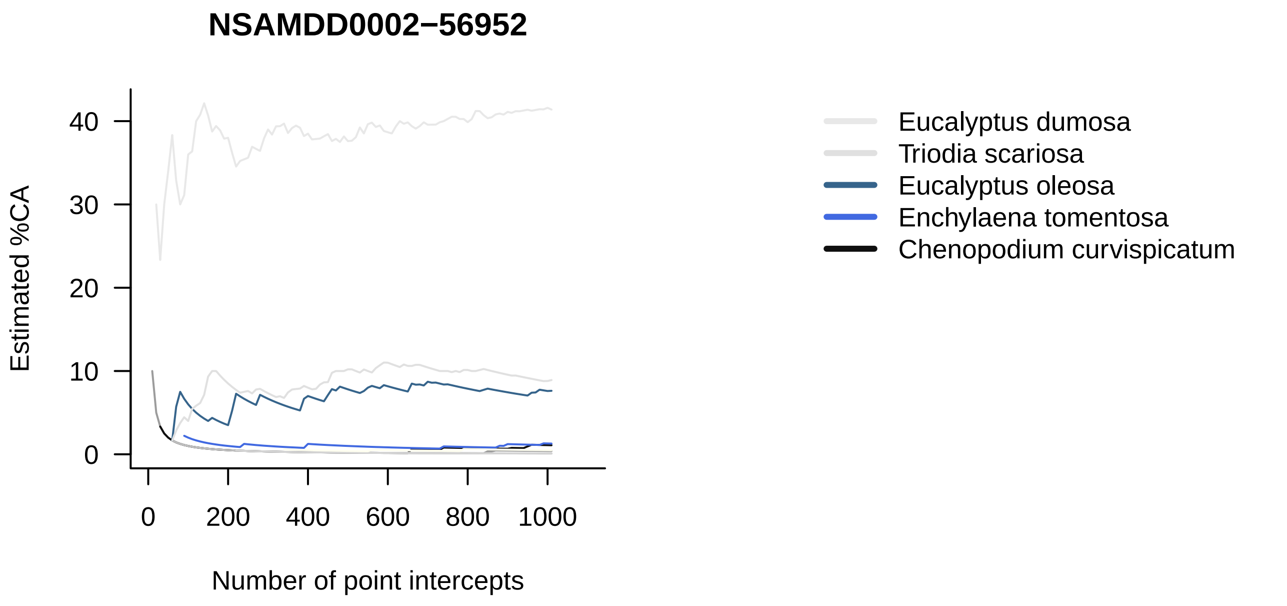
NSAMDD0002−56952
Estimated %CA
0
10
20
30
40
0
200
400
600
800
1000
Number of point intercepts
Eucalyptus dumosa
Triodia scariosa
Eucalyptus oleosa
Enchylaena tomentosa
Chenopodium curvispicatum

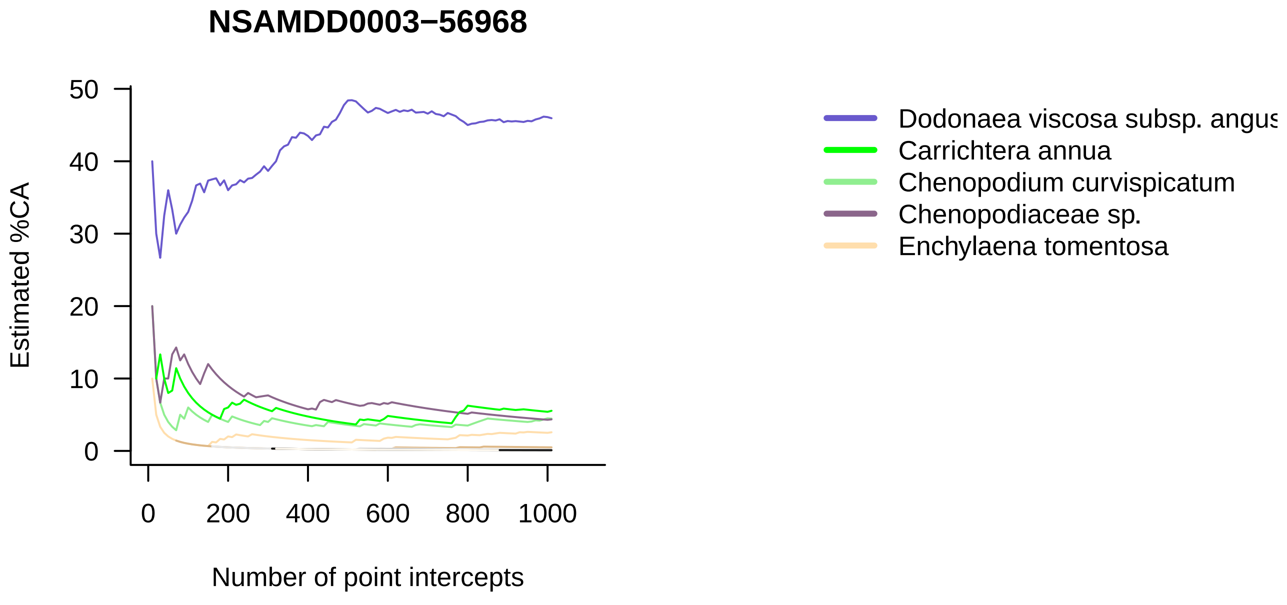
NSAMDD0003−56968
Estimated %CA
0
10
20
30
40
50
0
200
400
600
800
1000
Number of point intercepts
Dodonaea viscosa subsp. angus
Carrichtera annua
Chenopodium curvispicatum
Chenopodiaceae sp.
Enchylaena tomentosa

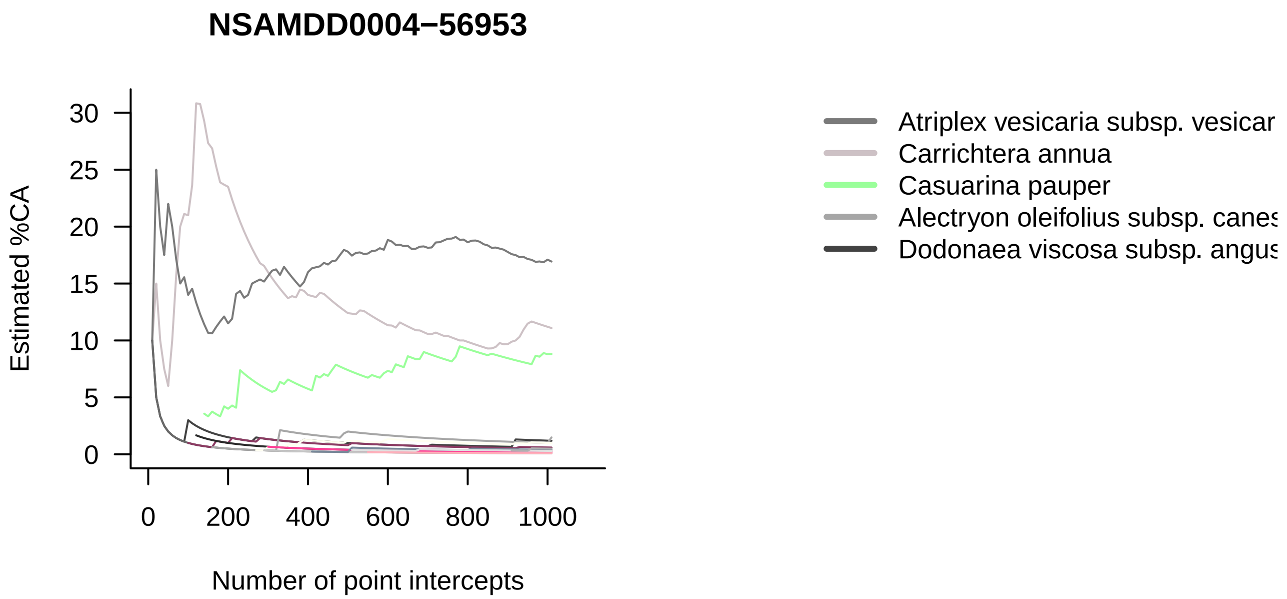
NSAMDD0004−56953
Estimated %CA
Number of point intercepts
0
5
10
15
20
25
30
0
200
400
600
800
1000
Atriplex vesicaria subsp. vesicar
Carrichtera annua
Casuarina pauper
Alectryon oleifolius subsp. canes
Dodonaea viscosa subsp. angus

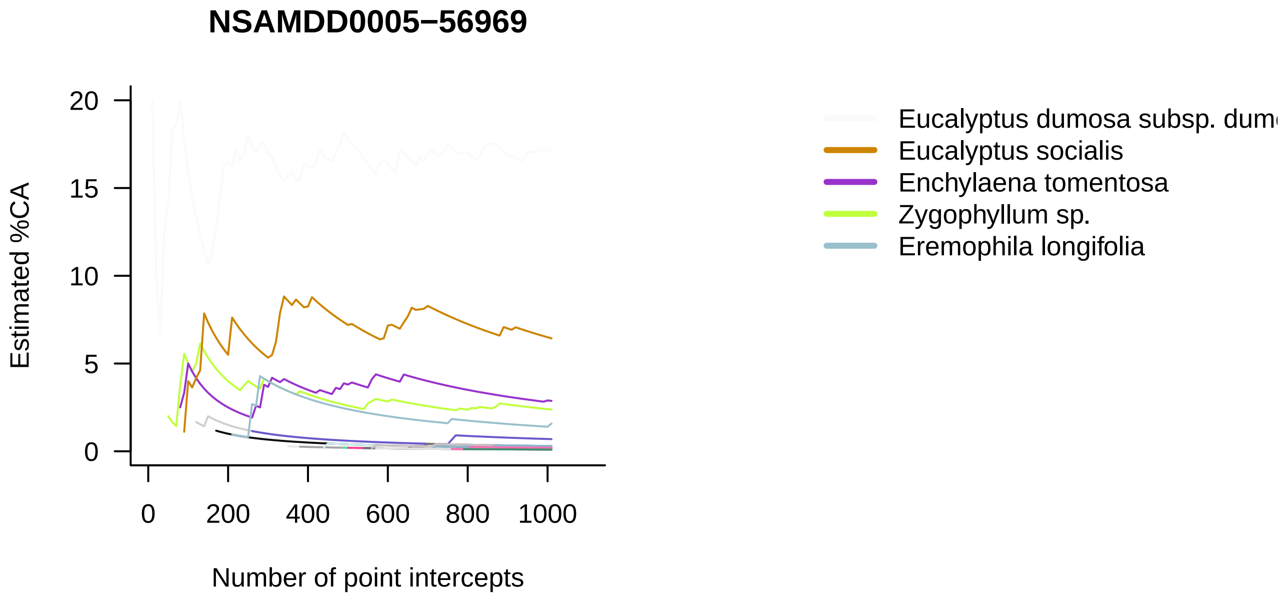
NSAMDD0005−56969
Estimated %CA
Number of point intercepts
0
5
10
15
20
0
200
400
600
800
1000
Eucalyptus dumosa subsp. dum
Eucalyptus socialis
Enchylaena tomentosa
Zygophyllum sp.
Eremophila longifolia

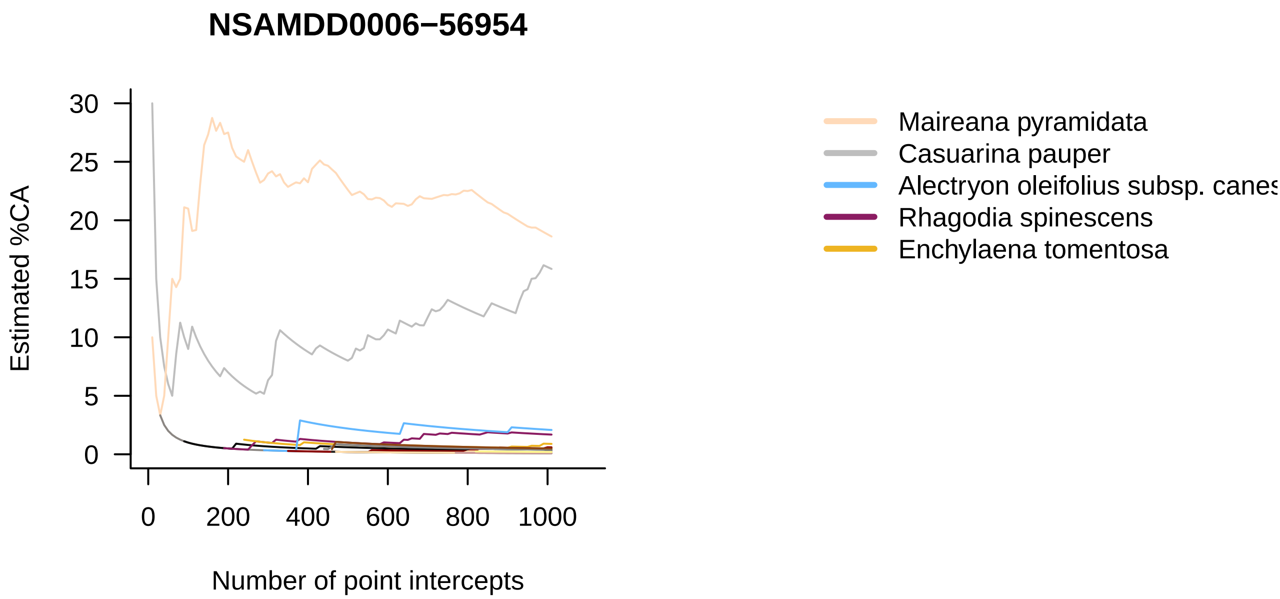

NSAMDD0006−56954
Estimated %CA
Number of point intercepts
0
5
10
15
20
25
30
0
200
400
600
800
1000
Maireana pyramidata
Casuarina pauper
Alectryon oleifolius subsp. canes
Rhagodia spinescens
Enchylaena tomentosa


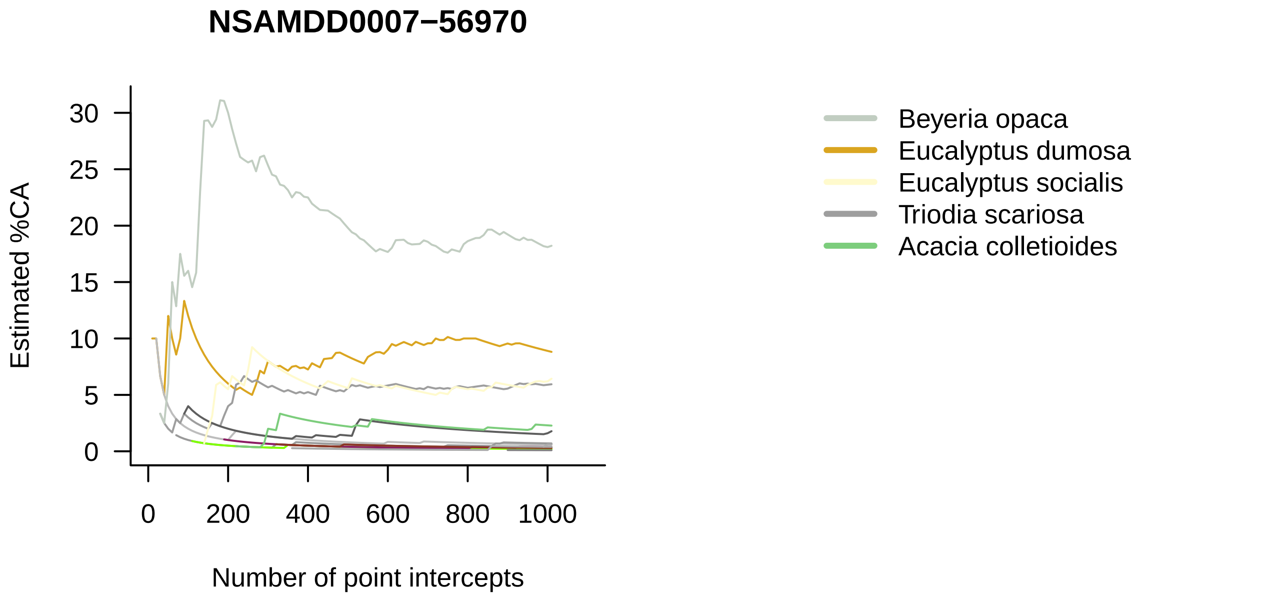

NSAMDD0007−56970
Estimated %CA
Number of point intercepts
0
5
10
15
20
25
30
0
200
400
600
800
1000
Beyeria opaca
Eucalyptus dumosa
Eucalyptus socialis
Triodia scariosa
Acacia colletioides

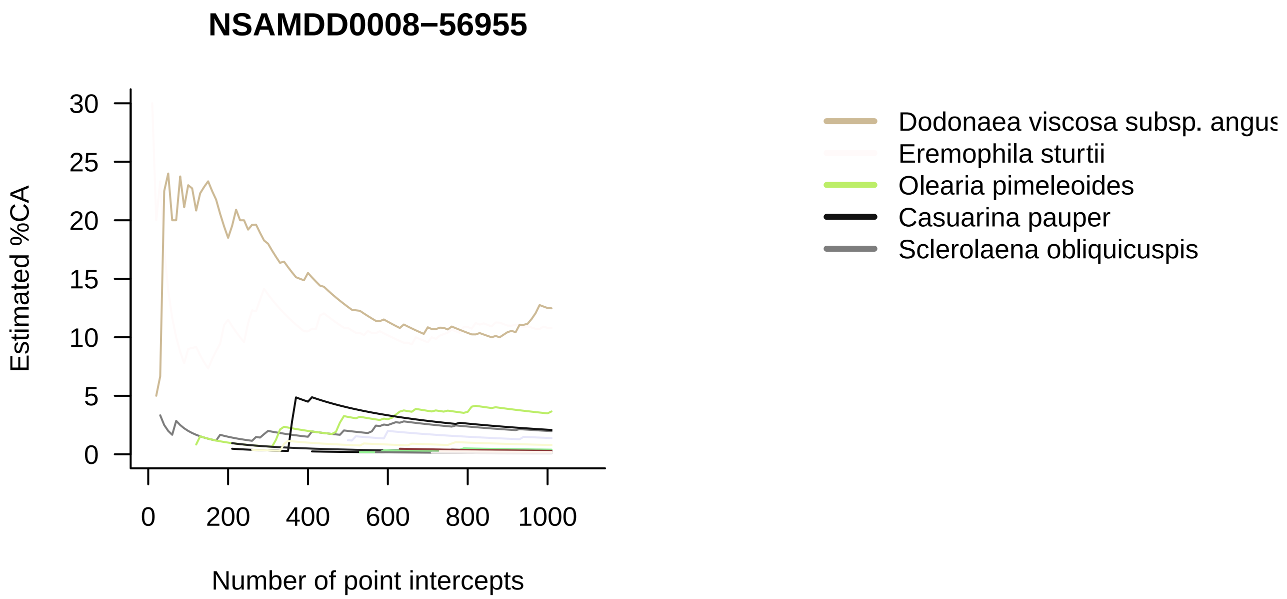
NSAMDD0008−56955
Estimated %CA
30
25
20
15
10
5
0
0
200
400
600
800
1000
Number of point intercepts
Dodonaea viscosa subsp. angus
Eremophila sturtii
Olearia pimeleoides
Casuarina pauper
Sclerolaena obliquicuspis

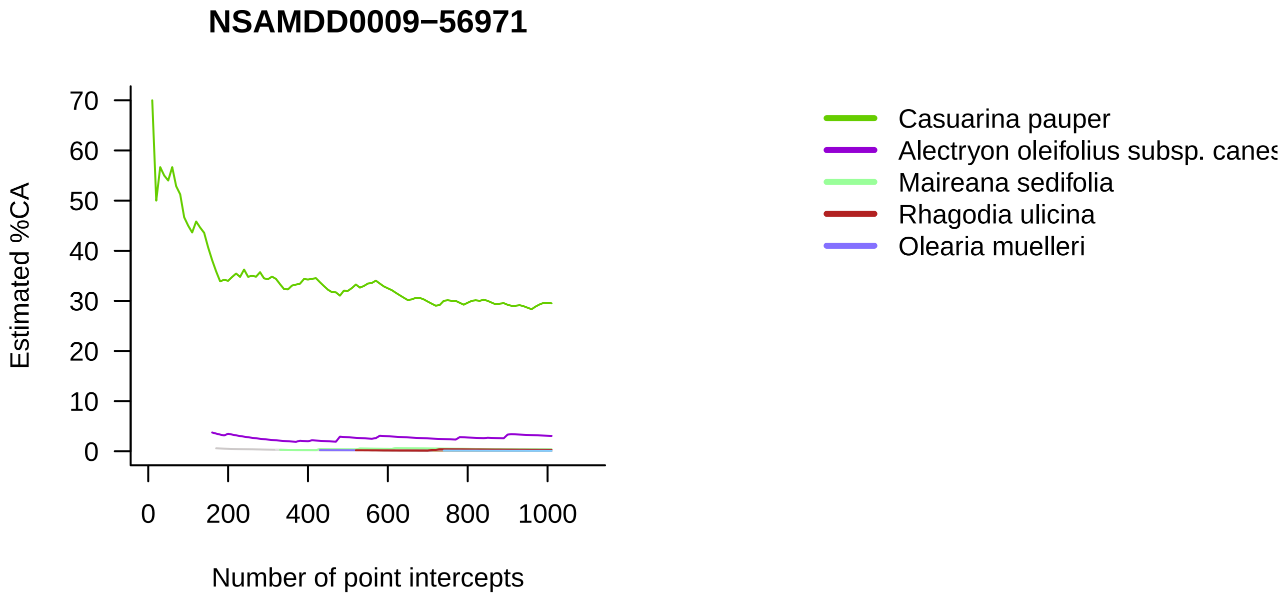
NSAMDD0009−56971
Estimated %CA
70
60
50
40
30
20
10
0
0
200
400
600
800
1000
Number of point intercepts
Casuarina pauper
Alectryon oleifolius subsp. canes
Maireana sedifolia
Rhagodia ulicina
Olearia muelleri

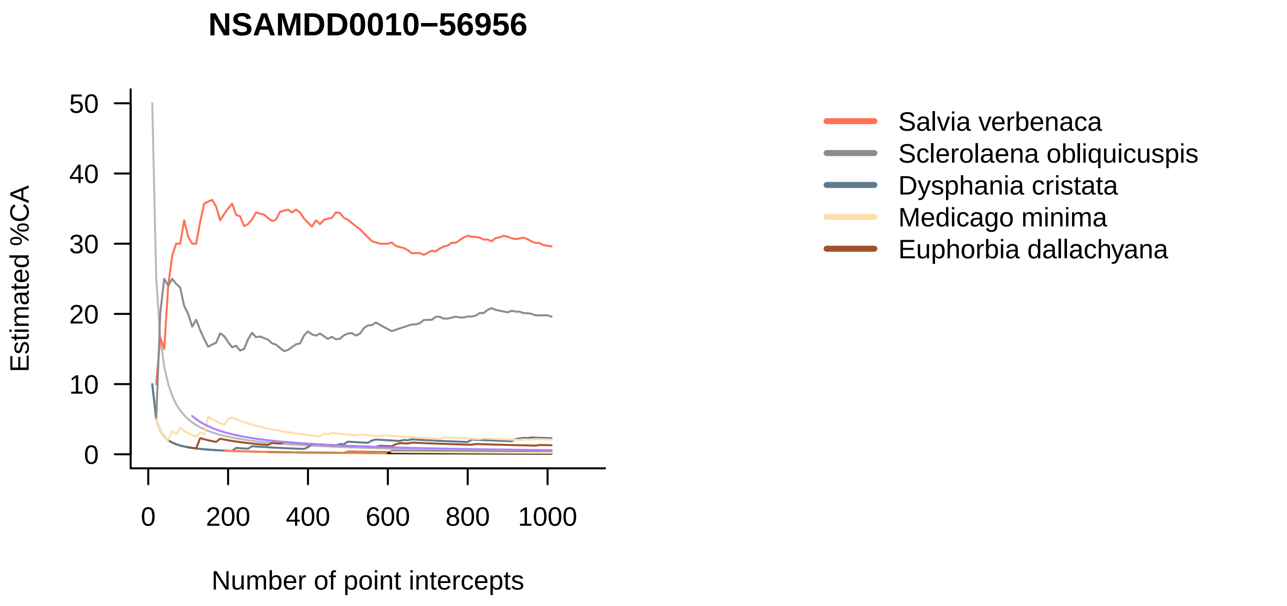
NSAMDD0010−56956
Estimated %CA
0
10
20
30
40
50
0
200
400
600
800
1000
Number of point intercepts
Salvia verbenaca
Sclerolaena obliquicuspis
Dysphania cristata
Medicago minima
Euphorbia dallachyana

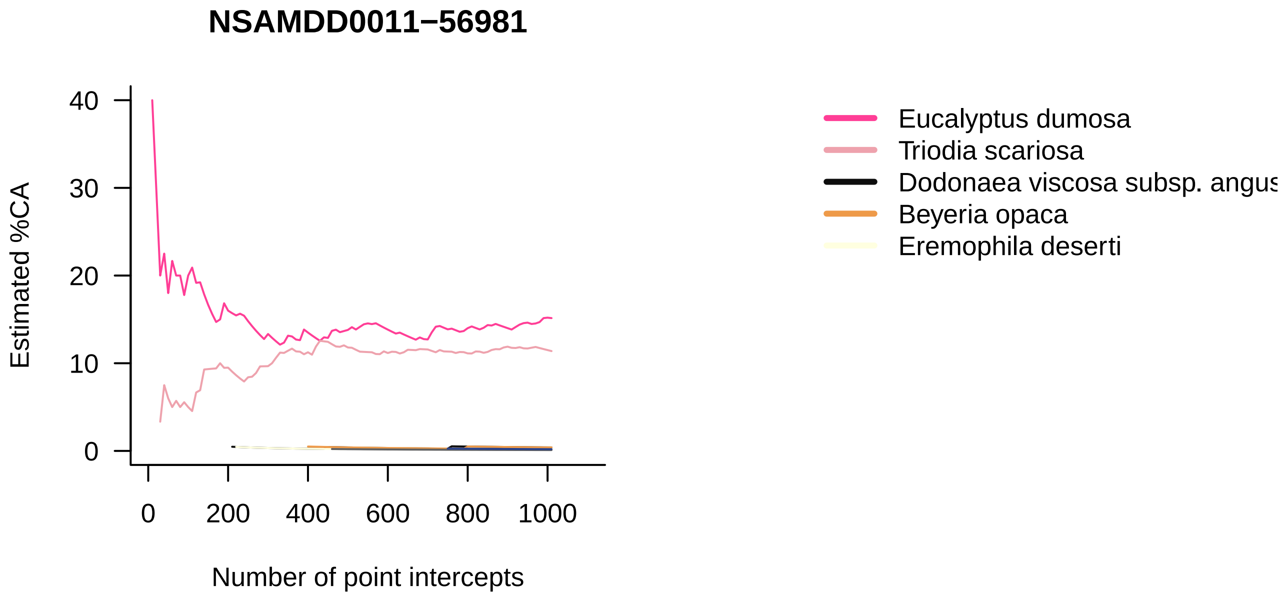
NSAMDD0011−56981
Estimated %CA
0
10
20
30
40
0
200
400
600
800
1000
Number of point intercepts
Eucalyptus dumosa
Triodia scariosa
Dodonaea viscosa subsp. angus
Beyeria opaca
Eremophila deserti

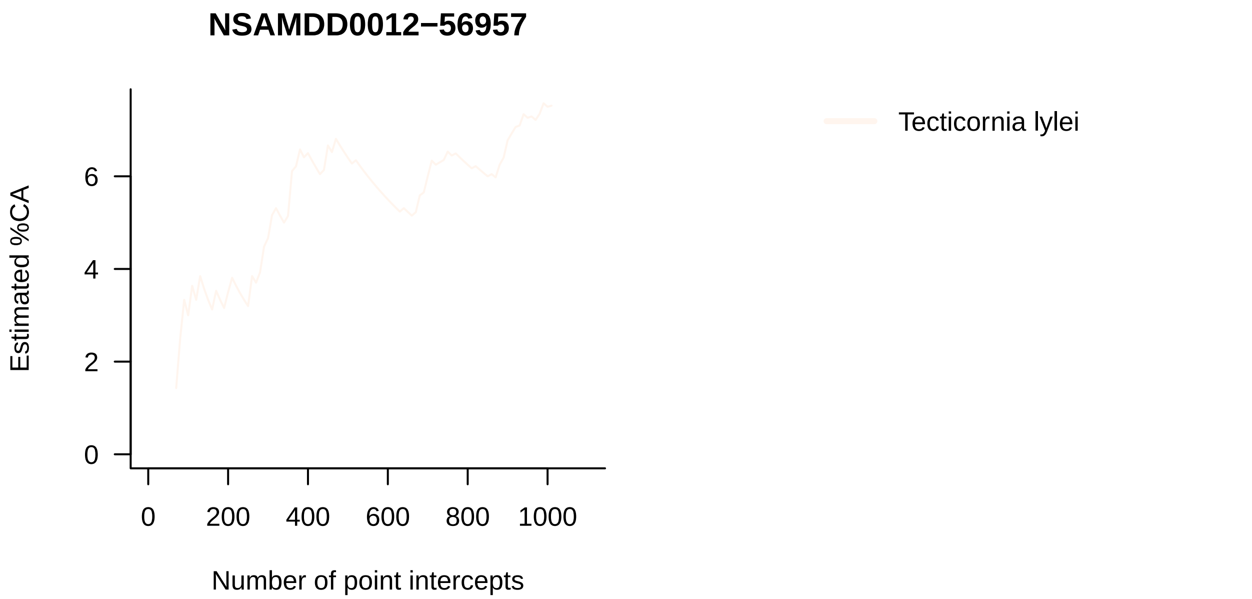
NSAMDD0012−56957
Estimated %CA
0
2
4
6
0
200
400
600
800
1000
Number of point intercepts
Tecticornia lylei

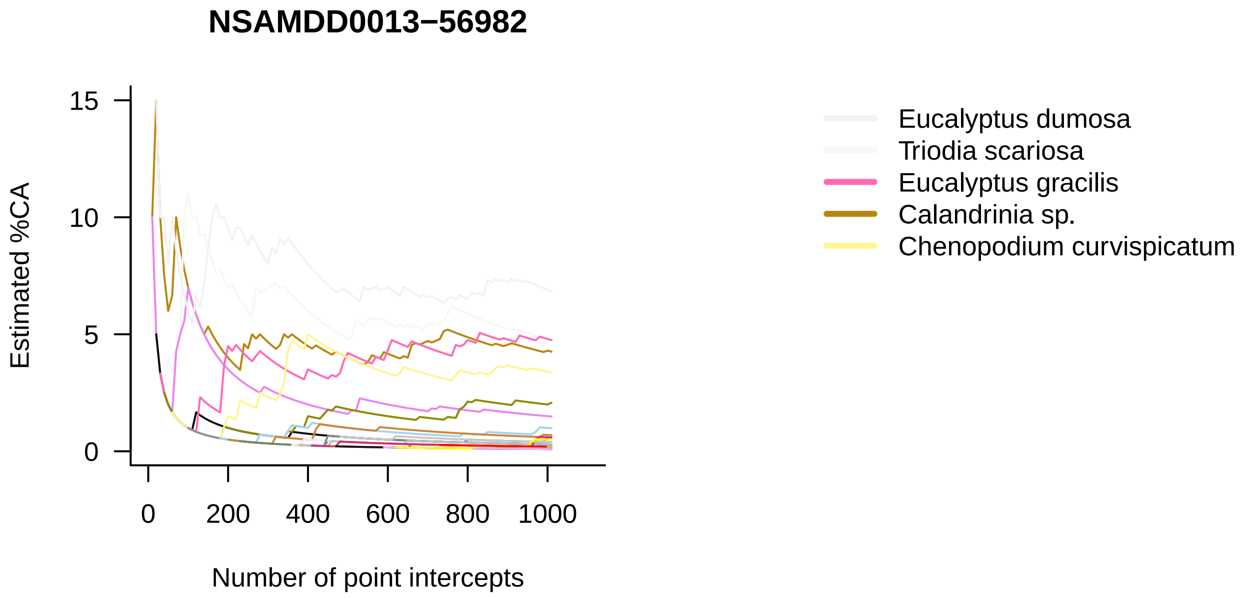
NSAMDD0013−56982
Estimated %CA
0
5
10
15
0
200
400
600
800
1000
Number of point intercepts
Eucalyptus dumosa
Triodia scariosa
Eucalyptus gracilis
Calandrinia sp.
Chenopodium curvispicatum

## NSAMDD0014−56963

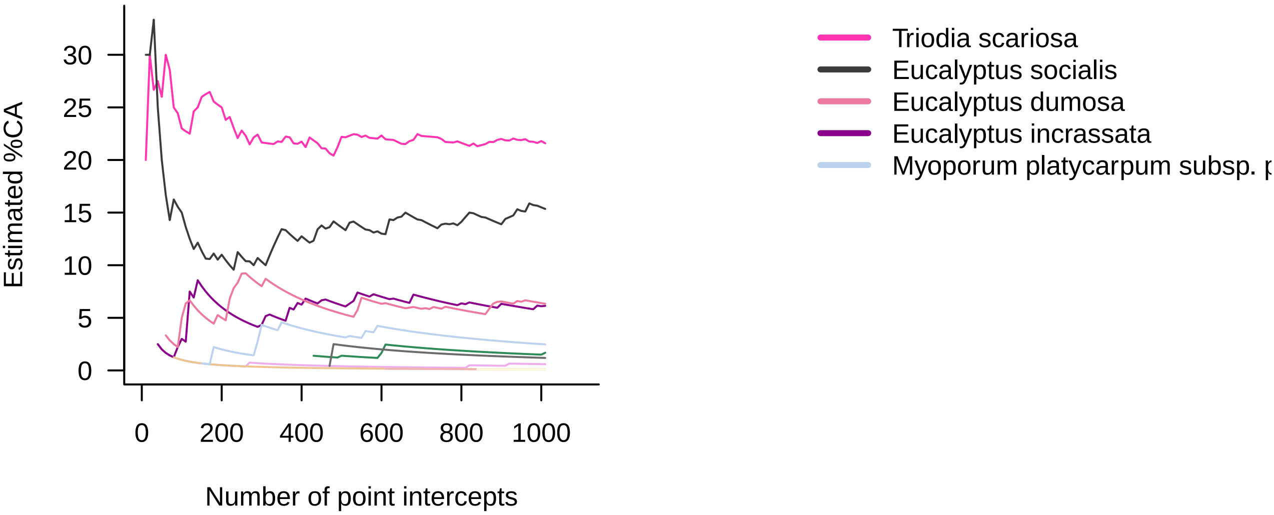


## NSAMDD0015−57636

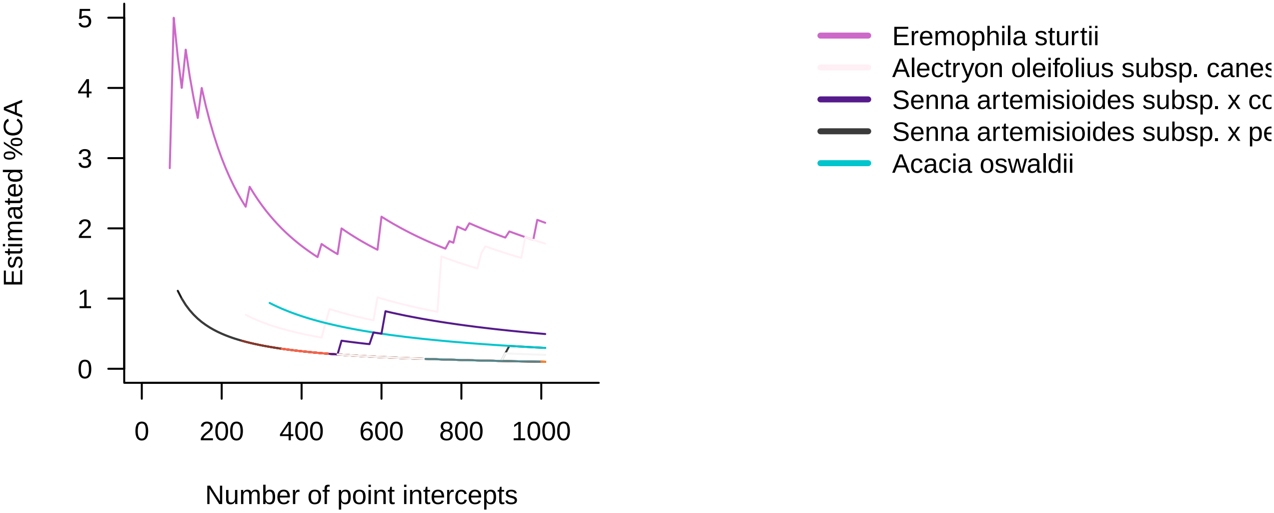

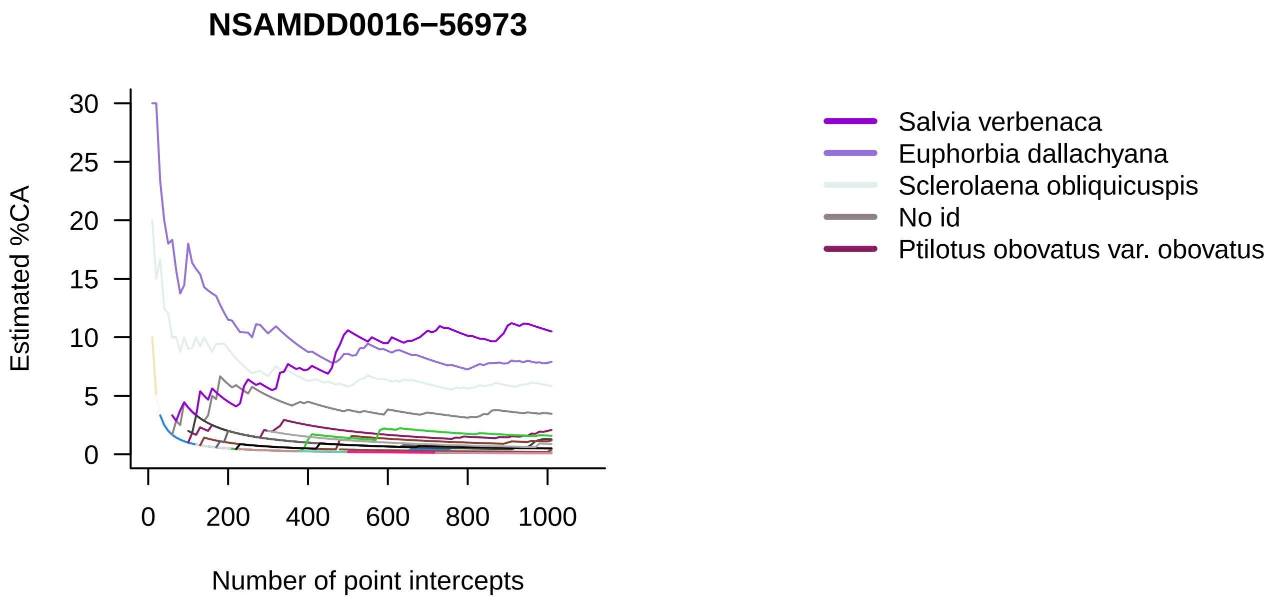
NSAMDD0016−56973
Estimated %CA
Number of point intercepts
0
5
10
15
20
25
30
0
200
400
600
800
1000
Salvia verbenaca
Euphorbia dallachyana
Sclerolaena obliquicuspis
No id
Ptilotus obovatus var. obovatus

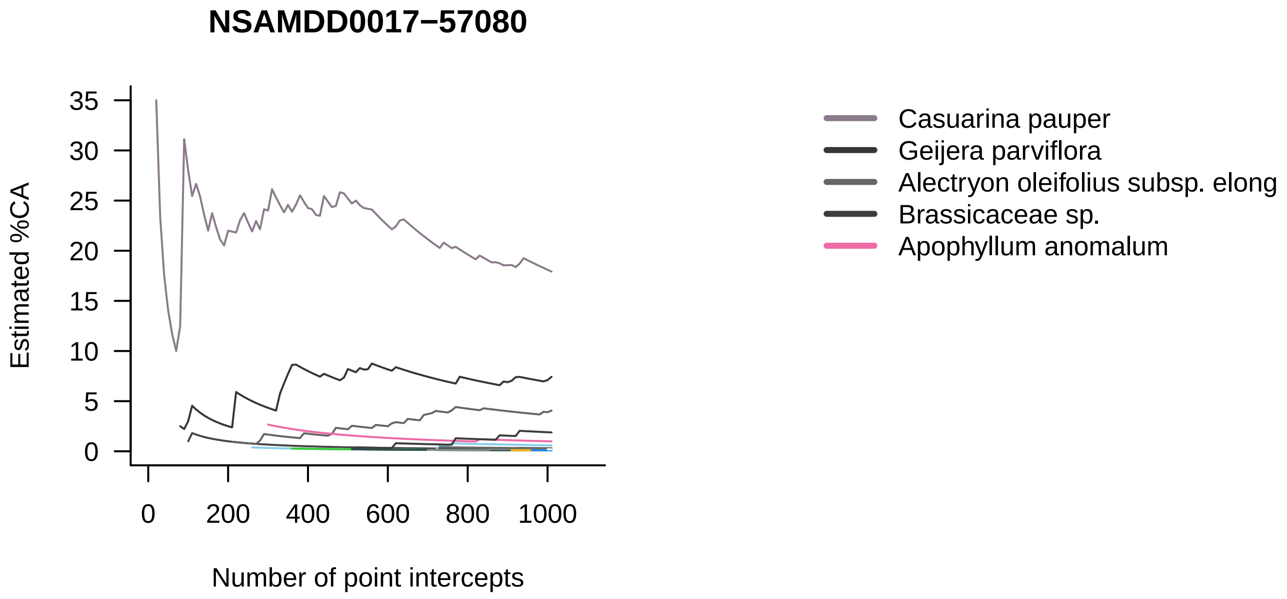
NSAMDD0017−57080
Estimated %CA
Number of point intercepts
0
5
10
15
20
25
30
35
0
200
400
600
800
1000
Casuarina pauper
Geijera parviflora
Alectryon oleifolius subsp. elong
Brassicaceae sp.
Apophyllum anomalum

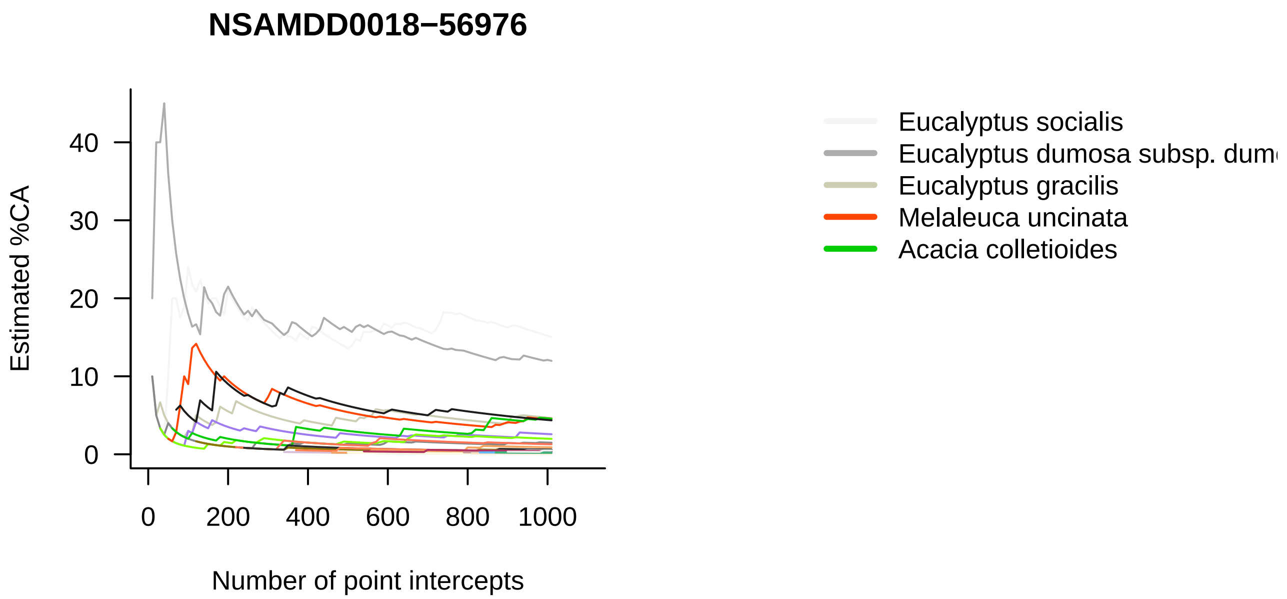
NSAMDD0018−56976
Estimated %CA
0
10
20
30
40
0
200
400
600
800
1000
Number of point intercepts
Eucalyptus socialis
Eucalyptus dumosa subsp. dum
Eucalyptus gracilis
Melaleuca uncinata
Acacia colletioides

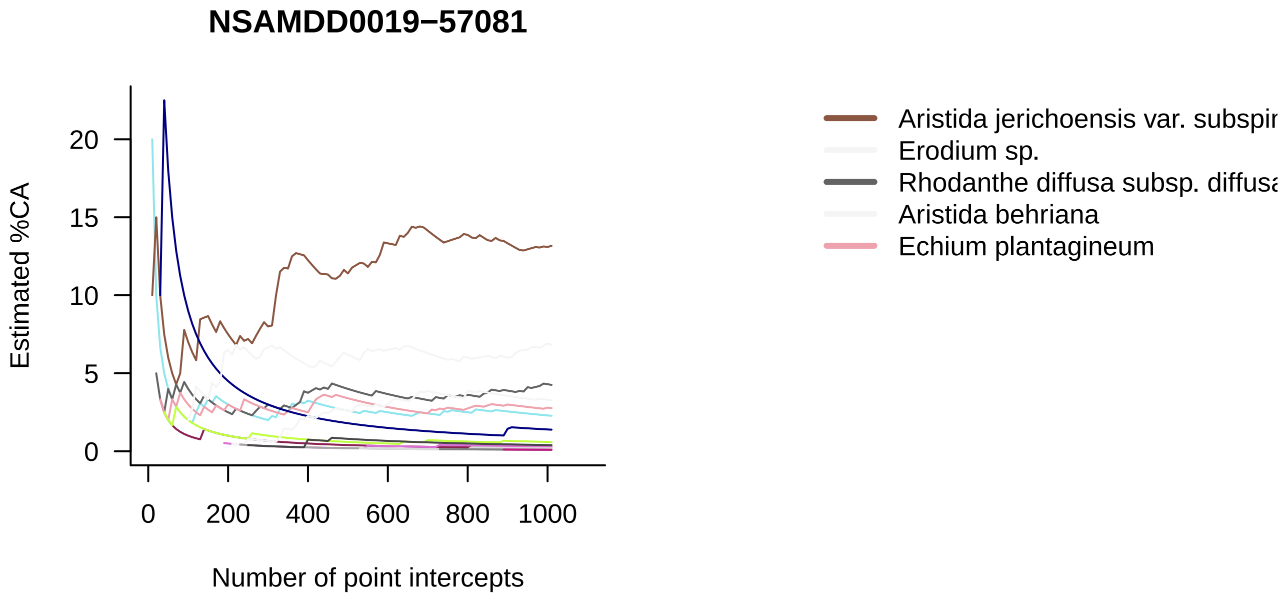
NSAMDD0019−57081
Estimated %CA
0
5
10
15
20
0
200
400
600
800
1000
Number of point intercepts
Aristida jerichoensis var. subspir
Erodium sp.
Rhodanthe diffusa subsp. diffusa
Aristida behriana
Echium plantagineum

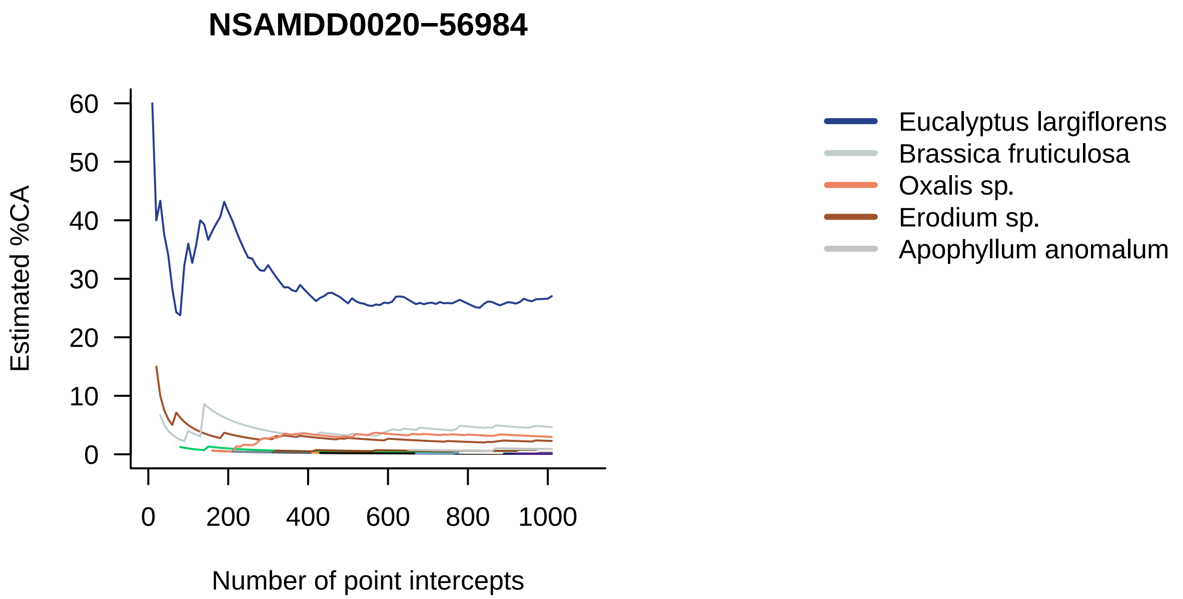
NSAMDD0020−56984
Estimated %CA
0
10
20
30
40
50
60
0
200
400
600
800
1000
Number of point intercepts
Eucalyptus largiflorens
Brassica fruticulosa
Oxalis sp.
Erodium sp.
Apophyllum anomalum

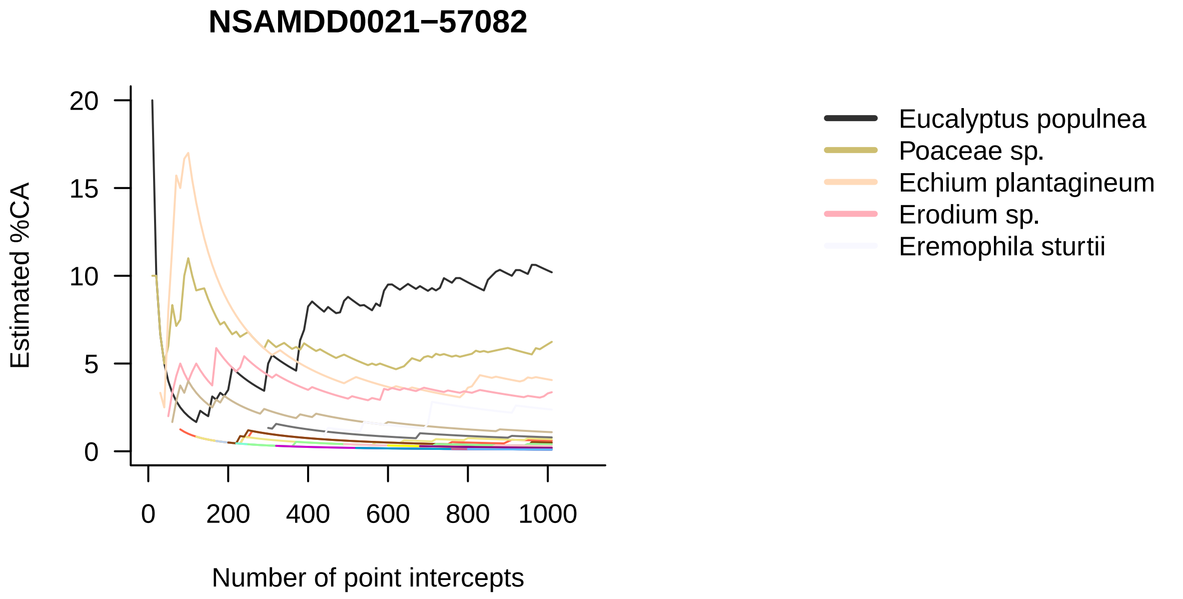
NSAMDD0021−57082
Estimated %CA
0
5
10
15
20
0
200
400
600
800
1000
Number of point intercepts
Eucalyptus populnea
Poaceae sp.
Echium plantagineum
Erodium sp.
Eremophila sturtii

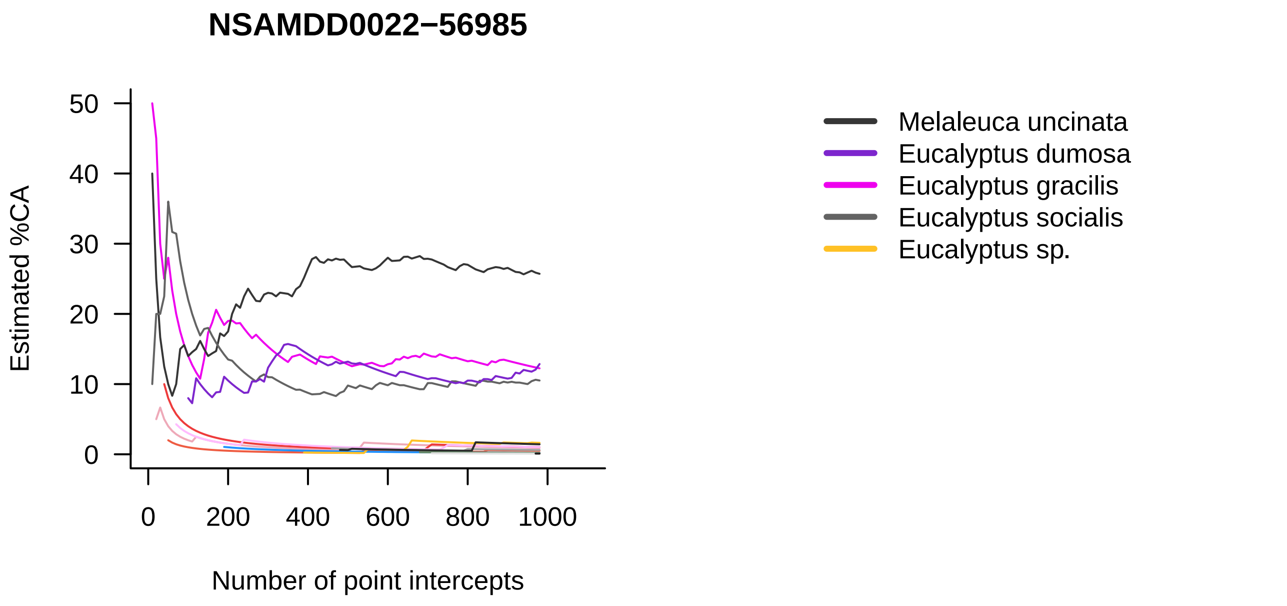
NSAMDD0022−56985
Estimated %CA
0
10
20
30
40
50
0
200
400
600
800
1000
Number of point intercepts
Melaleuca uncinata
Eucalyptus dumosa
Eucalyptus gracilis
Eucalyptus socialis
Eucalyptus sp.

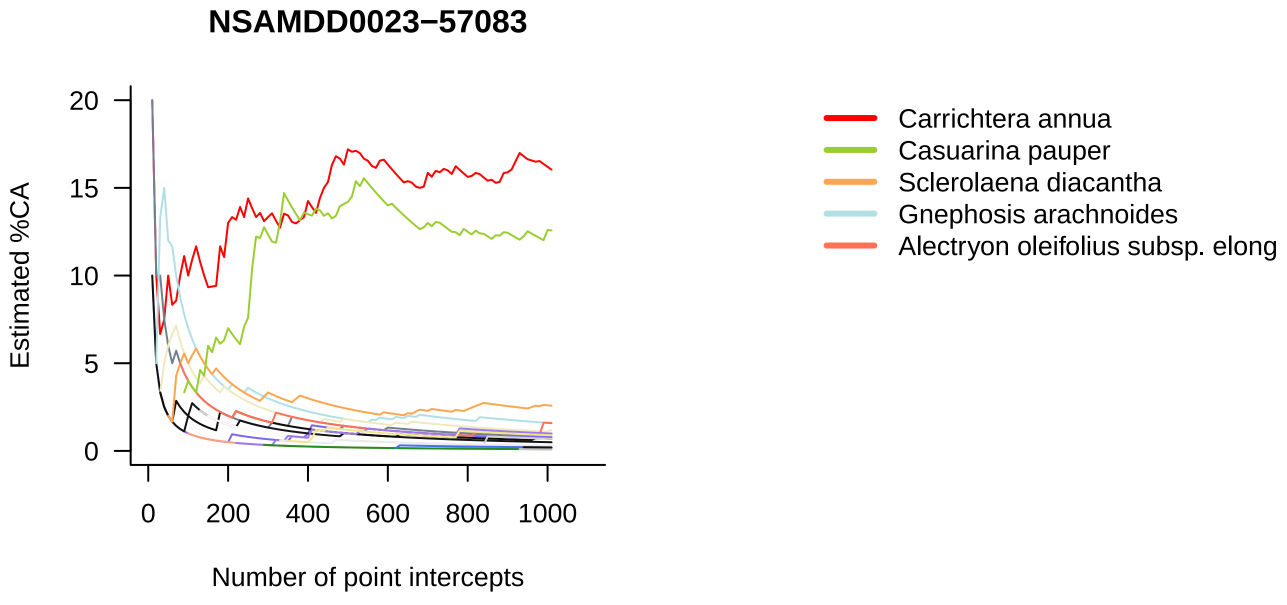
NSAMDD0023−57083
Estimated %CA
0
5
10
15
20
0
200
400
600
800
1000
Number of point intercepts
Carrichtera annua
Casuarina pauper
Sclerolaena diacantha
Gnephosis arachnoides
Alectryon oleifolius subsp. elong

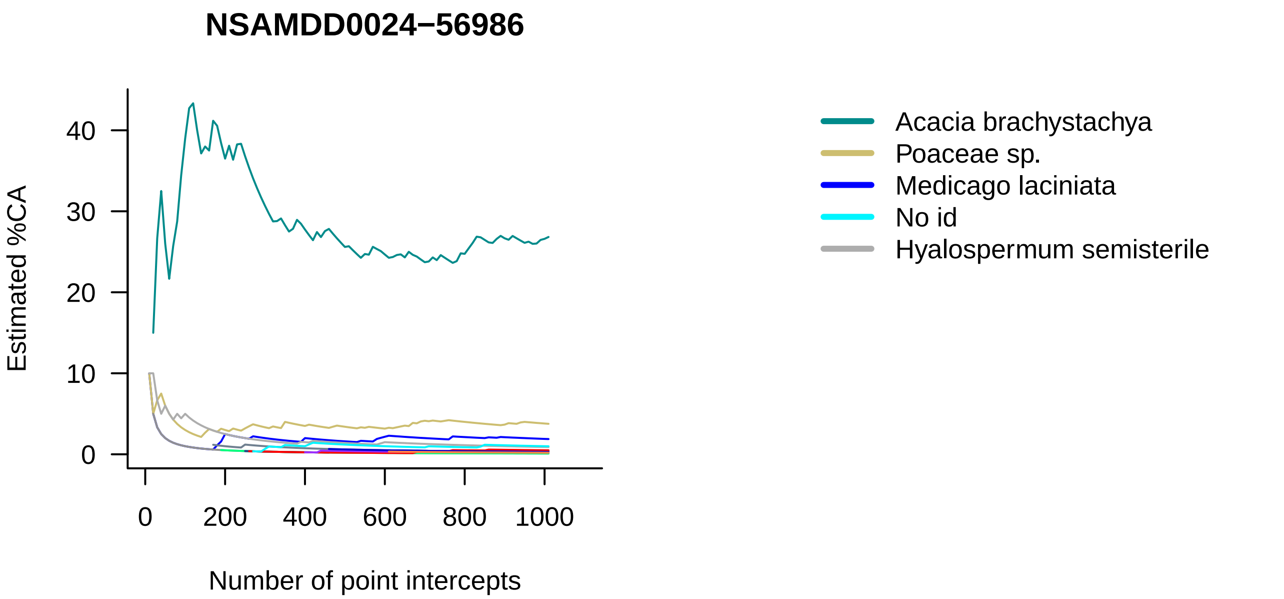
NSAMDD0024−56986
Estimated %CA
0
10
20
30
40
0
200
400
600
800
1000
Number of point intercepts
Acacia brachystachya
Poaceae sp.
Medicago laciniata
No id
Hyalospermum semisterile

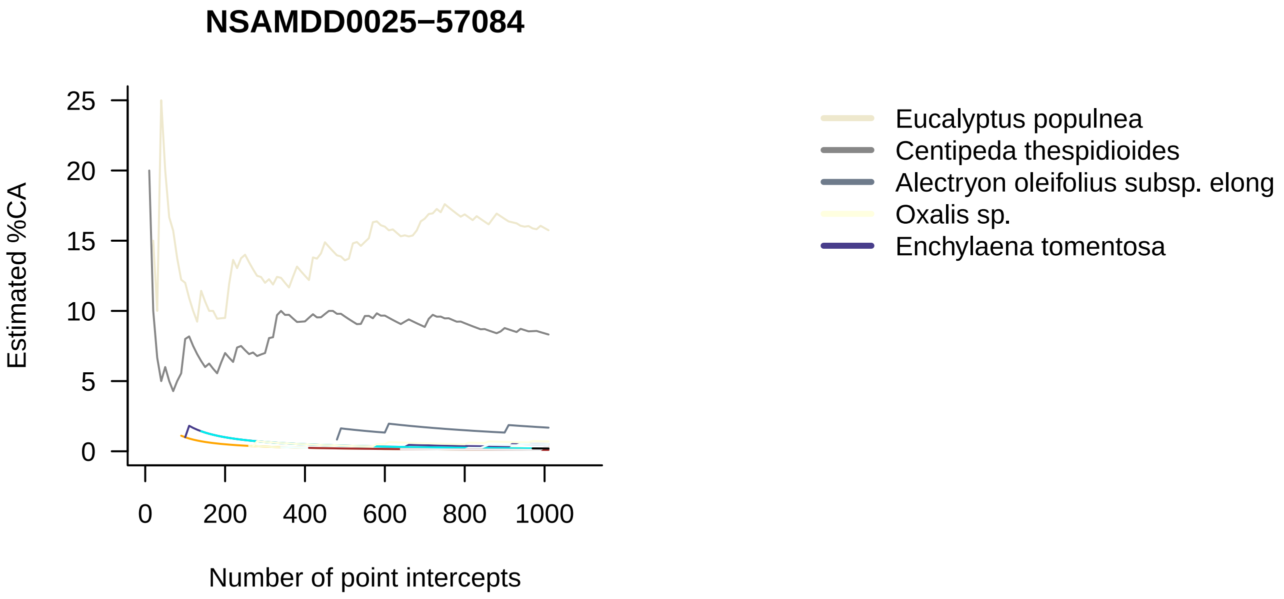
NSAMDD0025−57084
Estimated %CA
0
5
10
15
20
25
0
200
400
600
800
1000
Number of point intercepts
Eucalyptus populnea
Centipeda thespidioides
Alectryon oleifolius subsp. elong
Oxalis sp.
Enchylaena tomentosa

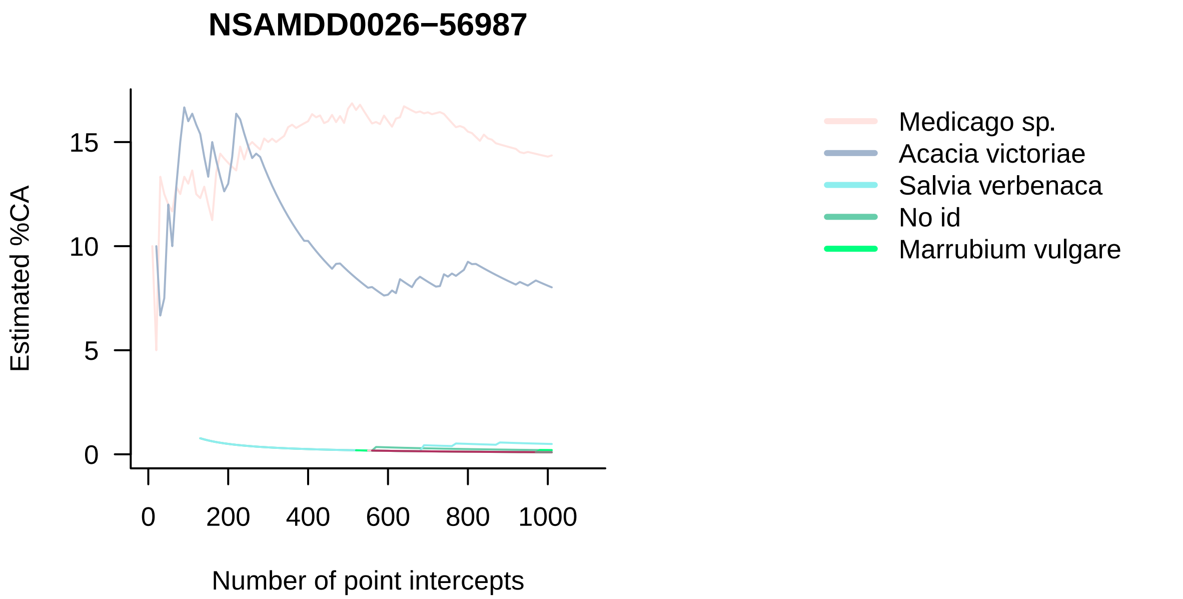
NSAMDD0026−56987
Estimated %CA
0
5
10
15
0
200
400
600
800
1000
Number of point intercepts
Medicago sp.
Acacia victoriae
Salvia verbenaca
No id
Marrubium vulgare

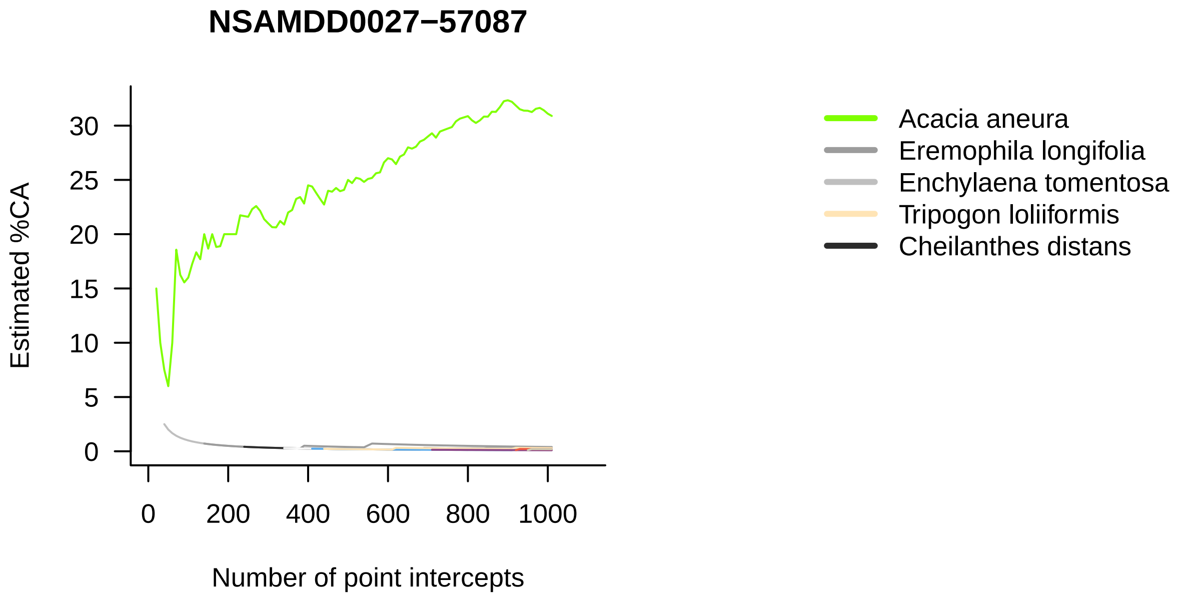
NSAMDD0027−57087
Estimated %CA
0
5
10
15
20
25
30
0
200
400
600
800
1000
Number of point intercepts
Acacia aneura
Eremophila longifolia
Enchylaena tomentosa
Tripogon loliiformis
Cheilanthes distans

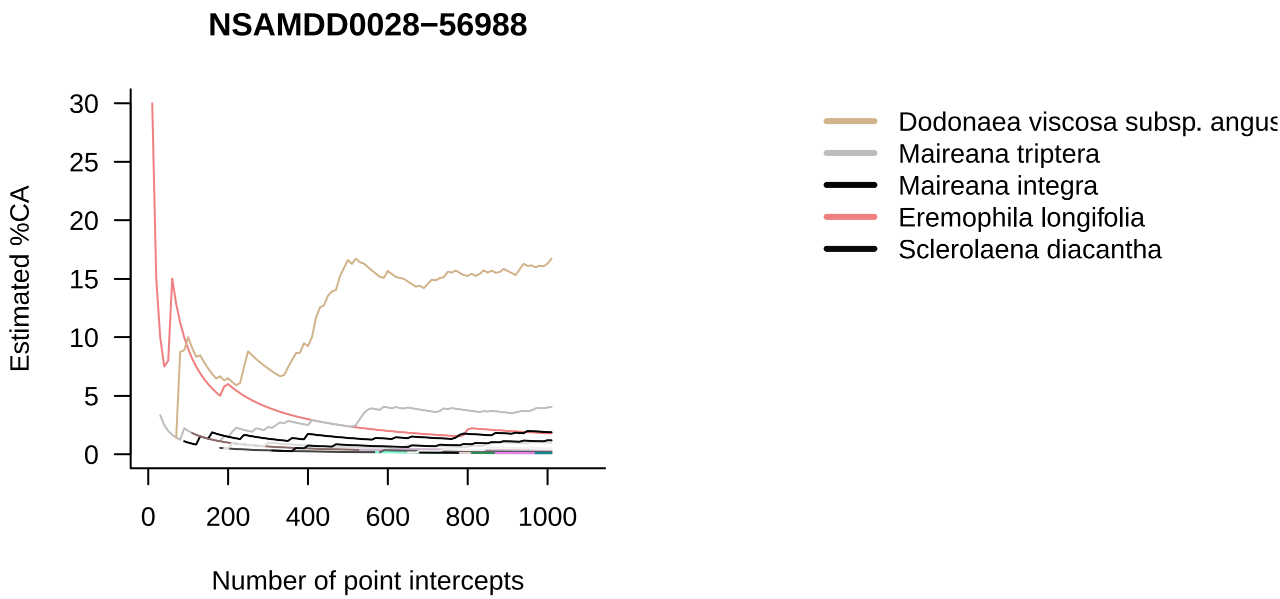

NSAMDD0028−56988
30
25
20
15
10
5
0
Estimated %CA
0
200
400
600
800
1000
Number of point intercepts
Dodonaea viscosa subsp. angus
Maireana triptera
Maireana integra
Eremophila longifolia
Sclerolaena diacantha


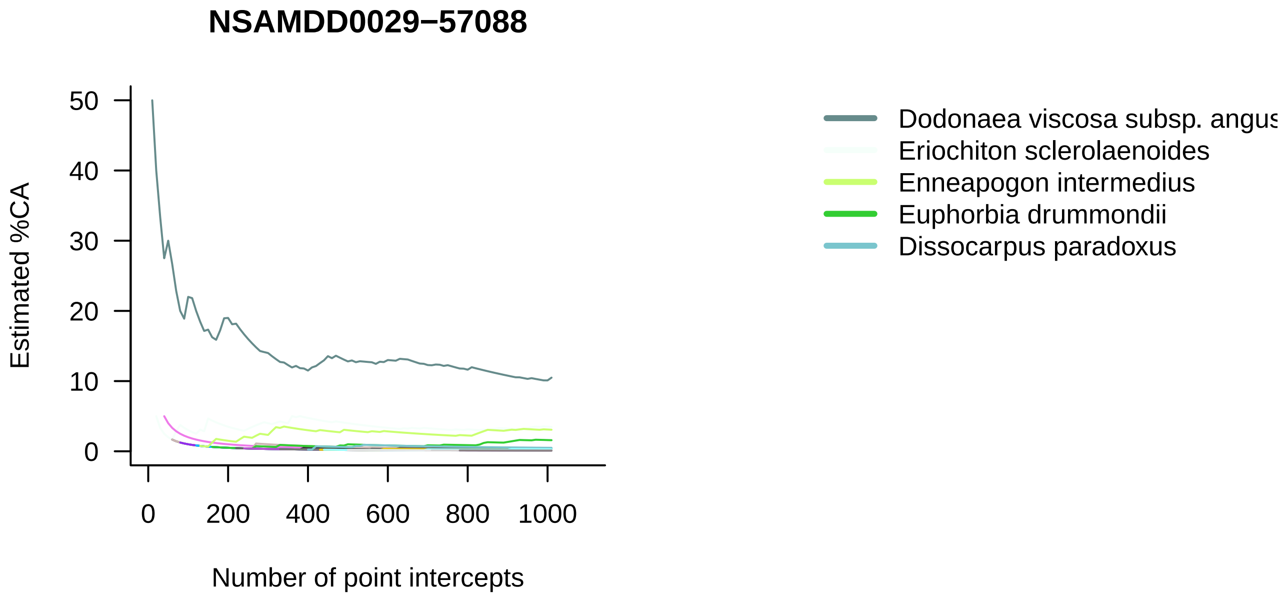

NSAMDD0029−57088
50
40
30
20
10
0
Estimated %CA
0
200
400
600
800
1000
Number of point intercepts
Dodonaea viscosa subsp. angus
Eriochiton sclerolaenoides
Enneapogon intermedius
Euphorbia drummondii
Dissocarpus paradoxus

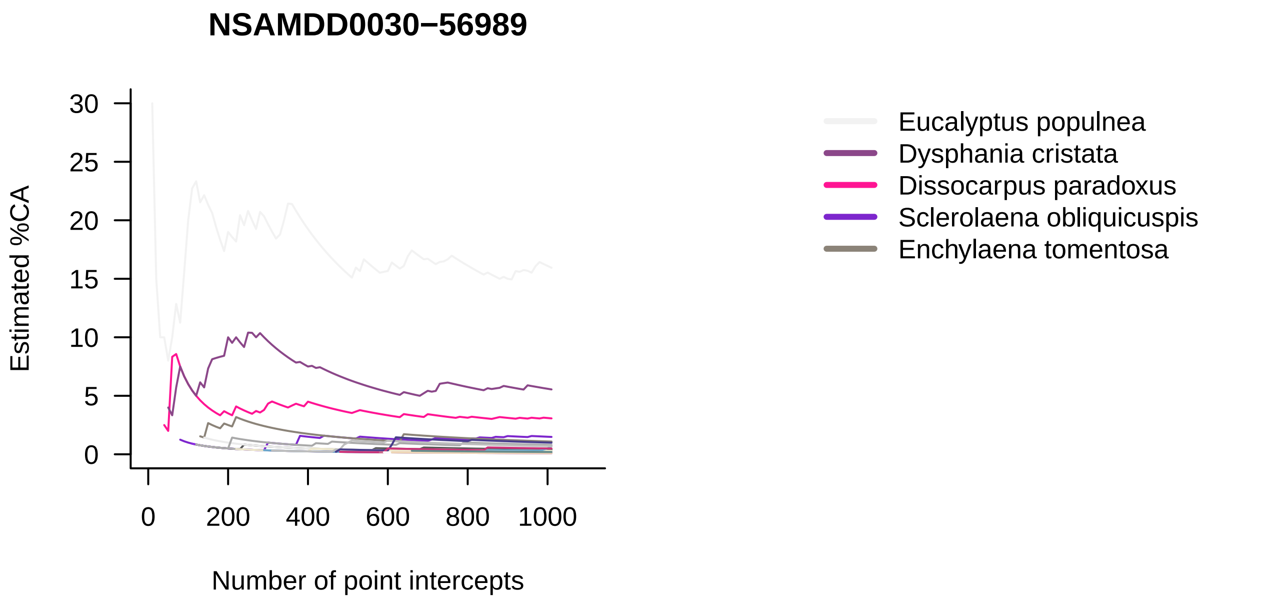
NSAMDD0030−56989
Estimated %CA
30
25
20
15
10
5
0
0
200
400
600
800
1000
Number of point intercepts
Eucalyptus populnea
Dysphania cristata
Dissocarpus paradoxus
Sclerolaena obliquicuspis
Enchylaena tomentosa

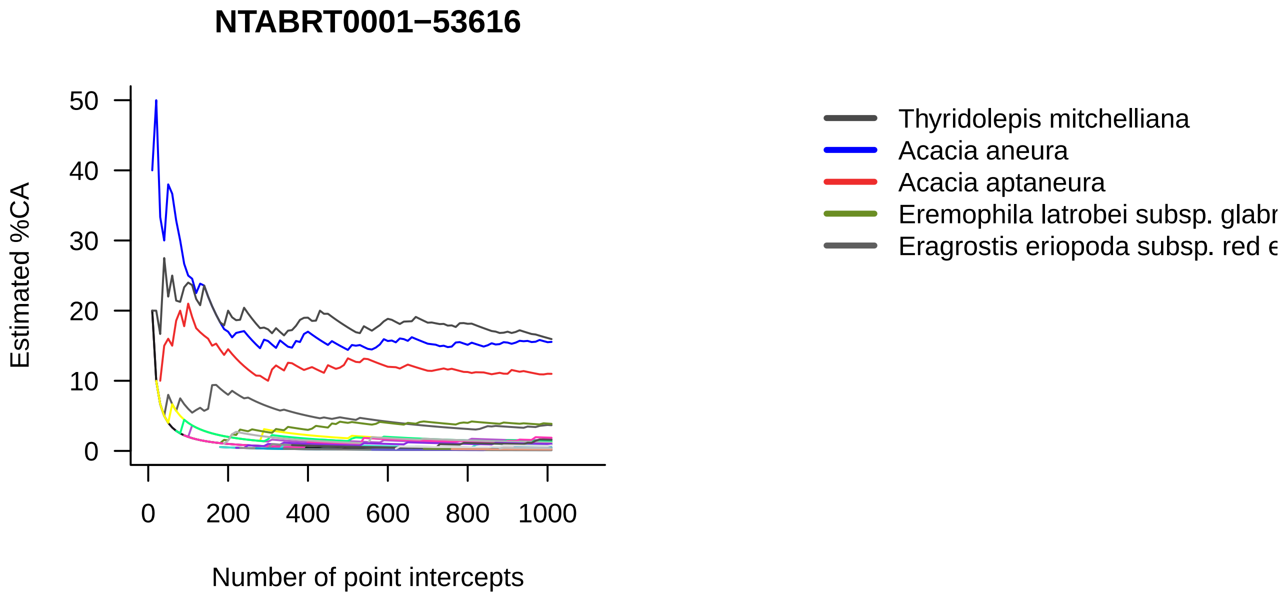
NTABRT0001−53616
Estimated %CA
50
40
30
20
10
0
0
200
400
600
800
1000
Number of point intercepts
Thyridolepis mitchelliana
Acacia aneura
Acacia aptaneura
Eremophila latrobei subsp. glabr
Eragrostis eriopoda subsp. red e

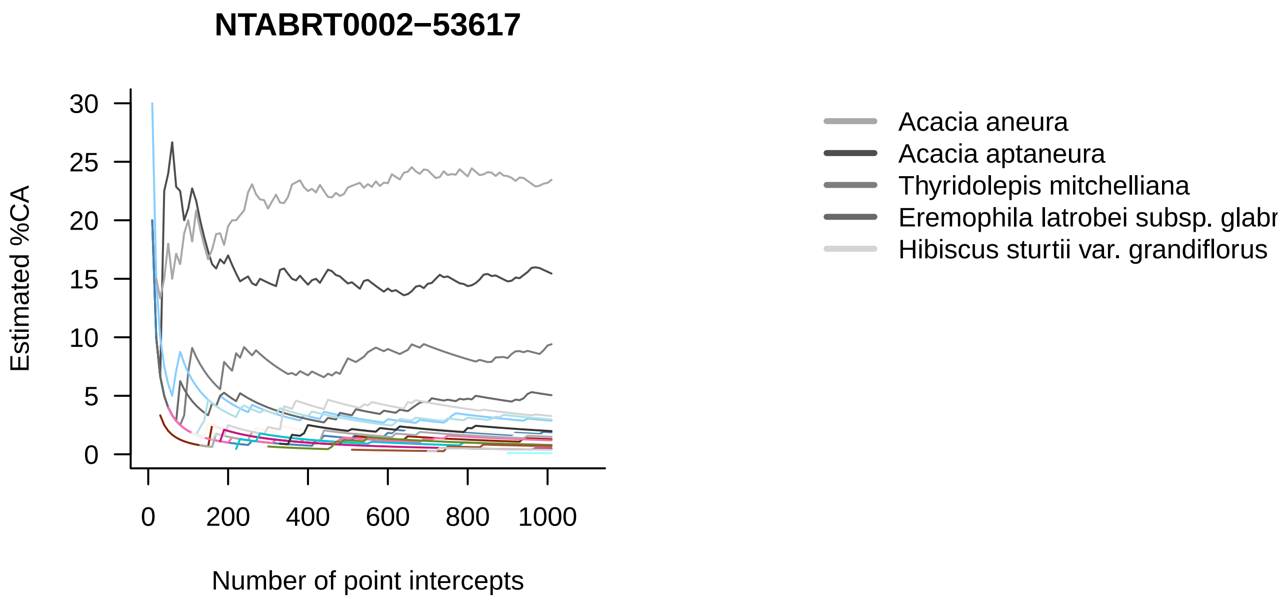
NTABRT0002−53617
Estimated %CA
Number of point intercepts
0
5
10
15
20
25
30
0
200
400
600
800
1000
Acacia aneura
Acacia aptaneura
Thyridolepis mitchelliana
Eremophila latrobei subsp. glabr
Hibiscus sturtii var. grandiflorus

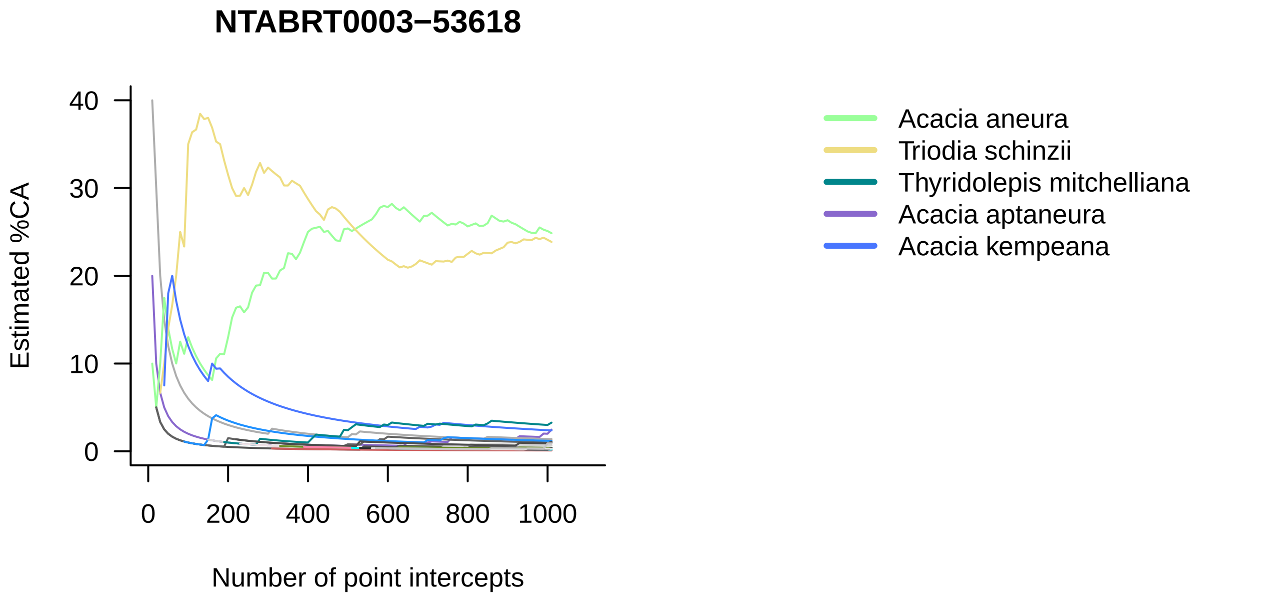
NTABRT0003−53618
Estimated %CA
Number of point intercepts
0
10
20
30
40
0
200
400
600
800
1000
Acacia aneura
Triodia schinzii
Thyridolepis mitchelliana
Acacia aptaneura
Acacia kempeana

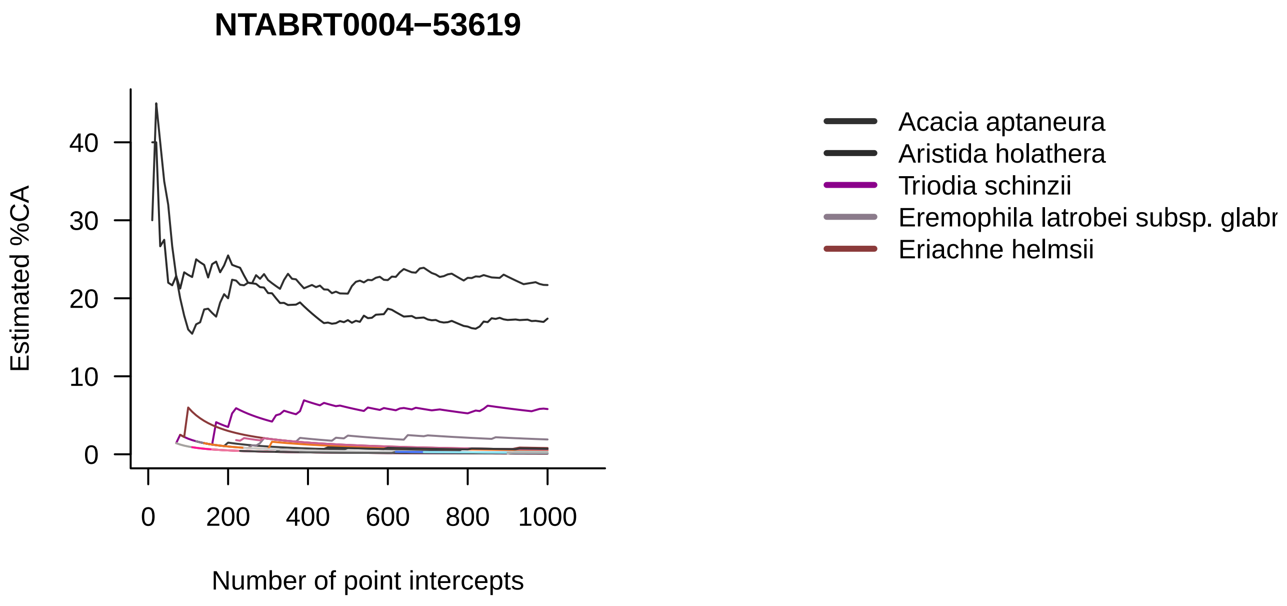
NTABRT0004−53619
Estimated %CA
Number of point intercepts
0
10
20
30
40
0
200
400
600
800
1000
Acacia aptaneura
Aristida holathera
Triodia schinzii
Eremophila latrobei subsp. glabr
Eriachne helmsii

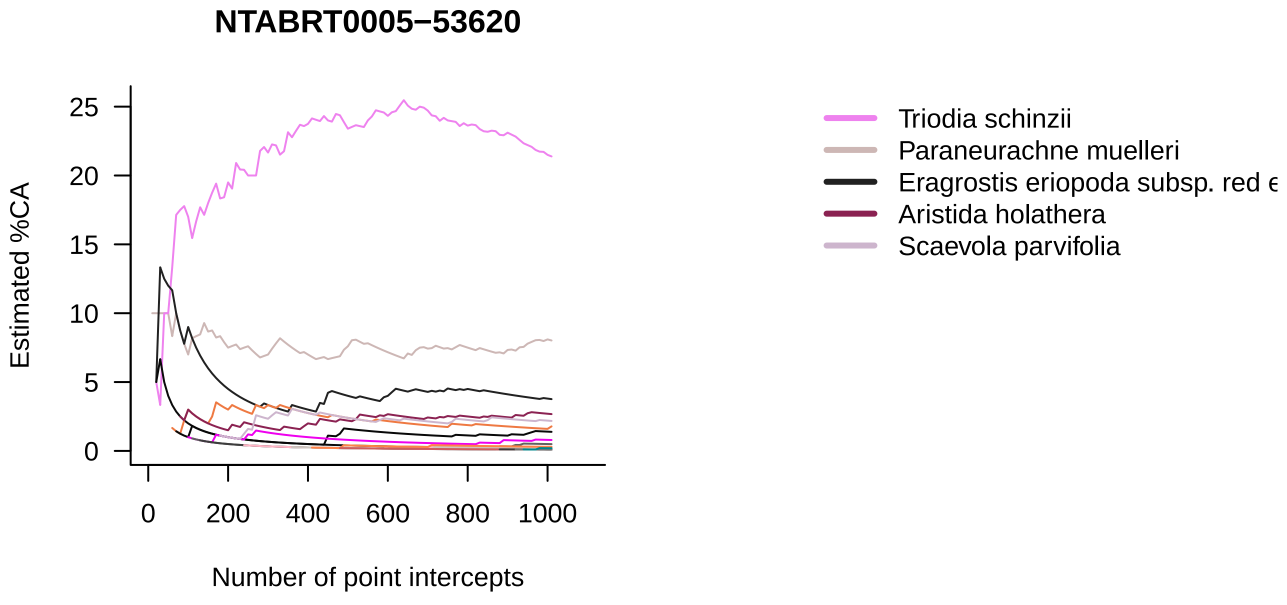
NTABRT0005−53620
Estimated %CA
Number of point intercepts
0
5
10
15
20
25
0
200
400
600
800
1000
Triodia schinzii
Paraneurachne muelleri
Eragrostis eriopoda subsp. red e
Aristida holathera
Scaevola parvifolia

## NTABRT0006−53621

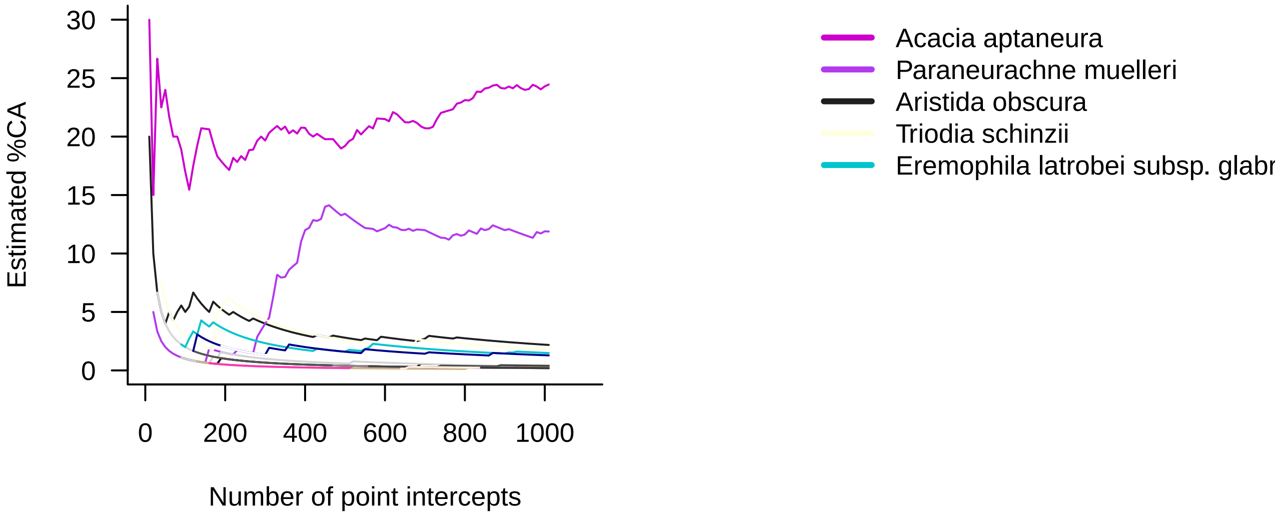


## NTADAC0001−53518

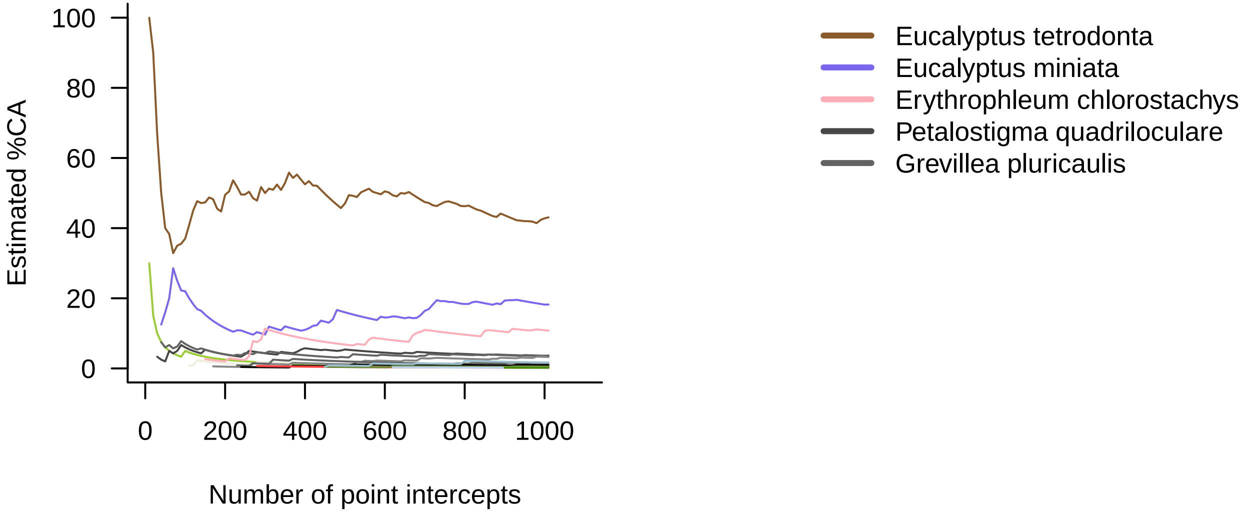

**NTAFIN0001−53519**

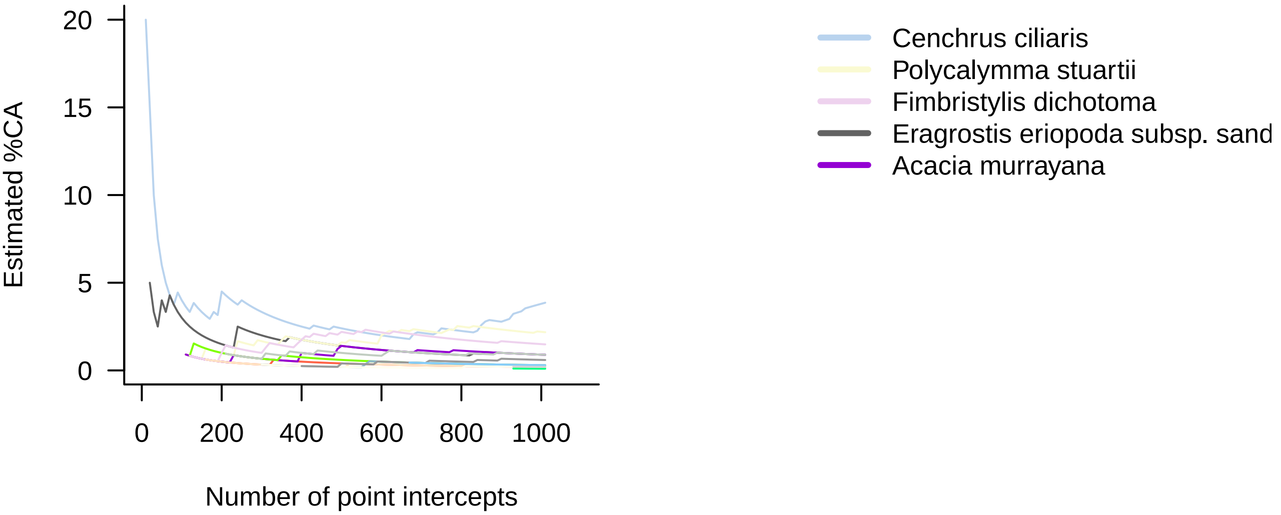


**NTAFIN0002−53622**

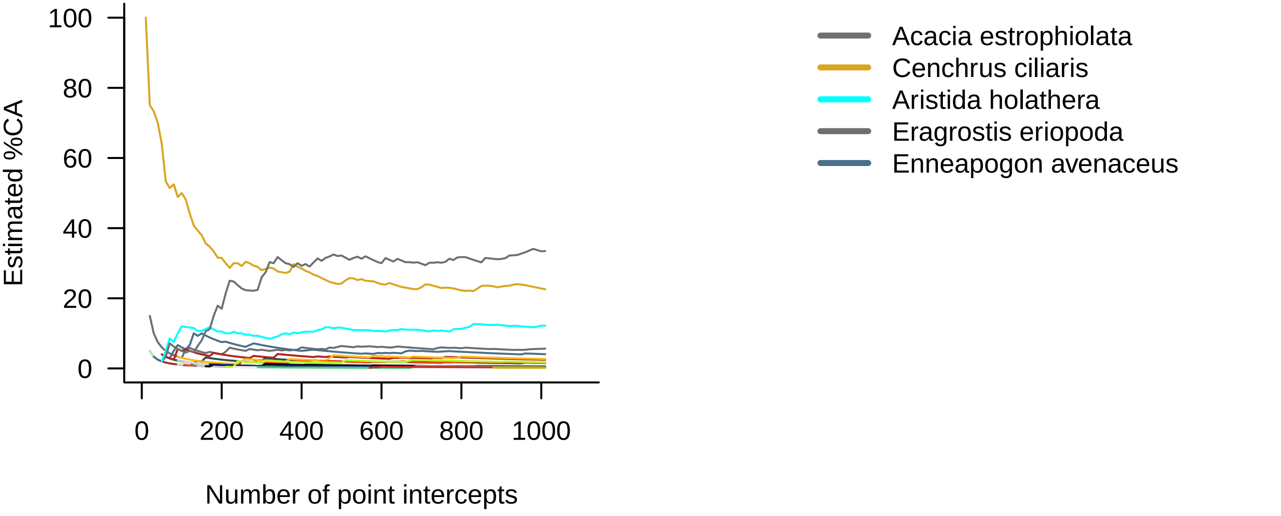

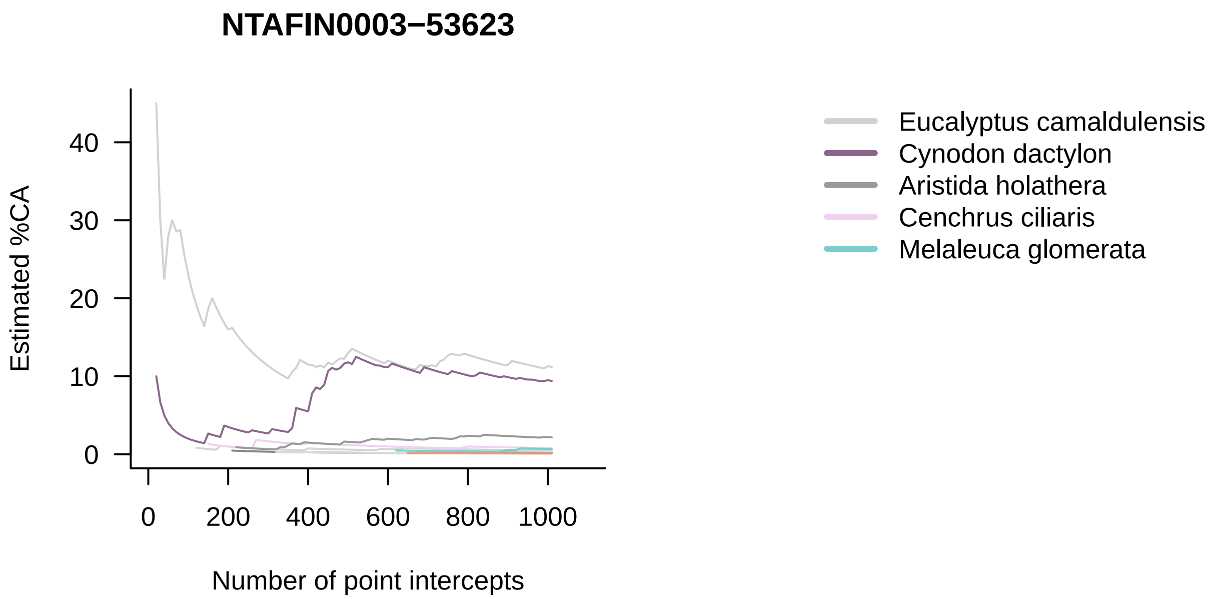
NTAFIN0003−53623
Estimated %CA
0
10
20
30
40
0
200
400
600
800
1000
Number of point intercepts
Eucalyptus camaldulensis
Cynodon dactylon
Aristida holathera
Cenchrus ciliaris
Melaleuca glomerata

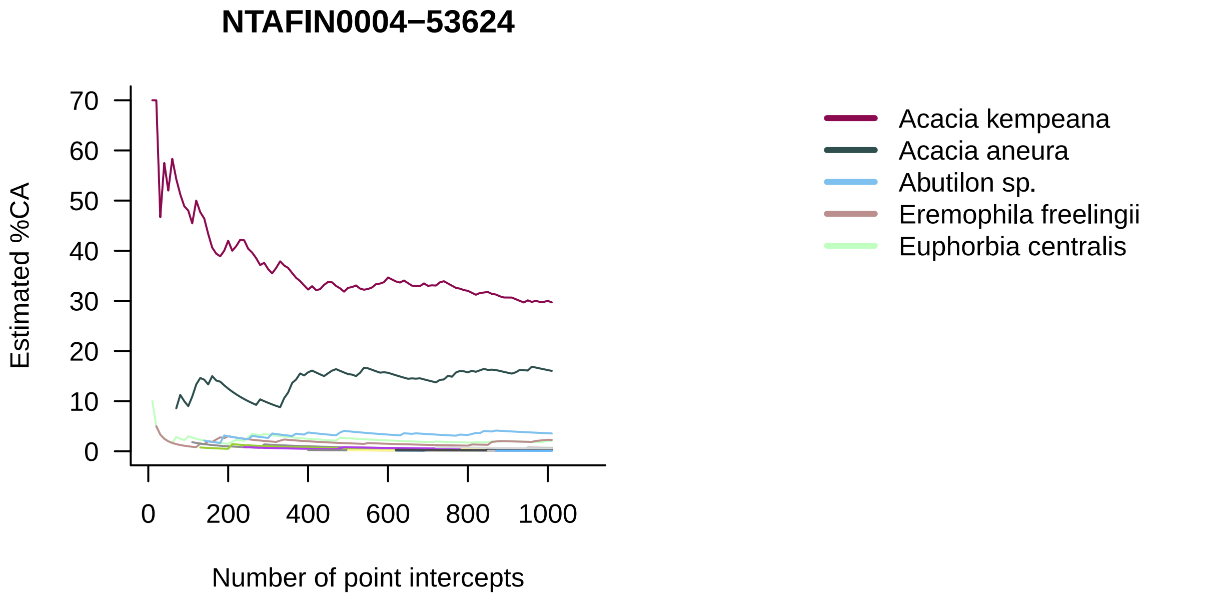
NTAFIN0004−53624
Estimated %CA
0
10
20
30
40
50
60
70
0
200
400
600
800
1000
Number of point intercepts
Acacia kempeana
Acacia aneura
Abutilon sp.
Eremophila freelingii
Euphorbia centralis

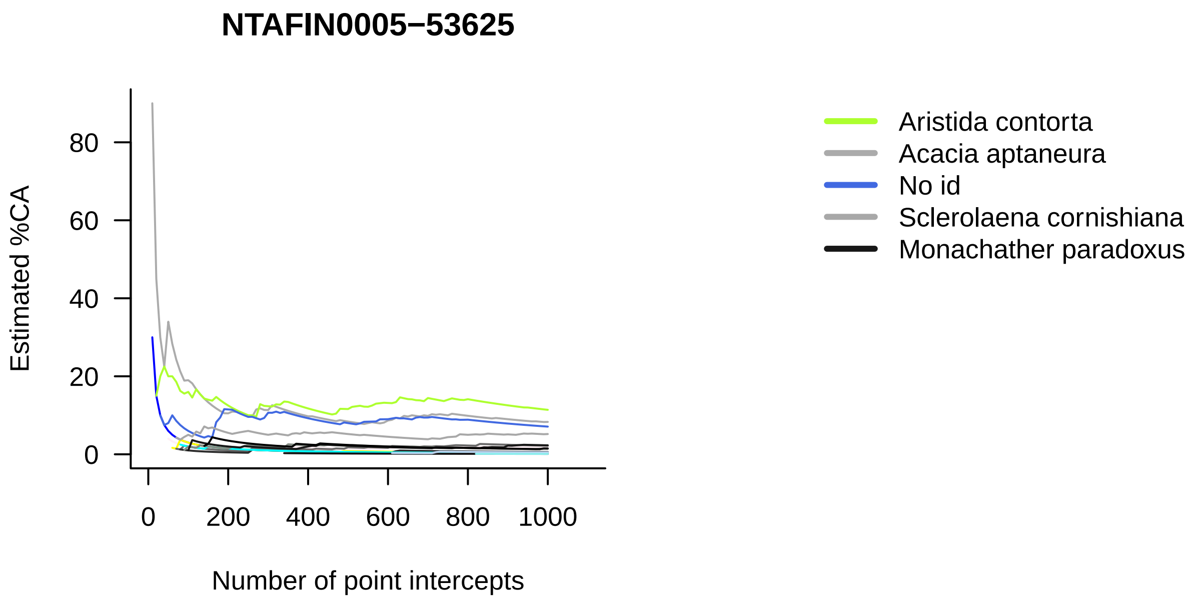
NTAFIN0005−53625
Estimated %CA
0
20
40
60
80
0
200
400
600
800
1000
Number of point intercepts
Aristida contorta
Acacia aptaneura
No id
Sclerolaena cornishiana
Monachather paradoxus

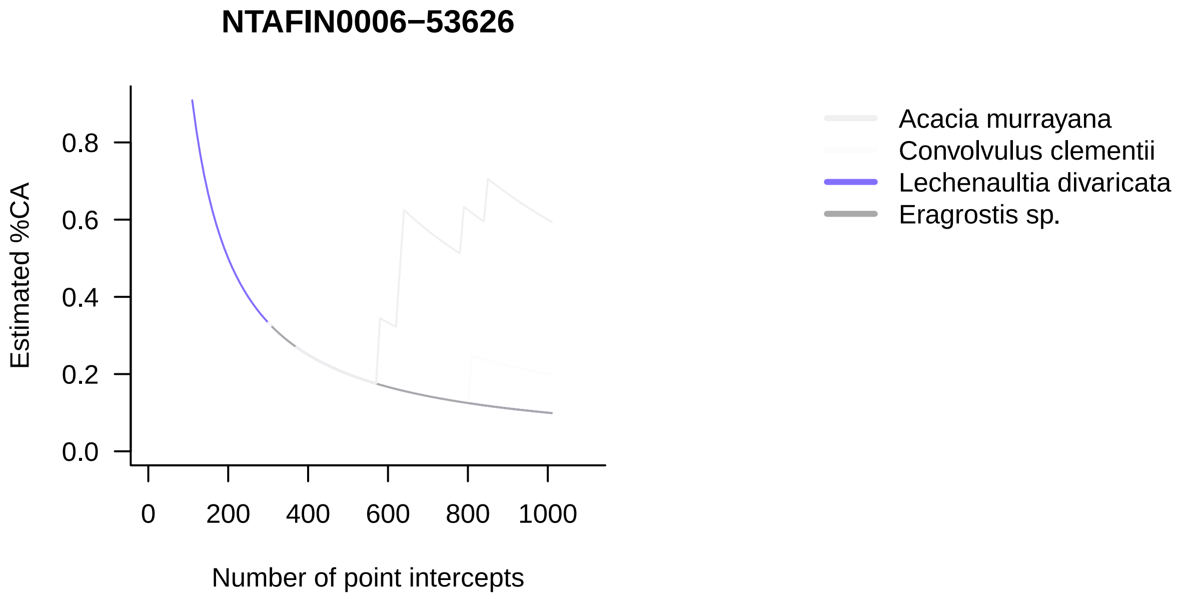
NTAFIN0006−53626
Estimated %CA
0.0
0.2
0.4
0.6
0.8
0
200
400
600
800
1000
Number of point intercepts
Acacia murrayana
Convolvulus clementii
Lechenaultia divaricata
Eragrostis sp.

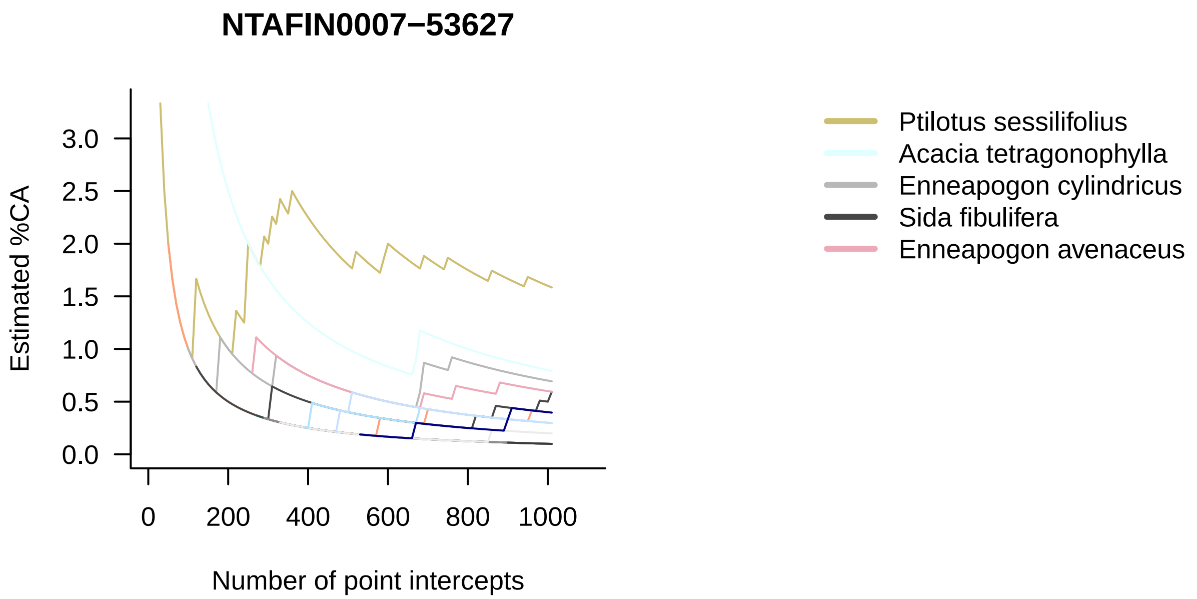
NTAFIN0007−53627
Estimated %CA
3.0
2.5
2.0
1.5
1.0
0.5
0.0
0
200
400
600
800
1000
Number of point intercepts
Ptilotus sessilifolius
Acacia tetragonophylla
Enneapogon cylindricus
Sida fibulifera
Enneapogon avenaceus

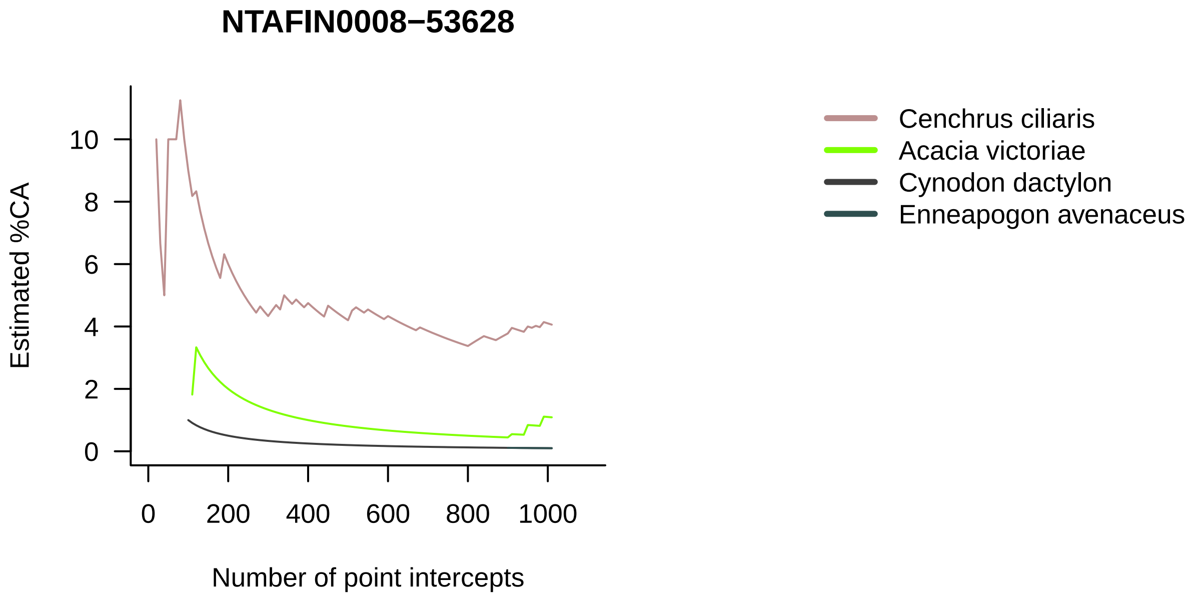
NTAFIN0008−53628
Estimated %CA
10
8
6
4
2
0
0
200
400
600
800
1000
Number of point intercepts
Cenchrus ciliaris
Acacia victoriae
Cynodon dactylon
Enneapogon avenaceus

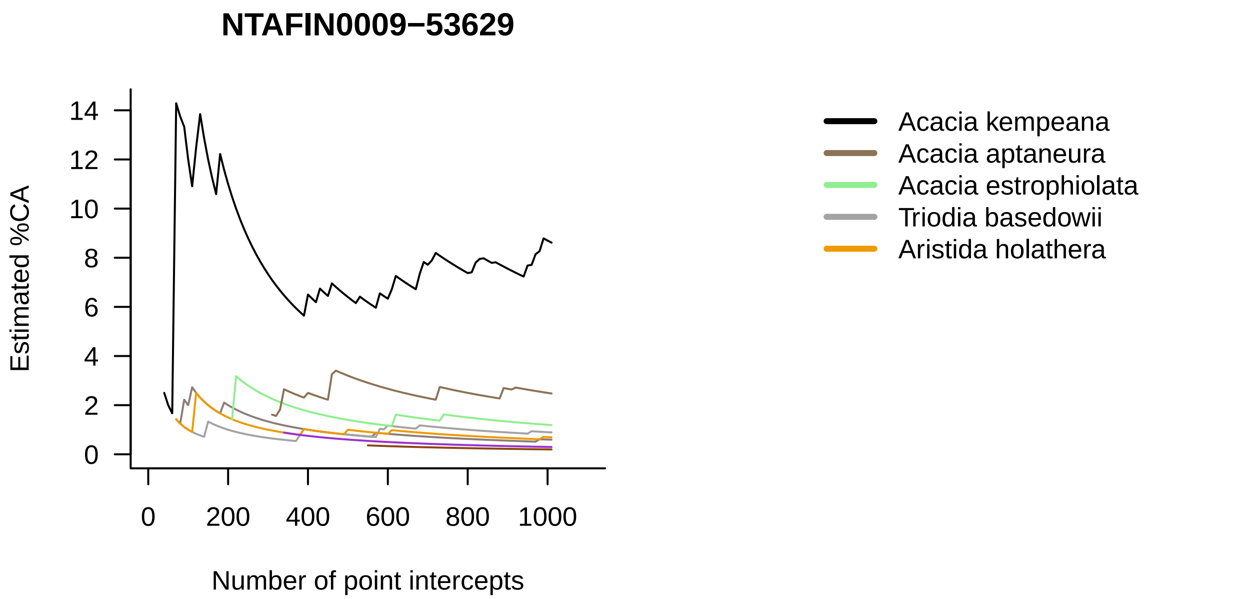
NTAFIN0009−53629
Estimated %CA
0
2
4
6
8
10
12
14
0
200
400
600
800
1000
Number of point intercepts
Acacia kempeana
Acacia aptaneura
Acacia estrophiolata
Triodia basedowii
Aristida holathera

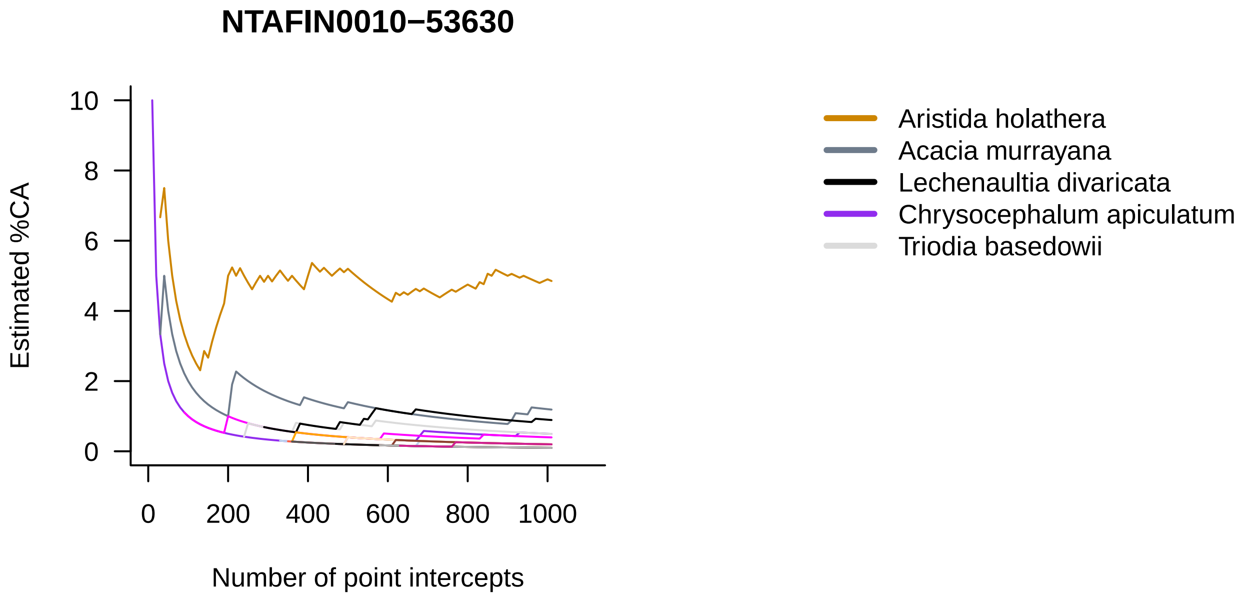
NTAFIN0010−53630
Estimated %CA
0
2
4
6
8
10
0
200
400
600
800
1000
Number of point intercepts
Aristida holathera
Acacia murrayana
Lechenaultia divaricata
Chrysocephalum apiculatum
Triodia basedowii

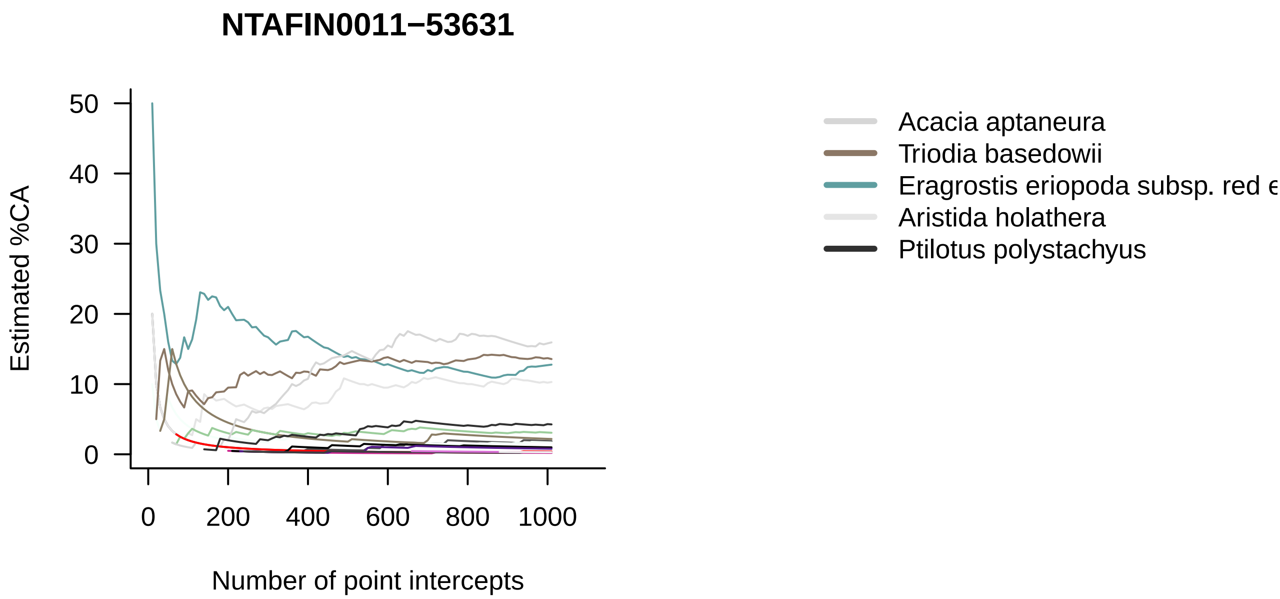
NTAFIN0011−53631
Estimated %CA
50
40
30
20
10
0
0
200
400
600
800
1000
Number of point intercepts
Acacia aptaneura
Triodia basedowii
Eragrostis eriopoda subsp. red e
Aristida holathera
Ptilotus polystachyus

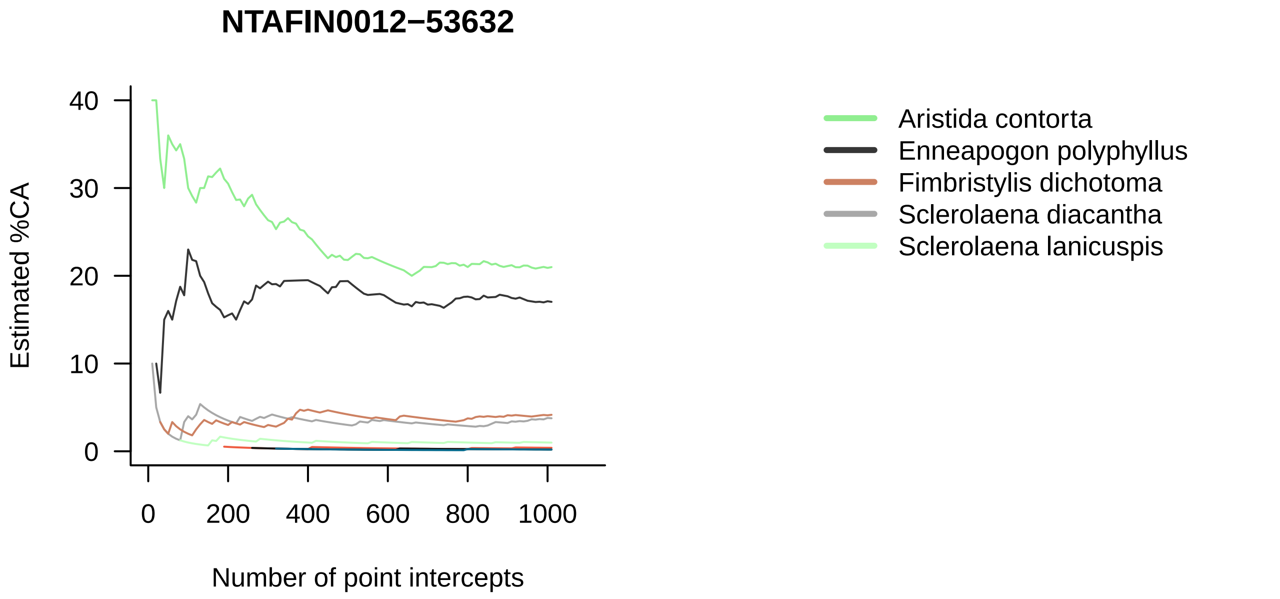
NTAFIN0012−53632
Estimated %CA
40
30
20
10
0
0
200
400
600
800
1000
Number of point intercepts
Aristida contorta
Enneapogon polyphyllus
Fimbristylis dichotoma
Sclerolaena diacantha
Sclerolaena lanicuspis

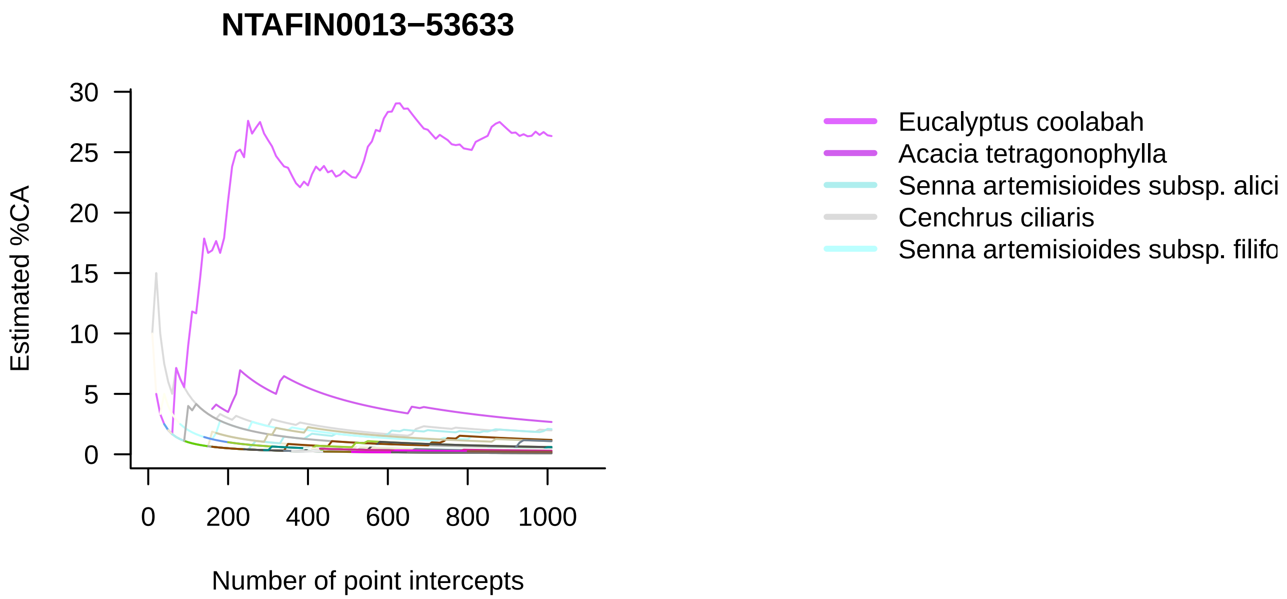
NTAFIN0013−53633
Estimated %CA
0
5
10
15
20
25
30
0
200
400
600
800
1000
Number of point intercepts
Eucalyptus coolabah
Acacia tetragonophylla
Senna artemisioides subsp. alici
Cenchrus ciliaris
Senna artemisioides subsp. filifo

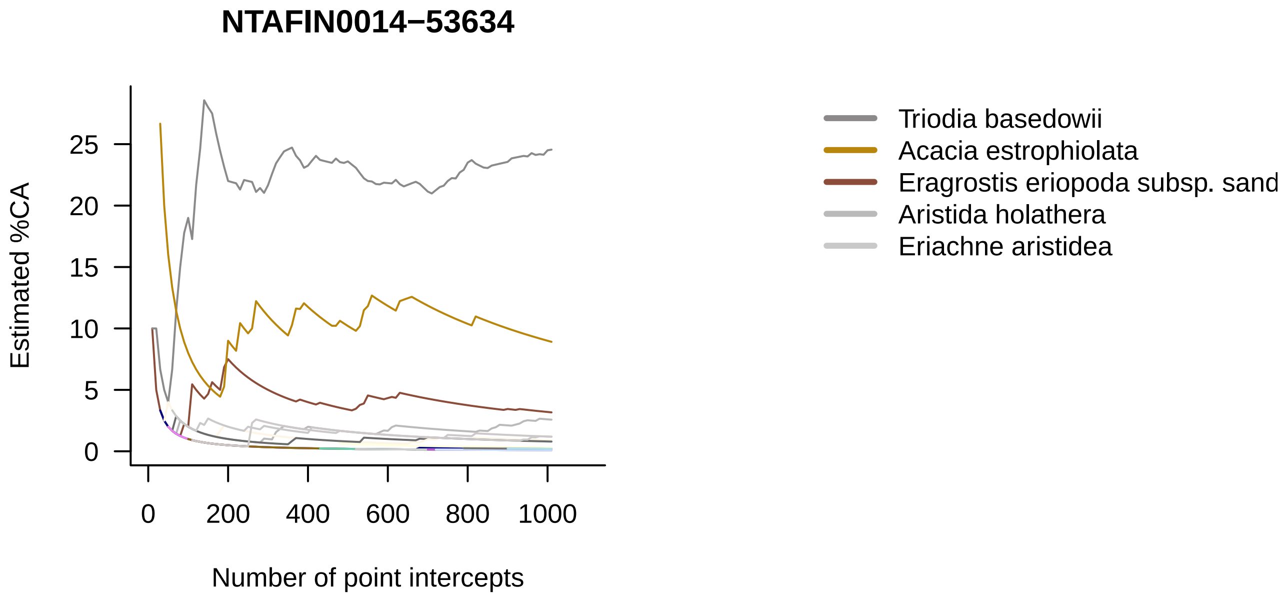
NTAFIN0014−53634
Estimated %CA
0
5
10
15
20
25
0
200
400
600
800
1000
Number of point intercepts
Triodia basedowii
Acacia estrophiolata
Eragrostis eriopoda subsp. sand
Aristida holathera
Eriachne aristidea

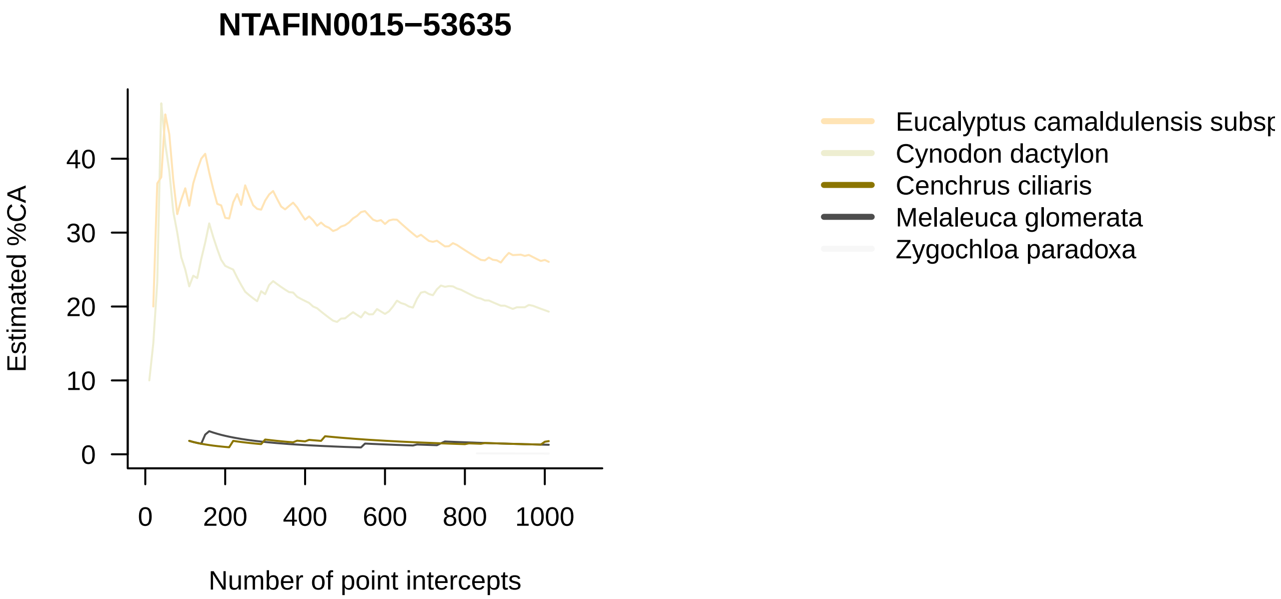
NTAFIN0015−53635
Estimated %CA
Number of point intercepts
0
10
20
30
40
0
200
400
600
800
1000
Eucalyptus camaldulensis subsp
Cynodon dactylon
Cenchrus ciliaris
Melaleuca glomerata
Zygochloa paradoxa

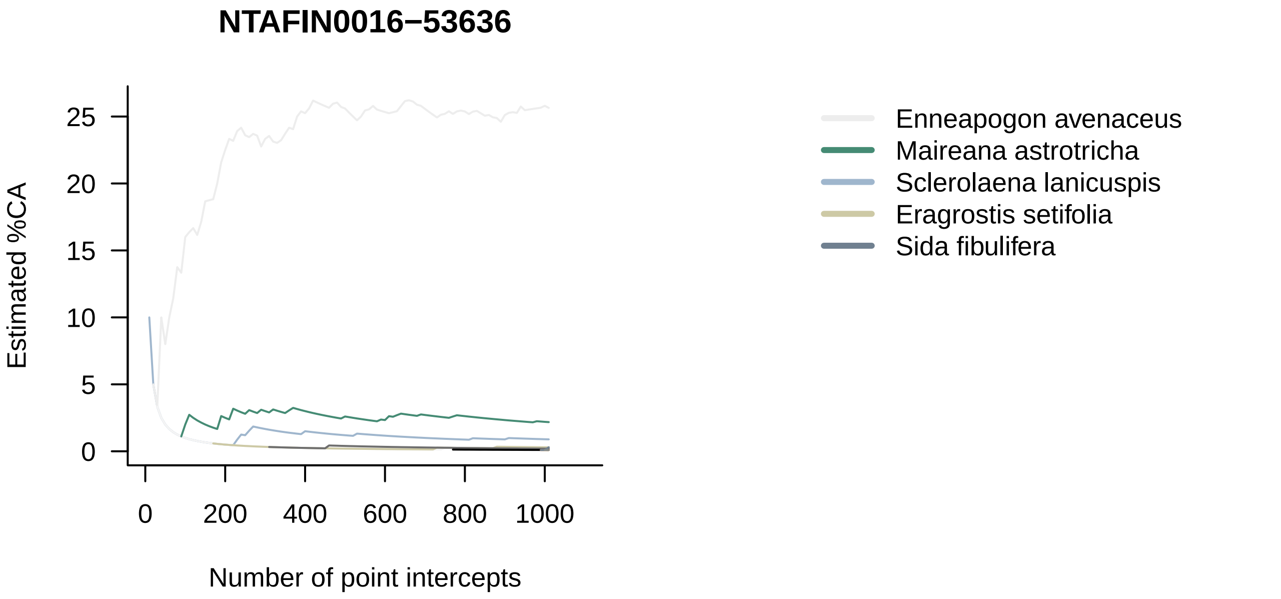
NTAFIN0016−53636
Estimated %CA
Number of point intercepts
0
5
10
15
20
25
0
200
400
600
800
1000
Enneapogon avenaceus
Maireana astrotricha
Sclerolaena lanicuspis
Eragrostis setifolia
Sida fibulifera

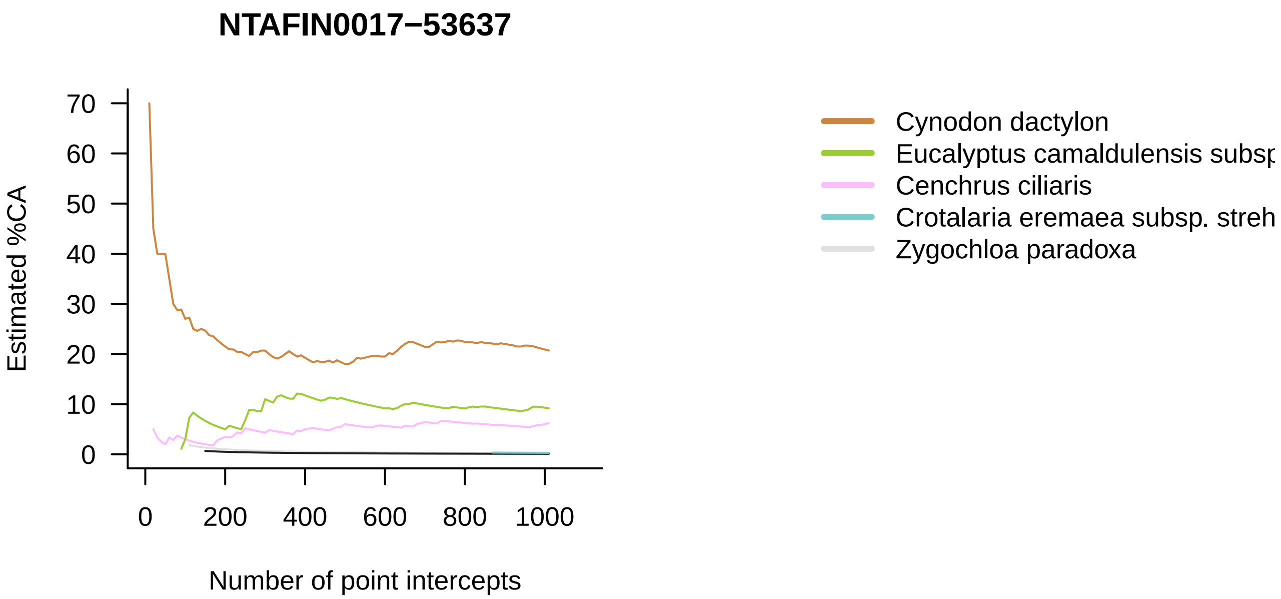
NTAFIN0017−53637
Estimated %CA
70
60
50
40
30
20
10
0
0
200
400
600
800
1000
Number of point intercepts
Cynodon dactylon
Eucalyptus camaldulensis subsp
Cenchrus ciliaris
Crotalaria eremaea subsp. streh
Zygochloa paradoxa

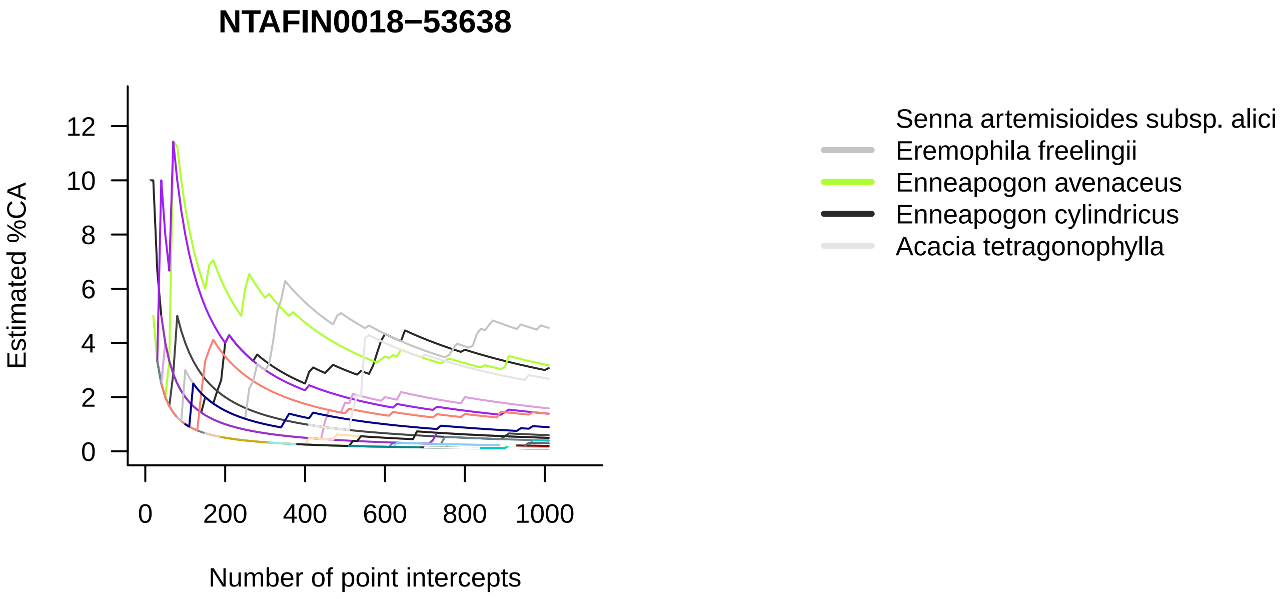
NTAFIN0018−53638
Estimated %CA
12
10
8
6
4
2
0
0
200
400
600
800
1000
Number of point intercepts
Senna artemisioides subsp. alici
Eremophila freelingii
Enneapogon avenaceus
Enneapogon cylindricus
Acacia tetragonophylla

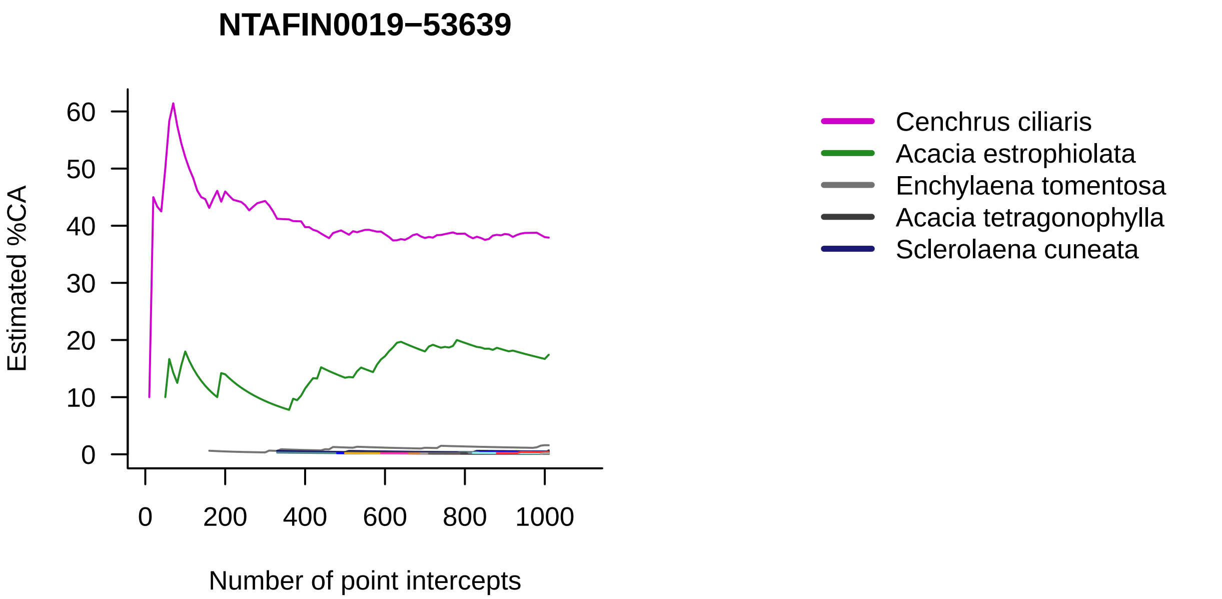
NTAFIN0019−53639
Estimated %CA
0
10
20
30
40
50
60
0
200
400
600
800
1000
Number of point intercepts
Cenchrus ciliaris
Acacia estrophiolata
Enchylaena tomentosa
Acacia tetragonophylla
Sclerolaena cuneata

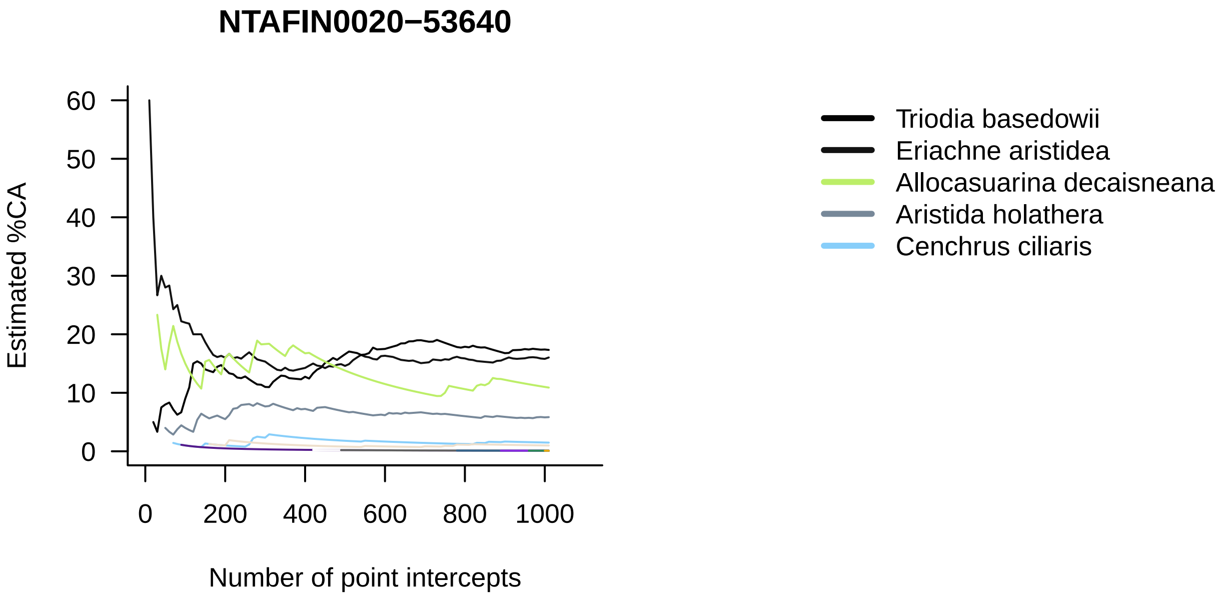
NTAFIN0020−53640
Estimated %CA
0
10
20
30
40
50
60
0
200
400
600
800
1000
Number of point intercepts
Triodia basedowii
Eriachne aristidea
Allocasuarina decaisneana
Aristida holathera
Cenchrus ciliaris

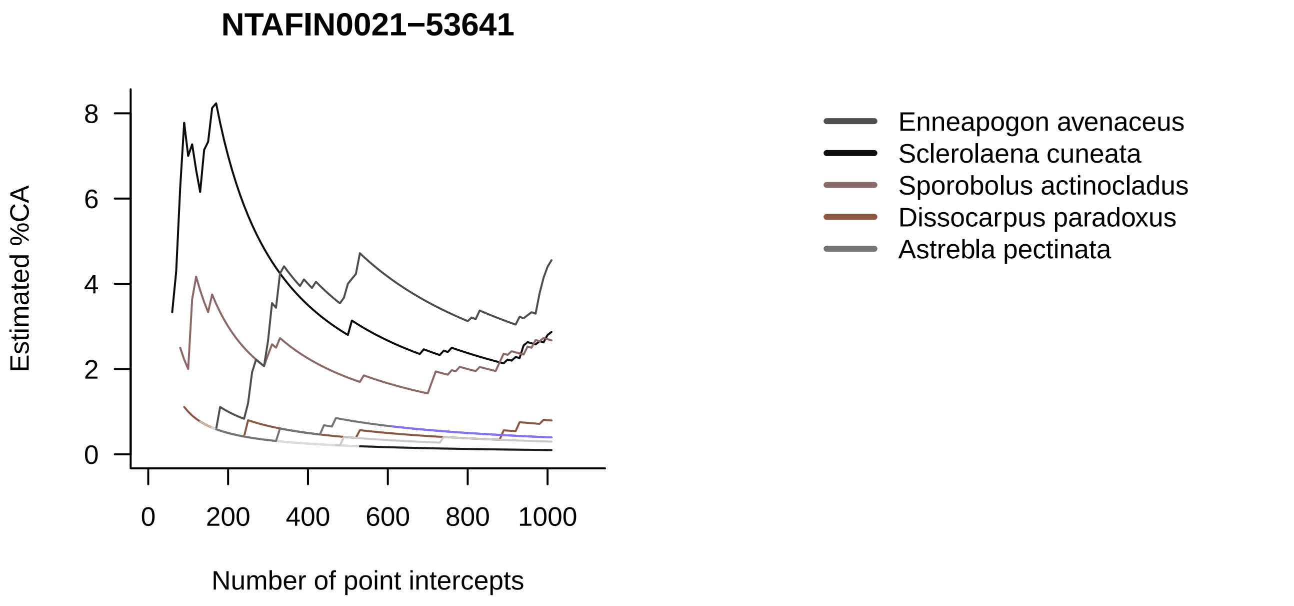
NTAFIN0021−53641
Estimated %CA
0
2
4
6
8
0
200
400
600
800
1000
Number of point intercepts
Enneapogon avenaceus
Sclerolaena cuneata
Sporobolus actinocladus
Dissocarpus paradoxus
Astrebla pectinata

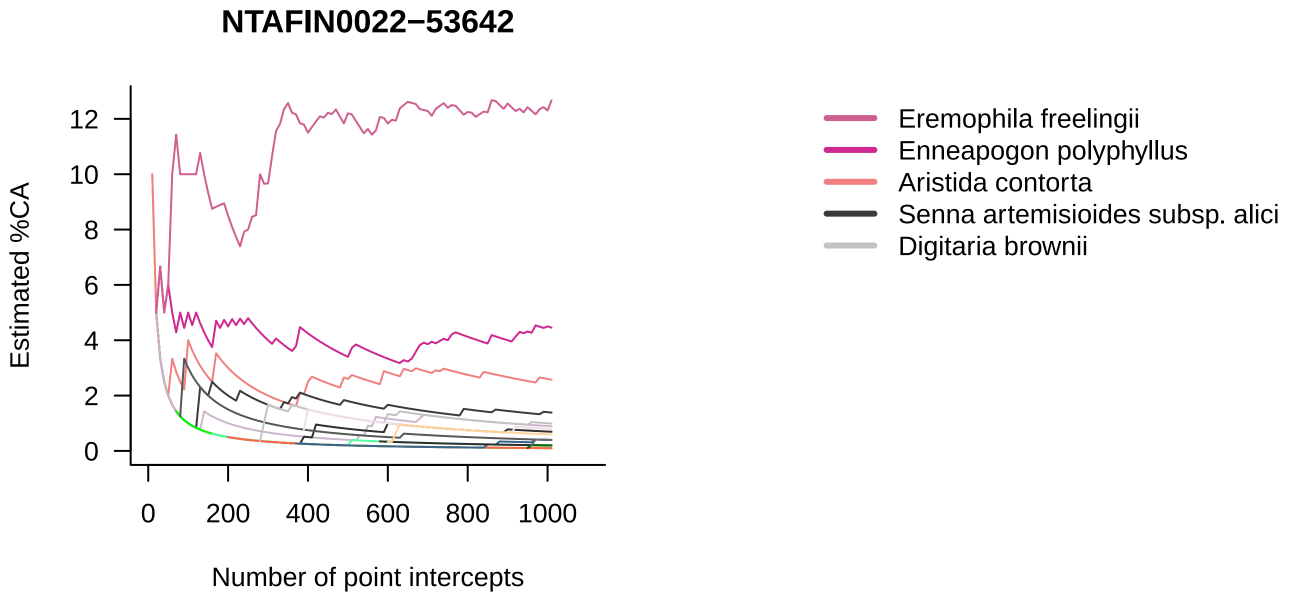
NTAFIN0022−53642
Estimated %CA
0
2
4
6
8
10
12
0
200
400
600
800
1000
Number of point intercepts
Eremophila freelingii
Enneapogon polyphyllus
Aristida contorta
Senna artemisioides subsp. alici
Digitaria brownii

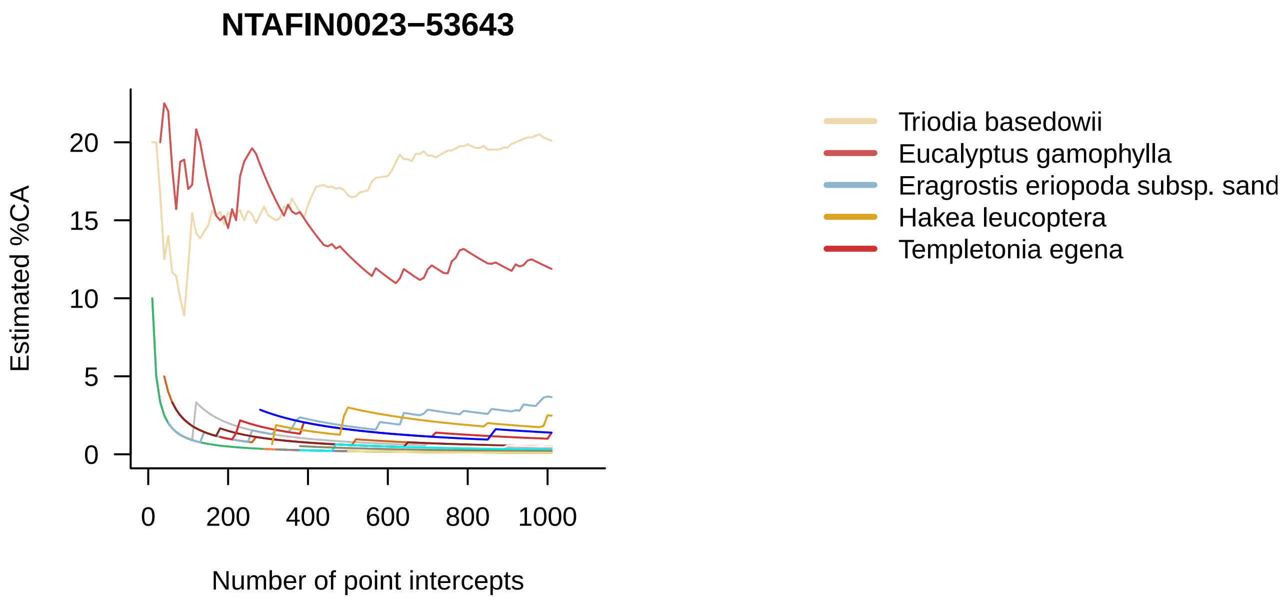
NTAFIN0023−53643
Estimated %CA
0
5
10
15
20
0
200
400
600
800
1000
Number of point intercepts
Triodia basedowii
Eucalyptus gamophylla
Eragrostis eriopoda subsp. sand
Hakea leucoptera
Templetonia egena

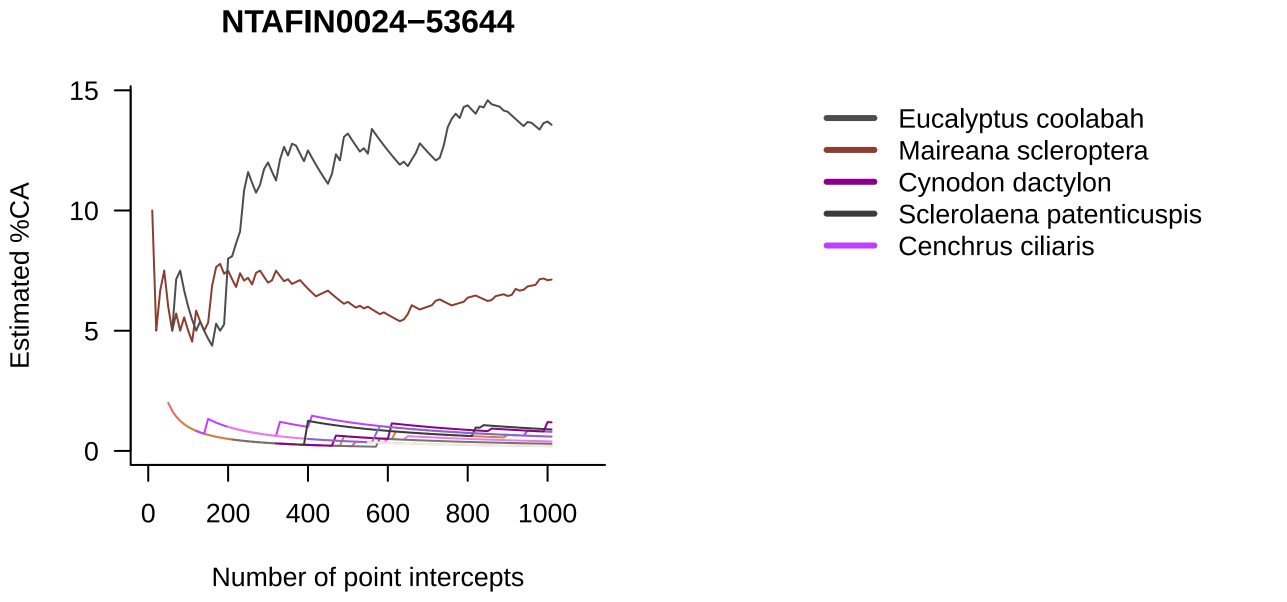
NTAFIN0024−53644
Estimated %CA
0
5
10
15
0
200
400
600
800
1000
Number of point intercepts
Eucalyptus coolabah
Maireana scleroptera
Cynodon dactylon
Sclerolaena patenticuspis
Cenchrus ciliaris

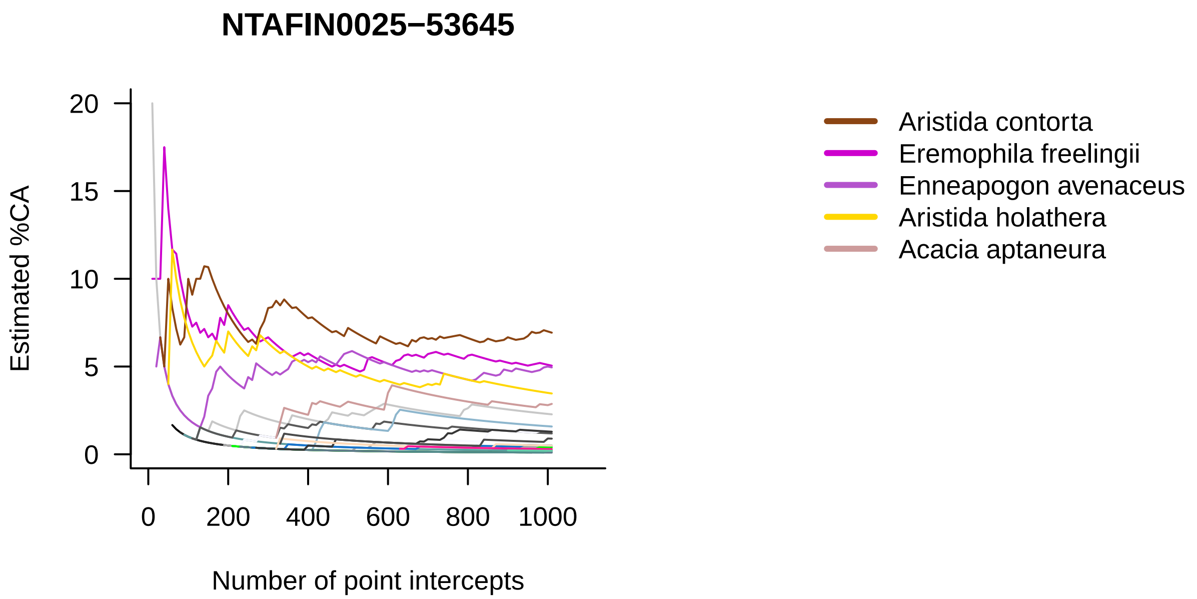

NTAFIN0025−53645
Estimated %CA
Number of point intercepts
0
200
400
600
800
1000
0
5
10
15
20
Aristida contorta
Eremophila freelingii
Enneapogon avenaceus
Aristida holathera
Acacia aptaneura


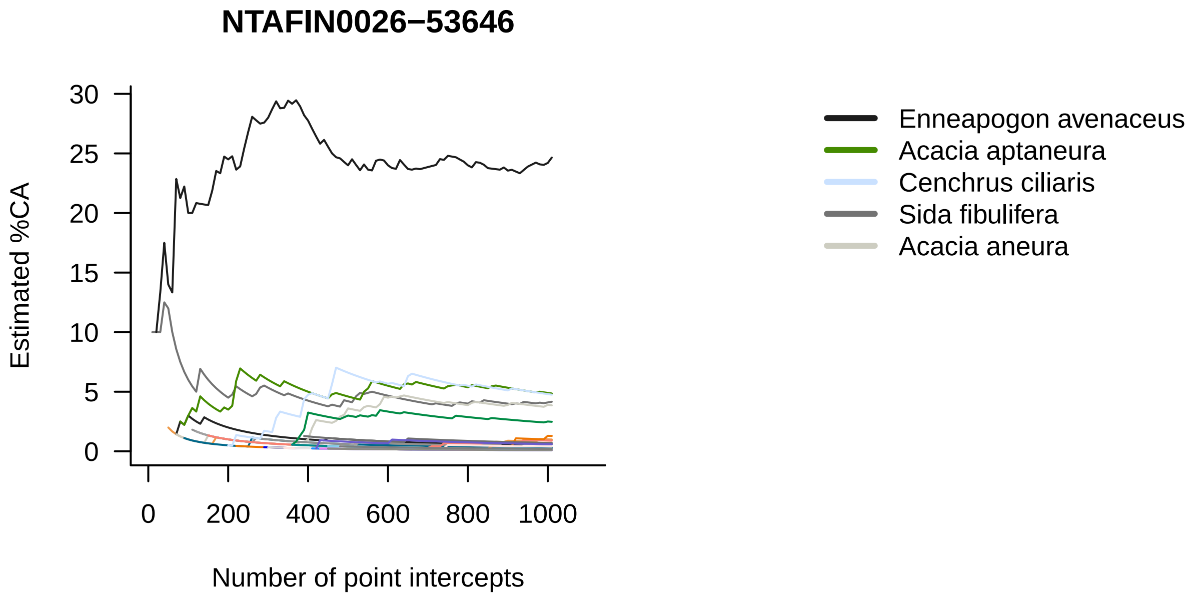

NTAFIN0026−53646
Estimated %CA
Number of point intercepts
0
200
400
600
800
1000
0
5
10
15
20
25
30
Enneapogon avenaceus
Acacia aptaneura
Cenchrus ciliaris
Sida fibulifera
Acacia aneura

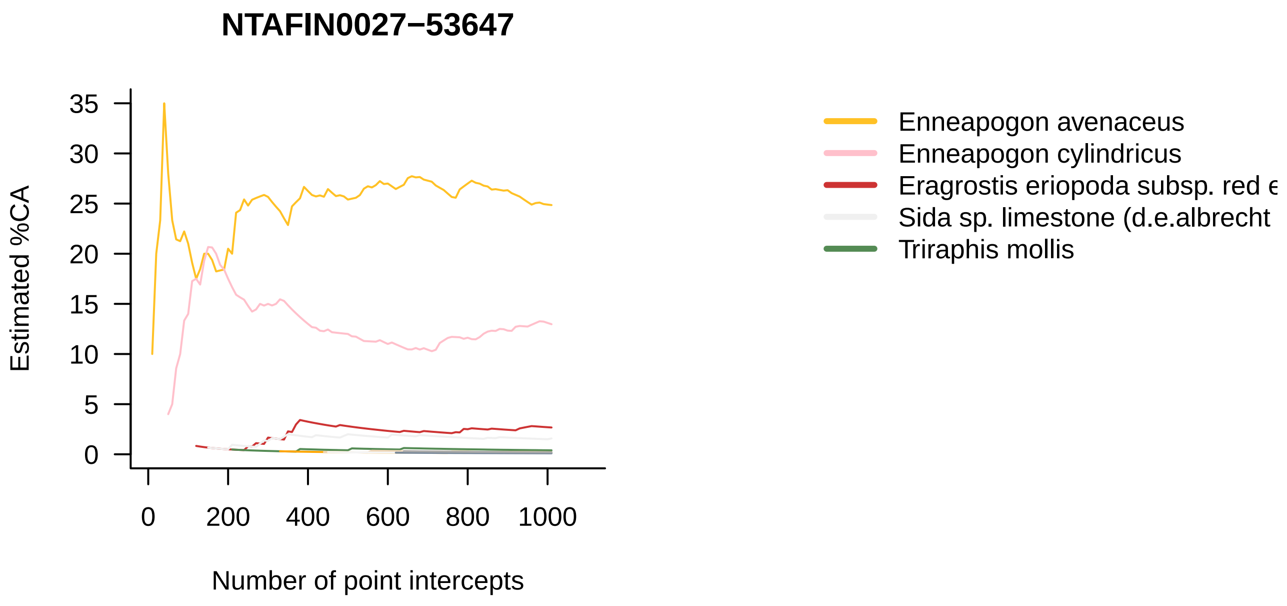
NTAFIN0027−53647
Estimated %CA
35
30
25
20
15
10
5
0
0
200
400
600
800
1000
Number of point intercepts
Enneapogon avenaceus
Enneapogon cylindricus
Eragrostis eriopoda subsp. red e
Sida sp. limestone (d.e.albrecht
Triraphis mollis

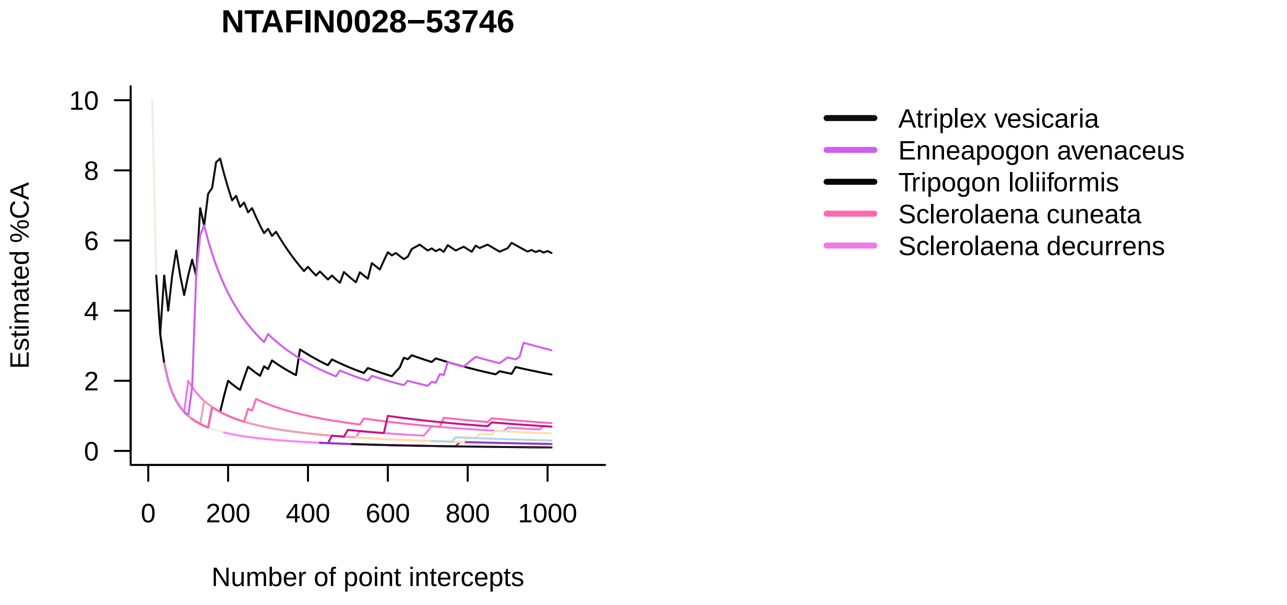
NTAFIN0028−53746
Estimated %CA
10
8
6
4
2
0
0
200
400
600
800
1000
Number of point intercepts
Atriplex vesicaria
Enneapogon avenaceus
Tripogon loliiformis
Sclerolaena cuneata
Sclerolaena decurrens

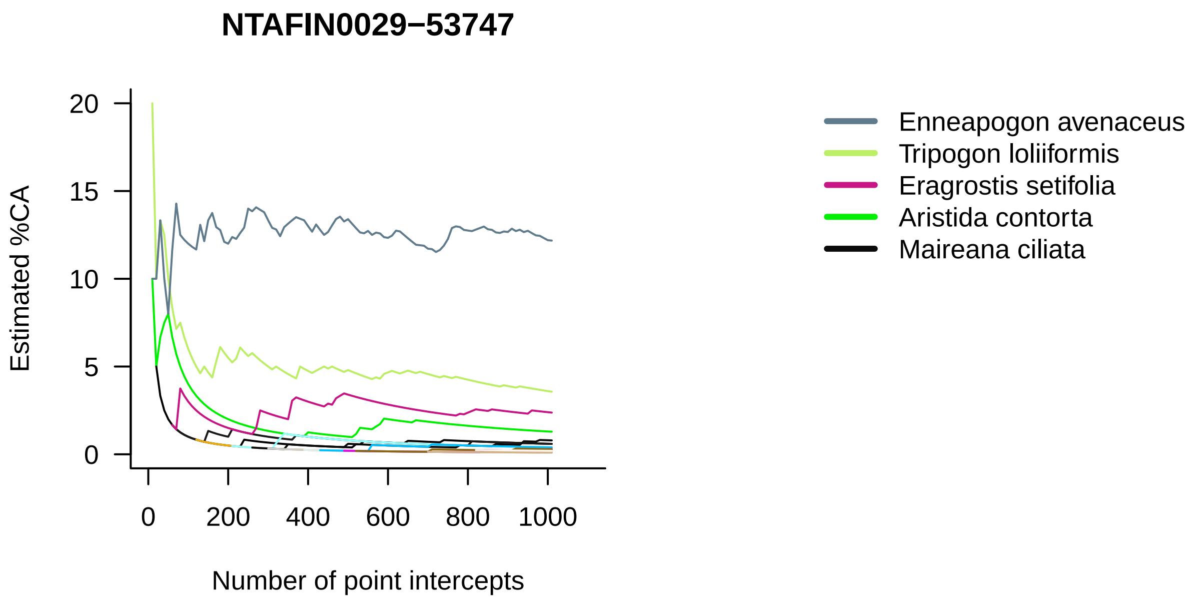
NTAFIN0029−53747
Estimated %CA
0
5
10
15
20
0
200
400
600
800
1000
Number of point intercepts
Enneapogon avenaceus
Tripogon loliiformis
Eragrostis setifolia
Aristida contorta
Maireana ciliata

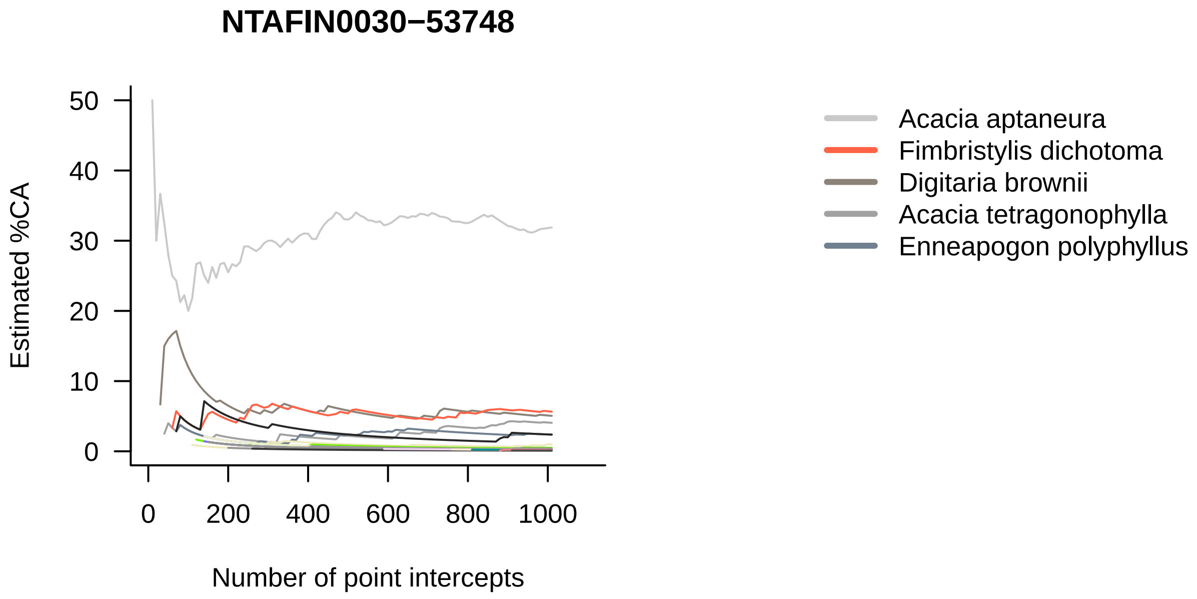
NTAFIN0030−53748
Estimated %CA
0
10
20
30
40
50
0
200
400
600
800
1000
Number of point intercepts
Acacia aptaneura
Fimbristylis dichotoma
Digitaria brownii
Acacia tetragonophylla
Enneapogon polyphyllus

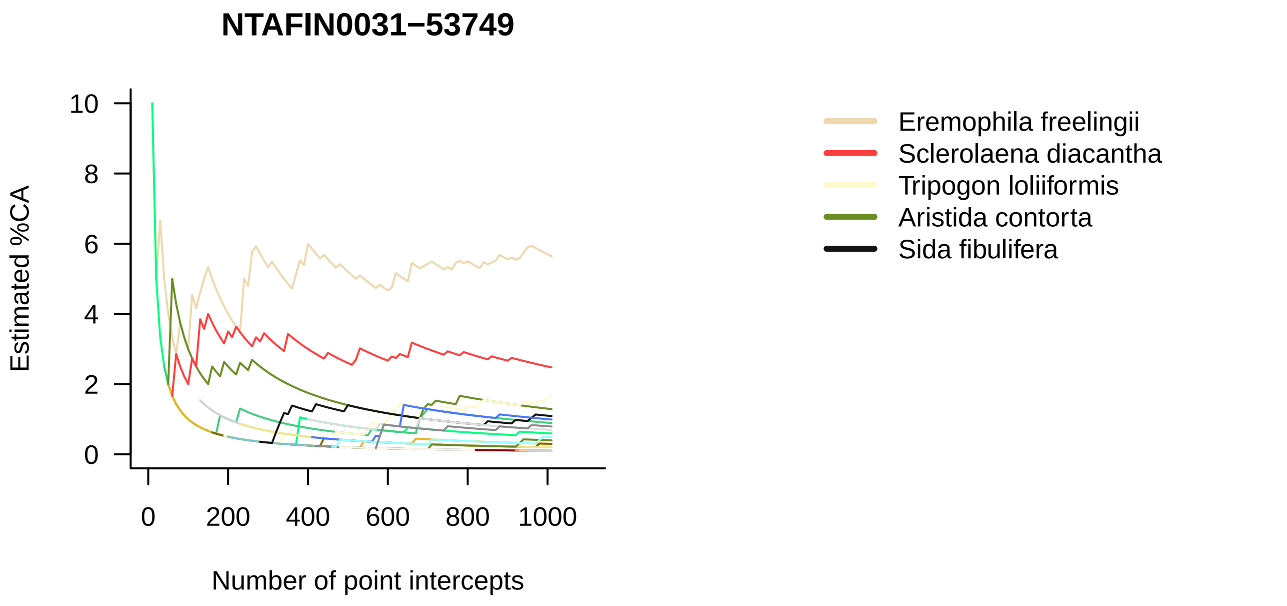
NTAFIN0031−53749
Estimated %CA
Number of point intercepts
0
2
4
6
8
10
0
200
400
600
800
1000
Eremophila freelingii
Sclerolaena diacantha
Tripogon loliiformis
Aristida contorta
Sida fibulifera

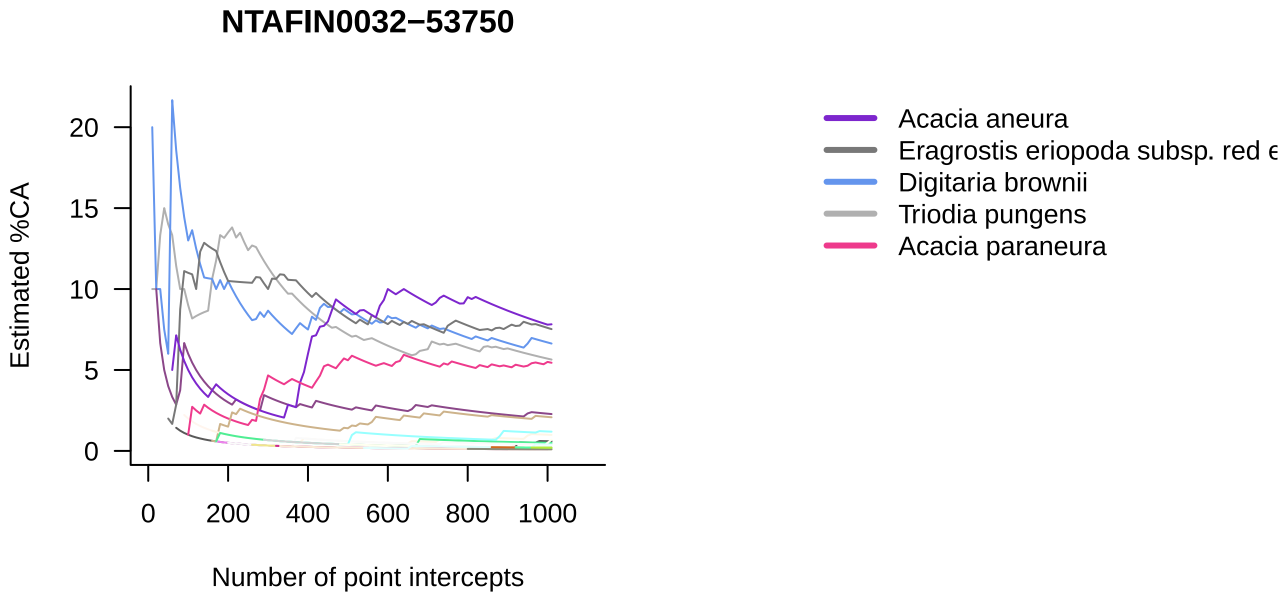
NTAFIN0032−53750
Estimated %CA
Number of point intercepts
0
5
10
15
20
0
200
400
600
800
1000
Acacia aneura
Eragrostis eriopoda subsp. red e
Digitaria brownii
Triodia pungens
Acacia paraneura

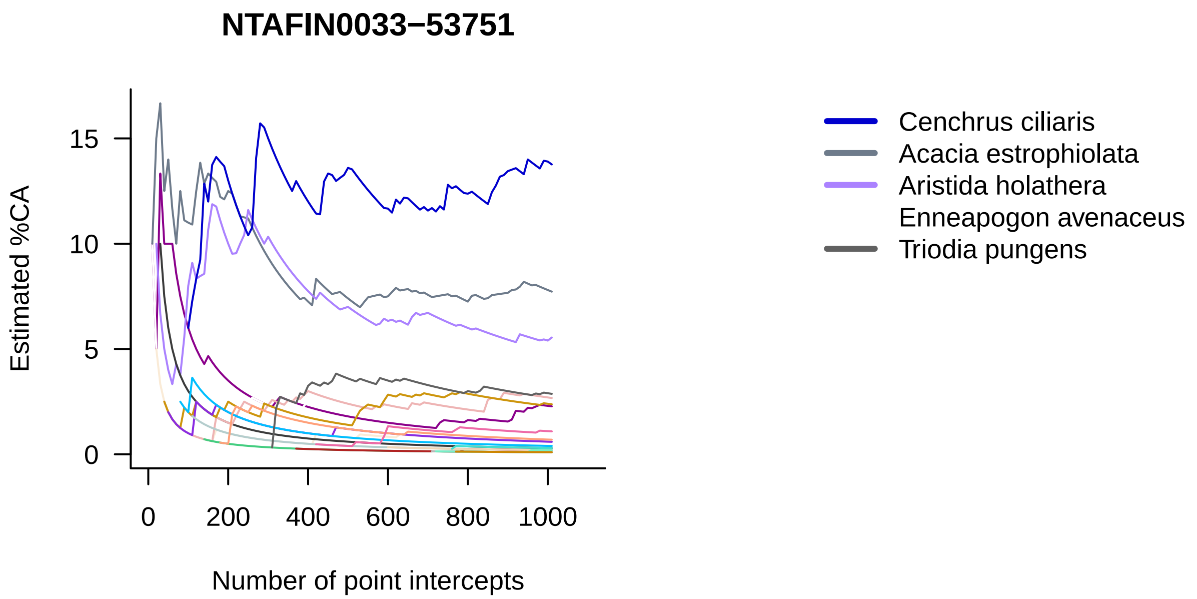
NTAFIN0033−53751
Estimated %CA
0
5
10
15
0
200
400
600
800
1000
Number of point intercepts
Cenchrus ciliaris
Acacia estrophiolata
Aristida holathera
Enneapogon avenaceus
Triodia pungens

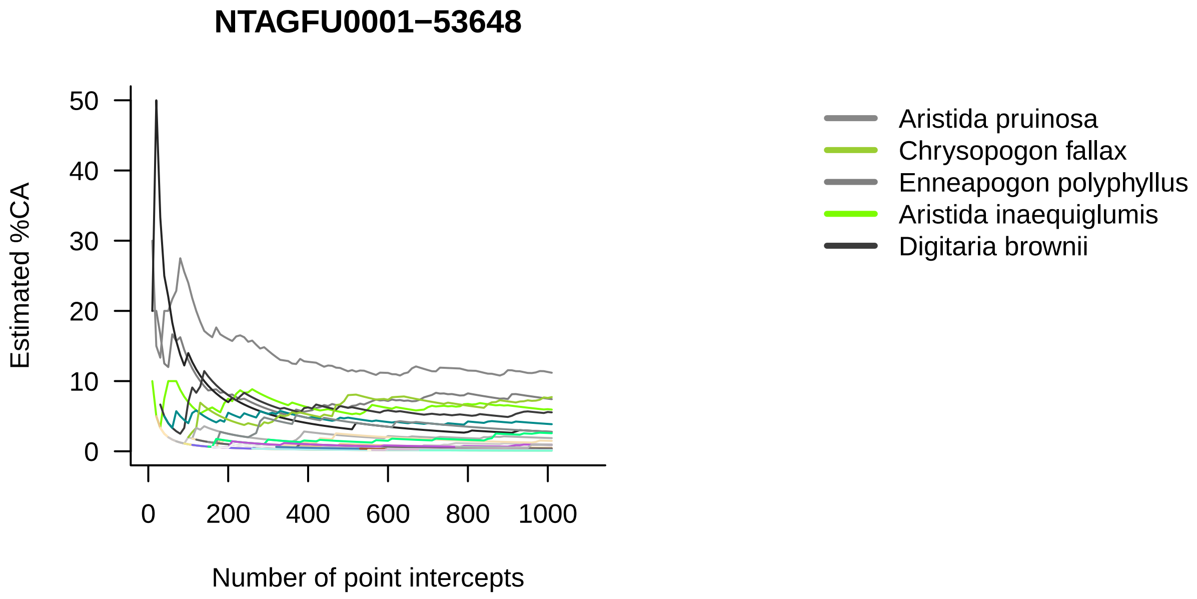
NTAGFU0001−53648
Estimated %CA
0
10
20
30
40
50
0
200
400
600
800
1000
Number of point intercepts
Aristida pruinosa
Chrysopogon fallax
Enneapogon polyphyllus
Aristida inaequiglumis
Digitaria brownii

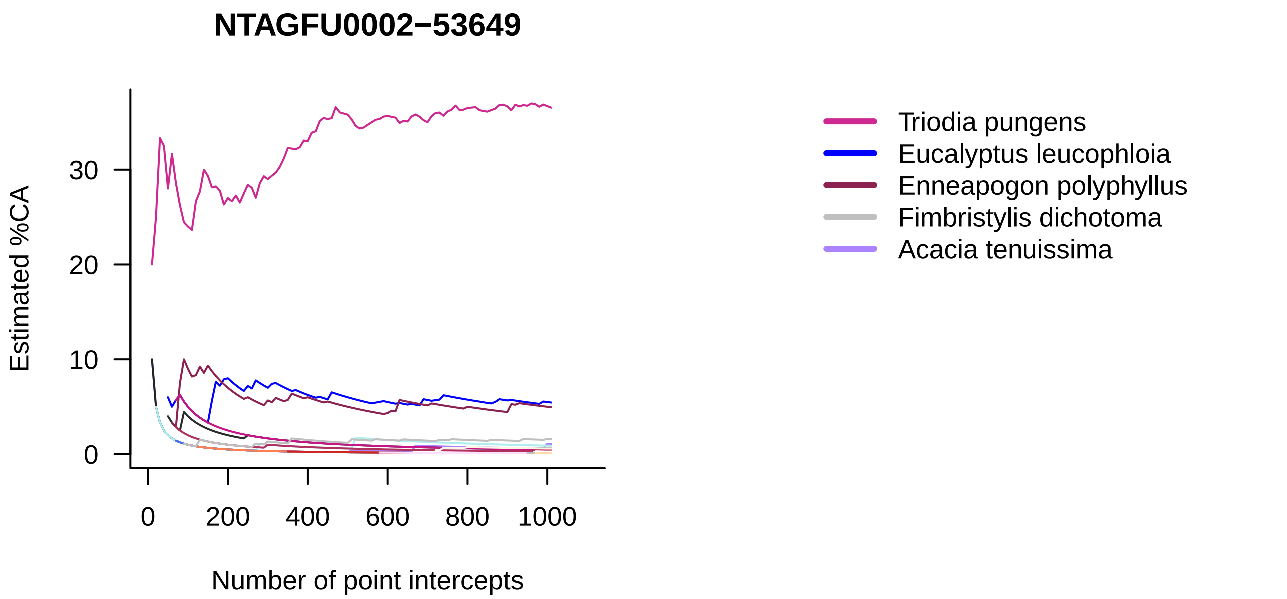
NTAGFU0002−53649
Estimated %CA
0
10
20
30
0
200
400
600
800
1000
Number of point intercepts
Triodia pungens
Eucalyptus leucophloia
Enneapogon polyphyllus
Fimbristylis dichotoma
Acacia tenuissima

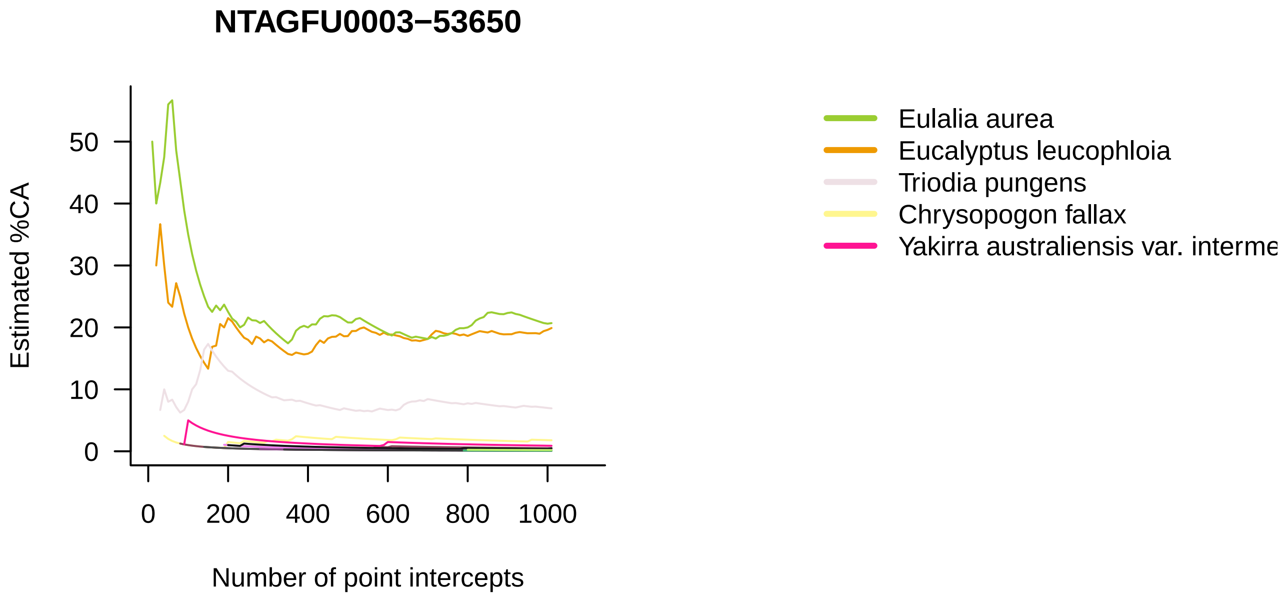
NTAGFU0003−53650
Estimated %CA
0
10
20
30
40
50
0
200
400
600
800
1000
Number of point intercepts
Eulalia aurea
Eucalyptus leucophloia
Triodia pungens
Chrysopogon fallax
Yakirra australiensis var. interme

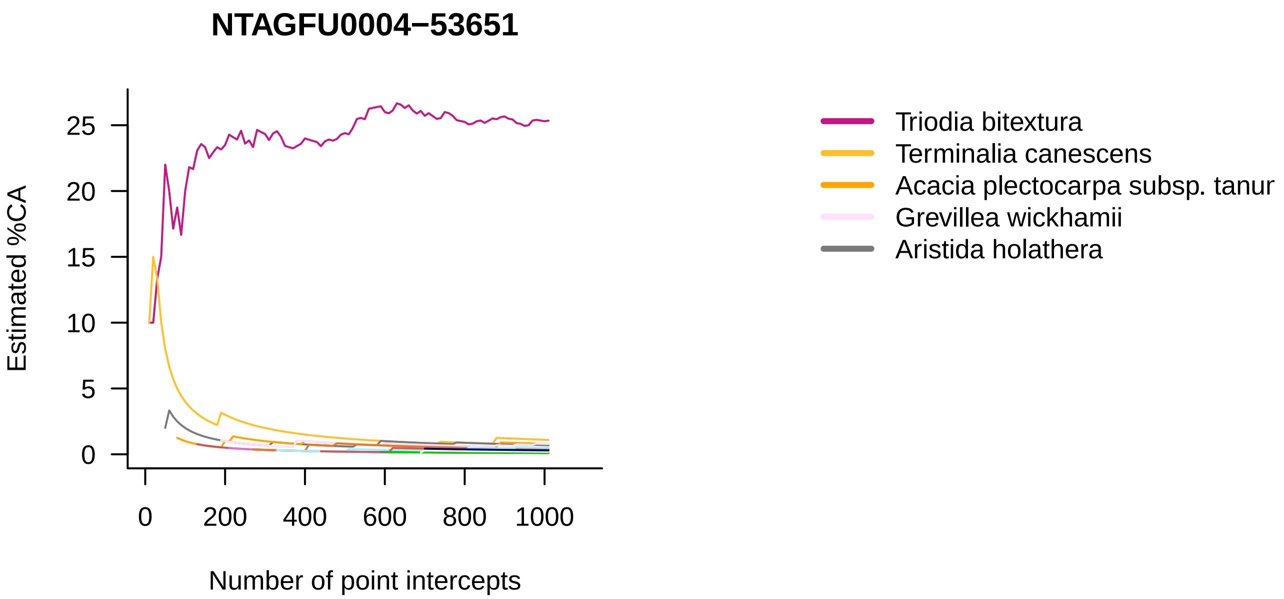
NTAGFU0004−53651
Estimated %CA
Number of point intercepts
0
5
10
15
20
25
0
200
400
600
800
1000
Triodia bitextura
Terminalia canescens
Acacia plectocarpa subsp. tanur
Grevillea wickhamii
Aristida holathera

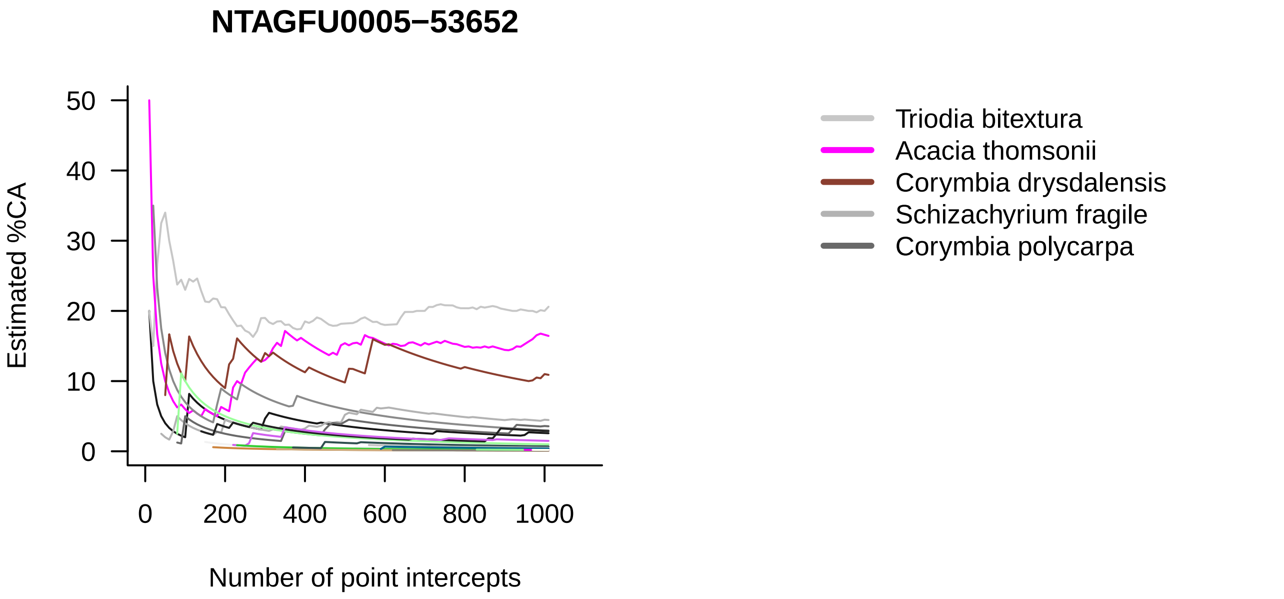
NTAGFU0005−53652
Estimated %CA
Number of point intercepts
0
10
20
30
40
50
0
200
400
600
800
1000
Triodia bitextura
Acacia thomsonii
Corymbia drysdalensis
Schizachyrium fragile
Corymbia polycarpa

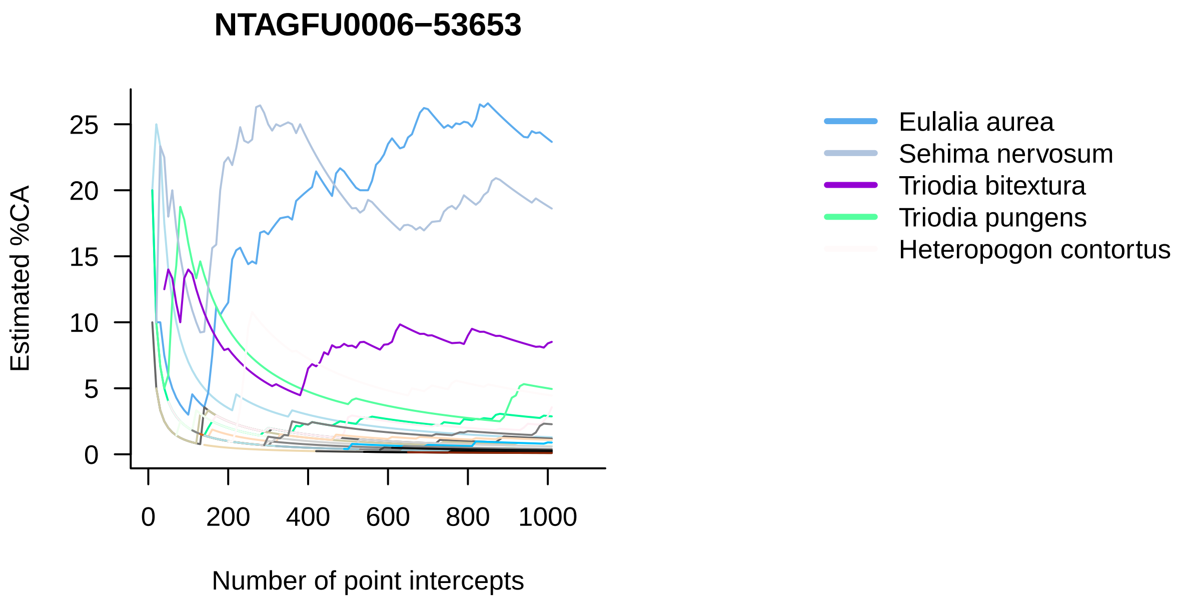
NTAGFU0006−53653
Estimated %CA
0
5
10
15
20
25
0
200
400
600
800
1000
Number of point intercepts
Eulalia aurea
Sehima nervosum
Triodia bitextura
Triodia pungens
Heteropogon contortus

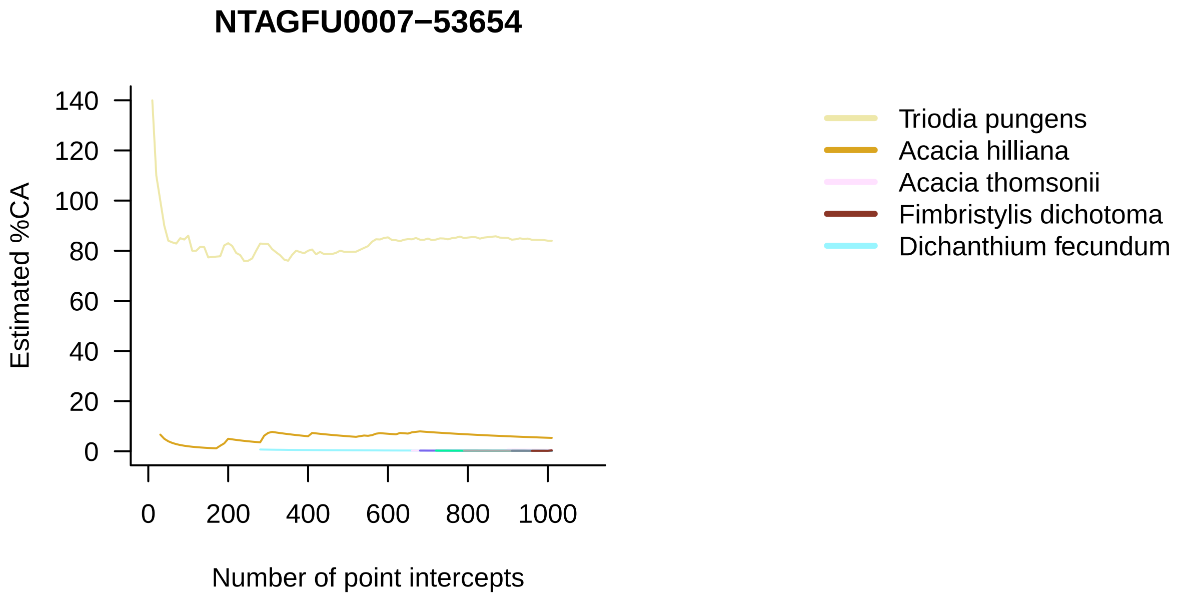
NTAGFU0007−53654
Estimated %CA
0
20
40
60
80
100
120
140
0
200
400
600
800
1000
Number of point intercepts
Triodia pungens
Acacia hilliana
Acacia thomsonii
Fimbristylis dichotoma
Dichanthium fecundum

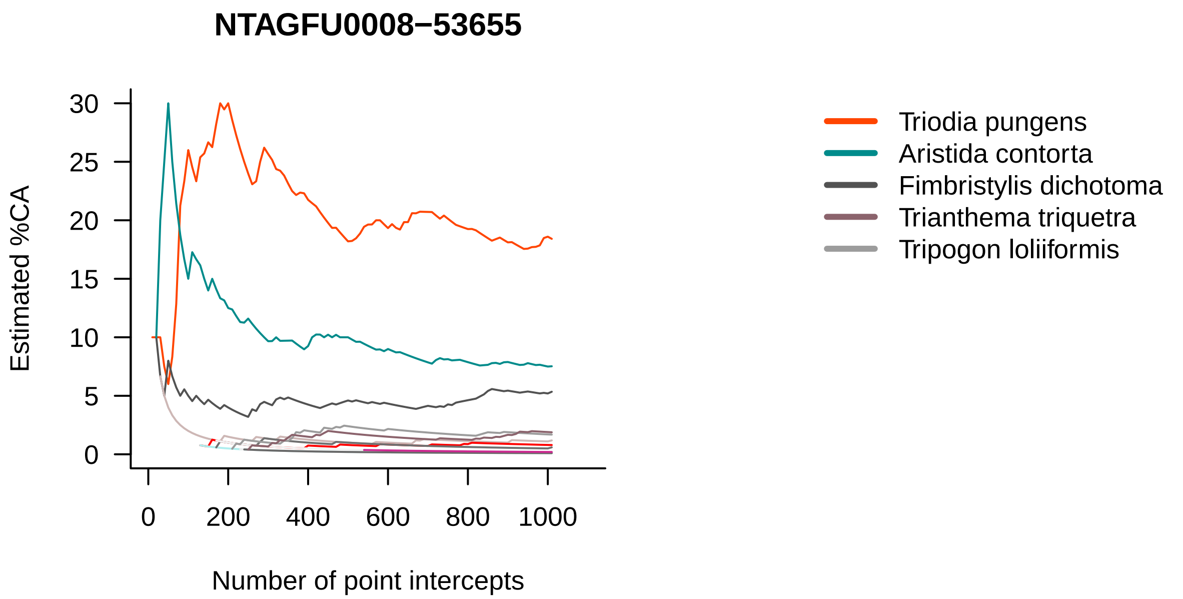

NTAGFU0008−53655
Estimated %CA
Number of point intercepts
0
5
10
15
20
25
30
0
200
400
600
800
1000
Triodia pungens
Aristida contorta
Fimbristylis dichotoma
Trianthema triquetra
Tripogon loliiformis


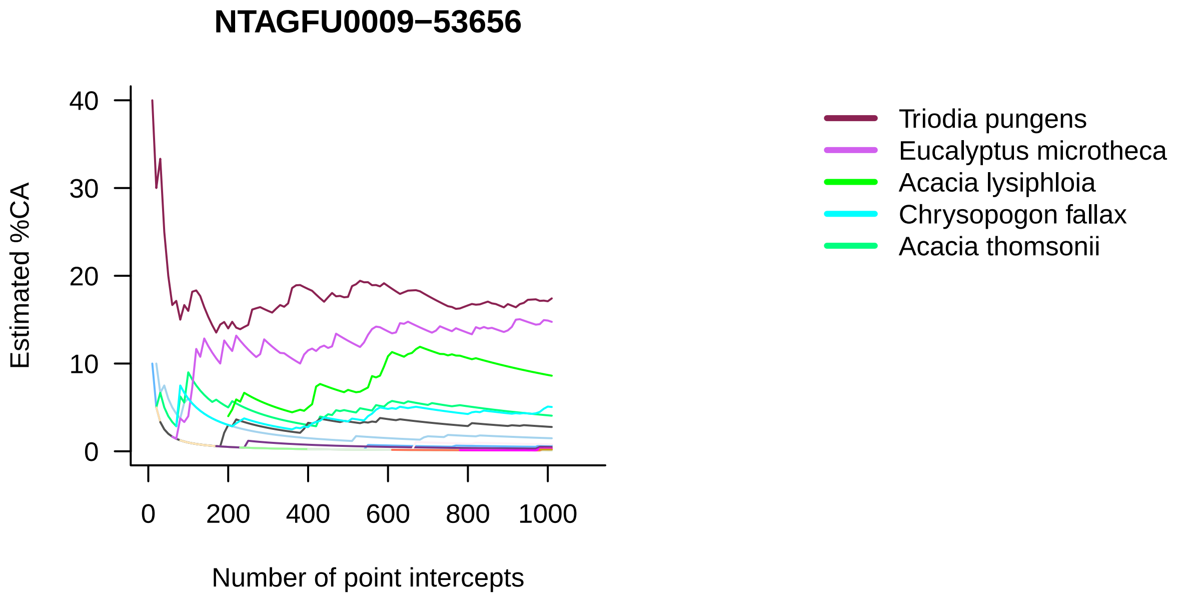

NTAGFU0009−53656
Estimated %CA
Number of point intercepts
0
10
20
30
40
0
200
400
600
800
1000
Triodia pungens
Eucalyptus microtheca
Acacia lysiphloia
Chrysopogon fallax
Acacia thomsonii

## NTAGFU0010−53657

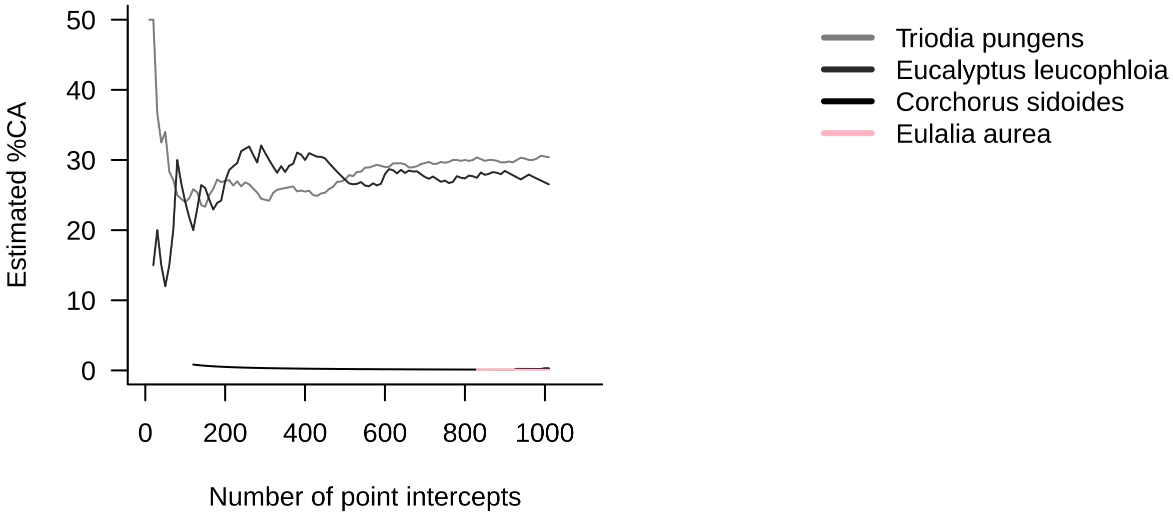


## NTAGFU0011−53658

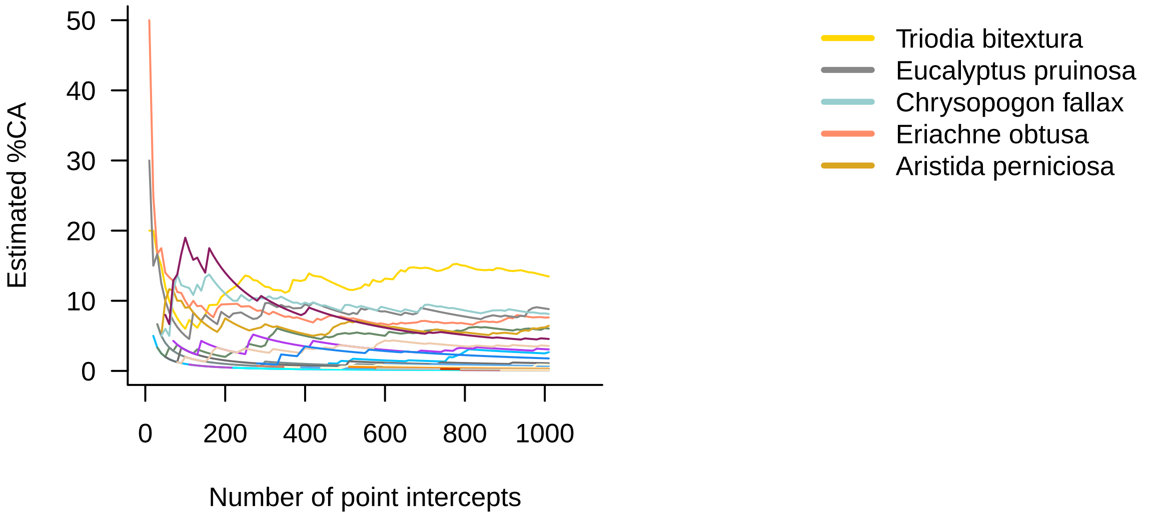

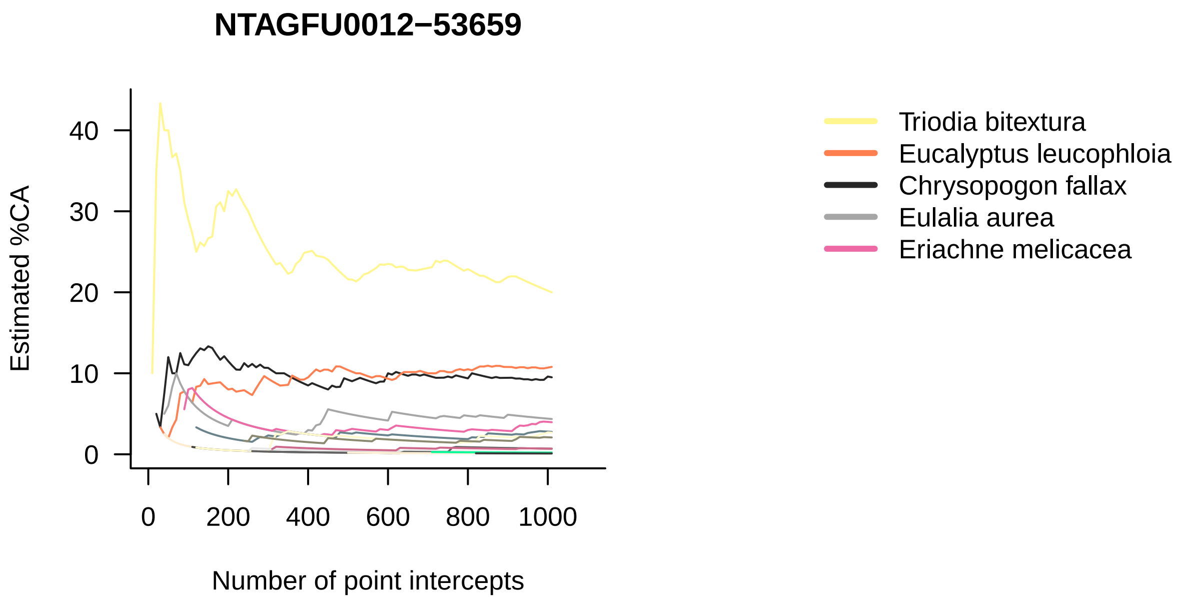
NTAGFU0012−53659
Estimated %CA
0
10
20
30
40
0
200
400
600
800
1000
Number of point intercepts
Triodia bitextura
Eucalyptus leucophloia
Chrysopogon fallax
Eulalia aurea
Eriachne melicacea

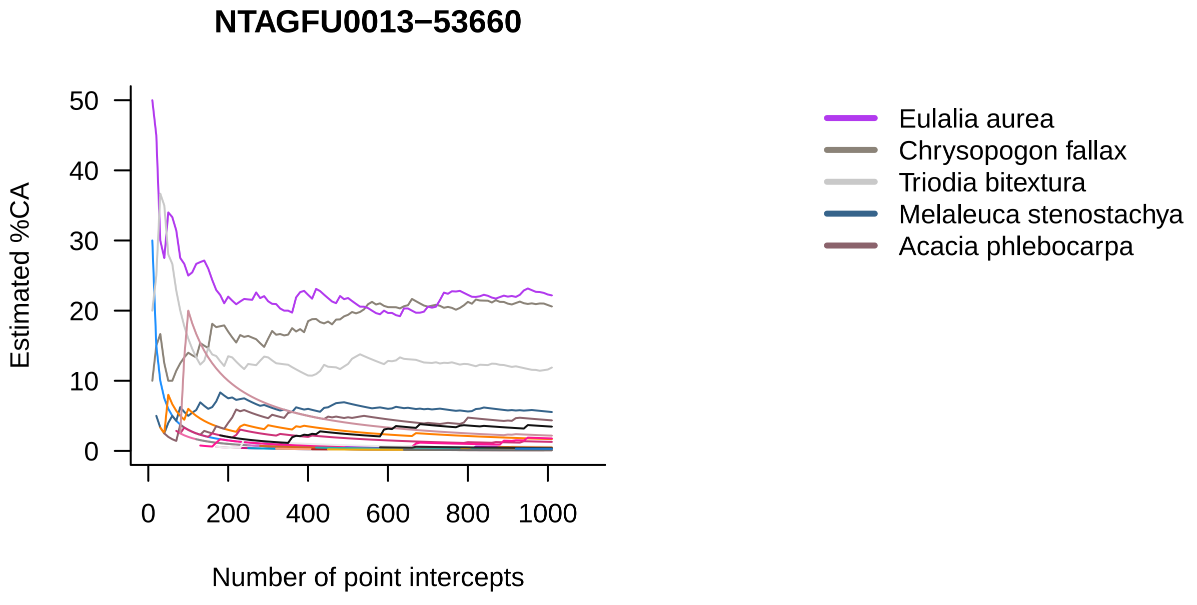
NTAGFU0013−53660
Estimated %CA
0
10
20
30
40
50
0
200
400
600
800
1000
Number of point intercepts
Eulalia aurea
Chrysopogon fallax
Triodia bitextura
Melaleuca stenostachya
Acacia phlebocarpa

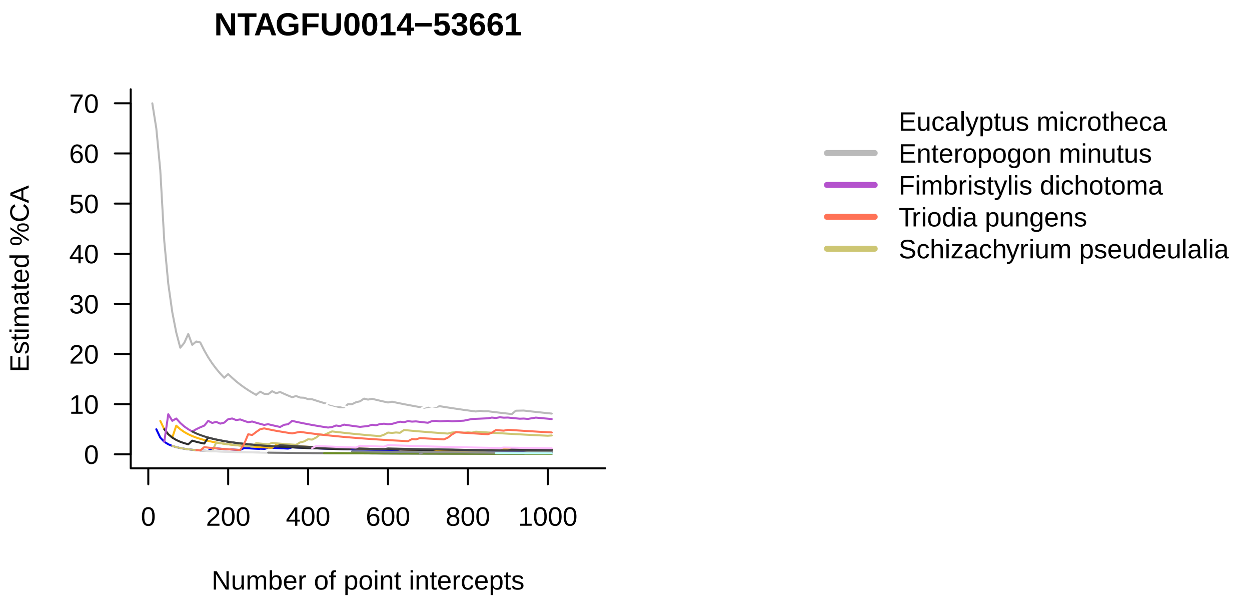
NTAGFU0014−53661
Estimated %CA
70
60
50
40
30
20
10
0
0
200
400
600
800
1000
Number of point intercepts
Eucalyptus microtheca
Enteropogon minutus
Fimbristylis dichotoma
Triodia pungens
Schizachyrium pseudeulalia

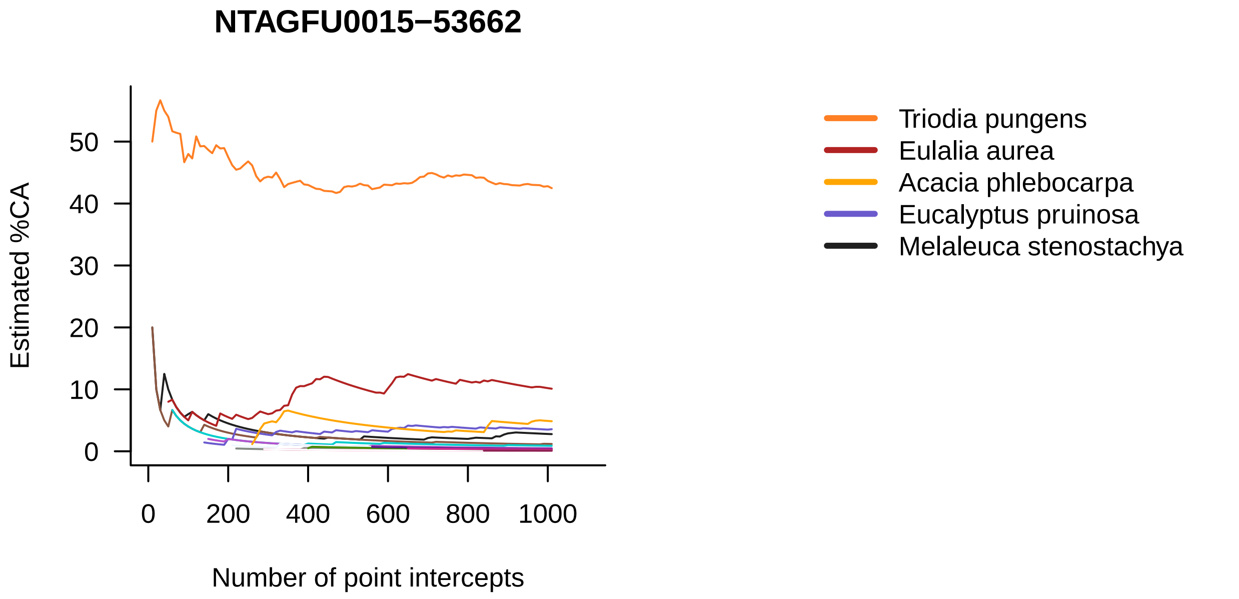
NTAGFU0015−53662
Estimated %CA
50
40
30
20
10
0
0
200
400
600
800
1000
Number of point intercepts
Triodia pungens
Eulalia aurea
Acacia phlebocarpa
Eucalyptus pruinosa
Melaleuca stenostachya

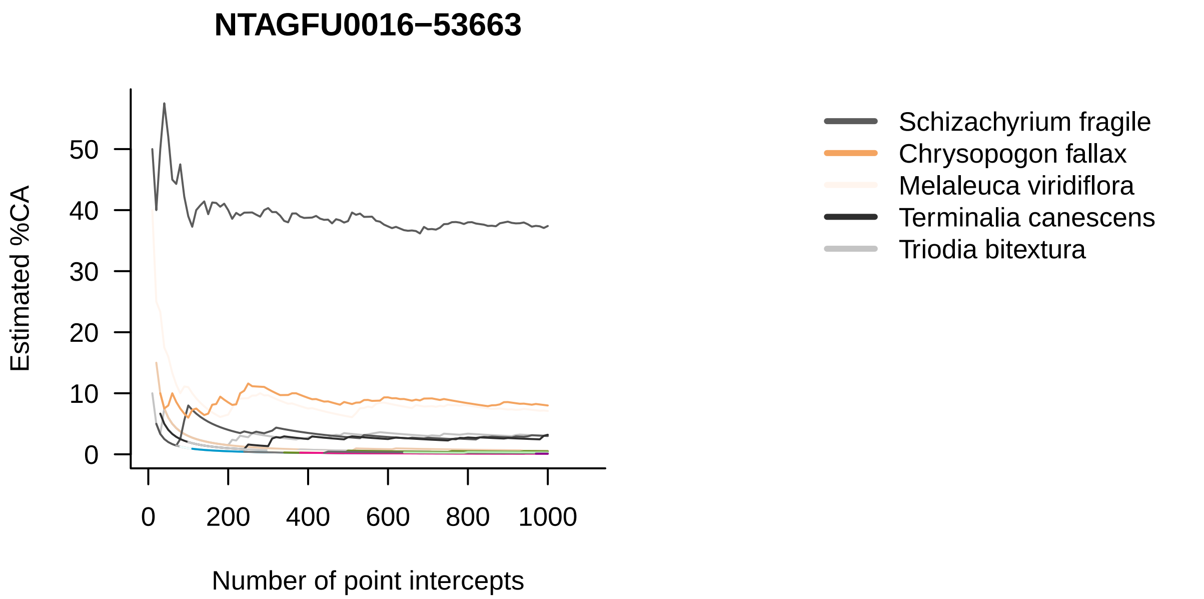
NTAGFU0016−53663
Estimated %CA
0
10
20
30
40
50
0
200
400
600
800
1000
Number of point intercepts
Schizachyrium fragile
Chrysopogon fallax
Melaleuca viridiflora
Terminalia canescens
Triodia bitextura

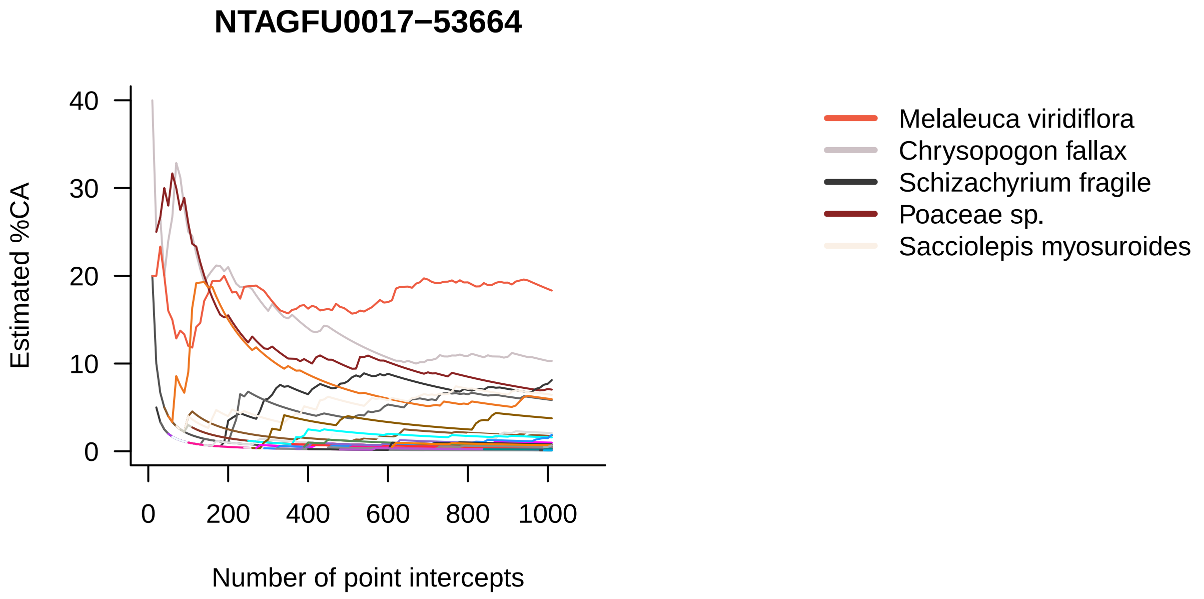
NTAGFU0017−53664
Estimated %CA
0
10
20
30
40
0
200
400
600
800
1000
Number of point intercepts
Melaleuca viridiflora
Chrysopogon fallax
Schizachyrium fragile
Poaceae sp.
Sacciolepis myosuroides

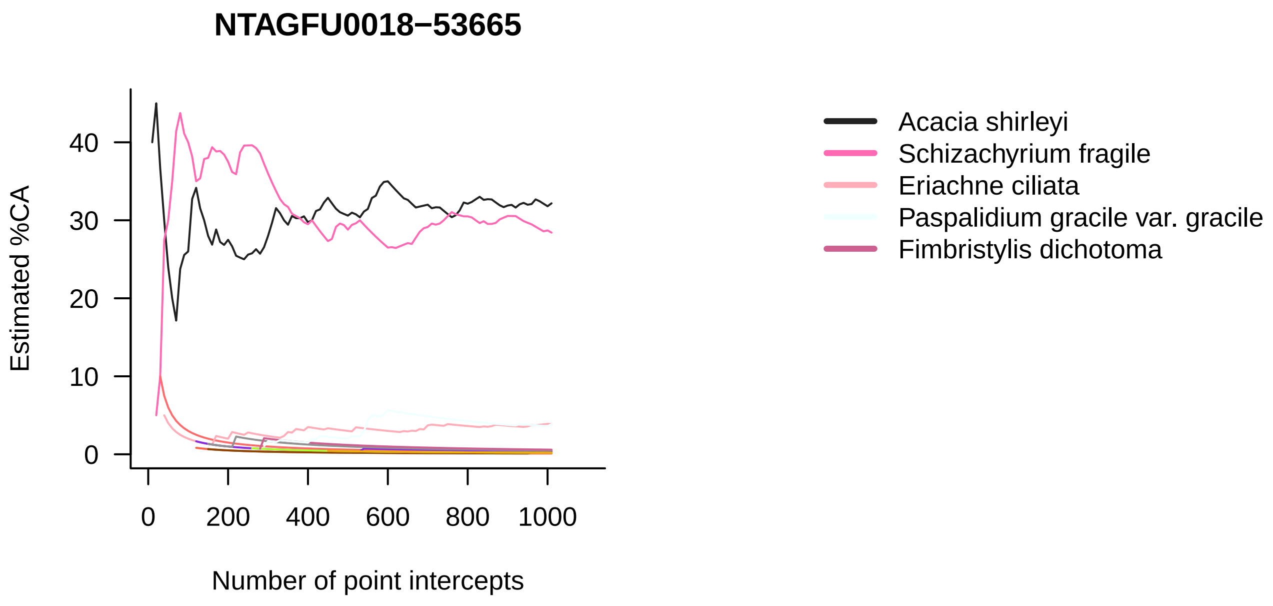
NTAGFU0018−53665
Estimated %CA
Number of point intercepts
0
10
20
30
40
0
200
400
600
800
1000
Acacia shirleyi
Schizachyrium fragile
Eriachne ciliata
Paspalidium gracile var. gracile
Fimbristylis dichotoma

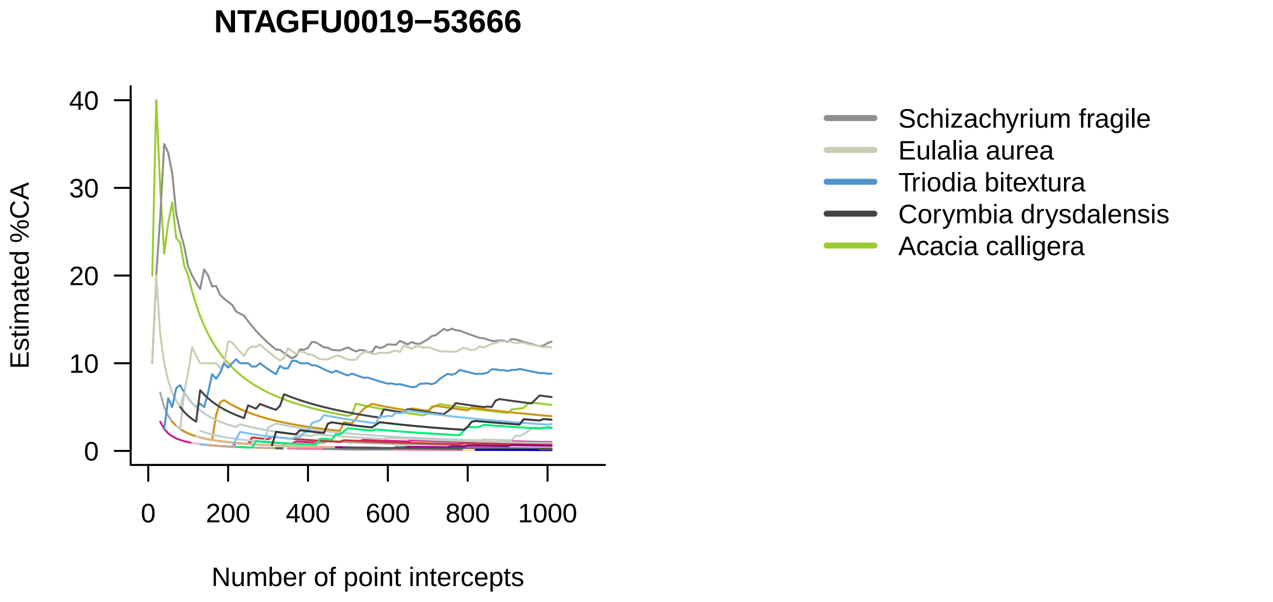
NTAGFU0019−53666
Estimated %CA
Number of point intercepts
0
10
20
30
40
0
200
400
600
800
1000
Schizachyrium fragile
Eulalia aurea
Triodia bitextura
Corymbia drysdalensis
Acacia calligera

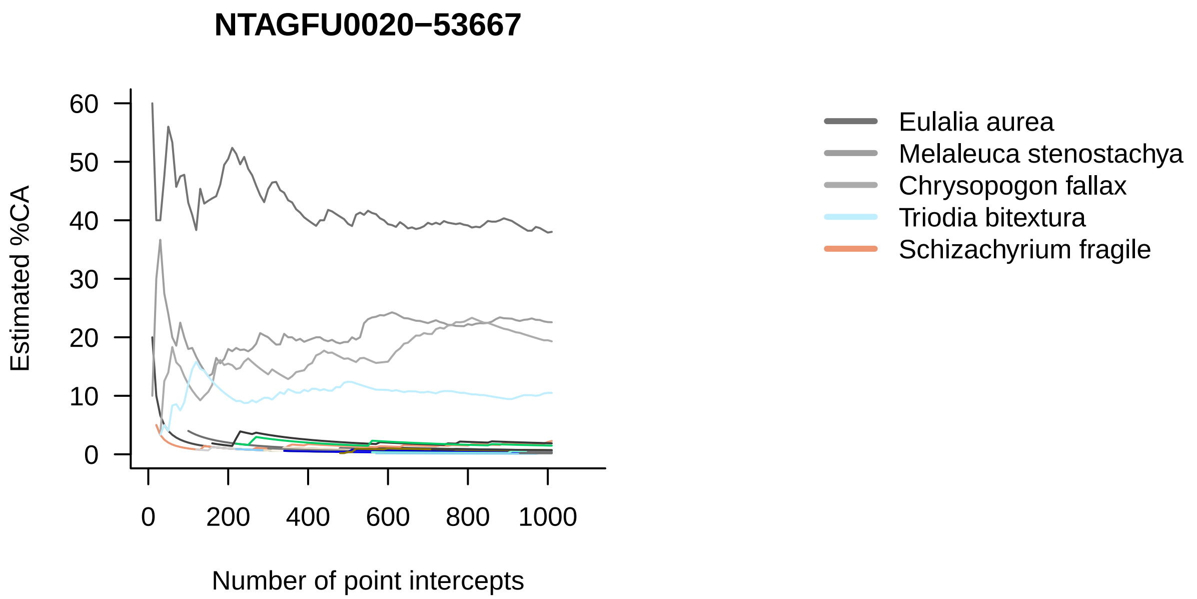
NTAGFU0020−53667
Estimated %CA
60
50
40
30
20
10
0
0
200
400
600
800
1000
Number of point intercepts
Eulalia aurea
Melaleuca stenostachya
Chrysopogon fallax
Triodia bitextura
Schizachyrium fragile

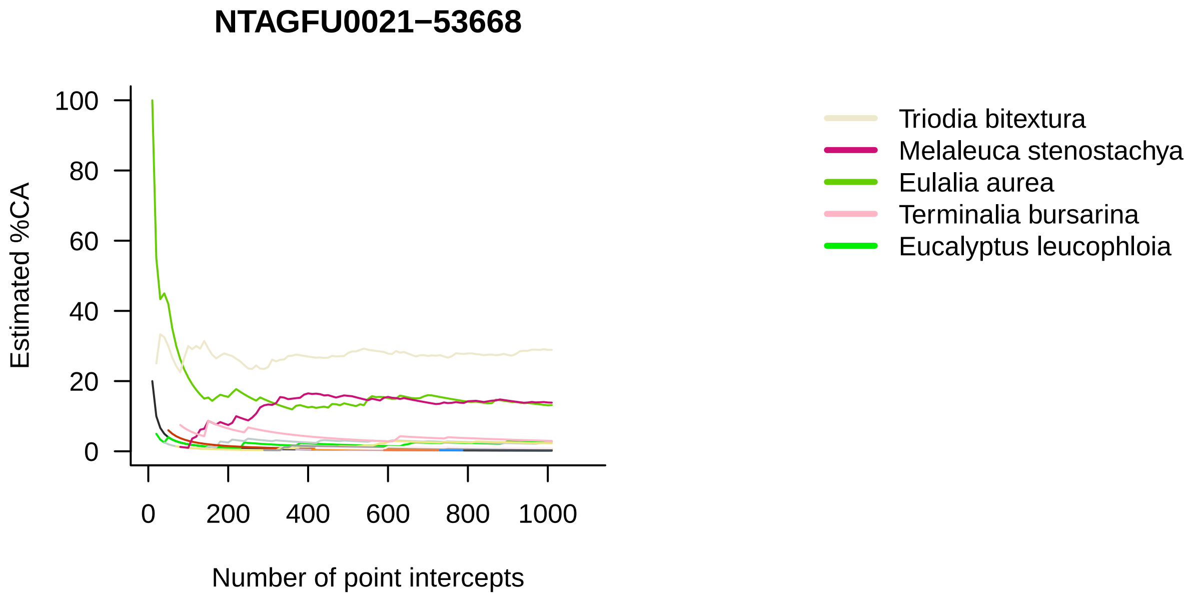
NTAGFU0021−53668
Estimated %CA
100
80
60
40
20
0
0
200
400
600
800
1000
Number of point intercepts
Triodia bitextura
Melaleuca stenostachya
Eulalia aurea
Terminalia bursarina
Eucalyptus leucophloia

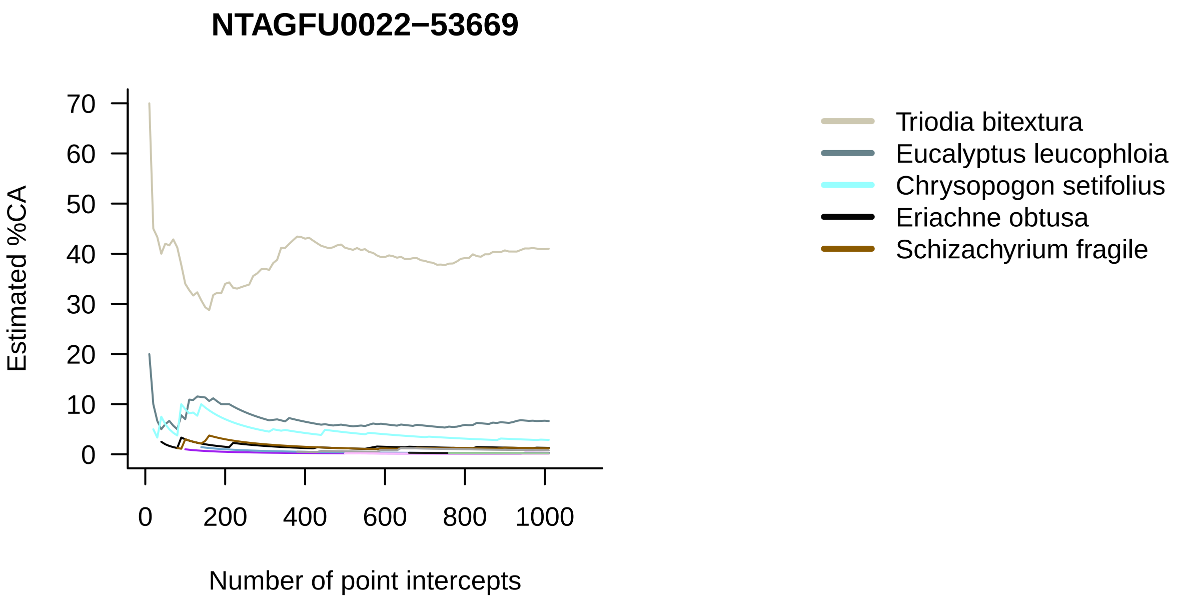

NTAGFU0022−53669
Estimated %CA
70
60
50
40
30
20
10
0
0
200
400
600
800
1000
Number of point intercepts
Triodia bitextura
Eucalyptus leucophloia
Chrysopogon setifolius
Eriachne obtusa
Schizachyrium fragile


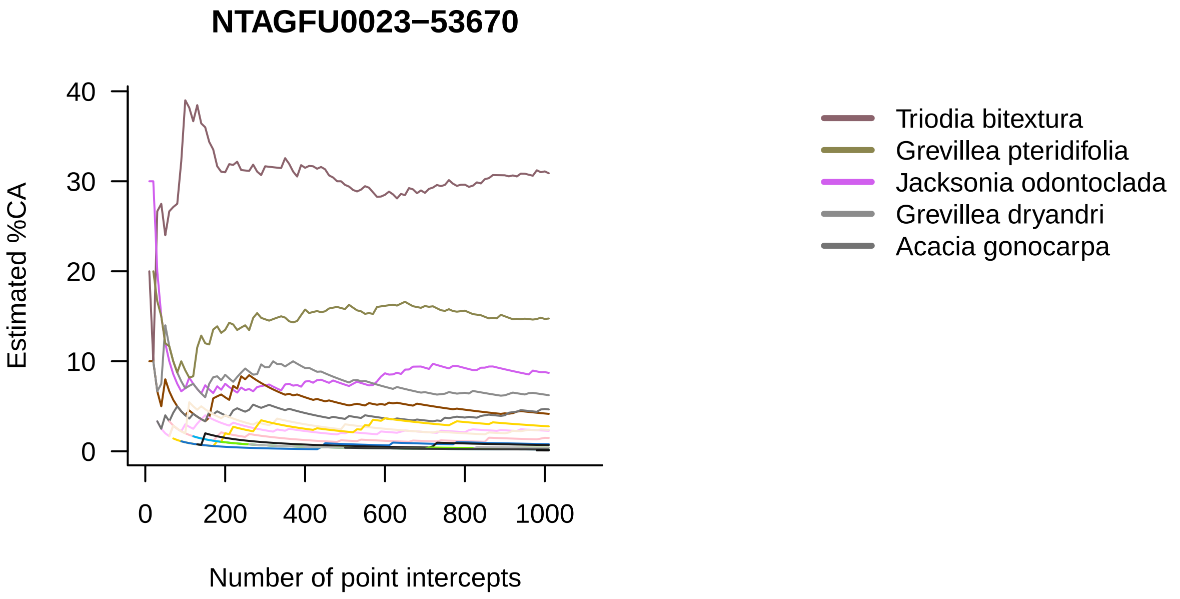

NTAGFU0023−53670
Estimated %CA
40
30
20
10
0
0
200
400
600
800
1000
Number of point intercepts
Triodia bitextura
Grevillea pteridifolia
Jacksonia odontoclada
Grevillea dryandri
Acacia gonocarpa

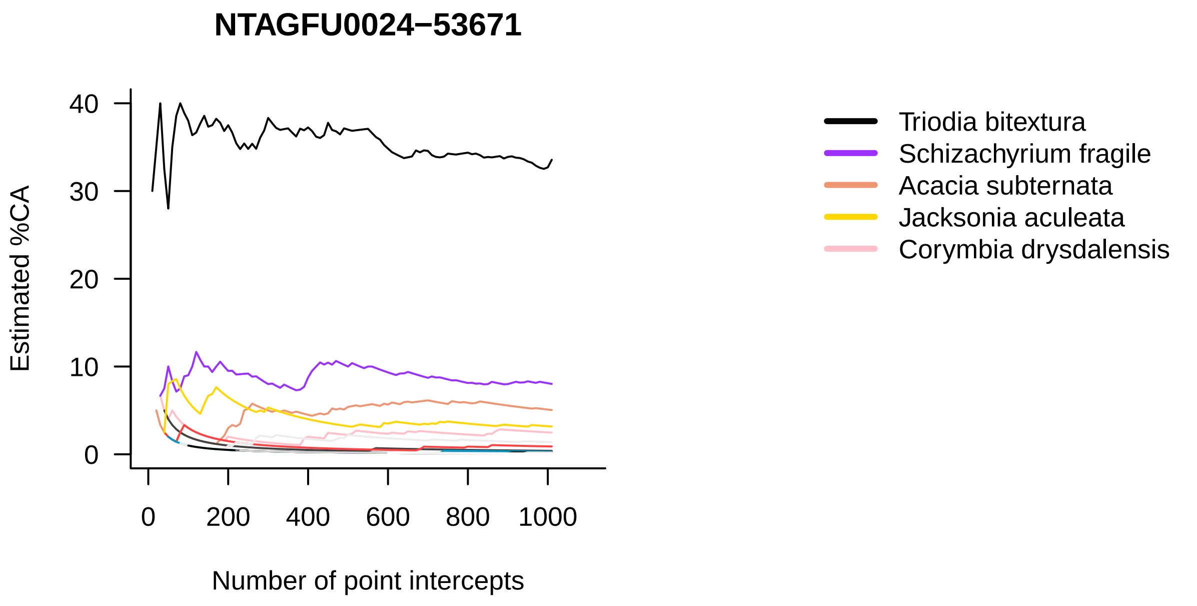
NTAGFU0024−53671
Estimated %CA
40
30
20
10
0
0
200
400
600
800
1000
Number of point intercepts
Triodia bitextura
Schizachyrium fragile
Acacia subternata
Jacksonia aculeata
Corymbia drysdalensis

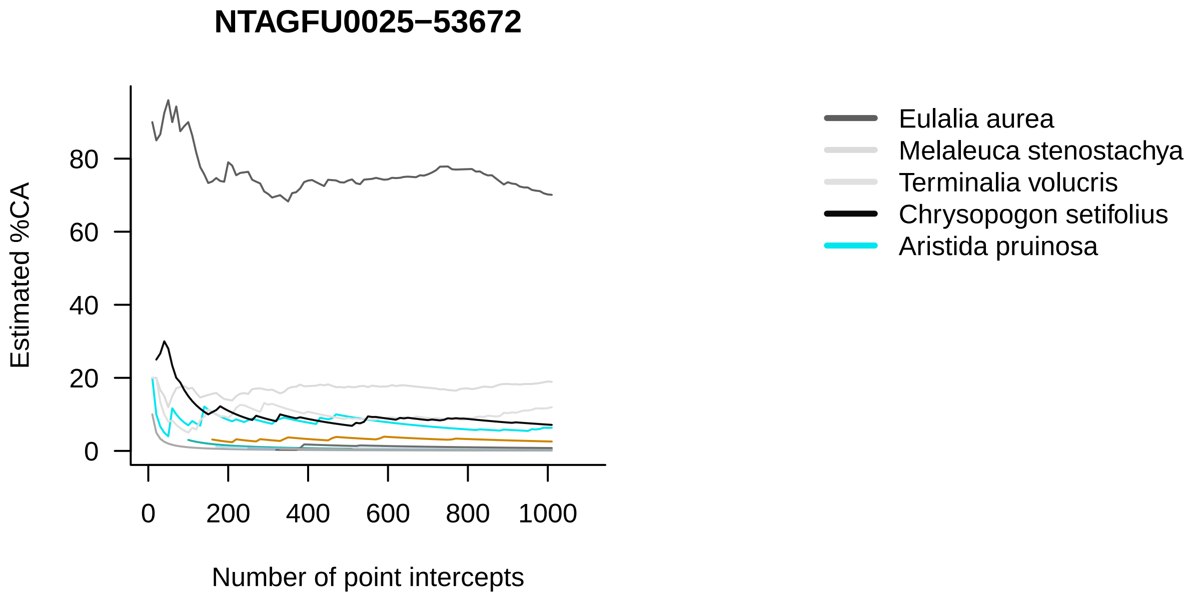
NTAGFU0025−53672
Estimated %CA
80
60
40
20
0
0
200
400
600
800
1000
Number of point intercepts
Eulalia aurea
Melaleuca stenostachya
Terminalia volucris
Chrysopogon setifolius
Aristida pruinosa

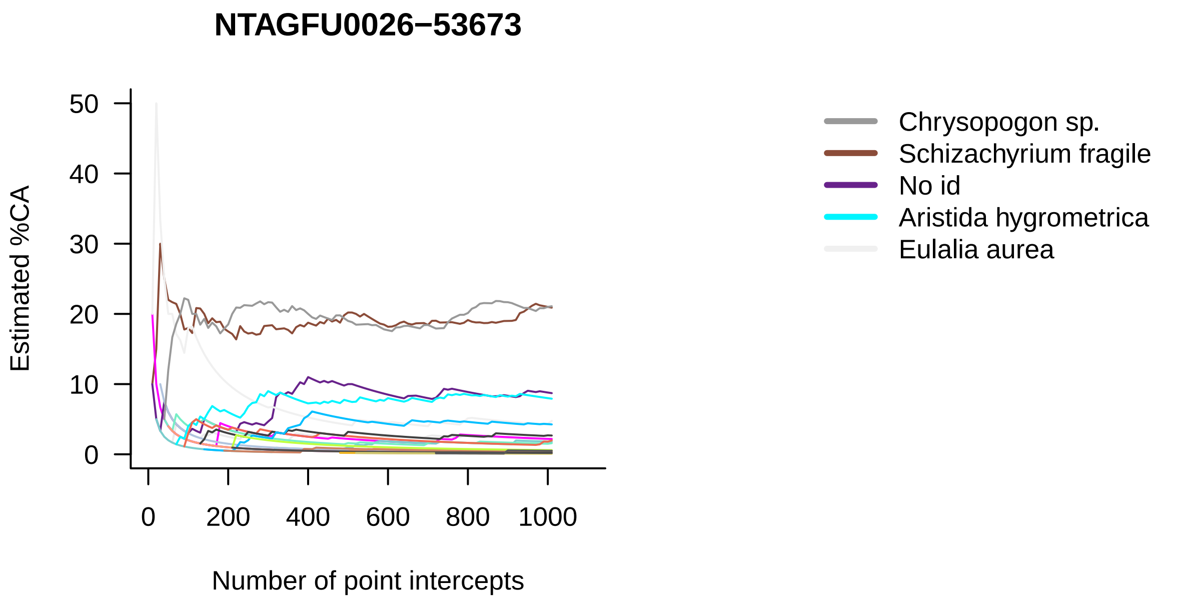
NTAGFU0026−53673
Estimated %CA
Number of point intercepts
0
10
20
30
40
50
0
200
400
600
800
1000
Chrysopogon sp.
Schizachyrium fragile
No id
Aristida hygrometrica
Eulalia aurea

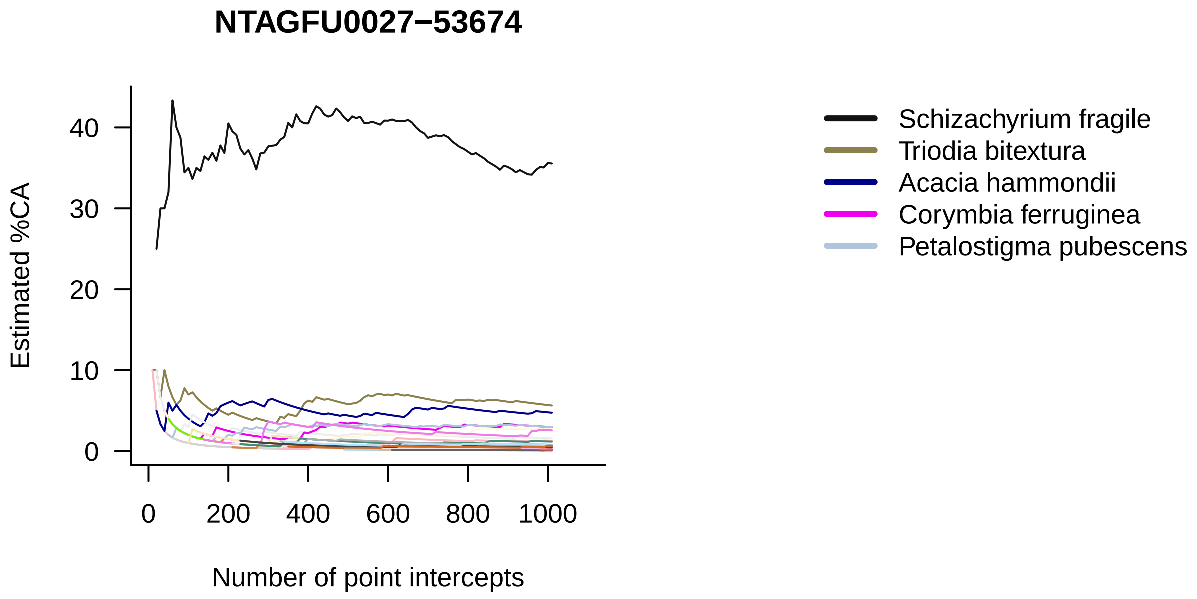
NTAGFU0027−53674
Estimated %CA
Number of point intercepts
0
10
20
30
40
0
200
400
600
800
1000
Schizachyrium fragile
Triodia bitextura
Acacia hammondii
Corymbia ferruginea
Petalostigma pubescens

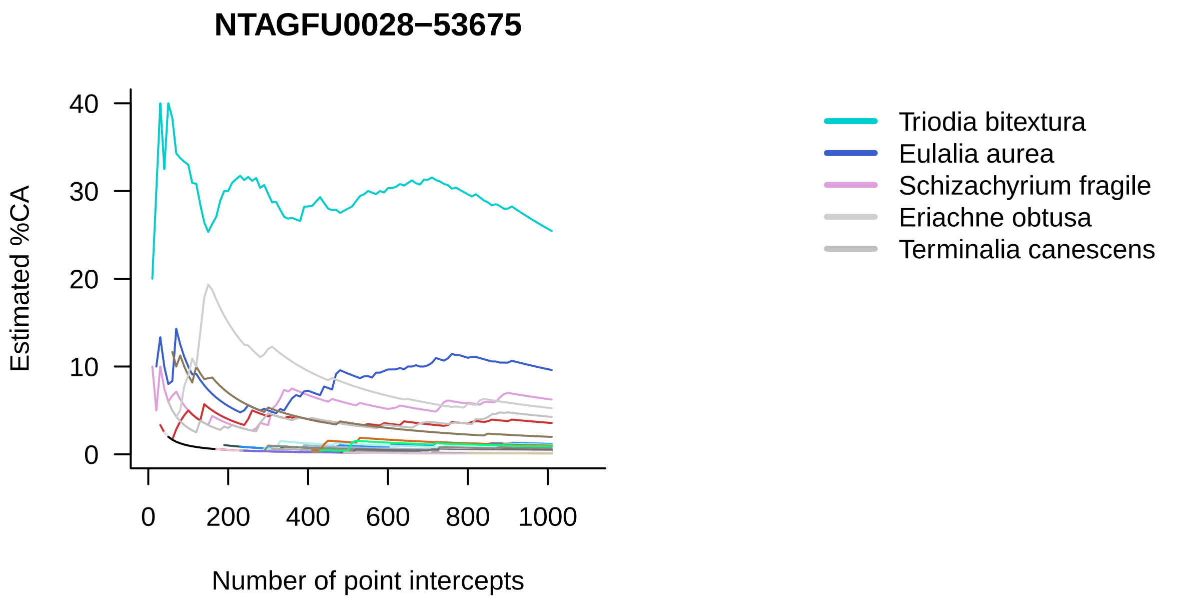
NTAGFU0028−53675
Estimated %CA
40
30
20
10
0
0
200
400
600
800
1000
Number of point intercepts
Triodia bitextura
Eulalia aurea
Schizachyrium fragile
Eriachne obtusa
Terminalia canescens

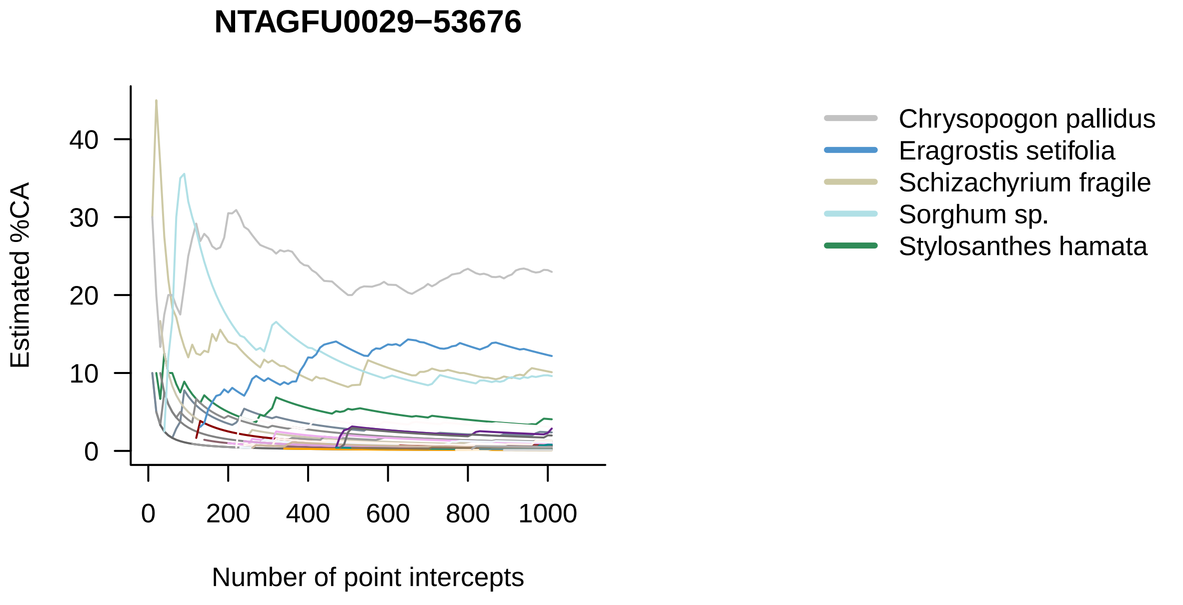
NTAGFU0029−53676
Estimated %CA
40
30
20
10
0
0
200
400
600
800
1000
Number of point intercepts
Chrysopogon pallidus
Eragrostis setifolia
Schizachyrium fragile
Sorghum sp.
Stylosanthes hamata

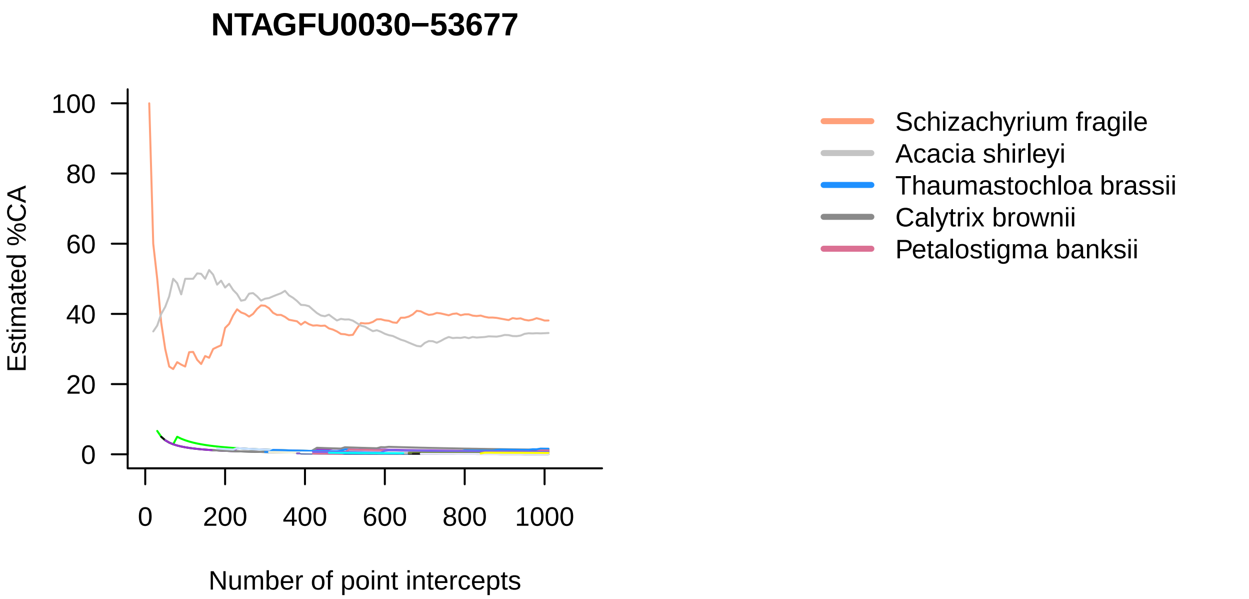
NTAGFU0030−53677
Estimated %CA
Number of point intercepts
0
20
40
60
80
100
0
200
400
600
800
1000
Schizachyrium fragile
Acacia shirleyi
Thaumastochloa brassii
Calytrix brownii
Petalostigma banksii

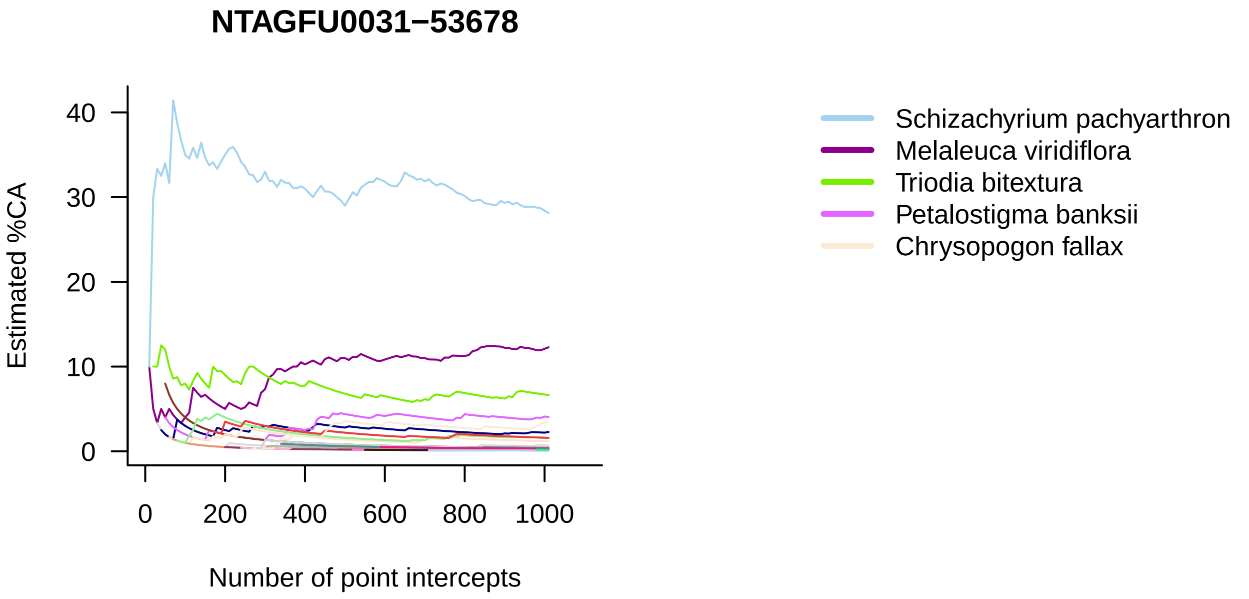
NTAGFU0031−53678
Estimated %CA
Number of point intercepts
0
10
20
30
40
0
200
400
600
800
1000
Schizachyrium pachyarthron
Melaleuca viridiflora
Triodia bitextura
Petalostigma banksii
Chrysopogon fallax

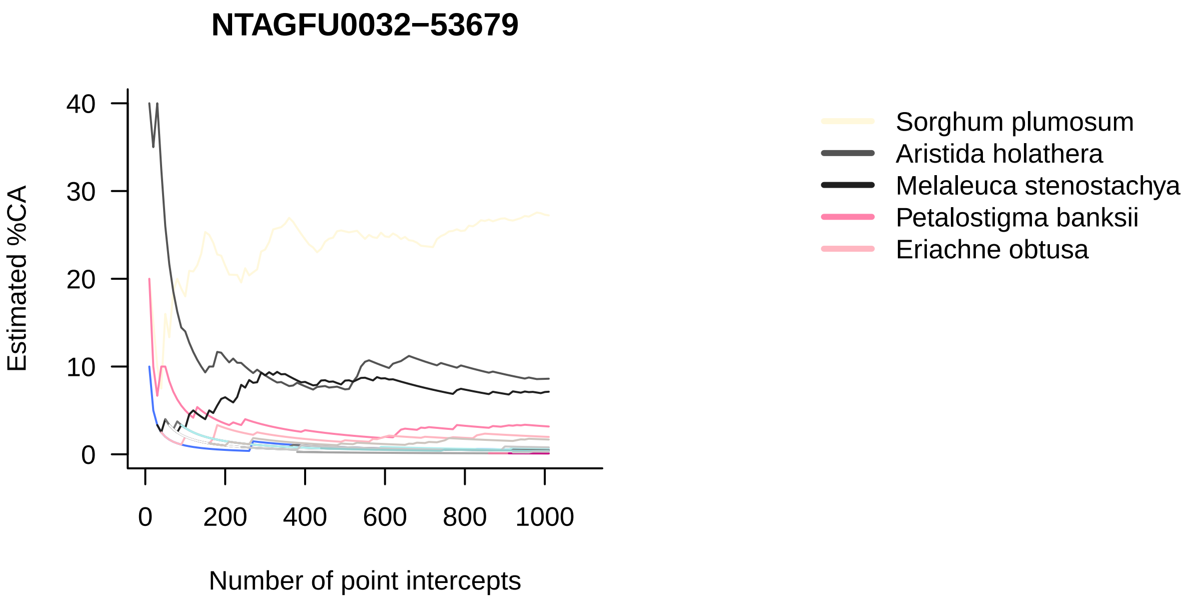
NTAGFU0032−53679
Estimated %CA
40
30
20
10
0
0
200
400
600
800
1000
Number of point intercepts
Sorghum plumosum
Aristida holathera
Melaleuca stenostachya
Petalostigma banksii
Eriachne obtusa

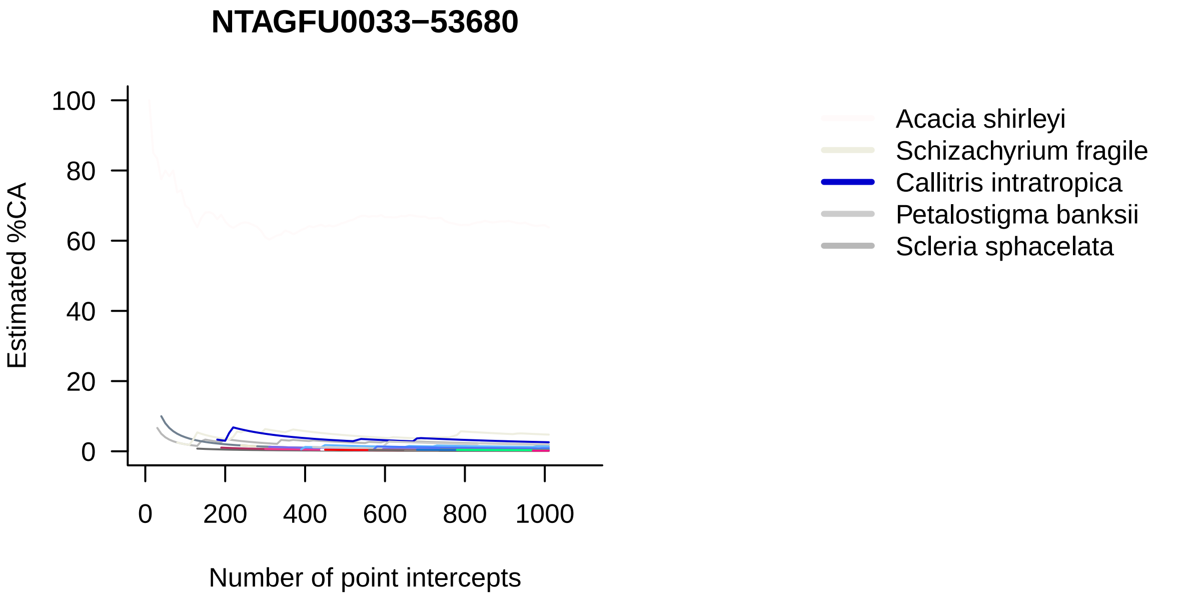
NTAGFU0033−53680
Estimated %CA
100
80
60
40
20
0
0
200
400
600
800
1000
Number of point intercepts
Acacia shirleyi
Schizachyrium fragile
Callitris intratropica
Petalostigma banksii
Scleria sphacelata

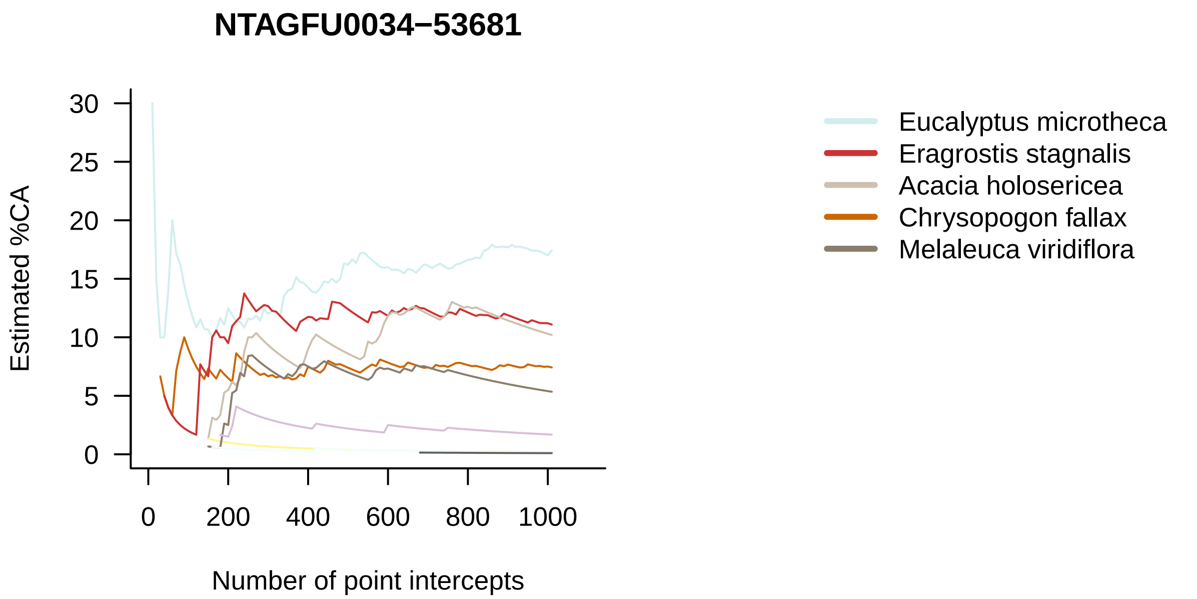
NTAGFU0034−53681
Estimated %CA
Number of point intercepts
0
5
10
15
20
25
30
0
200
400
600
800
1000
Eucalyptus microtheca
Eragrostis stagnalis
Acacia holosericea
Chrysopogon fallax
Melaleuca viridiflora

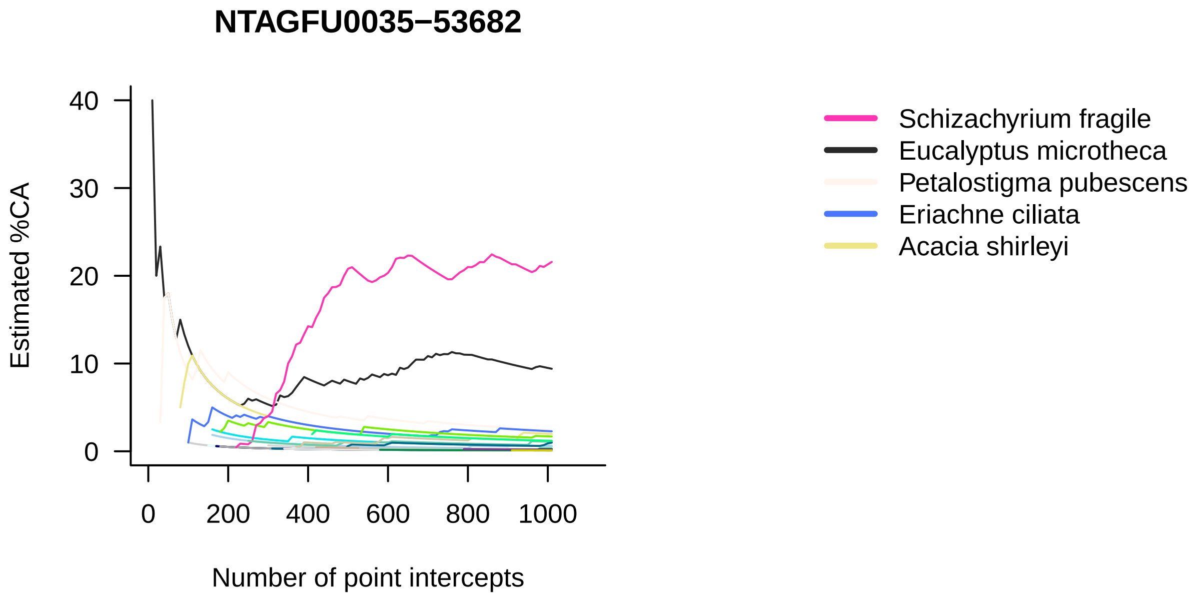
NTAGFU0035−53682
Estimated %CA
Number of point intercepts
0
10
20
30
40
0
200
400
600
800
1000
Schizachyrium fragile
Eucalyptus microtheca
Petalostigma pubescens
Eriachne ciliata
Acacia shirleyi

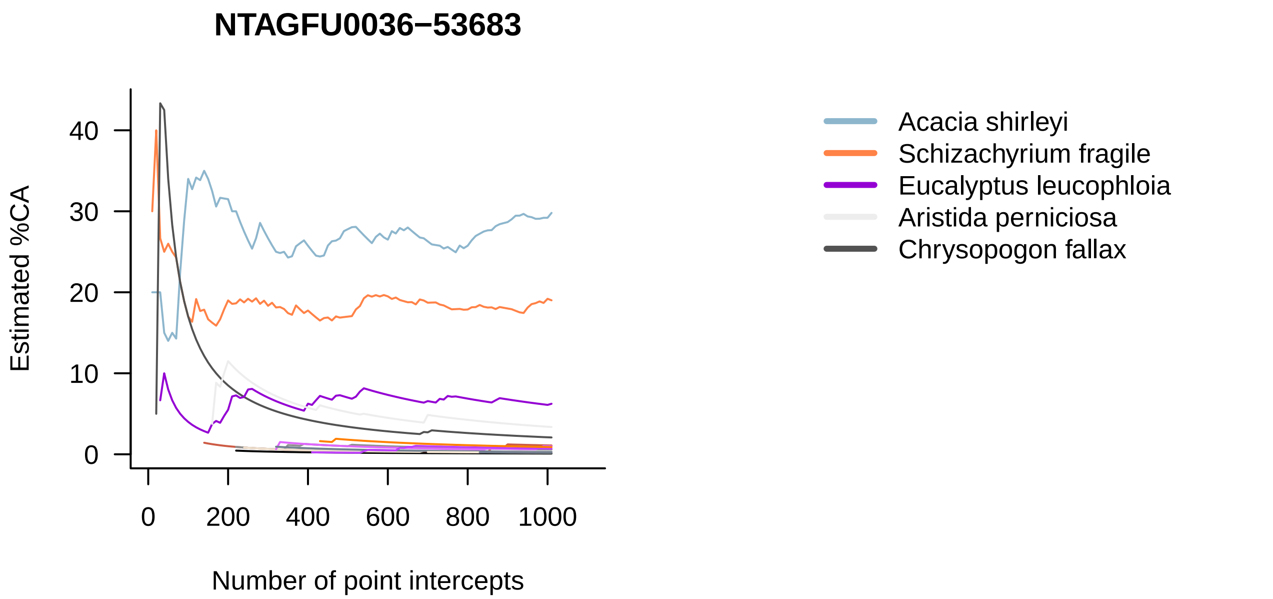

NTAGFU0036−53683
Estimated %CA
0
10
20
30
40
0
200
400
600
800
1000
Number of point intercepts
Acacia shirleyi
Schizachyrium fragile
Eucalyptus leucophloia
Aristida perniciosa
Chrysopogon fallax


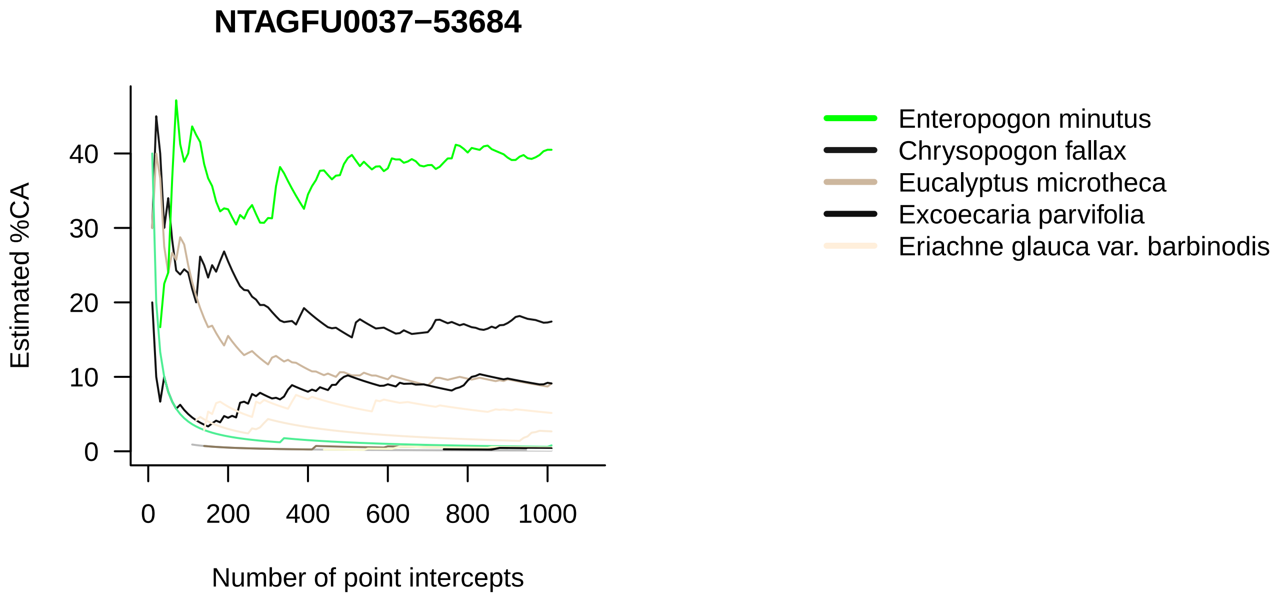

NTAGFU0037−53684
Estimated %CA
0
10
20
30
40
0
200
400
600
800
1000
Number of point intercepts
Enteropogon minutus
Chrysopogon fallax
Eucalyptus microtheca
Excoecaria parvifolia
Eriachne glauca var. barbinodis

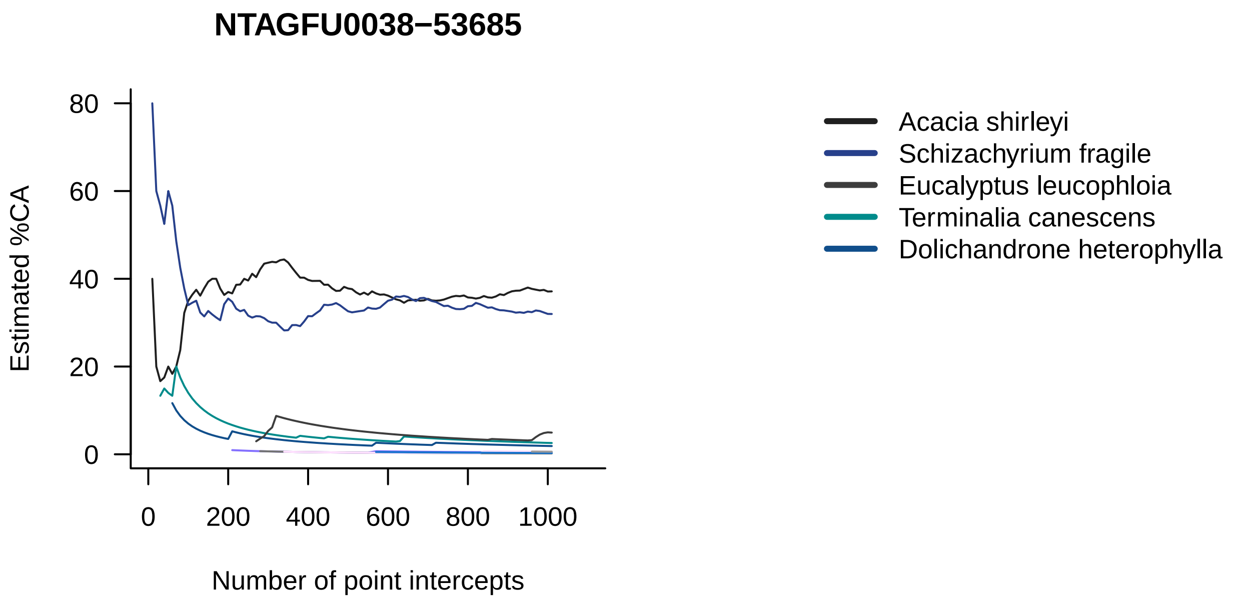
NTAGFU0038−53685
Estimated %CA
0
20
40
60
80
0
200
400
600
800
1000
Number of point intercepts
Acacia shirleyi
Schizachyrium fragile
Eucalyptus leucophloia
Terminalia canescens
Dolichandrone heterophylla

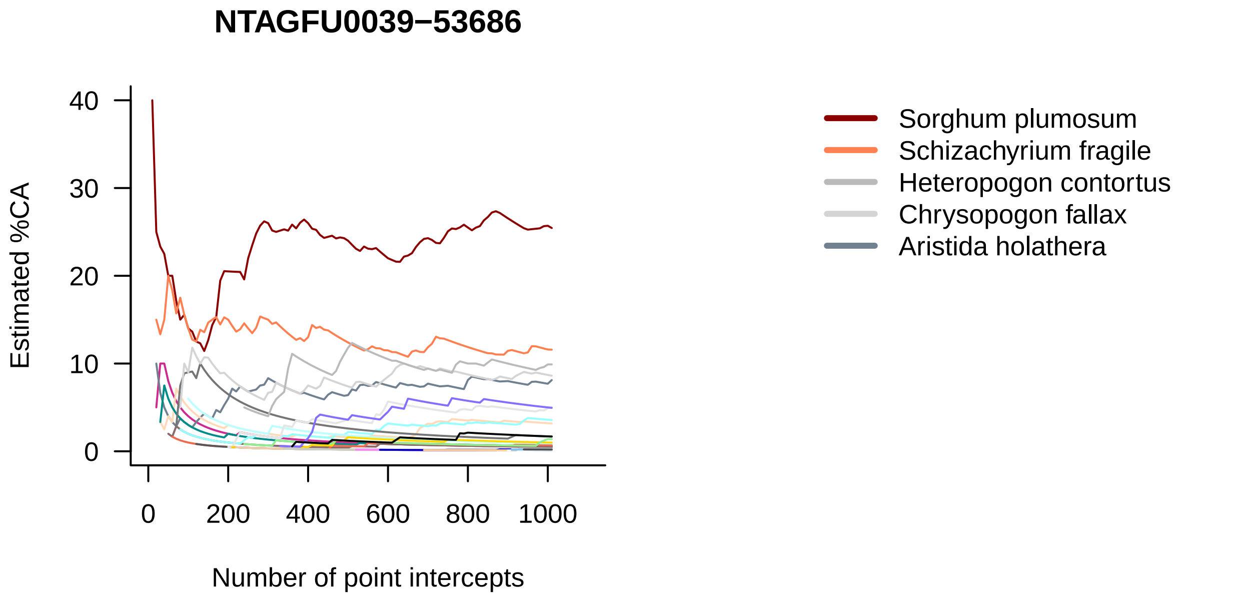
NTAGFU0039−53686
Estimated %CA
0
10
20
30
40
0
200
400
600
800
1000
Number of point intercepts
Sorghum plumosum
Schizachyrium fragile
Heteropogon contortus
Chrysopogon fallax
Aristida holathera

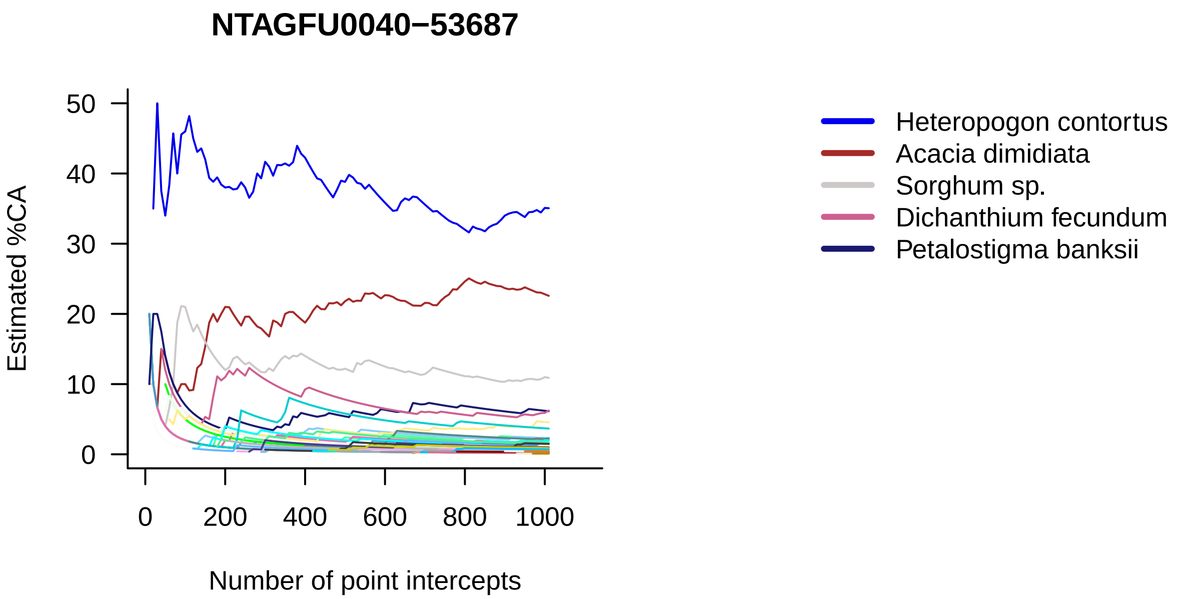
NTAGFU0040−53687
Estimated %CA
0
10
20
30
40
50
0
200
400
600
800
1000
Number of point intercepts
Heteropogon contortus
Acacia dimidiata
Sorghum sp.
Dichanthium fecundum
Petalostigma banksii

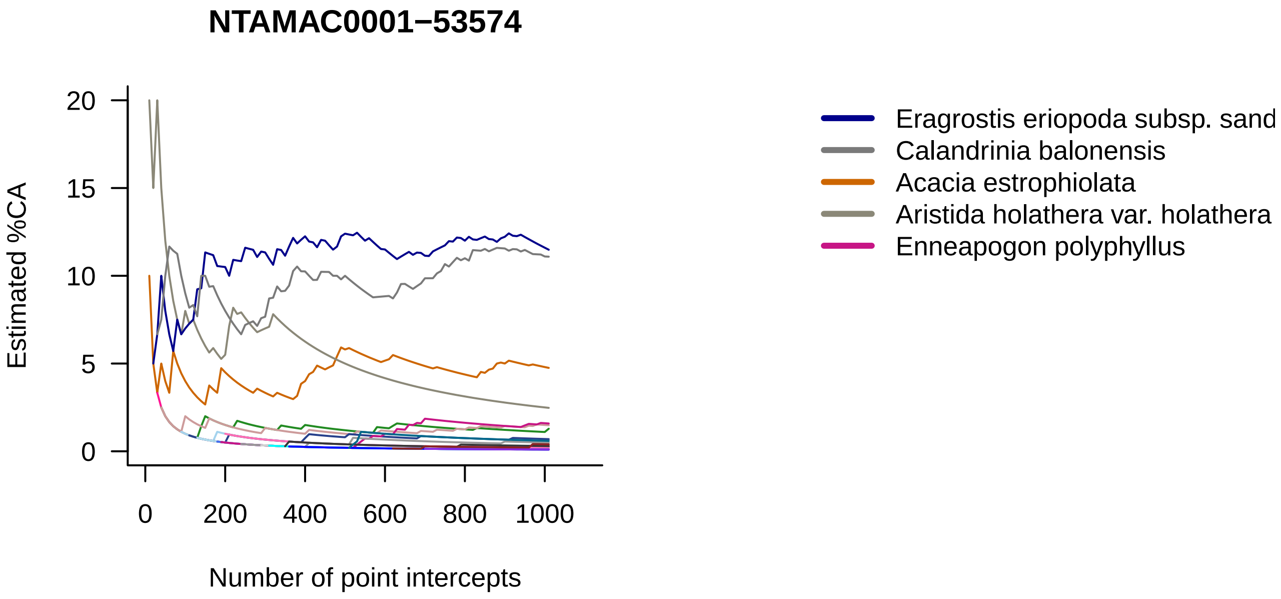
NTAMAC0001−53574
Estimated %CA
0
5
10
15
20
0
200
400
600
800
1000
Number of point intercepts
Eragrostis eriopoda subsp. sand
Calandrinia balonensis
Acacia estrophiolata
Aristida holathera var. holathera
Enneapogon polyphyllus

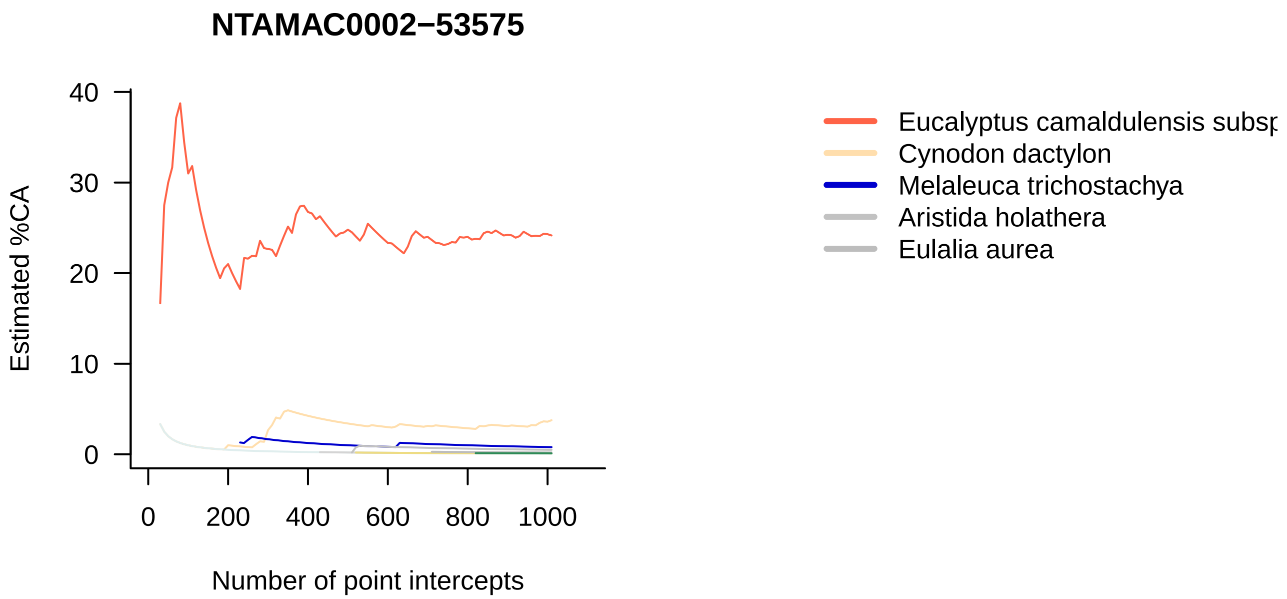
NTAMAC0002−53575
Estimated %CA
0
10
20
30
40
0
200
400
600
800
1000
Number of point intercepts
Eucalyptus camaldulensis subsp
Cynodon dactylon
Melaleuca trichostachya
Aristida holathera
Eulalia aurea

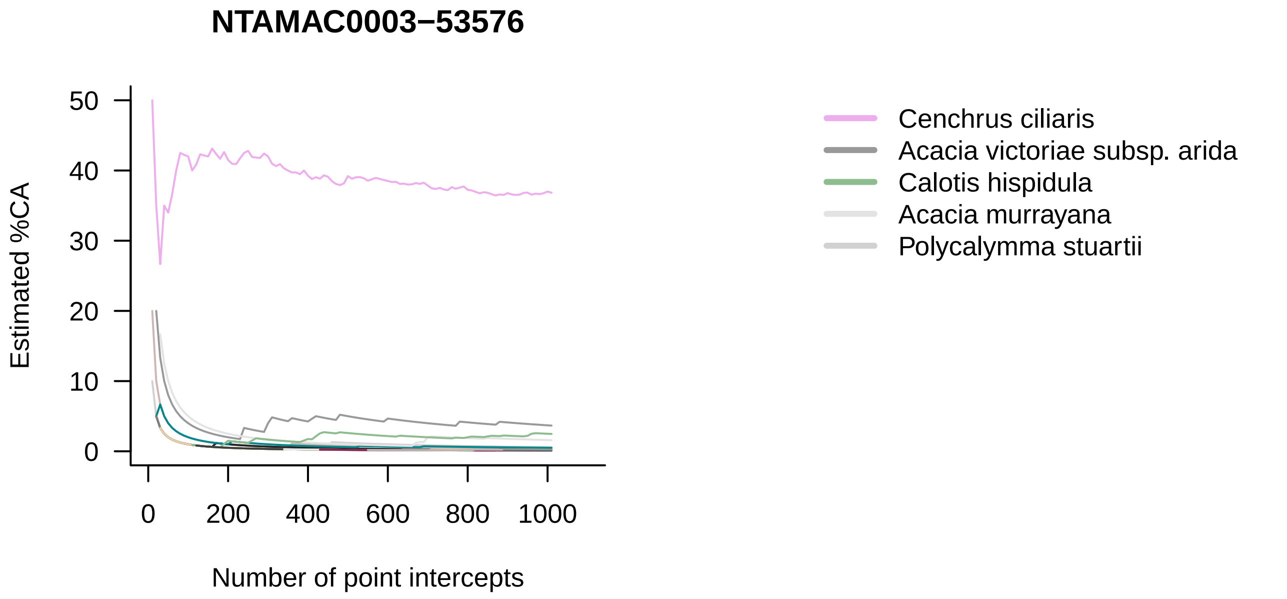
NTAMAC0003−53576
Estimated %CA
0
10
20
30
40
50
0
200
400
600
800
1000
Number of point intercepts
Cenchrus ciliaris
Acacia victoriae subsp. arida
Calotis hispidula
Acacia murrayana
Polycalymma stuartii

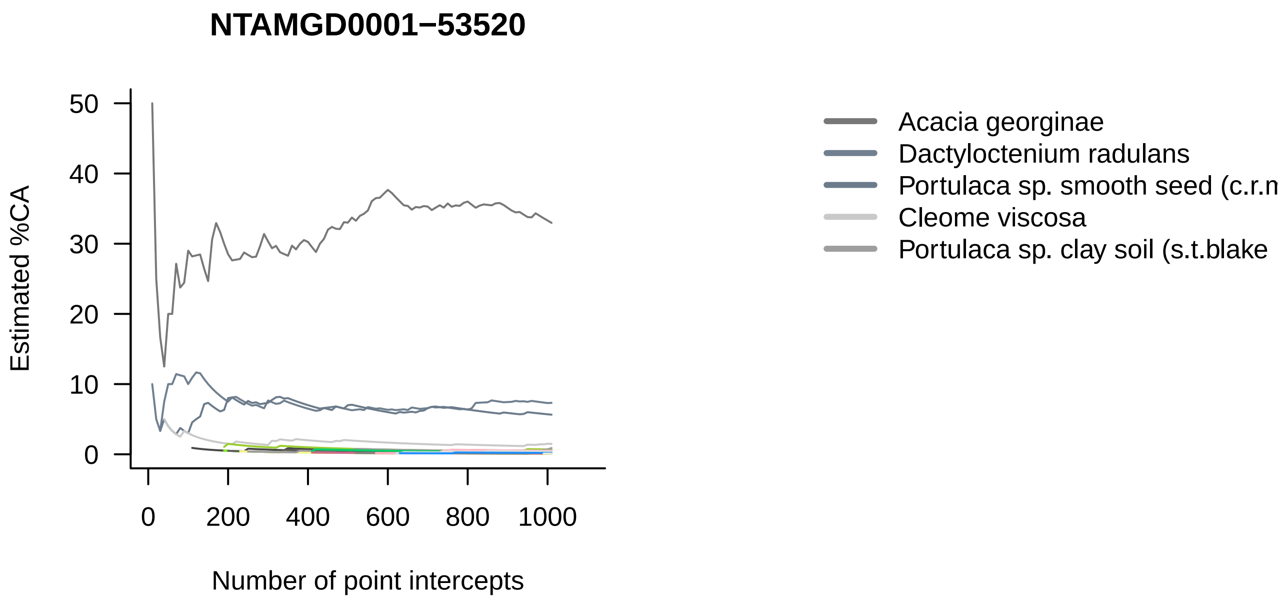
NTAMGD0001−53520
Estimated %CA
Number of point intercepts
Acacia georginae
Dactyloctenium radulans
Portulaca sp. smooth seed (c.r.n
Cleome viscosa
Portulaca sp. clay soil (s.t.blake

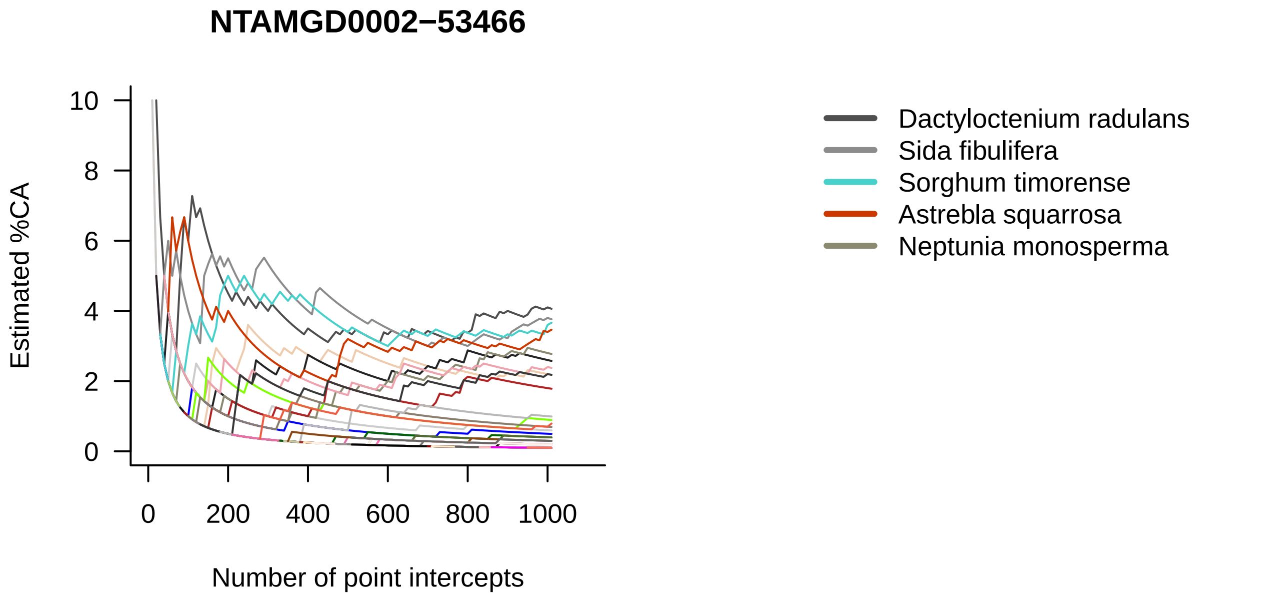
NTAMGD0002−53466
Estimated %CA
Number of point intercepts
Dactyloctenium radulans
Sida fibulifera
Sorghum timorense
Astrebla squarrosa
Neptunia monosperma

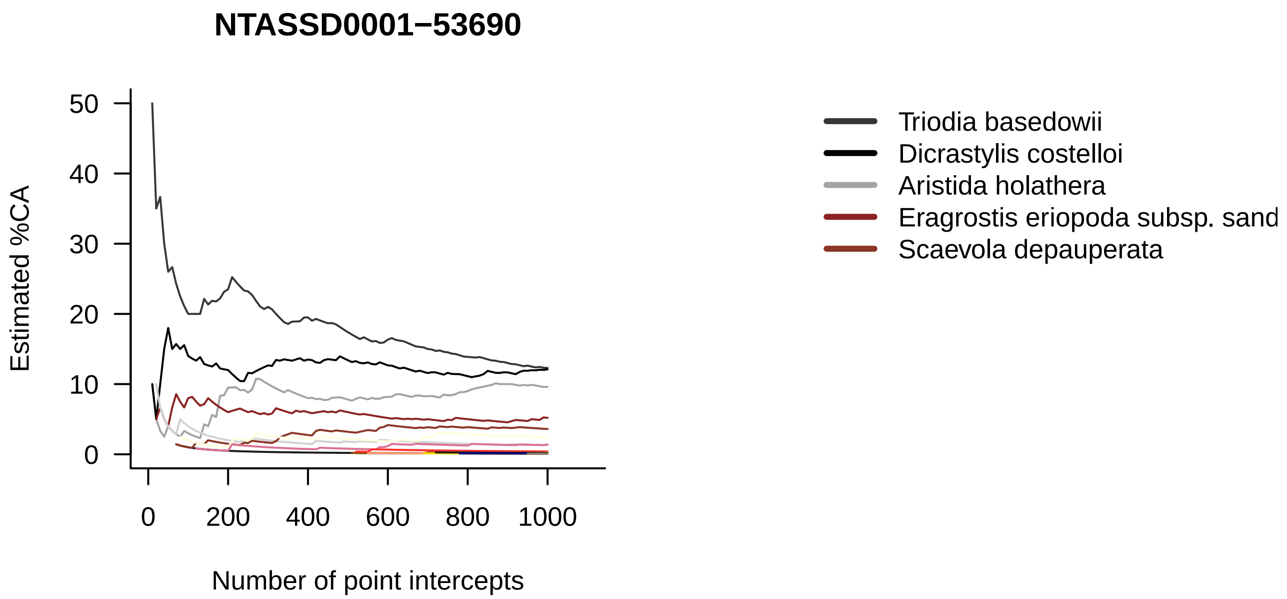
NTASSD0001−53690
Estimated %CA
0
10
20
30
40
50
0
200
400
600
800
1000
Number of point intercepts
Triodia basedowii
Dicrastylis costelloi
Aristida holathera
Eragrostis eriopoda subsp. sand
Scaevola depauperata

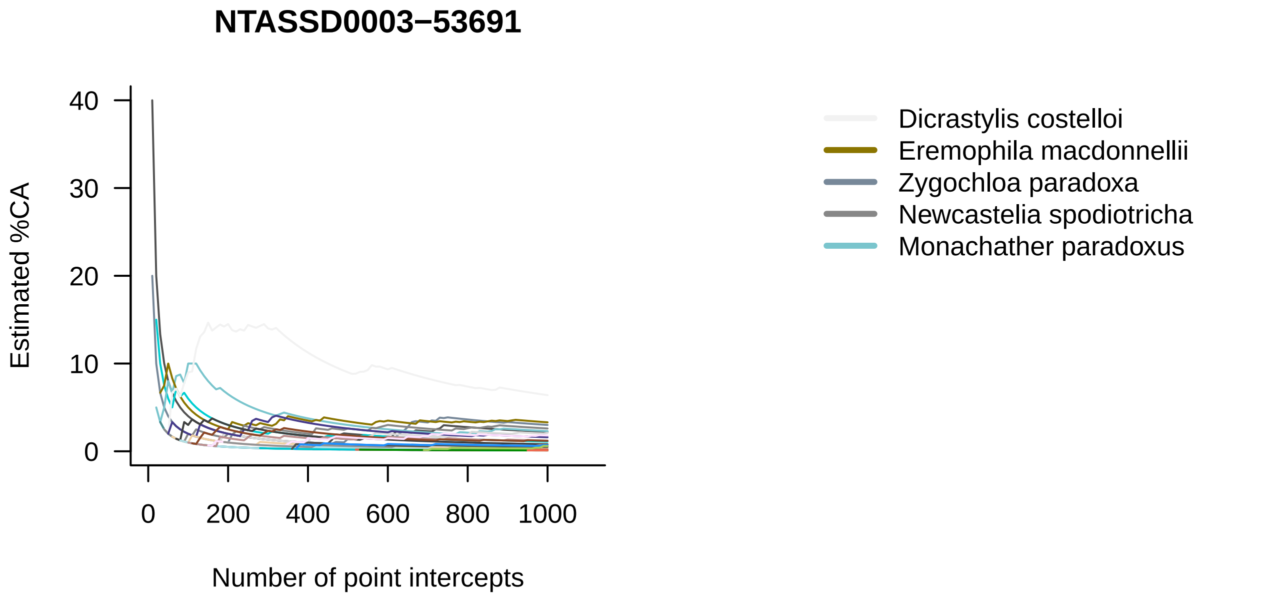
NTASSD0003−53691
Estimated %CA
0
10
20
30
40
0
200
400
600
800
1000
Number of point intercepts
Dicrastylis costelloi
Eremophila macdonnellii
Zygochloa paradoxa
Newcastelia spodiotricha
Monachather paradoxus

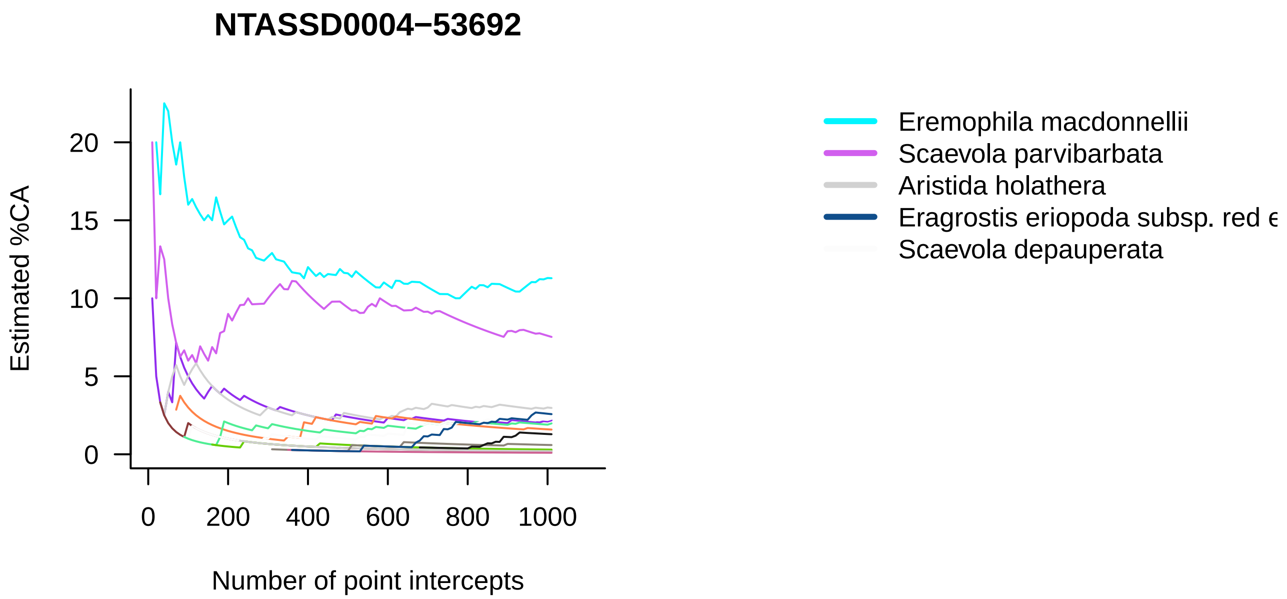
NTASSD0004−53692
Estimated %CA
Number of point intercepts
0
5
10
15
20
0
200
400
600
800
1000
Eremophila macdonnellii
Scaevola parvibarbata
Aristida holathera
Eragrostis eriopoda subsp. red e
Scaevola depauperata

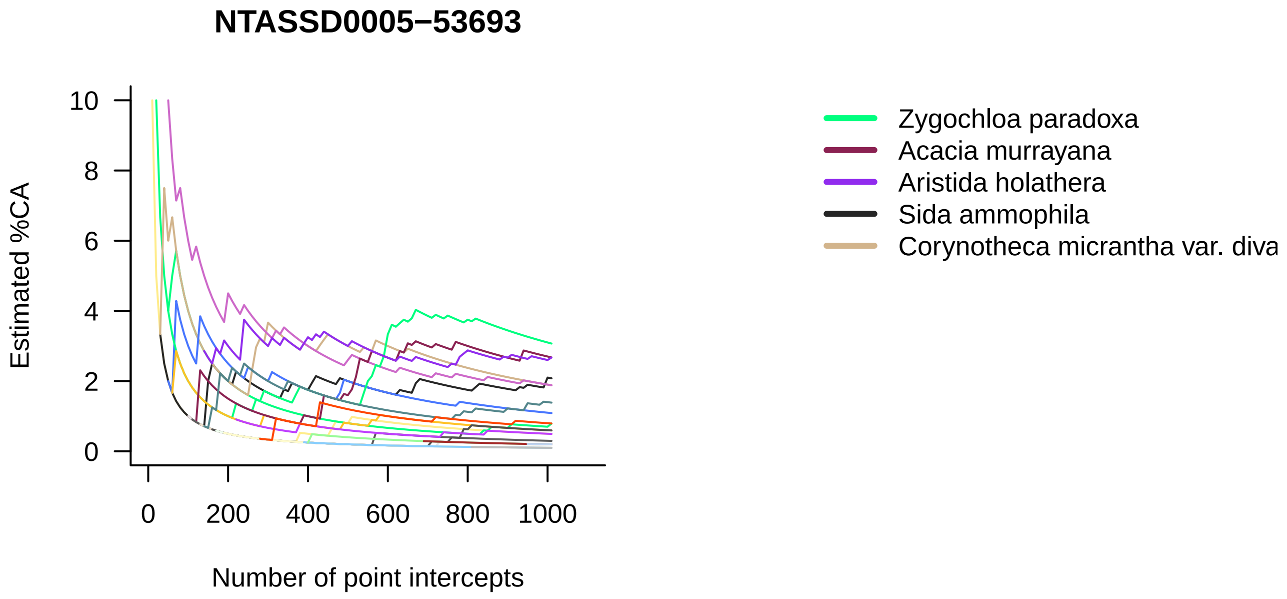
NTASSD0005−53693
Estimated %CA
Number of point intercepts
0
2
4
6
8
10
0
200
400
600
800
1000
Zygochloa paradoxa
Acacia murrayana
Aristida holathera
Sida ammophila
Corynotheca micrantha var. diva

## NTASSD0006−53694

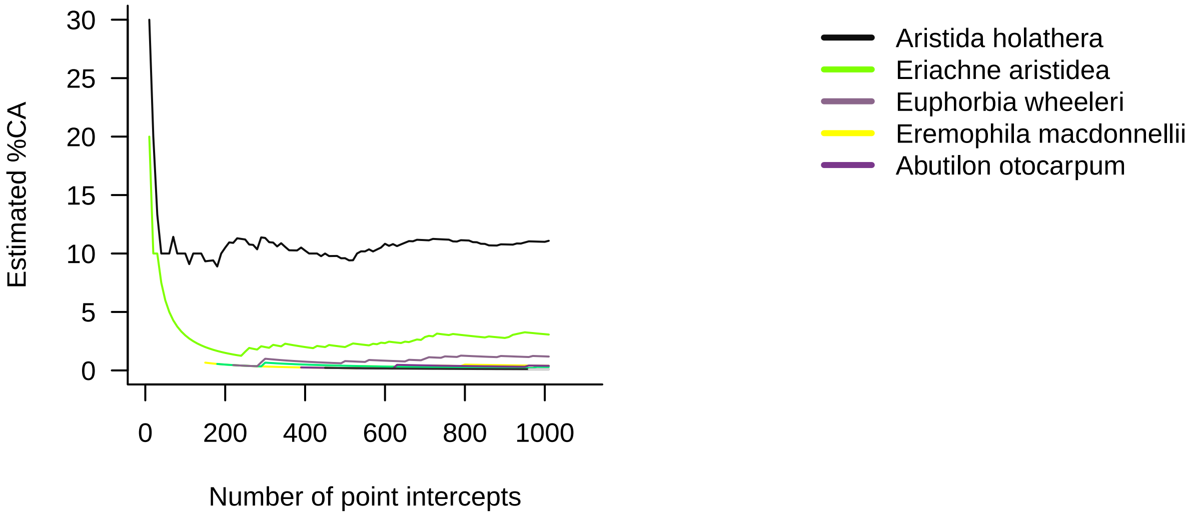


## NTASSD0007−53695

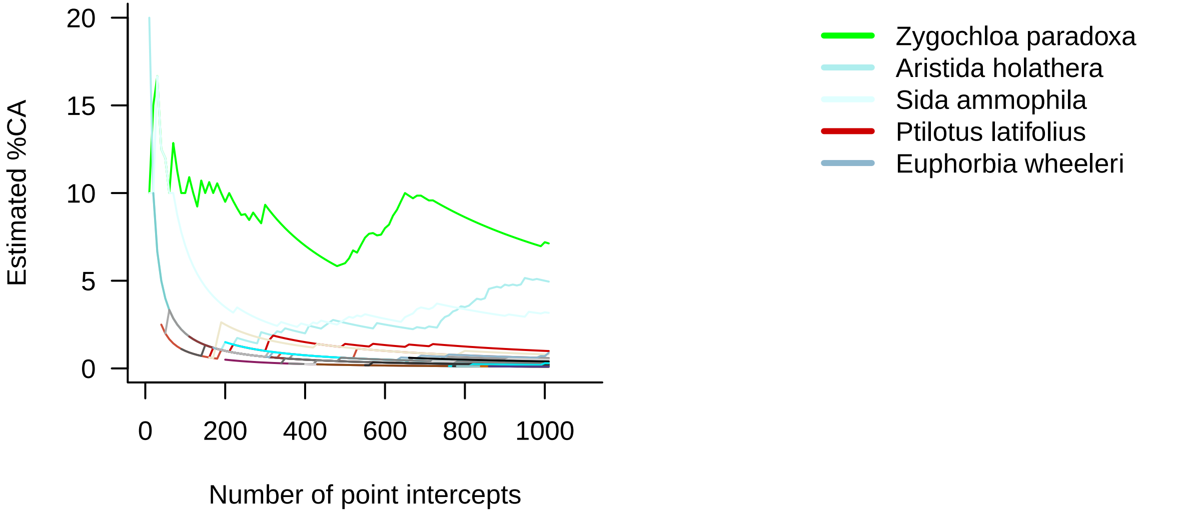

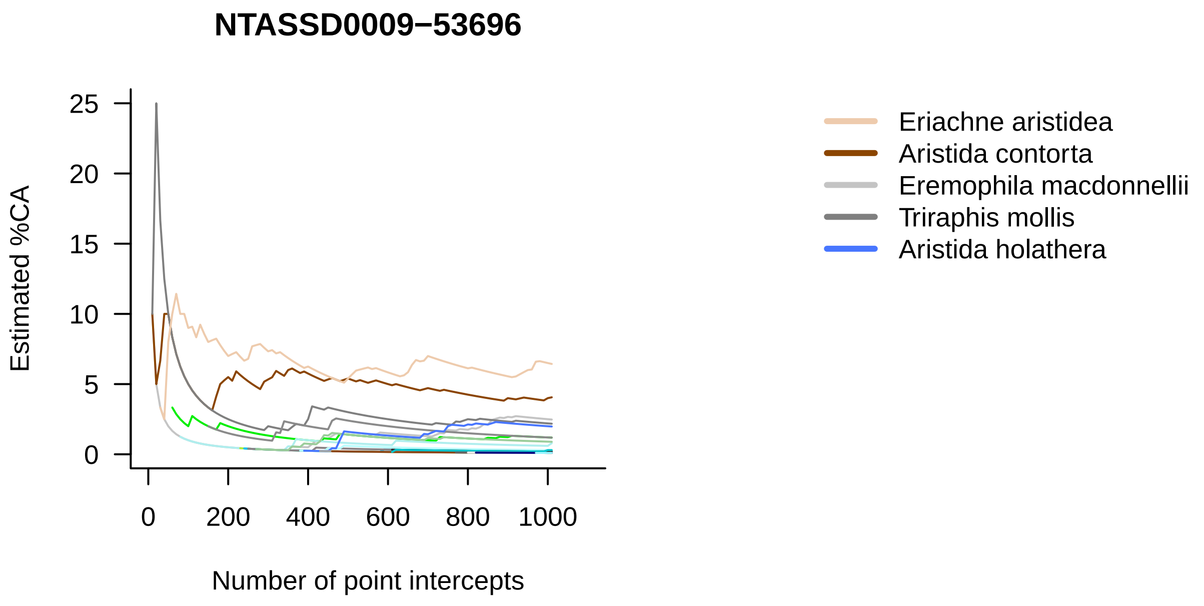
NTASSD0009−53696
Estimated %CA
0
5
10
15
20
25
0
200
400
600
800
1000
Number of point intercepts
Eriachne aristidea
Aristida contorta
Eremophila macdonnellii
Triraphis mollis
Aristida holathera

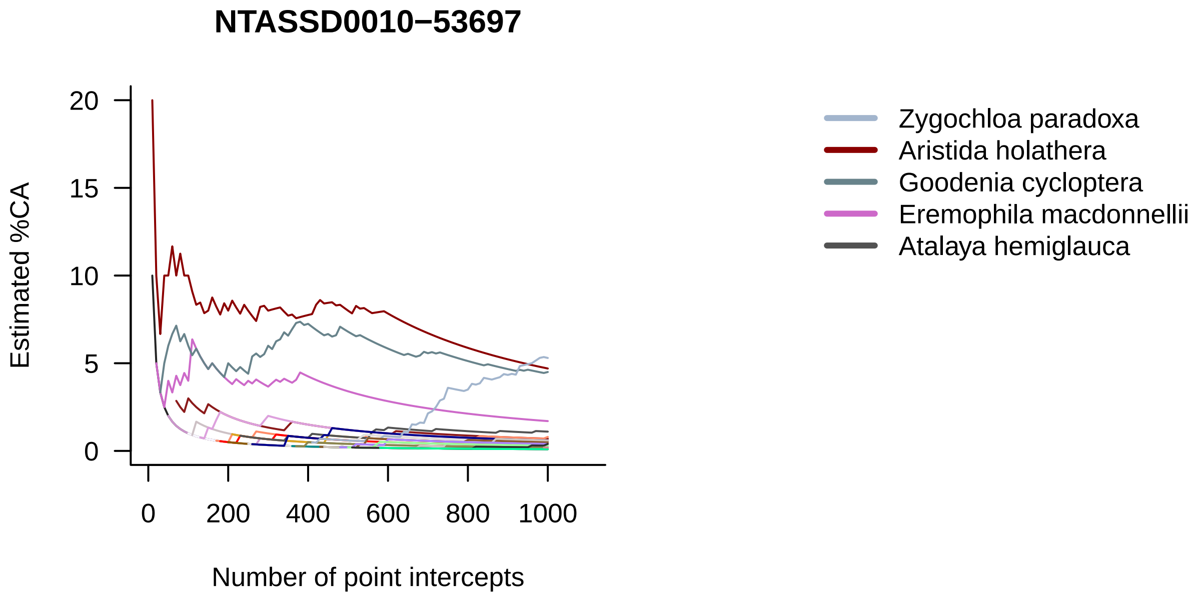
NTASSD0010−53697
Estimated %CA
0
5
10
15
20
0
200
400
600
800
1000
Number of point intercepts
Zygochloa paradoxa
Aristida holathera
Goodenia cycloptera
Eremophila macdonnellii
Atalaya hemiglauca

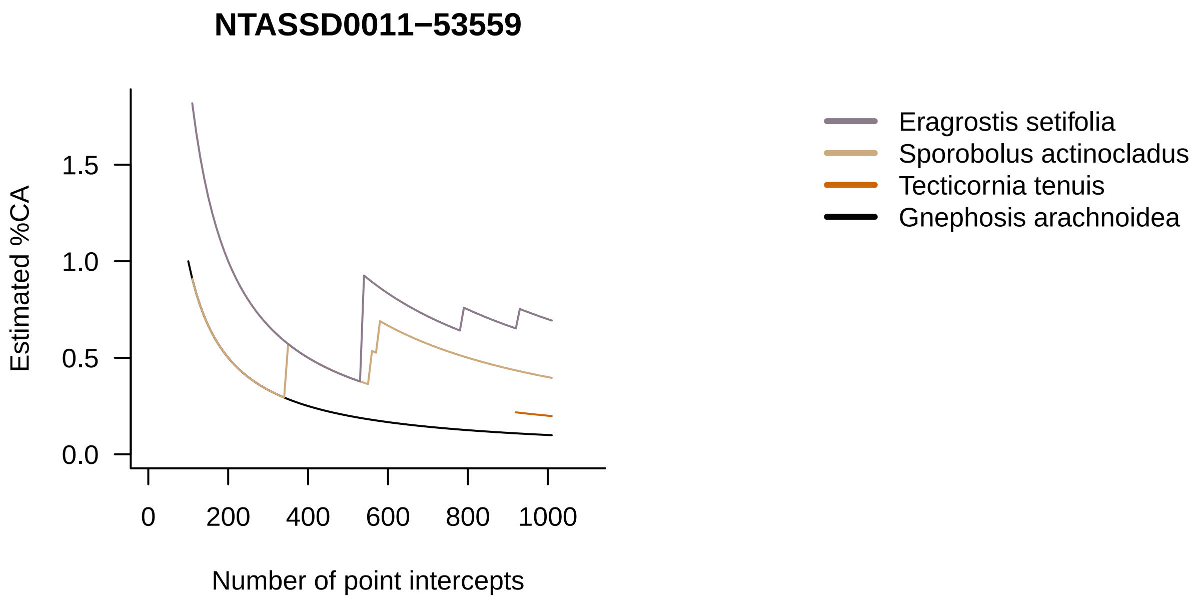
NTASSD0011−53559
Estimated %CA
0.0
0.5
1.0
1.5
0
200
400
600
800
1000
Number of point intercepts
Eragrostis setifolia
Sporobolus actinocladus
Tecticornia tenuis
Gnephosis arachnoidea

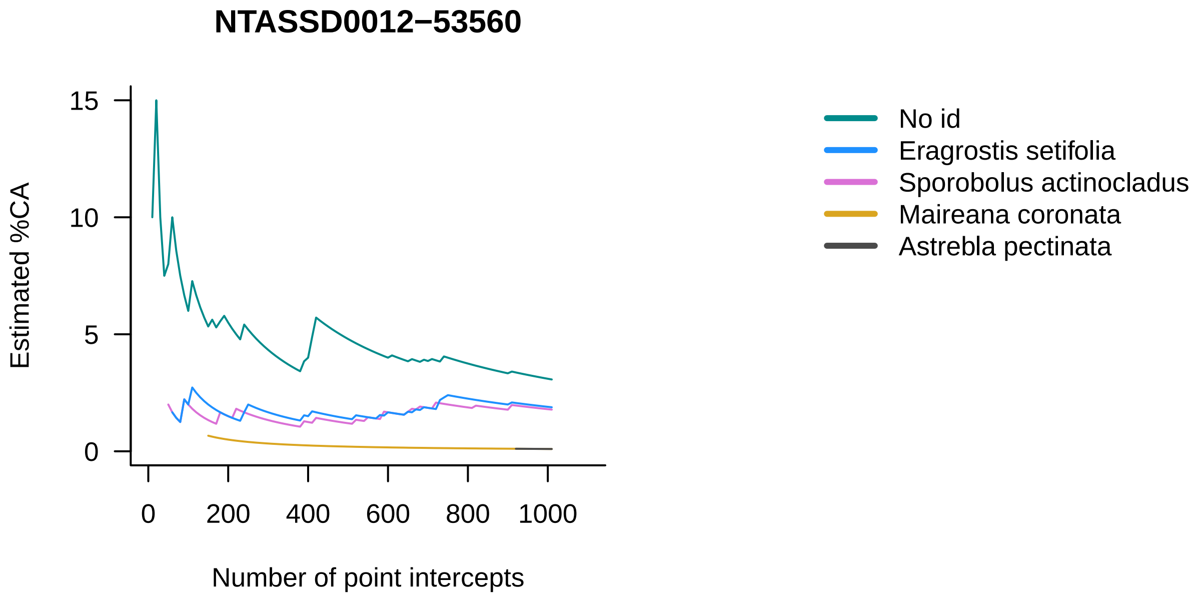
NTASSD0012−53560
Estimated %CA
0
5
10
15
0
200
400
600
800
1000
Number of point intercepts
No id
Eragrostis setifolia
Sporobolus actinocladus
Maireana coronata
Astrebla pectinata

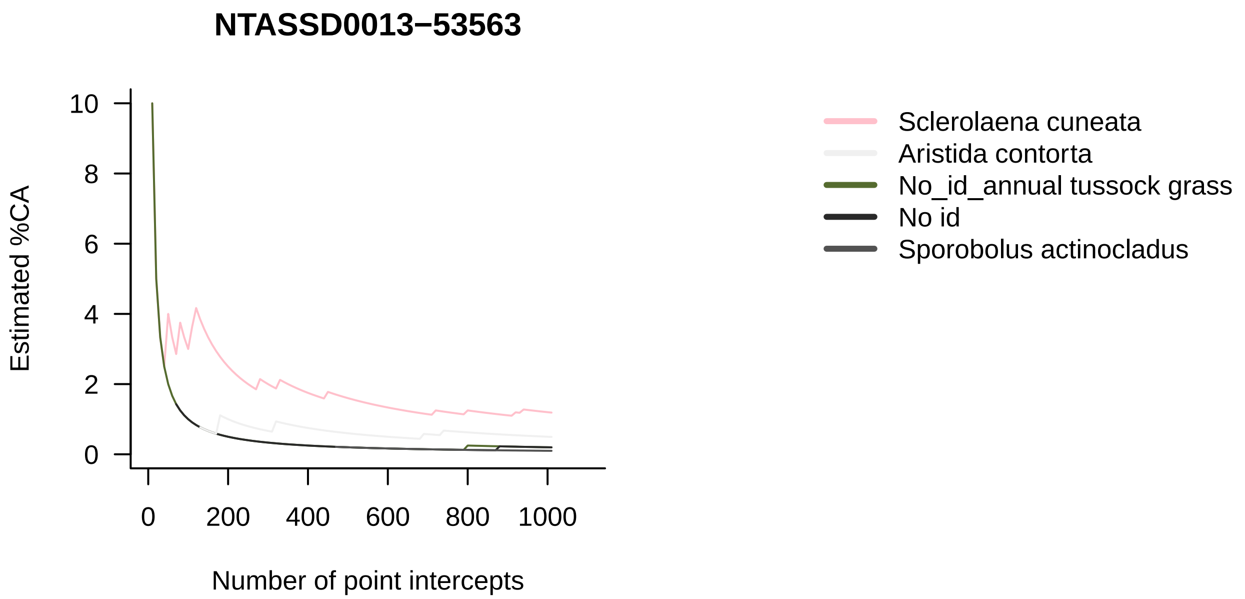
NTASSD0013−53563
Estimated %CA
Number of point intercepts
0
2
4
6
8
10
0
200
400
600
800
1000
Sclerolaena cuneata
Aristida contorta
No_id_annual tussock grass
No id
Sporobolus actinocladus

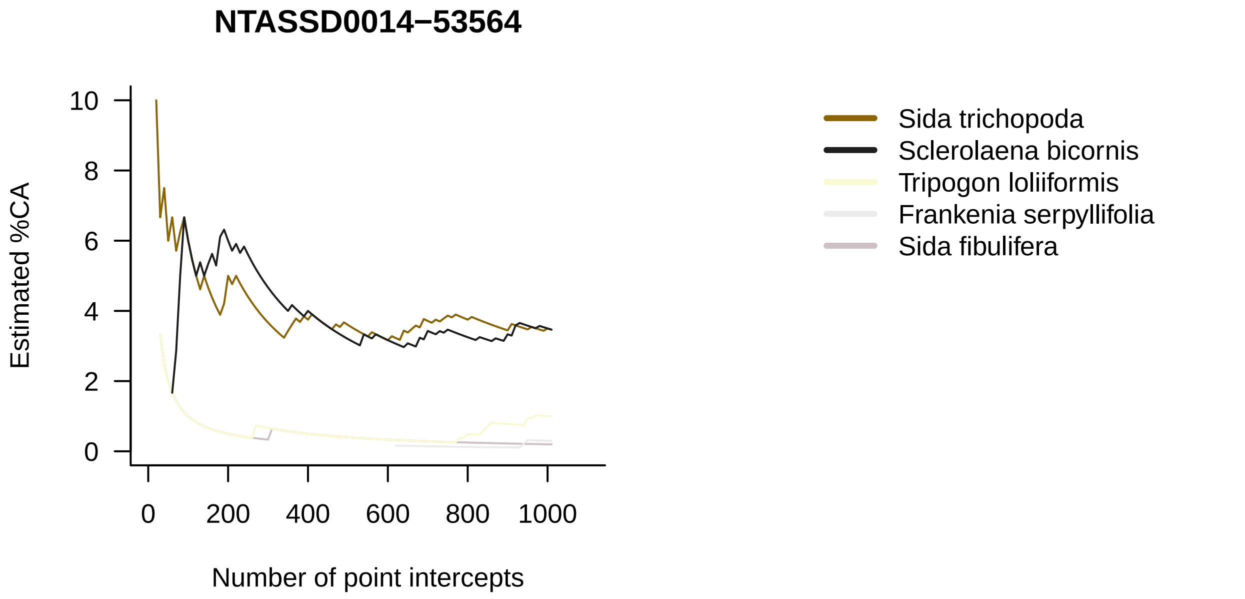
NTASSD0014−53564
Estimated %CA
Number of point intercepts
0
2
4
6
8
10
0
200
400
600
800
1000
Sida trichopoda
Sclerolaena bicornis
Tripogon loliiformis
Frankenia serpyllifolia
Sida fibulifera

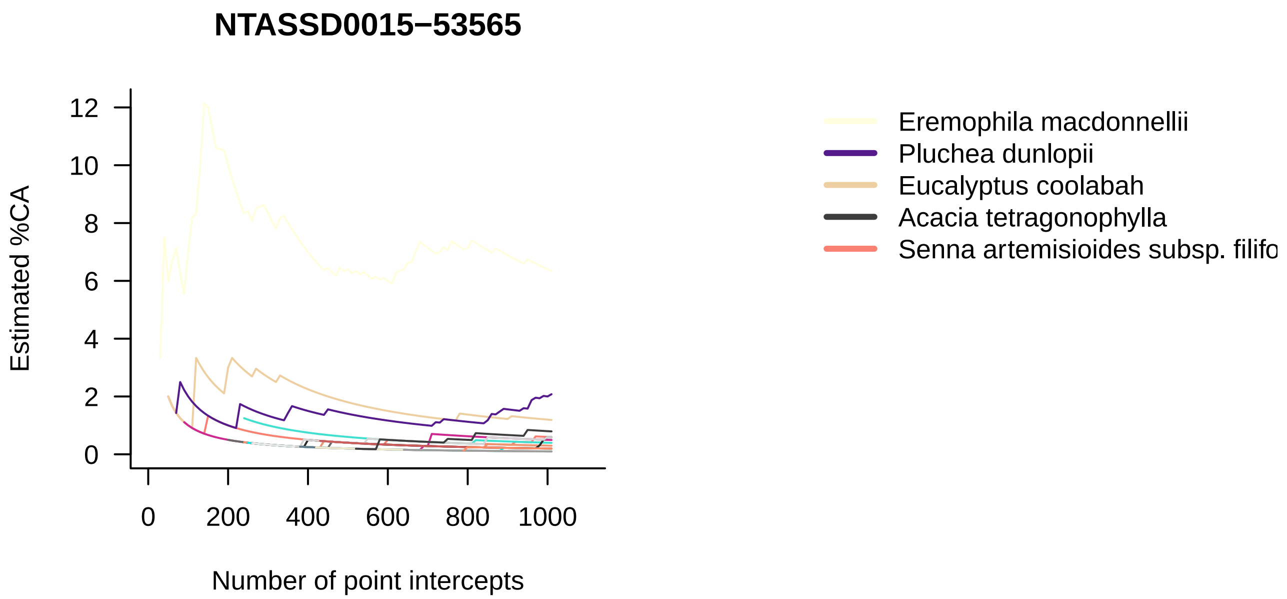
NTASSD0015−53565
Estimated %CA
Number of point intercepts
0
2
4
6
8
10
12
0
200
400
600
800
1000
Eremophila macdonnellii
Pluchea dunlopii
Eucalyptus coolabah
Acacia tetragonophylla
Senna artemisioides subsp. filifo

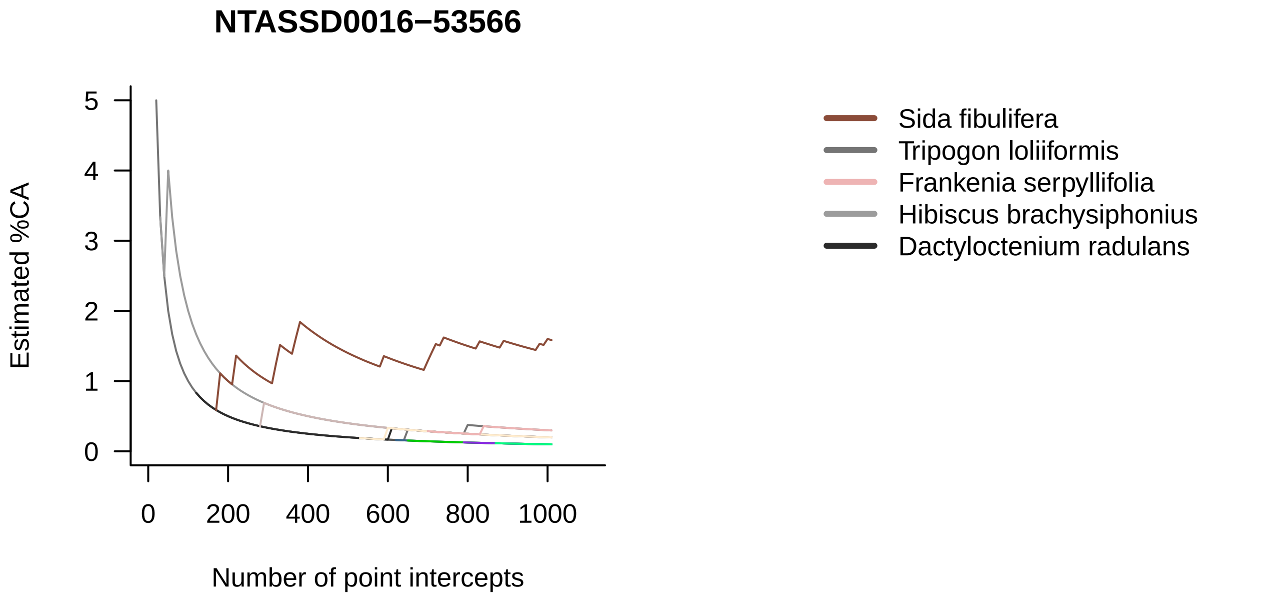
NTASSD0016−53566
Estimated %CA
Number of point intercepts
0
1
2
3
4
5
0
200
400
600
800
1000
Sida fibulifera
Tripogon loliiformis
Frankenia serpyllifolia
Hibiscus brachysiphonius
Dactyloctenium radulans

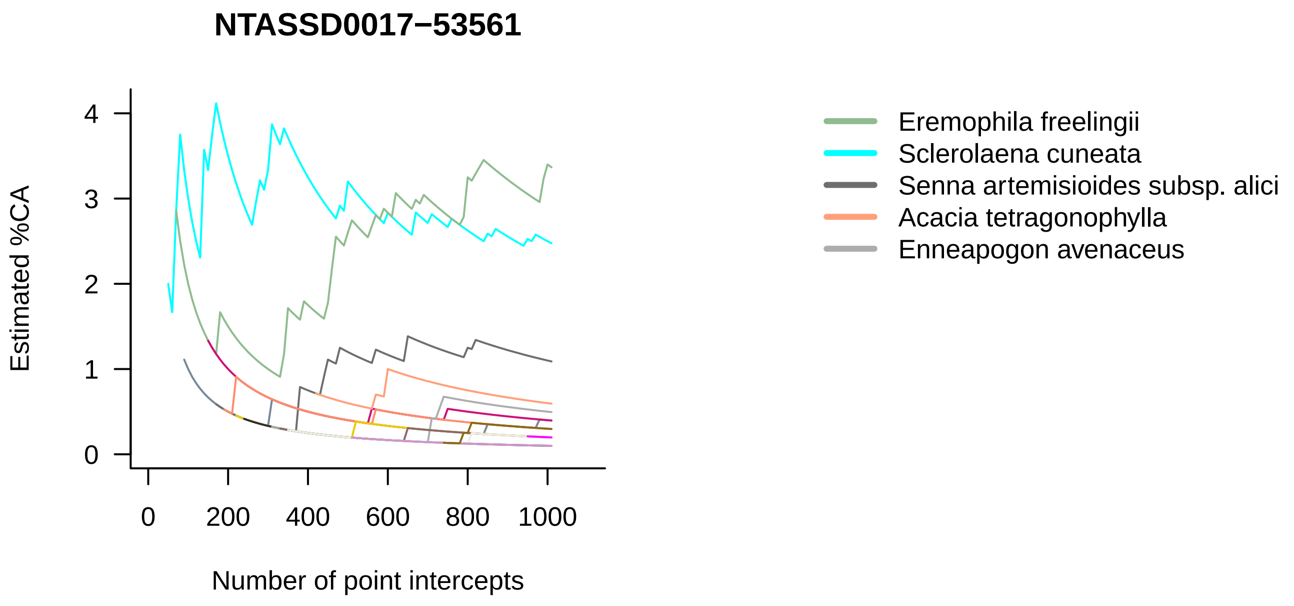

NTASSD0017−53561
Estimated %CA
0
1
2
3
4
0
200
400
600
800
1000
Number of point intercepts
Eremophila freelingii
Sclerolaena cuneata
Senna artemisioides subsp. alici
Acacia tetragonophylla
Enneapogon avenaceus


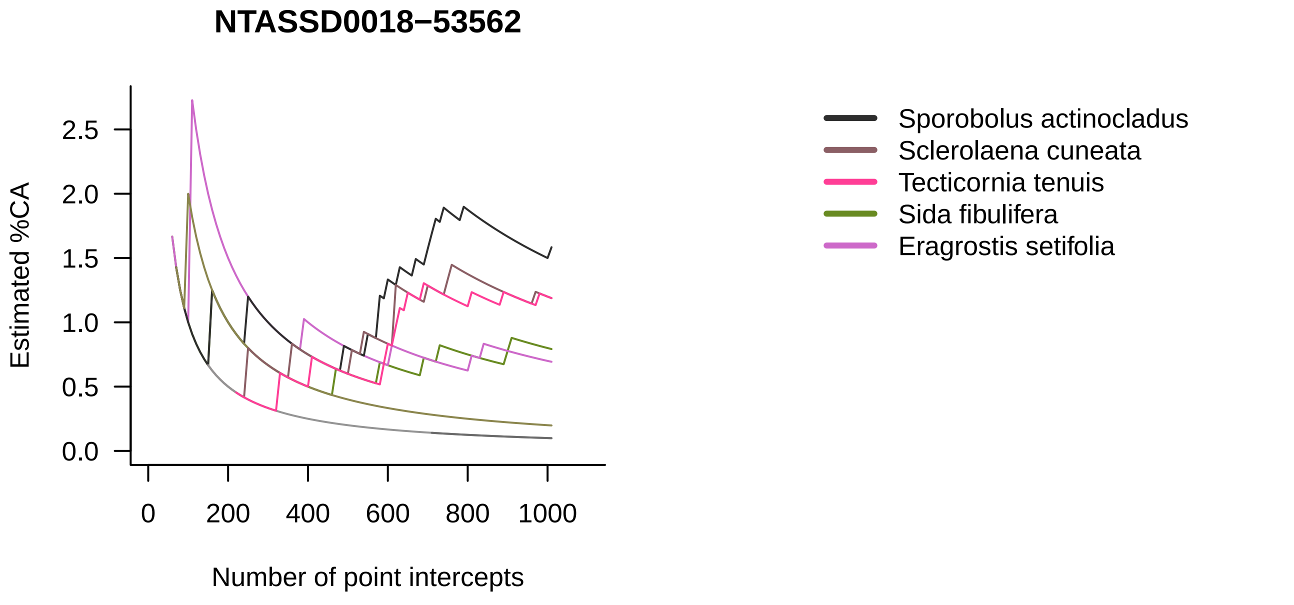

NTASSD0018−53562
Estimated %CA
0.0
0.5
1.0
1.5
2.0
2.5
0
200
400
600
800
1000
Number of point intercepts
Sporobolus actinocladus
Sclerolaena cuneata
Tecticornia tenuis
Sida fibulifera
Eragrostis setifolia

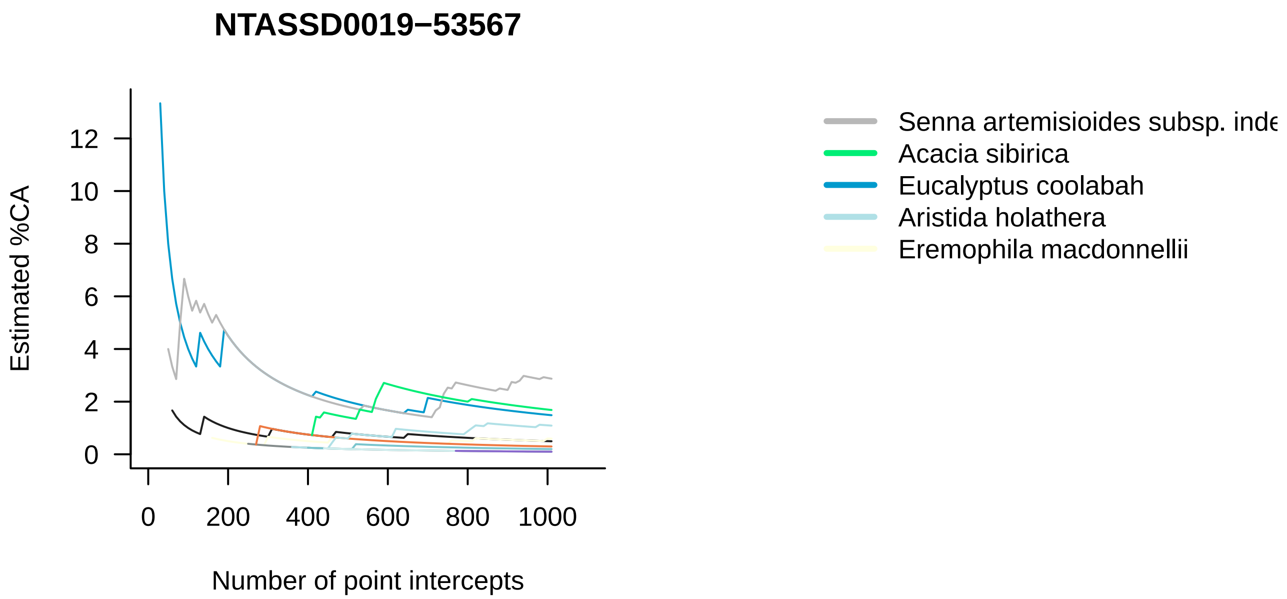
NTASSD0019−53567
Estimated %CA
0
2
4
6
8
10
12
0
200
400
600
800
1000
Number of point intercepts
Senna artemisioides subsp. inde
Acacia sibirica
Eucalyptus coolabah
Aristida holathera
Eremophila macdonnellii

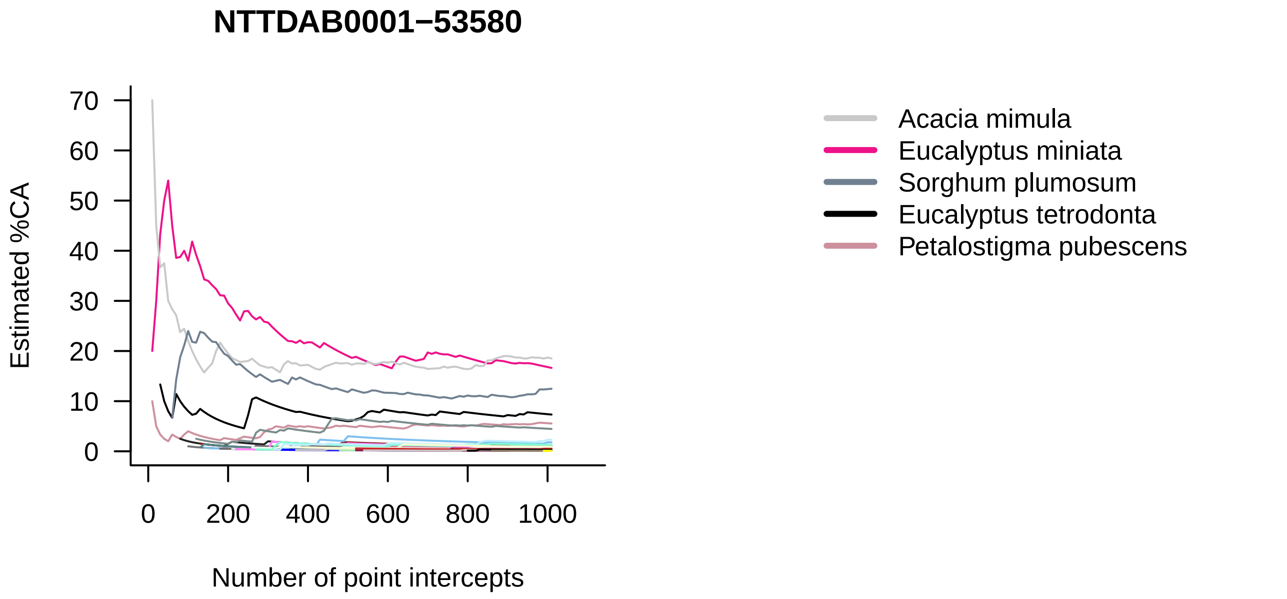
NTTDAB0001−53580
Estimated %CA
0
10
20
30
40
50
60
70
0
200
400
600
800
1000
Number of point intercepts
Acacia mimula
Eucalyptus miniata
Sorghum plumosum
Eucalyptus tetrodonta
Petalostigma pubescens

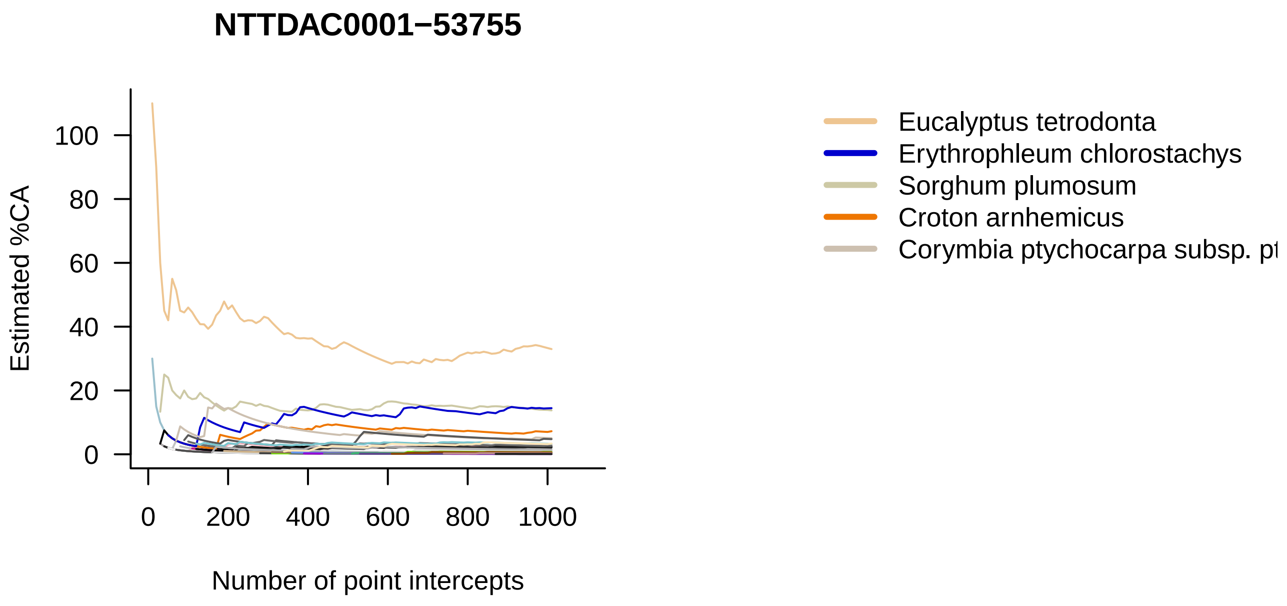

NTTDAC0001−53755
Estimated %CA
0
20
40
60
80
100
0
200
400
600
800
1000
Number of point intercepts
Eucalyptus tetrodonta
Erythrophleum chlorostachys
Sorghum plumosum
Croton arnhemicus
Corymbia ptychocarpa subsp. pt


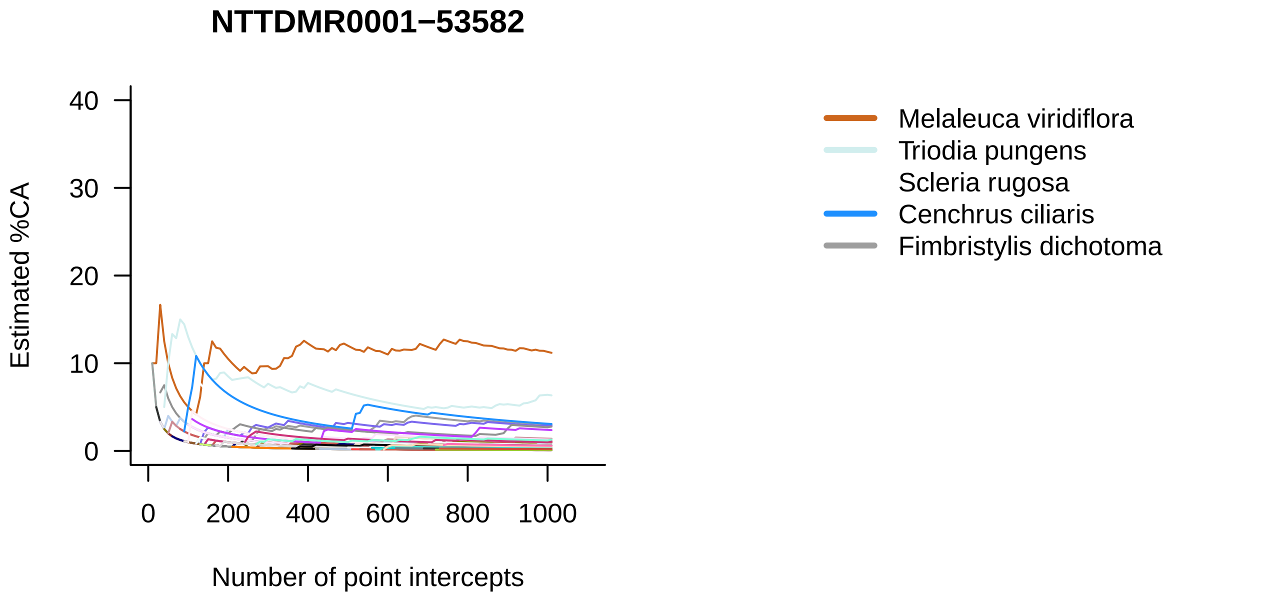

NTTDMR0001−53582
Estimated %CA
0
10
20
30
40
0
200
400
600
800
1000
Number of point intercepts
Melaleuca viridiflora
Triodia pungens
Scleria rugosa
Cenchrus ciliaris
Fimbristylis dichotoma

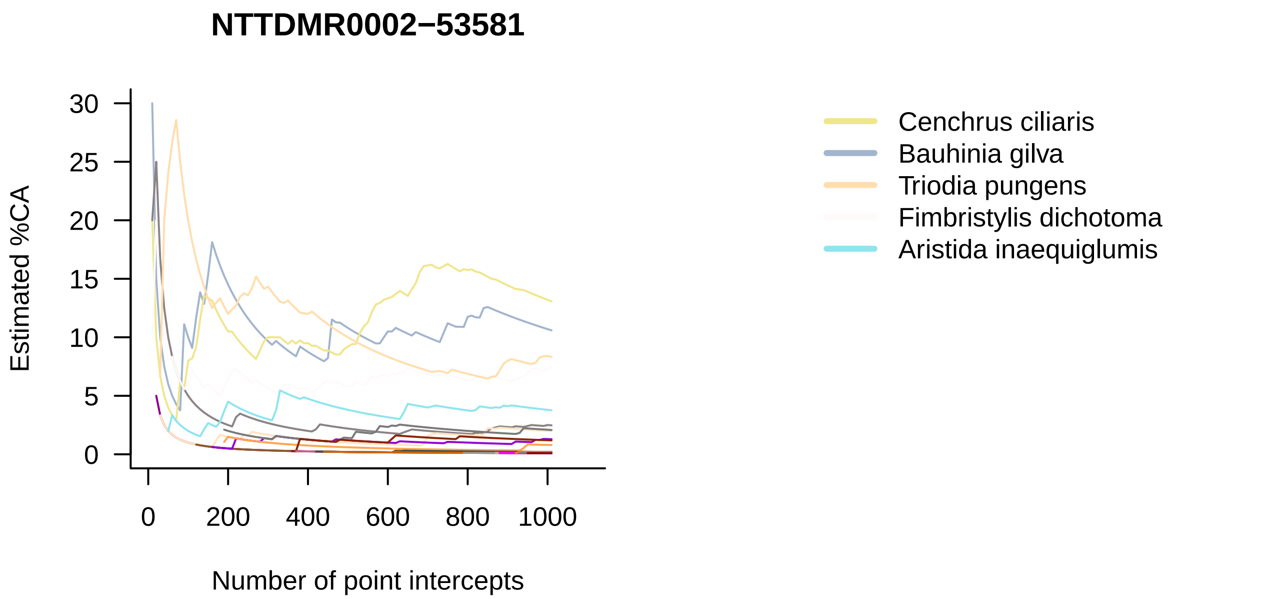
NTTDMR0002−53581
Estimated %CA
30
25
20
15
10
5
0
0
200
400
600
800
1000
Number of point intercepts
Cenchrus ciliaris
Bauhinia gilva
Triodia pungens
Fimbristylis dichotoma
Aristida inaequiglumis

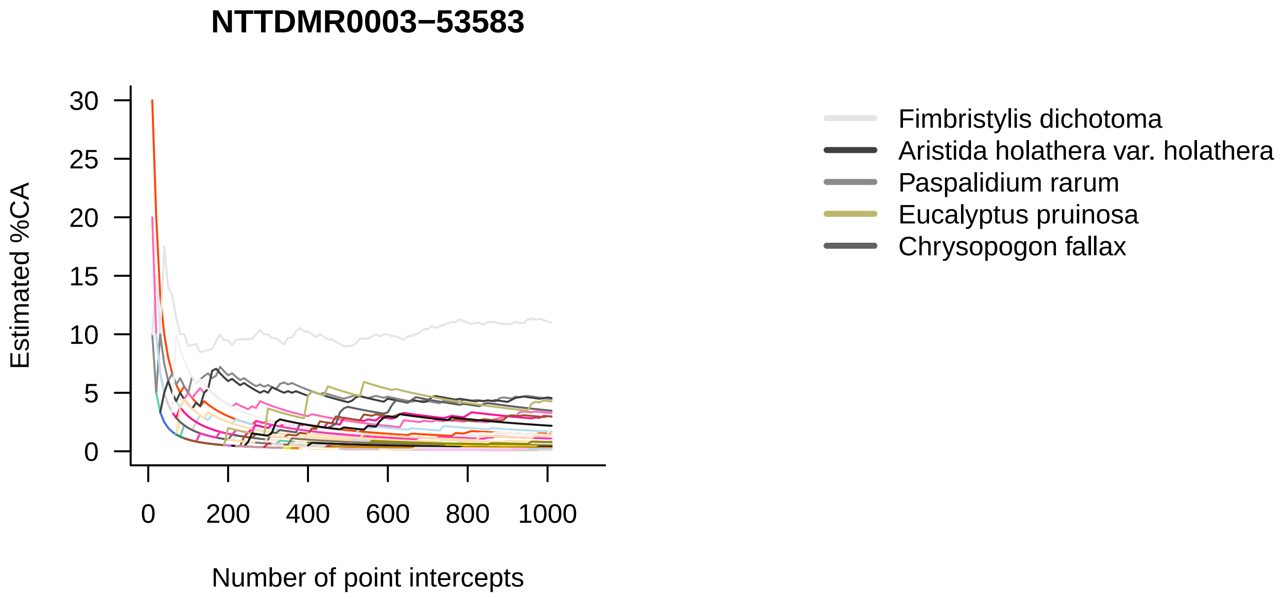
NTTDMR0003−53583
Estimated %CA
30
25
20
15
10
5
0
0
200
400
600
800
1000
Number of point intercepts
Fimbristylis dichotoma
Aristida holathera var. holathera
Paspalidium rarum
Eucalyptus pruinosa
Chrysopogon fallax

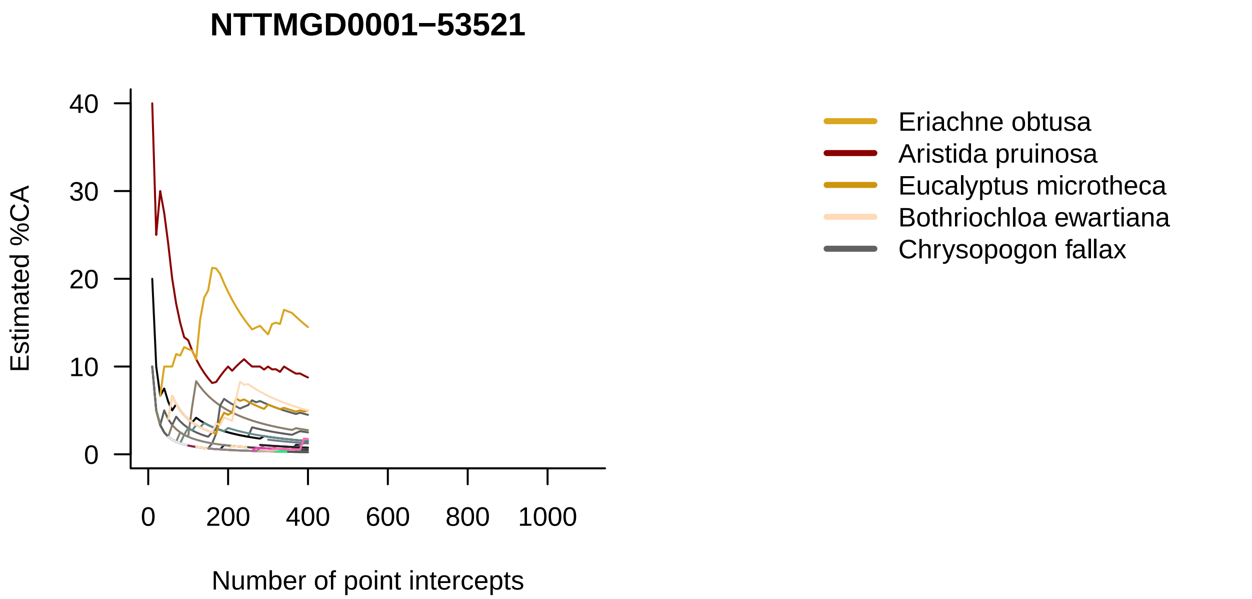
NTTMGD0001−53521
Estimated %CA
0
10
20
30
40
0
200
400
600
800
1000
Number of point intercepts
Eriachne obtusa
Aristida pruinosa
Eucalyptus microtheca
Bothriochloa ewartiana
Chrysopogon fallax

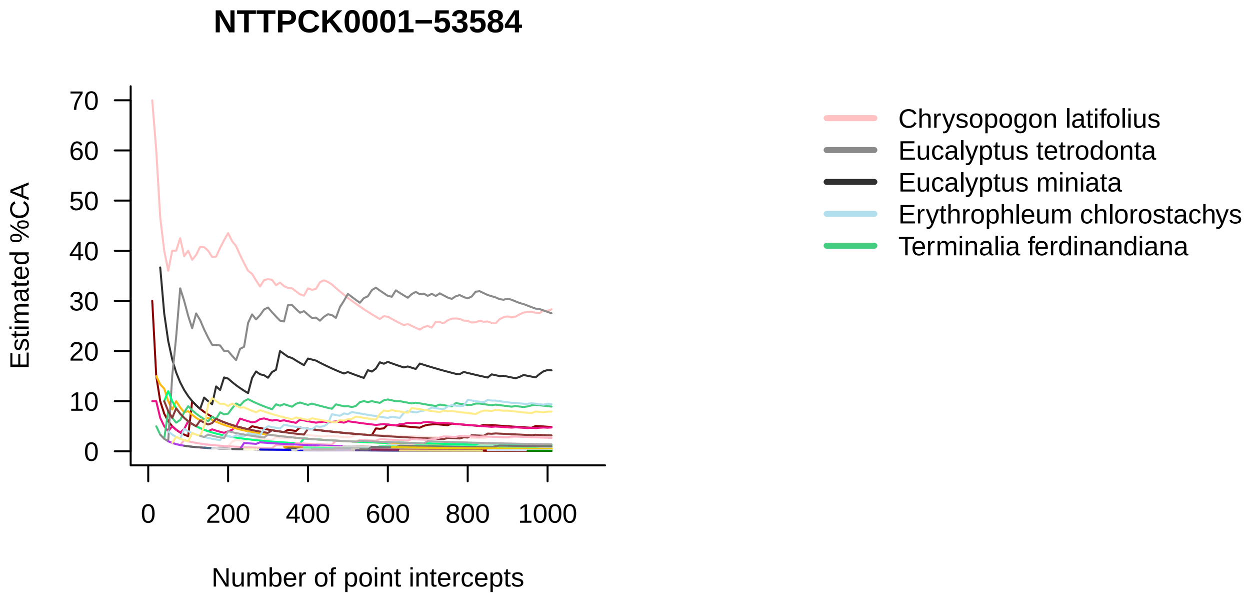
NTTPCK0001−53584
Estimated %CA
0
10
20
30
40
50
60
70
0
200
400
600
800
1000
Number of point intercepts
Chrysopogon latifolius
Eucalyptus tetrodonta
Eucalyptus miniata
Erythrophleum chlorostachys
Terminalia ferdinandiana

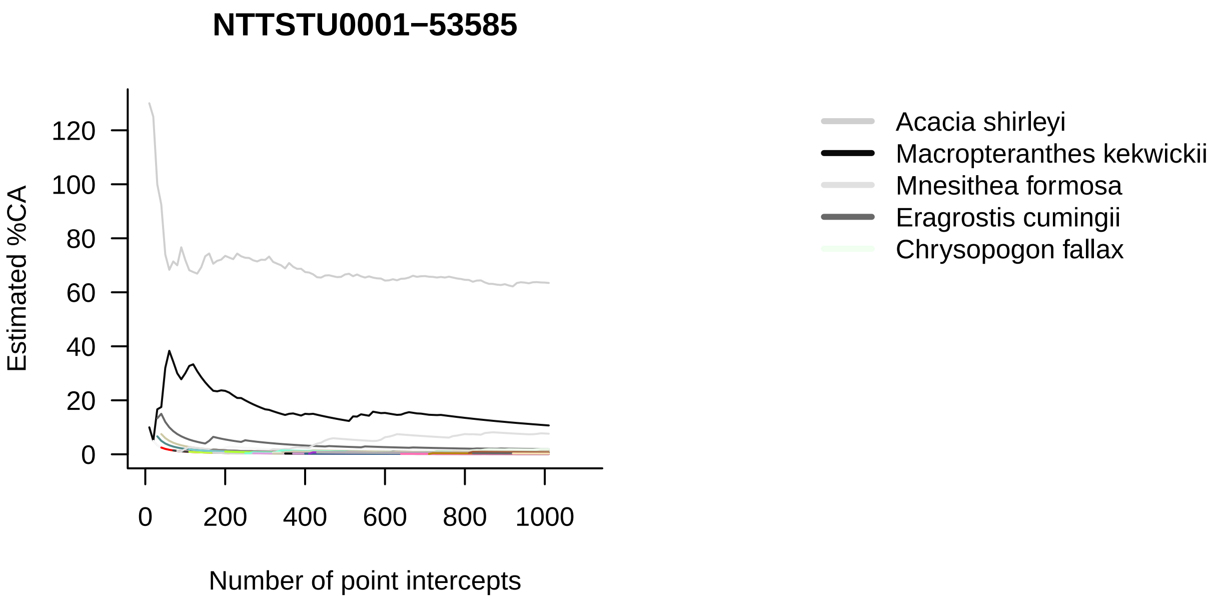
NTTSTU0001−53585
Estimated %CA
0
20
40
60
80
100
120
0
200
400
600
800
1000
Number of point intercepts
Acacia shirleyi
Macropteranthes kekwickii
Mnesithea formosa
Eragrostis cumingii
Chrysopogon fallax

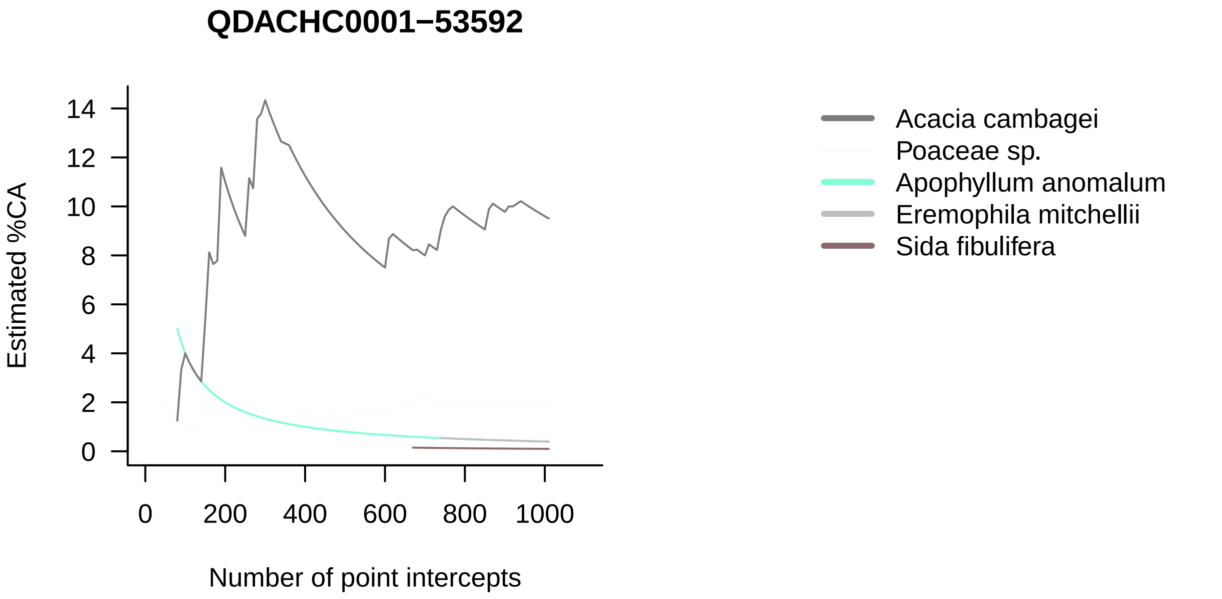
QDACHC0001−53592
Estimated %CA
0
2
4
6
8
10
12
14
0
200
400
600
800
1000
Number of point intercepts
Acacia cambagei
Poaceae sp.
Apophyllum anomalum
Eremophila mitchellii
Sida fibulifera

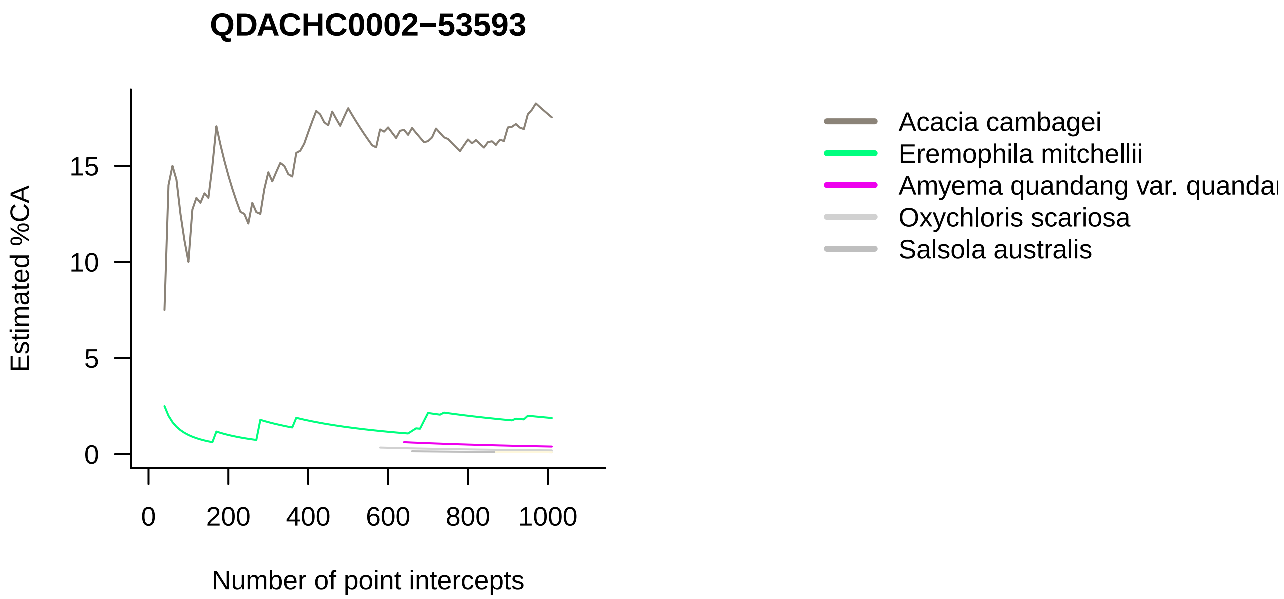
QDACHC0002−53593
Estimated %CA
0
5
10
15
0
200
400
600
800
1000
Number of point intercepts
Acacia cambagei
Eremophila mitchellii
Amyema quandang var. quandar
Oxychloris scariosa
Salsola australis

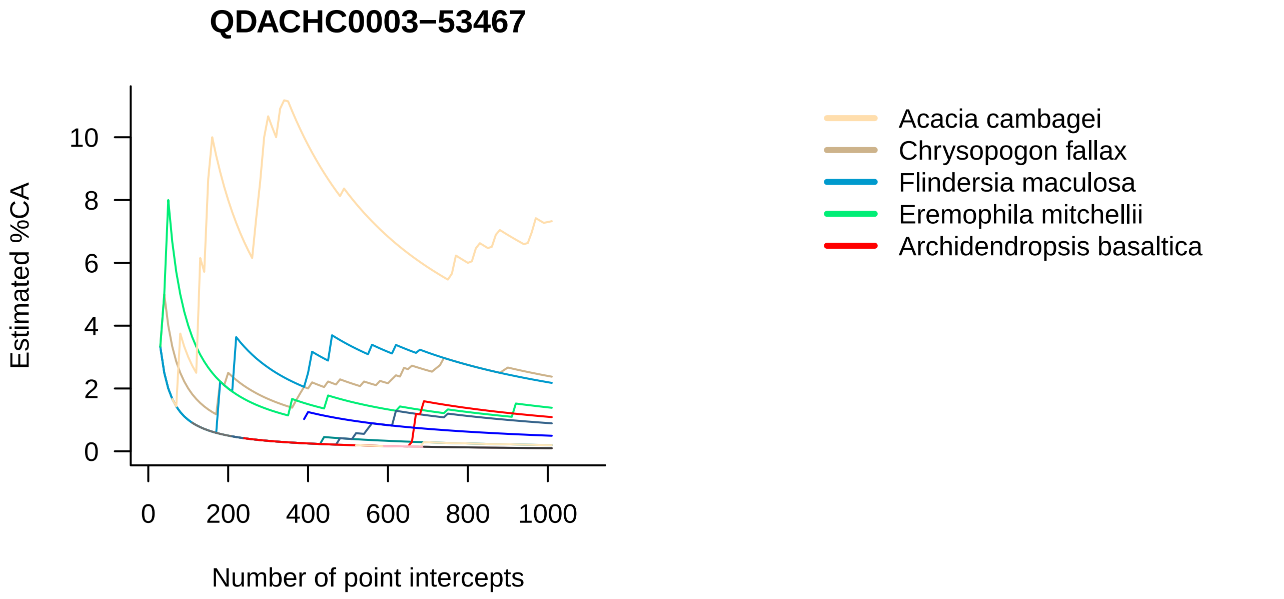
QDACHC0003−53467
Estimated %CA
0
2
4
6
8
10
0
200
400
600
800
1000
Number of point intercepts
Acacia cambagei
Chrysopogon fallax
Flindersia maculosa
Eremophila mitchellii
Archidendropsis basaltica

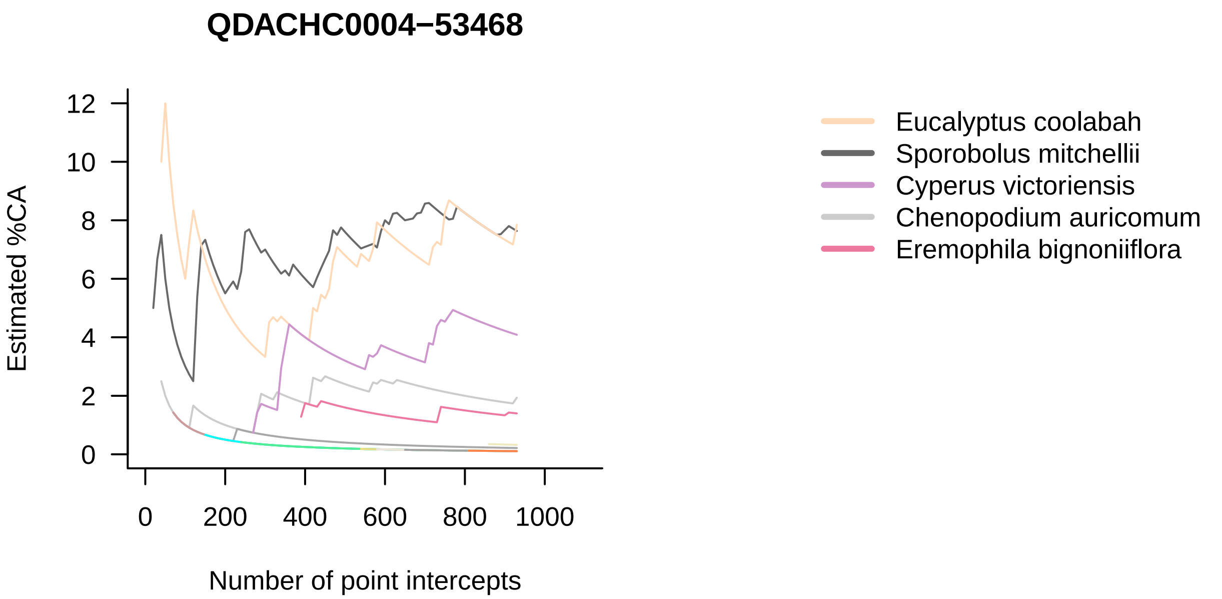
QDACHC0004−53468
Estimated %CA
Number of point intercepts
0
2
4
6
8
10
12
0
200
400
600
800
1000
Eucalyptus coolabah
Sporobolus mitchellii
Cyperus victoriensis
Chenopodium auricomum
Eremophila bignoniiflora

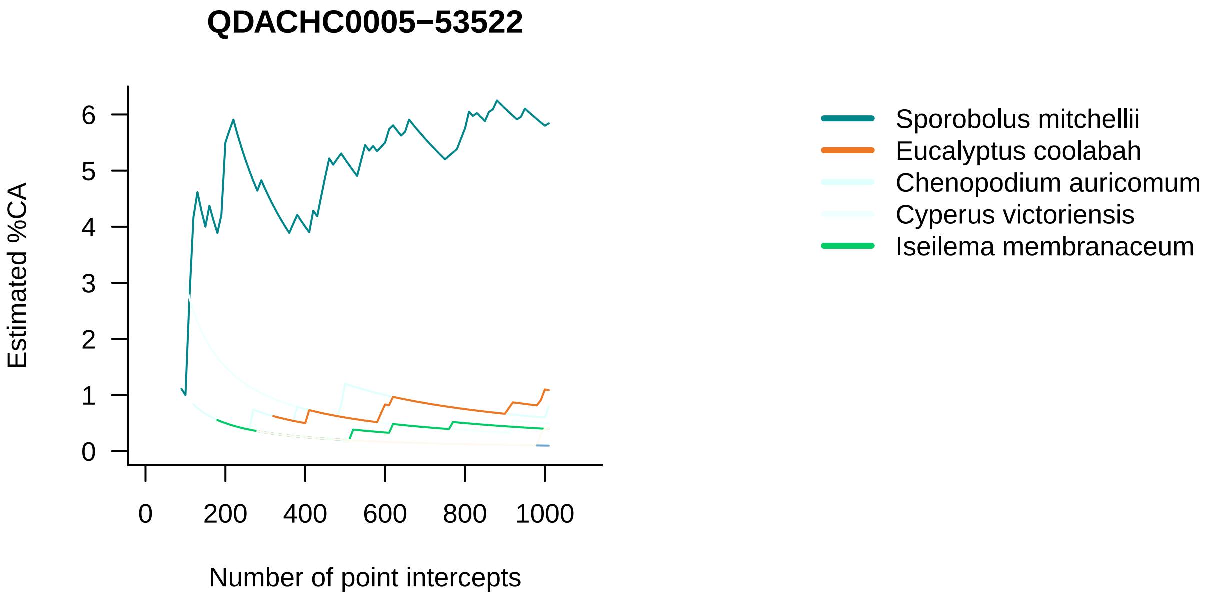
QDACHC0005−53522
Estimated %CA
Number of point intercepts
0
1
2
3
4
5
6
0
200
400
600
800
1000
Sporobolus mitchellii
Eucalyptus coolabah
Chenopodium auricomum
Cyperus victoriensis
Iseilema membranaceum

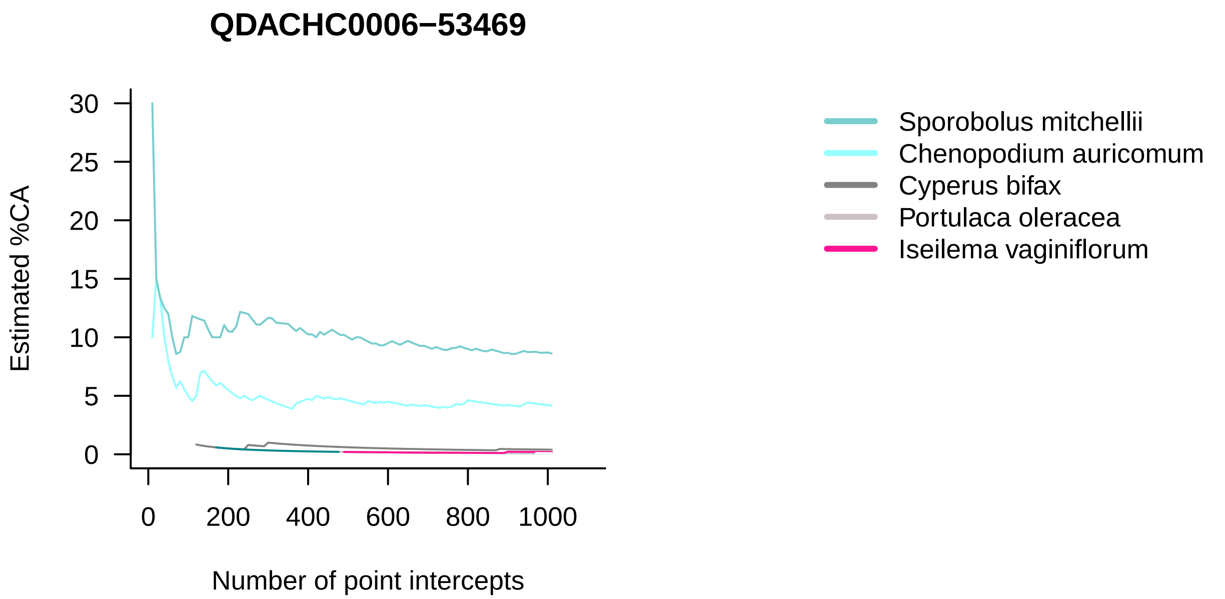
QDACHC0006−53469
Estimated %CA
30
25
20
15
10
5
0
0
200
400
600
800
1000
Number of point intercepts
Sporobolus mitchellii
Chenopodium auricomum
Cyperus bifax
Portulaca oleracea
Iseilema vaginiflorum

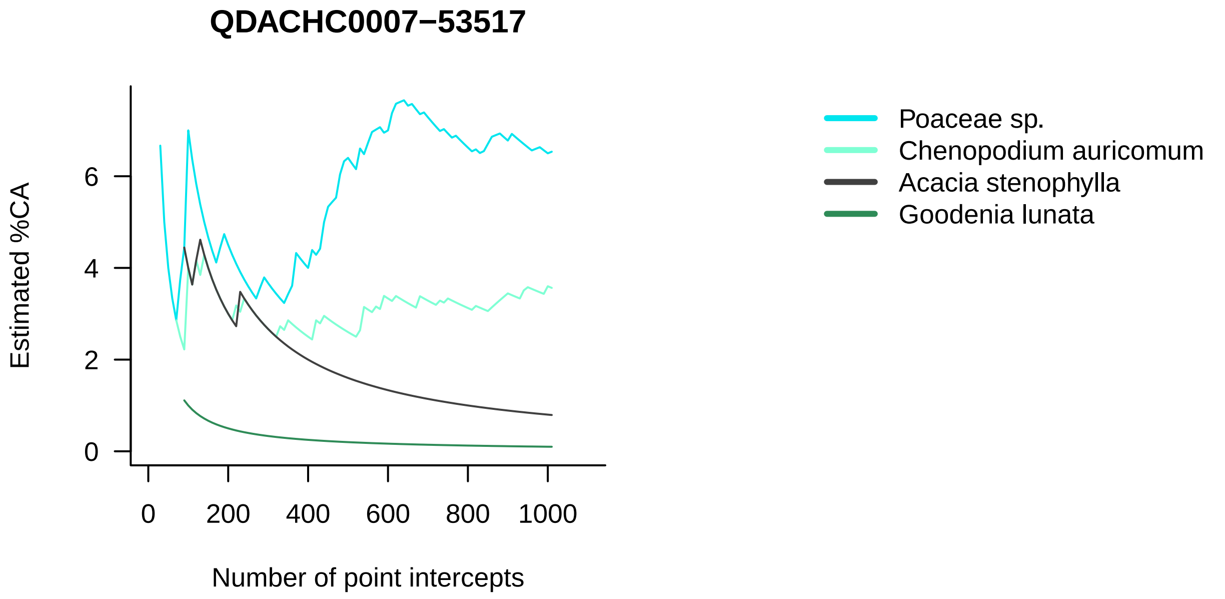
QDACHC0007−53517
Estimated %CA
6
4
2
0
0
200
400
600
800
1000
Number of point intercepts
Poaceae sp.
Chenopodium auricomum
Acacia stenophylla
Goodenia lunata

## QDAEIU0001−53470

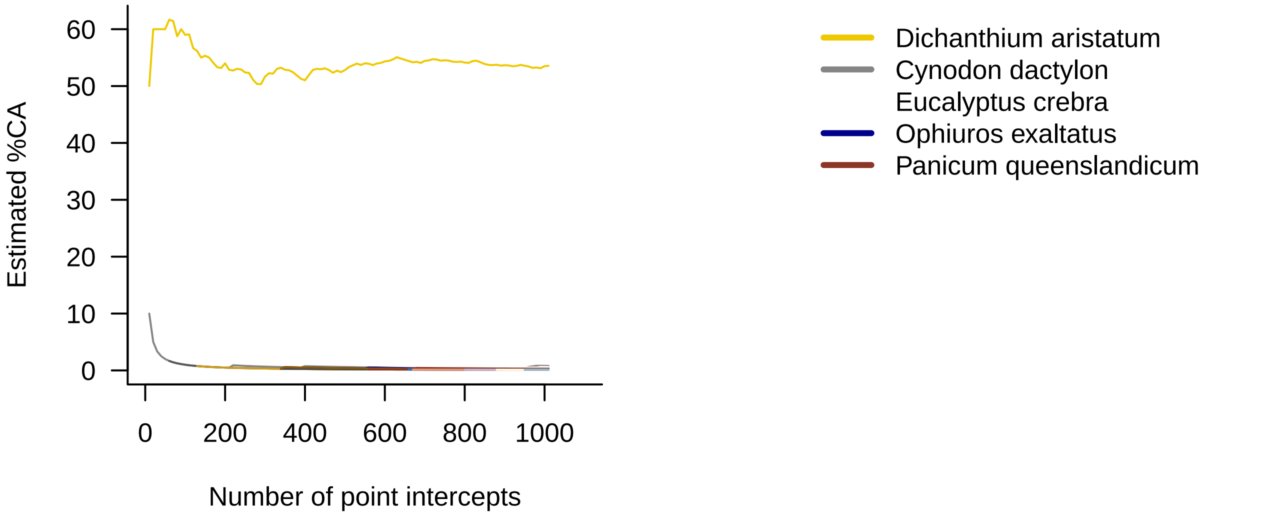


## QDAEIU0002−53523

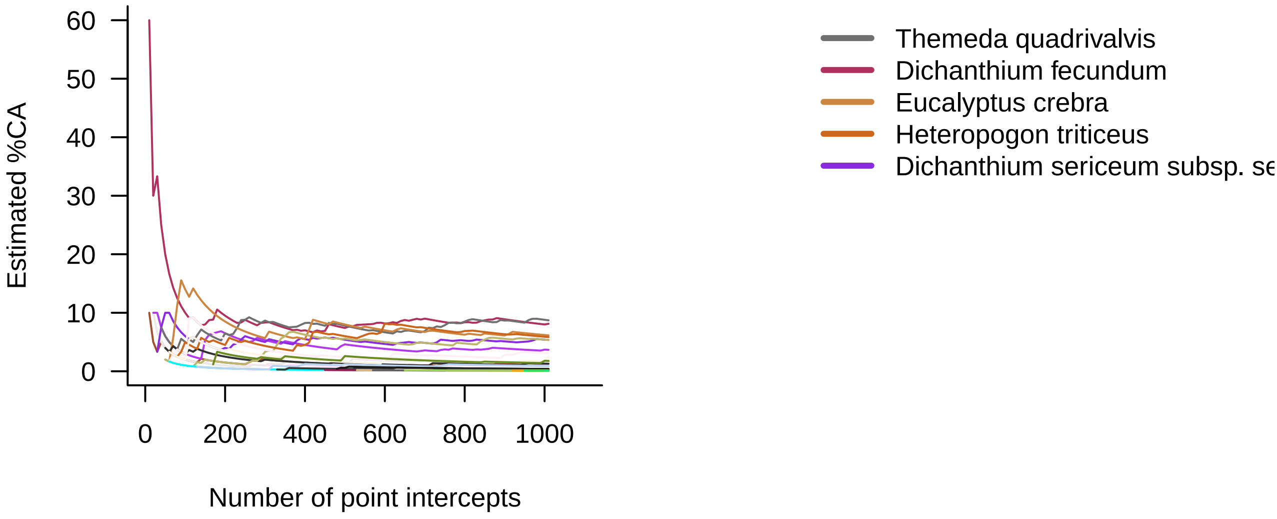

## QDAEIU0003−53471

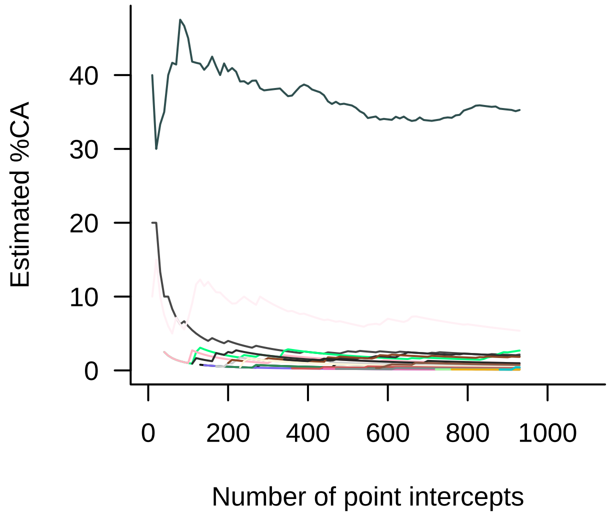


Heteropogon contortus
Themeda triandra
Heteropogon triticeus
Melinis repens
Eucalyptus crebra

## QDAEIU0004−53524

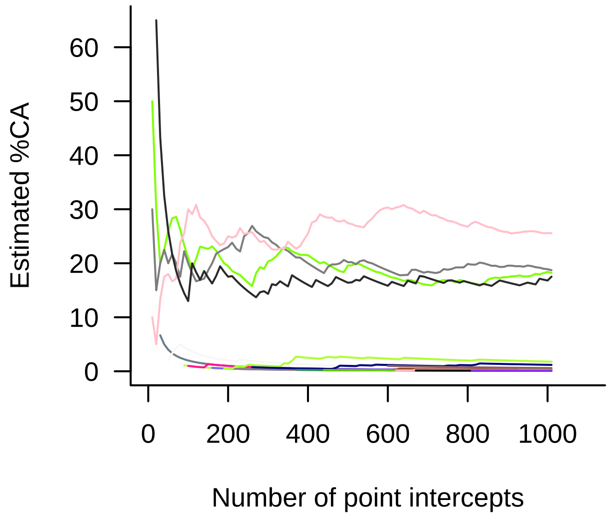


Heteropogon triticeus
Themeda triandra
Capillipedium parviflorum
Eucalyptus crebra
Poaceae sp.

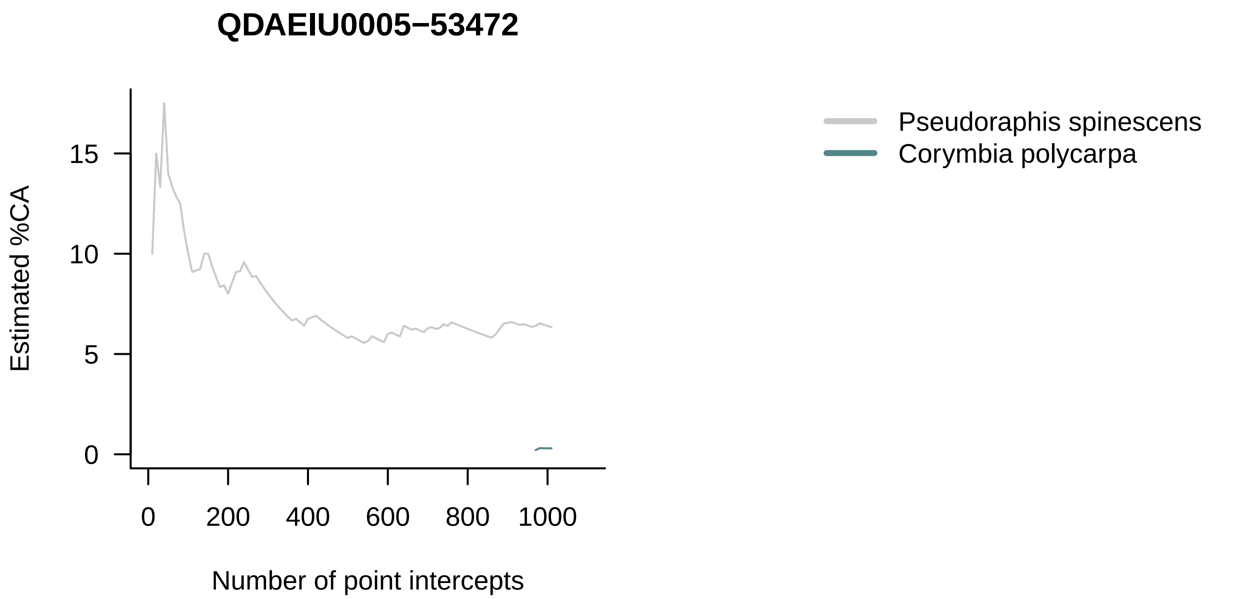
QDAEIU0005−53472
Estimated %CA
Number of point intercepts
Pseudoraphis spinescens
Corymbia polycarpa

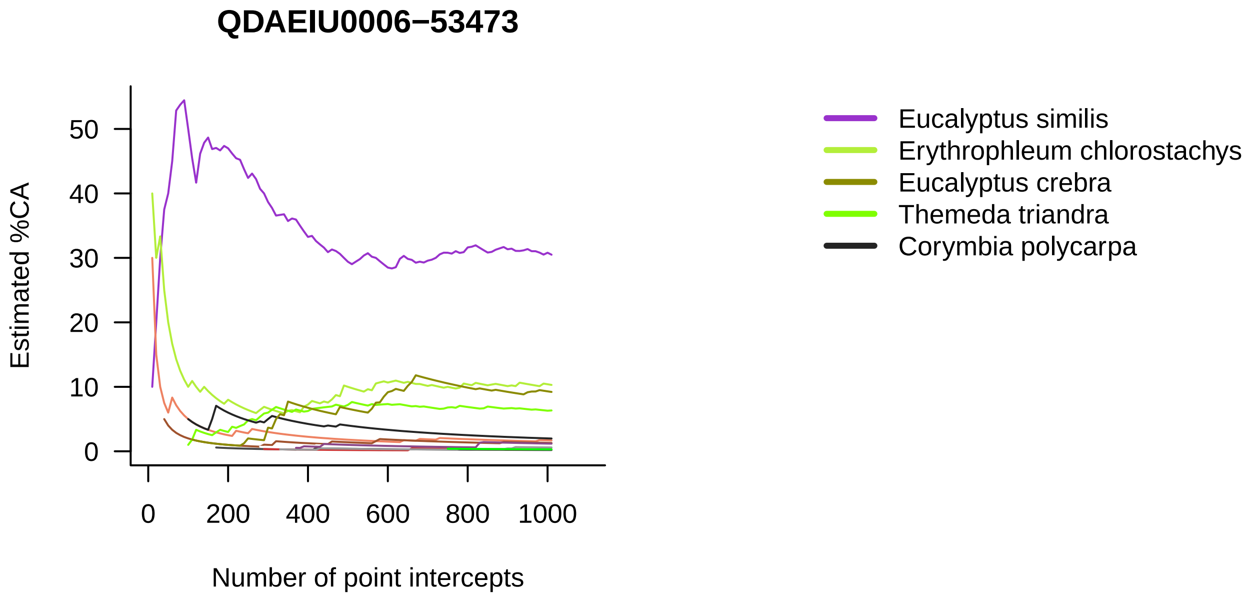
QDAEIU0006−53473
Estimated %CA
Number of point intercepts
Eucalyptus similis
Erythrophleum chlorostachys
Eucalyptus crebra
Themeda triandra
Corymbia polycarpa

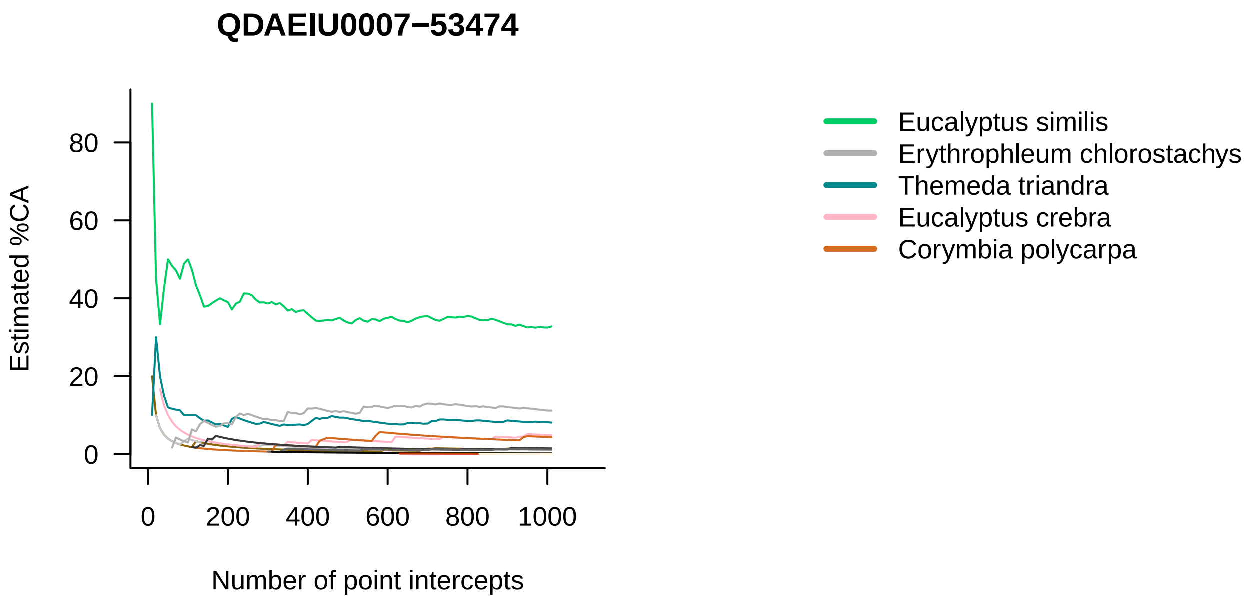
QDAEIU0007−53474
Estimated %CA
0
20
40
60
80
0
200
400
600
800
1000
Number of point intercepts
Eucalyptus similis
Erythrophleum chlorostachys
Themeda triandra
Eucalyptus crebra
Corymbia polycarpa

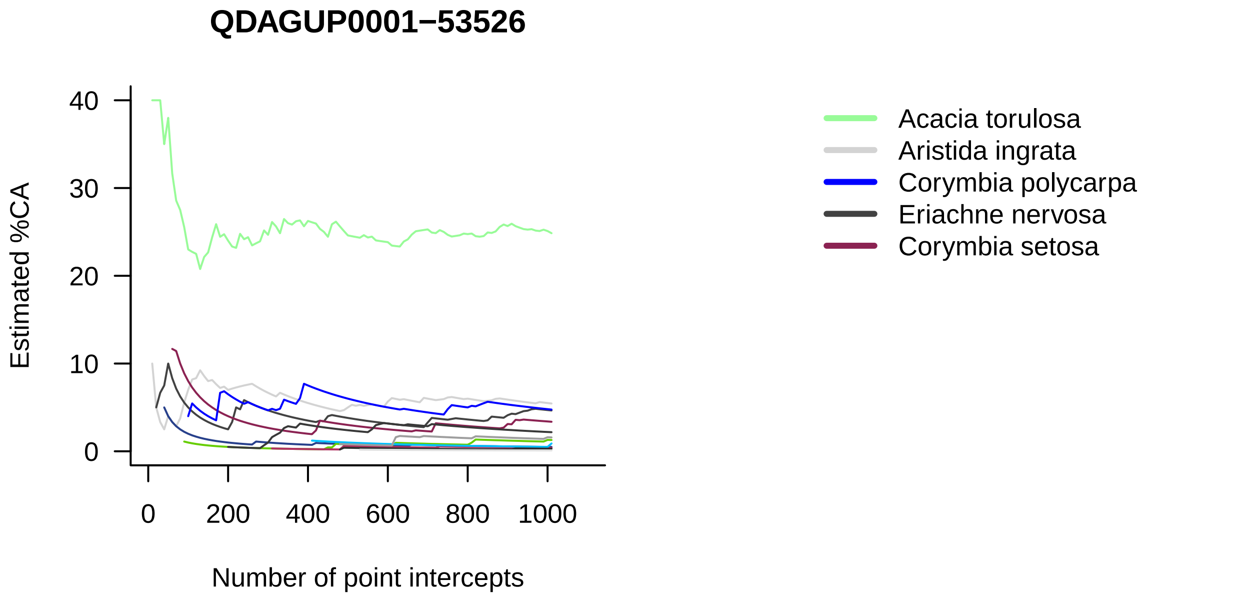
QDAGUP0001−53526
Estimated %CA
0
10
20
30
40
0
200
400
600
800
1000
Number of point intercepts
Acacia torulosa
Aristida ingrata
Corymbia polycarpa
Eriachne nervosa
Corymbia setosa

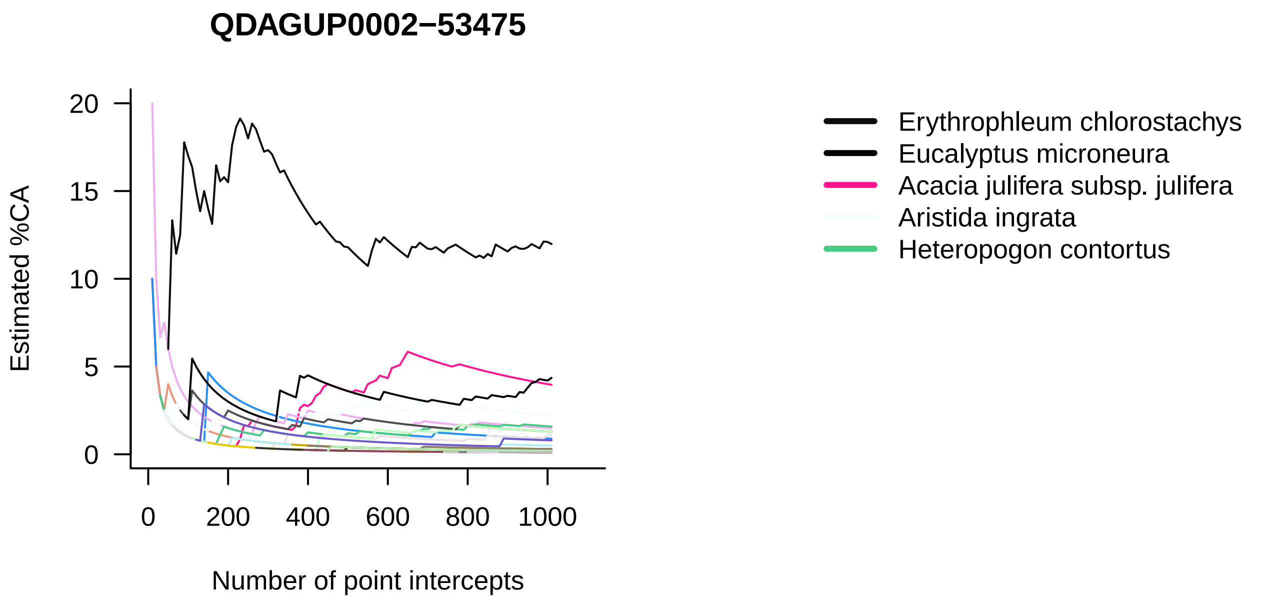
QDAGUP0002−53475
Estimated %CA
0
5
10
15
20
0
200
400
600
800
1000
Number of point intercepts
Erythrophleum chlorostachys
Eucalyptus microneura
Acacia julifera subsp. julifera
Aristida ingrata
Heteropogon contortus

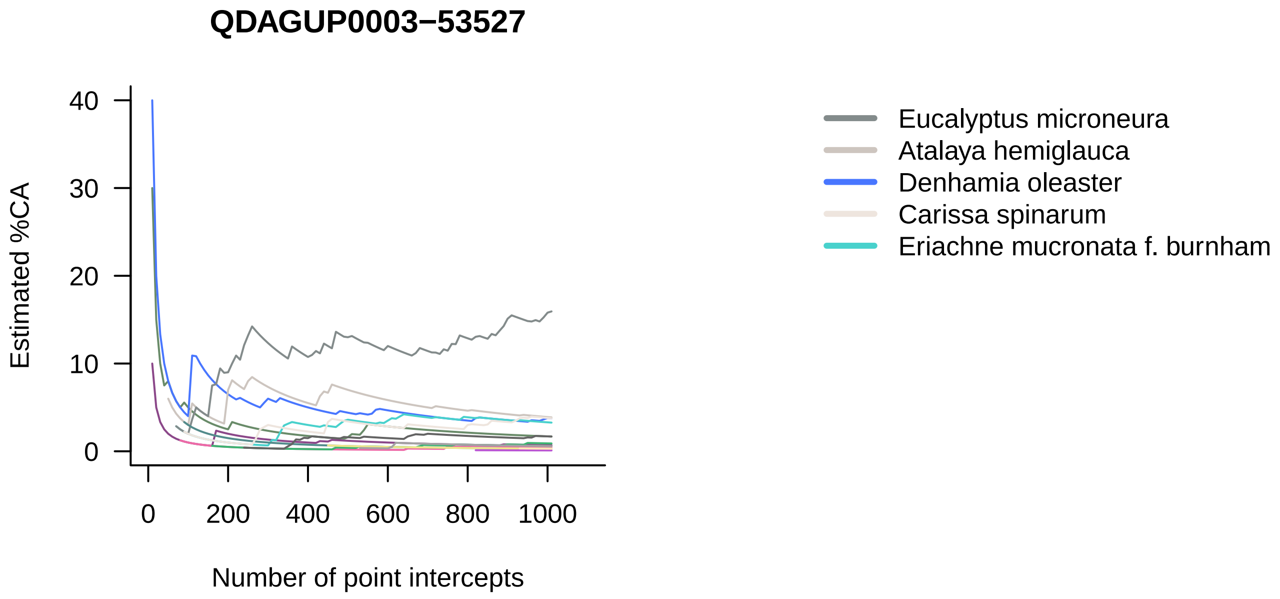
QDAGUP0003−53527
Estimated %CA
0
10
20
30
40
0
200
400
600
800
1000
Number of point intercepts
Eucalyptus microneura
Atalaya hemiglauca
Denhamia oleaster
Carissa spinarum
Eriachne mucronata f. burnham

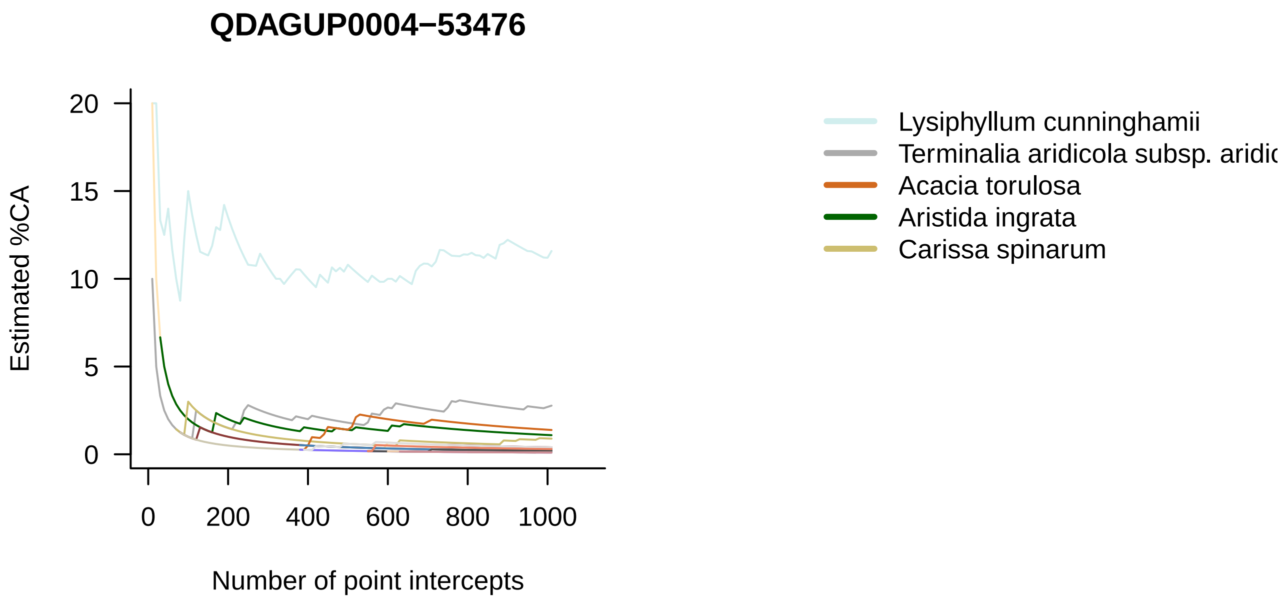
QDAGUP0004−53476
Estimated %CA
Number of point intercepts
0
5
10
15
20
0
200
400
600
800
1000
Lysiphyllum cunninghamii
Terminalia aridicola subsp. aridic
Acacia torulosa
Aristida ingrata
Carissa spinarum

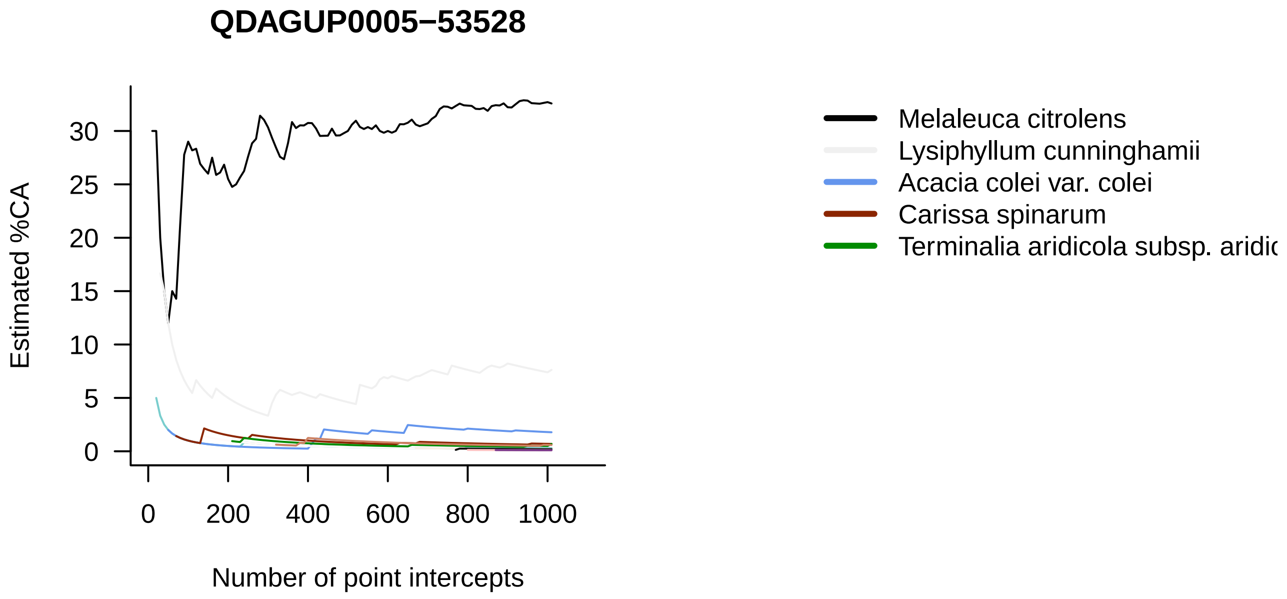
QDAGUP0005−53528
Estimated %CA
Number of point intercepts
0
5
10
15
20
25
30
0
200
400
600
800
1000
Melaleuca citrolens
Lysiphyllum cunninghamii
Acacia colei var. colei
Carissa spinarum
Terminalia aridicola subsp. aridic

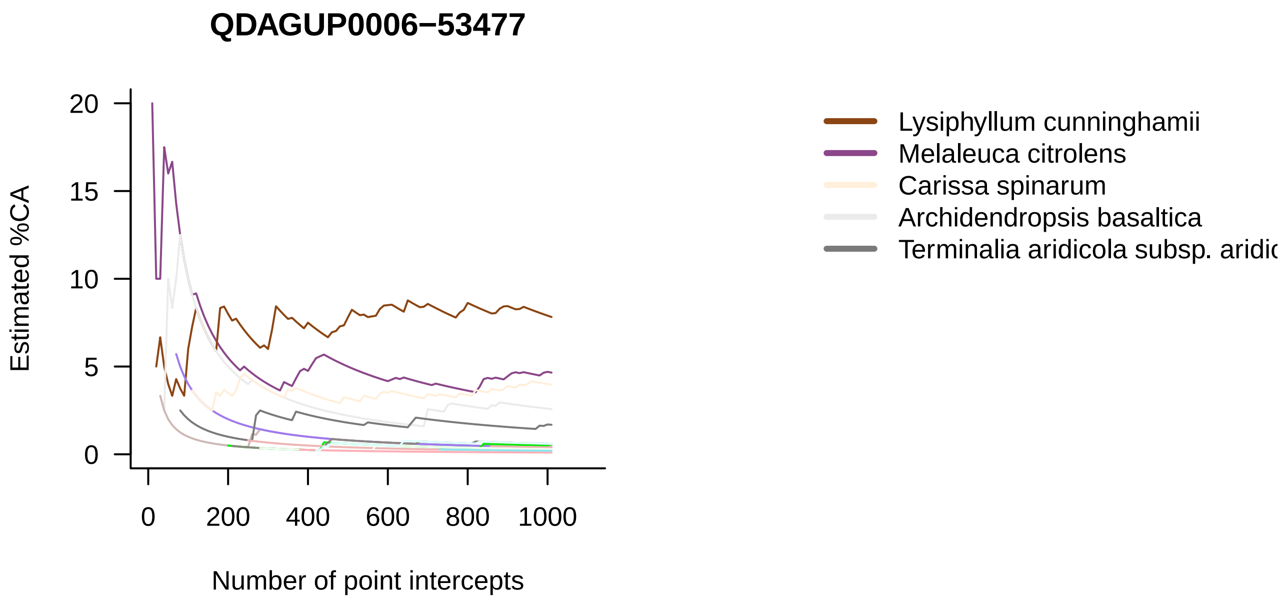
QDAGUP0006−53477
Estimated %CA
Number of point intercepts
0
5
10
15
20
0
200
400
600
800
1000
Lysiphyllum cunninghamii
Melaleuca citrolens
Carissa spinarum
Archidendropsis basaltica
Terminalia aridicola subsp. aridic

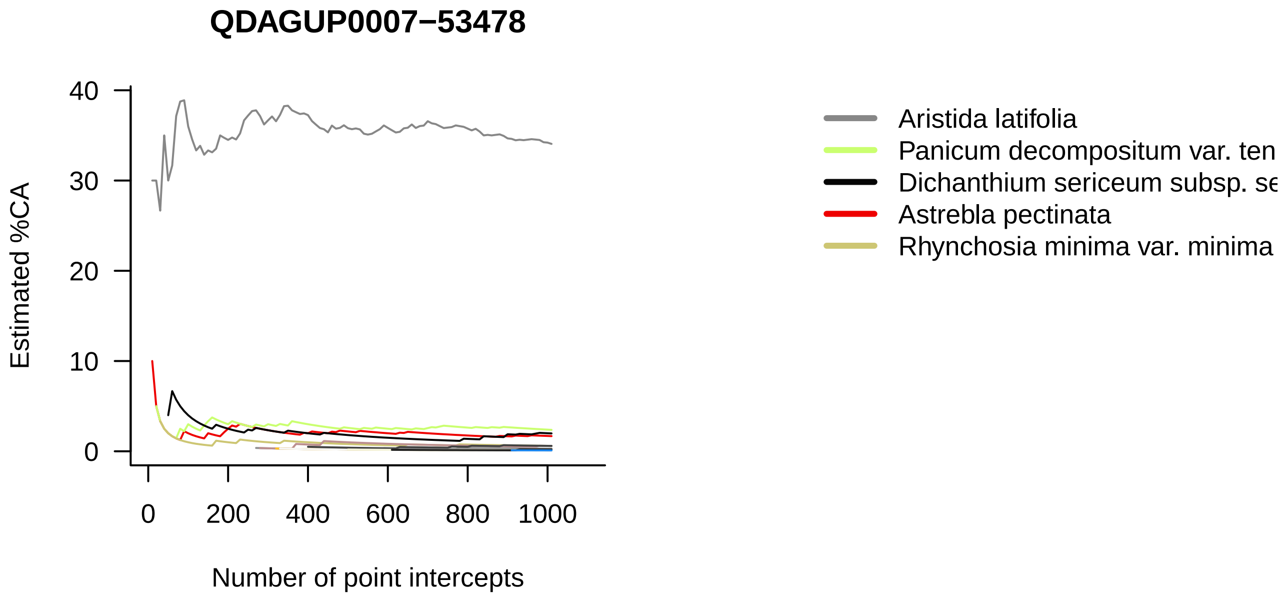
QDAGUP0007−53478
Estimated %CA
Number of point intercepts
0
10
20
30
40
0
200
400
600
800
1000
Aristida latifolia
Panicum decompositum var. ten
Dichanthium sericeum subsp. se
Astrebla pectinata
Rhynchosia minima var. minima

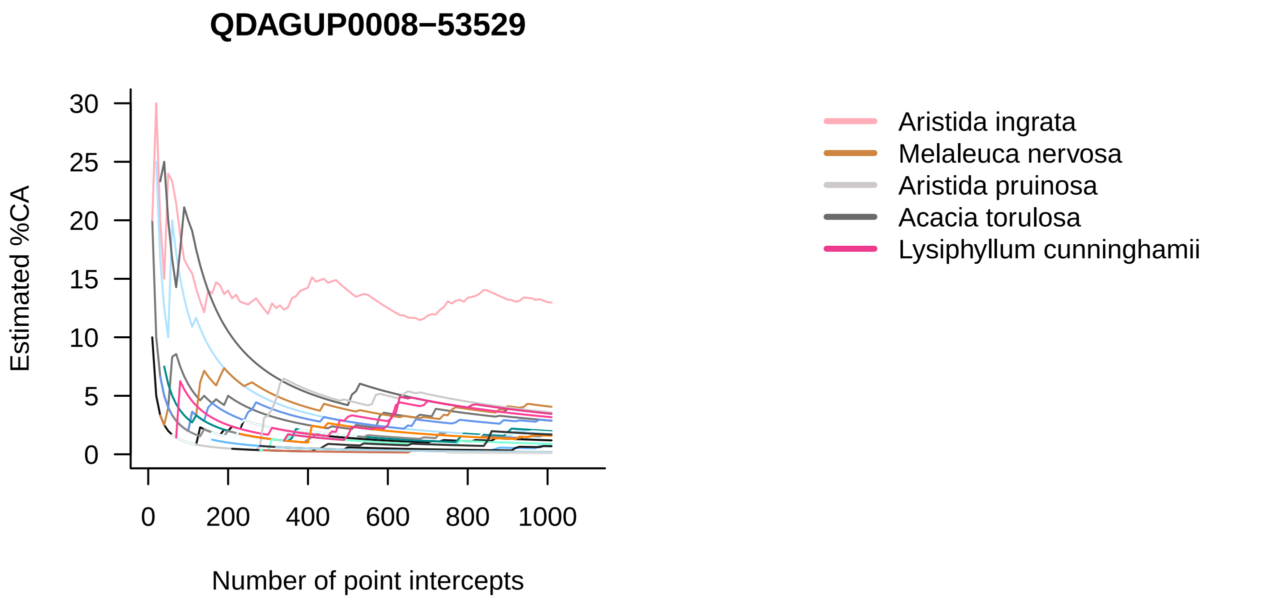
QDAGUP0008−53529
Estimated %CA
0
5
10
15
20
25
30
0
200
400
600
800
1000
Number of point intercepts
Aristida ingrata
Melaleuca nervosa
Aristida pruinosa
Acacia torulosa
Lysiphyllum cunninghamii

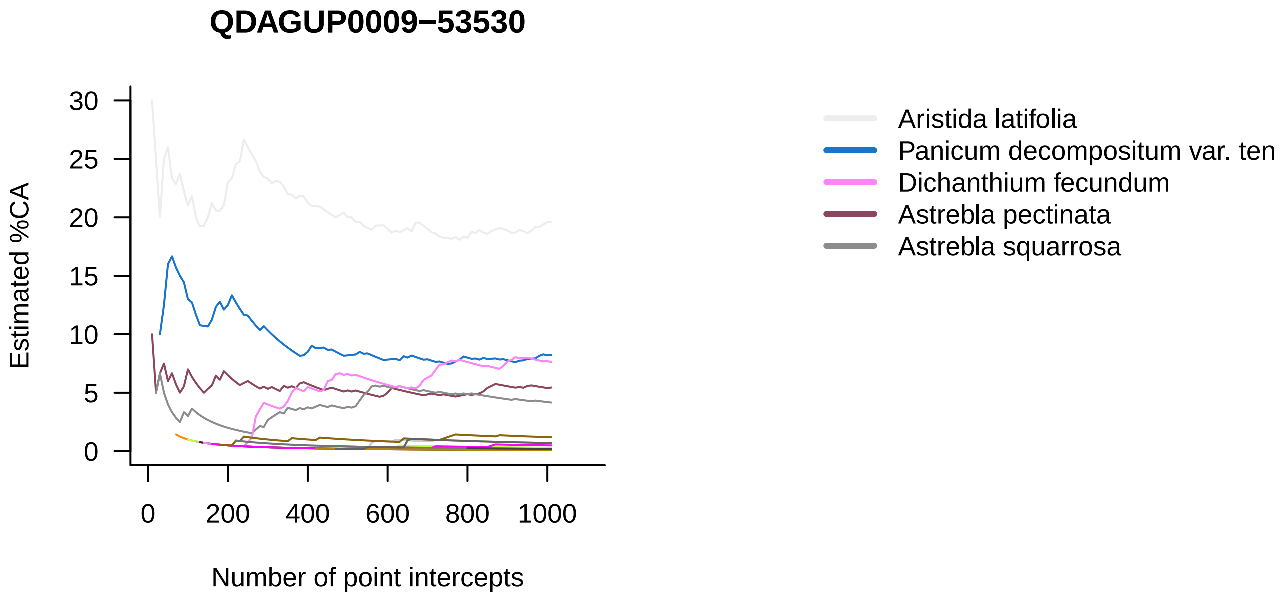
QDAGUP0009−53530
Estimated %CA
0
5
10
15
20
25
30
0
200
400
600
800
1000
Number of point intercepts
Aristida latifolia
Panicum decompositum var. ten
Dichanthium fecundum
Astrebla pectinata
Astrebla squarrosa

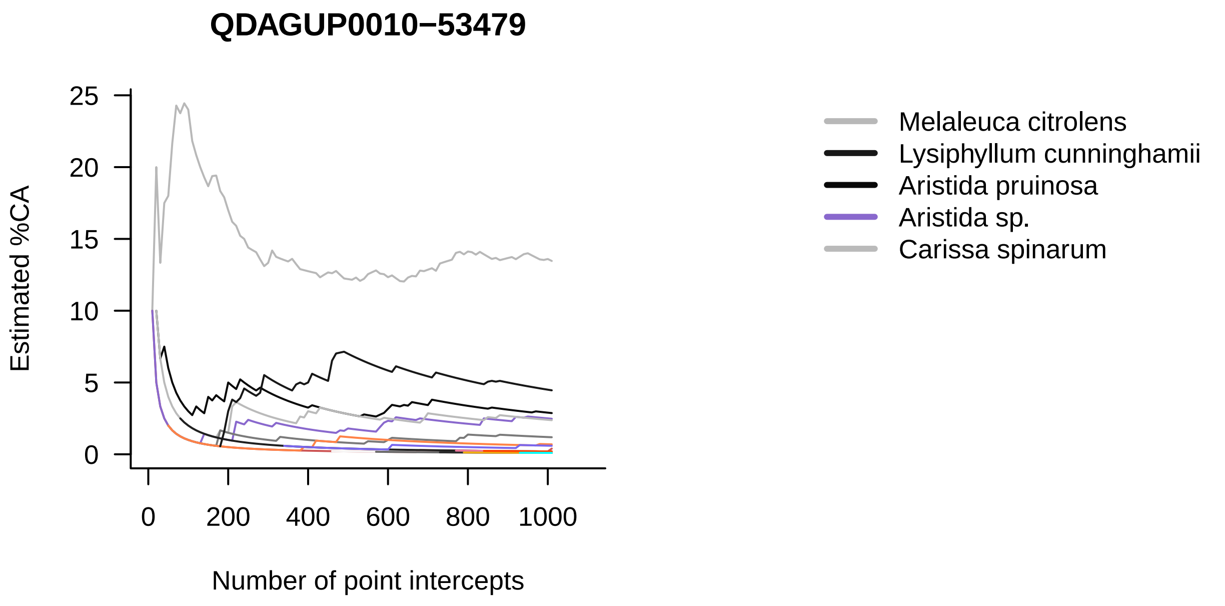
QDAGUP0010−53479
Estimated %CA
Number of point intercepts
0
5
10
15
20
25
0
200
400
600
800
1000
Melaleuca citrolens
Lysiphyllum cunninghamii
Aristida pruinosa
Aristida sp.
Carissa spinarum

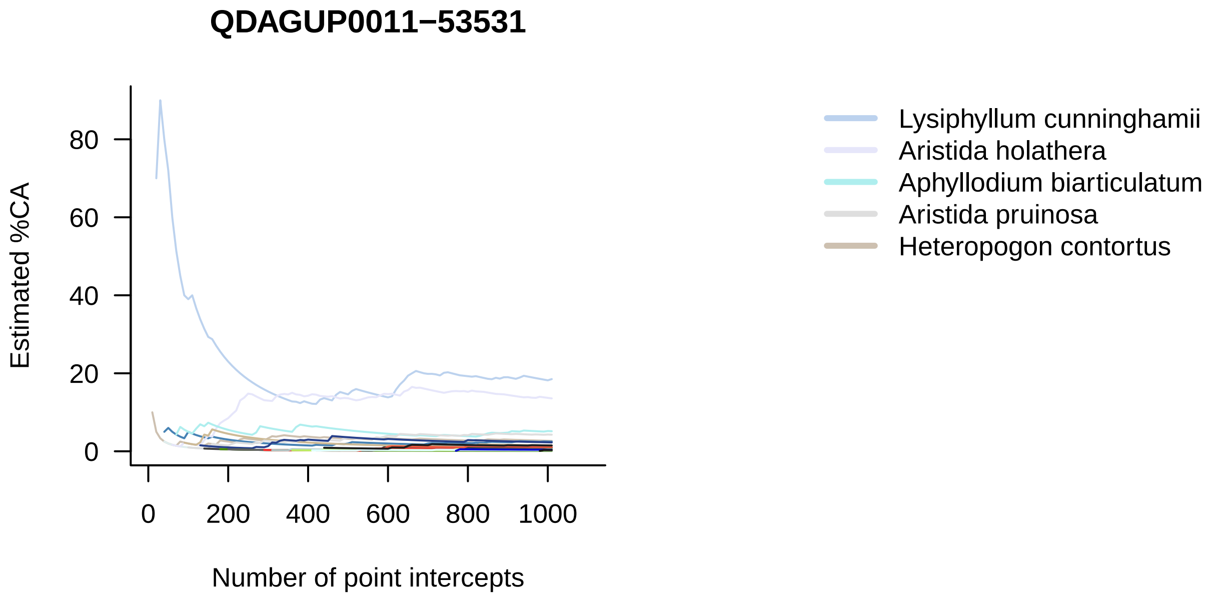
QDAGUP0011−53531
Estimated %CA
Number of point intercepts
0
20
40
60
80
0
200
400
600
800
1000
Lysiphyllum cunninghamii
Aristida holathera
Aphyllodium biarticulatum
Aristida pruinosa
Heteropogon contortus

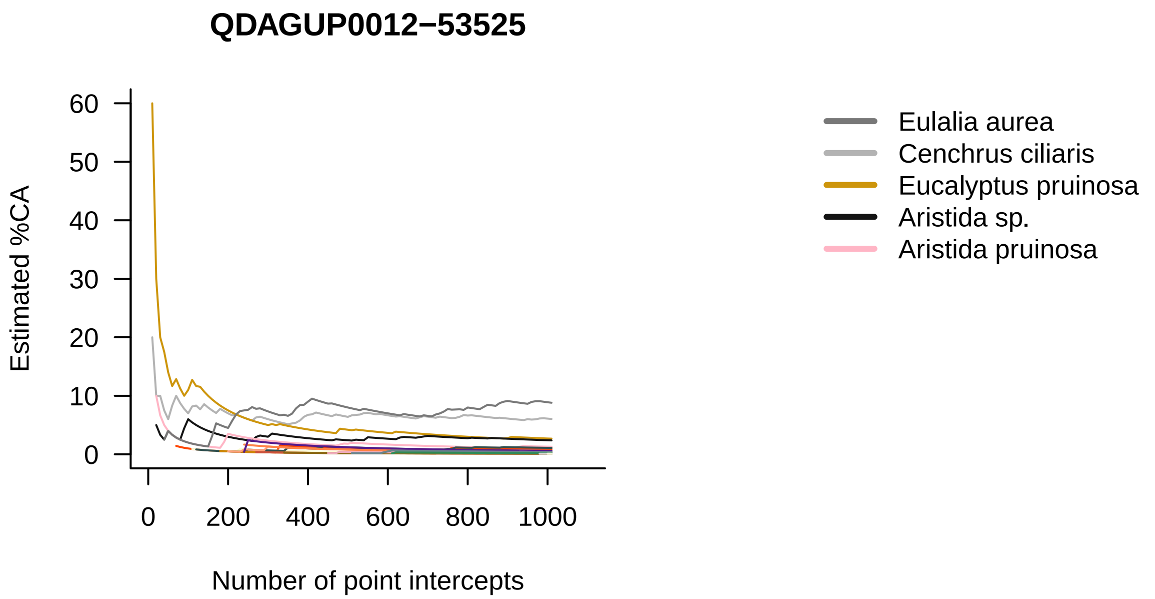
QDAGUP0012−53525
Estimated %CA
Number of point intercepts
0
10
20
30
40
50
60
0
200
400
600
800
1000
Eulalia aurea
Cenchrus ciliaris
Eucalyptus pruinosa
Aristida sp.
Aristida pruinosa

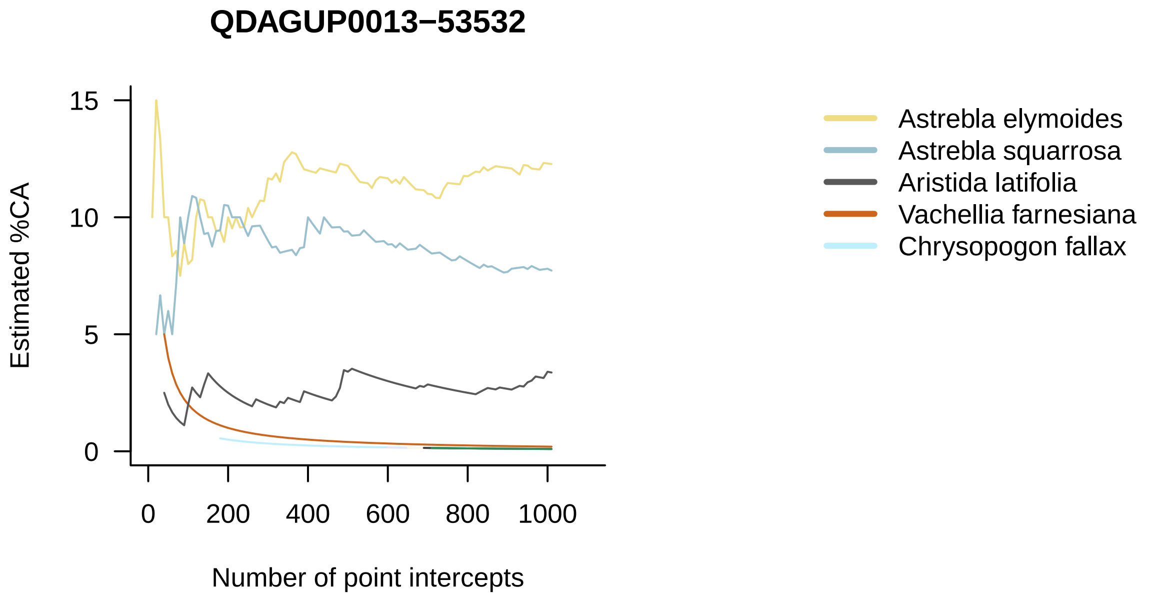
QDAGUP0013−53532
Estimated %CA
Number of point intercepts
0
5
10
15
0
200
400
600
800
1000
Astrebla elymoides
Astrebla squarrosa
Aristida latifolia
Vachellia farnesiana
Chrysopogon fallax

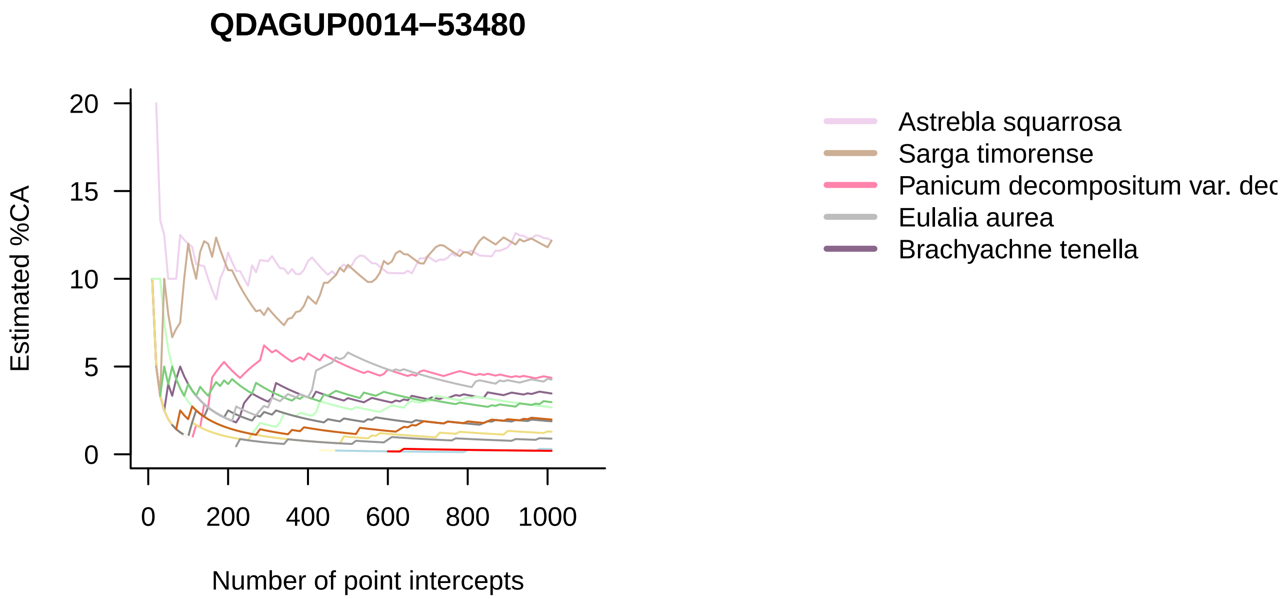
QDAGUP0014−53480
Estimated %CA
0
5
10
15
20
0
200
400
600
800
1000
Number of point intercepts
Astrebla squarrosa
Sarga timorense
Panicum decompositum var. dec
Eulalia aurea
Brachyachne tenella

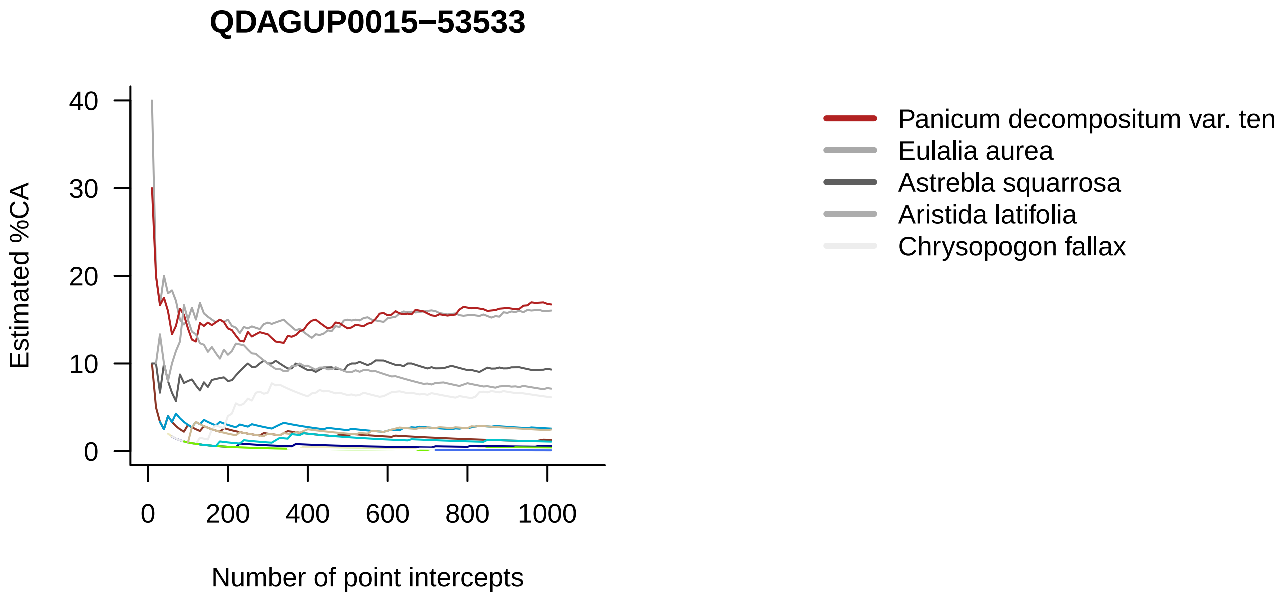
QDAGUP0015−53533
Estimated %CA
0
10
20
30
40
0
200
400
600
800
1000
Number of point intercepts
Panicum decompositum var. ten
Eulalia aurea
Astrebla squarrosa
Aristida latifolia
Chrysopogon fallax

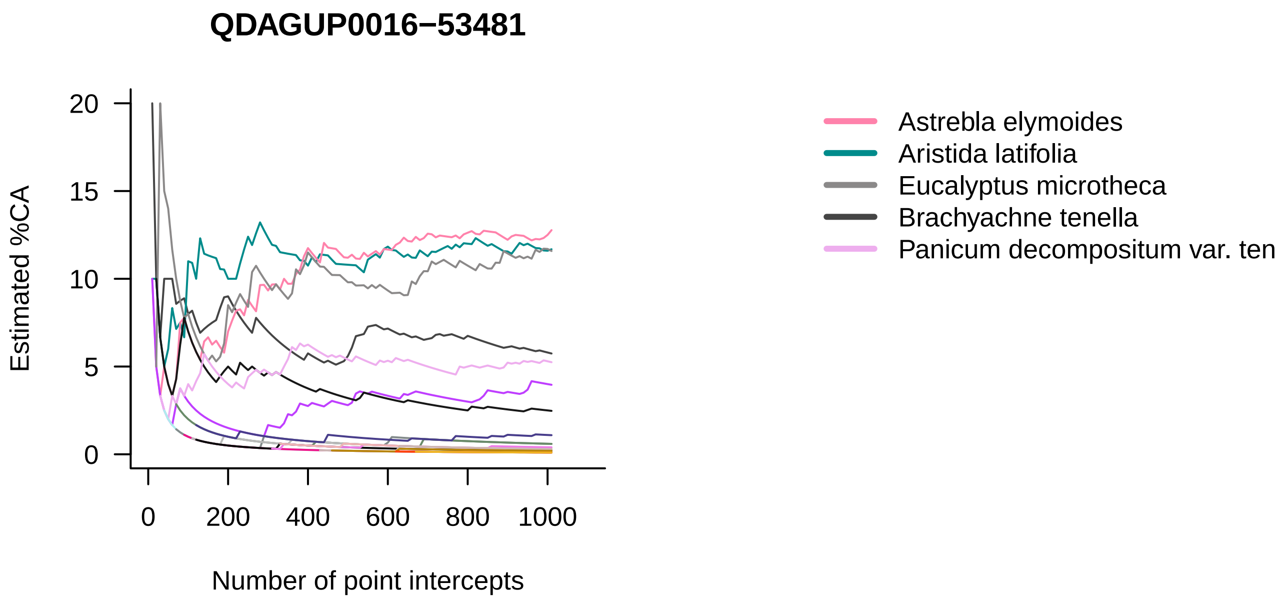
QDAGUP0016−53481
Estimated %CA
20
15
10
5
0
0
200
400
600
800
1000
Number of point intercepts
Astrebla elymoides
Aristida latifolia
Eucalyptus microtheca
Brachyachne tenella
Panicum decompositum var. ten

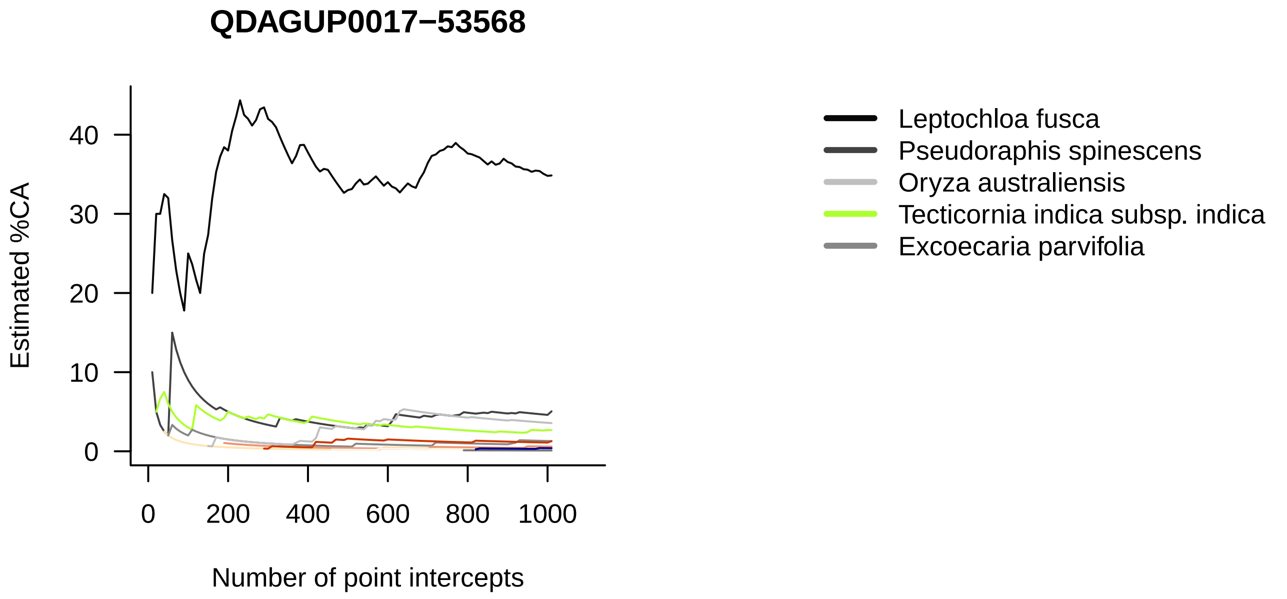
QDAGUP0017−53568
Estimated %CA
40
30
20
10
0
0
200
400
600
800
1000
Number of point intercepts
Leptochloa fusca
Pseudoraphis spinescens
Oryza australiensis
Tecticornia indica subsp. indica
Excoecaria parvifolia

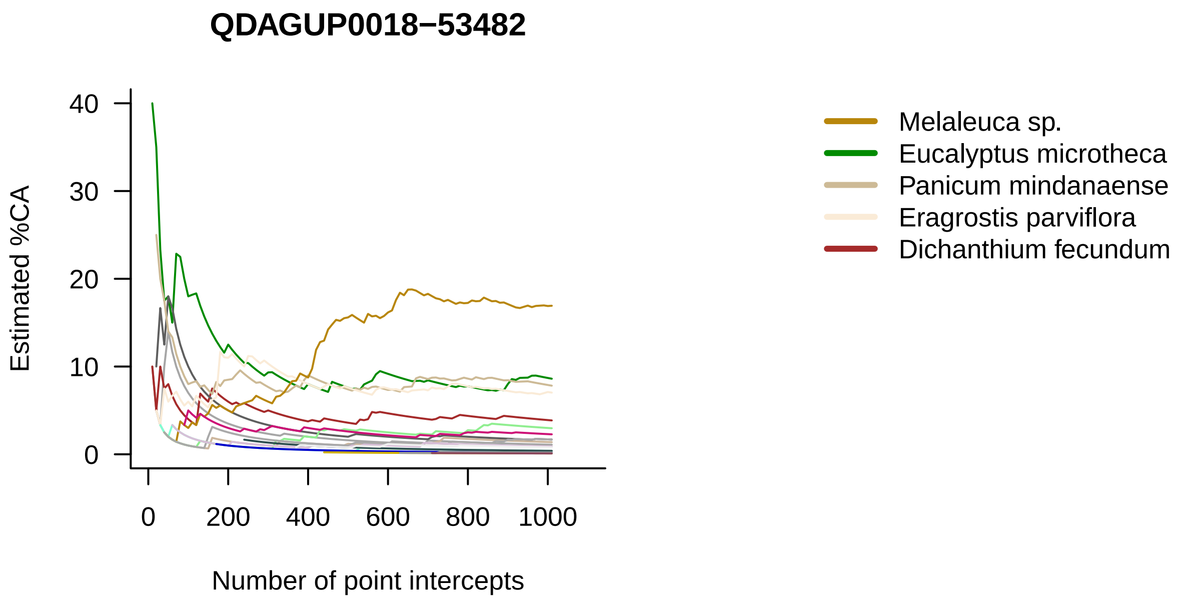
QDAGUP0018−53482
Estimated %CA
Number of point intercepts
0
10
20
30
40
0
200
400
600
800
1000
Melaleuca sp.
Eucalyptus microtheca
Panicum mindanaense
Eragrostis parviflora
Dichanthium fecundum

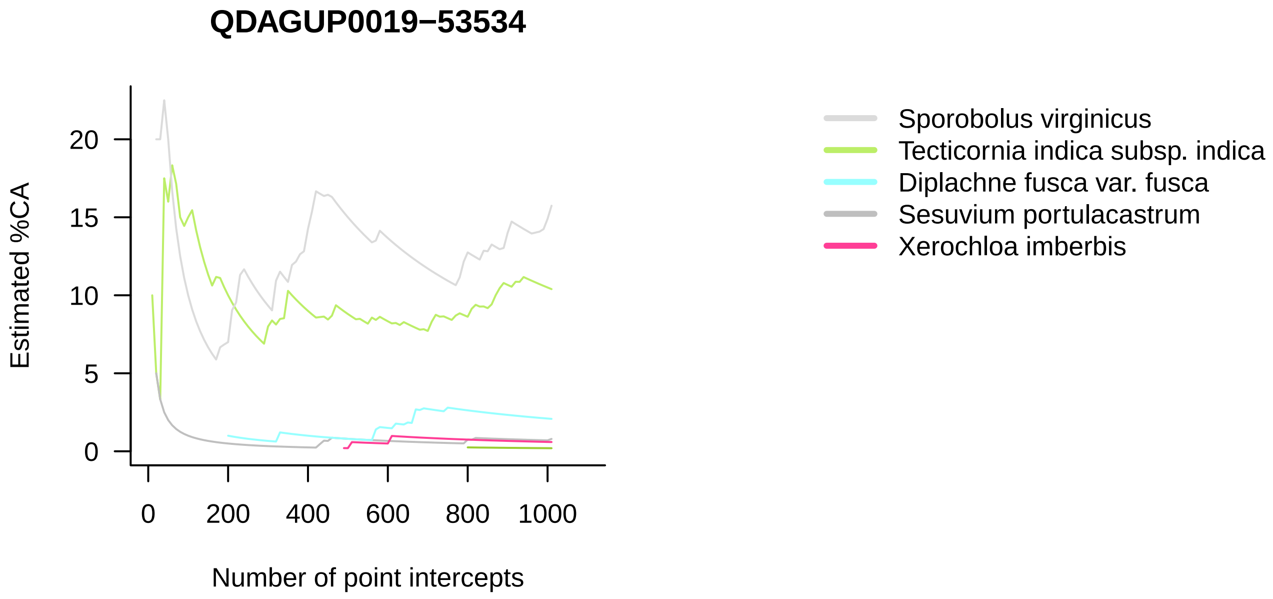
QDAGUP0019−53534
Estimated %CA
Number of point intercepts
0
5
10
15
20
0
200
400
600
800
1000
Sporobolus virginicus
Tecticornia indica subsp. indica
Diplachne fusca var. fusca
Sesuvium portulacastrum
Xerochloa imberbis

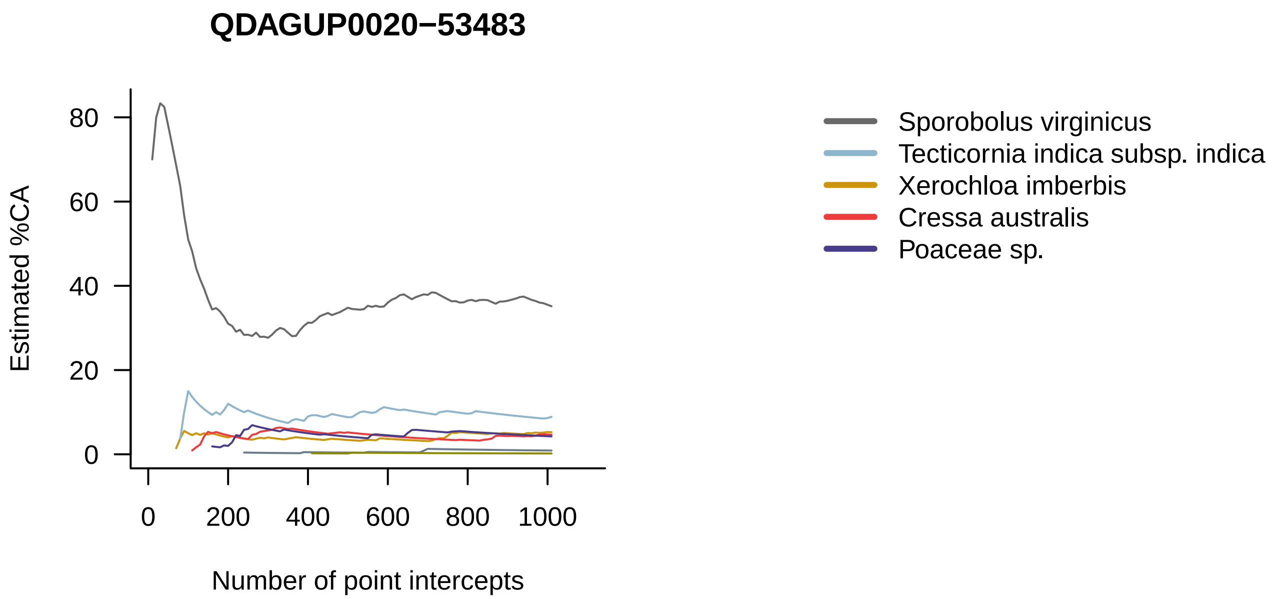
QDAGUP0020−53483
Estimated %CA
Number of point intercepts
0
20
40
60
80
0
200
400
600
800
1000
Sporobolus virginicus
Tecticornia indica subsp. indica
Xerochloa imberbis
Cressa australis
Poaceae sp.

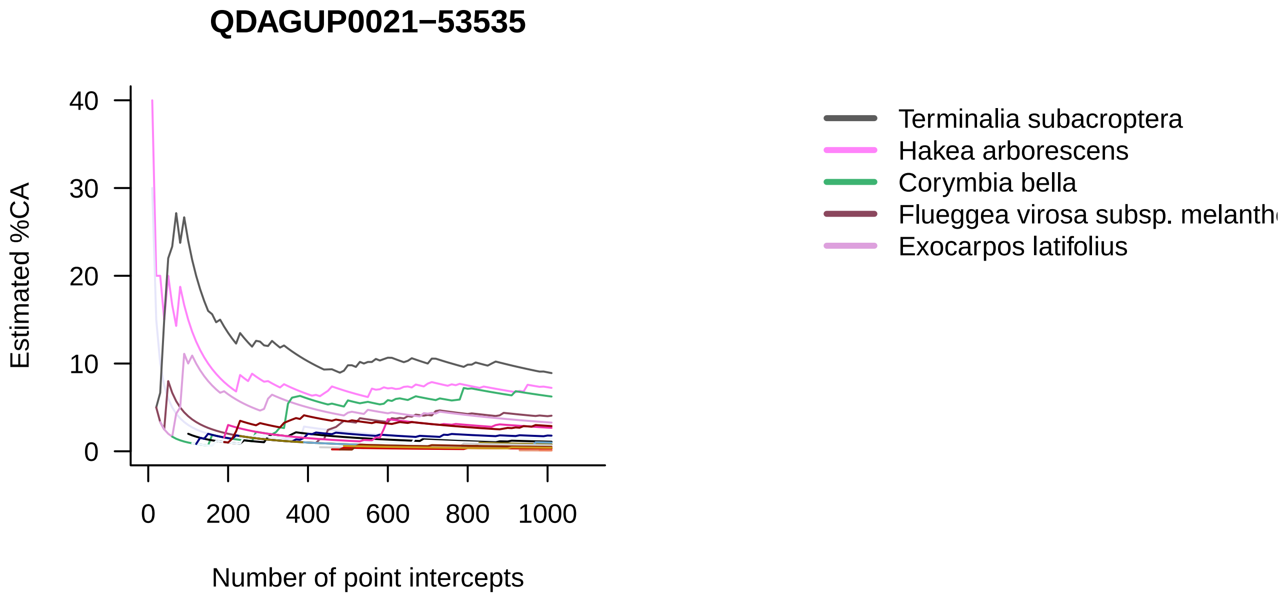
QDAGUP0021−53535
Estimated %CA
Number of point intercepts
0
10
20
30
40
0
200
400
600
800
1000
Terminalia subacroptera
Hakea arborescens
Corymbia bella
Flueggea virosa subsp. melanth
Exocarpos latifolius

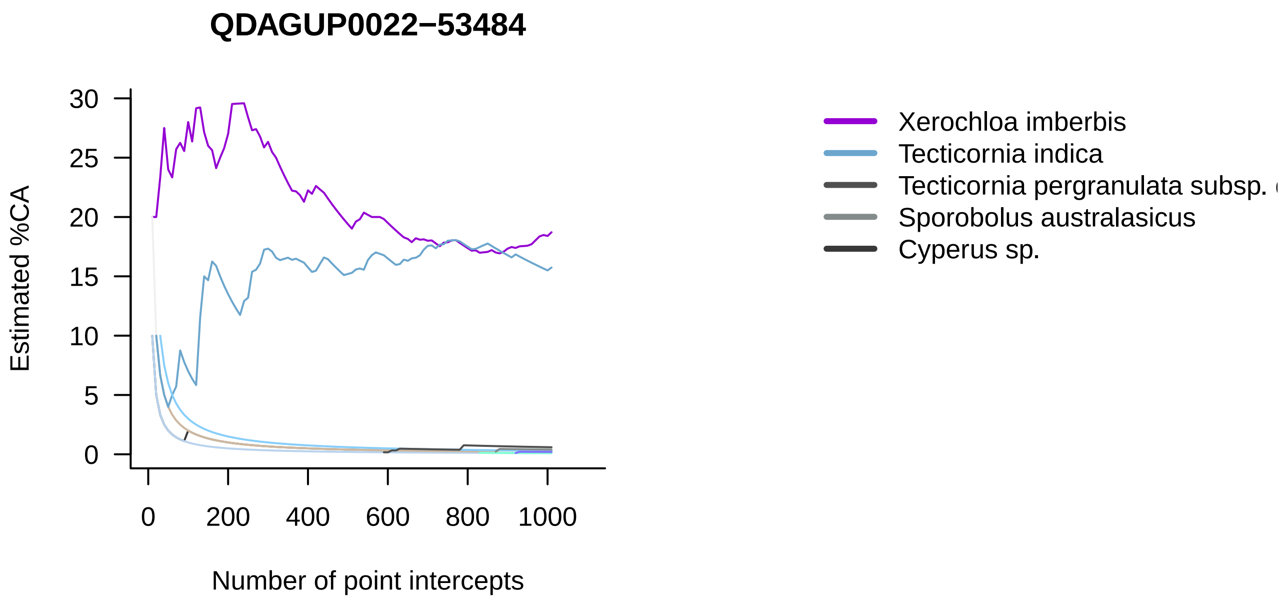
QDAGUP0022−53484
Estimated %CA
30
25
20
15
10
5
0
0
200
400
600
800
1000
Number of point intercepts
Xerochloa imberbis
Tecticornia indica
Tecticornia pergranulata subsp.
Sporobolus australasicus
Cyperus sp.

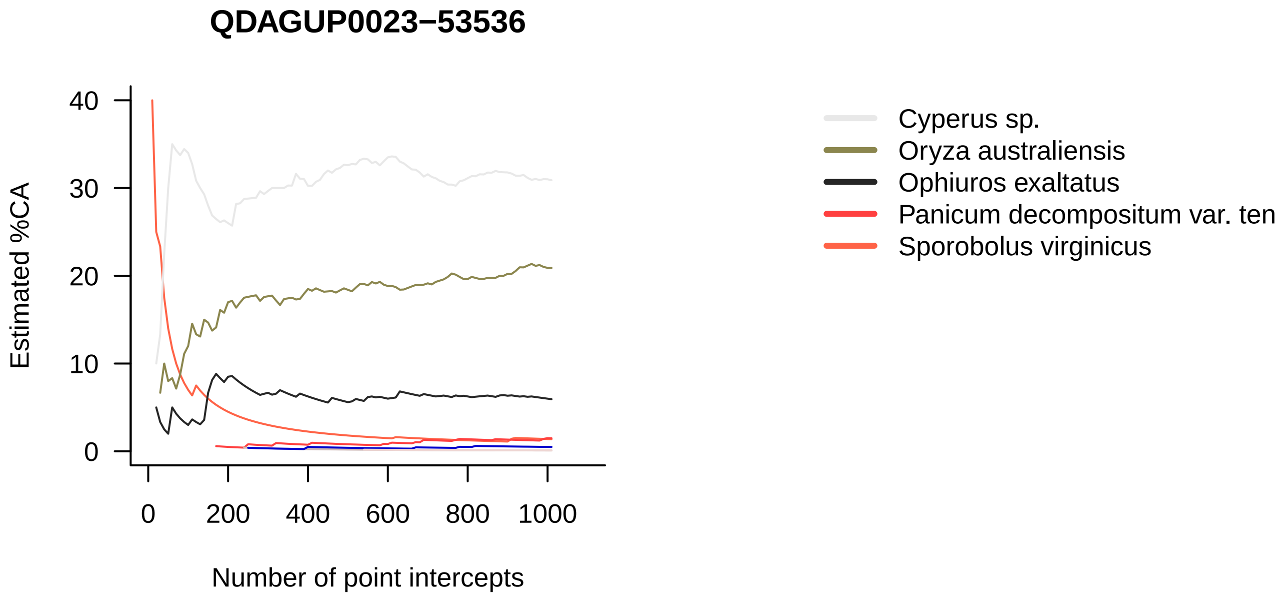
QDAGUP0023−53536
Estimated %CA
40
30
20
10
0
0
200
400
600
800
1000
Number of point intercepts
Cyperus sp.
Oryza australiensis
Ophiuros exaltatus
Panicum decompositum var. ten
Sporobolus virginicus

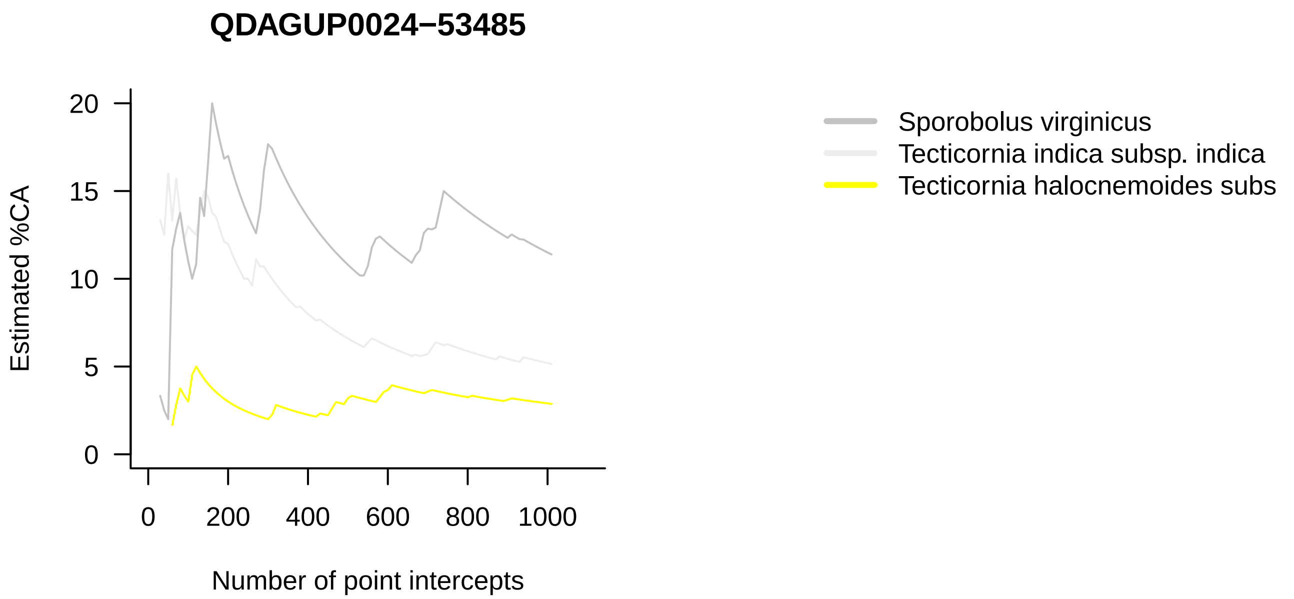
QDAGUP0024−53485
Estimated %CA
0
5
10
15
20
0
200
400
600
800
1000
Number of point intercepts
Sporobolus virginicus
Tecticornia indica subsp. indica
Tecticornia halocnemoides subs

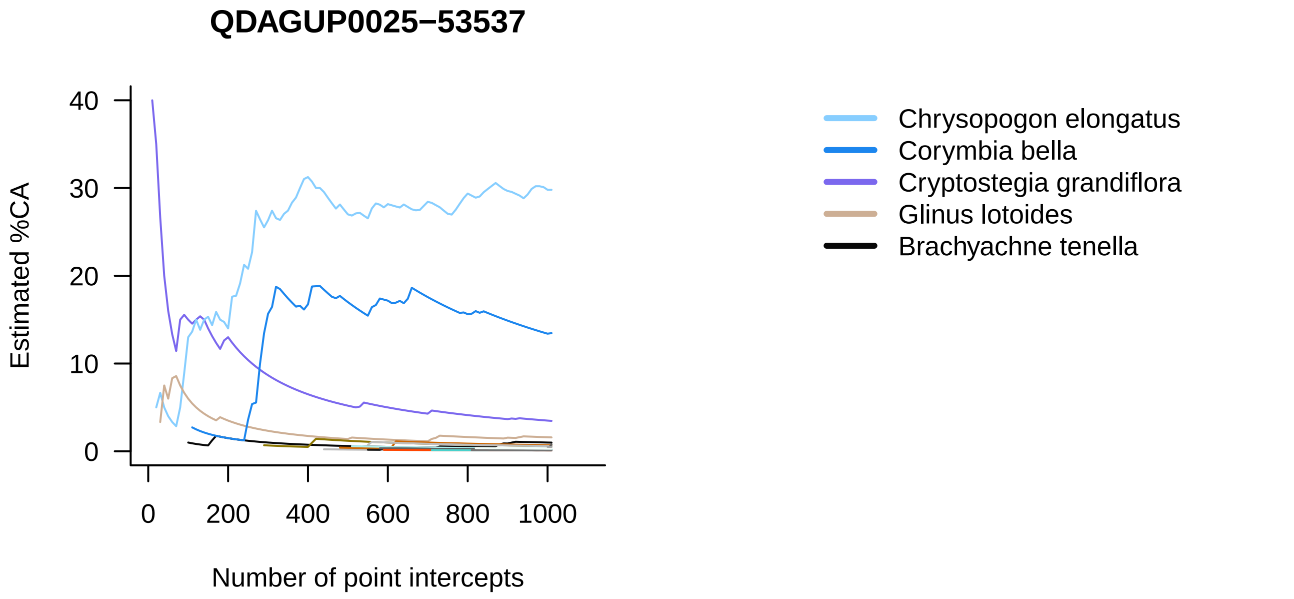
QDAGUP0025−53537
Estimated %CA
0
10
20
30
40
0
200
400
600
800
1000
Number of point intercepts
Chrysopogon elongatus
Corymbia bella
Cryptostegia grandiflora
Glinus lotoides
Brachyachne tenella

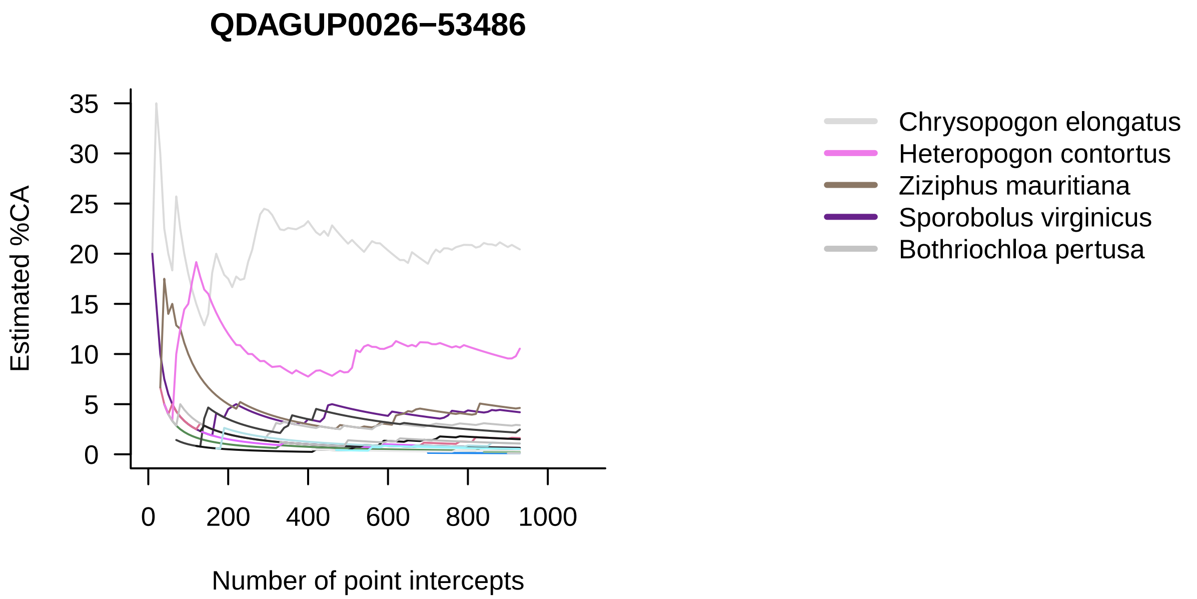
QDAGUP0026−53486
Estimated %CA
35
30
25
20
15
10
5
0
0
200
400
600
800
1000
Number of point intercepts
Chrysopogon elongatus
Heteropogon contortus
Ziziphus mauritiana
Sporobolus virginicus
Bothriochloa pertusa

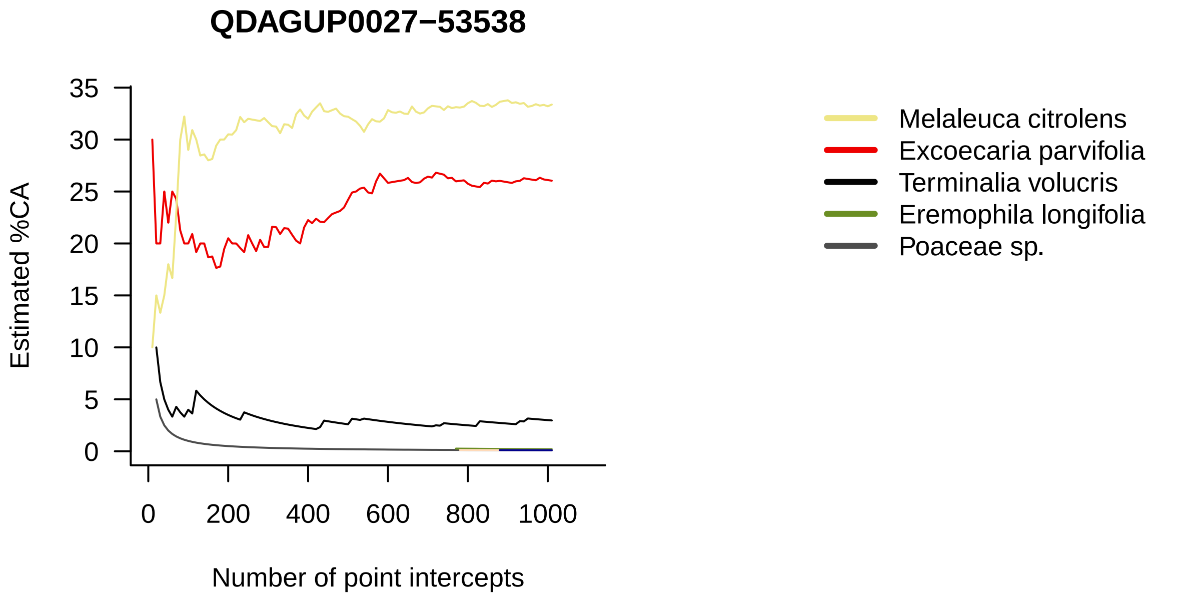
QDAGUP0027−53538
Estimated %CA
35
30
25
20
15
10
5
0
0
200
400
600
800
1000
Number of point intercepts
Melaleuca citrolens
Excoecaria parvifolia
Terminalia volucris
Eremophila longifolia
Poaceae sp.

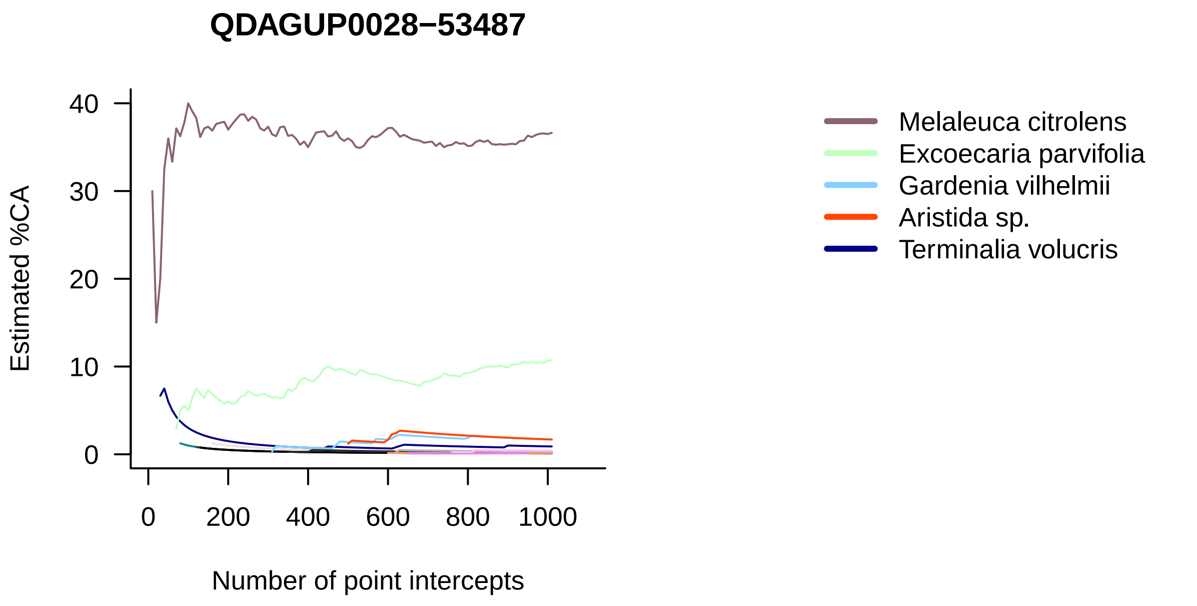
QDAGUP0028−53487
Estimated %CA
0
10
20
30
40
0
200
400
600
800
1000
Number of point intercepts
Melaleuca citrolens
Excoecaria parvifolia
Gardenia vilhelmii
Aristida sp.
Terminalia volucris

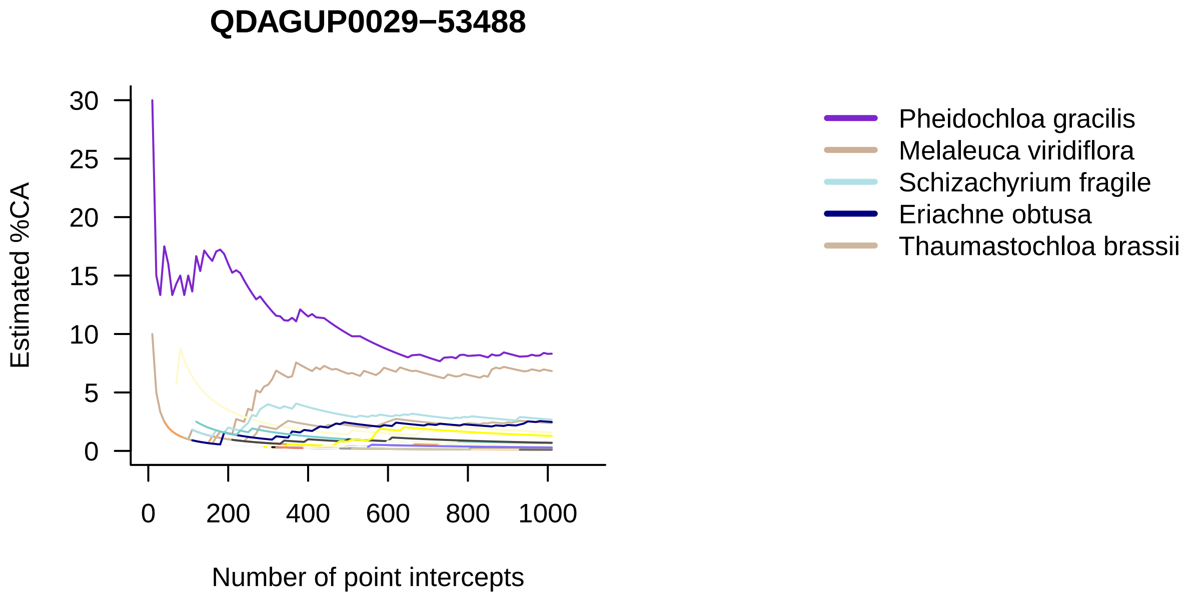
QDAGUP0029−53488
Estimated %CA
0
5
10
15
20
25
30
0
200
400
600
800
1000
Number of point intercepts
Pheidochloa gracilis
Melaleuca viridiflora
Schizachyrium fragile
Eriachne obtusa
Thaumastochloa brassii

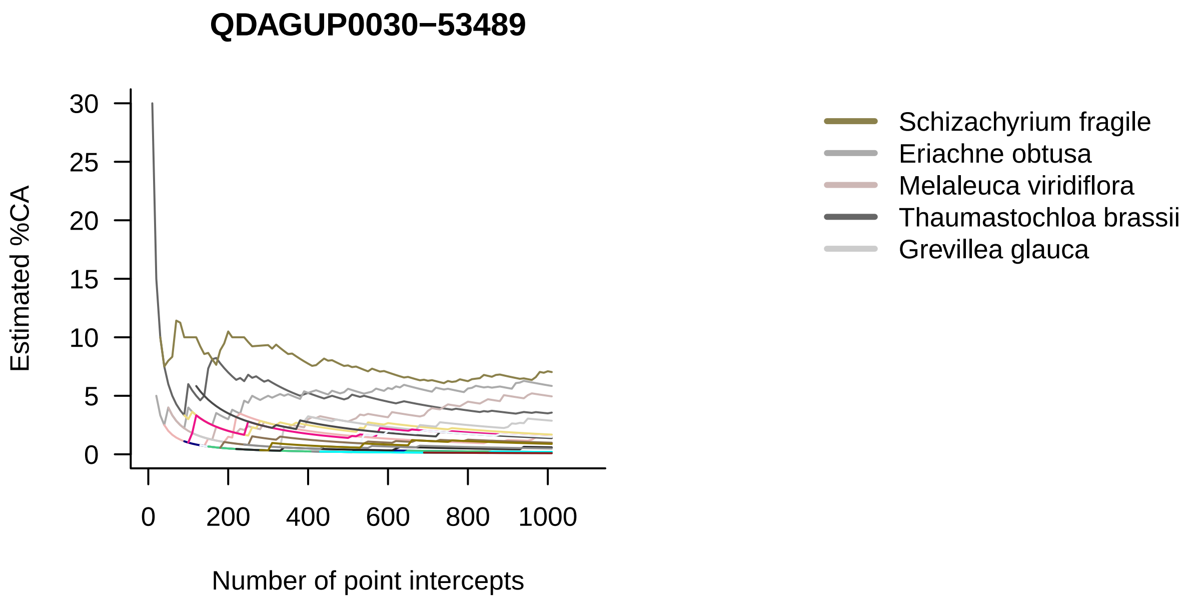

QDAGUP0030−53489
Estimated %CA
30
25
20
15
10
5
0
0
200
400
600
800
1000
Number of point intercepts
Schizachyrium fragile
Eriachne obtusa
Melaleuca viridiflora
Thaumastochloa brassii
Grevillea glauca


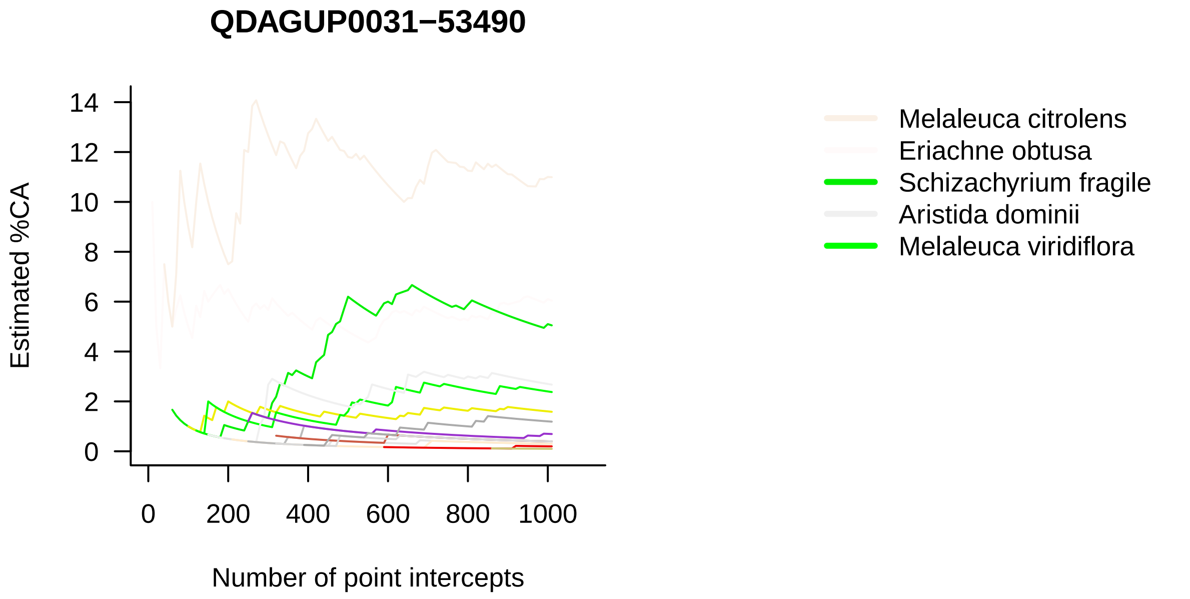

QDAGUP0031−53490
Estimated %CA
14
12
10
8
6
4
2
0
0
200
400
600
800
1000
Number of point intercepts
Melaleuca citrolens
Eriachne obtusa
Schizachyrium fragile
Aristida dominii
Melaleuca viridiflora

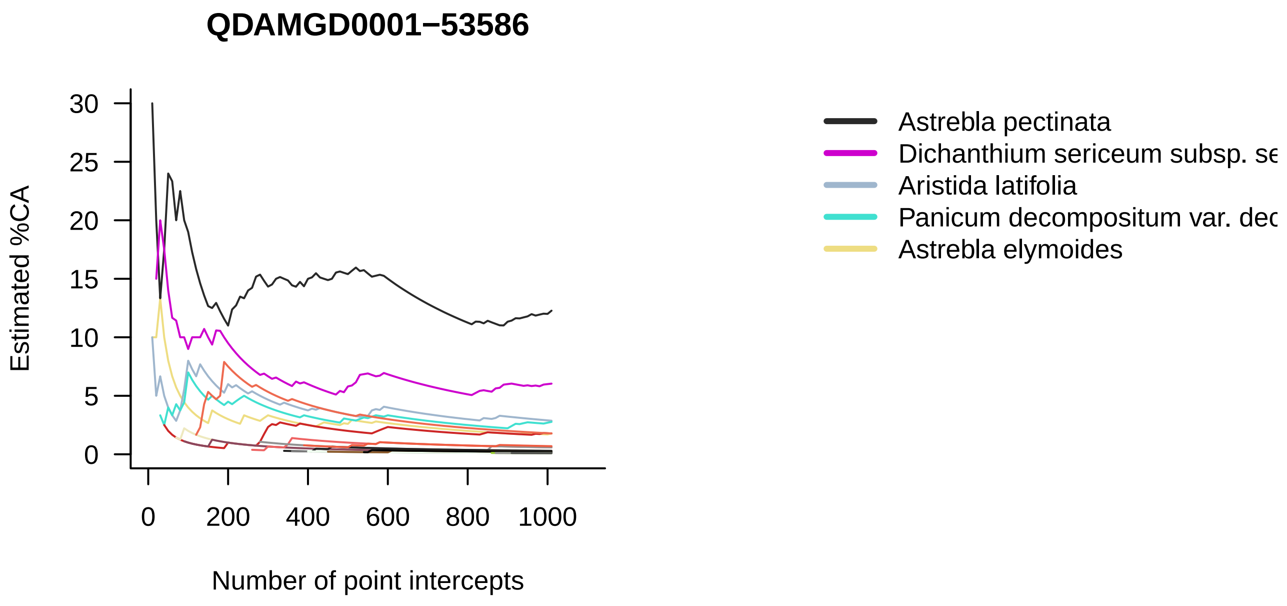
QDAMGD0001−53586
Estimated %CA
0
5
10
15
20
25
30
0
200
400
600
800
1000
Number of point intercepts
Astrebla pectinata
Dichanthium sericeum subsp. se
Aristida latifolia
Panicum decompositum var. dec
Astrebla elymoides

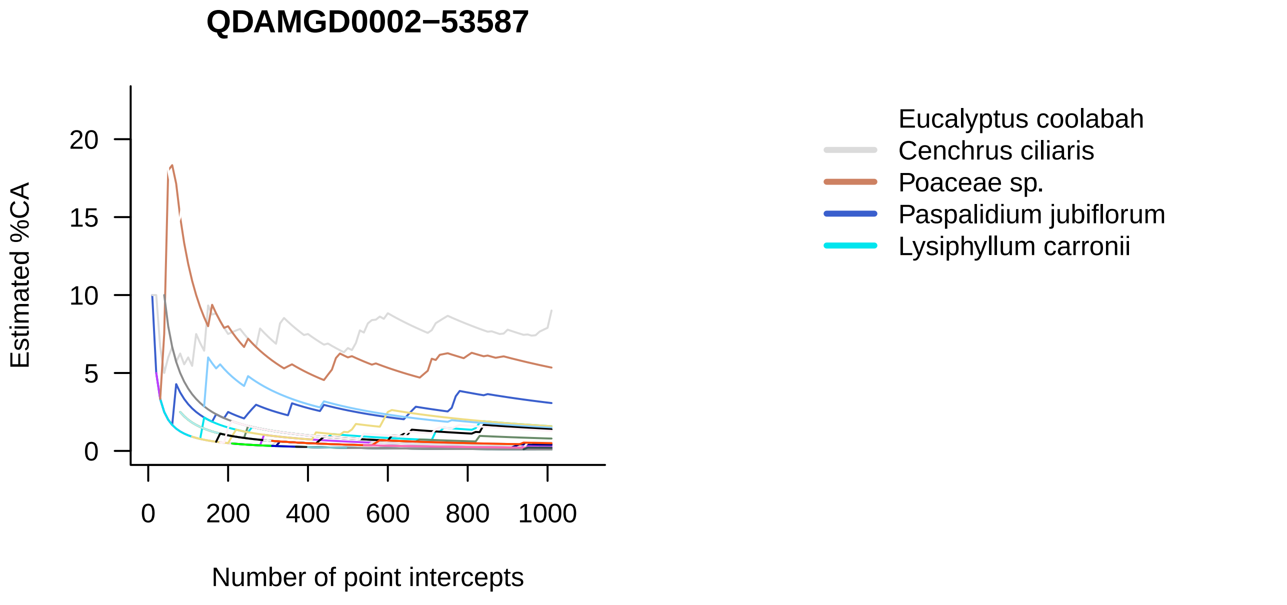
QDAMGD0002−53587
Estimated %CA
0
5
10
15
20
0
200
400
600
800
1000
Number of point intercepts
Eucalyptus coolabah
Cenchrus ciliaris
Poaceae sp.
Paspalidium jubiflorum
Lysiphyllum carronii

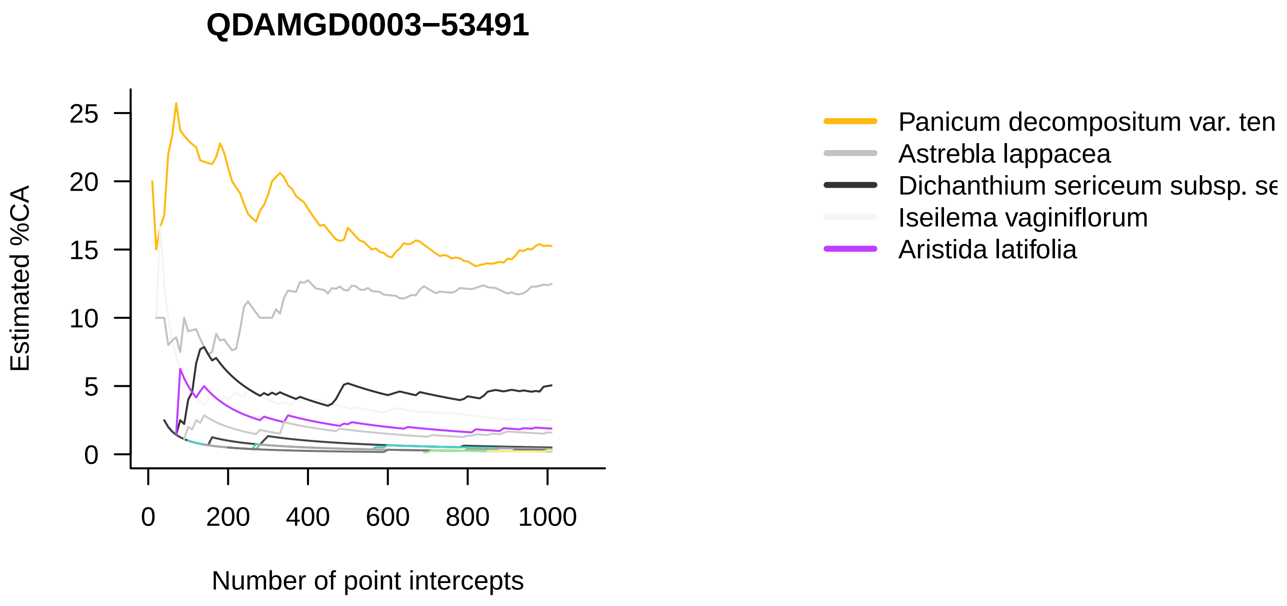
QDAMGD0003−53491
Estimated %CA
25
20
15
10
5
0
0
200
400
600
800
1000
Number of point intercepts
Panicum decompositum var. ten
Astrebla lappacea
Dichanthium sericeum subsp. se
Iseilema vaginiflorum
Aristida latifolia

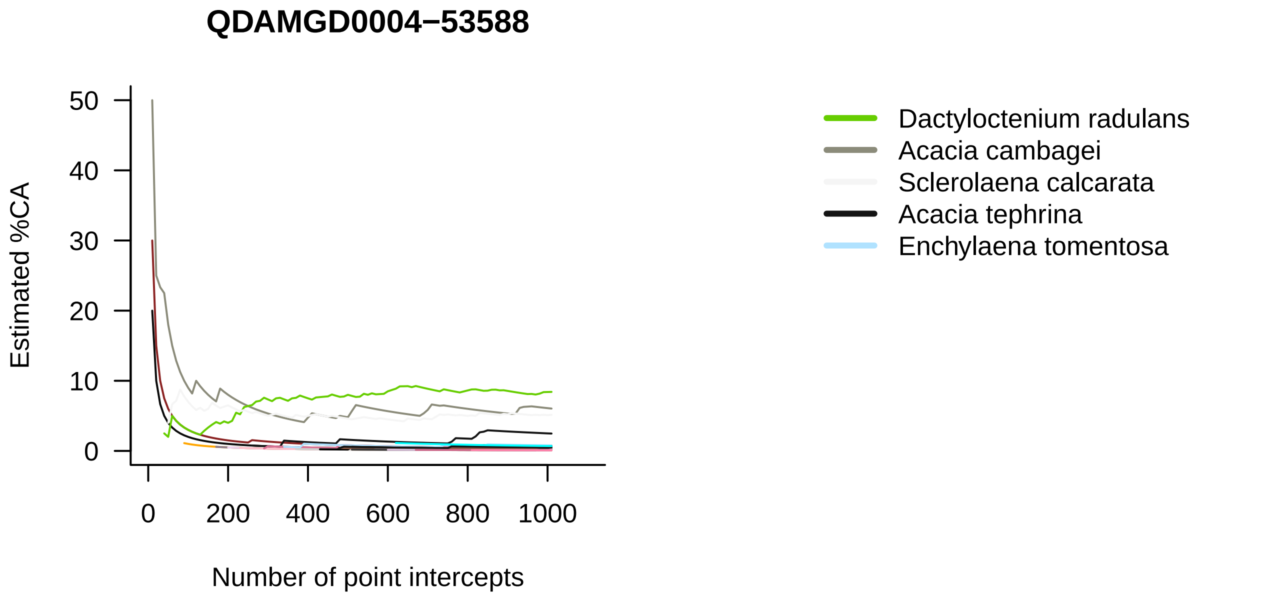
QDAMGD0004−53588
Estimated %CA
50
40
30
20
10
0
0
200
400
600
800
1000
Number of point intercepts
Dactyloctenium radulans
Acacia cambagei
Sclerolaena calcarata
Acacia tephrina
Enchylaena tomentosa

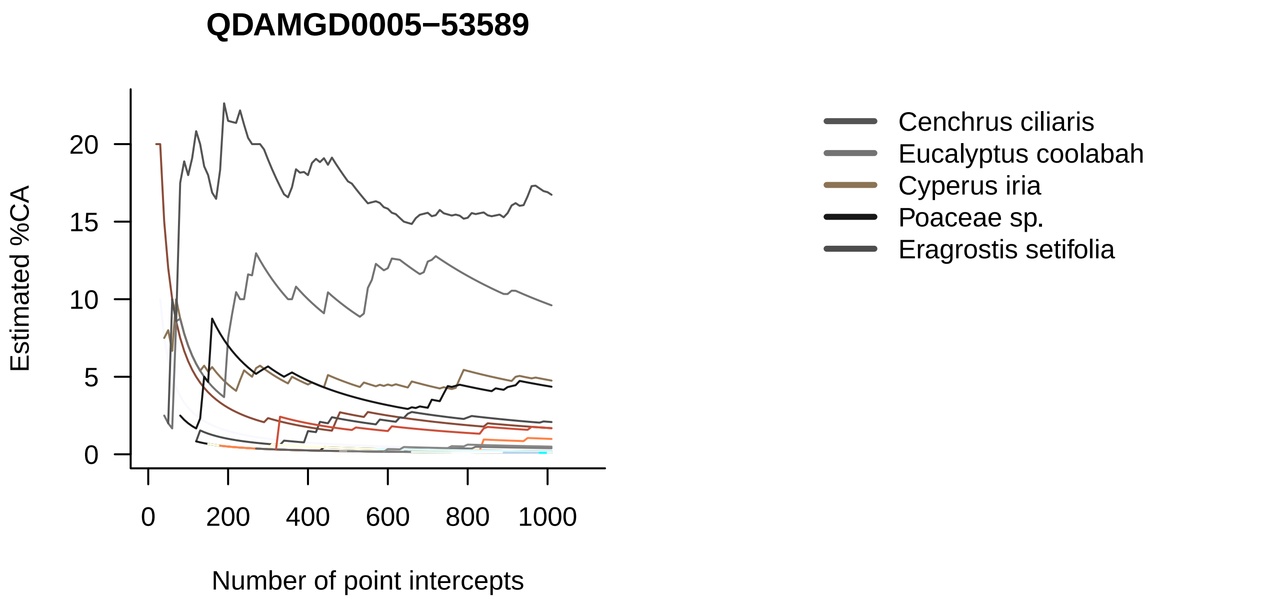
QDAMGD0005−53589
Estimated %CA
0
5
10
15
20
0
200
400
600
800
1000
Number of point intercepts
Cenchrus ciliaris
Eucalyptus coolabah
Cyperus iria
Poaceae sp.
Eragrostis setifolia

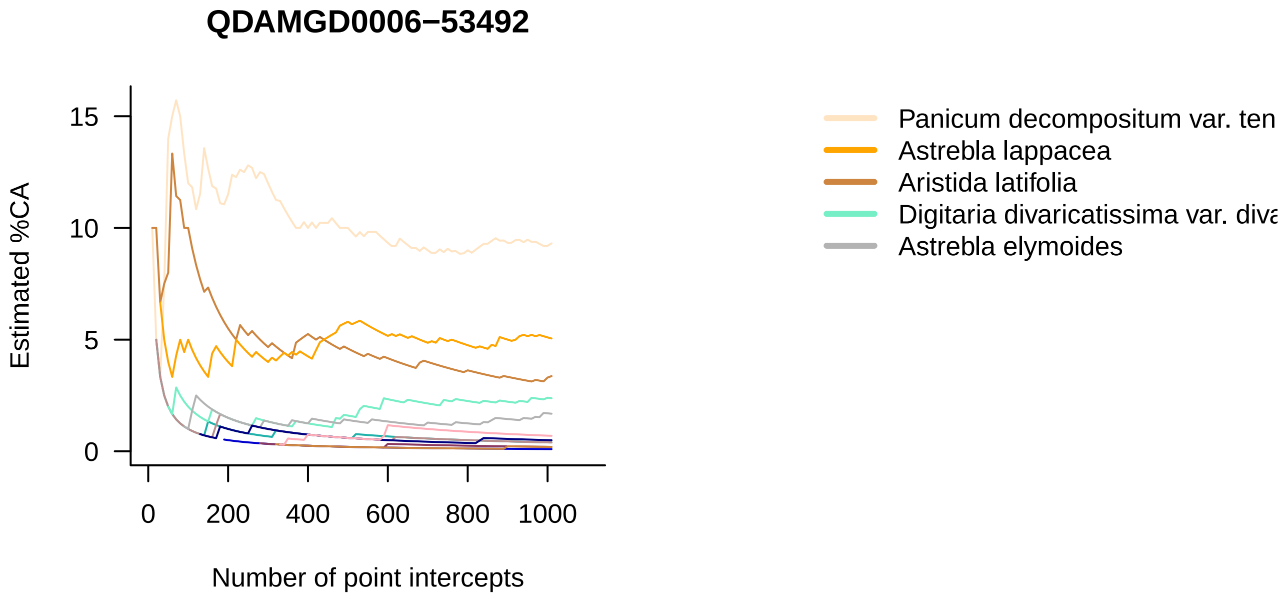
QDAMGD0006−53492
Estimated %CA
0
5
10
15
0
200
400
600
800
1000
Number of point intercepts
Panicum decompositum var. ten
Astrebla lappacea
Aristida latifolia
Digitaria divaricatissima var. diva
Astrebla elymoides

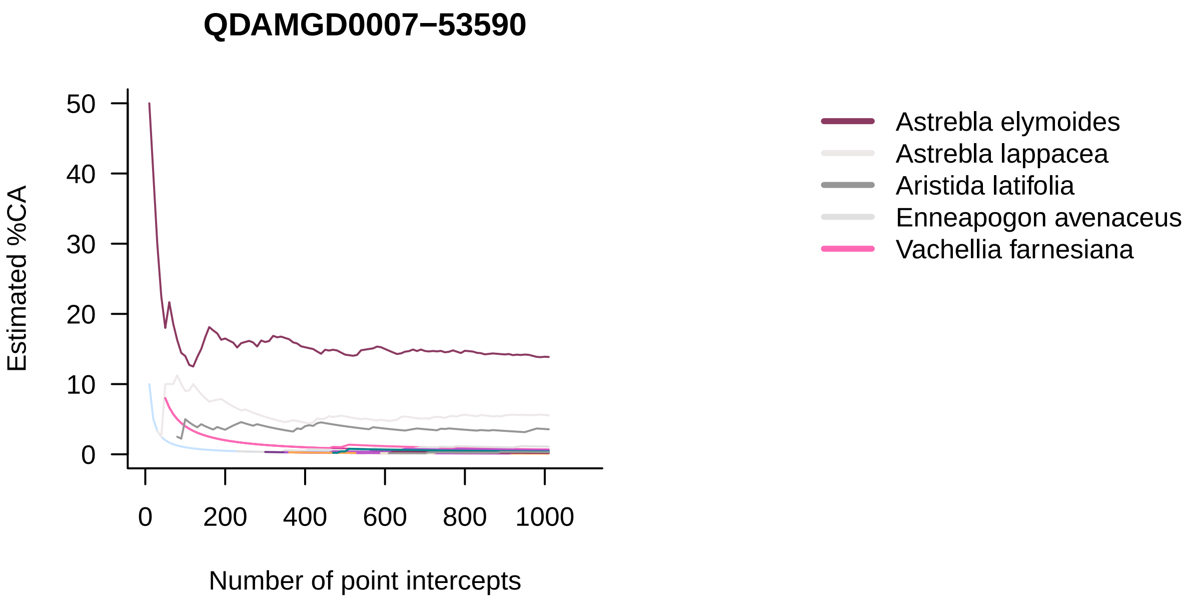
QDAMGD0007−53590
Estimated %CA
50
40
30
20
10
0
0
200
400
600
800
1000
Number of point intercepts
Astrebla elymoides
Astrebla lappacea
Aristida latifolia
Enneapogon avenaceus
Vachellia farnesiana

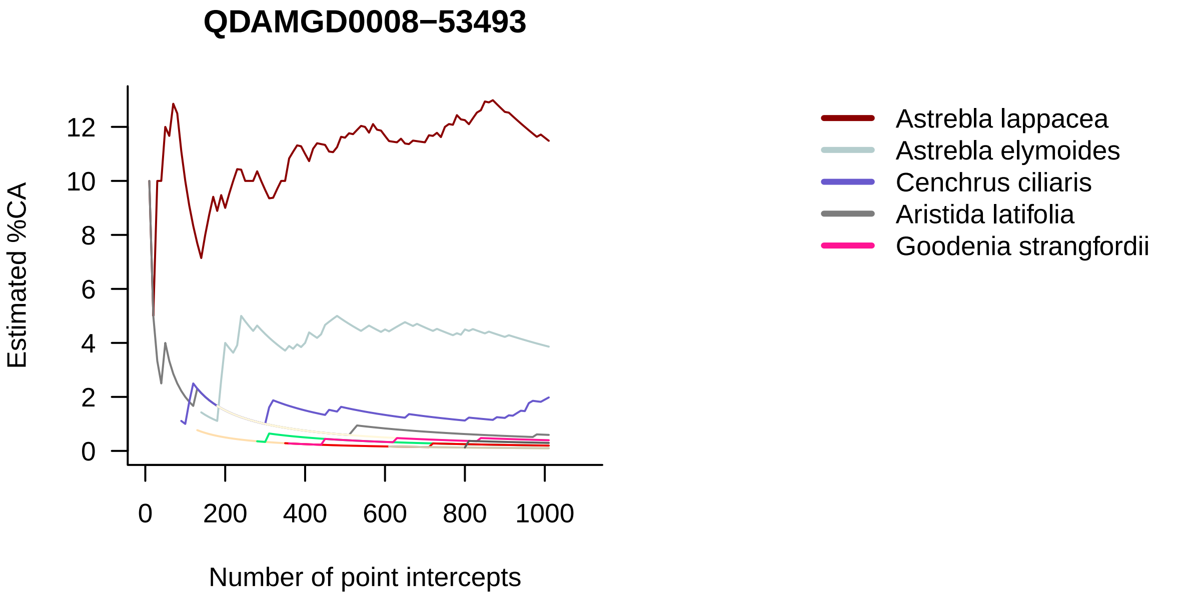
QDAMGD0008−53493
Estimated %CA
12
10
8
6
4
2
0
0
200
400
600
800
1000
Number of point intercepts
Astrebla lappacea
Astrebla elymoides
Cenchrus ciliaris
Aristida latifolia
Goodenia strangfordii

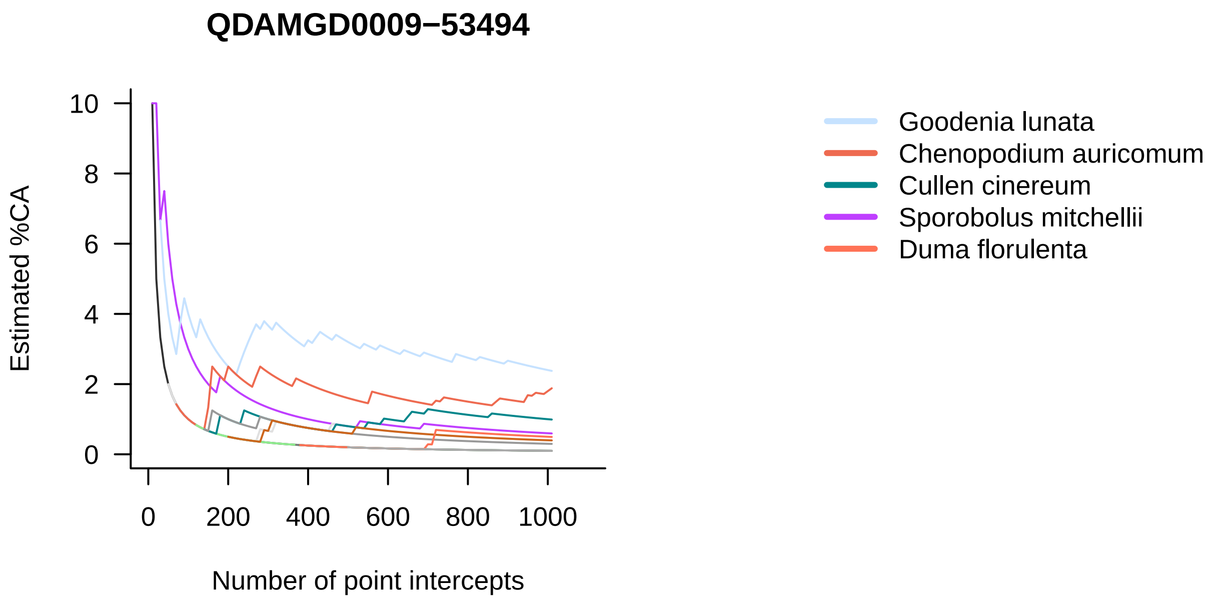
QDAMGD0009−53494
Estimated %CA
0
2
4
6
8
10
0
200
400
600
800
1000
Number of point intercepts
Goodenia lunata
Chenopodium auricomum
Cullen cinereum
Sporobolus mitchellii
Duma florulenta

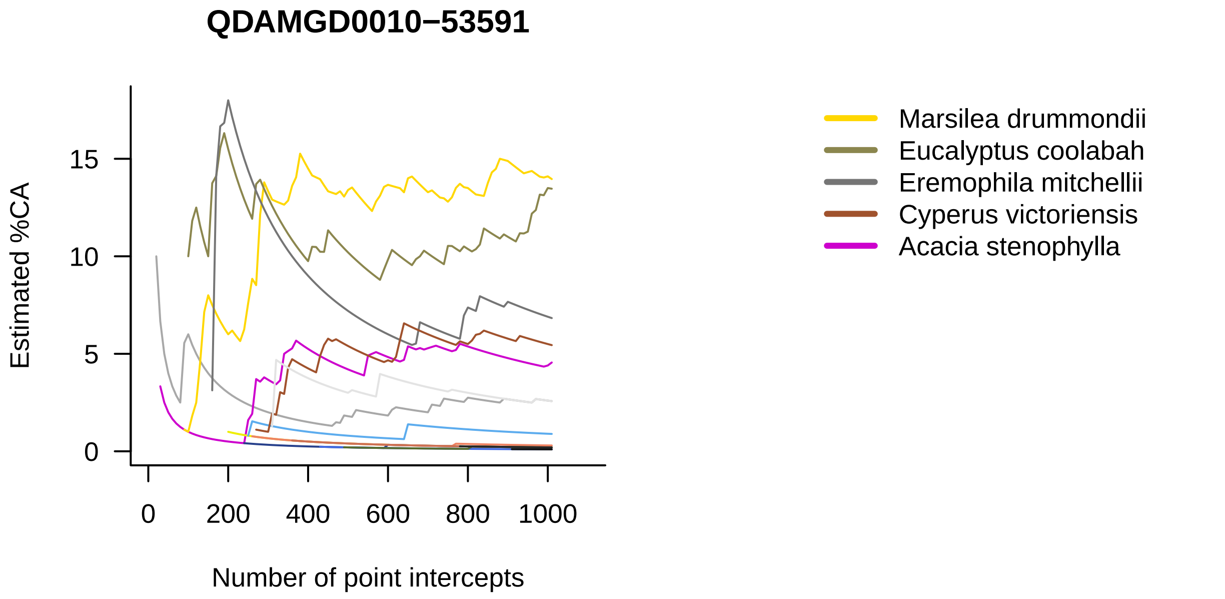
QDAMGD0010−53591
Estimated %CA
0
5
10
15
0
200
400
600
800
1000
Number of point intercepts
Marsilea drummondii
Eucalyptus coolabah
Eremophila mitchellii
Cyperus victoriensis
Acacia stenophylla

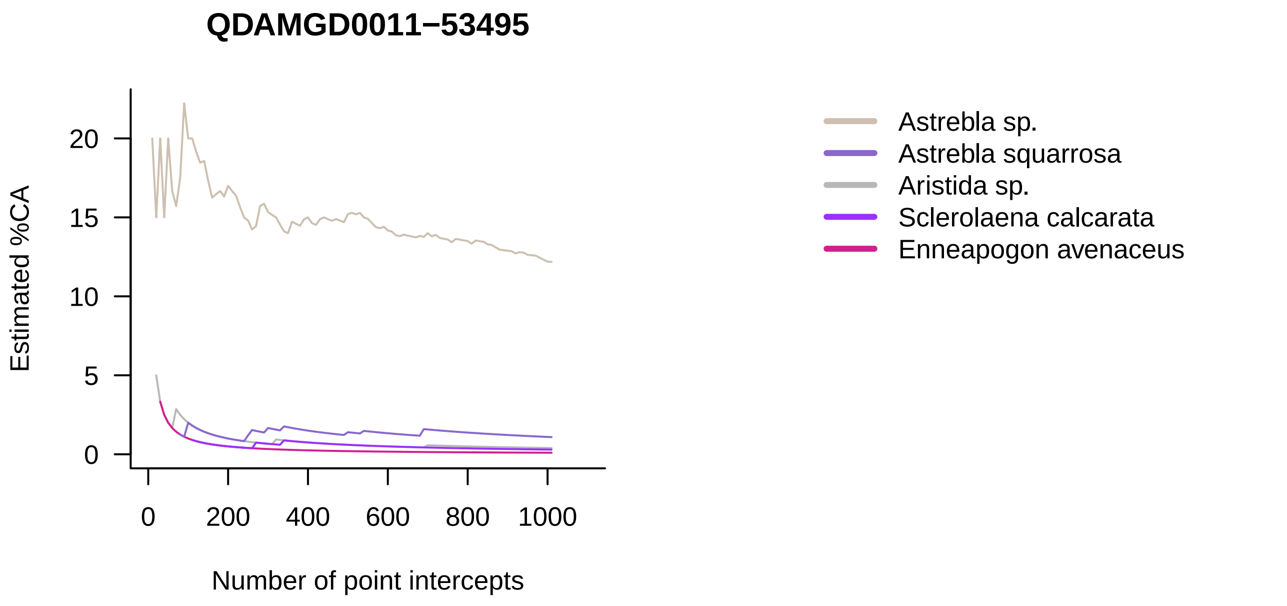
QDAMGD0011−53495
Estimated %CA
0
5
10
15
20
0
200
400
600
800
1000
Number of point intercepts
Astrebla sp.
Astrebla squarrosa
Aristida sp.
Sclerolaena calcarata
Enneapogon avenaceus

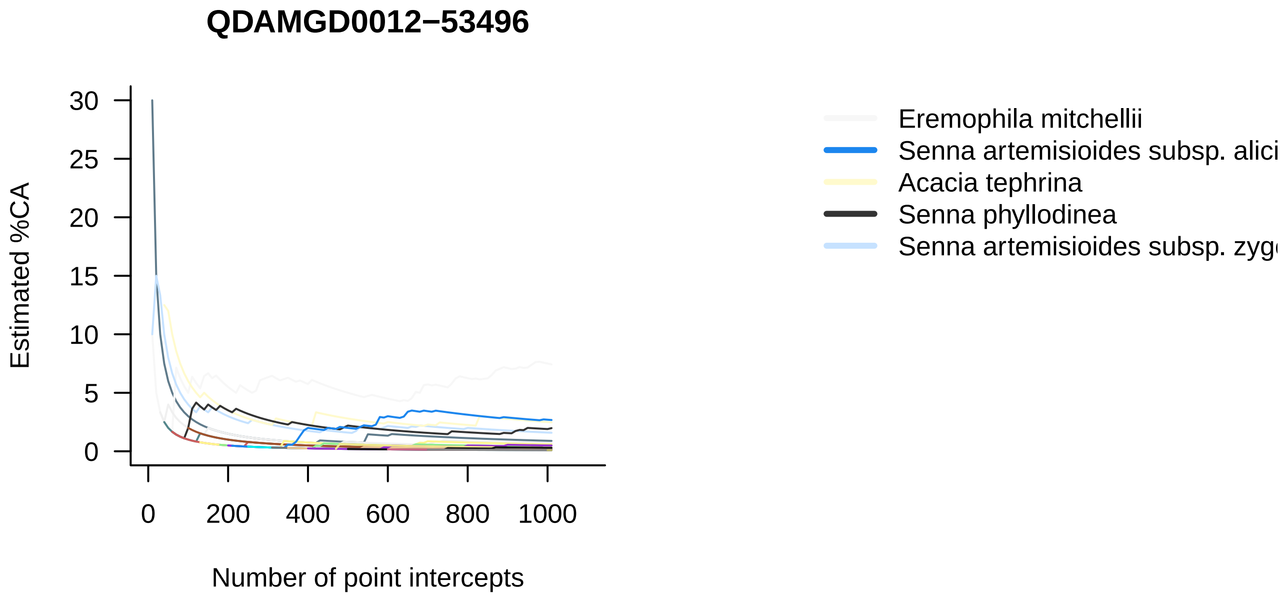
QDAMGD0012−53496
Estimated %CA
0
5
10
15
20
25
30
0
200
400
600
800
1000
Number of point intercepts
Eremophila mitchellii
Senna artemisioides subsp. alici
Acacia tephrina
Senna phyllodinea
Senna artemisioides subsp. zyg

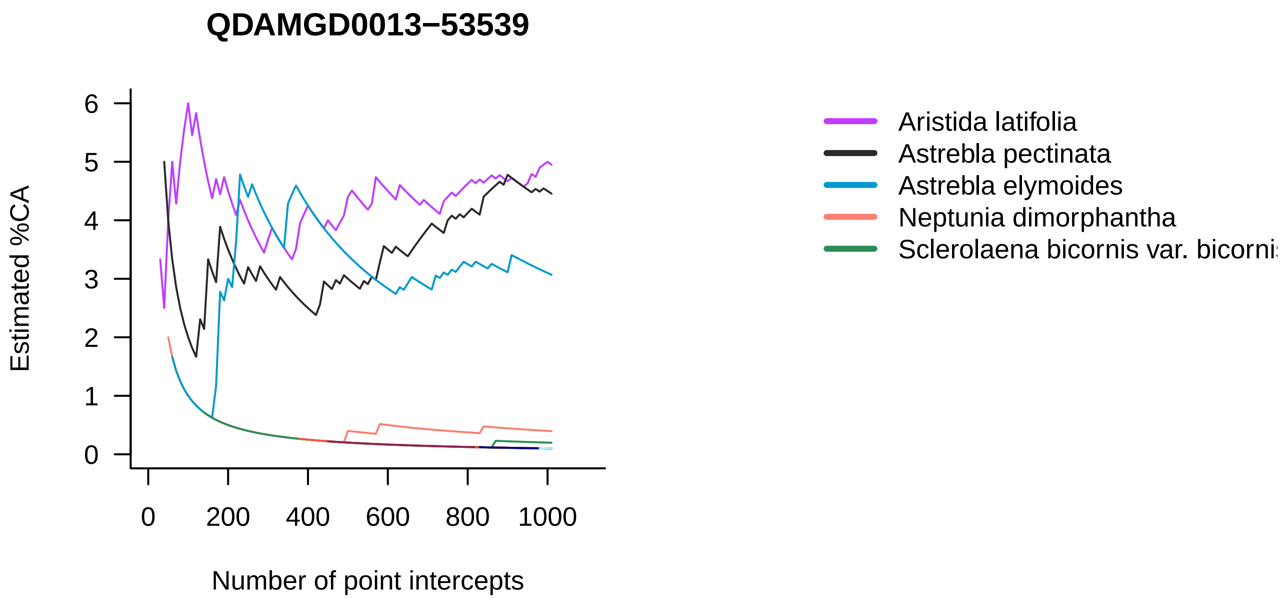
QDAMGD0013−53539
Estimated %CA
Number of point intercepts
0
1
2
3
4
5
6
0
200
400
600
800
1000
Aristida latifolia
Astrebla pectinata
Astrebla elymoides
Neptunia dimorphantha
Sclerolaena bicornis var. bicorni

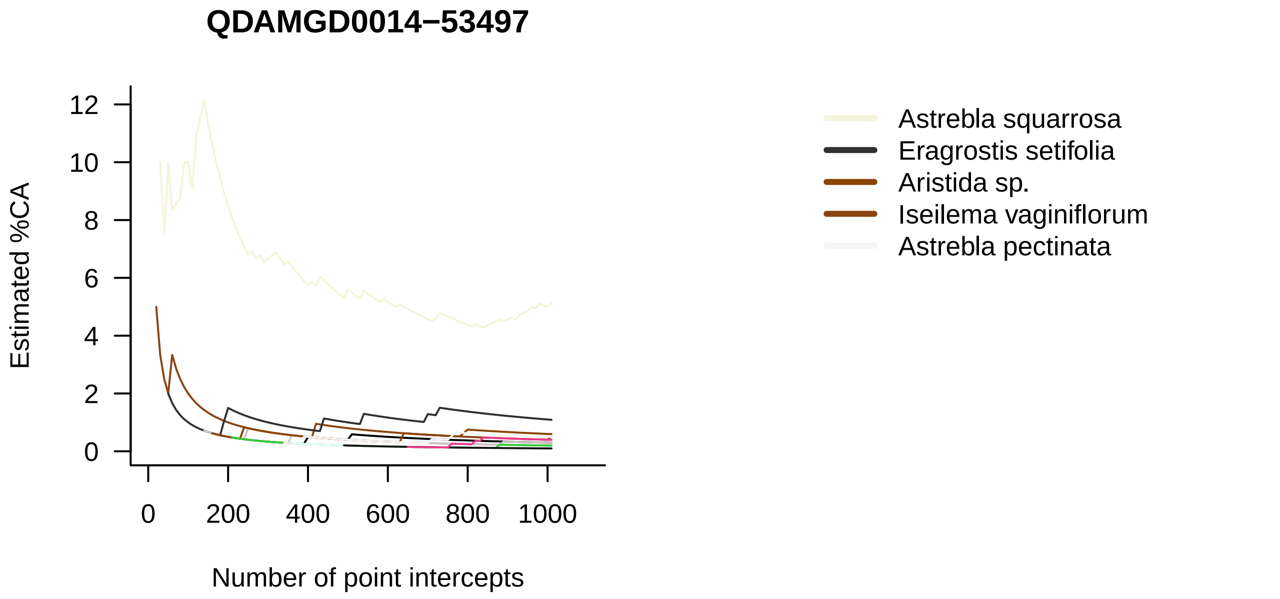
QDAMGD0014−53497
Estimated %CA
Number of point intercepts
0
2
4
6
8
10
12
0
200
400
600
800
1000
Astrebla squarrosa
Eragrostis setifolia
Aristida sp.
Iseilema vaginiflorum
Astrebla pectinata

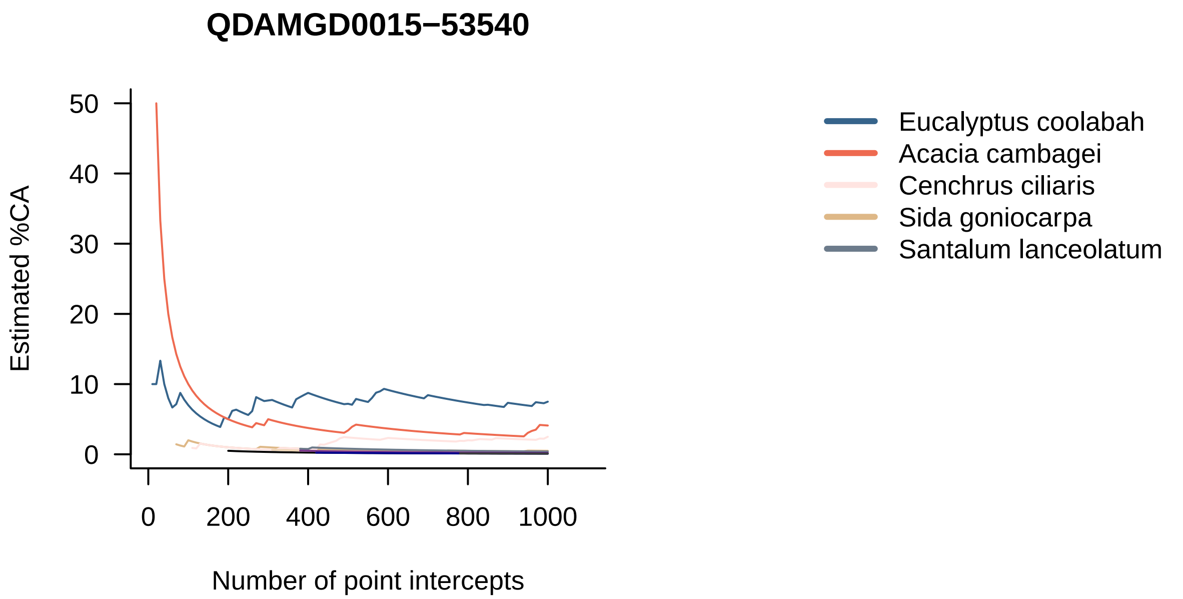
QDAMGD0015−53540
Estimated %CA
0
10
20
30
40
50
0
200
400
600
800
1000
Number of point intercepts
Eucalyptus coolabah
Acacia cambagei
Cenchrus ciliaris
Sida goniocarpa
Santalum lanceolatum

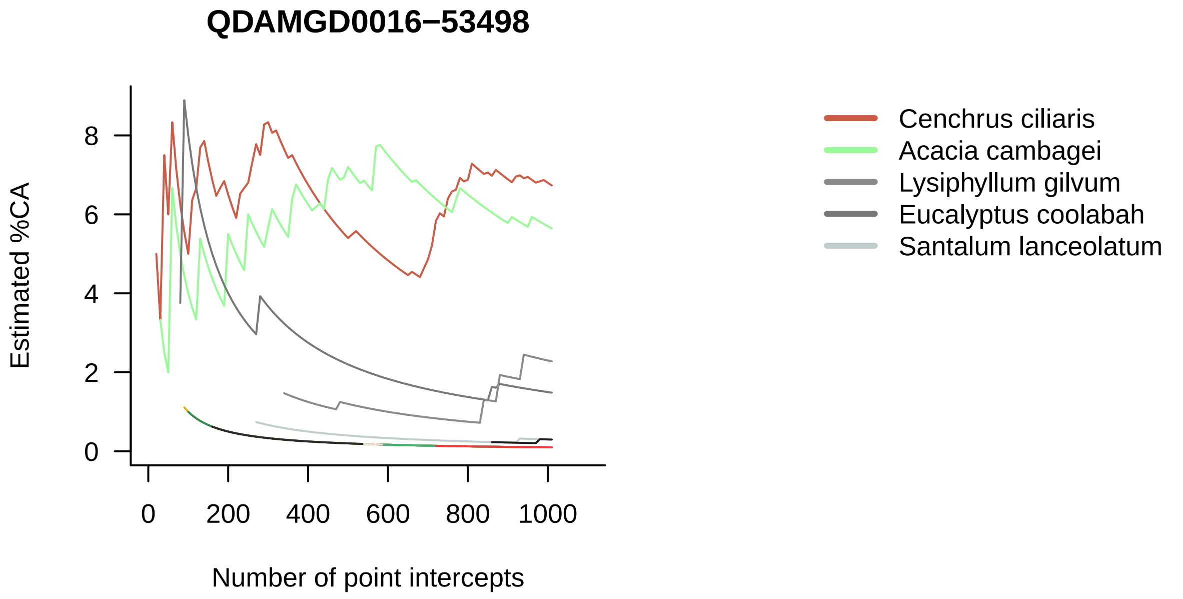
QDAMGD0016−53498
Estimated %CA
0
2
4
6
8
0
200
400
600
800
1000
Number of point intercepts
Cenchrus ciliaris
Acacia cambagei
Lysiphyllum gilvum
Eucalyptus coolabah
Santalum lanceolatum

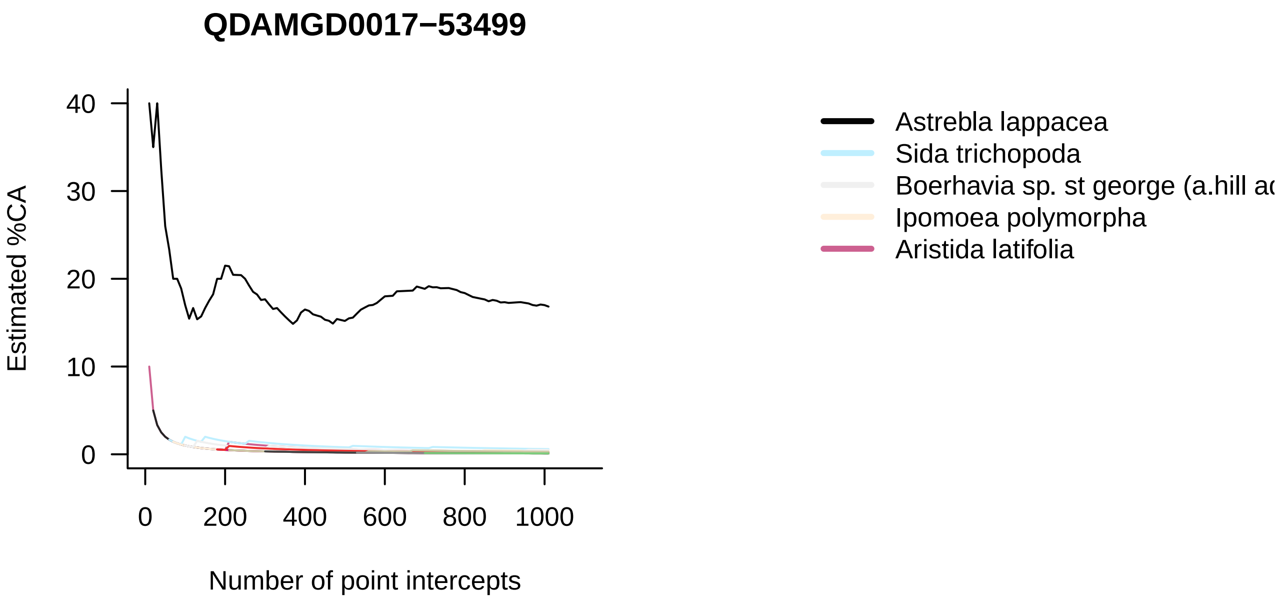
QDAMGD0017−53499
Estimated %CA
Number of point intercepts
0
10
20
30
40
0
200
400
600
800
1000
Astrebla lappacea
Sida trichopoda
Boerhavia sp. st george (a.hill ac
Ipomoea polymorpha
Aristida latifolia

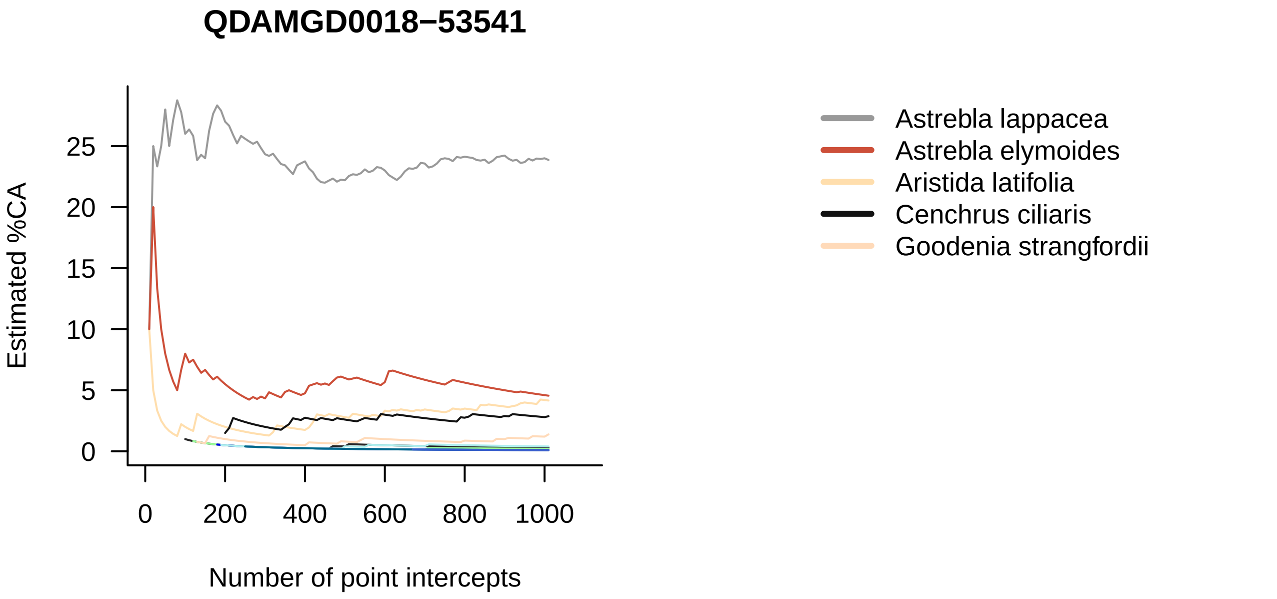
QDAMGD0018−53541
Estimated %CA
Number of point intercepts
0
5
10
15
20
25
0
200
400
600
800
1000
Astrebla lappacea
Astrebla elymoides
Aristida latifolia
Cenchrus ciliaris
Goodenia strangfordii

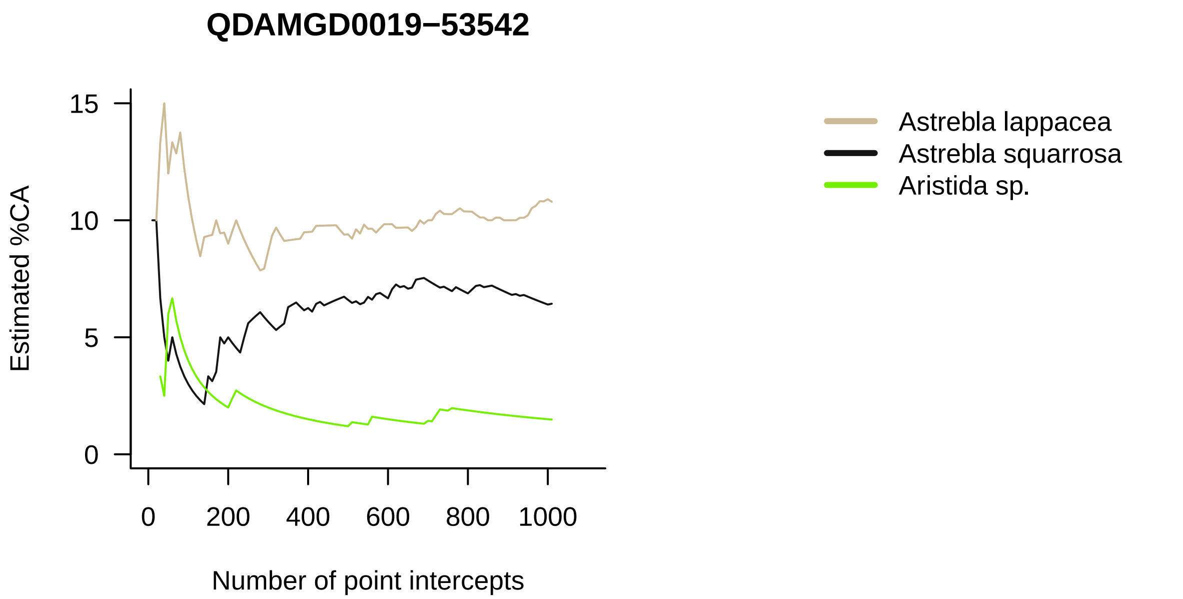

QDAMGD0019−53542
Estimated %CA
0
5
10
15
0
200
400
600
800
1000
Number of point intercepts
Astrebla lappacea
Astrebla squarrosa
Aristida sp.


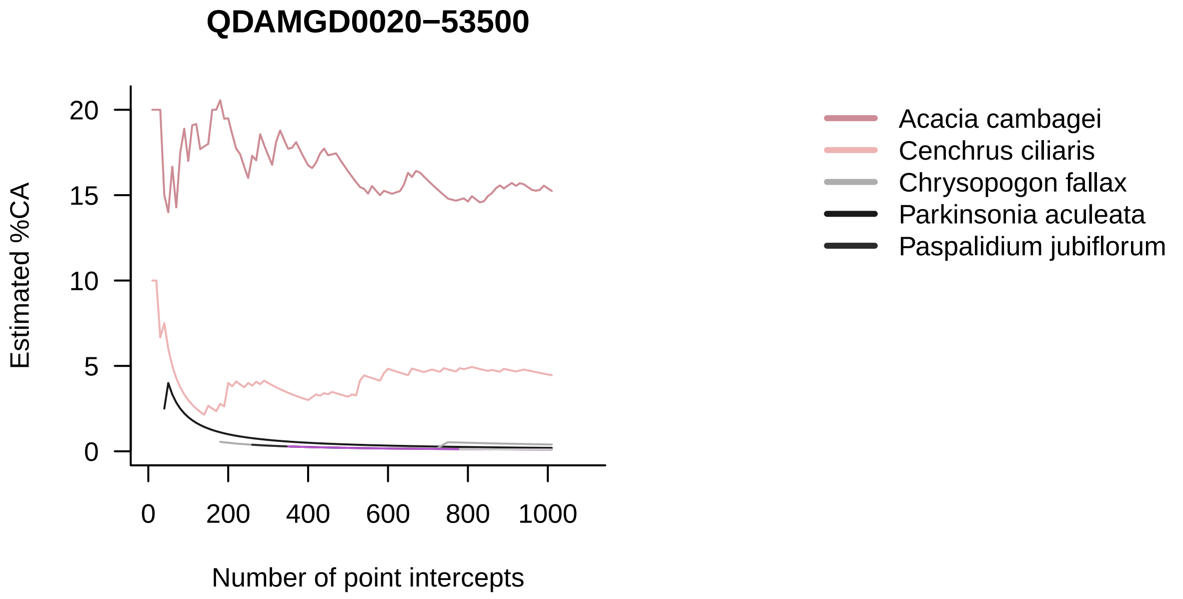

QDAMGD0020−53500
Estimated %CA
0
5
10
15
20
0
200
400
600
800
1000
Number of point intercepts
Acacia cambagei
Cenchrus ciliaris
Chrysopogon fallax
Parkinsonia aculeata
Paspalidium jubiflorum

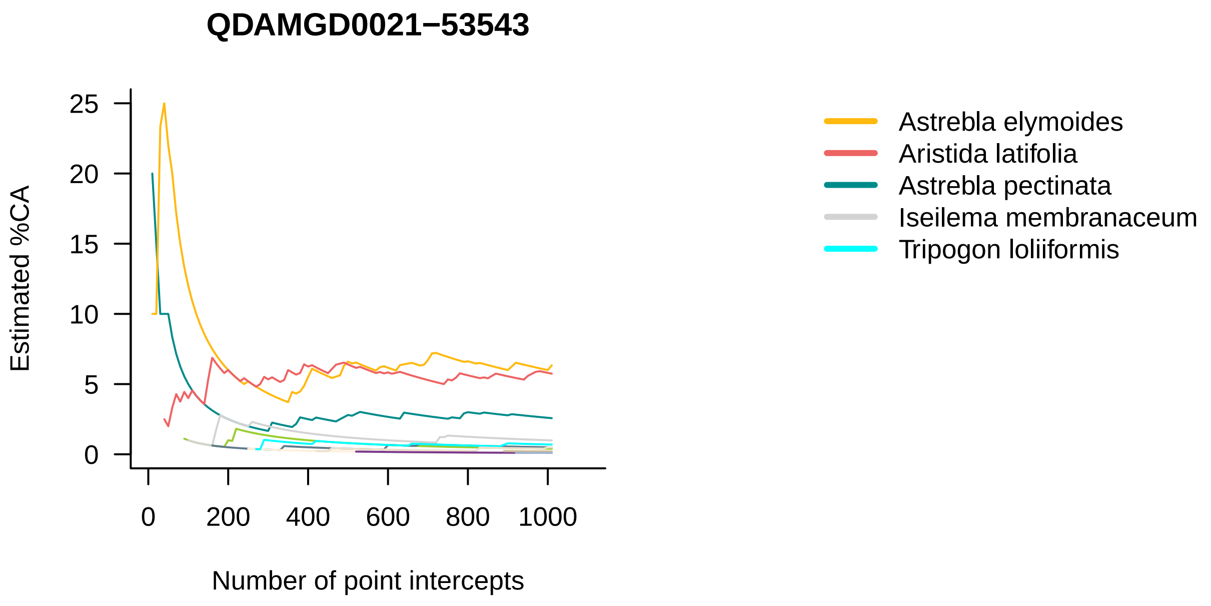
QDAMGD0021−53543
Estimated %CA
0
5
10
15
20
25
0
200
400
600
800
1000
Number of point intercepts
Astrebla elymoides
Aristida latifolia
Astrebla pectinata
Iseilema membranaceum
Tripogon loliiformis

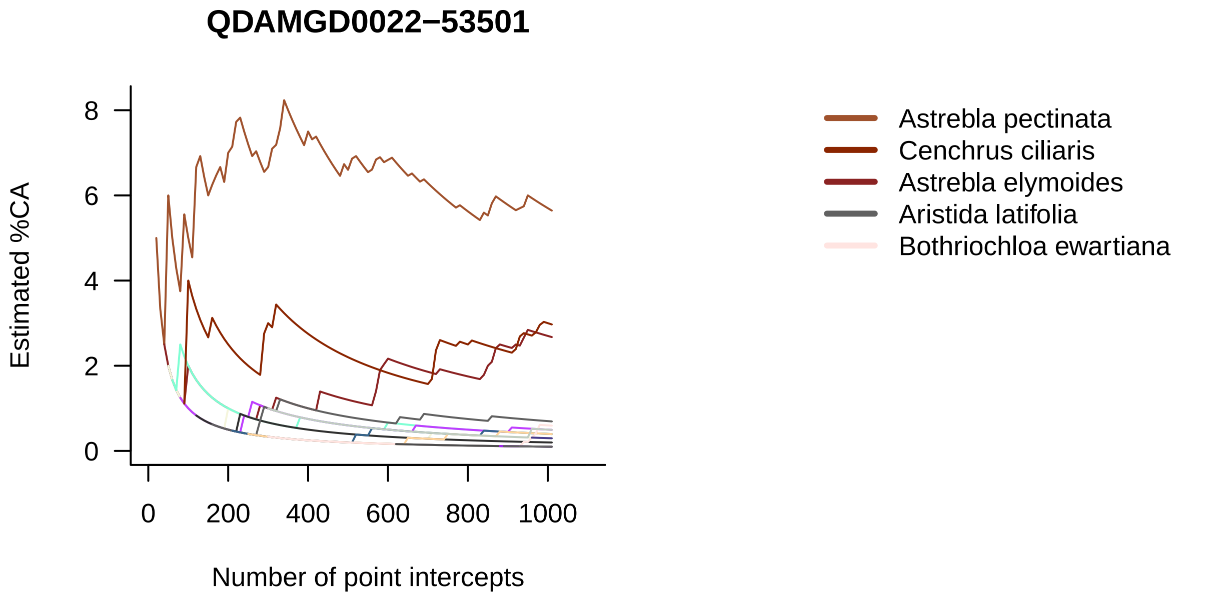
QDAMGD0022−53501
Estimated %CA
0
2
4
6
8
0
200
400
600
800
1000
Number of point intercepts
Astrebla pectinata
Cenchrus ciliaris
Astrebla elymoides
Aristida latifolia
Bothriochloa ewartiana

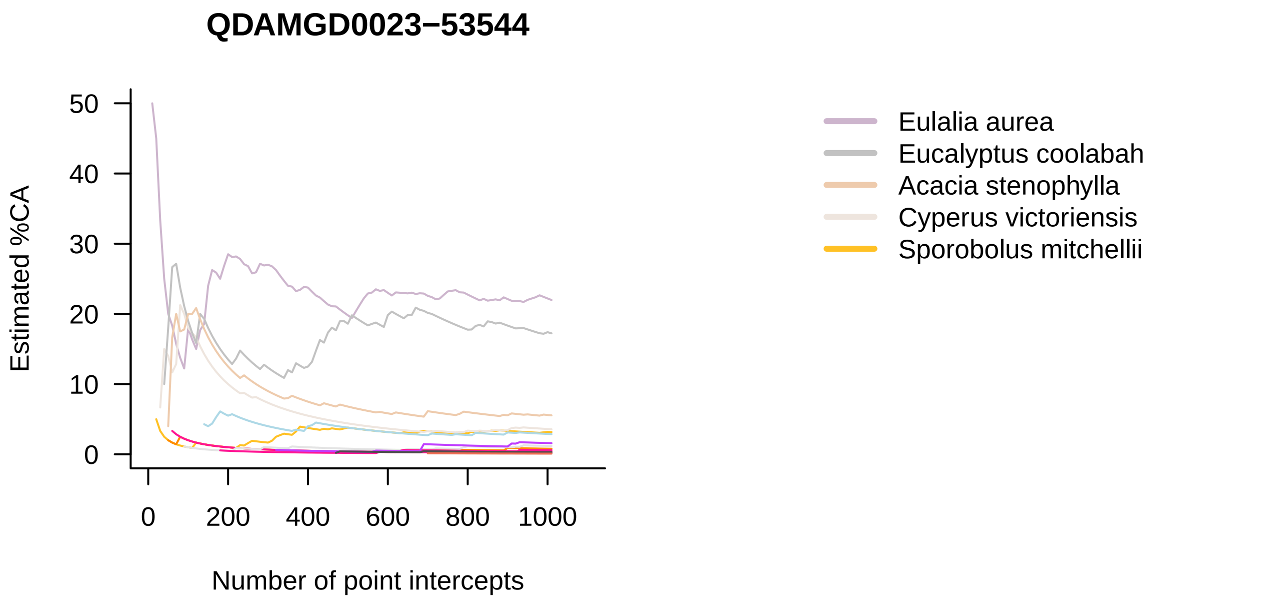
QDAMGD0023−53544
Estimated %CA
50
40
30
20
10
0
0
200
400
600
800
1000
Number of point intercepts
Eulalia aurea
Eucalyptus coolabah
Acacia stenophylla
Cyperus victoriensis
Sporobolus mitchellii

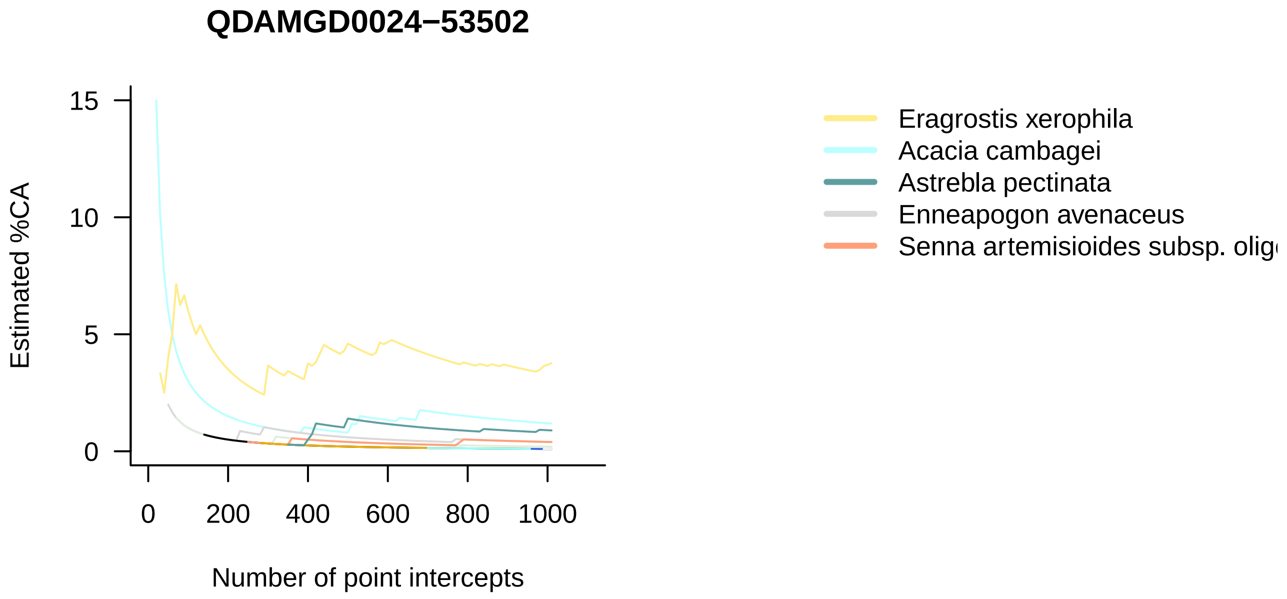
QDAMGD0024−53502
Estimated %CA
15
10
5
0
0
200
400
600
800
1000
Number of point intercepts
Eragrostis xerophila
Acacia cambagei
Astrebla pectinata
Enneapogon avenaceus
Senna artemisioides subsp. olig

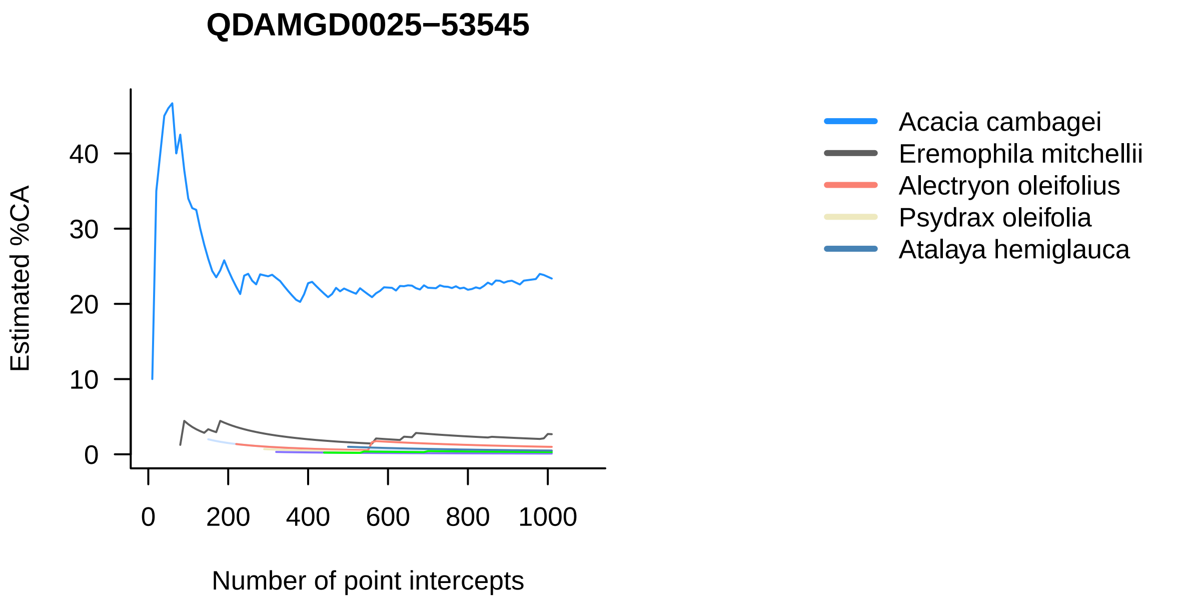
QDAMGD0025−53545
Estimated %CA
0
10
20
30
40
0
200
400
600
800
1000
Number of point intercepts
Acacia cambagei
Eremophila mitchellii
Alectryon oleifolius
Psydrax oleifolia
Atalaya hemiglauca

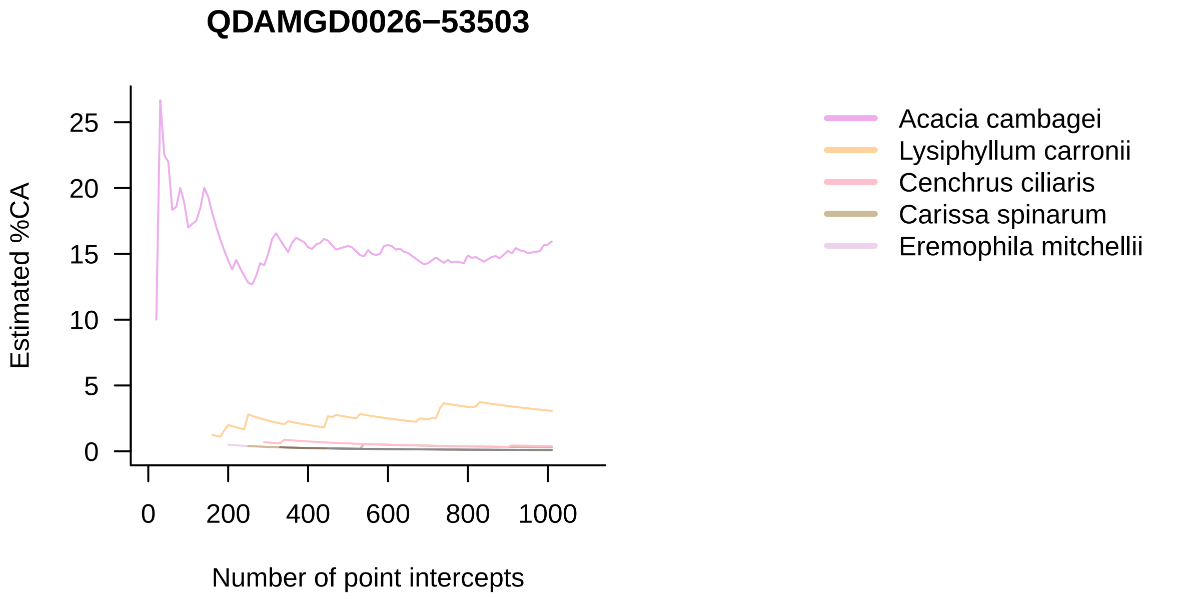
QDAMGD0026−53503
Estimated %CA
0
5
10
15
20
25
0
200
400
600
800
1000
Number of point intercepts
Acacia cambagei
Lysiphyllum carronii
Cenchrus ciliaris
Carissa spinarum
Eremophila mitchellii

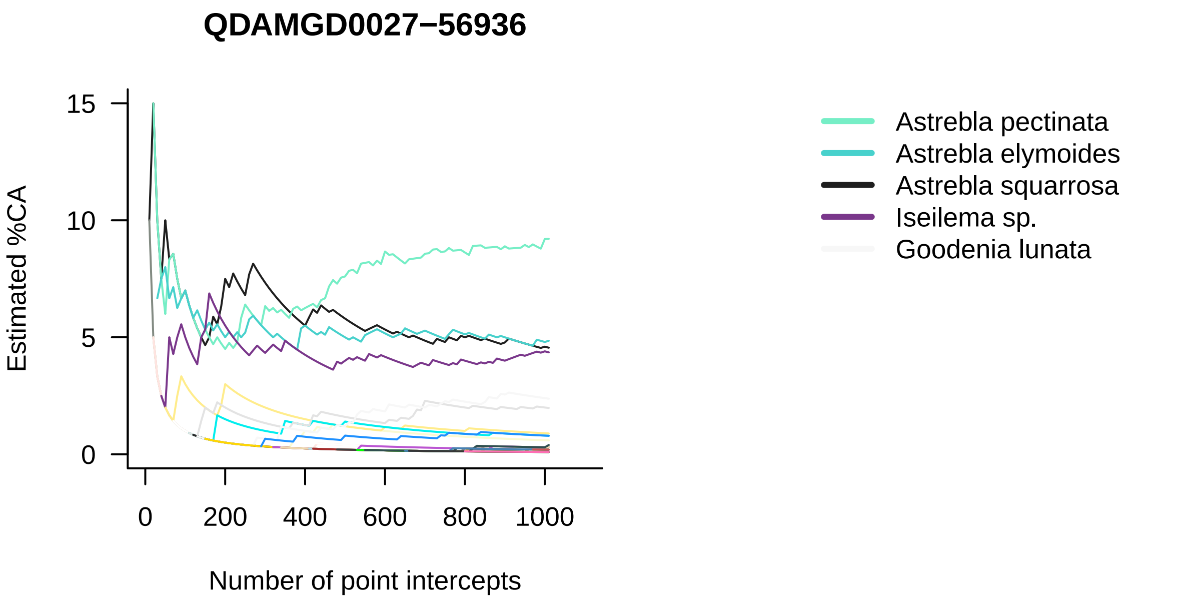
QDAMGD0027−56936
Estimated %CA
0
5
10
15
0
200
400
600
800
1000
Number of point intercepts
Astrebla pectinata
Astrebla elymoides
Astrebla squarrosa
Iseilema sp.
Goodenia lunata

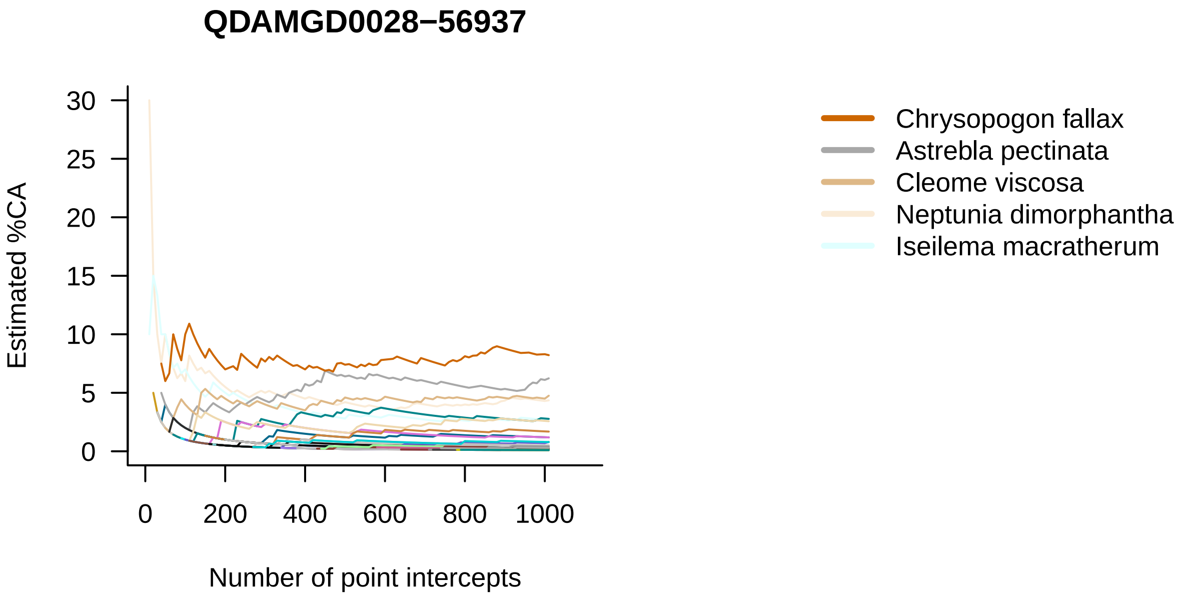
QDAMGD0028−56937
Estimated %CA
0
5
10
15
20
25
30
0
200
400
600
800
1000
Number of point intercepts
Chrysopogon fallax
Astrebla pectinata
Cleome viscosa
Neptunia dimorphantha
Iseilema macratherum

## QDAMII0001−53504

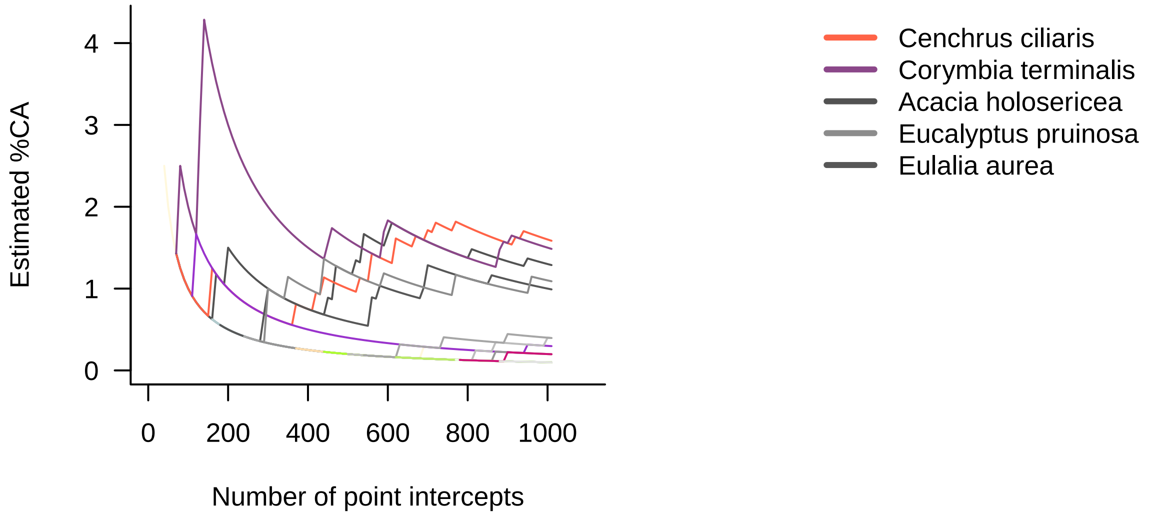


## QDAMII0002−53546

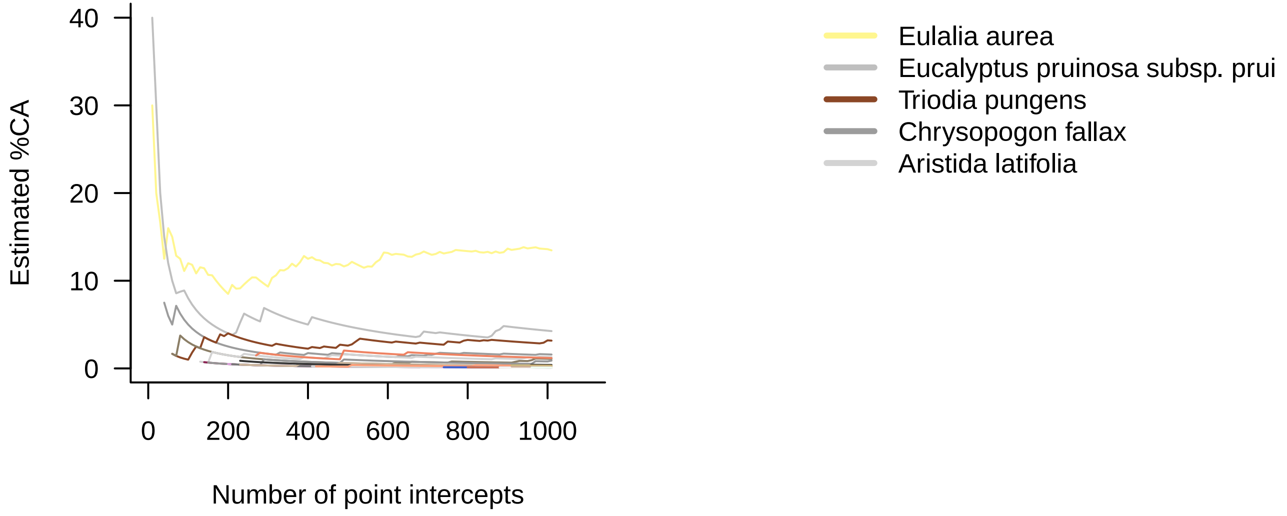

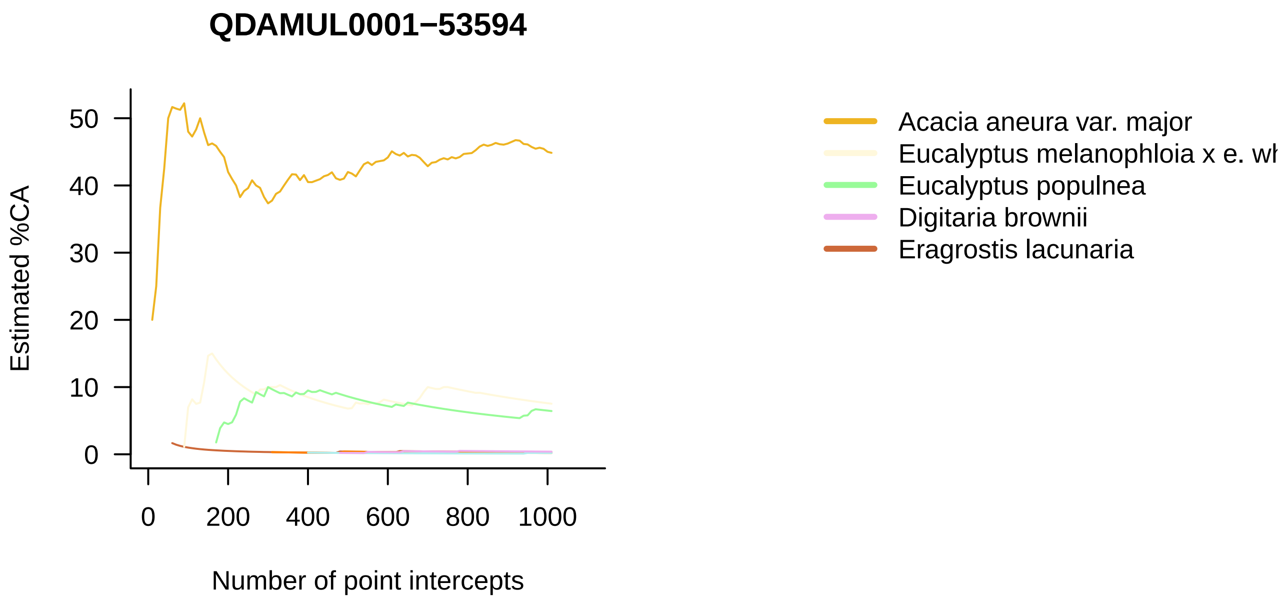
QDAMUL0001−53594
Estimated %CA
0
10
20
30
40
50
0
200
400
600
800
1000
Number of point intercepts
Acacia aneura var. major
Eucalyptus melanophloia x e. wh
Eucalyptus populnea
Digitaria brownii
Eragrostis lacunaria

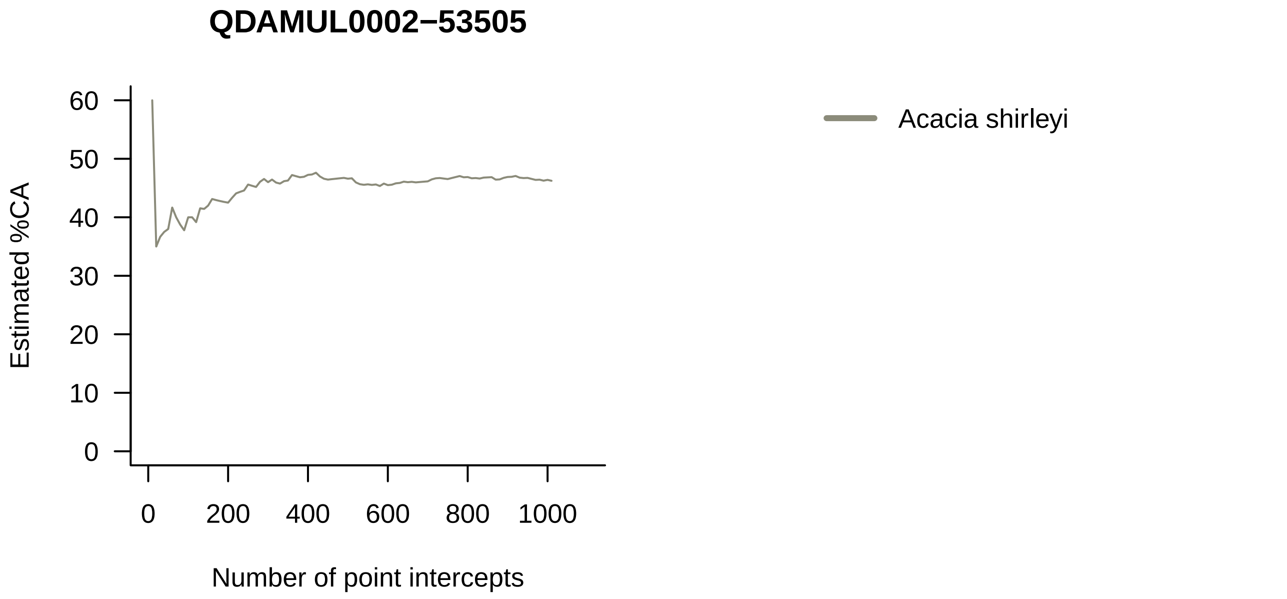
QDAMUL0002−53505
Estimated %CA
0
10
20
30
40
50
60
0
200
400
600
800
1000
Number of point intercepts
Acacia shirleyi

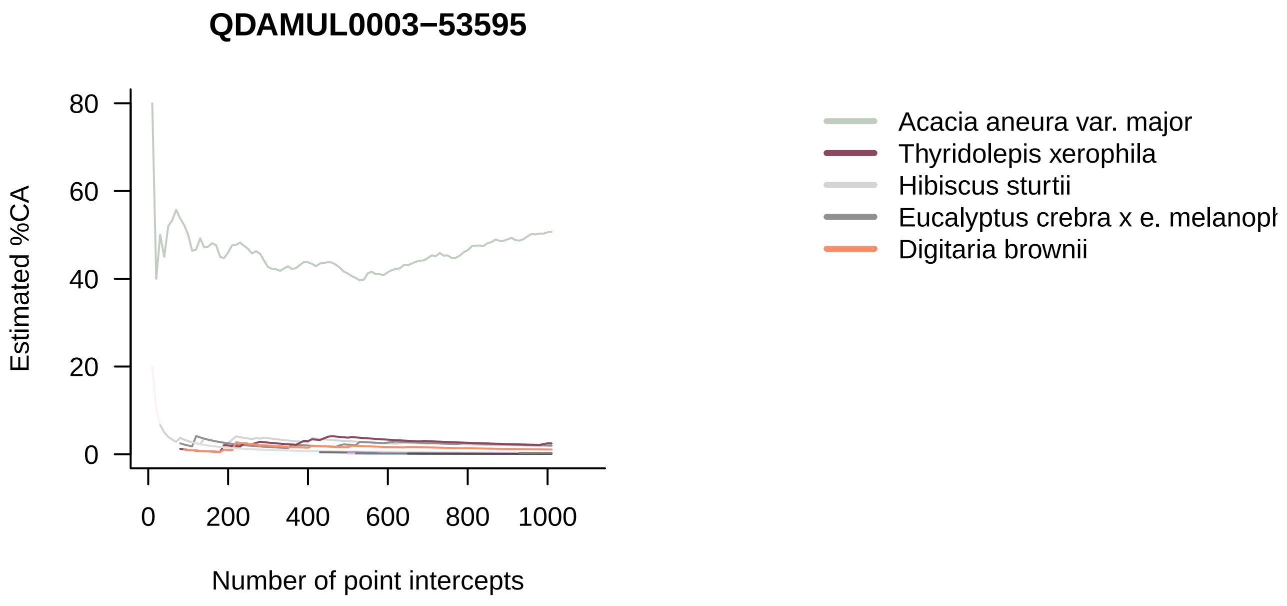

QDAMUL0003−53595
Estimated %CA
0
20
40
60
80
0
200
400
600
800
1000
Number of point intercepts
Acacia aneura var. major
Thyridolepis xerophila
Hibiscus sturtii
Eucalyptus crebra x e. melanopl
Digitaria brownii


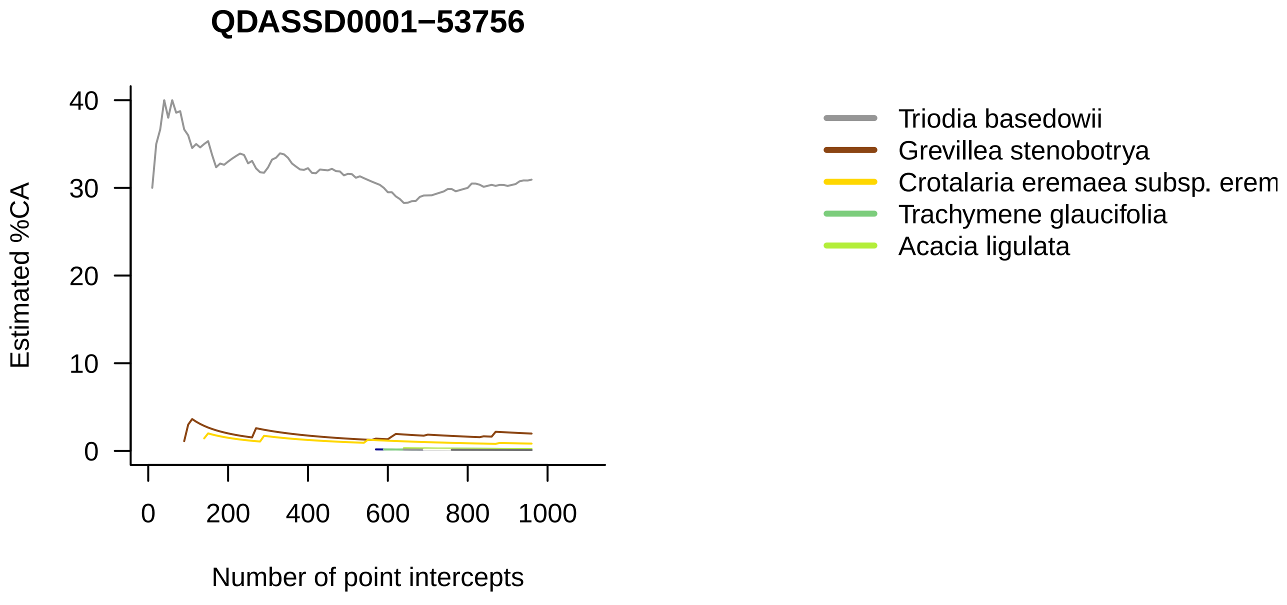

QDASSD0001−53756
Estimated %CA
0
10
20
30
40
0
200
400
600
800
1000
Number of point intercepts
Triodia basedowii
Grevillea stenobotrya
Crotalaria eremaea subsp. erem
Trachymene glaucifolia
Acacia ligulata

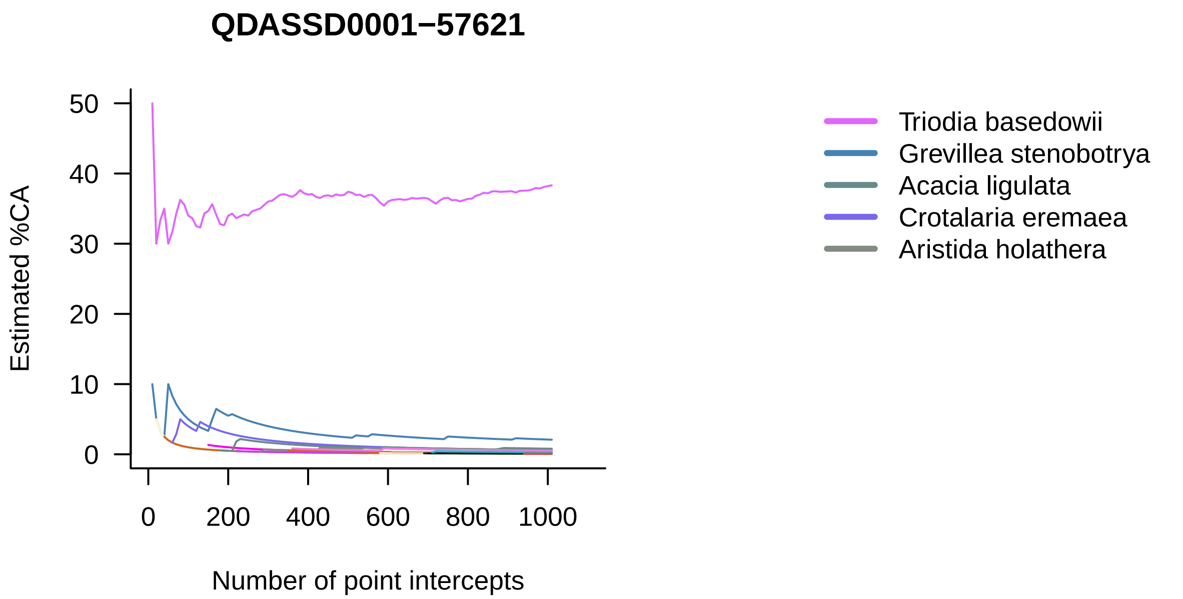
QDASSD0001−57621
Estimated %CA
Number of point intercepts
Triodia basedowii
Grevillea stenobotrya
Acacia ligulata
Crotalaria eremaea
Aristida holathera
0
10
20
30
40
50
0
200
400
600
800
1000

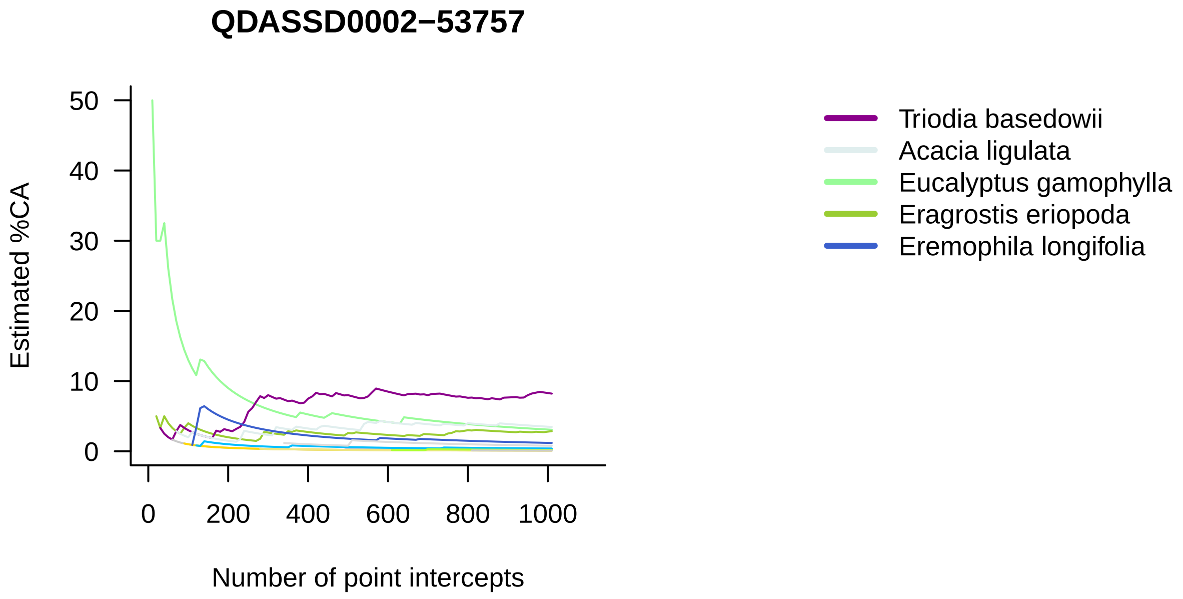
QDASSD0002−53757
Estimated %CA
Number of point intercepts
Triodia basedowii
Acacia ligulata
Eucalyptus gamophylla
Eragrostis eriopoda
Eremophila longifolia
0
10
20
30
40
50
0
200
400
600
800
1000

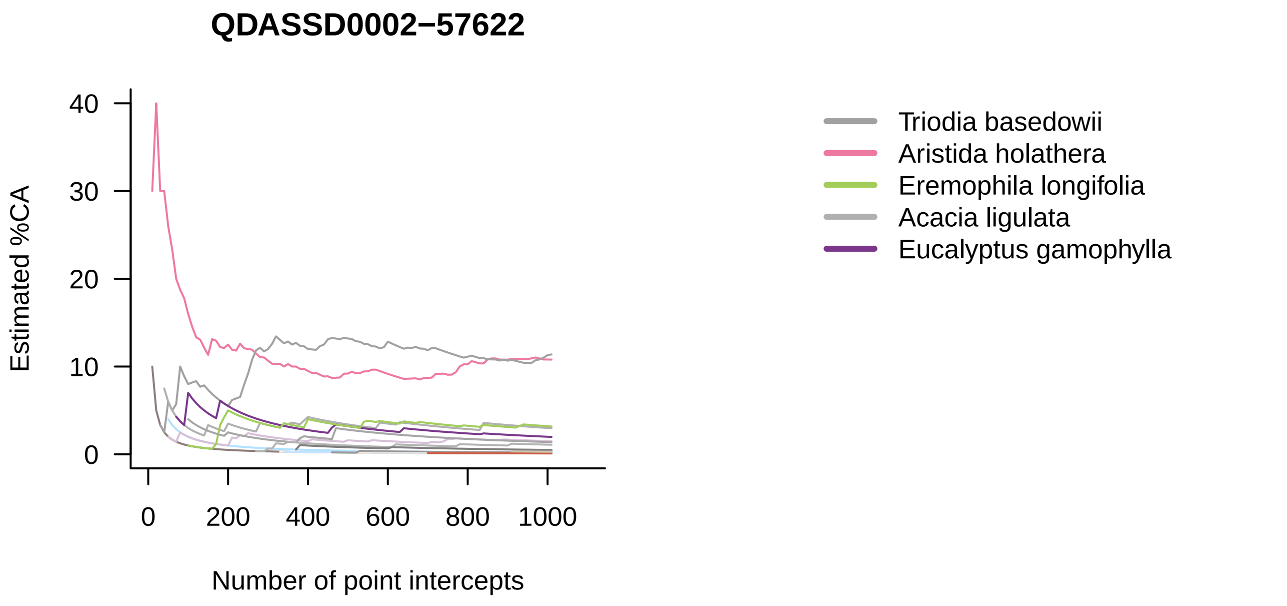
QDASSD0002−57622
Estimated %CA
0
10
20
30
40
0
200
400
600
800
1000
Number of point intercepts
Triodia basedowii
Aristida holathera
Eremophila longifolia
Acacia ligulata
Eucalyptus gamophylla

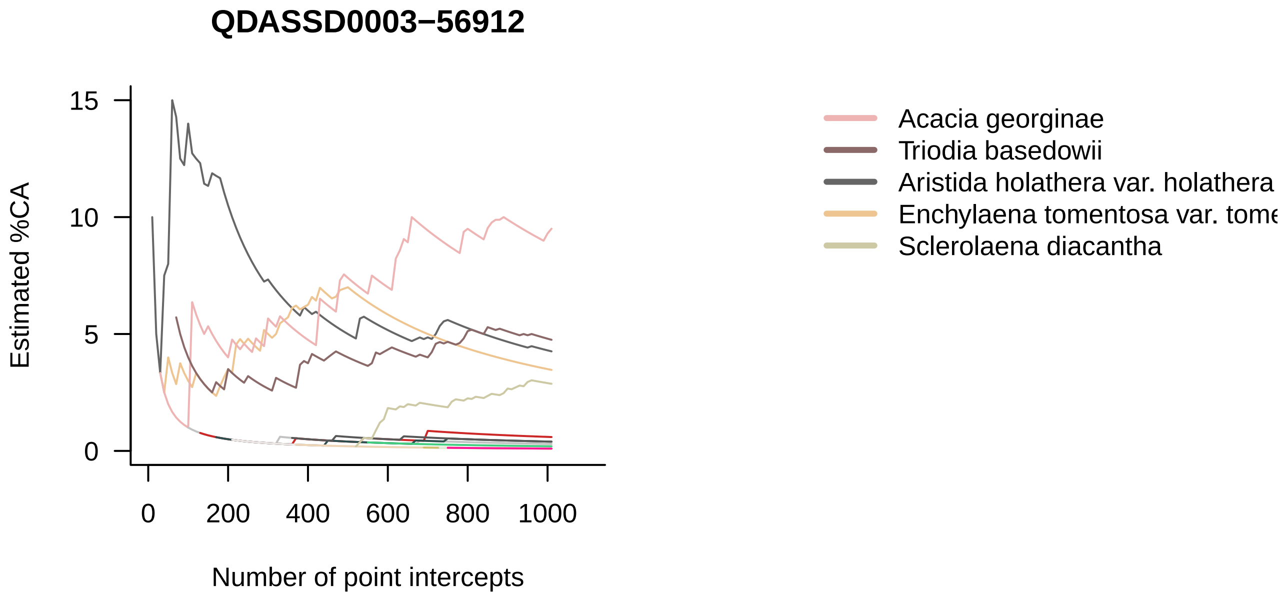
QDASSD0003−56912
Estimated %CA
0
5
10
15
0
200
400
600
800
1000
Number of point intercepts
Acacia georginae
Triodia basedowii
Aristida holathera var. holathera
Enchylaena tomentosa var. tome
Sclerolaena diacantha

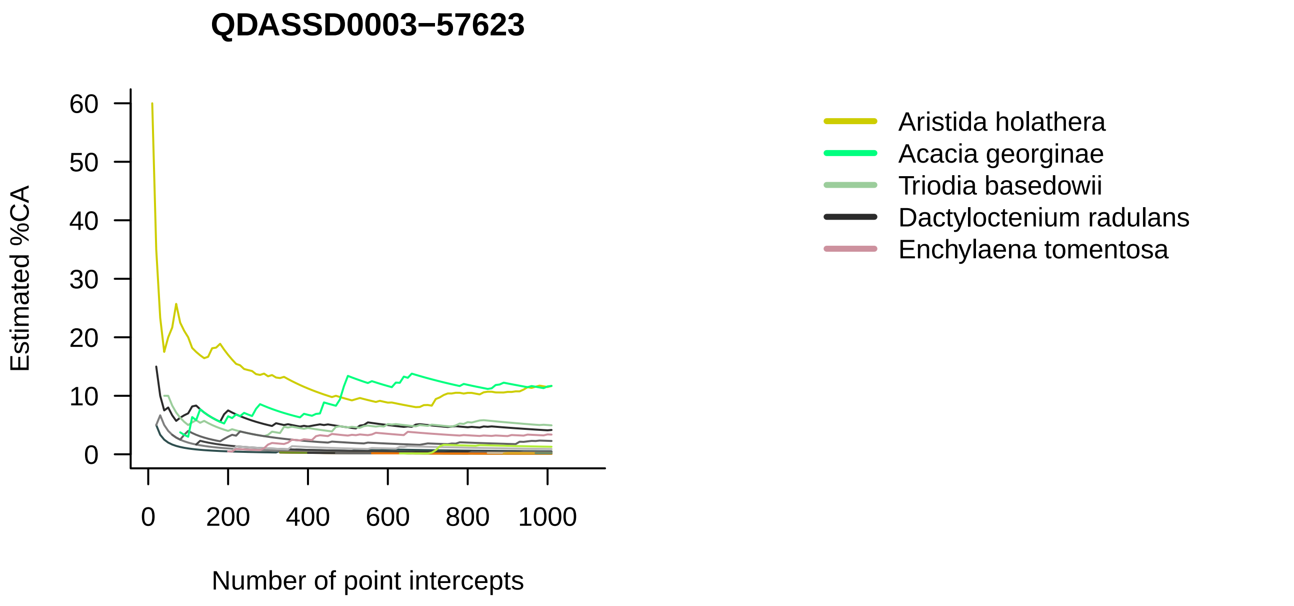
QDASSD0003−57623
Estimated %CA
60
50
40
30
20
10
0
0
200
400
600
800
1000
Number of point intercepts
Aristida holathera
Acacia georginae
Triodia basedowii
Dactyloctenium radulans
Enchylaena tomentosa

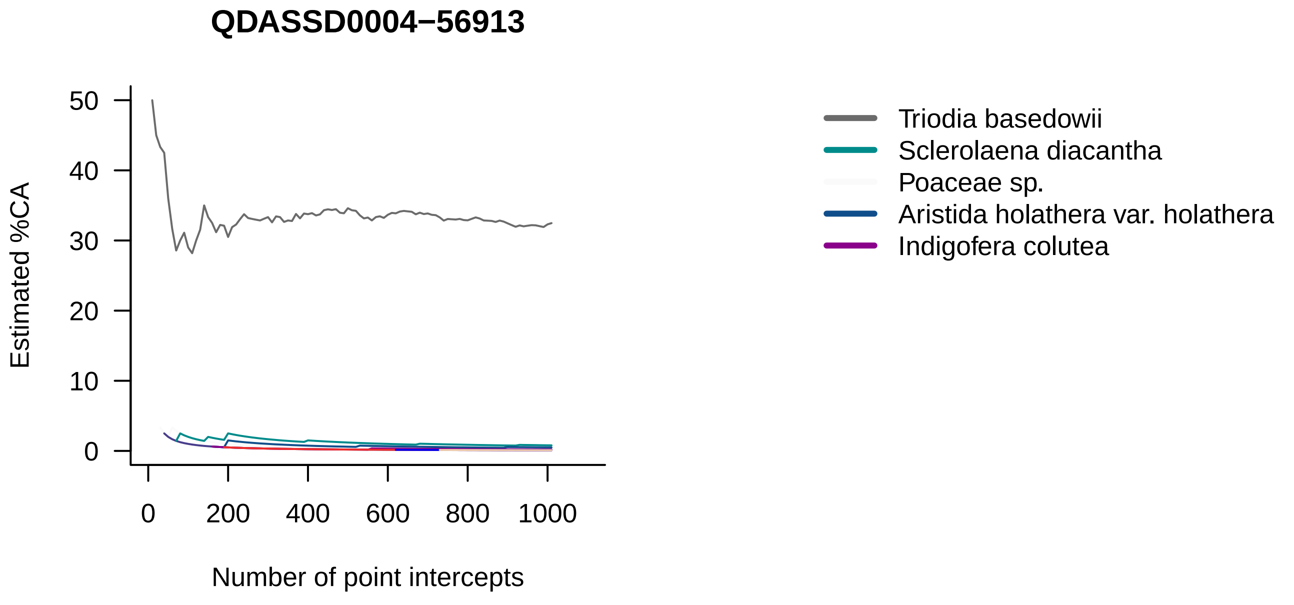
QDASSD0004−56913
Estimated %CA
50
40
30
20
10
0
0
200
400
600
800
1000
Number of point intercepts
Triodia basedowii
Sclerolaena diacantha
Poaceae sp.
Aristida holathera var. holathera
Indigofera colutea

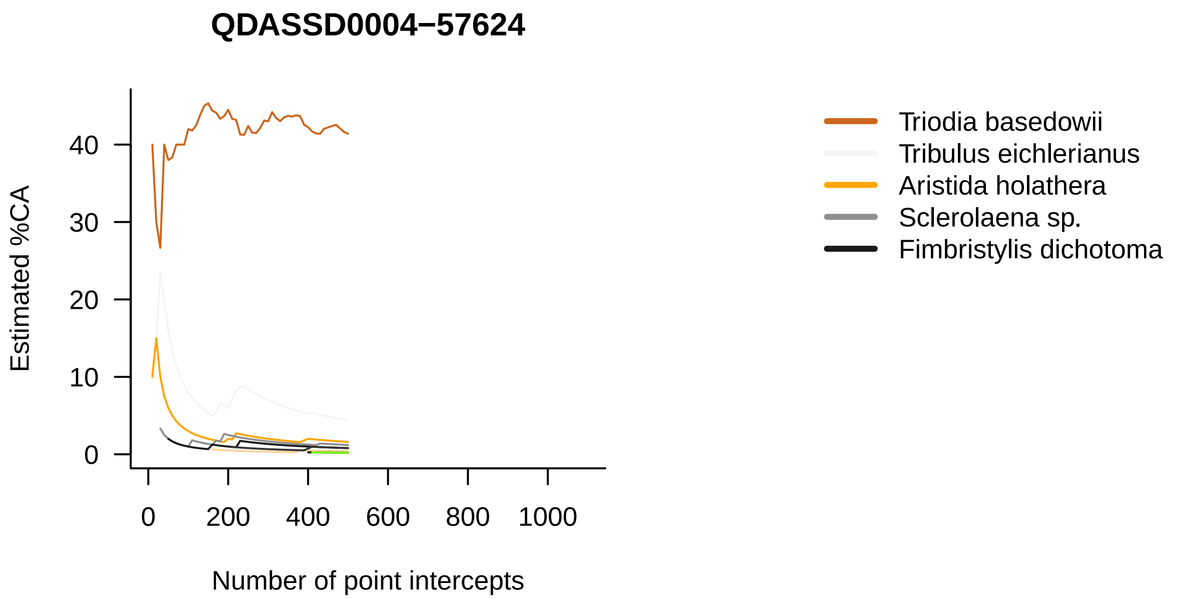
QDASSD0004−57624
Estimated %CA
0
10
20
30
40
0
200
400
600
800
1000
Number of point intercepts
Triodia basedowii
Tribulus eichlerianus
Aristida holathera
Sclerolaena sp.
Fimbristylis dichotoma

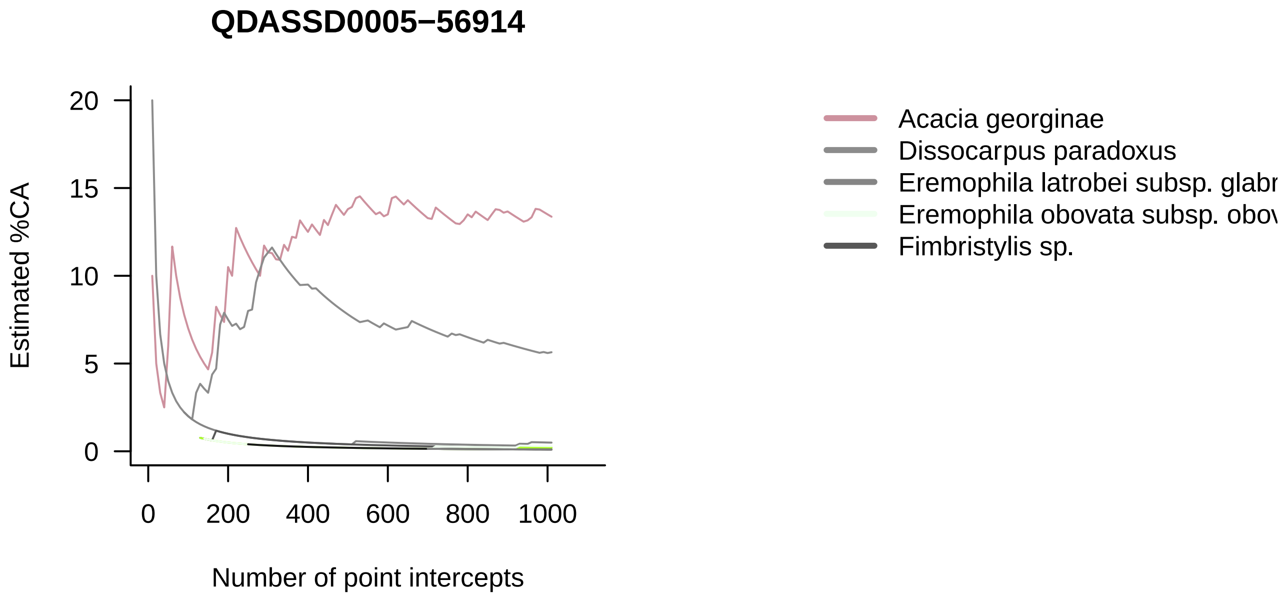
QDASSD0005−56914
Estimated %CA
0
5
10
15
20
0
200
400
600
800
1000
Number of point intercepts
Acacia georginae
Dissocarpus paradoxus
Eremophila latrobei subsp. glabr
Eremophila obovata subsp. obov
Fimbristylis sp.

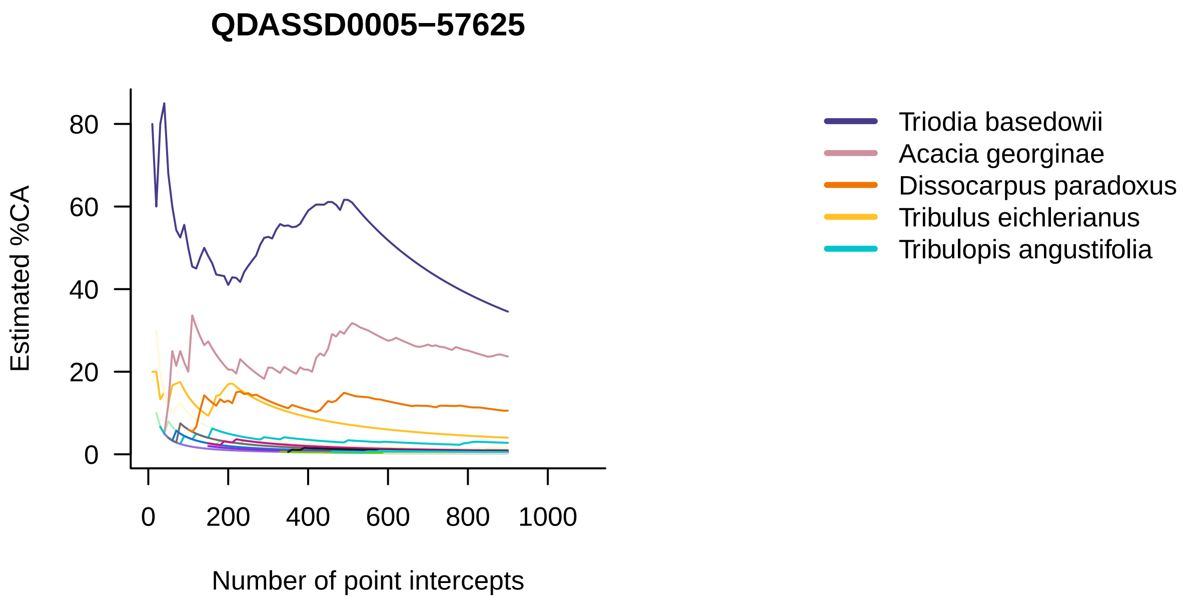
QDASSD0005−57625
Estimated %CA
0
20
40
60
80
0
200
400
600
800
1000
Number of point intercepts
Triodia basedowii
Acacia georginae
Dissocarpus paradoxus
Tribulus eichlerianus
Tribulopis angustifolia

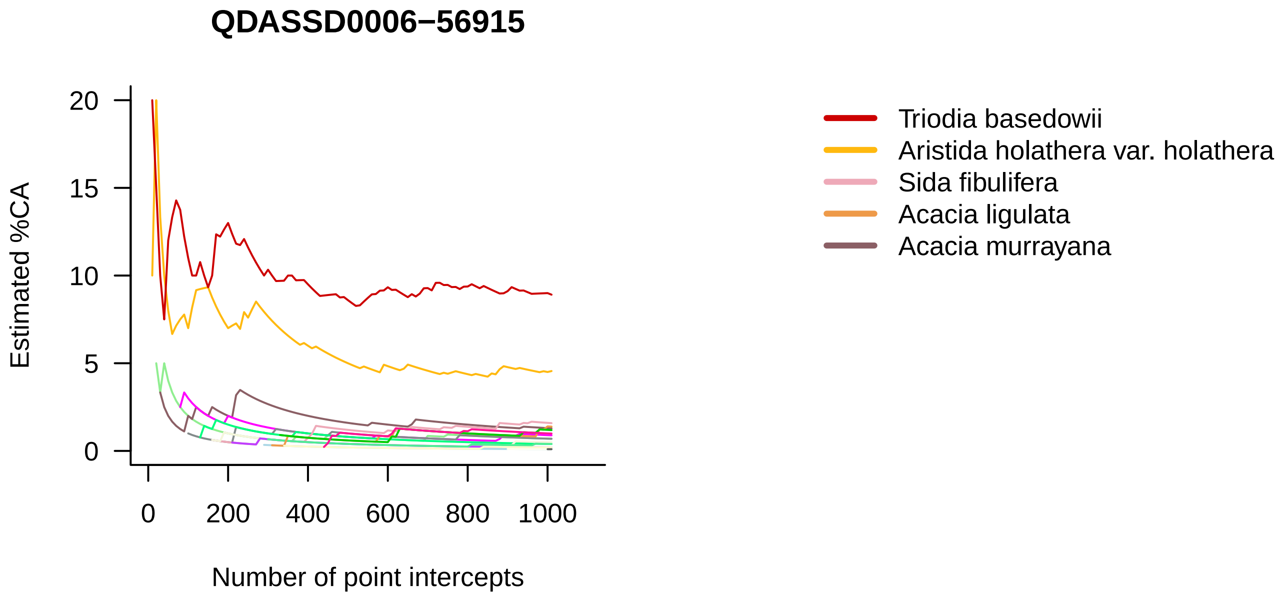
QDASSD0006−56915
Estimated %CA
0
5
10
15
20
0
200
400
600
800
1000
Number of point intercepts
Triodia basedowii
Aristida holathera var. holathera
Sida fibulifera
Acacia ligulata
Acacia murrayana

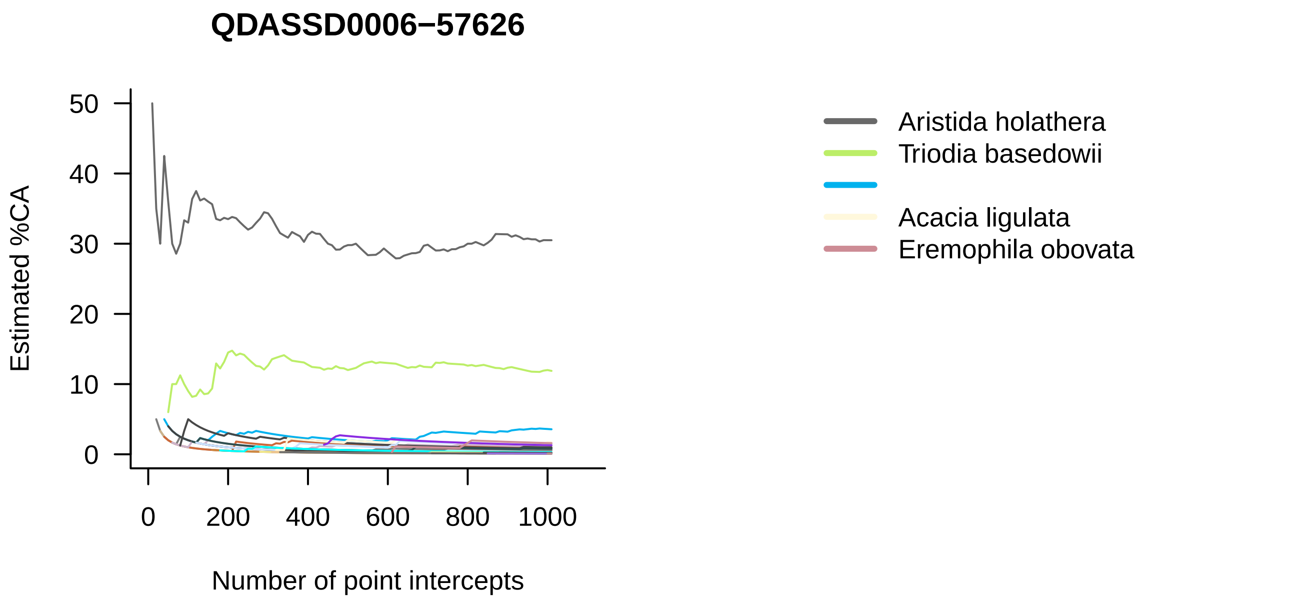
QDASSD0006−57626
Estimated %CA
Number of point intercepts
0
10
20
30
40
50
0
200
400
600
800
1000
Aristida holathera
Triodia basedowii
Acacia ligulata
Eremophila obovata

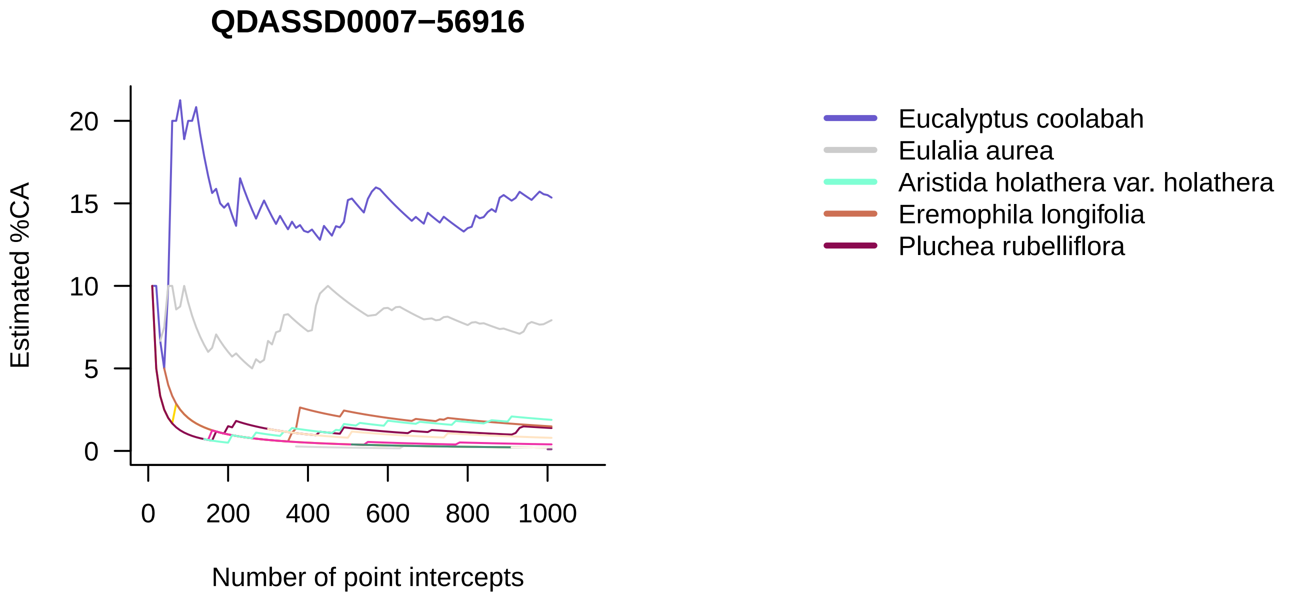
QDASSD0007−56916
Estimated %CA
Number of point intercepts
0
5
10
15
20
0
200
400
600
800
1000
Eucalyptus coolabah
Eulalia aurea
Aristida holathera var. holathera
Eremophila longifolia
Pluchea rubelliflora

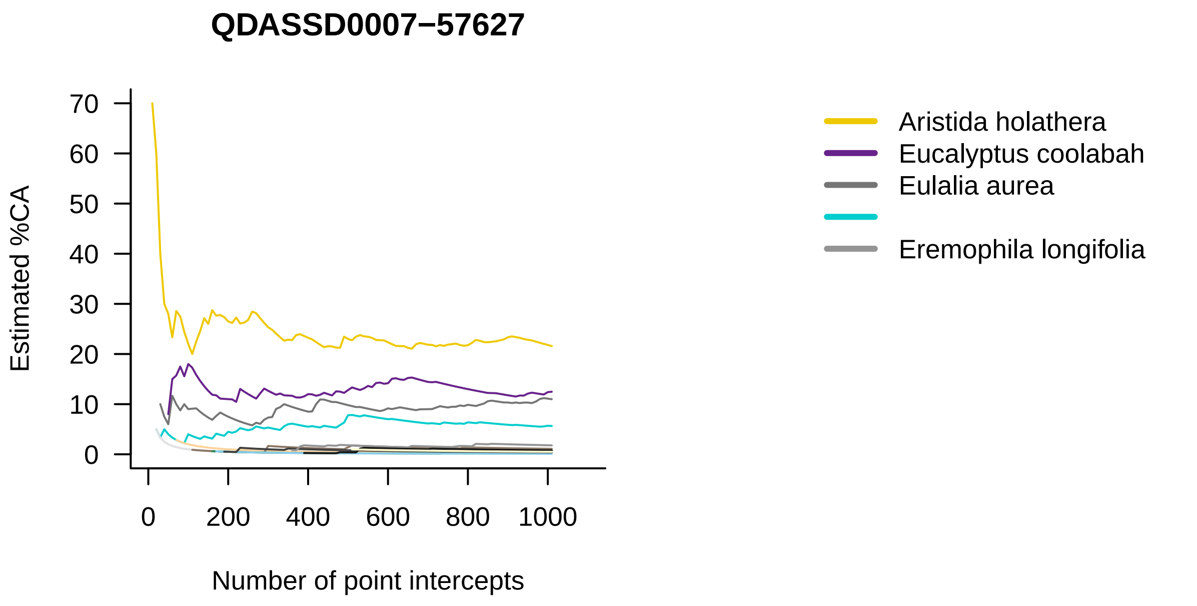
QDASSD0007−57627
Estimated %CA
70
60
50
40
30
20
10
0
0
200
400
600
800
1000
Number of point intercepts
Aristida holathera
Eucalyptus coolabah
Eulalia aurea
Eremophila longifolia

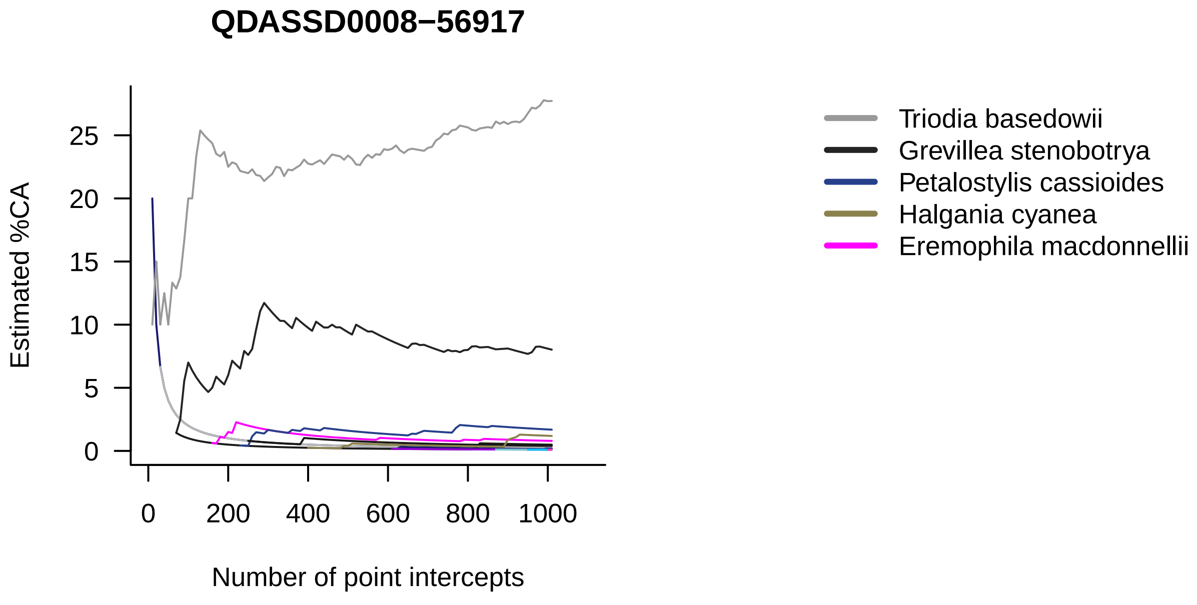
QDASSD0008−56917
Estimated %CA
25
20
15
10
5
0
0
200
400
600
800
1000
Number of point intercepts
Triodia basedowii
Grevillea stenobotrya
Petalostylis cassioides
Halgania cyanea
Eremophila macdonnellii

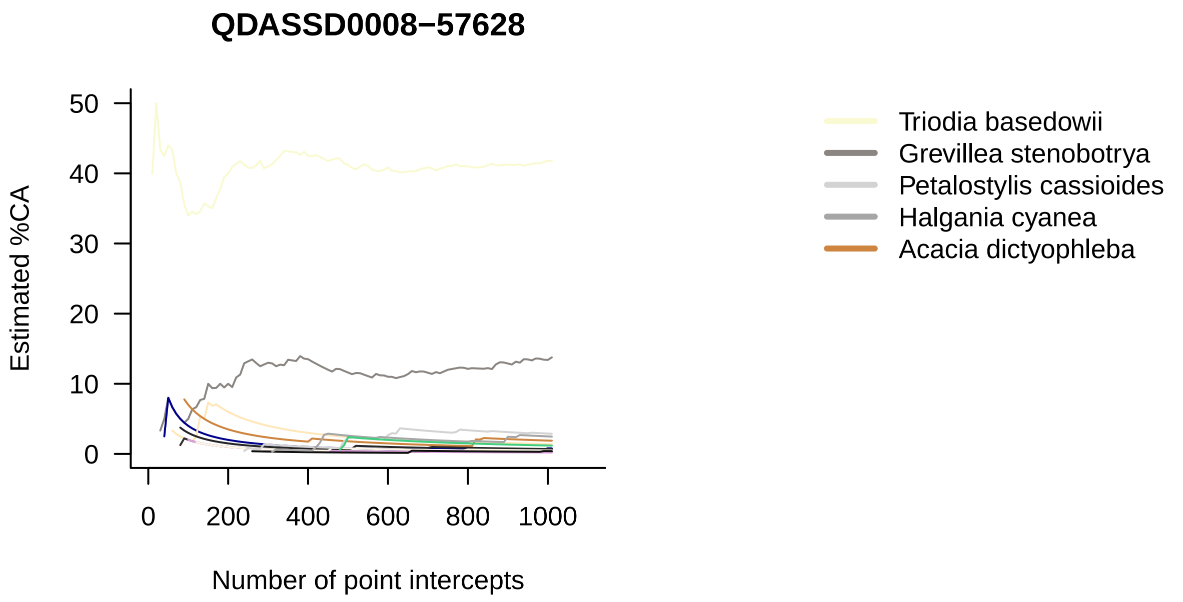
QDASSD0008−57628
Estimated %CA
50
40
30
20
10
0
0
200
400
600
800
1000
Number of point intercepts
Triodia basedowii
Grevillea stenobotrya
Petalostylis cassioides
Halgania cyanea
Acacia dictyophleba

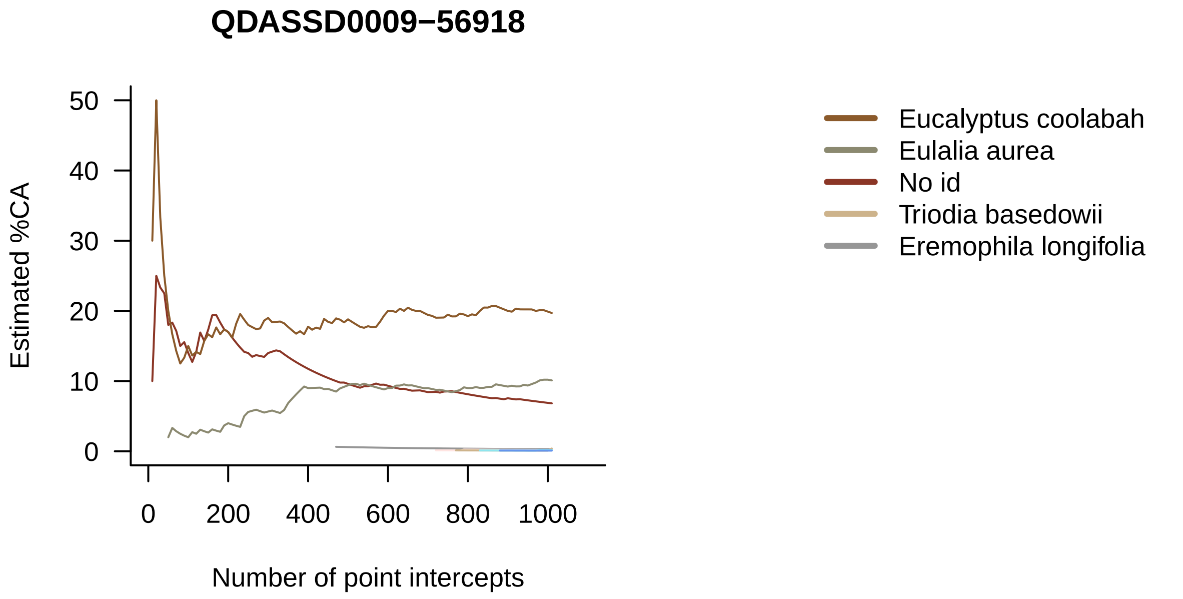
QDASSD0009−56918
Estimated %CA
50
40
30
20
10
0
0
200
400
600
800
1000
Number of point intercepts
Eucalyptus coolabah
Eulalia aurea
No id
Triodia basedowii
Eremophila longifolia

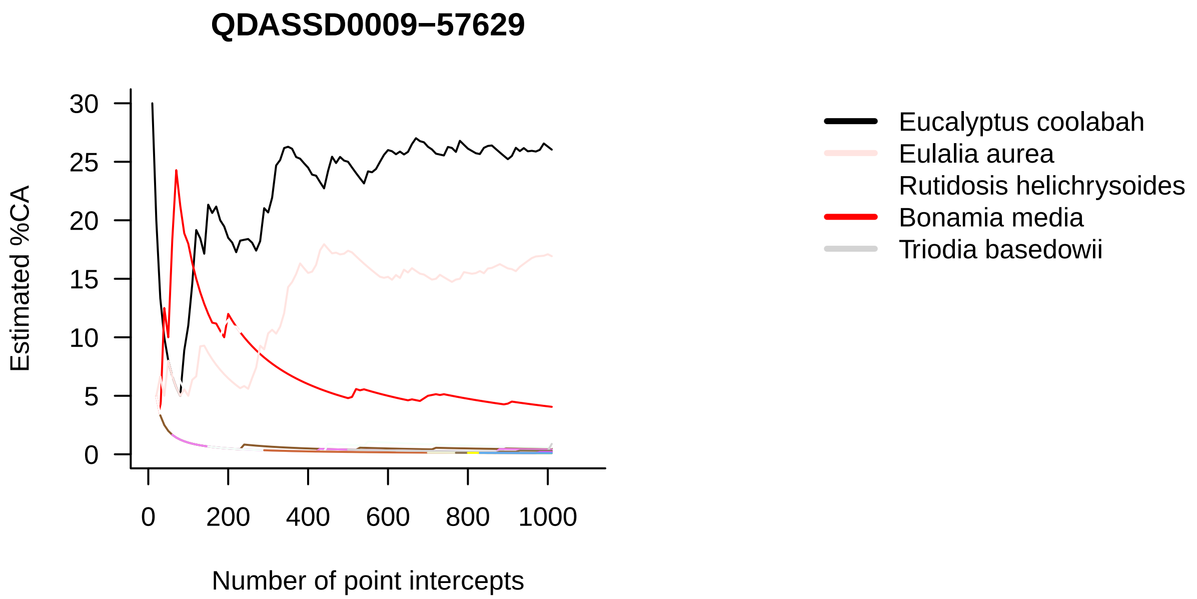

QDASSD0009−57629
Estimated %CA
30
25
20
15
10
5
0
0
200
400
600
800
1000
Number of point intercepts
Eucalyptus coolabah
Eulalia aurea
Rutidosis helichrysoides
Bonamia media
Triodia basedowii


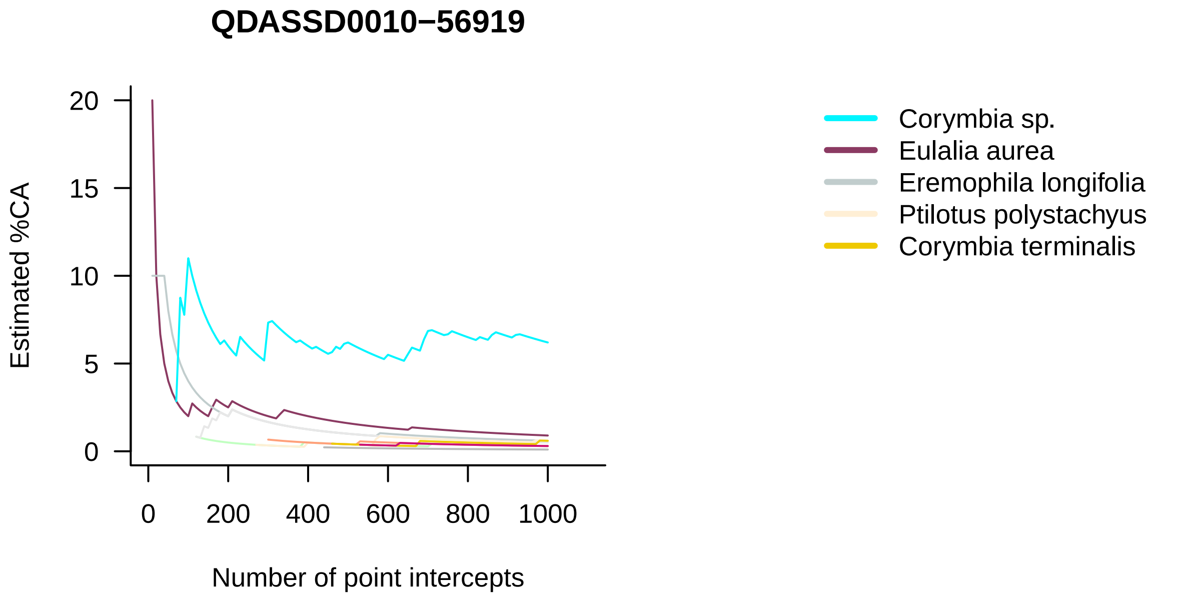

QDASSD0010−56919
Estimated %CA
20
15
10
5
0
0
200
400
600
800
1000
Number of point intercepts
Corymbia sp.
Eulalia aurea
Eremophila longifolia
Ptilotus polystachyus
Corymbia terminalis

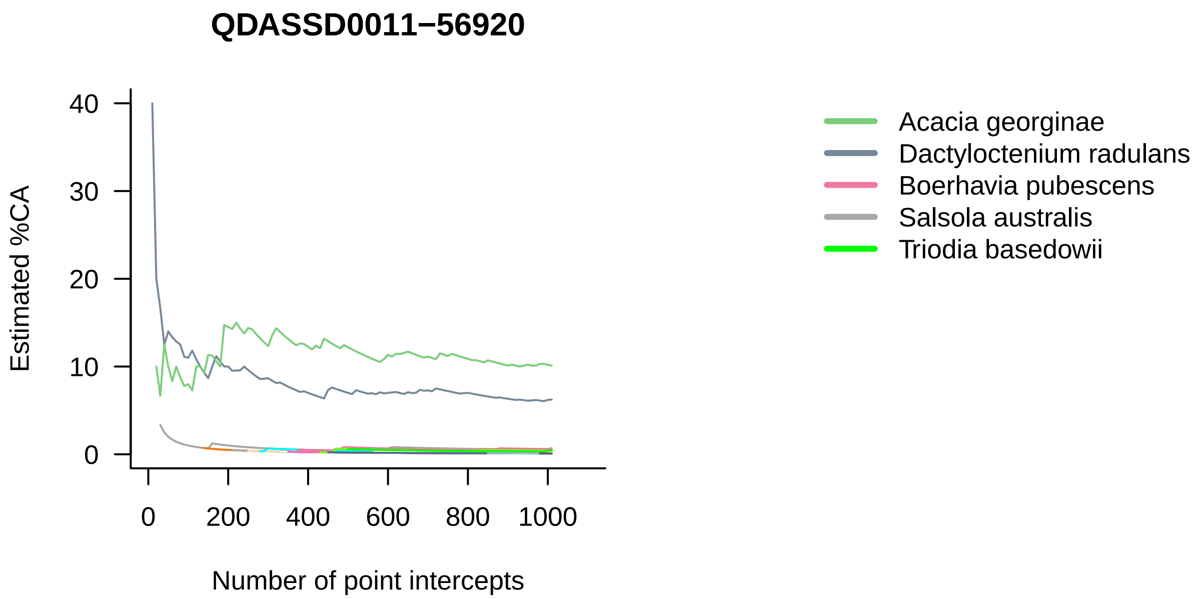
QDASSD0011−56920
Estimated %CA
Number of point intercepts
Acacia georginae
Dactyloctenium radulans
Boerhavia pubescens
Salsola australis
Triodia basedowii

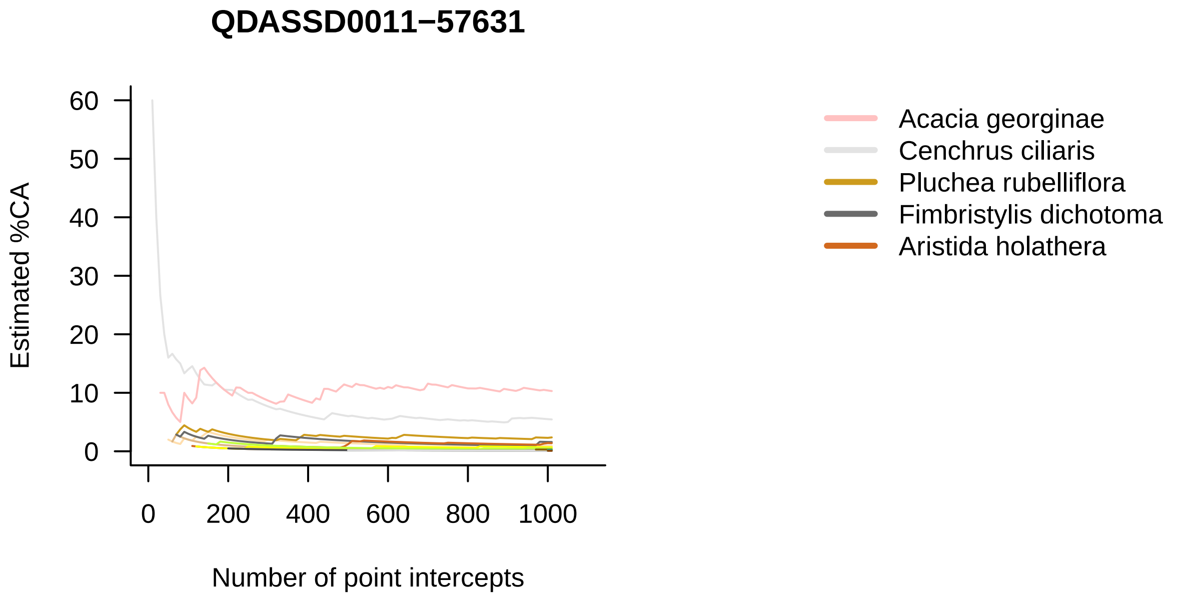
QDASSD0011−57631
Estimated %CA
Number of point intercepts
Acacia georginae
Cenchrus ciliaris
Pluchea rubelliflora
Fimbristylis dichotoma
Aristida holathera

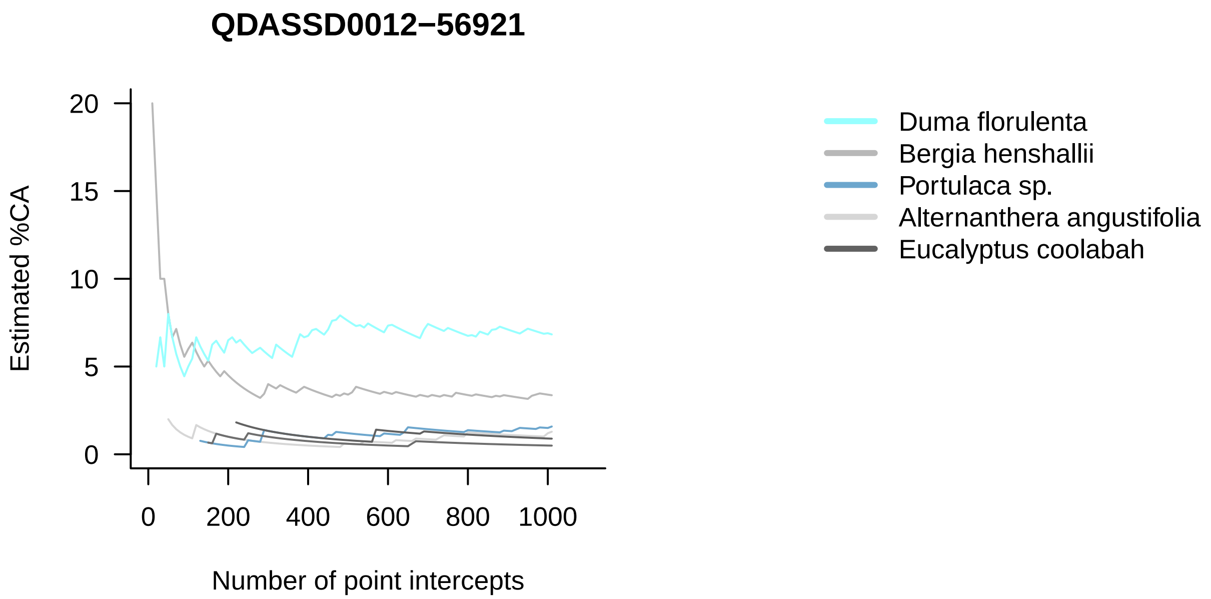
QDASSD0012−56921
Estimated %CA
20
15
10
5
0
0
200
400
600
800
1000
Number of point intercepts
Duma florulenta
Bergia henshallii
Portulaca sp.
Alternanthera angustifolia
Eucalyptus coolabah

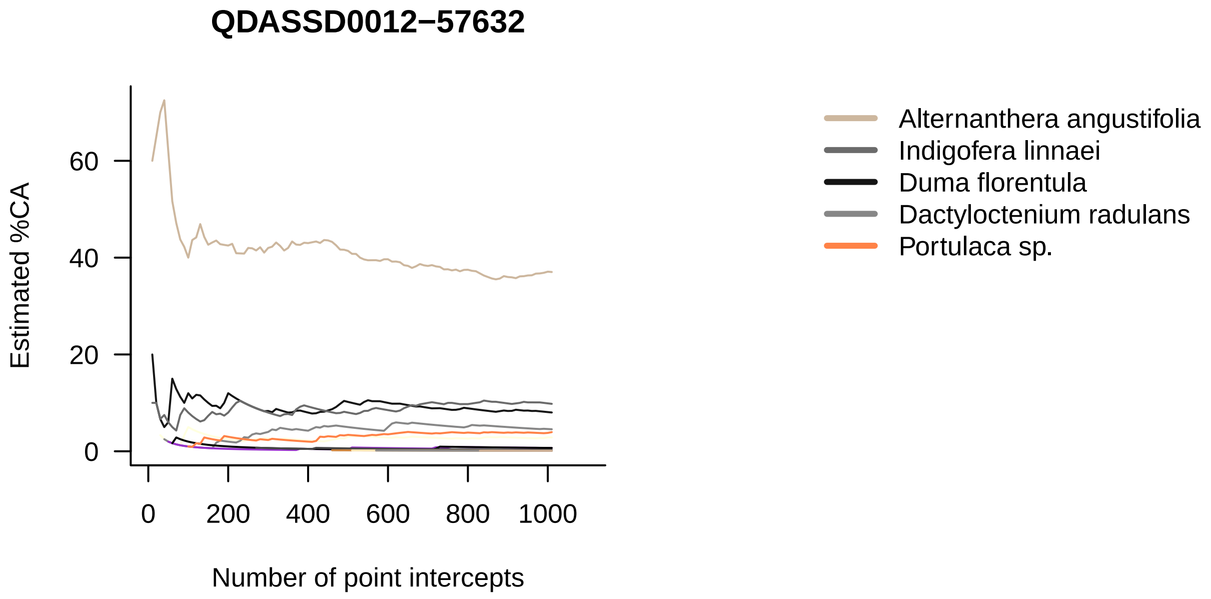
QDASSD0012−57632
Estimated %CA
60
40
20
0
0
200
400
600
800
1000
Number of point intercepts
Alternanthera angustifolia
Indigofera linnaei
Duma florentula
Dactyloctenium radulans
Portulaca sp.

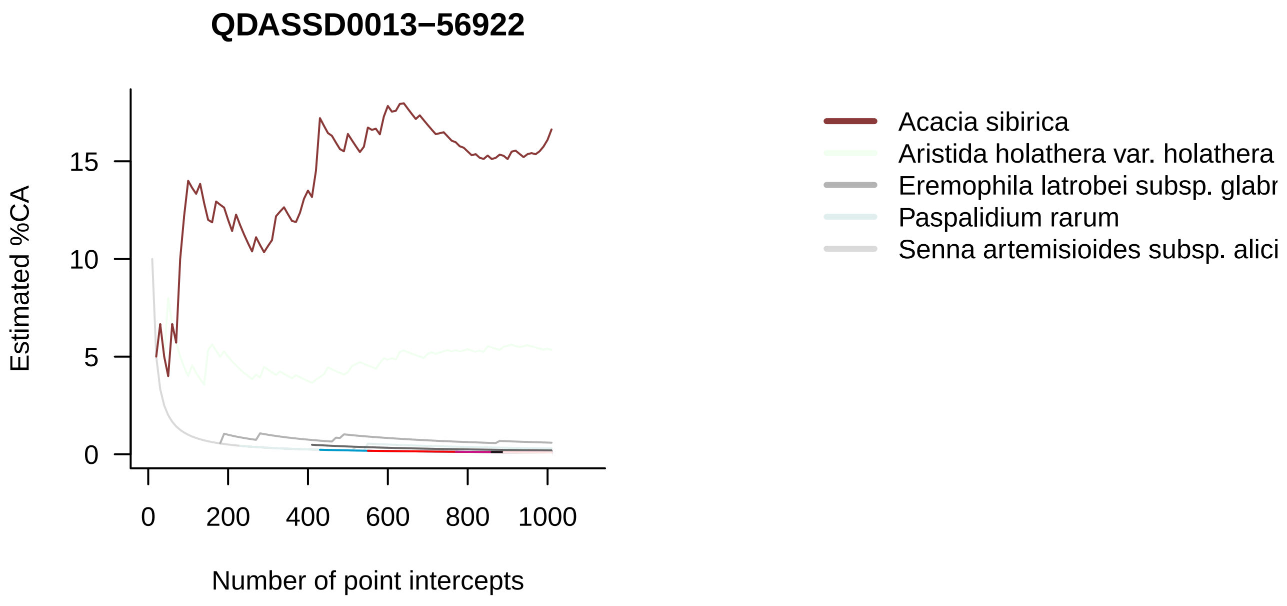
QDASSD0013−56922
Estimated %CA
0
5
10
15
0
200
400
600
800
1000
Number of point intercepts
Acacia sibirica
Aristida holathera var. holathera
Eremophila latrobei subsp. glabr
Paspalidium rarum
Senna artemisioides subsp. alici

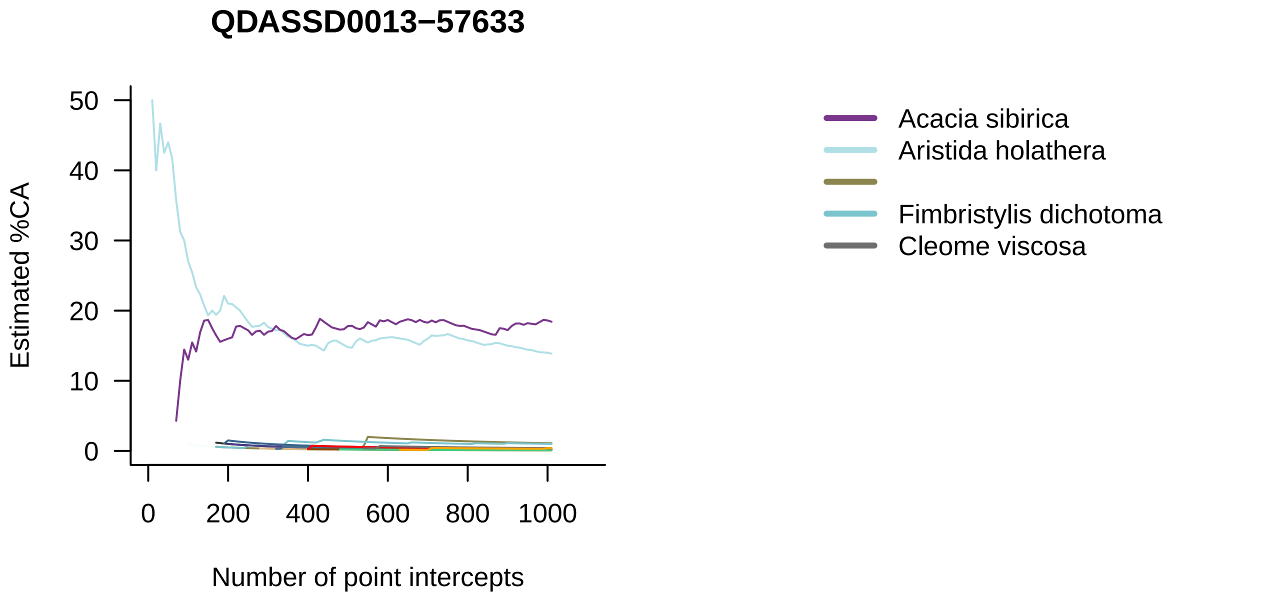
QDASSD0013−57633
Estimated %CA
0
10
20
30
40
50
0
200
400
600
800
1000
Number of point intercepts
Acacia sibirica
Aristida holathera
Fimbristylis dichotoma
Cleome viscosa

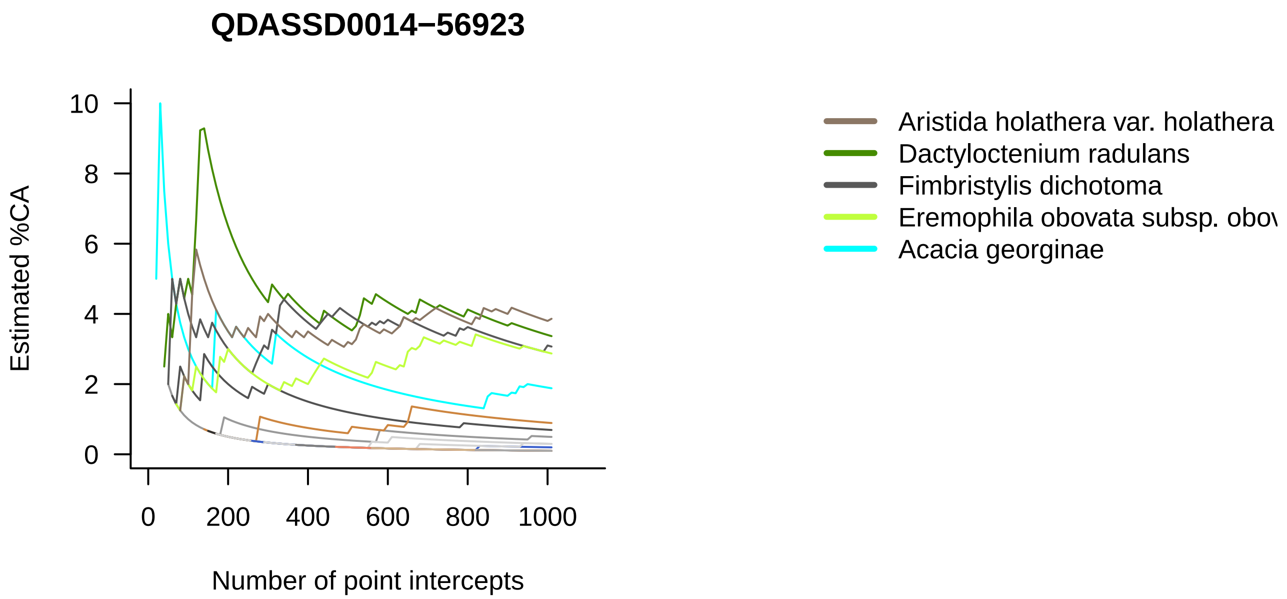
QDASSD0014−56923
Estimated %CA
Number of point intercepts
Aristida holathera var. holathera
Dactyloctenium radulans
Fimbristylis dichotoma
Eremophila obovata subsp. obov
Acacia georginae

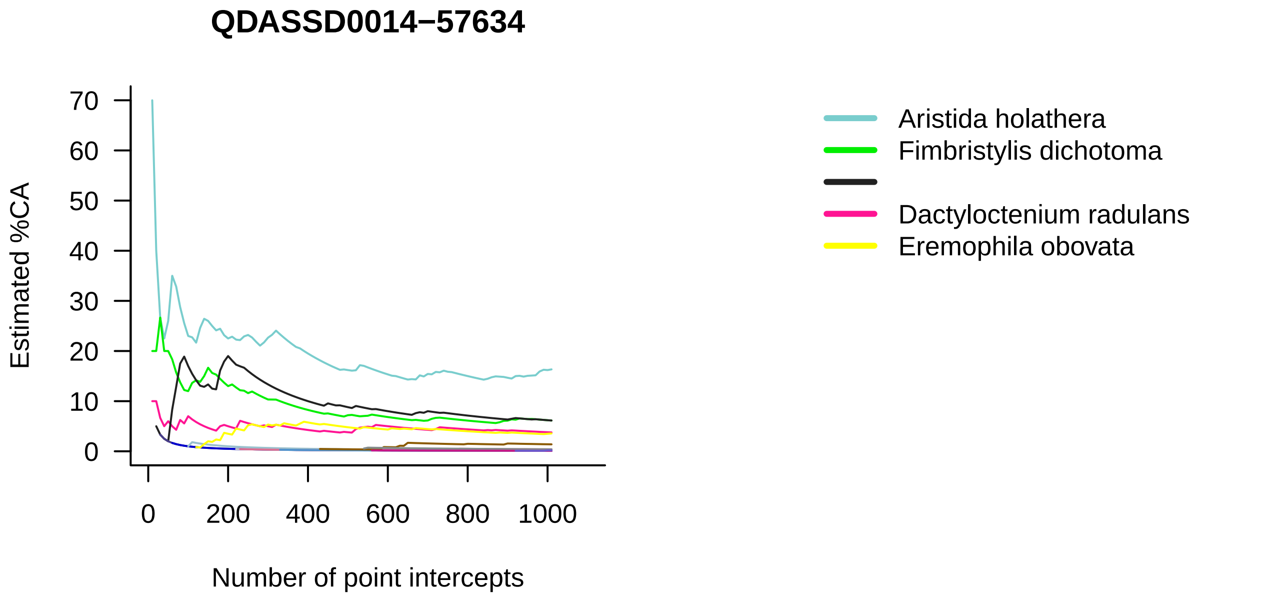
QDASSD0014−57634
Estimated %CA
Number of point intercepts
Aristida holathera
Fimbristylis dichotoma
Dactyloctenium radulans
Eremophila obovata

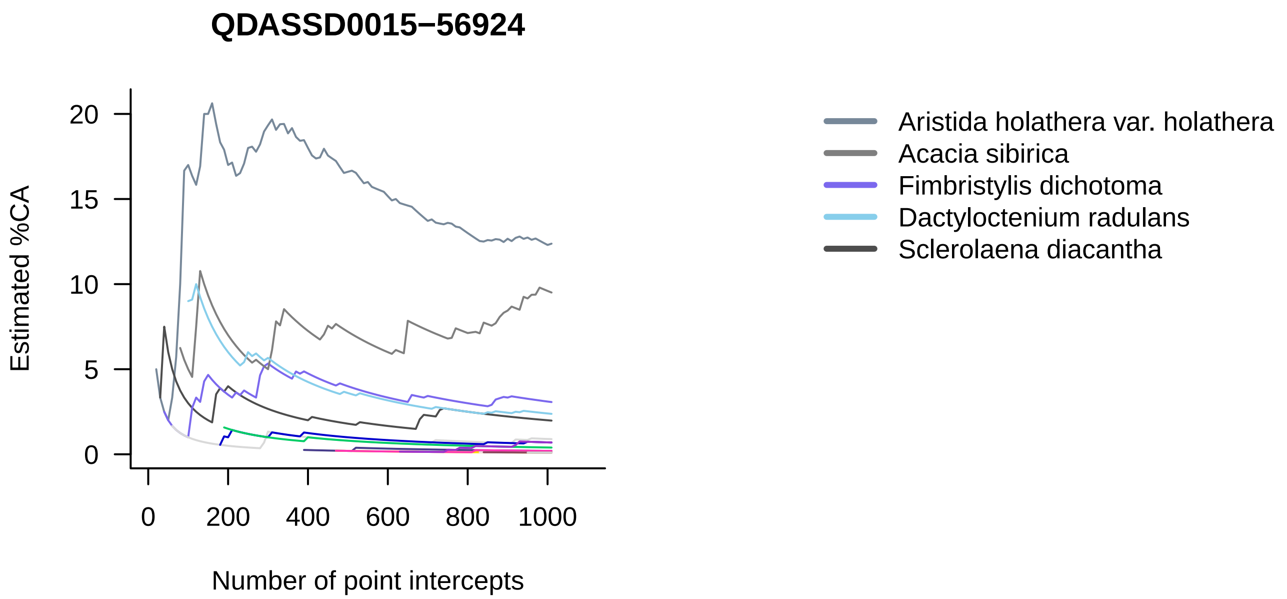
QDASSD0015−56924
Estimated %CA
Number of point intercepts
0
5
10
15
20
0
200
400
600
800
1000
Aristida holathera var. holathera
Acacia sibirica
Fimbristylis dichotoma
Dactyloctenium radulans
Sclerolaena diacantha

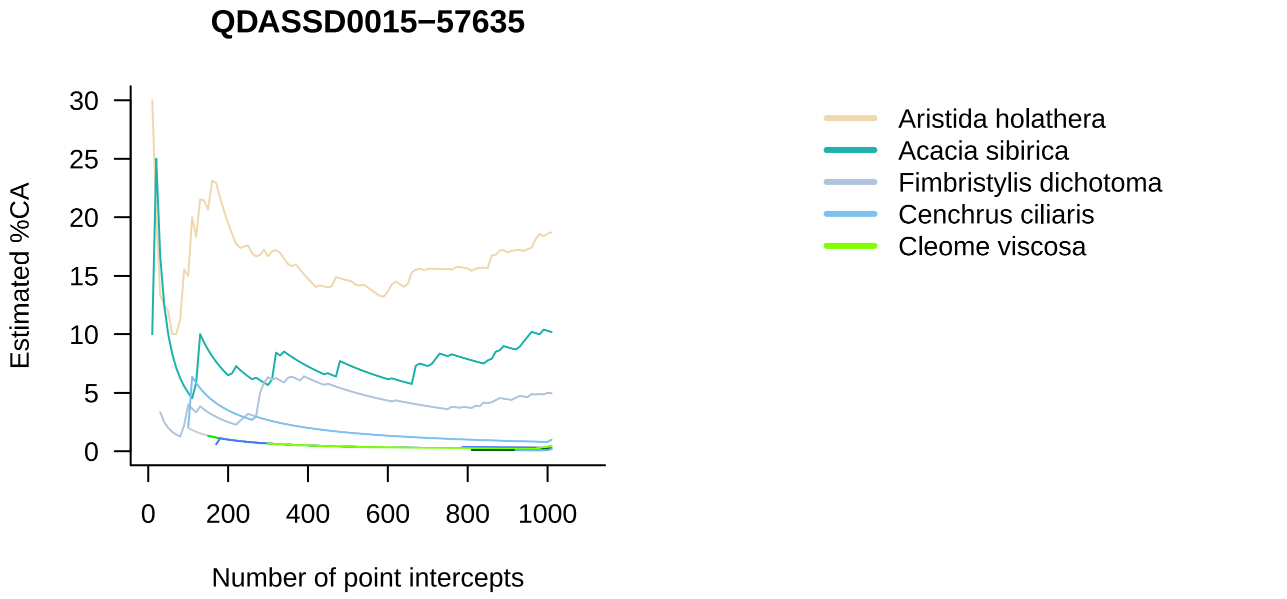
QDASSD0015−57635
Estimated %CA
Number of point intercepts
0
5
10
15
20
25
30
0
200
400
600
800
1000
Aristida holathera
Acacia sibirica
Fimbristylis dichotoma
Cenchrus ciliaris
Cleome viscosa

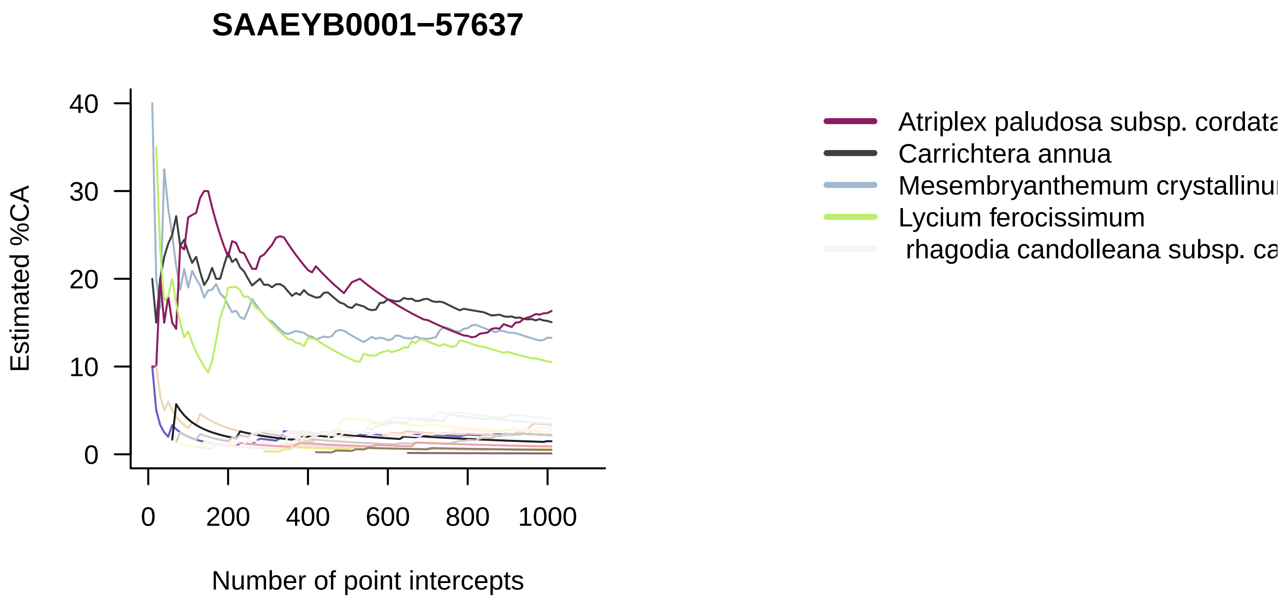
SAAEYB0001−57637
Estimated %CA
Number of point intercepts
0
10
20
30
40
0
200
400
600
800
1000
Atriplex paludosa subsp. cordata
Carrichtera annua
Mesembryanthemum crystallinu
Lycium ferocissimum
rhagodia candolleana subsp. ca

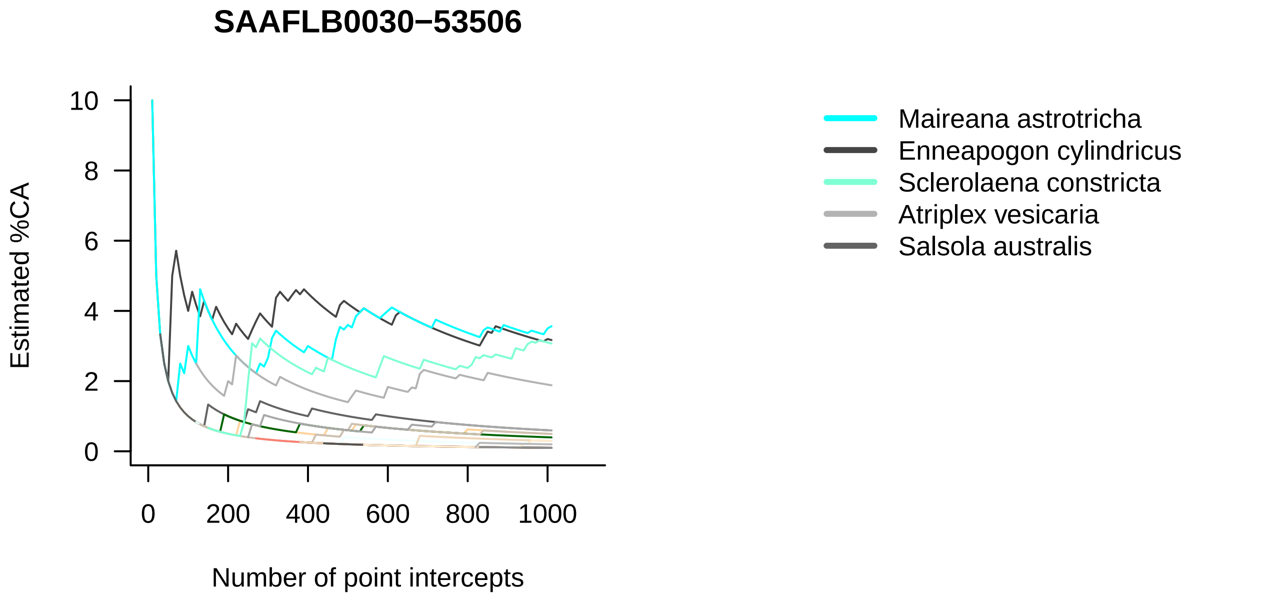
SAAFLB0030−53506
Estimated %CA
Number of point intercepts
0
2
4
6
8
10
0
200
400
600
800
1000
Maireana astrotricha
Enneapogon cylindricus
Sclerolaena constricta
Atriplex vesicaria
Salsola australis

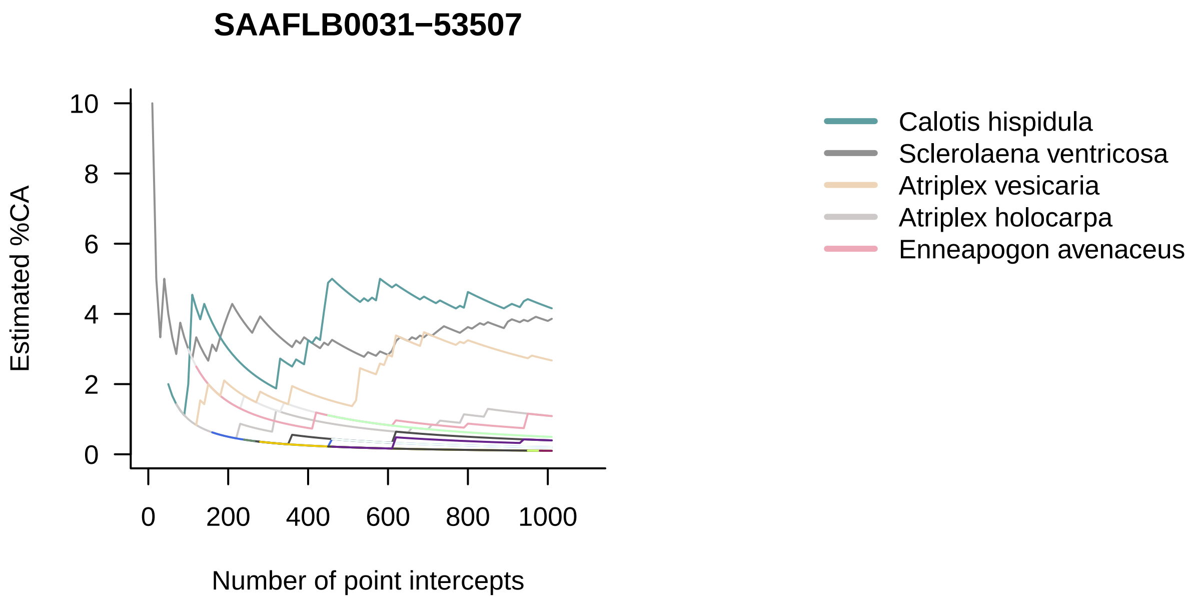
SAAFLB0031−53507
Estimated %CA
0
2
4
6
8
10
0
200
400
600
800
1000
Number of point intercepts
Calotis hispidula
Sclerolaena ventricosa
Atriplex vesicaria
Atriplex holocarpa
Enneapogon avenaceus

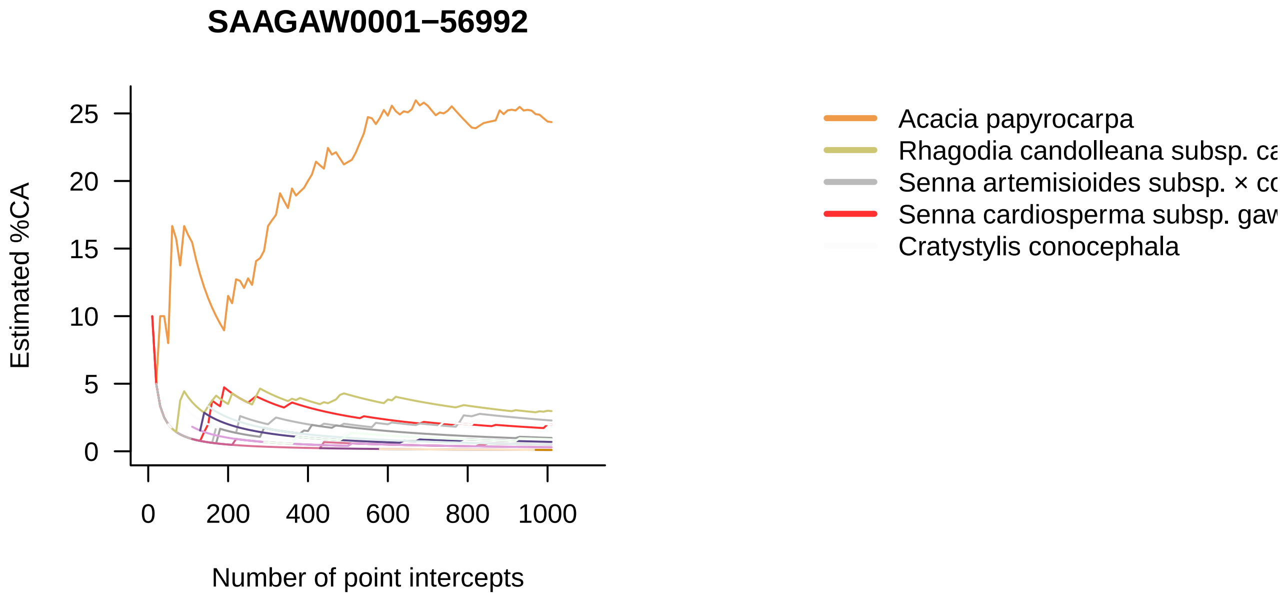
SAAGAW0001−56992
Estimated %CA
0
5
10
15
20
25
0
200
400
600
800
1000
Number of point intercepts
Acacia papyrocarpa
Rhagodia candolleana subsp. ca
Senna artemisioides subsp. × co
Senna cardiosperma subsp. gaw
Cratystylis conocephala

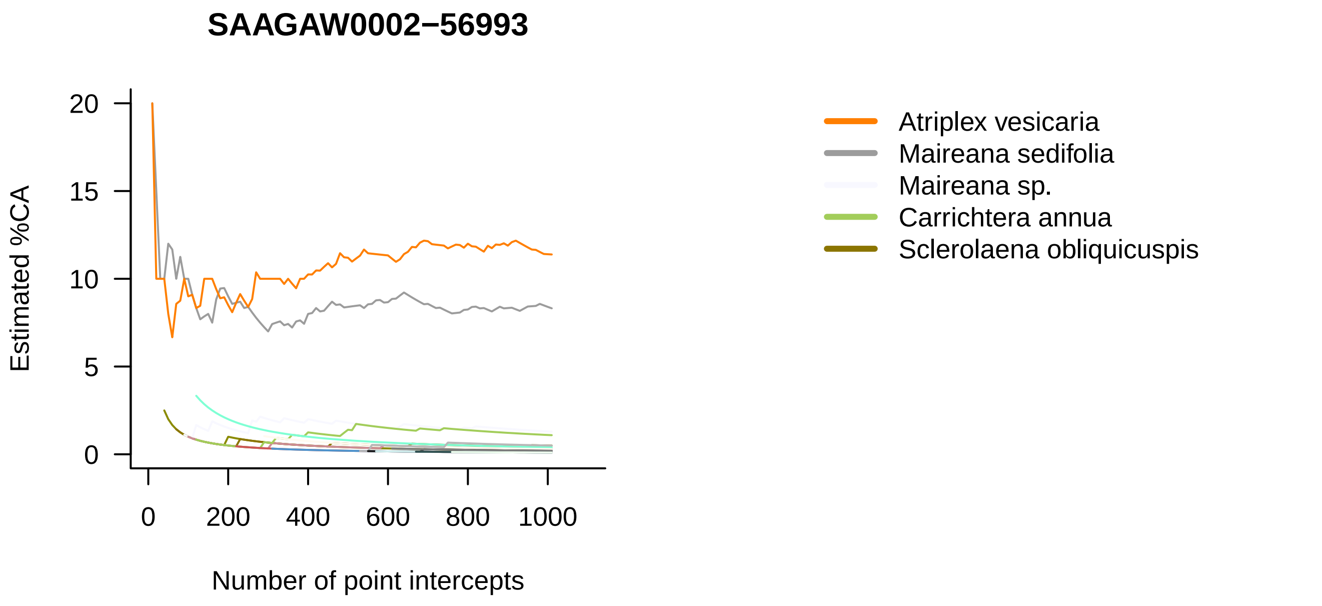
SAAGAW0002−56993
Estimated %CA
0
5
10
15
20
0
200
400
600
800
1000
Number of point intercepts
Atriplex vesicaria
Maireana sedifolia
Maireana sp.
Carrichtera annua
Sclerolaena obliquicuspis

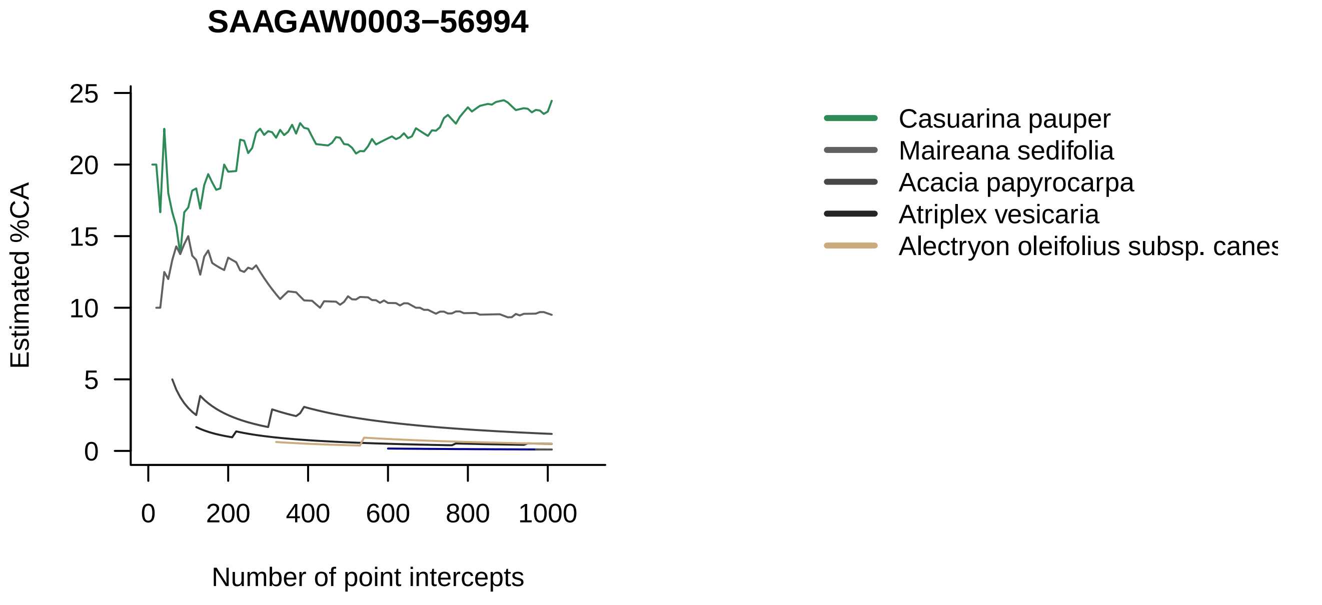
SAAGAW0003−56994
Estimated %CA
0
5
10
15
20
25
0
200
400
600
800
1000
Number of point intercepts
Casuarina pauper
Maireana sedifolia
Acacia papyrocarpa
Atriplex vesicaria
Alectryon oleifolius subsp. canes

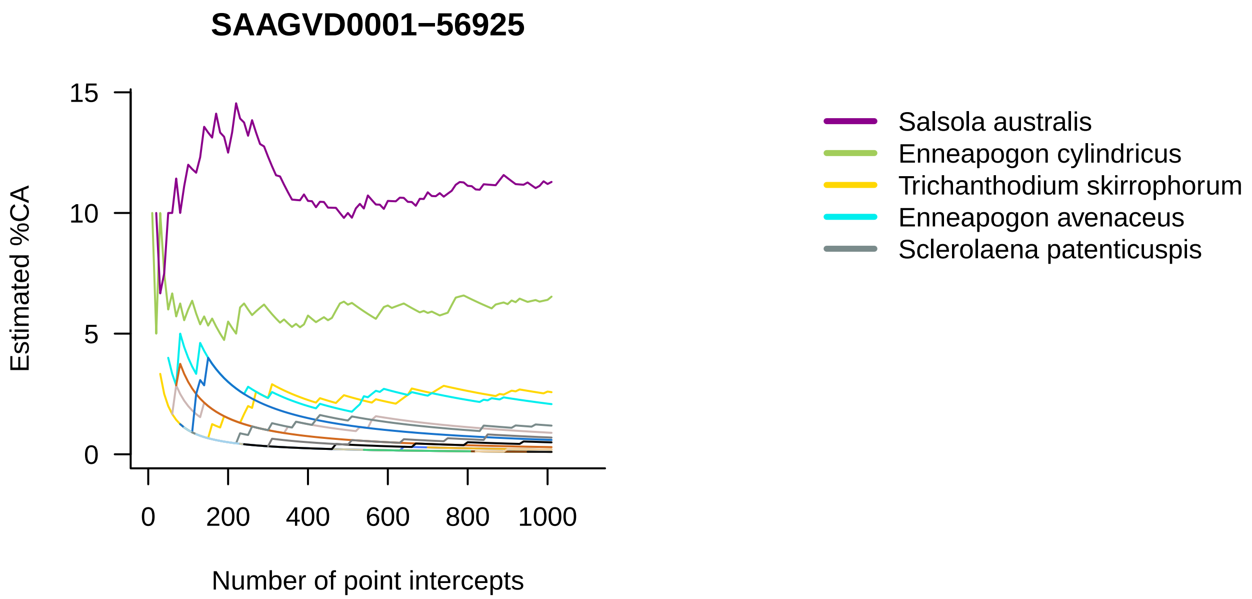
SAAGVD0001−56925
Estimated %CA
Number of point intercepts
0
5
10
15
0
200
400
600
800
1000
Salsola australis
Enneapogon cylindricus
Trichanthodium skirrophorum
Enneapogon avenaceus
Sclerolaena patenticuspis

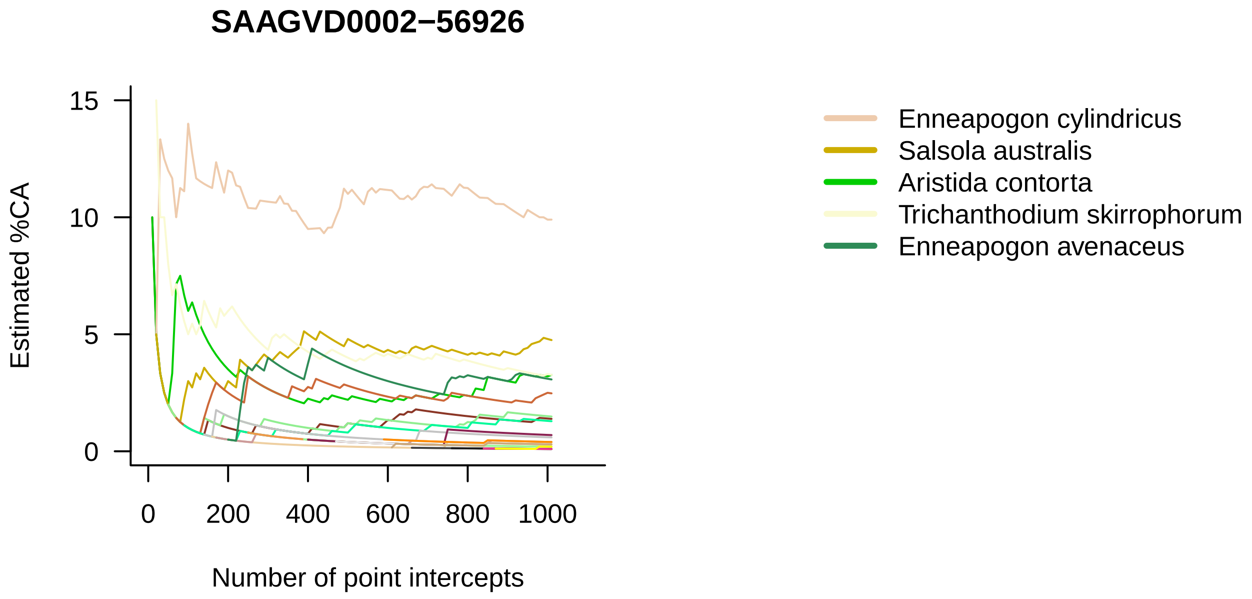
SAAGVD0002−56926
Estimated %CA
Number of point intercepts
0
5
10
15
0
200
400
600
800
1000
Enneapogon cylindricus
Salsola australis
Aristida contorta
Trichanthodium skirrophorum
Enneapogon avenaceus

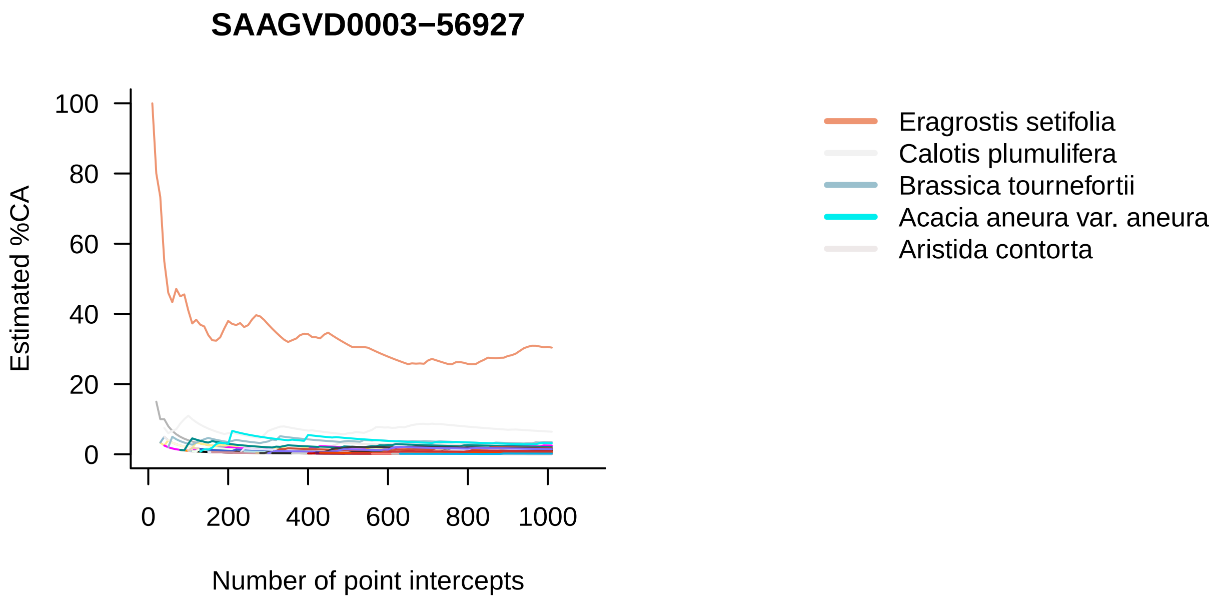
SAAGVD0003−56927
Estimated %CA
100
80
60
40
20
0
0
200
400
600
800
1000
Number of point intercepts
Eragrostis setifolia
Calotis plumulifera
Brassica tournefortii
Acacia aneura var. aneura
Aristida contorta

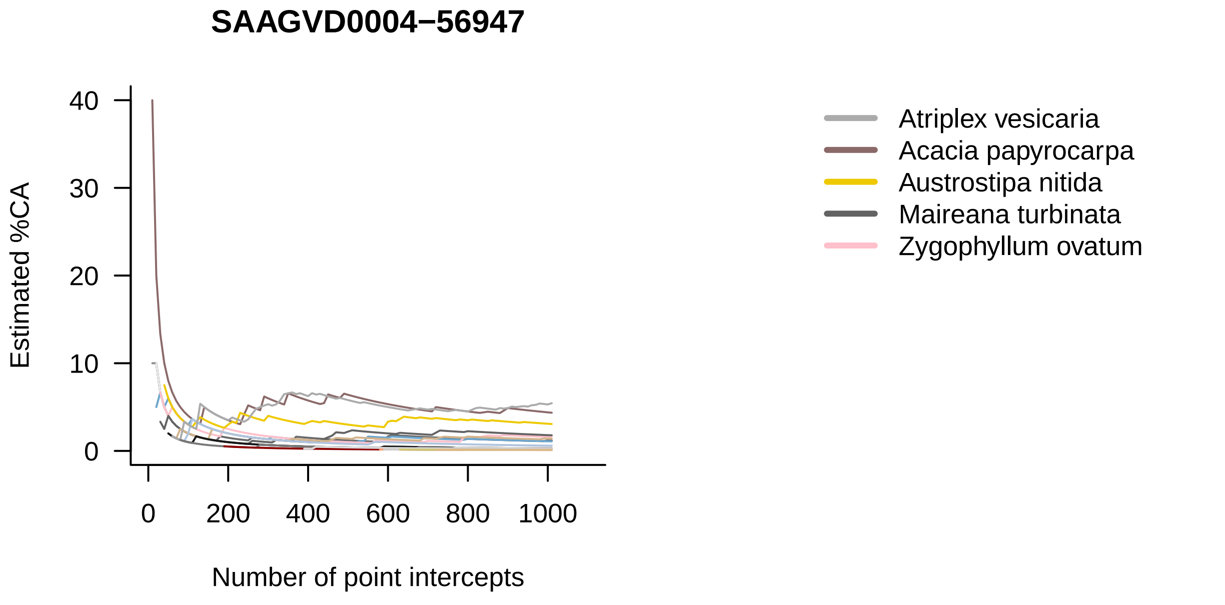
SAAGVD0004−56947
Estimated %CA
40
30
20
10
0
0
200
400
600
800
1000
Number of point intercepts
Atriplex vesicaria
Acacia papyrocarpa
Austrostipa nitida
Maireana turbinata
Zygophyllum ovatum

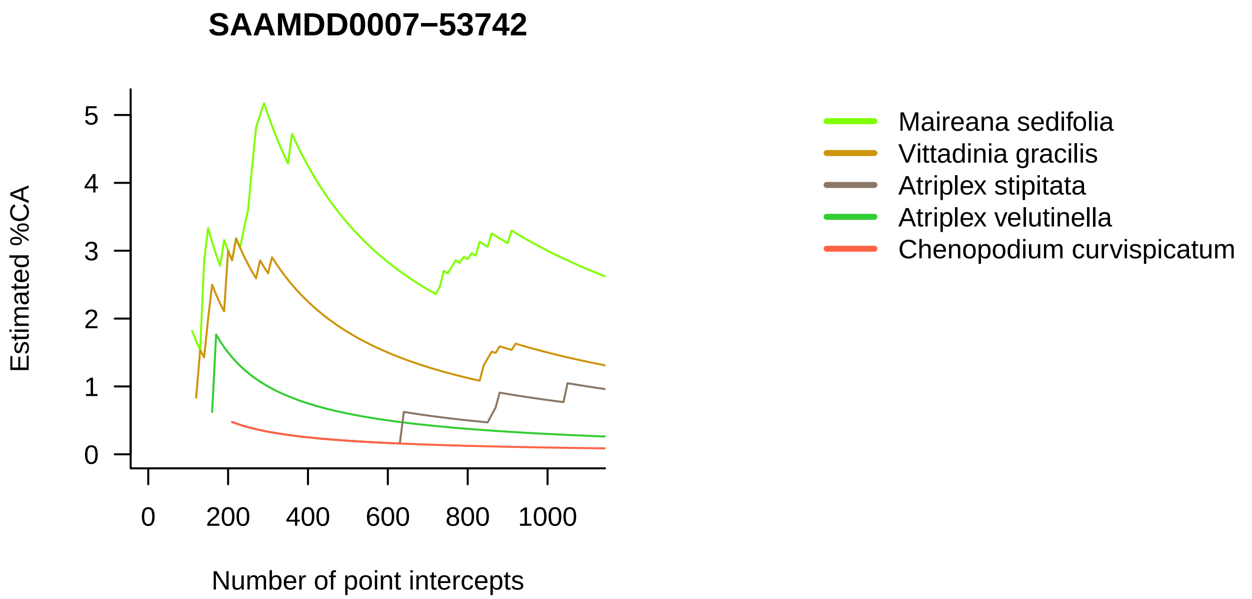
SAAMDD0007−53742
Estimated %CA
Number of point intercepts
0
1
2
3
4
5
0
200
400
600
800
1000
Maireana sedifolia
Vittadinia gracilis
Atriplex stipitata
Atriplex velutinella
Chenopodium curvispicatum

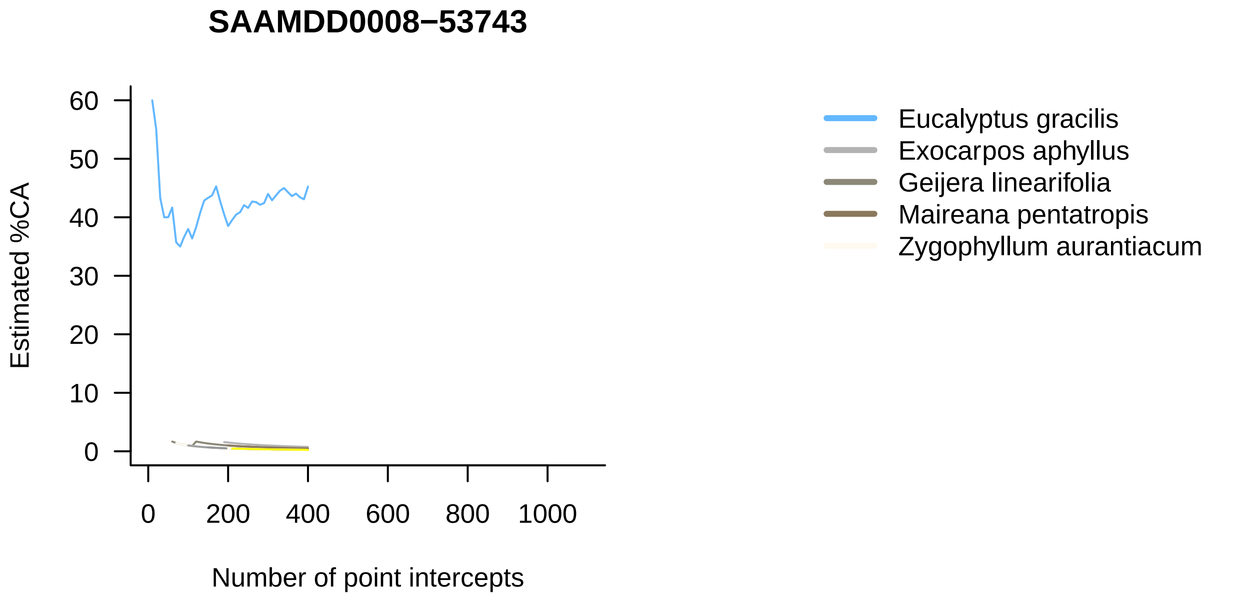
SAAMDD0008−53743
Estimated %CA
Number of point intercepts
0
10
20
30
40
50
60
0
200
400
600
800
1000
Eucalyptus gracilis
Exocarpos aphyllus
Geijera linearifolia
Maireana pentatropis
Zygophyllum aurantiacum

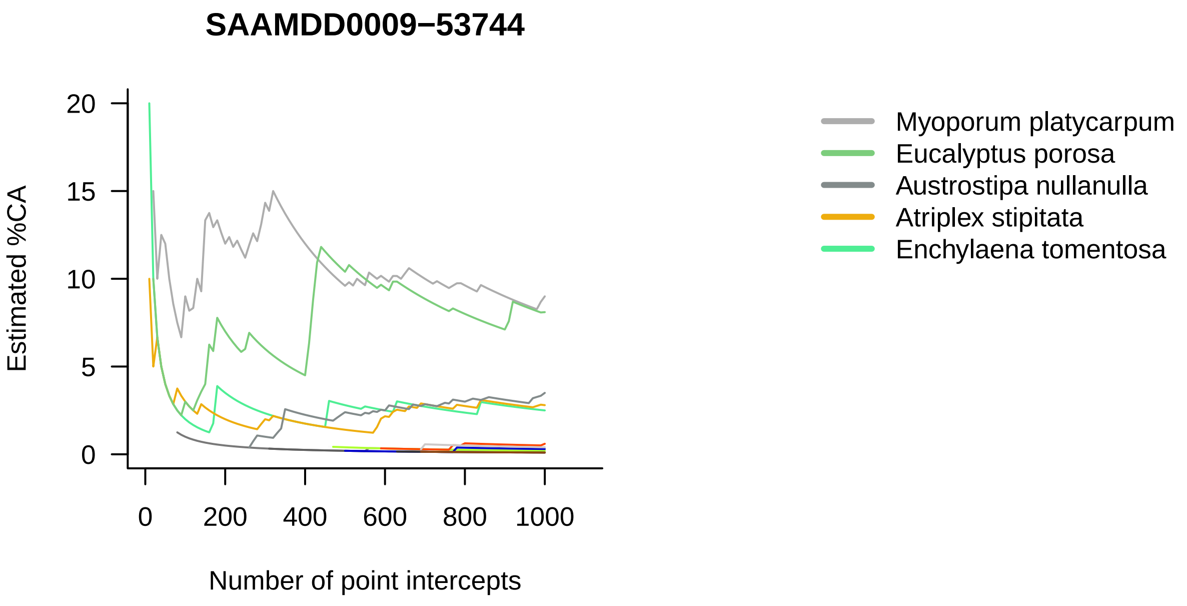
SAAMDD0009−53744
Estimated %CA
Number of point intercepts
0
5
10
15
20
0
200
400
600
800
1000
Myoporum platycarpum
Eucalyptus porosa
Austrostipa nullanulla
Atriplex stipitata
Enchylaena tomentosa

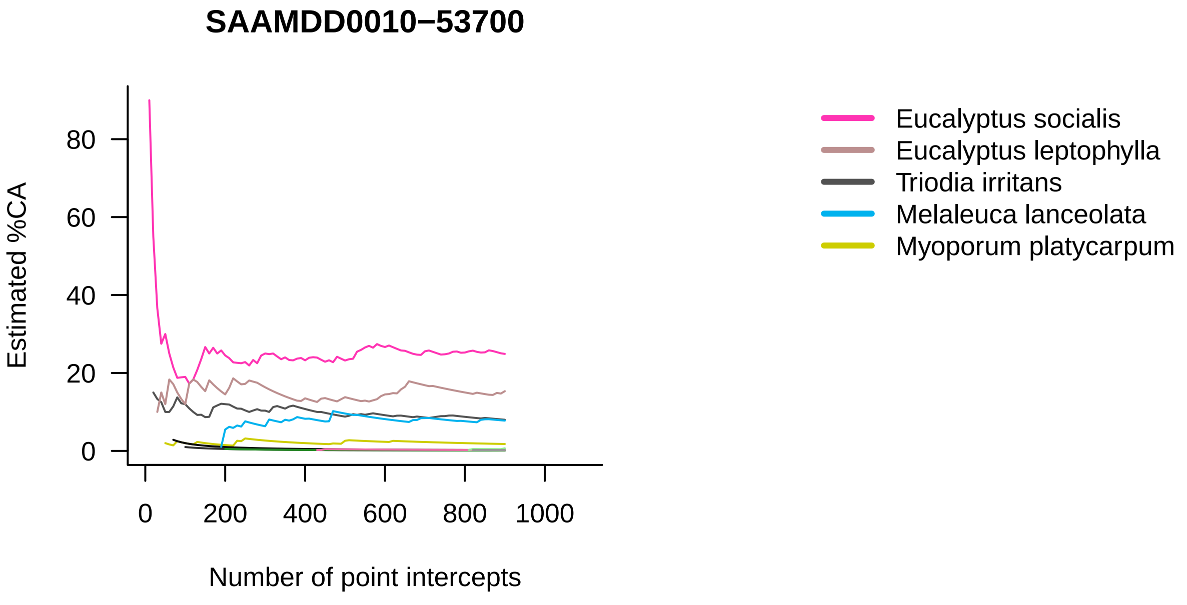
SAAMDD0010−53700
Estimated %CA
Number of point intercepts
0
20
40
60
80
0
200
400
600
800
1000
Eucalyptus socialis
Eucalyptus leptophylla
Triodia irritans
Melaleuca lanceolata
Myoporum platycarpum

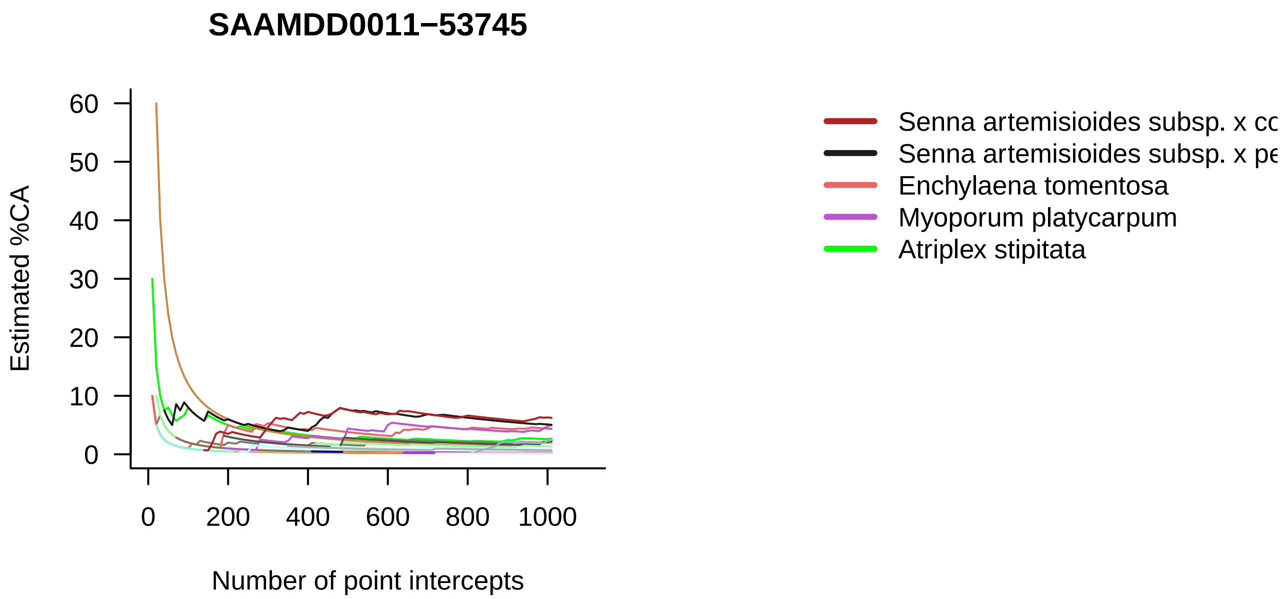
SAAMDD0011−53745
Estimated %CA
0
10
20
30
40
50
60
0
200
400
600
800
1000
Number of point intercepts
Senna artemisioides subsp. x cc
Senna artemisioides subsp. x pe
Enchylaena tomentosa
Myoporum platycarpum
Atriplex stipitata

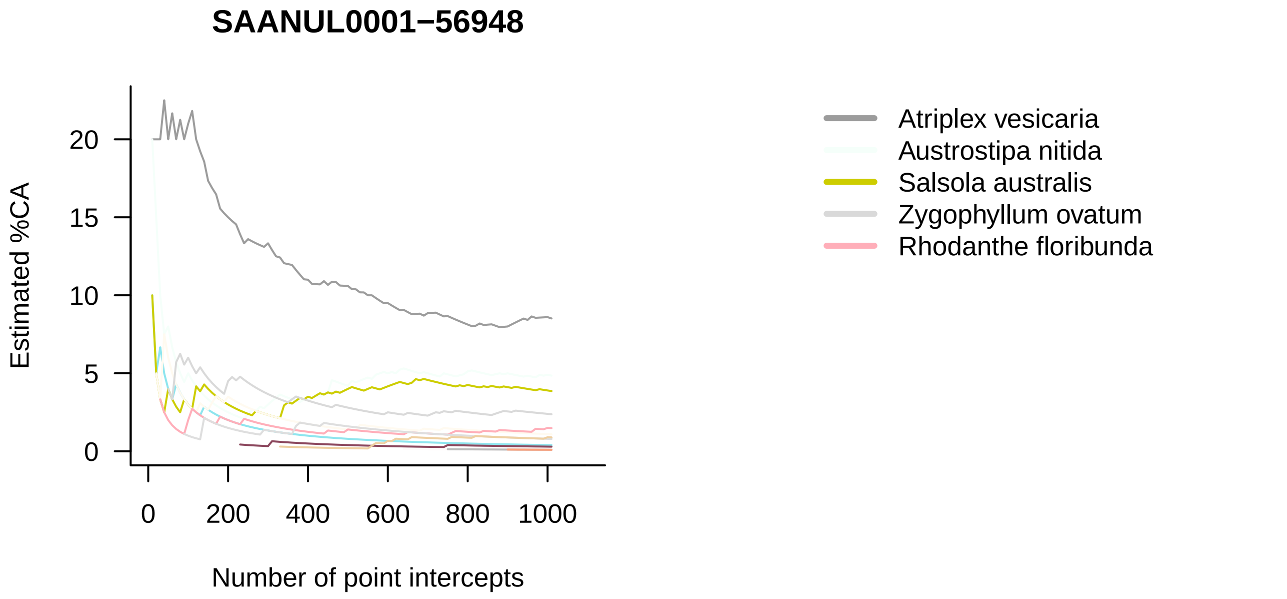
SAANUL0001−56948
Estimated %CA
0
5
10
15
20
0
200
400
600
800
1000
Number of point intercepts
Atriplex vesicaria
Austrostipa nitida
Salsola australis
Zygophyllum ovatum
Rhodanthe floribunda

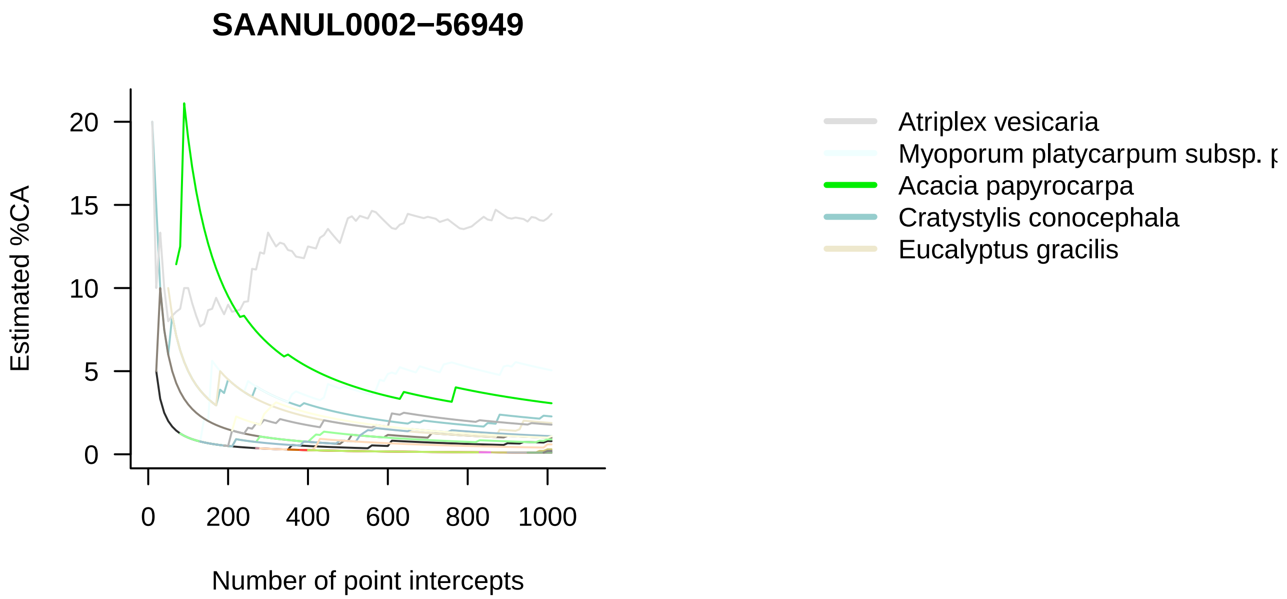
SAANUL0002−56949
Estimated %CA
0
5
10
15
20
0
200
400
600
800
1000
Number of point intercepts
Atriplex vesicaria
Myoporum platycarpum subsp. p
Acacia papyrocarpa
Cratystylis conocephala
Eucalyptus gracilis

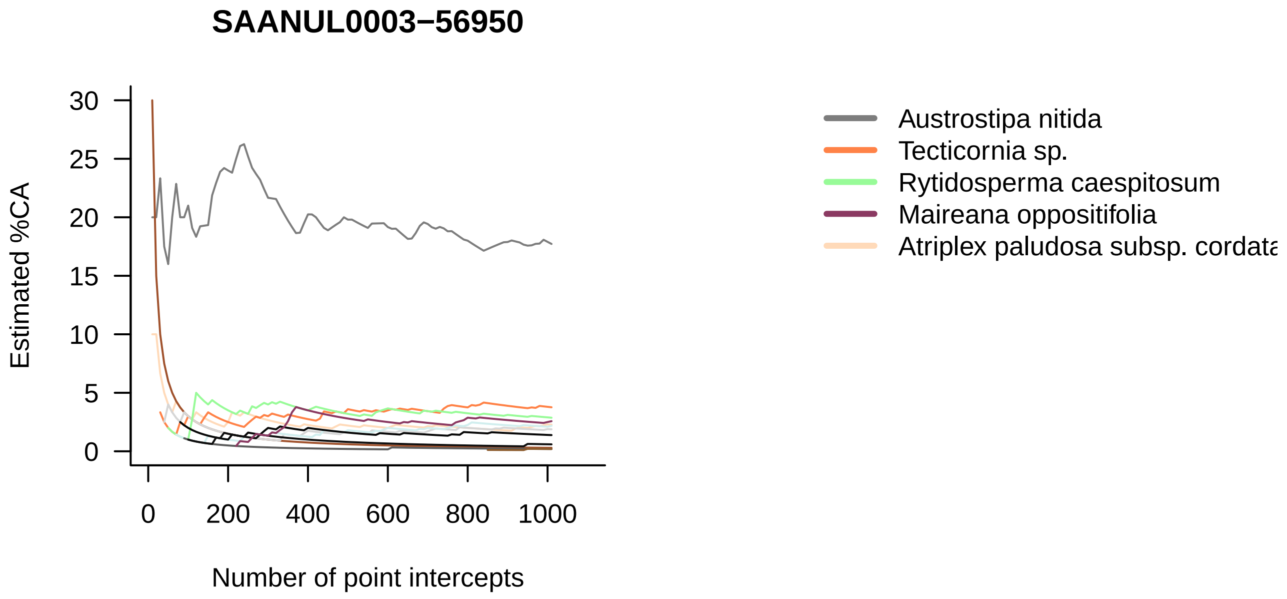
SAANUL0003−56950
Estimated %CA
0
5
10
15
20
25
30
0
200
400
600
800
1000
Number of point intercepts
Austrostipa nitida
Tecticornia sp.
Rytidosperma caespitosum
Maireana oppositifolia
Atriplex paludosa subsp. cordata

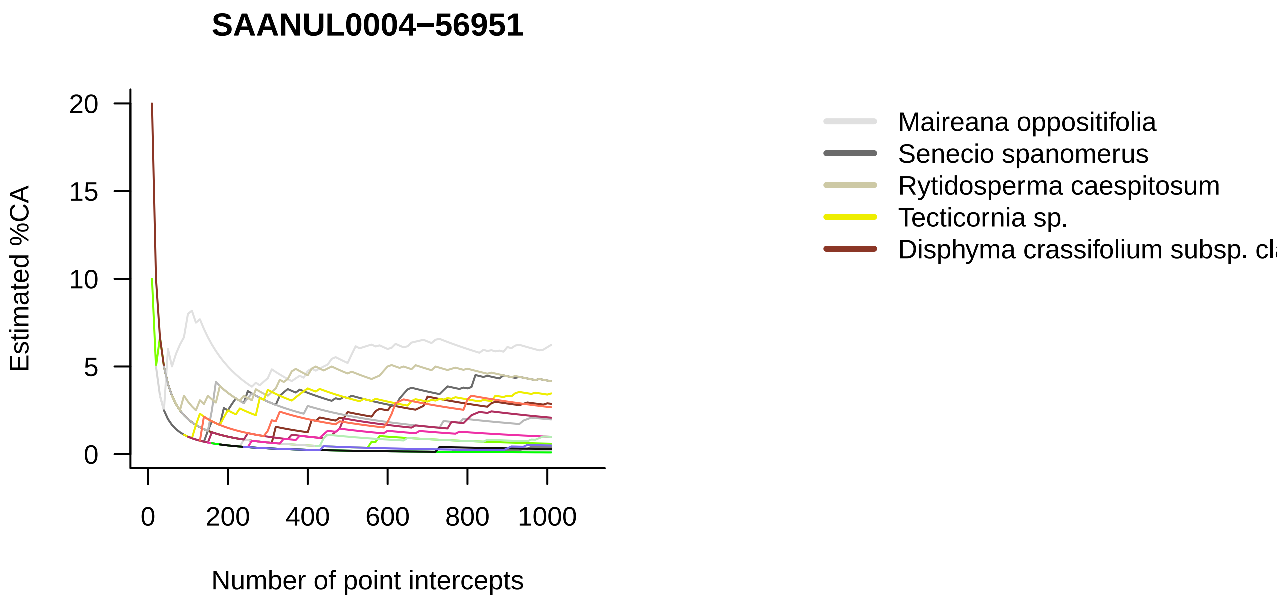
SAANUL0004−56951
Estimated %CA
Number of point intercepts
Maireana oppositifolia
Senecio spanomerus
Rytidosperma caespitosum
Tecticornia sp.
Disphyma crassifolium subsp. cl

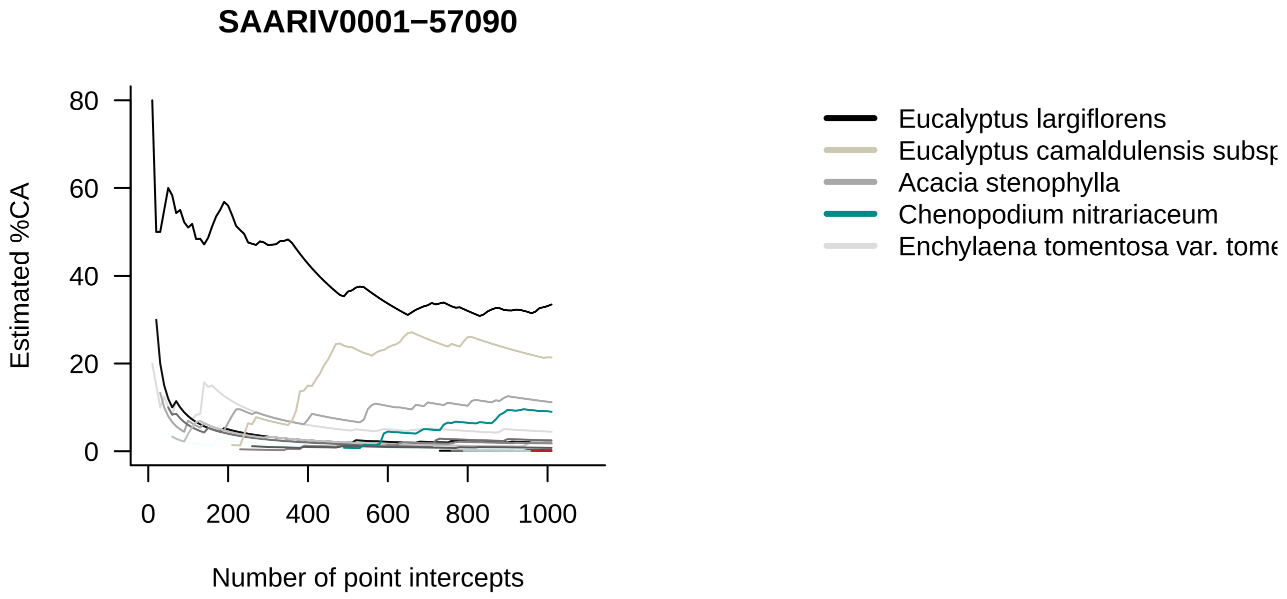
SAARIV0001−57090
Estimated %CA
Number of point intercepts
Eucalyptus largiflorens
Eucalyptus camaldulensis subsp
Acacia stenophylla
Chenopodium nitrariaceum
Enchylaena tomentosa var. tome

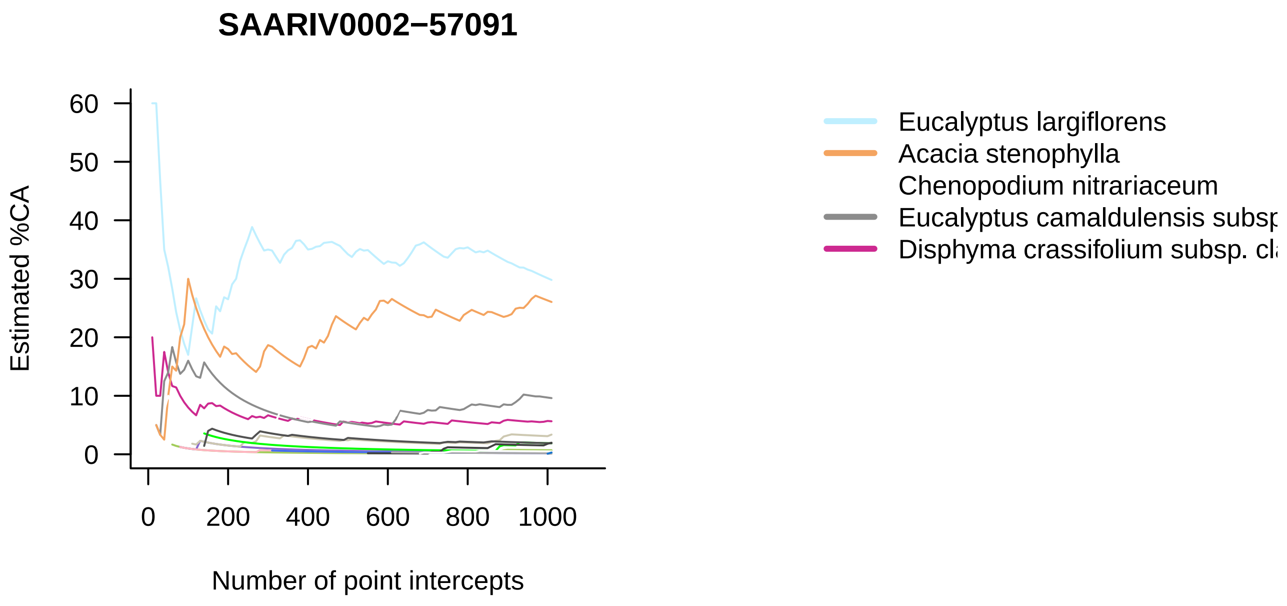
SAARIV0002−57091
Estimated %CA
Number of point intercepts
0
10
20
30
40
50
60
0
200
400
600
800
1000
Eucalyptus largiflorens
Acacia stenophylla
Chenopodium nitrariaceum
Eucalyptus camaldulensis subsp
Disphyma crassifolium subsp. cl

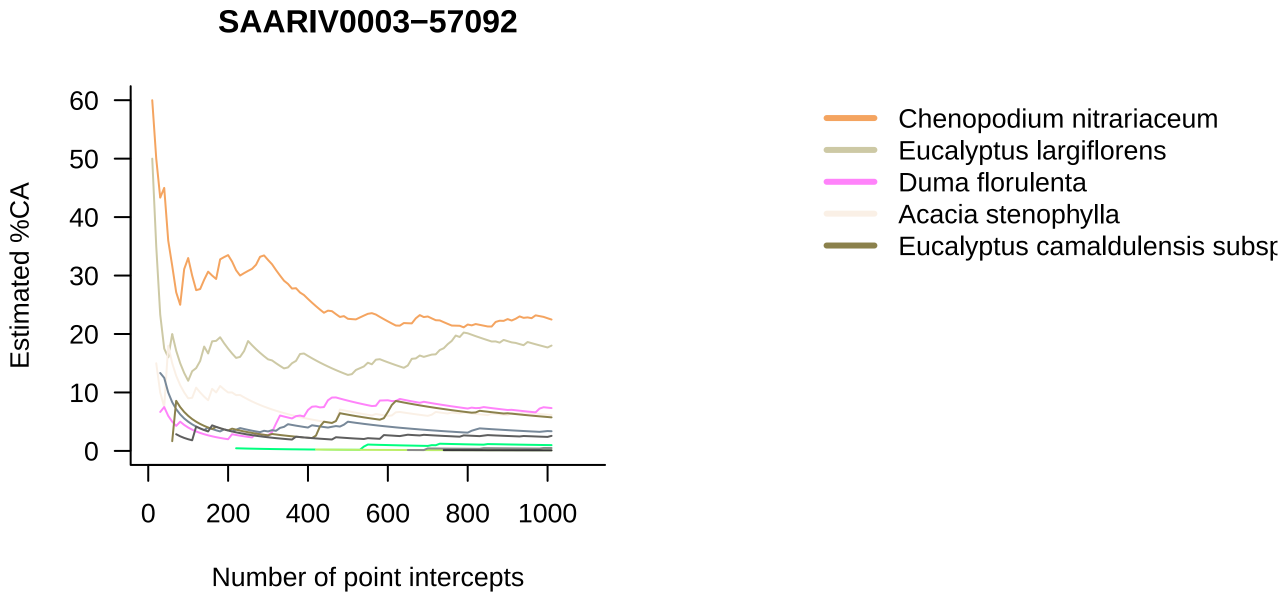
SAARIV0003−57092
Estimated %CA
Number of point intercepts
0
10
20
30
40
50
60
0
200
400
600
800
1000
Chenopodium nitrariaceum
Eucalyptus largiflorens
Duma florulenta
Acacia stenophylla
Eucalyptus camaldulensis subsp

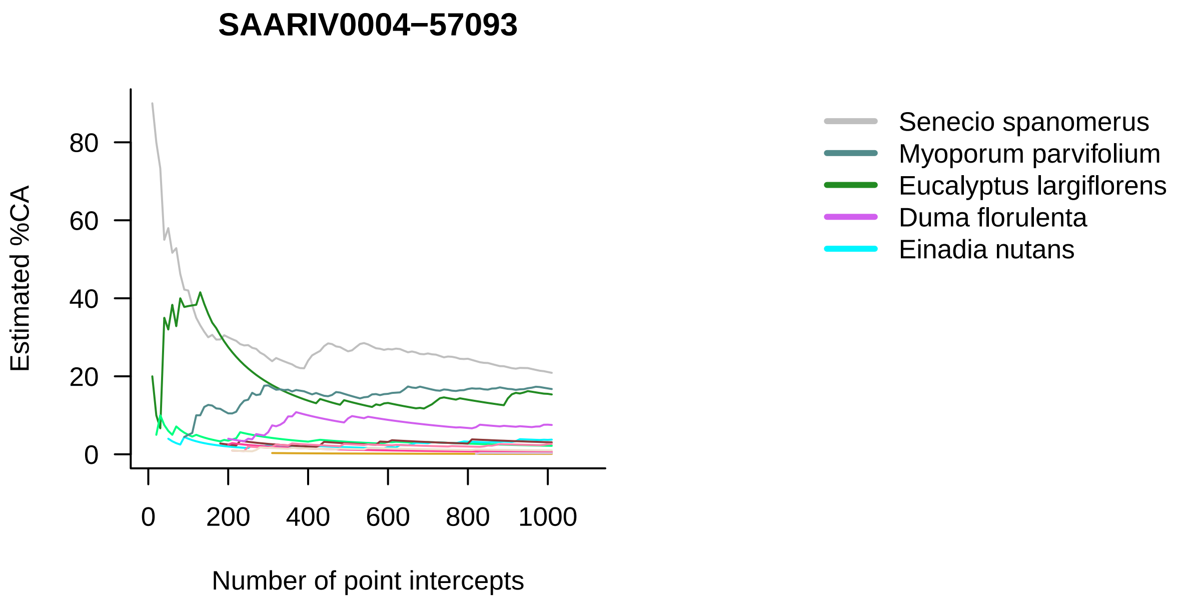
SAARIV0004−57093
Estimated %CA
0
20
40
60
80
0
200
400
600
800
1000
Number of point intercepts
Senecio spanomerus
Myoporum parvifolium
Eucalyptus largiflorens
Duma florulenta
Einadia nutans

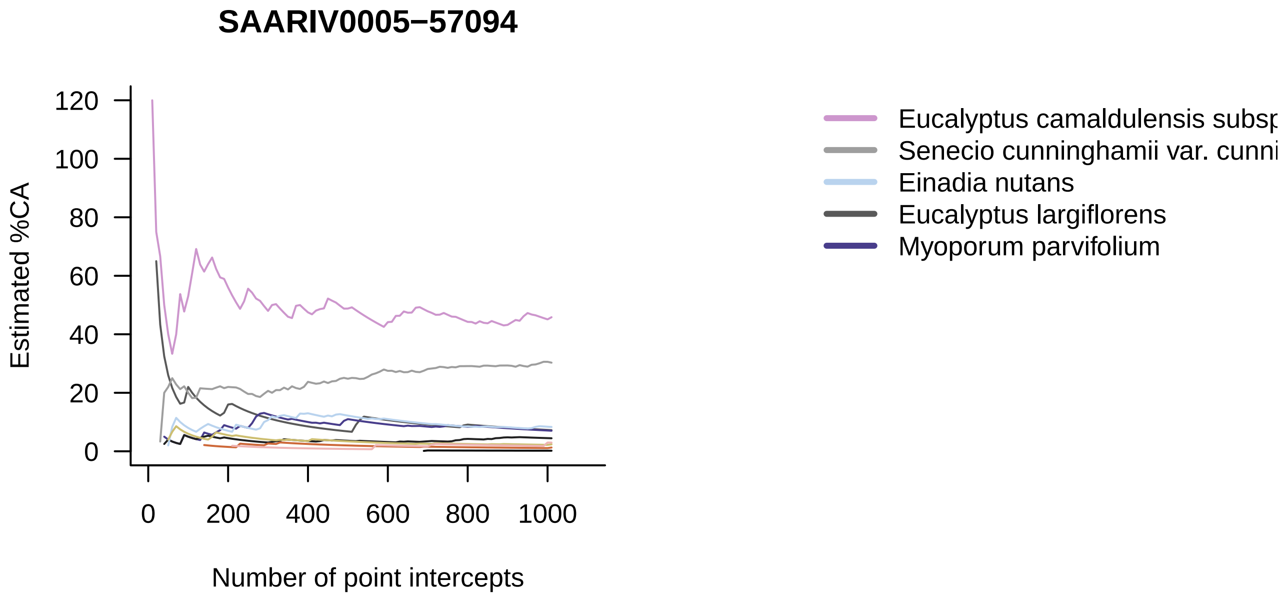
SAARIV0005−57094
Estimated %CA
0
20
40
60
80
100
120
0
200
400
600
800
1000
Number of point intercepts
Eucalyptus camaldulensis subsp
Senecio cunninghamii var. cunni
Einadia nutans
Eucalyptus largiflorens
Myoporum parvifolium

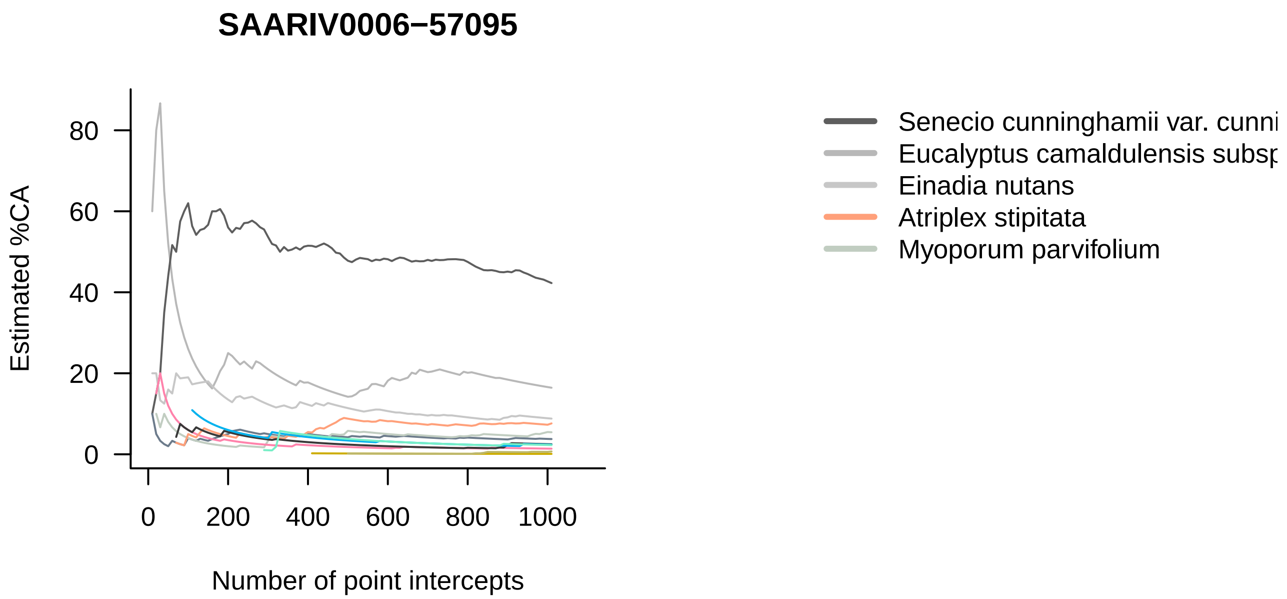
SAARIV0006−57095
Estimated %CA
0
20
40
60
80
0
200
400
600
800
1000
Number of point intercepts
Senecio cunninghamii var. cunni
Eucalyptus camaldulensis subsp
Einadia nutans
Atriplex stipitata
Myoporum parvifolium

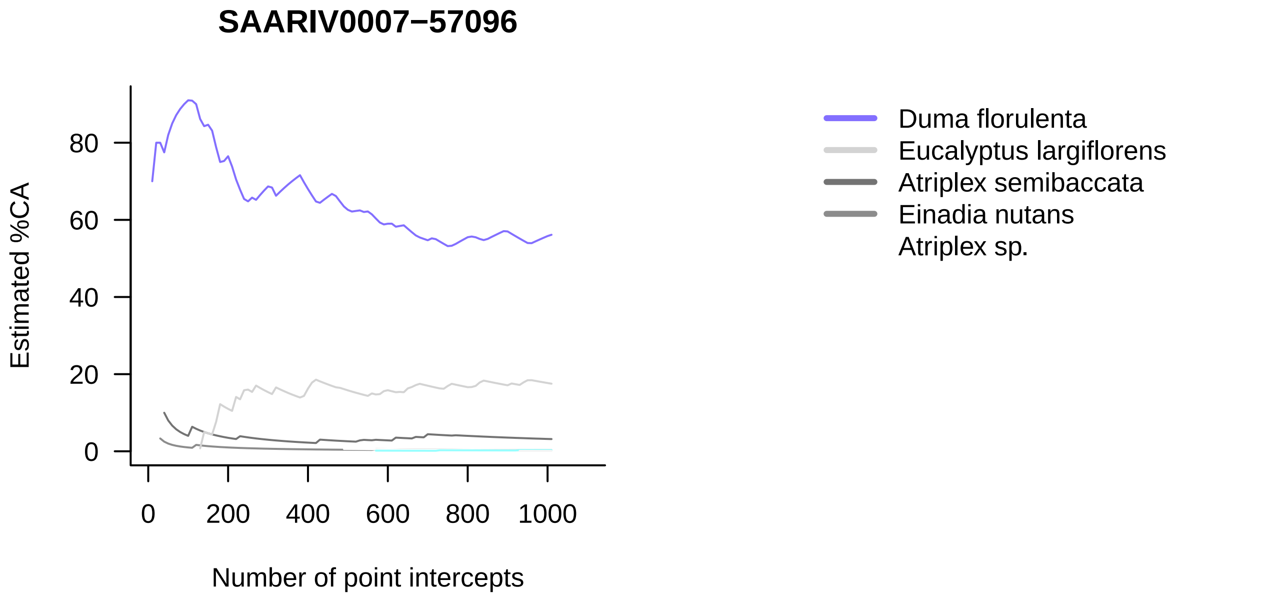
SAARIV0007−57096
Estimated %CA
0
20
40
60
80
0
200
400
600
800
1000
Number of point intercepts
Duma florulenta
Eucalyptus largiflorens
Atriplex semibaccata
Einadia nutans
Atriplex sp.

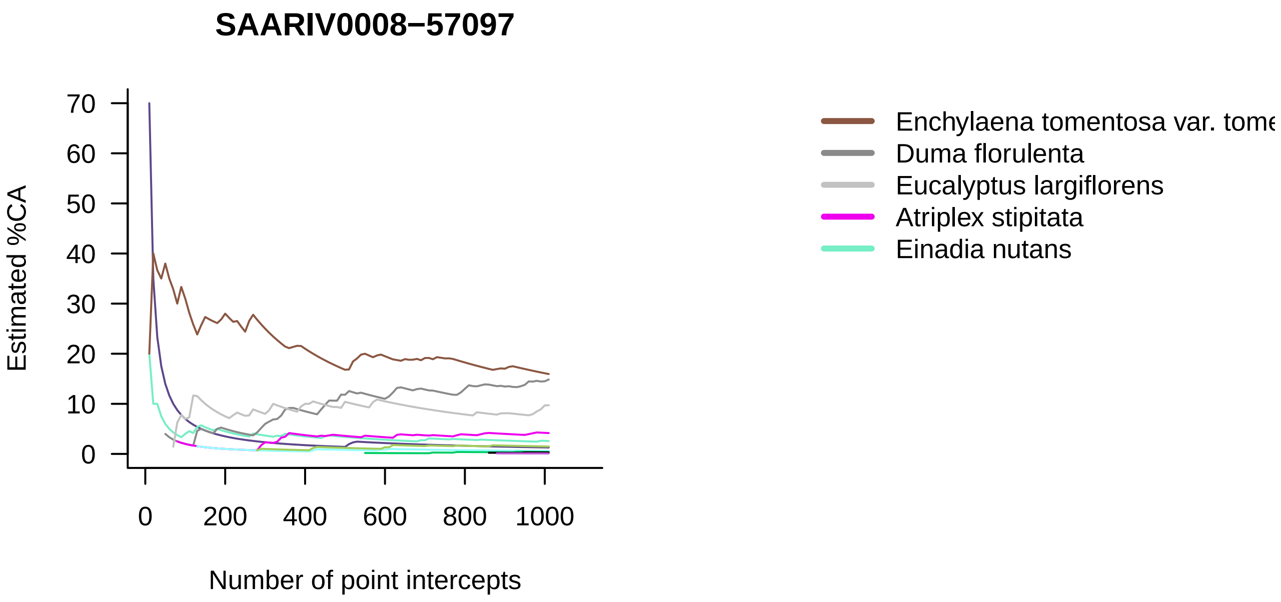
SAARIV0008−57097
Estimated %CA
Number of point intercepts
0
10
20
30
40
50
60
70
0
200
400
600
800
1000
Enchylaena tomentosa var. tome
Duma florulenta
Eucalyptus largiflorens
Atriplex stipitata
Einadia nutans

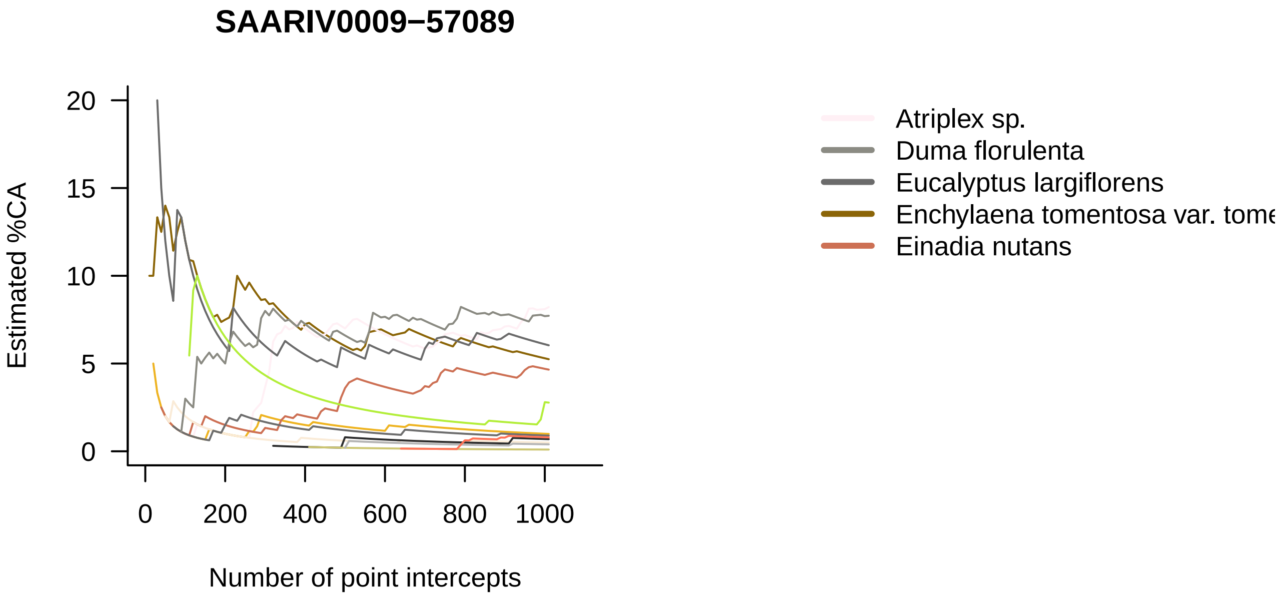
SAARIV0009−57089
Estimated %CA
Number of point intercepts
0
5
10
15
20
0
200
400
600
800
1000
Atriplex sp.
Duma florulenta
Eucalyptus largiflorens
Enchylaena tomentosa var. tome
Einadia nutans

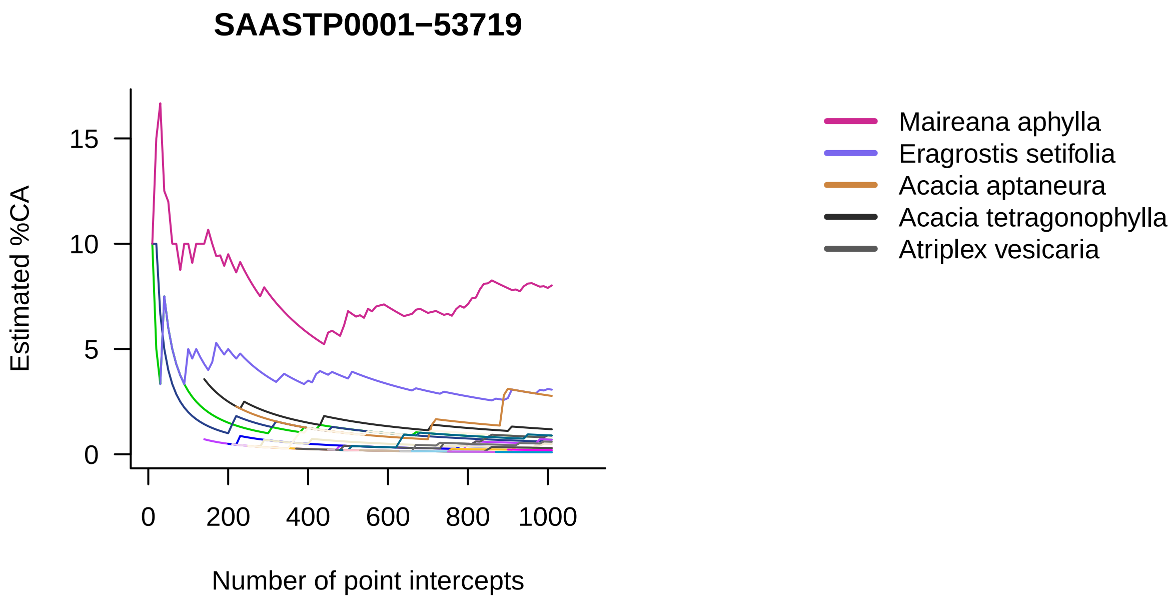
SAASTP0001−53719
Estimated %CA
0
5
10
15
0
200
400
600
800
1000
Number of point intercepts
Maireana aphylla
Eragrostis setifolia
Acacia aptaneura
Acacia tetragonophylla
Atriplex vesicaria

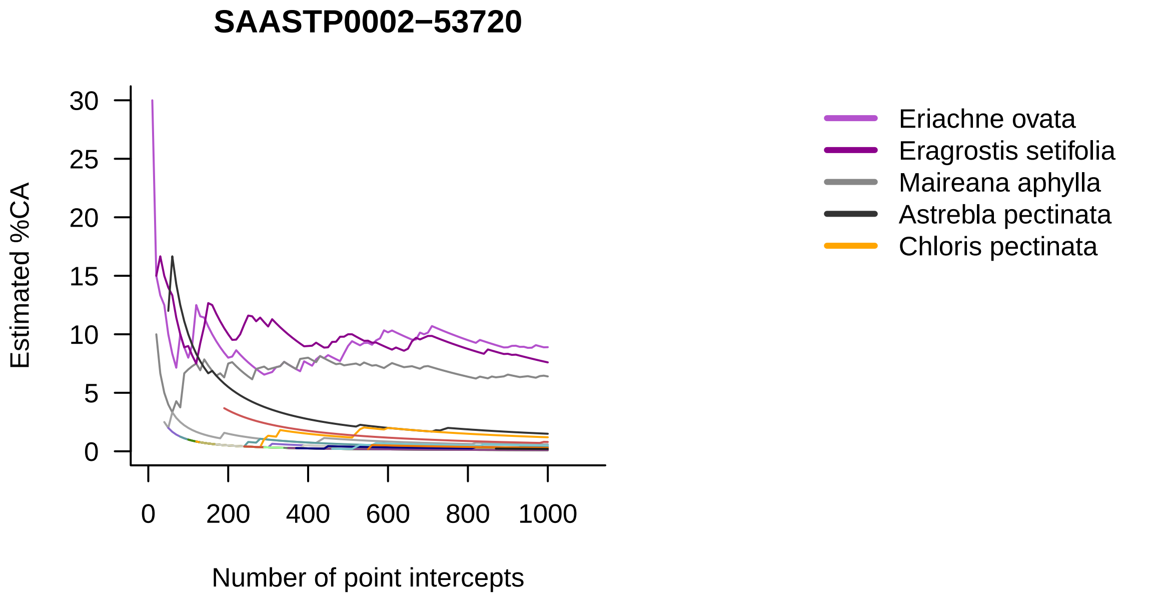
SAASTP0002−53720
Estimated %CA
0
5
10
15
20
25
30
0
200
400
600
800
1000
Number of point intercepts
Eriachne ovata
Eragrostis setifolia
Maireana aphylla
Astrebla pectinata
Chloris pectinata

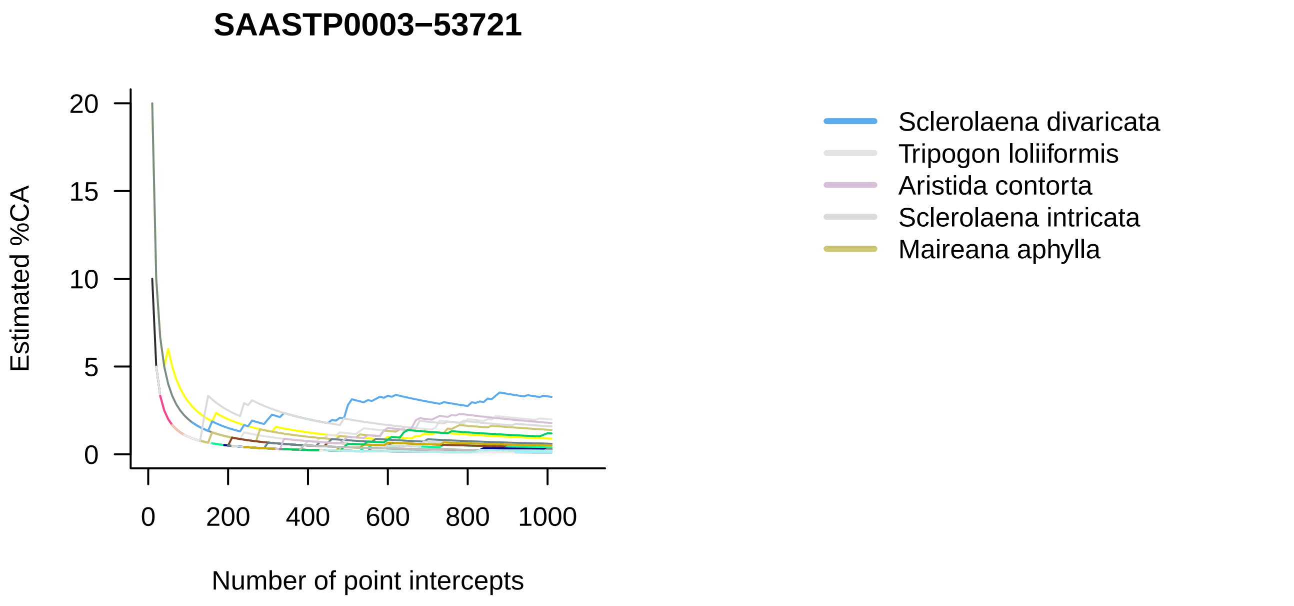
SAASTP0003−53721
Estimated %CA
20
15
10
5
0
0
200
400
600
800
1000
Number of point intercepts
Sclerolaena divaricata
Tripogon loliiformis
Aristida contorta
Sclerolaena intricata
Maireana aphylla

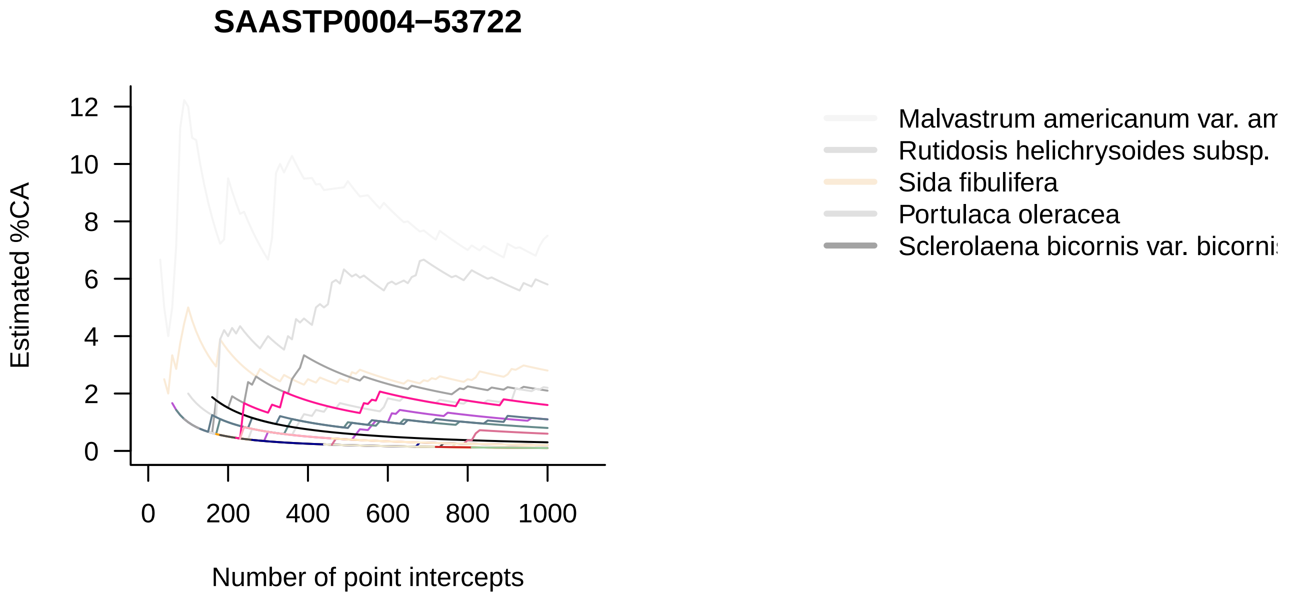
SAASTP0004−53722
Estimated %CA
12
10
8
6
4
2
0
0
200
400
600
800
1000
Number of point intercepts
Malvastrum americanum var. am
Rutidosis helichrysoides subsp.
Sida fibulifera
Portulaca oleracea
Sclerolaena bicornis var. bicorni

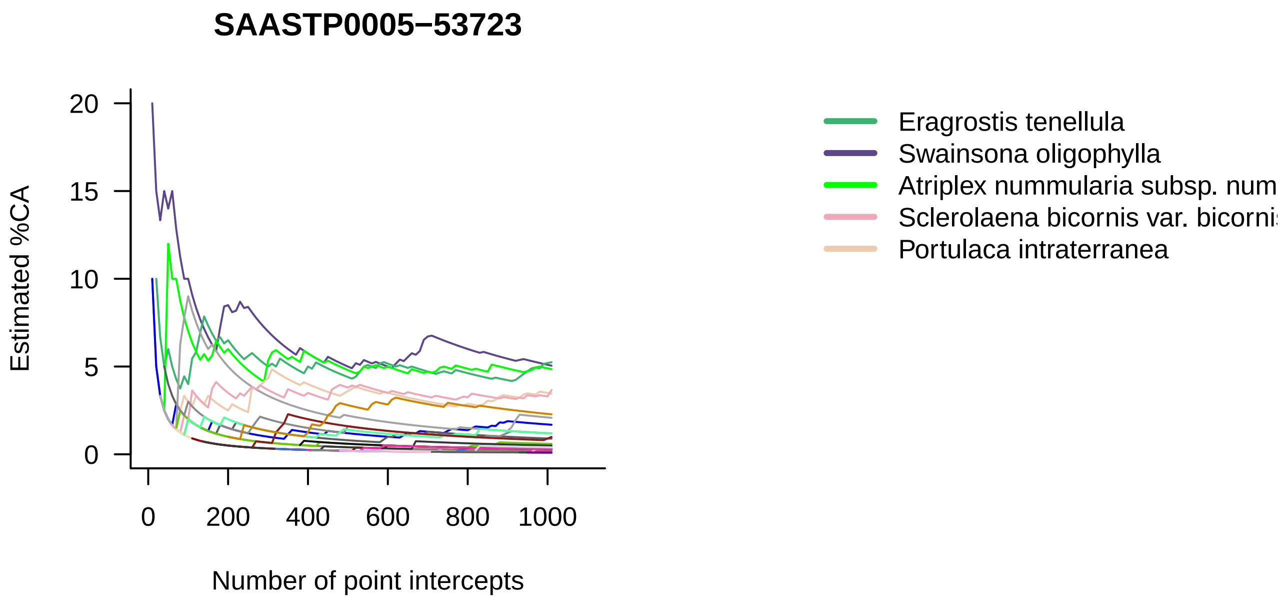
SAASTP0005−53723
Estimated %CA
Number of point intercepts
0
5
10
15
20
0
200
400
600
800
1000
Eragrostis tenellula
Swainsona oligophylla
Atriplex nummularia subsp. num
Sclerolaena bicornis var. bicorni
Portulaca intraterranea

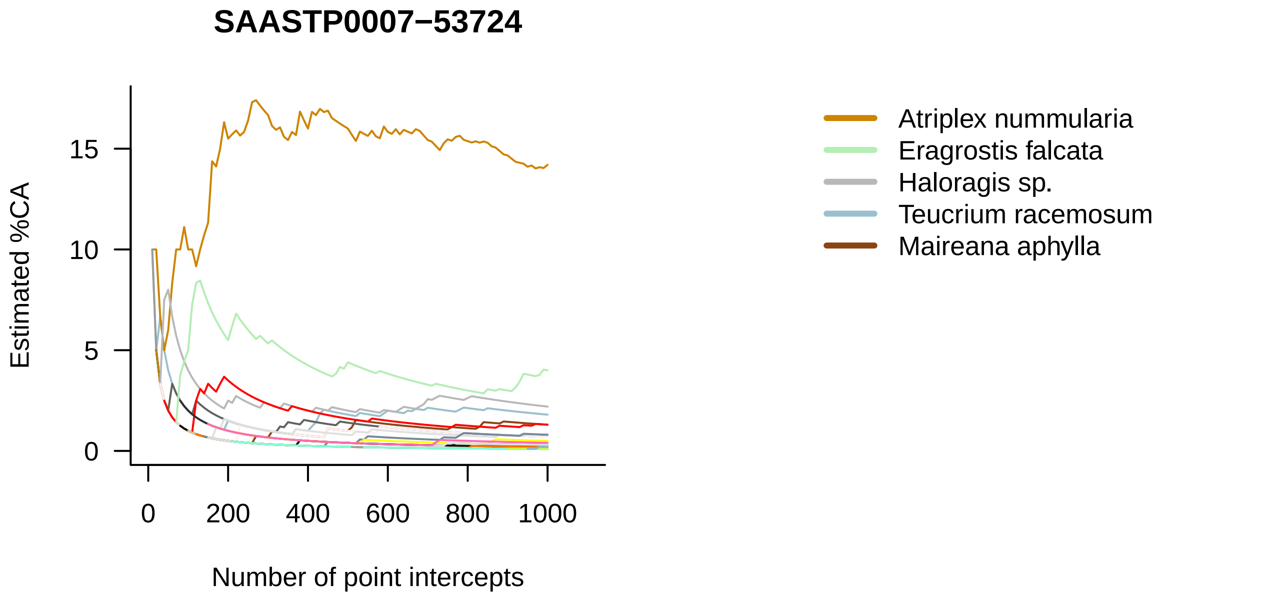
SAASTP0007−53724
Estimated %CA
Number of point intercepts
0
5
10
15
0
200
400
600
800
1000
Atriplex nummularia
Eragrostis falcata
Haloragis sp.
Teucrium racemosum
Maireana aphylla

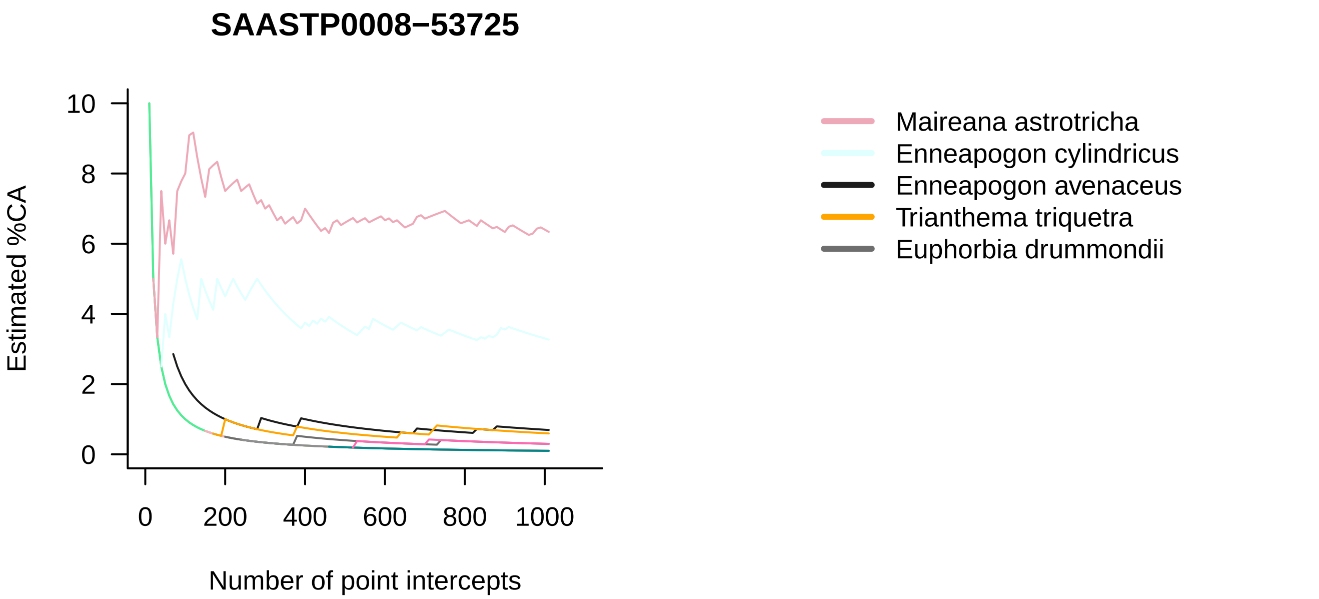
SAASTP0008−53725
Estimated %CA
Number of point intercepts
0
200
400
600
800
1000
0
2
4
6
8
10
Maireana astrotricha
Enneapogon cylindricus
Enneapogon avenaceus
Trianthema triquetra
Euphorbia drummondii

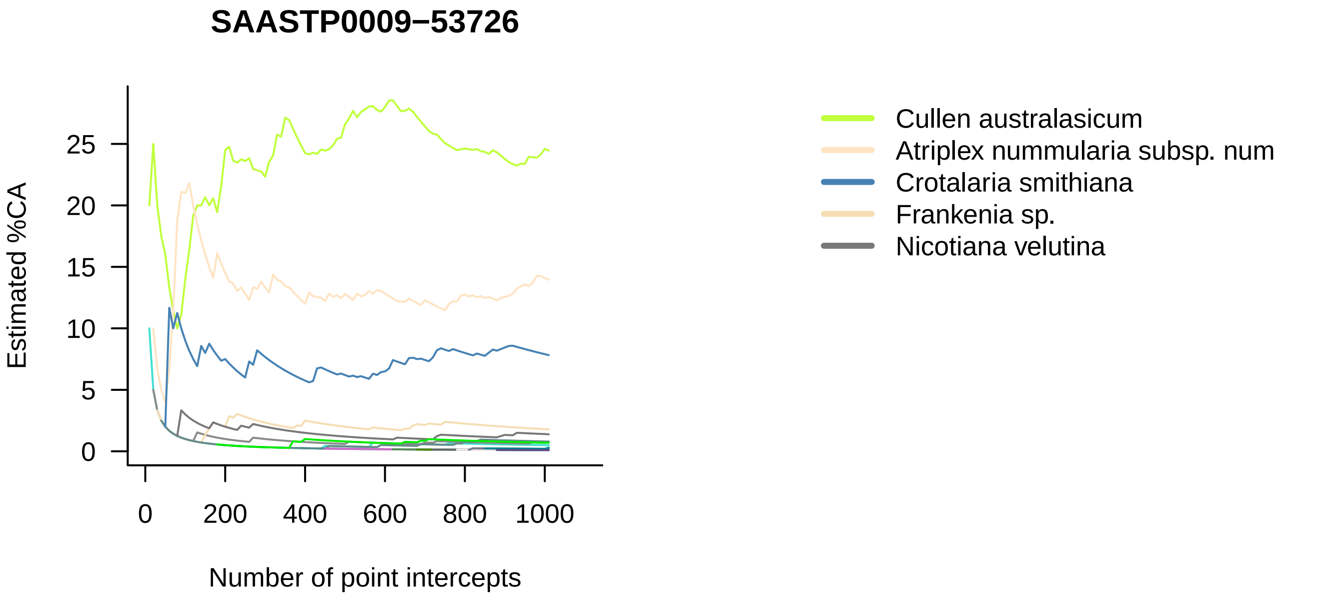
SAASTP0009−53726
Estimated %CA
Number of point intercepts
0
200
400
600
800
1000
0
5
10
15
20
25
Cullen australasicum
Atriplex nummularia subsp. num
Crotalaria smithiana
Frankenia sp.
Nicotiana velutina

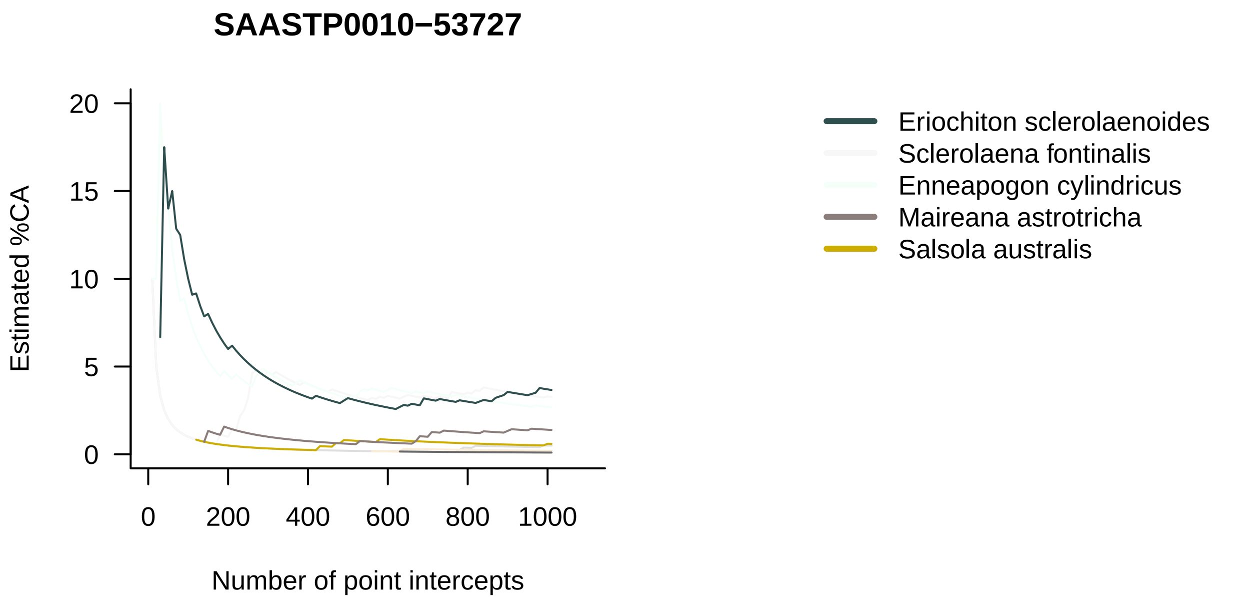
SAASTP0010−53727
Estimated %CA
20
15
10
5
0
0
200
400
600
800
1000
Number of point intercepts
Eriochiton sclerolaenoides
Sclerolaena fontinalis
Enneapogon cylindricus
Maireana astrotricha
Salsola australis

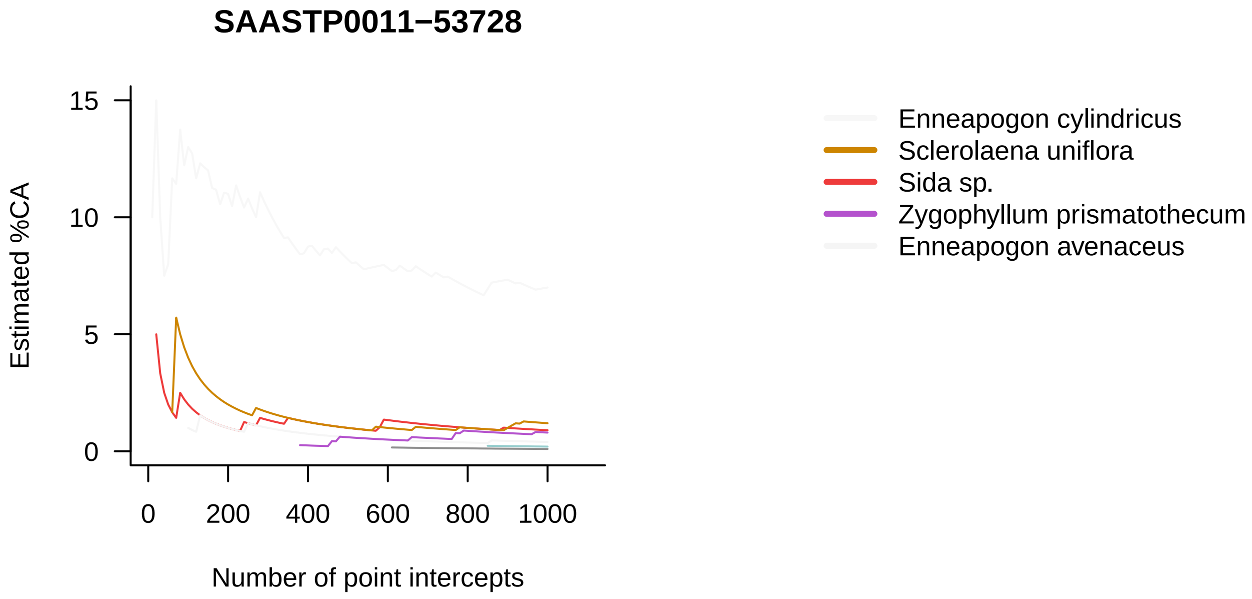
SAASTP0011−53728
Estimated %CA
15
10
5
0
0
200
400
600
800
1000
Number of point intercepts
Enneapogon cylindricus
Sclerolaena uniflora
Sida sp.
Zygophyllum prismatothecum
Enneapogon avenaceus

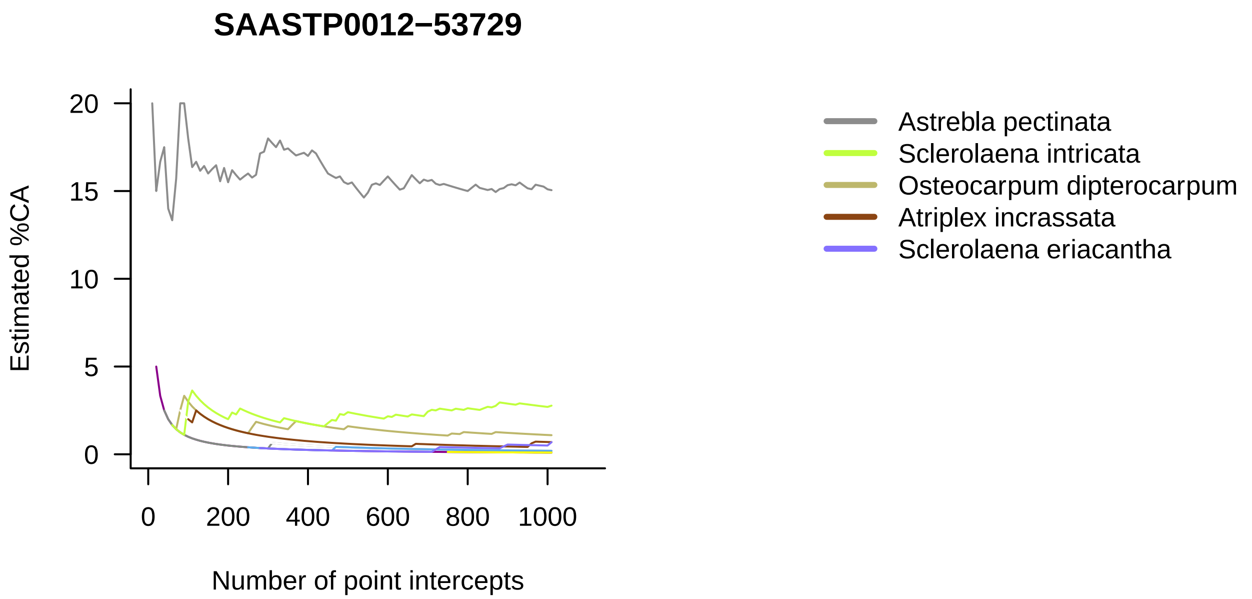

SAASTP0012−53729
Estimated %CA
0
5
10
15
20
0
200
400
600
800
1000
Number of point intercepts
Astrebla pectinata
Sclerolaena intricata
Osteocarpum dipterocarpum
Atriplex incrassata
Sclerolaena eriacantha


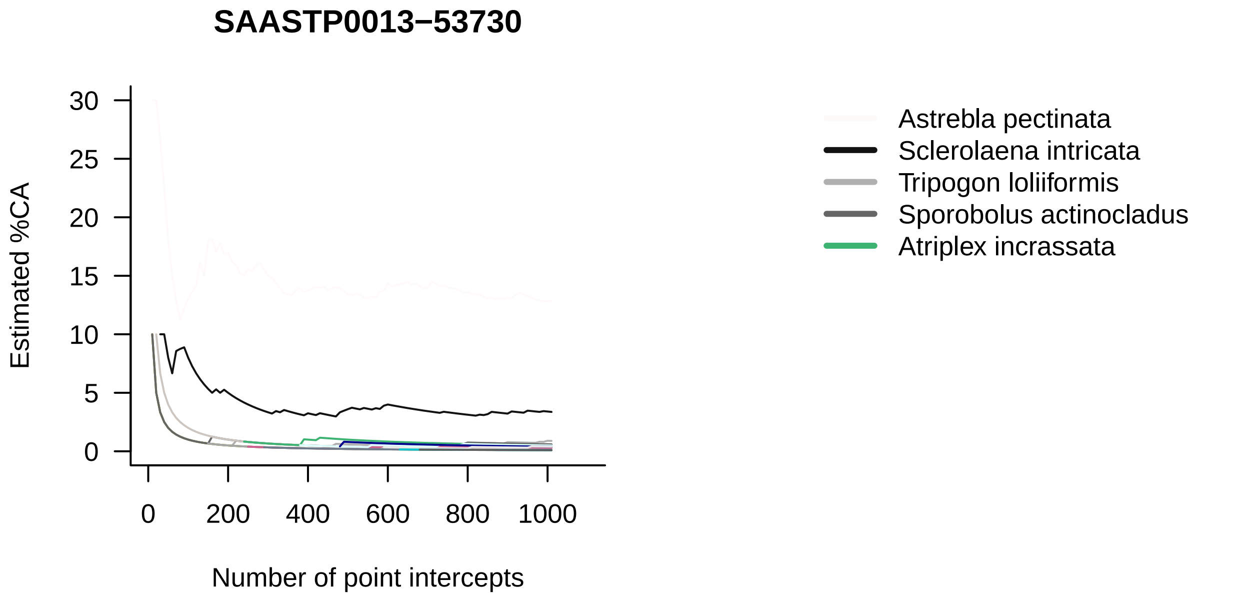

SAASTP0013−53730
Estimated %CA
0
5
10
15
20
25
30
0
200
400
600
800
1000
Number of point intercepts
Astrebla pectinata
Sclerolaena intricata
Tripogon loliiformis
Sporobolus actinocladus
Atriplex incrassata

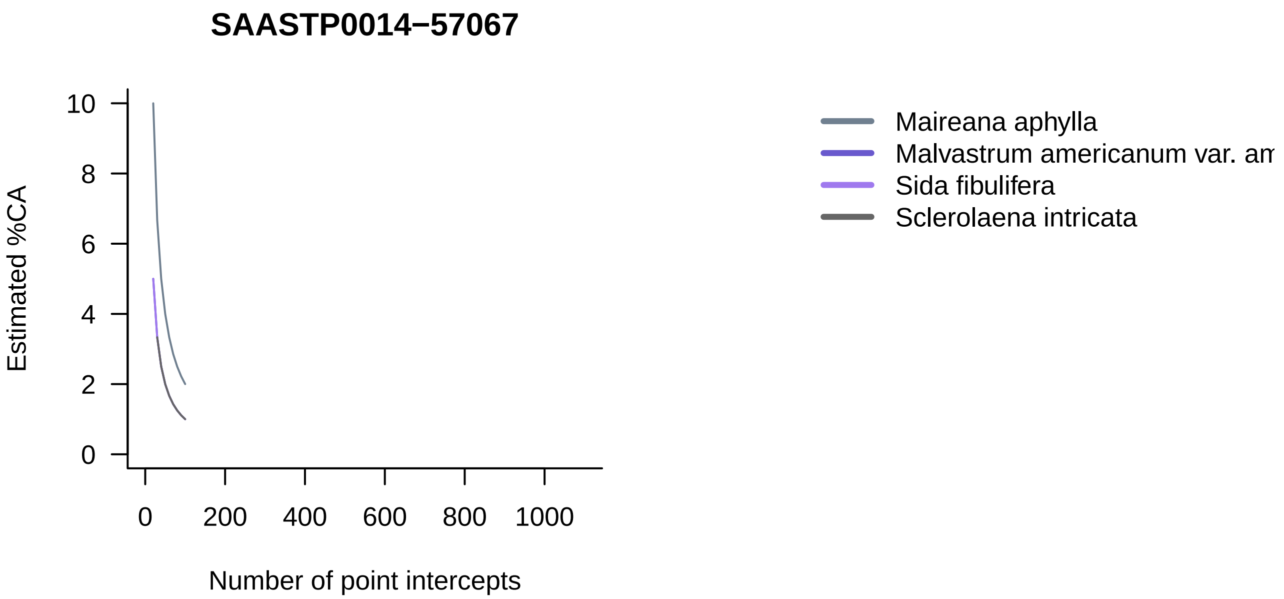
SAASTP0014−57067
Estimated %CA
Number of point intercepts
0
2
4
6
8
10
0
200
400
600
800
1000
Maireana aphylla
Malvastrum americanum var. am
Sida fibulifera
Sclerolaena intricata

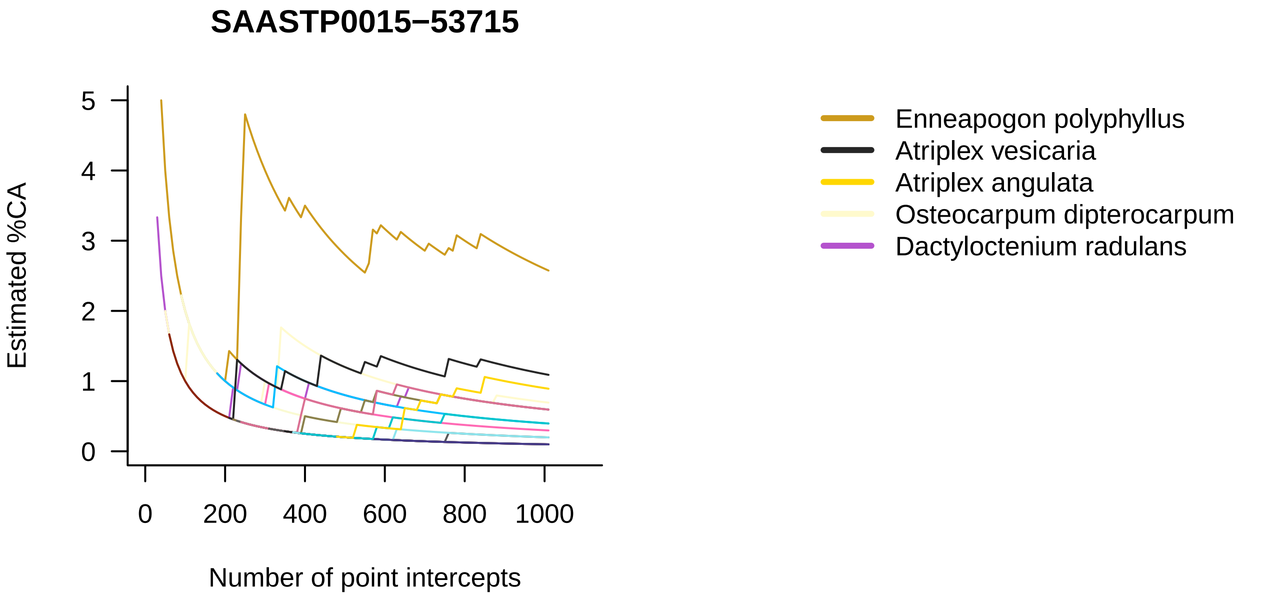
SAASTP0015−53715
Estimated %CA
Number of point intercepts
0
1
2
3
4
5
0
200
400
600
800
1000
Enneapogon polyphyllus
Atriplex vesicaria
Atriplex angulata
Osteocarpum dipterocarpum
Dactyloctenium radulans

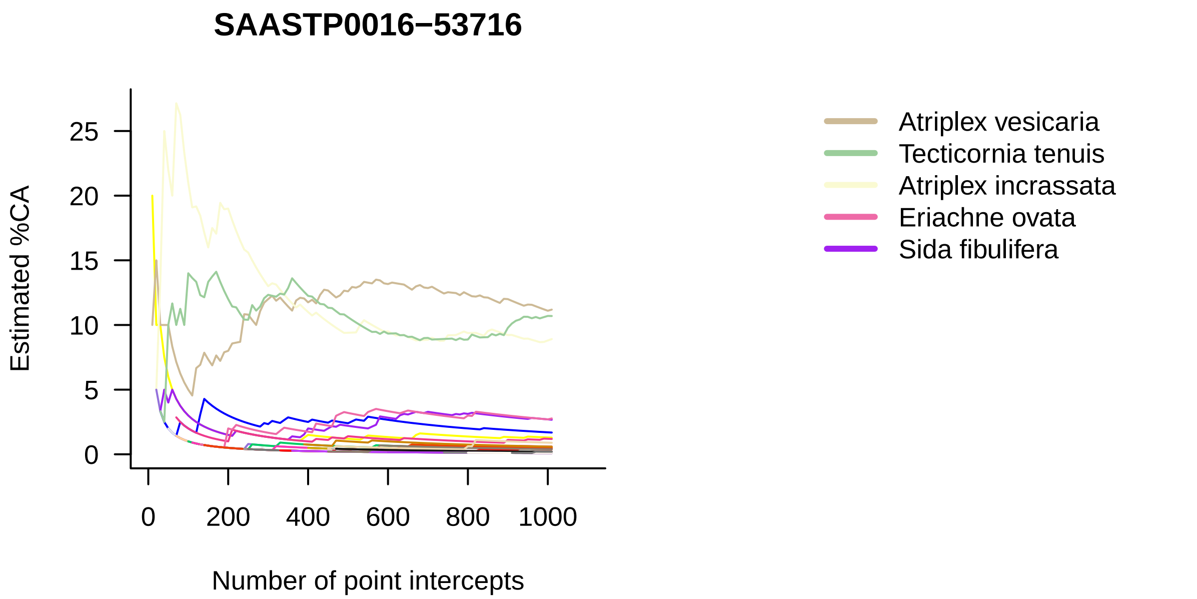
SAASTP0016−53716
Estimated %CA
Number of point intercepts
0
5
10
15
20
25
0
200
400
600
800
1000
Atriplex vesicaria
Tecticornia tenuis
Atriplex incrassata
Eriachne ovata
Sida fibulifera

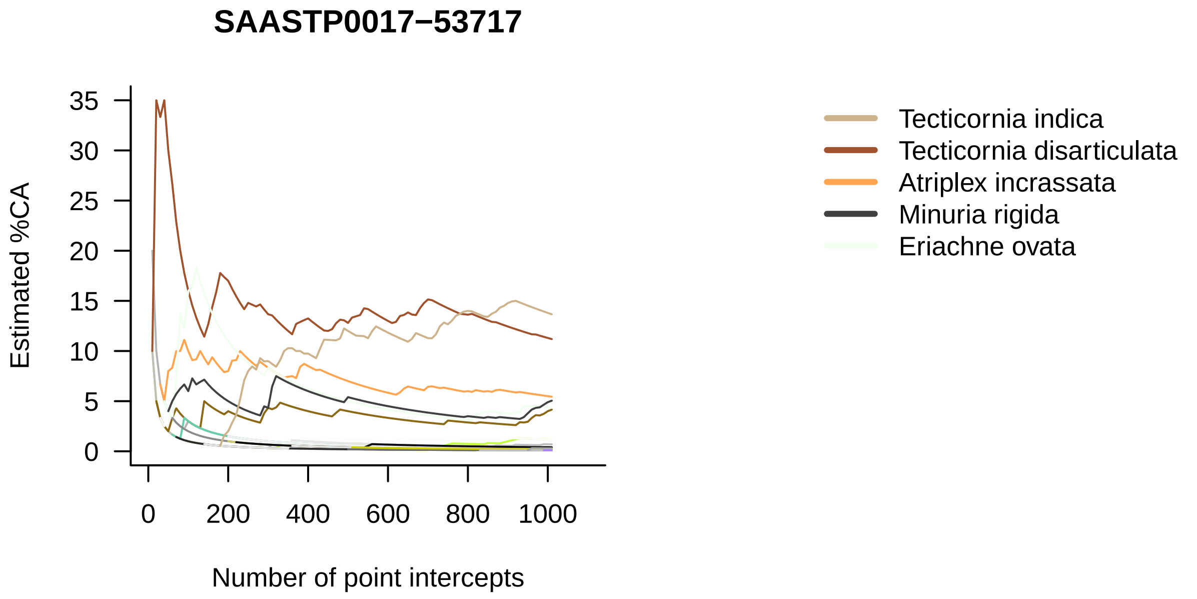
SAASTP0017−53717
Estimated %CA
Number of point intercepts
0
5
10
15
20
25
30
35
0
200
400
600
800
1000
Tecticornia indica
Tecticornia disarticulata
Atriplex incrassata
Minuria rigida
Eriachne ovata

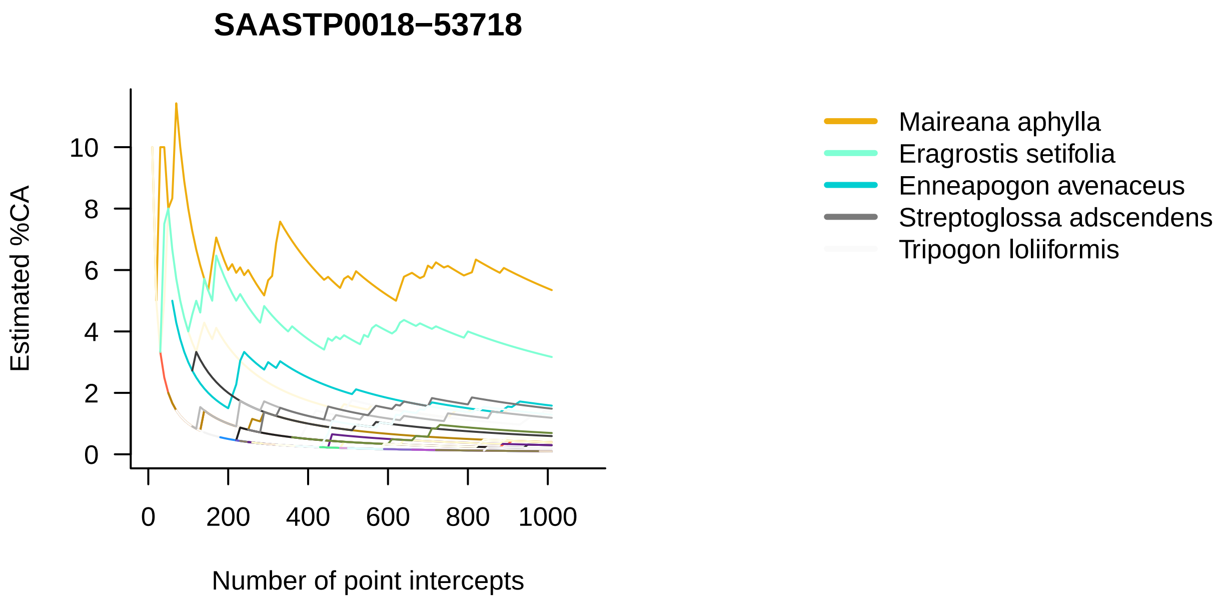
SAASTP0018−53718
Estimated %CA
0
2
4
6
8
10
0
200
400
600
800
1000
Number of point intercepts
Maireana aphylla
Eragrostis setifolia
Enneapogon avenaceus
Streptoglossa adscendens
Tripogon loliiformis

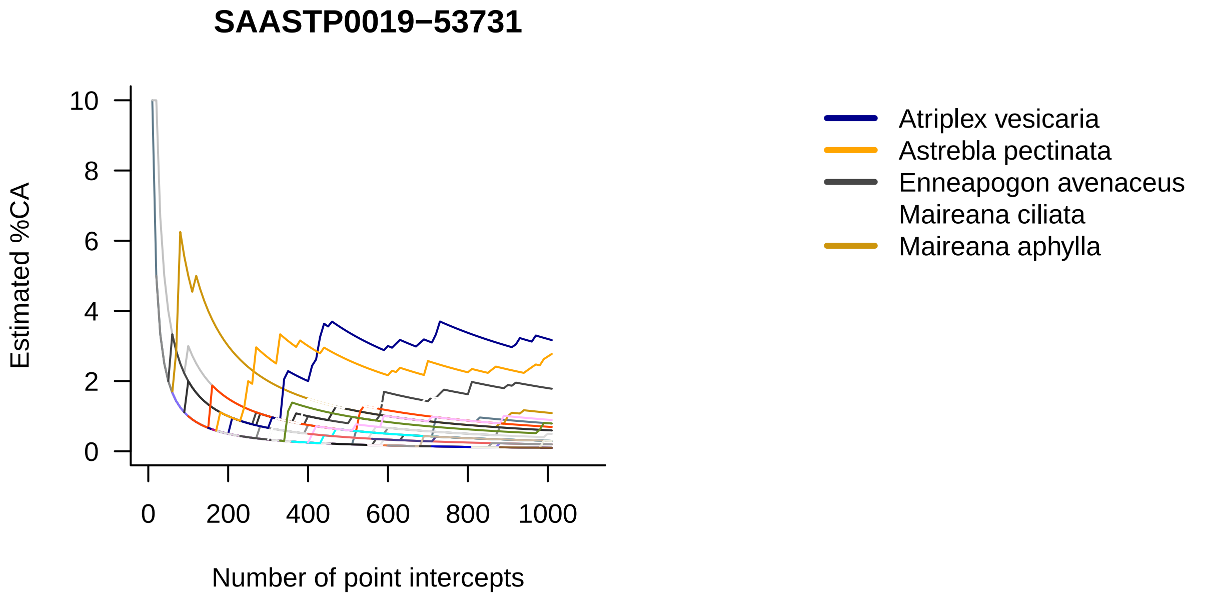
SAASTP0019−53731
Estimated %CA
0
2
4
6
8
10
0
200
400
600
800
1000
Number of point intercepts
Atriplex vesicaria
Astrebla pectinata
Enneapogon avenaceus
Maireana ciliata
Maireana aphylla

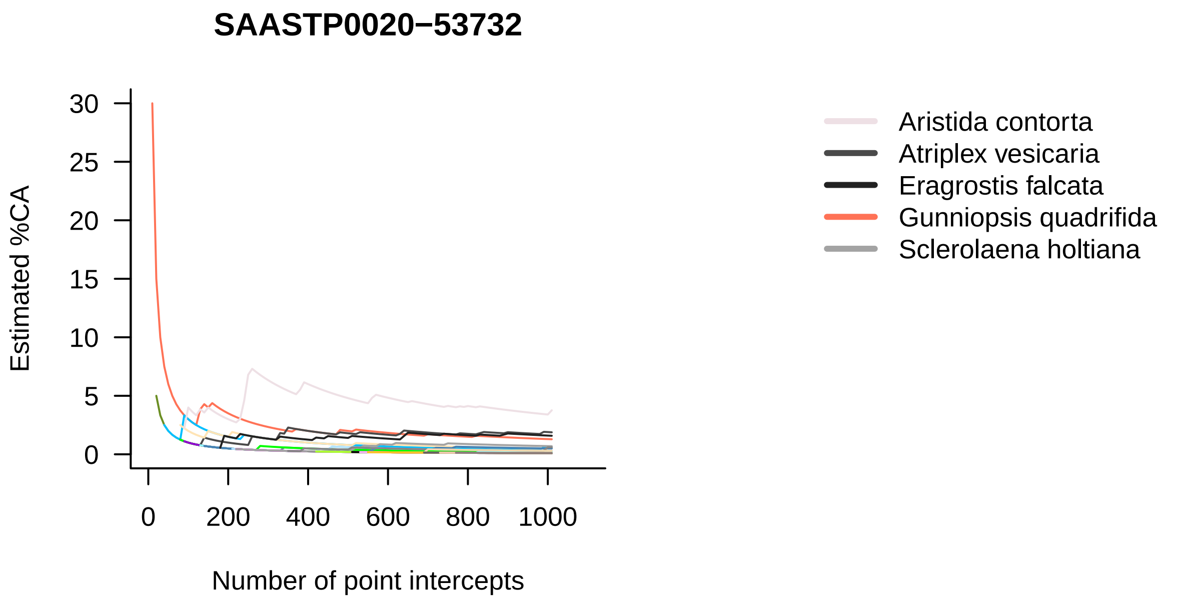
SAASTP0020−53732
Estimated %CA
0
5
10
15
20
25
30
0
200
400
600
800
1000
Number of point intercepts
Aristida contorta
Atriplex vesicaria
Eragrostis falcata
Gunniopsis quadrifida
Sclerolaena holtiana

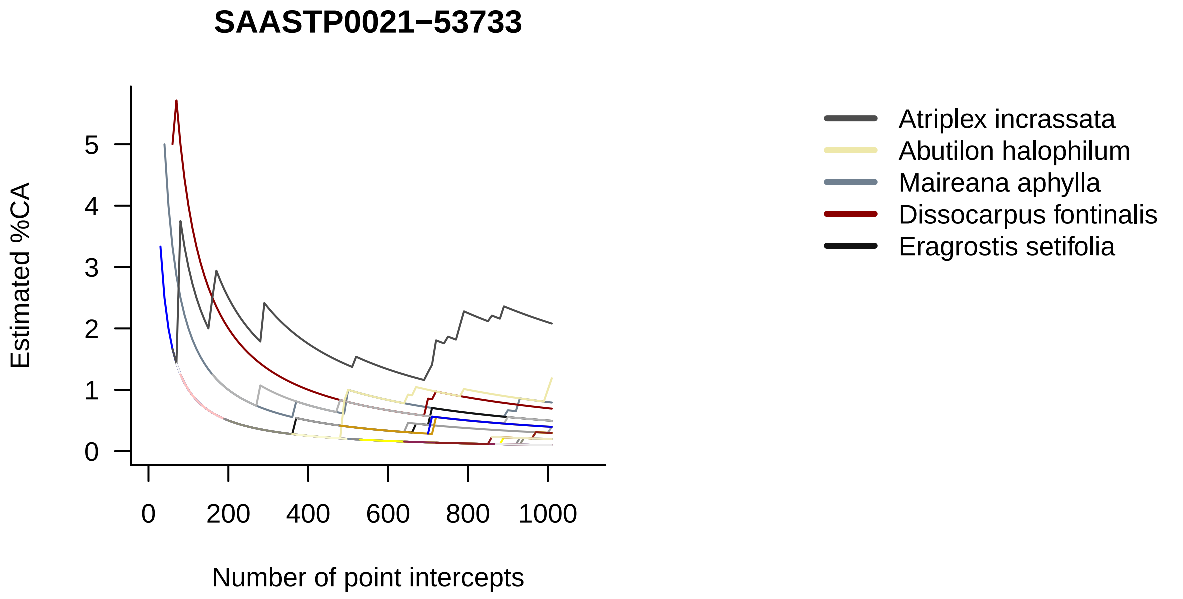
SAASTP0021−53733
Estimated %CA
0
1
2
3
4
5
0
200
400
600
800
1000
Number of point intercepts
Atriplex incrassata
Abutilon halophilum
Maireana aphylla
Dissocarpus fontinalis
Eragrostis setifolia

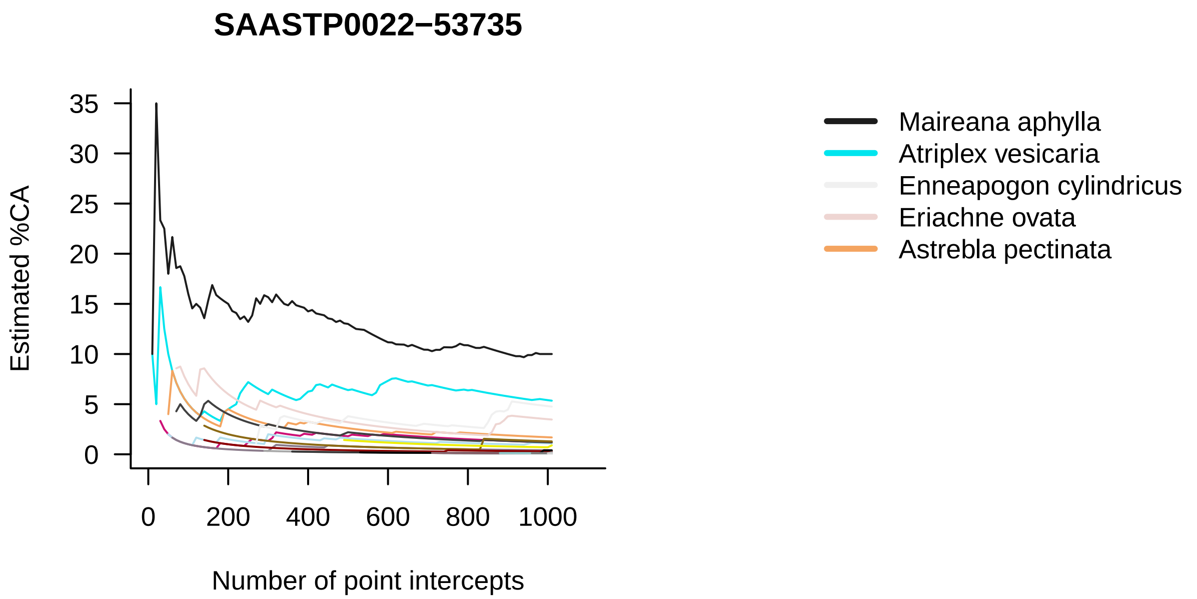
SAASTP0022−53735
Estimated %CA
35
30
25
20
15
10
5
0
0
200
400
600
800
1000
Number of point intercepts
Maireana aphylla
Atriplex vesicaria
Enneapogon cylindricus
Eriachne ovata
Astrebla pectinata

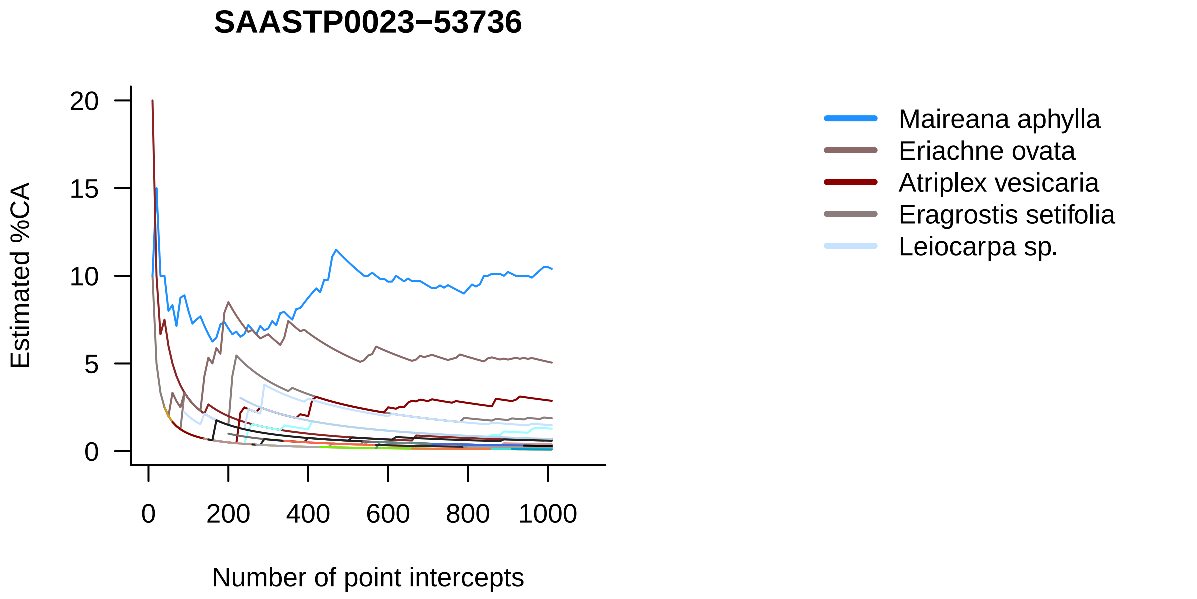
SAASTP0023−53736
Estimated %CA
20
15
10
5
0
0
200
400
600
800
1000
Number of point intercepts
Maireana aphylla
Eriachne ovata
Atriplex vesicaria
Eragrostis setifolia
Leiocarpa sp.

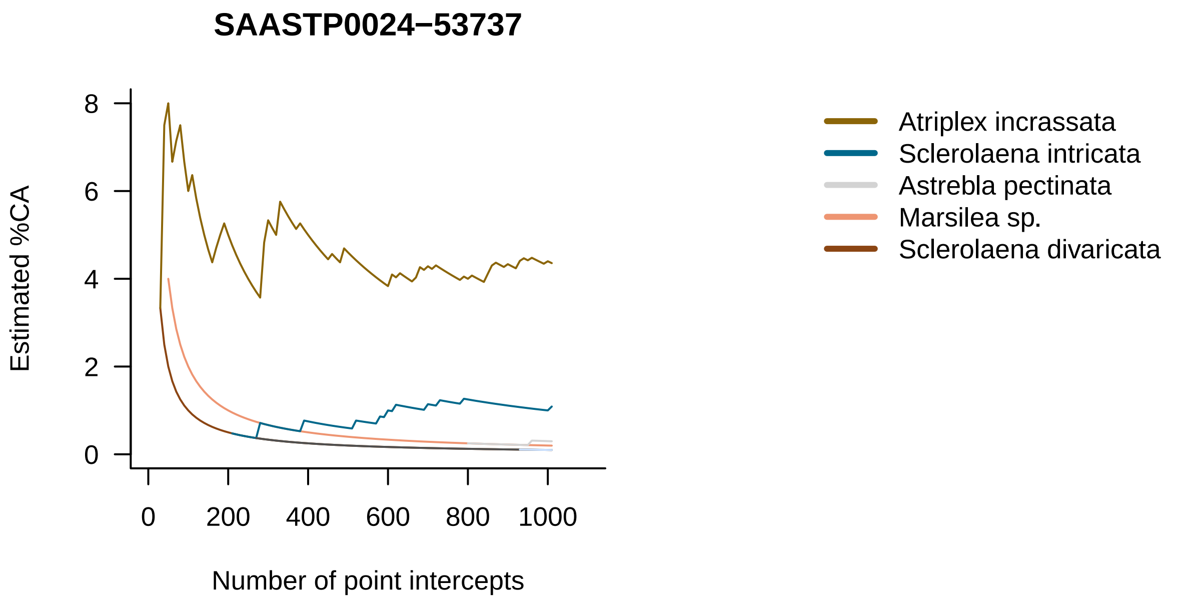
SAASTP0024−53737
Estimated %CA
8
6
4
2
0
0
200
400
600
800
1000
Number of point intercepts
Atriplex incrassata
Sclerolaena intricata
Astrebla pectinata
Marsilea sp.
Sclerolaena divaricata

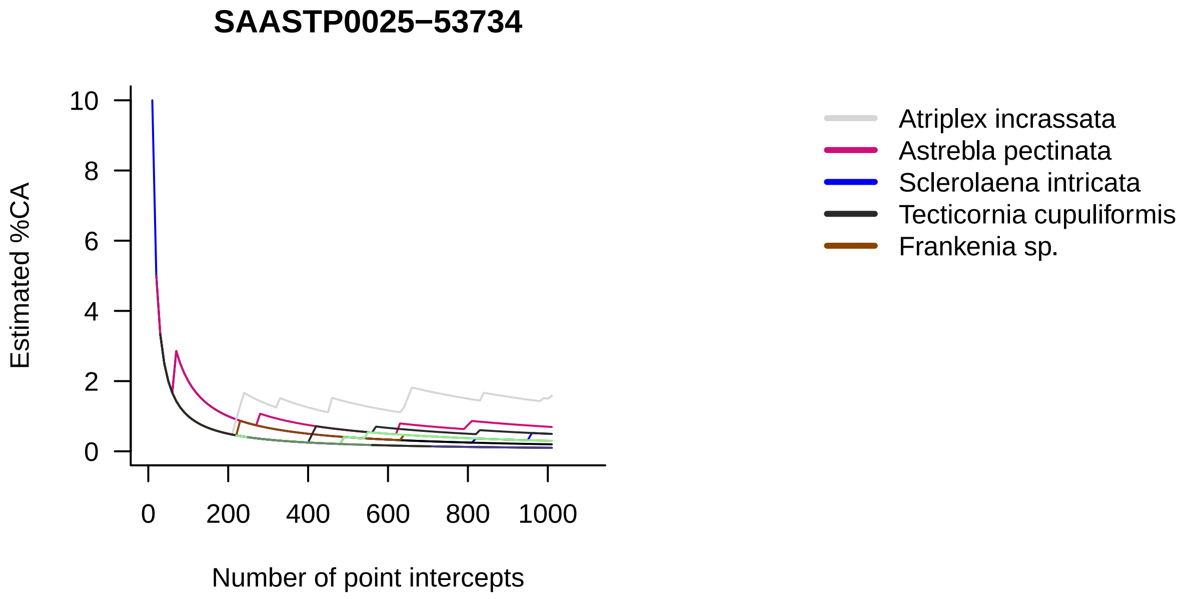
SAASTP0025−53734
Estimated %CA
10
8
6
4
2
0
0
200
400
600
800
1000
Number of point intercepts
Atriplex incrassata
Astrebla pectinata
Sclerolaena intricata
Tecticornia cupuliformis
Frankenia sp.

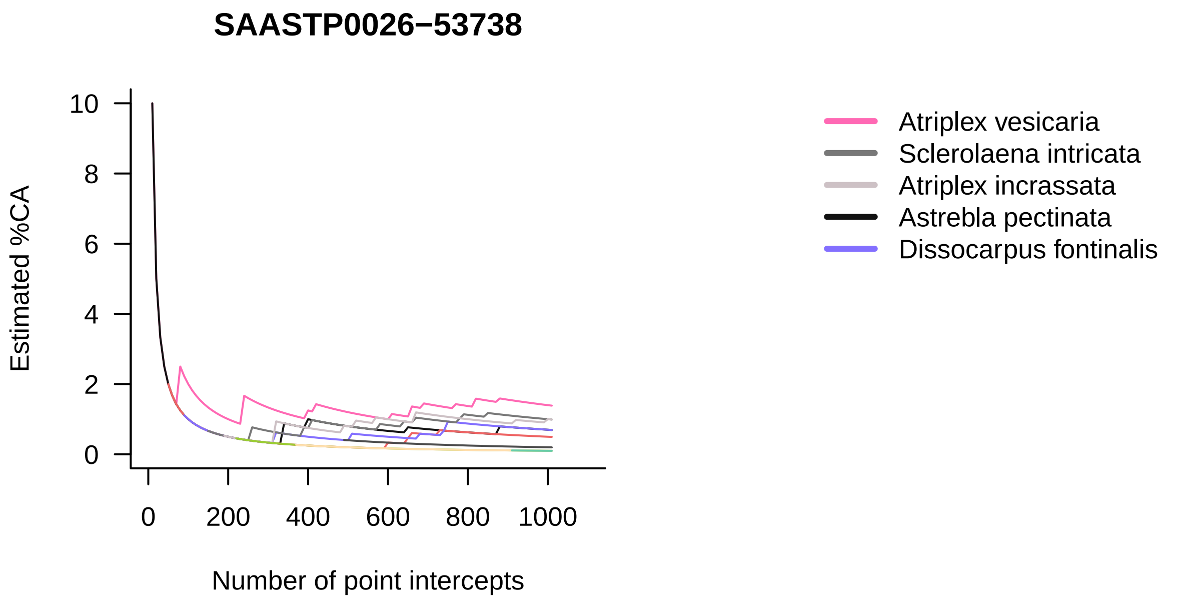
SAASTP0026−53738
Estimated %CA
Number of point intercepts
0
2
4
6
8
10
0
200
400
600
800
1000
Atriplex vesicaria
Sclerolaena intricata
Atriplex incrassata
Astrebla pectinata
Dissocarpus fontinalis

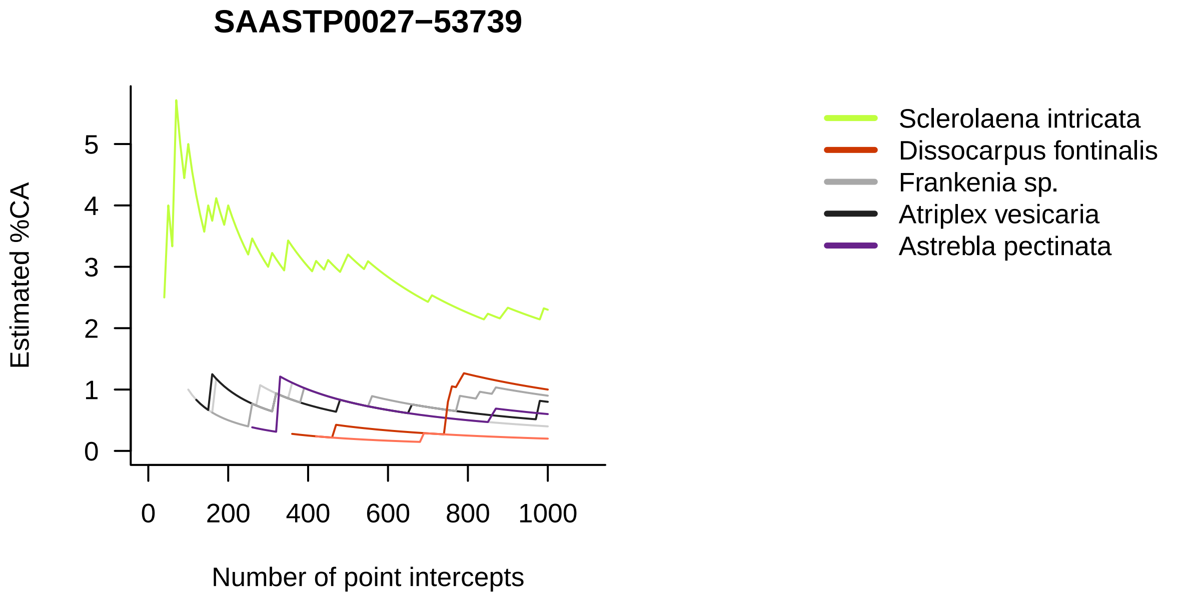
SAASTP0027−53739
Estimated %CA
Number of point intercepts
0
1
2
3
4
5
0
200
400
600
800
1000
Sclerolaena intricata
Dissocarpus fontinalis
Frankenia sp.
Atriplex vesicaria
Astrebla pectinata

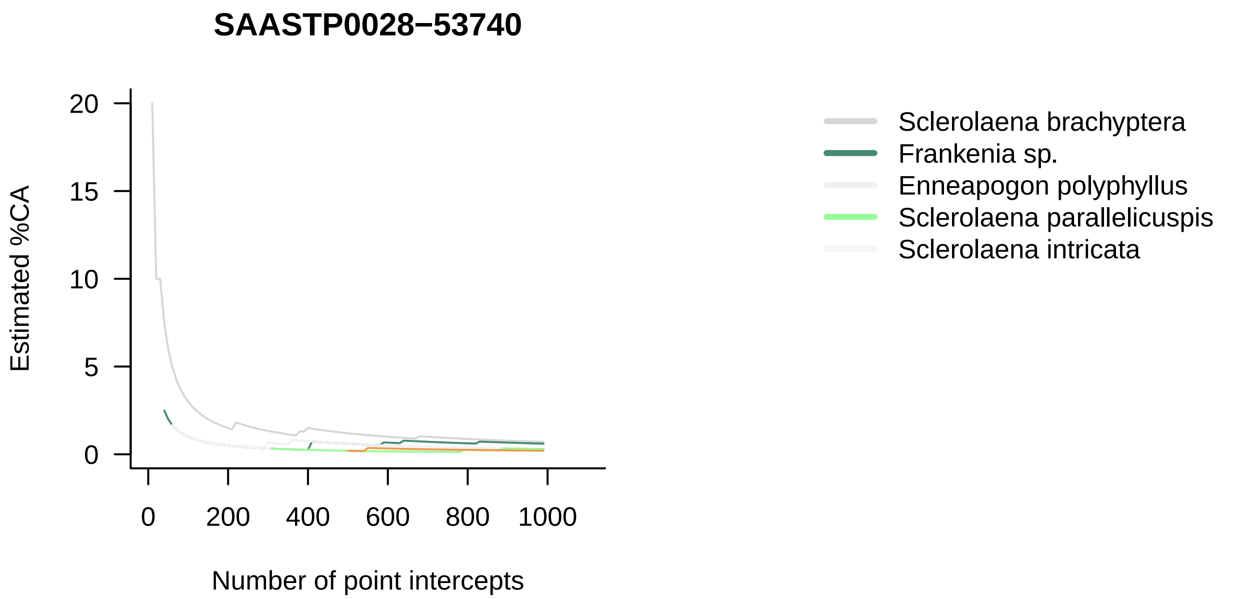
SAASTP0028−53740
Estimated %CA
20
15
10
5
0
0
200
400
600
800
1000
Number of point intercepts
Sclerolaena brachyptera
Frankenia sp.
Enneapogon polyphyllus
Sclerolaena parallelicuspis
Sclerolaena intricata

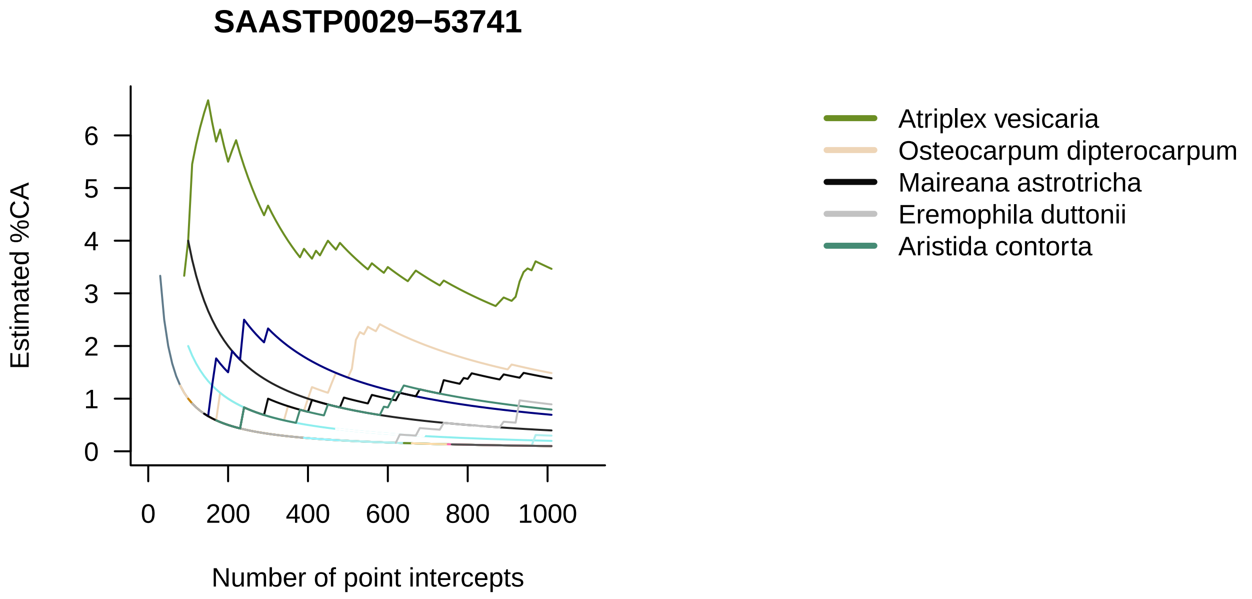
SAASTP0029−53741
Estimated %CA
6
5
4
3
2
1
0
0
200
400
600
800
1000
Number of point intercepts
Atriplex vesicaria
Osteocarpum dipterocarpum
Maireana astrotricha
Eremophila duttonii
Aristida contorta

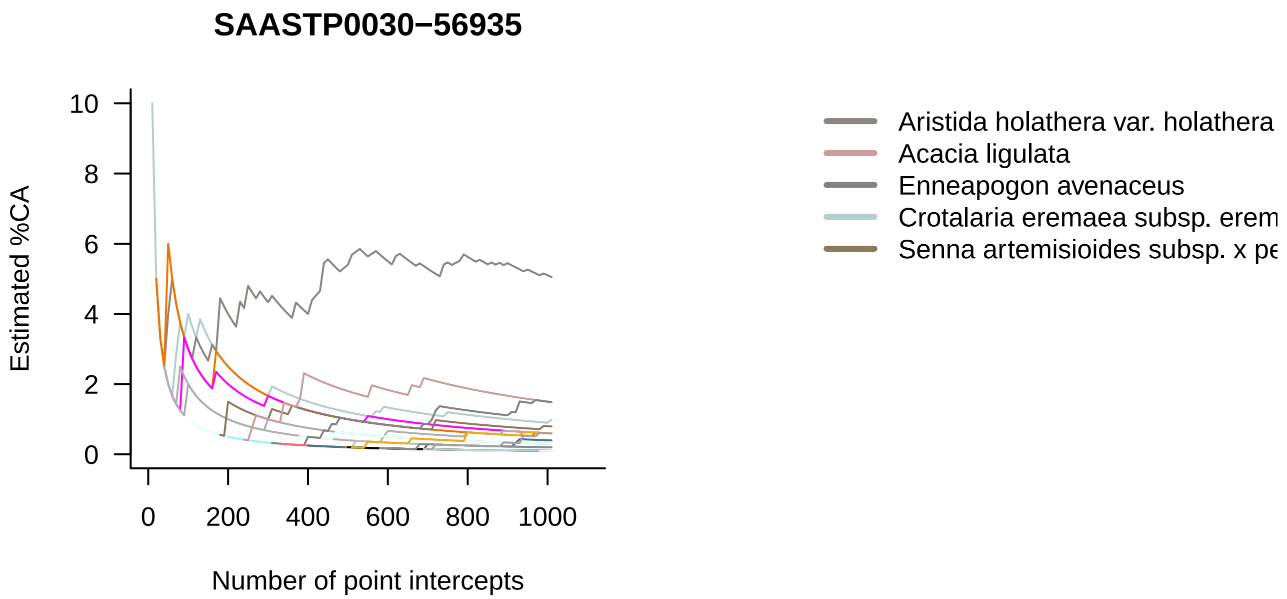
SAASTP0030−56935
Estimated %CA
Number of point intercepts
0
200
400
600
800
1000
0
2
4
6
8
10
Aristida holathera var. holathera
Acacia ligulata
Enneapogon avenaceus
Crotalaria eremaea subsp. erem
Senna artemisioides subsp. x pe

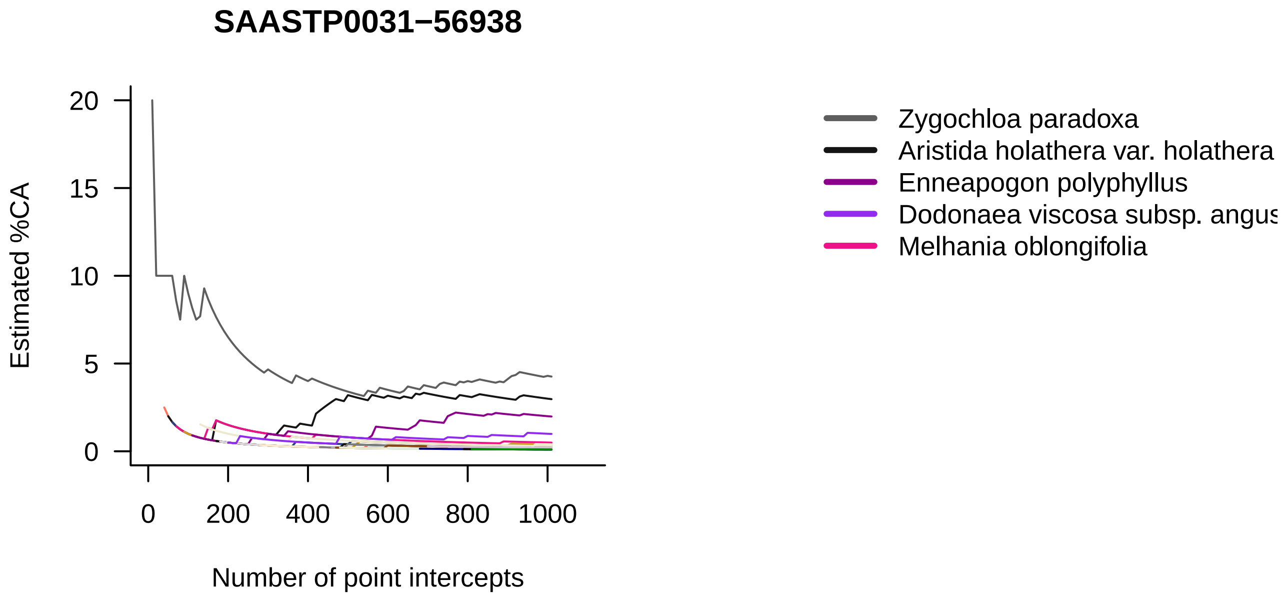
SAASTP0031−56938
Estimated %CA
Number of point intercepts
0
200
400
600
800
1000
0
5
10
15
20
Zygochloa paradoxa
Aristida holathera var. holathera
Enneapogon polyphyllus
Dodonaea viscosa subsp. angus
Melhania oblongifolia

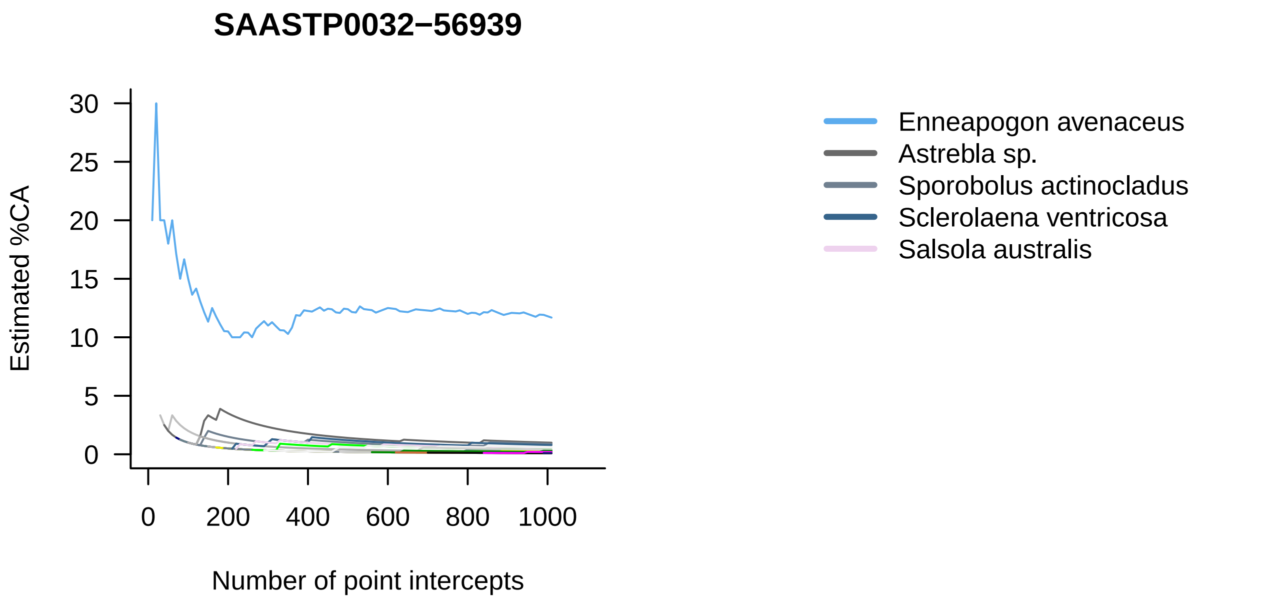
SAASTP0032−56939
Estimated %CA
0
5
10
15
20
25
30
0
200
400
600
800
1000
Number of point intercepts
Enneapogon avenaceus
Astrebla sp.
Sporobolus actinocladus
Sclerolaena ventricosa
Salsola australis

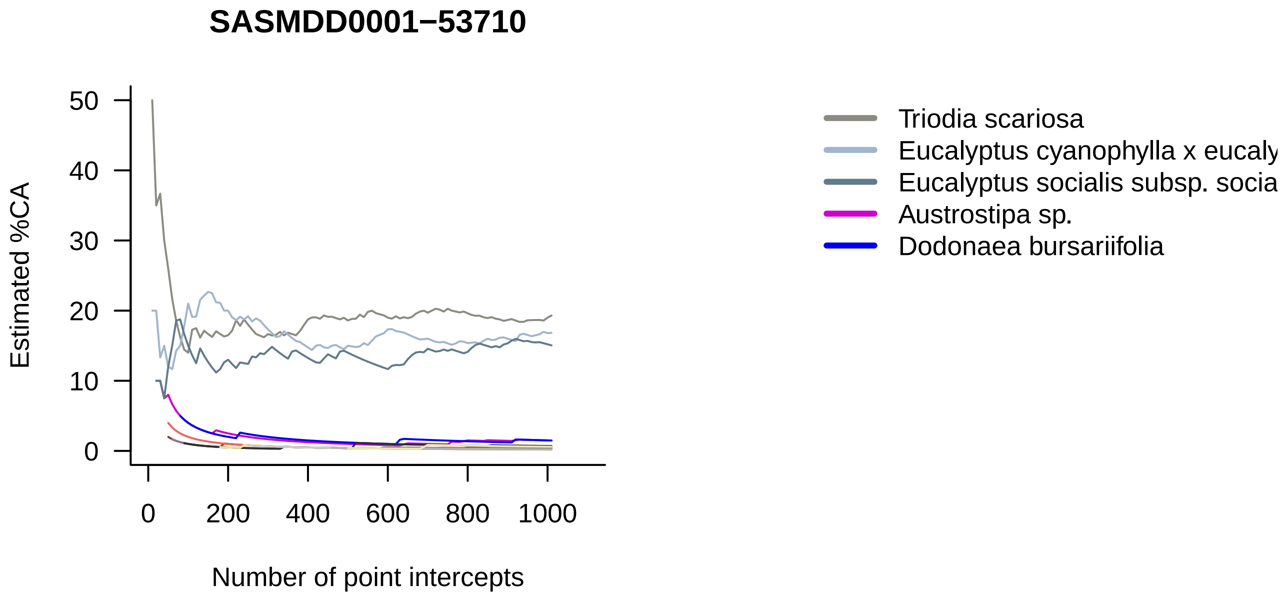
SASMDD0001−53710
Estimated %CA
0
10
20
30
40
50
0
200
400
600
800
1000
Number of point intercepts
Triodia scariosa
Eucalyptus cyanophylla x eucaly
Eucalyptus socialis subsp. socia
Austrostipa sp.
Dodonaea bursariifolia

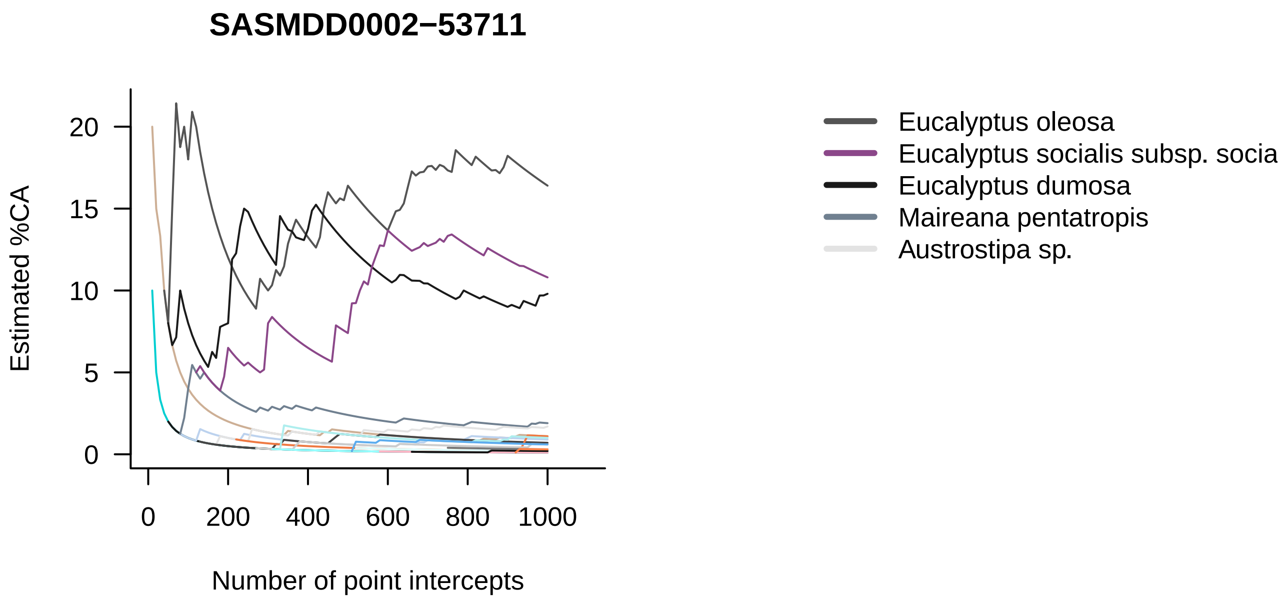
SASMDD0002−53711
Estimated %CA
Number of point intercepts
0
5
10
15
20
0
200
400
600
800
1000
Eucalyptus oleosa
Eucalyptus socialis subsp. socia
Eucalyptus dumosa
Maireana pentatropis
Austrostipa sp.

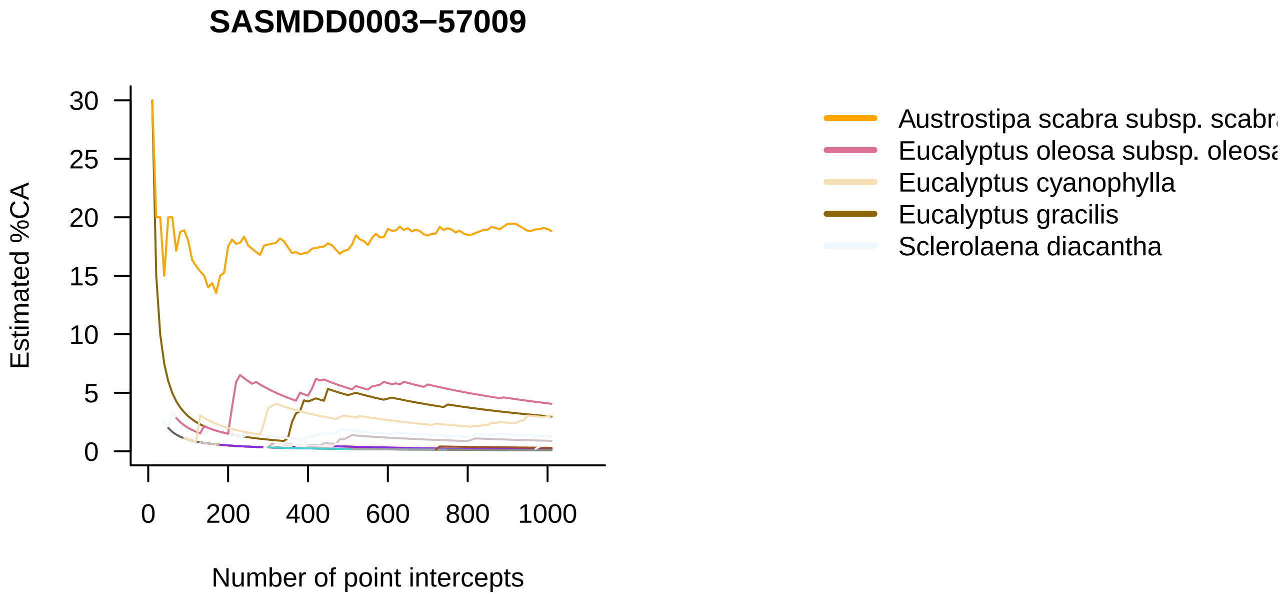
SASMDD0003−57009
Estimated %CA
Number of point intercepts
0
5
10
15
20
25
30
0
200
400
600
800
1000
Austrostipa scabra subsp. scabra
Eucalyptus oleosa subsp. oleosa
Eucalyptus cyanophylla
Eucalyptus gracilis
Sclerolaena diacantha

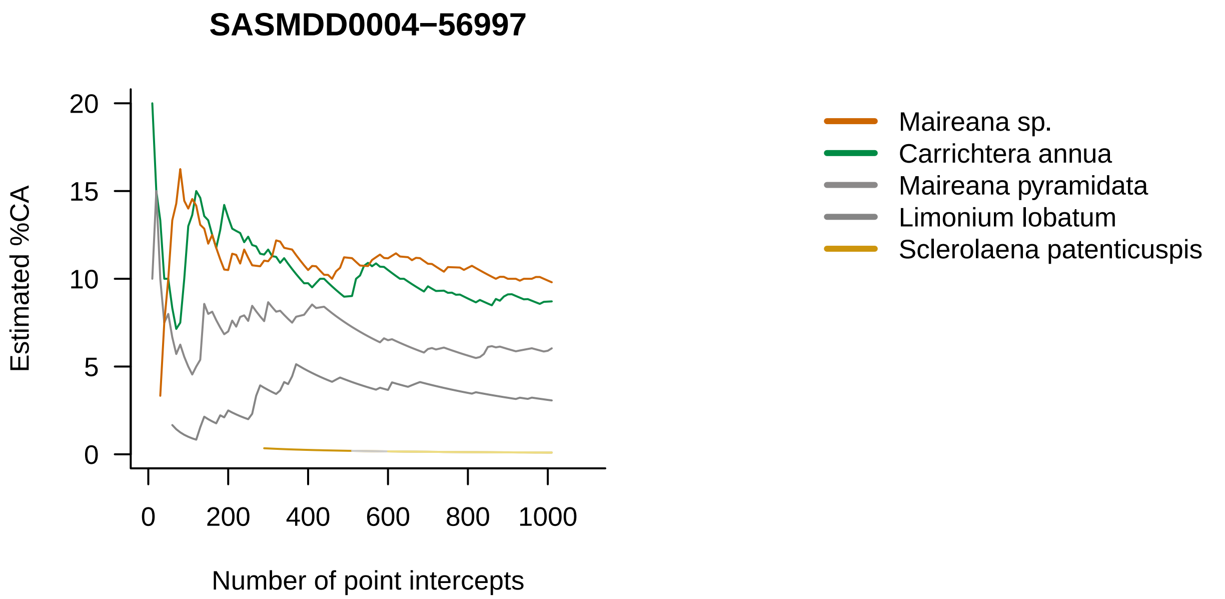
SASMDD0004−56997
Estimated %CA
20
15
10
5
0
0
200
400
600
800
1000
Number of point intercepts
Maireana sp.
Carrichtera annua
Maireana pyramidata
Limonium lobatum
Sclerolaena patenticuspis

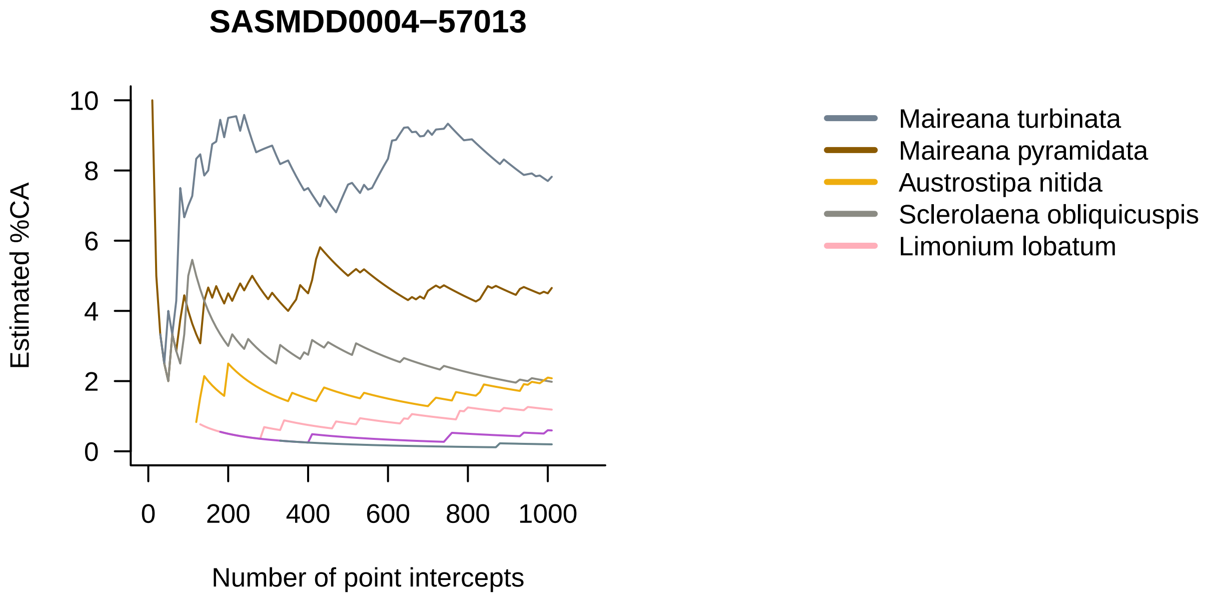
SASMDD0004−57013
Estimated %CA
10
8
6
4
2
0
0
200
400
600
800
1000
Number of point intercepts
Maireana turbinata
Maireana pyramidata
Austrostipa nitida
Sclerolaena obliquicuspis
Limonium lobatum

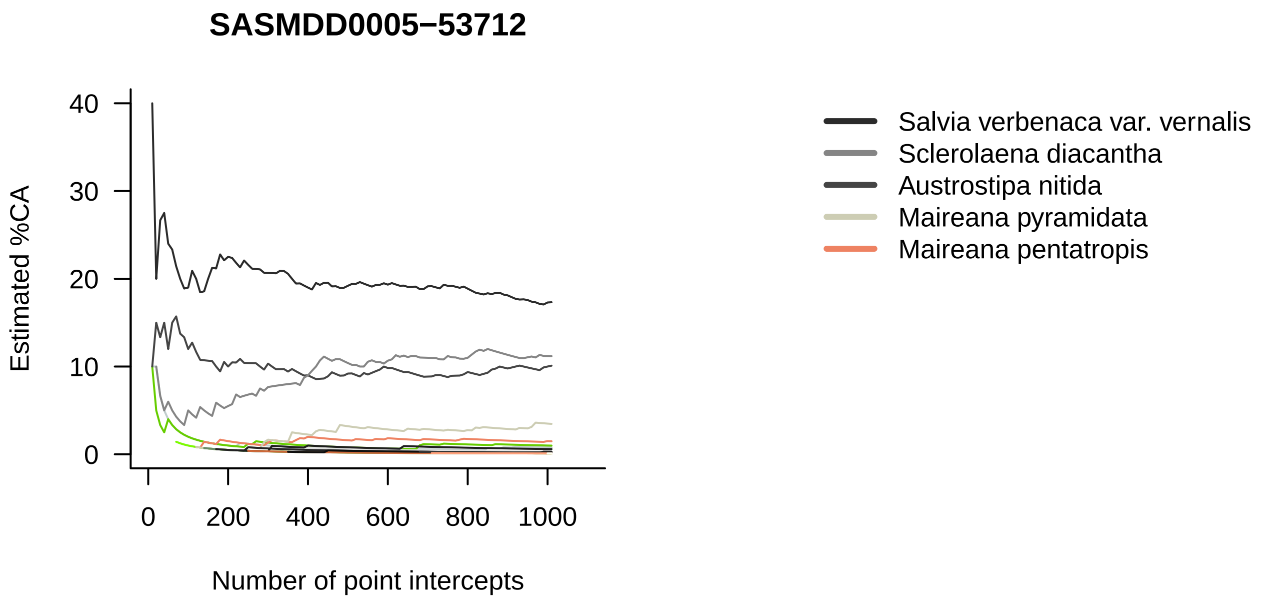
SASMDD0005−53712
Estimated %CA
0
10
20
30
40
0
200
400
600
800
1000
Number of point intercepts
Salvia verbenaca var. vernalis
Sclerolaena diacantha
Austrostipa nitida
Maireana pyramidata
Maireana pentatropis

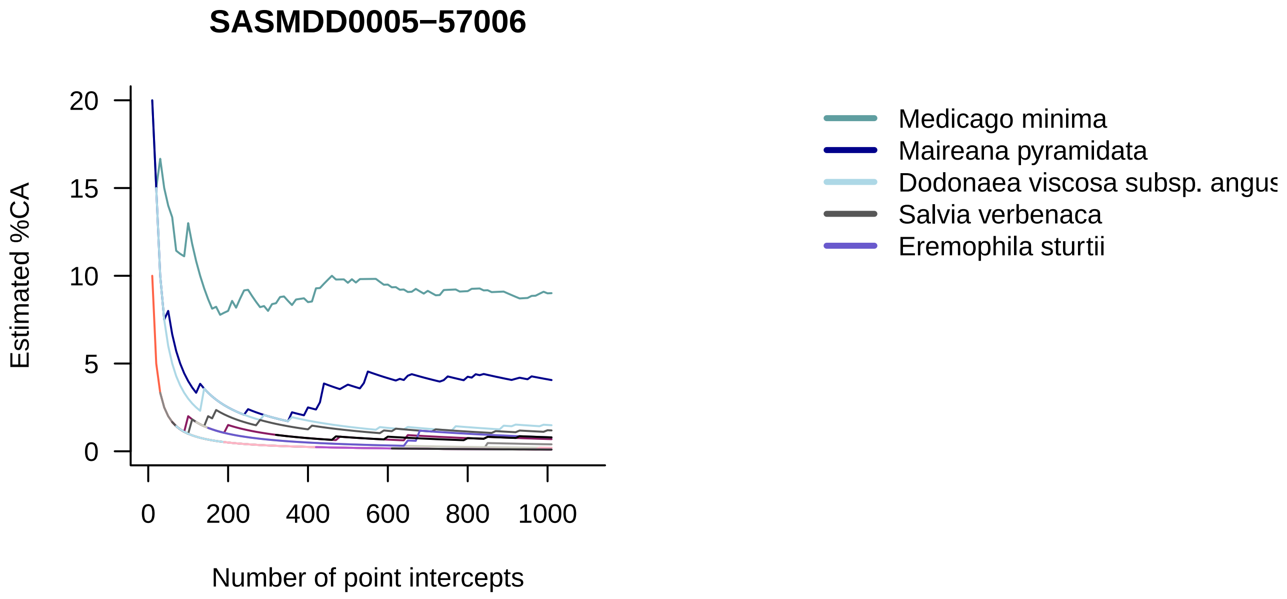
SASMDD0005−57006
Estimated %CA
0
5
10
15
20
0
200
400
600
800
1000
Number of point intercepts
Medicago minima
Maireana pyramidata
Dodonaea viscosa subsp. angus
Salvia verbenaca
Eremophila sturtii

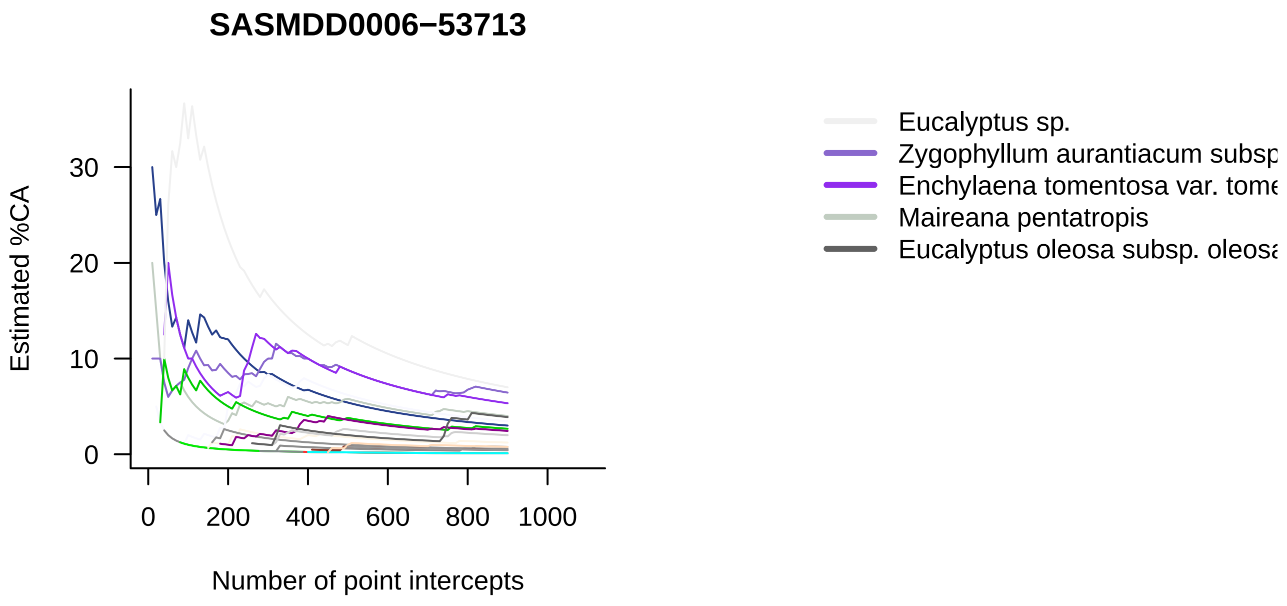
SASMDD0006−53713
Estimated %CA
Number of point intercepts
0
10
20
30
0
200
400
600
800
1000
Eucalyptus sp.
Zygophyllum aurantiacum subsp
Enchylaena tomentosa var. tome
Maireana pentatropis
Eucalyptus oleosa subsp. oleosa

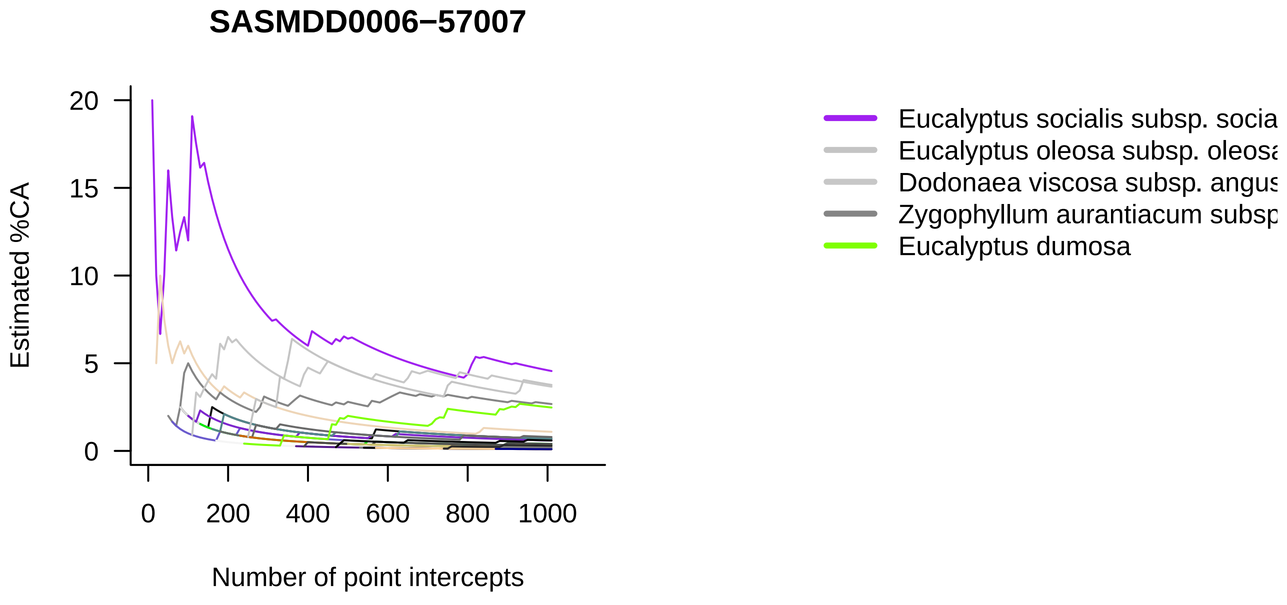
SASMDD0006−57007
Estimated %CA
Number of point intercepts
0
5
10
15
20
0
200
400
600
800
1000
Eucalyptus socialis subsp. socia
Eucalyptus oleosa subsp. oleosa
Dodonaea viscosa subsp. angus
Zygophyllum aurantiacum subsp
Eucalyptus dumosa

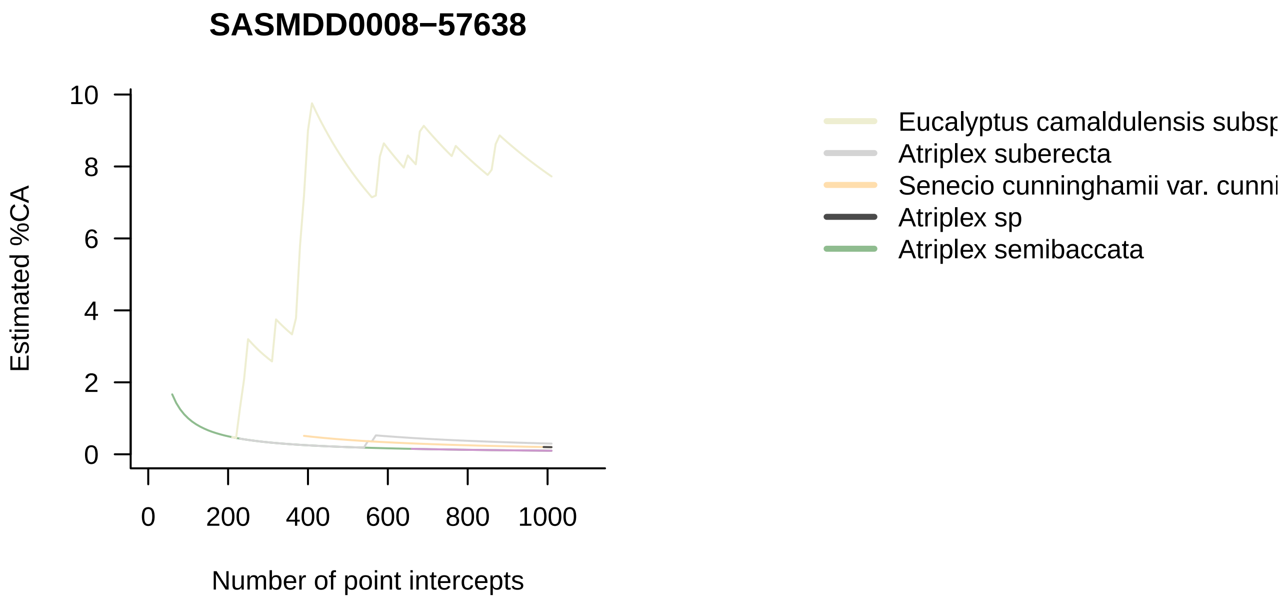
SASMDD0008−57638
Estimated %CA
Number of point intercepts
0
2
4
6
8
10
0
200
400
600
800
1000
Eucalyptus camaldulensis subsp
Atriplex suberecta
Senecio cunninghamii var. cunni
Atriplex sp
Atriplex semibaccata

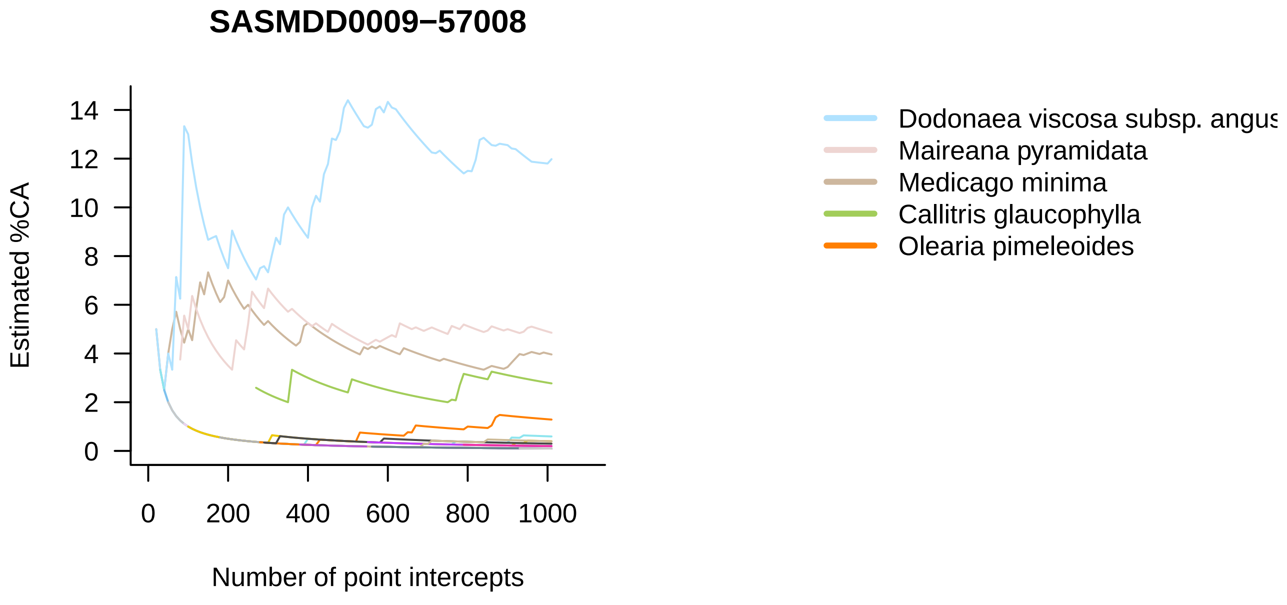
SASMDD0009−57008
Estimated %CA
Number of point intercepts
0
2
4
6
8
10
12
14
0
200
400
600
800
1000
Dodonaea viscosa subsp. angus
Maireana pyramidata
Medicago minima
Callitris glaucophylla
Olearia pimeleoides

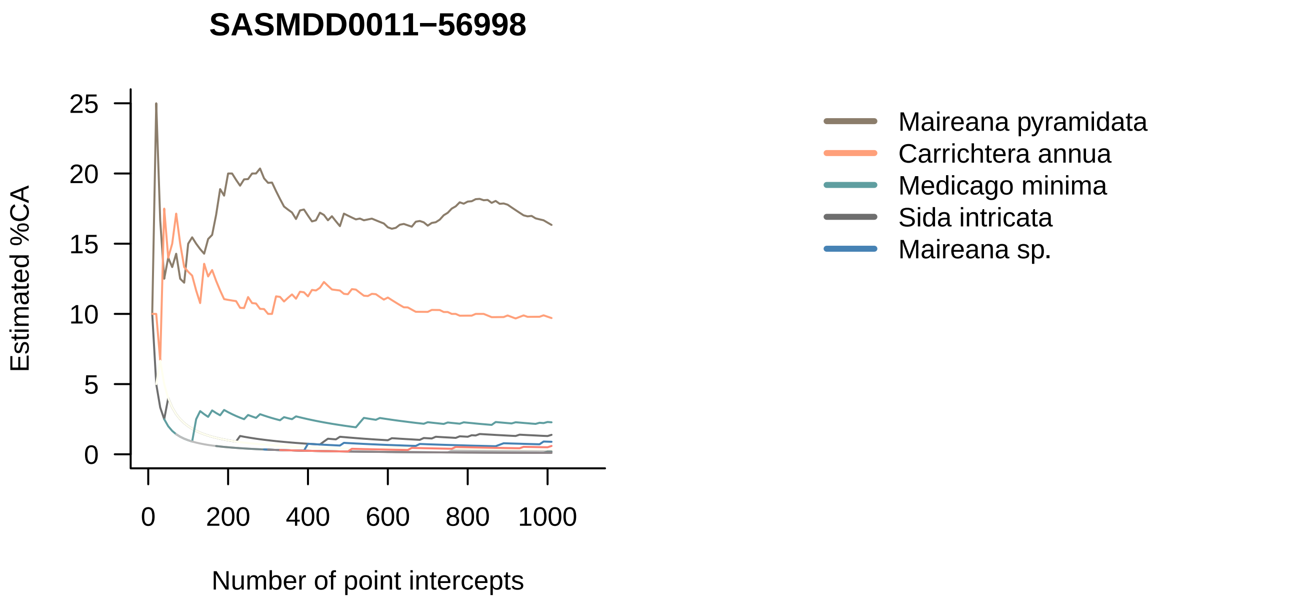
SASMDD0011−56998
Estimated %CA
Number of point intercepts
Maireana pyramidata
Carrichtera annua
Medicago minima
Sida intricata
Maireana sp.

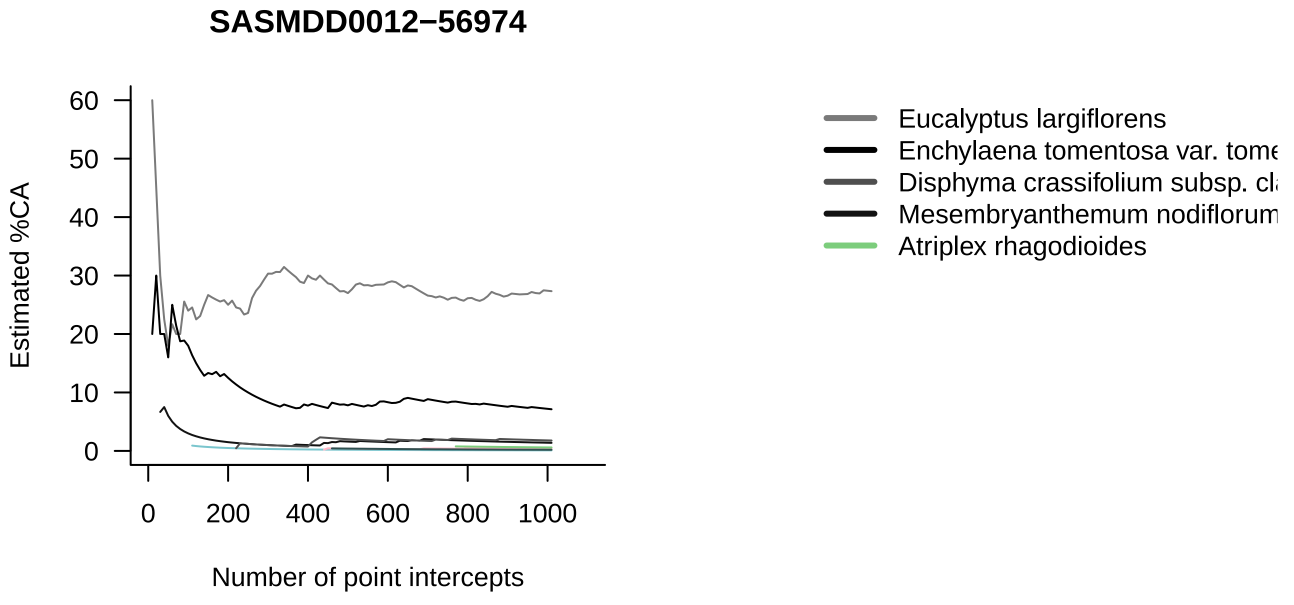
SASMDD0012−56974
Estimated %CA
Number of point intercepts
Eucalyptus largiflorens
Enchylaena tomentosa var. tome
Disphyma crassifolium subsp. cl
Mesembryanthemum nodiflorum
Atriplex rhagodioides

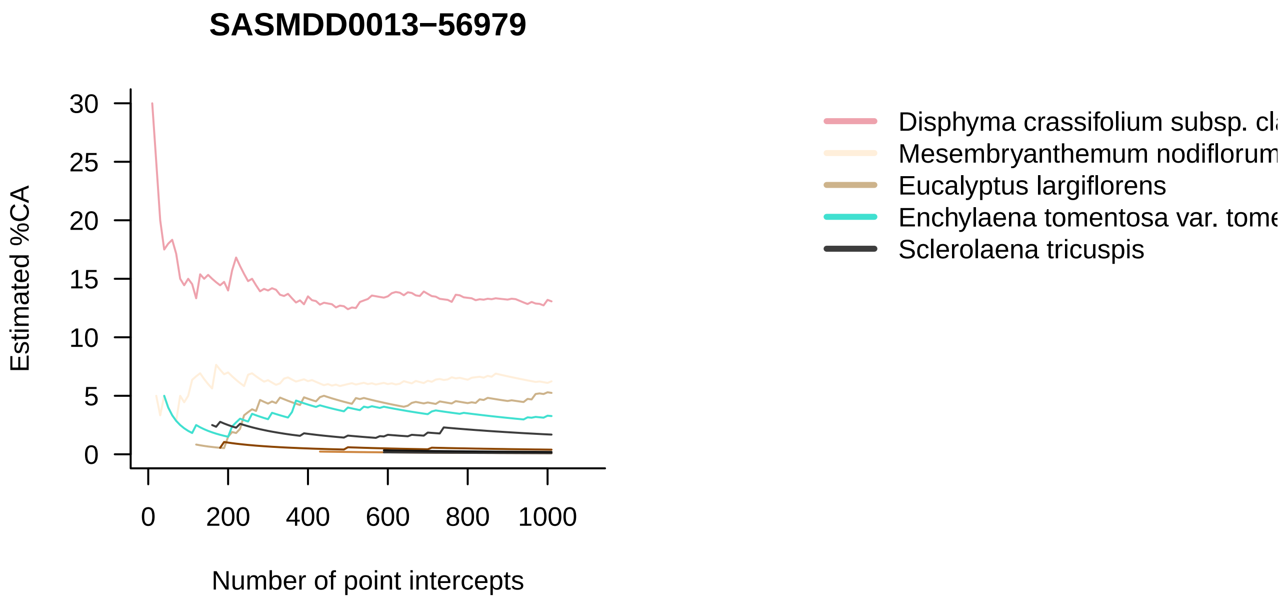
SASMDD0013−56979
Estimated %CA
30
25
20
15
10
5
0
0
200
400
600
800
1000
Number of point intercepts
Disphyma crassifolium subsp. cla
Mesembryanthemum nodiflorum
Eucalyptus largiflorens
Enchylaena tomentosa var. tome
Sclerolaena tricuspis

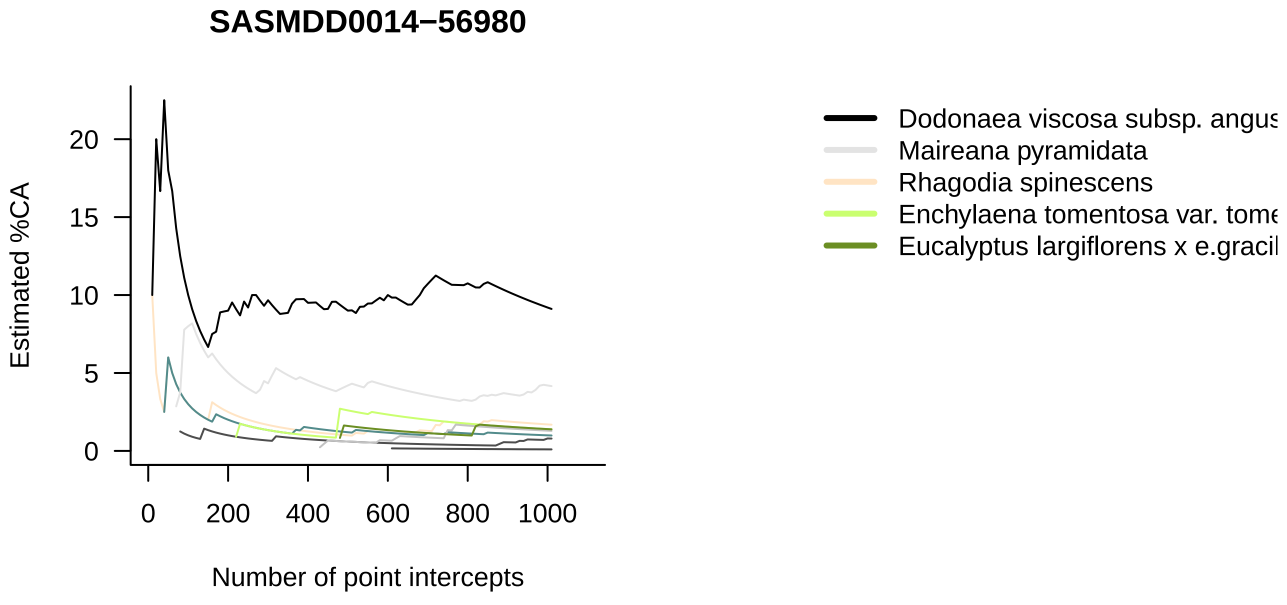
SASMDD0014−56980
Estimated %CA
20
15
10
5
0
0
200
400
600
800
1000
Number of point intercepts
Dodonaea viscosa subsp. angus
Maireana pyramidata
Rhagodia spinescens
Enchylaena tomentosa var. tome
Eucalyptus largiflorens x e.gracil

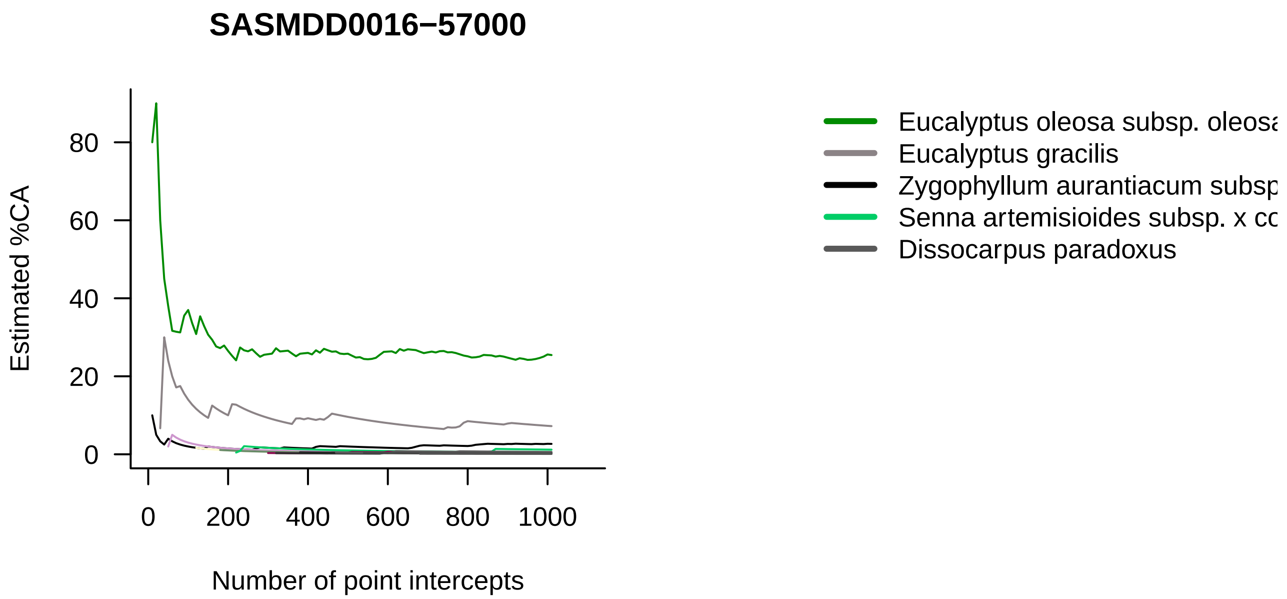
SASMDD0016−57000
Estimated %CA
80
60
40
20
0
0
200
400
600
800
1000
Number of point intercepts
Eucalyptus oleosa subsp. oleosa
Eucalyptus gracilis
Zygophyllum aurantiacum subsp
Senna artemisioides subsp. x co
Dissocarpus paradoxus

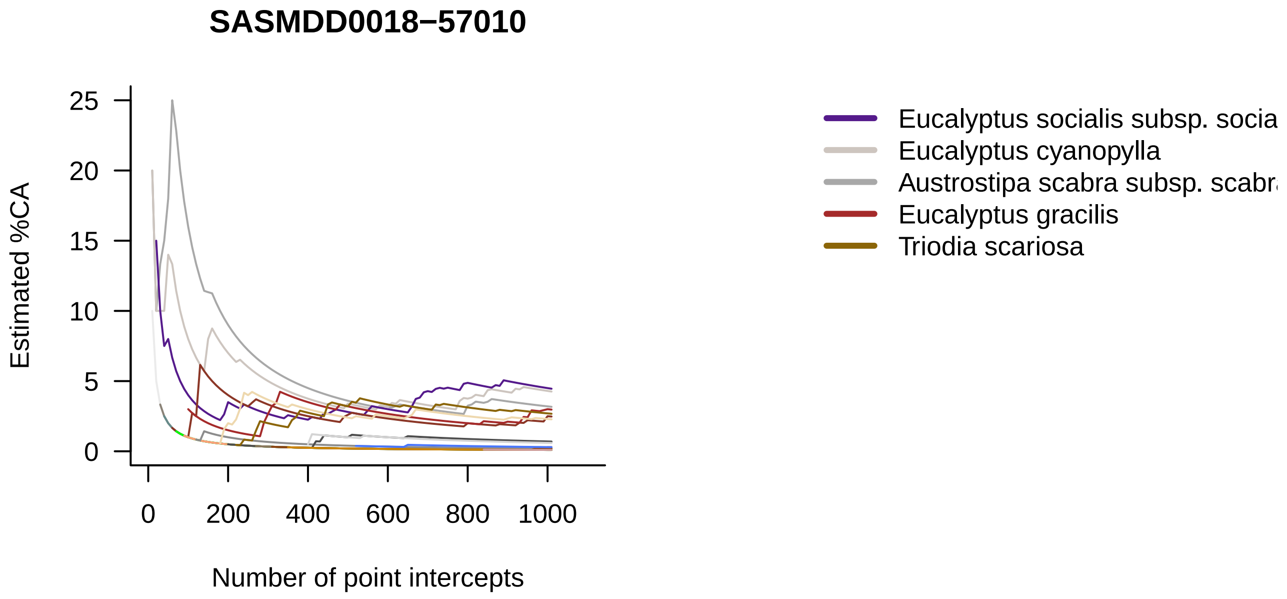
SASMDD0018−57010
Estimated %CA
25
20
15
10
5
0
0
200
400
600
800
1000
Number of point intercepts
Eucalyptus socialis subsp. socia
Eucalyptus cyanopylla
Austrostipa scabra subsp. scabra
Eucalyptus gracilis
Triodia scariosa

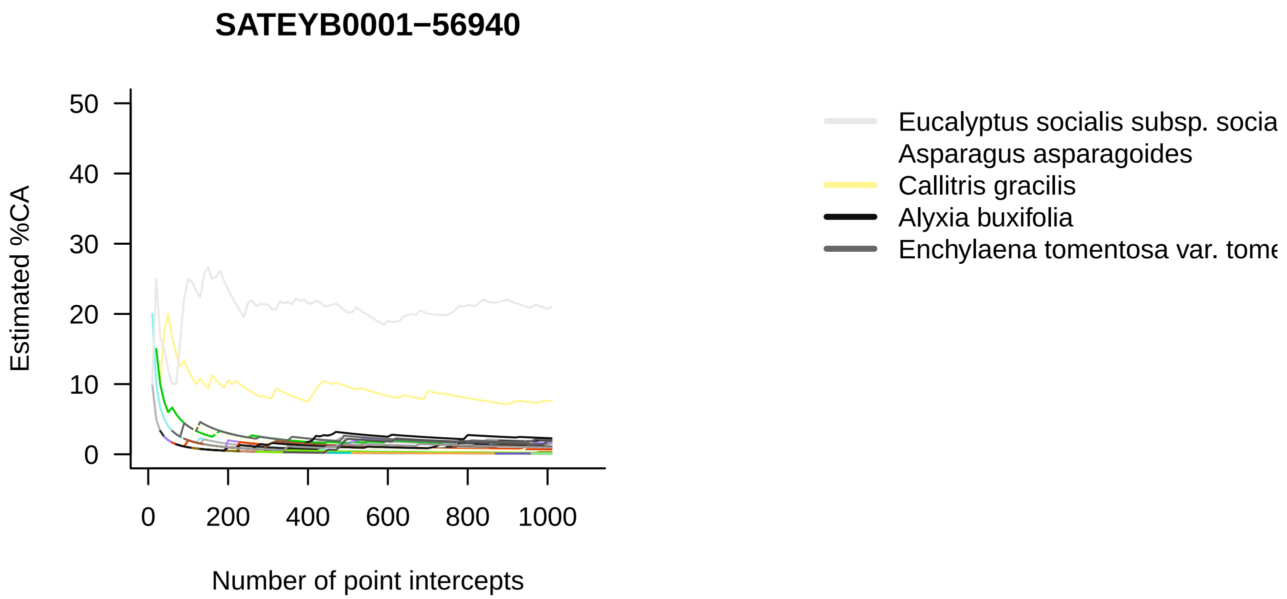

SATEYB0001−56940
Estimated %CA
0
10
20
30
40
50
0
200
400
600
800
1000
Number of point intercepts
Eucalyptus socialis subsp. socia
Asparagus asparagoides
Callitris gracilis
Alyxia buxifolia
Enchylaena tomentosa var. tome


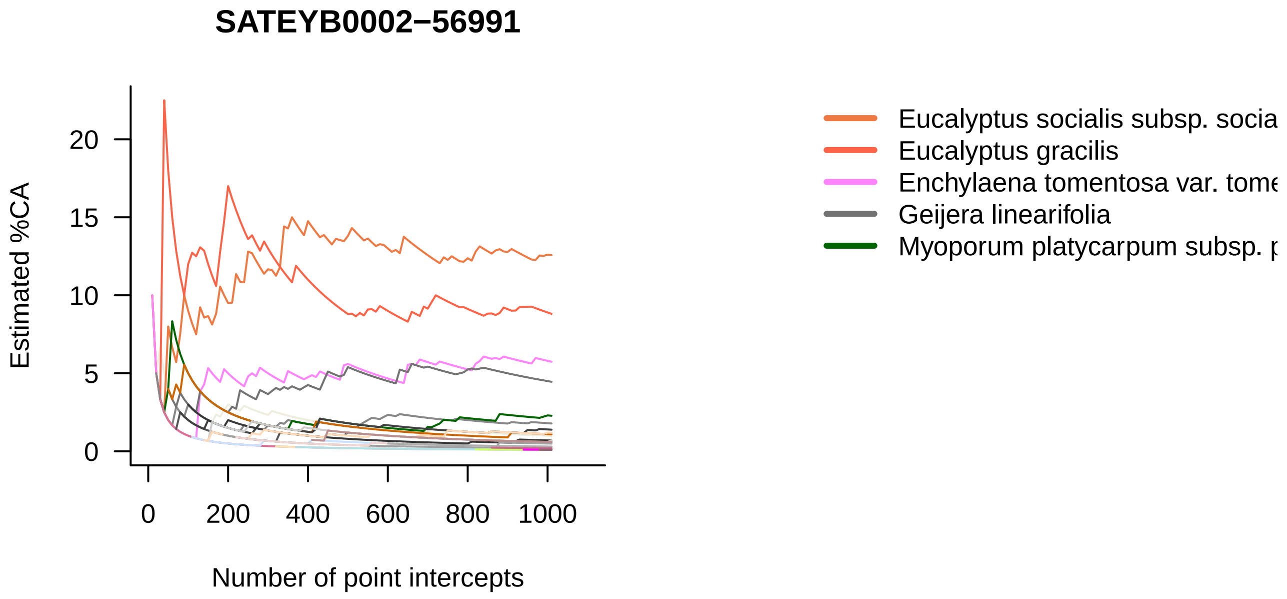

SATEYB0002−56991
Estimated %CA
0
5
10
15
20
0
200
400
600
800
1000
Number of point intercepts
Eucalyptus socialis subsp. socia
Eucalyptus gracilis
Enchylaena tomentosa var. tome
Geijera linearifolia
Myoporum platycarpum subsp. p

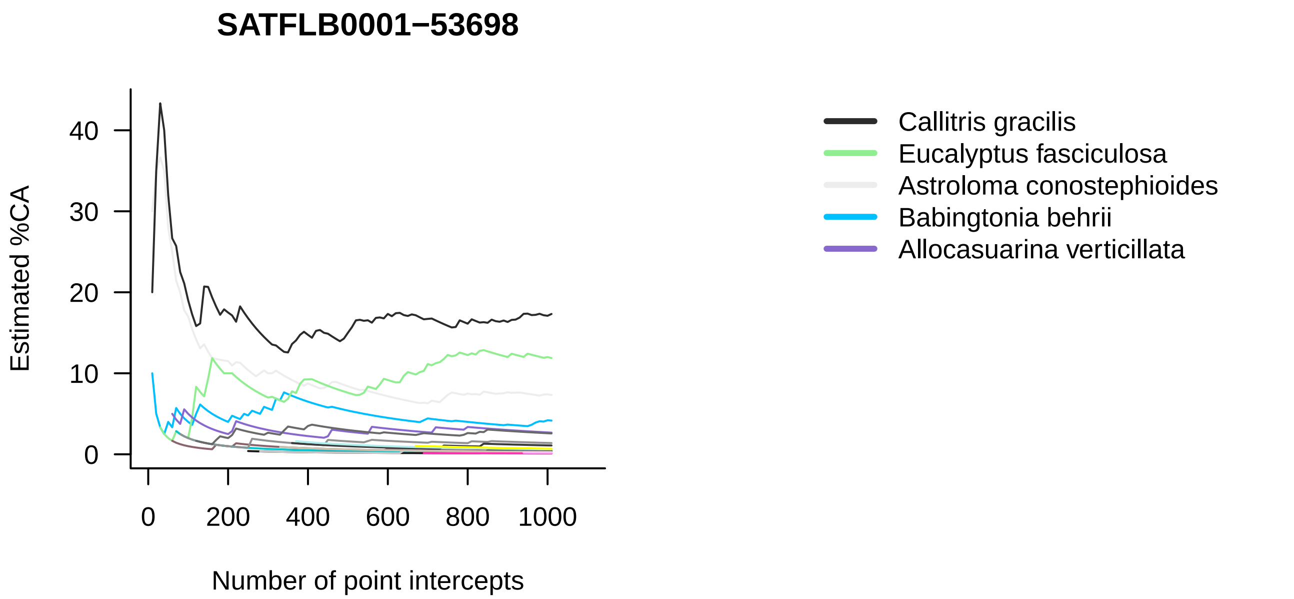
SATFLB0001−53698
Estimated %CA
0
10
20
30
40
0
200
400
600
800
1000
Number of point intercepts
Callitris gracilis
Eucalyptus fasciculosa
Astroloma conostephioides
Babingtonia behrii
Allocasuarina verticillata

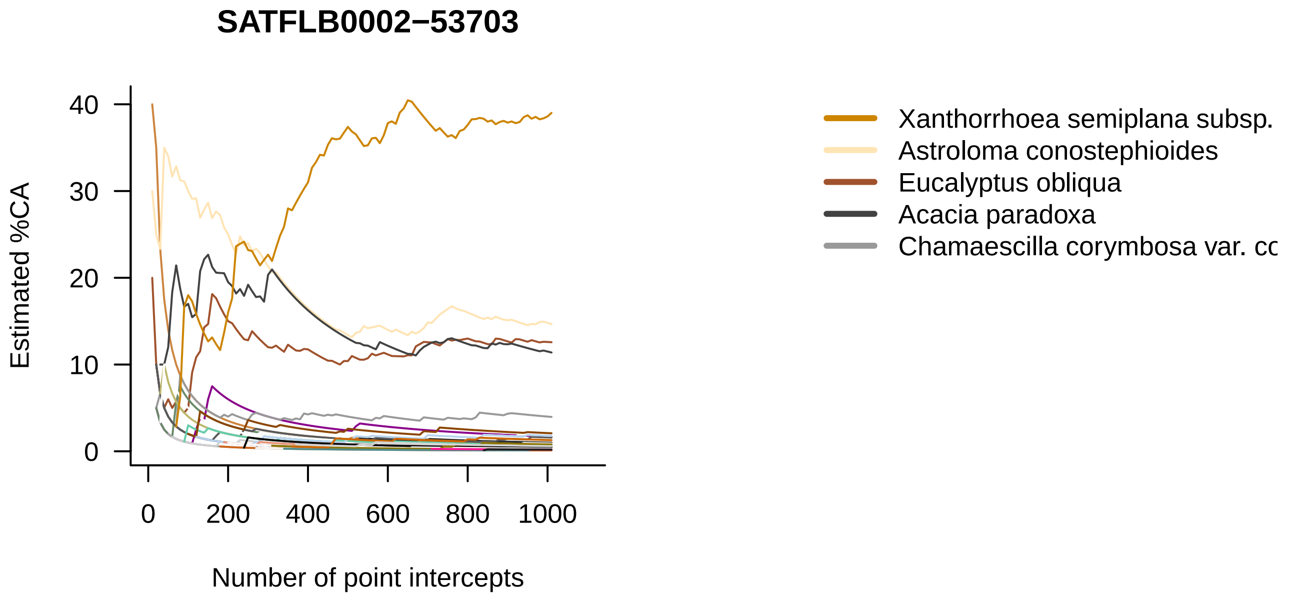
SATFLB0002−53703
Estimated %CA
0
10
20
30
40
0
200
400
600
800
1000
Number of point intercepts
Xanthorrhoea semiplana subsp.
Astroloma conostephioides
Eucalyptus obliqua
Acacia paradoxa
Chamaescilla corymbosa var. cc

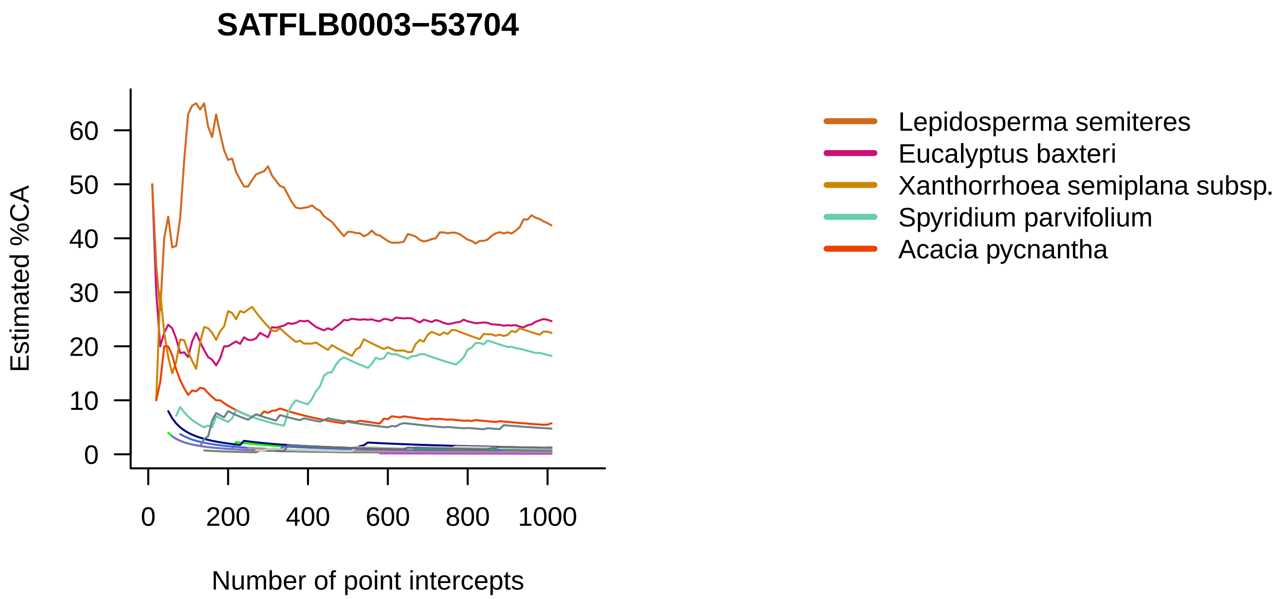
SATFLB0003−53704
Estimated %CA
0
10
20
30
40
50
60
0
200
400
600
800
1000
Number of point intercepts
Lepidosperma semiteres
Eucalyptus baxteri
Xanthorrhoea semiplana subsp.
Spyridium parvifolium
Acacia pycnantha

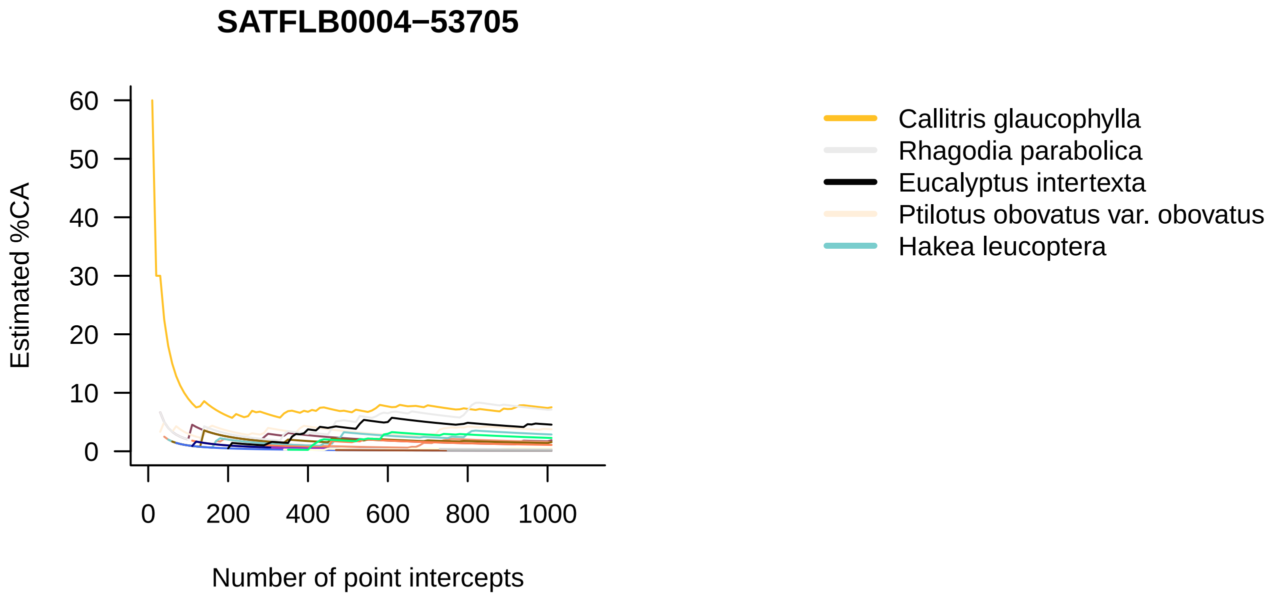
SATFLB0004−53705
Estimated %CA
0
10
20
30
40
50
60
0
200
400
600
800
1000
Number of point intercepts
Callitris glaucophylla
Rhagodia parabolica
Eucalyptus intertexta
Ptilotus obovatus var. obovatus
Hakea leucoptera

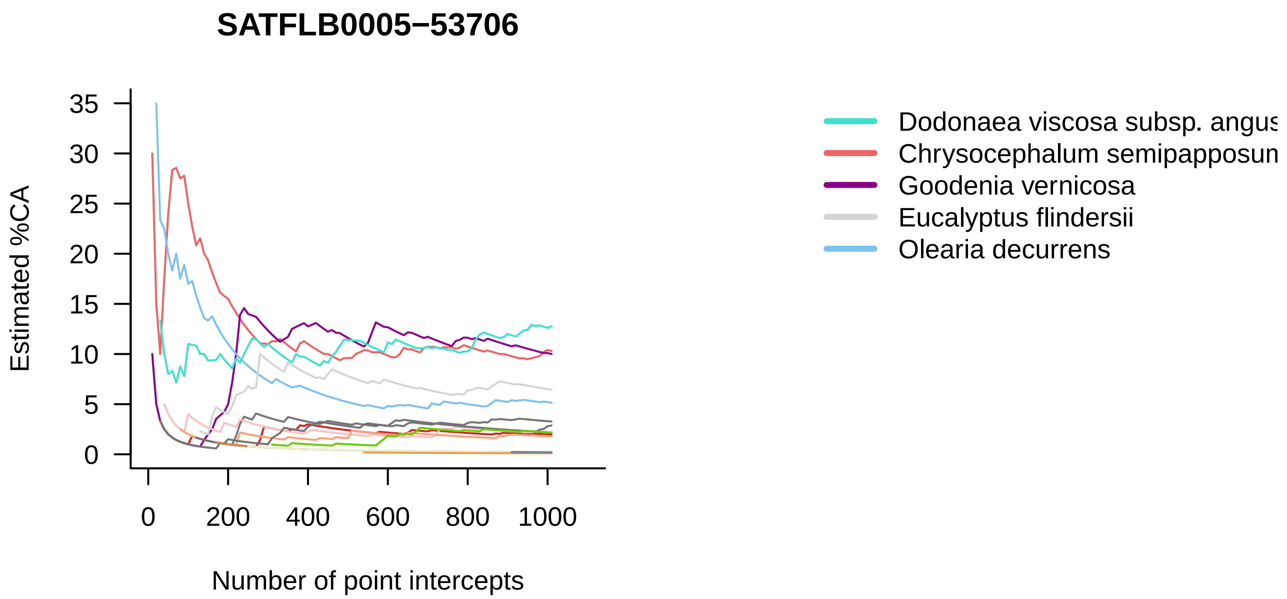
SATFLB0005−53706
Estimated %CA
Number of point intercepts
0
5
10
15
20
25
30
35
0
200
400
600
800
1000
Dodonaea viscosa subsp. angus
Chrysocephalum semipapposun
Goodenia vernicosa
Eucalyptus flindersii
Olearia decurrens

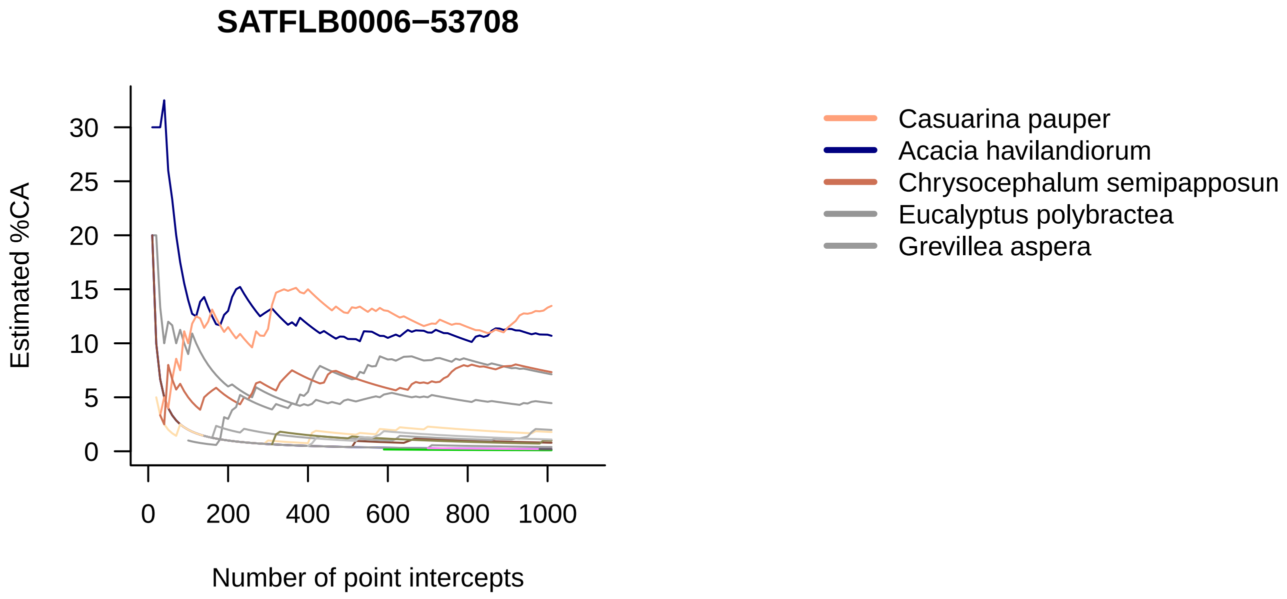
SATFLB0006−53708
Estimated %CA
Number of point intercepts
0
5
10
15
20
25
30
0
200
400
600
800
1000
Casuarina pauper
Acacia havilandiorum
Chrysocephalum semipapposun
Eucalyptus polybractea
Grevillea aspera

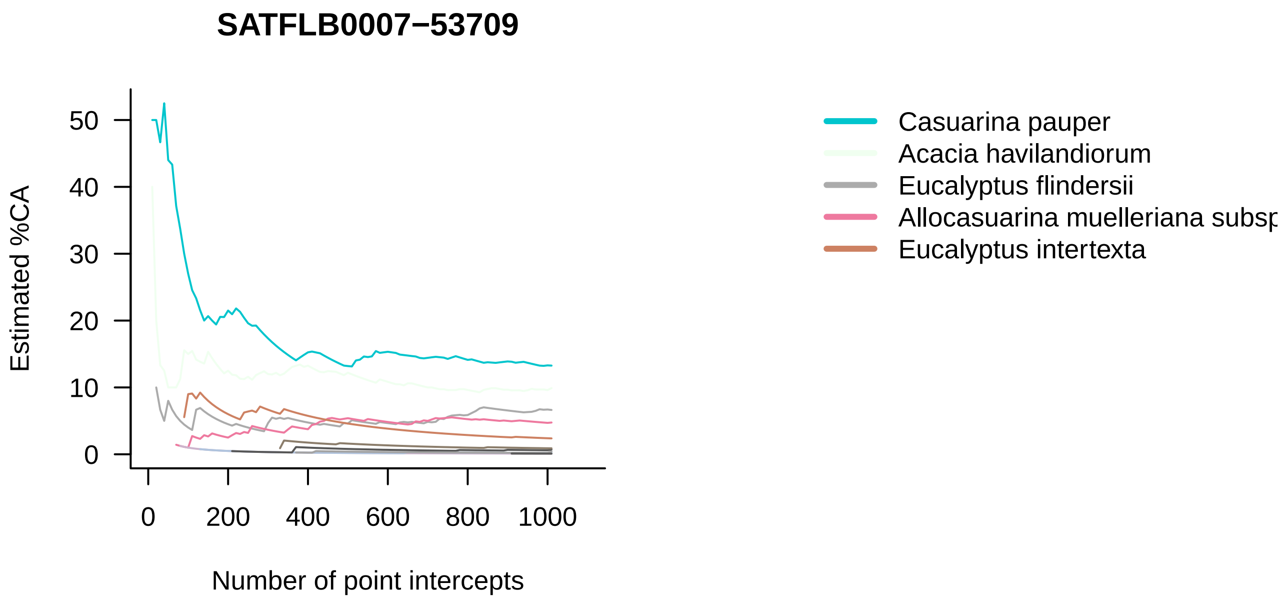
SATFLB0007−53709
Estimated %CA
Number of point intercepts
0
10
20
30
40
50
0
200
400
600
800
1000
Casuarina pauper
Acacia havilandiorum
Eucalyptus flindersii
Allocasuarina muelleriana subsp
Eucalyptus intertexta

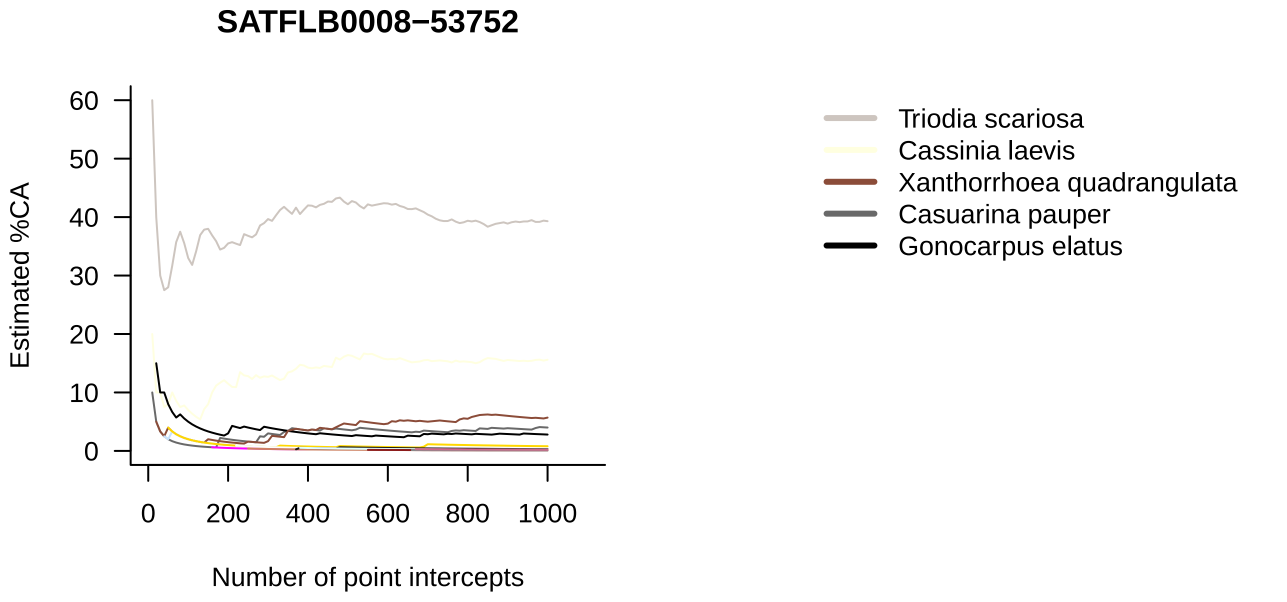
SATFLB0008−53752
Estimated %CA
Number of point intercepts
0
10
20
30
40
50
60
0
200
400
600
800
1000
Triodia scariosa
Cassinia laevis
Xanthorrhoea quadrangulata
Casuarina pauper
Gonocarpus elatus

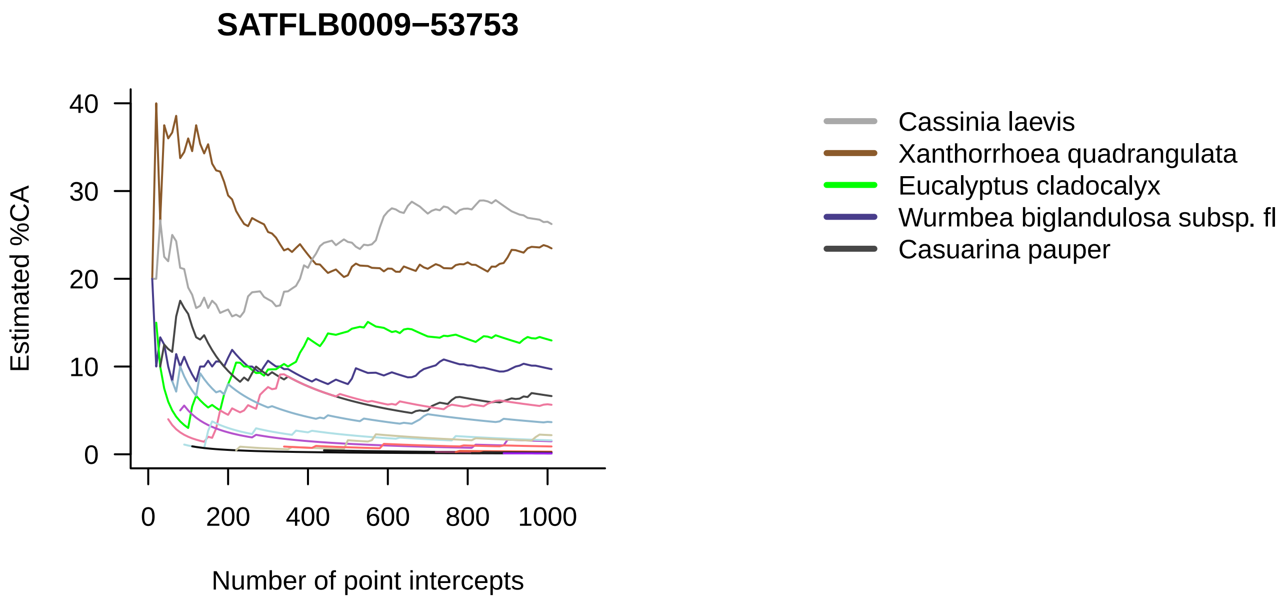
SATFLB0009−53753
Estimated %CA
40
30
20
10
0
0
200
400
600
800
1000
Number of point intercepts
Cassinia laevis
Xanthorrhoea quadrangulata
Eucalyptus cladocalyx
Wurmbea biglandulosa subsp. fl
Casuarina pauper

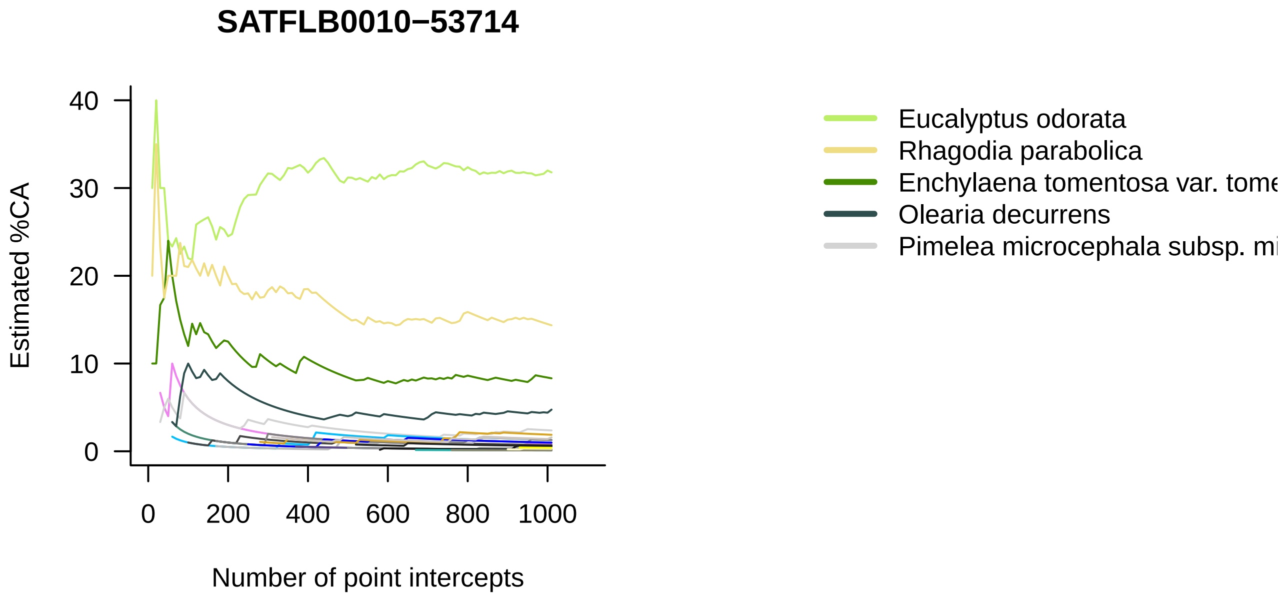
SATFLB0010−53714
Estimated %CA
40
30
20
10
0
0
200
400
600
800
1000
Number of point intercepts
Eucalyptus odorata
Rhagodia parabolica
Enchylaena tomentosa var. tome
Olearia decurrens
Pimelea microcephala subsp. mi

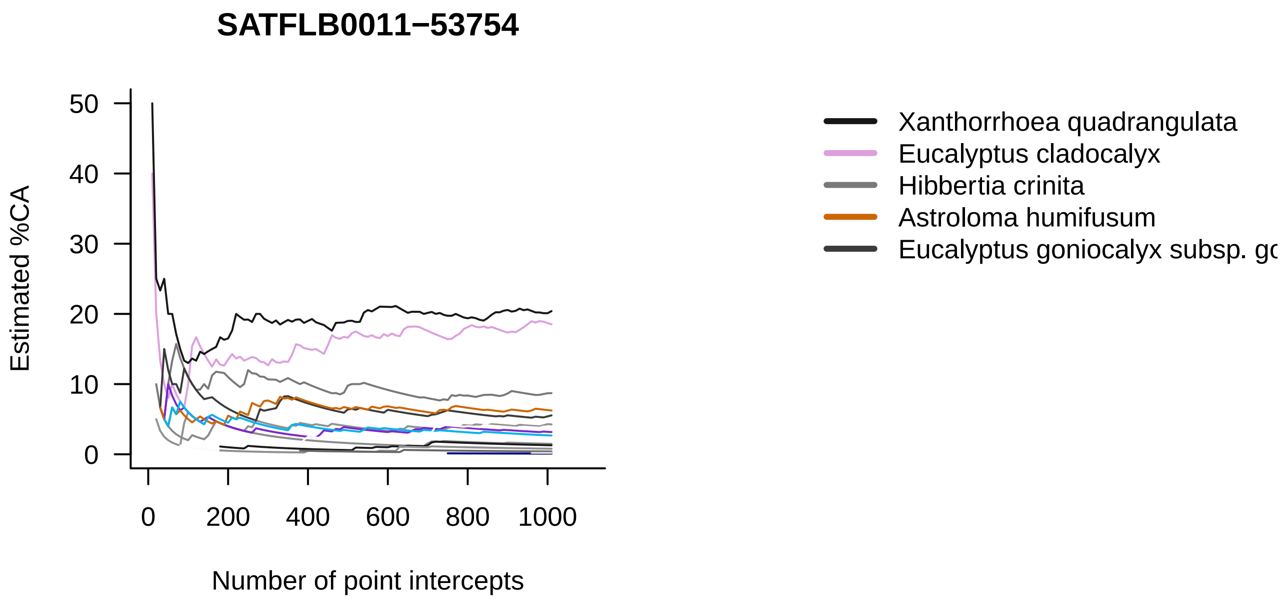
SATFLB0011−53754
Estimated %CA
Number of point intercepts
0
10
20
30
40
50
0
200
400
600
800
1000
Xanthorrhoea quadrangulata
Eucalyptus cladocalyx
Hibbertia crinita
Astroloma humifusum
Eucalyptus goniocalyx subsp. gc

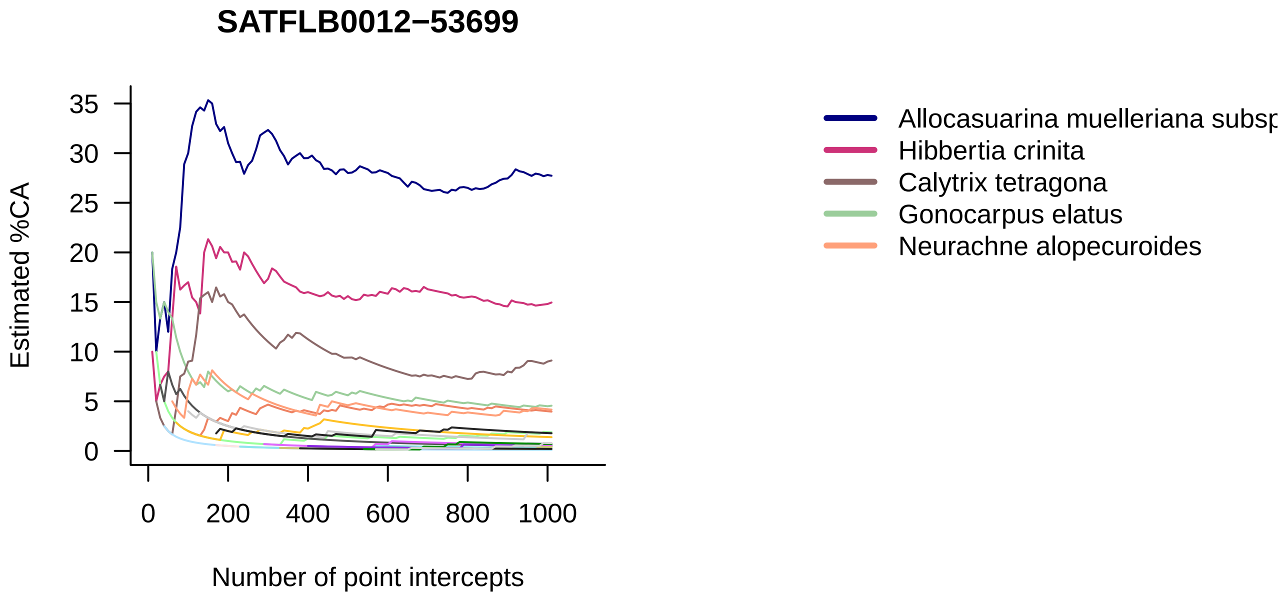
SATFLB0012−53699
Estimated %CA
Number of point intercepts
0
5
10
15
20
25
30
35
0
200
400
600
800
1000
Allocasuarina muelleriana subsp
Hibbertia crinita
Calytrix tetragona
Gonocarpus elatus
Neurachne alopecuroides

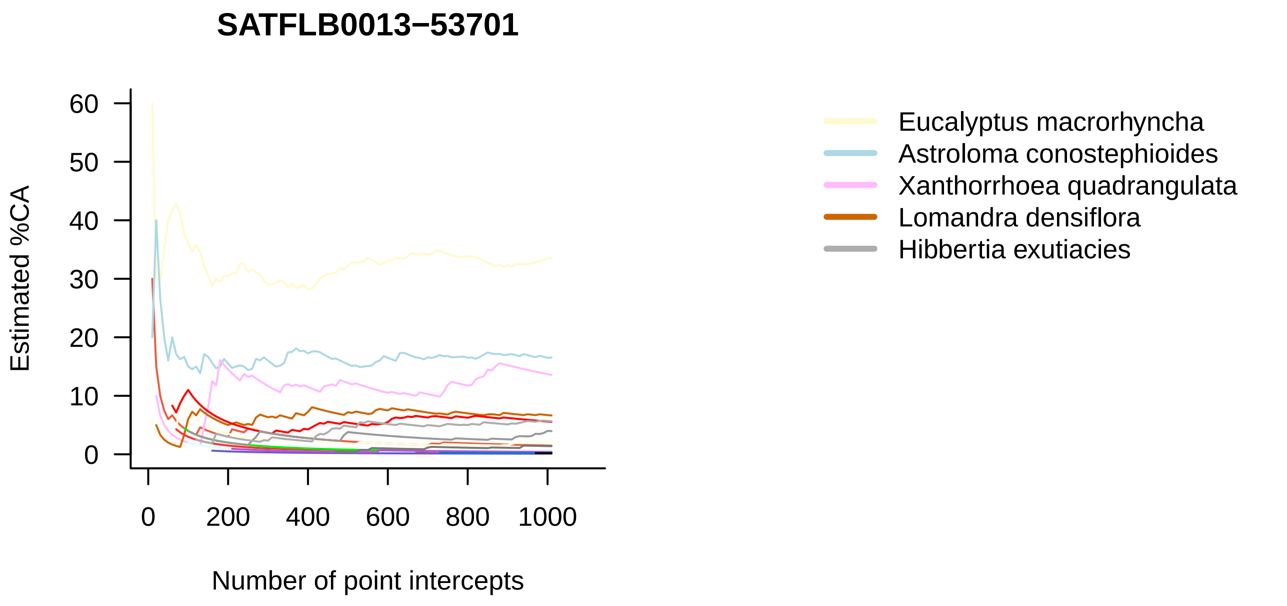
SATFLB0013−53701
Estimated %CA
Number of point intercepts
60
50
40
30
20
10
0
0
200
400
600
800
1000
Eucalyptus macrorhyncha
Astroloma conostephioides
Xanthorrhoea quadrangulata
Lomandra densiflora
Hibbertia exutiacies

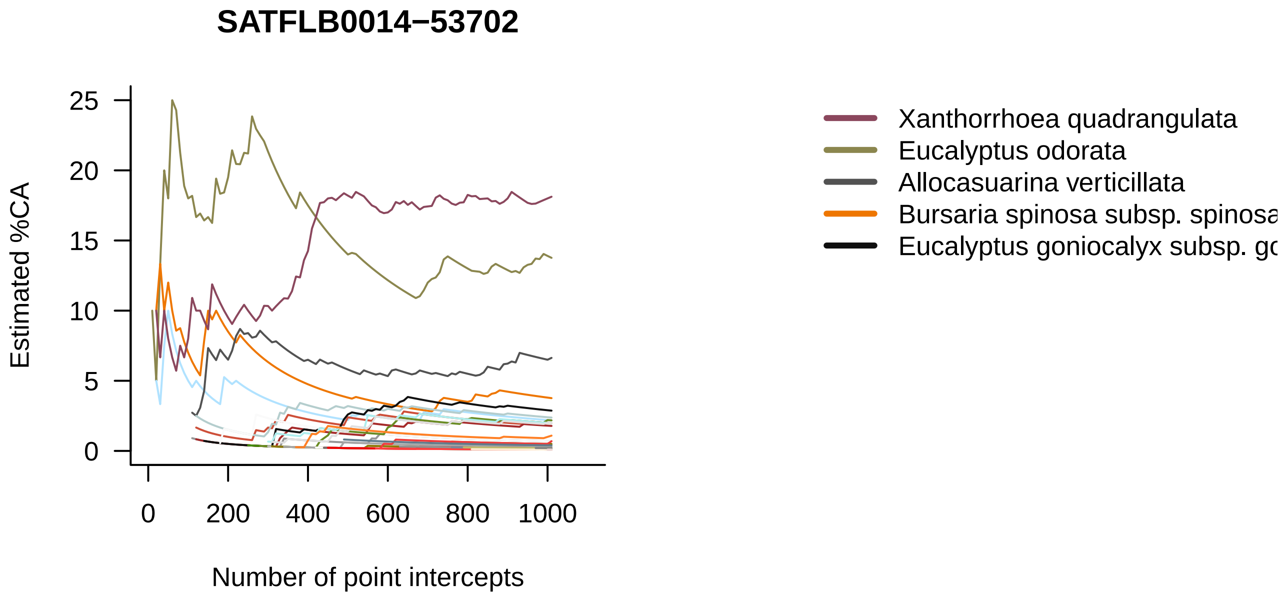
SATFLB0014−53702
Estimated %CA
Number of point intercepts
25
20
15
10
5
0
0
200
400
600
800
1000
Xanthorrhoea quadrangulata
Eucalyptus odorata
Allocasuarina verticillata
Bursaria spinosa subsp. spinosa
Eucalyptus goniocalyx subsp. gc

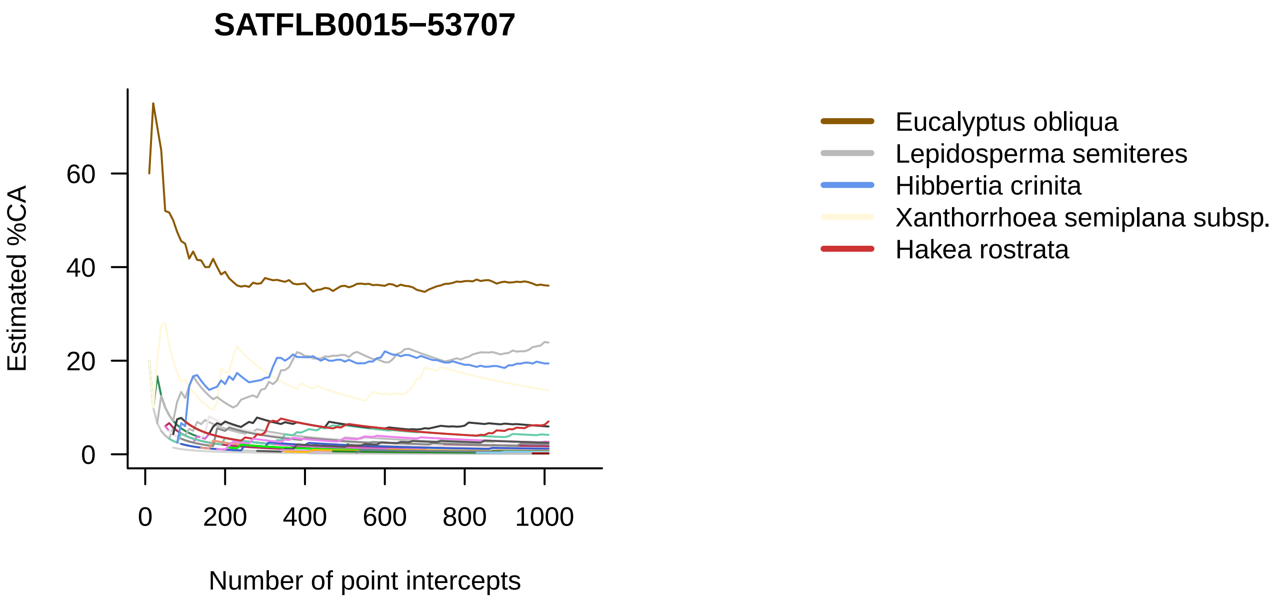
SATFLB0015−53707
Estimated %CA
Number of point intercepts
0
20
40
60
0
200
400
600
800
1000
Eucalyptus obliqua
Lepidosperma semiteres
Hibbertia crinita
Xanthorrhoea semiplana subsp.
Hakea rostrata

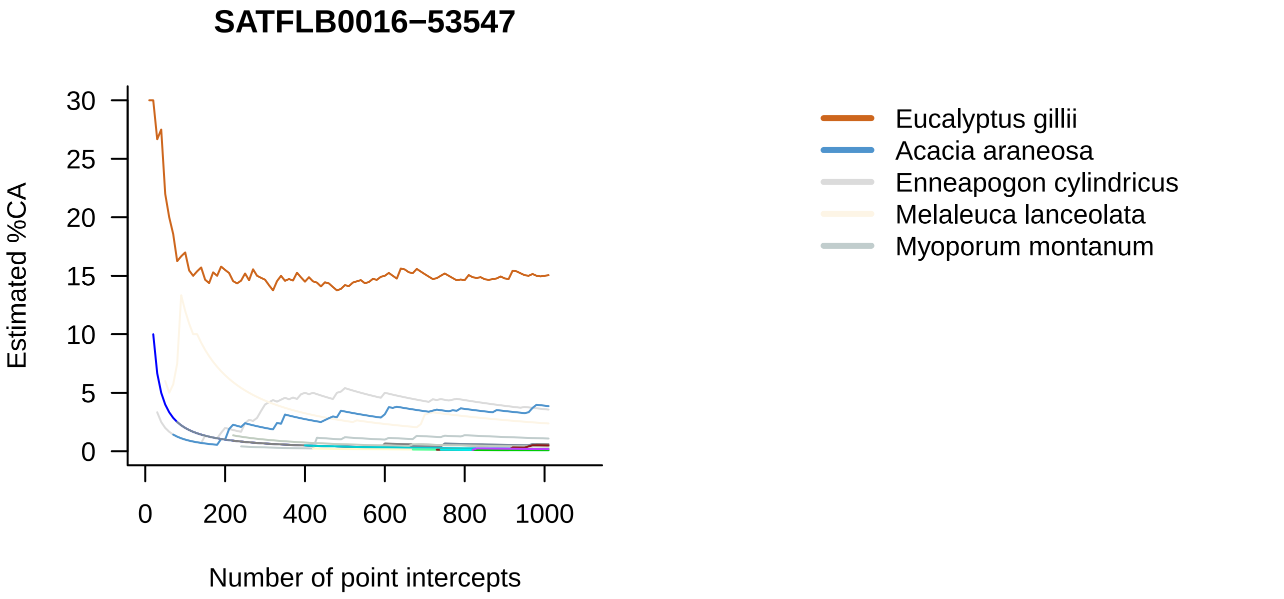
SATFLB0016−53547
Estimated %CA
Number of point intercepts
0
5
10
15
20
25
30
0
200
400
600
800
1000
Eucalyptus gillii
Acacia araneosa
Enneapogon cylindricus
Melaleuca lanceolata
Myoporum montanum

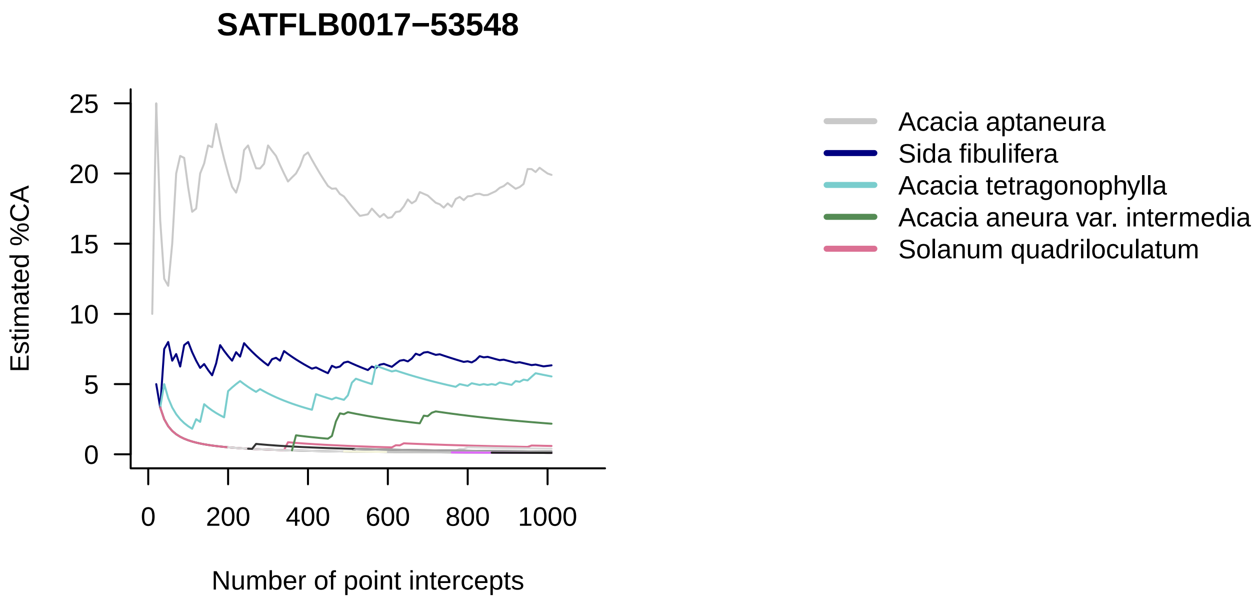
SATFLB0017−53548
Estimated %CA
Number of point intercepts
0
5
10
15
20
25
0
200
400
600
800
1000
Acacia aptaneura
Sida fibulifera
Acacia tetragonophylla
Acacia aneura var. intermedia
Solanum quadriloculatum

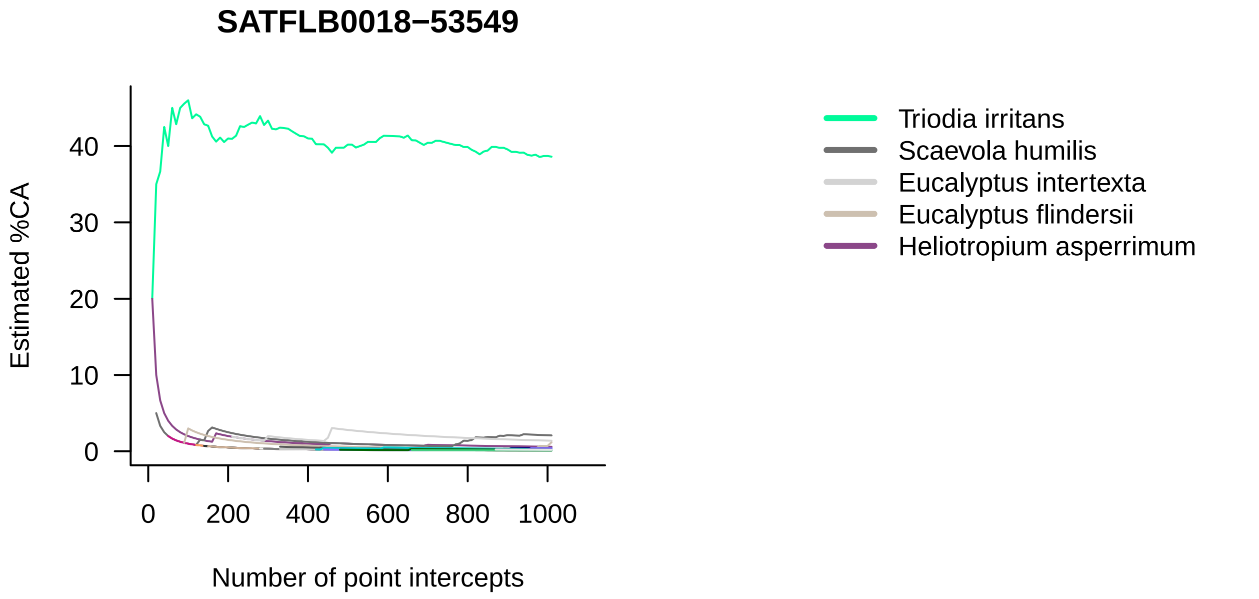
SATFLB0018−53549
Estimated %CA
Number of point intercepts
0
10
20
30
40
0
200
400
600
800
1000
Triodia irritans
Scaevola humilis
Eucalyptus intertexta
Eucalyptus flindersii
Heliotropium asperrimum

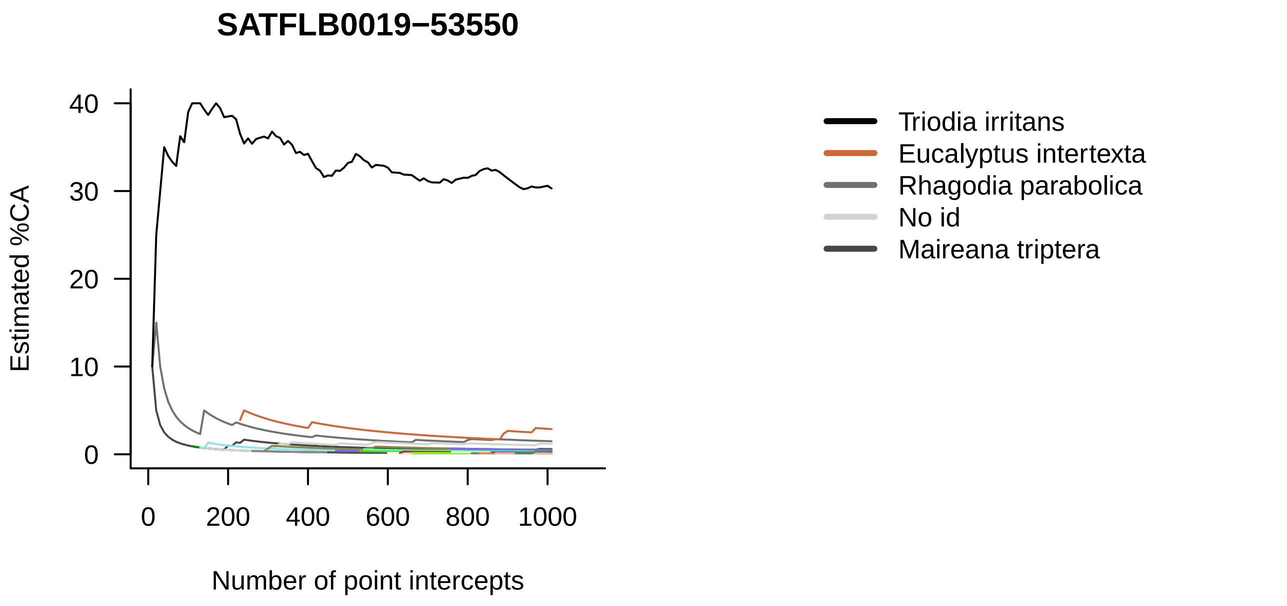
SATFLB0019−53550
Estimated %CA
0
10
20
30
40
0
200
400
600
800
1000
Number of point intercepts
Triodia irritans
Eucalyptus intertexta
Rhagodia parabolica
No id
Maireana triptera

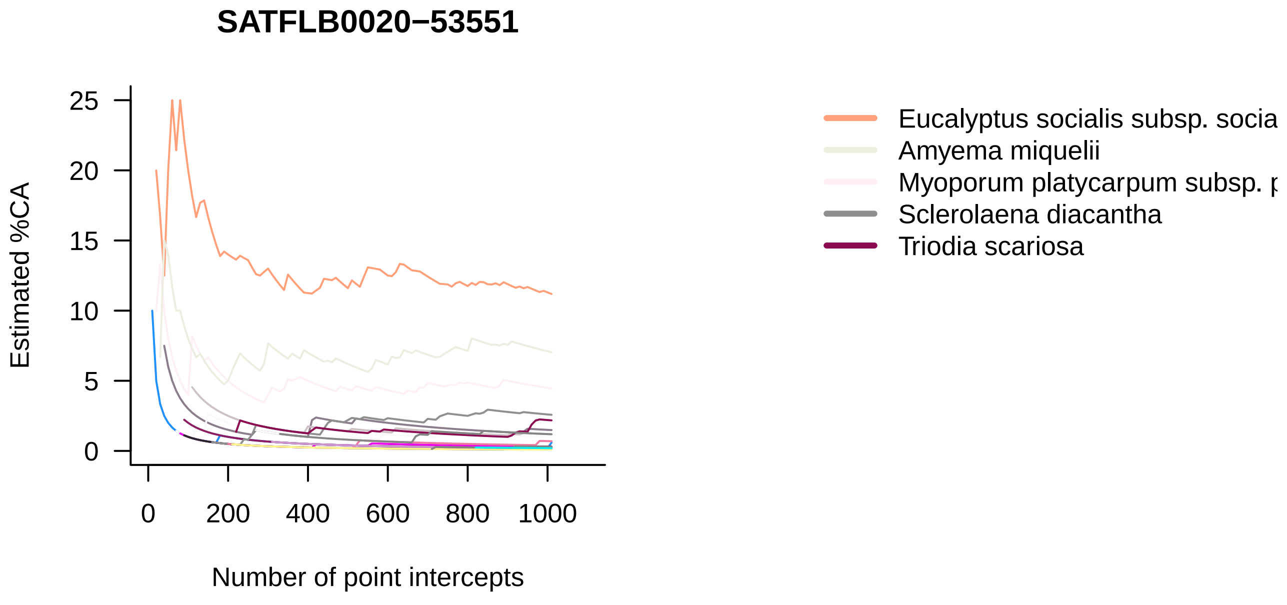
SATFLB0020−53551
Estimated %CA
0
5
10
15
20
25
0
200
400
600
800
1000
Number of point intercepts
Eucalyptus socialis subsp. socia
Amyema miquelii
Myoporum platycarpum subsp. p
Sclerolaena diacantha
Triodia scariosa

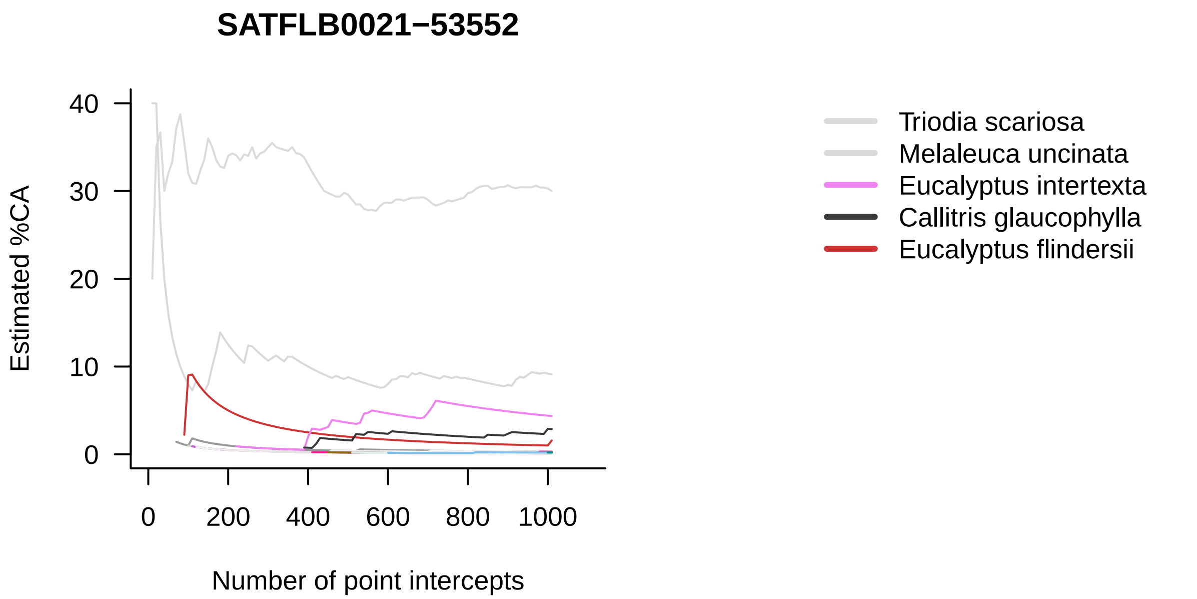
SATFLB0021−53552
Estimated %CA
Number of point intercepts
0
10
20
30
40
0
200
400
600
800
1000
Triodia scariosa
Melaleuca uncinata
Eucalyptus intertexta
Callitris glaucophylla
Eucalyptus flindersii

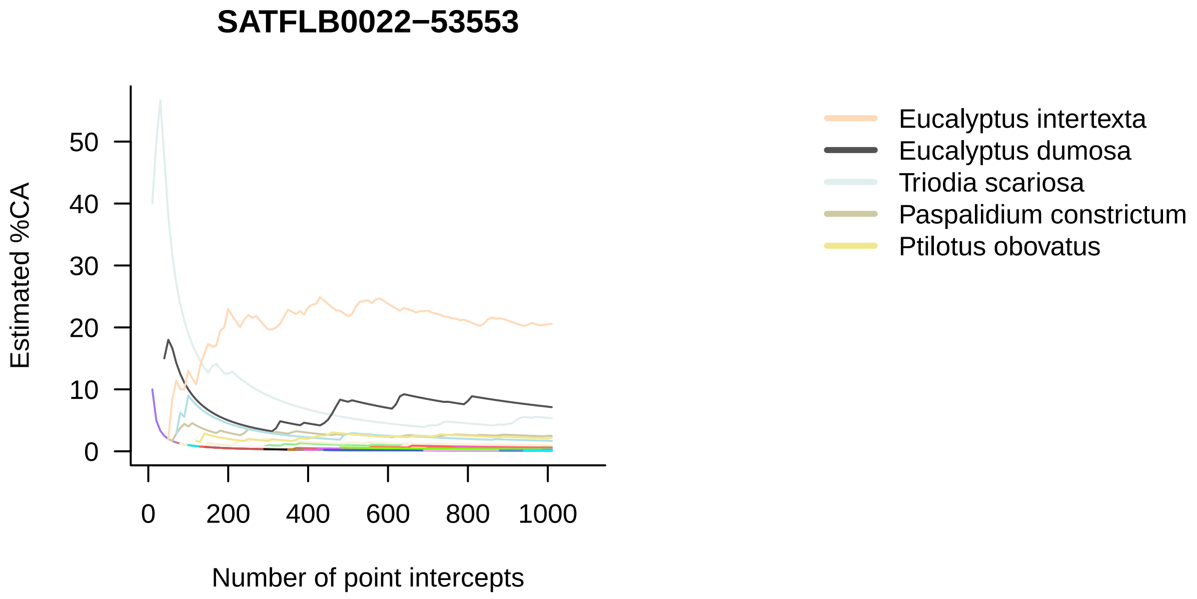
SATFLB0022−53553
Estimated %CA
Number of point intercepts
0
10
20
30
40
50
0
200
400
600
800
1000
Eucalyptus intertexta
Eucalyptus dumosa
Triodia scariosa
Paspalidium constrictum
Ptilotus obovatus

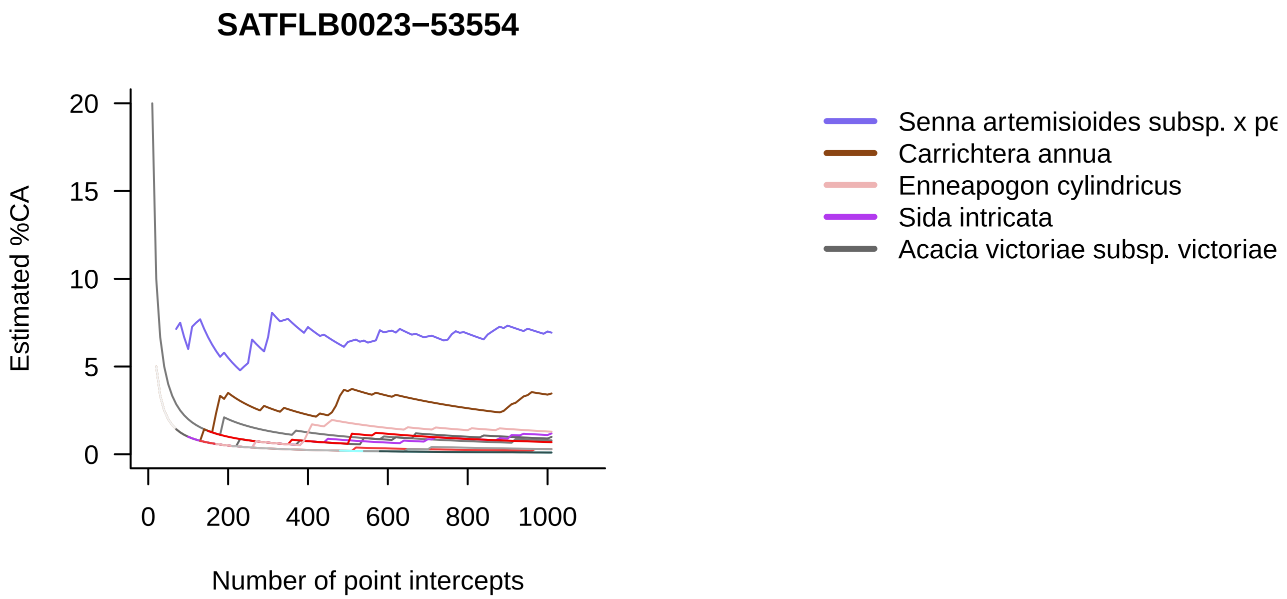
SATFLB0023−53554
Estimated %CA
Number of point intercepts
0
5
10
15
20
0
200
400
600
800
1000
Senna artemisioides subsp. x pe
Carrichtera annua
Enneapogon cylindricus
Sida intricata
Acacia victoriae subsp. victoriae

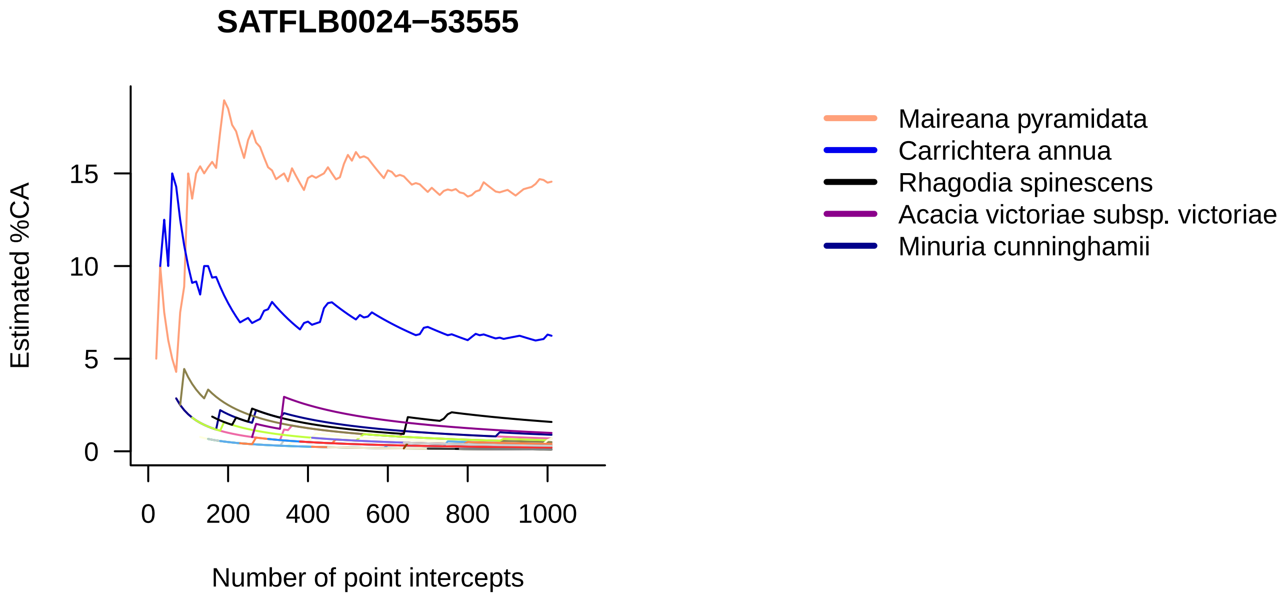
SATFLB0024−53555
Estimated %CA
Number of point intercepts
0
5
10
15
0
200
400
600
800
1000
Maireana pyramidata
Carrichtera annua
Rhagodia spinescens
Acacia victoriae subsp. victoriae
Minuria cunninghamii

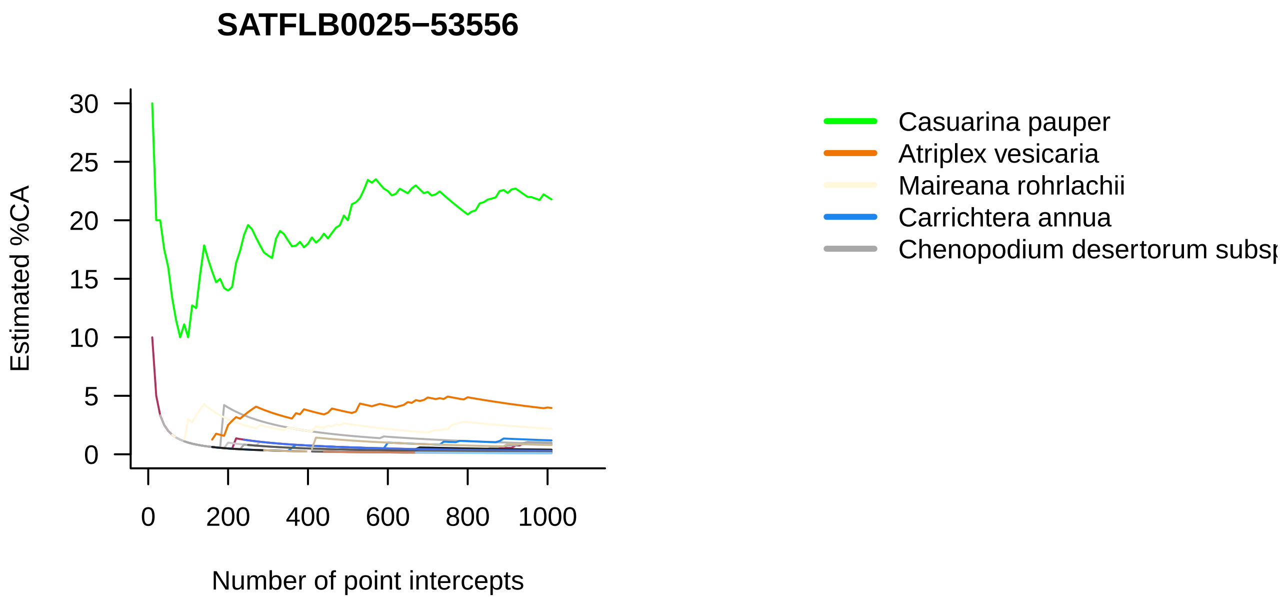
SATFLB0025−53556
Estimated %CA
30
25
20
15
10
5
0
0
200
400
600
800
1000
Number of point intercepts
Casuarina pauper
Atriplex vesicaria
Maireana rohrlachii
Carrichtera annua
Chenopodium desertorum subsp

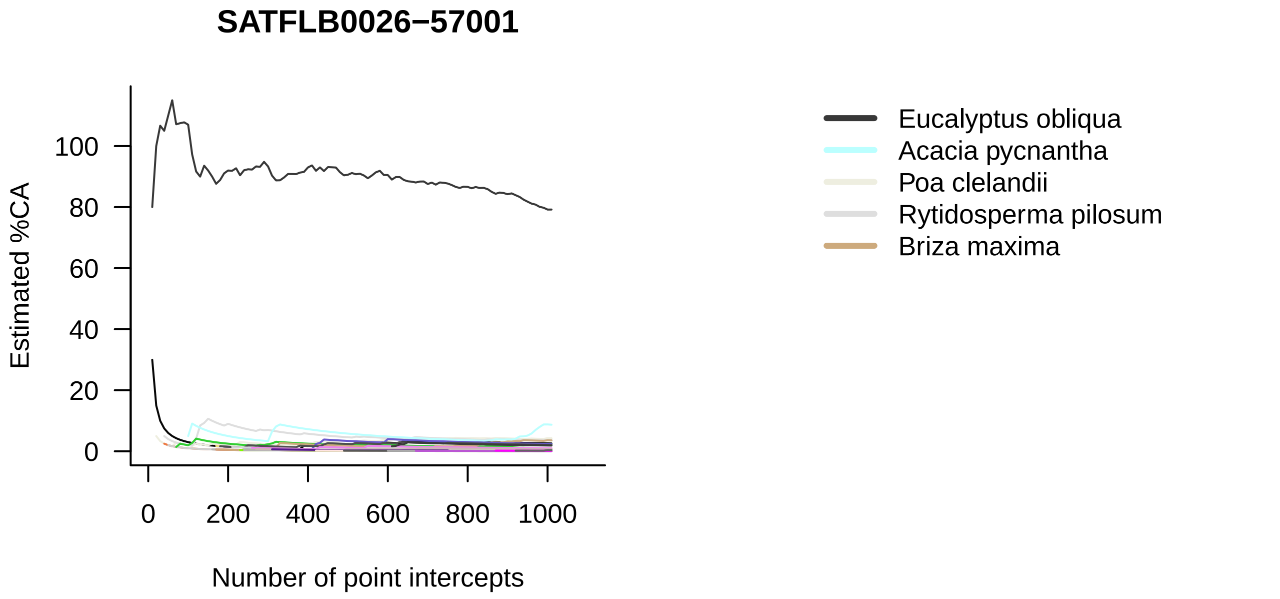
SATFLB0026−57001
Estimated %CA
100
80
60
40
20
0
0
200
400
600
800
1000
Number of point intercepts
Eucalyptus obliqua
Acacia pycnantha
Poa clelandii
Rytidosperma pilosum
Briza maxima

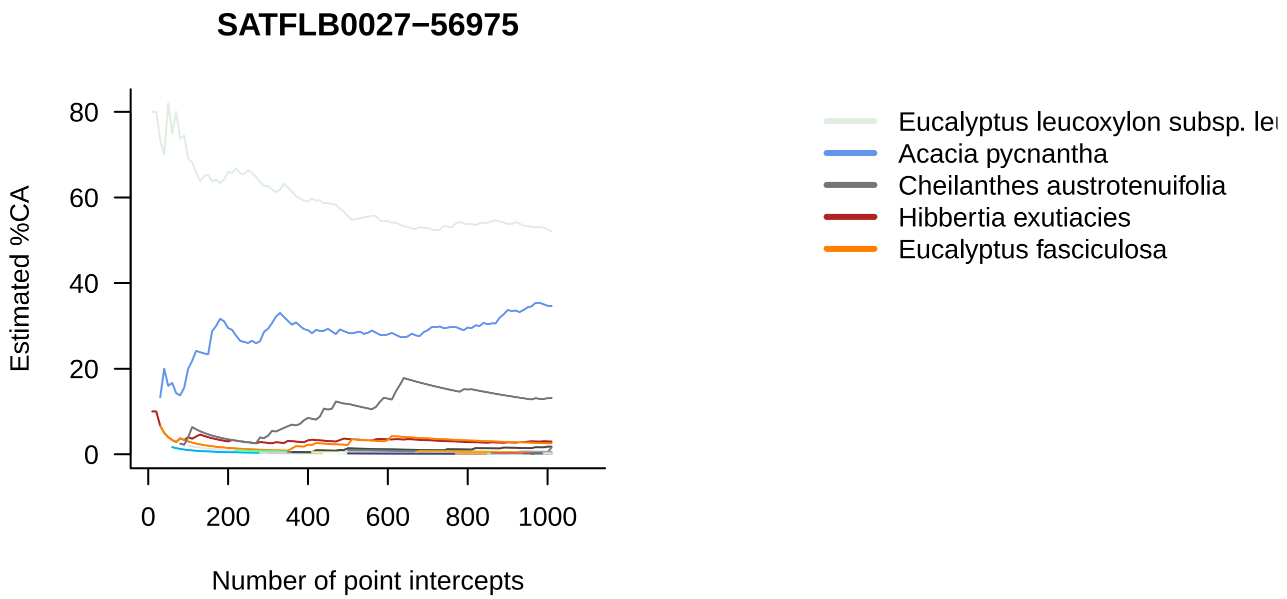
SATFLB0027−56975
Estimated %CA
80
60
40
20
0
0
200
400
600
800
1000
Number of point intercepts
Eucalyptus leucoxylon subsp. leu
Acacia pycnantha
Cheilanthes austrotenuifolia
Hibbertia exutiacies
Eucalyptus fasciculosa

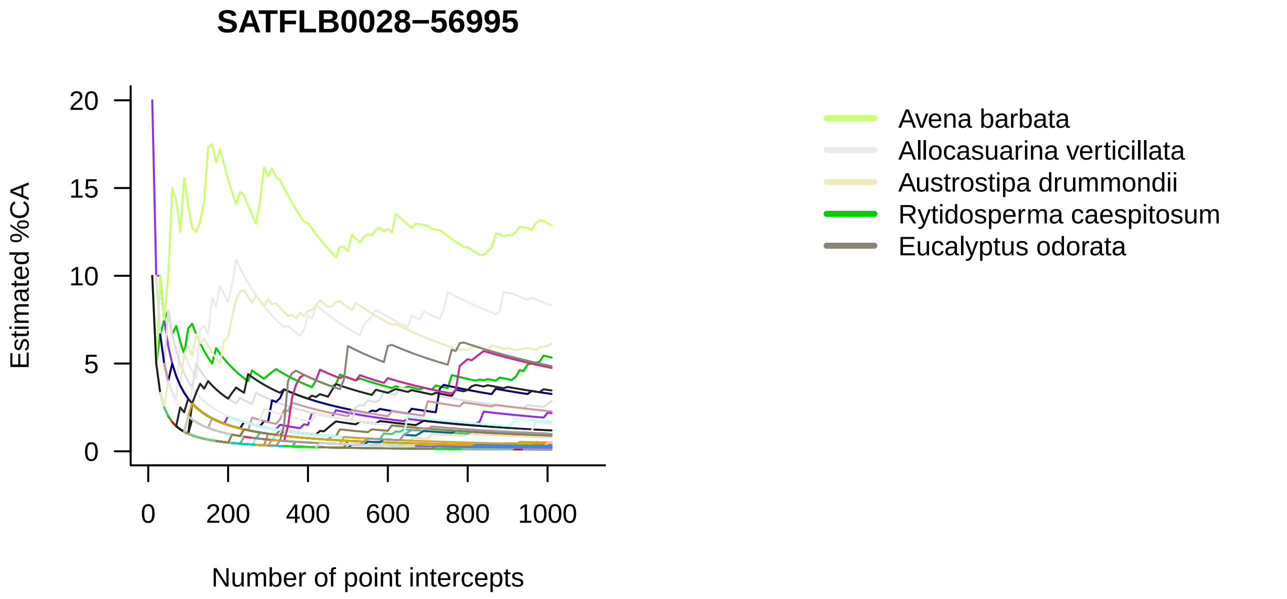
SATFLB0028−56995
Estimated %CA
20
15
10
5
0
0
200
400
600
800
1000
Number of point intercepts
Avena barbata
Allocasuarina verticillata
Austrostipa drummondii
Rytidosperma caespitosum
Eucalyptus odorata

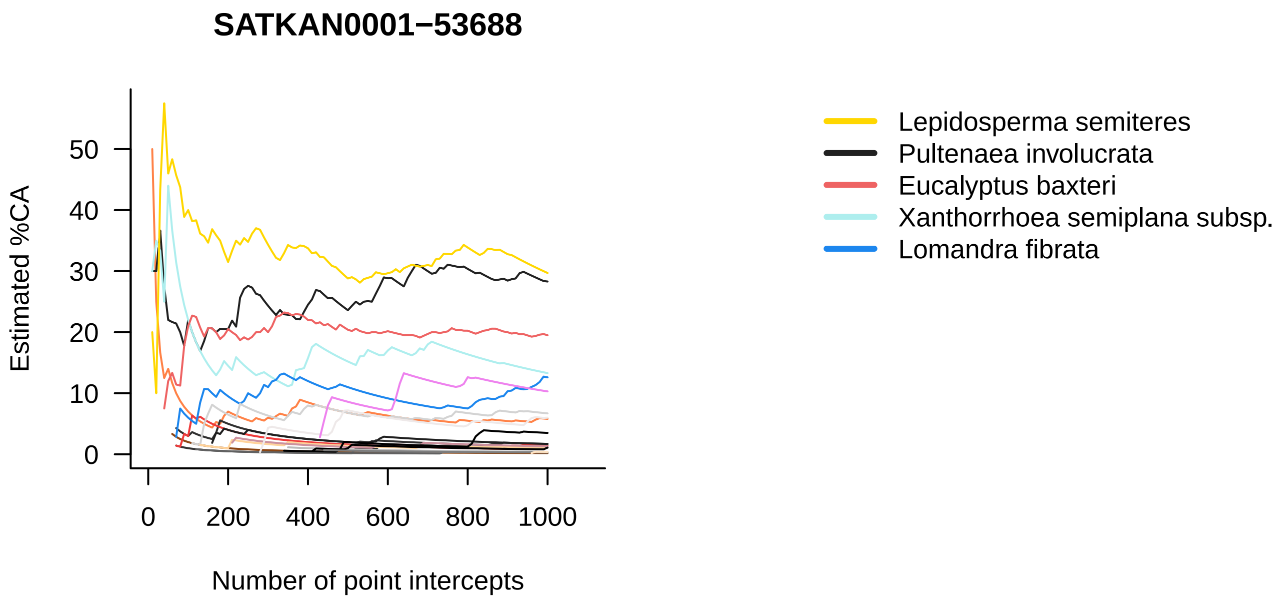
SATKAN0001−53688
Estimated %CA
0
10
20
30
40
50
0
200
400
600
800
1000
Number of point intercepts
Lepidosperma semiteres
Pultenaea involucrata
Eucalyptus baxteri
Xanthorrhoea semiplana subsp.
Lomandra fibrata

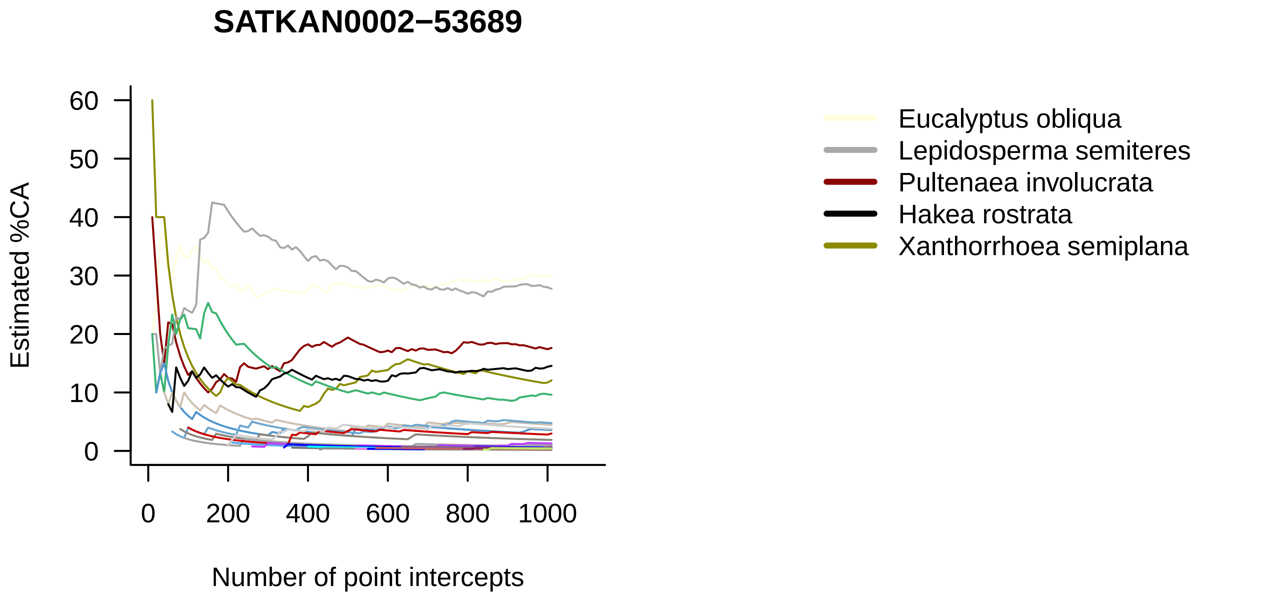
SATKAN0002−53689
Estimated %CA
0
10
20
30
40
50
60
0
200
400
600
800
1000
Number of point intercepts
Eucalyptus obliqua
Lepidosperma semiteres
Pultenaea involucrata
Hakea rostrata
Xanthorrhoea semiplana

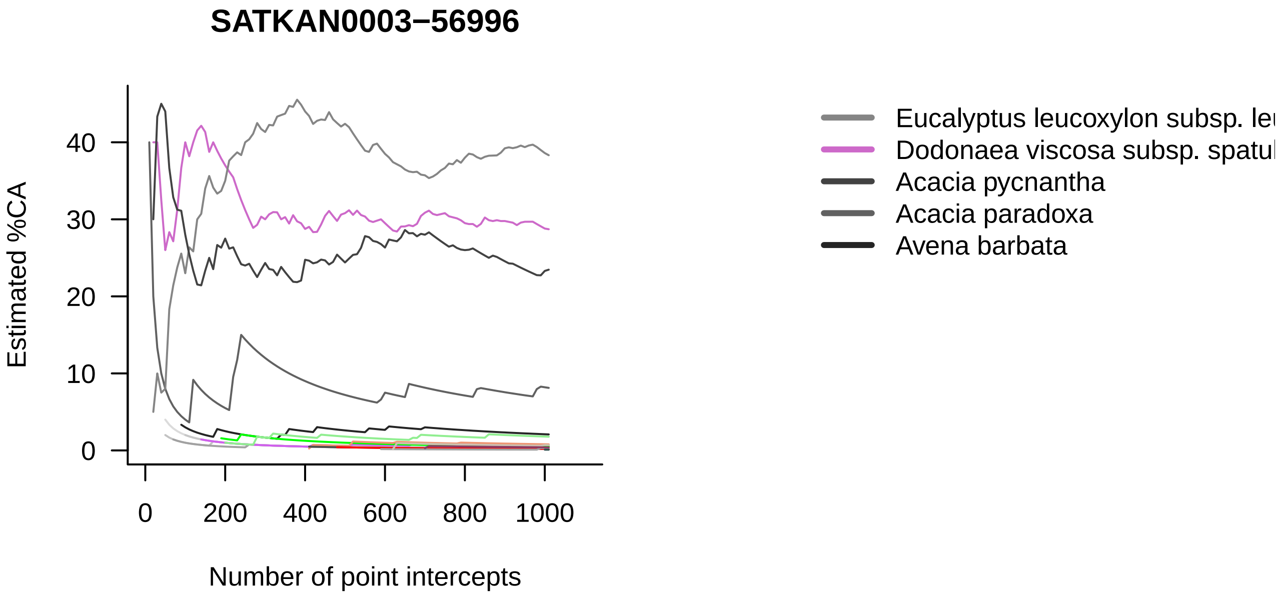
SATKAN0003−56996
Estimated %CA
0
10
20
30
40
0
200
400
600
800
1000
Number of point intercepts
Eucalyptus leucoxylon subsp. leu
Dodonaea viscosa subsp. spatul
Acacia pycnantha
Acacia paradoxa
Avena barbata

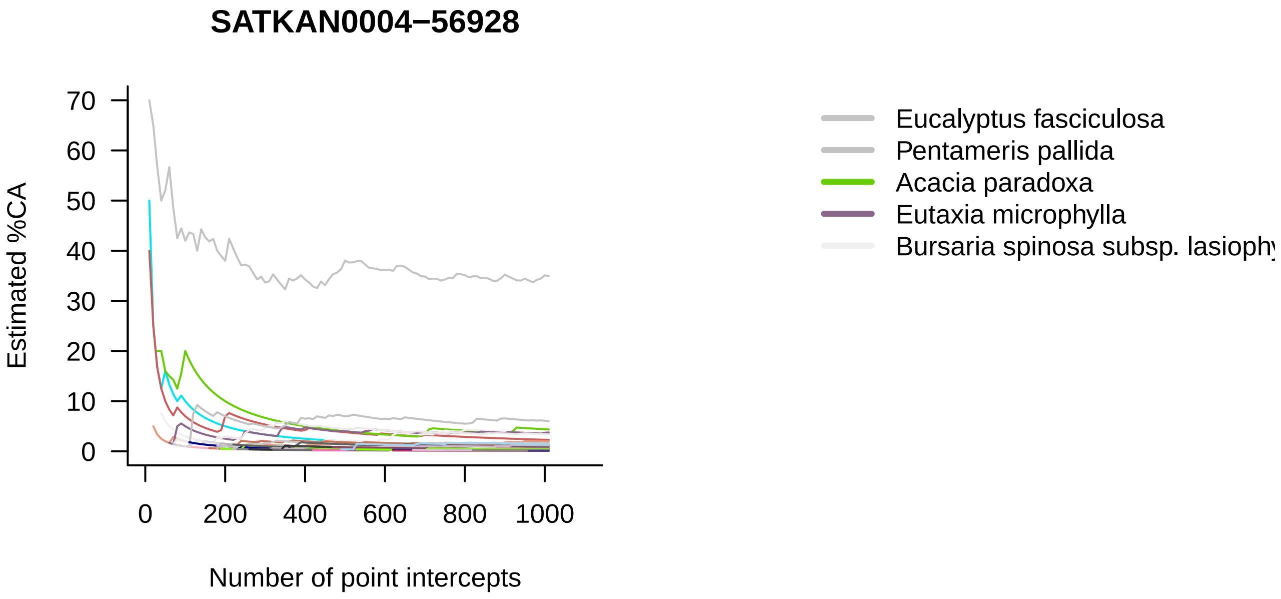
SATKAN0004−56928
Estimated %CA
0
10
20
30
40
50
60
70
0
200
400
600
800
1000
Number of point intercepts
Eucalyptus fasciculosa
Pentameris pallida
Acacia paradoxa
Eutaxia microphylla
Bursaria spinosa subsp. lasiophy

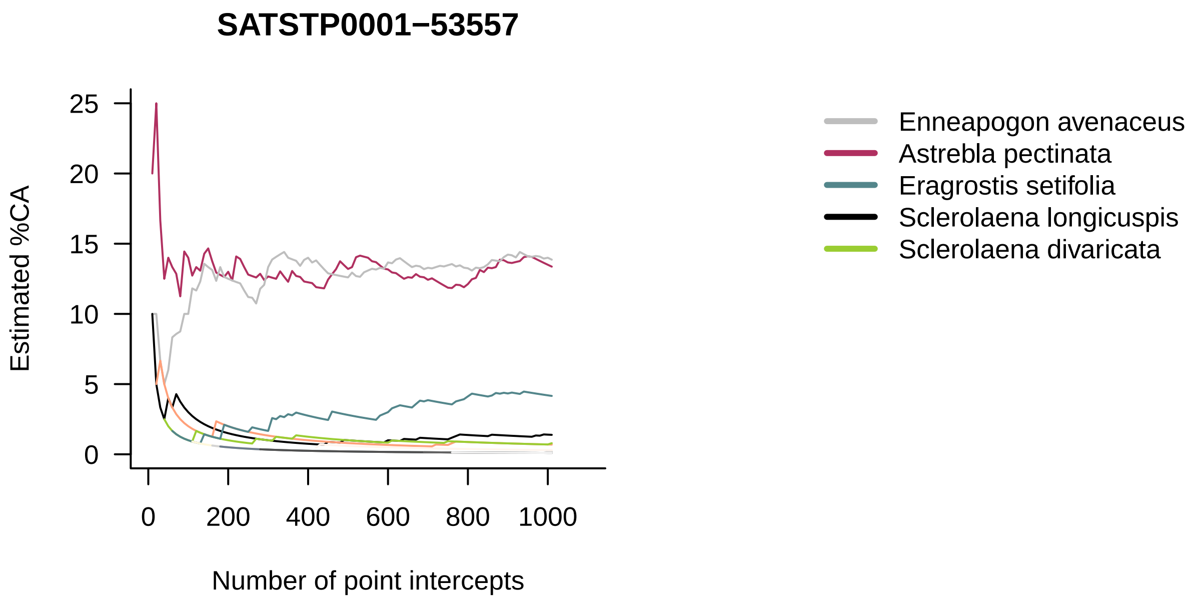
SATSTP0001−53557
Estimated %CA
Number of point intercepts
0
5
10
15
20
25
0
200
400
600
800
1000
Enneapogon avenaceus
Astrebla pectinata
Eragrostis setifolia
Sclerolaena longicuspis
Sclerolaena divaricata

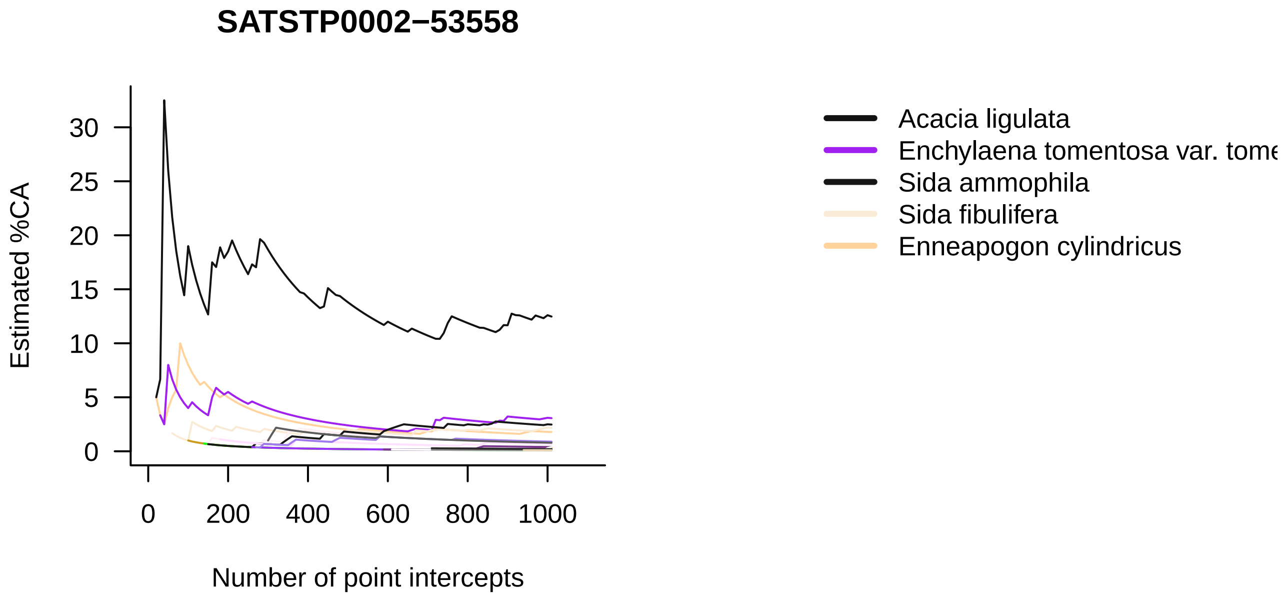
SATSTP0002−53558
Estimated %CA
Number of point intercepts
0
5
10
15
20
25
30
0
200
400
600
800
1000
Acacia ligulata
Enchylaena tomentosa var. tome
Sida ammophila
Sida fibulifera
Enneapogon cylindricus

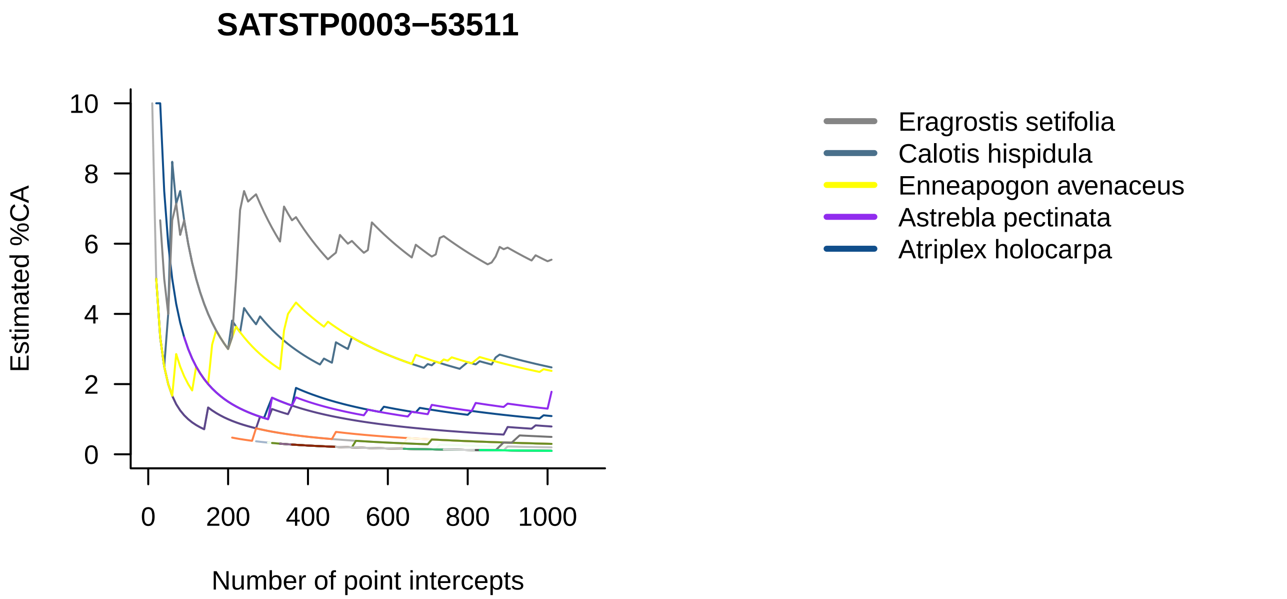

SATSTP0003−53511
Estimated %CA
Number of point intercepts
0
2
4
6
8
10
0
200
400
600
800
1000
Eragrostis setifolia
Calotis hispidula
Enneapogon avenaceus
Astrebla pectinata
Atriplex holocarpa


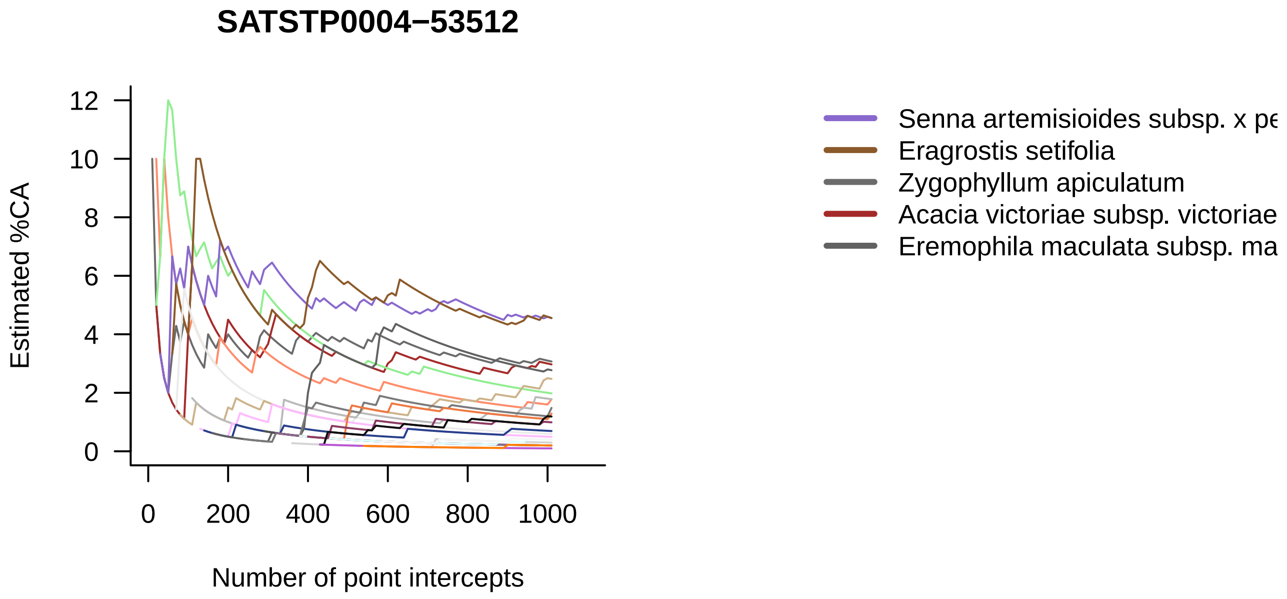

SATSTP0004−53512
Estimated %CA
Number of point intercepts
0
2
4
6
8
10
12
0
200
400
600
800
1000
Senna artemisioides subsp. x pe
Eragrostis setifolia
Zygophyllum apiculatum
Acacia victoriae subsp. victoriae
Eremophila maculata subsp. ma

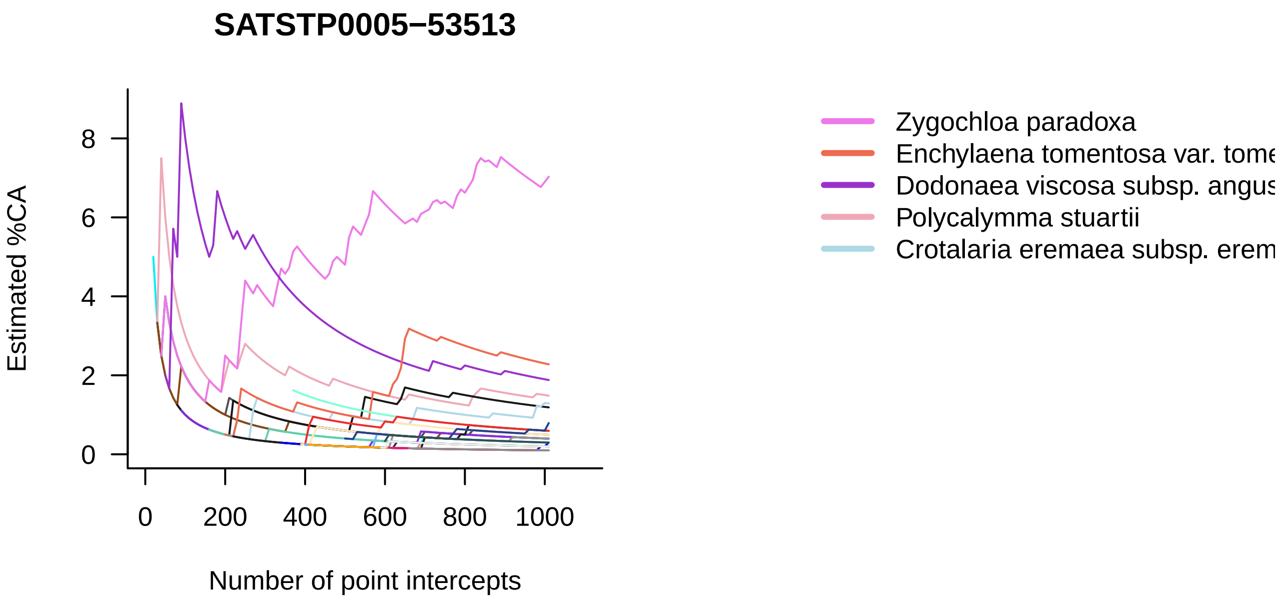
SATSTP0005−53513
Estimated %CA
0
2
4
6
8
0
200
400
600
800
1000
Number of point intercepts
Zygochloa paradoxa
Enchylaena tomentosa var. tome
Dodonaea viscosa subsp. angus
Polycalymma stuartii
Crotalaria eremaea subsp. erem

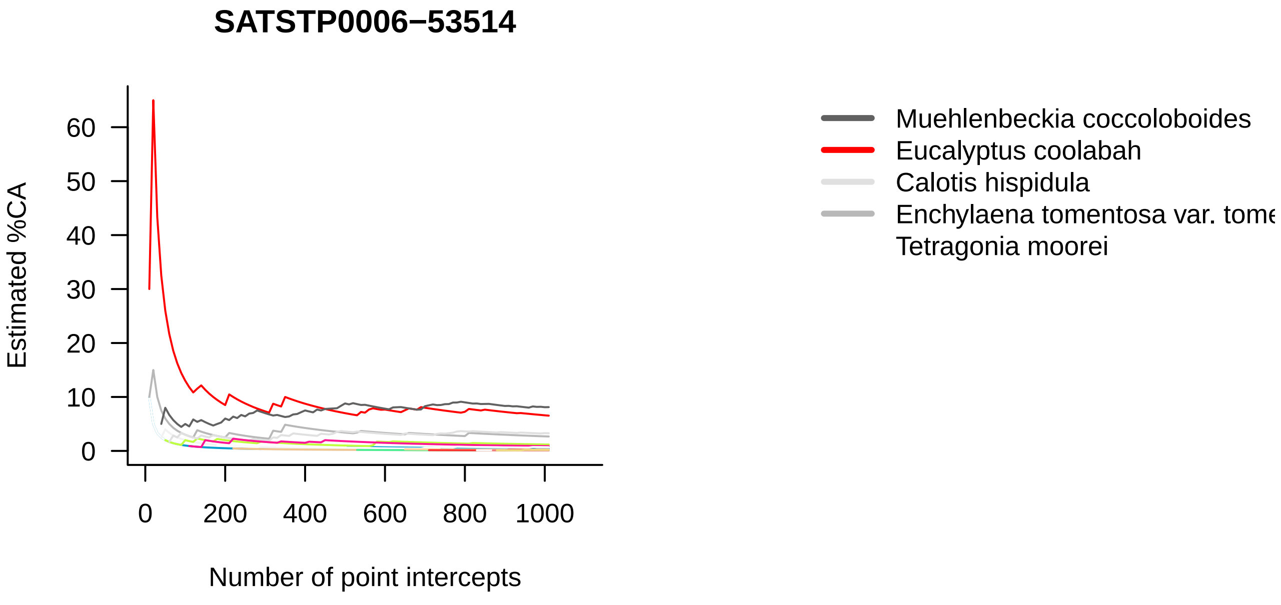
SATSTP0006−53514
Estimated %CA
0
10
20
30
40
50
60
0
200
400
600
800
1000
Number of point intercepts
Muehlenbeckia coccoloboides
Eucalyptus coolabah
Calotis hispidula
Enchylaena tomentosa var. tome
Tetragonia moorei

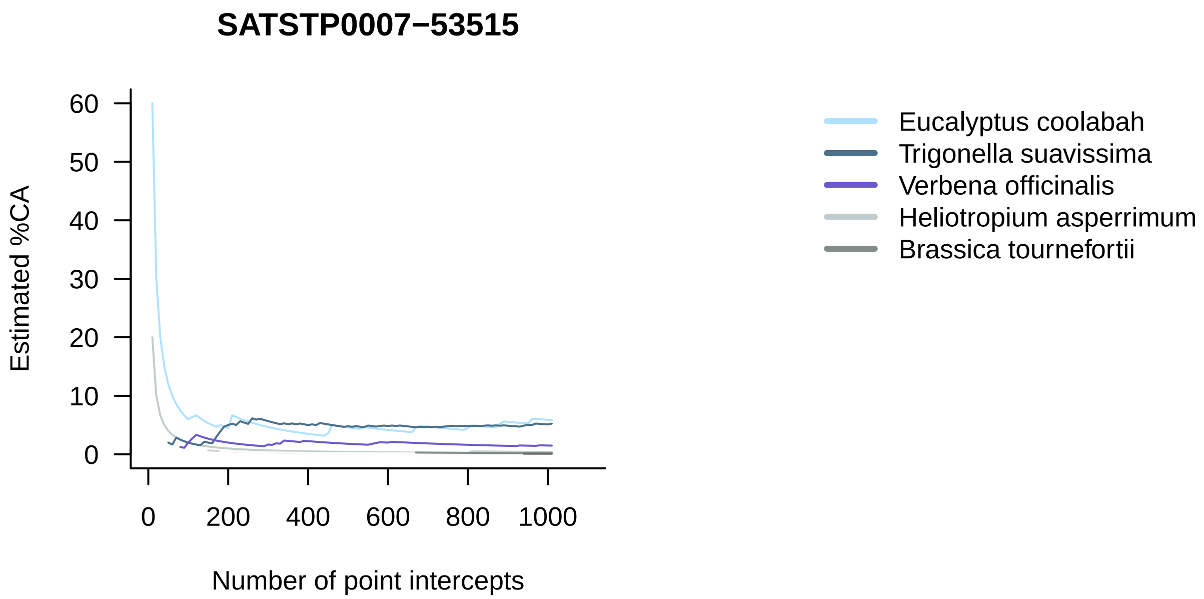
SATSTP0007−53515
Estimated %CA
0
10
20
30
40
50
60
0
200
400
600
800
1000
Number of point intercepts
Eucalyptus coolabah
Trigonella suavissima
Verbena officinalis
Heliotropium asperrimum
Brassica tournefortii

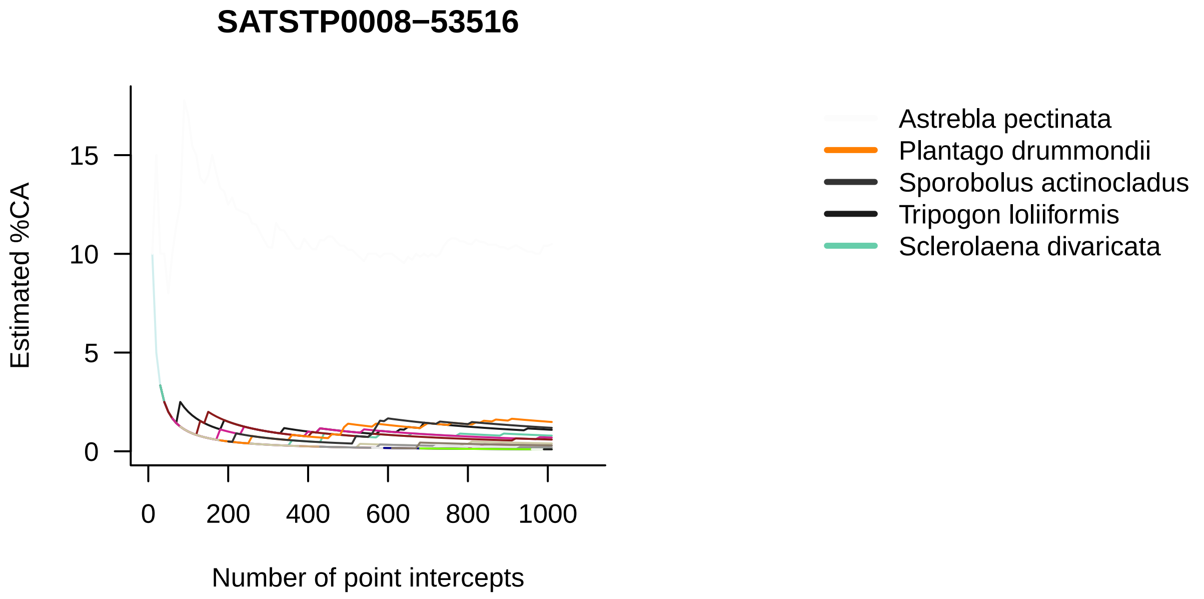
SATSTP0008−53516
Estimated %CA
0
5
10
15
0
200
400
600
800
1000
Number of point intercepts
Astrebla pectinata
Plantago drummondii
Sporobolus actinocladus
Tripogon loliiformis
Sclerolaena divaricata

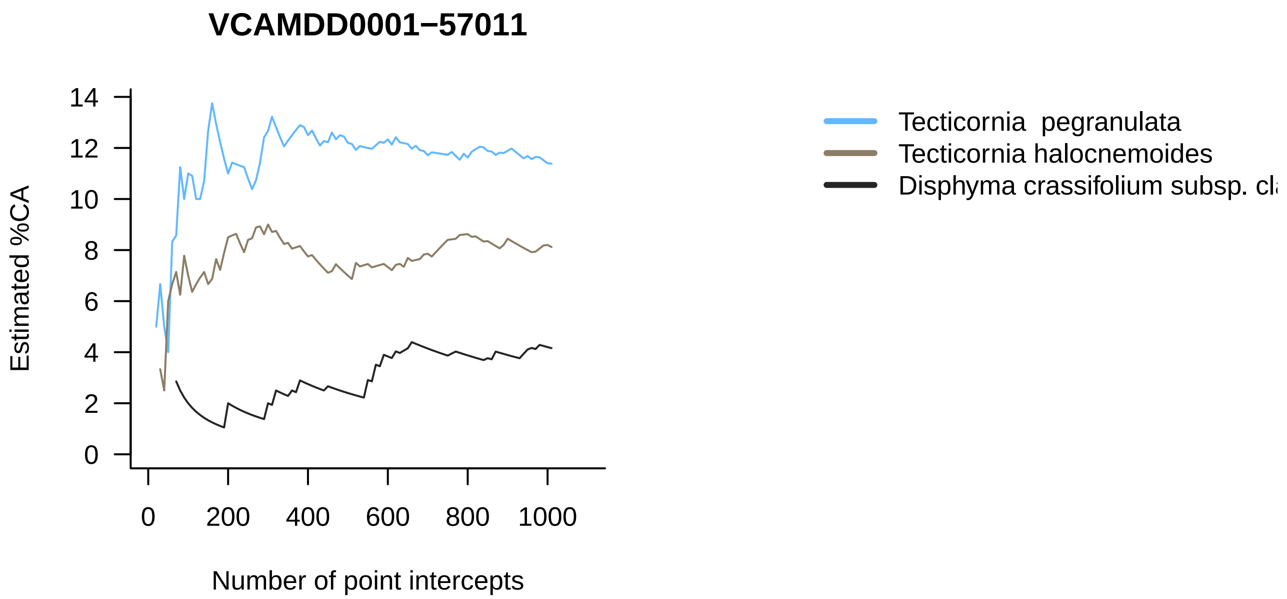
VCAMDD0001−57011
Estimated %CA
0
2
4
6
8
10
12
14
0
200
400
600
800
1000
Number of point intercepts
Tecticornia pegranulata
Tecticornia halocnemoides
Disphyma crassifolium subsp. cl

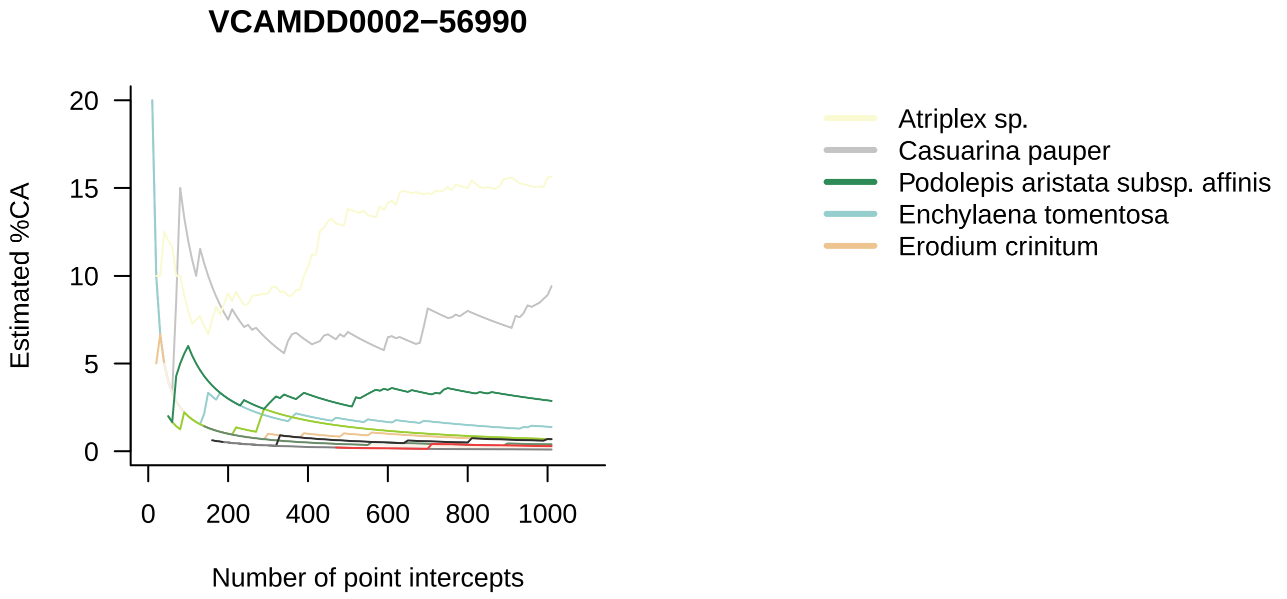
VCAMDD0002−56990
Estimated %CA
0
5
10
15
20
0
200
400
600
800
1000
Number of point intercepts
Atriplex sp.
Casuarina pauper
Podolepis aristata subsp. affinis
Enchylaena tomentosa
Erodium crinitum

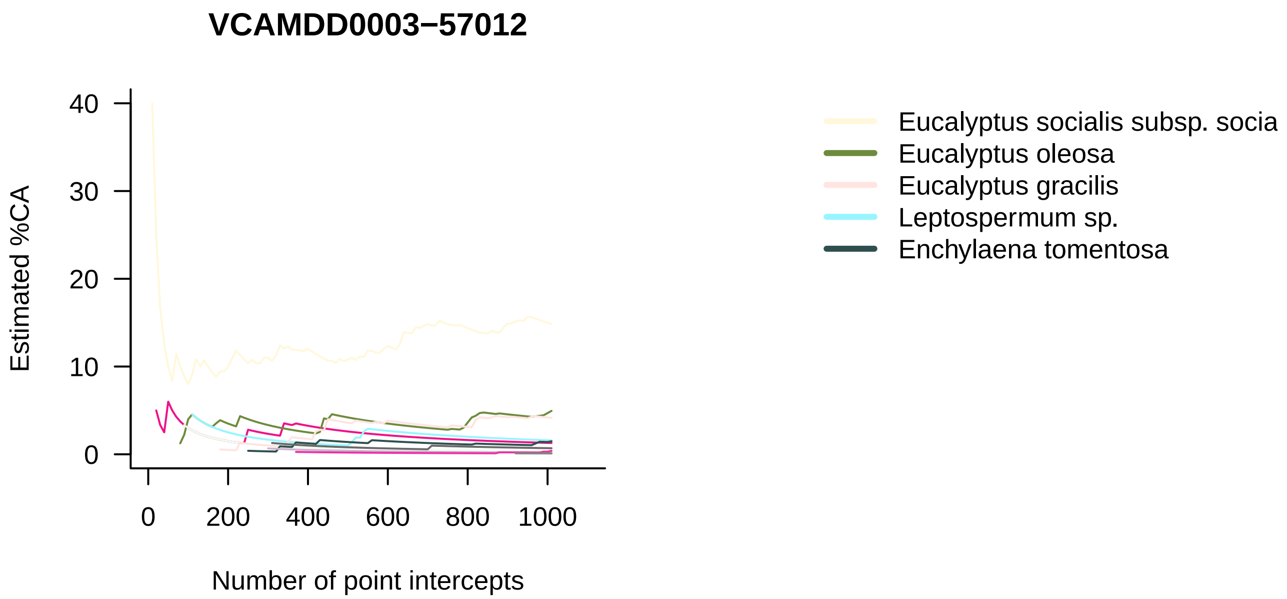
VCAMDD0003−57012
Estimated %CA
40
30
20
10
0
0
200
400
600
800
1000
Number of point intercepts
Eucalyptus socialis subsp. socia
Eucalyptus oleosa
Eucalyptus gracilis
Leptospermum sp.
Enchylaena tomentosa

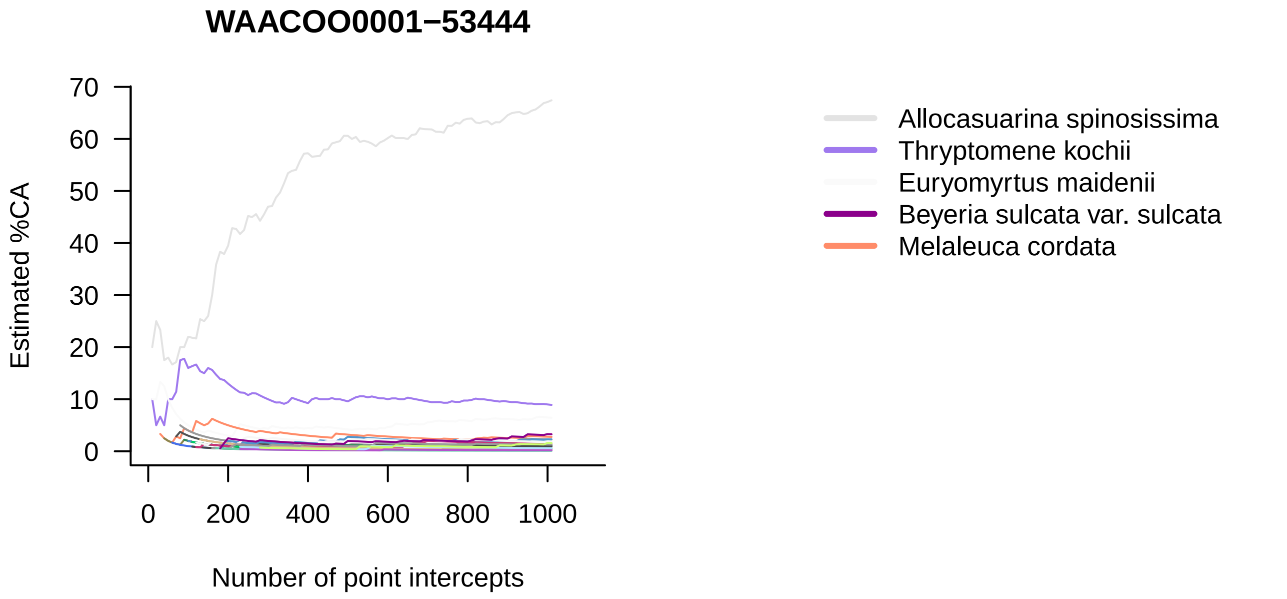
WAACOO0001−53444
Estimated %CA
70
60
50
40
30
20
10
0
0
200
400
600
800
1000
Number of point intercepts
Allocasuarina spinosissima
Thryptomene kochii
Euryomyrtus maidenii
Beyeria sulcata var. sulcata
Melaleuca cordata

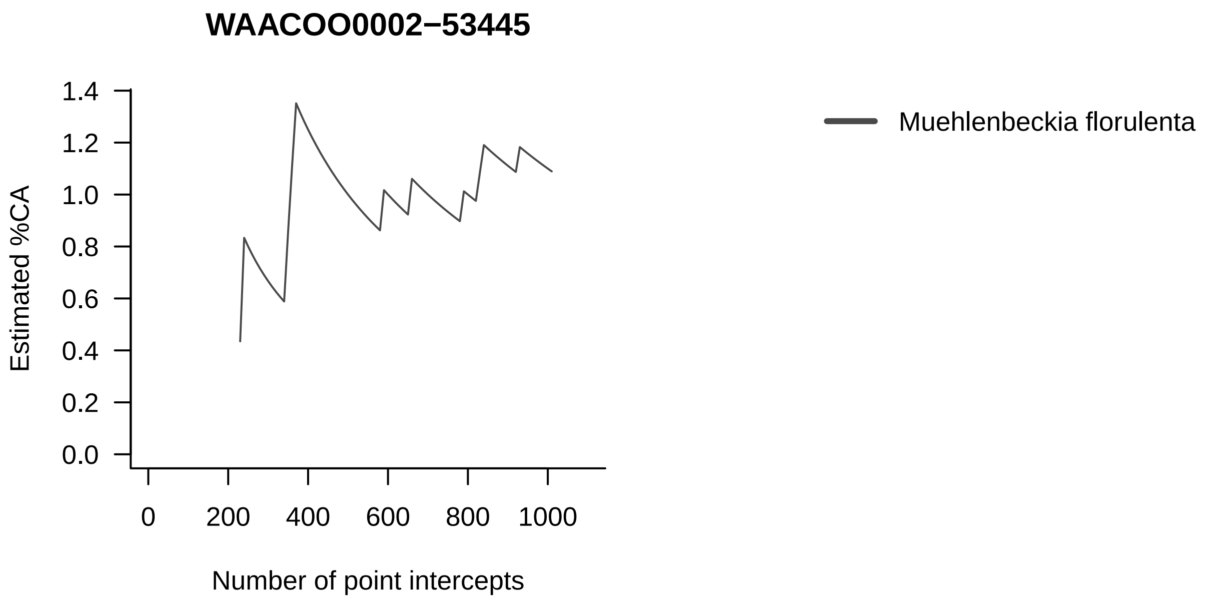
WAACOO0002−53445
Estimated %CA
1.4
1.2
1.0
0.8
0.6
0.4
0.2
0.0
0
200
400
600
800
1000
Number of point intercepts
Muehlenbeckia florulenta

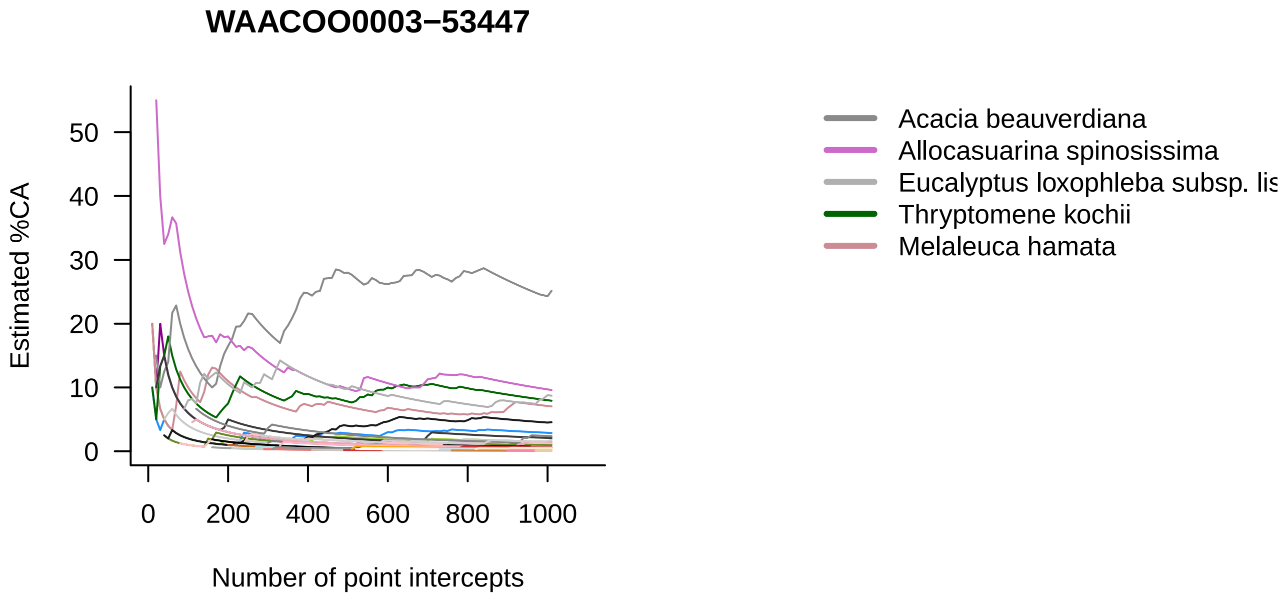
WAACOO0003−53447
Estimated %CA
50
40
30
20
10
0
0
200
400
600
800
1000
Number of point intercepts
Acacia beauverdiana
Allocasuarina spinosissima
Eucalyptus loxophleba subsp. lis
Thryptomene kochii
Melaleuca hamata

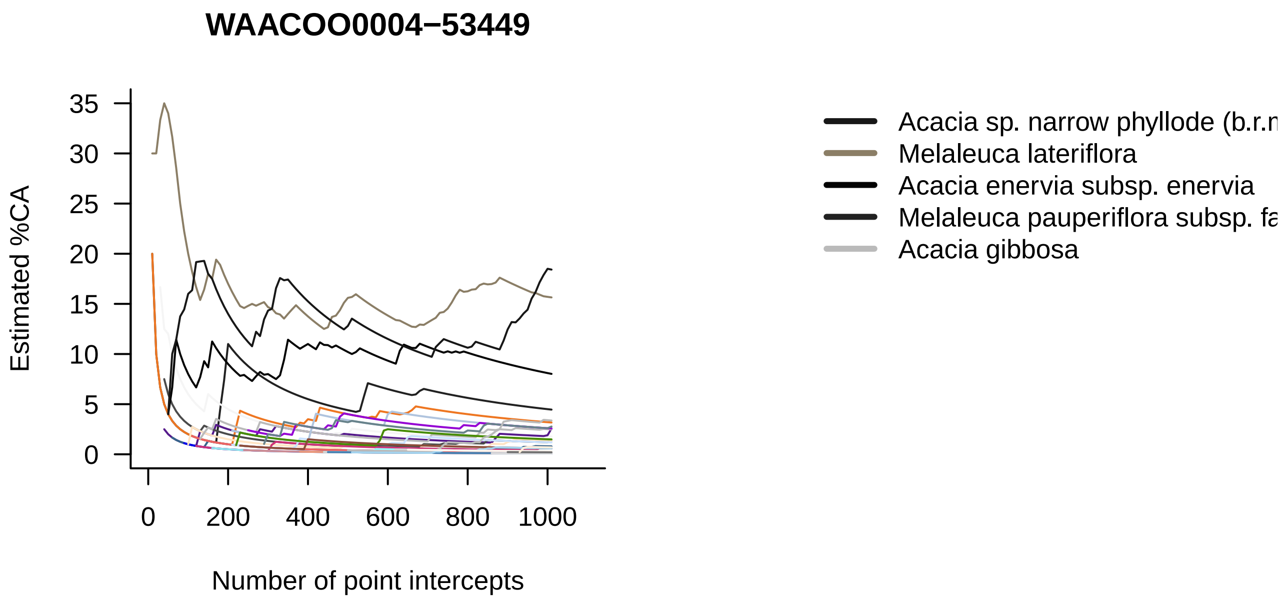
WAACOO0004−53449
Estimated %CA
35
30
25
20
15
10
5
0
0
200
400
600
800
1000
Number of point intercepts
Acacia sp. narrow phyllode (b.r.n
Melaleuca lateriflora
Acacia enervia subsp. enervia
Melaleuca pauperiflora subsp. fa
Acacia gibbosa

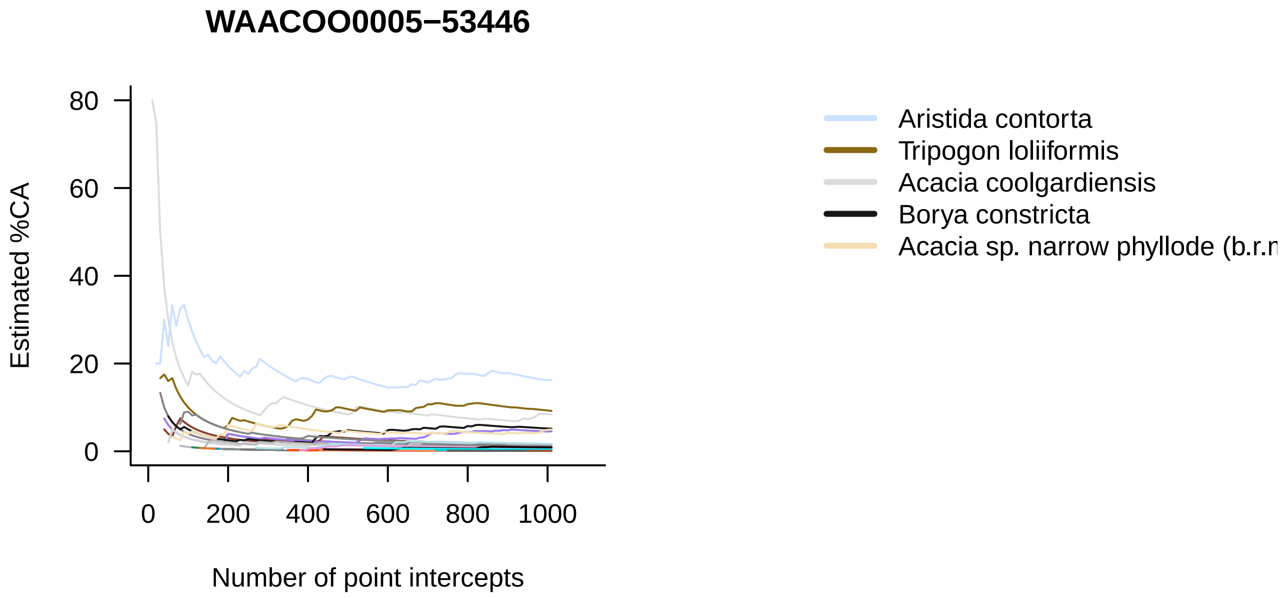
WAACOO0005−53446
Estimated %CA
80
60
40
20
0
0
200
400
600
800
1000
Number of point intercepts
Aristida contorta
Tripogon loliiformis
Acacia coolgardiensis
Borya constricta
Acacia sp. narrow phyllode (b.r.n

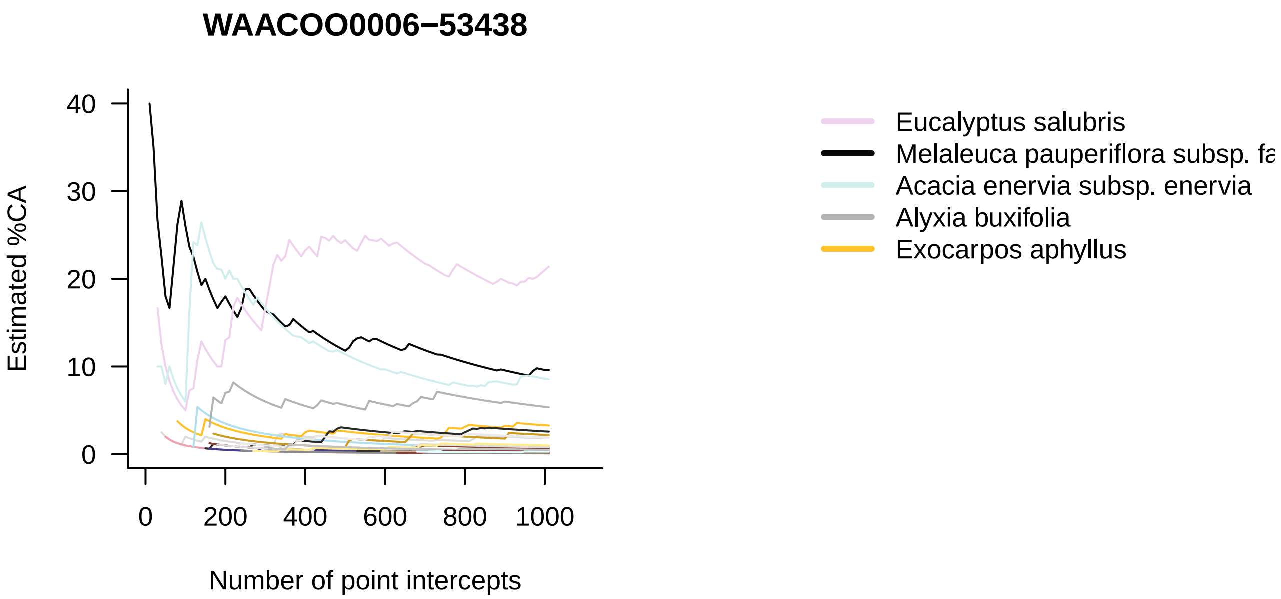
WAACOO0006−53438
Estimated %CA
40
30
20
10
0
0
200
400
600
800
1000
Number of point intercepts
Eucalyptus salubris
Melaleuca pauperiflora subsp. fa
Acacia enervia subsp. enervia
Alyxia buxifolia
Exocarpos aphyllus

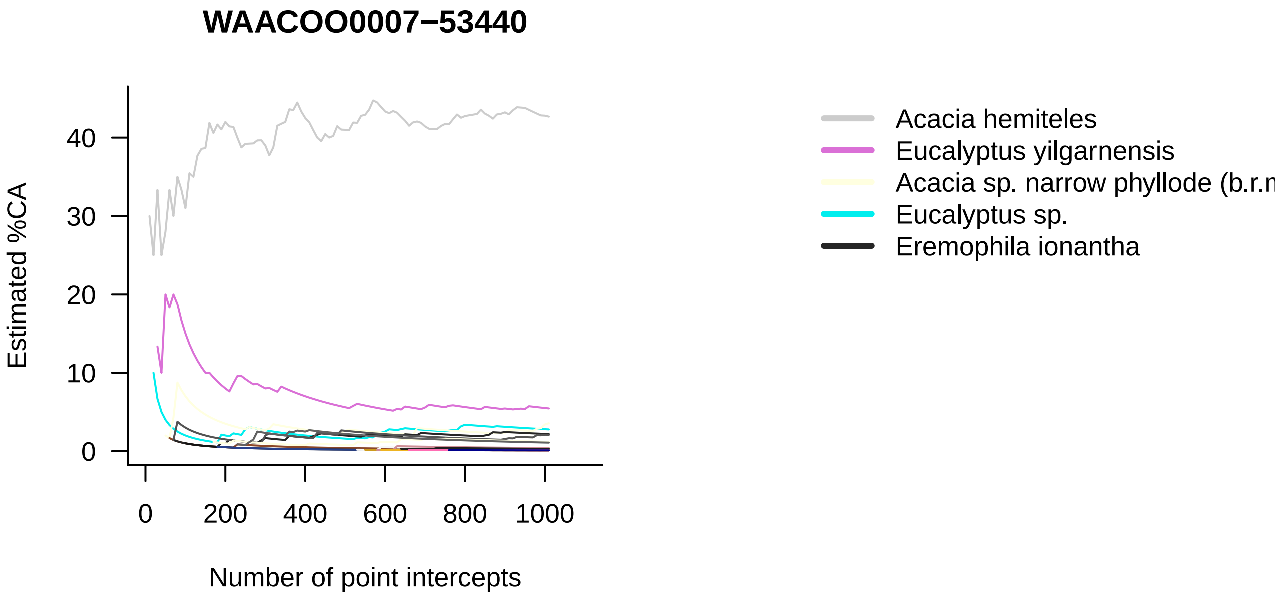
WAACOO0007−53440
Estimated %CA
40
30
20
10
0
0
200
400
600
800
1000
Number of point intercepts
Acacia hemiteles
Eucalyptus yilgarnensis
Acacia sp. narrow phyllode (b.r.m
Eucalyptus sp.
Eremophila ionantha

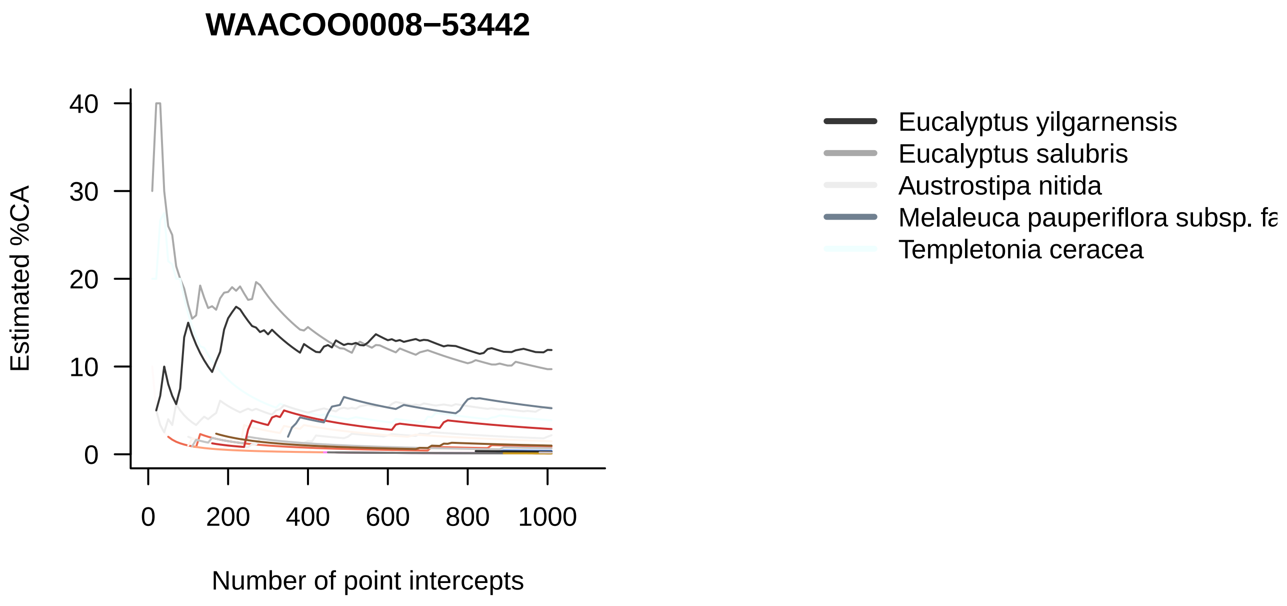
WAACOO0008−53442
Estimated %CA
Number of point intercepts
0
10
20
30
40
0
200
400
600
800
1000
Eucalyptus yilgarnensis
Eucalyptus salubris
Austrostipa nitida
Melaleuca pauperiflora subsp. fa
Templetonia ceracea

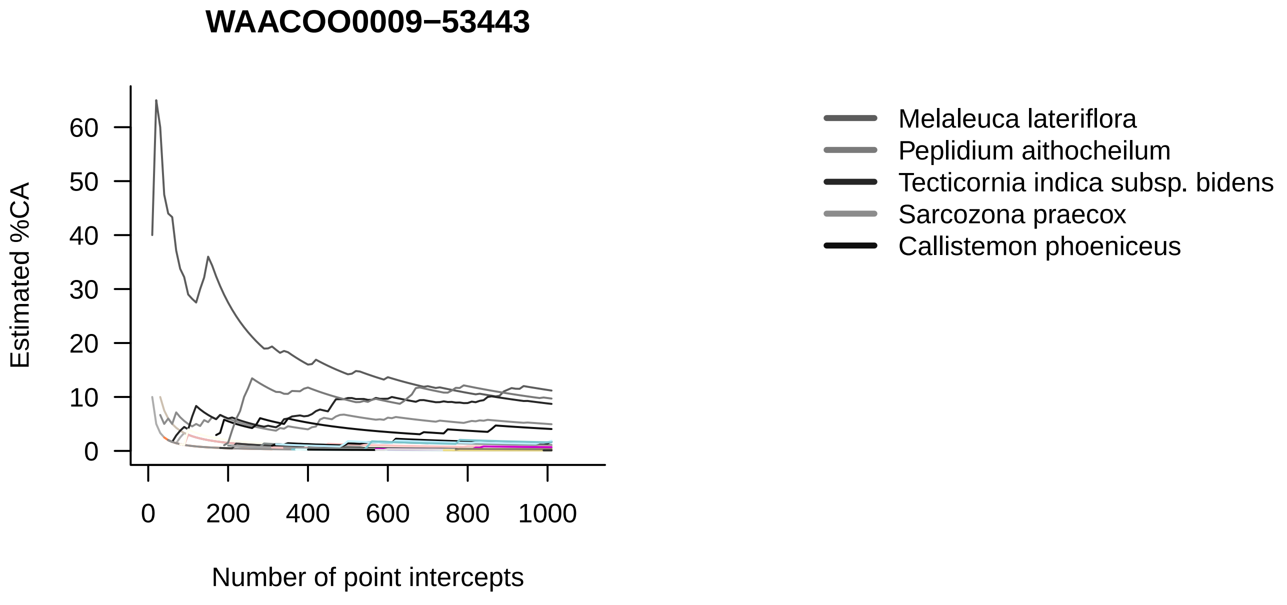
WAACOO0009−53443
Estimated %CA
Number of point intercepts
0
10
20
30
40
50
60
0
200
400
600
800
1000
Melaleuca lateriflora
Peplidium aithocheilum
Tecticornia indica subsp. bidens
Sarcozona praecox
Callistemon phoeniceus

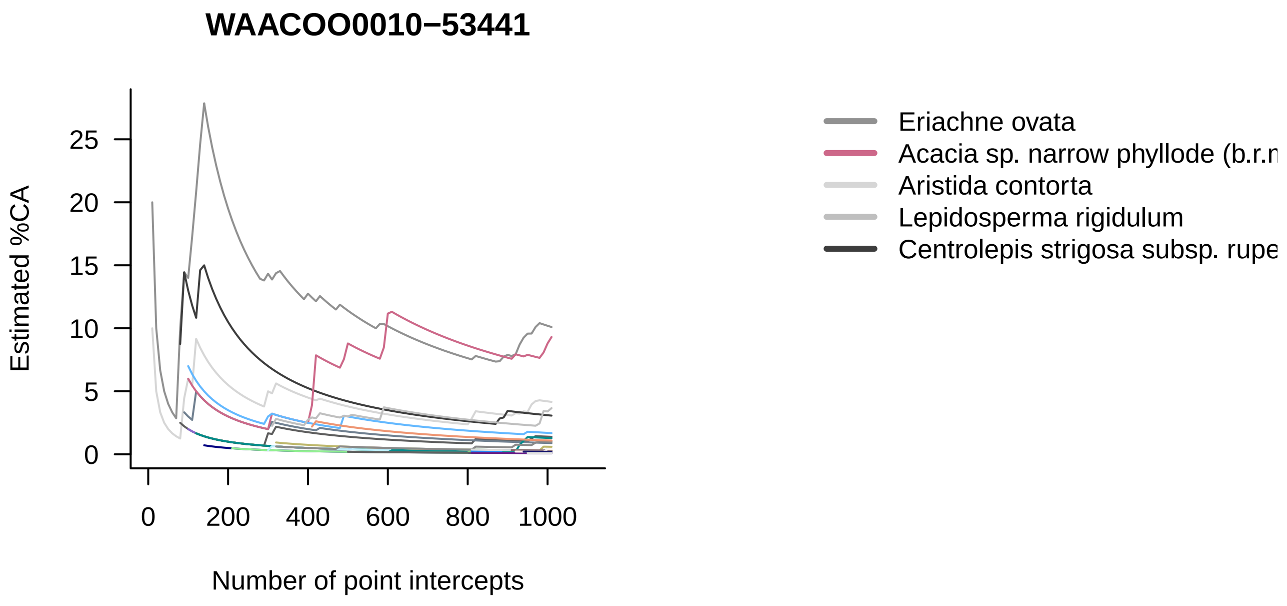
WAACOO0010−53441
Estimated %CA
Number of point intercepts
0
5
10
15
20
25
0
200
400
600
800
1000
Eriachne ovata
Acacia sp. narrow phyllode (b.r.n
Aristida contorta
Lepidosperma rigidulum
Centrolepis strigosa subsp. rupe

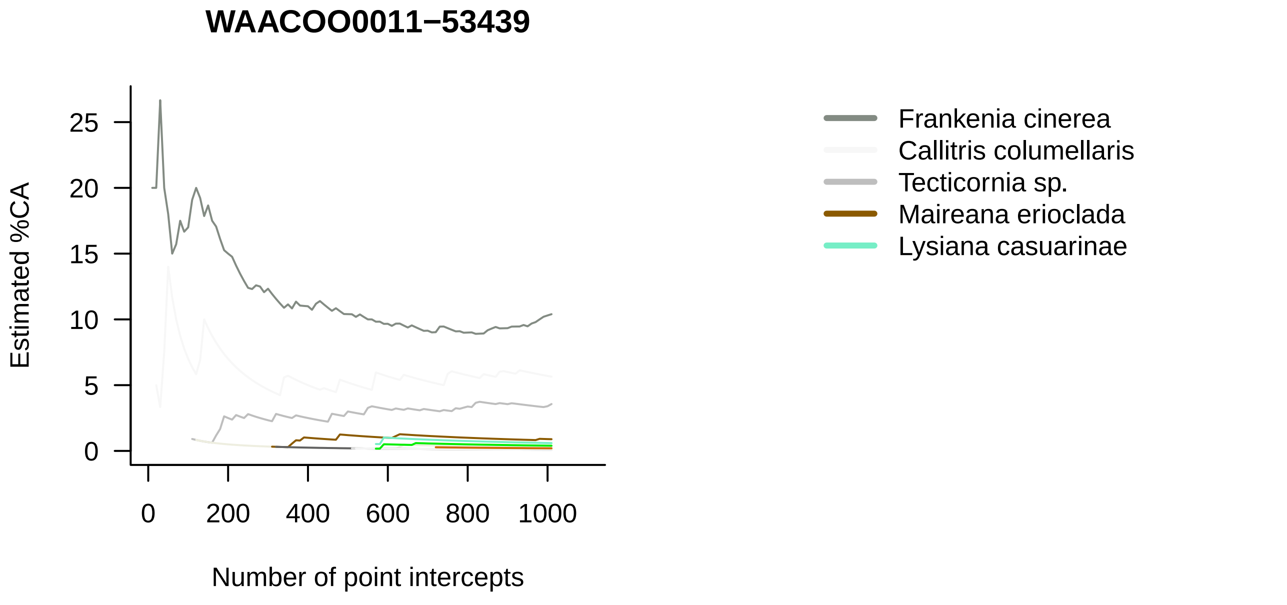
WAACOO0011−53439
Estimated %CA
Number of point intercepts
0
5
10
15
20
25
0
200
400
600
800
1000
Frankenia cinerea
Callitris columellaris
Tecticornia sp.
Maireana erioclada
Lysiana casuarinae

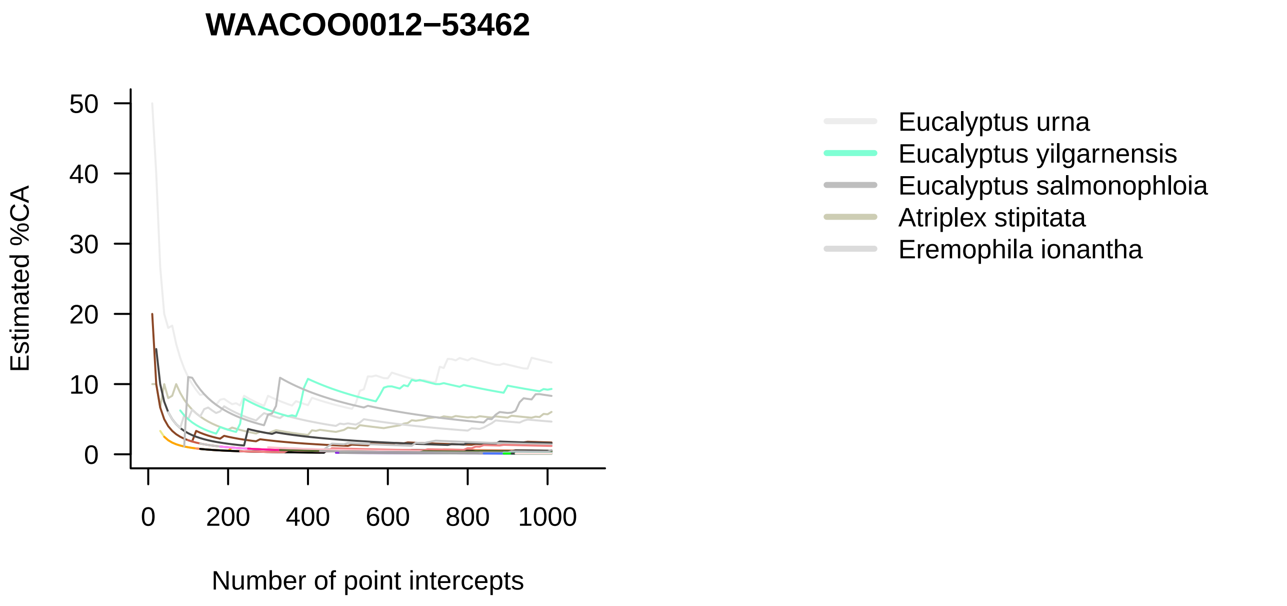
WAACOO0012−53462
Estimated %CA
50
40
30
20
10
0
0
200
400
600
800
1000
Number of point intercepts
Eucalyptus urna
Eucalyptus yilgarnensis
Eucalyptus salmonophloia
Atriplex stipitata
Eremophila ionantha

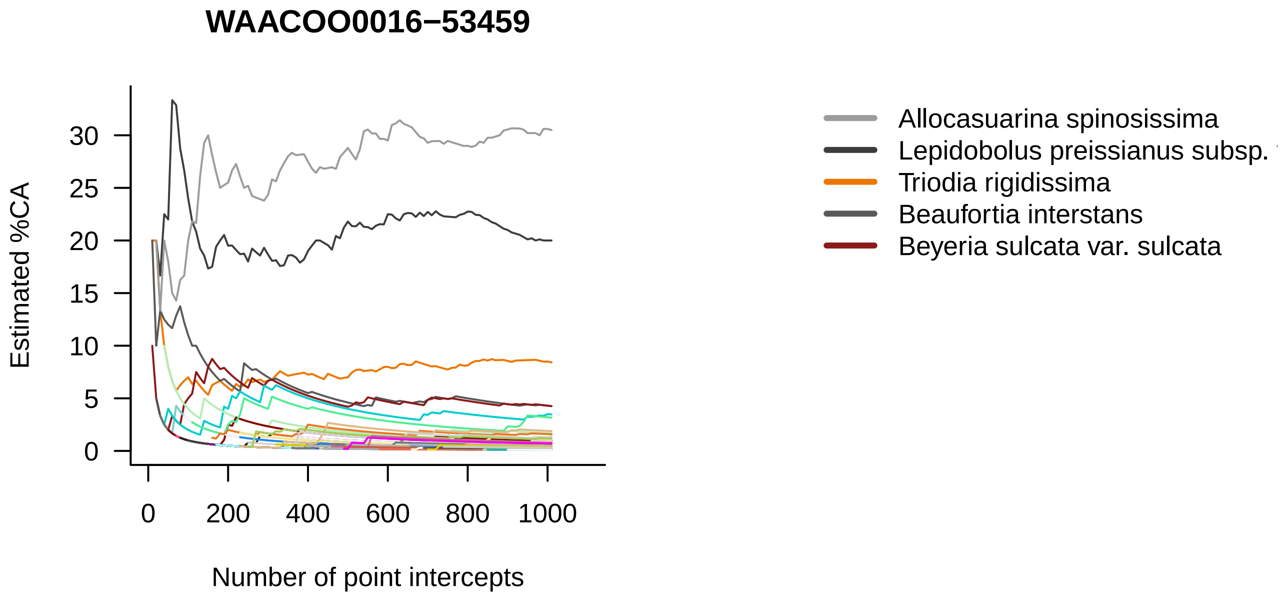
WAACOO0016−53459
Estimated %CA
30
25
20
15
10
5
0
0
200
400
600
800
1000
Number of point intercepts
Allocasuarina spinosissima
Lepidobolus preissianus subsp.
Triodia rigidissima
Beaufortia interstans
Beyeria sulcata var. sulcata

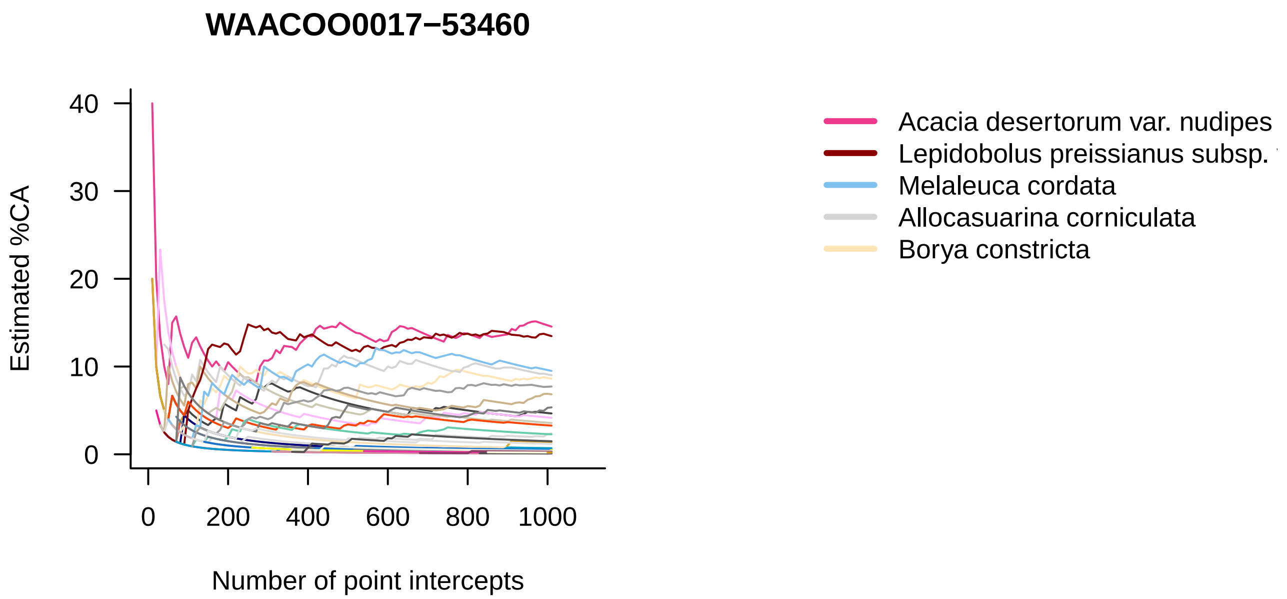
WAACOO0017−53460
Estimated %CA
40
30
20
10
0
0
200
400
600
800
1000
Number of point intercepts
Acacia desertorum var. nudipes
Lepidobolus preissianus subsp.
Melaleuca cordata
Allocasuarina corniculata
Borya constricta

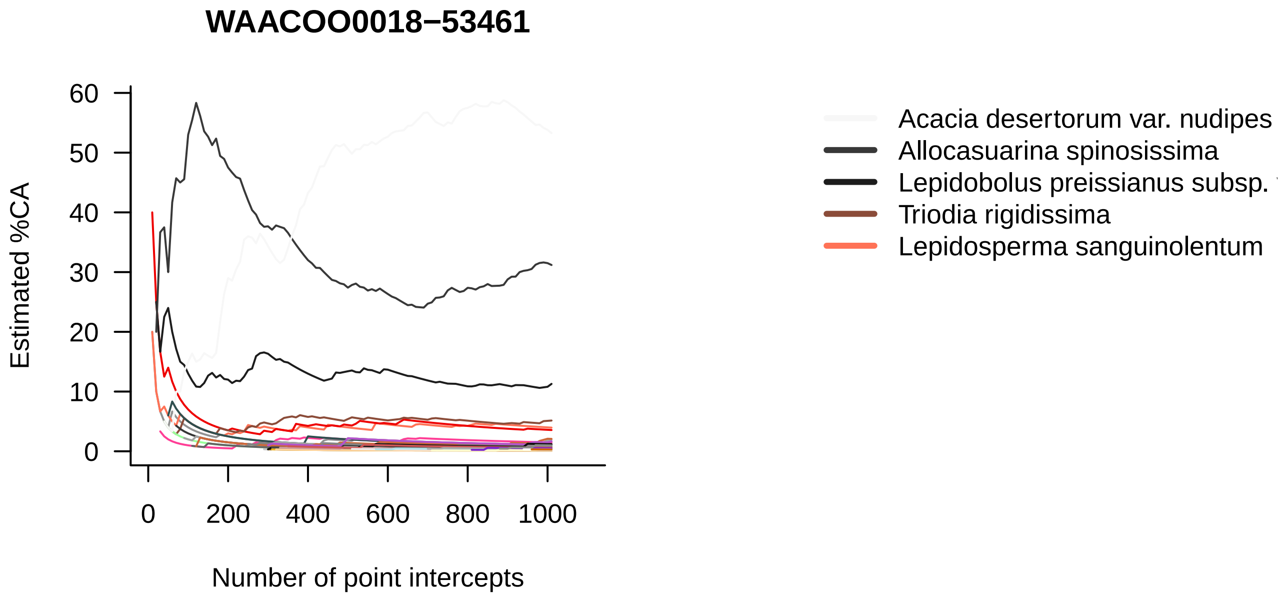
WAACOO0018−53461
Estimated %CA
60
50
40
30
20
10
0
0
200
400
600
800
1000
Number of point intercepts
Acacia desertorum var. nudipes
Allocasuarina spinosissima
Lepidobolus preissianus subsp.
Triodia rigidissima
Lepidosperma sanguinolentum

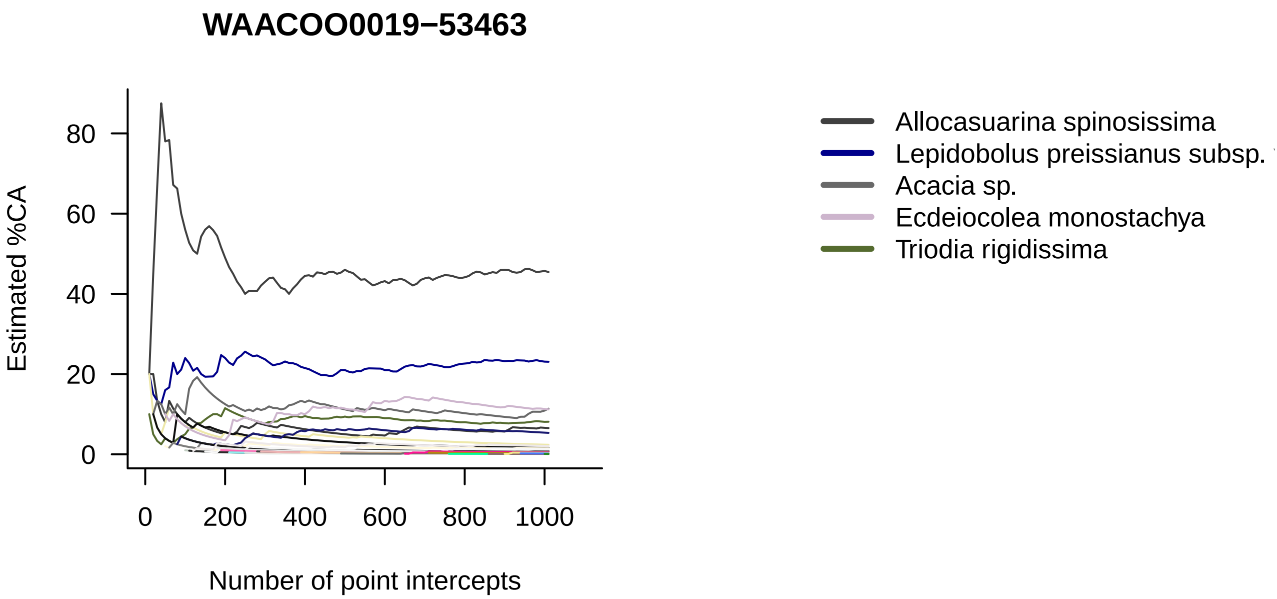
WAACOO0019−53463
Estimated %CA
0
20
40
60
80
0
200
400
600
800
1000
Number of point intercepts
Allocasuarina spinosissima
Lepidobolus preissianus subsp.
Acacia sp.
Ecdeiocolea monostachya
Triodia rigidissima

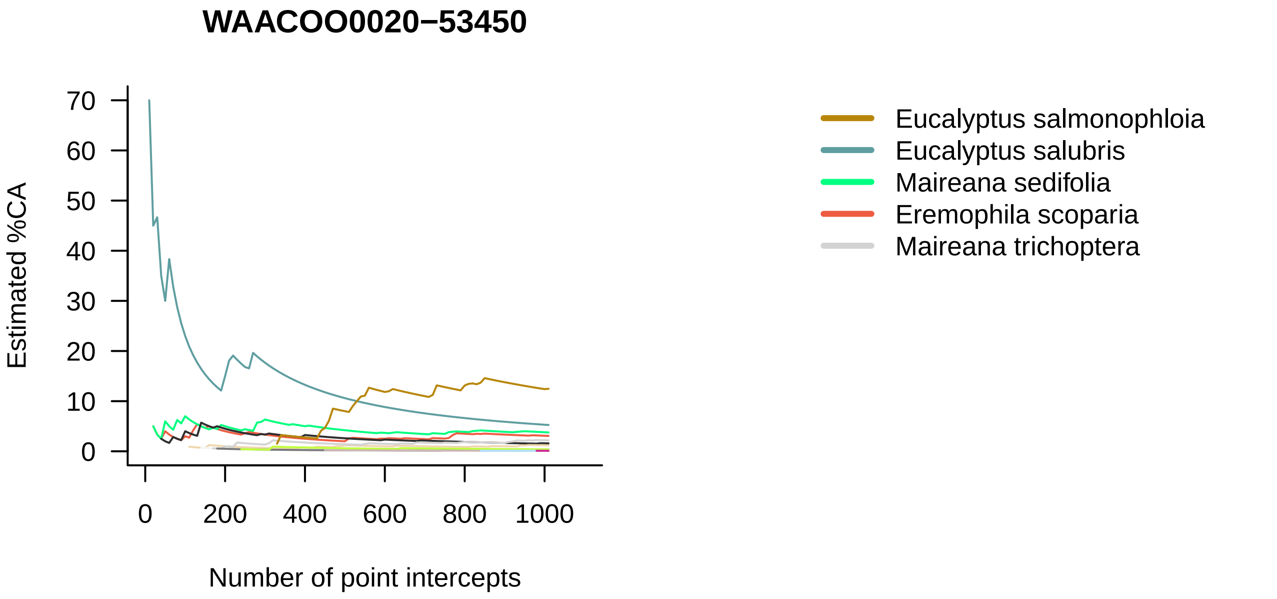
WAACOO0020−53450
Estimated %CA
0
10
20
30
40
50
60
70
0
200
400
600
800
1000
Number of point intercepts
Eucalyptus salmonophloia
Eucalyptus salubris
Maireana sedifolia
Eremophila scoparia
Maireana trichoptera

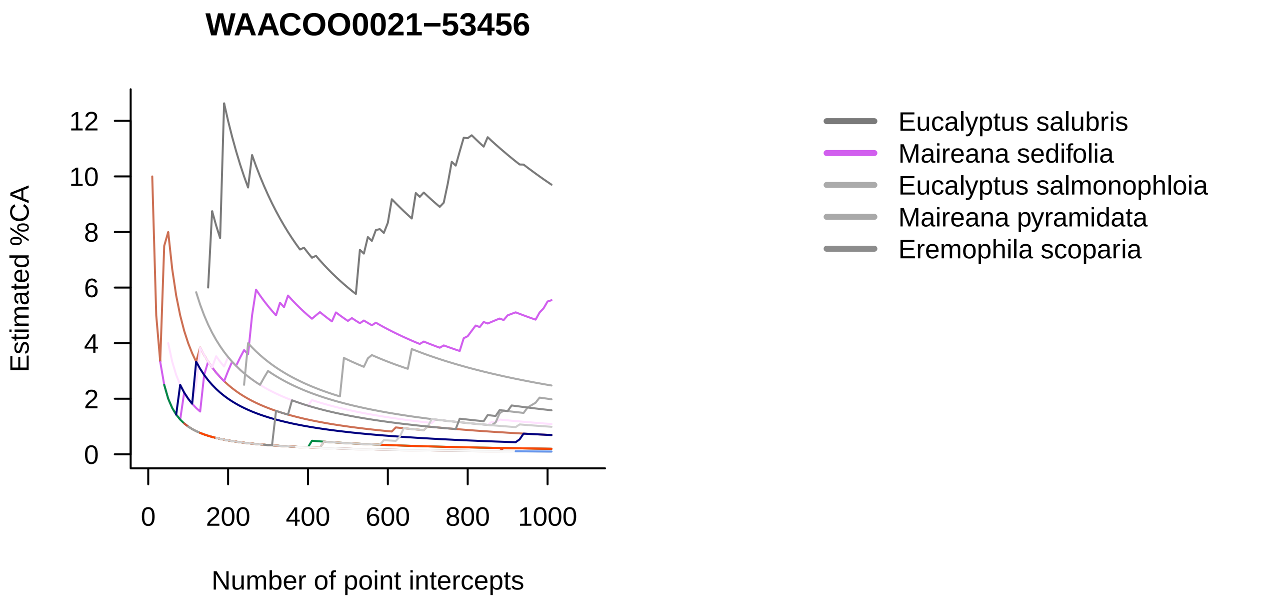
WAACOO0021−53456
Estimated %CA
0
2
4
6
8
10
12
0
200
400
600
800
1000
Number of point intercepts
Eucalyptus salubris
Maireana sedifolia
Eucalyptus salmonophloia
Maireana pyramidata
Eremophila scoparia

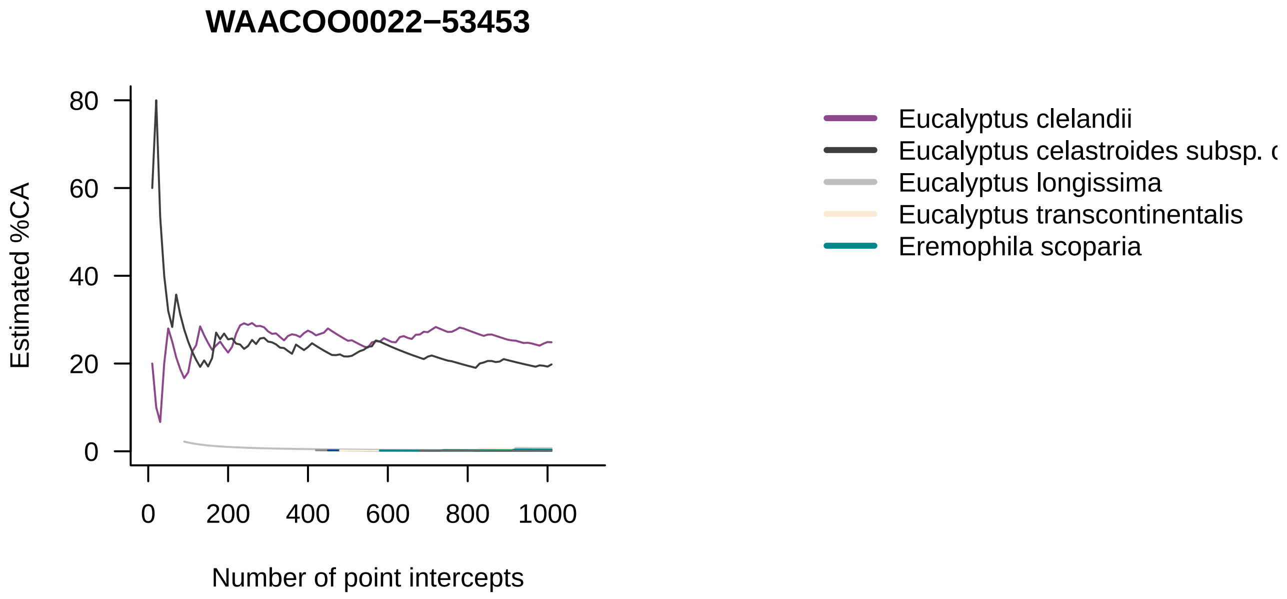
WAACOO0022−53453
Estimated %CA
0
20
40
60
80
0
200
400
600
800
1000
Number of point intercepts
Eucalyptus clelandii
Eucalyptus celastroides subsp. c
Eucalyptus longissima
Eucalyptus transcontinentalis
Eremophila scoparia

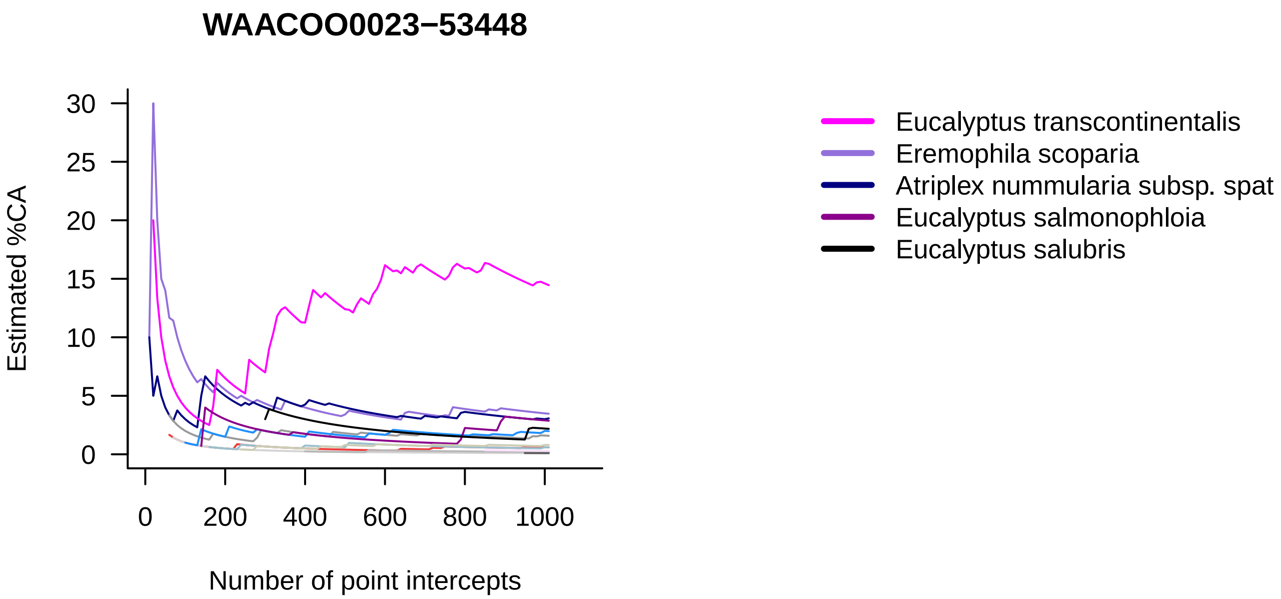
WAACOO0023−53448
Estimated %CA
0
5
10
15
20
25
30
0
200
400
600
800
1000
Number of point intercepts
Eucalyptus transcontinentalis
Eremophila scoparia
Atriplex nummularia subsp. spat
Eucalyptus salmonophloia
Eucalyptus salubris

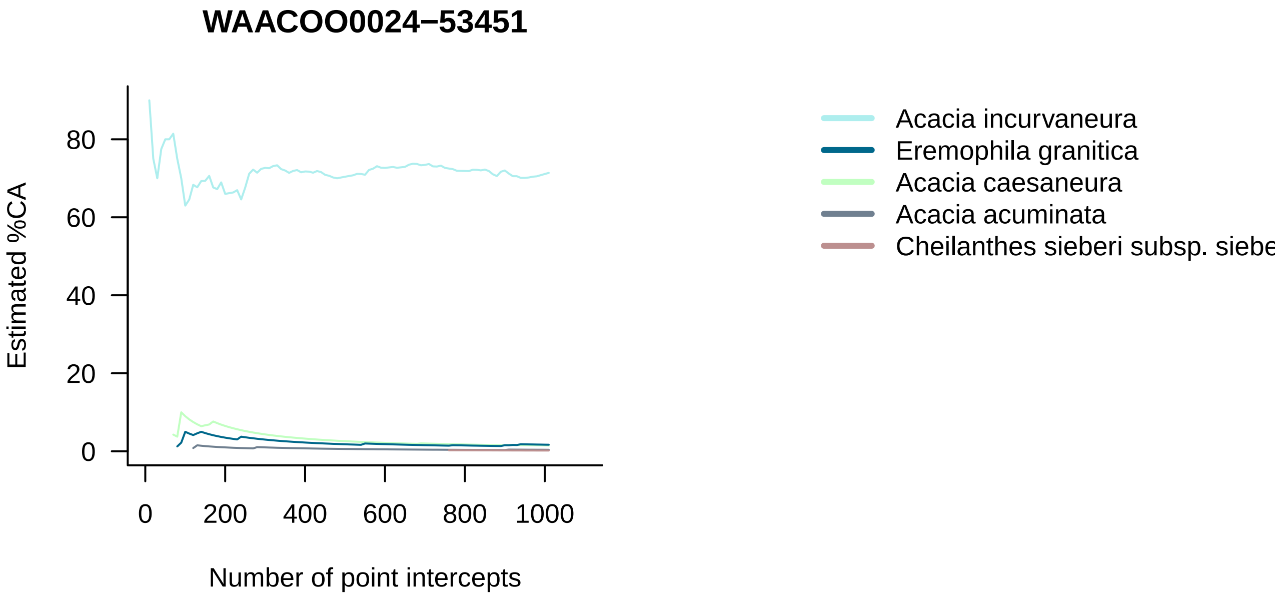
WAACOO0024−53451
Estimated %CA
0
20
40
60
80
0
200
400
600
800
1000
Number of point intercepts
Acacia incurvaneura
Eremophila granitica
Acacia caesaneura
Acacia acuminata
Cheilanthes sieberi subsp. siebe

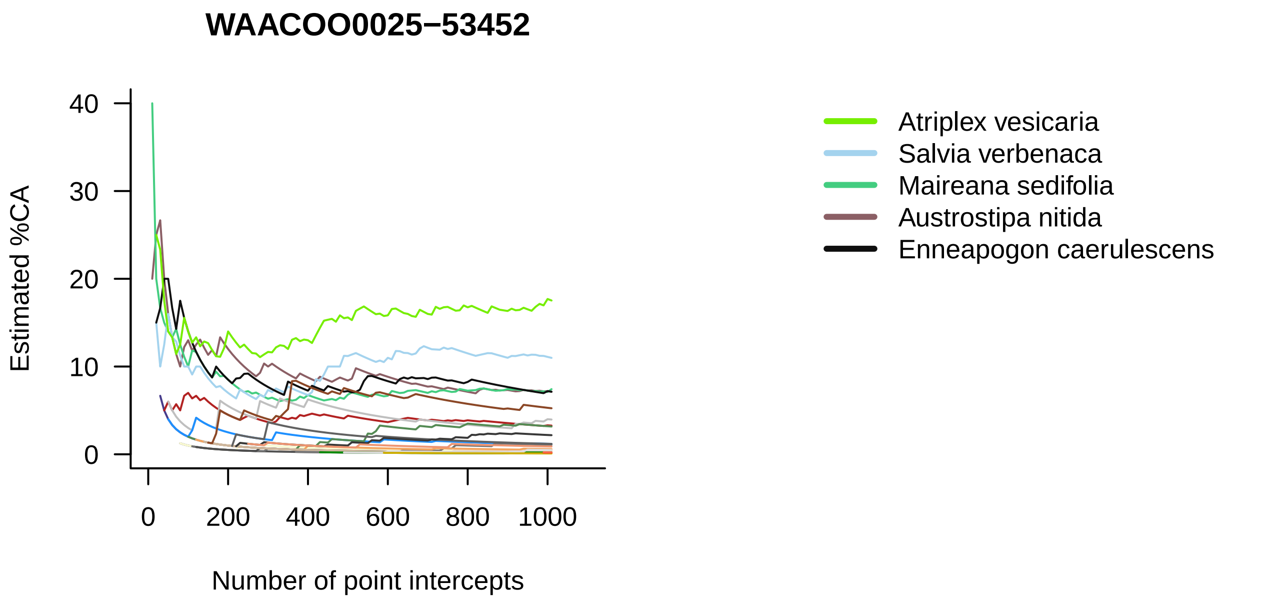
WAACOO0025−53452
Estimated %CA
40
30
20
10
0
0
200
400
600
800
1000
Number of point intercepts
Atriplex vesicaria
Salvia verbenaca
Maireana sedifolia
Austrostipa nitida
Enneapogon caerulescens

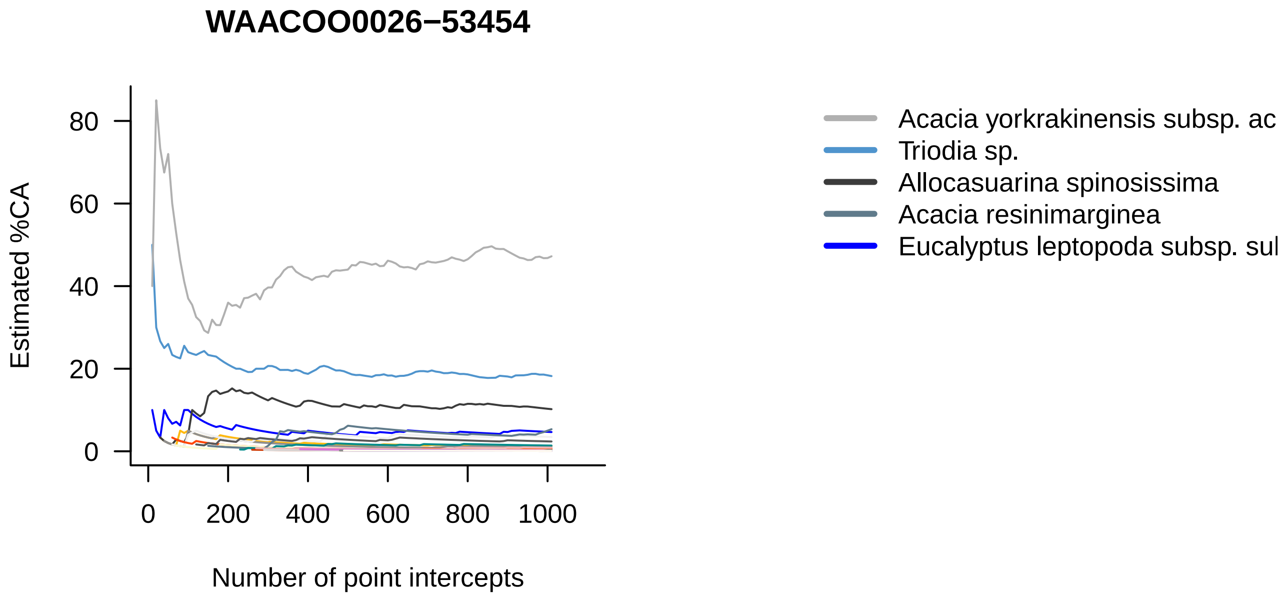
WAACOO0026−53454
Estimated %CA
80
60
40
20
0
0
200
400
600
800
1000
Number of point intercepts
Acacia yorkrakinensis subsp. ac
Triodia sp.
Allocasuarina spinosissima
Acacia resinimarginea
Eucalyptus leptopoda subsp. sul

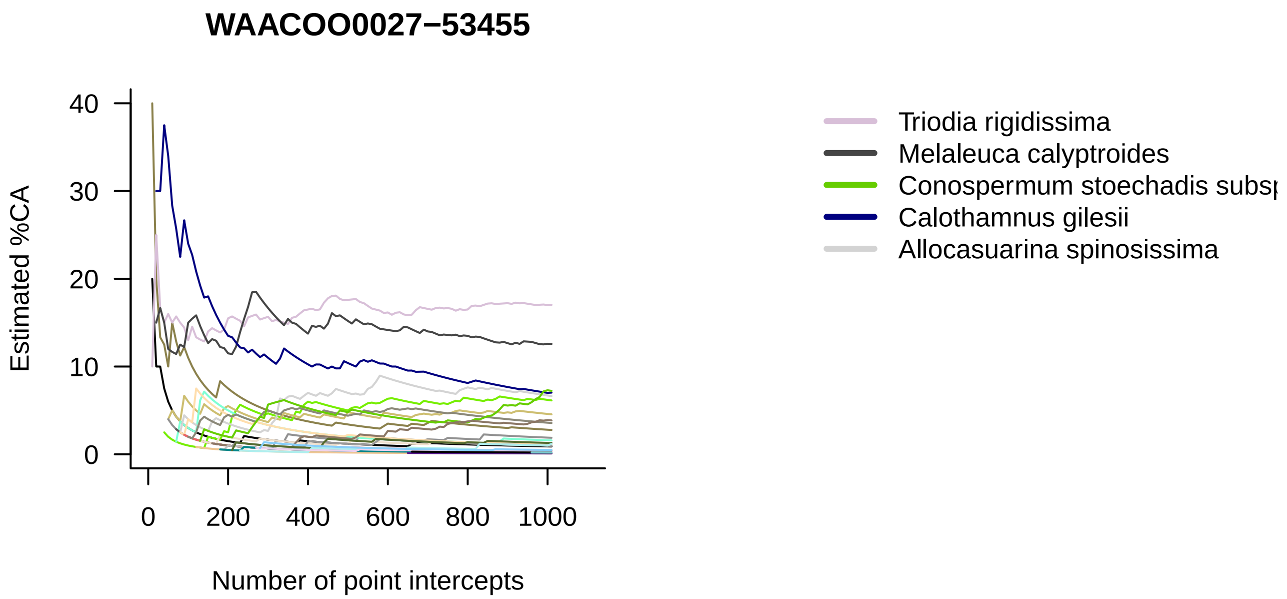
WAACOO0027−53455
Estimated %CA
40
30
20
10
0
0
200
400
600
800
1000
Number of point intercepts
Triodia rigidissima
Melaleuca calyptroides
Conospermum stoechadis subsp
Calothamnus gilesii
Allocasuarina spinosissima

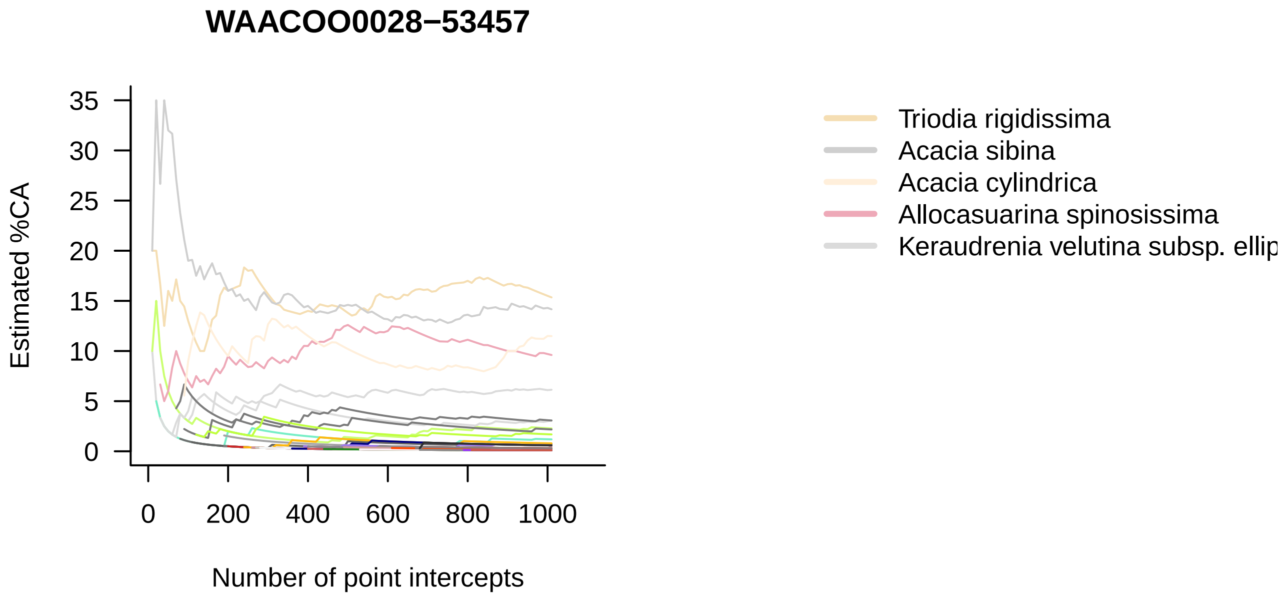
WAACOO0028−53457
Estimated %CA
35
30
25
20
15
10
5
0
0
200
400
600
800
1000
Number of point intercepts
Triodia rigidissima
Acacia sibina
Acacia cylindrica
Allocasuarina spinosissima
Keraudrenia velutina subsp. ellip

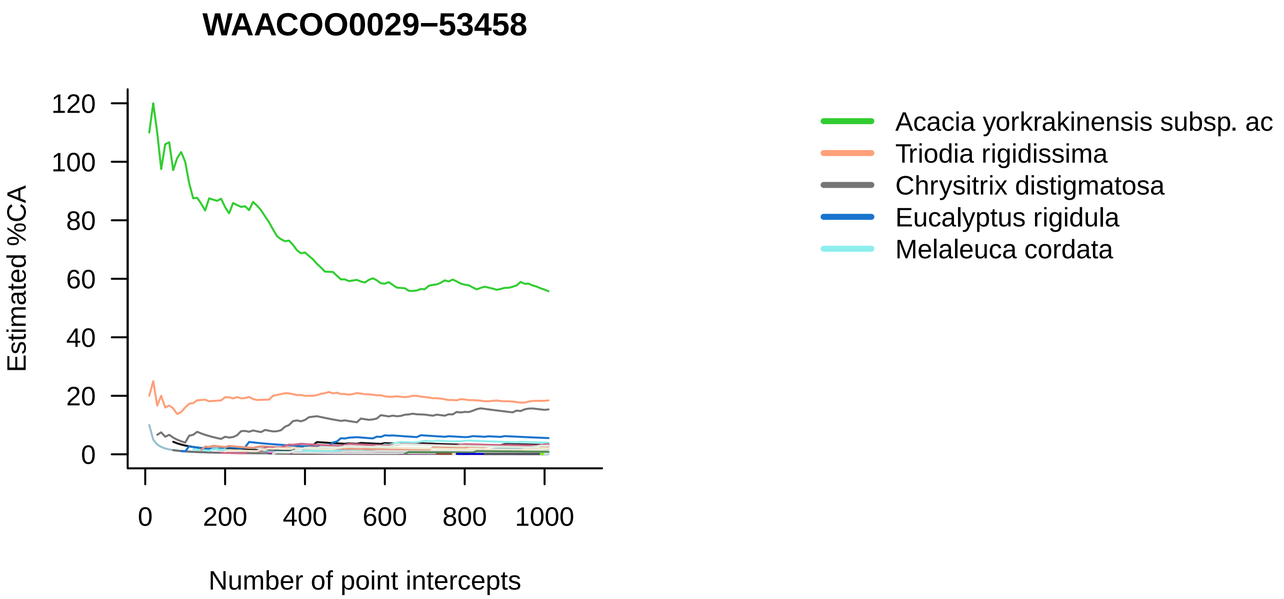
WAACOO0029−53458
Estimated %CA
0
20
40
60
80
100
120
0
200
400
600
800
1000
Number of point intercepts
Acacia yorkrakinensis subsp. ac
Triodia rigidissima
Chrysitrix distigmatosa
Eucalyptus rigidula
Melaleuca cordata

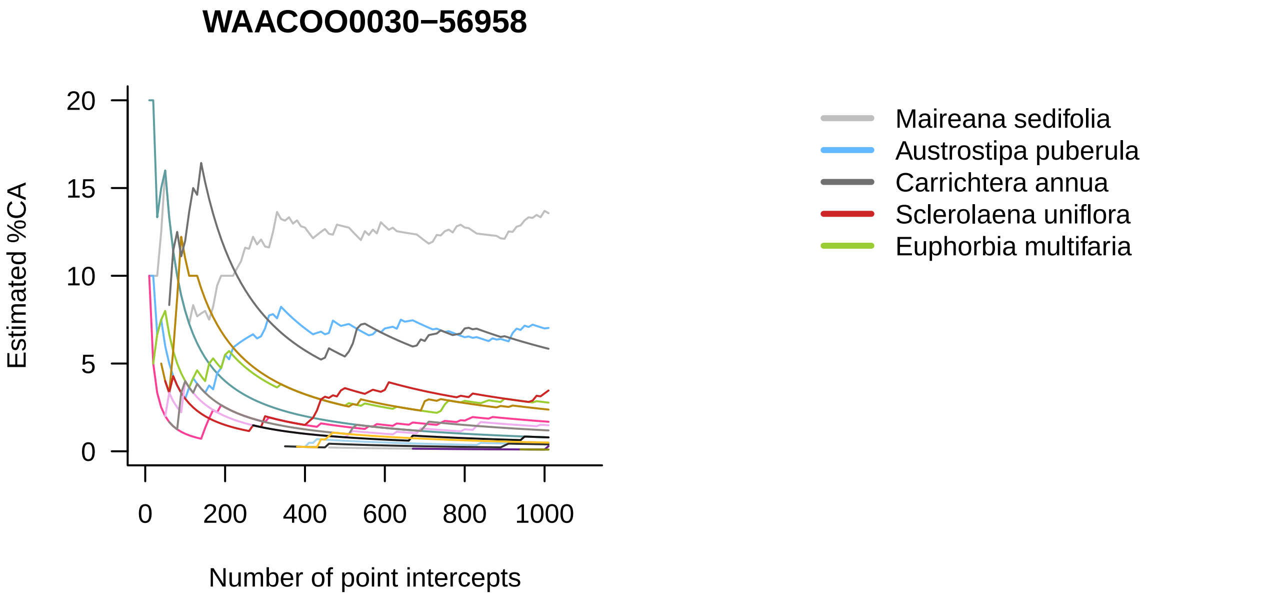
WAACOO0030−56958
Estimated %CA
0
5
10
15
20
0
200
400
600
800
1000
Number of point intercepts
Maireana sedifolia
Austrostipa puberula
Carrichtera annua
Sclerolaena uniflora
Euphorbia multifaria

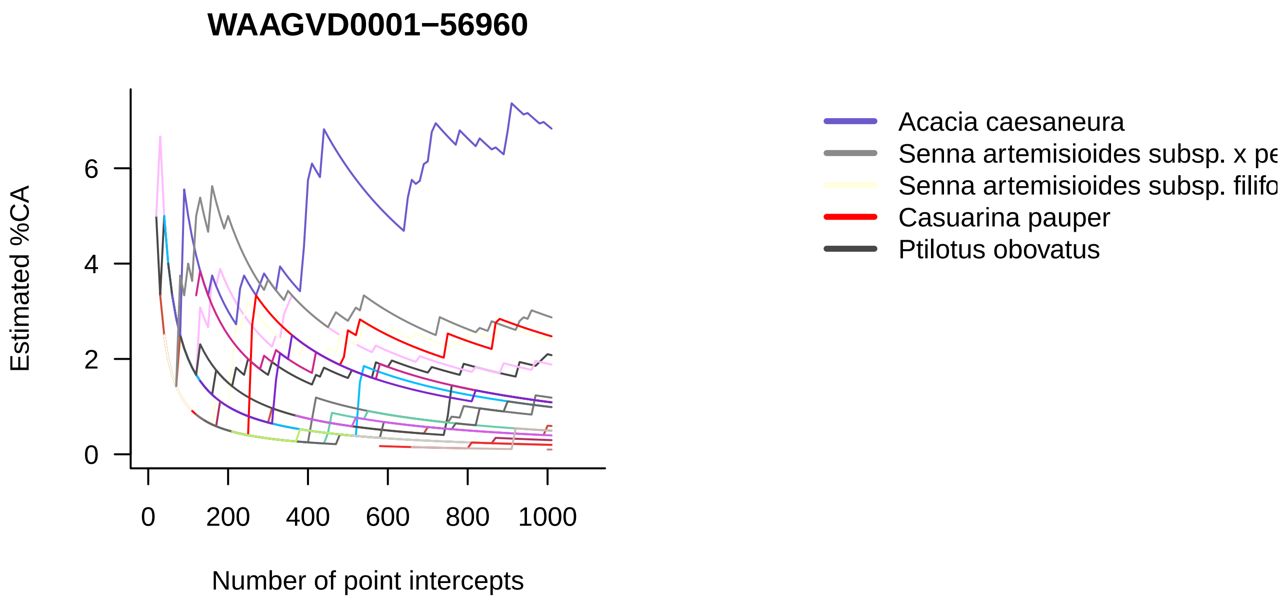
WAAGVD0001−56960
Estimated %CA
0
2
4
6
0
200
400
600
800
1000
Number of point intercepts
Acacia caesaneura
Senna artemisioides subsp. x pe
Senna artemisioides subsp. filifo
Casuarina pauper
Ptilotus obovatus

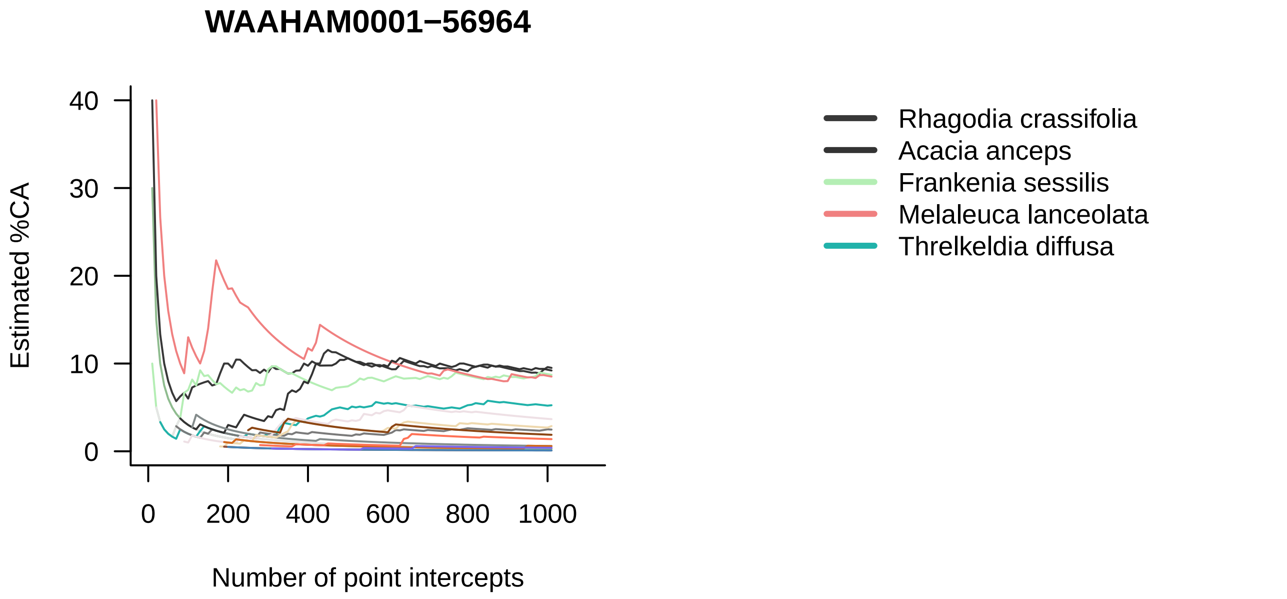
WAAHAM0001−56964
Estimated %CA
0
10
20
30
40
0
200
400
600
800
1000
Number of point intercepts
Rhagodia crassifolia
Acacia anceps
Frankenia sessilis
Melaleuca lanceolata
Threlkeldia diffusa

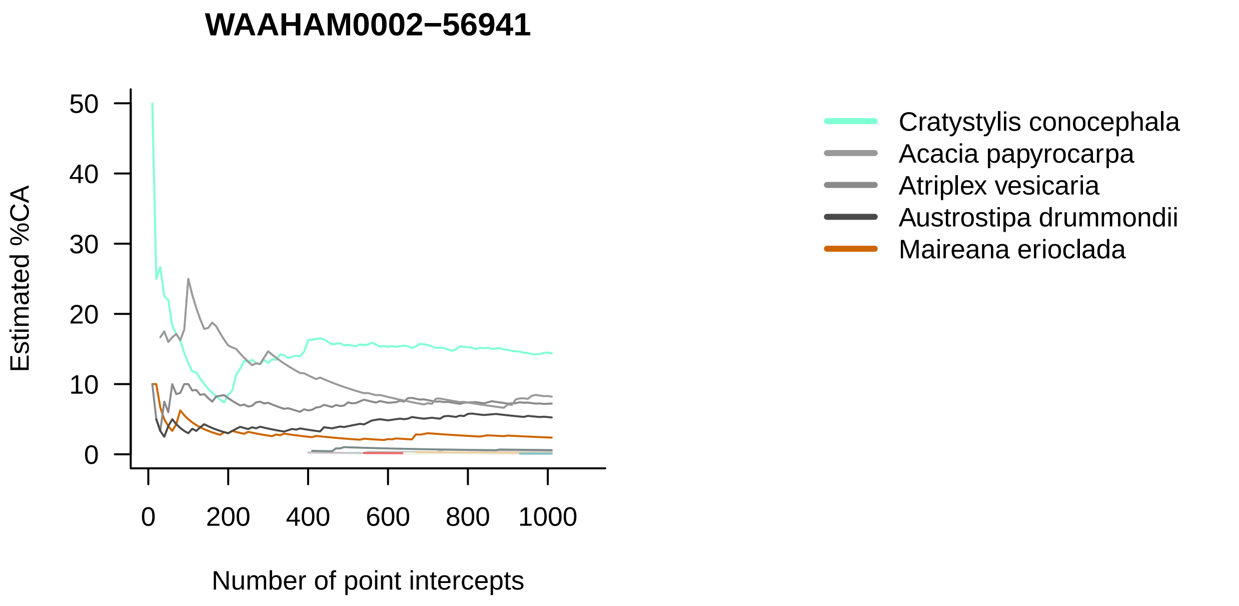
WAAHAM0002−56941
Estimated %CA
50
40
30
20
10
0
0
200
400
600
800
1000
Number of point intercepts
Cratystylis conocephala
Acacia papyrocarpa
Atriplex vesicaria
Austrostipa drummondii
Maireana erioclada

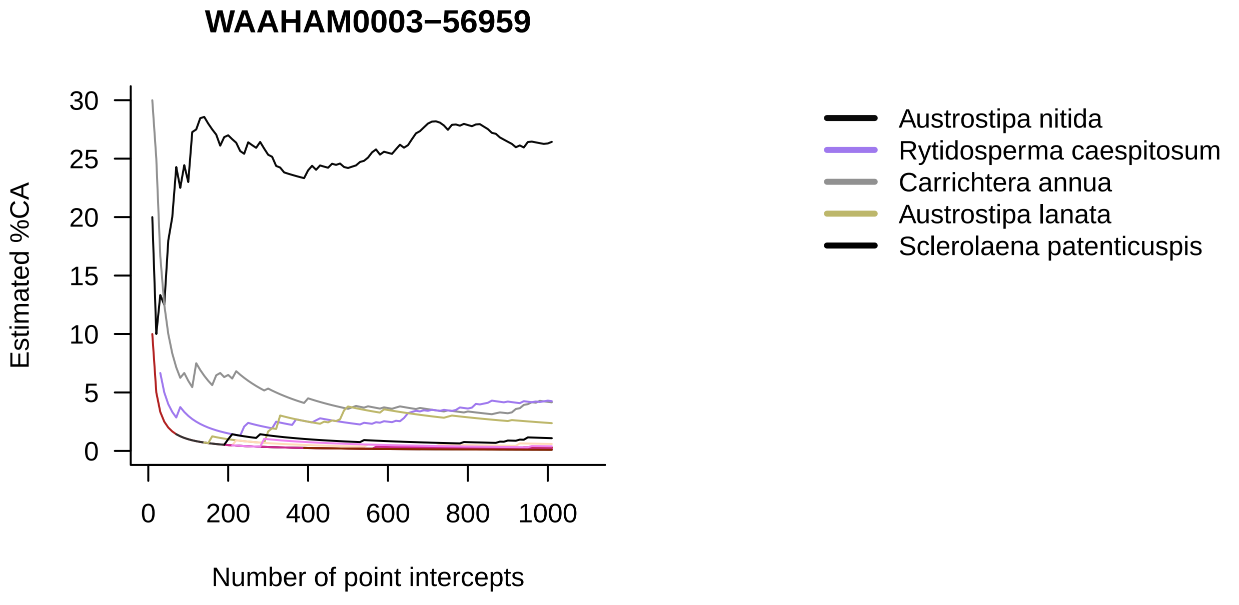
WAAHAM0003−56959
Estimated %CA
30
25
20
15
10
5
0
0
200
400
600
800
1000
Number of point intercepts
Austrostipa nitida
Rytidosperma caespitosum
Carrichtera annua
Austrostipa lanata
Sclerolaena patenticuspis

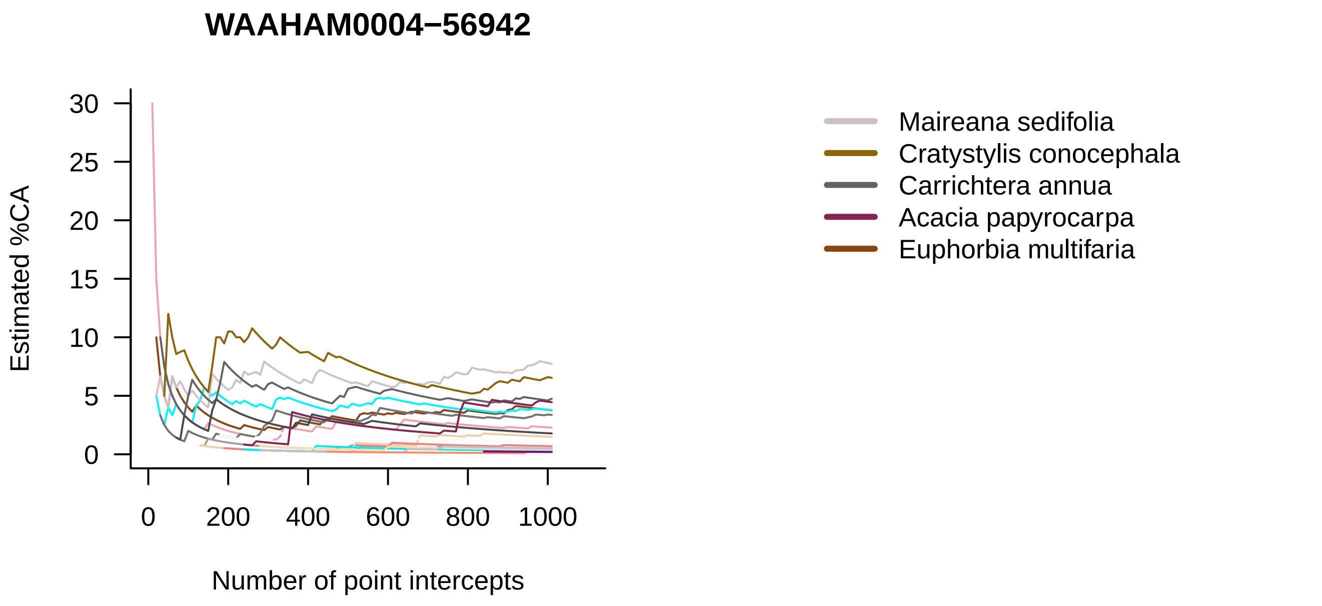
WAAHAM0004−56942
Estimated %CA
Number of point intercepts
0
5
10
15
20
25
30
0
200
400
600
800
1000
Maireana sedifolia
Cratystylis conocephala
Carrichtera annua
Acacia papyrocarpa
Euphorbia multifaria

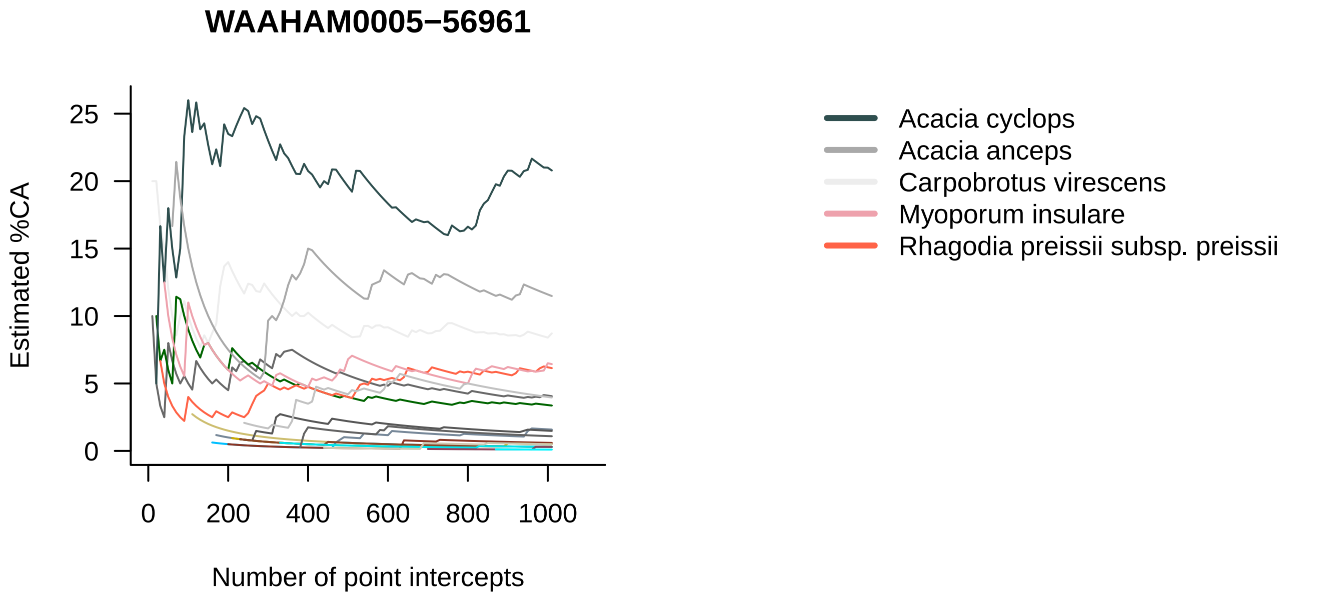
WAAHAM0005−56961
Estimated %CA
Number of point intercepts
0
5
10
15
20
25
0
200
400
600
800
1000
Acacia cyclops
Acacia anceps
Carpobrotus virescens
Myoporum insulare
Rhagodia preissii subsp. preissii

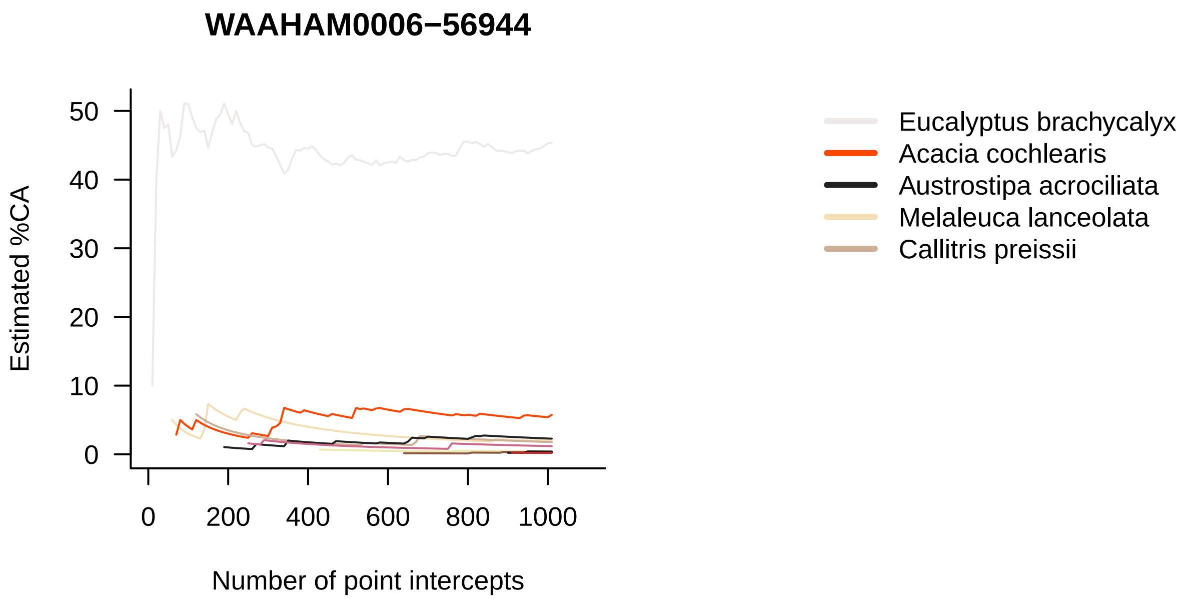
WAAHAM0006−56944
Estimated %CA
Number of point intercepts
0
10
20
30
40
50
0
200
400
600
800
1000
Eucalyptus brachycalyx
Acacia cochlearis
Austrostipa acrociliata
Melaleuca lanceolata
Callitris preissii

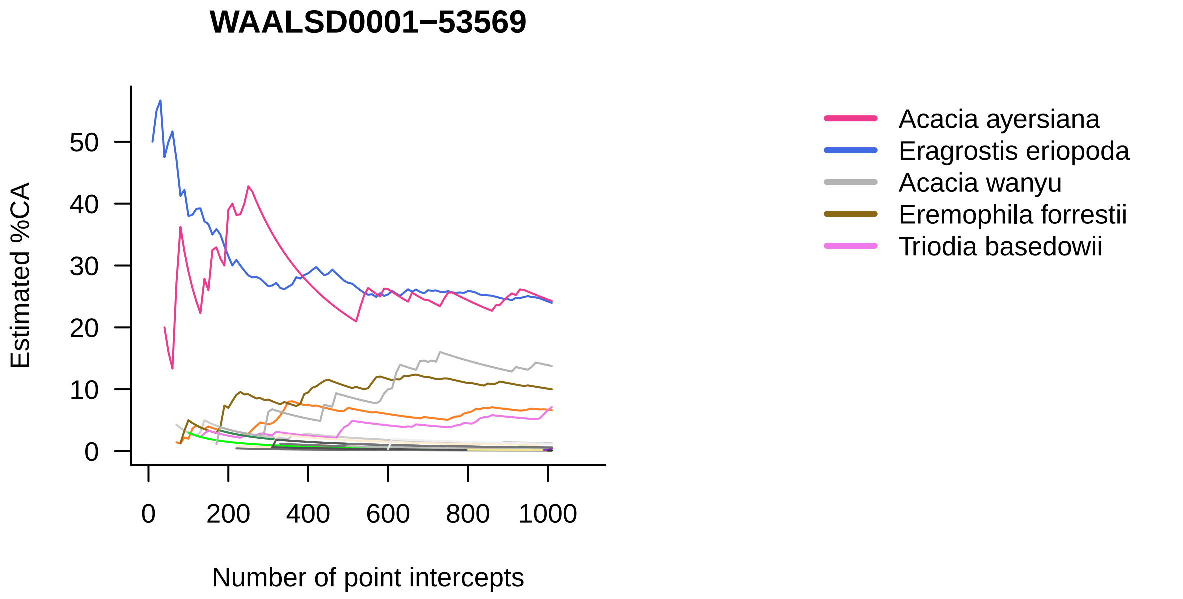
WAALSD0001−53569
Estimated %CA
Number of point intercepts
0
10
20
30
40
50
0
200
400
600
800
1000
Acacia ayersiana
Eragrostis eriopoda
Acacia wanyu
Eremophila forrestii
Triodia basedowii

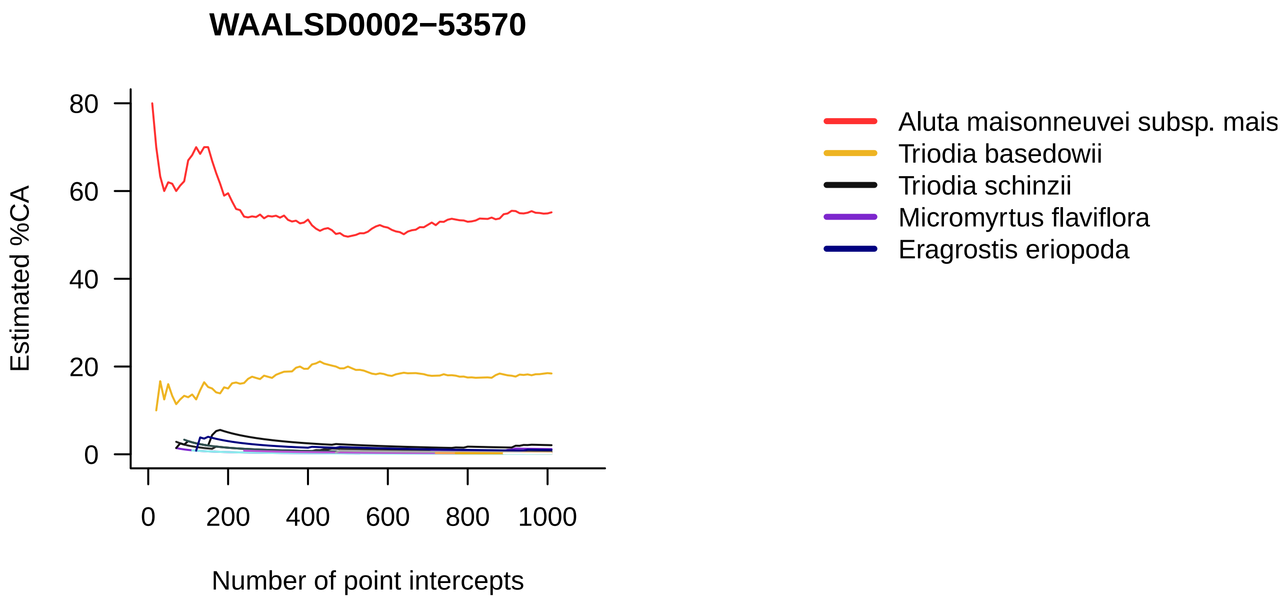
WAALSD0002−53570
Estimated %CA
0
20
40
60
80
0
200
400
600
800
1000
Number of point intercepts
Aluta maisonneuvei subsp. mais
Triodia basedowii
Triodia schinzii
Micromyrtus flaviflora
Eragrostis eriopoda

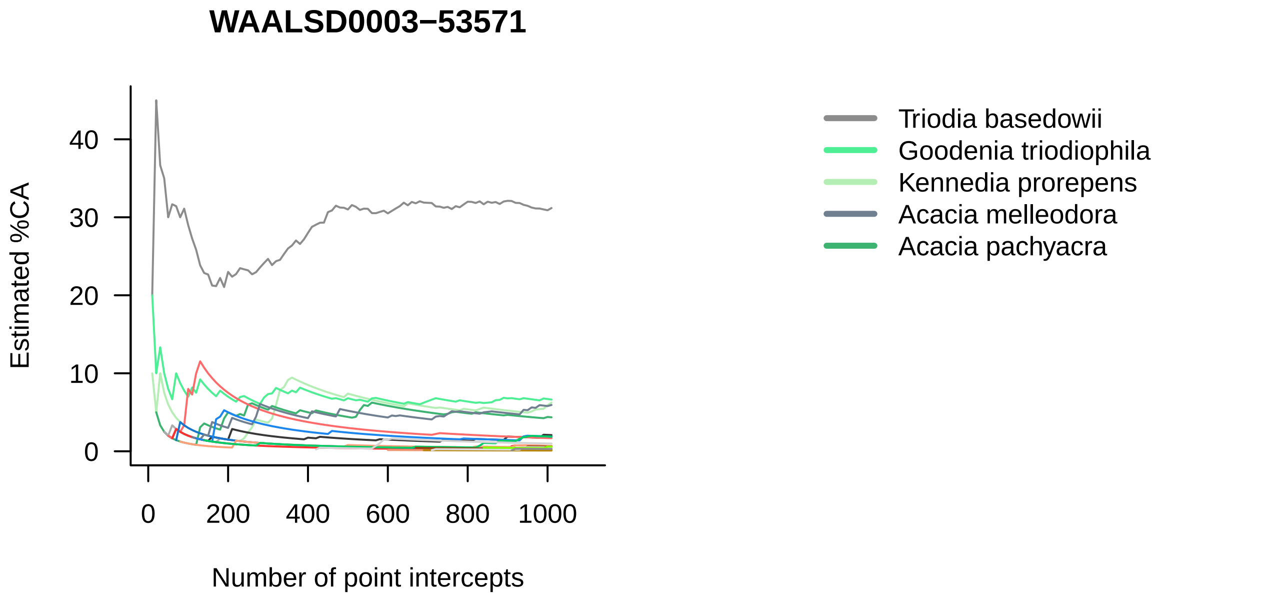
WAALSD0003−53571
Estimated %CA
0
10
20
30
40
0
200
400
600
800
1000
Number of point intercepts
Triodia basedowii
Goodenia triodiophila
Kennedia prorepens
Acacia melleodora
Acacia pachyacra

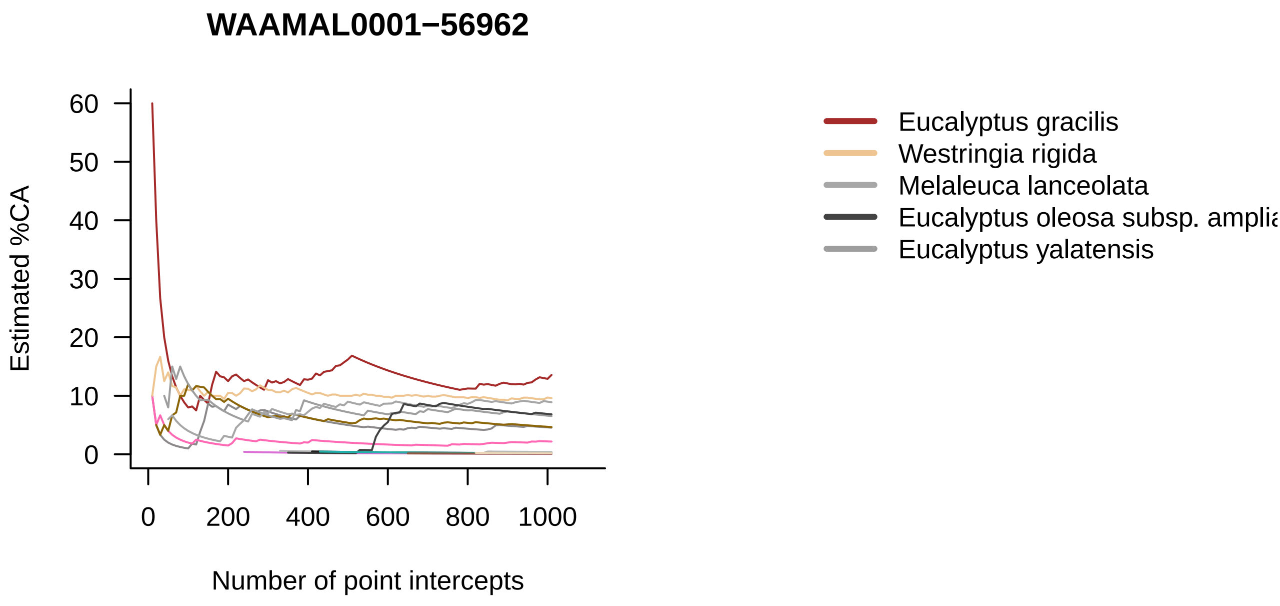
WAAMAL0001−56962
Estimated %CA
Number of point intercepts
0
10
20
30
40
50
60
0
200
400
600
800
1000
Eucalyptus gracilis
Westringia rigida
Melaleuca lanceolata
Eucalyptus oleosa subsp. amplia
Eucalyptus yalatensis

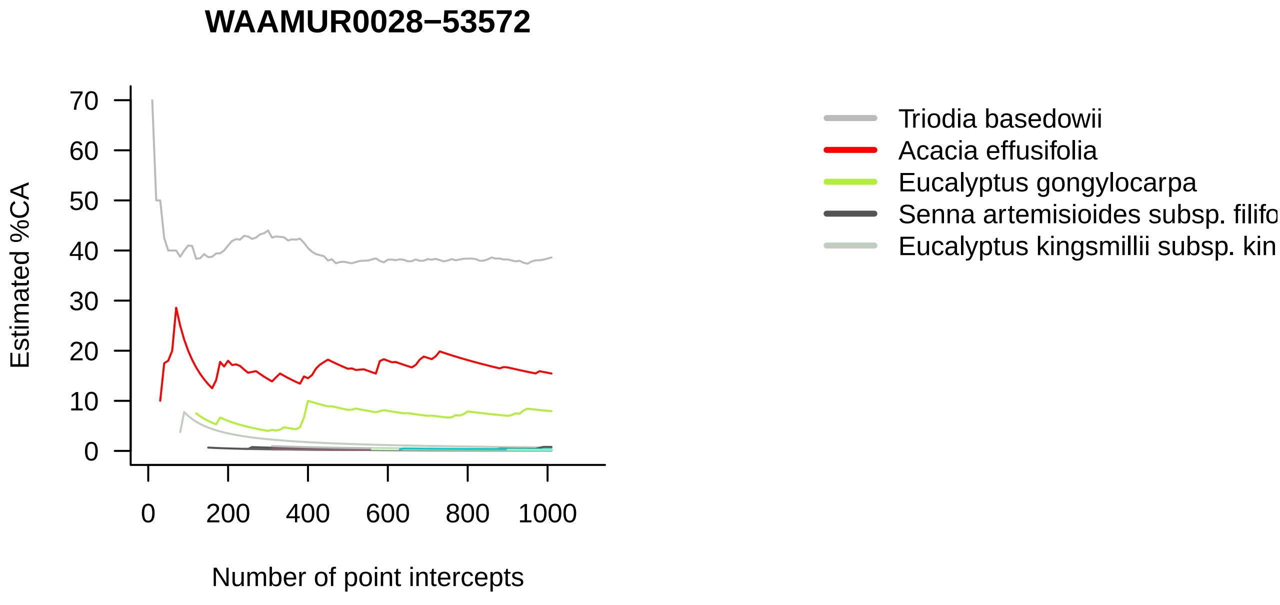
WAAMUR0028−53572
Estimated %CA
Number of point intercepts
0
10
20
30
40
50
60
70
0
200
400
600
800
1000
Triodia basedowii
Acacia effusifolia
Eucalyptus gongylocarpa
Senna artemisioides subsp. filifo
Eucalyptus kingsmillii subsp. kin

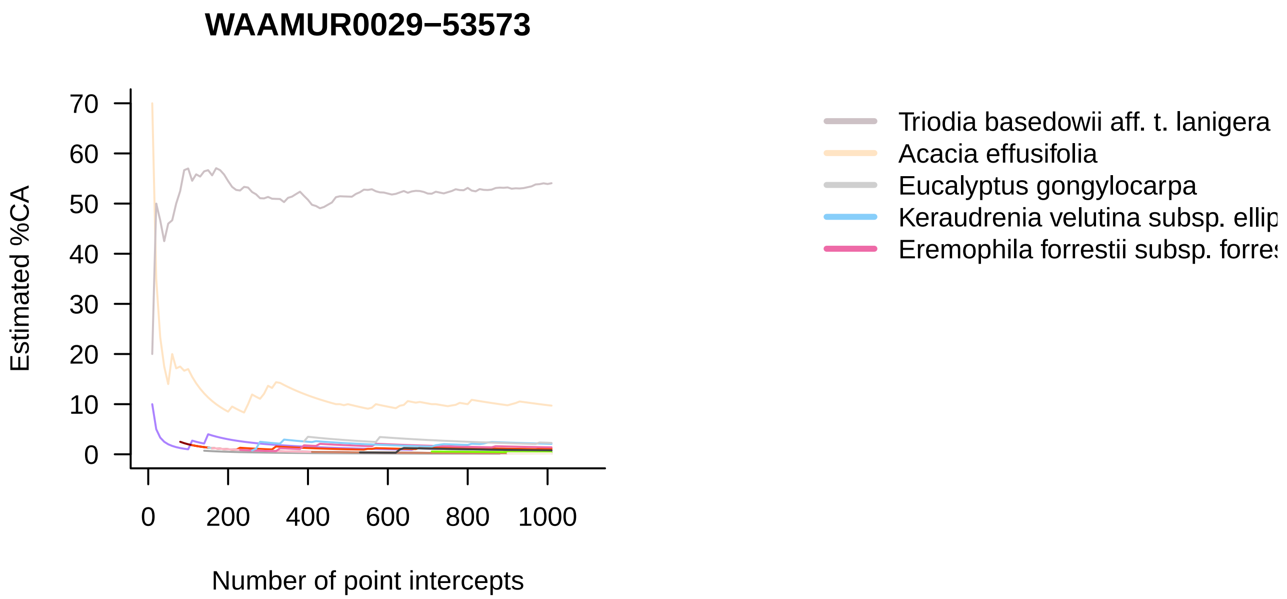
WAAMUR0029−53573
Estimated %CA
70
60
50
40
30
20
10
0
0
200
400
600
800
1000
Number of point intercepts
Triodia basedowii aff. t. lanigera
Acacia effusifolia
Eucalyptus gongylocarpa
Keraudrenia velutina subsp. ellip
Eremophila forrestii subsp. forres

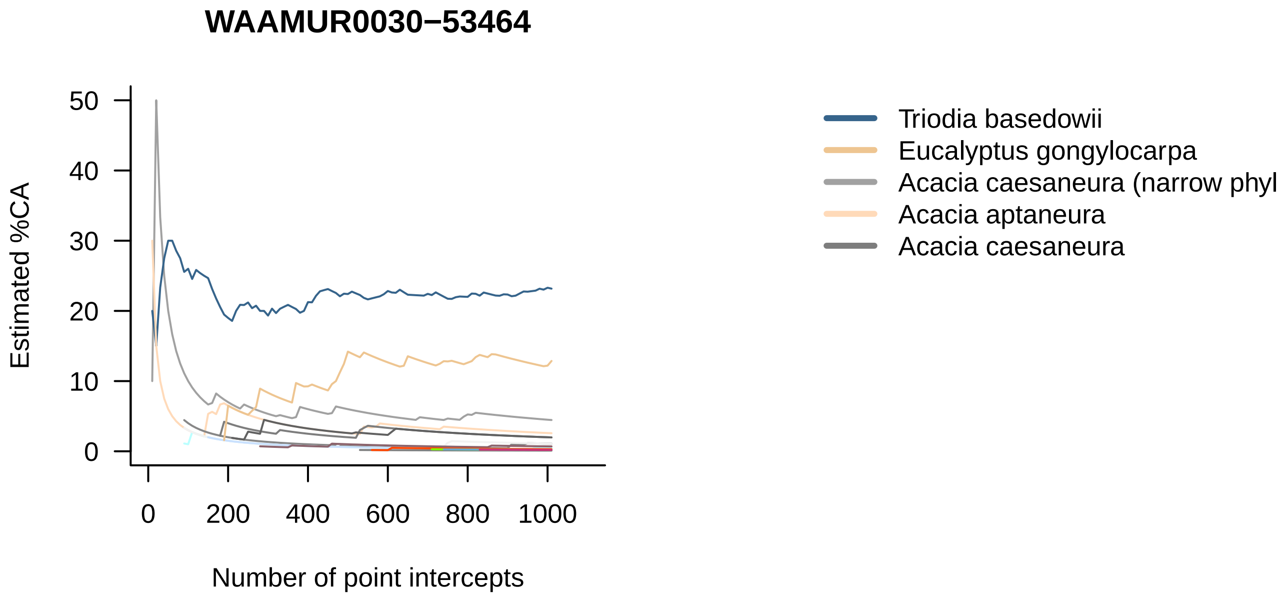
WAAMUR0030−53464
Estimated %CA
50
40
30
20
10
0
0
200
400
600
800
1000
Number of point intercepts
Triodia basedowii
Eucalyptus gongylocarpa
Acacia caesaneura (narrow phyl
Acacia aptaneura
Acacia caesaneura

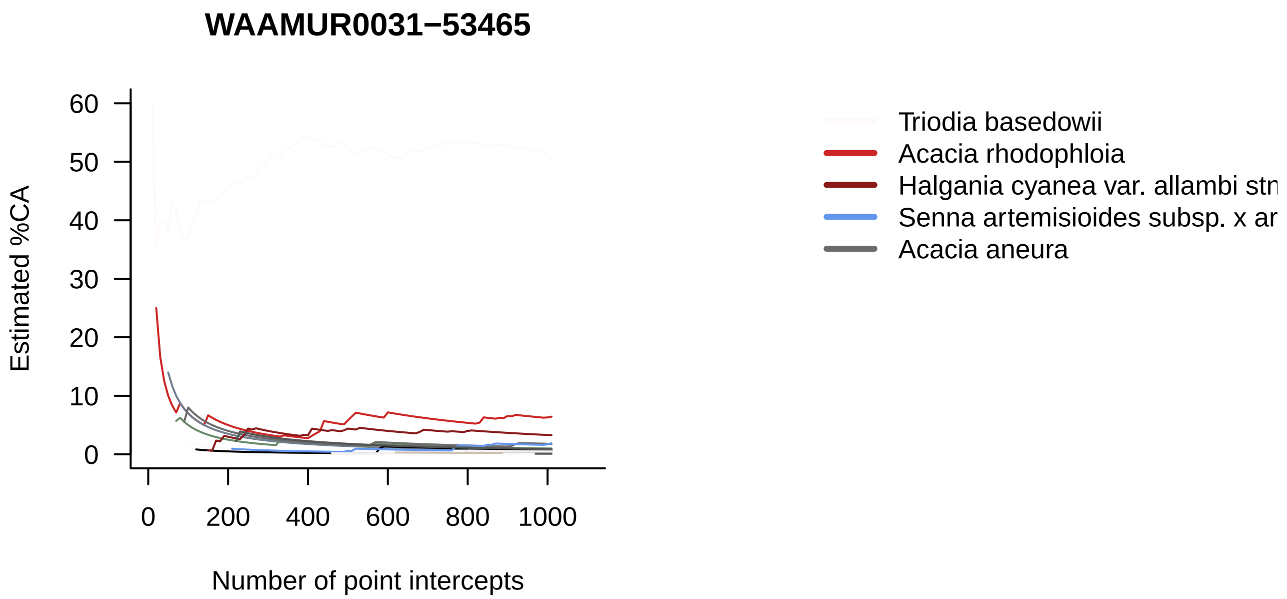
WAAMUR0031−53465
Estimated %CA
60
50
40
30
20
10
0
0
200
400
600
800
1000
Number of point intercepts
Triodia basedowii
Acacia rhodophloia
Halgania cyanea var. allambi str
Senna artemisioides subsp. x ar
Acacia aneura

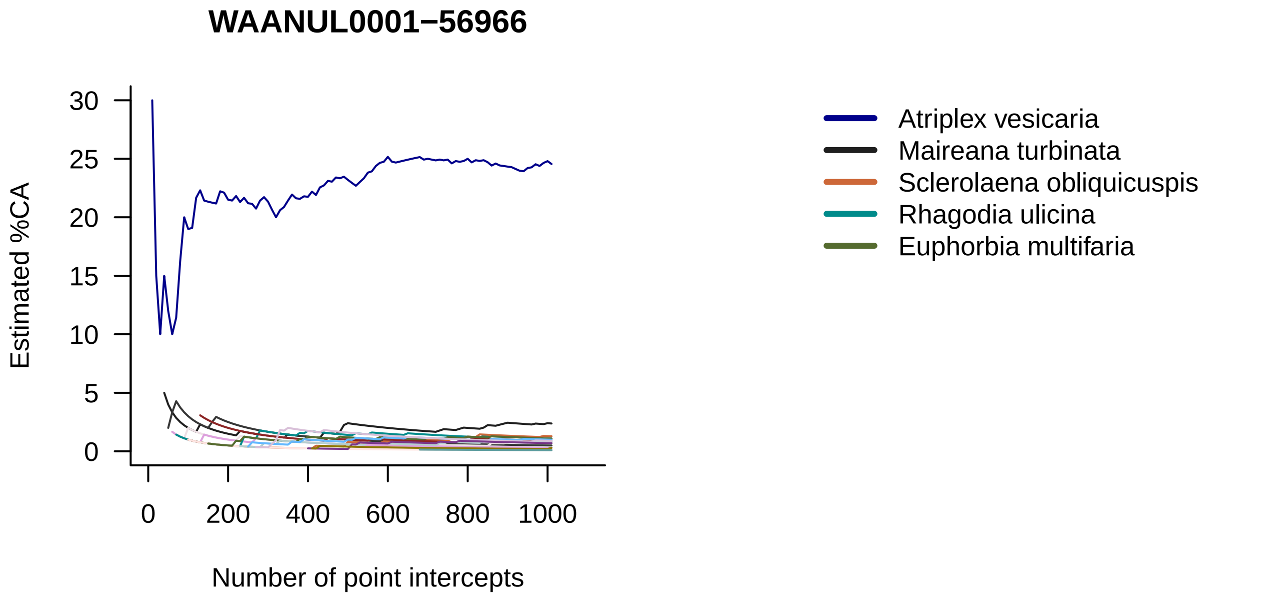
WAANUL0001−56966
Estimated %CA
30
25
20
15
10
5
0
0
200
400
600
800
1000
Number of point intercepts
Atriplex vesicaria
Maireana turbinata
Sclerolaena obliquicuspis
Rhagodia ulicina
Euphorbia multifaria

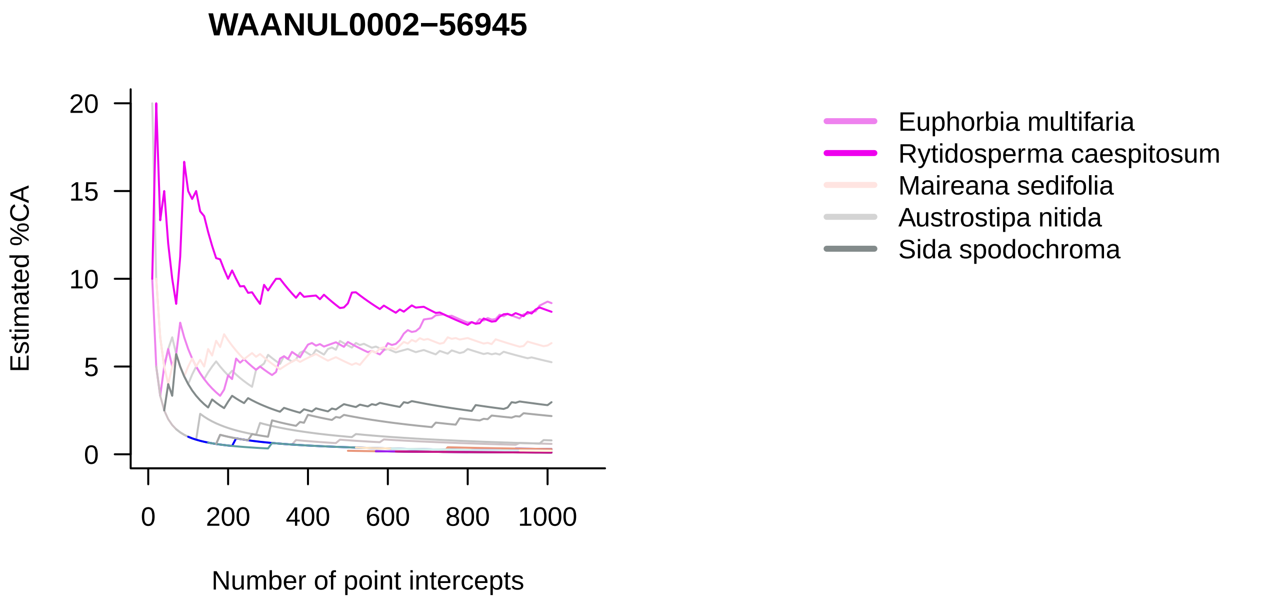
WAANUL0002−56945
Estimated %CA
0
5
10
15
20
0
200
400
600
800
1000
Number of point intercepts
Euphorbia multifaria
Rytidosperma caespitosum
Maireana sedifolia
Austrostipa nitida
Sida spodochroma

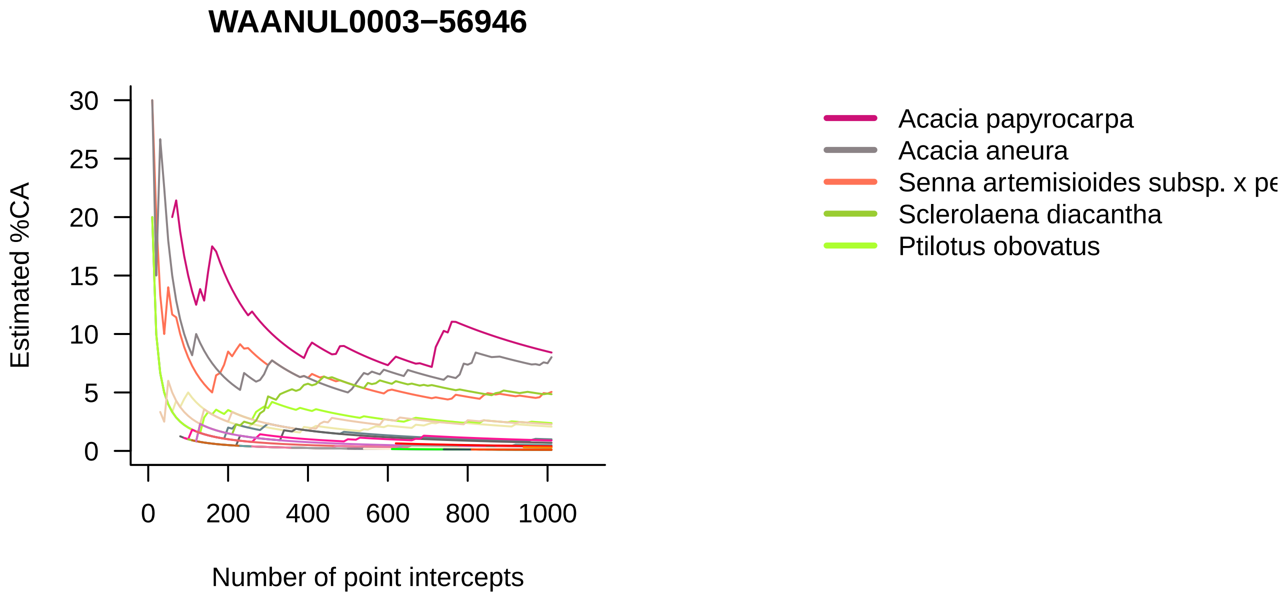
WAANUL0003−56946
Estimated %CA
0
5
10
15
20
25
30
0
200
400
600
800
1000
Number of point intercepts
Acacia papyrocarpa
Acacia aneura
Senna artemisioides subsp. x pe
Sclerolaena diacantha
Ptilotus obovatus

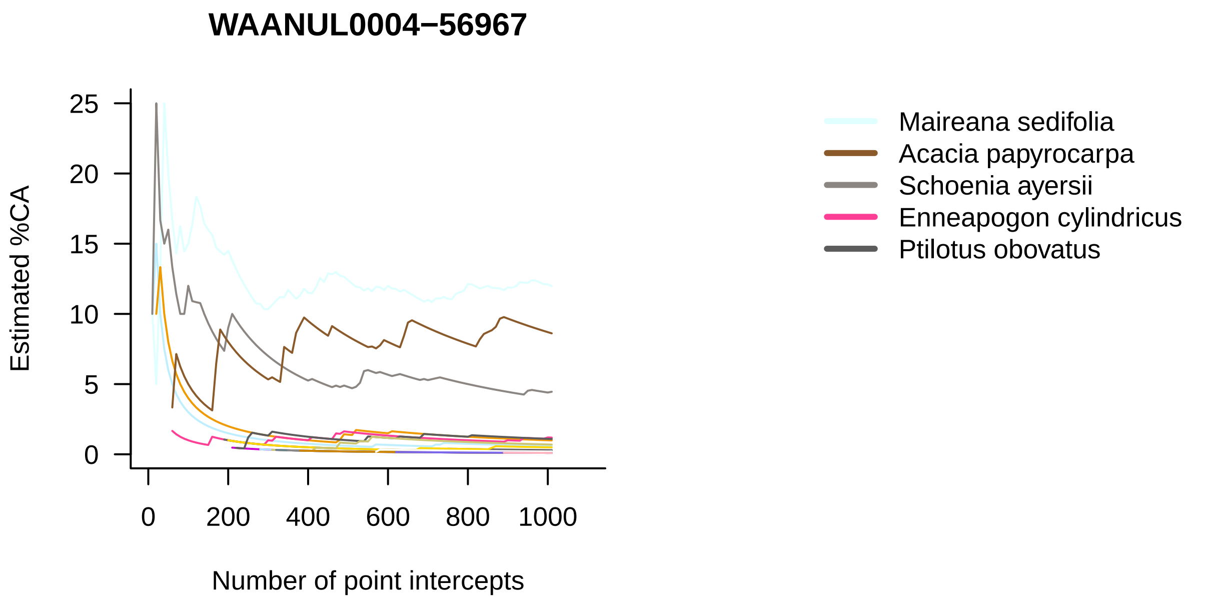
WAANUL0004−56967
Estimated %CA
25
20
15
10
5
0
0
200
400
600
800
1000
Number of point intercepts
Maireana sedifolia
Acacia papyrocarpa
Schoenia ayersii
Enneapogon cylindricus
Ptilotus obovatus

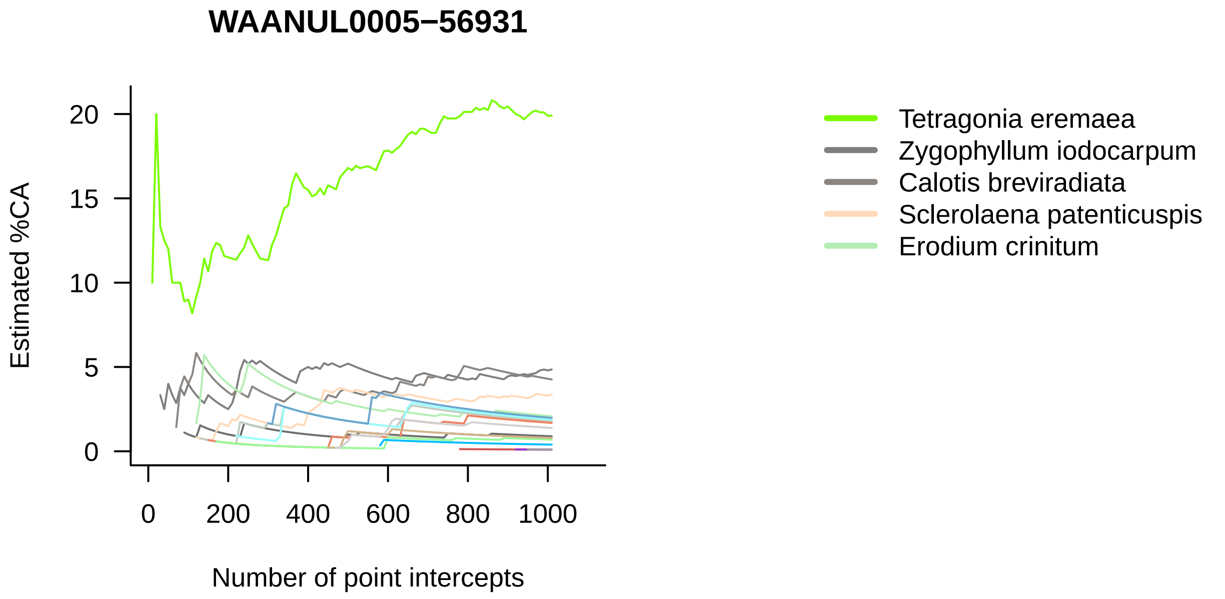
WAANUL0005−56931
Estimated %CA
20
15
10
5
0
0
200
400
600
800
1000
Number of point intercepts
Tetragonia eremaea
Zygophyllum iodocarpum
Calotis breviradiata
Sclerolaena patenticuspis
Erodium crinitum

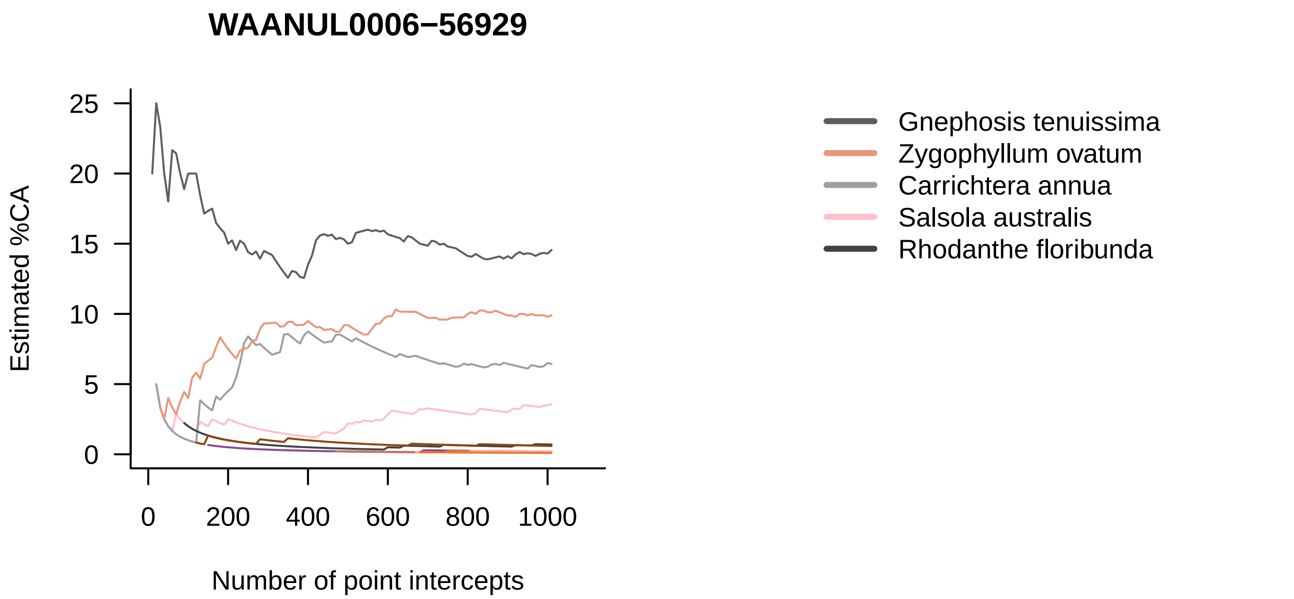
WAANUL0006−56929
Estimated %CA
Number of point intercepts
0
5
10
15
20
25
0
200
400
600
800
1000
Gnephosis tenuissima
Zygophyllum ovatum
Carrichtera annua
Salsola australis
Rhodanthe floribunda

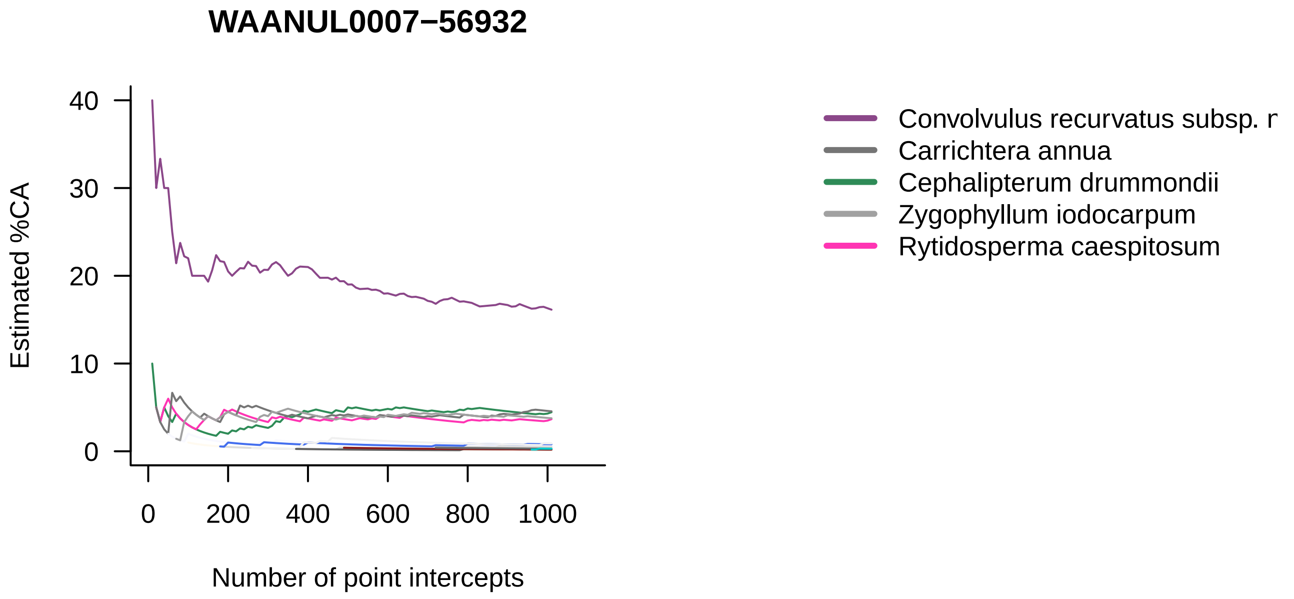
WAANUL0007−56932
Estimated %CA
Number of point intercepts
0
10
20
30
40
0
200
400
600
800
1000
Convolvulus recurvatus subsp. r
Carrichtera annua
Cephalipterum drummondii
Zygophyllum iodocarpum
Rytidosperma caespitosum

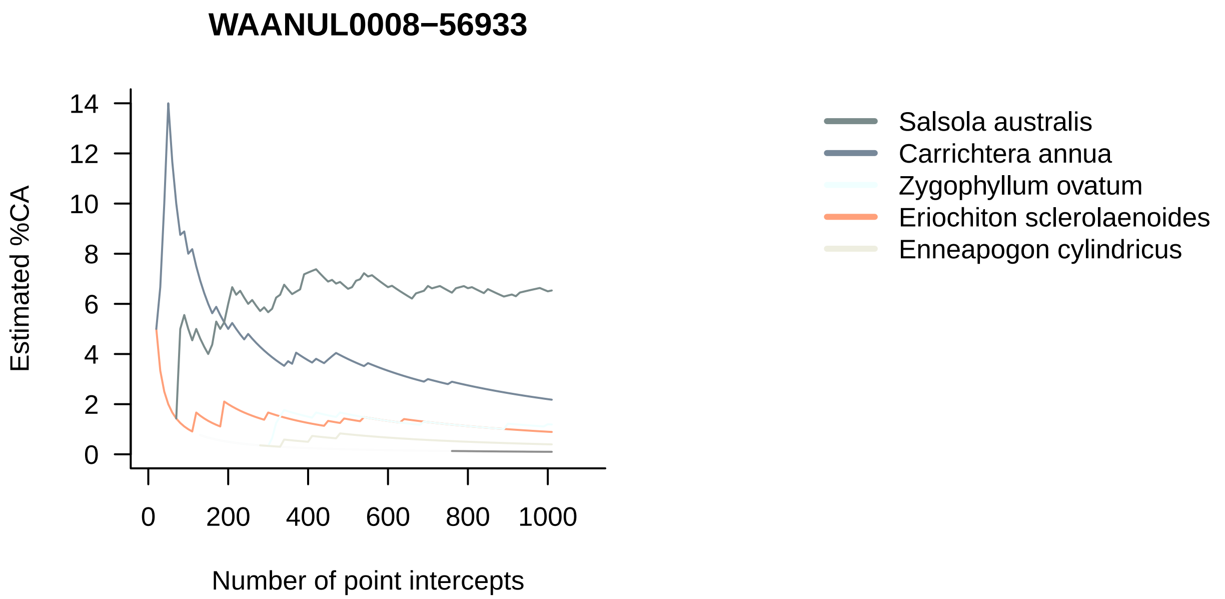
WAANUL0008−56933
Estimated %CA
0
2
4
6
8
10
12
14
0
200
400
600
800
1000
Number of point intercepts
Salsola australis
Carrichtera annua
Zygophyllum ovatum
Eriochiton sclerolaenoides
Enneapogon cylindricus

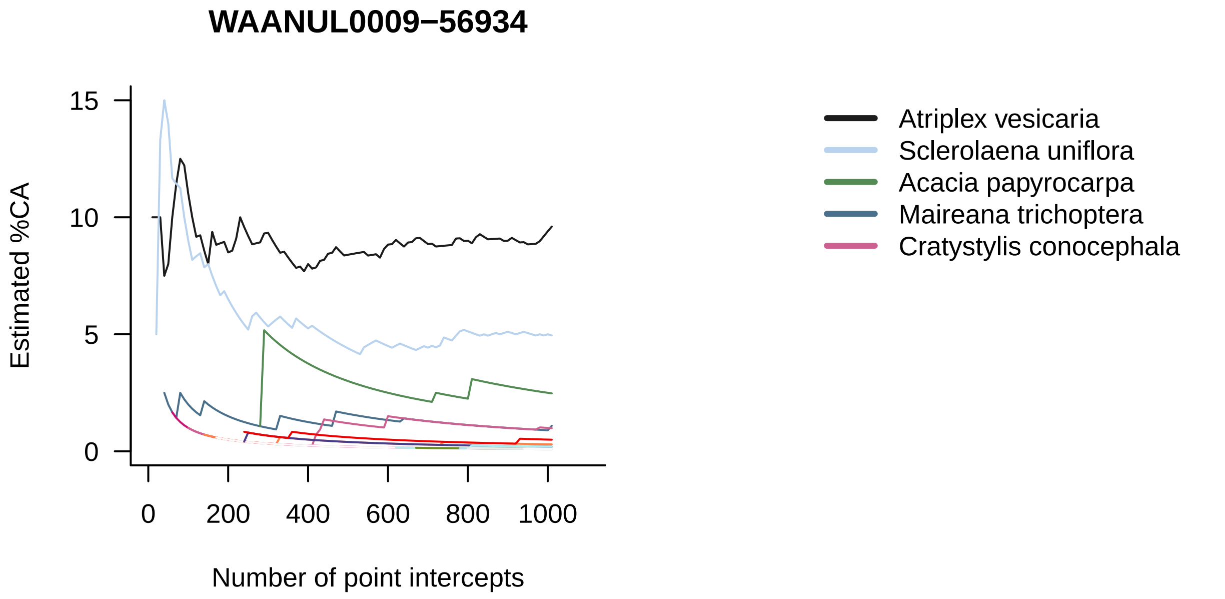
WAANUL0009−56934
Estimated %CA
0
5
10
15
0
200
400
600
800
1000
Number of point intercepts
Atriplex vesicaria
Sclerolaena uniflora
Acacia papyrocarpa
Maireana trichoptera
Cratystylis conocephala

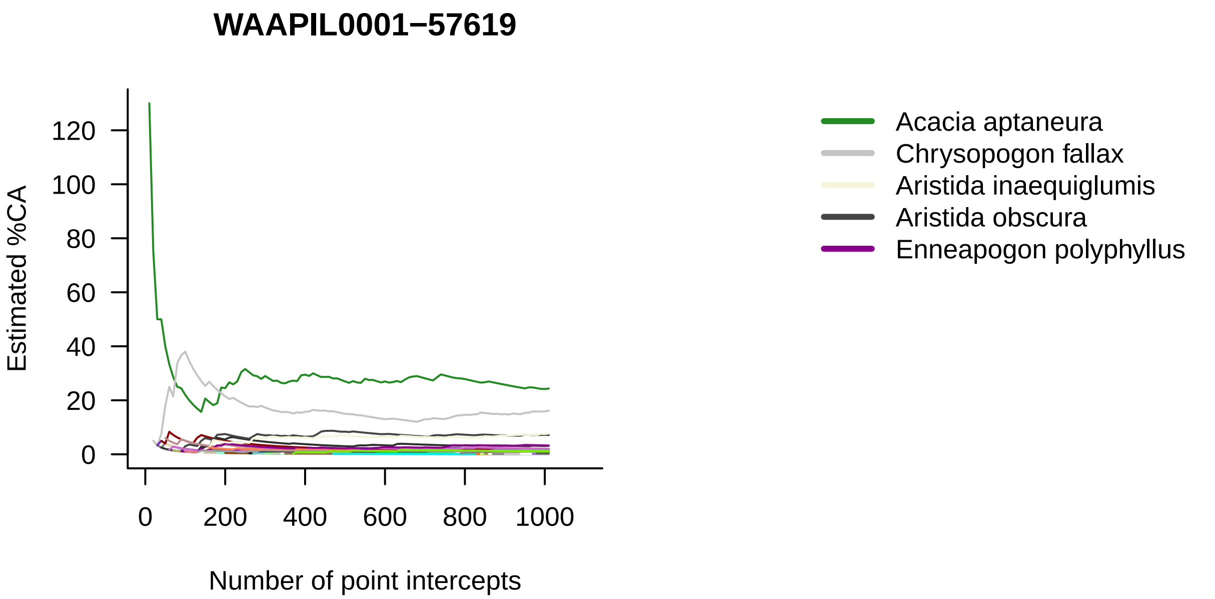
WAAPIL0001−57619
Estimated %CA
120
100
80
60
40
20
0
0
200
400
600
800
1000
Number of point intercepts
Acacia aptaneura
Chrysopogon fallax
Aristida inaequiglumis
Aristida obscura
Enneapogon polyphyllus

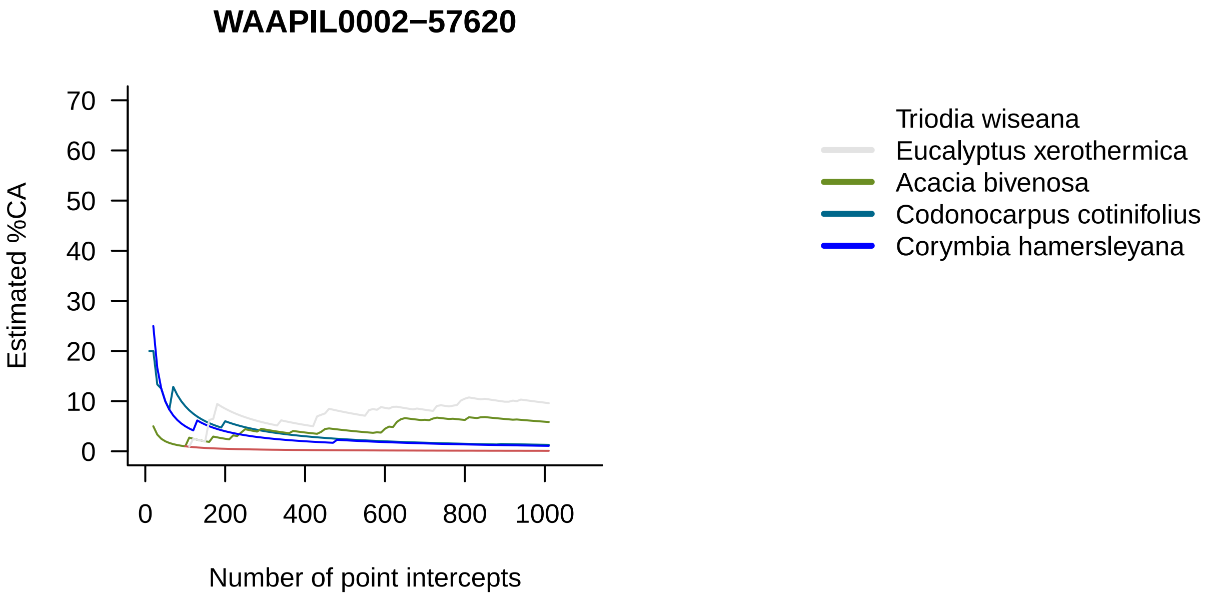
WAAPIL0002−57620
Estimated %CA
70
60
50
40
30
20
10
0
0
200
400
600
800
1000
Number of point intercepts
Triodia wiseana
Eucalyptus xerothermica
Acacia bivenosa
Codonocarpus cotinifolius
Corymbia hamersleyana

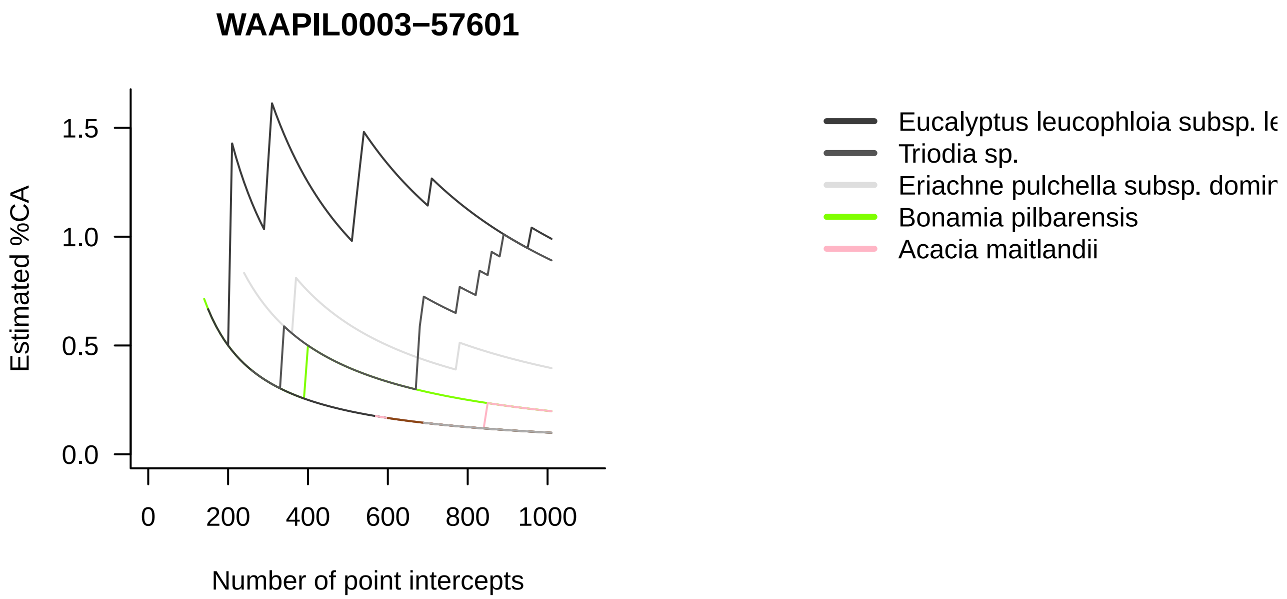
WAAPIL0003−57601
Estimated %CA
Number of point intercepts
0.0
0.5
1.0
1.5
0
200
400
600
800
1000
Eucalyptus leucophloia subsp. le
Triodia sp.
Eriachne pulchella subsp. domir
Bonamia pilbarensis
Acacia maitlandii

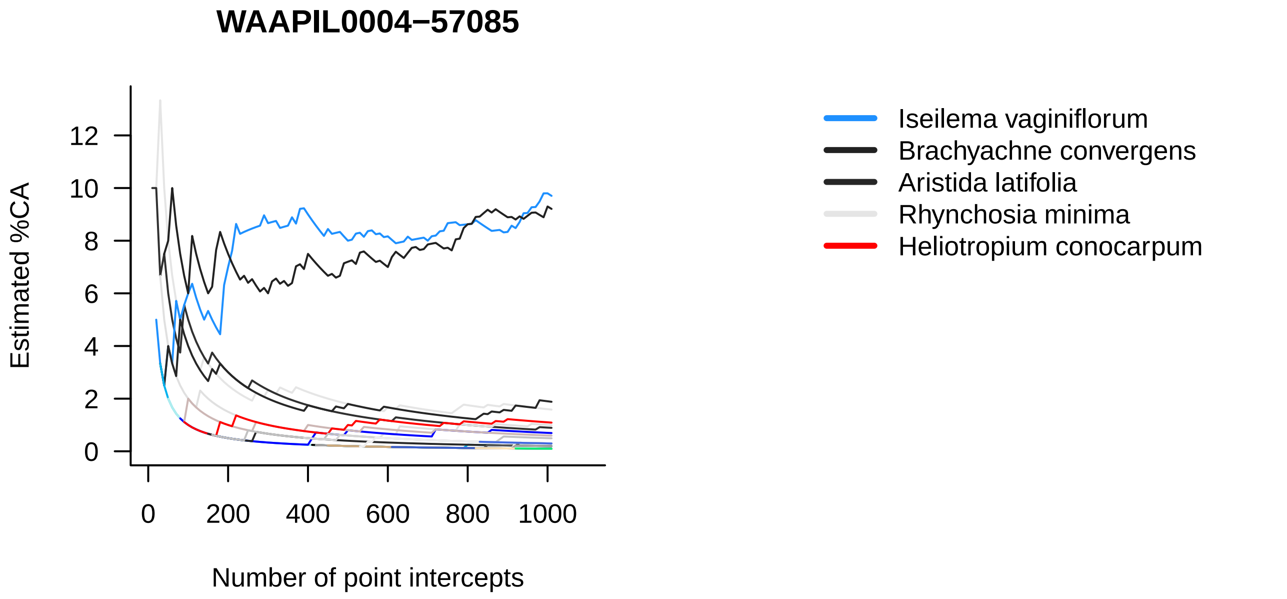
WAAPIL0004−57085
Estimated %CA
Number of point intercepts
0
2
4
6
8
10
12
0
200
400
600
800
1000
Iseilema vaginiflorum
Brachyachne convergens
Aristida latifolia
Rhynchosia minima
Heliotropium conocarpum

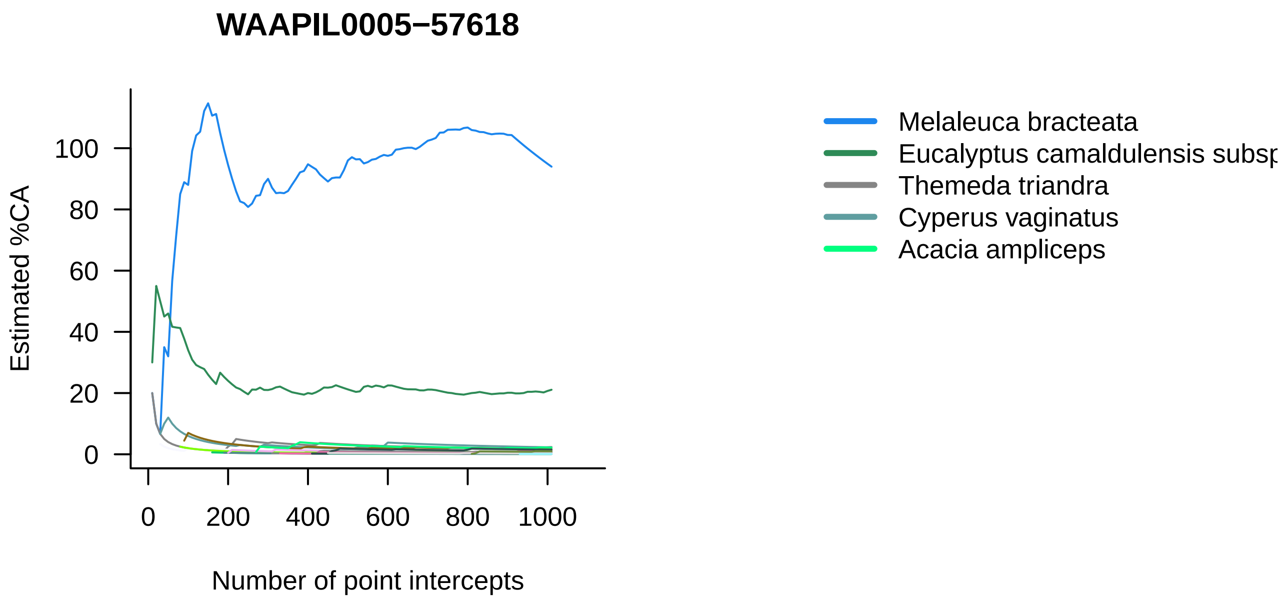

WAAPIL0005−57618
Estimated %CA
0
20
40
60
80
100
0
200
400
600
800
1000
Number of point intercepts
Melaleuca bracteata
Eucalyptus camaldulensis subsp
Themeda triandra
Cyperus vaginatus
Acacia ampliceps


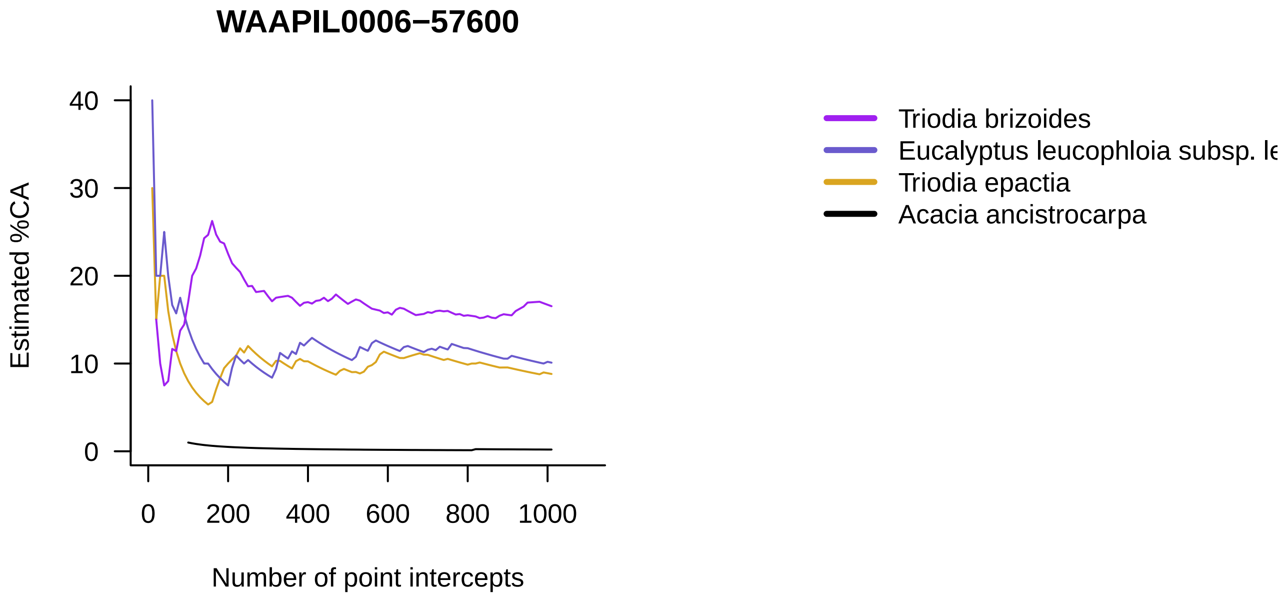

WAAPIL0006−57600
Estimated %CA
0
10
20
30
40
0
200
400
600
800
1000
Number of point intercepts
Triodia brizoides
Eucalyptus leucophloia subsp. le
Triodia epactia
Acacia ancistrocarpa

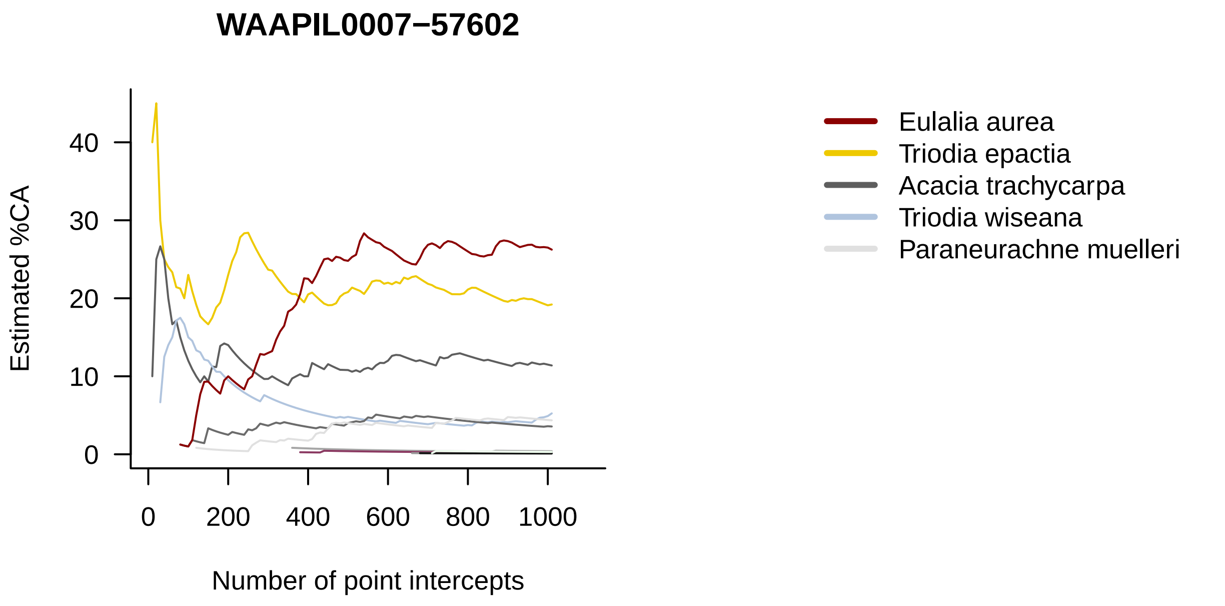
WAAPIL0007−57602
Estimated %CA
0
10
20
30
40
0
200
400
600
800
1000
Number of point intercepts
Eulalia aurea
Triodia epactia
Acacia trachycarpa
Triodia wiseana
Paraneurachne muelleri

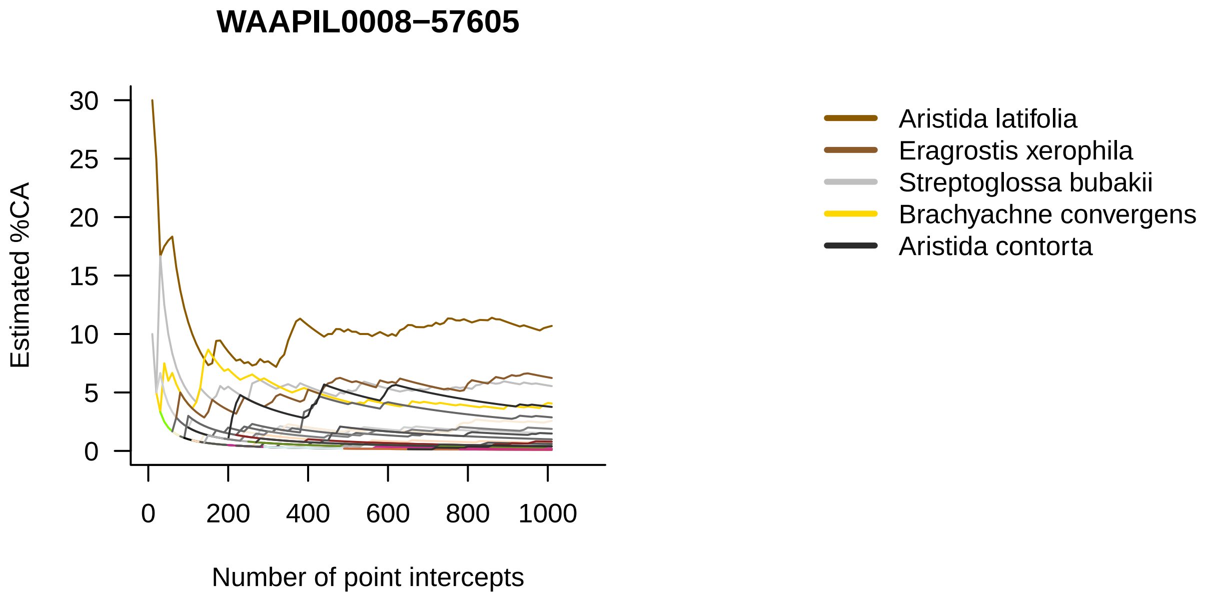
WAAPIL0008−57605
Estimated %CA
0
5
10
15
20
25
30
0
200
400
600
800
1000
Number of point intercepts
Aristida latifolia
Eragrostis xerophila
Streptoglossa bubakii
Brachyachne convergens
Aristida contorta

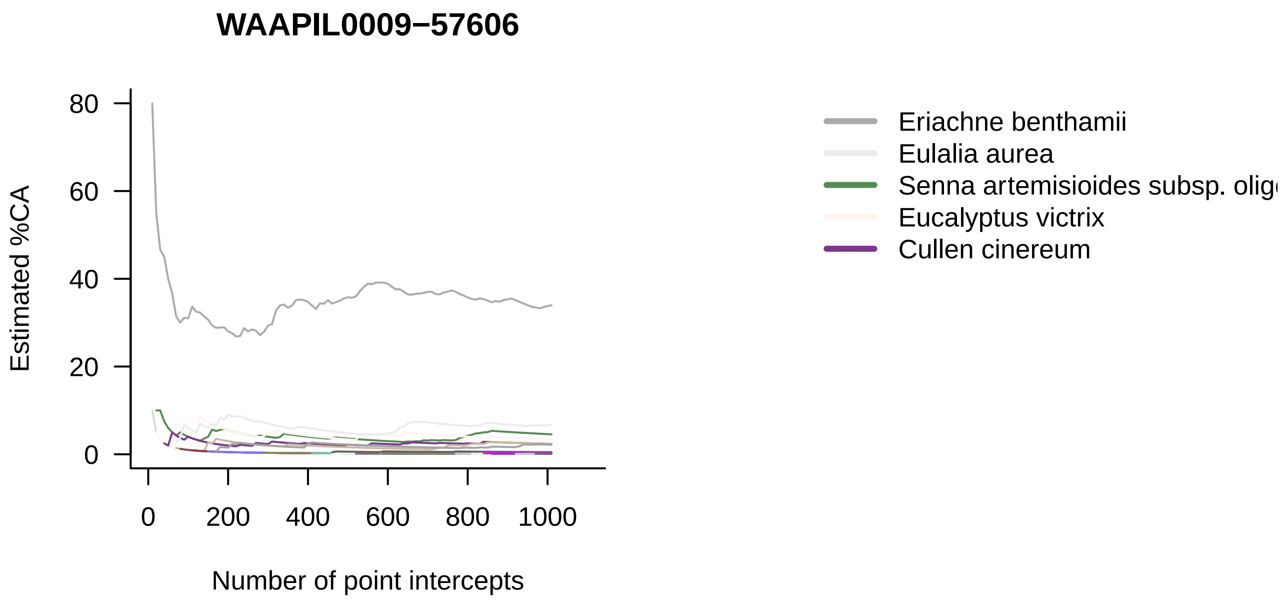
WAAPIL0009−57606
Estimated %CA
80
60
40
20
0
0
200
400
600
800
1000
Number of point intercepts
Eriachne benthamii
Eulalia aurea
Senna artemisioides subsp. olig
Eucalyptus victrix
Cullen cinereum

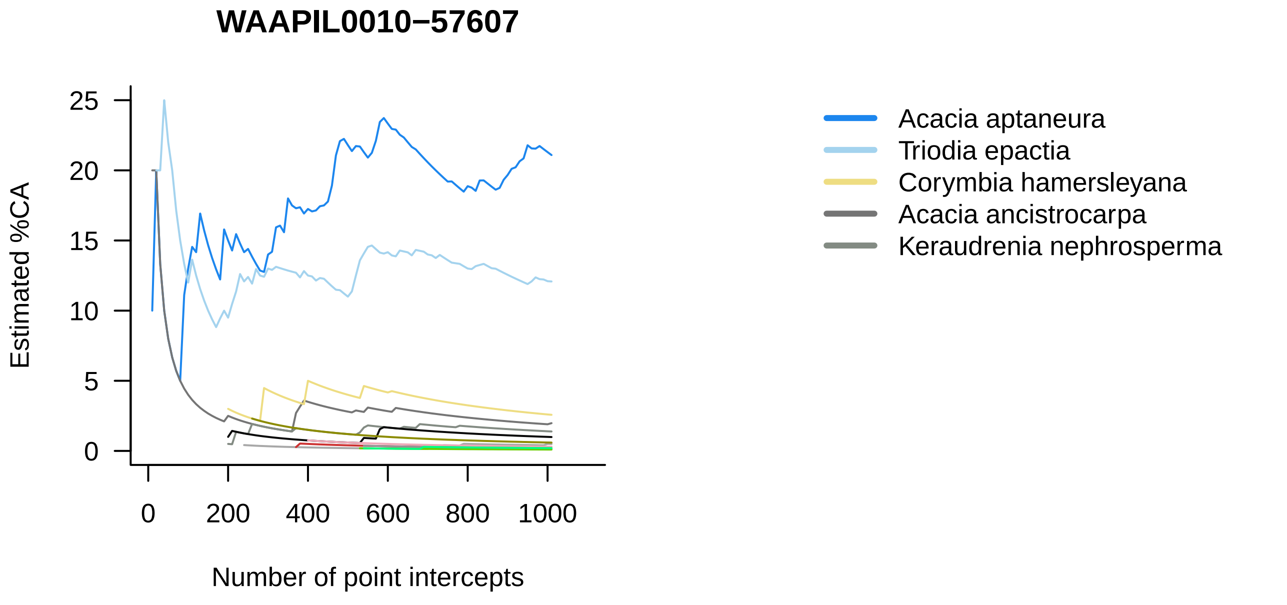
WAAPIL0010−57607
Estimated %CA
25
20
15
10
5
0
0
200
400
600
800
1000
Number of point intercepts
Acacia aptaneura
Triodia epactia
Corymbia hamersleyana
Acacia ancistrocarpa
Keraudrenia nephrosperma

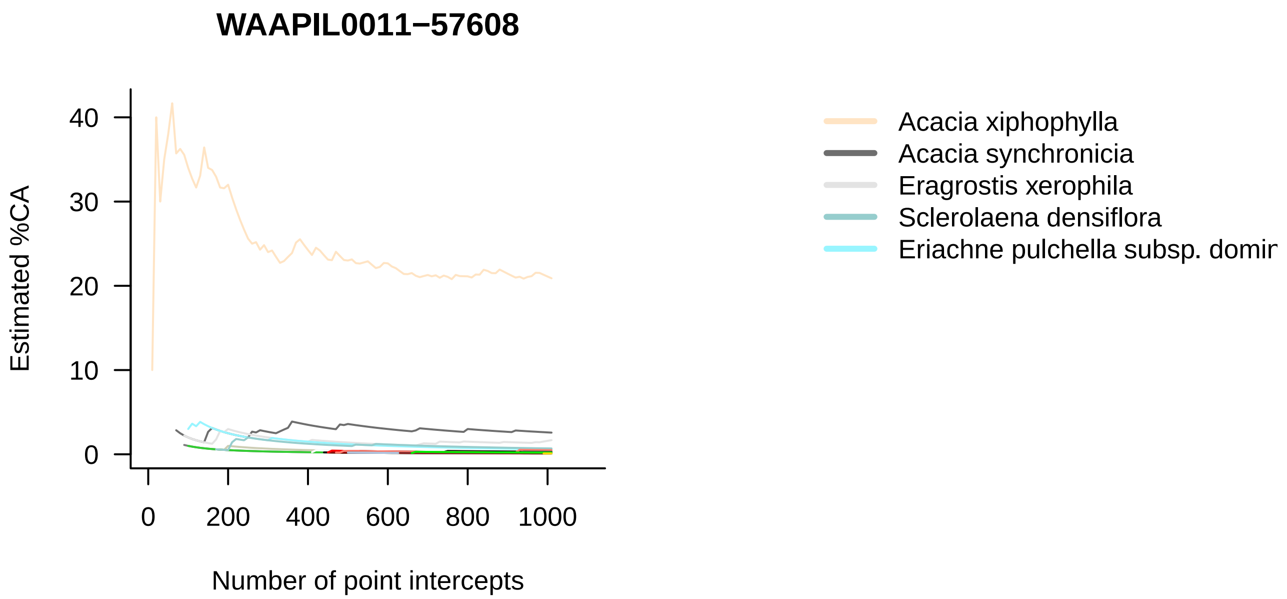
WAAPIL0011−57608
Estimated %CA
40
30
20
10
0
0
200
400
600
800
1000
Number of point intercepts
Acacia xiphophylla
Acacia synchronicia
Eragrostis xerophila
Sclerolaena densiflora
Eriachne pulchella subsp. domir

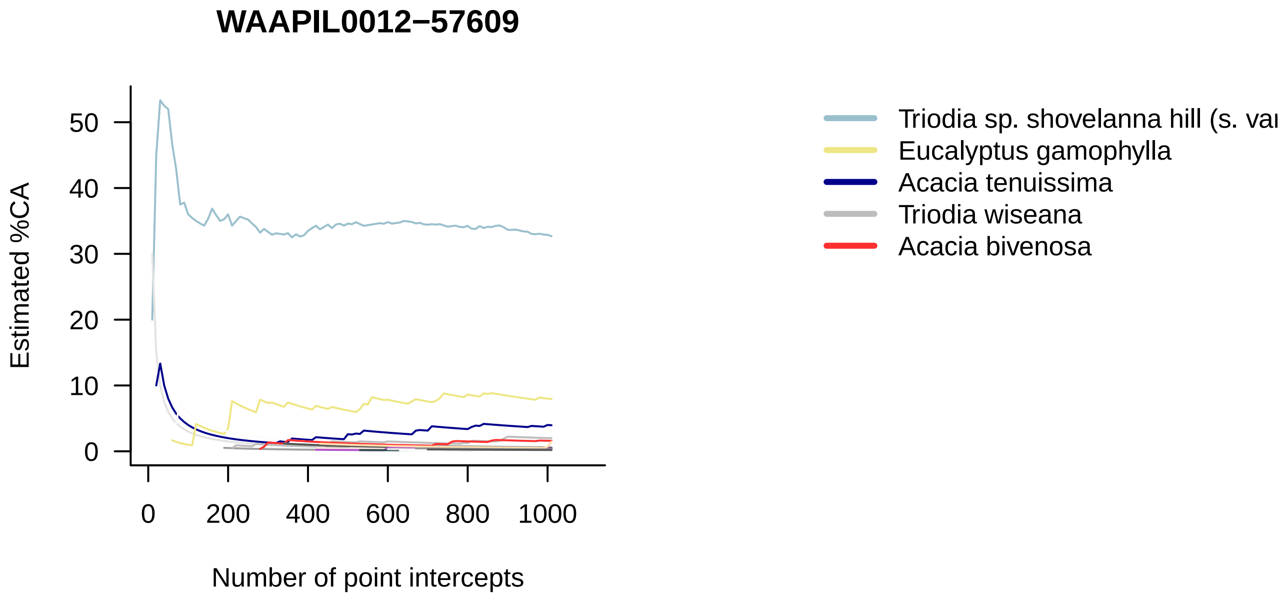
WAAPIL0012−57609
Estimated %CA
50
40
30
20
10
0
0
200
400
600
800
1000
Number of point intercepts
Triodia sp. shovelanna hill (s. vai
Eucalyptus gamophylla
Acacia tenuissima
Triodia wiseana
Acacia bivenosa

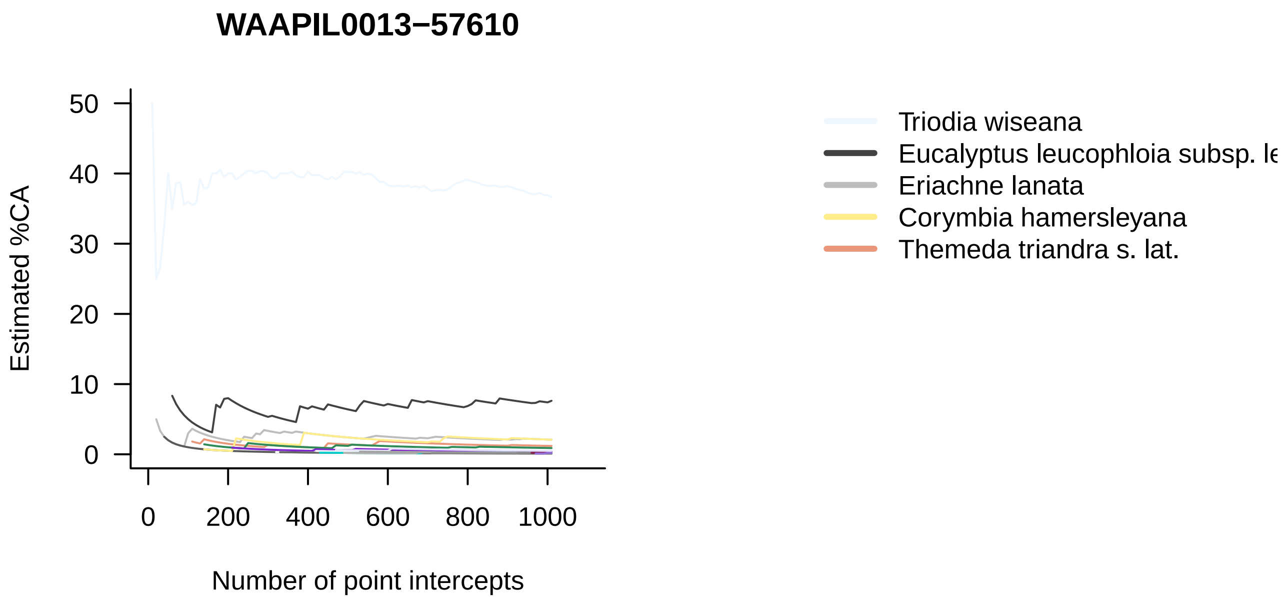

WAAPIL0013−57610
Estimated %CA
Number of point intercepts
Triodia wiseana
Eucalyptus leucophloia subsp. le
Eriachne lanata
Corymbia hamersleyana
Themeda triandra s. lat.


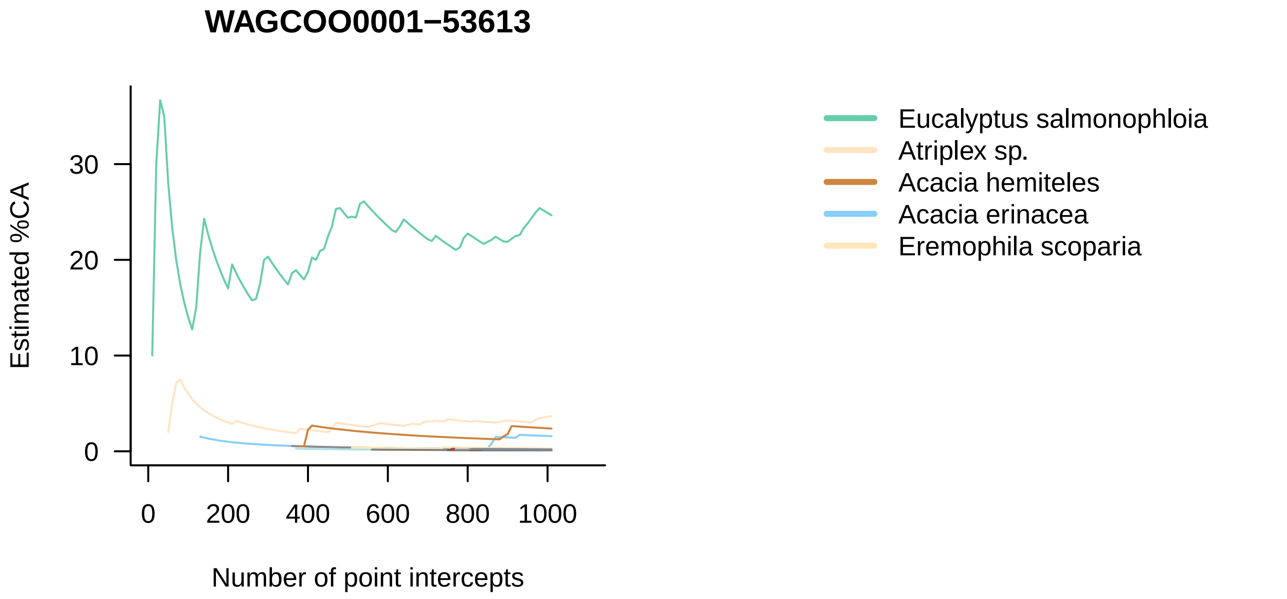

WAGCOO0001−53613
Estimated %CA
Number of point intercepts
Eucalyptus salmonophloia
Atriplex sp.
Acacia hemiteles
Acacia erinacea
Eremophila scoparia

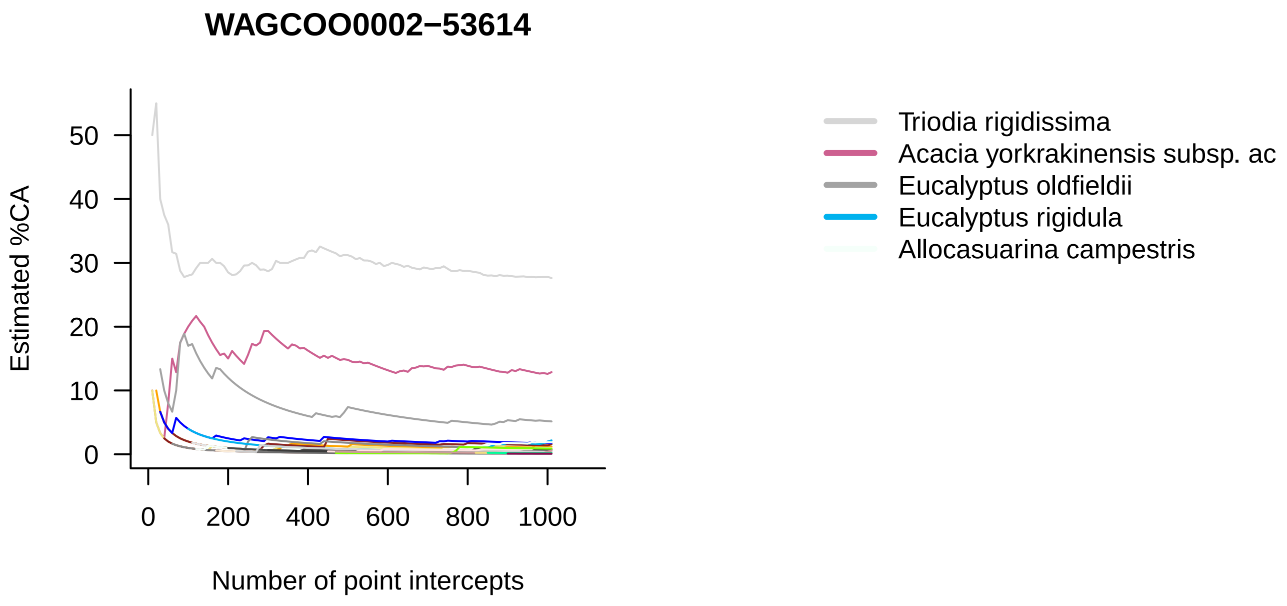
WAGCOO0002−53614
Estimated %CA
Number of point intercepts
Triodia rigidissima
Acacia yorkrakinensis subsp. ac
Eucalyptus oldfieldii
Eucalyptus rigidula
Allocasuarina campestris

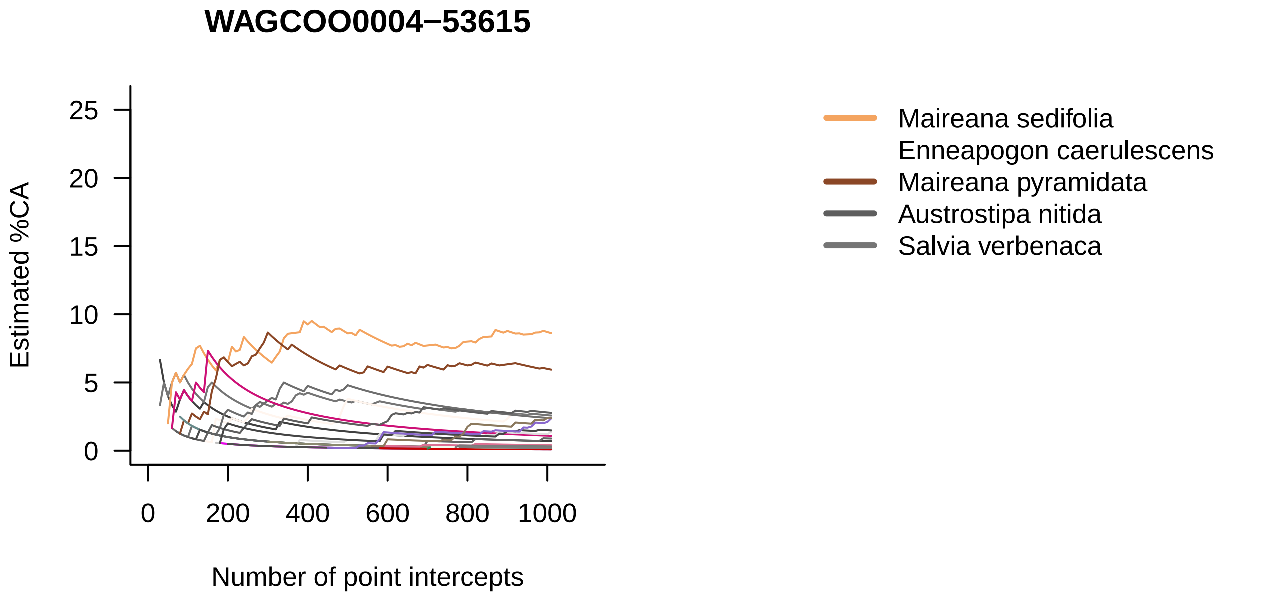
WAGCOO0004−53615
Estimated %CA
Number of point intercepts
Maireana sedifolia
Enneapogon caerulescens
Maireana pyramidata
Austrostipa nitida
Salvia verbenaca
