# Supplementary material for: Opportunities for Integrated Ecological Analysis across Inland Australia with Standardised Data from Ausplots Rangelands
Source: PLoS One. 2017 Jan 17;12(1):e0170137. doi: 10.1371/journal.pone.0170137 (PMC5241013; doi:10.1371/journal.pone.0170137)
Supplement: S5 Appendix — Plots of species abundance (percent cover) against species rank. (PDF) [file pone.0170137.s005.pdf]

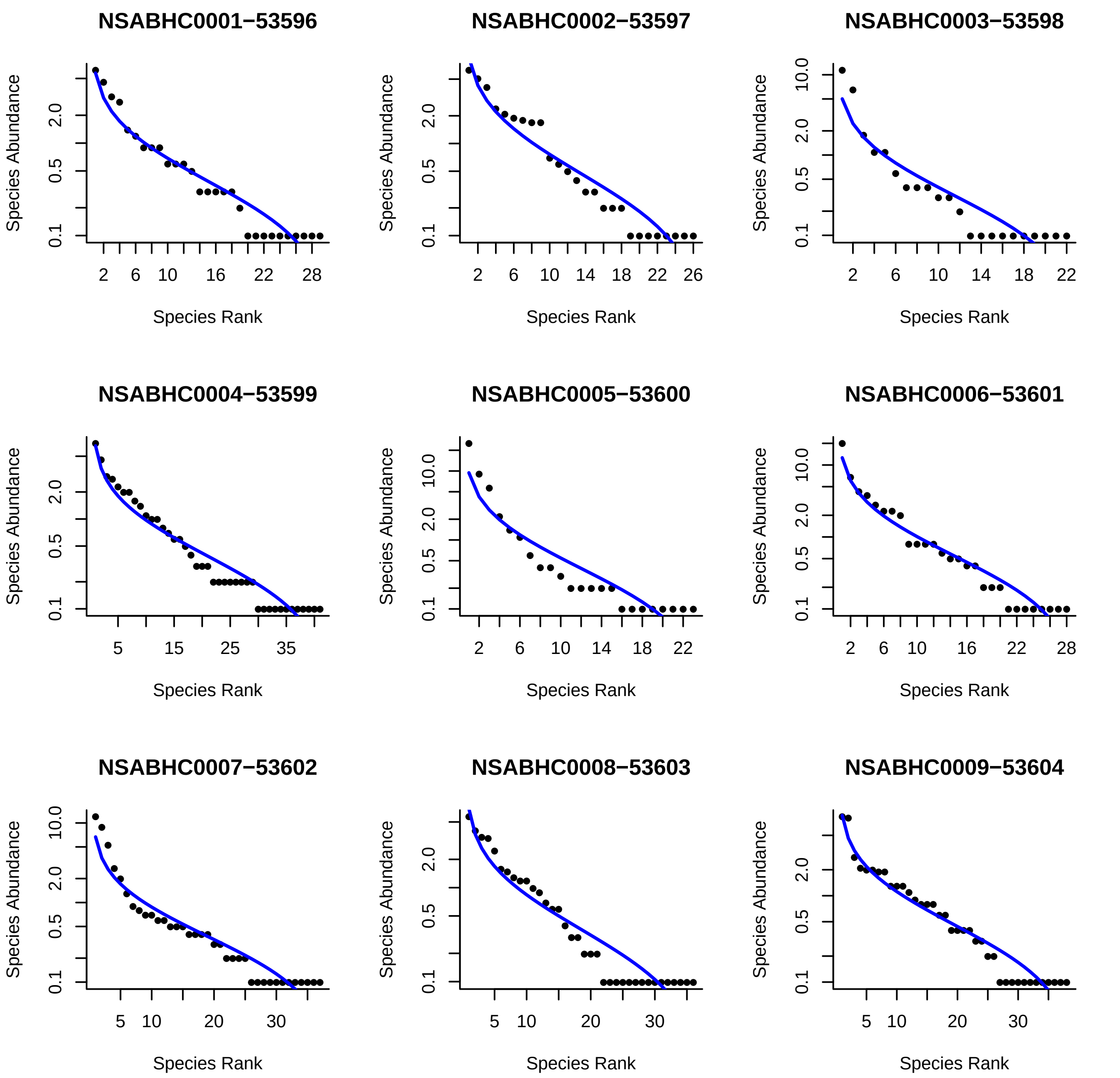
NSABHC0001−53596
NSABHC0002−53597
NSABHC0003−53598
NSABHC0004−53599
NSABHC0005−53600
NSABHC0006−53601
NSABHC0007−53602
NSABHC0008−53603
NSABHC0009−53604
Species Abundance
Species Rank
0.1
0.5
2.0
10.0
2
6
10
14
16
18
22
26
28
5
15
20
25
30
35

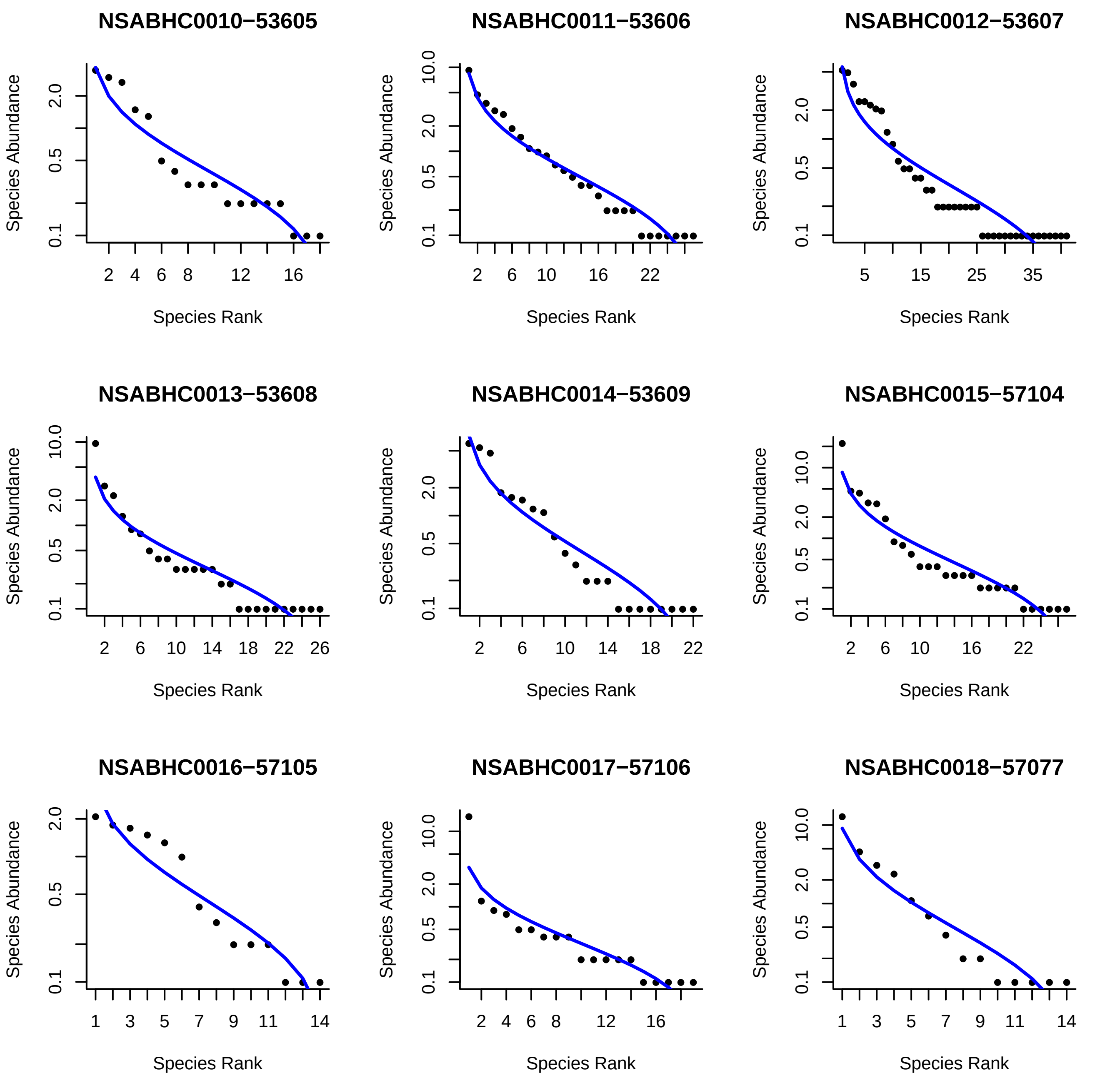
NSABHC0010−53605
Species Abundance
0.1
0.5
2.0
2
4
6
8
12
16
Species Rank
NSABHC0011−53606
Species Abundance
0.1
0.5
2.0
10.0
2
6
10
16
22
Species Rank
NSABHC0012−53607
Species Abundance
0.1
0.5
2.0
5
15
25
35
Species Rank
NSABHC0013−53608
Species Abundance
0.1
0.5
2.0
10.0
2
6
10
14
18
22
26
Species Rank
NSABHC0014−53609
Species Abundance
0.1
0.5
2.0
2
6
10
14
18
22
Species Rank
NSABHC0015−57104
Species Abundance
0.1
0.5
2.0
10.0
2
6
10
16
22
Species Rank
NSABHC0016−57105
Species Abundance
0.1
0.5
2.0
1
3
5
7
9
11
14
Species Rank
NSABHC0017−57106
Species Abundance
0.1
0.5
2.0
10.0
2
4
6
8
12
16
Species Rank
NSABHC0018−57077
Species Abundance
0.1
0.5
2.0
10.0
1
3
5
7
9
11
14
Species Rank

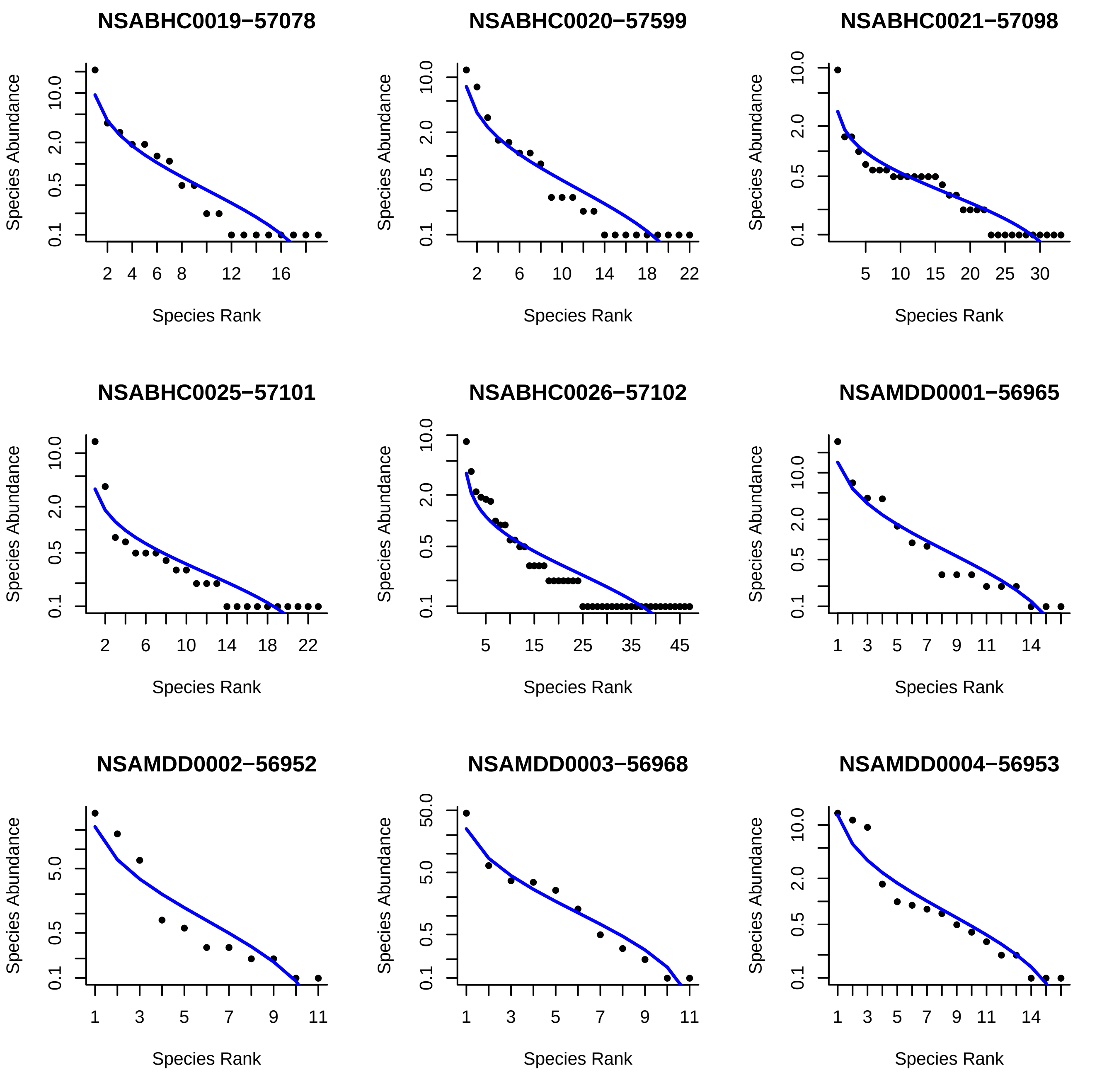

NSABHC0019−57078
Species Abundance
0.1
0.5
2.0
10.0
Species Rank
2
4
6
8
12
16
NSABHC0020−57599
Species Abundance
0.1
0.5
2.0
10.0
Species Rank
2
6
10
14
18
22
NSABHC0021−57098
Species Abundance
0.1
0.5
2.0
10.0
Species Rank
5
10
15
20
25
30
NSABHC0025−57101
Species Abundance
0.1
0.5
2.0
10.0
Species Rank
2
6
10
14
18
22
NSABHC0026−57102
Species Abundance
0.1
0.5
2.0
10.0
Species Rank
5
15
25
35
45
NSAMDD0001−56965
Species Abundance
0.1
0.5
2.0
10.0
Species Rank
1
3
5
7
9
11
14
NSAMDD0002−56952
Species Abundance
0.1
0.5
5.0
Species Rank
1
3
5
7
9
11
NSAMDD0003−56968
Species Abundance
0.1
0.5
5.0
50.0
Species Rank
1
3
5
7
9
11
NSAMDD0004−56953
Species Abundance
0.1
0.5
2.0
10.0
Species Rank
1
3
5
7
9
11
14

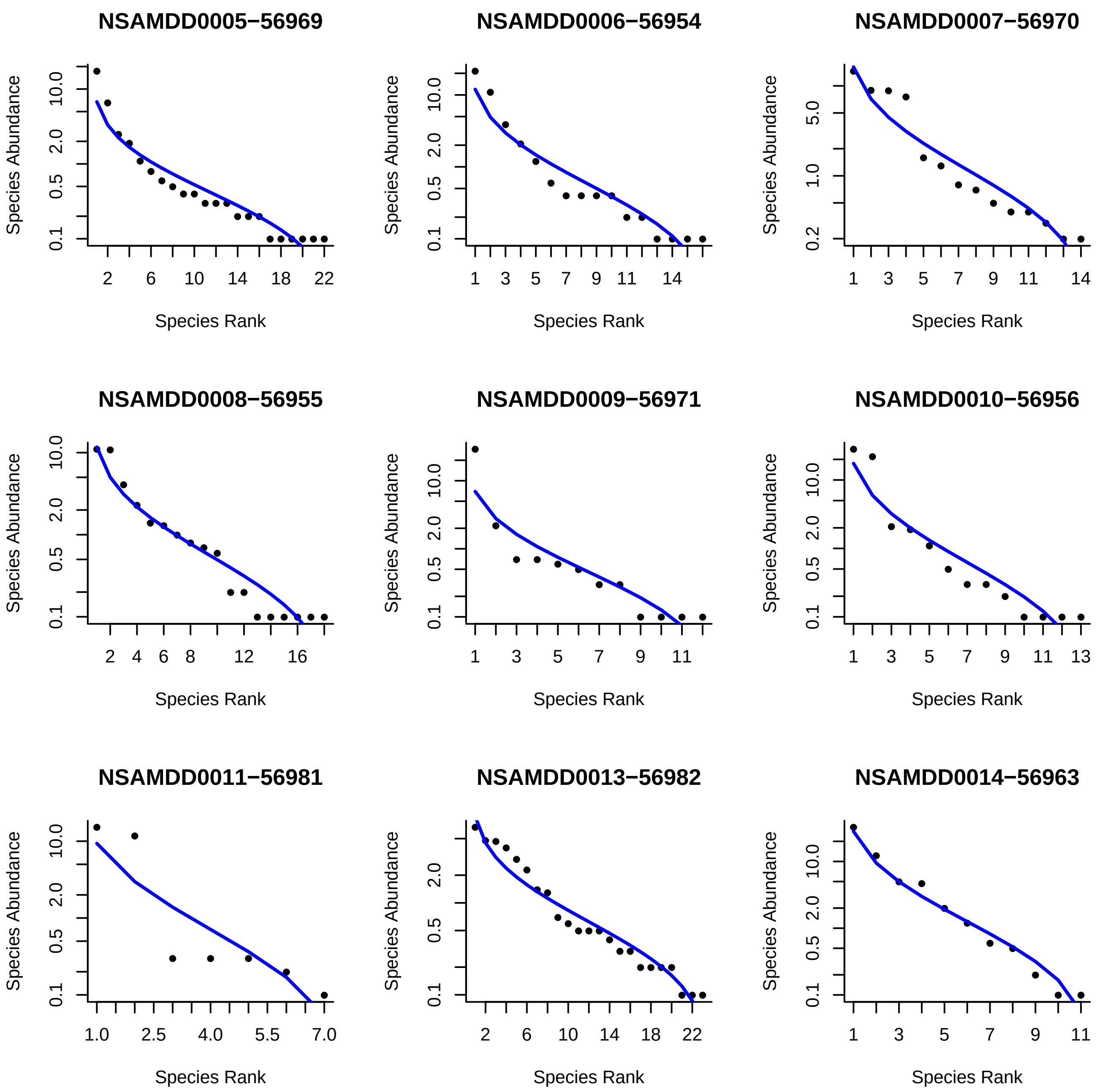
NSAMDD0005−56969
Species Abundance
0.1 0.5 2.0 10.0
2 6 10 14 18 22
Species Rank
NSAMDD0006−56954
Species Abundance
0.1 0.5 2.0 10.0
1 3 5 7 9 11 14
Species Rank
NSAMDD0007−56970
Species Abundance
0.2 1.0 5.0
1 3 5 7 9 11 14
Species Rank
NSAMDD0008−56955
Species Abundance
0.1 0.5 2.0 10.0
2 4 6 8 12 16
Species Rank
NSAMDD0009−56971
Species Abundance
0.1 0.5 2.0 10.0
1 3 5 7 9 11
Species Rank
NSAMDD0010−56956
Species Abundance
0.1 0.5 2.0 10.0
1 3 5 7 9 11 13
Species Rank
NSAMDD0011−56981
Species Abundance
0.1 0.5 2.0 10.0
1.0 2.5 4.0 5.5 7.0
Species Rank
NSAMDD0013−56982
Species Abundance
0.1 0.5 2.0
2 6 10 14 18 22
Species Rank
NSAMDD0014−56963
Species Abundance
0.1 0.5 2.0 10.0
1 3 5 7 9 11
Species Rank

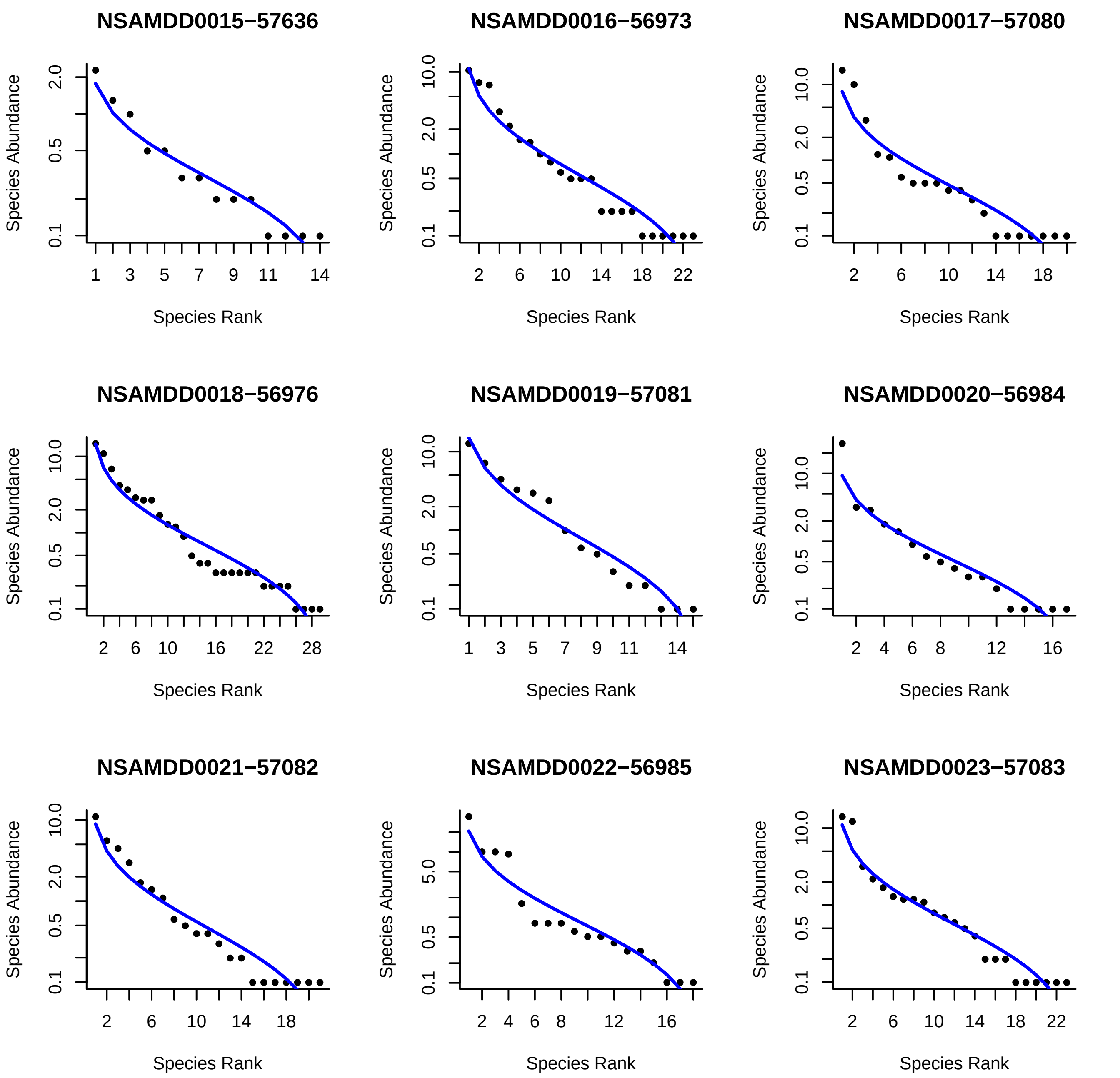
NSAMDD0015−57636
Species Abundance
0.1
0.5
2.0
1
3
5
7
9
11
14
Species Rank
NSAMDD0016−56973
Species Abundance
0.1
0.5
2.0
10.0
2
6
10
14
18
22
Species Rank
NSAMDD0017−57080
Species Abundance
0.1
0.5
2.0
10.0
2
6
10
14
18
Species Rank
NSAMDD0018−56976
Species Abundance
0.1
0.5
2.0
10.0
2
6
10
16
22
28
Species Rank
NSAMDD0019−57081
Species Abundance
0.1
0.5
2.0
10.0
1
3
5
7
9
11
14
Species Rank
NSAMDD0020−56984
Species Abundance
0.1
0.5
2.0
10.0
2
4
6
8
12
16
Species Rank
NSAMDD0021−57082
Species Abundance
0.1
0.5
2.0
10.0
2
6
10
14
18
Species Rank
NSAMDD0022−56985
Species Abundance
0.1
0.5
5.0
2
4
6
8
12
16
Species Rank
NSAMDD0023−57083
Species Abundance
0.1
0.5
2.0
10.0
2
6
10
14
18
22
Species Rank

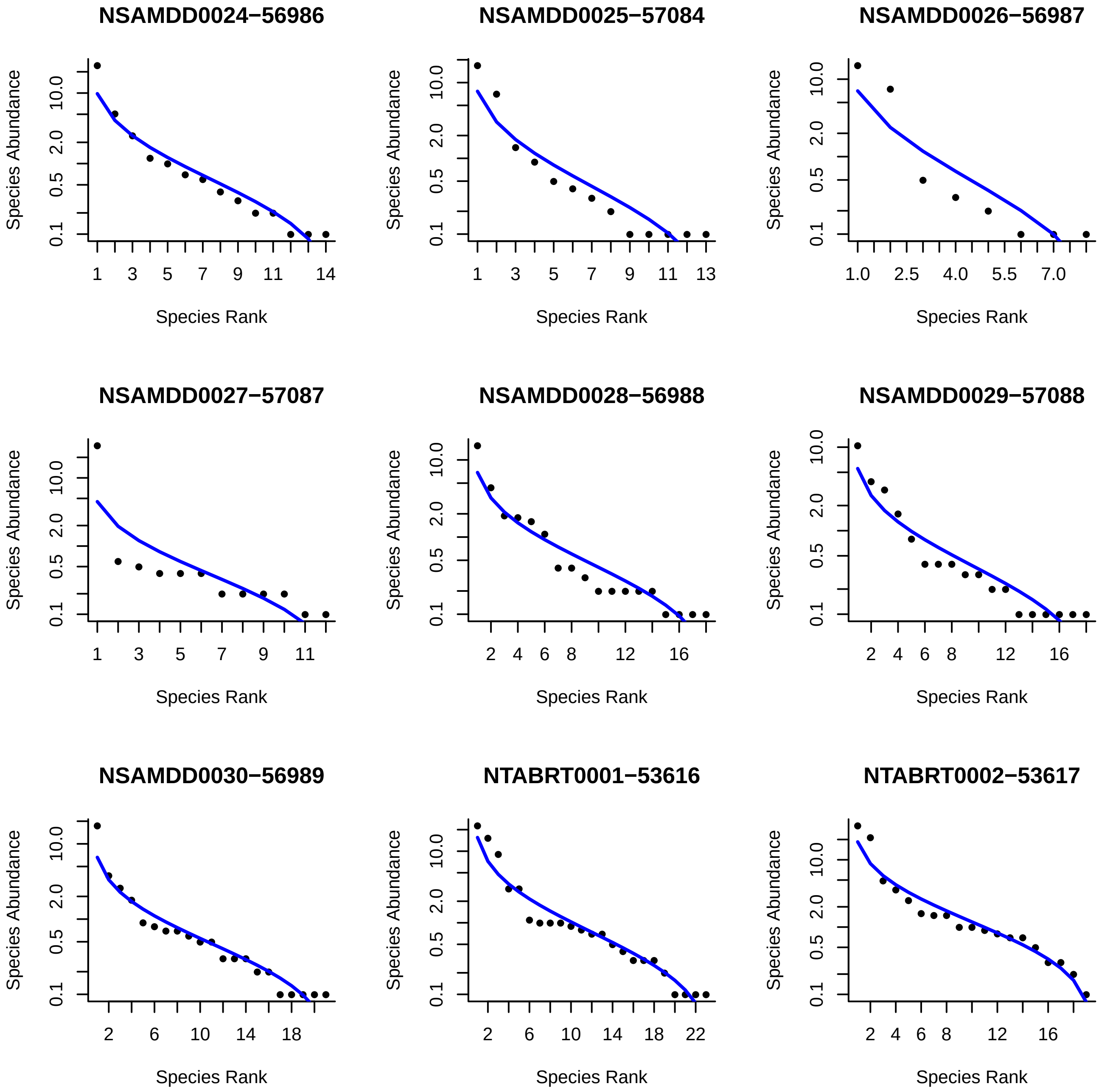
NSAMDD0024−56986
NSAMDD0025−57084
NSAMDD0026−56987
NSAMDD0027−57087
NSAMDD0028−56988
NSAMDD0029−57088
NSAMDD0030−56989
NTABRT0001−53616
NTABRT0002−53617
Species Abundance
Species Rank
0.1
0.5
2.0
10.0

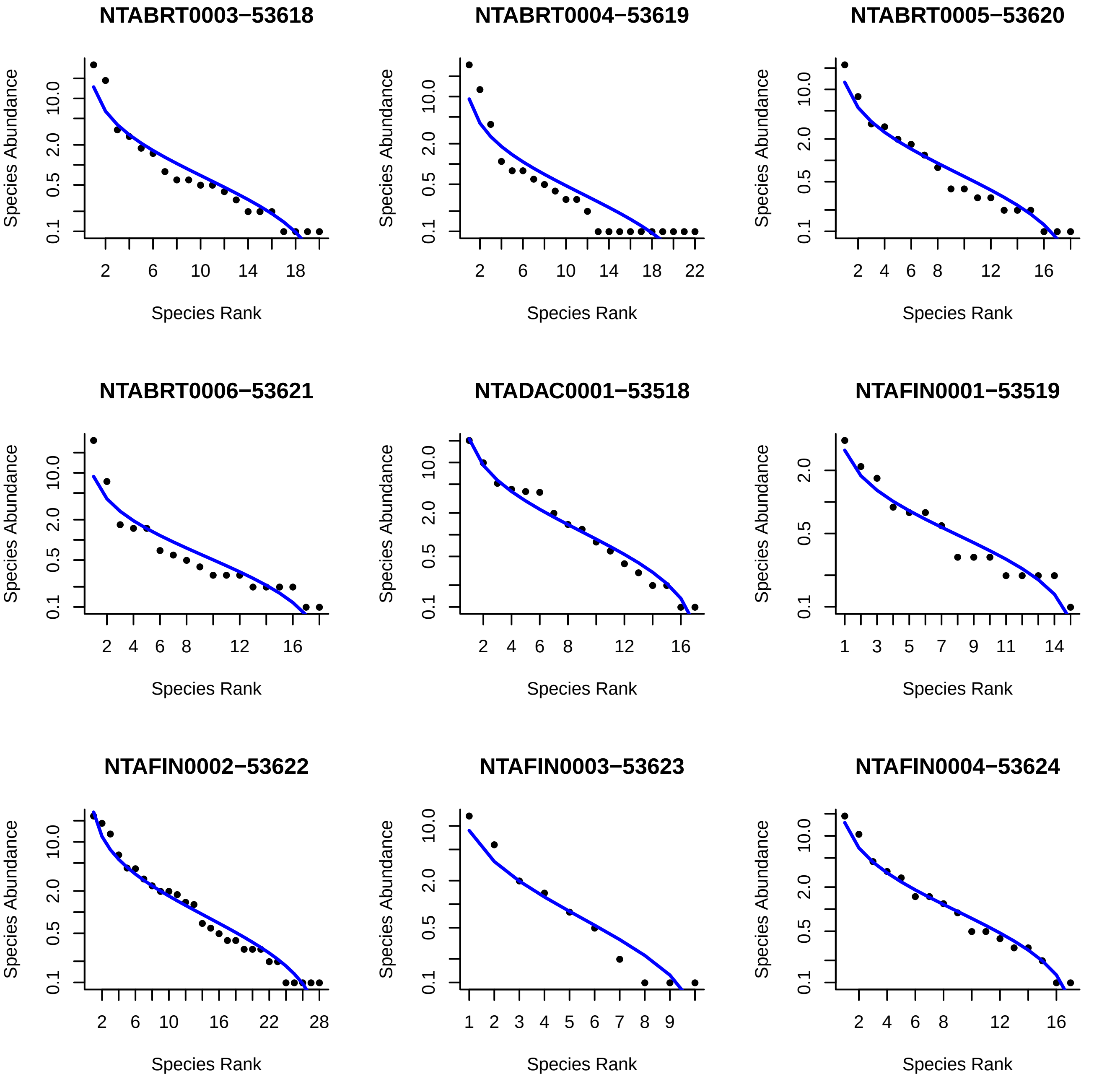
NTABRT0003−53618
NTABRT0004−53619
NTABRT0005−53620
NTABRT0006−53621
NTADAC0001−53518
NTAFIN0001−53519
NTAFIN0002−53622
NTAFIN0003−53623
NTAFIN0004−53624
Species Abundance
Species Rank
0.1
0.5
2.0
10.0
2
6
10
14
18
22
1
3
5
7
9
11
4
8
12
16
28

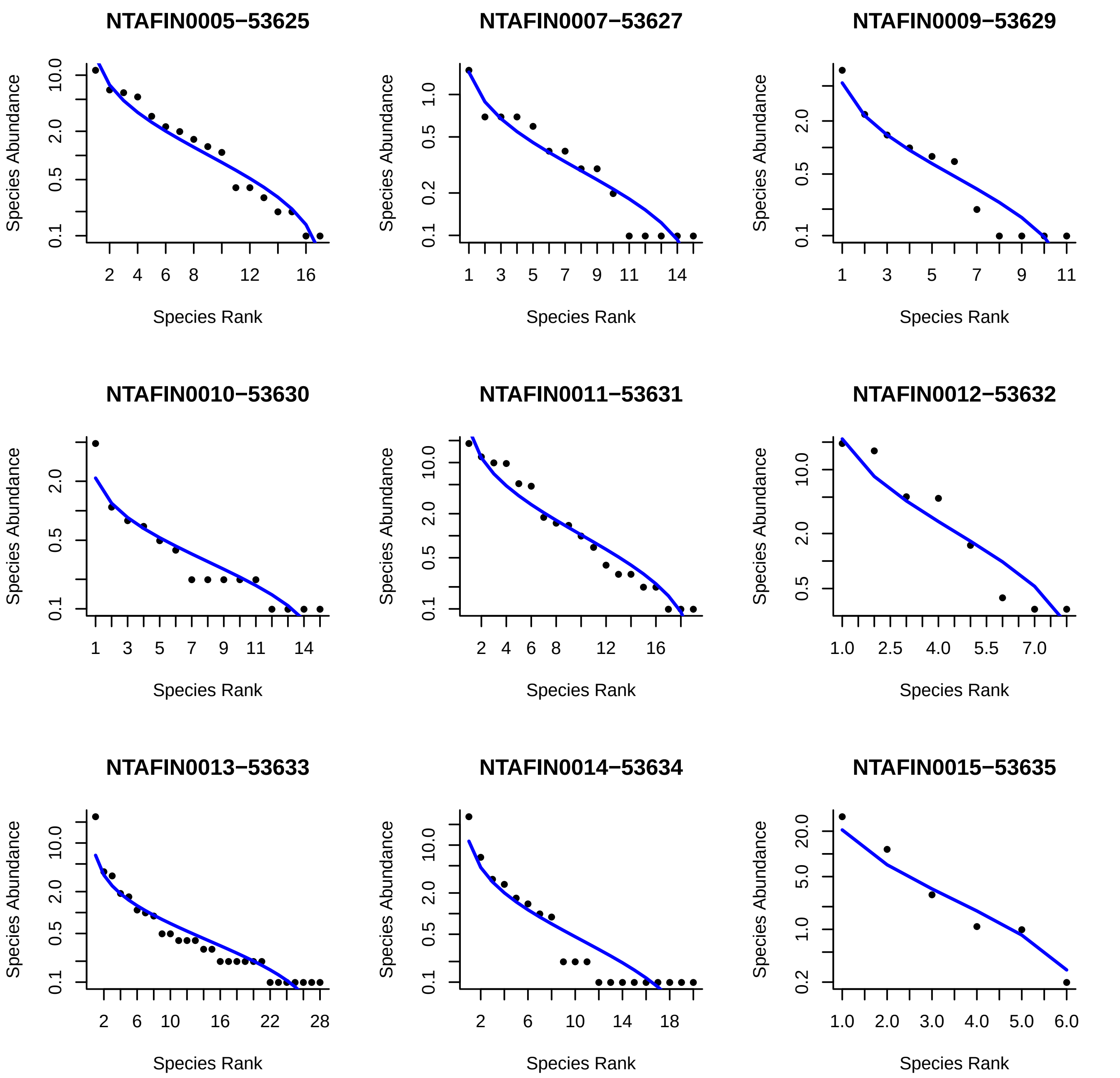
NTAFIN0005−53625
NTAFIN0007−53627
NTAFIN0009−53629
NTAFIN0010−53630
NTAFIN0011−53631
NTAFIN0012−53632
NTAFIN0013−53633
NTAFIN0014−53634
NTAFIN0015−53635
Species Abundance
Species Rank

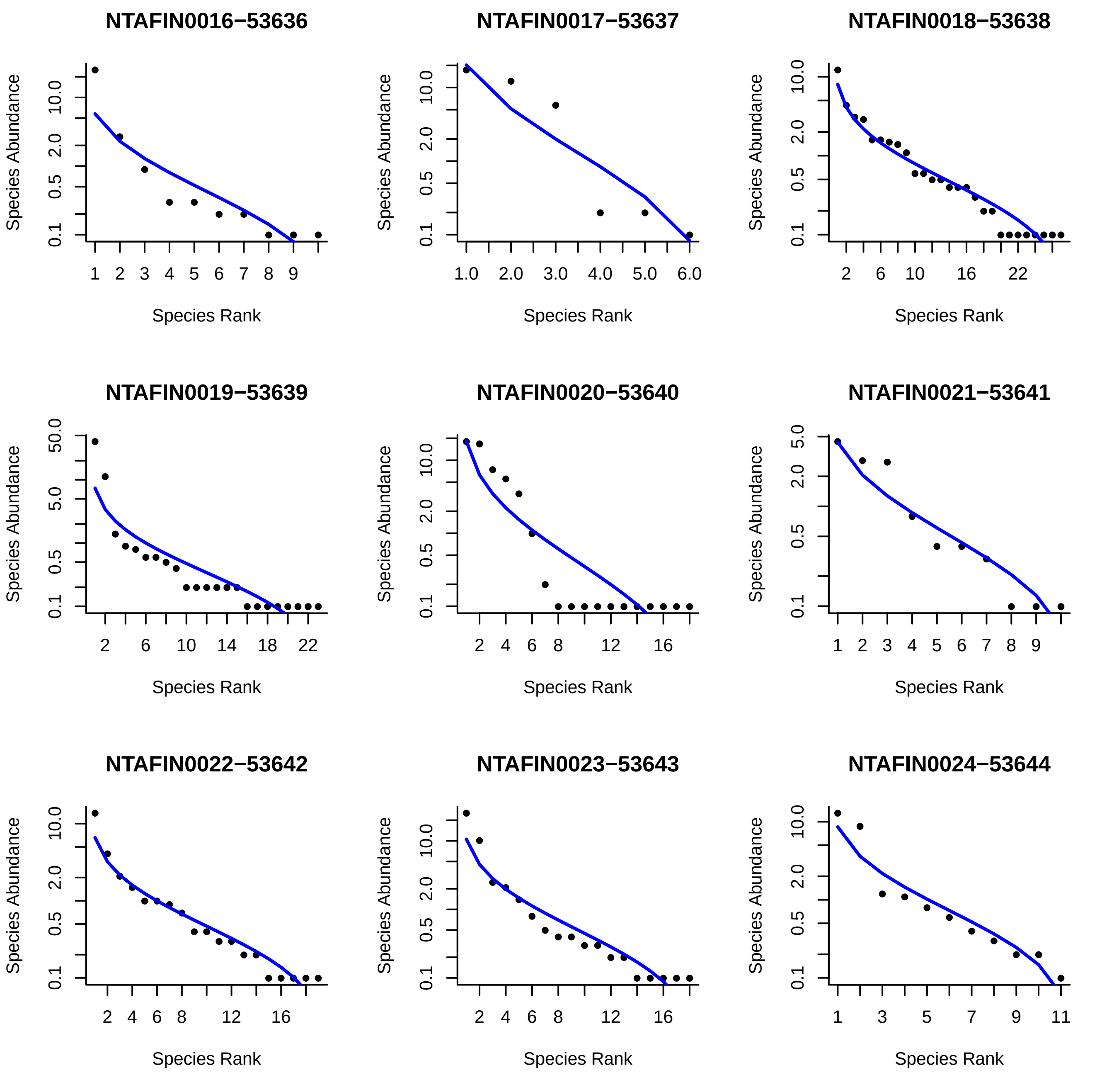
NTAFIN0016−53636
Species Abundance
0.1 0.5 2.0 10.0
1 2 3 4 5 6 7 8 9
Species Rank
NTAFIN0017−53637
Species Abundance
0.1 0.5 2.0 10.0
1.0 2.0 3.0 4.0 5.0 6.0
Species Rank
NTAFIN0018−53638
Species Abundance
0.1 0.5 2.0 10.0
2 6 10 16 22
Species Rank
NTAFIN0019−53639
Species Abundance
0.1 0.5 5.0 50.0
2 6 10 14 18 22
Species Rank
NTAFIN0020−53640
Species Abundance
0.1 0.5 2.0 10.0
2 4 6 8 12 16
Species Rank
NTAFIN0021−53641
Species Abundance
0.1 0.5 2.0 5.0
1 2 3 4 5 6 7 8 9
Species Rank
NTAFIN0022−53642
Species Abundance
0.1 0.5 2.0 10.0
2 4 6 8 12 16
Species Rank
NTAFIN0023−53643
Species Abundance
0.1 0.5 2.0 10.0
2 4 6 8 12 16
Species Rank
NTAFIN0024−53644
Species Abundance
0.1 0.5 2.0 10.0
1 3 5 7 9 11
Species Rank

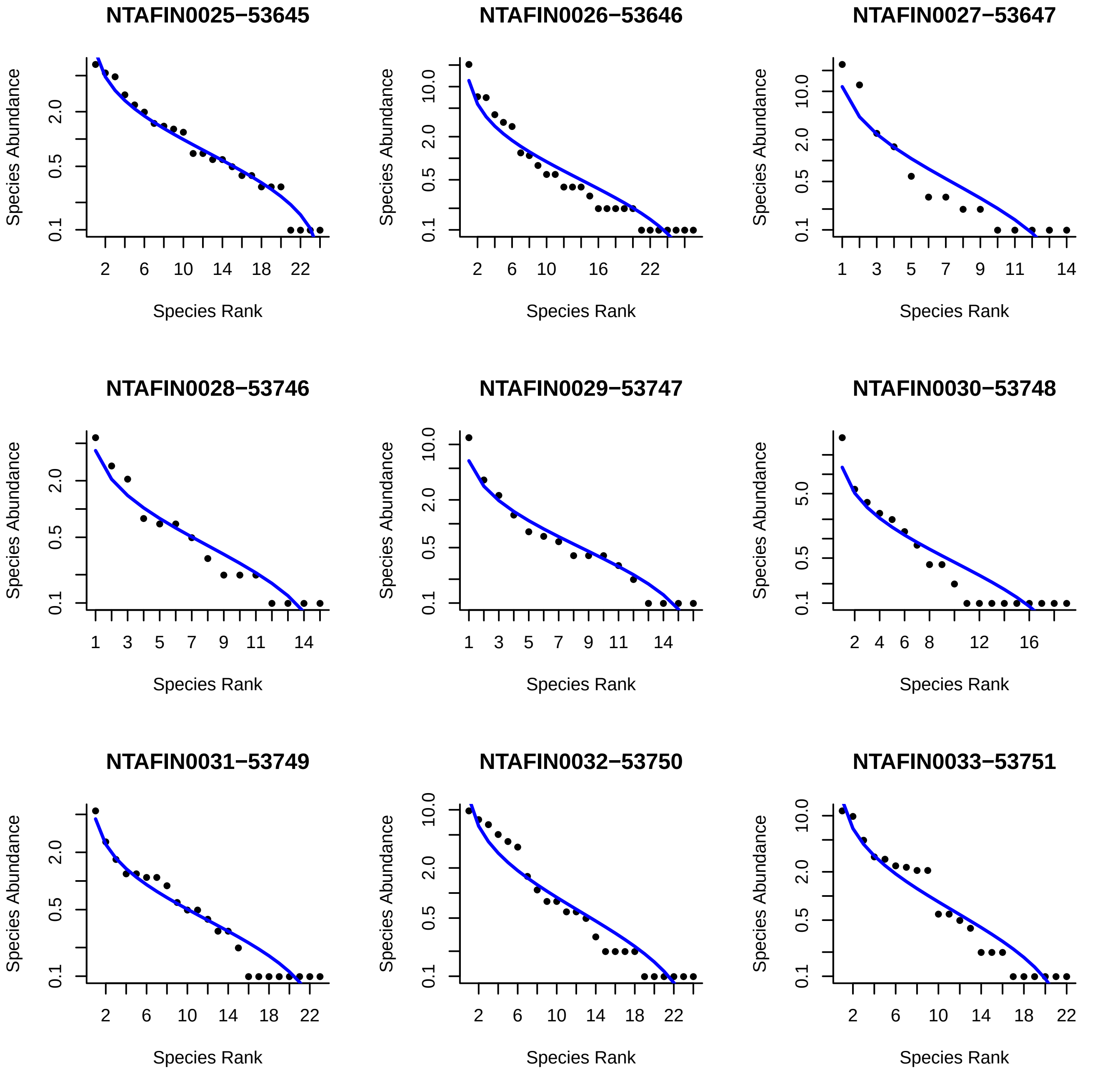

NTAFIN0025−53645
NTAFIN0026−53646
NTAFIN0027−53647
NTAFIN0028−53746
NTAFIN0029−53747
NTAFIN0030−53748
NTAFIN0031−53749
NTAFIN0032−53750
NTAFIN0033−53751
Species Abundance
Species Rank
0.1
0.5
2.0
5.0
10.0

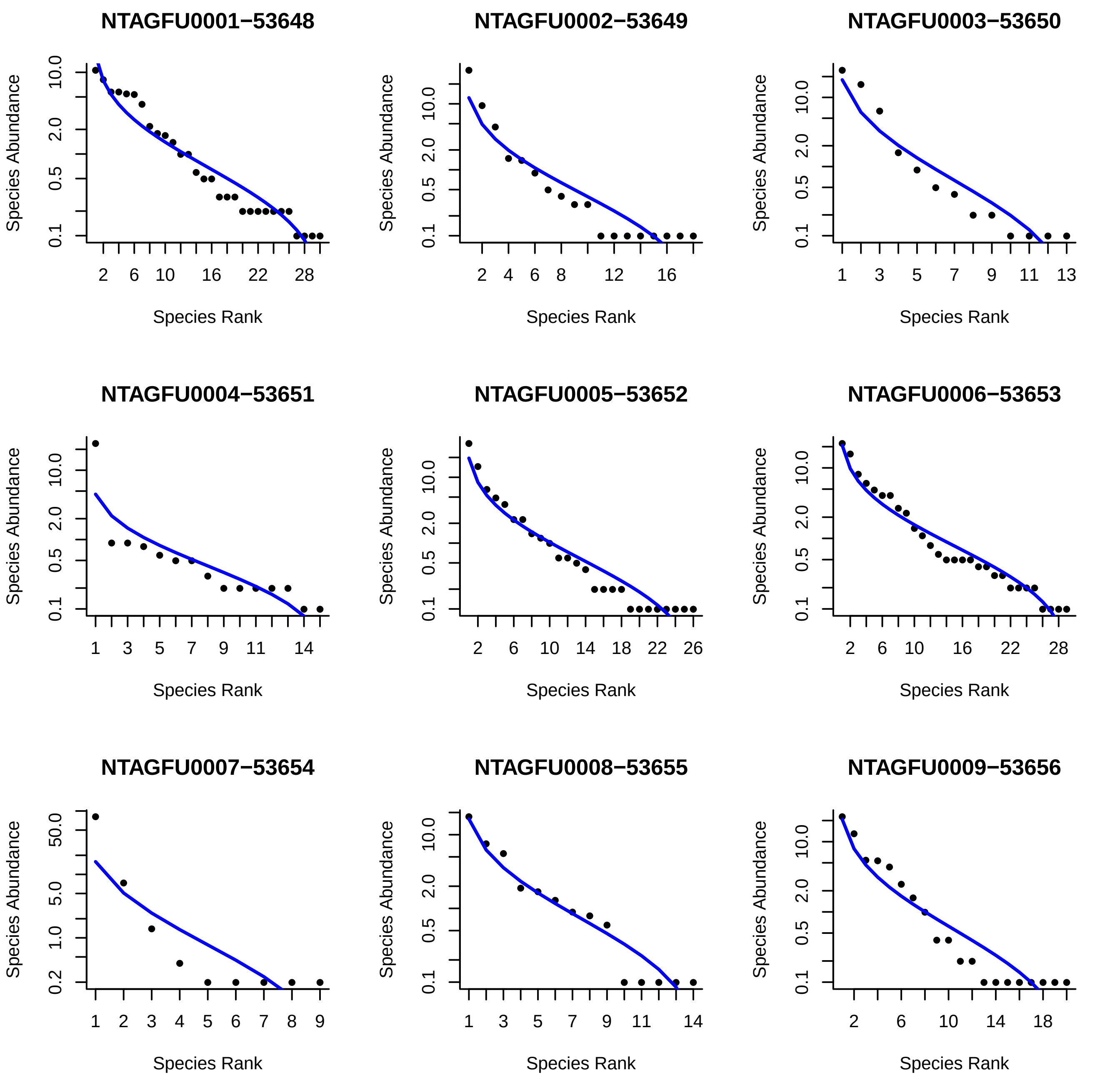
NTAGFU0001−53648
Species Abundance
Species Rank
NTAGFU0002−53649
Species Abundance
Species Rank
NTAGFU0003−53650
Species Abundance
Species Rank
NTAGFU0004−53651
Species Abundance
Species Rank
NTAGFU0005−53652
Species Abundance
Species Rank
NTAGFU0006−53653
Species Abundance
Species Rank
NTAGFU0007−53654
Species Abundance
Species Rank
NTAGFU0008−53655
Species Abundance
Species Rank
NTAGFU0009−53656
Species Abundance
Species Rank

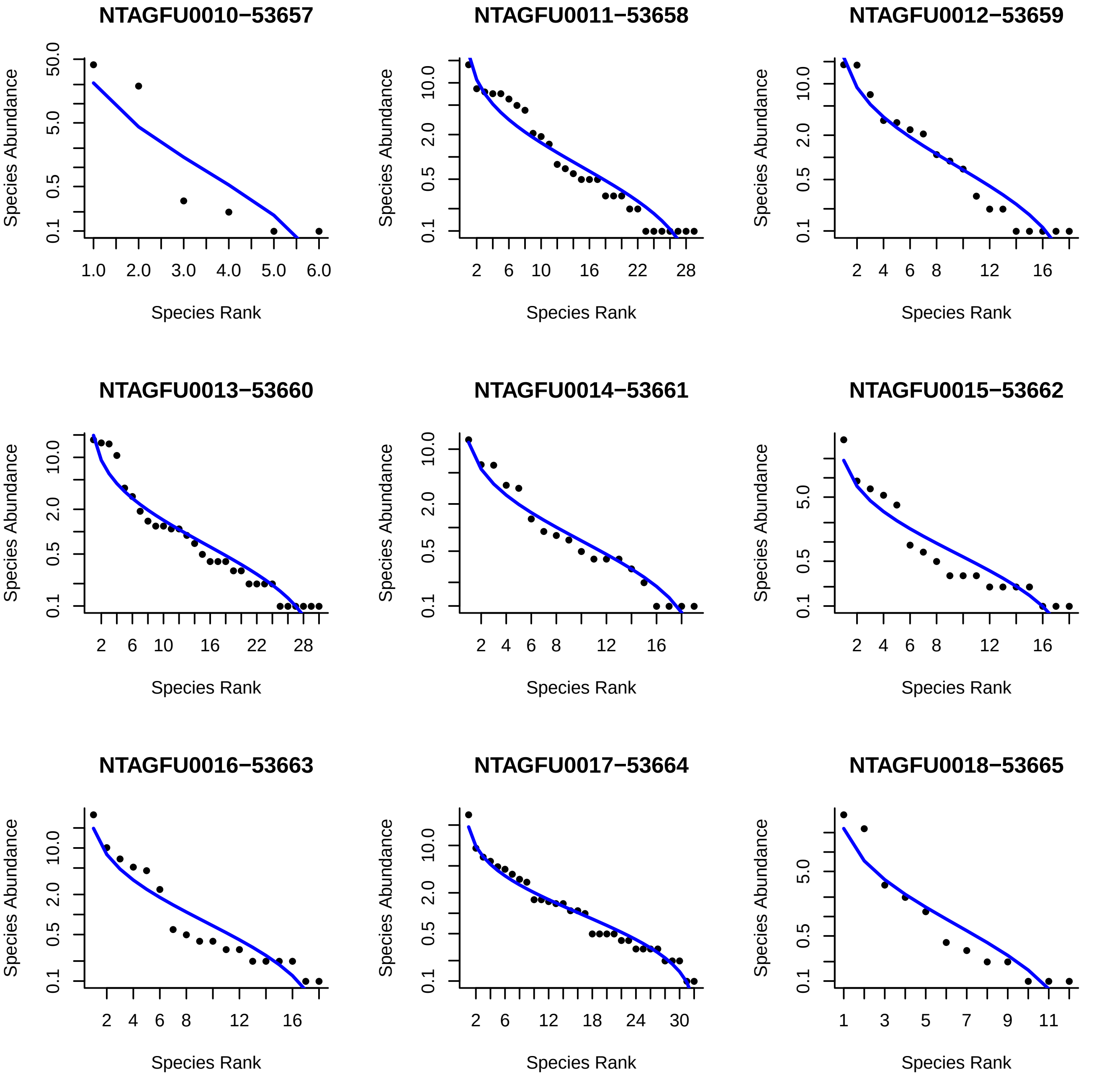
NTAGFU0010−53657
Species Abundance
Species Rank
0.1
0.5
5.0
50.0
1.0
2.0
3.0
4.0
5.0
6.0
NTAGFU0011−53658
Species Abundance
Species Rank
0.1
0.5
2.0
10.0
2
6
10
16
22
28
NTAGFU0012−53659
Species Abundance
Species Rank
0.1
0.5
2.0
10.0
2
4
6
8
12
16
NTAGFU0013−53660
Species Abundance
Species Rank
0.1
0.5
2.0
10.0
2
6
10
16
22
28
NTAGFU0014−53661
Species Abundance
Species Rank
0.1
0.5
2.0
10.0
2
4
6
8
12
16
NTAGFU0015−53662
Species Abundance
Species Rank
0.1
0.5
5.0
2
4
6
8
12
16
NTAGFU0016−53663
Species Abundance
Species Rank
0.1
0.5
2.0
10.0
2
4
6
8
12
16
NTAGFU0017−53664
Species Abundance
Species Rank
0.1
0.5
2.0
10.0
2
6
12
18
24
30
NTAGFU0018−53665
Species Abundance
Species Rank
0.1
0.5
5.0
1
3
5
7
9
11

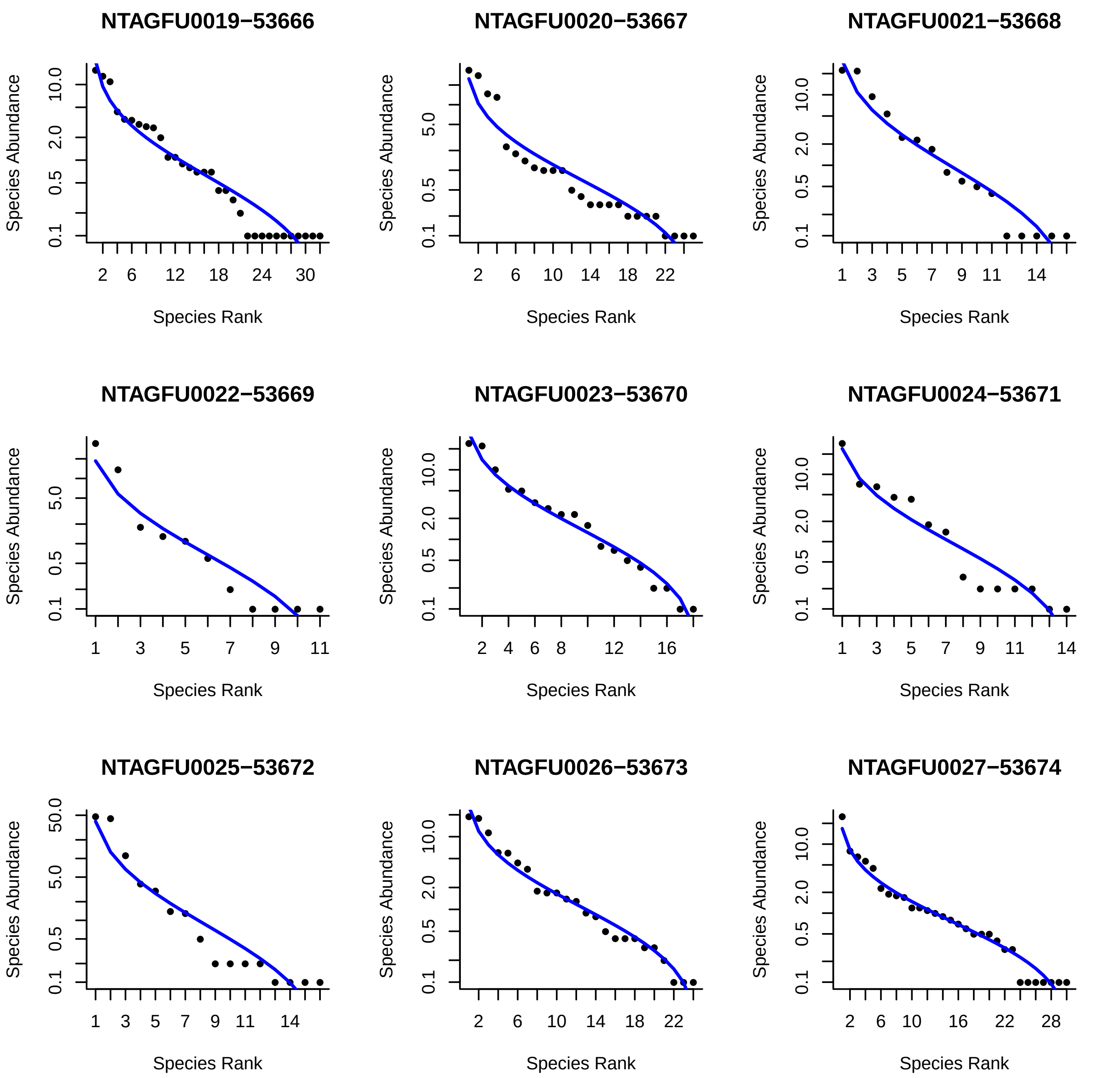
NTAGFU0019−53666
Species Abundance
10.0
2.0
0.5
0.1
2
6
12
18
24
30
Species Rank
NTAGFU0020−53667
Species Abundance
5.0
0.5
0.1
2
6
10
14
18
22
Species Rank
NTAGFU0021−53668
Species Abundance
10.0
2.0
0.5
0.1
1
3
5
7
9
11
14
Species Rank
NTAGFU0022−53669
Species Abundance
5.0
0.5
0.1
1
3
5
7
9
11
Species Rank
NTAGFU0023−53670
Species Abundance
10.0
2.0
0.5
0.1
2
4
6
8
12
16
Species Rank
NTAGFU0024−53671
Species Abundance
10.0
2.0
0.5
0.1
1
3
5
7
9
11
14
Species Rank
NTAGFU0025−53672
Species Abundance
50.0
5.0
0.5
0.1
1
3
5
7
9
11
14
Species Rank
NTAGFU0026−53673
Species Abundance
10.0
2.0
0.5
0.1
2
6
10
14
18
22
Species Rank
NTAGFU0027−53674
Species Abundance
10.0
2.0
0.5
0.1
2
6
10
16
22
28
Species Rank

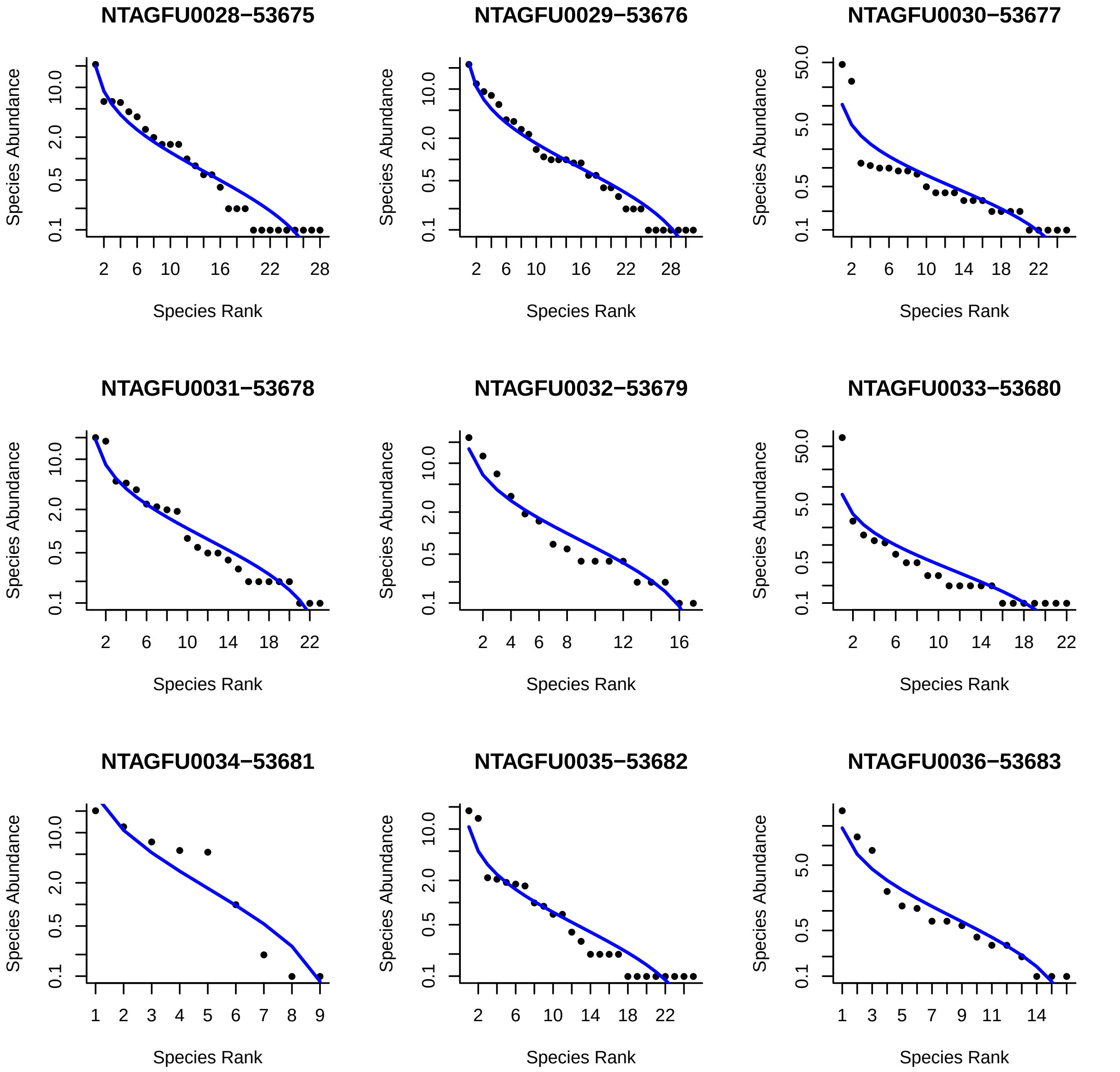
NTAGFU0028−53675
Species Abundance
0.1 0.5 2.0 10.0
2 6 10 16 22 28
Species Rank
NTAGFU0029−53676
Species Abundance
0.1 0.5 2.0 10.0
2 6 10 16 22 28
Species Rank
NTAGFU0030−53677
Species Abundance
0.1 0.5 5.0 50.0
2 6 10 14 18 22
Species Rank
NTAGFU0031−53678
Species Abundance
0.1 0.5 2.0 10.0
2 6 10 14 18 22
Species Rank
NTAGFU0032−53679
Species Abundance
0.1 0.5 2.0 10.0
2 4 6 8 12 16
Species Rank
NTAGFU0033−53680
Species Abundance
0.1 0.5 5.0 50.0
2 6 10 14 18 22
Species Rank
NTAGFU0034−53681
Species Abundance
0.1 0.5 2.0 10.0
1 2 3 4 5 6 7 8 9
Species Rank
NTAGFU0035−53682
Species Abundance
0.1 0.5 2.0 10.0
2 6 10 14 18 22
Species Rank
NTAGFU0036−53683
Species Abundance
0.1 0.5 5.0
1 3 5 7 9 11 14
Species Rank

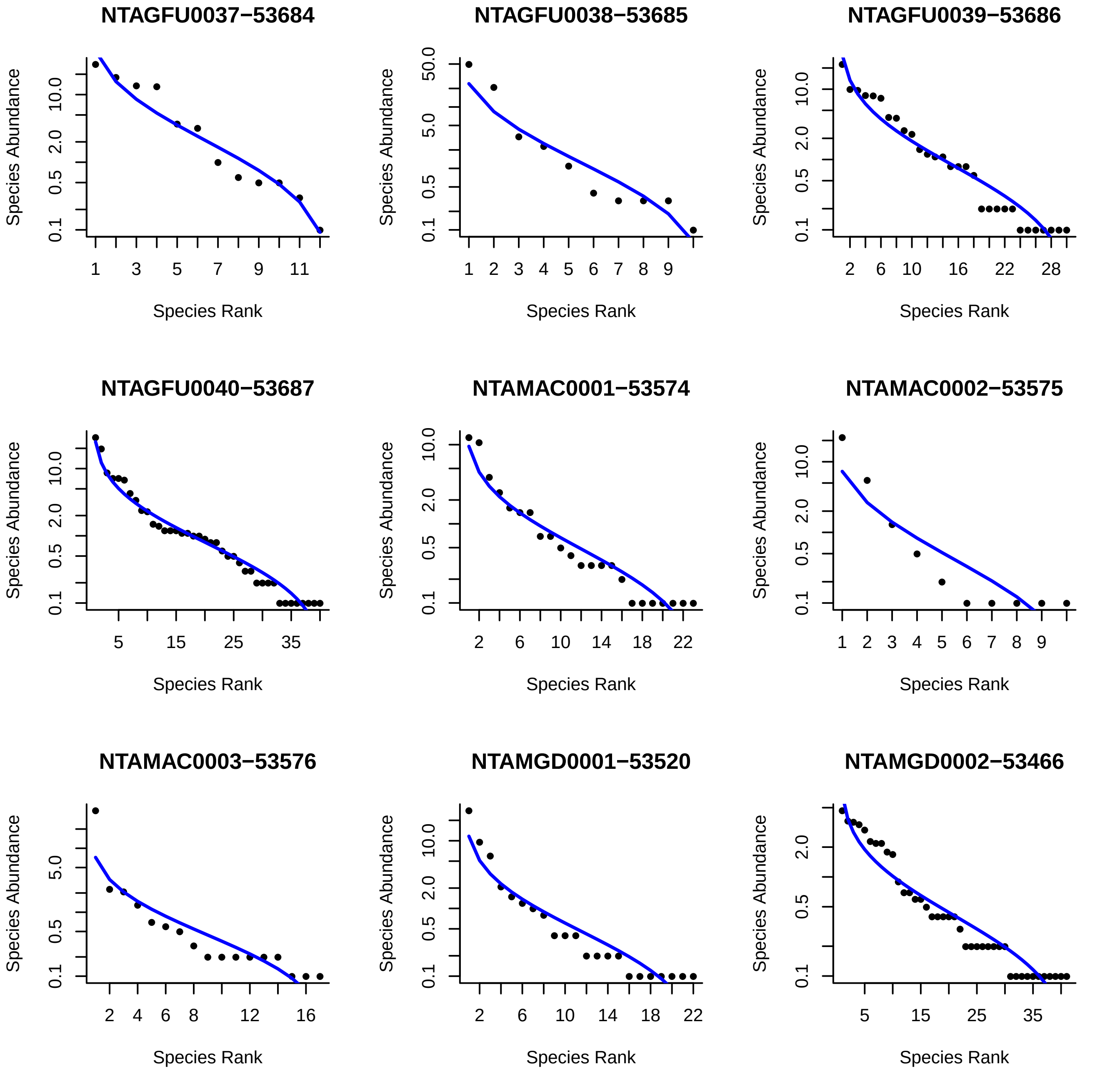
NTAGFU0037−53684
NTAGFU0038−53685
NTAGFU0039−53686
NTAGFU0040−53687
NTAMAC0001−53574
NTAMAC0002−53575
NTAMAC0003−53576
NTAMGD0001−53520
NTAMGD0002−53466
Species Abundance
Species Rank

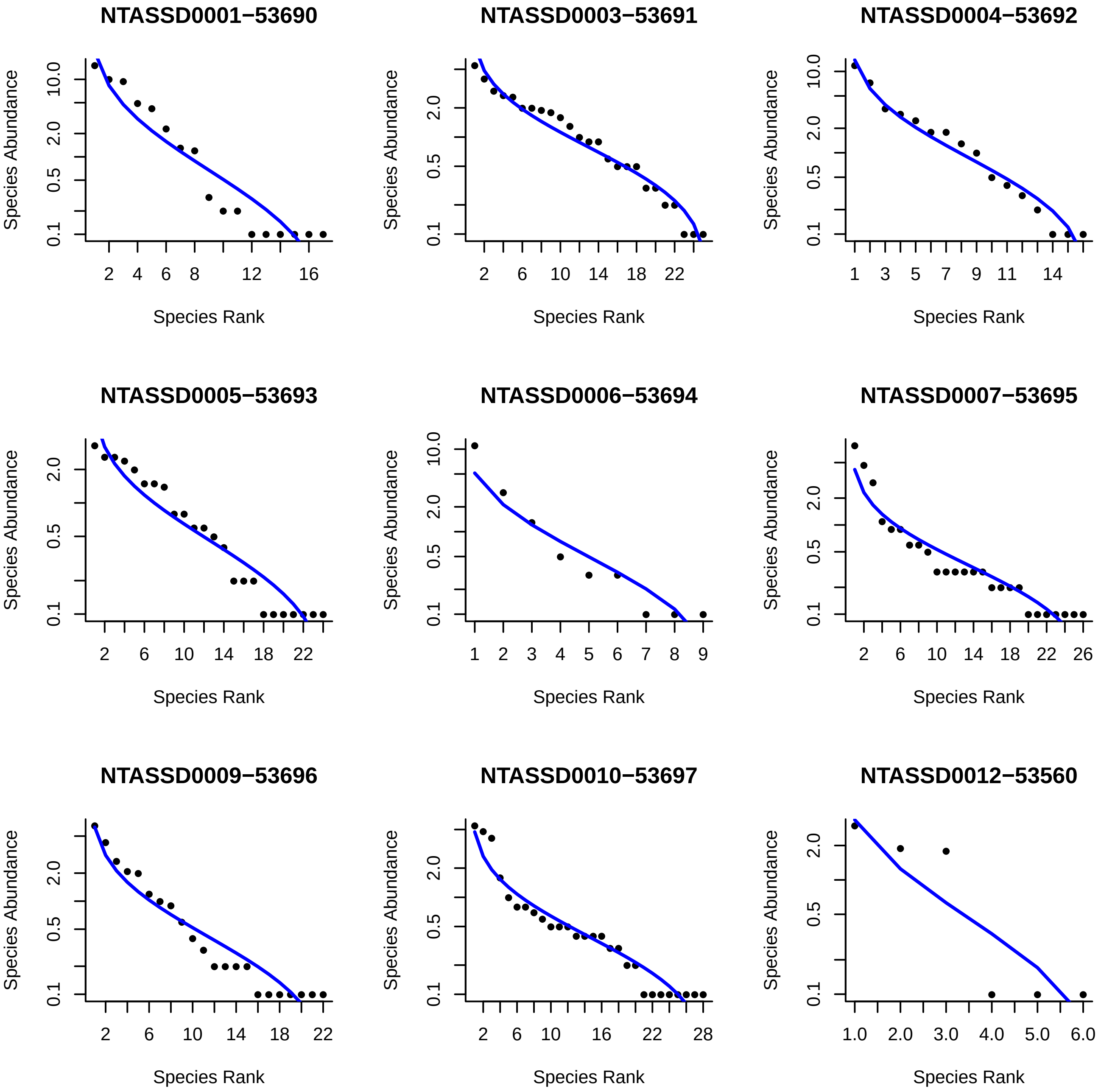
NTASSD0001−53690
NTASSD0003−53691
NTASSD0004−53692
NTASSD0005−53693
NTASSD0006−53694
NTASSD0007−53695
NTASSD0009−53696
NTASSD0010−53697
NTASSD0012−53560
Species Abundance
Species Rank
0.1
0.5
2.0
10.0
2 4 6 8 12 16
2 6 10 14 18 22
1 3 5 7 9 11 14
2 6 10 14 18 22
1 2 3 4 5 6 7 8 9
2 6 10 14 18 22 26
2 6 10 14 18 22
2 6 10 16 22 28
1.0 2.0 3.0 4.0 5.0 6.0

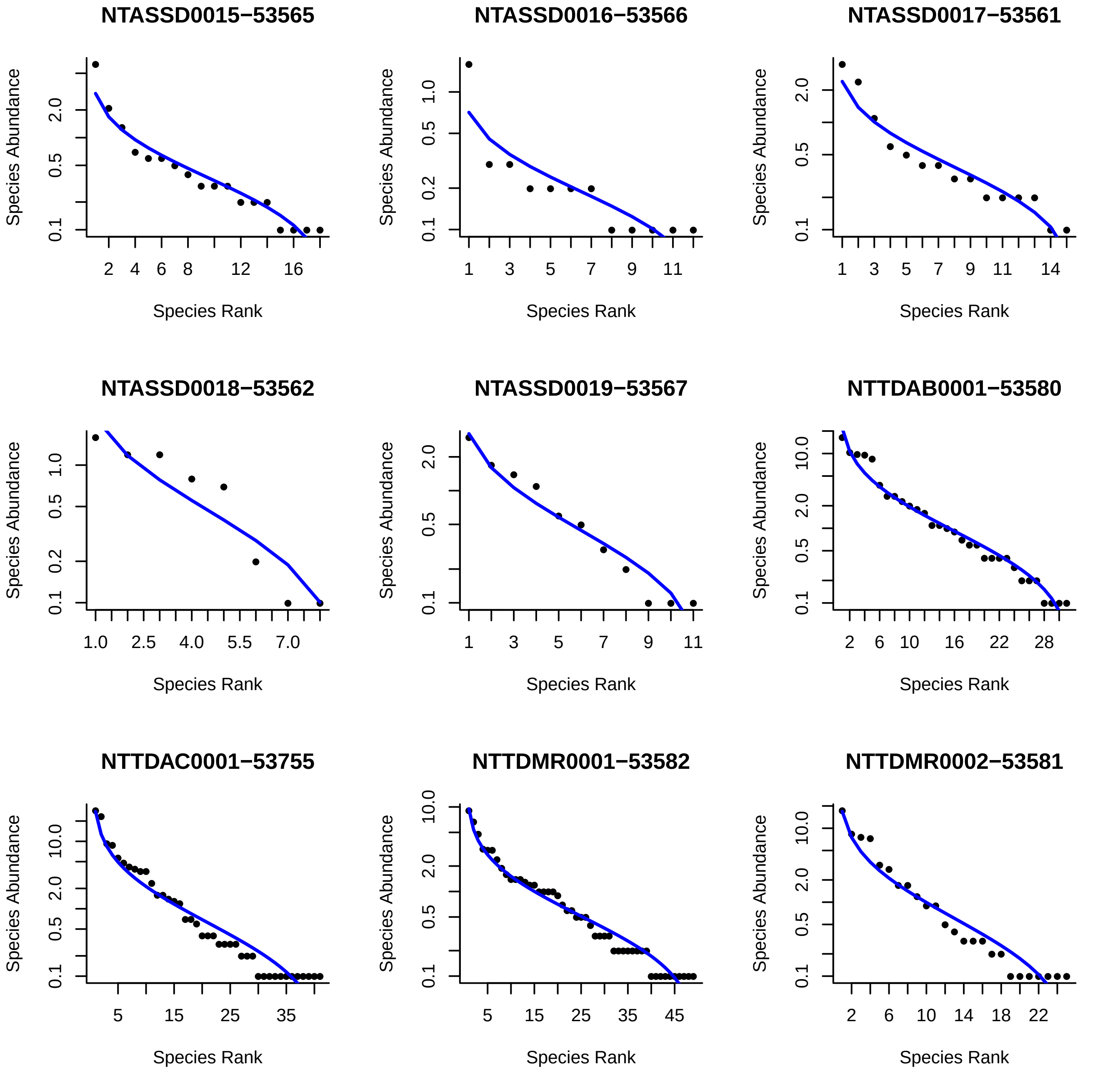
NTASSD0015−53565
Species Abundance
0.1 0.5 2.0
Species Rank
2 4 6 8 12 16
NTASSD0016−53566
Species Abundance
0.1 0.2 0.5 1.0
Species Rank
1 3 5 7 9 11
NTASSD0017−53561
Species Abundance
0.1 0.5 2.0
Species Rank
1 3 5 7 9 11 14
NTASSD0018−53562
Species Abundance
0.1 0.2 0.5 1.0
Species Rank
1.0 2.5 4.0 5.5 7.0
NTASSD0019−53567
Species Abundance
0.1 0.5 2.0
Species Rank
1 3 5 7 9 11
NTTDAB0001−53580
Species Abundance
0.1 0.5 2.0 10.0
Species Rank
2 6 10 16 22 28
NTTDAC0001−53755
Species Abundance
0.1 0.5 2.0 10.0
Species Rank
5 15 25 35
NTTDMR0001−53582
Species Abundance
0.1 0.5 2.0 10.0
Species Rank
5 15 25 35 45
NTTDMR0002−53581
Species Abundance
0.1 0.5 2.0 10.0
Species Rank
2 6 10 14 18 22

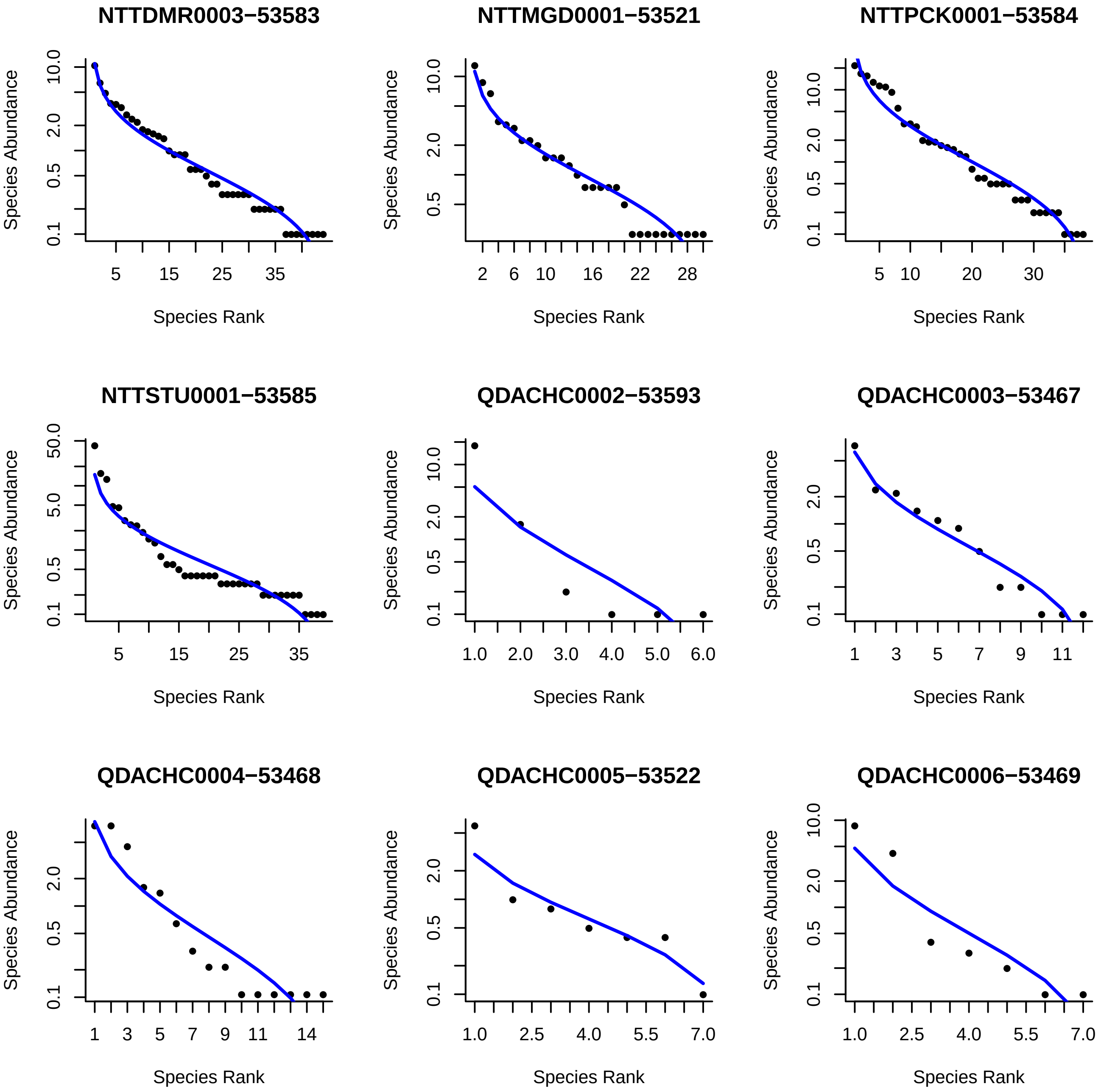
NTTDMR0003−53583
NTTMGD0001−53521
NTTPCK0001−53584
NTTSTU0001−53585
QDACHC0002−53593
QDACHC0003−53467
QDACHC0004−53468
QDACHC0005−53522
QDACHC0006−53469
Species Abundance
Species Rank
0.1
0.5
2.0
5.0
10.0
50.0
1
2
3
5
6
7
9
10
11
14
15
16
20
22
25
28
30
35
1.0
2.0
2.5
3.0
4.0
5.0
5.5
6.0
7.0

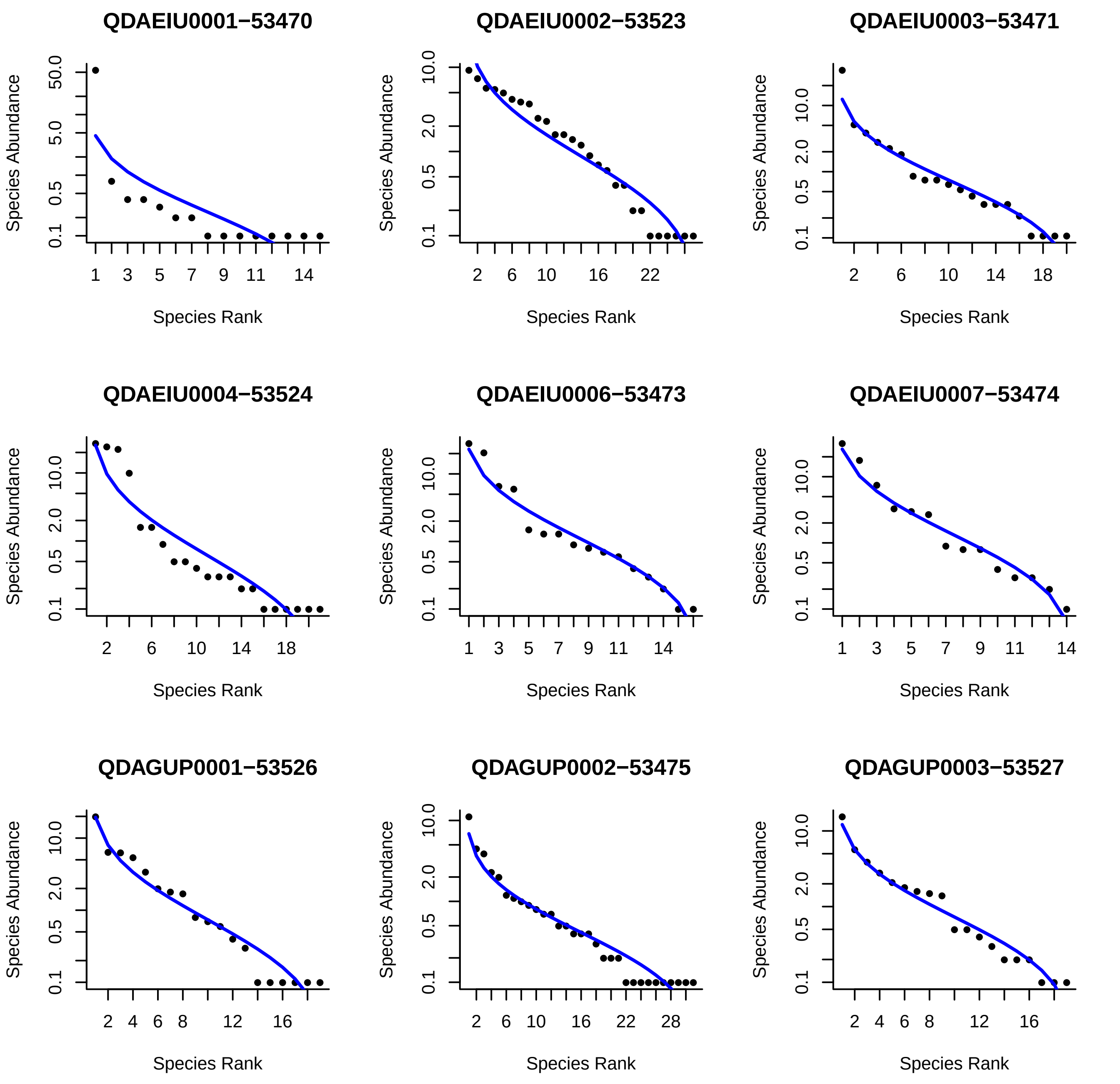
QDAEIU0001−53470
Species Abundance
0.1 0.5 5.0 50.0
Species Rank
1 3 5 7 9 11 14
QDAEIU0002−53523
Species Abundance
0.1 0.5 2.0 10.0
Species Rank
2 6 10 16 22
QDAEIU0003−53471
Species Abundance
0.1 0.5 2.0 10.0
Species Rank
2 6 10 14 18
QDAEIU0004−53524
Species Abundance
0.1 0.5 2.0 10.0
Species Rank
2 6 10 14 18
QDAEIU0006−53473
Species Abundance
0.1 0.5 2.0 10.0
Species Rank
1 3 5 7 9 11 14
QDAEIU0007−53474
Species Abundance
0.1 0.5 2.0 10.0
Species Rank
1 3 5 7 9 11 14
QDAGUP0001−53526
Species Abundance
0.1 0.5 2.0 10.0
Species Rank
2 4 6 8 12 16
QDAGUP0002−53475
Species Abundance
0.1 0.5 2.0 10.0
Species Rank
2 6 10 16 22 28
QDAGUP0003−53527
Species Abundance
0.1 0.5 2.0 10.0
Species Rank
2 4 6 8 12 16

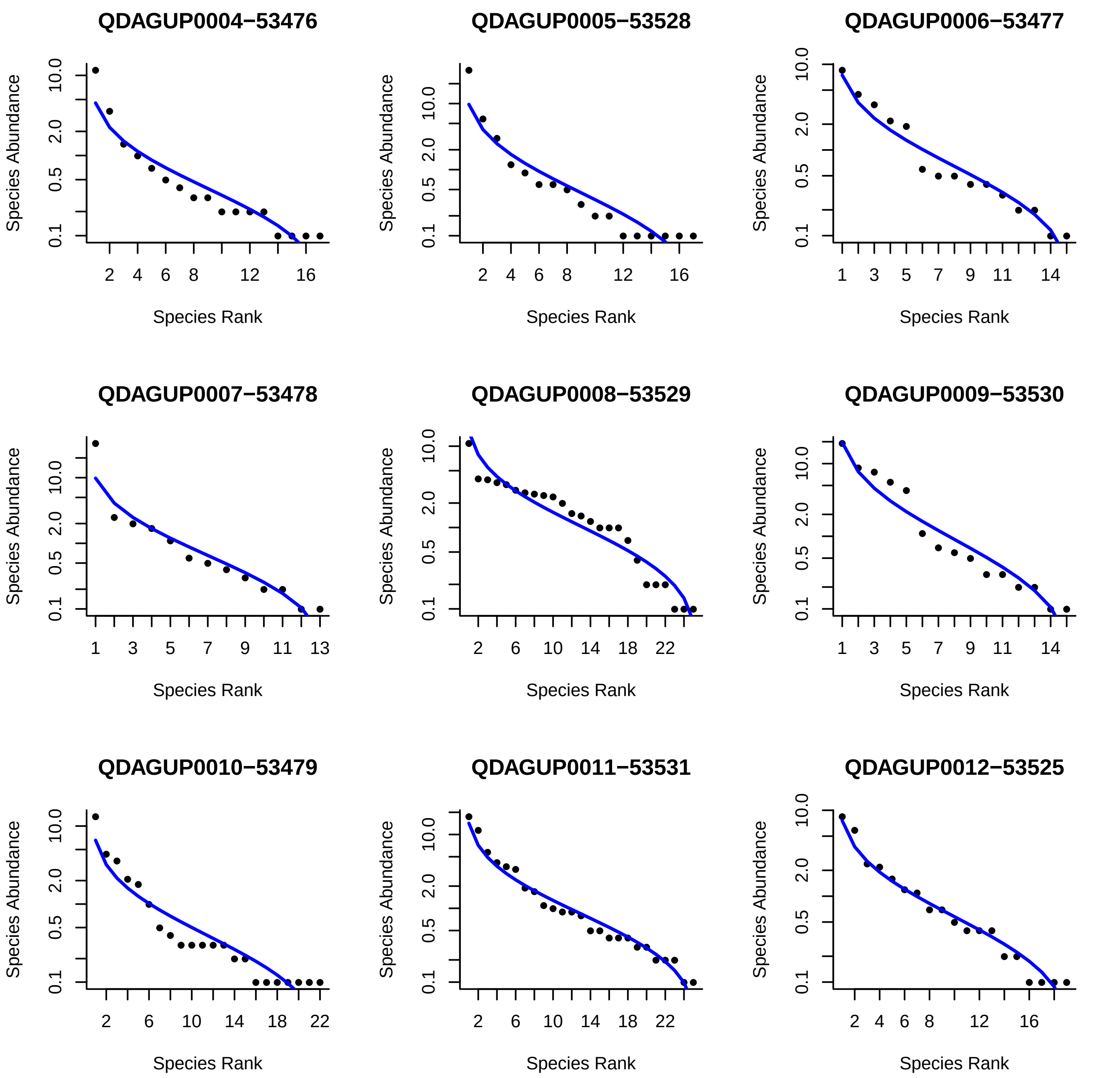
QDAGUP0004−53476
QDAGUP0005−53528
QDAGUP0006−53477
QDAGUP0007−53478
QDAGUP0008−53529
QDAGUP0009−53530
QDAGUP0010−53479
QDAGUP0011−53531
QDAGUP0012−53525
Species Abundance
Species Rank

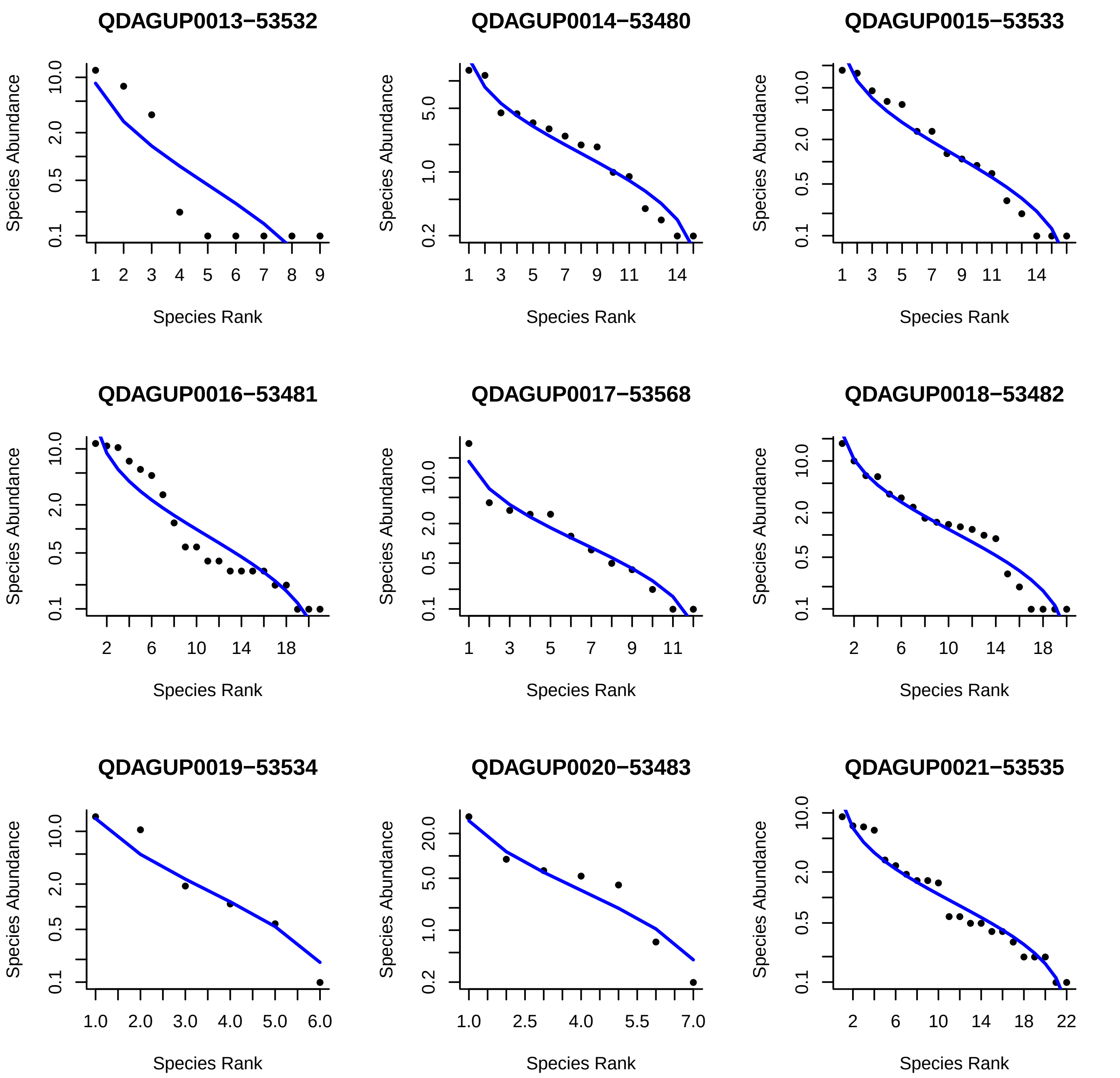
QDAGUP0013−53532
QDAGUP0014−53480
QDAGUP0015−53533
QDAGUP0016−53481
QDAGUP0017−53568
QDAGUP0018−53482
QDAGUP0019−53534
QDAGUP0020−53483
QDAGUP0021−53535
Species Abundance
Species Rank
0.1
0.5
2.0
10.0
0.2
1.0
5.0
20.0
1 2 3 4 5 6 7 8 9
1 3 5 7 9 11 14
1 3 5 7 9 11 14
2 6 10 14 18
1 3 5 7 9 11
2 6 10 14 18
1.0 2.0 3.0 4.0 5.0 6.0
1.0 2.5 4.0 5.5 7.0
2 6 10 14 18 22

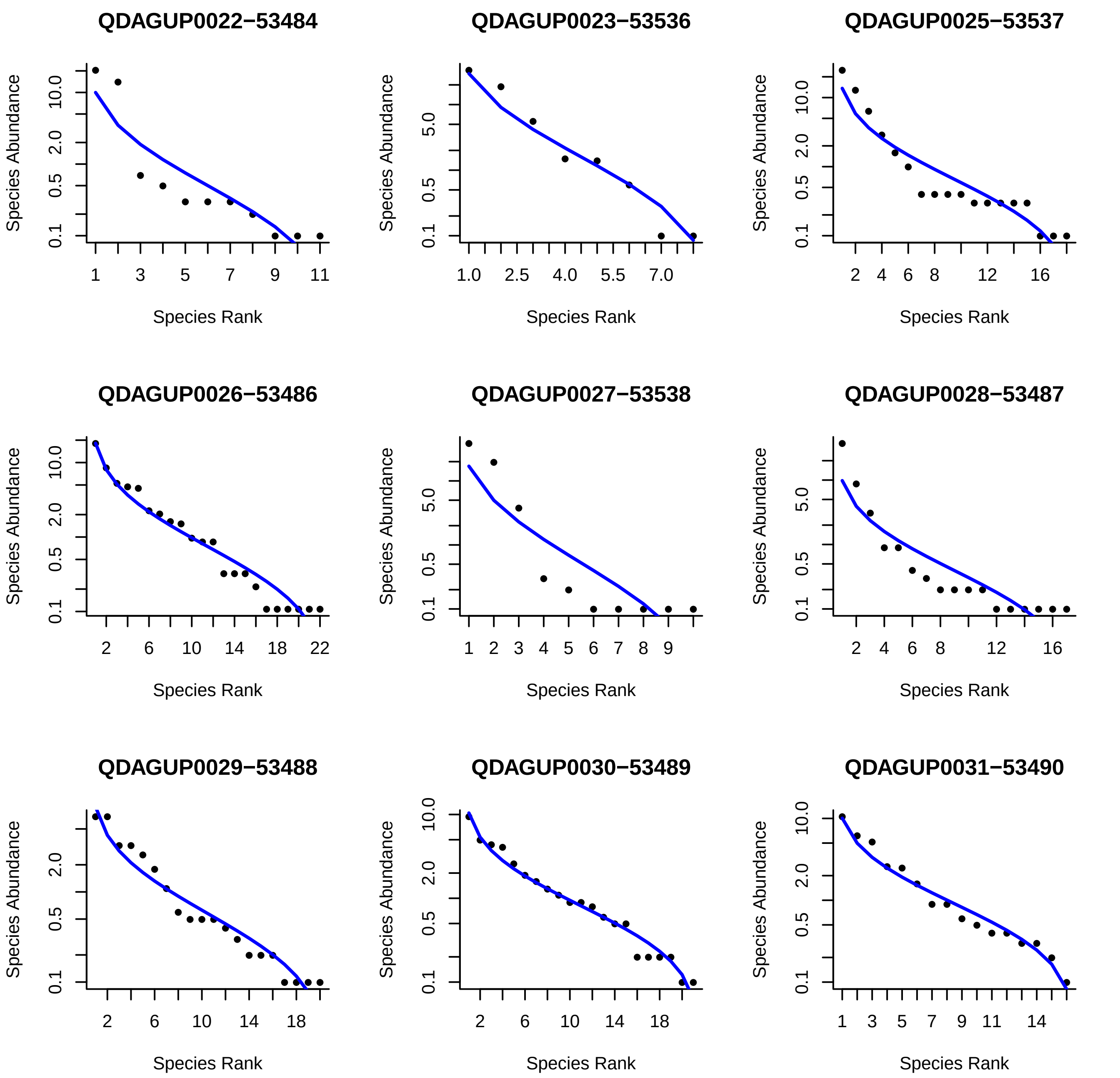
QDAGUP0022−53484
QDAGUP0023−53536
QDAGUP0025−53537
QDAGUP0026−53486
QDAGUP0027−53538
QDAGUP0028−53487
QDAGUP0029−53488
QDAGUP0030−53489
QDAGUP0031−53490
Species Abundance
Species Rank
0.1
0.5
2.0
5.0
10.0
1 3 5 7 9 11
1.0 2.5 4.0 5.5 7.0
2 4 6 8 12 16
2 6 10 14 18 22
1 2 3 4 5 6 7 8 9
2 4 6 8 12 16
2 6 10 14 18
2 6 10 14 18
1 3 5 7 9 11 14

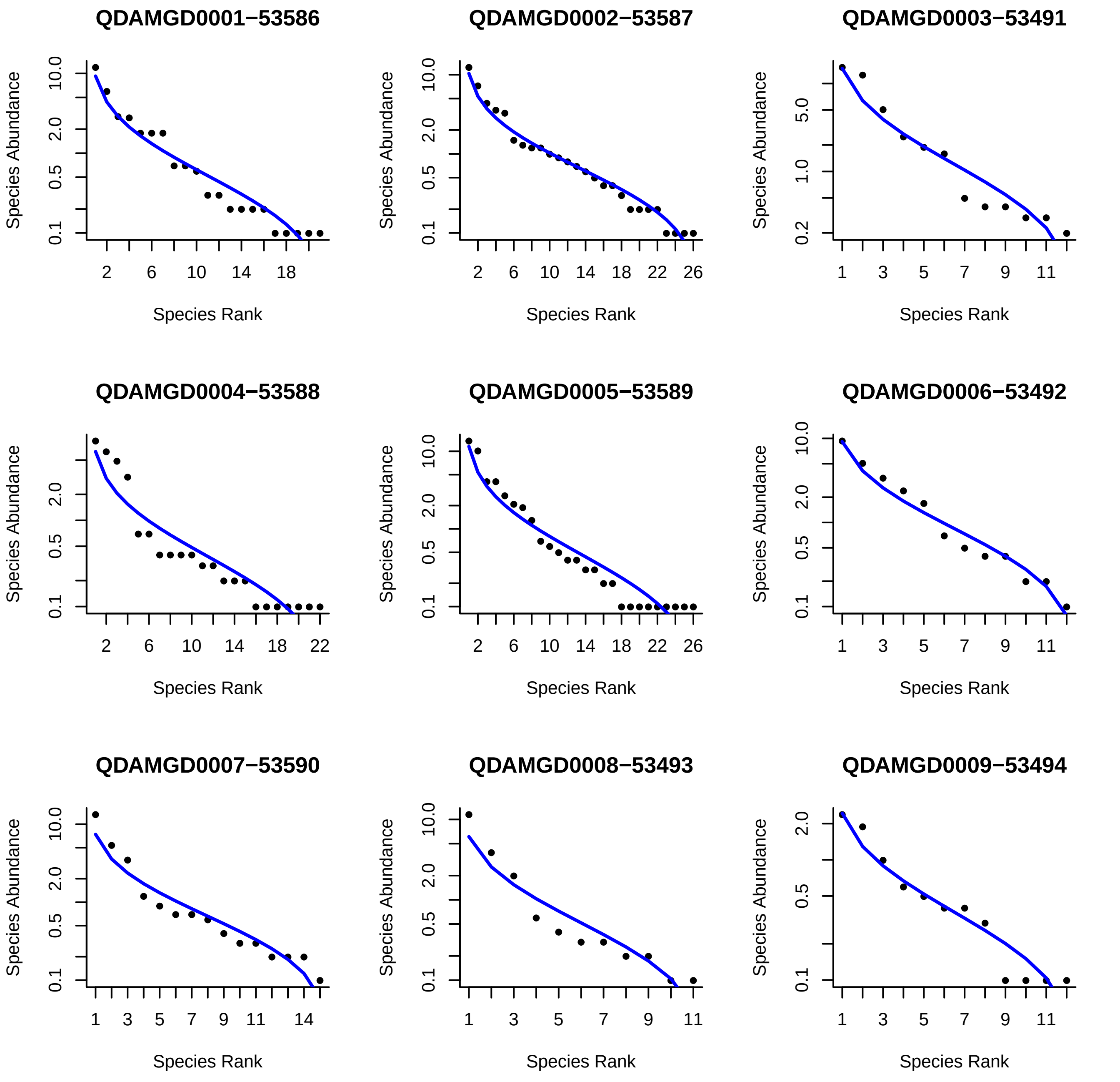
QDAMGD0001−53586
QDAMGD0002−53587
QDAMGD0003−53491
QDAMGD0004−53588
QDAMGD0005−53589
QDAMGD0006−53492
QDAMGD0007−53590
QDAMGD0008−53493
QDAMGD0009−53494
Species Abundance
Species Rank

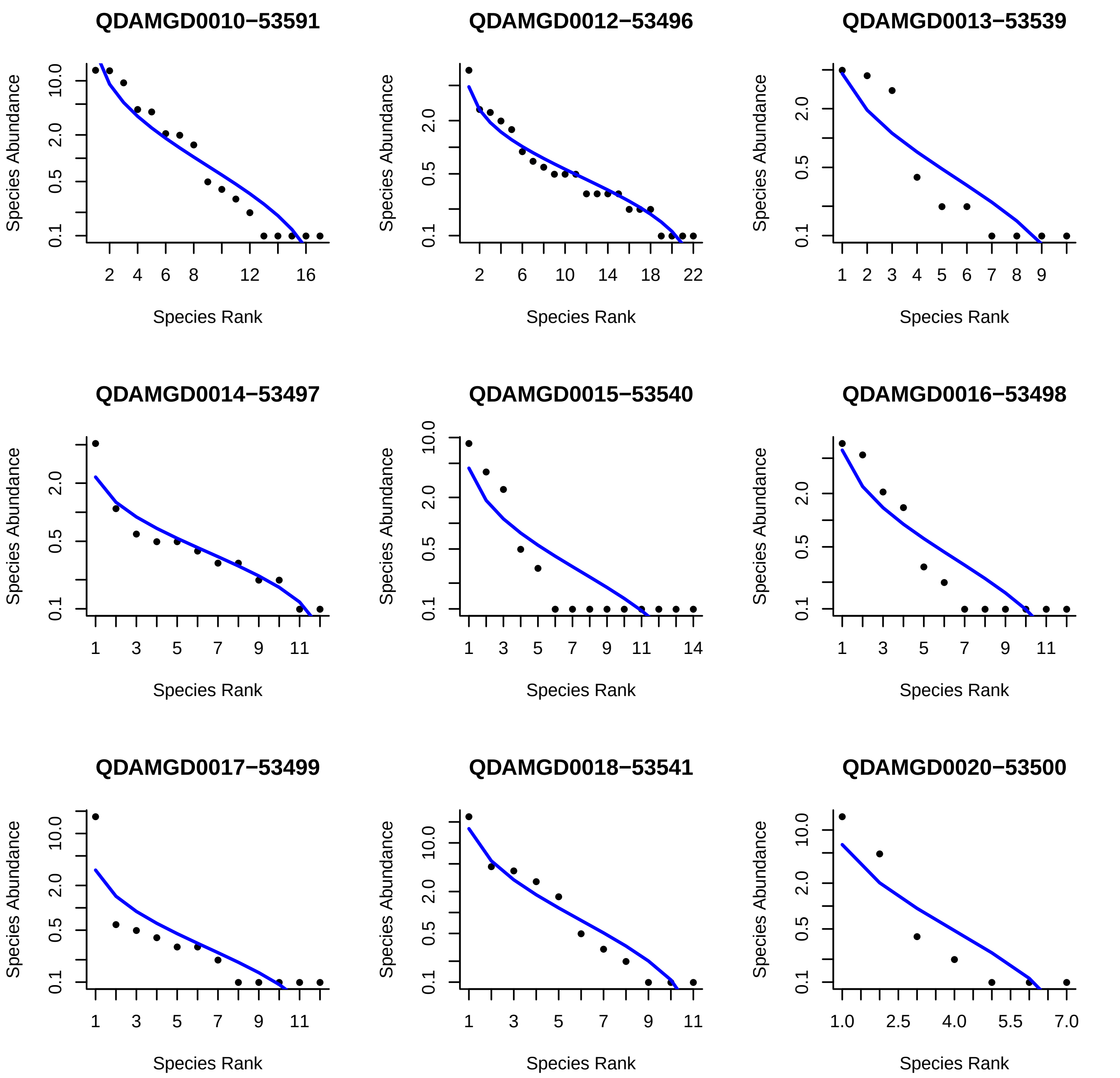
QDAMGD0010−53591
Species Abundance
0.1 0.5 2.0 10.0
2 4 6 8 12 16
Species Rank
QDAMGD0012−53496
Species Abundance
0.1 0.5 2.0
2 6 10 14 18 22
Species Rank
QDAMGD0013−53539
Species Abundance
0.1 0.5 2.0
1 2 3 4 5 6 7 8 9
Species Rank
QDAMGD0014−53497
Species Abundance
0.1 0.5 2.0
1 3 5 7 9 11
Species Rank
QDAMGD0015−53540
Species Abundance
0.1 0.5 2.0 10.0
1 3 5 7 9 11 14
Species Rank
QDAMGD0016−53498
Species Abundance
0.1 0.5 2.0
1 3 5 7 9 11
Species Rank
QDAMGD0017−53499
Species Abundance
0.1 0.5 2.0 10.0
1 3 5 7 9 11
Species Rank
QDAMGD0018−53541
Species Abundance
0.1 0.5 2.0 10.0
1 3 5 7 9 11
Species Rank
QDAMGD0020−53500
Species Abundance
0.1 0.5 2.0 10.0
1.0 2.5 4.0 5.5 7.0
Species Rank

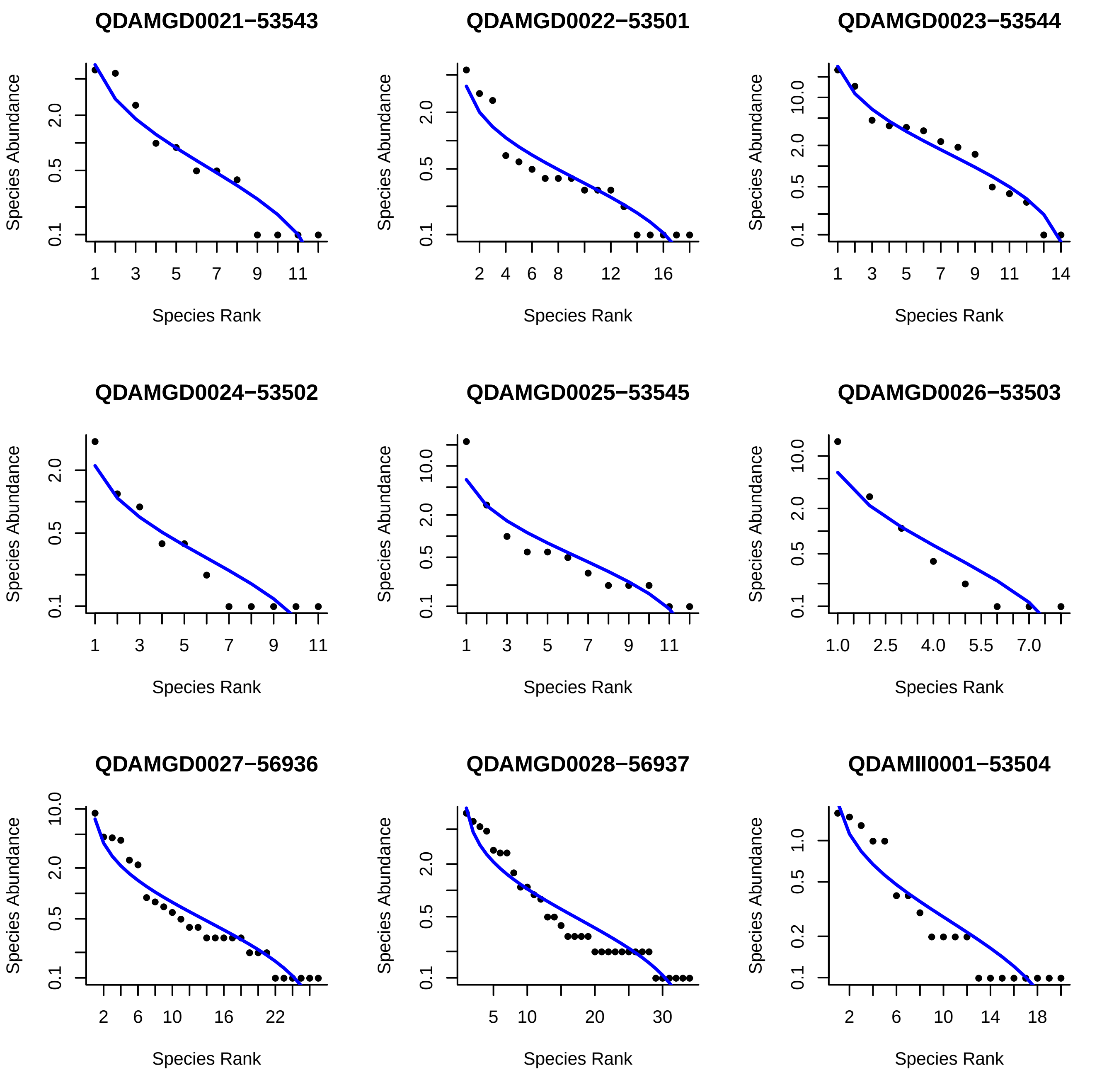
QDAMGD0021−53543
Species Abundance
0.1
0.5
2.0
Species Rank
1
3
5
7
9
11
QDAMGD0022−53501
Species Abundance
0.1
0.5
2.0
Species Rank
2
4
6
8
12
16
QDAMGD0023−53544
Species Abundance
0.1
0.5
2.0
10.0
Species Rank
1
3
5
7
9
11
14
QDAMGD0024−53502
Species Abundance
0.1
0.5
2.0
Species Rank
1
3
5
7
9
11
QDAMGD0025−53545
Species Abundance
0.1
0.5
2.0
10.0
Species Rank
1
3
5
7
9
11
QDAMGD0026−53503
Species Abundance
0.1
0.5
2.0
10.0
Species Rank
1.0
2.5
4.0
5.5
7.0
QDAMGD0027−56936
Species Abundance
0.1
0.5
2.0
10.0
Species Rank
2
6
10
16
22
QDAMGD0028−56937
Species Abundance
0.1
0.5
2.0
Species Rank
5
10
20
30
QDAMII0001−53504
Species Abundance
0.1
0.2
0.5
1.0
Species Rank
2
6
10
14
18

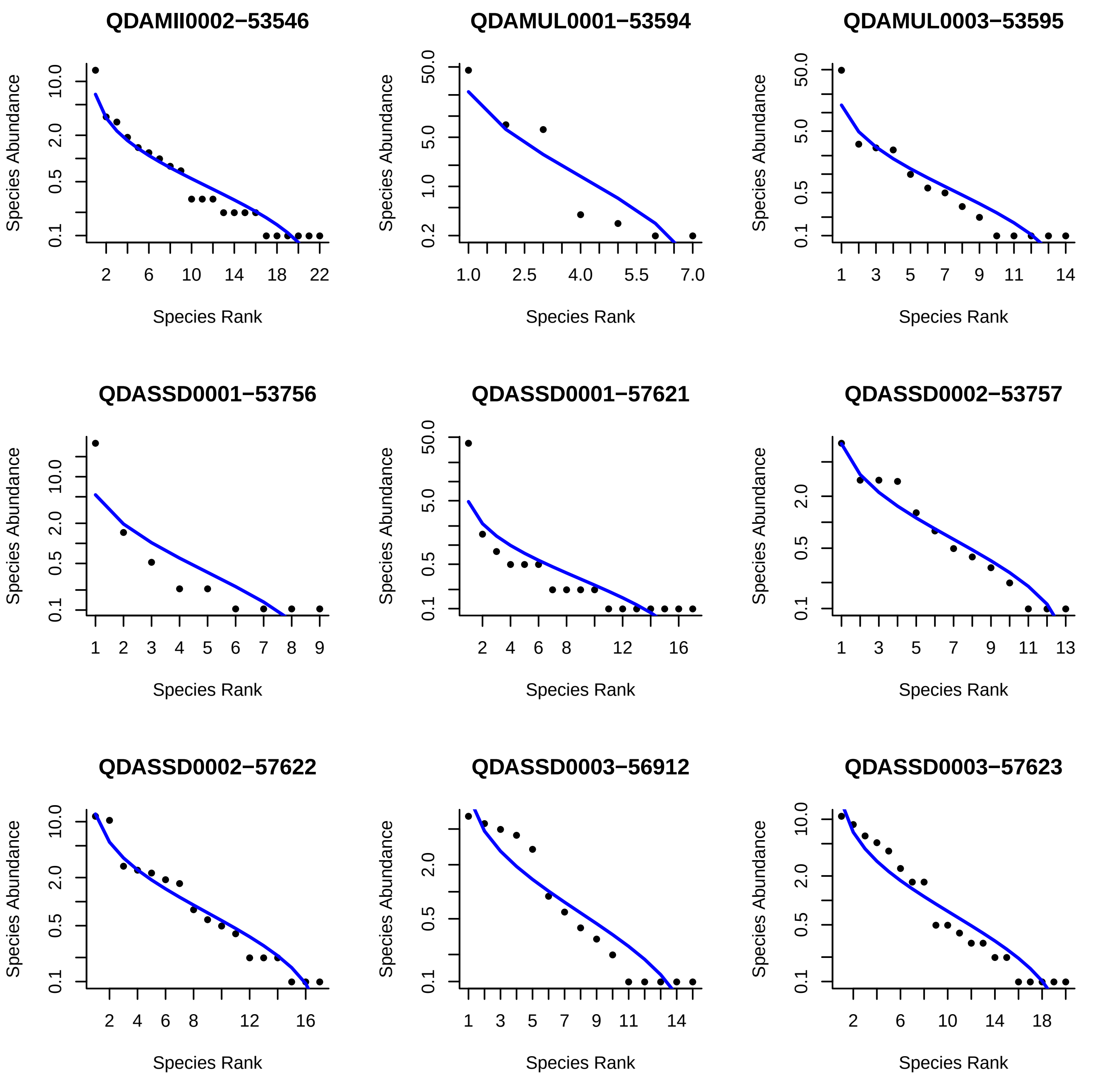
QDAMII0002−53546
QDAMUL0001−53594
QDAMUL0003−53595
QDASSD0001−53756
QDASSD0001−57621
QDASSD0002−53757
QDASSD0002−57622
QDASSD0003−56912
QDASSD0003−57623
Species Abundance
Species Rank
0.1 0.5 2.0 10.0
0.2 1.0 5.0 50.0
0.1 0.5 5.0 50.0
2 6 10 14 18 22
1.0 2.5 4.0 5.5 7.0
1 3 5 7 9 11 14
1 2 3 4 5 6 7 8 9
2 4 6 8 12 16
1 3 5 7 9 11 13
1 3 5 7 9 11 14
2 6 10 14 18

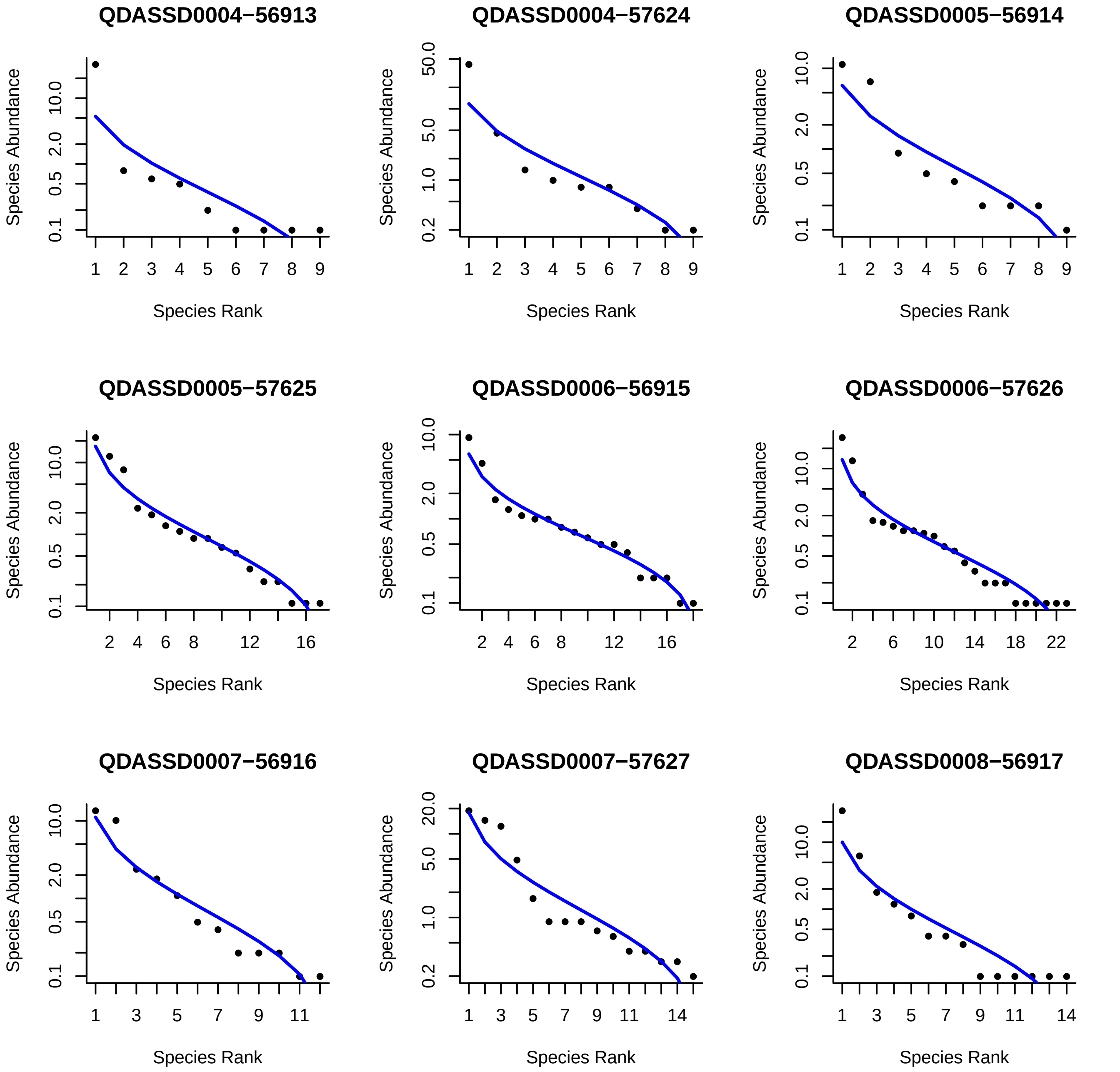

QDASSD0004−56913
Species Abundance
0.1 0.5 2.0 10.0
1 2 3 4 5 6 7 8 9
Species Rank
QDASSD0004−57624
Species Abundance
0.2 1.0 5.0 50.0
1 2 3 4 5 6 7 8 9
Species Rank
QDASSD0005−56914
Species Abundance
0.1 0.5 2.0 10.0
1 2 3 4 5 6 7 8 9
Species Rank
QDASSD0005−57625
Species Abundance
0.1 0.5 2.0 10.0
2 4 6 8 12 16
Species Rank
QDASSD0006−56915
Species Abundance
0.1 0.5 2.0 10.0
2 4 6 8 12 16
Species Rank
QDASSD0006−57626
Species Abundance
0.1 0.5 2.0 10.0
2 6 10 14 18 22
Species Rank
QDASSD0007−56916
Species Abundance
0.1 0.5 2.0 10.0
1 3 5 7 9 11
Species Rank
QDASSD0007−57627
Species Abundance
0.2 1.0 5.0 20.0
1 3 5 7 9 11 14
Species Rank
QDASSD0008−56917
Species Abundance
0.1 0.5 2.0 10.0
1 3 5 7 9 11 14
Species Rank

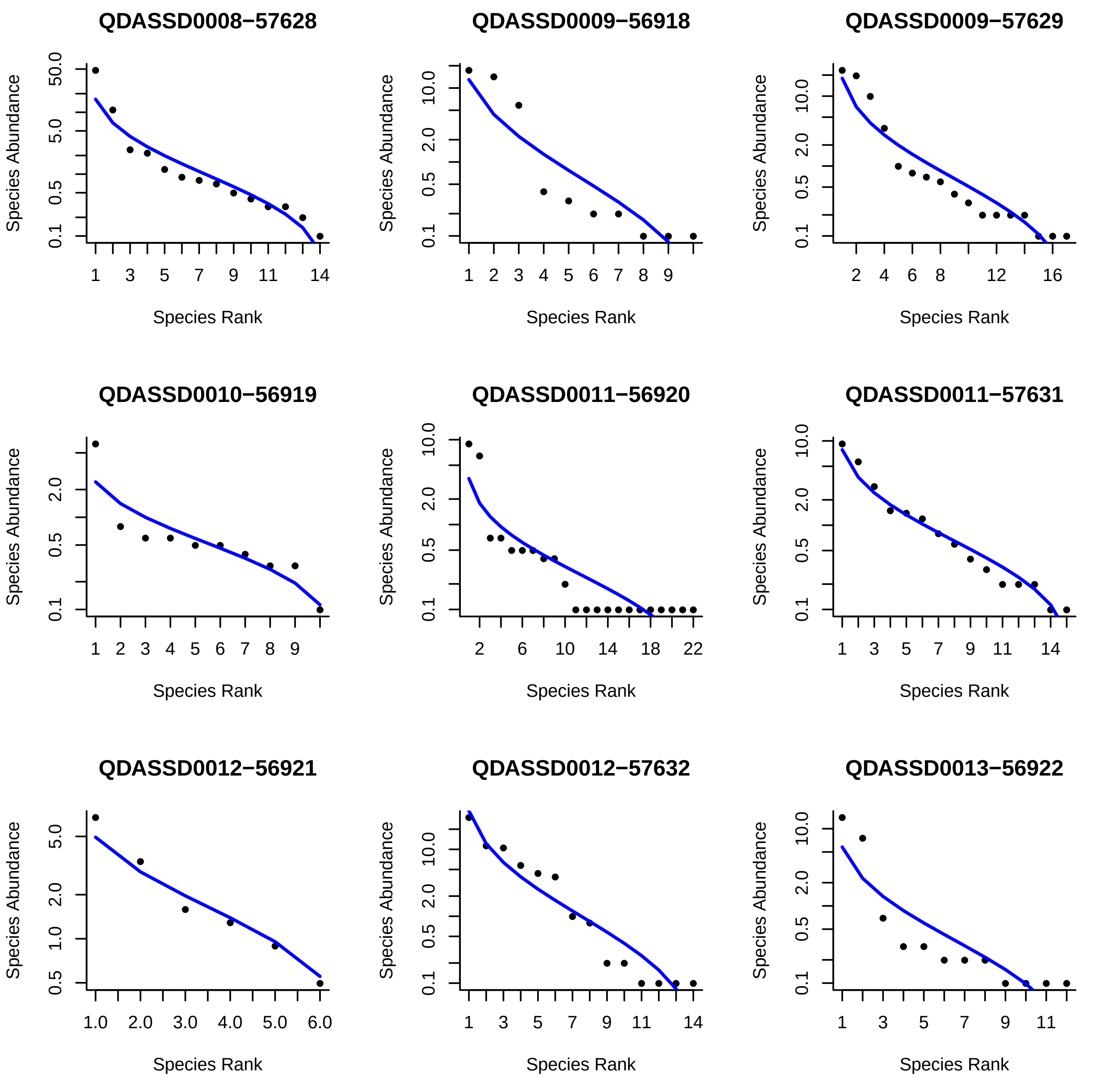
QDASSD0008−57628
QDASSD0009−56918
QDASSD0009−57629
QDASSD0010−56919
QDASSD0011−56920
QDASSD0011−57631
QDASSD0012−56921
QDASSD0012−57632
QDASSD0013−56922
Species Abundance
Species Rank
0.1
0.5
2.0
5.0
10.0
50.0
1.0
2.0
3.0
4.0
5.0
6.0

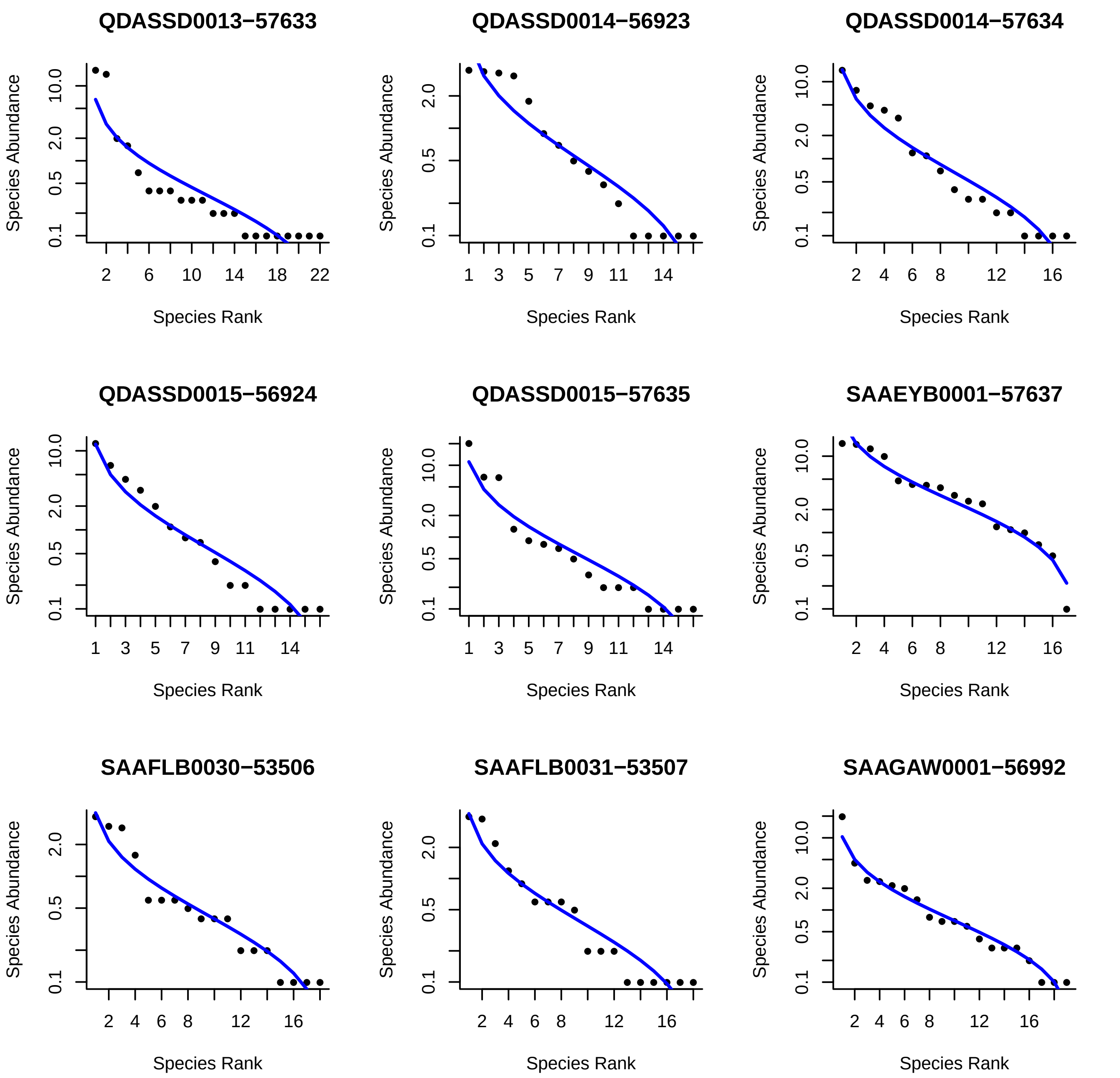
QDASSD0013−57633
QDASSD0014−56923
QDASSD0014−57634
QDASSD0015−56924
QDASSD0015−57635
SAAEYB0001−57637
SAAFLB0030−53506
SAAFLB0031−53507
SAAGAW0001−56992
Species Abundance
Species Rank
0.1
0.5
2.0
10.0
2
6
10
14
18
22
1
3
5
7
9
11
4
8
12
16

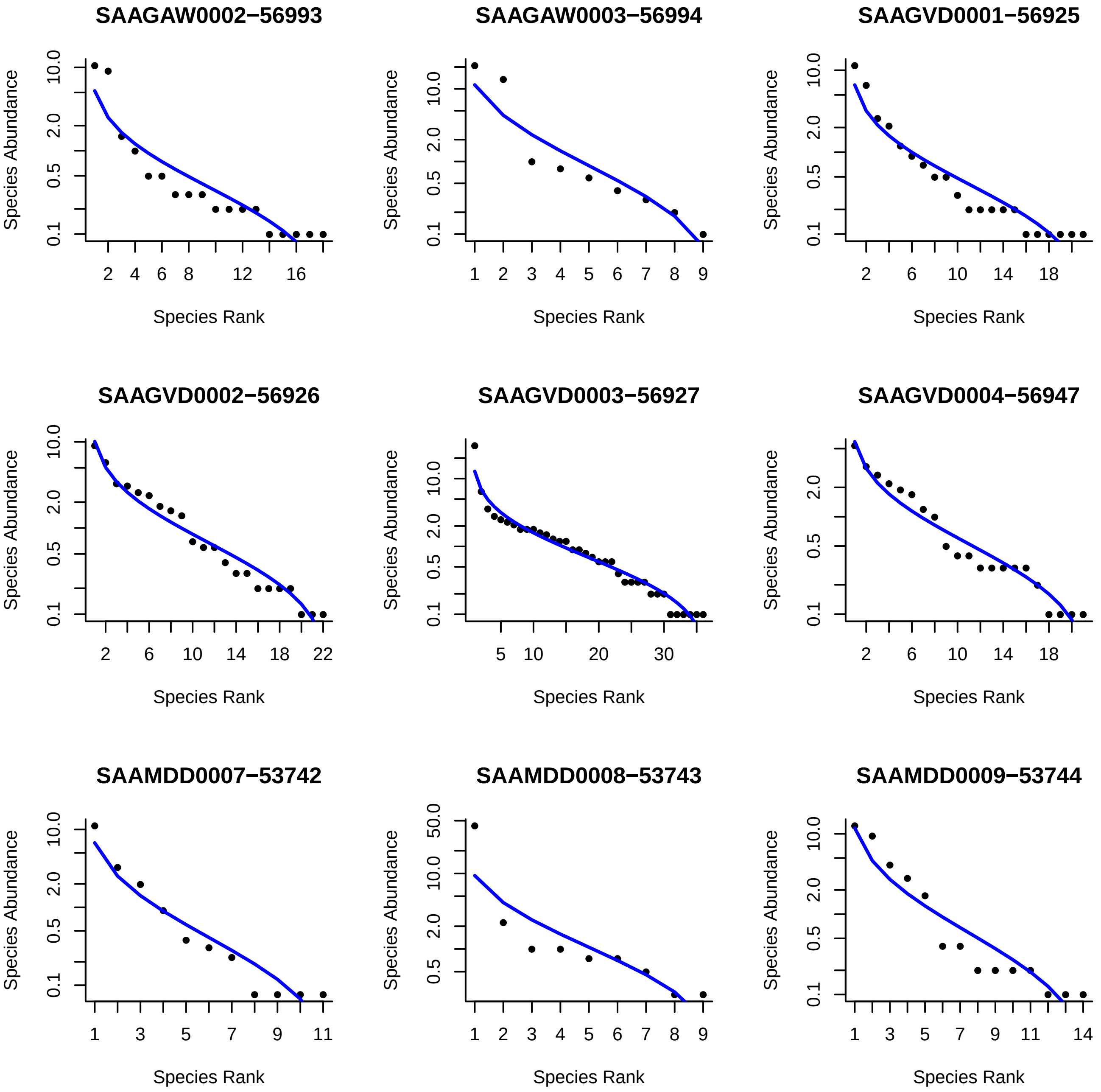
SAAGAW0002−56993
SAAGAW0003−56994
SAAGVD0001−56925
SAAGVD0002−56926
SAAGVD0003−56927
SAAGVD0004−56947
SAAMDD0007−53742
SAAMDD0008−53743
SAAMDD0009−53744
Species Abundance
Species Rank
0.1
0.5
2.0
10.0
50.0
1
2
3
4
5
6
7
8
9
10
11
12
14
16
18
20
22
30

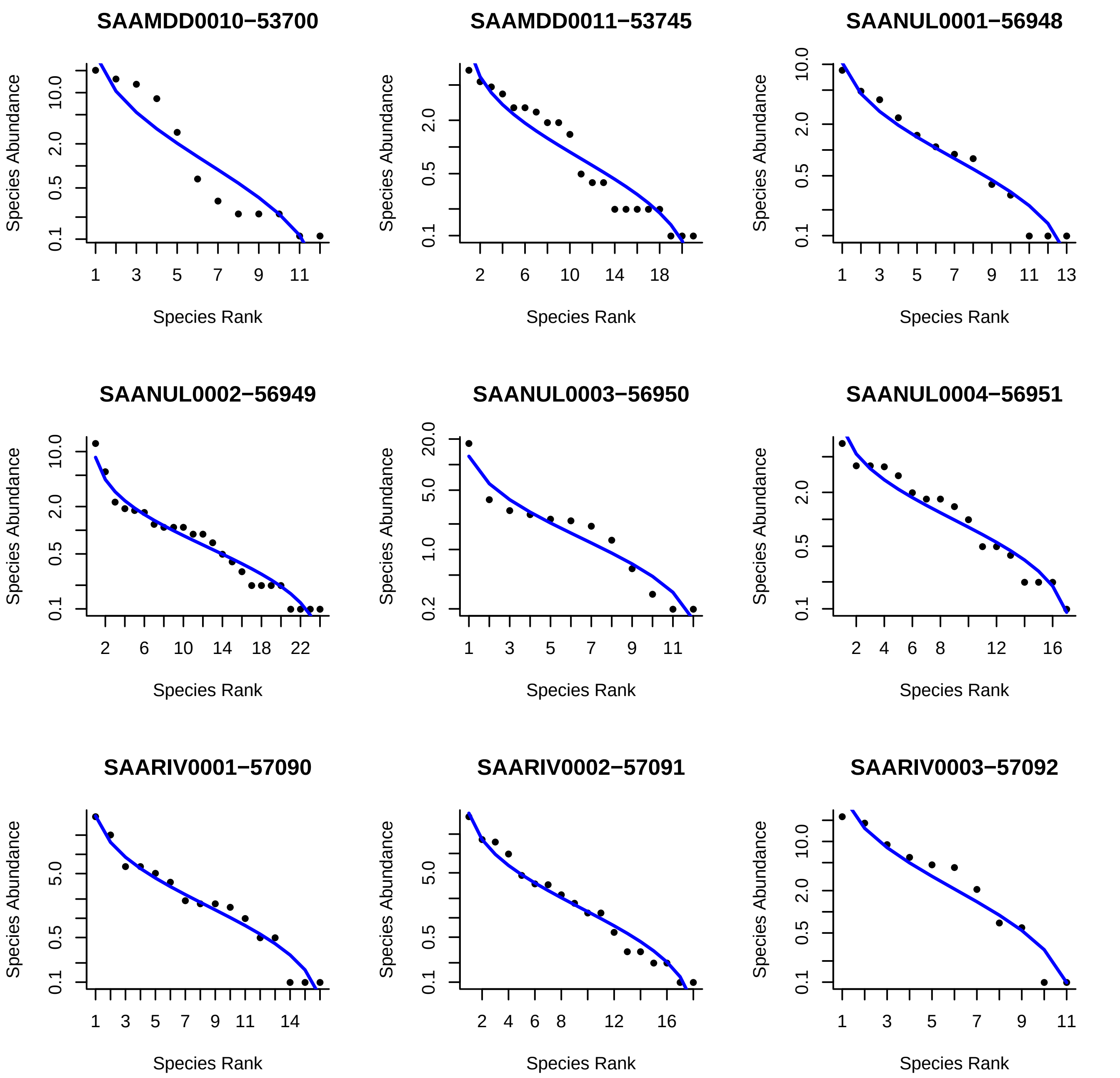
SAAMDD0010−53700
SAAMDD0011−53745
SAANUL0001−56948
SAANUL0002−56949
SAANUL0003−56950
SAANUL0004−56951
SAARIV0001−57090
SAARIV0002−57091
SAARIV0003−57092
Species Abundance
Species Rank

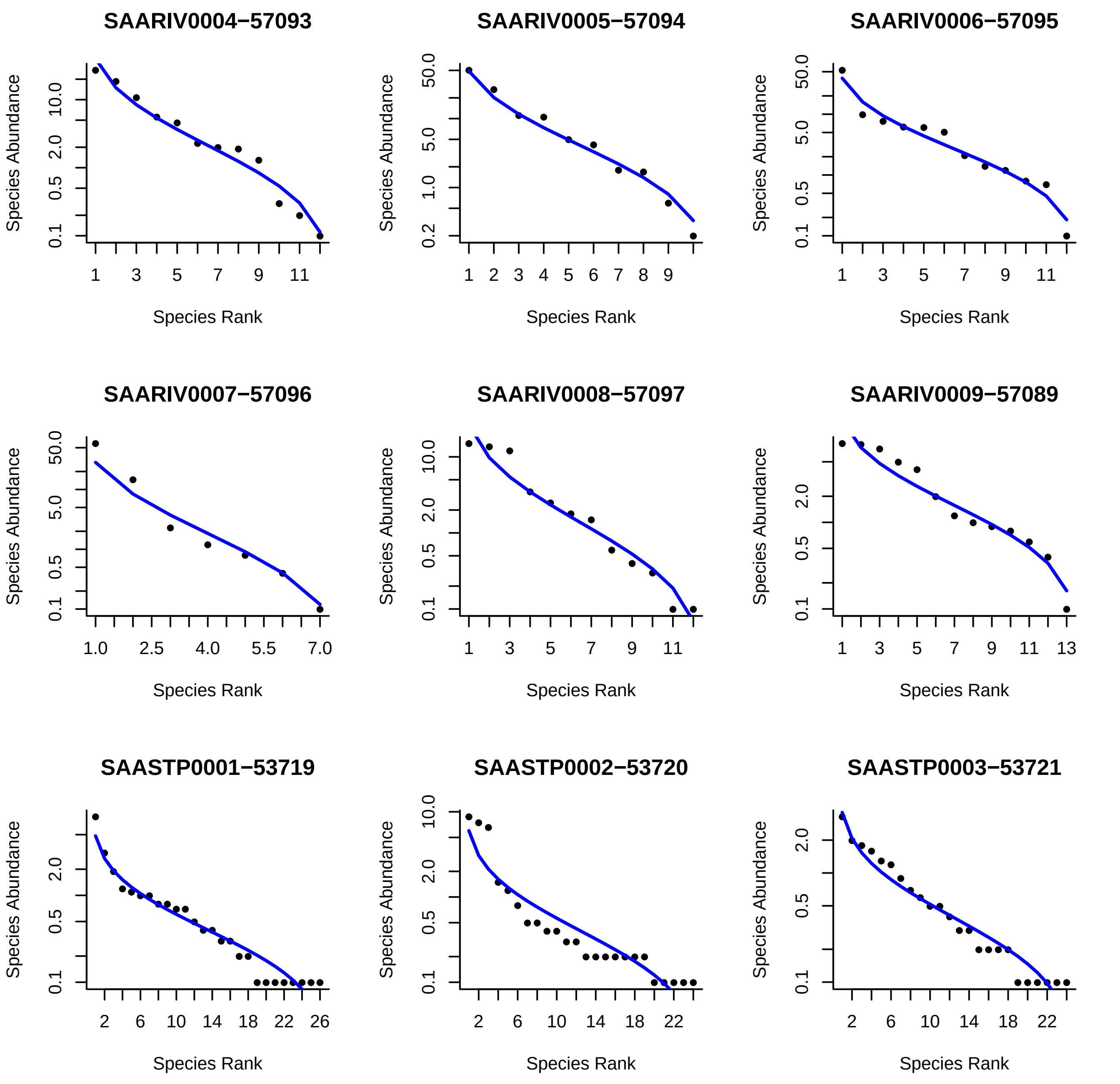

SAARIV0004−57093
Species Abundance
0.1
0.5
2.0
10.0
1
3
5
7
9
11
Species Rank
SAARIV0005−57094
Species Abundance
0.2
1.0
5.0
50.0
1
2
3
4
5
6
7
8
9
Species Rank
SAARIV0006−57095
Species Abundance
0.1
0.5
5.0
50.0
1
3
5
7
9
11
Species Rank
SAARIV0007−57096
Species Abundance
0.1
0.5
5.0
50.0
1.0
2.5
4.0
5.5
7.0
Species Rank
SAARIV0008−57097
Species Abundance
0.1
0.5
2.0
10.0
1
3
5
7
9
11
Species Rank
SAARIV0009−57089
Species Abundance
0.1
0.5
2.0
1
3
5
7
9
11
13
Species Rank
SAASTP0001−53719
Species Abundance
0.1
0.5
2.0
2
6
10
14
18
22
26
Species Rank
SAASTP0002−53720
Species Abundance
0.1
0.5
2.0
10.0
2
6
10
14
18
22
Species Rank
SAASTP0003−53721
Species Abundance
0.1
0.5
2.0
2
6
10
14
18
22
Species Rank

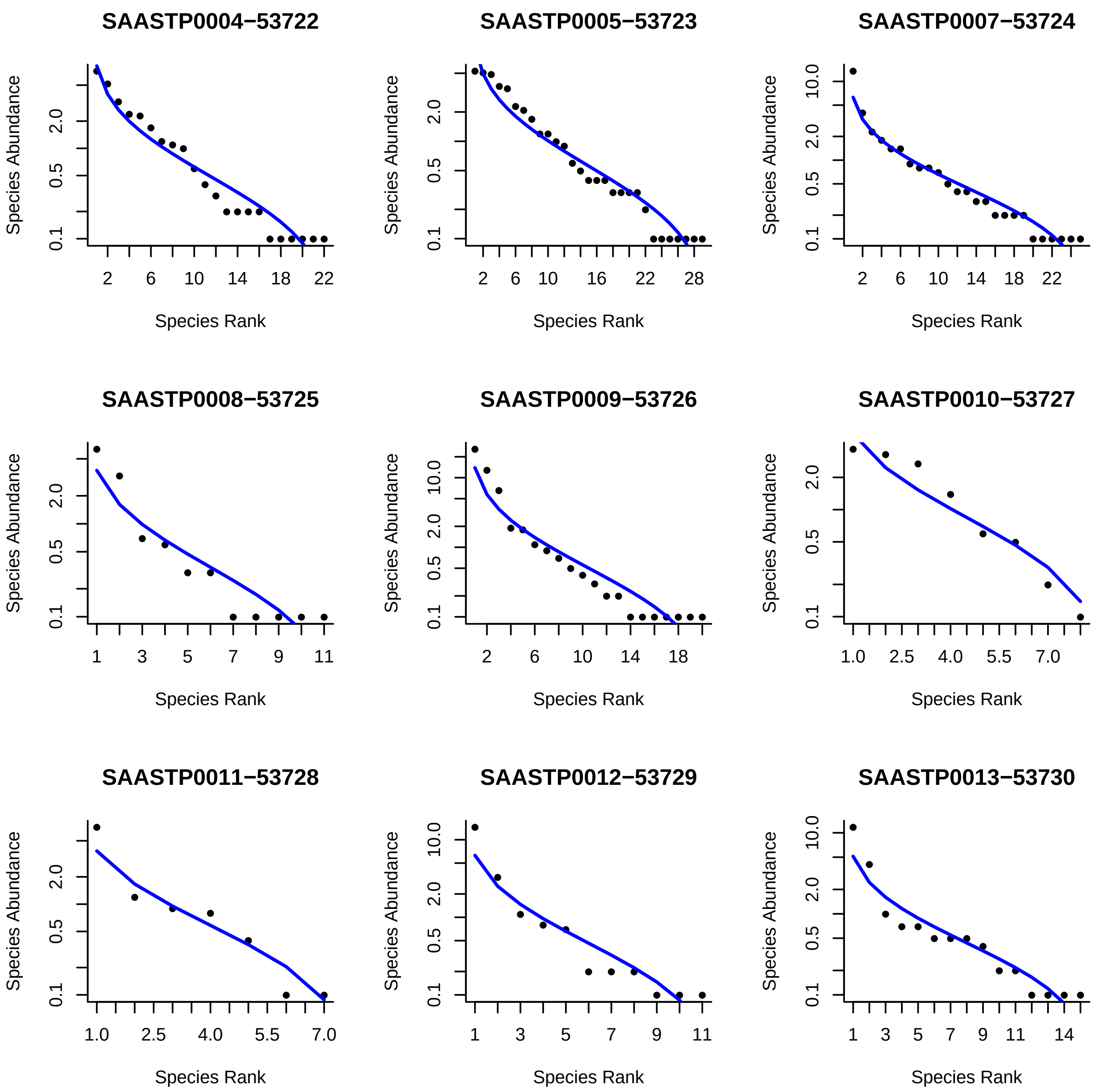
SAASTP0004−53722
SAASTP0005−53723
SAASTP0007−53724
SAASTP0008−53725
SAASTP0009−53726
SAASTP0010−53727
SAASTP0011−53728
SAASTP0012−53729
SAASTP0013−53730
Species Abundance
Species Rank
0.1
0.5
2.0
10.0
2 6 10 14 18 22
2 6 10 16 22 28
2 6 10 14 18 22
1 3 5 7 9 11
2 6 10 14 18
1.0 2.5 4.0 5.5 7.0
1.0 2.5 4.0 5.5 7.0
1 3 5 7 9 11
1 3 5 7 9 11 14

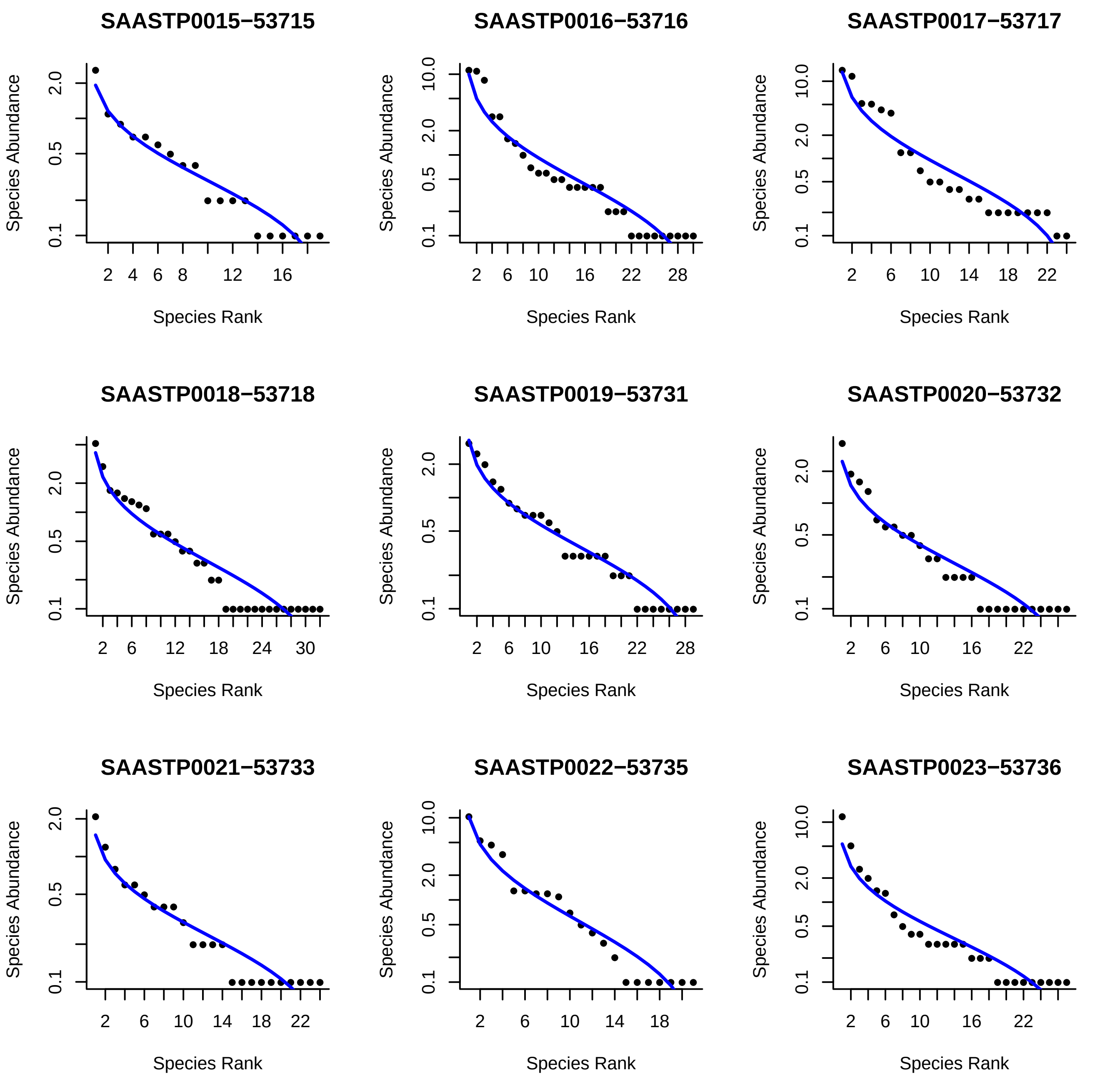

SAASTP0015−53715
Species Abundance
0.1
0.5
2.0
2
4
6
8
12
16
Species Rank
SAASTP0016−53716
Species Abundance
0.1
0.5
2.0
10.0
2
6
10
16
22
28
Species Rank
SAASTP0017−53717
Species Abundance
0.1
0.5
2.0
10.0
2
6
10
14
18
22
Species Rank
SAASTP0018−53718
Species Abundance
0.1
0.5
2.0
2
6
12
18
24
30
Species Rank
SAASTP0019−53731
Species Abundance
0.1
0.5
2.0
2
6
10
16
22
28
Species Rank
SAASTP0020−53732
Species Abundance
0.1
0.5
2.0
2
6
10
16
22
Species Rank
SAASTP0021−53733
Species Abundance
0.1
0.5
2.0
2
6
10
14
18
22
Species Rank
SAASTP0022−53735
Species Abundance
0.1
0.5
2.0
10.0
2
6
10
14
18
Species Rank
SAASTP0023−53736
Species Abundance
0.1
0.5
2.0
10.0
2
6
10
16
22
Species Rank

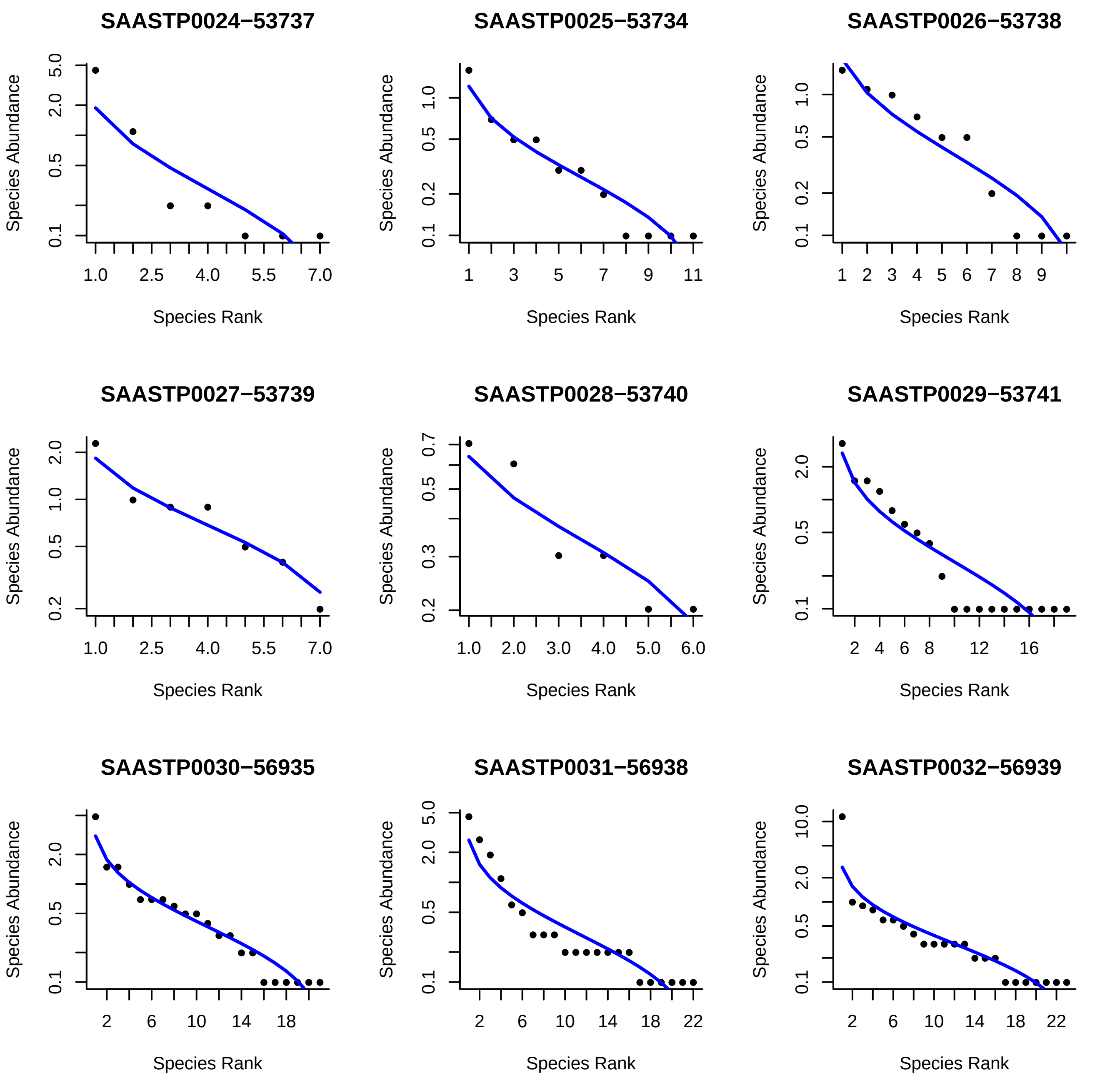
SAASTP0024−53737
Species Abundance
Species Rank
SAASTP0025−53734
Species Abundance
Species Rank
SAASTP0026−53738
Species Abundance
Species Rank
SAASTP0027−53739
Species Abundance
Species Rank
SAASTP0028−53740
Species Abundance
Species Rank
SAASTP0029−53741
Species Abundance
Species Rank
SAASTP0030−56935
Species Abundance
Species Rank
SAASTP0031−56938
Species Abundance
Species Rank
SAASTP0032−56939
Species Abundance
Species Rank

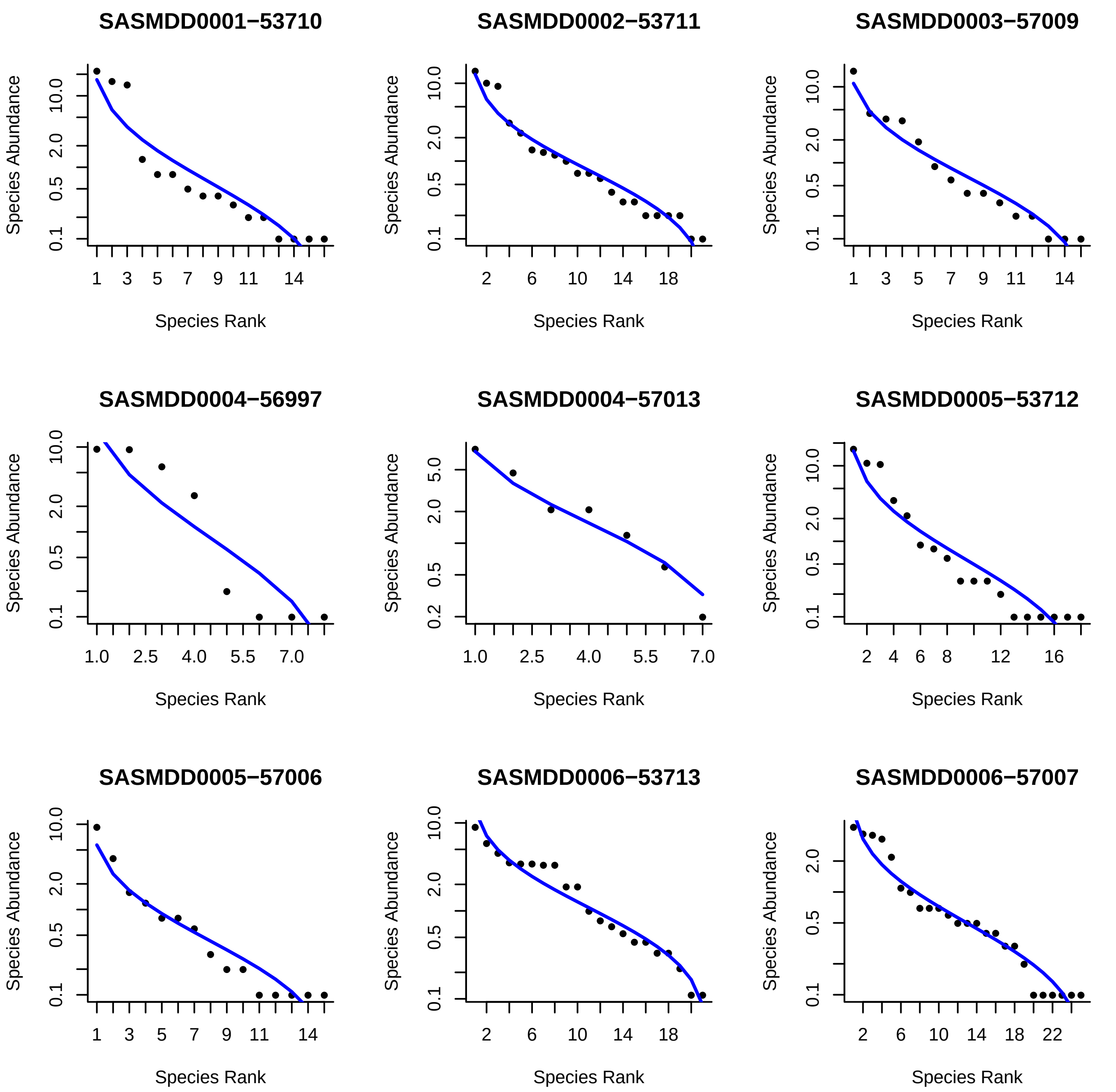
SASMDD0001−53710
SASMDD0002−53711
SASMDD0003−57009
SASMDD0004−56997
SASMDD0004−57013
SASMDD0005−53712
SASMDD0005−57006
SASMDD0006−53713
SASMDD0006−57007
Species Abundance
Species Rank

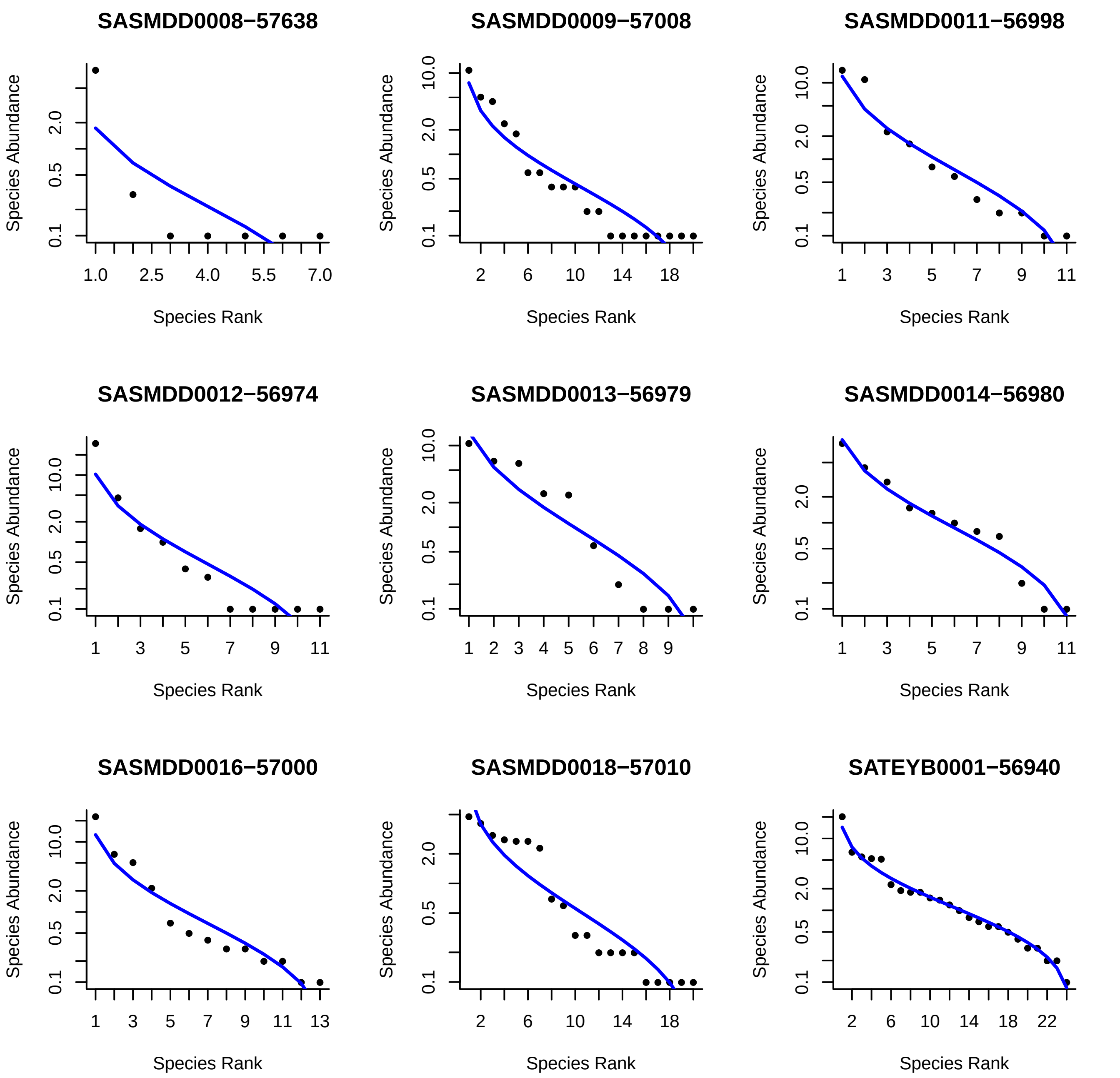
SASMDD0008−57638
SASMDD0009−57008
SASMDD0011−56998
SASMDD0012−56974
SASMDD0013−56979
SASMDD0014−56980
SASMDD0016−57000
SASMDD0018−57010
SATEYB0001−56940
Species Abundance
Species Rank
0.1
0.5
2.0
10.0
1.0
2.5
4.0
5.5
7.0
2
6
10
14
18
22
1
3
5
7
9
11
13
4
8

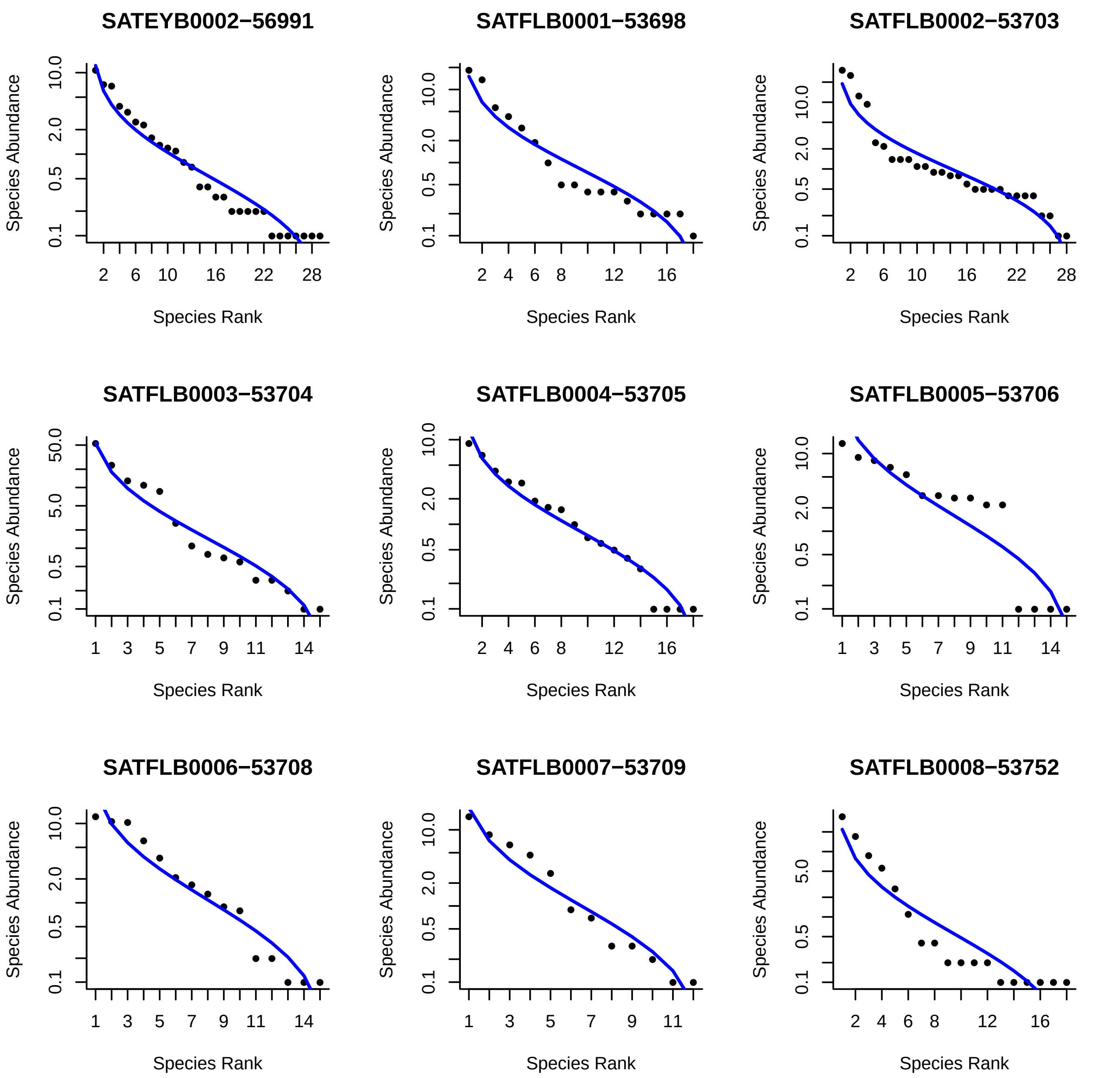
SATEYB0002−56991
SATFLB0001−53698
SATFLB0002−53703
SATFLB0003−53704
SATFLB0004−53705
SATFLB0005−53706
SATFLB0006−53708
SATFLB0007−53709
SATFLB0008−53752
Species Abundance
Species Rank

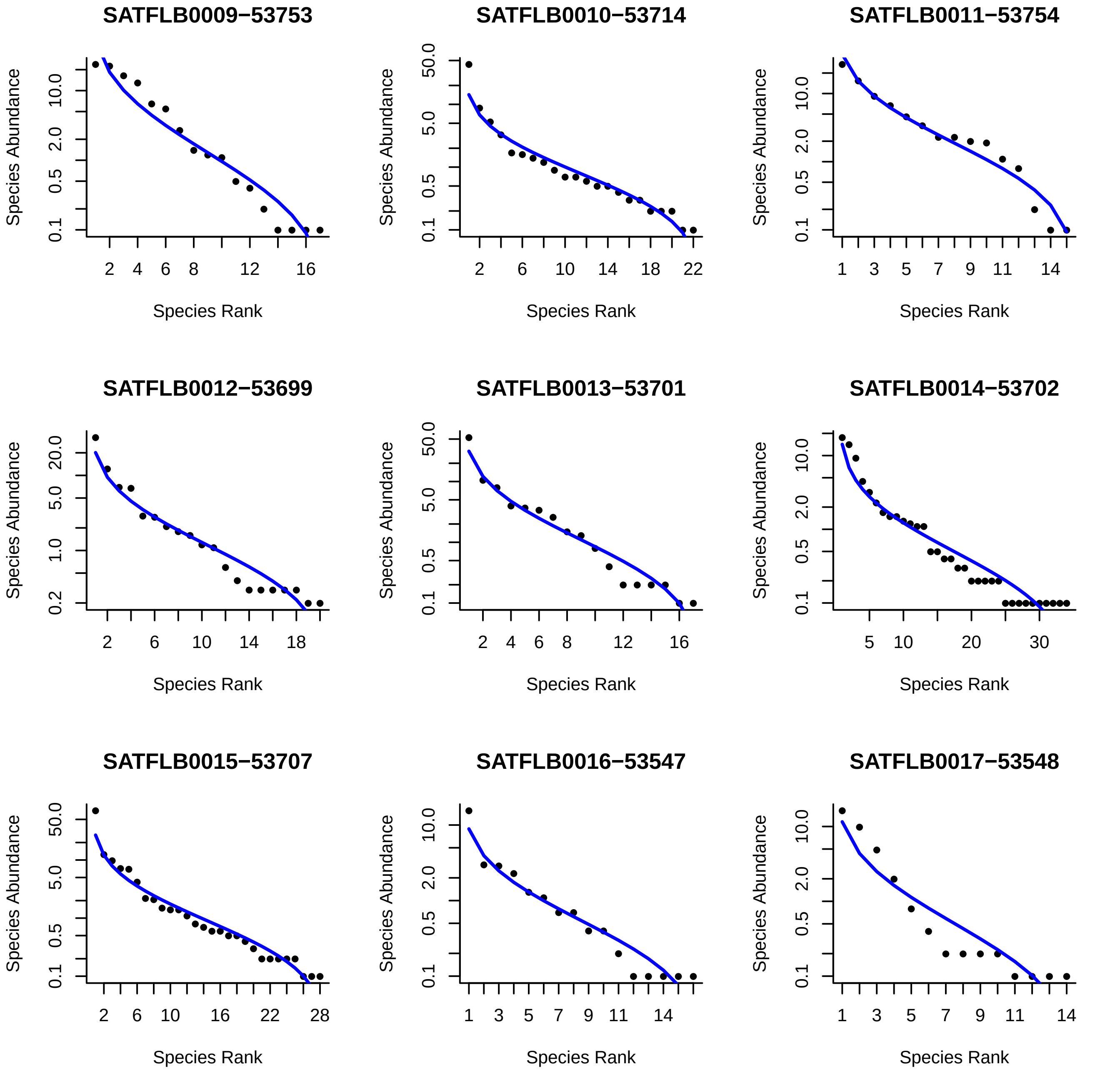

SATFLB0009−53753
Species Abundance
0.1
0.5
2.0
10.0
2
4
6
8
12
16
Species Rank
SATFLB0010−53714
Species Abundance
0.1
0.5
5.0
50.0
2
6
10
14
18
22
Species Rank
SATFLB0011−53754
Species Abundance
0.1
0.5
2.0
10.0
1
3
5
7
9
11
14
Species Rank
SATFLB0012−53699
Species Abundance
0.2
1.0
5.0
20.0
2
6
10
14
18
Species Rank
SATFLB0013−53701
Species Abundance
0.1
0.5
5.0
50.0
2
4
6
8
12
16
Species Rank
SATFLB0014−53702
Species Abundance
0.1
0.5
2.0
10.0
5
10
20
30
Species Rank
SATFLB0015−53707
Species Abundance
0.1
0.5
5.0
50.0
2
6
10
16
22
28
Species Rank
SATFLB0016−53547
Species Abundance
0.1
0.5
2.0
10.0
1
3
5
7
9
11
14
Species Rank
SATFLB0017−53548
Species Abundance
0.1
0.5
2.0
10.0
1
3
5
7
9
11
14
Species Rank

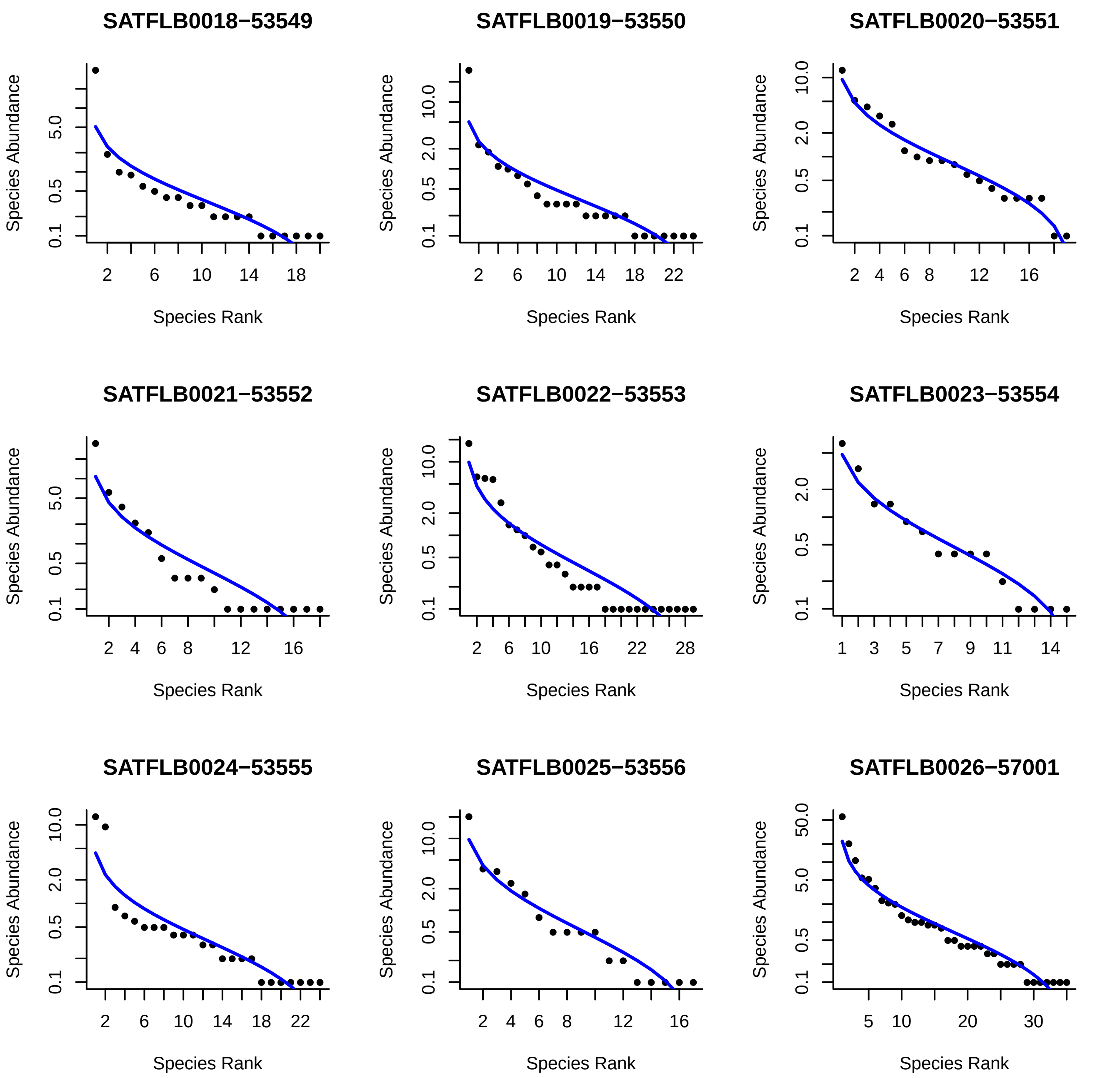

SATFLB0018−53549
SATFLB0019−53550
SATFLB0020−53551
SATFLB0021−53552
SATFLB0022−53553
SATFLB0023−53554
SATFLB0024−53555
SATFLB0025−53556
SATFLB0026−57001
Species Abundance
Species Rank
0.1
0.5
2.0
5.0
10.0
50.0
2 6 10 14 18 22
2 4 6 8 12 16
2 6 10 16 22 28
1 3 5 7 9 11 14
5 10 20 30

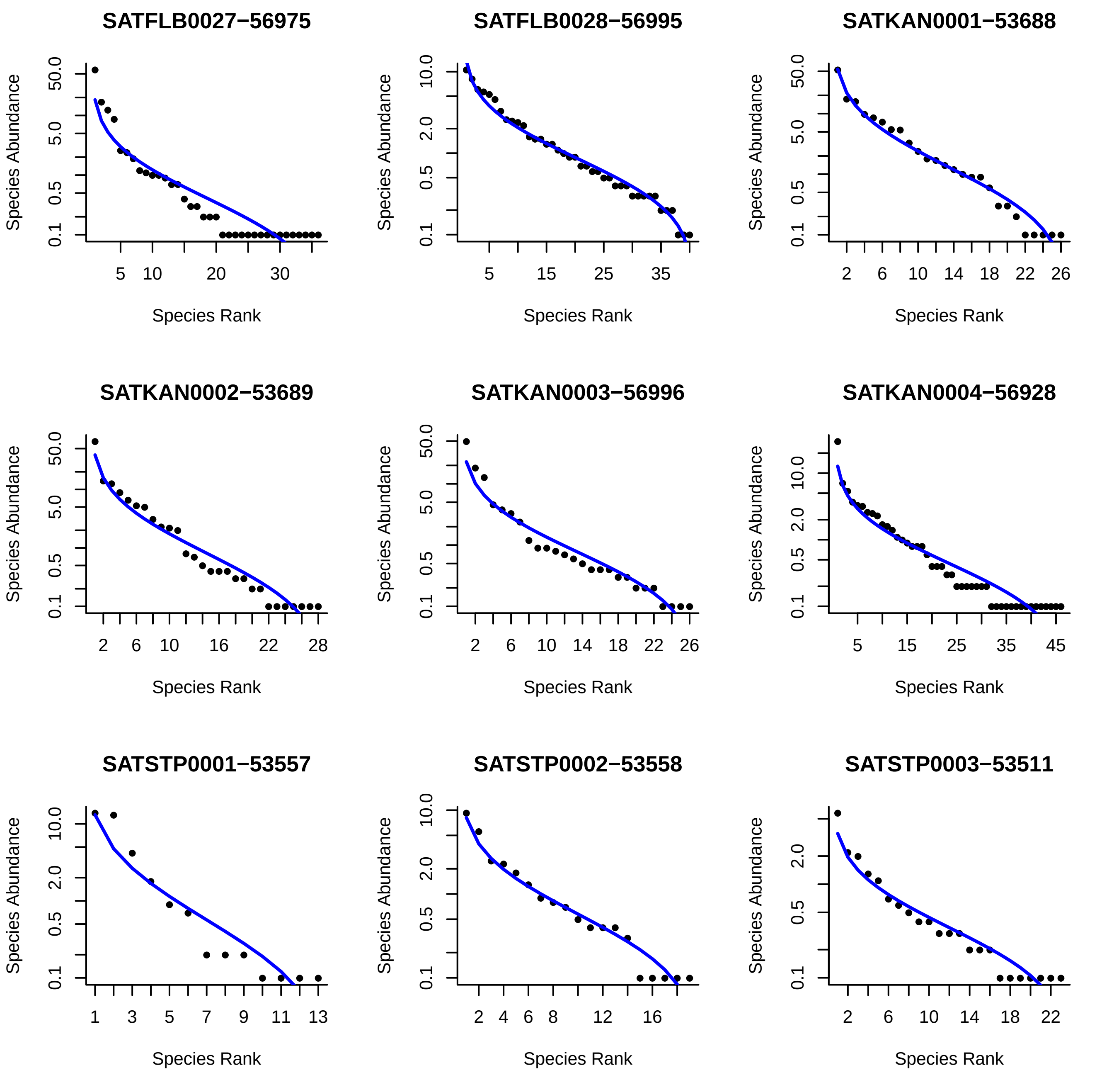
SATFLB0027−56975
SATFLB0028−56995
SATKAN0001−53688
SATKAN0002−53689
SATKAN0003−56996
SATKAN0004−56928
SATSTP0001−53557
SATSTP0002−53558
SATSTP0003−53511
Species Abundance
Species Rank
0.1
0.5
2.0
5.0
10.0
50.0
1
2
3
4
5
6
7
8
9
10
11
12
13
14
15
16
18
20
22
25
26
28
30
35
45

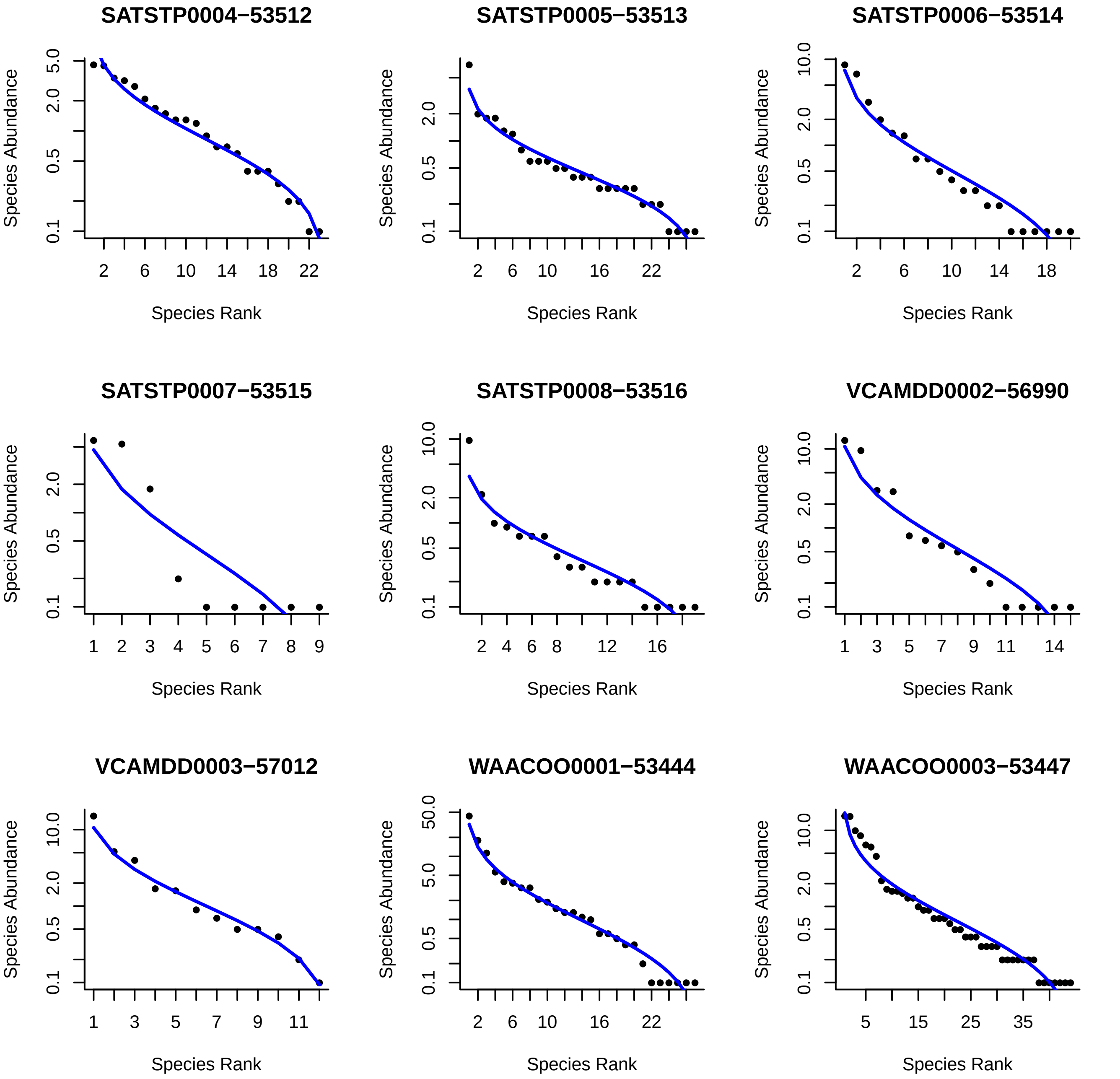
SATSTP0004−53512
SATSTP0005−53513
SATSTP0006−53514
SATSTP0007−53515
SATSTP0008−53516
VCAMDD0002−56990
VCAMDD0003−57012
WAACOO0001−53444
WAACOO0003−53447
Species Abundance
Species Rank
0.1
0.5
2.0
5.0
10.0
50.0

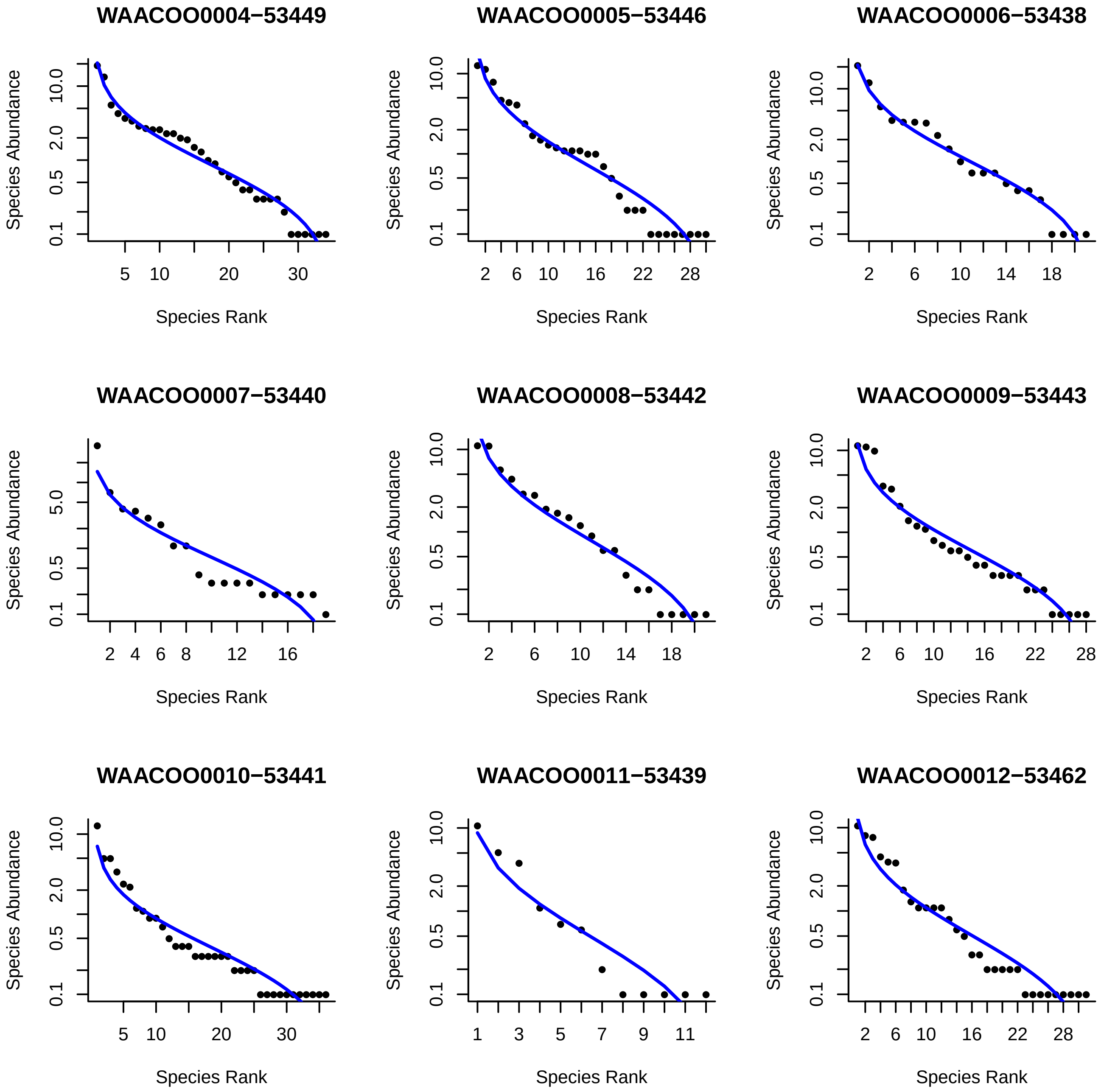
WAACOO0004−53449
WAACOO0005−53446
WAACOO0006−53438
WAACOO0007−53440
WAACOO0008−53442
WAACOO0009−53443
WAACOO0010−53441
WAACOO0011−53439
WAACOO0012−53462
Species Abundance
Species Rank
0.1
0.5
2.0
5.0
10.0
5
10
20
30
2
6
16
22
28
14
18
4
8
12
1
3
7
9
11

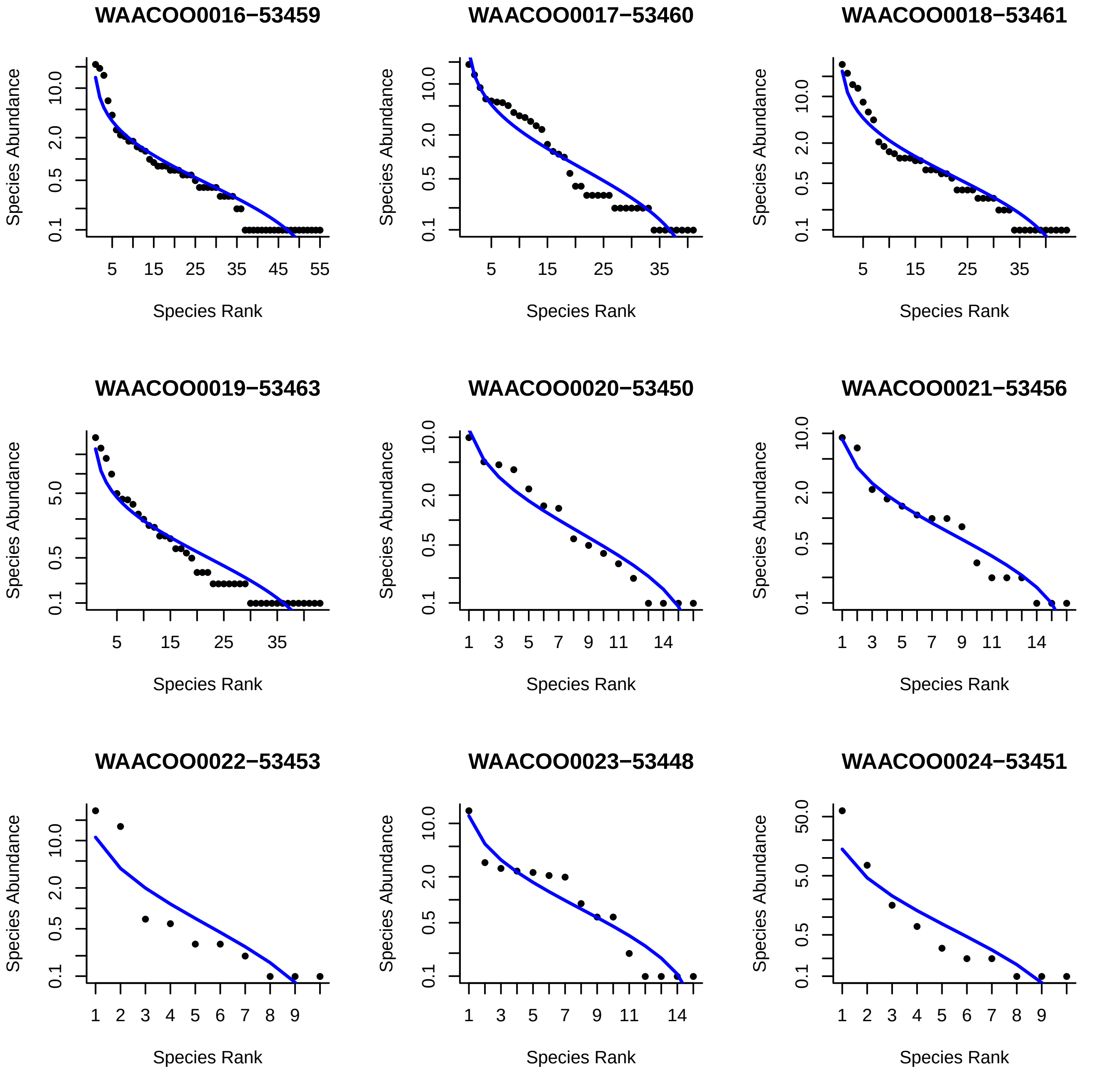
WAACOO0016−53459
WAACOO0017−53460
WAACOO0018−53461
WAACOO0019−53463
WAACOO0020−53450
WAACOO0021−53456
WAACOO0022−53453
WAACOO0023−53448
WAACOO0024−53451
Species Abundance
Species Rank

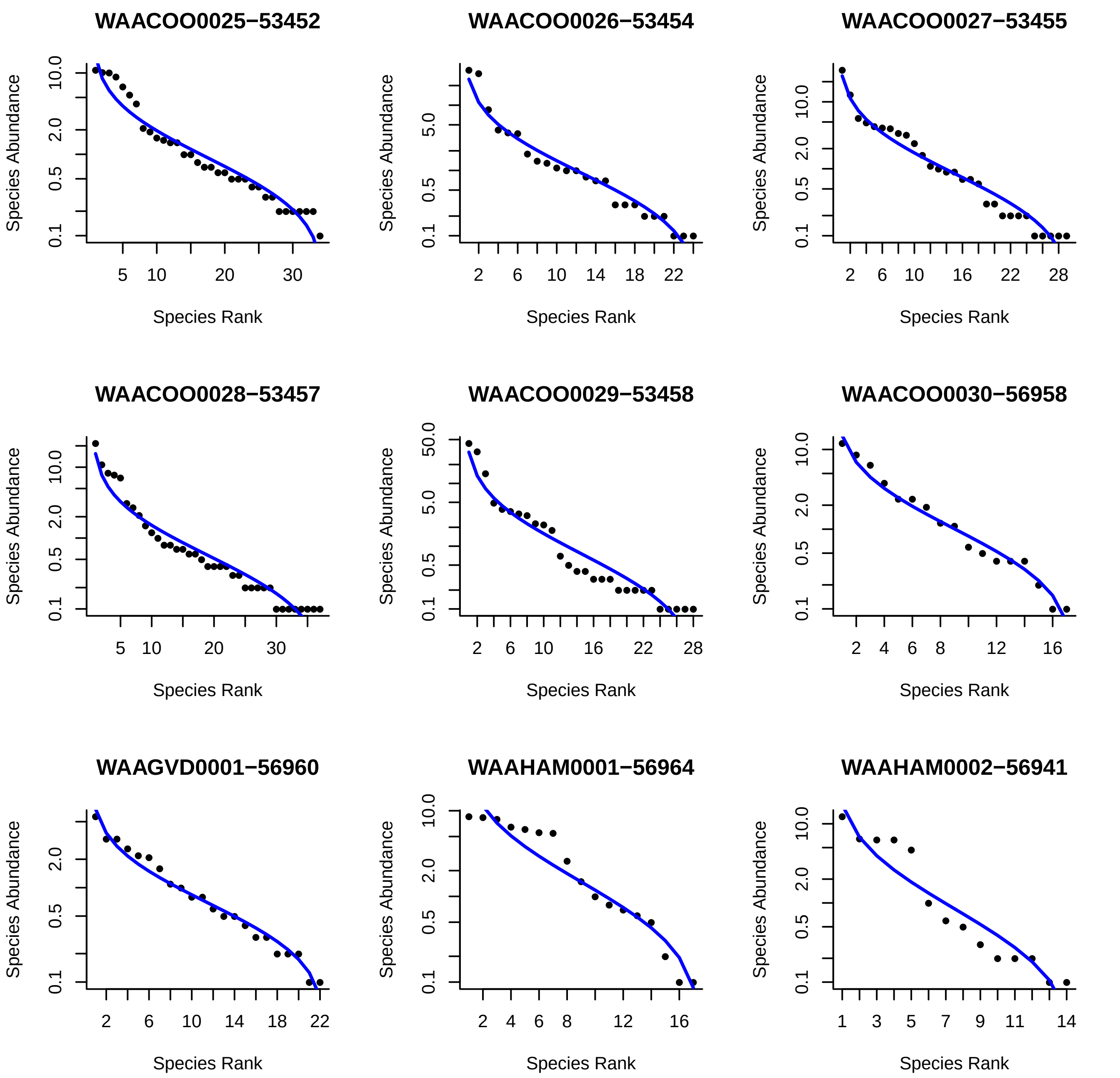
WAACOO0025−53452
Species Abundance
0.1 0.5 2.0 10.0
5 10 20 30
Species Rank
WAACOO0026−53454
Species Abundance
0.1 0.5 5.0
2 6 10 14 18 22
Species Rank
WAACOO0027−53455
Species Abundance
0.1 0.5 2.0 10.0
2 6 10 16 22 28
Species Rank
WAACOO0028−53457
Species Abundance
0.1 0.5 2.0 10.0
5 10 20 30
Species Rank
WAACOO0029−53458
Species Abundance
0.1 0.5 5.0 50.0
2 6 10 16 22 28
Species Rank
WAACOO0030−56958
Species Abundance
0.1 0.5 2.0 10.0
2 4 6 8 12 16
Species Rank
WAAGVD0001−56960
Species Abundance
0.1 0.5 2.0
2 6 10 14 18 22
Species Rank
WAAHAM0001−56964
Species Abundance
0.1 0.5 2.0 10.0
2 4 6 8 12 16
Species Rank
WAAHAM0002−56941
Species Abundance
0.1 0.5 2.0 10.0
1 3 5 7 9 11 14
Species Rank

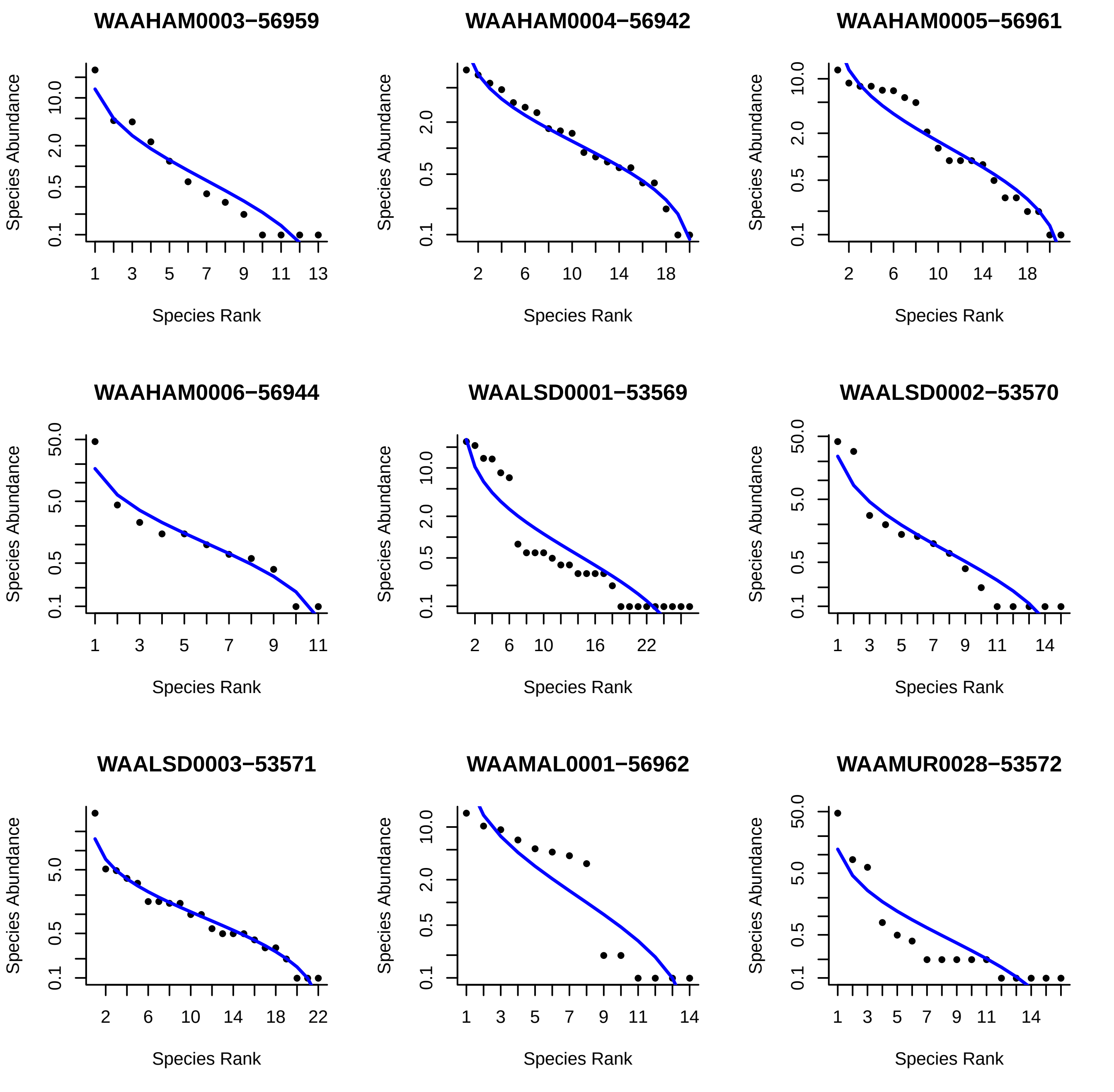
WAAHAM0003−56959
Species Abundance
10.0
2.0
0.5
0.1
1 3 5 7 9 11 13
Species Rank
WAAHAM0004−56942
Species Abundance
2.0
0.5
0.1
2 6 10 14 18
Species Rank
WAAHAM0005−56961
Species Abundance
10.0
2.0
0.5
0.1
2 6 10 14 18
Species Rank
WAAHAM0006−56944
Species Abundance
50.0
5.0
0.5
0.1
1 3 5 7 9 11
Species Rank
WAALSD0001−53569
Species Abundance
10.0
2.0
0.5
0.1
2 6 10 16 22
Species Rank
WAALSD0002−53570
Species Abundance
50.0
5.0
0.5
0.1
1 3 5 7 9 11 14
Species Rank
WAALSD0003−53571
Species Abundance
5.0
0.5
0.1
2 6 10 14 18 22
Species Rank
WAAMAL0001−56962
Species Abundance
10.0
2.0
0.5
0.1
1 3 5 7 9 11 14
Species Rank
WAAMUR0028−53572
Species Abundance
50.0
5.0
0.5
0.1
1 3 5 7 9 11 14
Species Rank

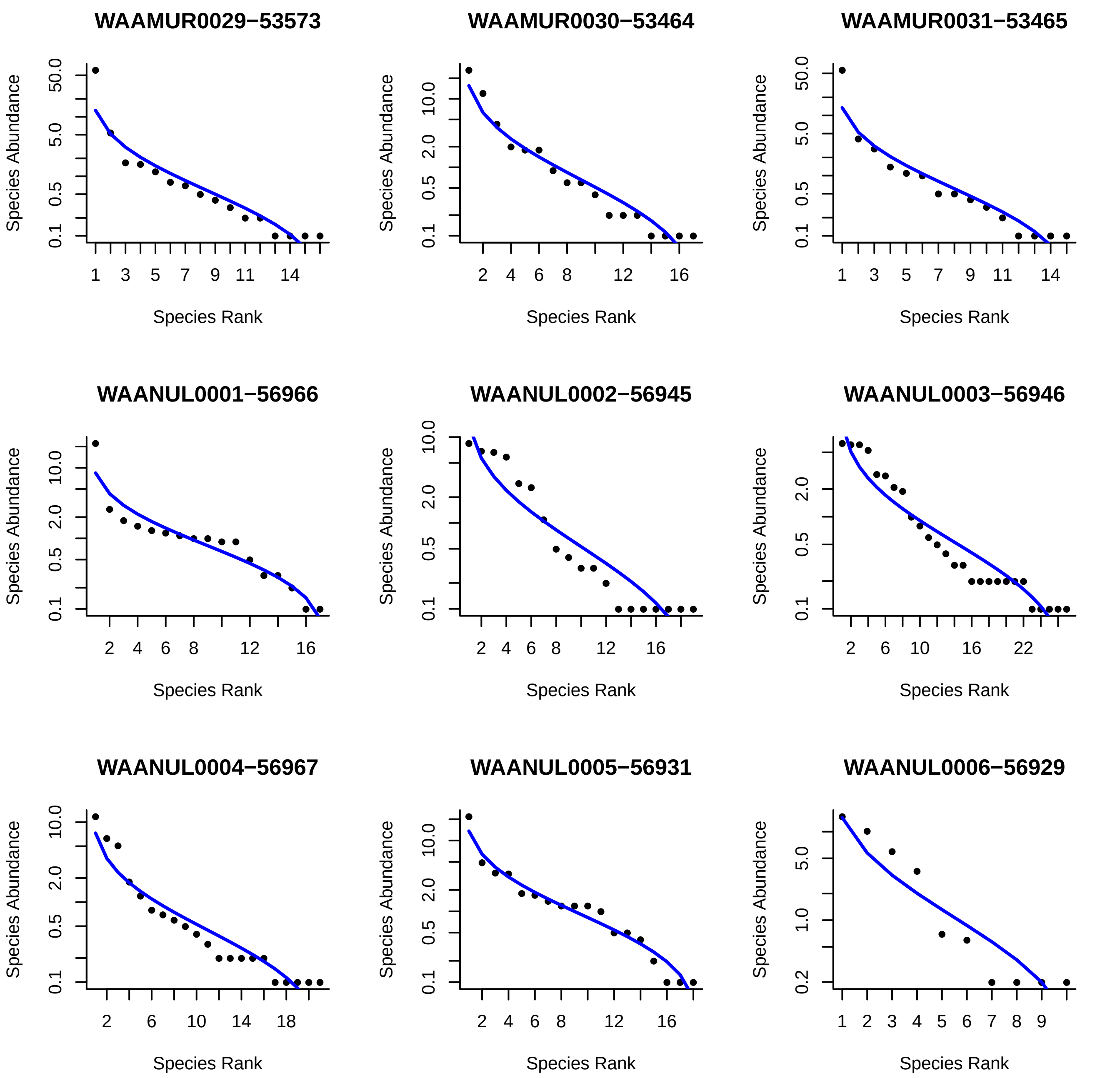
WAAMUR0029−53573
WAAMUR0030−53464
WAAMUR0031−53465
WAANUL0001−56966
WAANUL0002−56945
WAANUL0003−56946
WAANUL0004−56967
WAANUL0005−56931
WAANUL0006−56929
Species Abundance
Species Rank

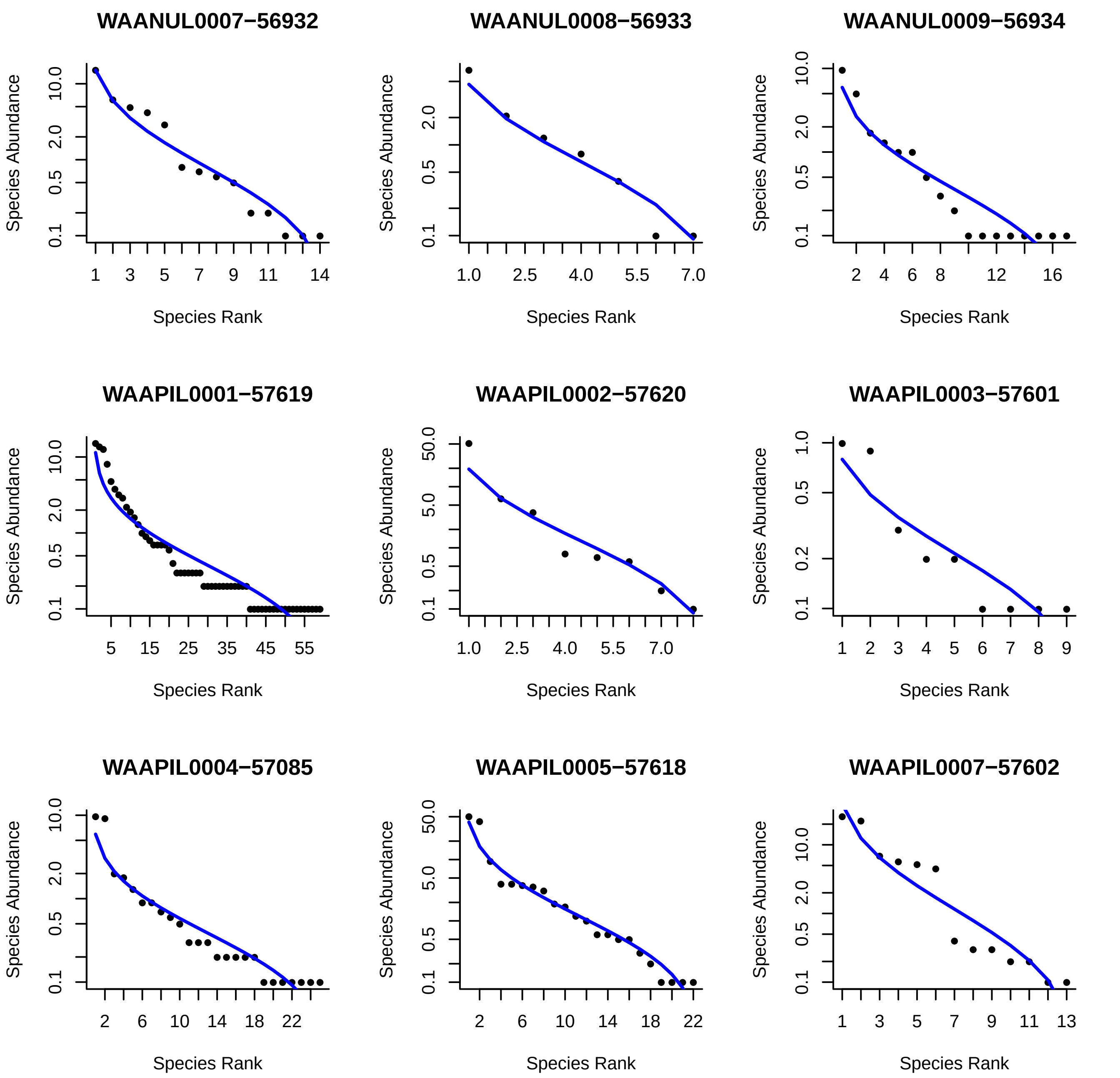
WAANUL0007−56932
Species Abundance
Species Rank
0.1
0.5
2.0
10.0
1
3
5
7
9
11
14
WAANUL0008−56933
Species Abundance
Species Rank
0.1
0.5
2.0
1.0
2.5
4.0
5.5
7.0
WAANUL0009−56934
Species Abundance
Species Rank
0.1
0.5
2.0
10.0
2
4
6
8
12
16
WAAPIL0001−57619
Species Abundance
Species Rank
0.1
0.5
2.0
10.0
5
15
25
35
45
55
WAAPIL0002−57620
Species Abundance
Species Rank
0.1
0.5
5.0
50.0
1.0
2.5
4.0
5.5
7.0
WAAPIL0003−57601
Species Abundance
Species Rank
0.1
0.2
0.5
1.0
1
2
3
4
5
6
7
8
9
WAAPIL0004−57085
Species Abundance
Species Rank
0.1
0.5
2.0
10.0
2
6
10
14
18
22
WAAPIL0005−57618
Species Abundance
Species Rank
0.1
0.5
5.0
50.0
2
6
10
14
18
22
WAAPIL0007−57602
Species Abundance
Species Rank
0.1
0.5
2.0
10.0
1
3
5
7
9
11
13

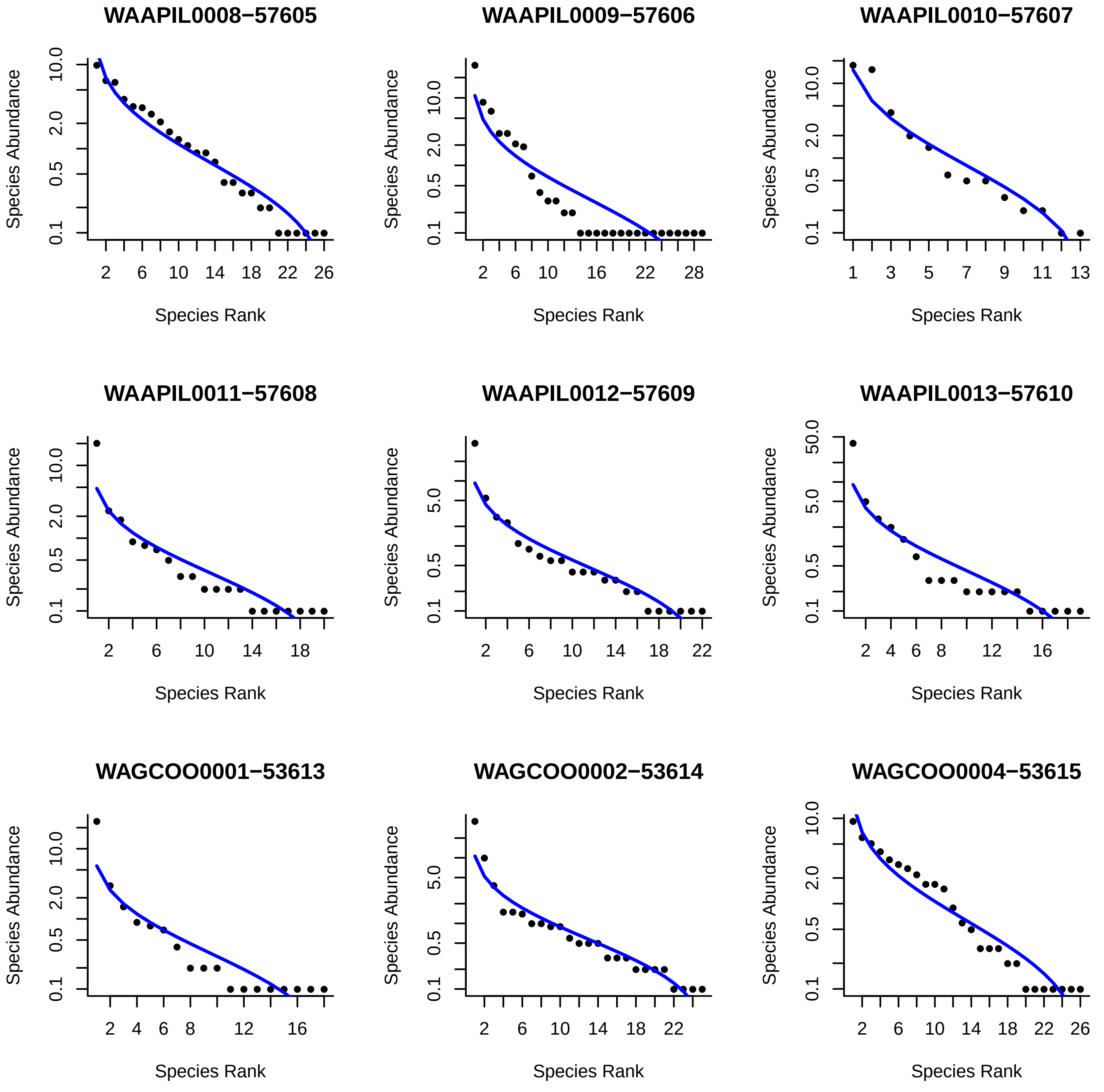
WAAPIL0008−57605
WAAPIL0009−57606
WAAPIL0010−57607
WAAPIL0011−57608
WAAPIL0012−57609
WAAPIL0013−57610
WAGCOO0001−53613
WAGCOO0002−53614
WAGCOO0004−53615
Species Abundance
Species Rank
0.1
0.5
2.0
5.0
10.0
50.0
2
6
10
14
18
22
26
28
1
3
5
7
9
11
13
4
8
12
16
20
