# Supplementary material for: Opportunities for Integrated Ecological Analysis across Inland Australia with Standardised Data from Ausplots Rangelands
Source: PLoS One. 2017 Jan 17;12(1):e0170137. doi: 10.1371/journal.pone.0170137 (PMC5241013; doi:10.1371/journal.pone.0170137)
Supplement: S6 Appendix — Regressions in subsets by vegetation group with predictor variable Mean annual precipitation (MAP) and response variable shape coefficients of SADs models fit to abundance data for AuspPlots using the lognormal distribution. (PDF) [file pone.0170137.s006.pdf]

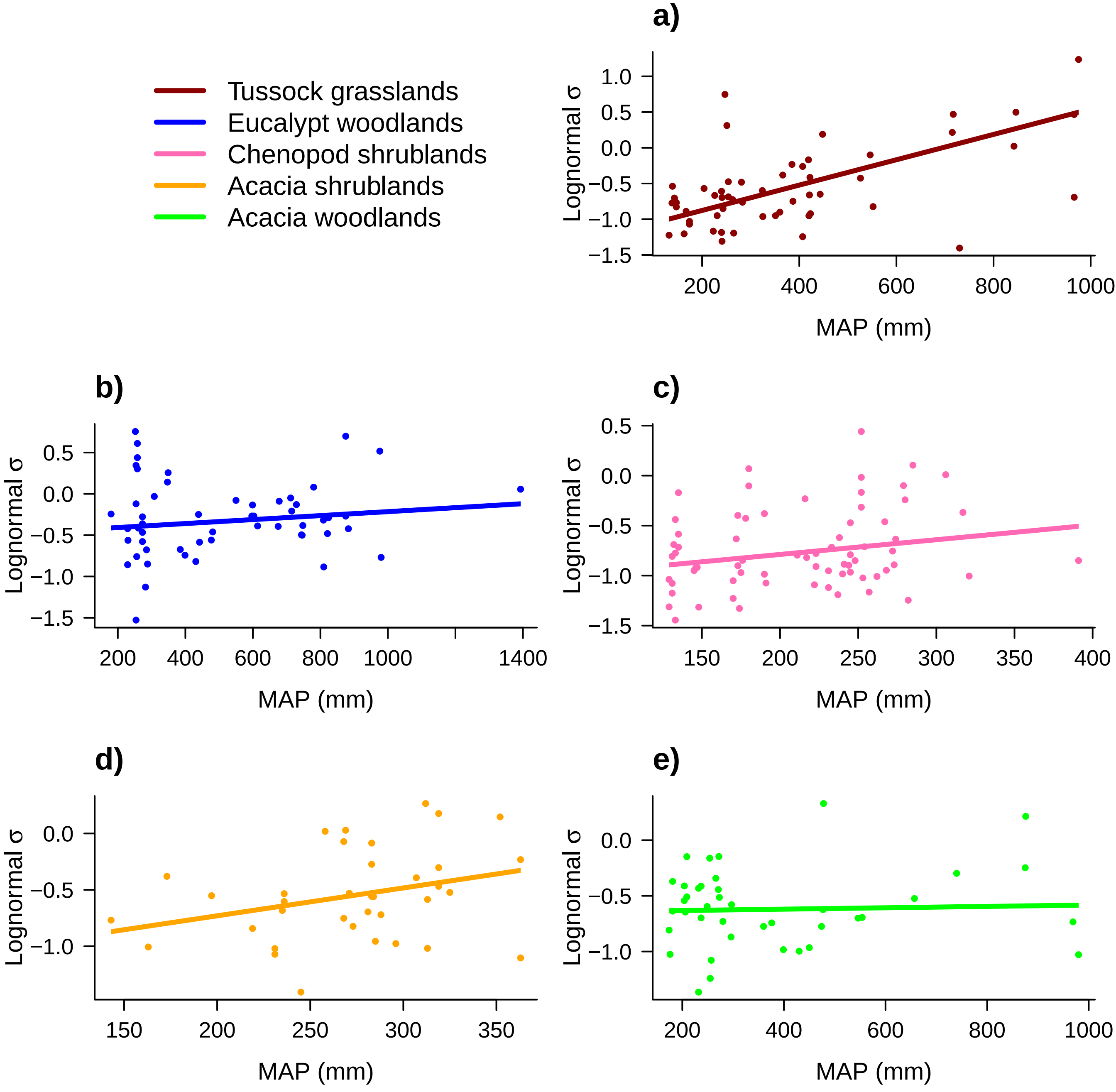
a)
b)
c)
d)
e)
Tussock grasslands
Eucalypt woodlands
Chenopod shrublands
Acacia shrublands
Acacia woodlands
Lognormal σ
MAP (mm)
